# High Performance Thin-Layer Chromatography (HPTLC) data of Cannabinoids in ten mobile phase systems

**DOI:** 10.1016/j.dib.2020.105955

**Published:** 2020-06-30

**Authors:** Yifan Liu, Justin Victoria, Matthew Wood, Marianne E. Staretz, Thomas A. Brettell

**Affiliations:** a Forensic Science Program, Department of Chemical and Physical Sciences, Cedar Crest College, Allentown, PA, 18104, USA; b Ocean County Sheriff's Department, Toms River, NJ, 08753, USA

**Keywords:** Forensic chemistry, Cannabis, Cannabinoids, Hptlc, Thin-layer chromatography

## Abstract

This article contains data from triplicate analyses of ten different TLC mobile phase systems from the analysis of 11 cannabinoid standards using HPTLC. The data includes 1) example plate images for 8 mobile phases during evaluation; 2) example table of calculated average, standard deviation, CV% of R_F_ for hexane:acetone (87:13) system during evaluation; 3) images of all case sample plates. The validation of the two chosen mobile phases were done according to the SWGDRUG validation requirements [1]. This data can be used for choosing an alternative mobile phase TLC system for the analysis of cannabinoids. The data described in this article is related to a previous published research article “High Performance Thin-Layer Chromatography (HPTLC) analysis of cannabinoids in cannabis extracts” by Liu, et al. (2020) [2].

**Data in brief**

Specifications table

**Table:** 

Subject	Forensic Chemistry
Specific subject area	HPTLC, cannabinoids
Type of data	Tables Images Figures
How data were acquired	Plates were run on CAMAG HPTLC instruments and data were collected using VisionCATS software.
Data format	Raw: HPTLC original reports; Images of plates Analyzed: Excel tables and figures.
Parameters for data collection	11 certified reference cannabinoid standards were run on 10 different mobile phases in triplicates. Reports were automatically generated after each run using the instrument software. R_F_ values in each report were used to calculate resolution.
Description of data collection	R_F_ values were exported to an excel spreadsheet, where the calculations were performed.
Data source location	Cedar Crest College Allentown, Pennsylvania
Data accessibility	All the data are provided with the article.
Related research article	Author's name: Y. Liu et al. Title: High Performance Thin-Layer Chromatography (HPTLC) analysis of cannabinoids in cannabis extracts Journal: Forensic Chemistry (2020) DOI:https://doi.org/10.1016/j.forc.2020.100249

**Value of the data**

• Example images of each mobile phase system with cannabinoid standards can visually help researchers understand which mobile phase results in better separation in the analysis of cannabinoids. Figures that compare each mobile phase can provide a visual of the relationship between the positions of cannabinoids to assist researchers in choosing the proper mobile phase.
• The tables of R_F_ values and resolution calculation for different cannabinoids in different mobile phase systems can provide numerical support when selecting a mobile phase to perform analyses. The data will serve as references for agencies and crime labs in the analysis of seized cannabis products.
• Case sample images can provide a reference to determine the proper mobile phase for crime laboratories and researchers performing HPTLC analysis of cannabinoids.

## 1 Data description

This data article includes 1) example plate images for 8 mobile phases during evaluation; 2) example of calculated average, standard deviation, CV% of R_F_ values and resolution for 11 cannabinoid standards in one mobile phase after three replicates; 3) images of example case samples analyzed on two mobile systems.

**Fig. 1:**
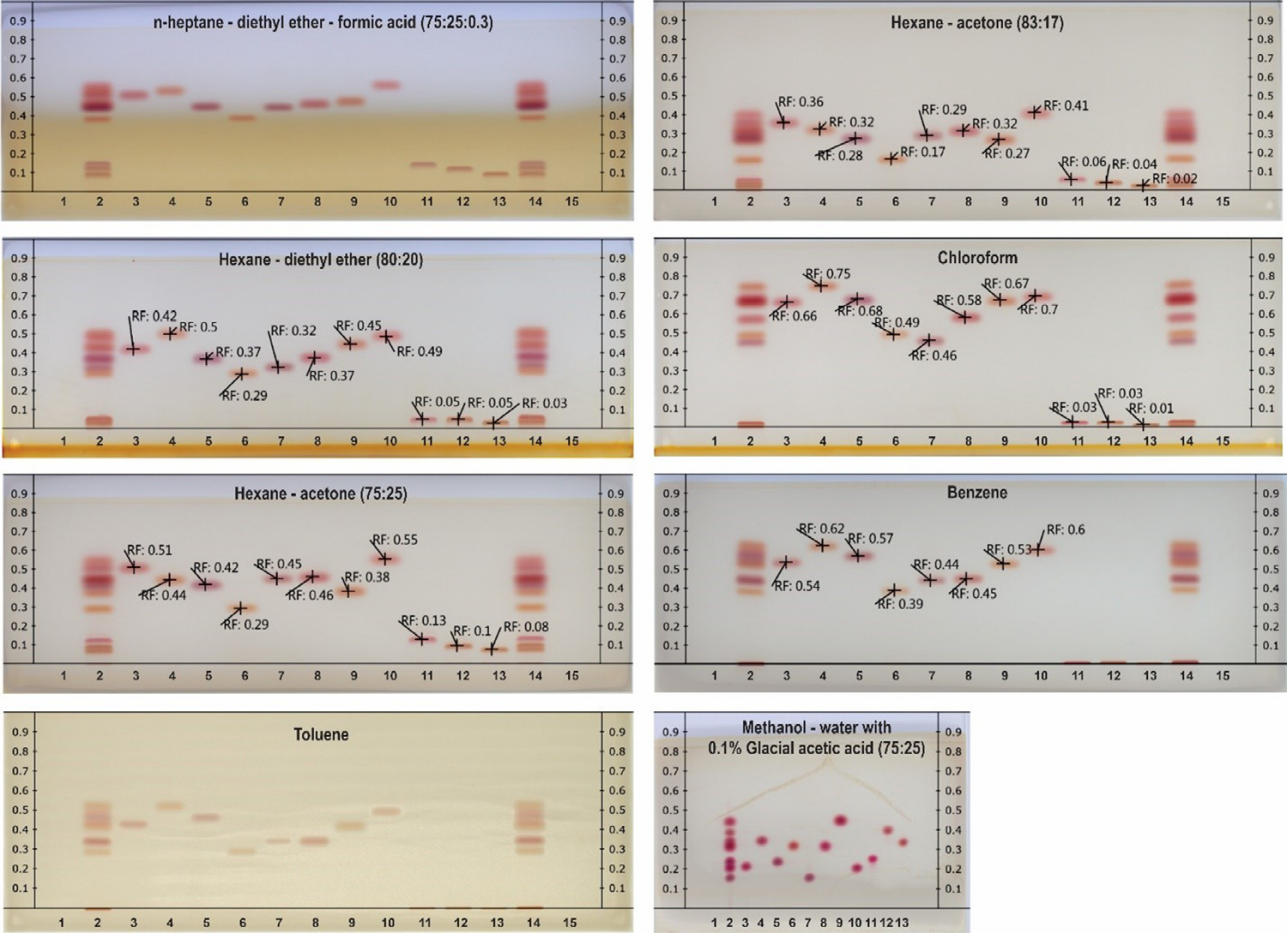
Example plates for eight mobile phases. Y-axis denotes R_F_ value. Lane 1 and 15: methanol blank, lane 2 and 14: mixture of 11 cannabinoid standards which can be seen in lanes 3–13 respectively: Δ9-THC, CBD, CBN, CBG, CBC, THCV, CBDV, Δ8-THC, THCA-A, CBDA and CBGA.

Fig. 1 shows example plates for the eight mobile phases evaluated. The R_F_ values were determined for the 11 cannabinoid standards, delta-9-tetrahydrocannabinol (Δ9-THC), cannabidiol (CBD), cannabinol (CBN), cannabigerol (CBG), cannabichromene (CBC), tetrahydrocannabivarin (THCV), cannabidivarin (CBDV), delta-8-tetrahydrocannabinol (Δ8-THC), delta-9-tetrahydrocannabinolic acid (THCA-A), cannabinolic acid (CBDA) and cannabigerolic acid (CBGA) in lane 3–13. The original HPTLC reports of the triplicate analyses of the eight mobile phase systems can be found in the supplemental material (S1). S1 includes 24 pdf files of original HPTLC reports generated from VisionCATS software. Each file includes application format, instrument parameters, images of each plate and raw R_F_ values.

**Fig. 2:**
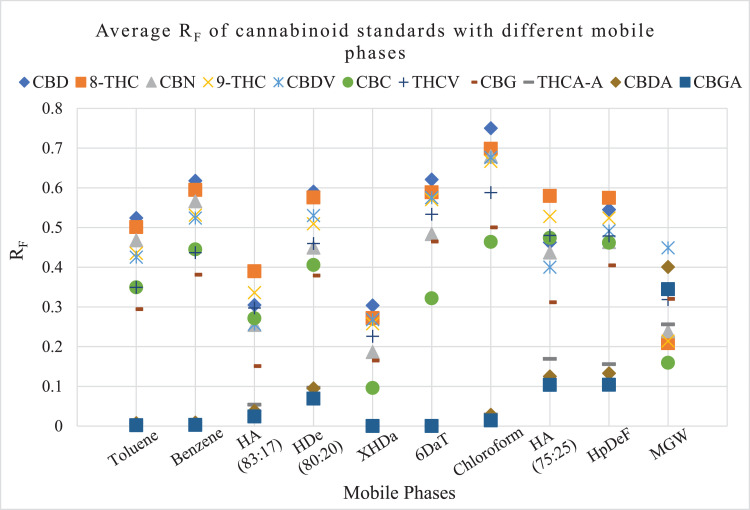
Average R_F_ of 11 cannabinoid standards in 10 mobile phase systems. Y-axis denotes R_F_ values and x-axis denotes mobile phases. Different color and shape in the figure denote different cannabinoid standards.

Fig. 2 shows a diagram of the average R_F_ values of 11 cannabinoid standards in 10 mobile phase systems after triplicate analyses. Different colors and shapes in the figure denote different cannabinoid standards and allow the reader to see the relative position of each standard in relation to the others for each of the mobile phase systems.

**Fig. 3:**
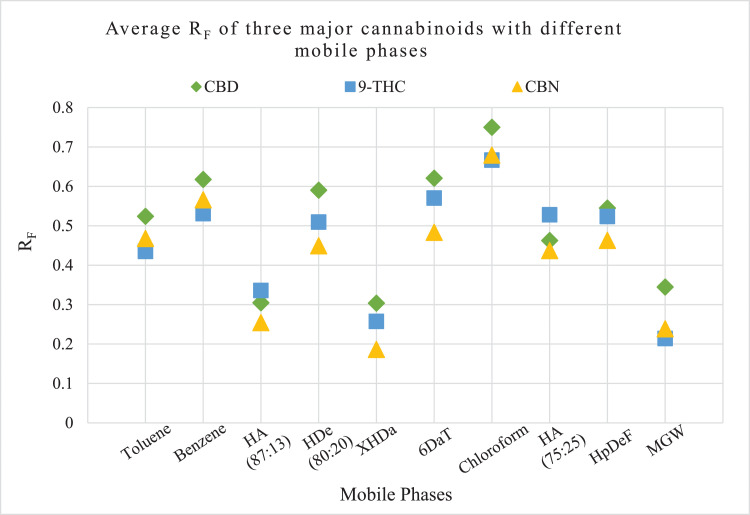
Position of Δ9-THC (), CBD (), and CBN () on 10 mobile phases. Y-axis denotes R_F_ values and x-axis denotes mobile phases.

Similarly, Fig. 3 shows a diagram of the average R_F_ values and relative elution order of the three major cannabinoid standards, Δ9-THC, CBD, and CBN, in 10 mobile phase systems.

Table 1 shows an example of the calculated average, standard deviation, CV% of R_F_ values and resolution for 11 cannabinoid standards in one mobile phase, hexane:acetone (87:13) after triplicate analysis. The raw data from which these calculations were made can be found in the excel spreadsheets provided in the supplemental material (S2, S3).

**Table 1:** Hexane:acetone (87:13).

Cannabinoids	Average R_F_(+/- St. Dev.)	R_F_ CV%	Resolution(+/- St. Dev.)
8-THC	0.390 (+/- 0.014)	3.47	
9-THC	0.336 (+/- 0.017)	5.09	0.394 (+/- 0.085)
CBD	0.305 (+/- 0.012)	3.85	0.092 (+/- 0.041)
THCV	0.297 (+/- 0.013)	4.39	0.330 (+/- 0.021)
CBC	0.271 (+/- 0.015)	5.40	0.186 (+/- 0.082)
CBDV	0.257 (+/- 0.008)	3.12	0.029 (+/- 0.073)
CBN	0.254 (+/- 0.013)	5.06	1.432 (+/- 0.128)
CBG	0.151 (+/- 0.008)	5.42	1.797 (+/- 0.146)
THCA-A	0.054 (+/- 0.001)	2.13	0.383 (+/- 0.039)
CBDA	0.038 (+/- 0.001)	3.01	0.347 (+/- 0.035)
CBGA	0.024 (+/- 0.003)	11.86	1.250 (+/- 0.140)

S2, S3 shows R_F_ values generated from original reports after each run, where raw and calculated R_F_ values such as average, standard deviation, CV% and resolution of each mobile phase can be found.

**Fig. 4:**
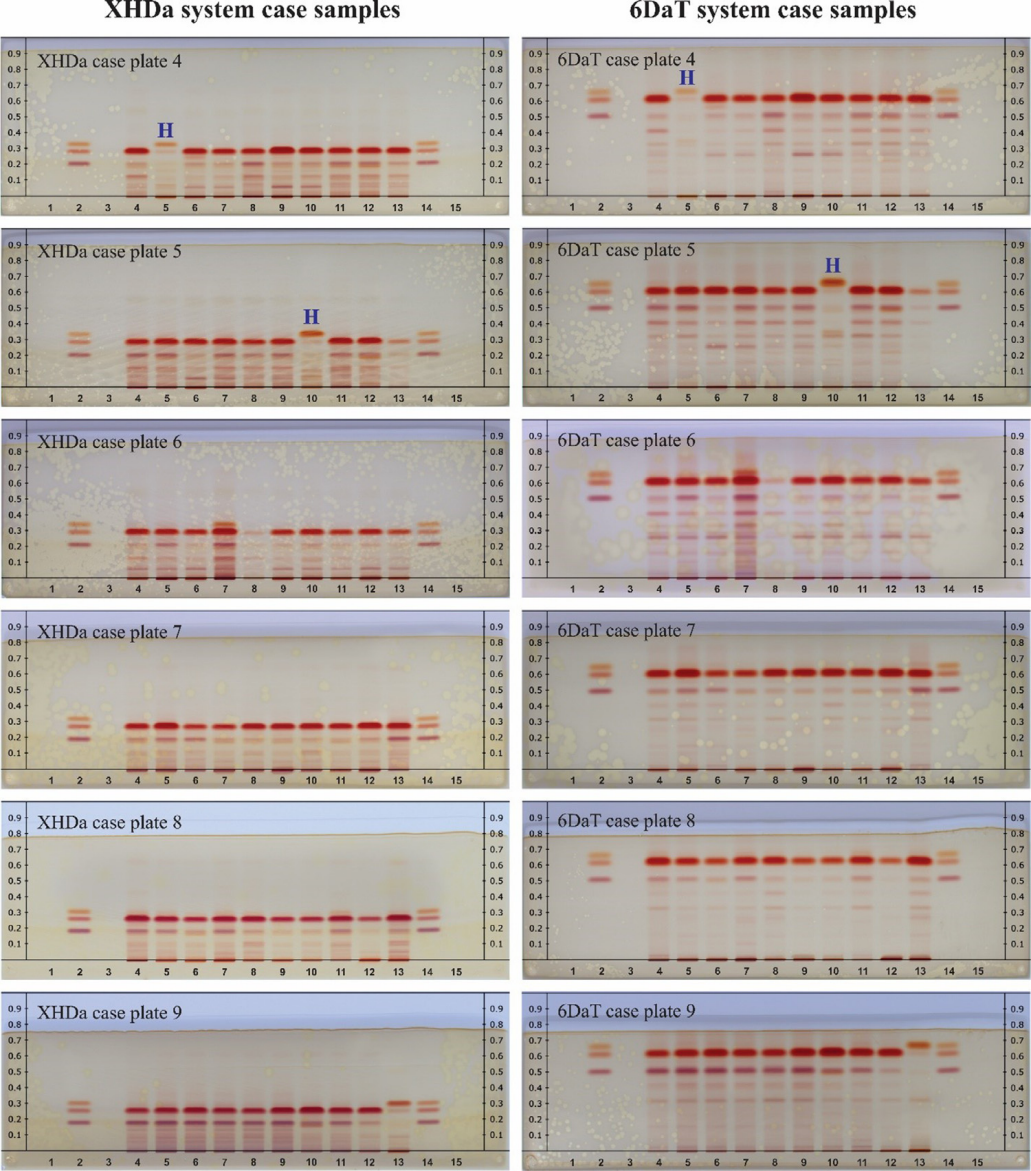
Images of HPTLC plates of case samples analyzed on two mobile systems, XHDa (left column) and 6DaT (right column). Y-axis denotes R_F_ value. Lane 1 and 15: methanol blank, lane 2 and 14: mixture of CBD (Top), Δ9-THC (middle), and CBN (bottom). Lane 3: tetracosane internal standard. Lane 4–13: case samples. “H” denotes hemp sample. Application volume for all lanes is 2µL.

A total of 89 samples of suspected cannabis products (77 vegetation, 4 edible gummies, 4 e-cigarettes) were obtained from two crime laboratories and prepared as described previously [2]. The first three case sample plates from both xylene:hexane:diethylamine (25:10:1) (XHDa) and 6% diethylamine:toluene (6DaT) mobile phase systems, as well as Pennsylvania State Police case samples were shown in the previous article [2]. Here we report the rest of the case sample plate images on both mobile phase systems (Fig. 4). Results of all of the case samples by HPTLC can be found in the supplemental material (S4).

Fig. 4 shows case sample images on both XHDa (left) and 6DaT (right) mobile phase systems. Methanol blanks, reference standards, and internal standards were included on the plates. Lane 4–13 were all vegetation samples. Hemp samples that were confirmed by GC–MS were marked with purple letter H in the figure.

S4 includes all original HPTLC reports for all 89 case samples in both mobile phase systems. Each file includes application format, instrument parameters, images of each plate and R_F_ values.

## 2 Experimental design, materials, and methods

### 2.1 Cannabinoid standards and sample preparation

Working solutions (100 µg/mL) of 11 cannabinoid standards were prepared from the 1-mg/mL certified standard solutions acquired from Cerilliant Corporation by diluting with LC-grade methanol (VWR, Radnor, PA, USA). A mixture of all 11 cannabinoids was prepared by combining a 136 *µ*L aliquot of each standard for a final concentration of 91 *µ*g/mL for each cannabinoid. The 11 cannabinoid standards were analyzed in triplicate by HPTLC with ten mobile phase systems. These same cannabinoid standards were then analyzed an additional seven times on two of the most optimal mobile phase systems, 6DaT and XHDa to validate these systems for case analysis of seized cannabis samples.

A total of 89 samples of suspected cannabis products (77 vegetation, 4 edible gummies, 4 e-cigarettes) were obtained from two crime laboratories and prepared as described in a previous publication [2]. These samples were prepared by personnel at the crime laboratories for analysis by GC–MS. Case samples in this article were all vegetation samples from Ocean County Sheriff's Department in Toms River, New Jersey. To prepare the sample 500 mg of cannabis sample was dried at 60 °C overnight before it was ground. Sample was ground and 100 mg of ground sample was then extracted with 50 mL of chloroform, evaporated to dryness, and reconstituted with 5 mL of 0.2% (w/v) tetracosane in methanol. The extracts were analyzed as received and applied to the HPTLC plates. Case sample application format can be found in (S10) in a previous publication [2].

### 2.2 Methods

All HPTLC plate runs were carried out using the following experimental procedure. A clean plate was put into the TLC visualizer for background documentation purposes prior to each run. Methanol blanks, standard working solutions or case samples were then applied on the plate either in the form of bands (20 × 10 cm plate) or dots (10 × 10 cm plate) using CAMAG automatic TLC sampler 4. The plate was then transferred to the CAMAG automatic development chamber-2 to develop with a selected mobile phase. During development, the plate was first dried for 30 s. A glass chamber underwent a 20 min humidity control and tank saturation step before the plate was lowered into the chamber. Migration distance was set at 70 mm. Once the migration distance was reached, the plate was removed from the mobile phase and dried for five minutes. The plate was then transferred to the CAMAG TLC visualizer for documentation under white light at 254 nm and 366 nm wavelengths. A background image was taken at the beginning of the run which was automatically subtracted by the software. The plate was transferred to the TLC scanner 3 for an intensity scan of samples on the plate after development. The plate was then dipped into a 5% Fast Blue B solution for derivatizing purposes using the CAMAG Chromatogram Immersion Device III. The dipping speed was set to 3 with a dipping time of 5 s. After derivatization, the plate was transferred to the visualizer for the 3rd set of pictures, which were the final images reported in this article.

## Declaration of Competing Interest

The authors declare that they have no known competing financial interests or personal relationships which have, or could be perceived to have, influenced the work reported in this article.

## Appendix Supplementary materials

Image, application 1
