# Supplementary material for: High Performance Thin-Layer Chromatography (HPTLC) data of Cannabinoids in ten mobile phase systems
Source: Data Brief. 2020 Jun 30;31:105955. doi: 10.1016/j.dib.2020.105955 (PMC7352075; doi:10.1016/j.dib.2020.105955)
Supplement: Supplementary file 1 [file mmc1.zip › S1-Triplicate reports/Chloroform-3.pdf]

Analysis: Chloroform-3

Path: Home/YL Research

Based on method: Triplets Method

Created	26-May-2019 17:44:45	visionCATSuser
Modified	04-Jun-2019 14:11:35	visionCATSuser
Last HPTLC log	04-Jun-2019 14:11:35	Analysis modified
Explorer notes		

Track	Vial ID	Description	Volume	Position	Type
1	MeOH blank	MeOH Blank	2.0 µl	A1	Sample
2	Mixture 100	Mixture 500ng	5.0 µl	B1	Sample
3	9-THC 100	D9-THC 500ng	5.0 µl	C1	Reference
4	CBD 100	CBD 500ng	5.0 µl	D1	Reference
5	CBN 100	CBN 500ng	5.0 µl	E1	Reference
6	CBG 100	CBG 500ng	5.0 µl	F1	Reference
7	CBC 100	CBC 500ng	5.0 µl	A2	Reference
8	THCV 100	THCV 500ng	5.0 µl	B2	Reference
9	CBDV 100	CBDV 500ng	5.0 µl	C2	Reference
10	8-THC 100	D8-THC 500ng	5.0 µl	D2	Reference
11	THCA-A 100	THCA-A 500ng	5.0 µl	E2	Reference
12	CBDA 100	CBDA 500ng	5.0 µl	F2	Reference
13	CBGA 100	CBGA 500ng	5.0 µl	A3	Reference
14	Mixture 100	Mixture 500ng	5.0 µl	B1	Sample
15	MeOH blank	MeOH Blank	2.0 µl	A1	Sample
Sequence table notes					

A track marked with ⚠ means: the application type is overridden in some evaluation(s).

System setup:

Software	Server User-PC, version 2.5.18072.1
ATS4	S/N:080713
Chamber	N/A
Derivatization dip	N/A
Scanner3	S/N:031025
Visualizer	S/N:230515

Chromatography

Plate layout:

Stationary phase	Merck, HPTLC plates silica gel 60 F 254
Plate format	200.0 x 100.0 mm
Application type	User
Application	Position Y: 10.0 mm, length: 8.0 mm, width: 0.0 mm
Track	First position X: 20.0 mm, distance: 11.4 mm
Solvent front position	70.0 mm
Notes	

Take image clean plate 1a - Visualizer (S/N: 230515):

Quality	Enhanced
RT White	auto capture, Auto, level 85 %, Band
R 254	auto capture, Auto, level 85 %, Band
Instrument diagnostics	Valid diagnostics
Documentation step label	
Notes	

Application 1 - ATS 4 (S/N: 080713):

Spray gas	NI
Sample solvent type	Methanol
Filling speed	15 µl/s
Predosage volume	200 nl
Retraction volume	200 nl
Dosage speed	150 nl/s
Filling quality	User
Rinsing cycles / vacuum	1 / 4 s
Filling cycles / vacuum	1 / 4 s
Rinsing solvent name	Methanol
Nozzle temperature	Unheated
Rack in use	Standard
Instrument diagnostics	Valid diagnostics
Notes	

Development 1 - Chamber:

Tank	TTC 20x10
Mobile phase	
Saturation time	20 min
Use saturation pad	true
Use smartALERT	false
Volume front through	10 ml
Volume rear through	20 ml
Drying time	5 min
Drying temperature	Room temperature
Notes	

Take image developed plate 1a - Visualizer (S/N: 230515):

Quality	Enhanced
RT White	auto capture, Auto, level 85 %, Band
R 254	auto capture, Auto, level 85 %, Band
R 366	auto capture, Auto, level 85 %, Band
Instrument diagnostics	Valid diagnostics
Documentation step label	
Notes	

Scan developed plate 1b - Scanner 3 (S/N: 031025):

Scanner type	Single λ
Optimization for	Resolution
Measurement mode	Absorption
Filter	n/a
Detector mode	Automatic
Scanning speed	20 mm/s
Data resolution	100 µm/step
Slit	5 x 0.2 mm, micro
Partial scan	No
Lamp	Deuterium & Tungsten
Wavelength(s)	254 nm
Instrument diagnostics	Valid diagnostics
Documentation step label	
Notes	

Derivatization 1 - dip:

Reagent name	Fast Blue B salt
Dipping speed	3
Dipping time	5 s
Reagent preparation	1g Fast Blue B salt in 200mL water
Heating	none
Notes	Air dry for 5 minutes

Take image derivatized plate 1a - Visualizer (S/N: 230515):

Quality	Enhanced
RT White	auto capture, Auto, level 85 %, Band
R 366	auto capture, Auto, level 85 %, Band
Instrument diagnostics	Valid diagnostics
Documentation step label	
Notes	

System suitability tests:

SST settings:

SST tracks	

Data acquisition

Application 1 - ATS 4 (S/N: 080713):

Executed	26-May-2019 18:19:45 visionCATSuser

Development 1 - Chamber:

Executed	26-May-2019 18:46:26 visionCATSuser

Take image developed plate 1a - Visualizer (S/N: 230515):

Executed	26-May-2019 19:43:59 visionCATSuser

RT White

Developed, RemTransVis

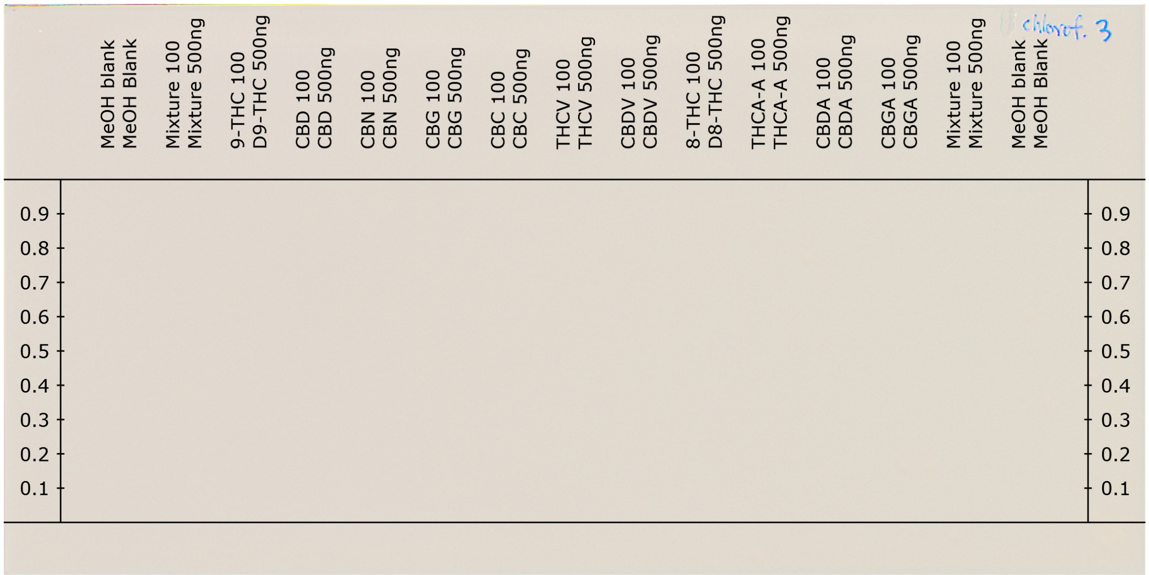

Exposure	0.087 s
Contrast	1
Normalized exposure	Disabled
Clarify	Disabled
White balance	1.00, 1.00, 1.00

R 254

Developed, Remission254

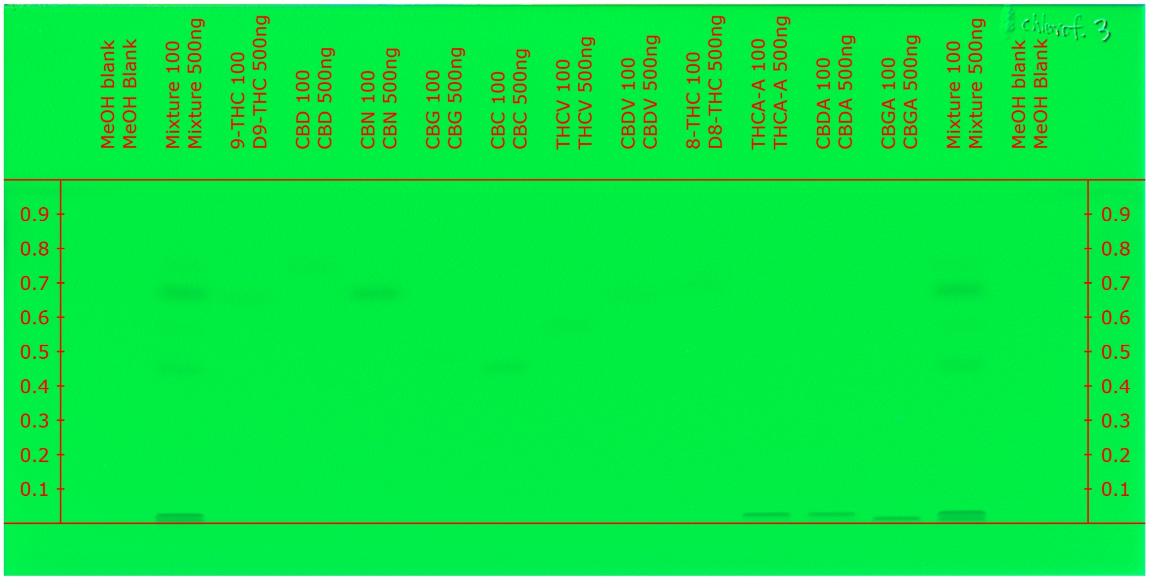

Exposure	0.287 s
Contrast	1
Normalized exposure	Disabled
Clarify	Disabled
White balance	1.00, 1.00, 1.00

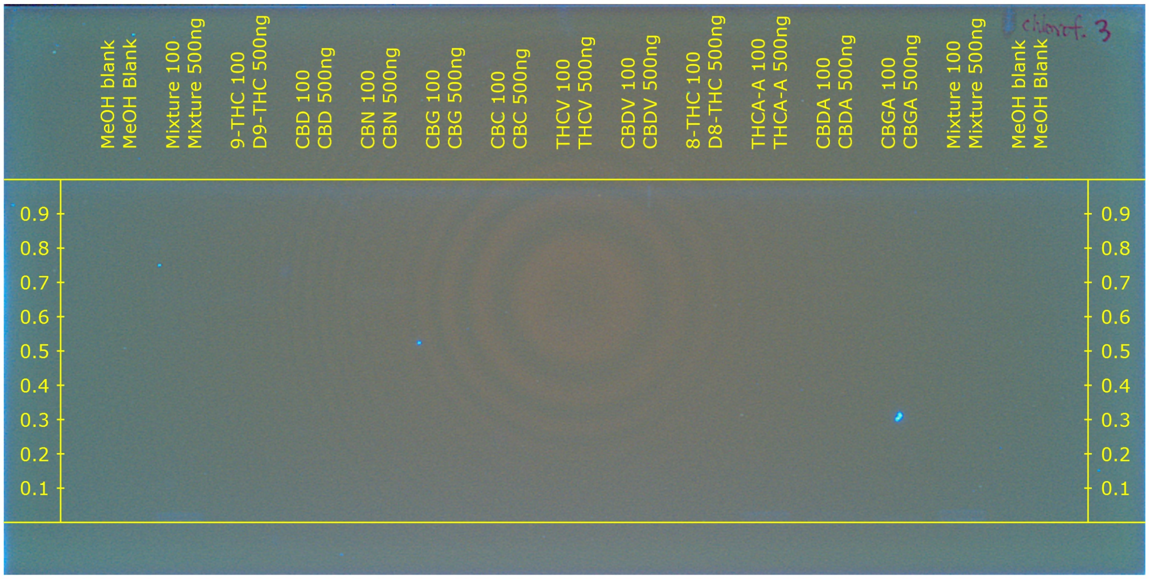

Exposure	9.999 s
Contrast	1
Normalized exposure	Disabled
Clarify	Disabled
White balance	1.00, 1.00, 1.00

Scan developed plate 1b - Scanner 3 (S/N: 031025):

Executed	26-May-2019 19:47:40 visionCATSuser

Scan:

Wavelength	254 nm

Track 1:

Type	Single λ

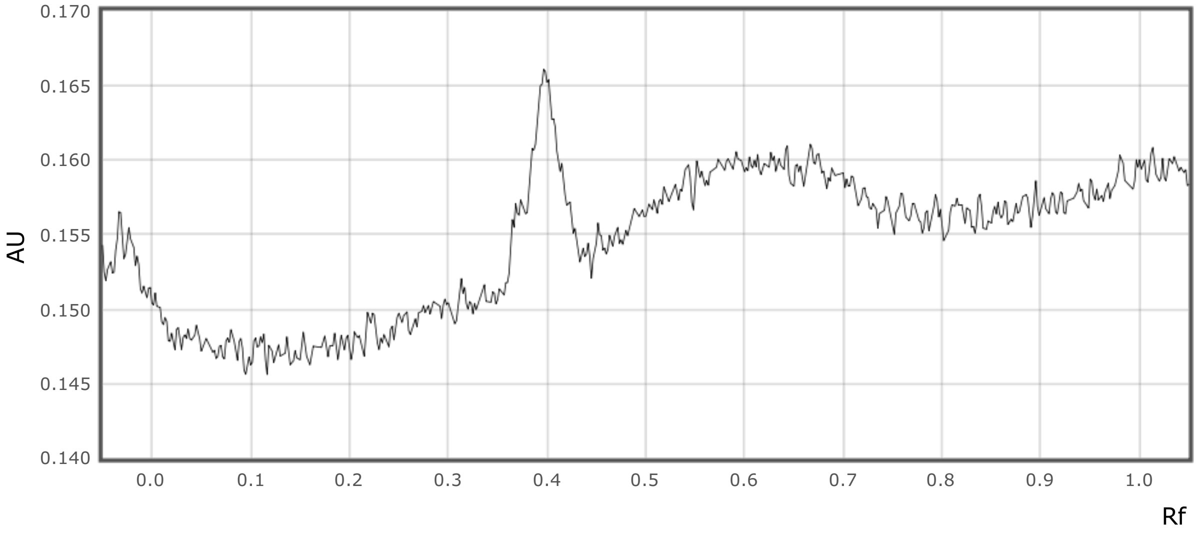

Track 2:

Type	Single λ

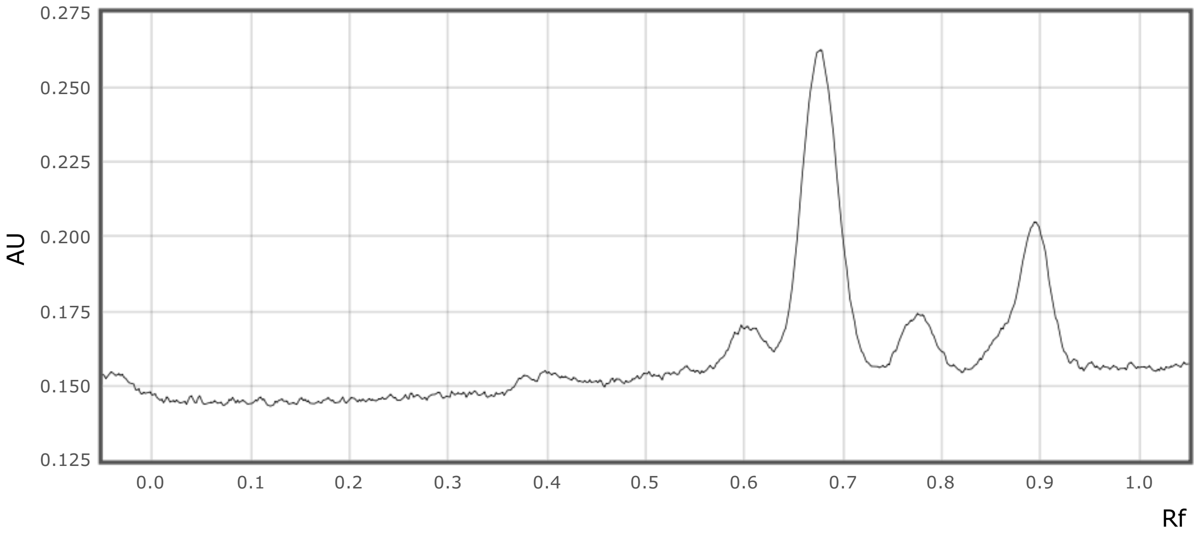

Track 3:

Type	Single λ

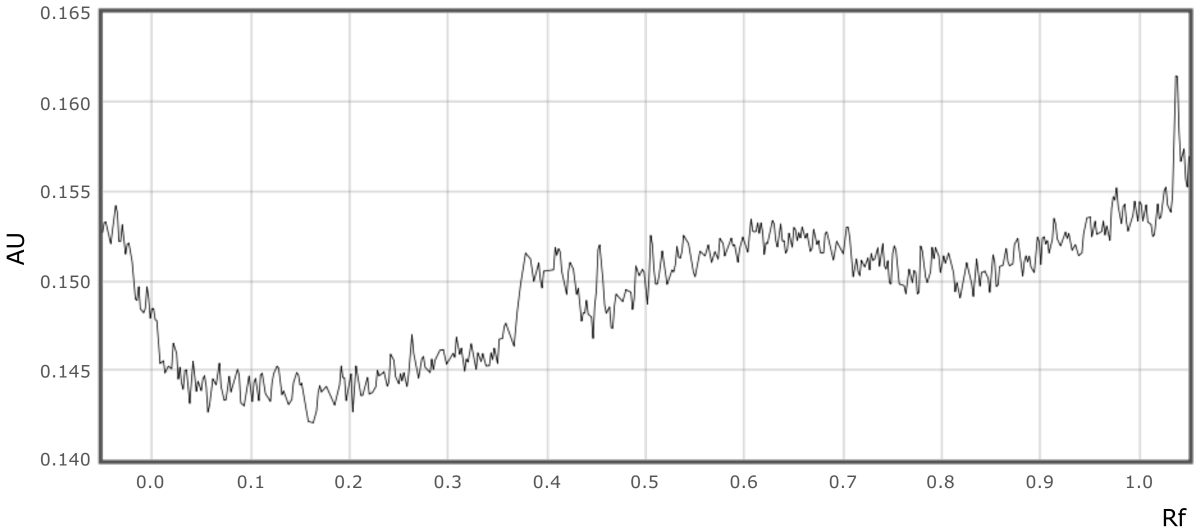

Track 4:

Type	Single λ

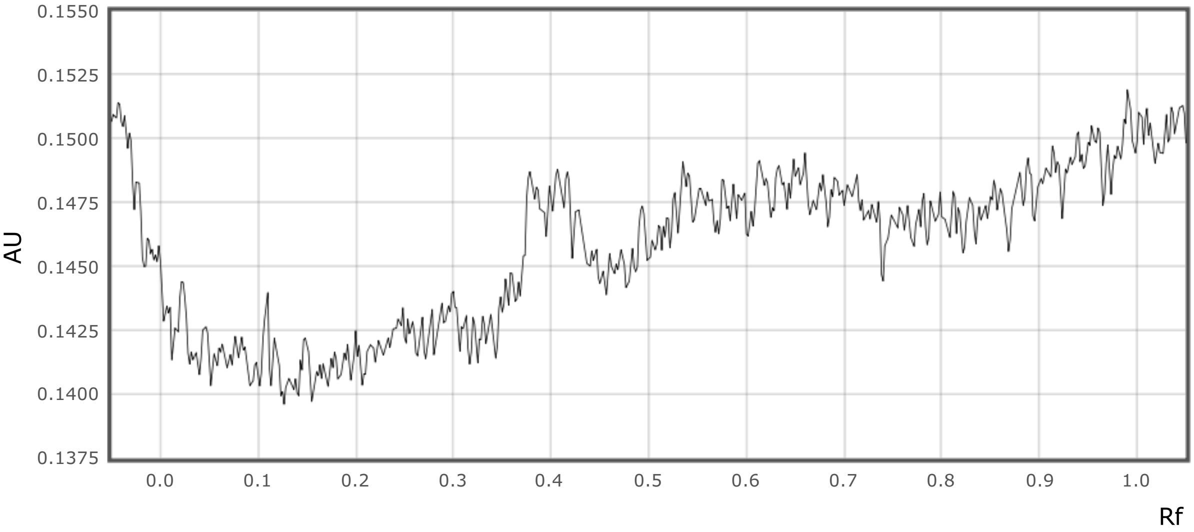

Track 5:	
Type	Single λ

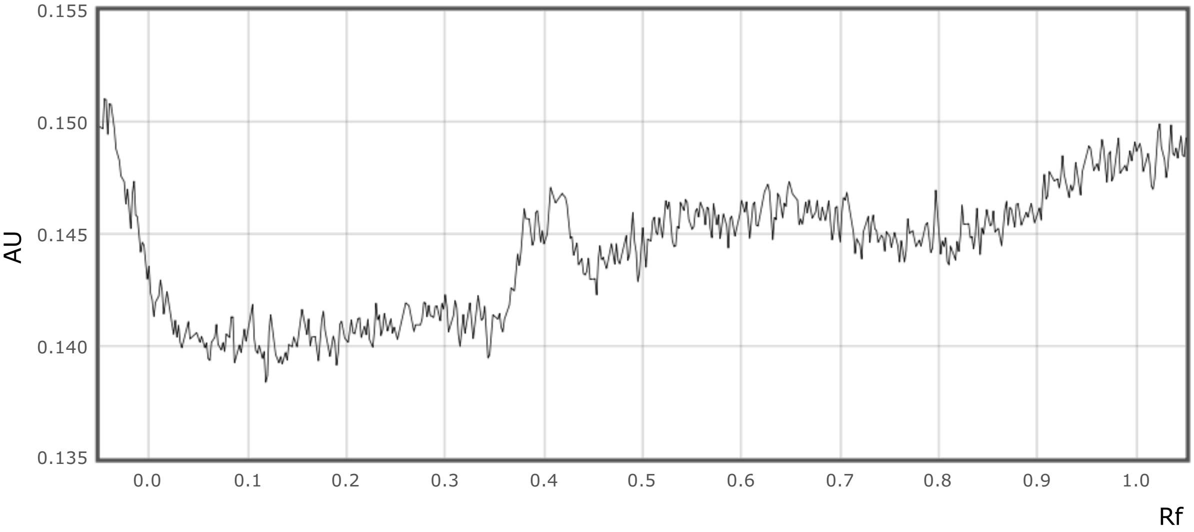

Track 6:	
Type	Single λ

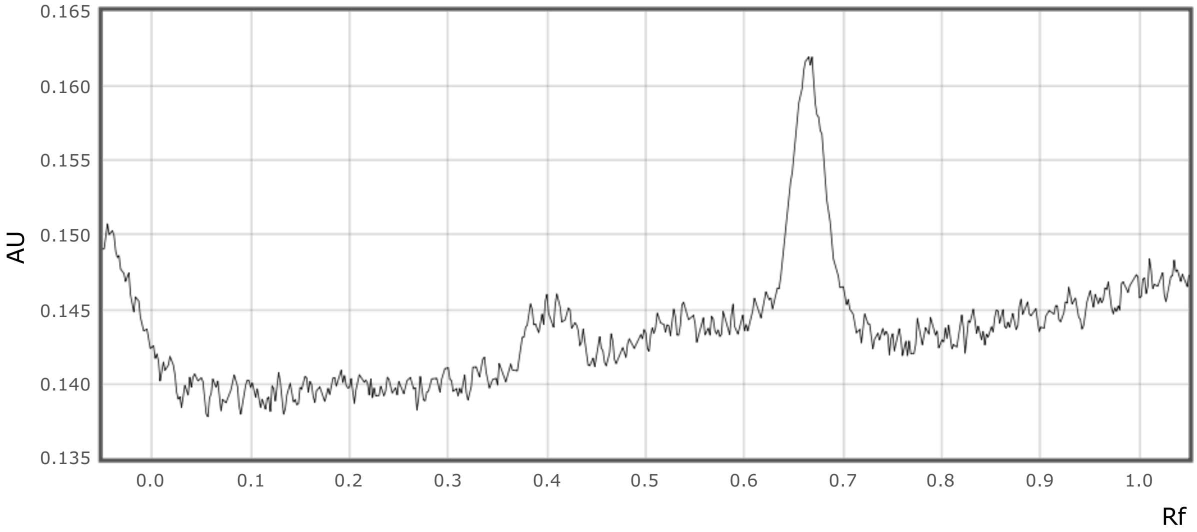

Track 7:

Type	Single λ

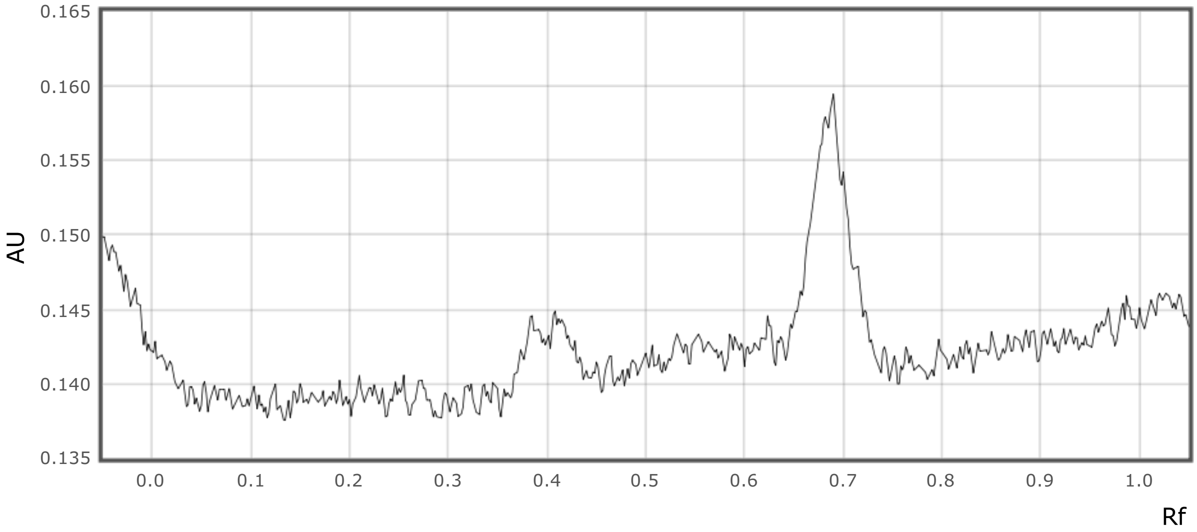

Track 8:

Type	Single λ

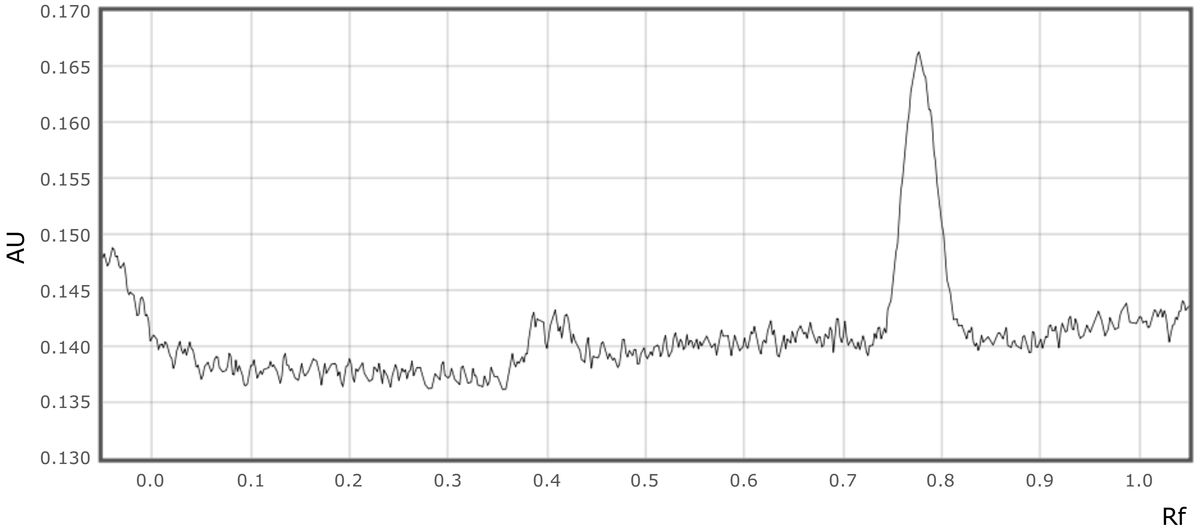

Track 9:

Type	Single λ

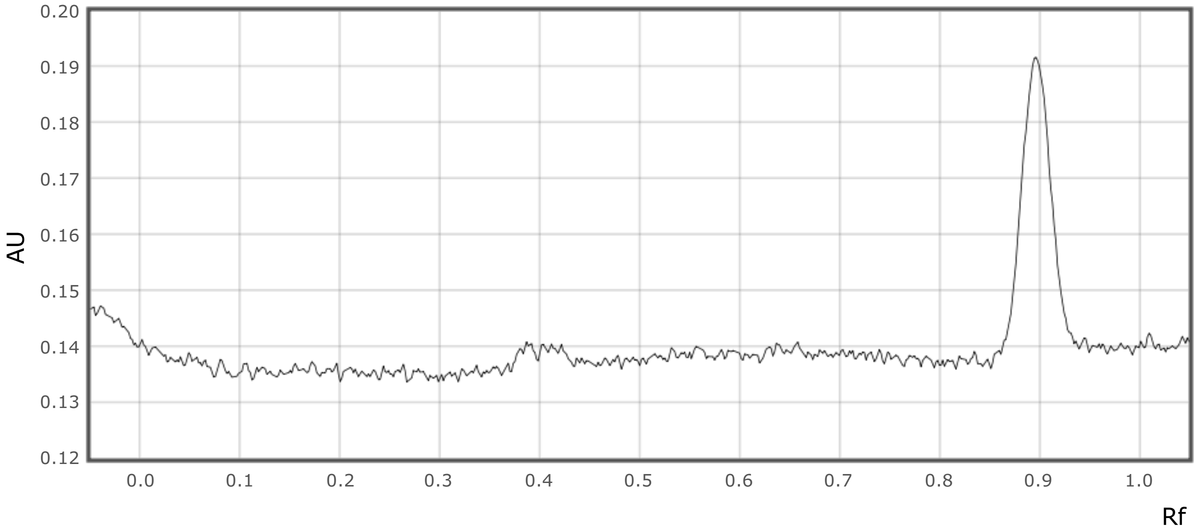

Track 10:

Type	Single λ

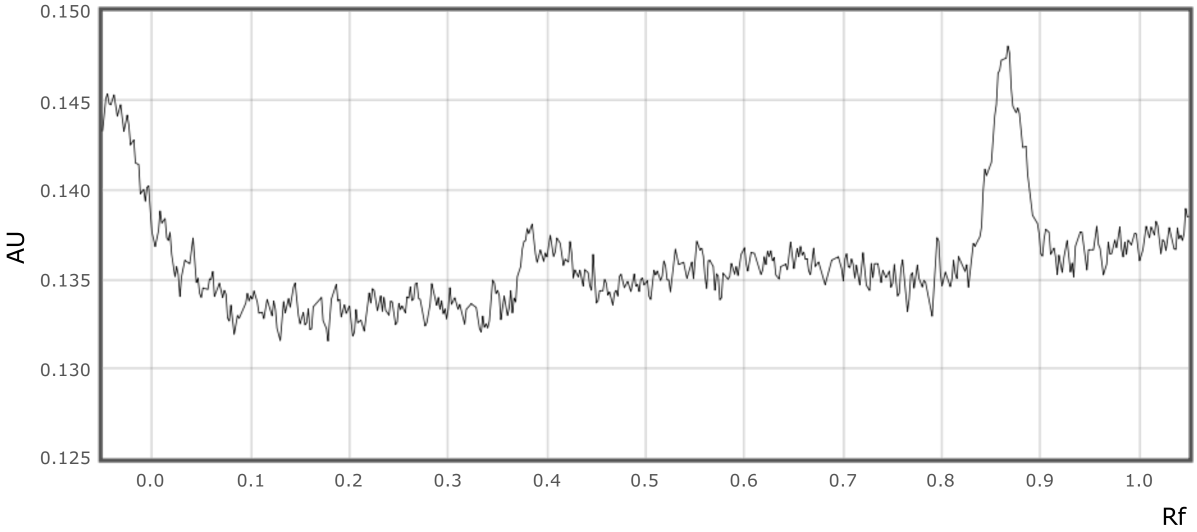

Track 11:	
Type	Single λ

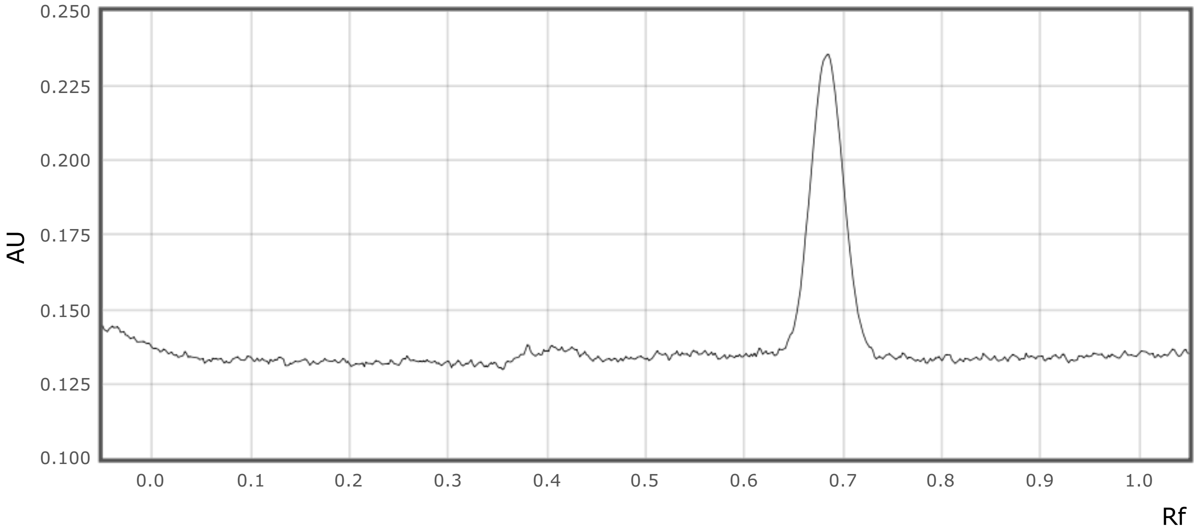

Track 12:	
Type	Single λ

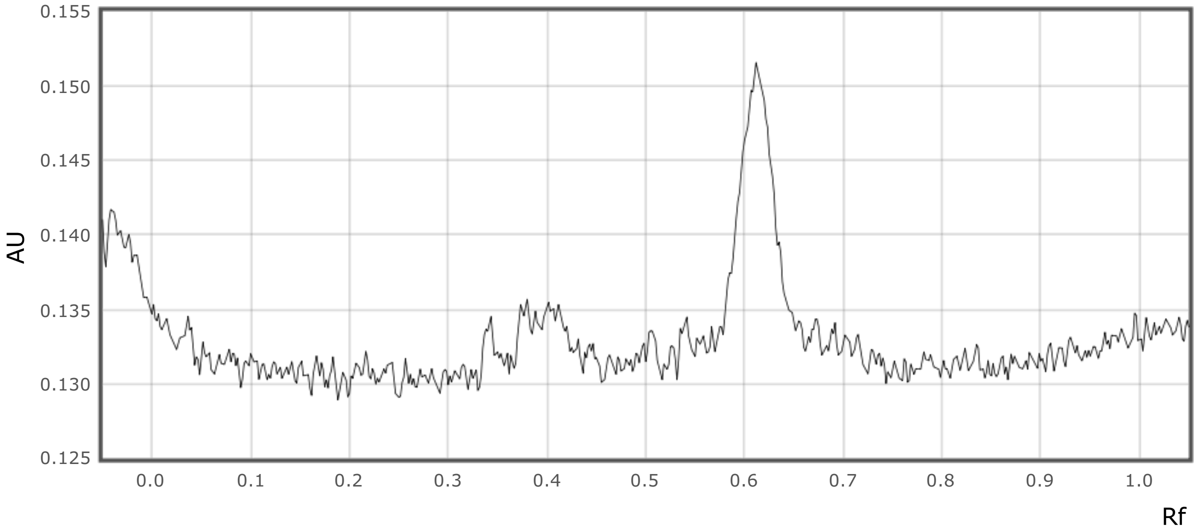

Track 13:	
Type	Single λ

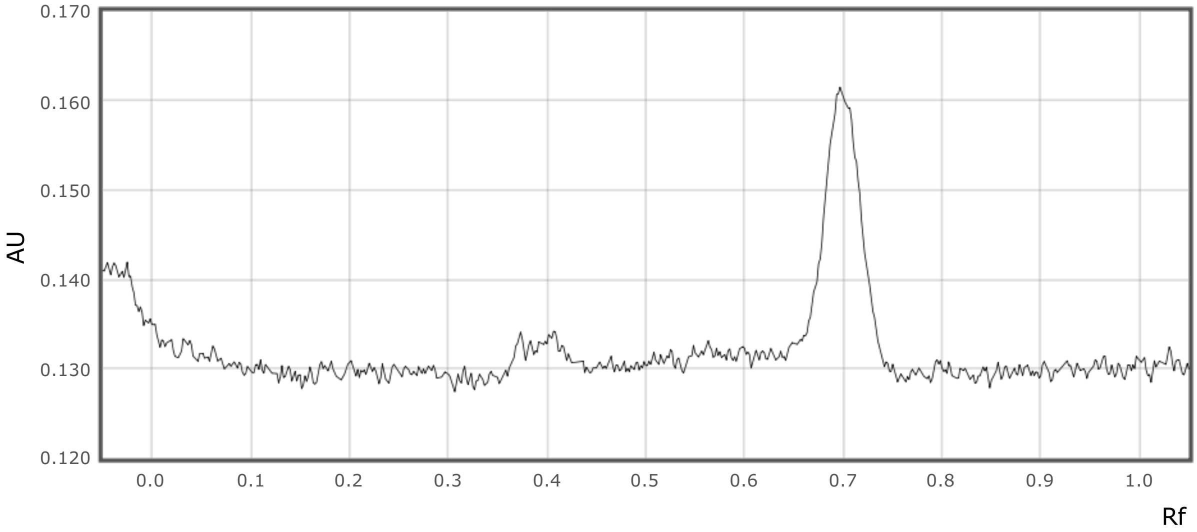

Track 14:	
Type	Single λ

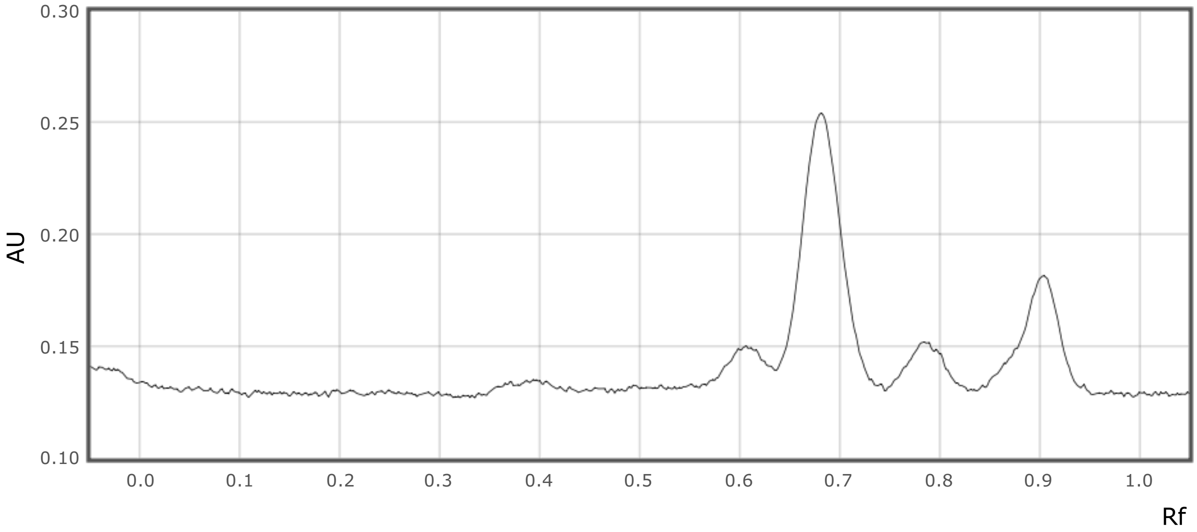

Track 15:	
Type	Single λ

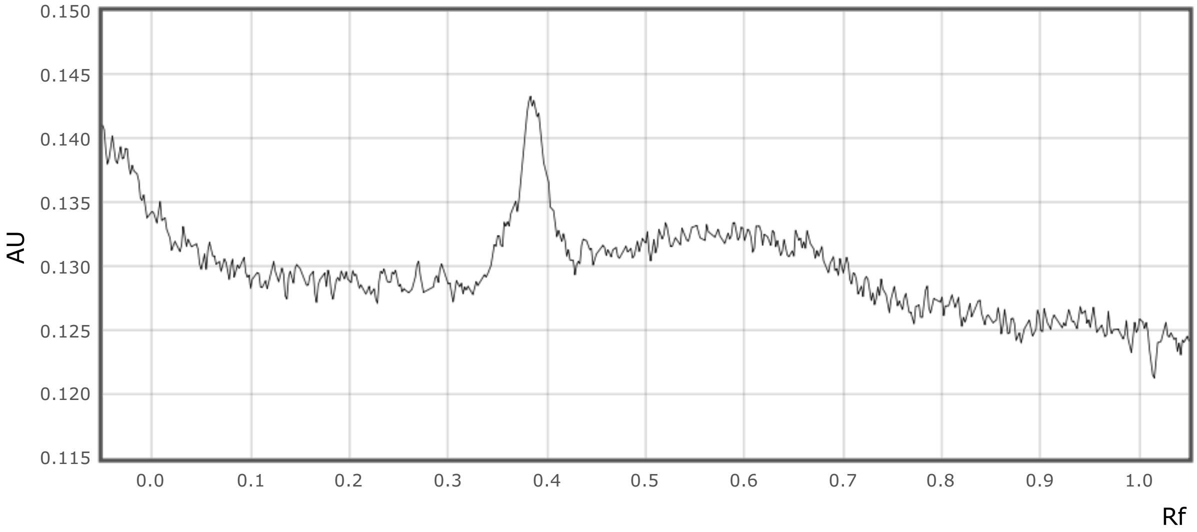

Derivatization 1 - dip:

Executed	26-May-2019 19:49:52 visionCATSuser

Take image derivatized plate 1a - Visualizer (S/N: 230515):

Executed	26-May-2019 19:53:07 visionCATSuser

World Leader in Planar Chromatography

Chloroform-3 | visionCATS

RT White | Derivatized, RemTransVis

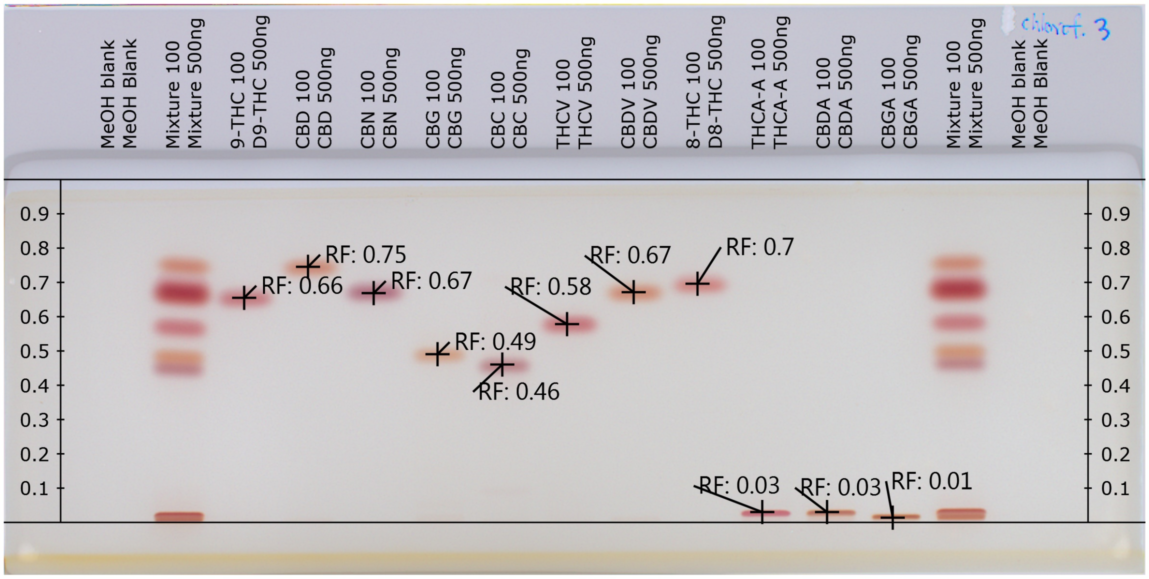

Exposure	0.059 s
Contrast	1
Normalized exposure	Disabled
Clarify	Disabled
White balance	1.10, 1.05, 0.88

R 366 | Derivatized, Remission366

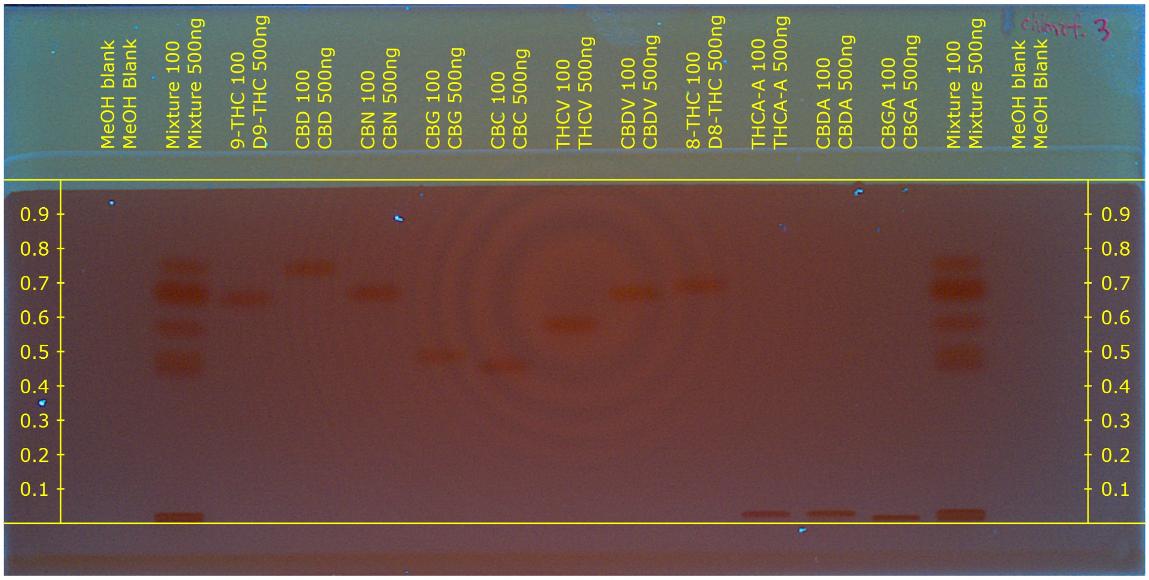

Exposure	9.999 s
Contrast	1
Normalized exposure	Disabled
Clarify	Disabled
White balance	1.00, 1.00, 1.00

Evaluation 1 :

Validated	false
Step	Take image derivatized plate 1a
Concentration unit type	Mass / volume
Notes	

Definition:

References:

9-THC 100

Substance Name	Concentration	Purity
9-THC	100.000 µg/ml	100.00 %

CBD 100

Substance Name	Concentration	Purity
CBD	100.000 µg/ml	100.00 %

CBN 100

Substance Name	Concentration	Purity
CBN	100.000 µg/ml	100.00 %

CBG 100

Substance Name	Concentration	Purity
CBG	100.000 µg/ml	100.00 %

CBC 100

Substance Name	Concentration	Purity
CBC	100.000 µg/ml	100.00 %

THCV 100

Substance Name	Concentration	Purity
THCV	100.000 µg/ml	100.00 %

CBDV 100

Substance Name	Concentration	Purity
CBDV	100.000 µg/ml	100.00 %

8-THC 100

Substance Name	Concentration	Purity
8-THC	100.000 µg/ml	100.00 %

THCA-A 100

Substance Name	Concentration	Purity
THCA-A	100.000 µg/ml	100.00 %

CBDA 100

Substance Name	Concentration	Purity
CBDA	100.000 µg/ml	100.00 %

CBGA 100

Substance Name	Concentration	Purity
CBGA	100.000 µg/ml	100.00 %

Samples:

Vial ID	Amount	Volume solution	Reference amount	Related to
MeOH blank		0.00 ml		
Mixture 100		0.00 ml		

Integration parameters:

Bounds	[0.000,1.000]
Smoothing	Savitzky–Golay of order 3 and window 7
Baseline correction	Lowest slope with noise 0.05
Profile subtraction	Profile subtraction from track 1
Peaks detection	Gauss (legacy) with sensitivity 0.1, separation 0.1 and threshold 0.1

Scan:

Wavelength	RT White

Track 1:

Type	Sample
Vial ID	MeOH blank
Description	MeOH Blank
Volume	2.0 µl

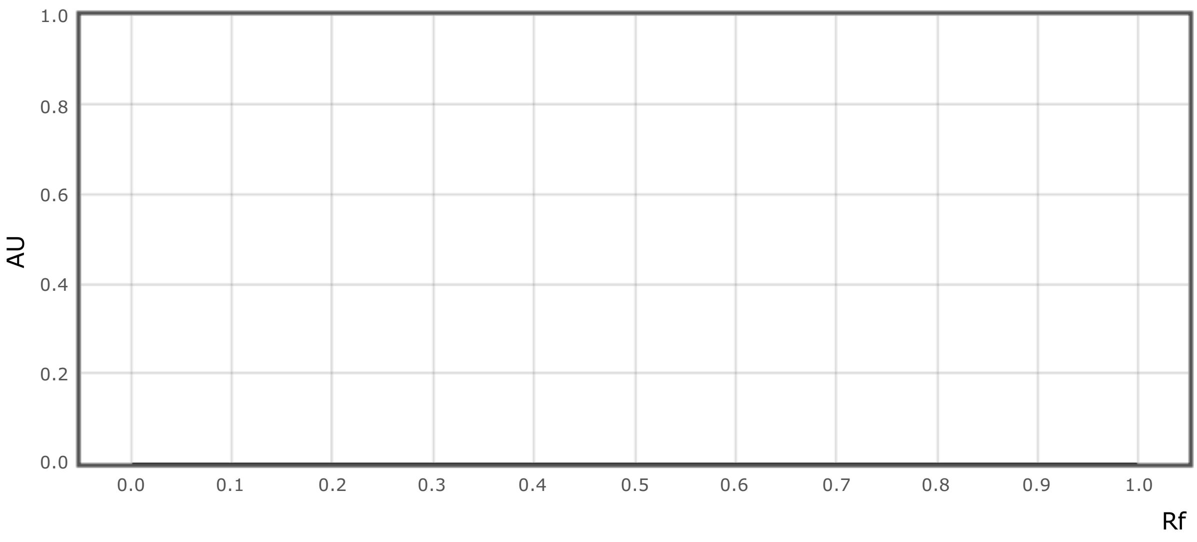

Peak #	Start		Max			End		Area		Manual peak	Substance Name
	Rf	H	Rf	H	%	Rf	H	A	%		

Track 2:

Type	Sample
Vial ID	Mixture 100
Description	Mixture 500ng
Volume	5.0 µl

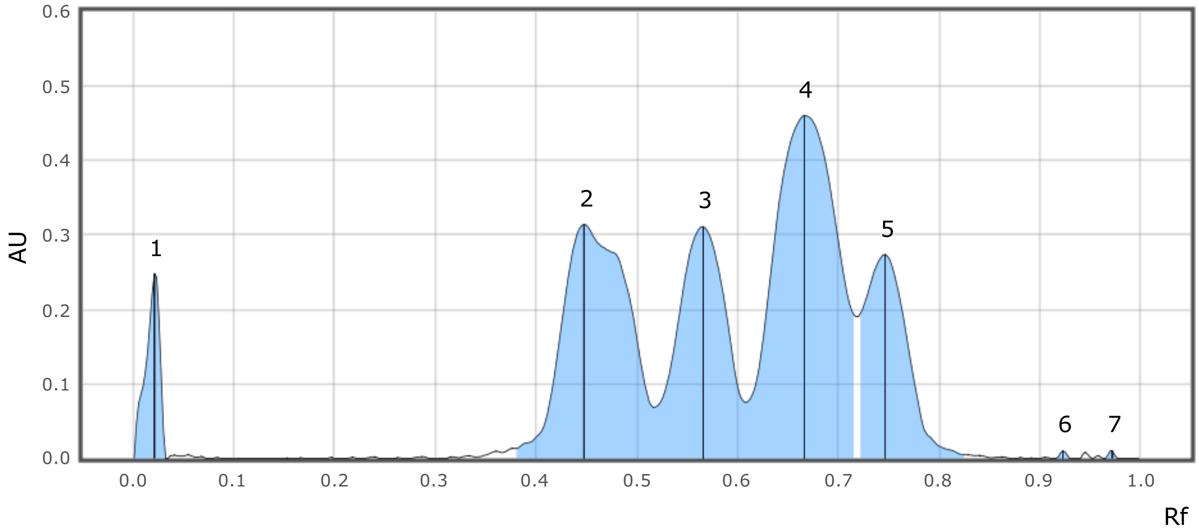

Peak #	Start		Max			End		Area		Manual peak	Substance Name
	Rf	H	Rf	H	%	Rf	H	A	%		
1	0.001	0.0000	0.021	0.2479	15.25	0.033	0.0000	0.00397	4.26	No	
2	0.378	0.0133	0.448	0.3137	19.29	0.517	0.0680	0.02404	25.78	No	
3	0.517	0.0680	0.566	0.3108	19.12	0.608	0.0749	0.01762	18.90	No	
4	0.608	0.0749	0.666	0.4600	28.29	0.718	0.1901	0.03390	36.36	No	
5	0.720	0.1901	0.747	0.2734	16.82	0.825	0.0047	0.01357	14.55	No	
6	0.916	0.0000	0.923	0.0101	0.62	0.930	0.0000	0.00007	0.08	No	
7	0.963	0.0000	0.972	0.0100	0.62	0.979	0.0000	0.00007	0.07	No	

Track 3:

Type	Reference
Vial ID	9-THC 100
Description	D9-THC 500ng
Volume	5.0 µl

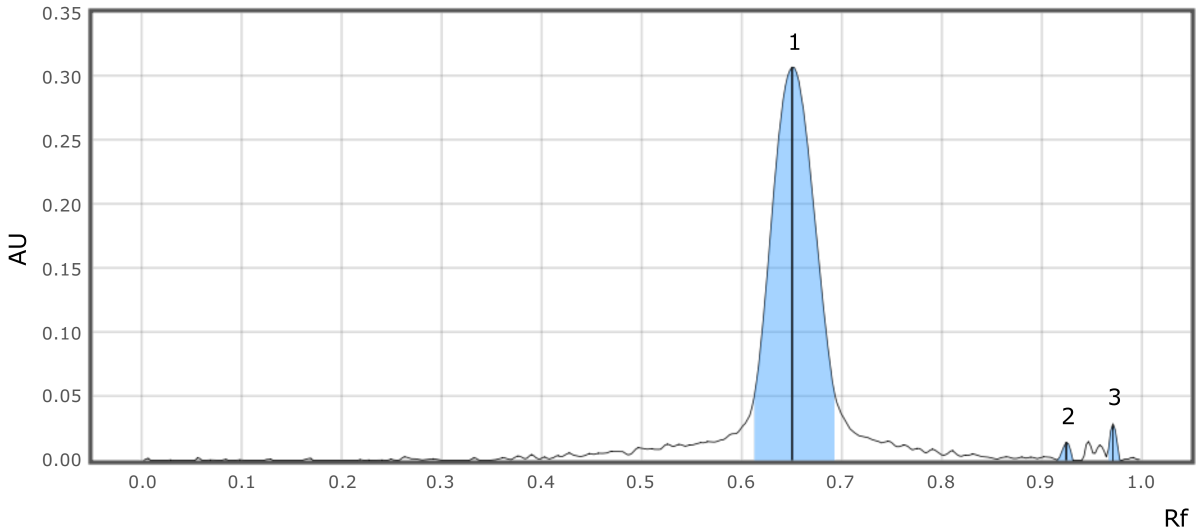

Peak #	Start Rf	Start H	Max Rf	Max H	Max %	End Rf	End H	Area A	Area %	Manual peak	Substance Name
1	0.612	0.0497	0.651	0.3072	87.83	0.694	0.0525	0.01562	97.87	Yes	9-THC
2	0.916	0.0002	0.925	0.0140	4.01	0.932	0.0000	0.00012	0.76	No	
3	0.965	0.0027	0.972	0.0286	8.16	0.981	0.0000	0.00022	1.37	No	

Track 4:

Type	Reference
Vial ID	CBD 100
Description	CBD 500ng
Volume	5.0 µl

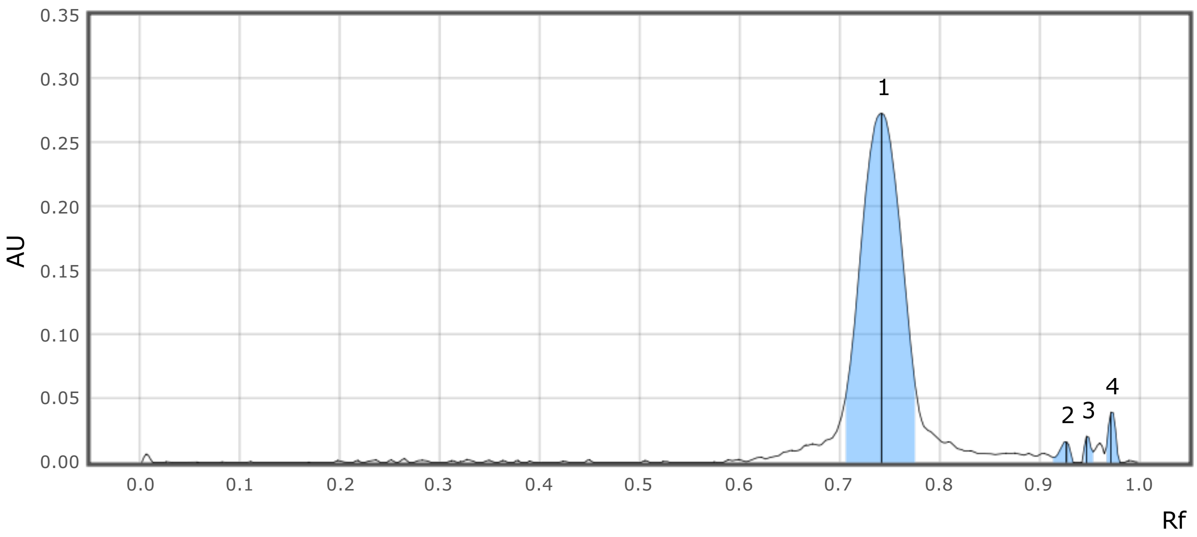

Peak #	Start Rf	Start H	Max Rf	Max H	Max %	End Rf	End H	Area A	Area %	Manual peak	Substance Name
1	0.705	0.0435	0.742	0.2732	78.27	0.780	0.0395	0.01304	94.98	Yes	CBD
2	0.914	0.0040	0.927	0.0161	4.61	0.934	0.0000	0.00019	1.41	No	
3	0.943	0.0000	0.947	0.0205	5.87	0.954	0.0081	0.00015	1.09	No	
4	0.965	0.0068	0.972	0.0393	11.26	0.981	0.0000	0.00035	2.52	No	

Track 5:

Type	Reference
Vial ID	CBN 100
Description	CBN 500ng
Volume	5.0 µl

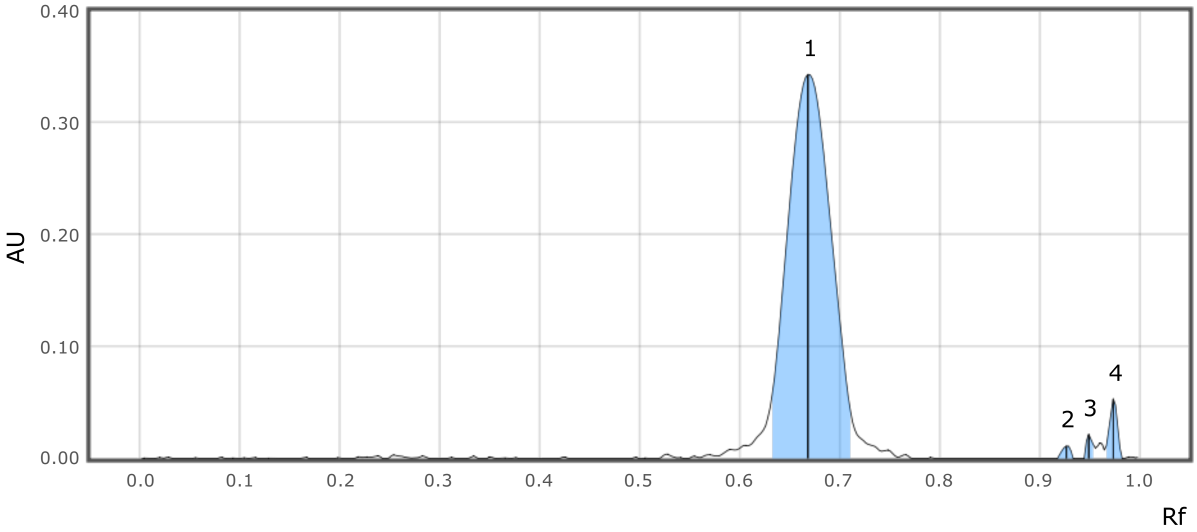

Peak #	Start		Max			End		Area		Manual peak	Substance Name
	Rf	H	Rf	H	%	Rf	H	A	%		
1	0.632	0.0452	0.668	0.3433	80.02	0.712	0.0430	0.01706	95.77	Yes	CBN
2	0.918	0.0000	0.927	0.0109	2.55	0.934	0.0000	0.00011	0.61	No	
3	0.945	0.0000	0.950	0.0215	5.01	0.956	0.0092	0.00016	0.90	No	
4	0.965	0.0075	0.974	0.0533	12.42	0.983	0.0000	0.00049	2.73	No	

Track 6:

Type	Reference
Vial ID	CBG 100
Description	CBG 500ng
Volume	5.0 µl

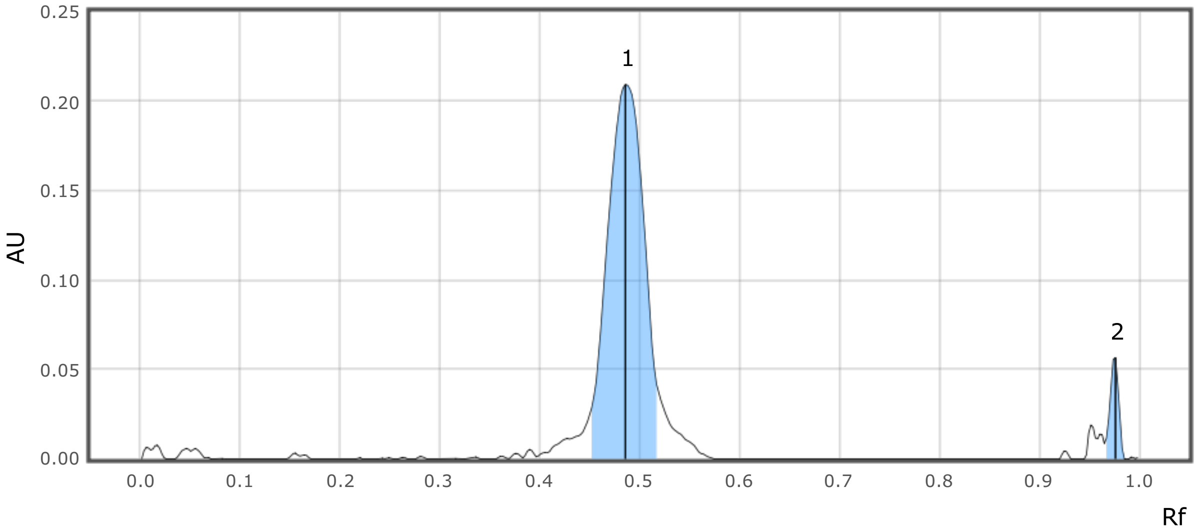

Peak #	Start		Max			End		Area		Manual peak	Substance Name
	Rf	H	Rf	H	%	Rf	H	A	%		
1	0.451	0.0248	0.485	0.2091	78.71	0.518	0.0418	0.00878	93.81	Yes	CBG
2	0.965	0.0086	0.976	0.0566	21.29	0.985	0.0000	0.00058	6.19	No	

Track 7:

Type	Reference
Vial ID	CBC 100
Description	CBC 500ng
Volume	5.0 µl

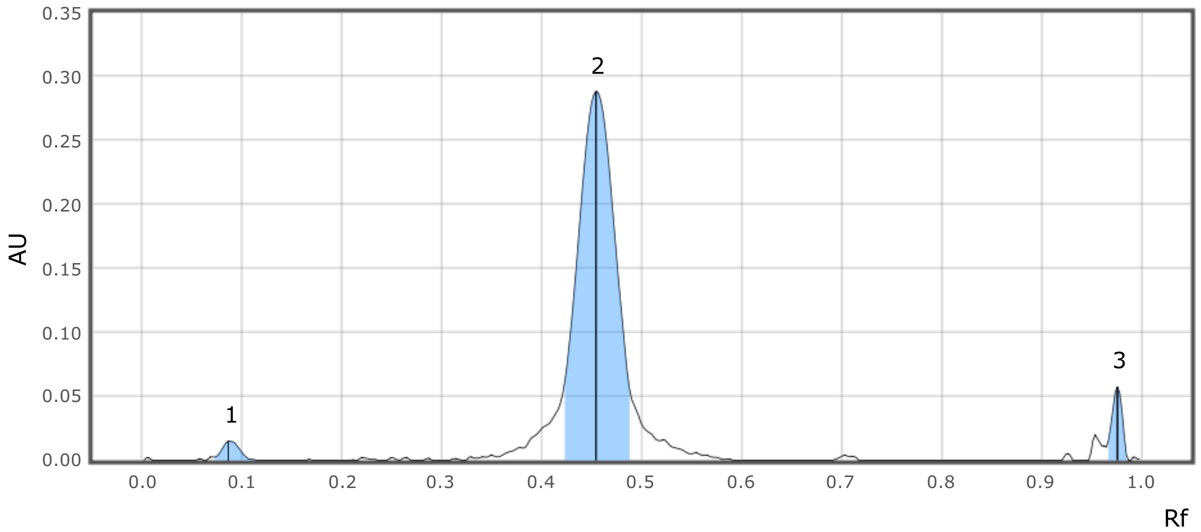

Peak	Start		Max			End		Area		Manual	Substance
#	Rf	H	Rf	H	%	Rf	H	A	%	peak	Name
1	0.073	0.0025	0.086	0.0150	4.15	0.115	0.0000	0.00031	2.31	No	
2	0.421	0.0515	0.454	0.2886	79.94	0.488	0.0556	0.01226	92.41	Yes	CBC
3	0.965	0.0103	0.976	0.0574	15.91	0.988	0.0000	0.00070	5.27	No	

Track 8:

Type	Reference
Vial ID	THCV 100
Description	THCV 500ng
Volume	5.0 µl

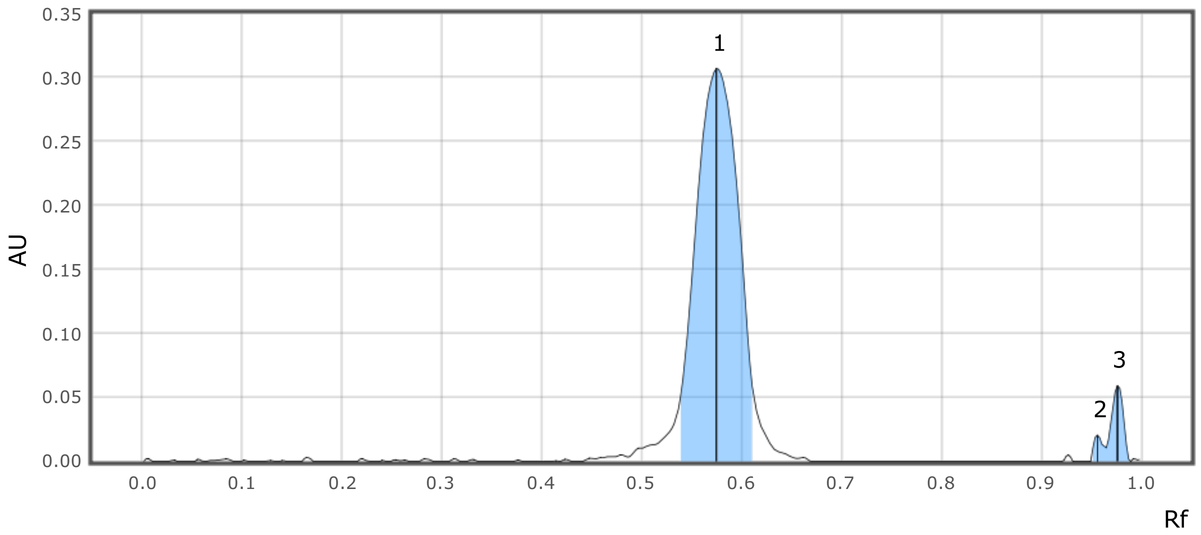

Peak #	Start Rf	Start H	Max Rf	Max H	Max %	End Rf	End H	Area A	Area %	Manual peak	Substance Name
1	0.538	0.0514	0.575	0.3069	79.42	0.614	0.0395	0.01504	93.68	Yes	THCV
2	0.950	0.0000	0.956	0.0205	5.31	0.965	0.0105	0.00022	1.36	No	
3	0.965	0.0105	0.976	0.0590	15.27	0.990	0.0000	0.00080	4.96	No	

Track 9:

Type	Reference
Vial ID	CBDV 100
Description	CBDV 500ng
Volume	5.0 µl

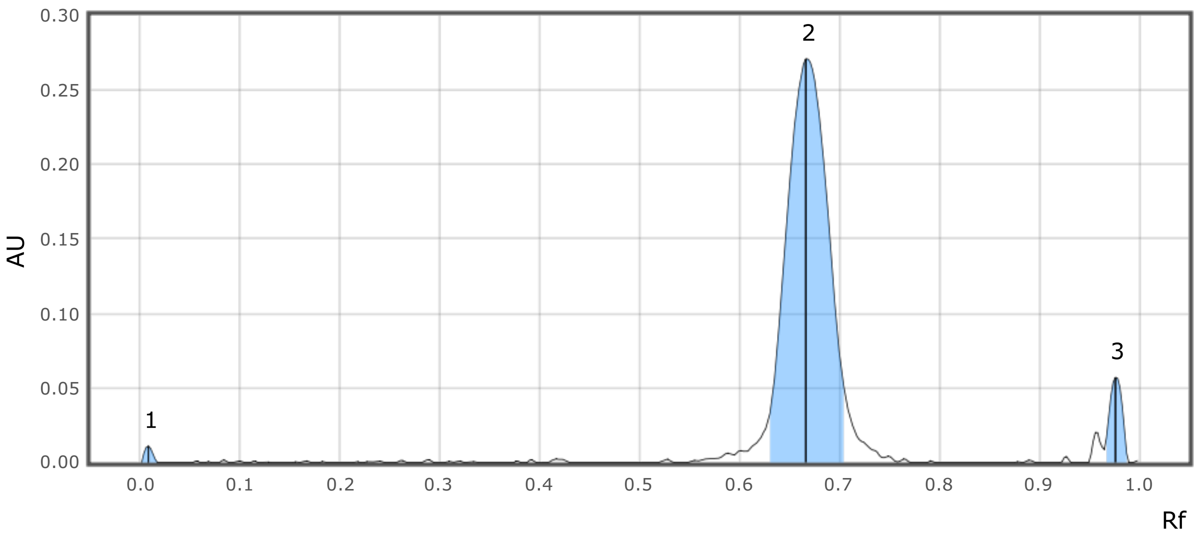

Peak #	Start Rf	Start H	Max Rf	Max H	Max %	End Rf	End H	Area A	Area %	Manual peak	Substance Name
1	0.001	0.0000	0.008	0.0109	3.22	0.017	0.0000	0.00010	0.72	No	
2	0.631	0.0330	0.666	0.2705	79.93	0.705	0.0514	0.01271	93.09	Yes	CBDV
3	0.965	0.0086	0.976	0.0570	16.85	0.990	0.0000	0.00084	6.19	No	

Track 10:

Type	Reference
Vial ID	8-THC 100
Description	D8-THC 500ng
Volume	5.0 µl

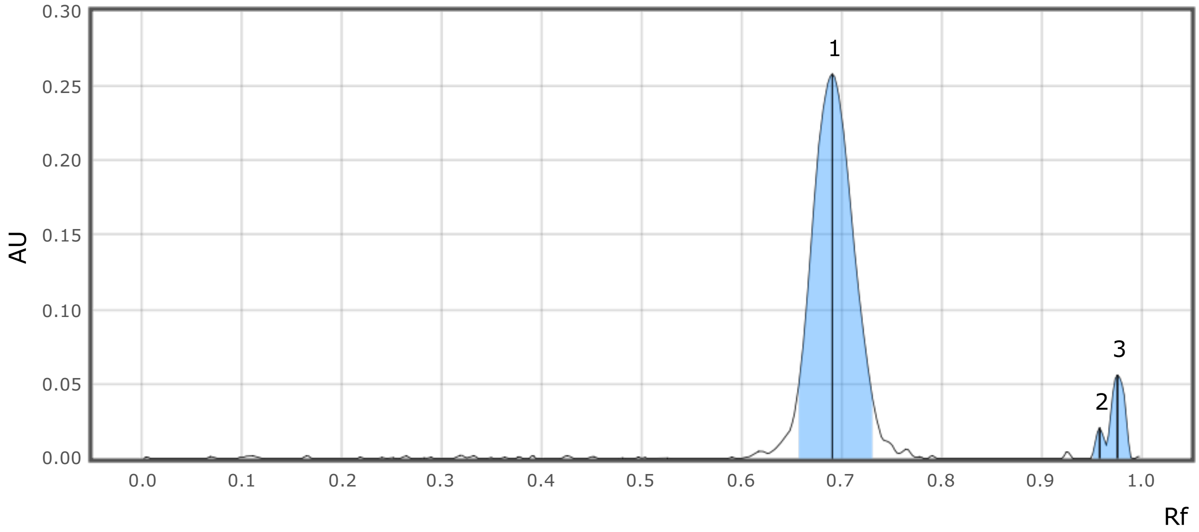

Peak #	Start		Max			End		Area		Manual peak	Substance Name
	Rf	H	Rf	H	%	Rf	H	A	%		
1	0.655	0.0392	0.691	0.2578	77.12	0.732	0.0329	0.01218	91.99	Yes	8-THC
2	0.950	0.0000	0.959	0.0205	6.14	0.965	0.0087	0.00019	1.47	No	
3	0.965	0.0087	0.976	0.0560	16.74	0.992	0.0000	0.00087	6.54	No	

Track 11:

Type	Reference
Vial ID	THCA-A 100
Description	THCA-A 500ng
Volume	5.0 µl

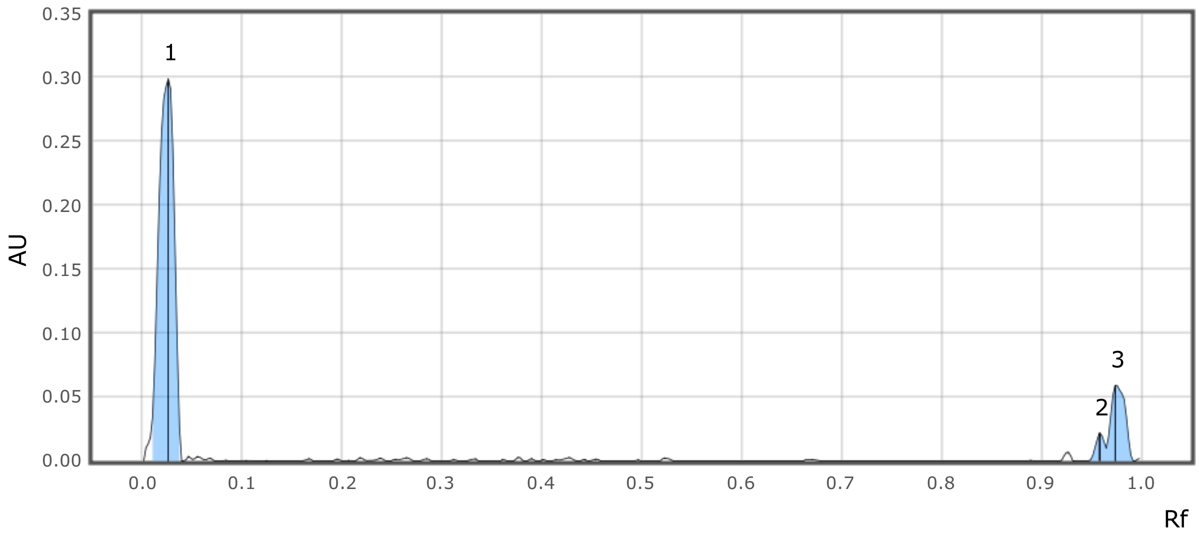

Peak #	Start		Max			End		Area		Manual peak	Substance Name
	Rf	H	Rf	H	%	Rf	H	A	%		
1	0.010	0.0346	0.026	0.2987	78.71	0.037	0.0265	0.00532	81.67	Yes	THCA-A
2	0.947	0.0000	0.959	0.0218	5.75	0.965	0.0098	0.00021	3.23	No	
3	0.965	0.0098	0.974	0.0590	15.54	0.994	0.0000	0.00098	15.10	No	

Track 12:

Type	Reference
Vial ID	CBDA 100
Description	CBDA 500ng
Volume	5.0 µl

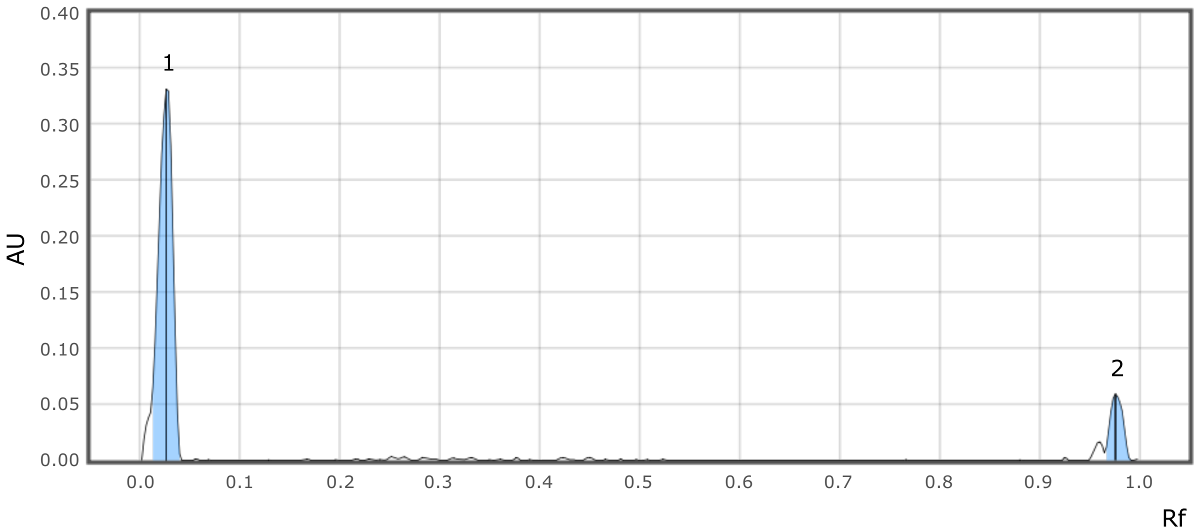

Peak #	Start		Max			End		Area		Manual peak	Substance Name
	Rf	H	Rf	H	%	Rf	H	A	%		
1	0.011	0.0428	0.026	0.3316	84.84	0.041	0.0000	0.00550	86.11	Yes	CBDV
2	0.965	0.0069	0.976	0.0592	15.16	0.994	0.0000	0.00089	13.89	No	

Track 13:

Type	Reference
Vial ID	CBGA 100
Description	CBGA 500ng
Volume	5.0 µl

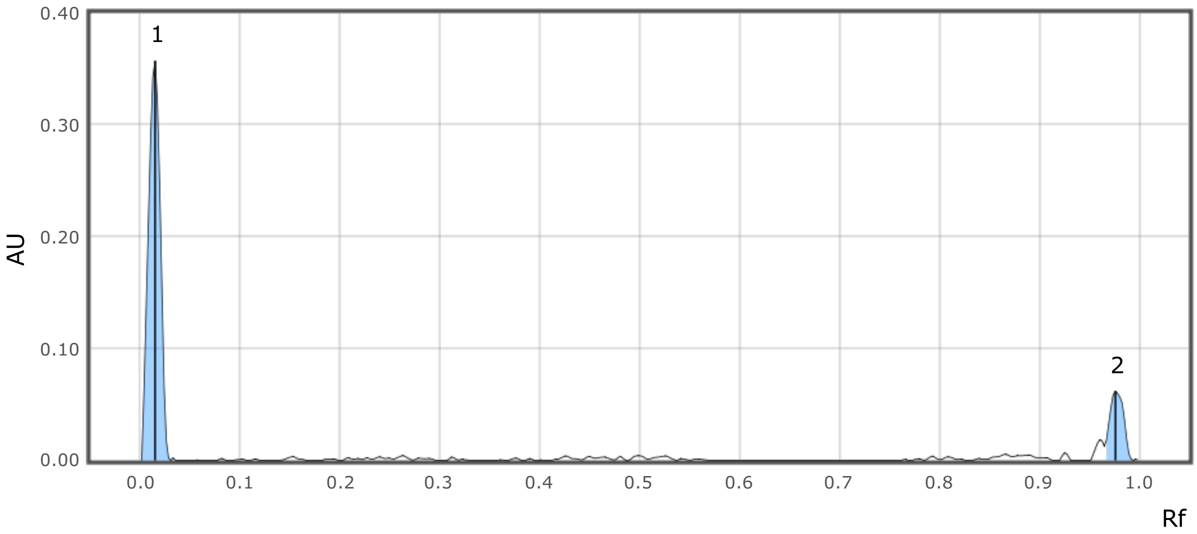

Peak #	Start Rf	Start H	Max Rf	Max H	Max %	End Rf	End H	Area A	Area %	Manual peak	Substance Name
1	0.001	0.0000	0.015	0.3572	85.22	0.030	0.0000	0.00492	82.87	No	CBGA
2	0.965	0.0127	0.976	0.0619	14.78	0.994	0.0000	0.00102	17.13	No	

Track 14:

Type	Sample
Vial ID	Mixture 100
Description	Mixture 500ng
Volume	5.0 µl

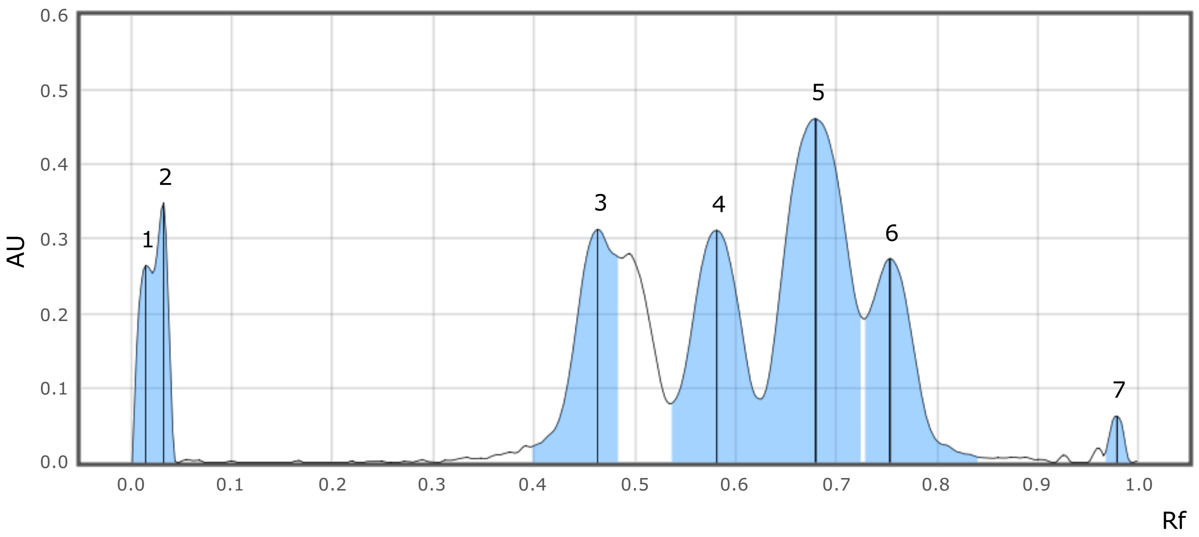

Peak #	Start Rf	Start H	Max Rf	Max H	Max %	End Rf	End H	Area A	Area %	Manual peak	Substance Name
1	0.001	0.0000	0.015	0.2639	12.99	0.021	0.2536	0.00412	4.58	No	
2	0.021	0.2536	0.033	0.3480	17.13	0.046	0.0000	0.00526	5.85	No	
3	0.396	0.0208	0.463	0.3126	15.38	0.488	0.2737	0.01559	17.35	No	
4	0.535	0.0785	0.581	0.3112	15.31	0.624	0.0849	0.01767	19.66	No	
5	0.624	0.0849	0.680	0.4607	22.67	0.726	0.1927	0.03261	36.28	No	
6	0.729	0.1925	0.753	0.2735	13.46	0.842	0.0066	0.01364	15.18	No	
7	0.965	0.0090	0.979	0.0621	3.06	0.994	0.0000	0.00098	1.09	No	

Track 15:

Type	Sample
Vial ID	MeOH blank
Description	MeOH Blank
Volume	2.0 µl

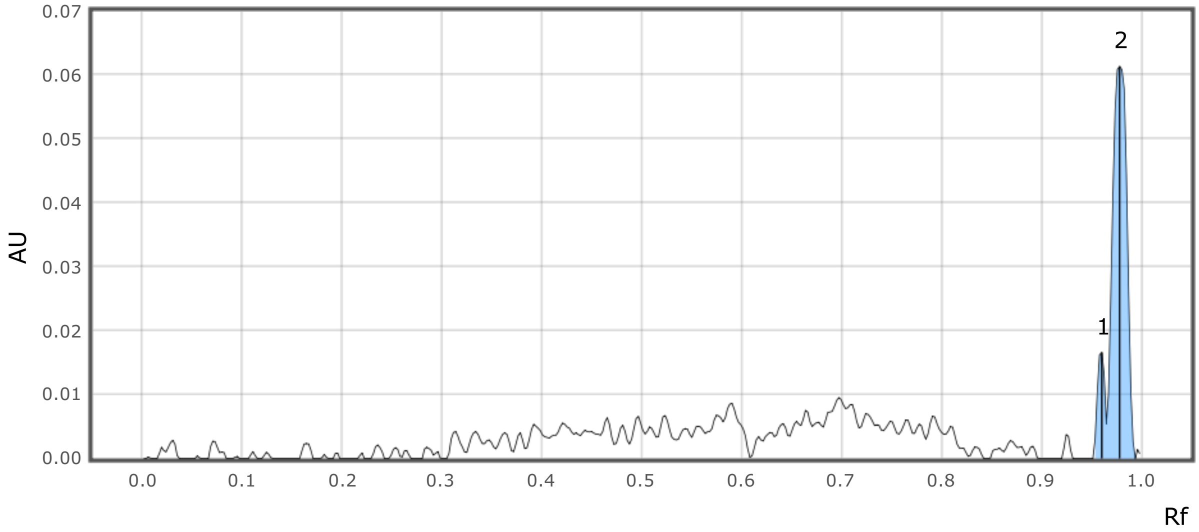

Peak #	Start Rf	Start H	Max Rf	Max H	Max %	End Rf	End H	Area A	Area %	Manual peak	Substance Name
1	0.952	0.0000	0.961	0.0166	21.28	0.965	0.0054	0.00014	11.94	No	
2	0.965	0.0054	0.979	0.0613	78.72	0.994	0.0000	0.00102	88.06	No	

Calibration results:

Height calibration for substance 8-THC @ RT White:

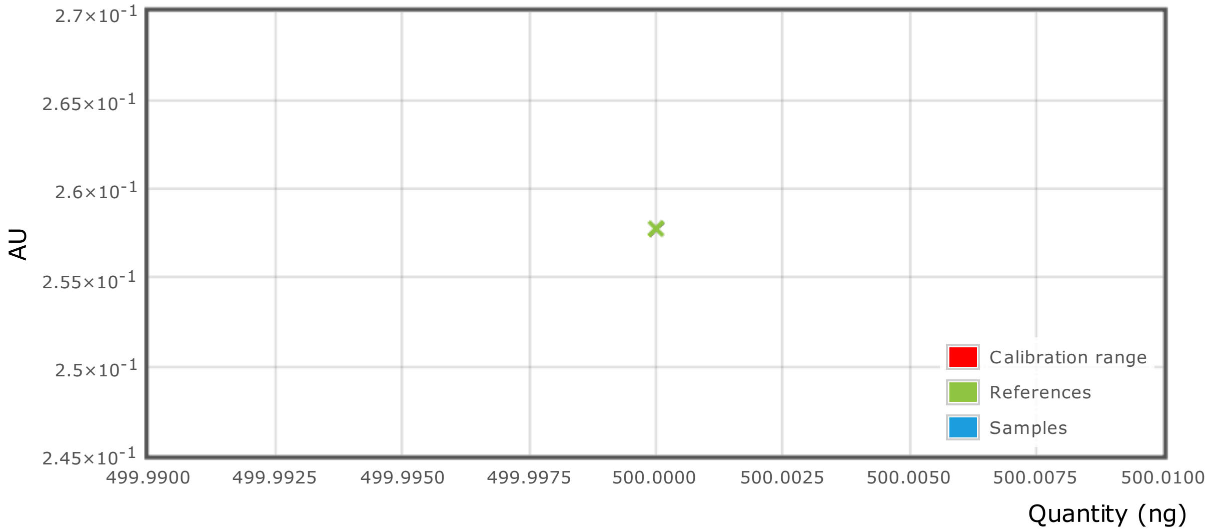

Regression mode	Linear-2
Range deviation	5.00 %
Related substances	Default
Number of references	1
Calibration function	$y=0x$
Coefficient of variation	CV 0.00 %
Correlation coefficient	n/a
⚠	Unable to compute the results for this substance because there wasn't enough groups of references replicas (at least 1 for Linear-1, 2 for Linear2 and Mime-1 and 3 for Polynomial and MiMe-2)

Height calibration for substance 9-THC @ RT White:

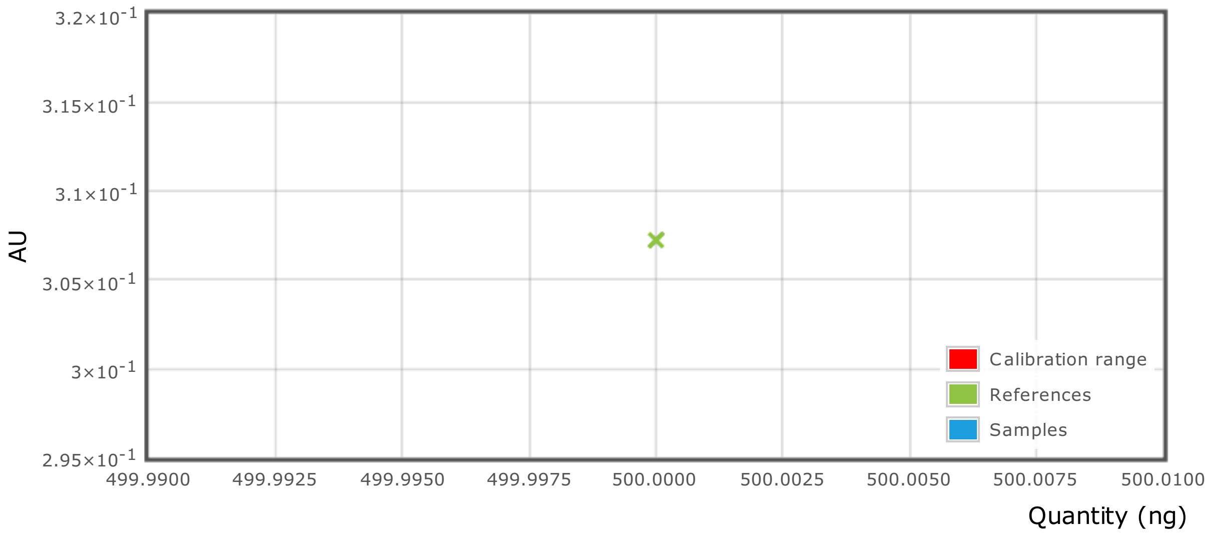

Regression mode	Linear-2
Range deviation	5.00 %
Related substances	Default
Number of references	1
Calibration function	$y=0x$
Coefficient of variation	CV 0.00 %
Correlation coefficient	n/a
⚠	Unable to compute the results for this substance because there wasn't enough groups of references replicas (at least 1 for Linear-1, 2 for Linear2 and Mime-1 and 3 for Polynomial and MiMe-2)

Height calibration for substance CBC @ RT White:

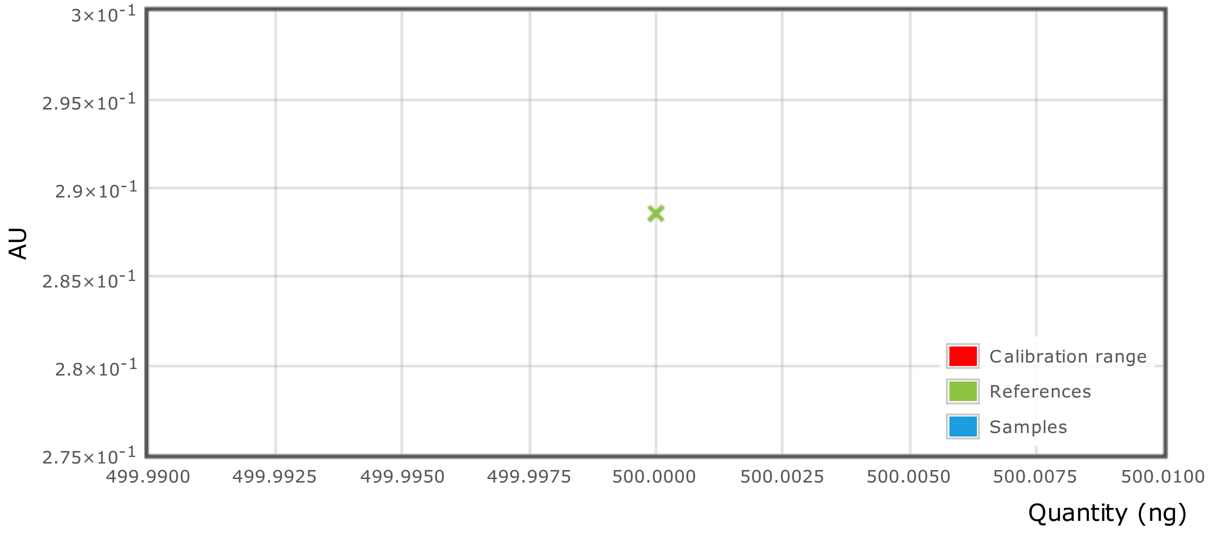

Regression mode	Linear-2
Range deviation	5.00 %
Related substances	Default
Number of references	1
Calibration function	$y=0x$
Coefficient of variation	CV 0.00 %
Correlation coefficient	n/a
⚠	Unable to compute the results for this substance because there wasn't enough groups of references replicas (at least 1 for Linear-1, 2 for Linear2 and Mime-1 and 3 for Polynomial and MiMe-2)

Height calibration for substance CBD @ RT White:

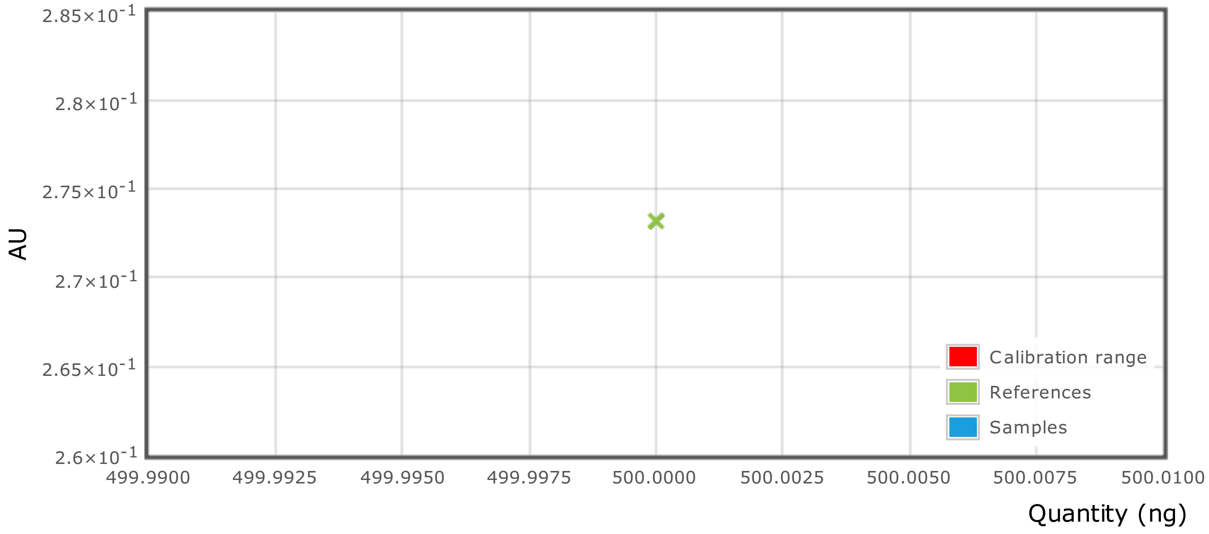

Regression mode	Linear-2
Range deviation	5.00 %
Related substances	Default
Number of references	1
Calibration function	$y=0x$
Coefficient of variation	CV 0.00 %
Correlation coefficient	n/a
⚠	Unable to compute the results for this substance because there wasn't eno ugh groups of references replicas (at least 1 for Linear-1, 2 for Linear2 and Mime-1 and 3 for Polynomial and MiMe-2)

Height calibration for substance CBDA @ RT White:

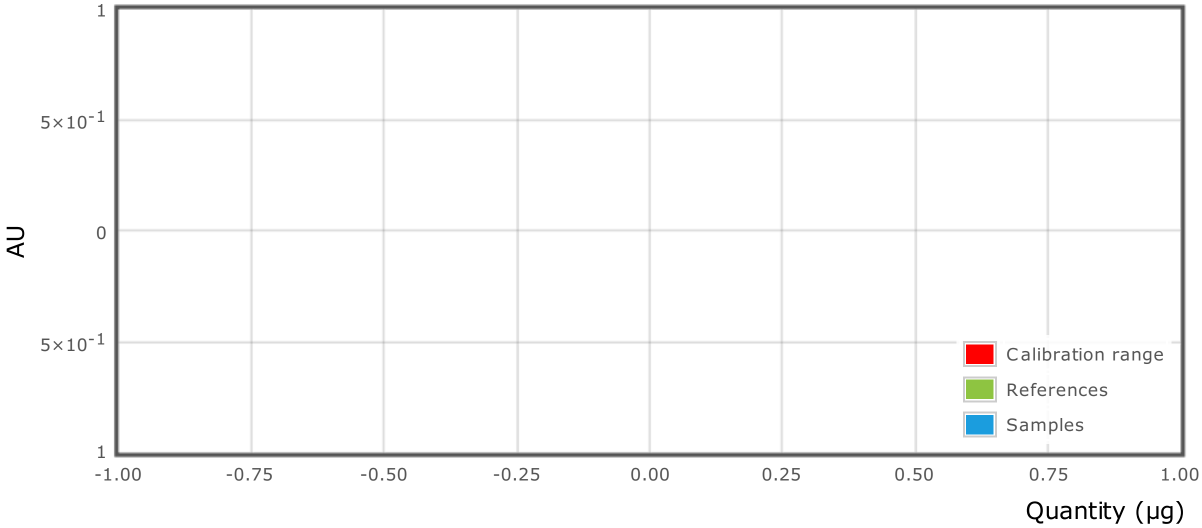

Regression mode	Linear-2
Range deviation	5.00 %
Related substances	Default
Number of references	0
Calibration function	$y=0x$
Coefficient of variation	CV 0.00 %
Correlation coefficient	n/a
⚠	There wasn't any reference application available in the assignments for this substance. Please check that the peaks were correctly detected and assigne d for this substance.

Height calibration for substance CBDV @ RT White:

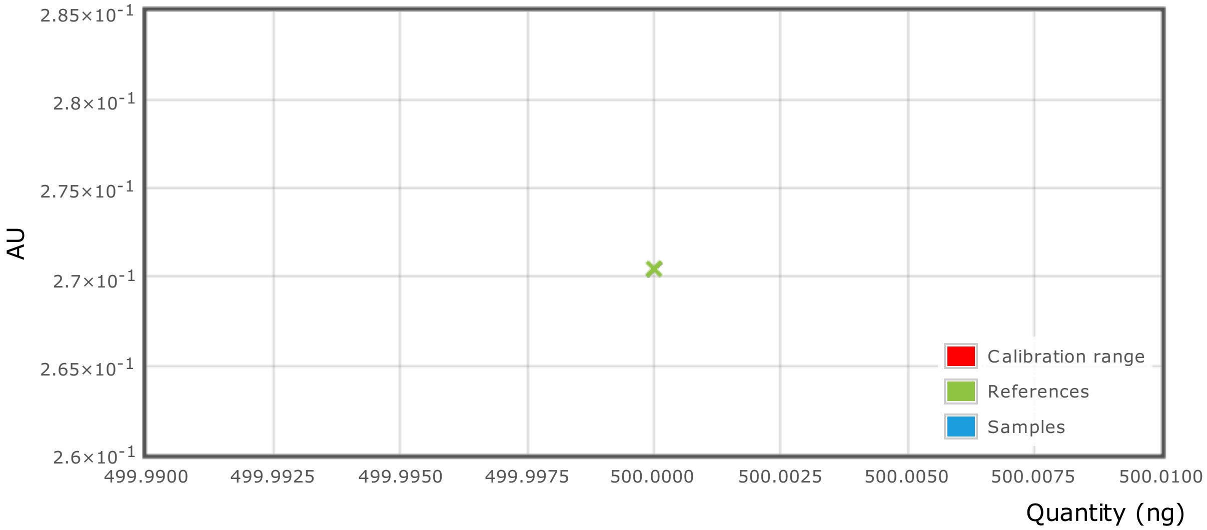

Regression mode	Linear-2
Range deviation	5.00 %
Related substances	Default
Number of references	1
Calibration function	$y=0x$
Coefficient of variation	CV 0.00 %
Correlation coefficient	n/a
⚠	Unable to compute the results for this substance because there wasn't eno ugh groups of references replicas (at least 1 for Linear-1, 2 for Linear2 and Mime-1 and 3 for Polynomial and MiMe-2)

Height calibration for substance CBG @ RT White:

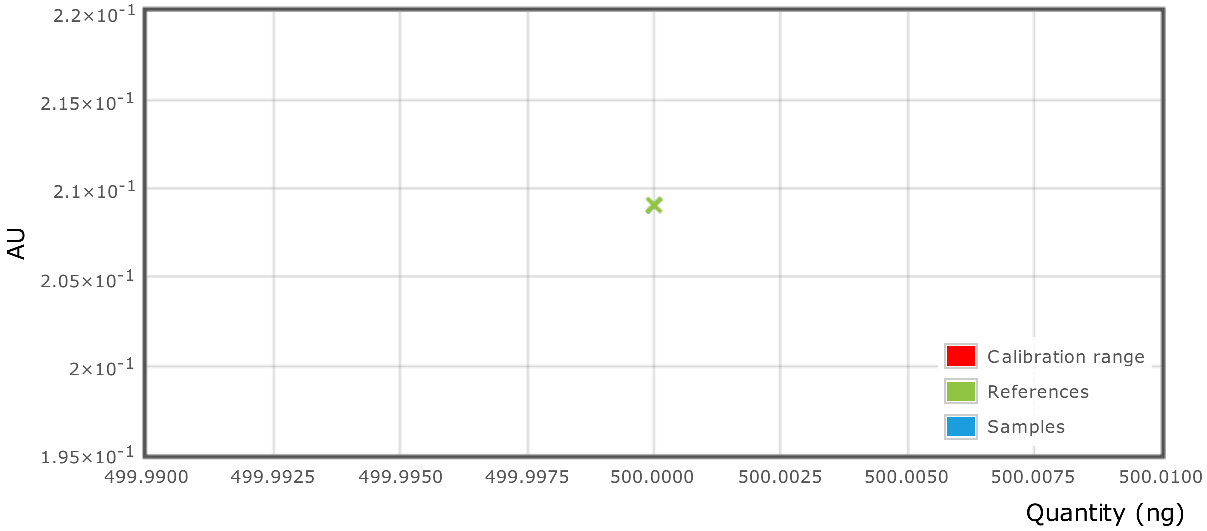

Regression mode	Linear-2
Range deviation	5.00 %
Related substances	Default
Number of references	1
Calibration function	$y=0x$
Coefficient of variation	CV 0.00 %
Correlation coefficient	n/a
⚠	Unable to compute the results for this substance because there wasn't enough groups of references replicas (at least 1 for Linear-1, 2 for Linear2 and Mime-1 and 3 for Polynomial and MiMe-2)

Height calibration for substance CBGA @ RT White:

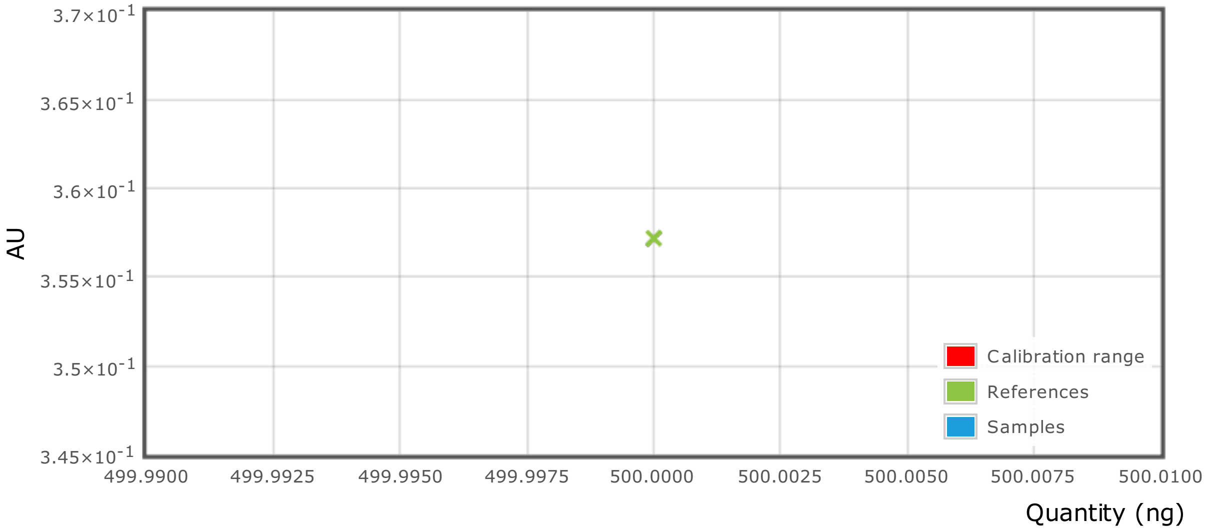

Regression mode	Linear-2
Range deviation	5.00 %
Related substances	Default
Number of references	1
Calibration function	$y=0x$
Coefficient of variation	CV 0.00 %
Correlation coefficient	n/a
⚠	Unable to compute the results for this substance because there wasn't enough groups of references replicas (at least 1 for Linear-1, 2 for Linear2 and Mime-1 and 3 for Polynomial and MiMe-2)

Height calibration for substance CBN @ RT White:

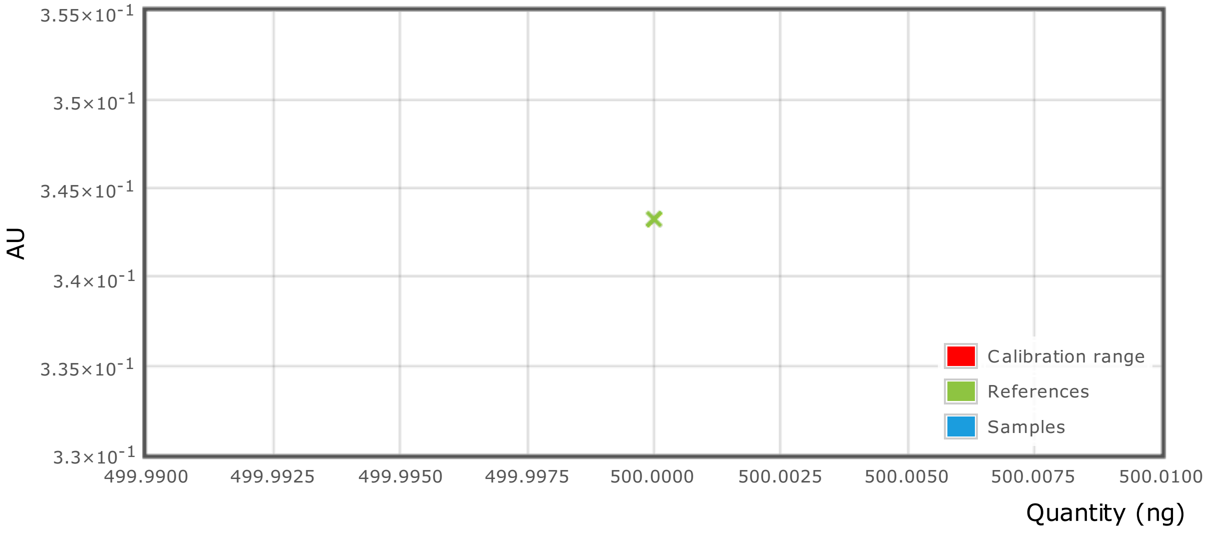

Regression mode	Linear-2
Range deviation	5.00 %
Related substances	Default
Number of references	1
Calibration function	$y=0x$
Coefficient of variation	CV 0.00 %
Correlation coefficient	n/a
⚠	Unable to compute the results for this substance because there wasn't eno ugh groups of references replicas (at least 1 for Linear-1, 2 for Linear2 and Mime-1 and 3 for Polynomial and MiMe-2)

Height calibration for substance THCA-A @ RT White:

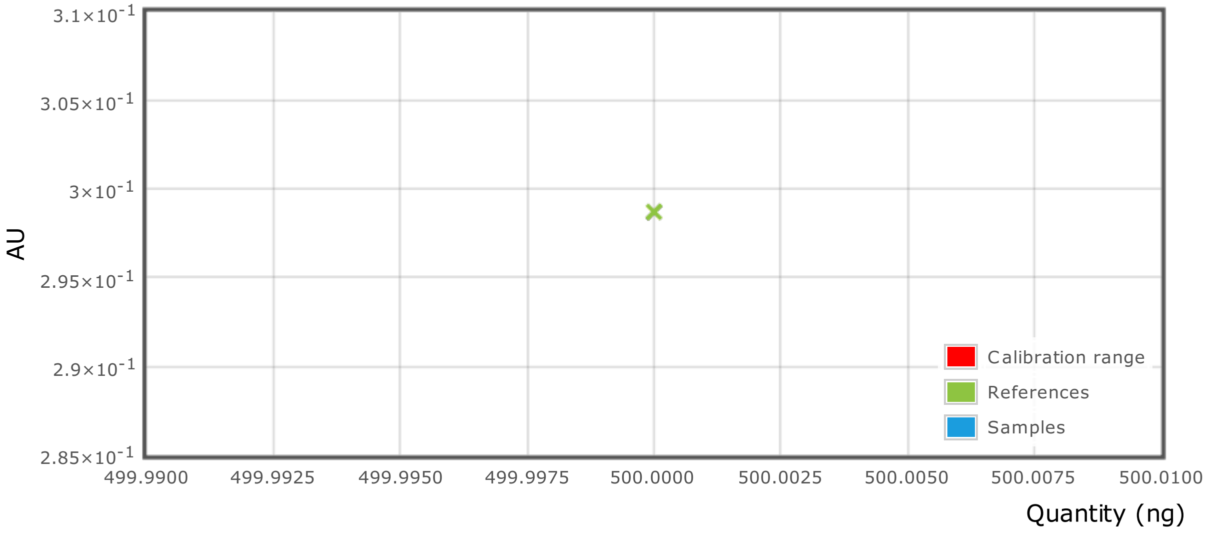

Regression mode	Linear-2
Range deviation	5.00 %
Related substances	Default
Number of references	1
Calibration function	$y=0x$
Coefficient of variation	CV 0.00 %
Correlation coefficient	n/a
⚠	Unable to compute the results for this substance because there wasn't enough groups of references replicas (at least 1 for Linear-1, 2 for Linear2 and Mime-1 and 3 for Polynomial and MiMe-2)

Height calibration for substance THCV @ RT White:

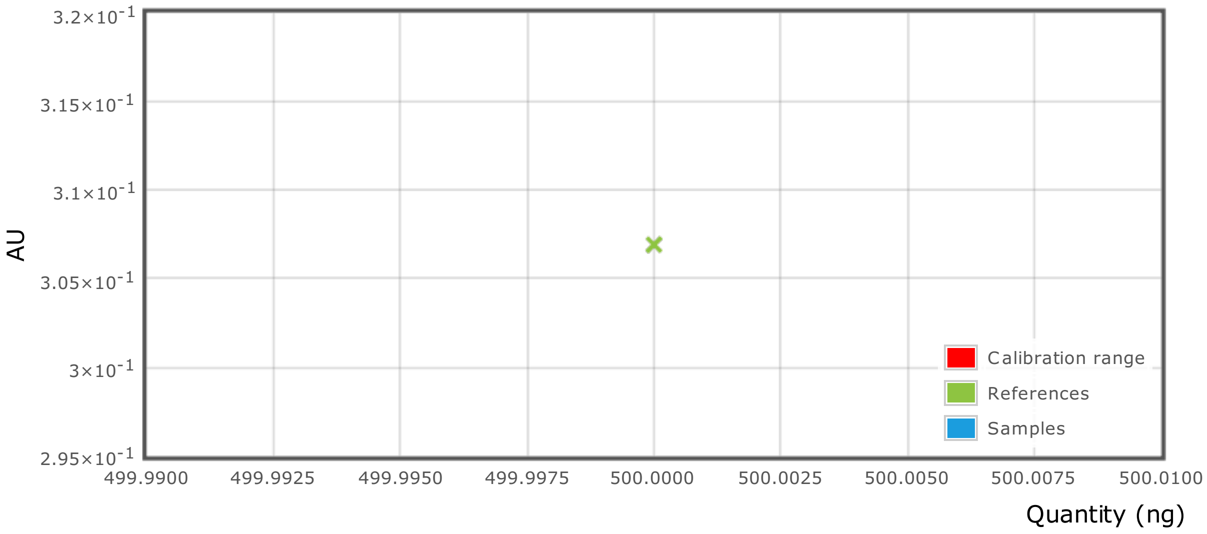

Regression mode	Linear-2
Range deviation	5.00 %
Related substances	Default
Number of references	1
Calibration function	$y=0x$
Coefficient of variation	CV 0.00 %
Correlation coefficient	n/a
⚠	Unable to compute the results for this substance because there wasn't enough groups of references replicas (at least 1 for Linear-1, 2 for Linear2 and Mime-1 and 3 for Polynomial and MiMe-2)

Results:

Substance having no available results		
⚠	CBN	There wasn't any sample application available in the assignments for this substance. Please check that the peaks were correctly detected and assig ned for this substance.
⚠	CBGA	There wasn't any sample application available in the assignments for this substance. Please check that the peaks were correctly detected and assig ned for this substance.
⚠	CBDA	There wasn't any sample application available in the assignments for this substance. Please check that the peaks were correctly detected and assig ned for this substance.
⚠	8-THC	There wasn't any sample application available in the assignments for this substance. Please check that the peaks were correctly detected and assig ned for this substance.
⚠	9-THC	There wasn't any sample application available in the assignments for this substance. Please check that the peaks were correctly detected and assig ned for this substance.
⚠	CBC	There wasn't any sample application available in the assignments for this substance. Please check that the peaks were correctly detected and assig ned for this substance.
⚠	THCV	There wasn't any sample application available in the assignments for this substance. Please check that the peaks were correctly detected and assig ned for this substance.
⚠	THCA-A	There wasn't any sample application available in the assignments for this substance. Please check that the peaks were correctly detected and assig ned for this substance.
⚠	CBD	There wasn't any sample application available in the assignments for this substance. Please check that the peaks were correctly detected and assig ned for this substance.
⚠	CBG	There wasn't any sample application available in the assignments for this substance. Please check that the peaks were correctly detected and assig ned for this substance.
⚠	CBDV	There wasn't any sample application available in the assignments for this substance. Please check that the peaks were correctly detected and assig ned for this substance.

A track marked with ⚠ means: this result is outside the regression range given by the reference assignments, but is included in the results because it is in the allowed range deviation.

Analyst:	Reviewer:
