# Supplementary material for: High Performance Thin-Layer Chromatography (HPTLC) data of Cannabinoids in ten mobile phase systems
Source: Data Brief. 2020 Jun 30;31:105955. doi: 10.1016/j.dib.2020.105955 (PMC7352075; doi:10.1016/j.dib.2020.105955)
Supplement: Supplementary file 1 [file mmc1.zip › S1-Triplicate reports/HDe-2.pdf]

# Analysis: HDe-re-2

**Path: Home/YL Research**

**Based on method: Triplets Method**

| | | |
|---|---|---|
| Created | 07-Jun-2019 22:31:22 | visionCATSuser |
| Modified | 09-Jun-2019 17:47:04 | visionCATSuser |
| Last HPTLC log | 09-Jun-2019 17:47:04 | Analysis modified |
| Explorer notes | | |

| Track | Vial ID | Description | Volume | Position | Type |
|---|---|---|---|---|---|
| 1 | MeOH blank | MeOH Blank | 2.0 µl | A1 | Sample |
| 2 | Mixture 100 | Mixture 500ng | 5.0 µl | B1 | Sample |
| 3 | 9-THC 100 | D9-THC 500ng | 5.0 µl | C1 | Reference |
| 4 | CBD 100 | CBD 500ng | 5.0 µl | D1 | Reference |
| 5 | CBN 100 | CBN 500ng | 5.0 µl | E1 | Reference |
| 6 | CBG 100 | CBG 500ng | 5.0 µl | F1 | Reference |
| 7 | CBC 100 | CBC 500ng | 5.0 µl | A2 | Reference |
| 8 | THCV 100 | THCV 500ng | 5.0 µl | B2 | Reference |
| 9 | CBDV 100 | CBDV 500ng | 5.0 µl | C2 | Reference |
| 10 | 8-THC 100 | D8-THC 500ng | 5.0 µl | D2 | Reference |
| 11 | THCA-A 100 | THCA-A 500ng | 5.0 µl | E2 | Reference |
| 12 | CBDA 100 | CBDA 500ng | 5.0 µl | F2 | Reference |
| 13 | CBGA 100 | CBGA 500ng | 5.0 µl | A3 | Reference |
| 14 | Mixture 100 | Mixture 500ng | 5.0 µl | B1 | Sample |
| 15 | MeOH blank | MeOH Blank | 2.0 µl | A1 | Sample |
| Sequence table notes | | | | | |

A track marked with ⚠ means: the application type is overridden in some evaluation(s).

## System setup:

| | |
|---|---|
| Software | Server User-PC, version 2.5.18072.1 |
| ATS4 | S/N:080713 |
| Chamber | N/A |
| Derivatization dip | N/A |
| Scanner3 | S/N:031025 |
| Visualizer | S/N:230515 |

# Chromatography

## Plate layout:

| | |
|---|---|
| Stationary phase | Merck, HPTLC plates silica gel 60 F 254 |
| Plate format | 200.0 x 100.0 mm |
| Application type | User |
| Application | Position Y: 10.0 mm, length: 8.0 mm, width: 0.0 mm |
| Track | First position X: 20.0 mm, distance: 11.4 mm |
| Solvent front position | 70.0 mm |
| Notes | |

## Take image clean plate 1a - Visualizer (S/N: 230515):

| | |
|---|---|
| Quality | Enhanced |
| RT White | auto capture, Auto, level 85 %, Band |
| R 254 | auto capture, Auto, level 85 %, Band |
| Instrument diagnostics | Valid diagnostics |
| Documentation step label | |
| Notes | |

## Application 1 - ATS 4 (S/N: 080713):

| | |
|---|---|
| Spray gas | NI |
| Sample solvent type | Methanol |
| Filling speed | 15 µl/s |
| Predosage volume | 200 nl |
| Retraction volume | 200 nl |
| Dosage speed | 150 nl/s |
| Filling quality | User |
| Rinsing cycles / vacuum | 1 / 4 s |
| Filling cycles / vacuum | 1 / 4 s |
| Rinsing solvent name | Methanol |
| Nozzle temperature | Unheated |
| Rack in use | Standard |
| Instrument diagnostics | Valid diagnostics |
| Notes | |

## Development 1 - Chamber:

| | |
|---|---|
| Tank | TTC 20x10 |
| Mobile phase | |
| Saturation time | 20 min |
| Use saturation pad | true |
| Use smartALERT | false |
| Volume front through | 10 ml |
| Volume rear through | 20 ml |
| Drying time | 5 min |
| Drying temperature | Room temperature |
| Notes | |

## Take image developed plate 1a - Visualizer (S/N: 230515):

| | |
|---|---|
| Quality | Enhanced |
| RT White | auto capture, Auto, level 85 %, Band |
| R 254 | auto capture, Auto, level 85 %, Band |
| R 366 | auto capture, Auto, level 85 %, Band |
| Instrument diagnostics | Valid diagnostics |
| Documentation step label | |
| Notes | |

## Scan developed plate 1b - Scanner 3 (S/N: 031025):

| | |
|---|---|
| Scanner type | Single λ |
| Optimization for | Resolution |
| Measurement mode | Absorption |
| Filter | n/a |
| Detector mode | Automatic |
| Scanning speed | 20 mm/s |
| Data resolution | 100 µm/step |
| Slit | 5 x 0.2 mm, micro |
| Partial scan | No |
| Lamp | Deuterium & Tungsten |
| Wavelength(s) | 254 nm |
| Instrument diagnostics | Valid diagnostics |
| Documentation step label | |
| Notes | |

## Derivatization 1 - dip:

| | |
|---|---|
| Reagent name | Fast Blue B salt |
| Dipping speed | 3 |
| Dipping time | 5 s |
| Reagent preparation | 1g Fast Blue B salt in 200mL water |
| Heating | none |
| Notes | Air dry for 5 minutes |

## Take image derivatized plate 1a - Visualizer (S/N: 230515):

| | |
|---|---|
| Quality | Enhanced |
| RT White | auto capture, Auto, level 85 %, Band |
| R 366 | auto capture, Auto, level 85 %, Band |
| Instrument diagnostics | Valid diagnostics |
| Documentation step label | |
| Notes | |

# System suitability tests:

## SST settings:

| | |
|---|---|
| SST tracks | |

# Data acquisition

## Application 1 - ATS 4 (S/N: 080713):

| | |
|---|---|
| Executed | 09-Jun-2019 16:12:55 visionCATSuser |

## Development 1 - Chamber:

| | |
|---|---|
| Executed | 09-Jun-2019 17:33:25 visionCATSuser |

## Take image developed plate 1a - Visualizer (S/N: 230515):

| | |
|---|---|
| Executed | 09-Jun-2019 17:33:28 visionCATSuser |

RT White Developed, RemTransVis

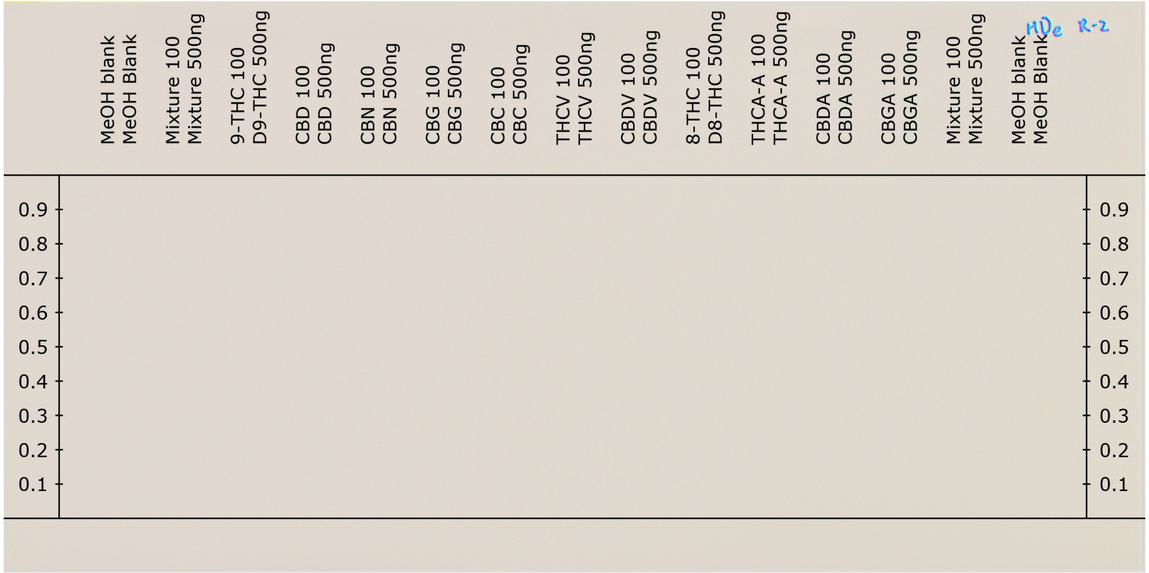


| | |
|---|---|
| Exposure | 0.079 s |
| Contrast | 1 |
| Normalized exposure | Disabled |
| Clarify | Disabled |
| White balance | 1.00, 1.00, 1.00 |

R 254 Developed, Remission254

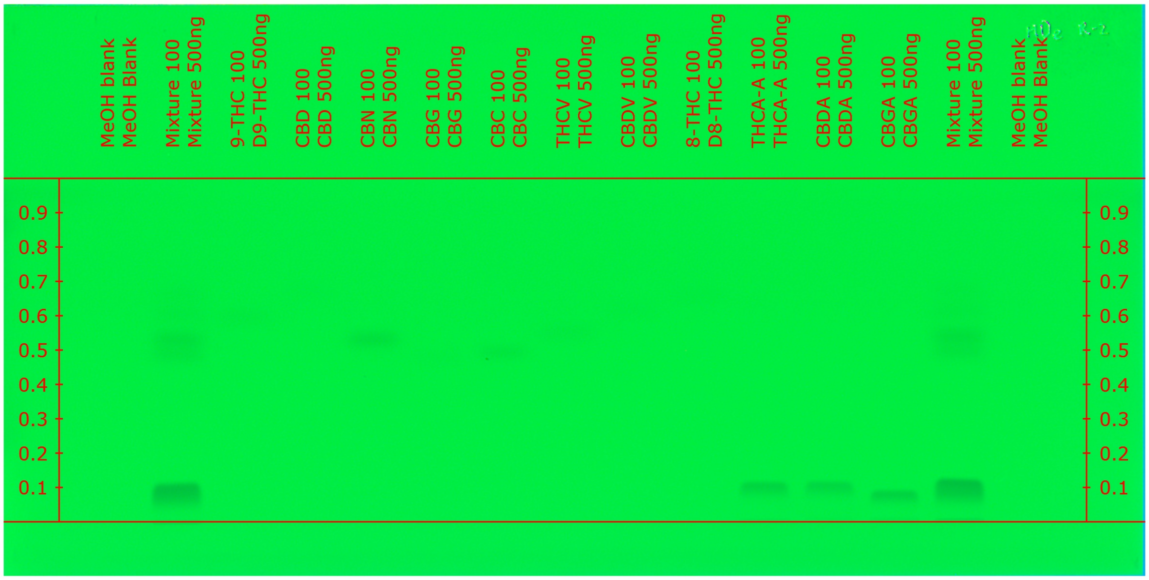


| | |
|---|---|
| Exposure | 0.254 s |
| Contrast | 1 |
| Normalized exposure | Disabled |
| Clarify | Disabled |
| White balance | 1.00, 1.00, 1.00 |

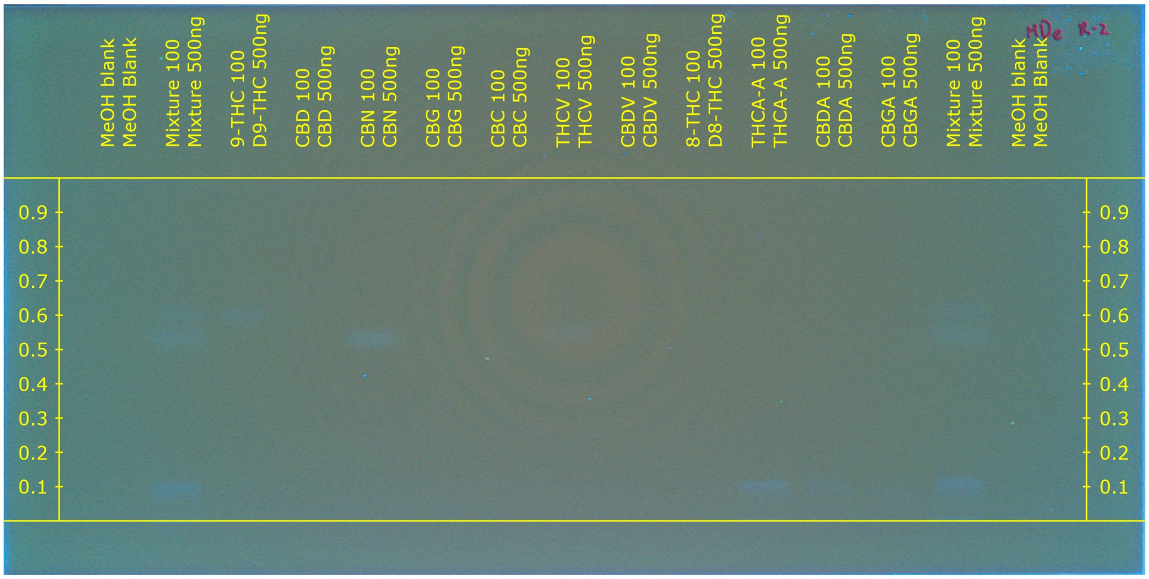


| | |
|---|---|
| Exposure | 10.000 s |
| Contrast | 1 |
| Normalized exposure | Disabled |
| Clarify | Disabled |
| White balance | 1.00, 1.00, 1.00 |

## Scan developed plate 1b - Scanner 3 (S/N: 031025):

| | |
|---|---|
| Executed | 09-Jun-2019 17:34:51 visionCATSuser |

## Scan:

| | |
|---|---|
| Wavelength | 254 nm |

## Track 1:

| | |
|---|---|
| Type | Single λ |

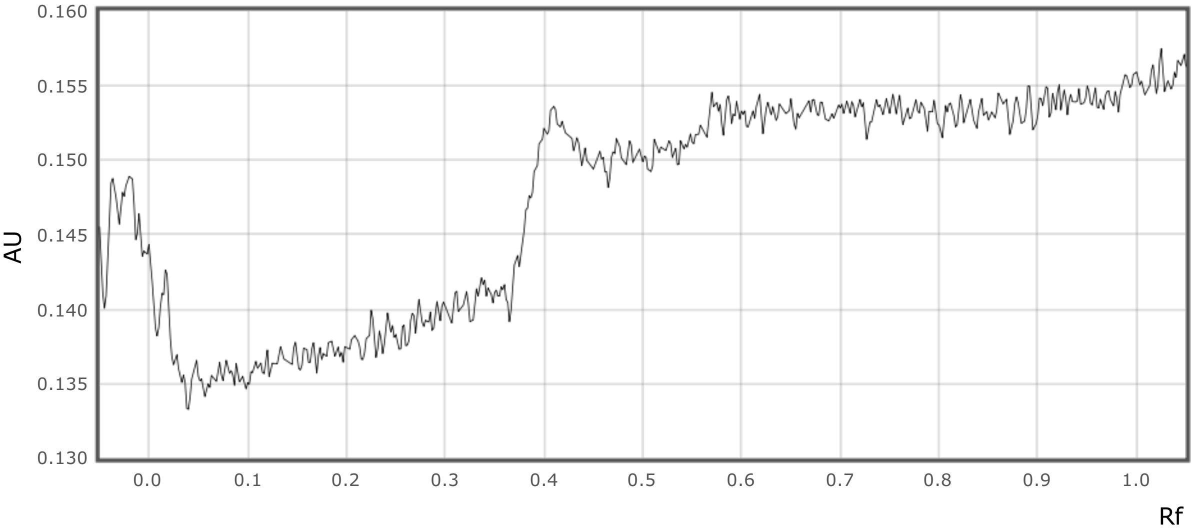

## Track 2:

| Type | Single λ |
| --- | --- |

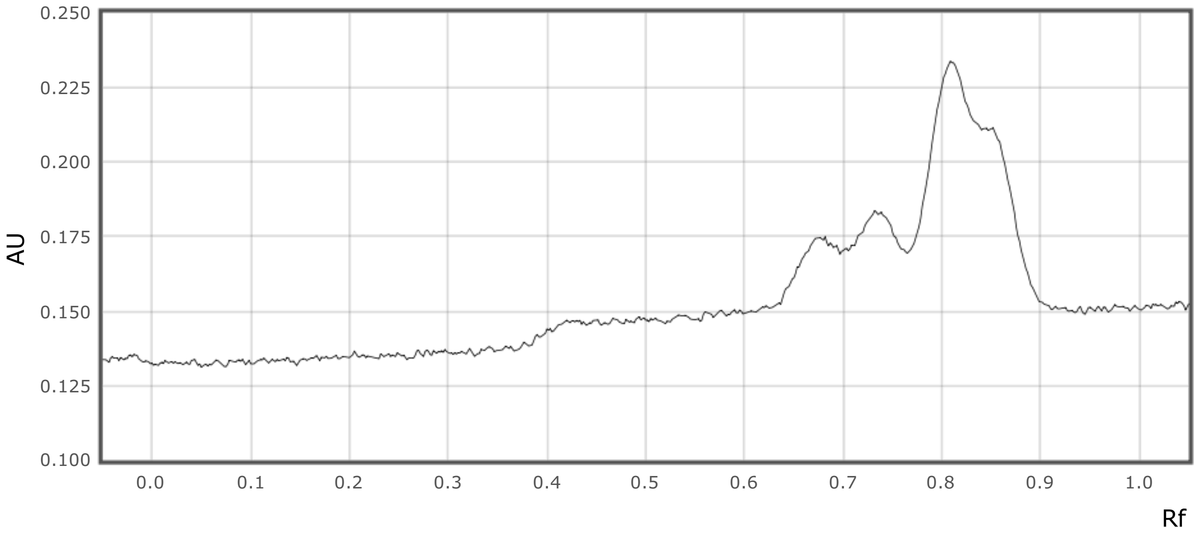


## Track 3:

| Type | Single λ |
| --- | --- |

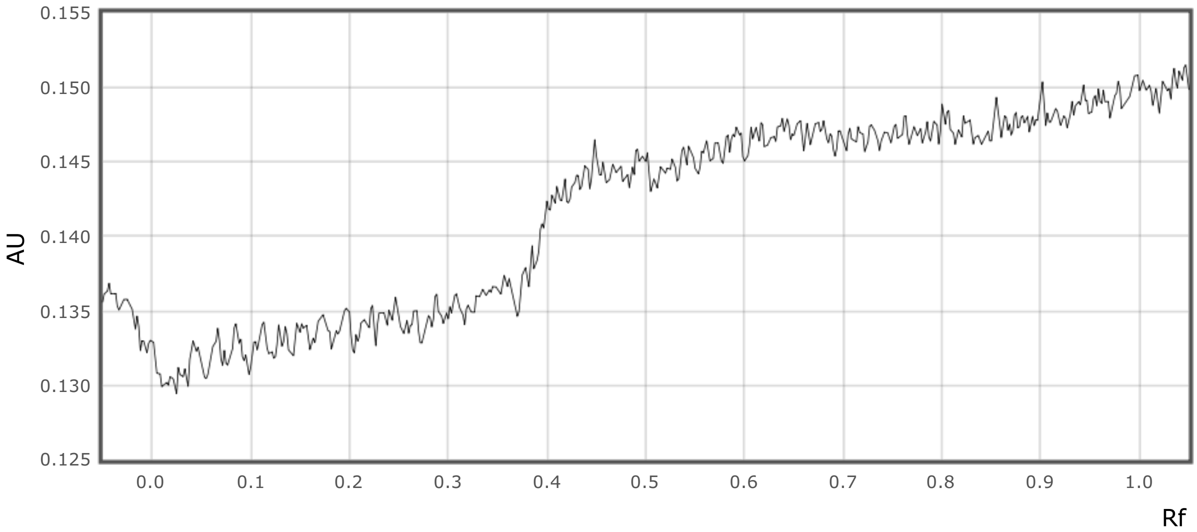


## Track 4:

| Type | Single λ |
| --- | --- |

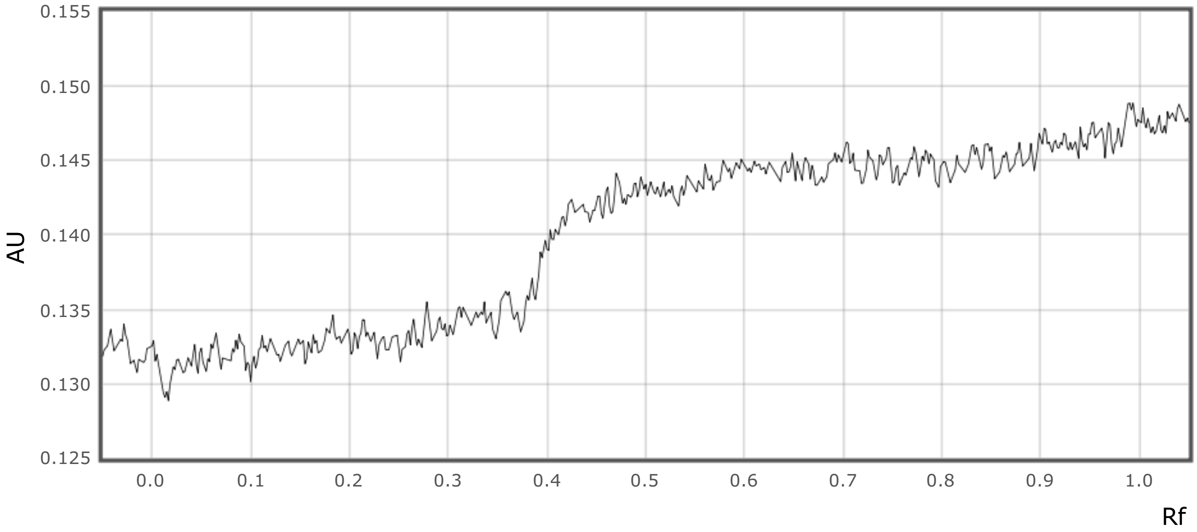


| Track 5: | |
|---|---|
| Type | Single λ |

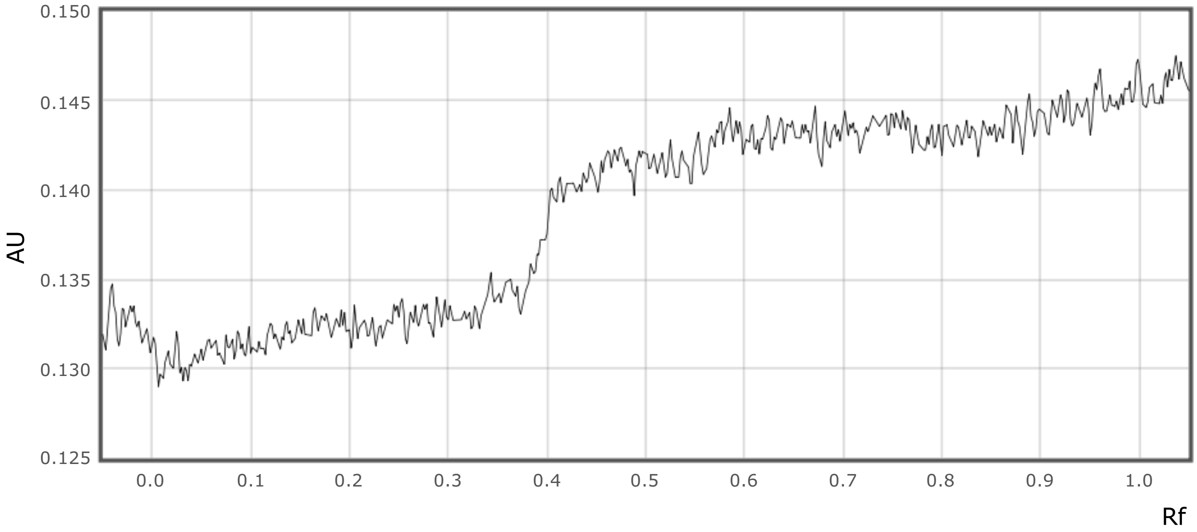


| Track 6: | |
|---|---|
| Type | Single λ |

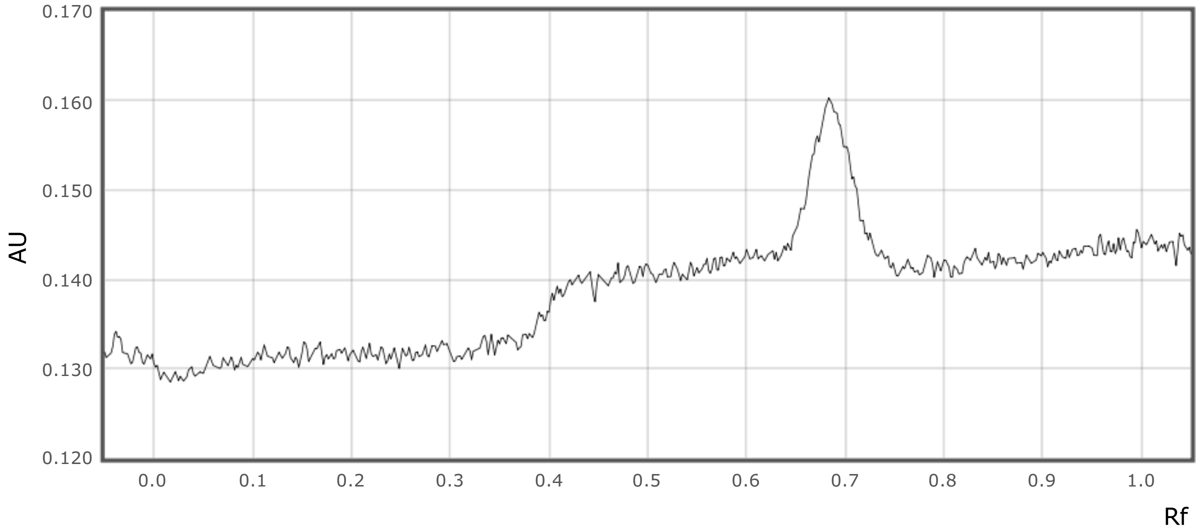


## Track 7:

| Type | Single λ |
|---|---|

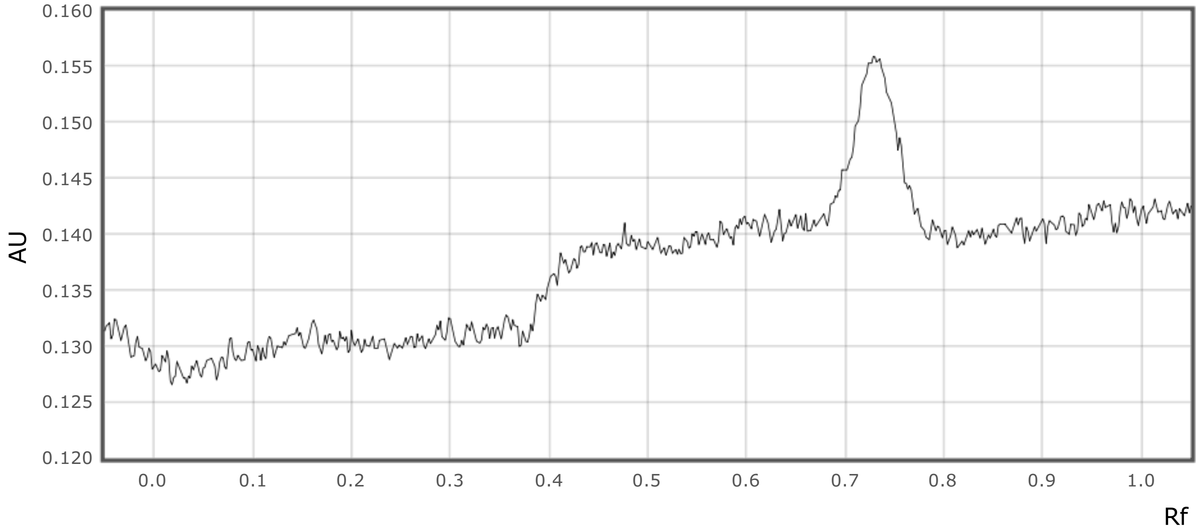


## Track 8:

| Type | Single λ |
|---|---|

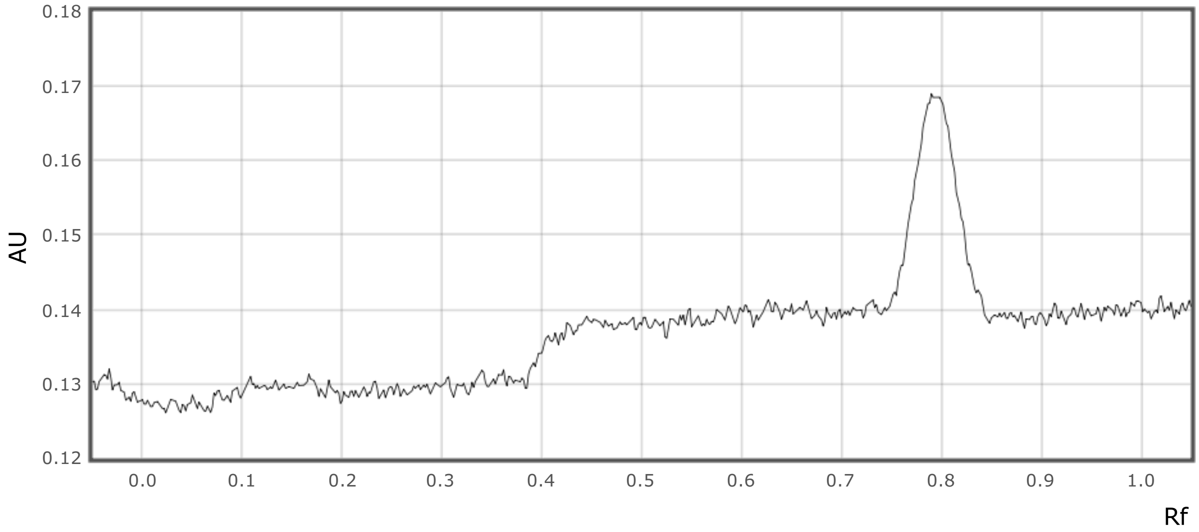


| Track 9: | |
|---|---|
| Type | Single λ |

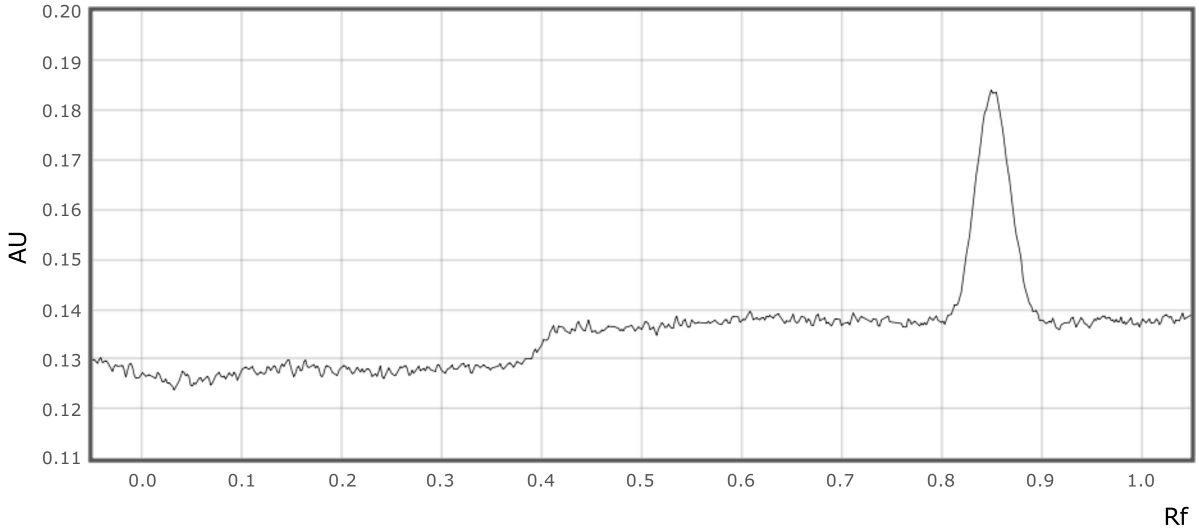


| Track 10: | |
|---|---|
| Type | Single λ |

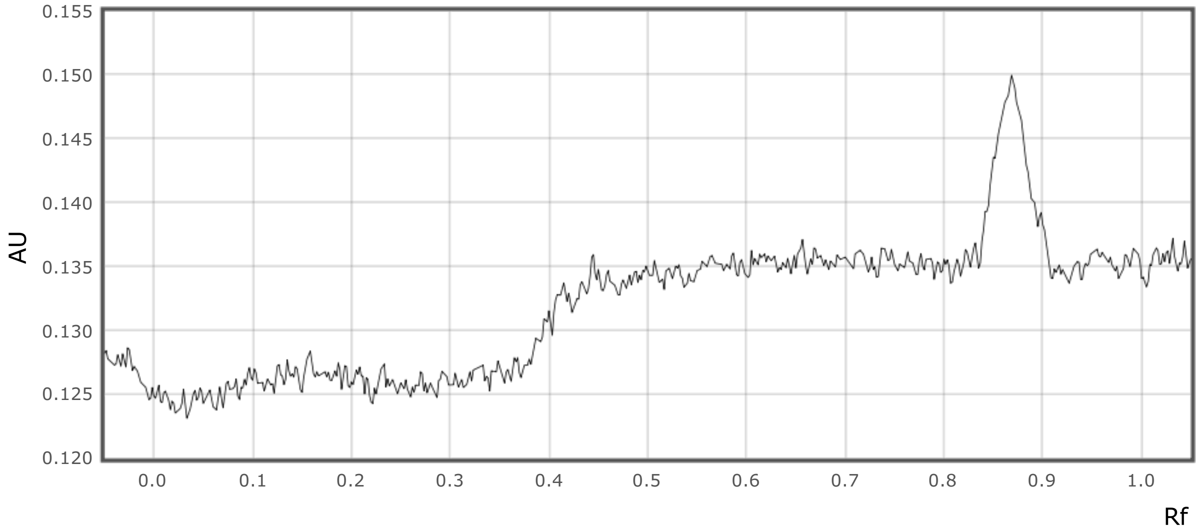


| Track 11: | |
|---|---|
| Type | Single λ |

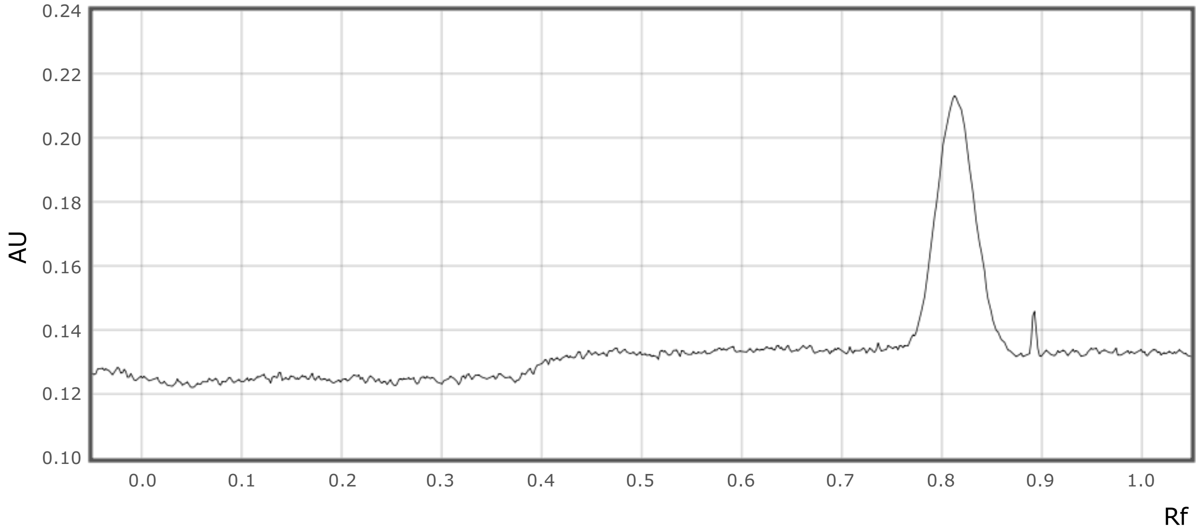


| Track 12: | |
|---|---|
| Type | Single λ |

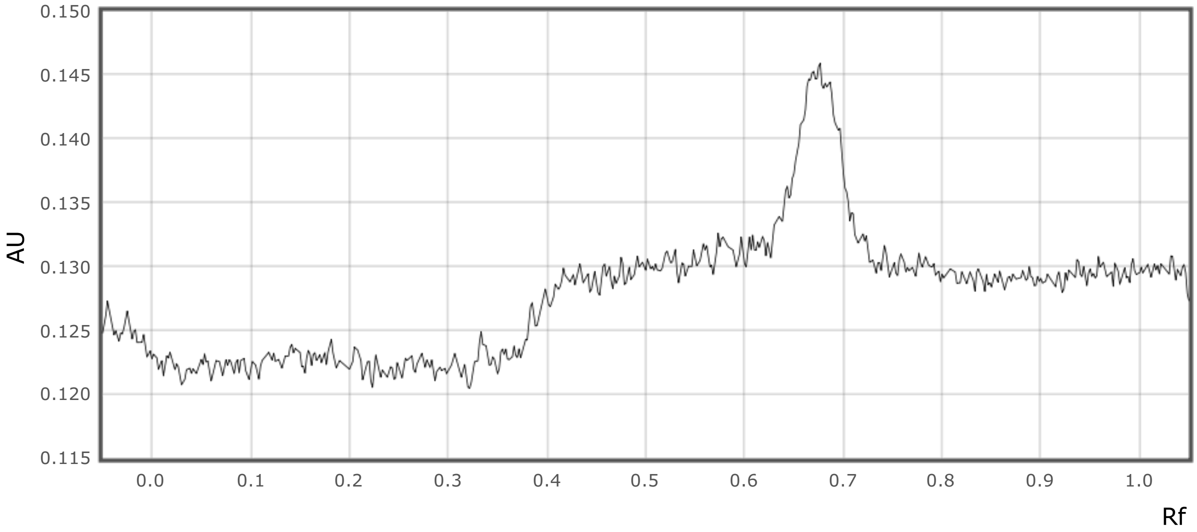


| Track 13: | |
|---|---|
| Type | Single λ |

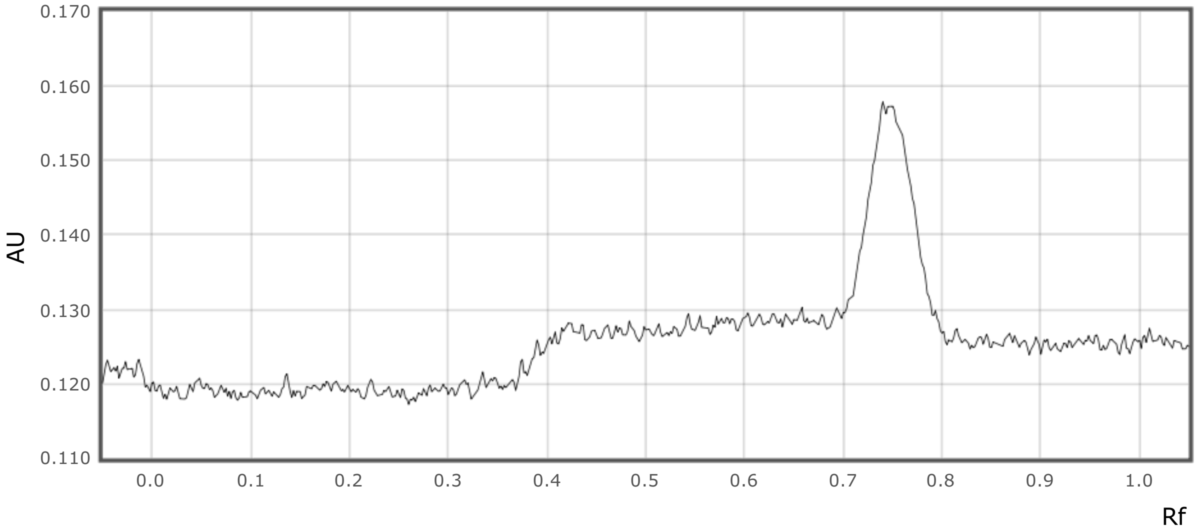


| Track 14: | |
|---|---|
| Type | Single λ |

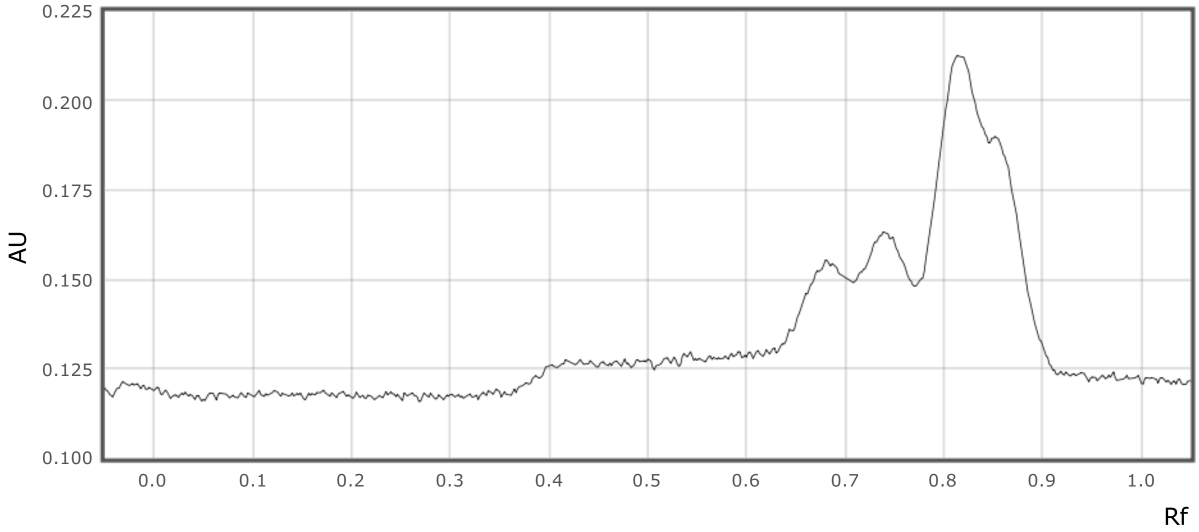


| Track 15: | |
|---|---|
| Type | Single λ |

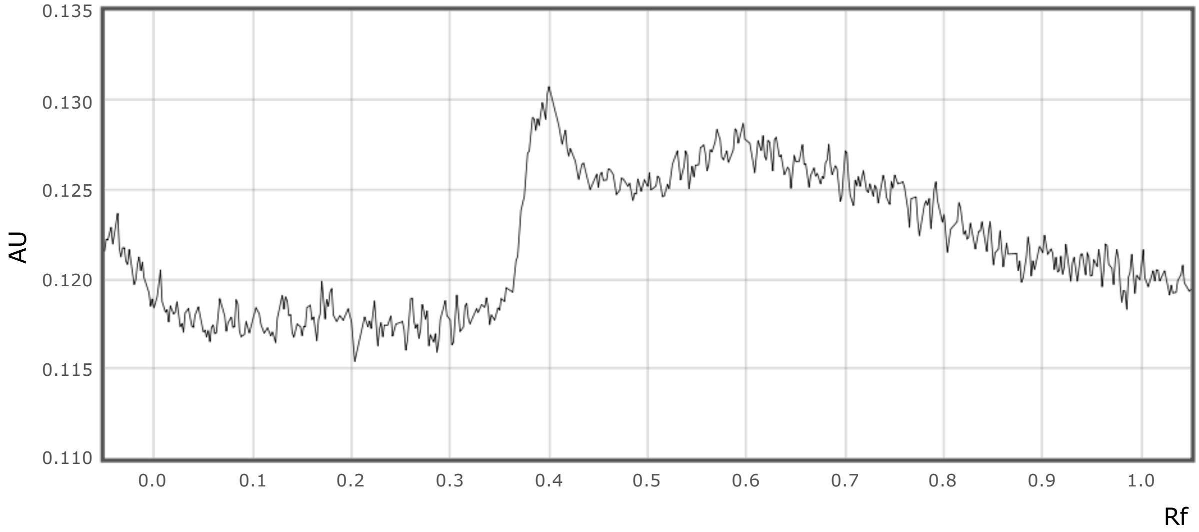


## Derivatization 1 - dip:

| | |
|---|---|
| Executed | 09-Jun-2019 17:39:11 visionCATSuser |

## Take image derivatized plate 1a - Visualizer (S/N: 230515):

| | |
|---|---|
| Executed | 09-Jun-2019 17:41:47 visionCATSuser |

RT White

Derivatized, RemTransVis

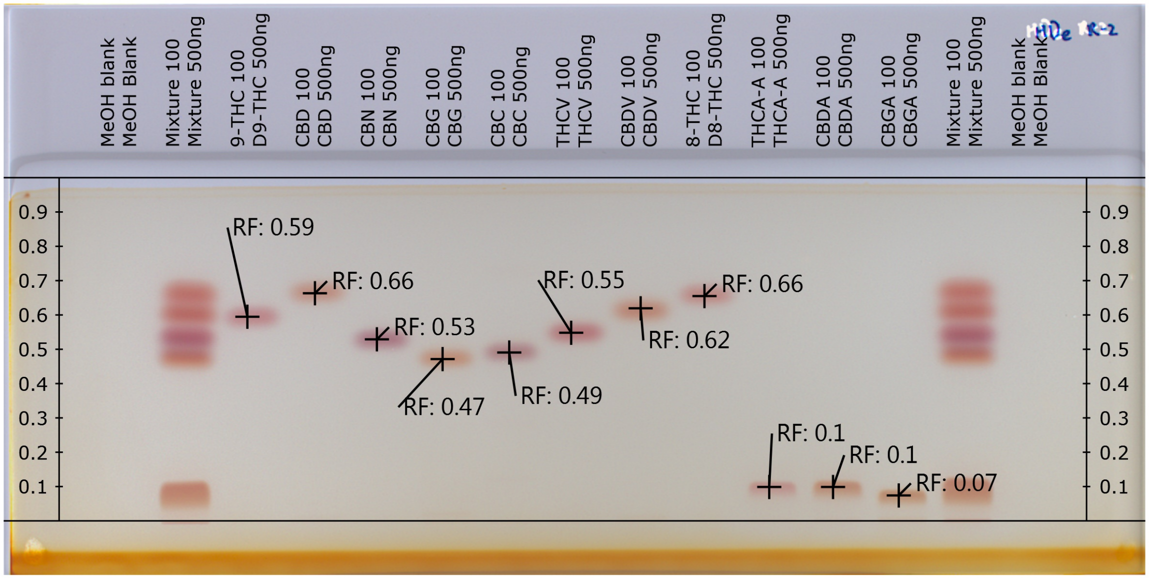


| | |
|---|---|
| Exposure | 0.052 s |
| Contrast | 1 |
| Normalized exposure | Disabled |
| Clarify | Disabled |
| White balance | 1.13, 1.07, 0.85 |

R 366

Derivatized, Remission366

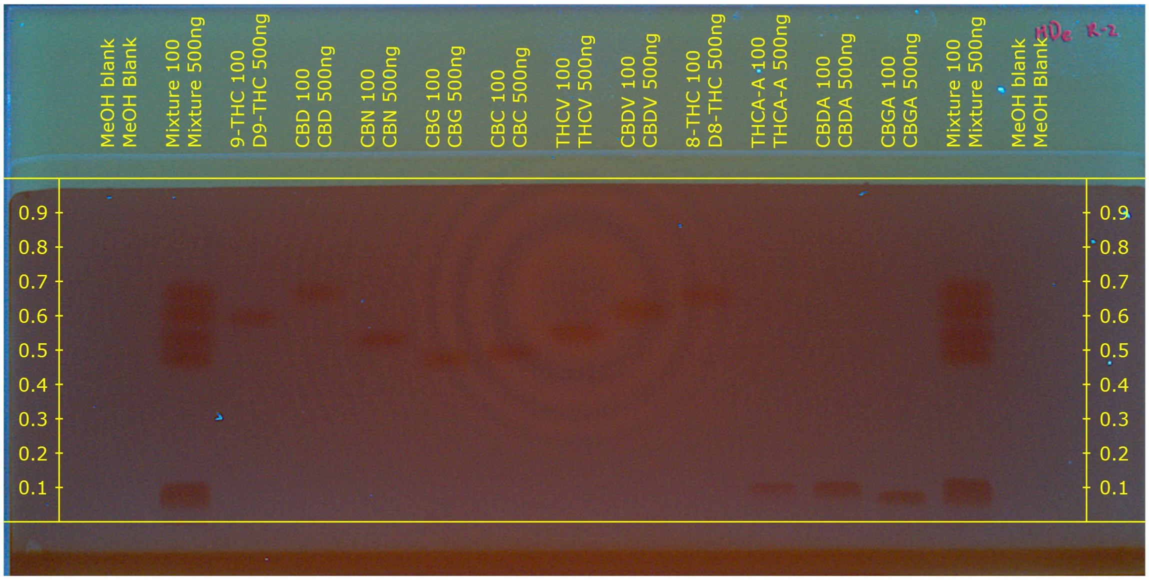


| | |
|---|---|
| Exposure | 10.000 s |
| Contrast | 1 |
| Normalized exposure | Disabled |
| Clarify | Disabled |
| White balance | 1.00, 1.00, 1.00 |

## Evaluation 1 :

| | |
|---|---|
| Validated | false |
| Step | Take image derivatized plate 1a |
| Concentration unit type | Mass / volume |
| Notes | |

# Definition:

## References:

| 9-THC 100 | | |
|---|---|---|
| Substance Name | Concentration | Purity |
| 9-THC | 100.000 µg/ml | 100.00 % |

| CBD 100 | | |
|---|---|---|
| Substance Name | Concentration | Purity |
| CBD | 100.000 µg/ml | 100.00 % |

| CBN 100 | | |
|---|---|---|
| Substance Name | Concentration | Purity |
| CBN | 100.000 µg/ml | 100.00 % |

| CBG 100 | | |
|---|---|---|
| Substance Name | Concentration | Purity |
| CBG | 100.000 µg/ml | 100.00 % |

| CBC 100 | | |
|---|---|---|
| Substance Name | Concentration | Purity |
| CBC | 100.000 µg/ml | 100.00 % |

| THCV 100 | | |
|---|---|---|
| Substance Name | Concentration | Purity |
| THCV | 100.000 µg/ml | 100.00 % |

| CBDV 100 | | |
|---|---|---|
| Substance Name | Concentration | Purity |
| CBDV | 100.000 µg/ml | 100.00 % |

| 8-THC 100 | | |
|---|---|---|
| Substance Name | Concentration | Purity |
| 8-THC | 100.000 µg/ml | 100.00 % |

| THCA-A 100 | | |
|---|---|---|
| Substance Name | Concentration | Purity |
| THCA-A | 100.000 µg/ml | 100.00 % |

| CBDA 100 | | |
|---|---|---|
| Substance Name | Concentration | Purity |
| CBDA | 100.000 µg/ml | 100.00 % |

| CBGA 100 | | |
|---|---|---|
| Substance Name | Concentration | Purity |
| CBGA | 100.000 µg/ml | 100.00 % |

## Samples:

| Vial ID | Amount | Volume solution | Reference amount | Related to |
|---|---|---|---|---|
| MeOH blank | | 0.00 ml | | |
| Mixture 100 | | 0.00 ml | | |

## Integration parameters:

| | |
|---|---|
| Bounds | [0.000,1.000] |
| Smoothing | Savitzky–Golay of order 3 and window 7 |
| Baseline correction | Lowest slope with noise 0.05 |
| Profile subtraction | Profile subtraction from track 1 |
| Peaks detection | Gauss (legacy) with sensitivity 0.1, separation 1 and threshold 0.1 |

## Scan:

| | |
|---|---|
| Wavelength | RT White |

## Track 1:

| | |
|---|---|
| Type | Sample |
| Vial ID | MeOH blank |
| Description | MeOH Blank |
| Volume | 2.0 µl |

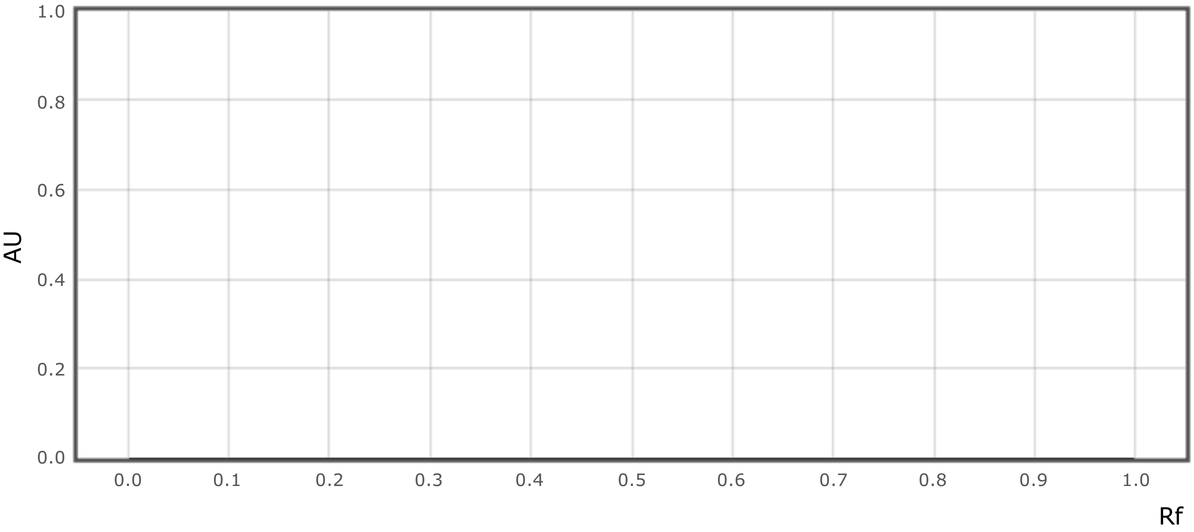


| Peak # | Start | | Max | | | End | | Area | | Manual peak | Substance Name |
|---|---|---|---|---|---|---|---|---|---|---|---|
| | Rf | H | Rf | H | % | Rf | H | A | % | | |

## Track 2:

| | |
|---|---|
| Type | Sample |
| Vial ID | Mixture 100 |
| Description | Mixture 500ng |
| Volume | 5.0 µl |

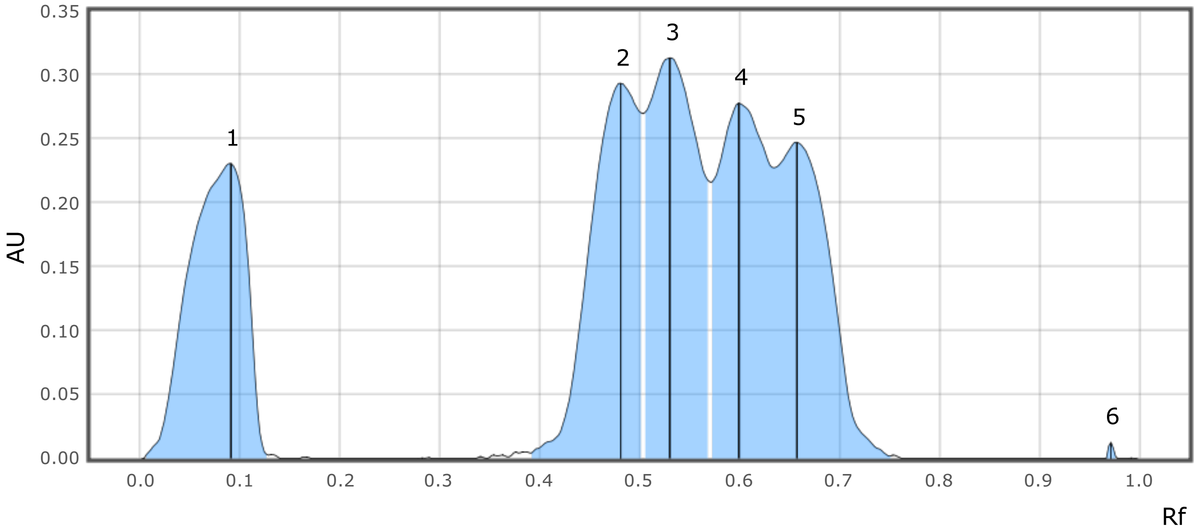


| Peak # | Start | | Max | | | End | | Area | | Manual peak | Substance Name |
|---|---|---|---|---|---|---|---|---|---|---|---|
| | Rf | H | Rf | H | % | Rf | H | A | % | | |
| 1 | 0.004 | 0.0000 | 0.091 | 0.2306 | 16.77 | 0.128 | 0.0025 | 0.01559 | 19.18 | No | |
| 2 | 0.392 | 0.0042 | 0.481 | 0.2933 | 21.33 | 0.501 | 0.2704 | 0.01622 | 19.95 | No | |
| 3 | 0.503 | 0.2697 | 0.530 | 0.3133 | 22.79 | 0.570 | 0.2163 | 0.01861 | 22.89 | No | |
| 4 | 0.573 | 0.2162 | 0.599 | 0.2778 | 20.21 | 0.635 | 0.2273 | 0.01575 | 19.37 | No | |
| 5 | 0.635 | 0.2273 | 0.657 | 0.2473 | 17.98 | 0.751 | 0.0018 | 0.01505 | 18.52 | No | |
| 6 | 0.965 | 0.0000 | 0.972 | 0.0126 | 0.91 | 0.979 | 0.0000 | 0.00007 | 0.09 | No | |

## Track 3:

| | |
|---|---|
| Type | Reference |
| Vial ID | 9-THC 100 |
| Description | D9-THC 500ng |
| Volume | 5.0 µl |

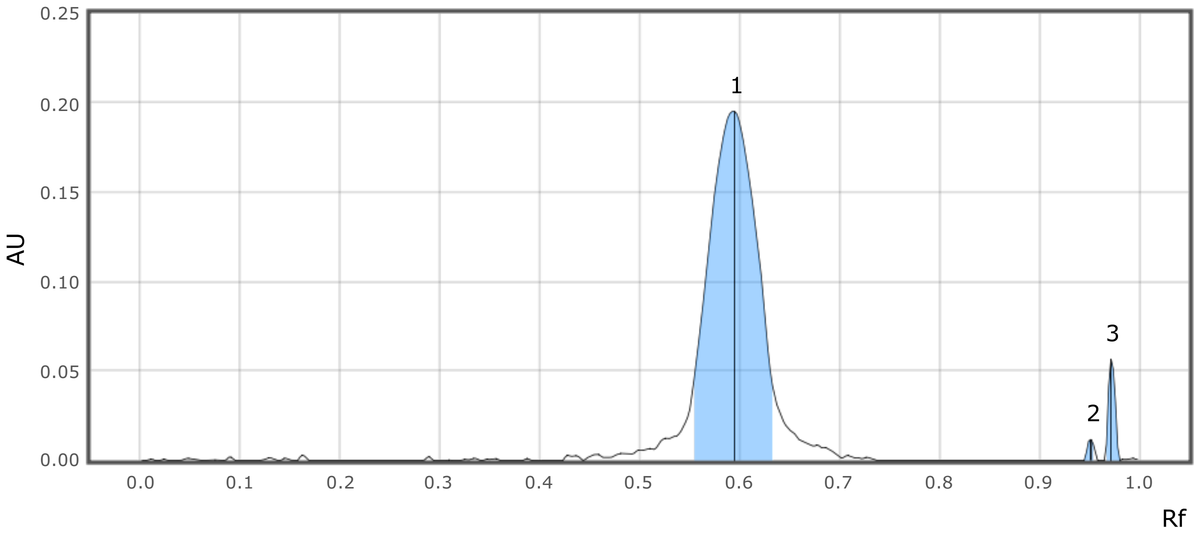

| Peak # | Start Rf | Start H | Max Rf | Max H | Max % | End Rf | End H | Area A | Area % | Manual peak | Substance Name |
|---|---|---|---|---|---|---|---|---|---|---|---|
| 1 | 0.552 | 0.0369 | 0.595 | 0.1951 | 74.06 | 0.636 | 0.0364 | 0.01073 | 95.26 | Yes | 9-THC |
| 2 | 0.945 | 0.0000 | 0.952 | 0.0117 | 4.44 | 0.959 | 0.0000 | 0.00009 | 0.82 | No | |
| 3 | 0.965 | 0.0000 | 0.972 | 0.0566 | 21.50 | 0.981 | 0.0000 | 0.00044 | 3.92 | No | |

## Track 4:

| | |
|---|---|
| Type | Reference |
| Vial ID | CBD 100 |
| Description | CBD 500ng |
| Volume | 5.0 µl |

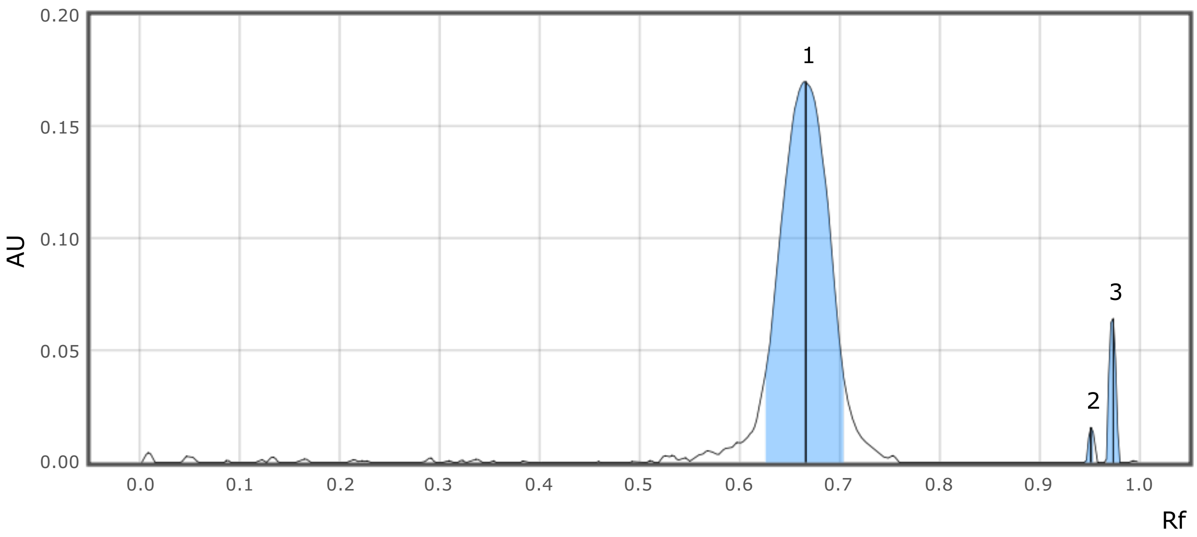


| Peak # | Start Rf | Start H | Max Rf | Max H | Max % | End Rf | End H | Area A | Area % | Manual peak | Substance Name |
|---|---|---|---|---|---|---|---|---|---|---|---|
| 1 | 0.621 | 0.0293 | 0.666 | 0.1701 | 68.02 | 0.707 | 0.0323 | 0.00948 | 93.90 | Yes | CBD |
| 2 | 0.945 | 0.0000 | 0.952 | 0.0157 | 6.26 | 0.959 | 0.0000 | 0.00011 | 1.07 | No | |
| 3 | 0.965 | 0.0000 | 0.974 | 0.0643 | 25.72 | 0.981 | 0.0000 | 0.00051 | 5.03 | No | |

## Track 5:

| | |
|---|---|
| Type | Reference |
| Vial ID | CBN 100 |
| Description | CBN 500ng |
| Volume | 5.0 µl |

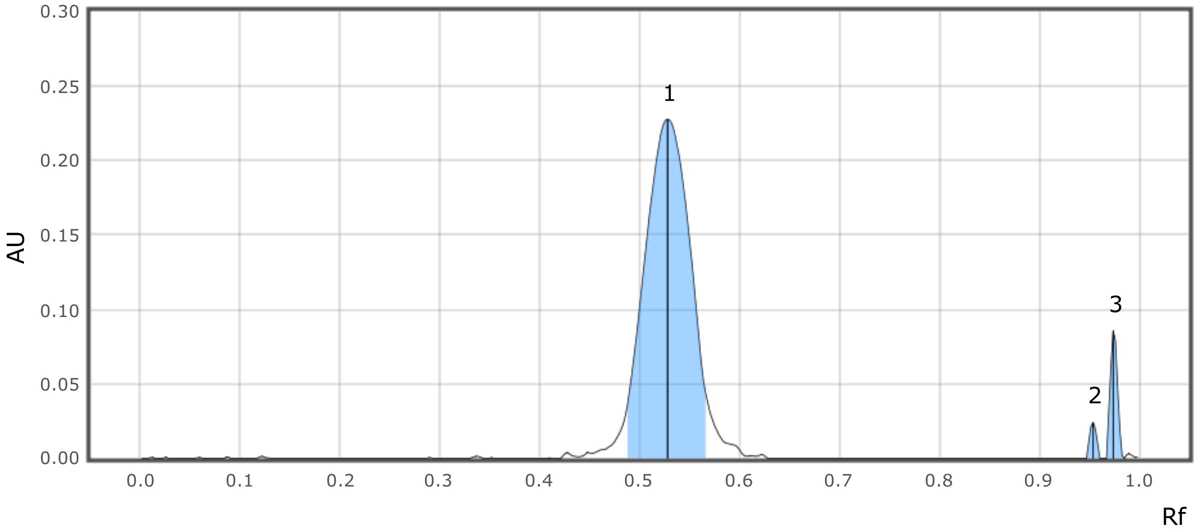


| Peak # | Start Rf | Start H | Max Rf | Max H | Max % | End Rf | End H | Area A | Area % | Manual peak | Substance Name |
|---|---|---|---|---|---|---|---|---|---|---|---|
| 1 | 0.486 | 0.0281 | 0.528 | 0.2275 | 67.46 | 0.570 | 0.0310 | 0.01190 | 92.90 | Yes | CBN |
| 2 | 0.947 | 0.0000 | 0.954 | 0.0242 | 7.17 | 0.961 | 0.0000 | 0.00019 | 1.49 | No | |
| 3 | 0.967 | 0.0000 | 0.974 | 0.0855 | 25.37 | 0.985 | 0.0000 | 0.00072 | 5.61 | No | |

## Track 6:

| | |
|---|---|
| Type | Reference |
| Vial ID | CBG 100 |
| Description | CBG 500ng |
| Volume | 5.0 µl |

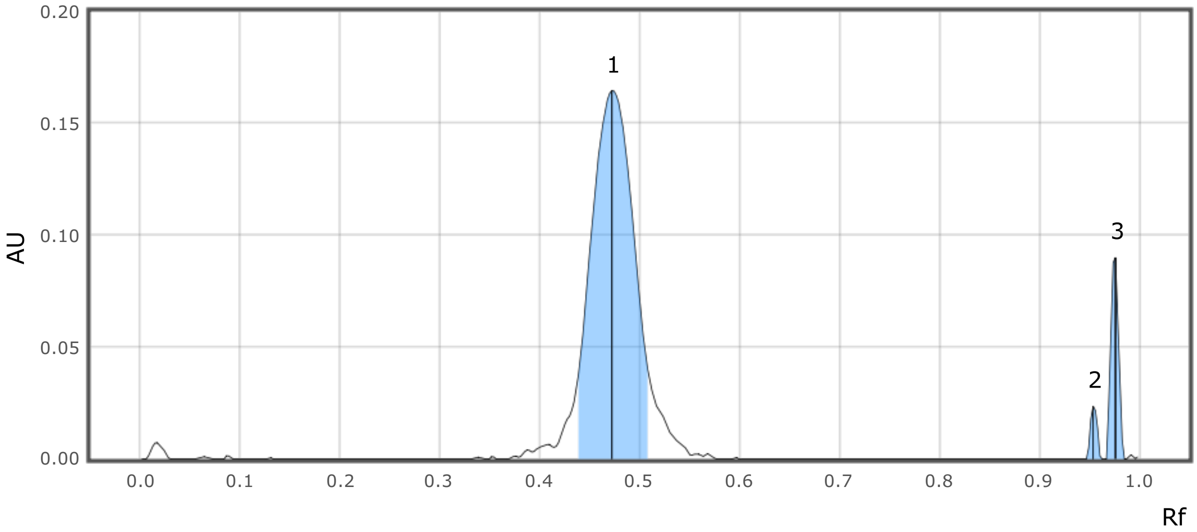


| Peak # | Start Rf | Start H | Max Rf | Max H | Max % | End Rf | End H | Area A | Area % | Manual peak | Substance Name |
|---|---|---|---|---|---|---|---|---|---|---|---|
| 1 | 0.436 | 0.0312 | 0.472 | 0.1647 | 59.21 | 0.512 | 0.0305 | 0.00807 | 89.01 | Yes | CBG |
| 2 | 0.947 | 0.0000 | 0.954 | 0.0235 | 8.45 | 0.963 | 0.0000 | 0.00018 | 2.03 | No | |
| 3 | 0.967 | 0.0000 | 0.976 | 0.0899 | 32.34 | 0.985 | 0.0000 | 0.00081 | 8.96 | No | |

## Track 7:

| | |
|---|---|
| Type | Reference |
| Vial ID | CBC 100 |
| Description | CBC 500ng |
| Volume | 5.0 µl |

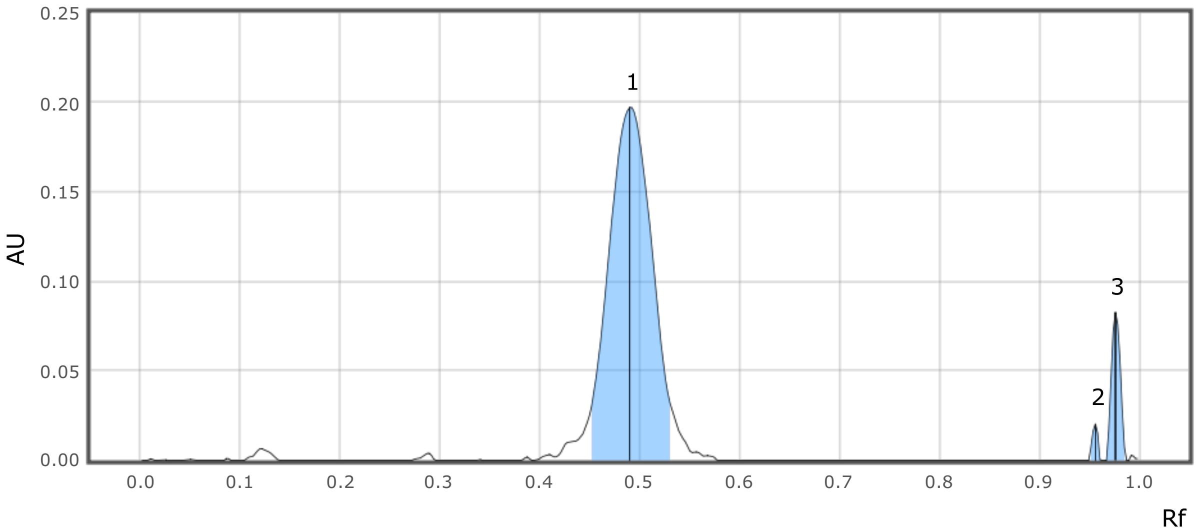


| Peak | Start | | Max | | | End | | Area | | Manual | Substance |
|---|---|---|---|---|---|---|---|---|---|---|---|
| # | Rf | H | Rf | H | % | Rf | H | A | % | peak | Name |
| 1 | 0.451 | 0.0253 | 0.490 | 0.1969 | 65.66 | 0.532 | 0.0284 | 0.00978 | 90.79 | Yes | CBC |
| 2 | 0.950 | 0.0000 | 0.956 | 0.0204 | 6.79 | 0.963 | 0.0000 | 0.00013 | 1.23 | No | |
| 3 | 0.967 | 0.0000 | 0.976 | 0.0826 | 27.55 | 0.988 | 0.0000 | 0.00086 | 7.98 | No | |

## Track 8:

| | |
|---|---|
| Type | Reference |
| Vial ID | THCV 100 |
| Description | THCV 500ng |
| Volume | 5.0 µl |

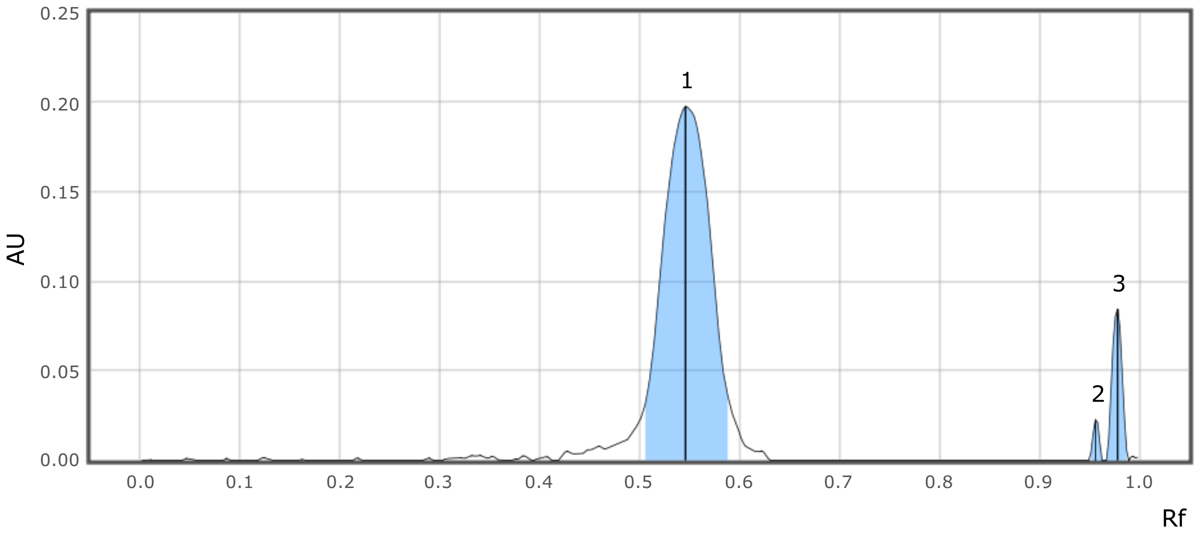

| Peak # | Start | | Max | | | End | | Area | | Manual peak | Substance Name |
|---|---|---|---|---|---|---|---|---|---|---|---|
| | Rf | H | Rf | H | % | Rf | H | A | % | | |
| 1 | 0.505 | 0.0313 | 0.546 | 0.1976 | 64.85 | 0.590 | 0.0310 | 0.01080 | 90.53 | Yes | THCV |
| 2 | 0.950 | 0.0000 | 0.956 | 0.0226 | 7.43 | 0.963 | 0.0000 | 0.00016 | 1.34 | No | |
| 3 | 0.967 | 0.0000 | 0.979 | 0.0845 | 27.72 | 0.990 | 0.0000 | 0.00097 | 8.13 | No | |

## Track 9:

| | |
|---|---|
| Type | Reference |
| Vial ID | CBDV 100 |
| Description | CBDV 500ng |
| Volume | 5.0 µl |

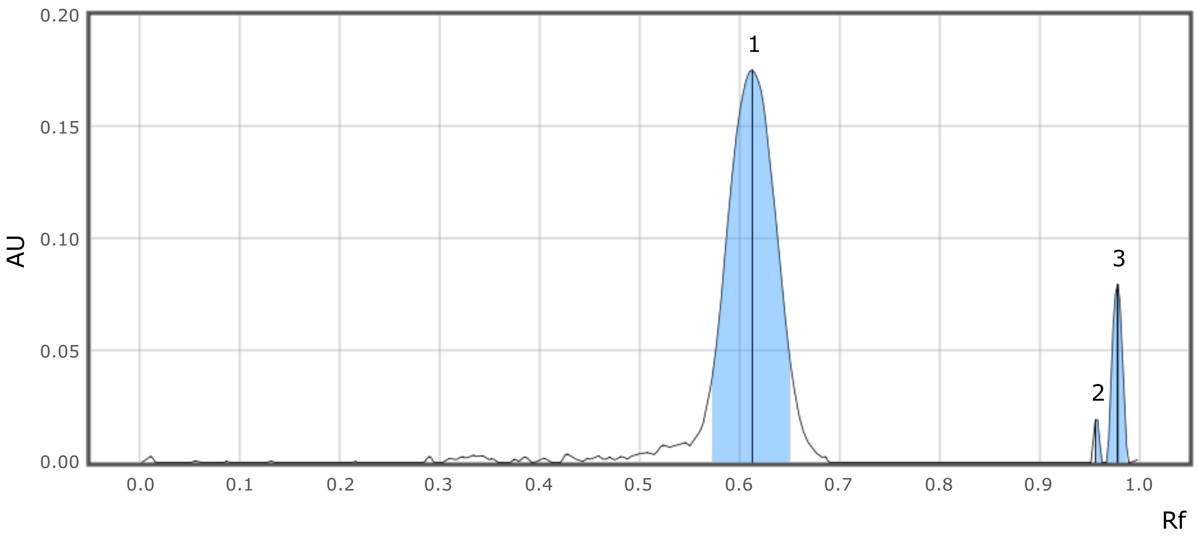


| Peak # | Start | | Max | | | End | | Area | | Manual peak | Substance Name |
|---|---|---|---|---|---|---|---|---|---|---|---|
| | Rf | H | Rf | H | % | Rf | H | A | % | | |
| 1 | 0.571 | 0.0321 | 0.613 | 0.1753 | 63.95 | 0.655 | 0.0319 | 0.00972 | 90.12 | Yes | CBDV |
| 2 | 0.952 | 0.0000 | 0.956 | 0.0191 | 6.97 | 0.963 | 0.0000 | 0.00013 | 1.17 | No | |
| 3 | 0.967 | 0.0000 | 0.979 | 0.0797 | 29.08 | 0.990 | 0.0000 | 0.00094 | 8.71 | No | |

## Track 10:

| | |
|---|---|
| Type | Reference |
| Vial ID | 8-THC 100 |
| Description | D8-THC 500ng |
| Volume | 5.0 µl |

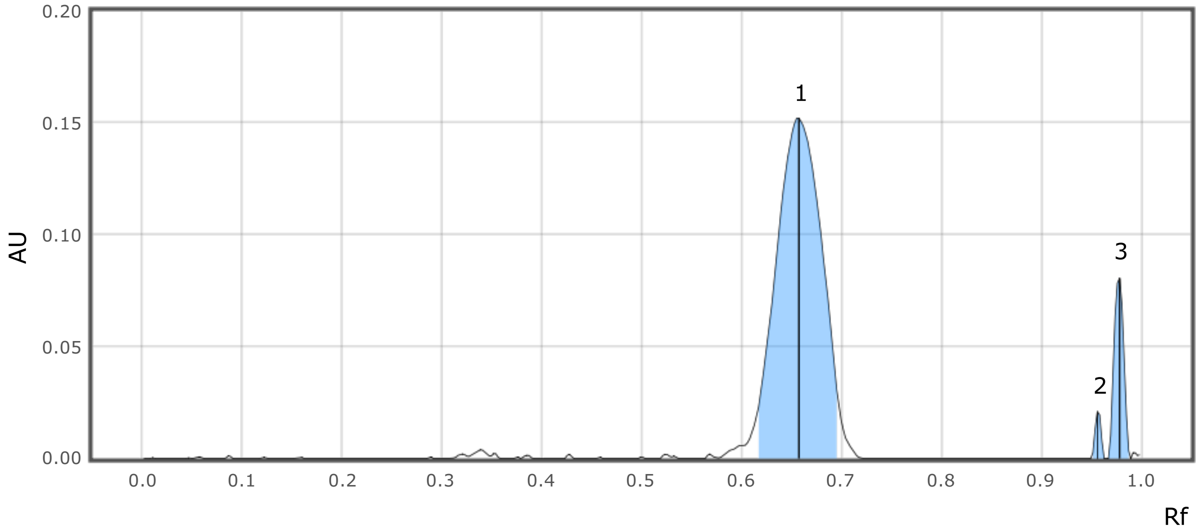


| Peak # | Start Rf | Start H | Max Rf | Max H | Max % | End Rf | End H | Area A | Area % | Manual peak | Substance Name |
|---|---|---|---|---|---|---|---|---|---|---|---|
| 1 | 0.616 | 0.0231 | 0.657 | 0.1520 | 59.96 | 0.697 | 0.0232 | 0.00791 | 88.30 | Yes | 8-THC |
| 2 | 0.950 | 0.0000 | 0.956 | 0.0210 | 8.27 | 0.963 | 0.0000 | 0.00014 | 1.59 | No | |
| 3 | 0.967 | 0.0000 | 0.979 | 0.0805 | 31.77 | 0.990 | 0.0000 | 0.00091 | 10.11 | No | |

## Track 11:

| | |
|---|---|
| Type | Reference |
| Vial ID | THCA-A 100 |
| Description | THCA-A 500ng |
| Volume | 5.0 µl |

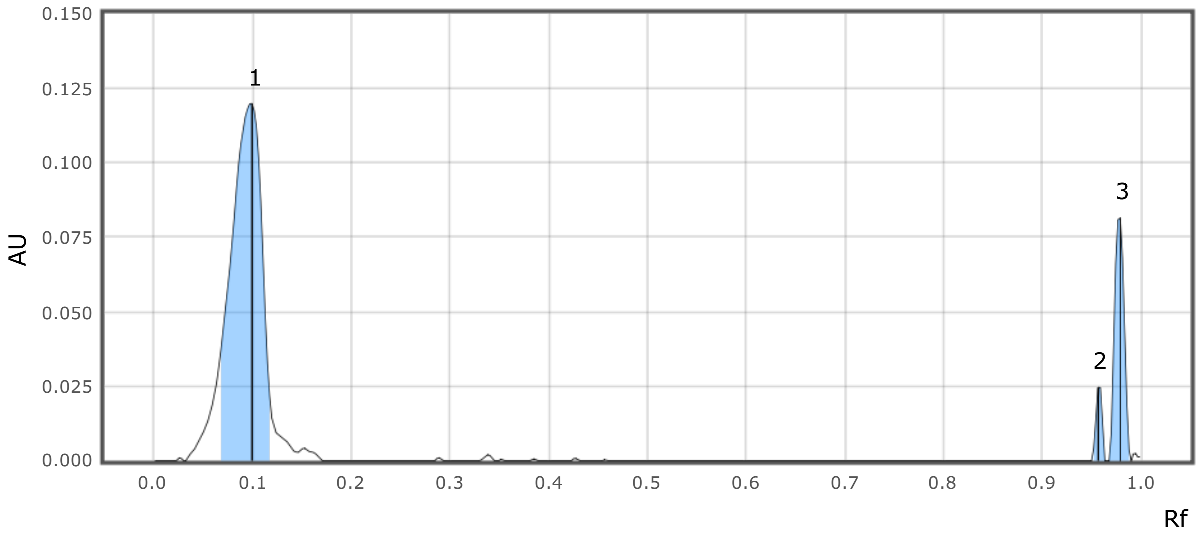


| Peak # | Start Rf | Start H | Max Rf | Max H | Max % | End Rf | End H | Area A | Area % | Manual peak | Substance Name |
|---|---|---|---|---|---|---|---|---|---|---|---|
| 1 | 0.065 | 0.0261 | 0.099 | 0.1197 | 53.06 | 0.118 | 0.0217 | 0.00427 | 79.61 | Yes | THCA-A |
| 2 | 0.950 | 0.0000 | 0.956 | 0.0246 | 10.89 | 0.963 | 0.0000 | 0.00018 | 3.39 | No | |
| 3 | 0.967 | 0.0000 | 0.979 | 0.0813 | 36.05 | 0.990 | 0.0000 | 0.00091 | 17.00 | No | |

## Track 12:

| | |
|---|---|
| Type | Reference |
| Vial ID | CBDA 100 |
| Description | CBDA 500ng |
| Volume | 5.0 µl |

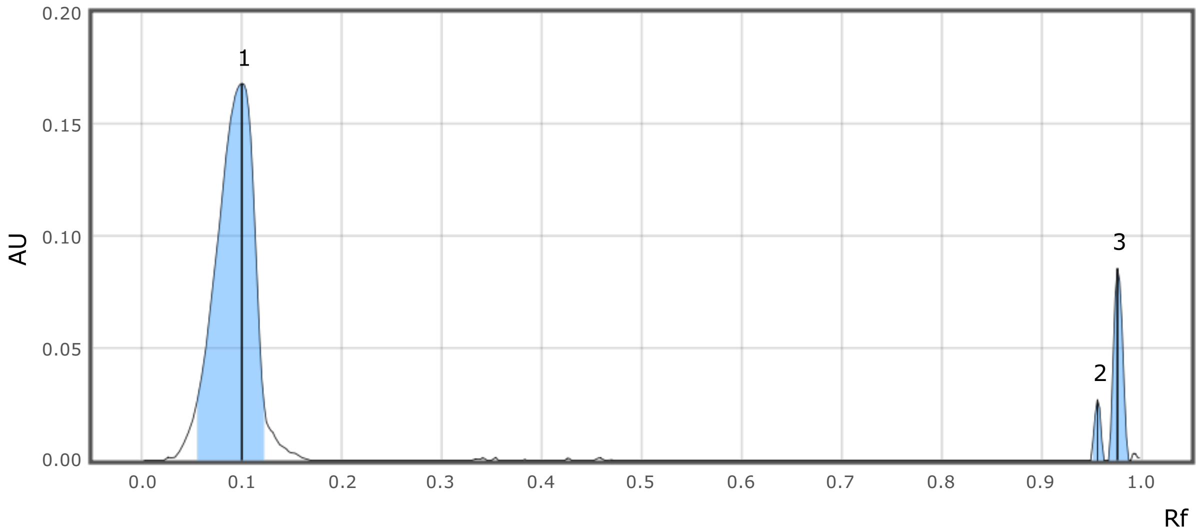


| Peak # | Start Rf | Start H | Max Rf | Max H | Max % | End Rf | End H | Area A | Area % | Manual peak | Substance Name |
|---|---|---|---|---|---|---|---|---|---|---|---|
| 1 | 0.055 | 0.0266 | 0.099 | 0.1683 | 59.87 | 0.123 | 0.0249 | 0.00716 | 86.81 | Yes | CBDA |
| 2 | 0.950 | 0.0000 | 0.956 | 0.0271 | 9.65 | 0.963 | 0.0000 | 0.00020 | 2.37 | No | |
| 3 | 0.967 | 0.0000 | 0.976 | 0.0857 | 30.47 | 0.990 | 0.0000 | 0.00089 | 10.82 | No | |

## Track 13:

| | |
|---|---|
| Type | Reference |
| Vial ID | CBGA 100 |
| Description | CBGA 500ng |
| Volume | 5.0 µl |

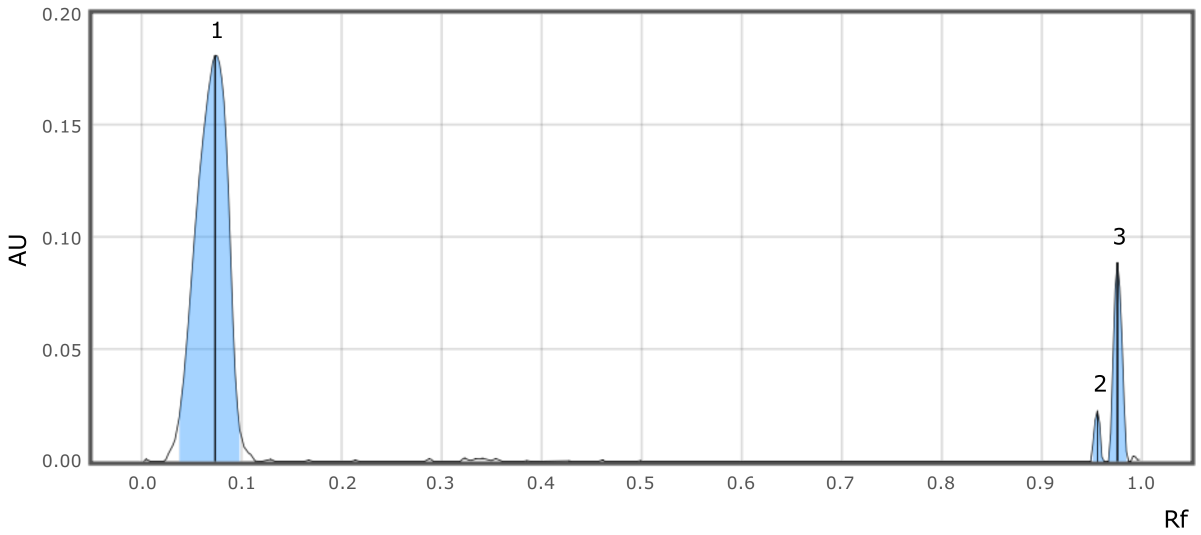

| Peak # | Start | | Max | | | End | | Area | | Manual peak | Substance Name |
|---|---|---|---|---|---|---|---|---|---|---|---|
| | Rf | H | Rf | H | % | Rf | H | A | % | | |
| 1 | 0.033 | 0.0096 | 0.073 | 0.1813 | 61.93 | 0.099 | 0.0102 | 0.00682 | 87.07 | Yes | CBGA |
| 2 | 0.950 | 0.0000 | 0.956 | 0.0226 | 7.72 | 0.963 | 0.0000 | 0.00015 | 1.92 | No | |
| 3 | 0.967 | 0.0000 | 0.976 | 0.0888 | 30.35 | 0.988 | 0.0000 | 0.00086 | 11.01 | No | |

## Track 14:

| | |
|---|---|
| Type | Sample |
| Vial ID | Mixture 100 |
| Description | Mixture 500ng |
| Volume | 5.0 µl |

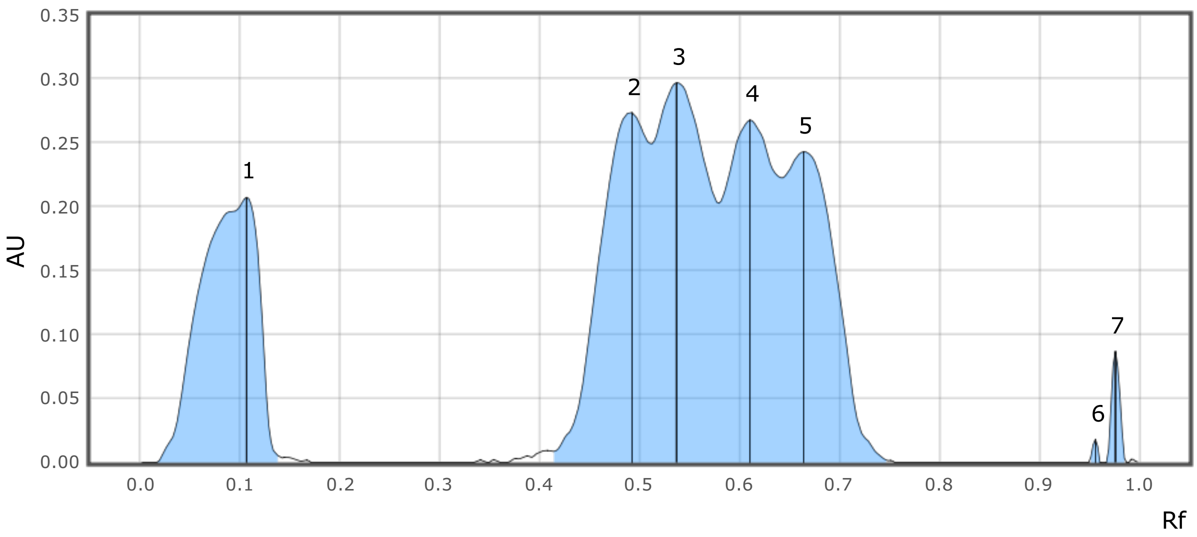


| Peak # | Start | | Max | | | End | | Area | | Manual peak | Substance Name |
|---|---|---|---|---|---|---|---|---|---|---|---|
| | Rf | H | Rf | H | % | Rf | H | A | % | | |
| 1 | 0.015 | 0.0000 | 0.106 | 0.2073 | 14.88 | 0.142 | 0.0036 | 0.01424 | 18.41 | No | |
| 2 | 0.414 | 0.0086 | 0.492 | 0.2736 | 19.64 | 0.512 | 0.2492 | 0.01554 | 20.09 | No | |
| 3 | 0.512 | 0.2492 | 0.537 | 0.2968 | 21.30 | 0.577 | 0.2035 | 0.01709 | 22.09 | No | |
| 4 | 0.579 | 0.2027 | 0.610 | 0.2678 | 19.22 | 0.642 | 0.2226 | 0.01512 | 19.54 | No | |
| 5 | 0.642 | 0.2226 | 0.664 | 0.2429 | 17.43 | 0.749 | 0.0014 | 0.01445 | 18.67 | No | |
| 6 | 0.950 | 0.0000 | 0.956 | 0.0180 | 1.29 | 0.961 | 0.0000 | 0.00011 | 0.14 | No | |
| 7 | 0.967 | 0.0000 | 0.976 | 0.0869 | 6.24 | 0.988 | 0.0000 | 0.00083 | 1.07 | No | |

## Track 15:

| | |
|---|---|
| Type | Sample |
| Vial ID | MeOH blank |
| Description | MeOH Blank |
| Volume | 2.0 µl |

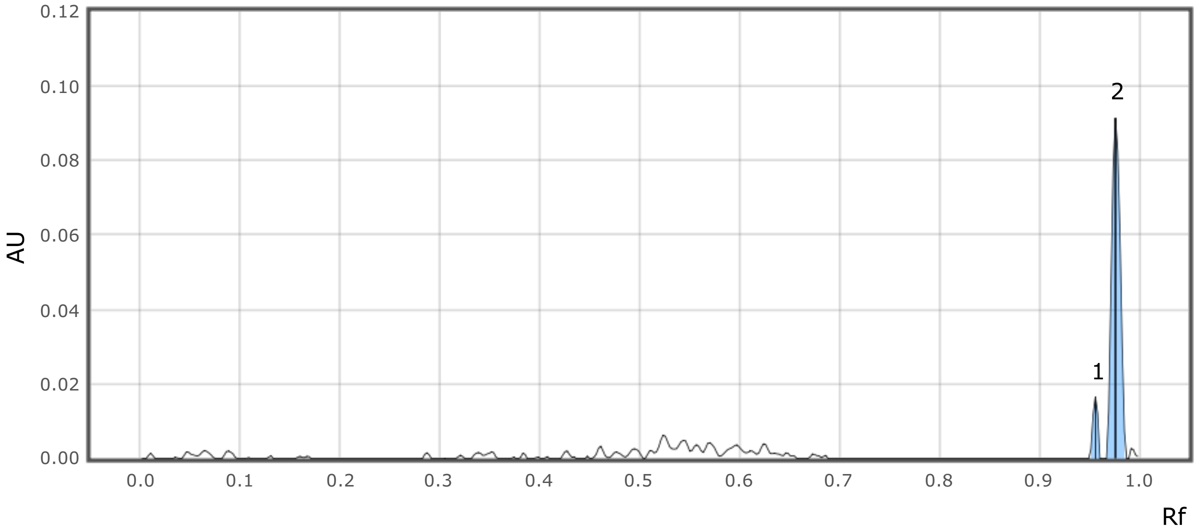


| Peak # | Start | | Max | | | End | | Area | | Manual peak | Substance Name |
|---|---|---|---|---|---|---|---|---|---|---|---|
| | Rf | H | Rf | H | % | Rf | H | A | % | | |
| 1 | 0.950 | 0.0000 | 0.956 | 0.0165 | 15.33 | 0.961 | 0.0000 | 0.00010 | 9.49 | No | |
| 2 | 0.967 | 0.0000 | 0.976 | 0.0913 | 84.67 | 0.988 | 0.0000 | 0.00091 | 90.51 | No | |

# Calibration results:

## Height calibration for substance 8-THC @ :

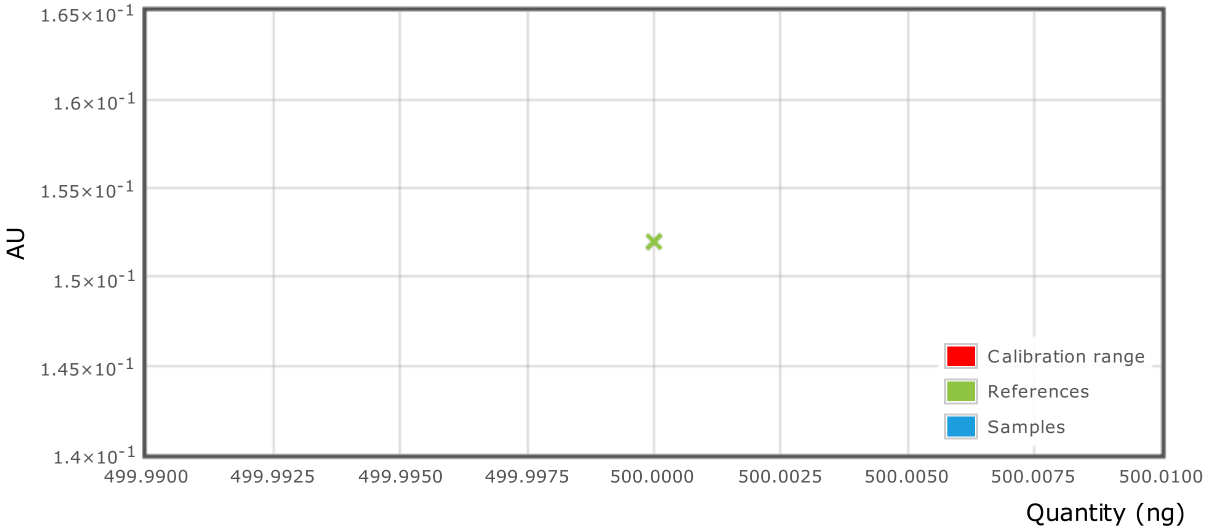

| | |
|---|---|
| Regression mode | Linear-2 |
| Range deviation | 5.00 % |
| Related substances | Default |
| Number of references | 1 |
| Calibration function | $y=0x$ |
| Coefficient of variation | CV 0.00 % |
| Correlation coefficient | n/a |
| ⚠ | Unable to compute the results for this substance because there wasn't enough groups of references replicas (at least 1 for Linear-1, 2 for Linear2 and Mime-1 and 3 for Polynomial and MiMe-2) |

## Height calibration for substance 9-THC @ :

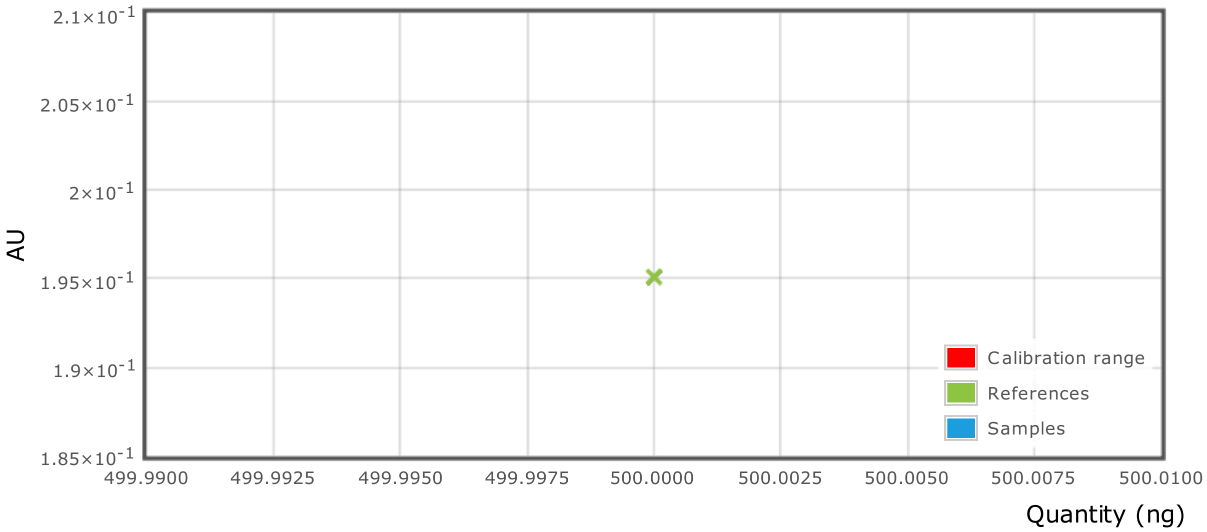


| | |
|---|---|
| Regression mode | Linear-2 |
| Range deviation | 5.00 % |
| Related substances | Default |
| Number of references | 1 |
| Calibration function | $y=0x$ |
| Coefficient of variation | CV 0.00 % |
| Correlation coefficient | n/a |
| ⚠ | Unable to compute the results for this substance because there wasn't enough groups of references replicas (at least 1 for Linear-1, 2 for Linear2 and Mime-1 and 3 for Polynomial and MiMe-2) |

## Height calibration for substance CBC @ :

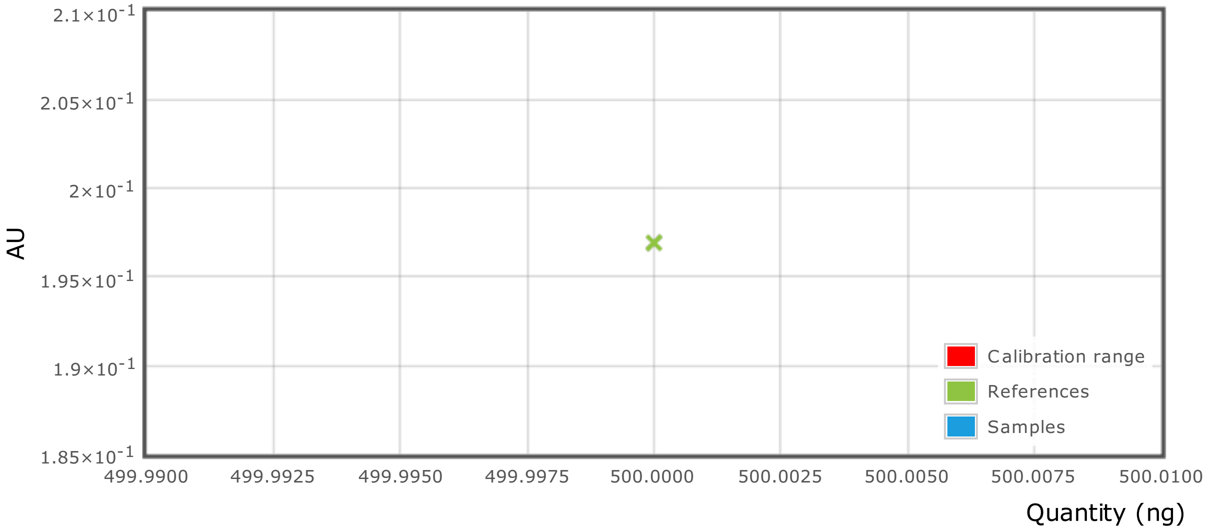


| | |
|---|---|
| Regression mode | Linear-2 |
| Range deviation | 5.00 % |
| Related substances | Default |
| Number of references | 1 |
| Calibration function | $y=0x$ |
| Coefficient of variation | CV 0.00 % |
| Correlation coefficient | n/a |
| ⚠ | Unable to compute the results for this substance because there wasn't eno ugh groups of references replicas (at least 1 for Linear-1, 2 for Linear2 and Mime-1 and 3 for Polynomial and MiMe-2) |

## Height calibration for substance CBD @ :

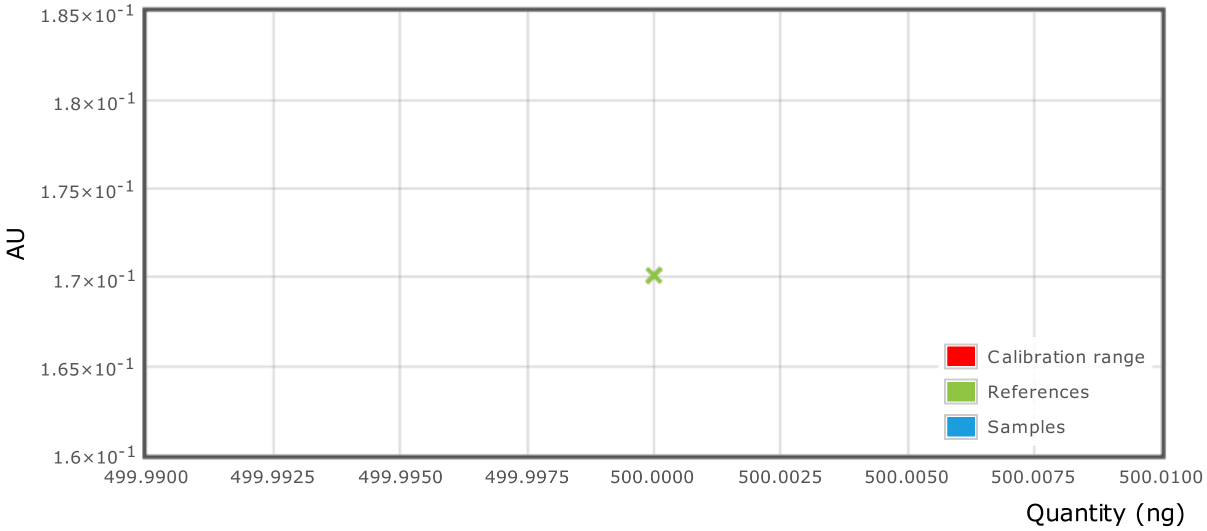

| | |
|---|---|
| Regression mode | Linear-2 |
| Range deviation | 5.00 % |
| Related substances | Default |
| Number of references | 1 |
| Calibration function | $y=0x$ |
| Coefficient of variation | CV 0.00 % |
| Correlation coefficient | n/a |
| ⚠ | Unable to compute the results for this substance because there wasn't enough groups of references replicas (at least 1 for Linear-1, 2 for Linear2 and Mime-1 and 3 for Polynomial and MiMe-2) |

## Height calibration for substance CBDA @ :

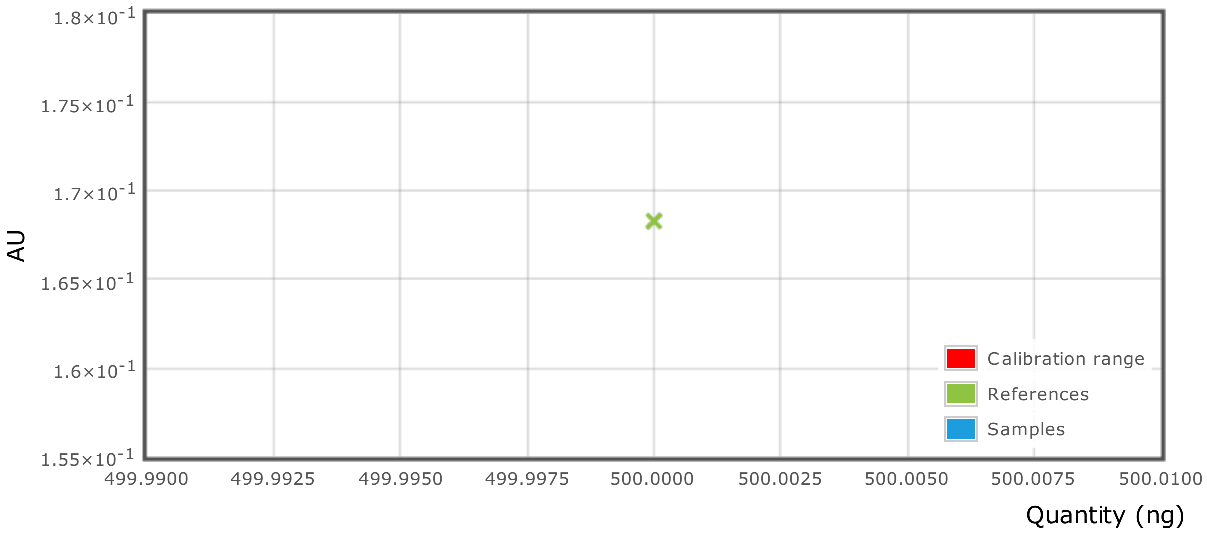


| | |
|---|---|
| Regression mode | Linear-2 |
| Range deviation | 5.00 % |
| Related substances | Default |
| Number of references | 1 |
| Calibration function | $y=0x$ |
| Coefficient of variation | CV 0.00 % |
| Correlation coefficient | n/a |
| ⚠ | Unable to compute the results for this substance because there wasn't enough groups of references replicas (at least 1 for Linear-1, 2 for Linear2 and Mime-1 and 3 for Polynomial and MiMe-2) |

## Height calibration for substance CBDV @ :

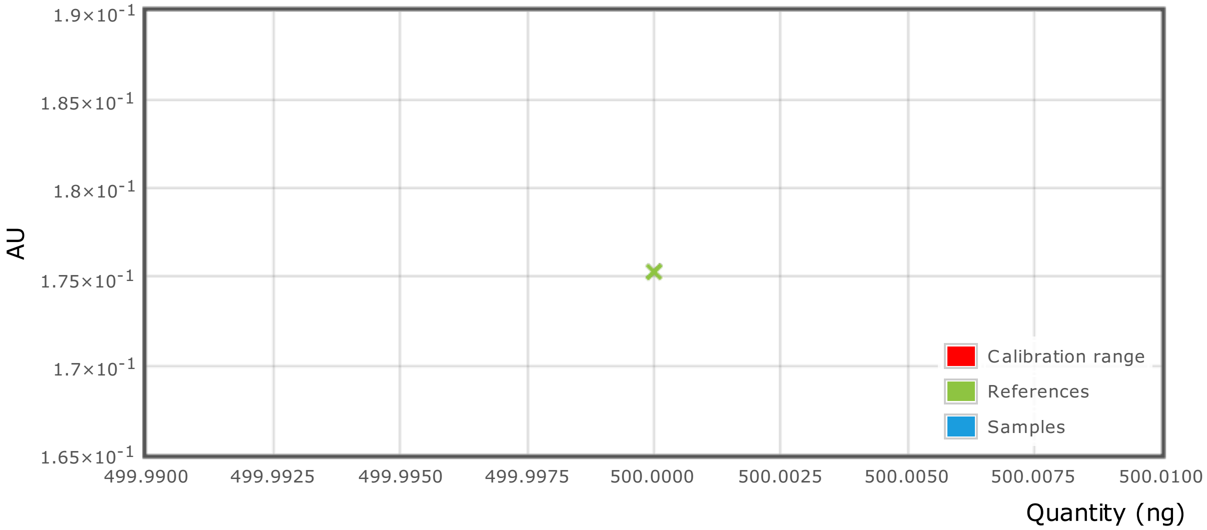


| | |
|---|---|
| Regression mode | Linear-2 |
| Range deviation | 5.00 % |
| Related substances | Default |
| Number of references | 1 |
| Calibration function | $y=0x$ |
| Coefficient of variation | CV 0.00 % |
| Correlation coefficient | n/a |
| ⚠ | Unable to compute the results for this substance because there wasn't eno ugh groups of references replicas (at least 1 for Linear-1, 2 for Linear2 and Mime-1 and 3 for Polynomial and MiMe-2) |

## Height calibration for substance CBG @ :

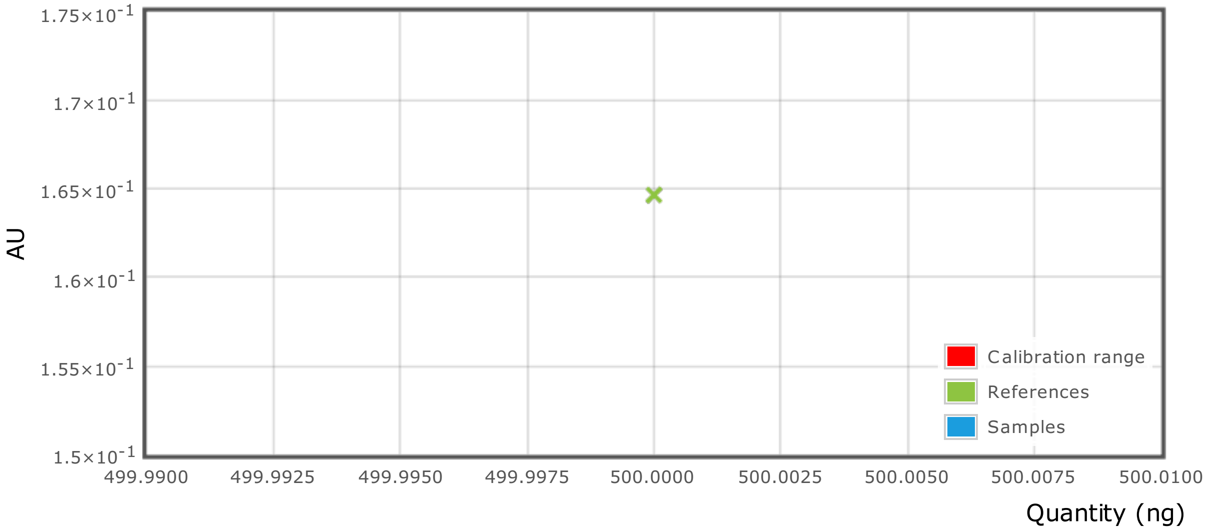

| | |
|---|---|
| Regression mode | Linear-2 |
| Range deviation | 5.00 % |
| Related substances | Default |
| Number of references | 1 |
| Calibration function | $y=0x$ |
| Coefficient of variation | CV 0.00 % |
| Correlation coefficient | n/a |
| ⚠ | Unable to compute the results for this substance because there wasn't enough groups of references replicas (at least 1 for Linear-1, 2 for Linear2 and Mime-1 and 3 for Polynomial and MiMe-2) |

## Height calibration for substance CBGA @ :

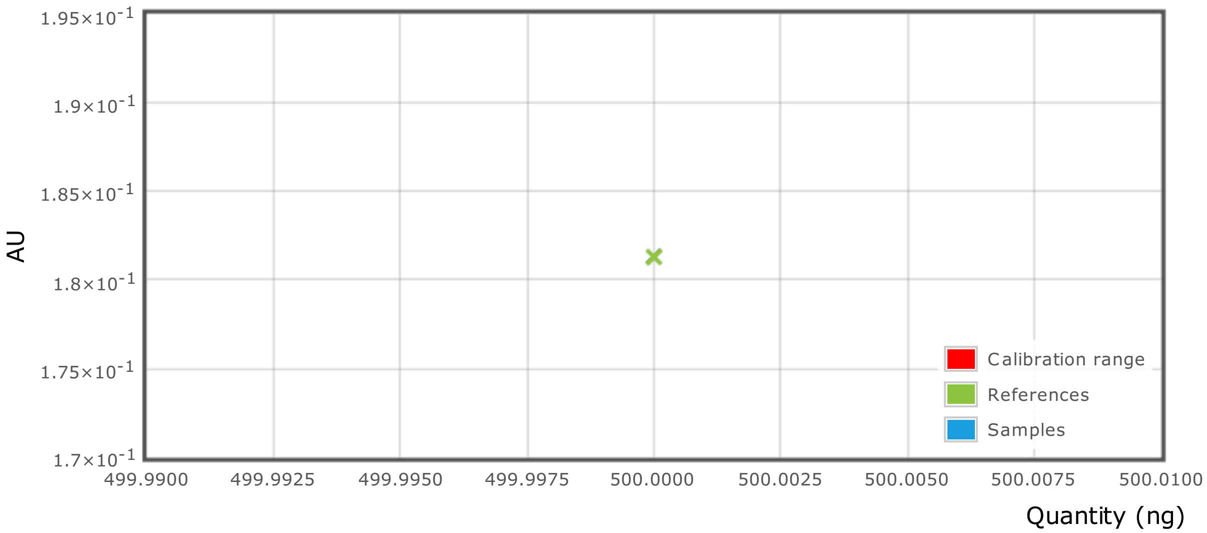


| | |
|---|---|
| Regression mode | Linear-2 |
| Range deviation | 5.00 % |
| Related substances | Default |
| Number of references | 1 |
| Calibration function | $y=0x$ |
| Coefficient of variation | CV 0.00 % |
| Correlation coefficient | n/a |
| ⚠ | Unable to compute the results for this substance because there wasn't enough groups of references replicas (at least 1 for Linear-1, 2 for Linear2 and Mime-1 and 3 for Polynomial and MiMe-2) |

## Height calibration for substance CBN @ :

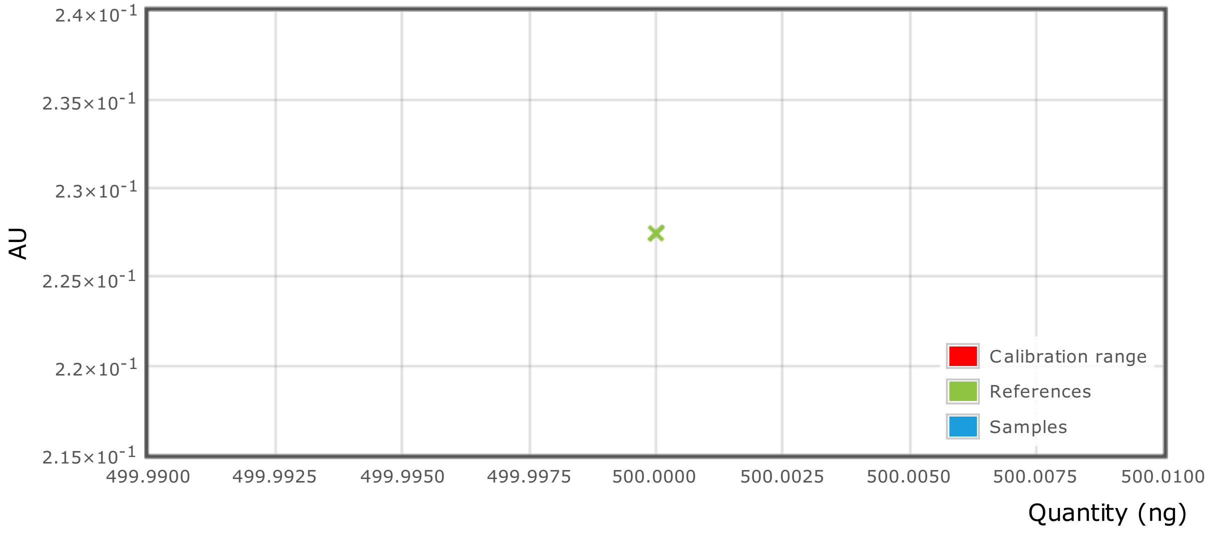


| | |
|---|---|
| Regression mode | Linear-2 |
| Range deviation | 5.00 % |
| Related substances | Default |
| Number of references | 1 |
| Calibration function | $y=0x$ |
| Coefficient of variation | CV 0.00 % |
| Correlation coefficient | n/a |
| ⚠ | Unable to compute the results for this substance because there wasn't enough groups of references replicas (at least 1 for Linear-1, 2 for Linear2 and Mime-1 and 3 for Polynomial and MiMe-2) |

## Height calibration for substance THCA-A @ :

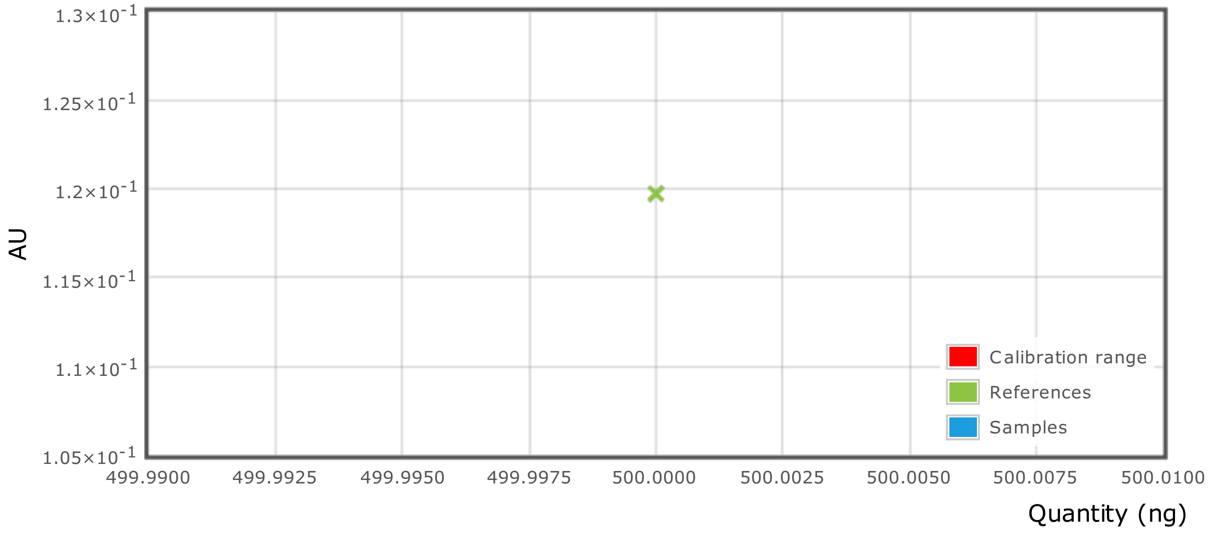

| | |
|---|---|
| Regression mode | Linear-2 |
| Range deviation | 5.00 % |
| Related substances | Default |
| Number of references | 1 |
| Calibration function | $y=0x$ |
| Coefficient of variation | CV 0.00 % |
| Correlation coefficient | n/a |
| ⚠ | Unable to compute the results for this substance because there wasn't enough groups of references replicas (at least 1 for Linear-1, 2 for Linear2 and Mime-1 and 3 for Polynomial and MiMe-2) |

## Height calibration for substance THCV @ :

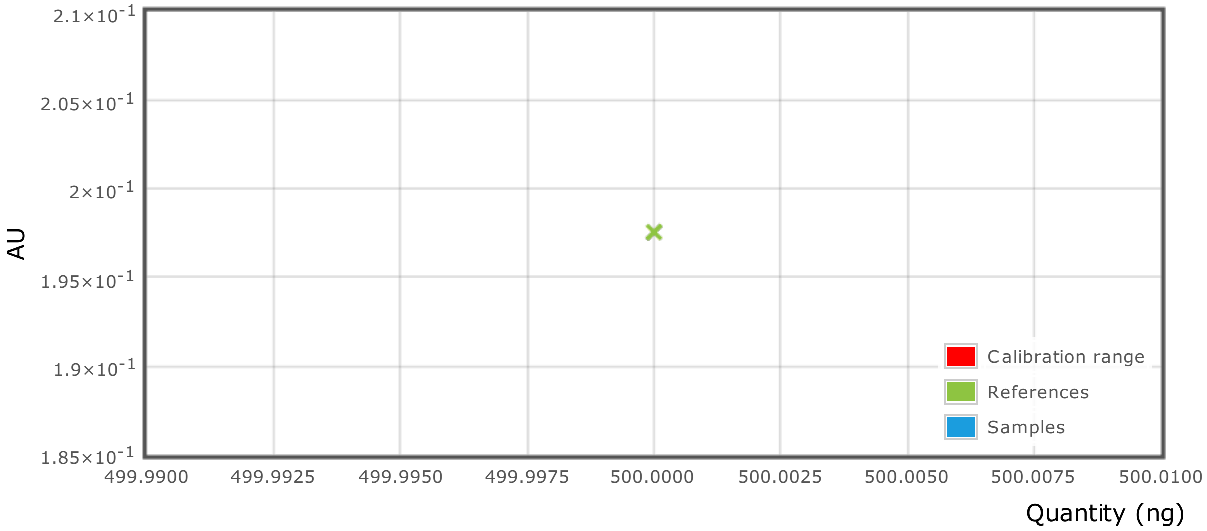


| | |
|---|---|
| Regression mode | Linear-2 |
| Range deviation | 5.00 % |
| Related substances | Default |
| Number of references | 1 |
| Calibration function | $y=0x$ |
| Coefficient of variation | CV 0.00 % |
| Correlation coefficient | n/a |
| ⚠ | Unable to compute the results for this substance because there wasn't enough groups of references replicas (at least 1 for Linear-1, 2 for Linear2 and Mime-1 and 3 for Polynomial and MiMe-2) |

## Results:

| Substance having no available results | | |
|---|---|---|
| ⚠ | CBDA | There wasn't any sample application available in the assignments for this substance. Please check that the peaks were correctly detected and assig ned for this substance. |
| ⚠ | THCA-A | There wasn't any sample application available in the assignments for this substance. Please check that the peaks were correctly detected and assig ned for this substance. |
| ⚠ | 9-THC | There wasn't any sample application available in the assignments for this substance. Please check that the peaks were correctly detected and assig ned for this substance. |
| ⚠ | 8-THC | There wasn't any sample application available in the assignments for this substance. Please check that the peaks were correctly detected and assig ned for this substance. |
| ⚠ | CBG | There wasn't any sample application available in the assignments for this substance. Please check that the peaks were correctly detected and assig ned for this substance. |
| ⚠ | CBD | There wasn't any sample application available in the assignments for this substance. Please check that the peaks were correctly detected and assig ned for this substance. |
| ⚠ | CBDV | There wasn't any sample application available in the assignments for this substance. Please check that the peaks were correctly detected and assig ned for this substance. |
| ⚠ | THCV | There wasn't any sample application available in the assignments for this substance. Please check that the peaks were correctly detected and assig ned for this substance. |
| ⚠ | CBN | There wasn't any sample application available in the assignments for this substance. Please check that the peaks were correctly detected and assig ned for this substance. |
| ⚠ | CBGA | There wasn't any sample application available in the assignments for this substance. Please check that the peaks were correctly detected and assig ned for this substance. |
| ⚠ | CBC | There wasn't any sample application available in the assignments for this substance. Please check that the peaks were correctly detected and assig ned for this substance. |

A track marked with ⚠ means: this result is outside the regression range given by the reference assignments, but is included in the results because it is in the allowed range deviation.

| Analyst: | Reviewer: |
|---|---|
