# Supplementary material for: High Performance Thin-Layer Chromatography (HPTLC) data of Cannabinoids in ten mobile phase systems
Source: Data Brief. 2020 Jun 30;31:105955. doi: 10.1016/j.dib.2020.105955 (PMC7352075; doi:10.1016/j.dib.2020.105955)
Supplement: Supplementary file 1 [file mmc1.zip › S1-Triplicate reports/HDe-3.pdf]

Analysis: HDe-re-3

Path: Home/YL Research

Based on method: Triplets Method

Created	07-Jun-2019 14:38:52	visionCATSuser
Modified	07-Jun-2019 16:51:42	visionCATSuser
Last HPTLC log	07-Jun-2019 16:51:42	Analysis modified
Explorer notes		

Track	Vial ID	Description	Volume	Position	Type
1	MeOH blank	MeOH Blank	2.0 µl	A1	Sample
2	Mixture 100	Mixture 500ng	5.0 µl	A2	Sample
3	9-THC 100	D9-THC 500ng	5.0 µl	A3	Reference
4	CBD 100	CBD 500ng	5.0 µl	A4	Reference
5	CBN 100	CBN 500ng	5.0 µl	A5	Reference
6	CBG 100	CBG 500ng	5.0 µl	A6	Reference
7	CBC 100	CBC 500ng	5.0 µl	A7	Reference
8	THCV 100	THCV 500ng	5.0 µl	A8	Reference
9	CBDV 100	CBDV 500ng	5.0 µl	A9	Reference
10	8-THC 100	D8-THC 500ng	5.0 µl	A10	Reference
11	THCA-A 100	THCA-A 500ng	5.0 µl	A11	Reference
12	CBDA 100	CBDA 500ng	5.0 µl	B1	Reference
13	CBGA 100	CBGA 500ng	5.0 µl	B2	Reference
14	Mixture 100	Mixture 500ng	5.0 µl	A2	Sample
15	MeOH blank	MeOH Blank	2.0 µl	A1	Sample
Sequence table notes					

A track marked with ⚠ means: the application type is overridden in some evaluation(s).

System setup:

Software	Server User-PC, version 2.5.18072.1
ATS4	S/N:080713
Chamber	N/A
Derivatization dip	N/A
Scanner3	S/N:031025
Visualizer	S/N:230515

Chromatography

Plate layout:

Stationary phase	Merck, HPTLC plates silica gel 60 F 254
Plate format	200.0 x 100.0 mm
Application type	User
Application	Position Y: 10.0 mm, length: 8.0 mm, width: 0.0 mm
Track	First position X: 20.0 mm, distance: 11.4 mm
Solvent front position	70.0 mm
Notes	

Take image clean plate 1a - Visualizer (S/N: 230515):

Quality	Enhanced
RT White	auto capture, Auto, level 85 %, Band
R 254	auto capture, Auto, level 85 %, Band
Instrument diagnostics	Valid diagnostics
Documentation step label	
Notes	

Application 1 - ATS 4 (S/N: 080713):

Spray gas	NI
Sample solvent type	Methanol
Filling speed	15 µl/s
Predosage volume	200 nl
Retraction volume	200 nl
Dosage speed	150 nl/s
Filling quality	User
Rinsing cycles / vacuum	1 / 4 s
Filling cycles / vacuum	1 / 4 s
Rinsing solvent name	Methanol
Nozzle temperature	Unheated
Rack in use	Standard
Instrument diagnostics	Valid diagnostics
Notes	

Development 1 - Chamber:

Tank	TTC 20x10
Mobile phase	
Saturation time	20 min
Use saturation pad	true
Use smartALERT	false
Volume front through	10 ml
Volume rear through	20 ml
Drying time	5 min
Drying temperature	Room temperature
Notes	

Take image developed plate 1a - Visualizer (S/N: 230515):

Quality	Enhanced
RT White	auto capture, Auto, level 85 %, Band
R 254	auto capture, Auto, level 85 %, Band
R 366	auto capture, Auto, level 85 %, Band
Instrument diagnostics	Valid diagnostics
Documentation step label	
Notes	

Scan developed plate 1b - Scanner 3 (S/N: 031025):

Scanner type	Single λ
Optimization for	Resolution
Measurement mode	Absorption
Filter	n/a
Detector mode	Automatic
Scanning speed	20 mm/s
Data resolution	100 µm/step
Slit	5 x 0.2 mm, micro
Partial scan	No
Lamp	Deuterium & Tungsten
Wavelength(s)	254 nm
Instrument diagnostics	Valid diagnostics
Documentation step label	
Notes	

Derivatization 1 - dip:

Reagent name	Fast Blue B salt
Dipping speed	3
Dipping time	5 s
Reagent preparation	1g Fast Blue B salt in 200mL water
Heating	none
Notes	Air dry for 5 minutes

Take image derivatized plate 1a - Visualizer (S/N: 230515):

Quality	Enhanced
RT White	auto capture, Auto, level 85 %, Band
R 366	auto capture, Auto, level 85 %, Band
Instrument diagnostics	Valid diagnostics
Documentation step label	
Notes	

System suitability tests:

SST settings:

SST tracks	

Data acquisition

Application 1 - ATS 4 (S/N: 080713):

Executed	07-Jun-2019 14:41:07 visionCATSuser

Development 1 - Chamber:

Executed	07-Jun-2019 15:25:40 visionCATSuser

Take image developed plate 1a - Visualizer (S/N: 230515):

Executed	07-Jun-2019 16:13:00 visionCATSuser

RT White Developed, RemTransVis

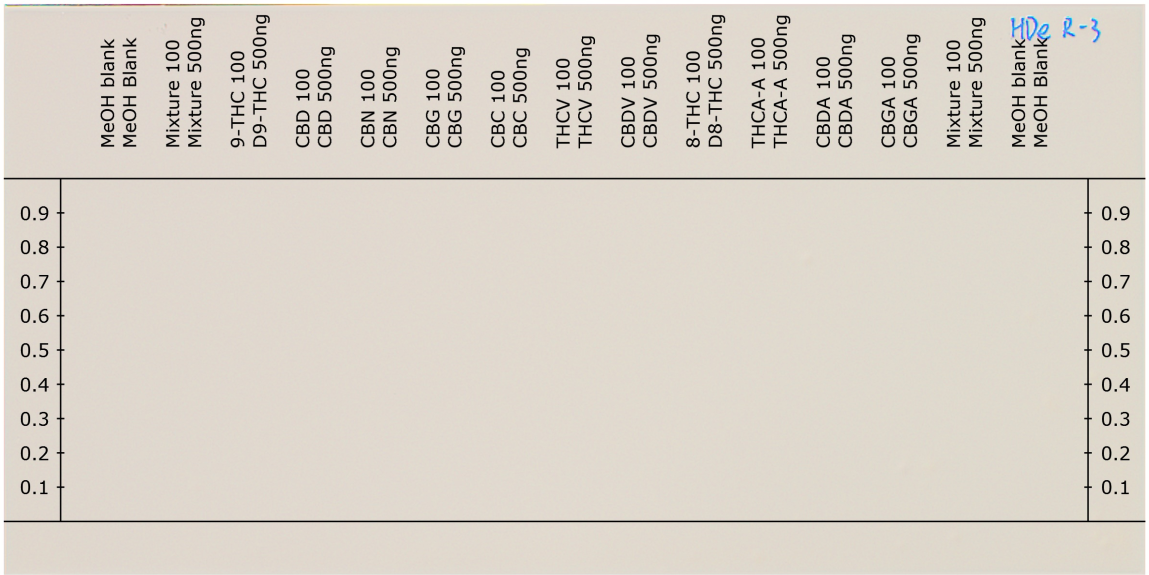

Exposure	0.089 s
Contrast	1
Normalized exposure	Disabled
Clarify	Disabled
White balance	1.00, 1.00, 1.00

R 254 Developed, Remission254

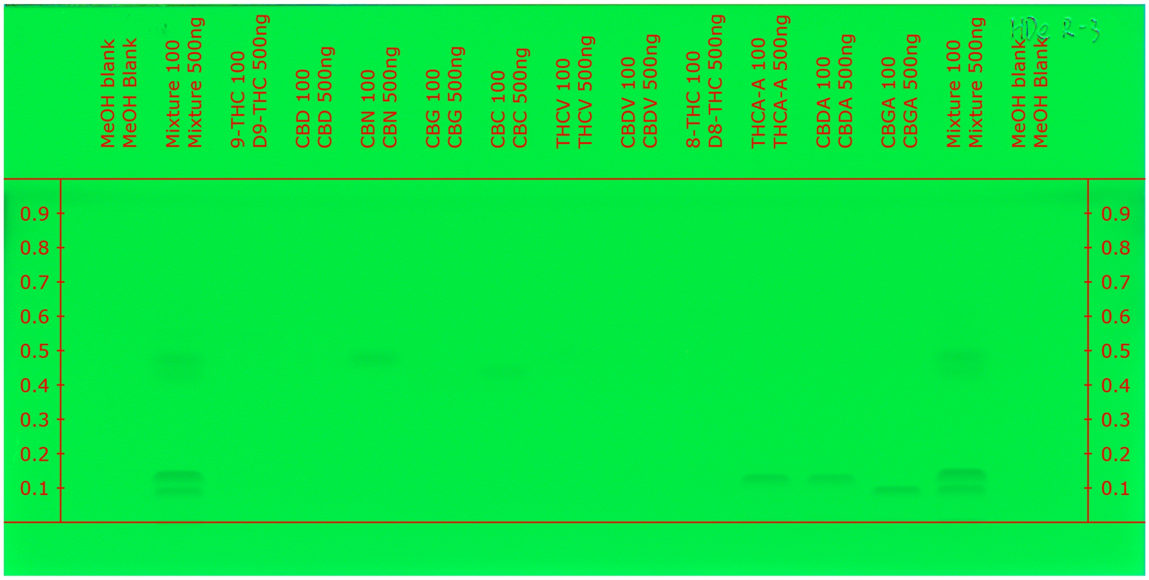

Exposure	0.270 s
Contrast	1
Normalized exposure	Disabled
Clarify	Disabled
White balance	1.00, 1.00, 1.00

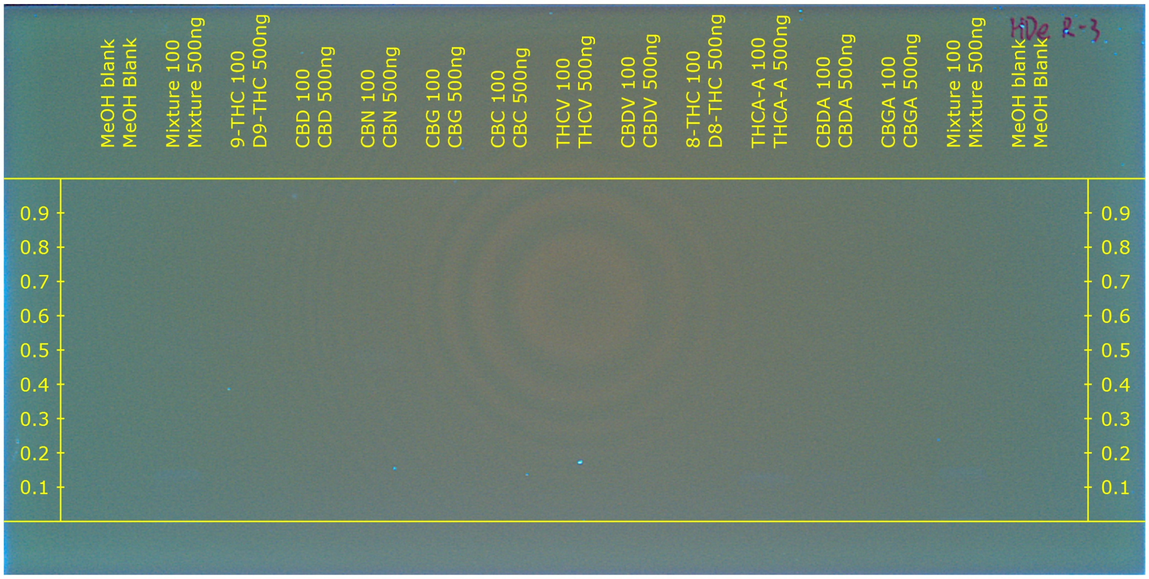

Exposure	9.999 s
Contrast	1
Normalized exposure	Disabled
Clarify	Disabled
White balance	1.00, 1.00, 1.00

Scan developed plate 1b - Scanner 3 (S/N: 031025):

Executed	07-Jun-2019 16:20:21 visionCATSuser

Scan:

Wavelength	254 nm

Track 1:

Type	Single λ

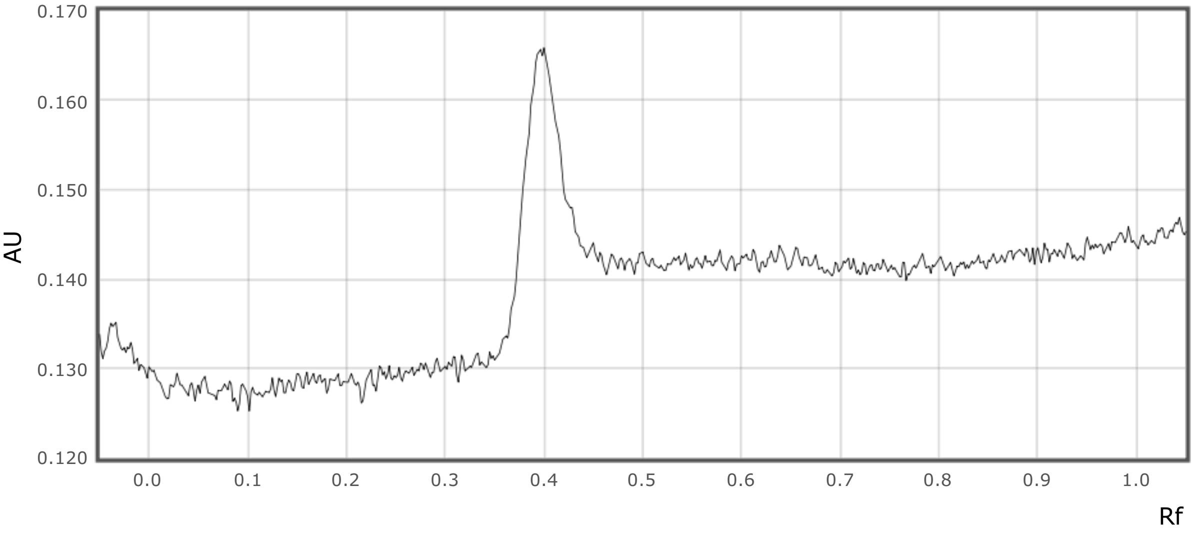

Track 2:

Type	Single λ

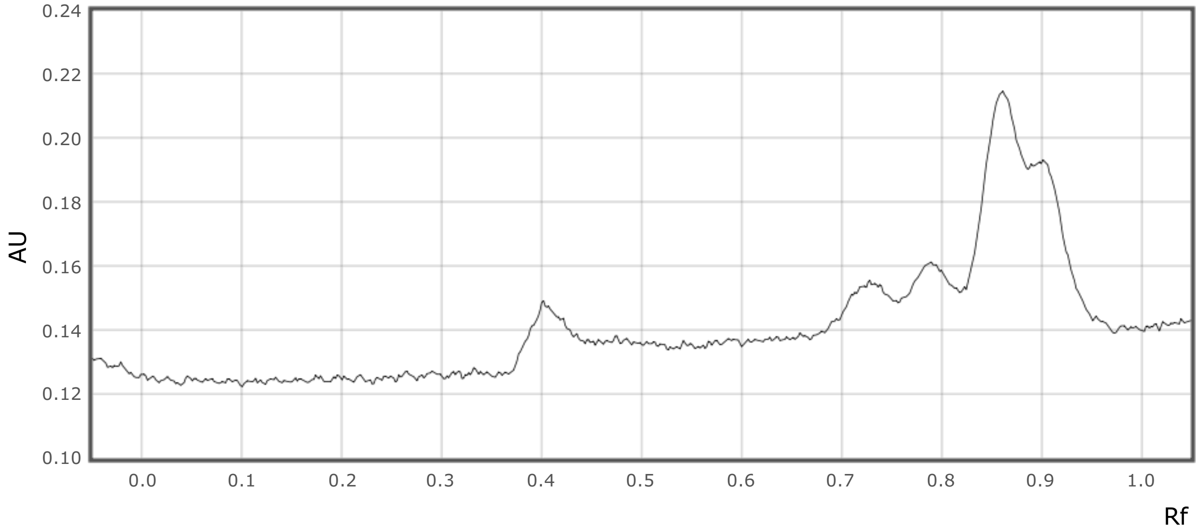

Track 3:

Type	Single λ

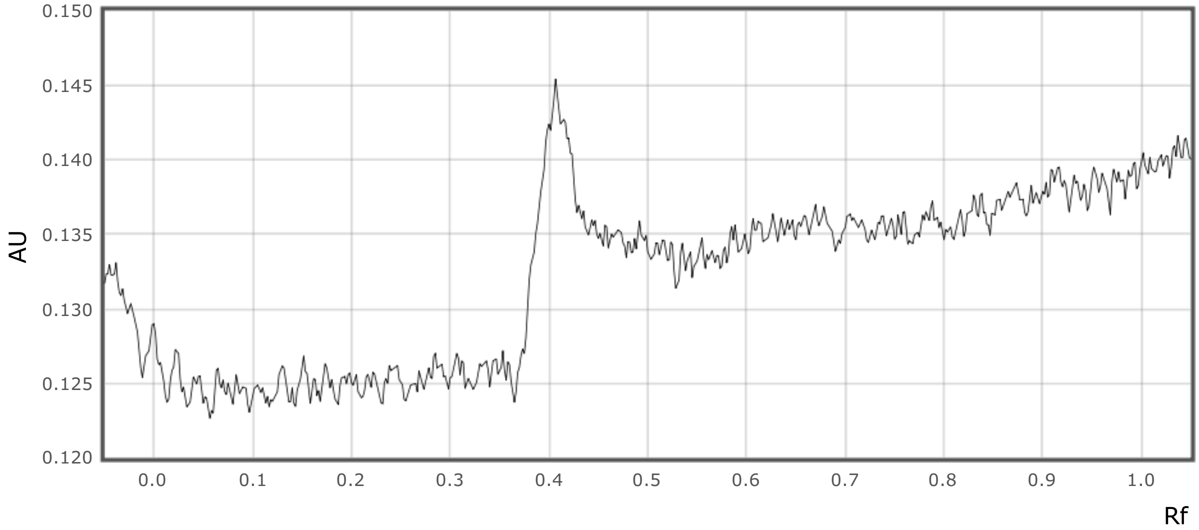

Track 4:

Type	Single λ

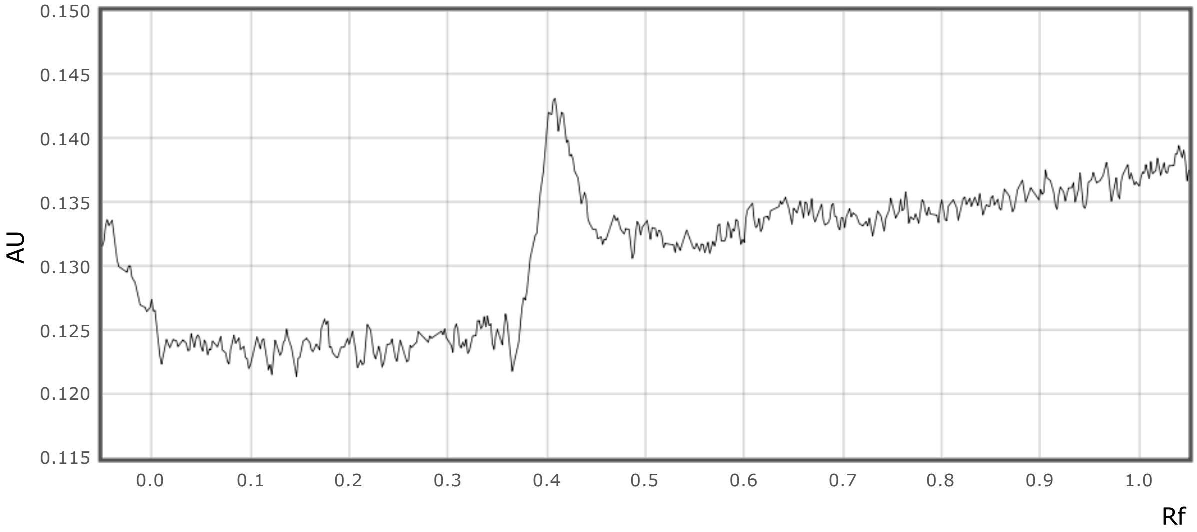

Track 5:	
Type	Single λ

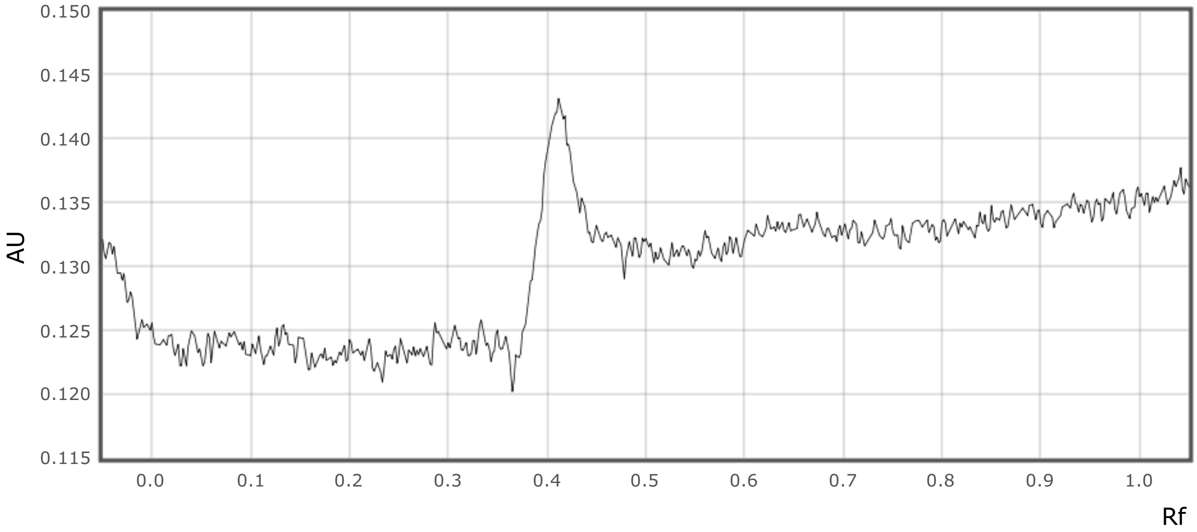

Track 6:	
Type	Single λ

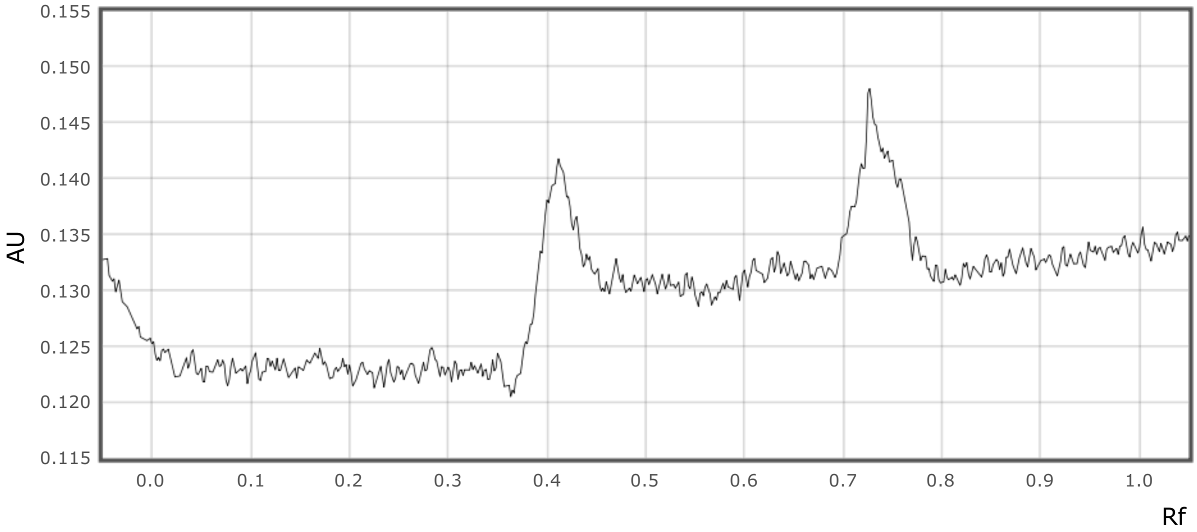

Track 7:	
Type	Single λ

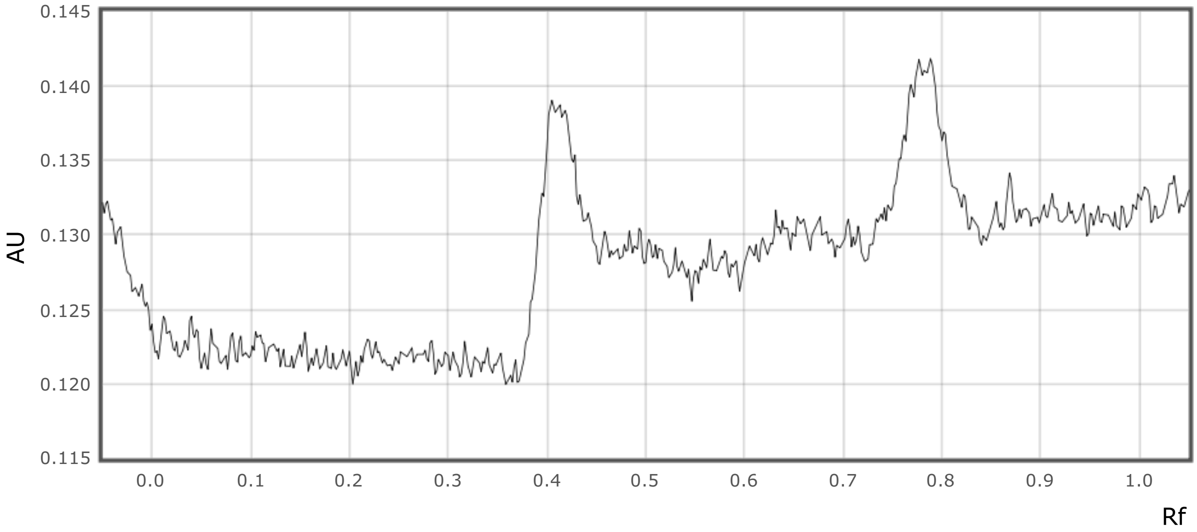

Track 8:	
Type	Single λ

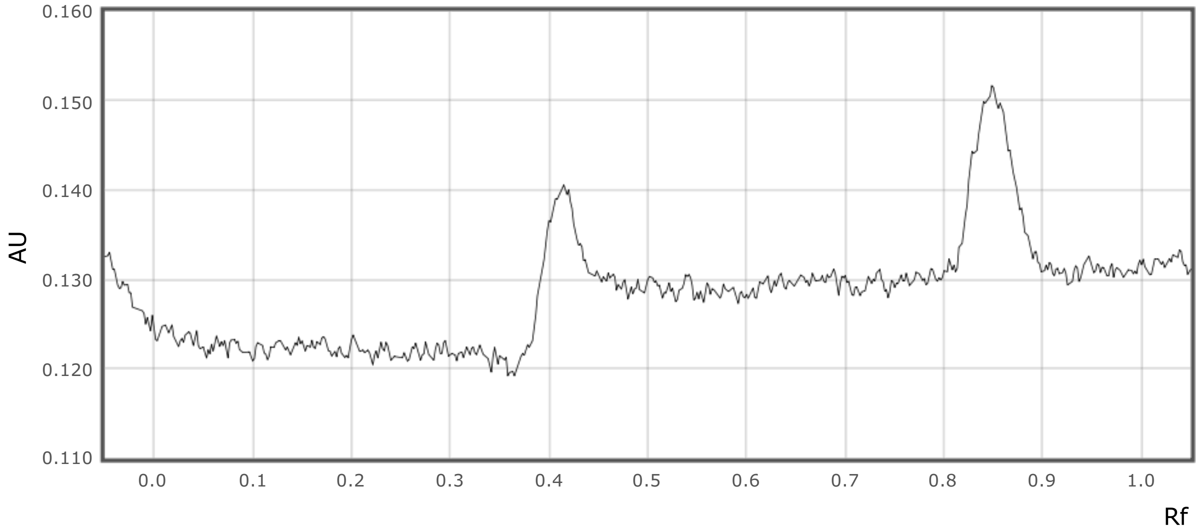

Track 9:	
Type	Single λ

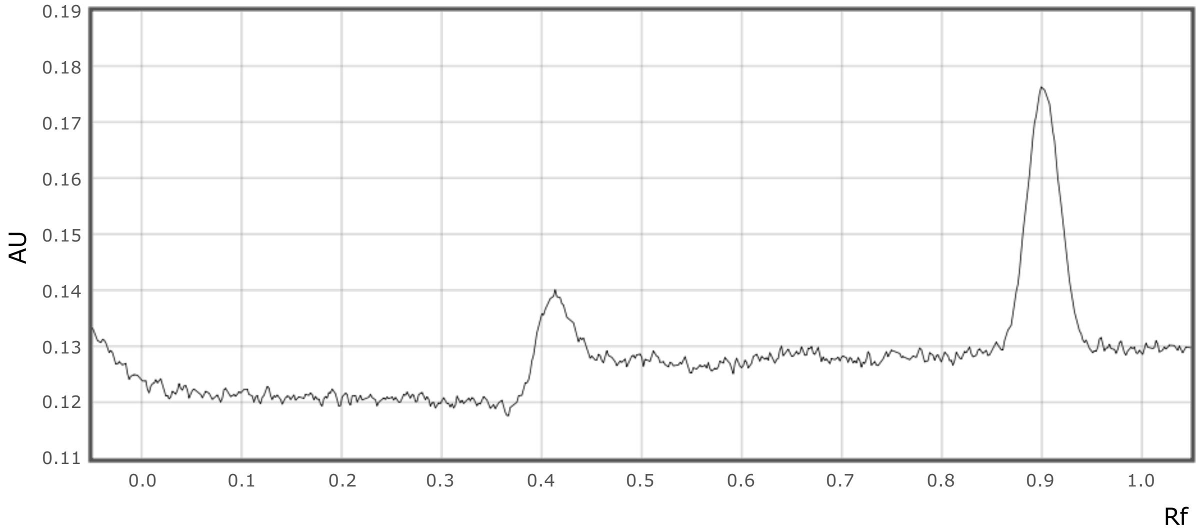

Track 10:	
Type	Single λ

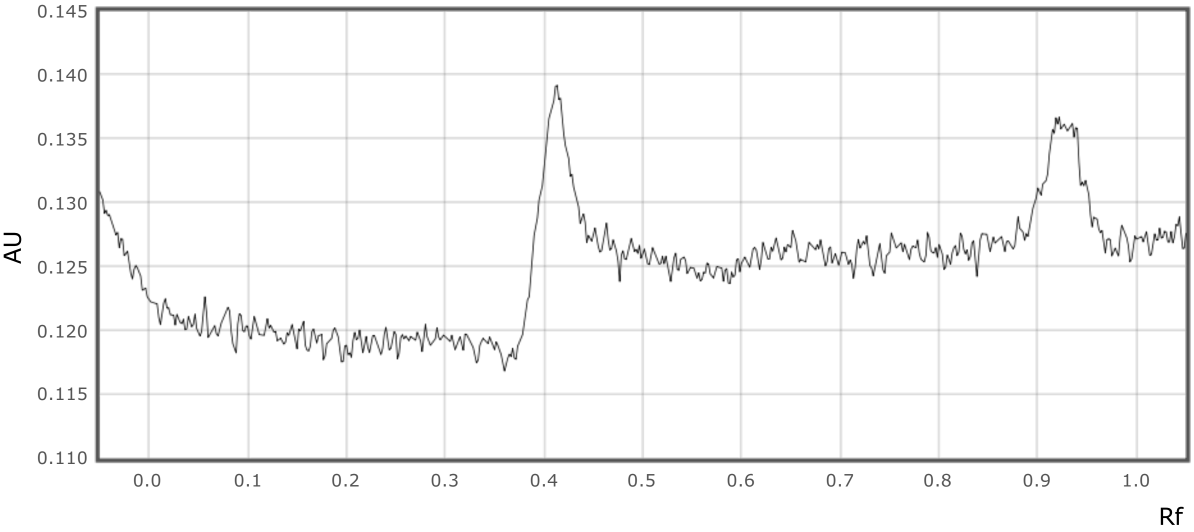

Track 11:	
Type	Single λ

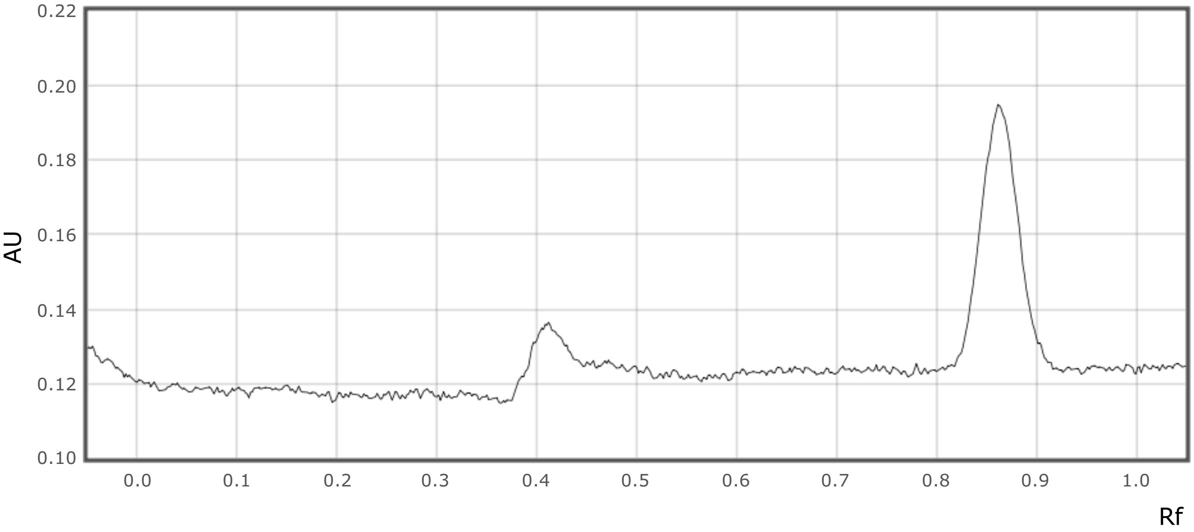

Track 12:	
Type	Single λ

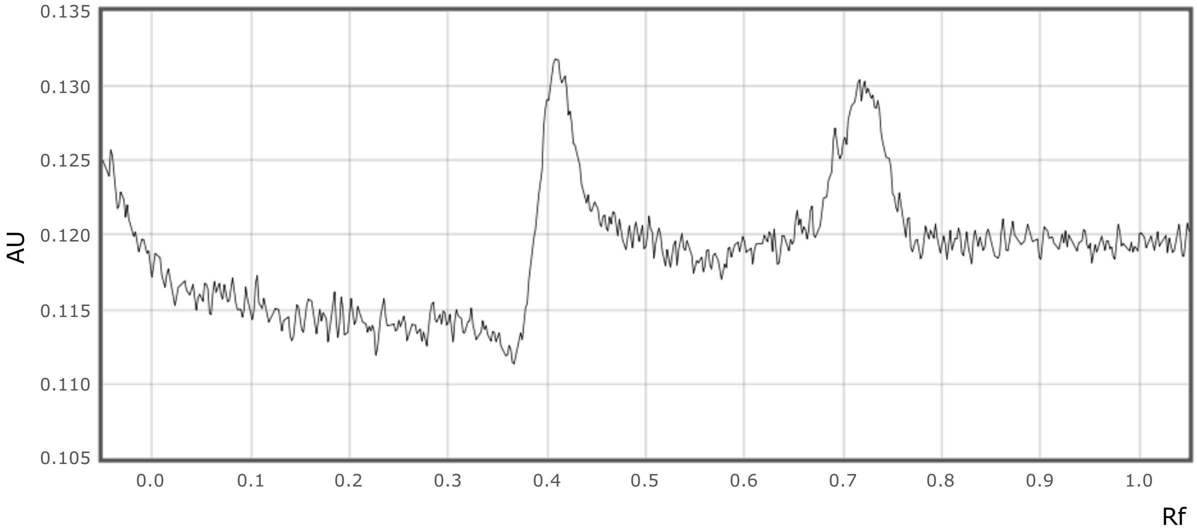

Track 13:	
Type	Single λ

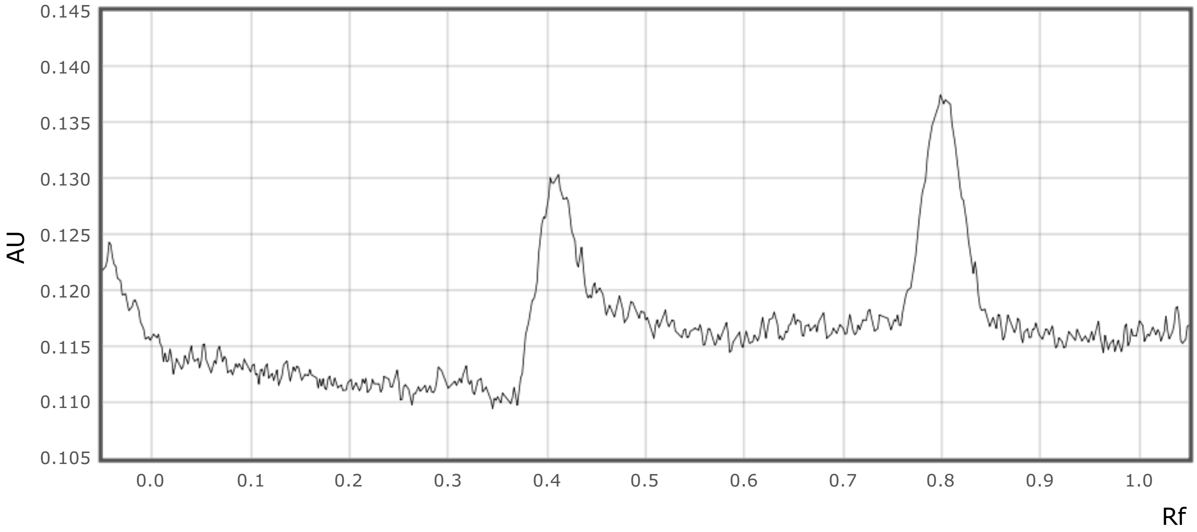

Track 14:	
Type	Single λ

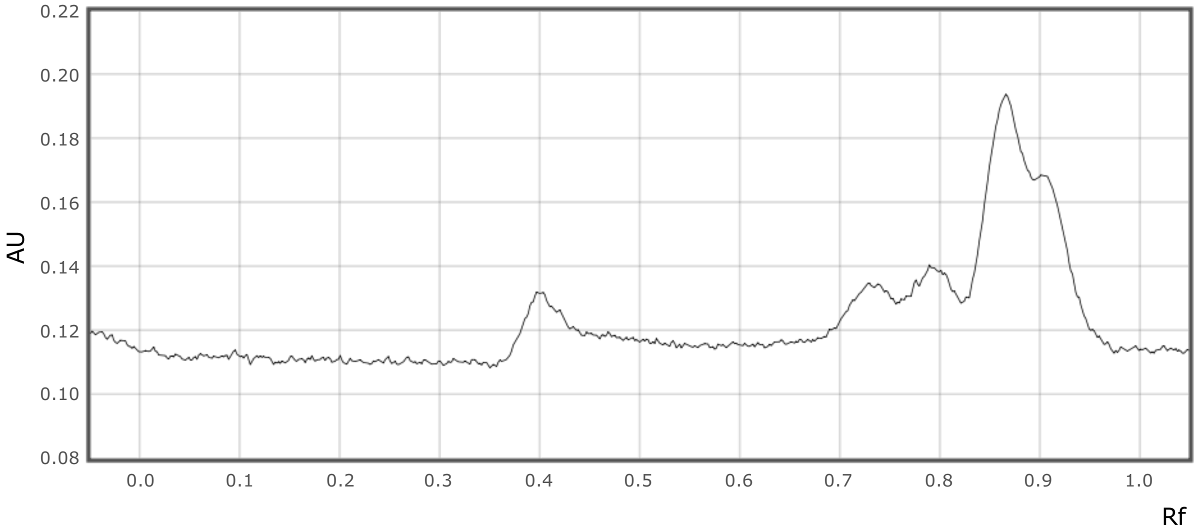

Track 15:	
Type	Single λ

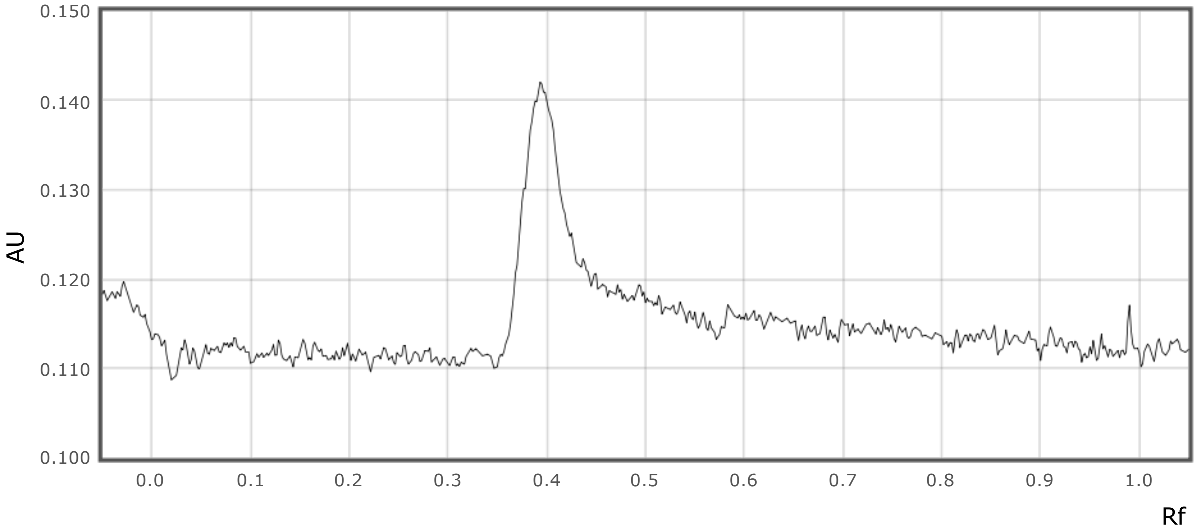

Derivatization 1 - dip:

Executed	07-Jun-2019 16:36:36 visionCATSuser

Take image derivatized plate 1a - Visualizer (S/N: 230515):

Executed	07-Jun-2019 16:36:38 visionCATSuser

RT White

Derivatized, RemTransVis

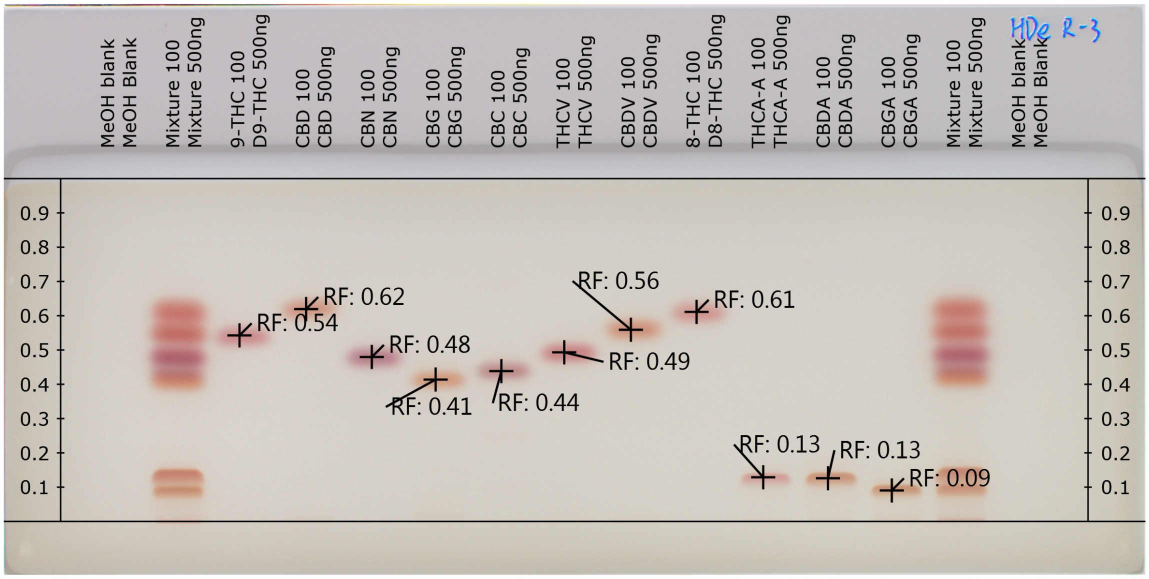

Exposure	0.060 s
Contrast	1
Normalized exposure	Disabled
Clarify	Disabled
White balance	1.05, 1.03, 0.93

R 366

Derivatized, Remission366

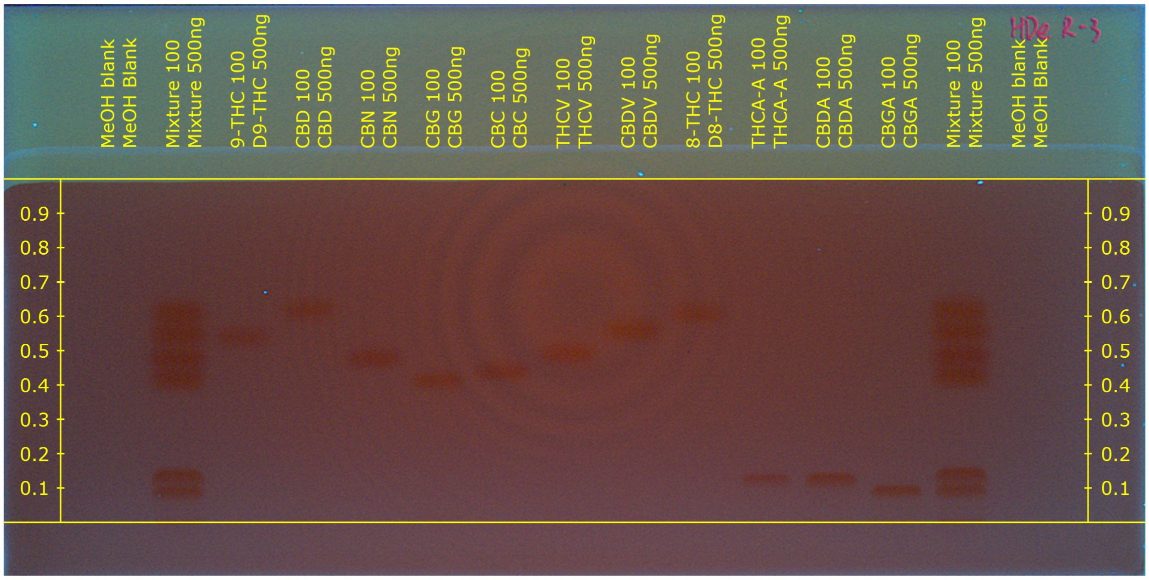

Exposure	9.999 s
Contrast	1
Normalized exposure	Disabled
Clarify	Disabled
White balance	1.00, 1.00, 1.00

Evaluation 1 :

Validated	false
Step	Take image derivatized plate 1a
Concentration unit type	Mass / volume
Notes	

Definition:

References:

9-THC 100		
Substance Name	Concentration	Purity
9-THC	100.000 µg/ml	100.00 %

CBD 100		
Substance Name	Concentration	Purity
CBD	100.000 µg/ml	100.00 %

CBN 100		
Substance Name	Concentration	Purity
CBN	100.000 µg/ml	100.00 %

CBG 100		
Substance Name	Concentration	Purity
CBG	100.000 µg/ml	100.00 %

CBC 100		
Substance Name	Concentration	Purity
CBC	100.000 µg/ml	100.00 %

THCV 100		
Substance Name	Concentration	Purity
THCV	100.000 µg/ml	100.00 %

CBDV 100		
Substance Name	Concentration	Purity
CBDV	100.000 µg/ml	100.00 %

8-THC 100		
Substance Name	Concentration	Purity
8-THC	100.000 µg/ml	100.00 %

THCA-A 100		
Substance Name	Concentration	Purity
THCA-A	100.000 µg/ml	100.00 %

CBDA 100		
Substance Name	Concentration	Purity
CBDA	100.000 µg/ml	100.00 %

CBGA 100		
Substance Name	Concentration	Purity
CBGA	100.000 µg/ml	100.00 %

Samples:

Vial ID	Amount	Volume solution	Reference amount	Related to
MeOH blank		0.00 ml		
Mixture 100		0.00 ml		

Integration parameters:

Bounds	[0.000,1.000]
Smoothing	Savitzky–Golay of order 3 and window 7
Baseline correction	Lowest slope with noise 0.05
Profile subtraction	Profile subtraction from track 1
Peaks detection	Gauss (legacy) with sensitivity 0.1, separation 1 and threshold 0.1

Scan:

Wavelength	RT White

Track 1:

Type	Sample
Vial ID	MeOH blank
Description	MeOH Blank
Volume	2.0 µl

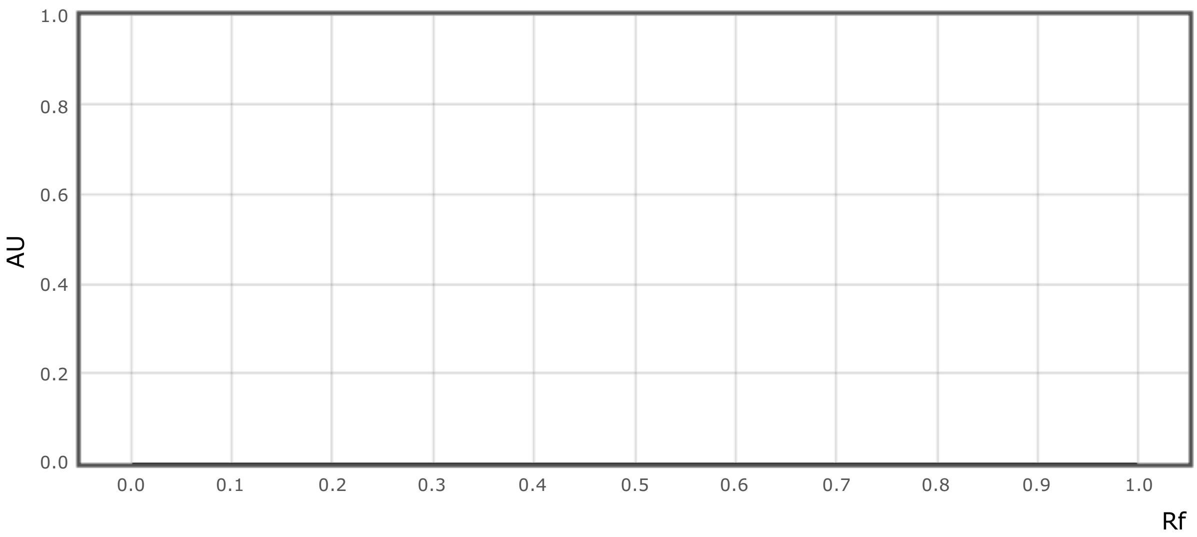

Peak #	Start		Max			End		Area		Manual peak	Substance Name
	Rf	H	Rf	H	%	Rf	H	A	%		

Track 2:

Type	Sample
Vial ID	Mixture 100
Description	Mixture 500ng
Volume	5.0 µl

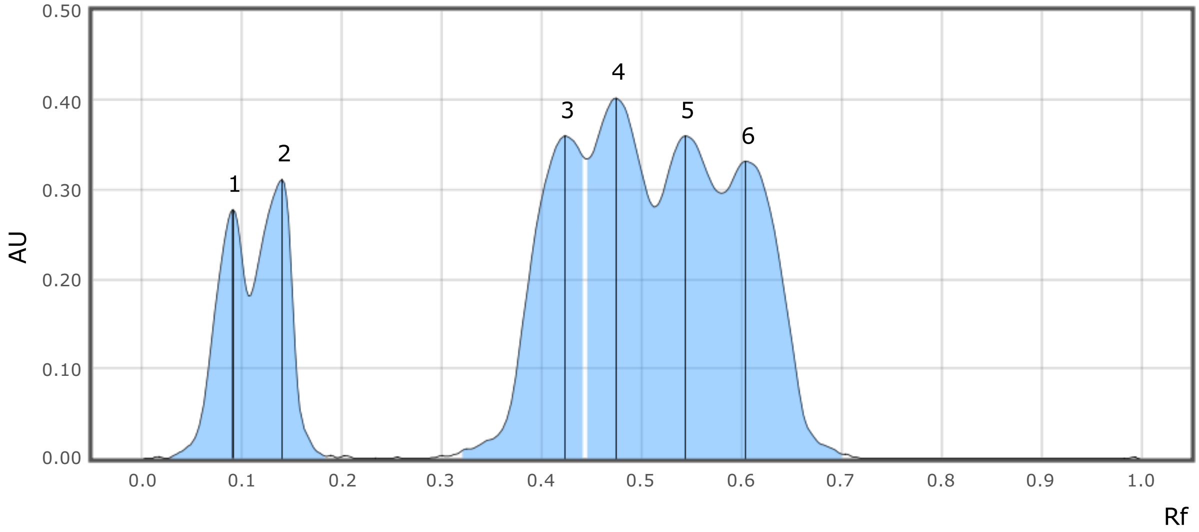

Peak #	Start		Max			End		Area		Manual peak	Substance Name
	Rf	H	Rf	H	%	Rf	H	A	%		
1	0.026	0.0000	0.091	0.2778	13.60	0.106	0.1812	0.00963	8.69	No	
2	0.106	0.1812	0.140	0.3112	15.23	0.184	0.0021	0.01254	11.30	No	
3	0.320	0.0088	0.423	0.3604	17.64	0.443	0.3347	0.02133	19.23	No	
4	0.445	0.3343	0.474	0.4021	19.68	0.512	0.2805	0.02387	21.52	No	
5	0.512	0.2805	0.544	0.3598	17.61	0.579	0.2960	0.02181	19.66	No	
6	0.579	0.2960	0.604	0.3318	16.24	0.702	0.0045	0.02174	19.60	No	

Track 3:

Type	Reference
Vial ID	9-THC 100
Description	D9-THC 500ng
Volume	5.0 µl

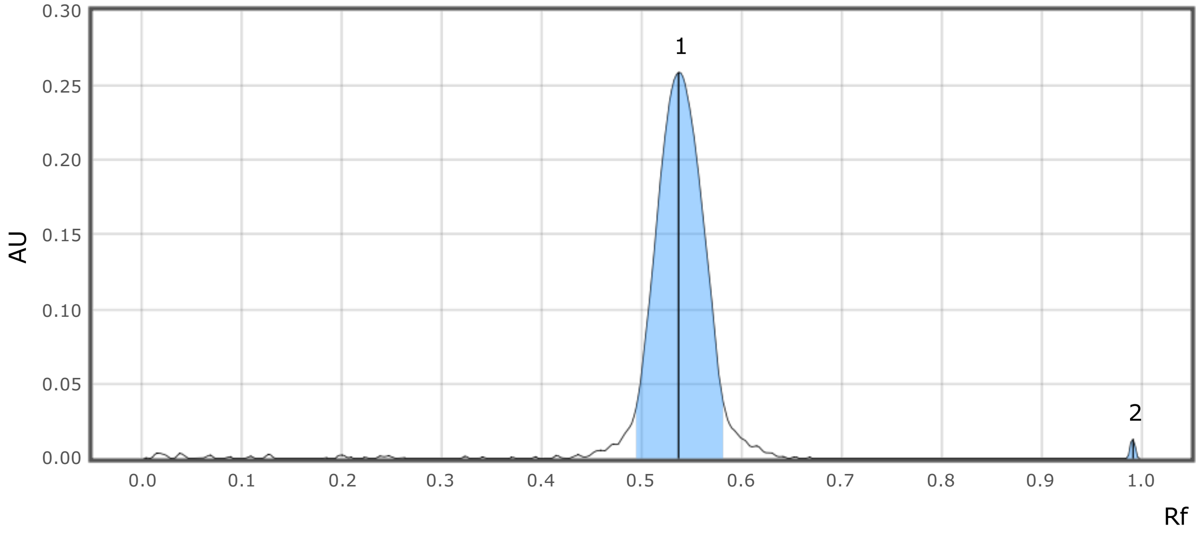

Peak #	Start		Max			End		Area		Manual peak	Substance Name
	Rf	H	Rf	H	%	Rf	H	A	%		
1	0.493	0.0337	0.537	0.2585	95.32	0.584	0.0317	0.01418	99.46	Yes	9-THC
2	0.985	0.0000	0.992	0.0127	4.68	0.999	0.0000	0.00008	0.54	No	

Track 4:

Type	Reference
Vial ID	CBD 100
Description	CBD 500ng
Volume	5.0 µl

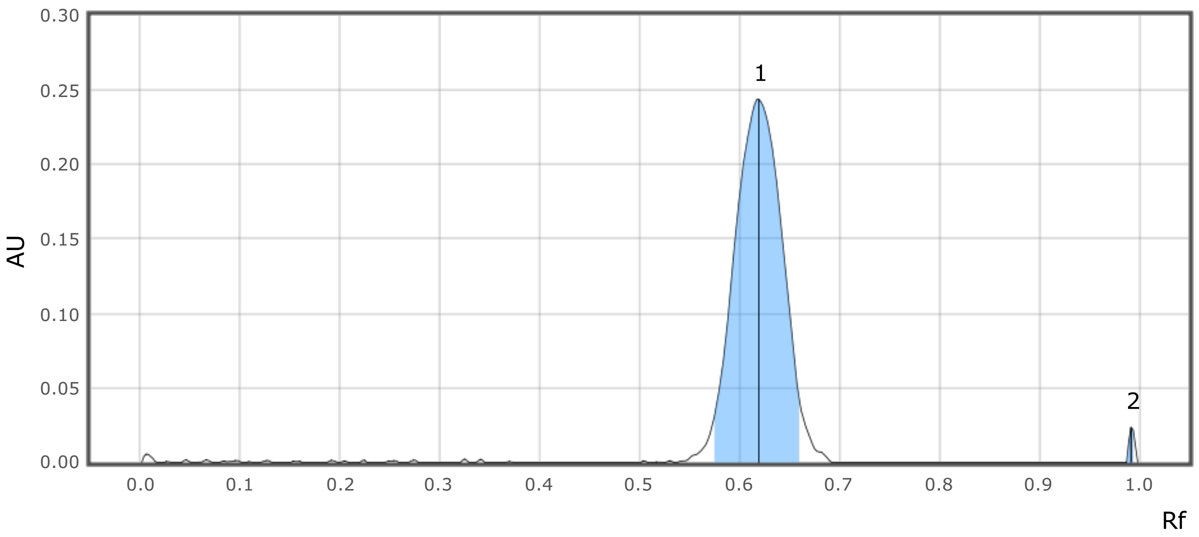

Peak #	Start		Max			End		Area		Manual peak	Substance Name
	Rf	H	Rf	H	%	Rf	H	A	%		
1	0.573	0.0249	0.619	0.2434	91.15	0.661	0.0344	0.01335	98.84	Yes	CBD
2	0.988	0.0000	0.992	0.0236	8.85	0.999	0.0000	0.00016	1.16	No	

Track 5:

Type	Reference
Vial ID	CBN 100
Description	CBN 500ng
Volume	5.0 µl

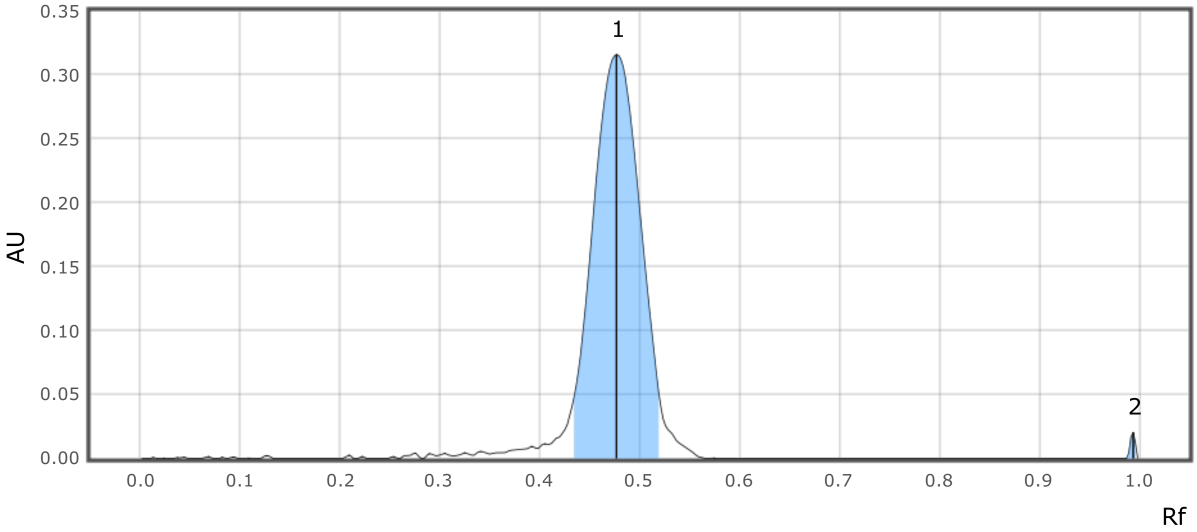

Peak #	Start		Max			End		Area		Manual peak	Substance Name
	Rf	H	Rf	H	%	Rf	H	A	%		
1	0.433	0.0477	0.477	0.3158	93.96	0.521	0.0400	0.01716	99.31	Yes	CBN
2	0.988	0.0000	0.994	0.0203	6.04	0.999	0.0000	0.00012	0.69	No	

Track 6:

Type	Reference
Vial ID	CBG 100
Description	CBG 500ng
Volume	5.0 µl

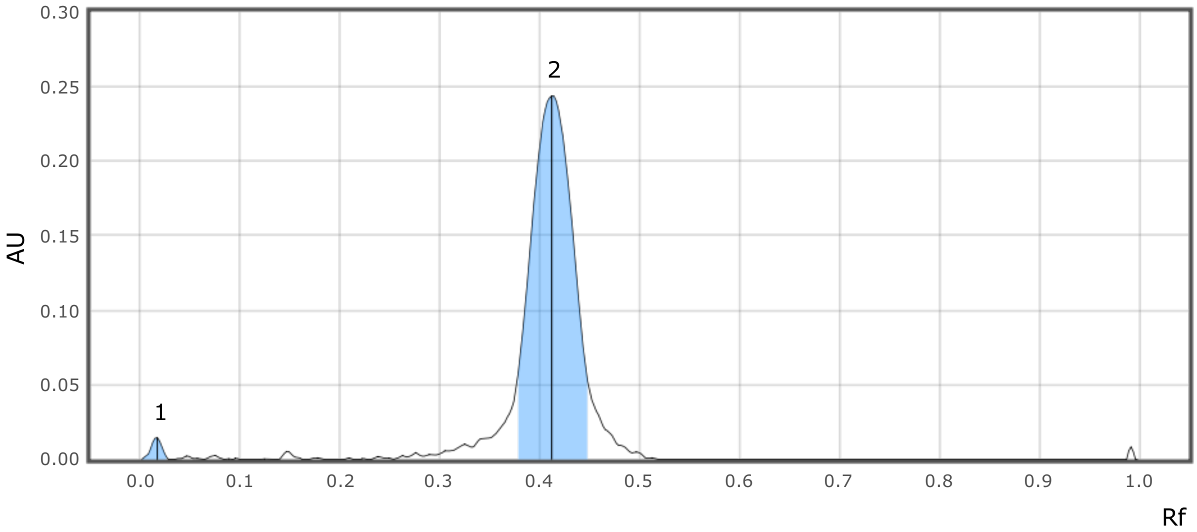

Peak #	Start		Max			End		Area		Manual peak	Substance Name
	Rf	H	Rf	H	%	Rf	H	A	%		
1	0.001	0.0000	0.017	0.0145	5.61	0.028	0.0000	0.00018	1.51	No	
2	0.375	0.0388	0.412	0.2434	94.39	0.451	0.0457	0.01186	98.49	Yes	CBG

Track 7:

Type	Reference
Vial ID	CBC 100
Description	CBC 500ng
Volume	5.0 µl

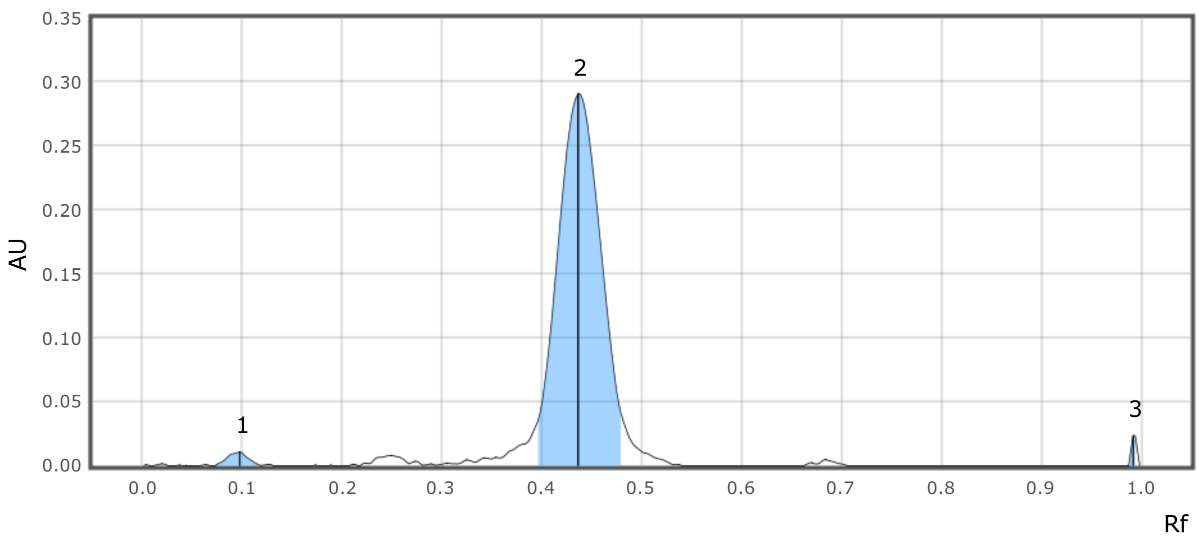

Peak #	Start		Max			End		Area		Manual peak	Substance Name
	Rf	H	Rf	H	%	Rf	H	A	%		
1	0.073	0.0000	0.097	0.0109	3.36	0.120	0.0003	0.00026	1.76	No	
2	0.396	0.0353	0.436	0.2909	89.40	0.479	0.0417	0.01432	97.15	Yes	CBC
3	0.988	0.0000	0.992	0.0236	7.24	0.999	0.0000	0.00016	1.09	No	

Track 8:

Type	Reference
Vial ID	THCV 100
Description	THCV 500ng
Volume	5.0 µl

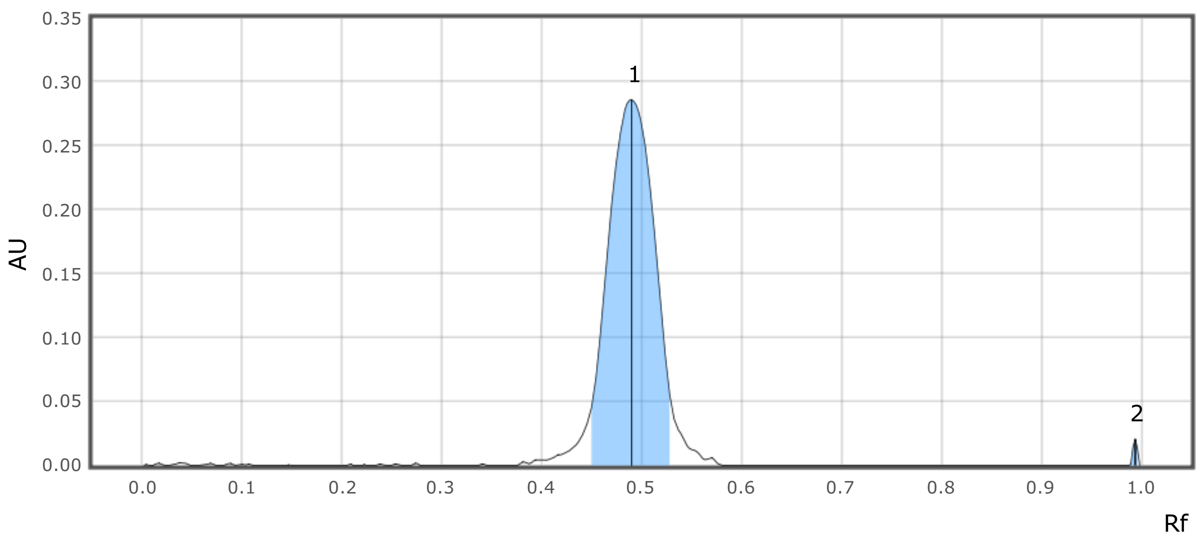

Peak #	Start		Max			End		Area		Manual peak	Substance Name
	Rf	H	Rf	H	%	Rf	H	A	%		
1	0.447	0.0386	0.490	0.2860	93.28	0.532	0.0364	0.01530	99.29	Yes	THCV
2	0.988	0.0000	0.994	0.0206	6.72	0.999	0.0000	0.00011	0.71	No	

Track 9:

Type	Reference
Vial ID	CBDV 100
Description	CBDV 500ng
Volume	5.0 µl

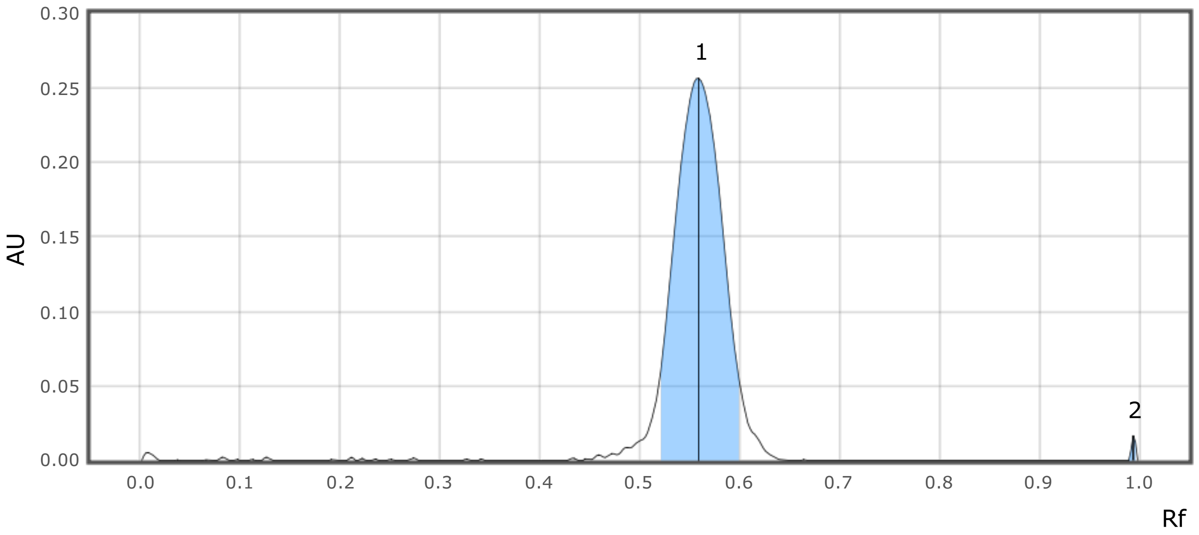

Peak #	Start		Max			End		Area		Manual peak	Substance Name
	Rf	H	Rf	H	%	Rf	H	A	%		
1	0.519	0.0499	0.559	0.2561	93.91	0.602	0.0466	0.01391	99.43	Yes	CBDV
2	0.990	0.0000	0.994	0.0166	6.09	0.999	0.0000	0.00008	0.57	No	

Track 10:

Type	Reference
Vial ID	8-THC 100
Description	D8-THC 500ng
Volume	5.0 µl

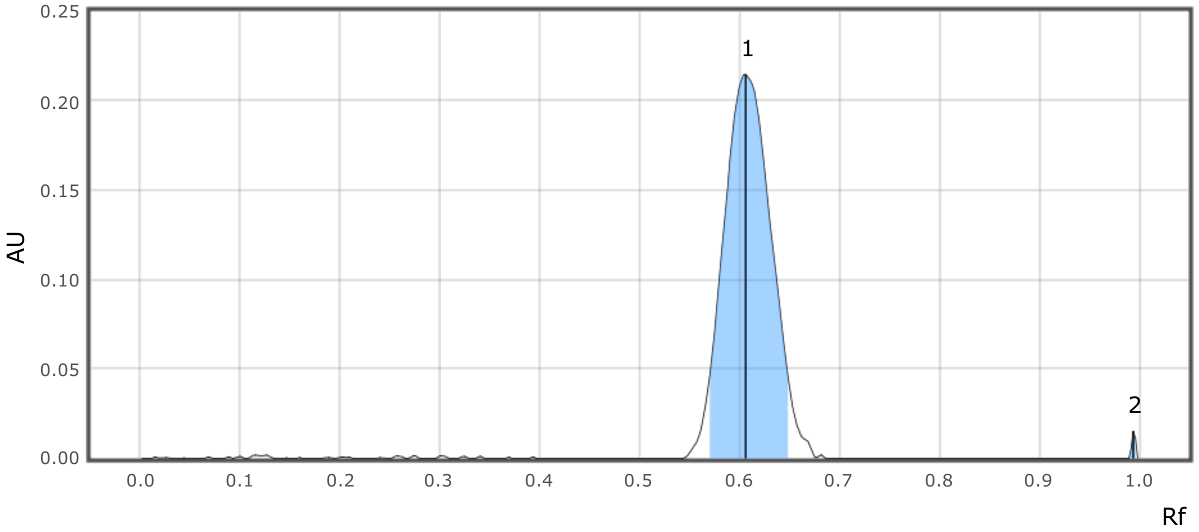

Peak #	Start		Max			End		Area		Manual peak	Substance Name
	Rf	H	Rf	H	%	Rf	H	A	%		
1	0.567	0.0294	0.606	0.2147	93.39	0.652	0.0294	0.01168	99.40	Yes	8-THC
2	0.990	0.0000	0.994	0.0152	6.61	0.999	0.0000	0.00007	0.60	No	

Track 11:

Type	Reference
Vial ID	THCA-A 100
Description	THCA-A 500ng
Volume	5.0 µl

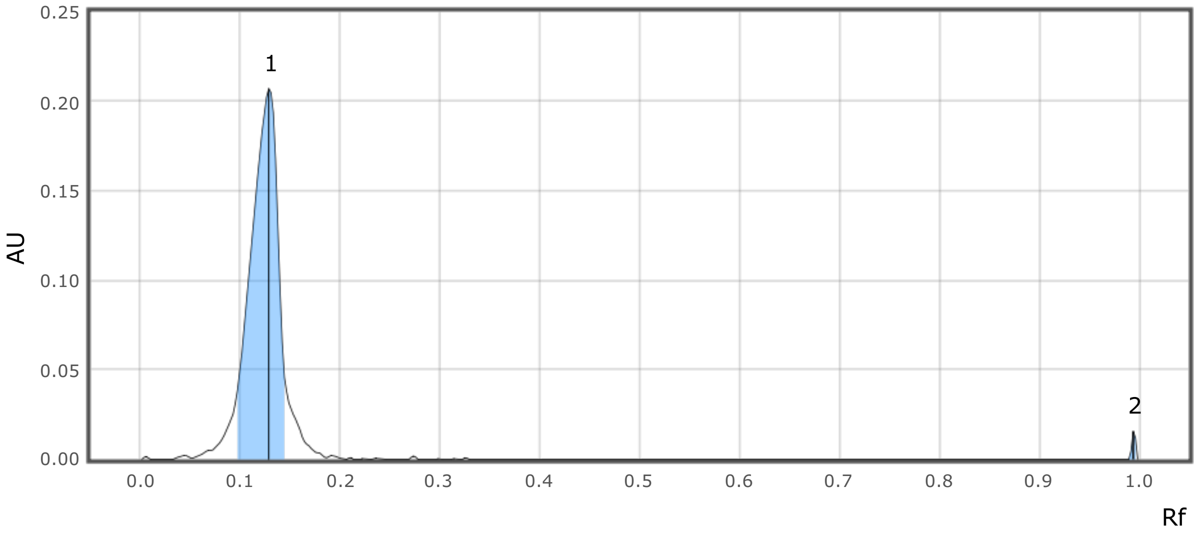

Peak #	Start		Max			End		Area		Manual peak	Substance Name
	Rf	H	Rf	H	%	Rf	H	A	%		
1	0.095	0.0314	0.128	0.2069	92.98	0.145	0.0458	0.00627	98.87	Yes	THCA-A
2	0.990	0.0000	0.994	0.0156	7.02	0.999	0.0000	0.00007	1.13	No	

Track 12:

Type	Reference
Vial ID	CBDA 100
Description	CBDA 500ng
Volume	5.0 µl

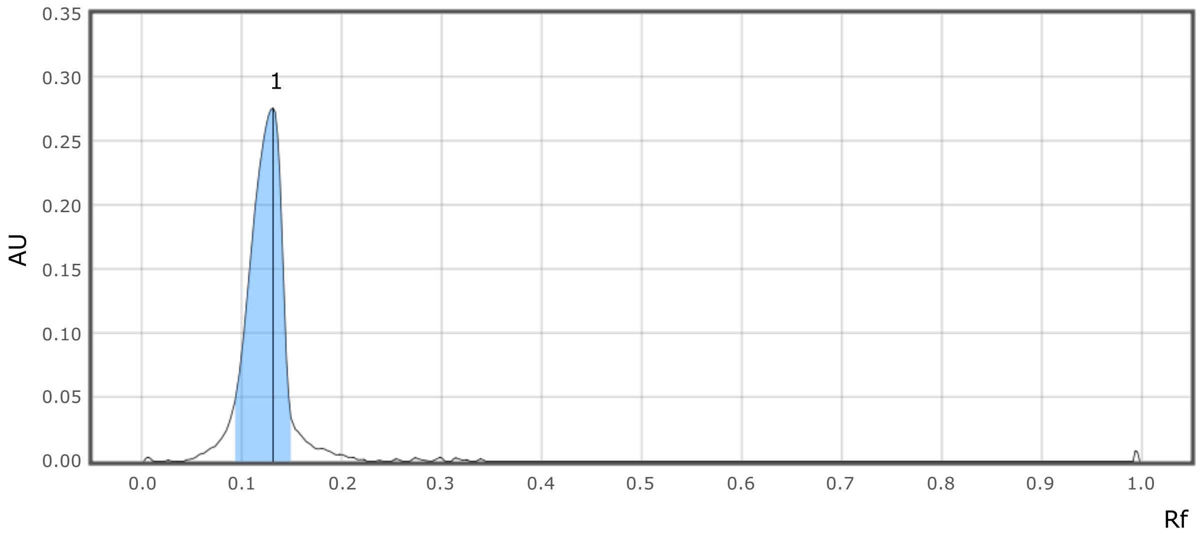

Peak #	Start		Max			End		Area		Manual peak	Substance Name
	Rf	H	Rf	H	%	Rf	H	A	%		
1	0.090	0.0405	0.131	0.2762	100.00	0.149	0.0341	0.00984	100.00	Yes	CBDA

Track 13:

Type	Reference
Vial ID	CBGA 100
Description	CBGA 500ng
Volume	5.0 µl

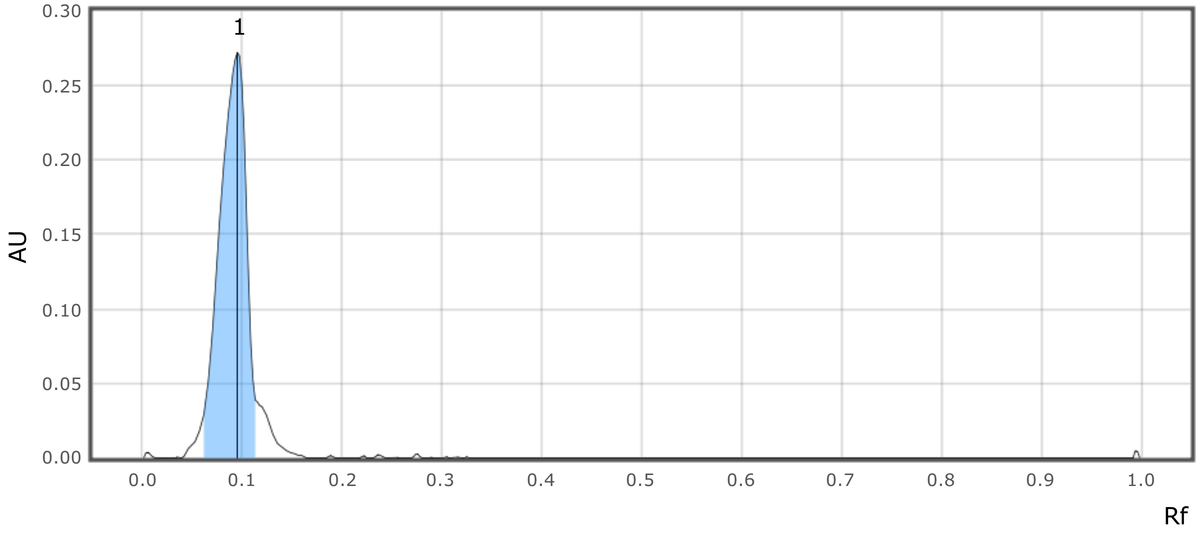

Peak #	Start		Max			End		Area		Manual peak	Substance Name
	Rf	H	Rf	H	%	Rf	H	A	%		
1	0.059	0.0234	0.095	0.2718	100.0<br>0	0.113	0.0389	0.00833	100.0<br>0	Yes	CBGA

Track 14:

Type	Sample
Vial ID	Mixture 100
Description	Mixture 500ng
Volume	5.0 µl

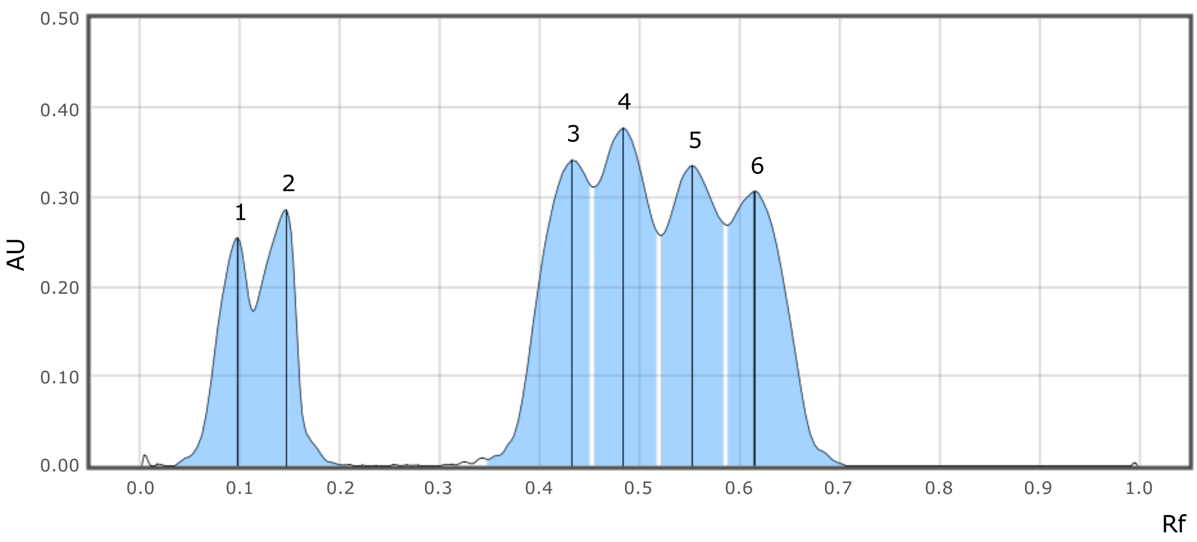

Peak #	Start		Max			End		Area		Manual peak	Substance Name
	Rf	H	Rf	H	%	Rf	H	A	%		
1	0.033	0.0000	0.097	0.2548	13.41	0.113	0.1723	0.00932	9.29	No	
2	0.113	0.1723	0.146	0.2856	15.03	0.202	0.0010	0.01147	11.44	No	
3	0.347	0.0071	0.432	0.3412	17.95	0.452	0.3115	0.01934	19.28	No	
4	0.454	0.3113	0.483	0.3771	19.85	0.519	0.2585	0.02155	21.48	No	
5	0.521	0.2569	0.552	0.3350	17.63	0.586	0.2691	0.01946	19.40	No	
6	0.588	0.2684	0.615	0.3065	16.13	0.709	0.0000	0.01916	19.11	No	

Track 15:

Type	Sample
Vial ID	MeOH blank
Description	MeOH Blank
Volume	2.0 µl

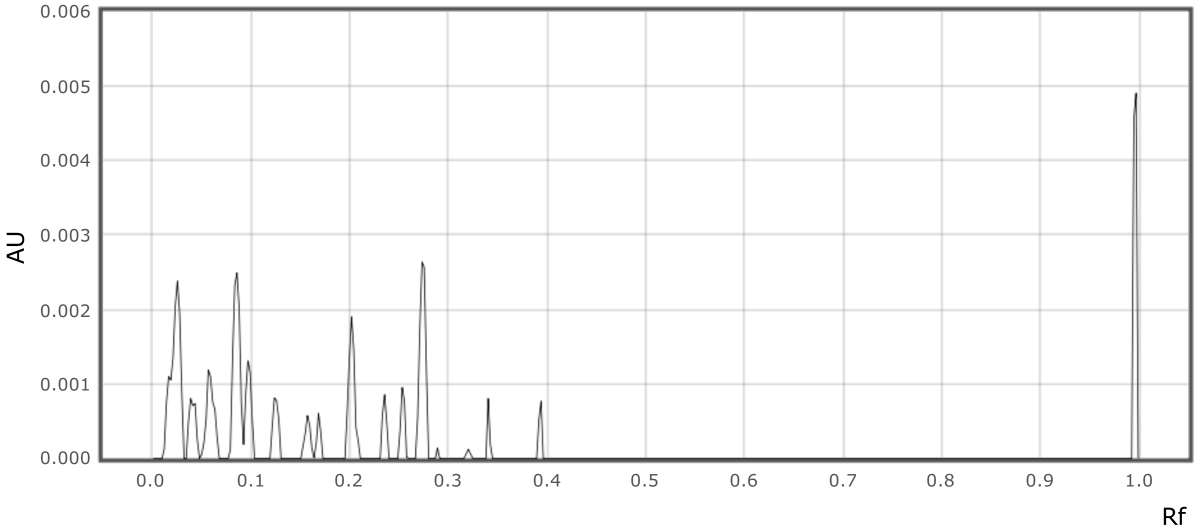

Peak #	Start		Max			End		Area		Manual peak	Substance Name
	Rf	H	Rf	H	%	Rf	H	A	%		

Calibration results:

Height calibration for substance 8-THC @ RT White:

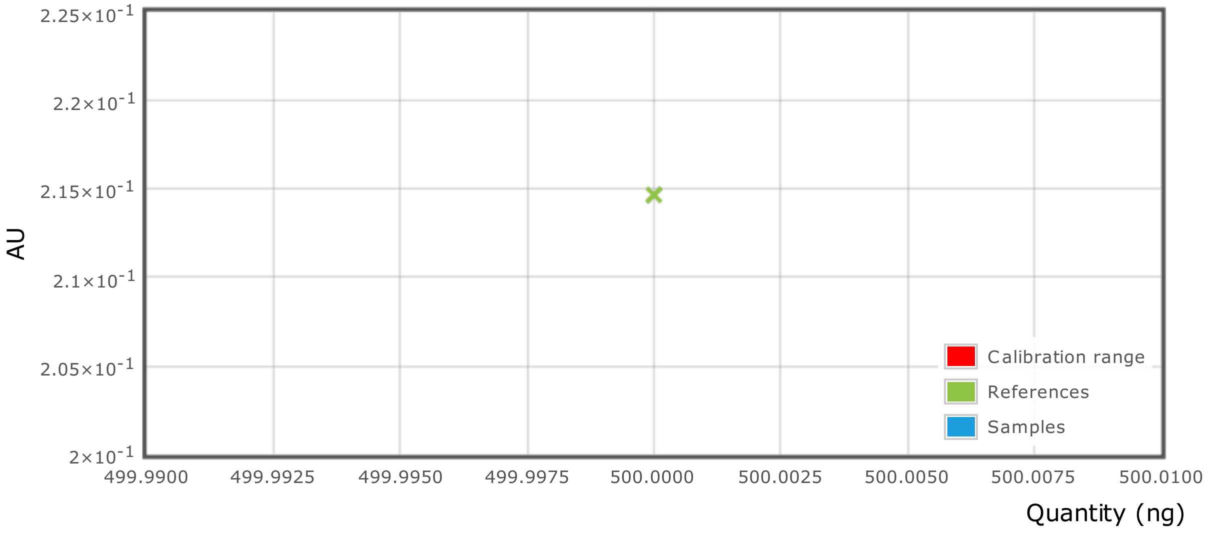

Regression mode	Linear-2
Range deviation	5.00 %
Related substances	Default
Number of references	1
Calibration function	$y=0x$
Coefficient of variation	CV 0.00 %
Correlation coefficient	n/a
⚠	Unable to compute the results for this substance because there wasn't eno ugh groups of references replicas (at least 1 for Linear-1, 2 for Linear2 and Mime-1 and 3 for Polynomial and MiMe-2)

Height calibration for substance 9-THC @ RT White:

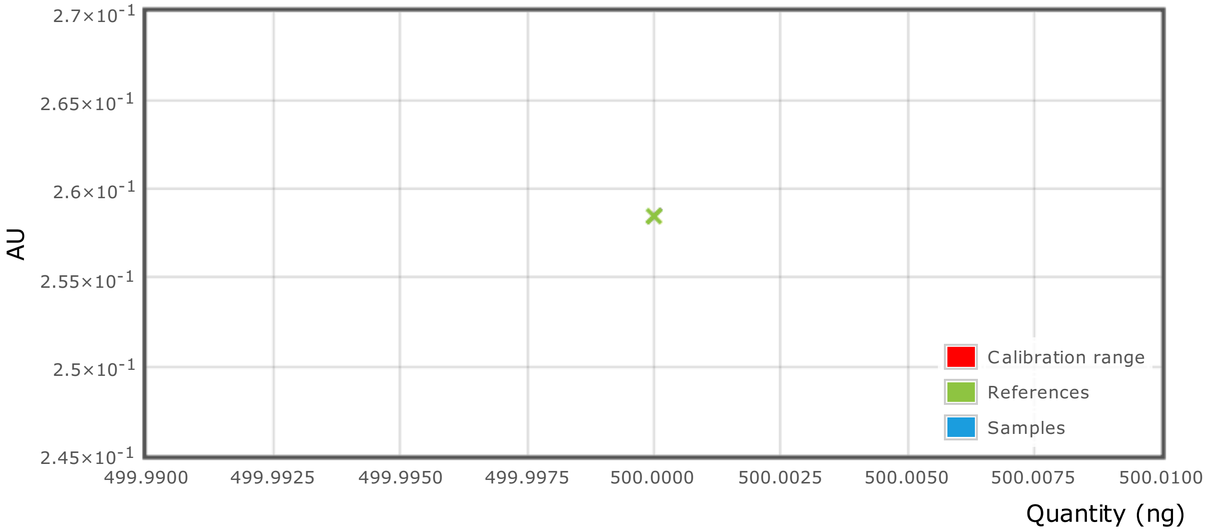

Regression mode	Linear-2
Range deviation	5.00 %
Related substances	Default
Number of references	1
Calibration function	$y=0x$
Coefficient of variation	CV 0.00 %
Correlation coefficient	n/a
⚠	Unable to compute the results for this substance because there wasn't enough groups of references replicas (at least 1 for Linear-1, 2 for Linear2 and Mime-1 and 3 for Polynomial and MiMe-2)

Height calibration for substance CBC @ RT White:

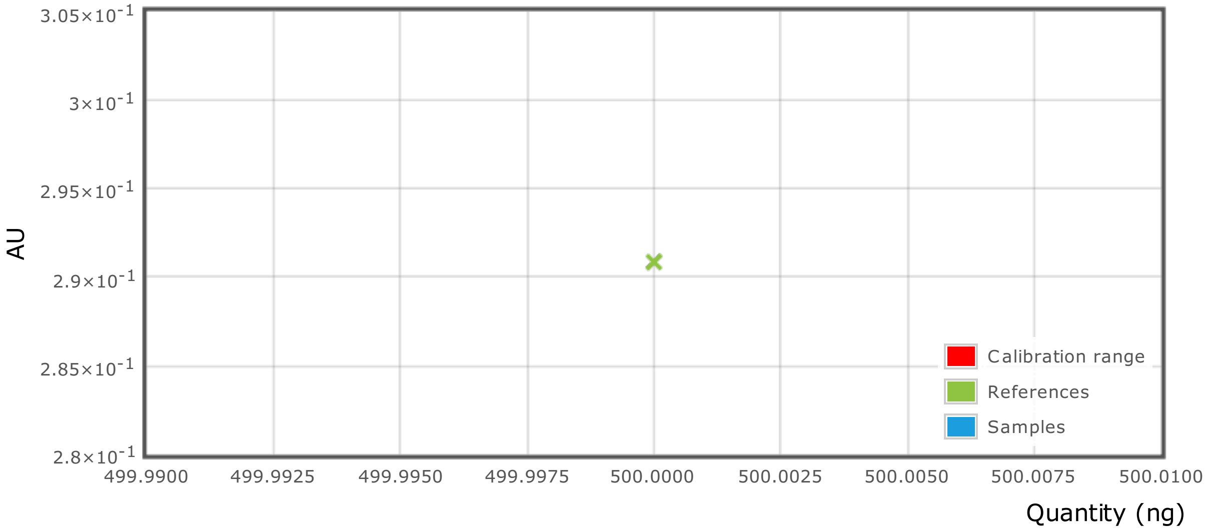

Regression mode	Linear-2
Range deviation	5.00 %
Related substances	Default
Number of references	1
Calibration function	$y=0x$
Coefficient of variation	CV 0.00 %
Correlation coefficient	n/a
⚠	Unable to compute the results for this substance because there wasn't enough groups of references replicas (at least 1 for Linear-1, 2 for Linear2 and Mime-1 and 3 for Polynomial and MiMe-2)

Height calibration for substance CBD @ RT White:

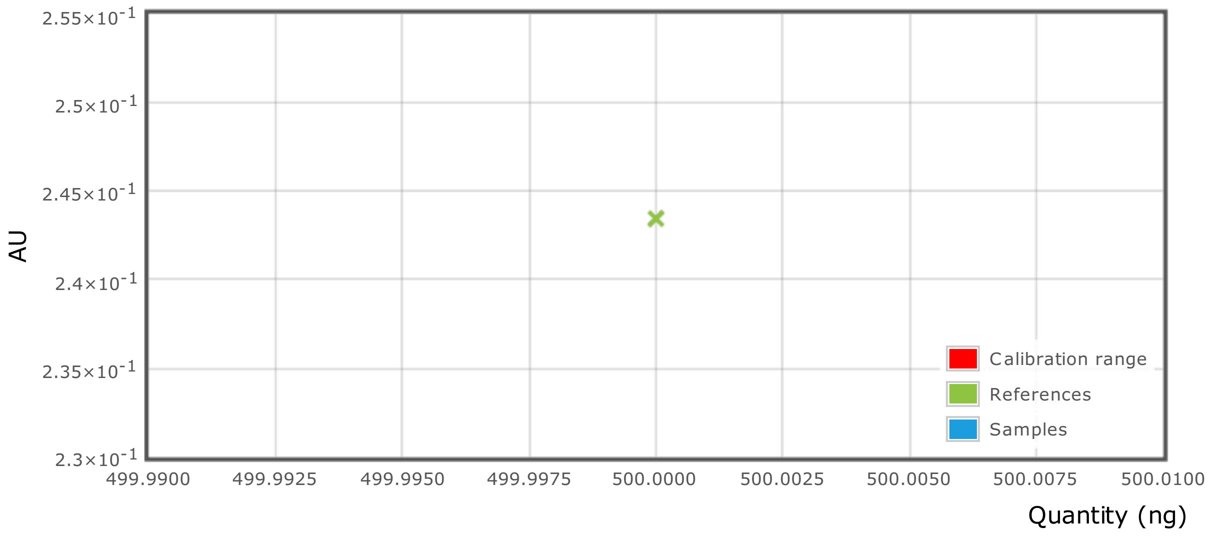

Regression mode	Linear-2
Range deviation	5.00 %
Related substances	Default
Number of references	1
Calibration function	$y=0x$
Coefficient of variation	CV 0.00 %
Correlation coefficient	n/a
⚠	Unable to compute the results for this substance because there wasn't enough groups of references replicas (at least 1 for Linear-1, 2 for Linear2 and Mime-1 and 3 for Polynomial and MiMe-2)

Height calibration for substance CBDA @ RT White:

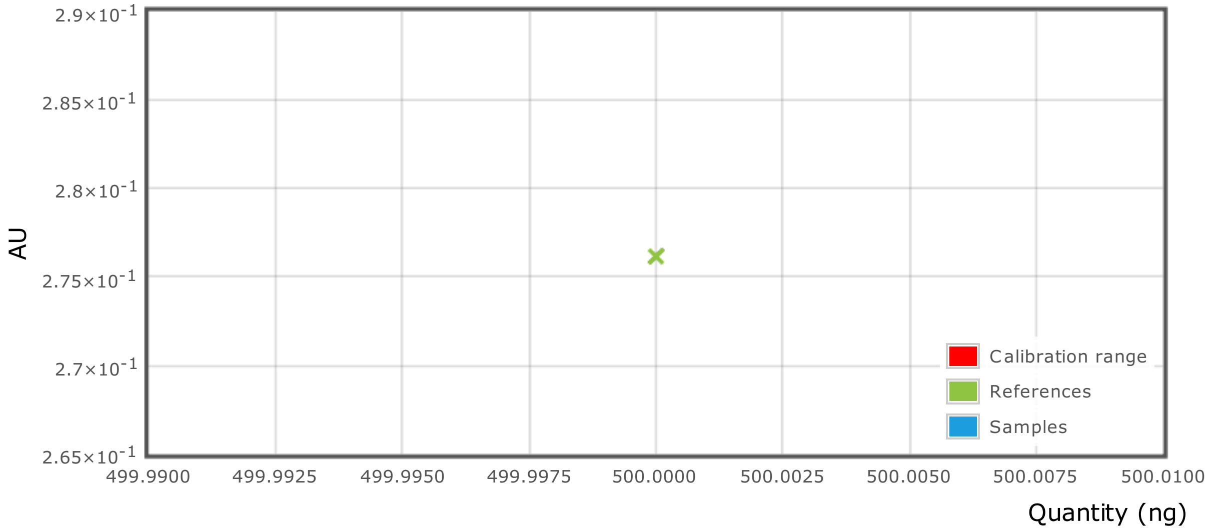

Regression mode	Linear-2
Range deviation	5.00 %
Related substances	Default
Number of references	1
Calibration function	$y=0x$
Coefficient of variation	CV 0.00 %
Correlation coefficient	n/a
⚠	Unable to compute the results for this substance because there wasn't enough groups of references replicas (at least 1 for Linear-1, 2 for Linear2 and Mime-1 and 3 for Polynomial and MiMe-2)

Height calibration for substance CBDV @ RT White:

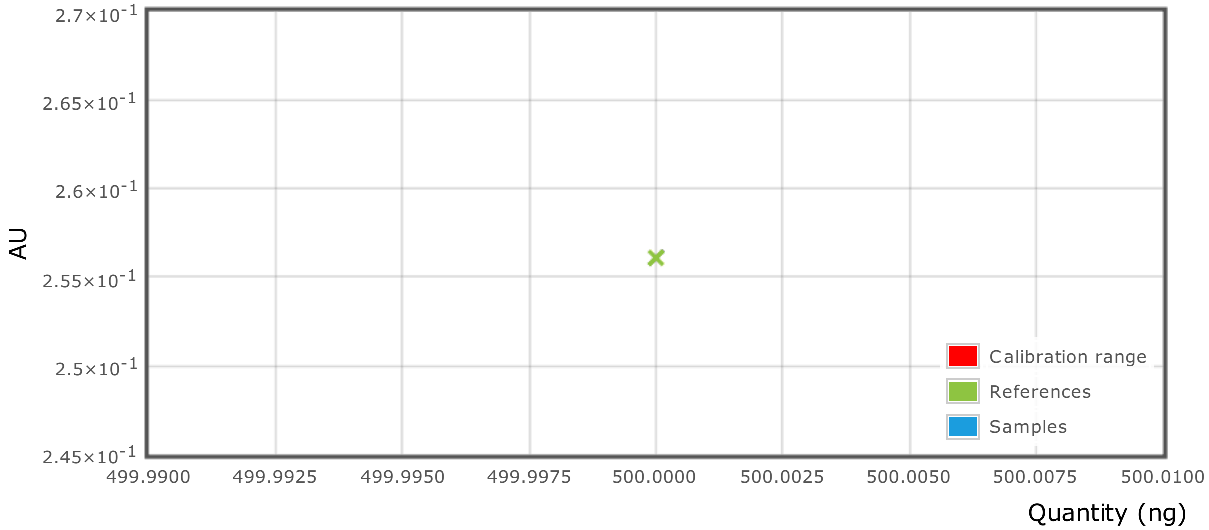

Regression mode	Linear-2
Range deviation	5.00 %
Related substances	Default
Number of references	1
Calibration function	$y=0x$
Coefficient of variation	CV 0.00 %
Correlation coefficient	n/a
⚠	Unable to compute the results for this substance because there wasn't enough groups of references replicas (at least 1 for Linear-1, 2 for Linear2 and Mime-1 and 3 for Polynomial and MiMe-2)

Height calibration for substance CBG @ RT White:

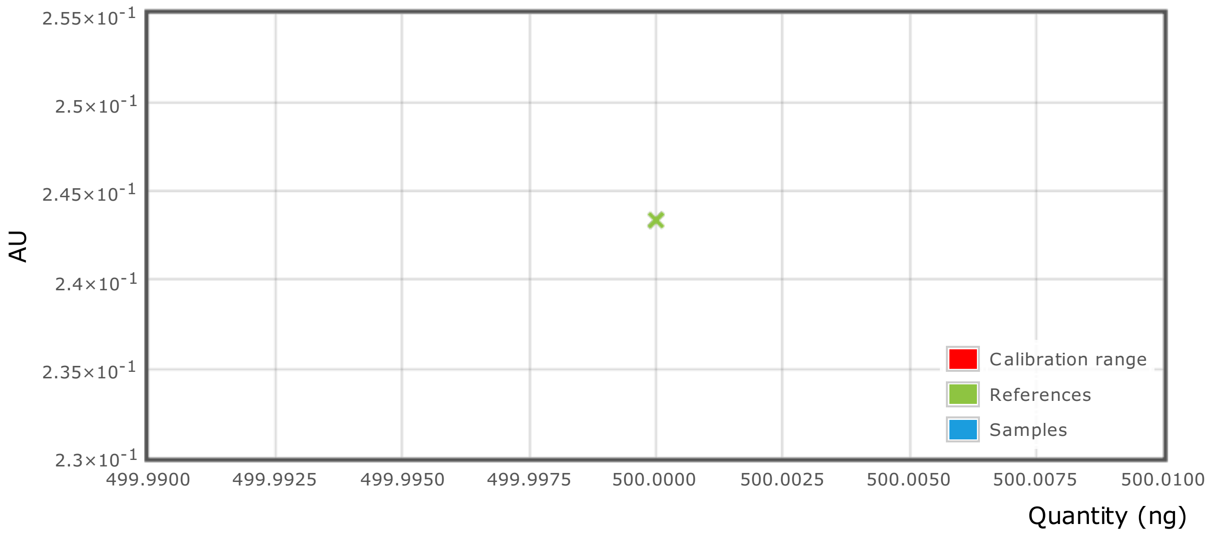

Regression mode	Linear-2
Range deviation	5.00 %
Related substances	Default
Number of references	1
Calibration function	$y=0x$
Coefficient of variation	CV 0.00 %
Correlation coefficient	n/a
⚠	Unable to compute the results for this substance because there wasn't enough groups of references replicas (at least 1 for Linear-1, 2 for Linear2 and Mime-1 and 3 for Polynomial and MiMe-2)

Height calibration for substance CBGA @ RT White:

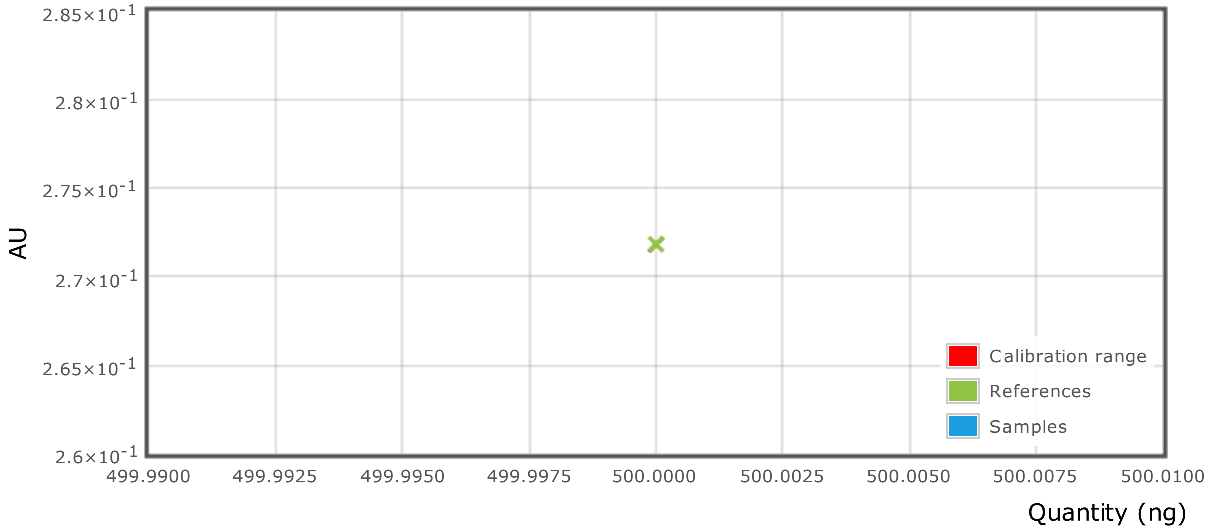

Regression mode	Linear-2
Range deviation	5.00 %
Related substances	Default
Number of references	1
Calibration function	$y=0x$
Coefficient of variation	CV 0.00 %
Correlation coefficient	n/a
⚠	Unable to compute the results for this substance because there wasn't eno ugh groups of references replicas (at least 1 for Linear-1, 2 for Linear2 and Mime-1 and 3 for Polynomial and MiMe-2)

Height calibration for substance CBN @ RT White:

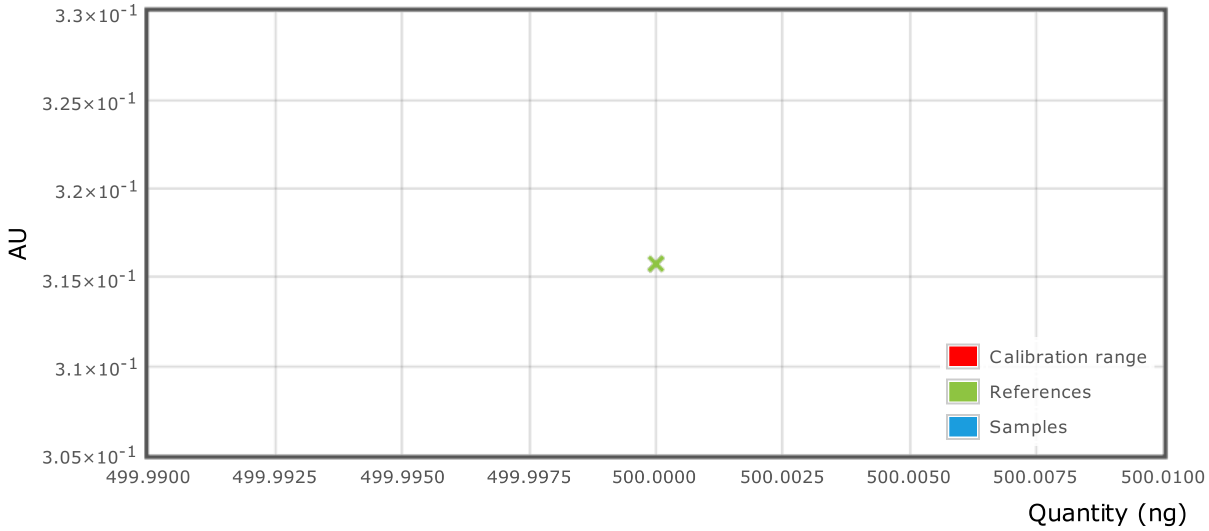

Regression mode	Linear-2
Range deviation	5.00 %
Related substances	Default
Number of references	1
Calibration function	$y=0x$
Coefficient of variation	CV 0.00 %
Correlation coefficient	n/a
⚠	Unable to compute the results for this substance because there wasn't enough groups of references replicas (at least 1 for Linear-1, 2 for Linear2 and Mime-1 and 3 for Polynomial and MiMe-2)

Height calibration for substance THCA-A @ RT White:

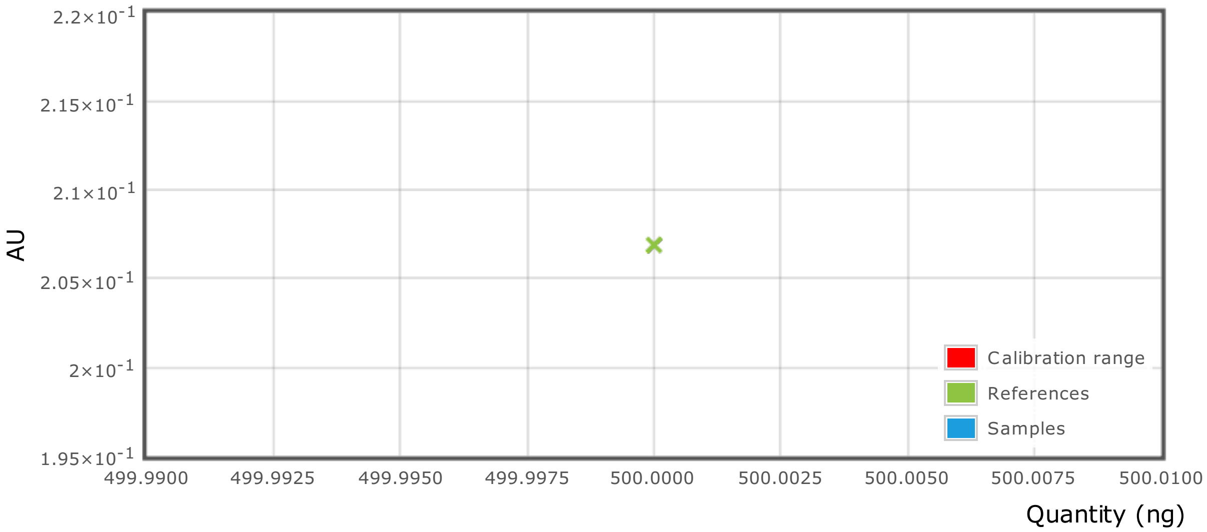

Regression mode	Linear-2
Range deviation	5.00 %
Related substances	Default
Number of references	1
Calibration function	$y=0x$
Coefficient of variation	CV 0.00 %
Correlation coefficient	n/a
⚠	Unable to compute the results for this substance because there wasn't enough groups of references replicas (at least 1 for Linear-1, 2 for Linear2 and Mime-1 and 3 for Polynomial and MiMe-2)

Height calibration for substance THCV @ RT White:

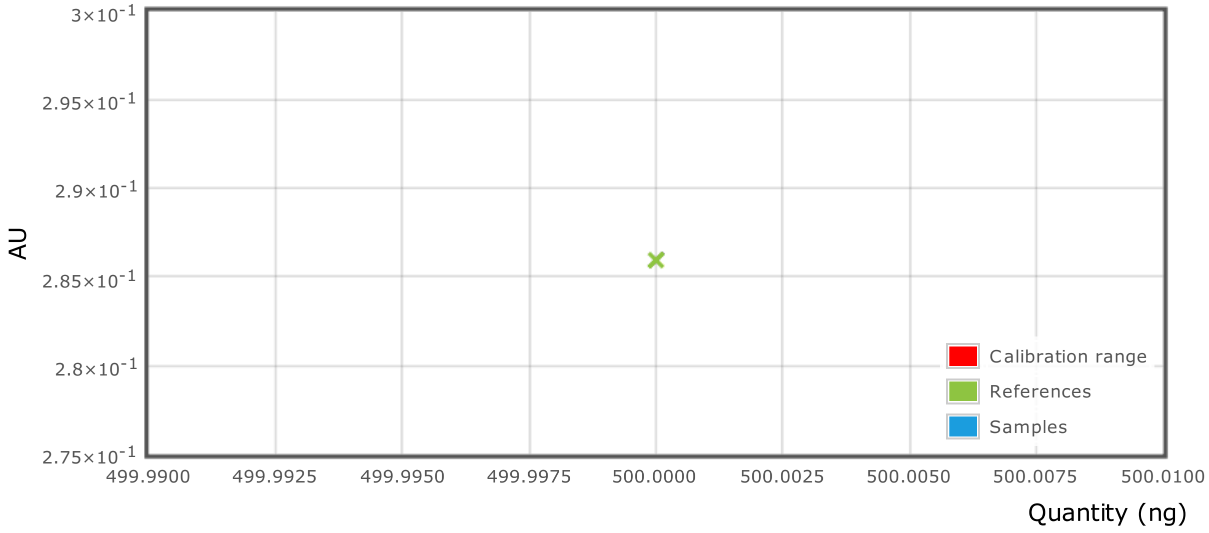

Regression mode	Linear-2
Range deviation	5.00 %
Related substances	Default
Number of references	1
Calibration function	$y=0x$
Coefficient of variation	CV 0.00 %
Correlation coefficient	n/a
⚠	Unable to compute the results for this substance because there wasn't enough groups of references replicas (at least 1 for Linear-1, 2 for Linear2 and Mime-1 and 3 for Polynomial and MiMe-2)

Results:

Substance having no available results		
⚠	THCA-A	There wasn't any sample application available in the assignments for this substance. Please check that the peaks were correctly detected and assig ned for this substance.
⚠	CBDA	There wasn't any sample application available in the assignments for this substance. Please check that the peaks were correctly detected and assig ned for this substance.
⚠	CBD	There wasn't any sample application available in the assignments for this substance. Please check that the peaks were correctly detected and assig ned for this substance.
⚠	CBC	There wasn't any sample application available in the assignments for this substance. Please check that the peaks were correctly detected and assig ned for this substance.
⚠	CBDV	There wasn't any sample application available in the assignments for this substance. Please check that the peaks were correctly detected and assig ned for this substance.
⚠	CBG	There wasn't any sample application available in the assignments for this substance. Please check that the peaks were correctly detected and assig ned for this substance.
⚠	CBN	There wasn't any sample application available in the assignments for this substance. Please check that the peaks were correctly detected and assig ned for this substance.
⚠	9-THC	There wasn't any sample application available in the assignments for this substance. Please check that the peaks were correctly detected and assig ned for this substance.
⚠	CBGA	There wasn't any sample application available in the assignments for this substance. Please check that the peaks were correctly detected and assig ned for this substance.
⚠	THCV	There wasn't any sample application available in the assignments for this substance. Please check that the peaks were correctly detected and assig ned for this substance.
⚠	8-THC	There wasn't any sample application available in the assignments for this substance. Please check that the peaks were correctly detected and assig ned for this substance.

A track marked with ⚠ means: this result is outside the regression range given by the reference assignments, but is included in the results because it is in the allowed range deviation.

Analyst:	Reviewer:
