# Supplementary material for: High Performance Thin-Layer Chromatography (HPTLC) data of Cannabinoids in ten mobile phase systems
Source: Data Brief. 2020 Jun 30;31:105955. doi: 10.1016/j.dib.2020.105955 (PMC7352075; doi:10.1016/j.dib.2020.105955)
Supplement: Supplementary file 1 [file mmc1.zip › S1-Triplicate reports/MGW-1.pdf]

Analysis: MGW-1

Path: Home/YL Research

Based on method: Triplets Method

Created	27-Jun-2019 13:32:48	visionCATSuser
Modified	27-Jun-2019 16:13:55	visionCATSuser
Last HPTLC log	27-Jun-2019 16:13:55	Analysis modified
Explorer notes		

Track	Vial ID	Description	Volume	Position	Type
1	MeOH blank	MeOH Blank	2.0 µl	A1	Sample
2	Mixture 100	200ng	2.0 µl	B1	Sample
3	9-THC 100	200ng	2.0 µl	C1	Reference
4	CBD 100	200ng	2.0 µl	D1	Reference
5	CBN 100	200ng	2.0 µl	E1	Reference
6	CBG 100	200ng	2.0 µl	F1	Reference
7	CBC 100	200ng	2.0 µl	A2	Reference
8	THCV 100	200ng	2.0 µl	B2	Reference
9	CBDV 100	200ng	2.0 µl	C2	Reference
10	8-THC 100	200ng	2.0 µl	D2	Reference
11	THCA-A 100	200ng	2.0 µl	E2	Reference
12	CBDA 100	200ng	2.0 µl	F2	Reference
13	CBGA 100	200ng	2.0 µl	A3	Reference
Sequence table notes					

A track marked with ⚠ means: the application type is overridden in some evaluation(s).

System setup:

Software	Server User-PC, version 2.5.18072.1
ATS4	S/N:080713
Chamber	N/A
Derivatization dip	N/A
Visualizer	S/N:230515

Chromatography

Plate layout:

Stationary phase	Merck, HPTLC plates RP-18 F 254s
Plate format	100.0 x 100.0 mm
Application type	Spot
Application	Position Y: 8.0 mm, length: 0.0 mm, width: 0.0 mm
Track	First position X: 20.0 mm, distance: 5.0 mm
Solvent front position	70.0 mm
Notes	

Take image clean plate 1a - Visualizer (S/N: 230515):

Quality	Enhanced
RT White	auto capture, Auto, level 85 %, Band
R 254	auto capture, Auto, level 85 %, Band
Instrument diagnostics	Valid diagnostics
Documentation step label	
Notes	

Application 1 - ATS 4 (S/N: 080713):

Spray gas	NI
Sample solvent type	Methanol
Filling speed	15 µl/s
Predosage volume	200 nl
Retraction volume	200 nl
Dosage speed	150 nl/s
Filling quality	User
Rinsing cycles / vacuum	1 / 4 s
Filling cycles / vacuum	1 / 4 s
Rinsing solvent name	Methanol
Nozzle temperature	Unheated
Rack in use	Standard
Instrument diagnostics	Valid diagnostics
Notes	

Development 1 - Chamber:

Tank	TTC 20x10
Mobile phase	
Saturation time	20 min
Use saturation pad	true
Use smartALERT	false
Volume front through	10 ml
Volume rear through	20 ml
Drying time	5 min
Drying temperature	Room temperature
Notes	

Take image developed plate 1a - Visualizer (S/N: 230515):

Quality	Enhanced
RT White	auto capture, Auto, level 85 %, Band
R 254	auto capture, Auto, level 85 %, Band
R 366	auto capture, Auto, level 85 %, Band
Instrument diagnostics	Valid diagnostics
Documentation step label	
Notes	

Scan developed plate 1b - Scanner 3 (S/N: 031025):

Scanner type	Single λ
Optimization for	Resolution
Measurement mode	Absorption
Filter	n/a
Detector mode	Automatic
Scanning speed	20 mm/s
Data resolution	100 µm/step
Slit	5 x 0.2 mm, micro
Partial scan	No
Lamp	Deuterium & Tungsten
Wavelength(s)	254 nm
Instrument diagnostics	Valid diagnostics
Documentation step label	
Notes	

Derivatization 1 - dip:

Reagent name	Fast Blue B salt
Dipping speed	3
Dipping time	5 s
Reagent preparation	1g Fast Blue B salt in 200mL water
Heating	none
Notes	Air dry for 5 minutes

Take image derivatized plate 1a - Visualizer (S/N: 230515):

Quality	Enhanced
RT White	auto capture, Auto, level 85 %, Band
R 366	auto capture, Auto, level 85 %, Band
Instrument diagnostics	Valid diagnostics
Documentation step label	
Notes	

Take image derivatized plate 1b - Visualizer (S/N: 230515):

Quality	Enhanced
RT White	auto capture, Auto, level 85 %, Band
R 366	auto capture, Auto, level 85 %, Band
Instrument diagnostics	Valid diagnostics
Documentation step label	
Notes	

System suitability tests:

SST settings:

SST tracks	

Data acquisition

Application 1 - ATS 4 (S/N: 080713):

Executed	27-Jun-2019 13:40:29 visionCATSuser

Development 1 - Chamber:

Executed	27-Jun-2019 15:12:25 visionCATSuser

Take image developed plate 1a - Visualizer (S/N: 230515):

Executed	27-Jun-2019 15:13:20 visionCATSuser

RT White Developed, RemTransVis

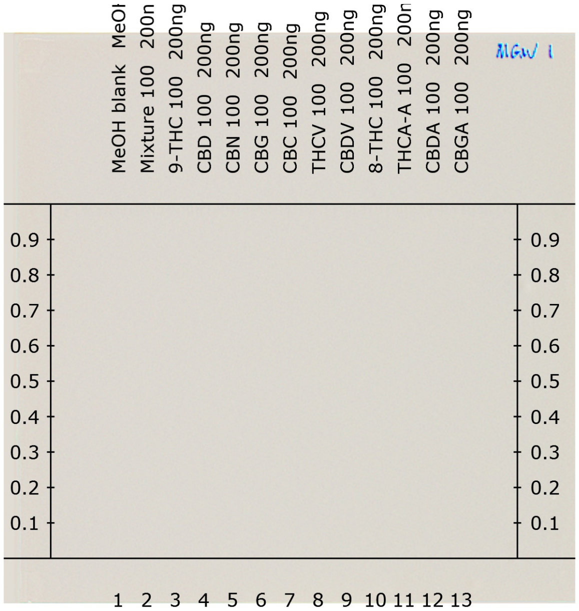

Exposure	0.047 s
Contrast	1
Normalized exposure	Disabled
Clarify	Disabled
White balance	1.00, 1.00, 1.00

R 254 Developed, Remission254

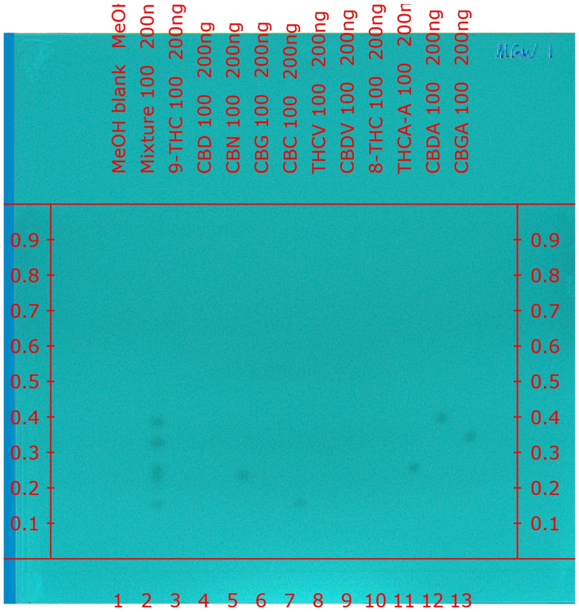

Exposure	0.209 s
Contrast	1
Normalized exposure	Disabled
Clarify	Disabled
White balance	1.00, 1.00, 1.00

R 366 Developed, Remission366

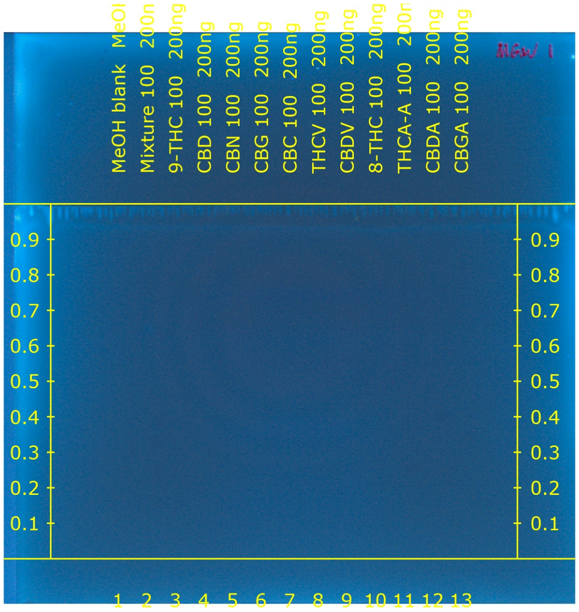

Exposure	6.388 s
Contrast	1
Normalized exposure	Disabled
Clarify	Disabled
White balance	1.00, 1.00, 1.00

Derivatization 1 - dip:

Executed	27-Jun-2019 15:27:24 visionCATSuser

Take image derivatized plate 1b - Visualizer (S/N: 230515):

Executed	27-Jun-2019 15:57:25 visionCATSuser

RT White Derivatized, RemTransVis

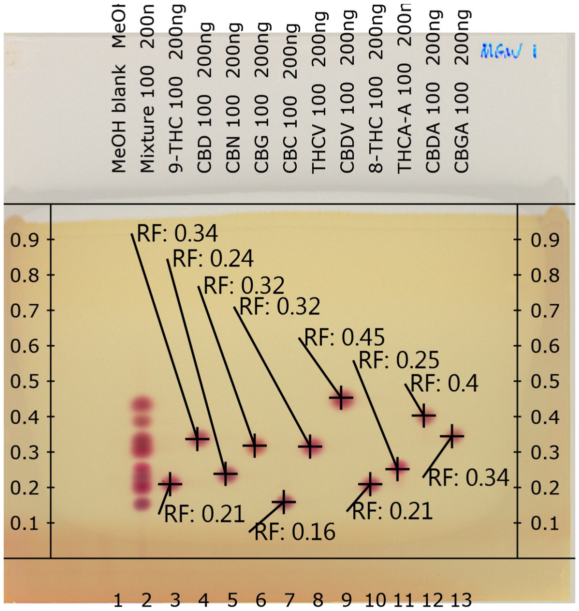

Exposure	0.063 s
Contrast	1
Normalized exposure	Disabled
Clarify	Disabled
White balance	1.01, 1.00, 1.00

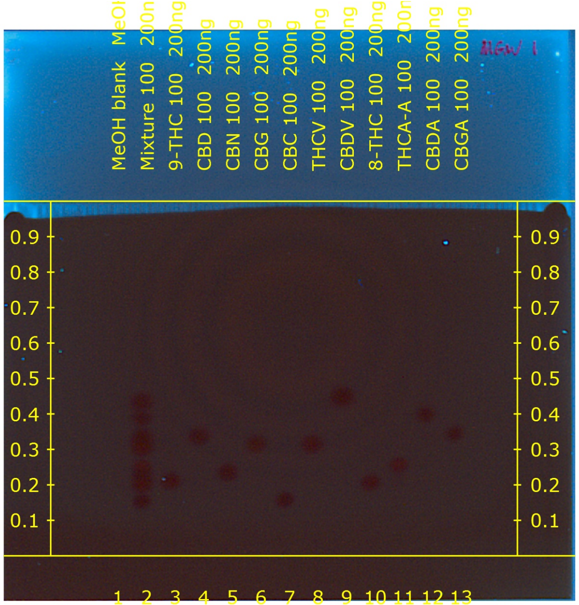

Exposure	8.050 s
Contrast	1
Normalized exposure	Disabled
Clarify	Disabled
White balance	1.00, 1.00, 1.00

Evaluation 1 :

Validated	false
Step	Take image derivatized plate 1b
Concentration unit type	Mass / volume
Notes	

Definition:

References:

9-THC 100

Substance Name	Concentration	Purity
9-THC	100.000 µg/ml	100.00 %

CBD 100

Substance Name	Concentration	Purity
CBD	100.000 µg/ml	100.00 %

CBN 100

Substance Name	Concentration	Purity
CBN	100.000 µg/ml	100.00 %

CBG 100

Substance Name	Concentration	Purity
CBG	100.000 µg/ml	100.00 %

CBC 100		
Substance Name	Concentration	Purity
CBC	100.000 µg/ml	100.00 %

THCV 100		
Substance Name	Concentration	Purity
THCV	100.000 µg/ml	100.00 %

CBDV 100		
Substance Name	Concentration	Purity
CBDV	100.000 µg/ml	100.00 %

8-THC 100		
Substance Name	Concentration	Purity
8-THC	100.000 µg/ml	100.00 %

THCA-A 100		
Substance Name	Concentration	Purity
THCA-A	100.000 µg/ml	100.00 %

CBDA 100		
Substance Name	Concentration	Purity
CBDA	100.000 µg/ml	100.00 %

CBGA 100		
Substance Name	Concentration	Purity
CBGA	100.000 µg/ml	100.00 %

Samples:

Vial ID	Amount	Volume solution	Reference amount	Related to
MeOH blank		0.00 ml		
Mixture 100		0.00 ml		

Integration parameters:

Bounds	[0.000,1.000]
Smoothing	Savitzky–Golay of order 3 and window 7
Baseline correction	Lowest slope with noise 0.05
Profile subtraction	Profile subtraction from track 1
Peaks detection	Gauss (legacy) with sensitivity 0.1, separation 1 and threshold 0.05

Scan:

Wavelength	RT White

Track 1:

Type	Sample
Vial ID	MeOH blank
Description	MeOH Blank
Volume	2.0 µl

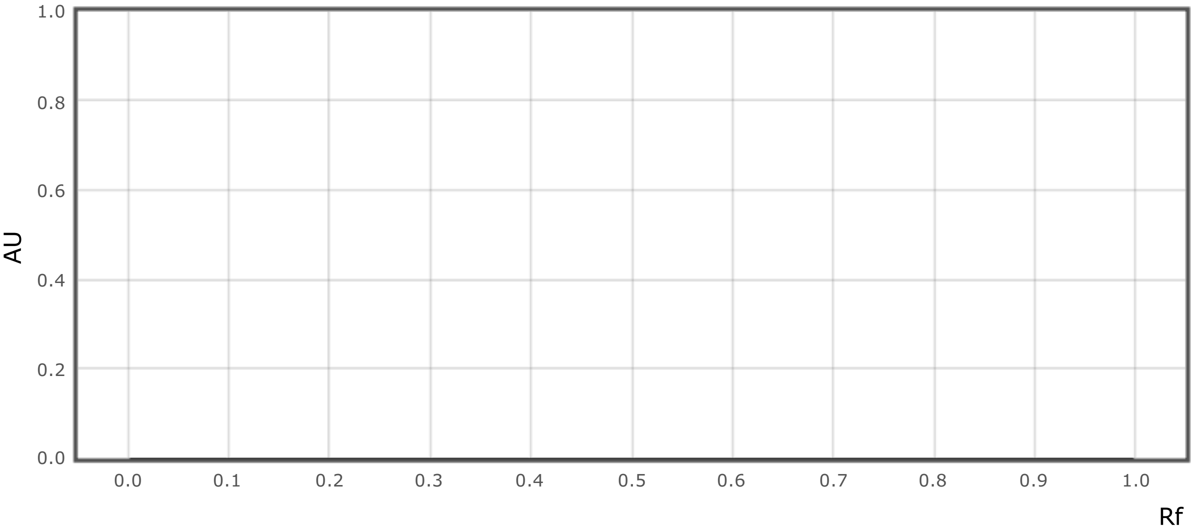

Peak	Start		Max			End		Area		Manual	Substance
#	Rf	H	Rf	H	%	Rf	H	A	%	peak	Name

Track 2:	
Type	Sample
Vial ID	Mixture 100
Description	200ng
Volume	2.0 µl

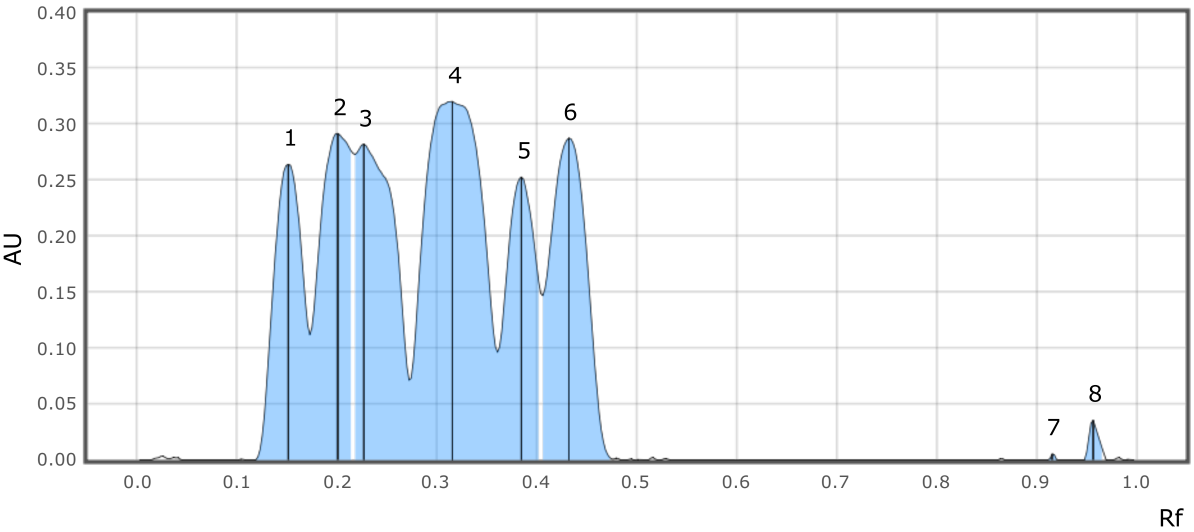

Peak #	Start Rf	Start H	Max Rf	Max H	Max %	End Rf	End H	Area A	Area %	Manual peak	Substance Name
1	0.116	0.0000	0.151	0.2642	15.19	0.173	0.1119	0.00875	11.77	No	
2	0.173	0.1119	0.201	0.2918	16.77	0.216	0.2741	0.01052	14.14	No	
3	0.218	0.2730	0.227	0.2822	16.22	0.272	0.0713	0.01244	16.73	No	
4	0.272	0.0713	0.315	0.3204	18.42	0.361	0.0963	0.02178	29.28	No	
5	0.361	0.0963	0.384	0.2529	14.54	0.404	0.1491	0.00847	11.38	No	
6	0.406	0.1469	0.432	0.2876	16.53	0.475	0.0007	0.01200	16.14	No	
7	0.912	0.0000	0.916	0.0053	0.30	0.922	0.0000	0.00002	0.03	No	
8	0.948	0.0000	0.957	0.0354	2.04	0.970	0.0000	0.00039	0.53	No	

Track 3:

Type	Reference
Vial ID	9-THC 100
Description	200ng
Volume	2.0 µl

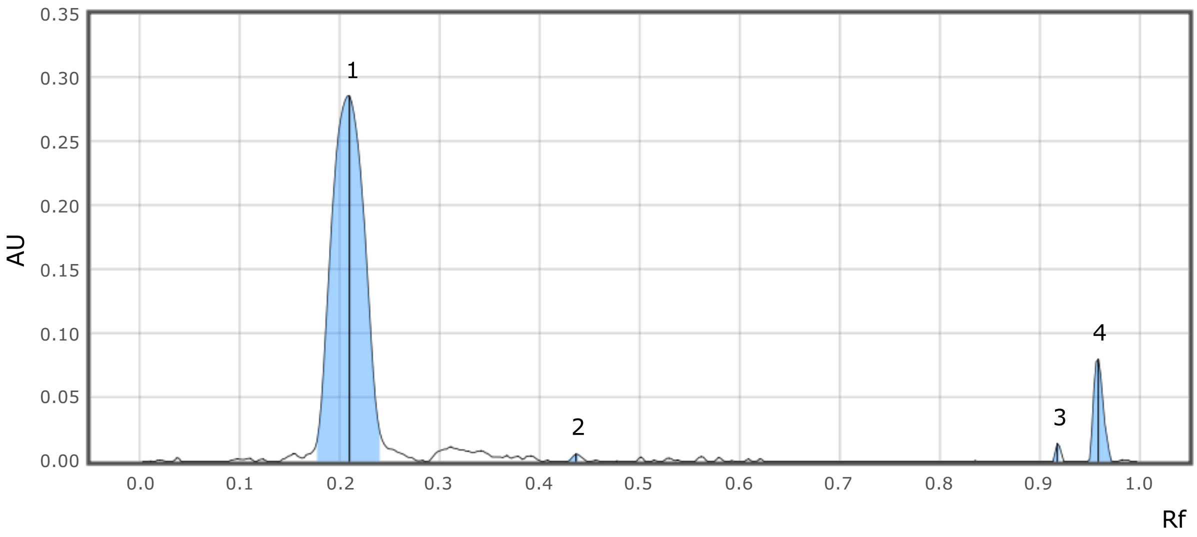

Peak #	Start Rf	Start H	Max Rf	Max H	Max %	End Rf	End H	Area A	Area %	Manual peak	Substance Name
1	0.176	0.0159	0.209	0.2859	74.13	0.241	0.0173	0.01106	91.45	Yes	9-THC
2	0.428	0.0000	0.436	0.0057	1.48	0.447	0.0000	0.00006	0.48	No	
3	0.914	0.0000	0.918	0.0141	3.65	0.925	0.0000	0.00008	0.69	No	
4	0.948	0.0000	0.959	0.0800	20.73	0.974	0.0000	0.00089	7.38	No	

Track 4:

Type	Reference
Vial ID	CBD 100
Description	200ng
Volume	2.0 µl

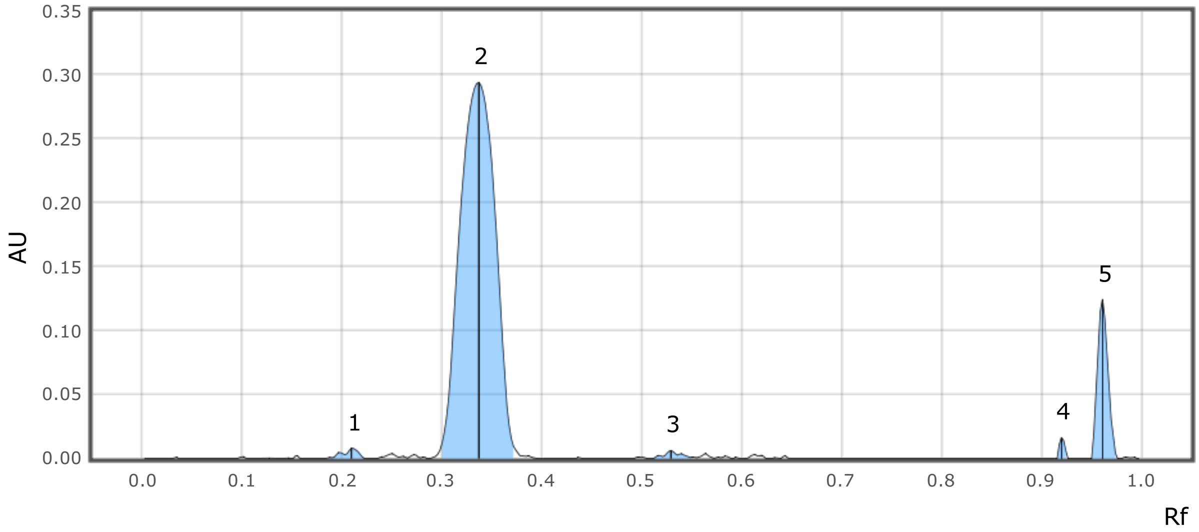

Peak #	Start		Max			End		Area		Manual peak	Substance Name
	Rf	H	Rf	H	%	Rf	H	A	%		
1	0.183	0.0000	0.209	0.0079	1.77	0.222	0.0000	0.00014	0.97	No	
2	0.299	0.0118	0.337	0.2939	65.52	0.373	0.0069	0.01210	86.71	Yes	CBD
3	0.512	0.0000	0.529	0.0060	1.34	0.555	0.0006	0.00011	0.79	No	
4	0.916	0.0000	0.920	0.0163	3.64	0.927	0.0000	0.00010	0.73	No	
5	0.951	0.0000	0.961	0.1244	27.73	0.977	0.0000	0.00151	10.80	No	

Track 5:

Type	Reference
Vial ID	CBN 100
Description	200ng
Volume	2.0 µl

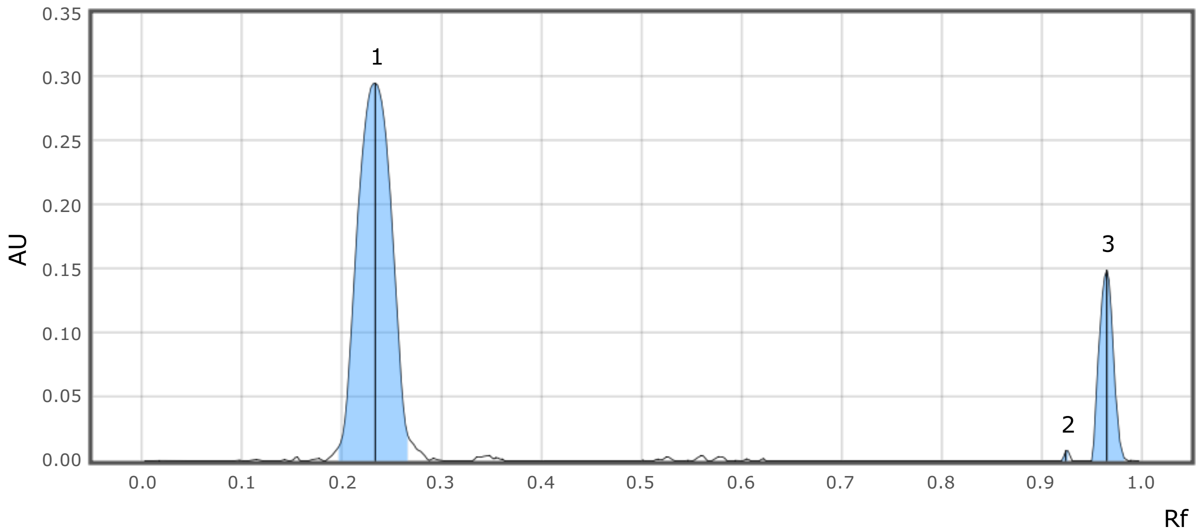

Peak #	Start		Max			End		Area		Manual peak	Substance Name
	Rf	H	Rf	H	%	Rf	H	A	%		
1	0.196	0.0106	0.233	0.2952	65.31	0.268	0.0164	0.01180	82.61	Yes	CBN
2	0.920	0.0000	0.925	0.0078	1.73	0.931	0.0000	0.00005	0.34	No	
3	0.951	0.0000	0.966	0.1490	32.96	0.987	0.0000	0.00244	17.05	No	

Track 6:

Type	Reference
Vial ID	CBG 100
Description	200ng
Volume	2.0 µl

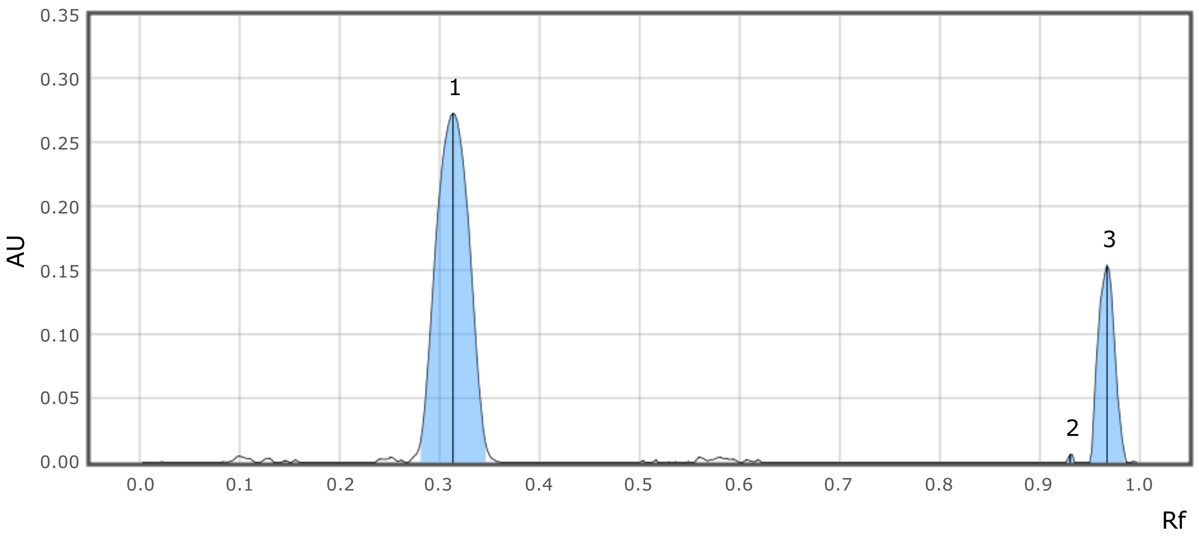

Peak #	Start		Max			End		Area		Manual peak	Substance Name
	Rf	H	Rf	H	%	Rf	H	A	%		
1	0.280	0.0164	0.313	0.2732	62.93	0.346	0.0153	0.01067	78.02	Yes	CBG
2	0.927	0.0000	0.931	0.0065	1.49	0.935	0.0000	0.00003	0.25	No	
3	0.951	0.0000	0.968	0.1545	35.57	0.987	0.0000	0.00297	21.73	No	

Track 7:

Type	Reference
Vial ID	CBC 100
Description	200ng
Volume	2.0 µl

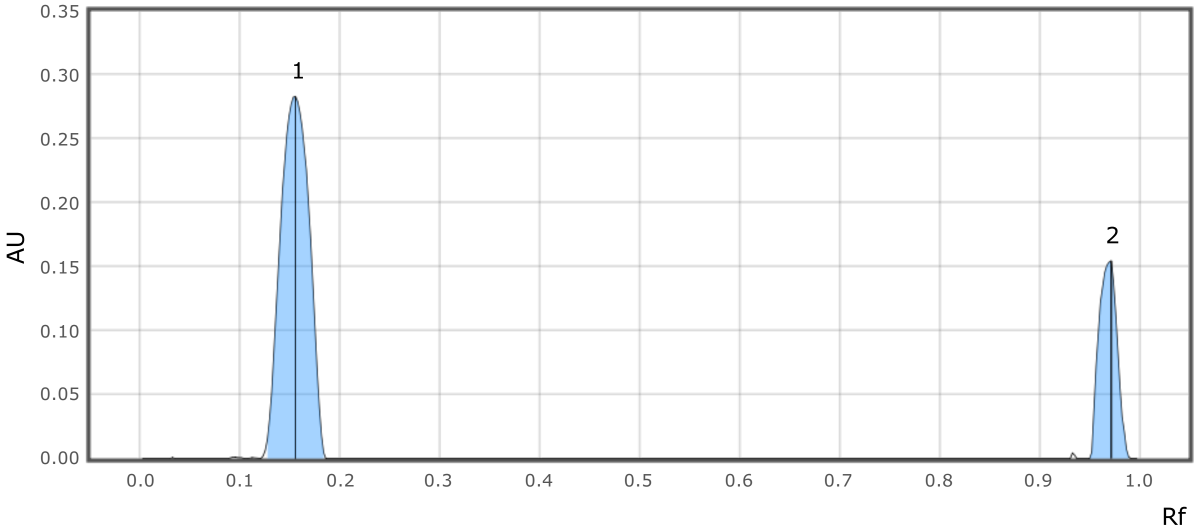

Peak #	Start		Max			End		Area		Manual peak	Substance Name
	Rf	H	Rf	H	%	Rf	H	A	%		
1	0.126	0.0064	0.155	0.2832	64.69	0.186	0.0000	0.00962	74.45	Yes	CBC
2	0.951	0.0000	0.972	0.1546	35.31	0.992	0.0000	0.00330	25.55	No	

Track 8:

Type	Reference
Vial ID	THCV 100
Description	200ng
Volume	2.0 µl

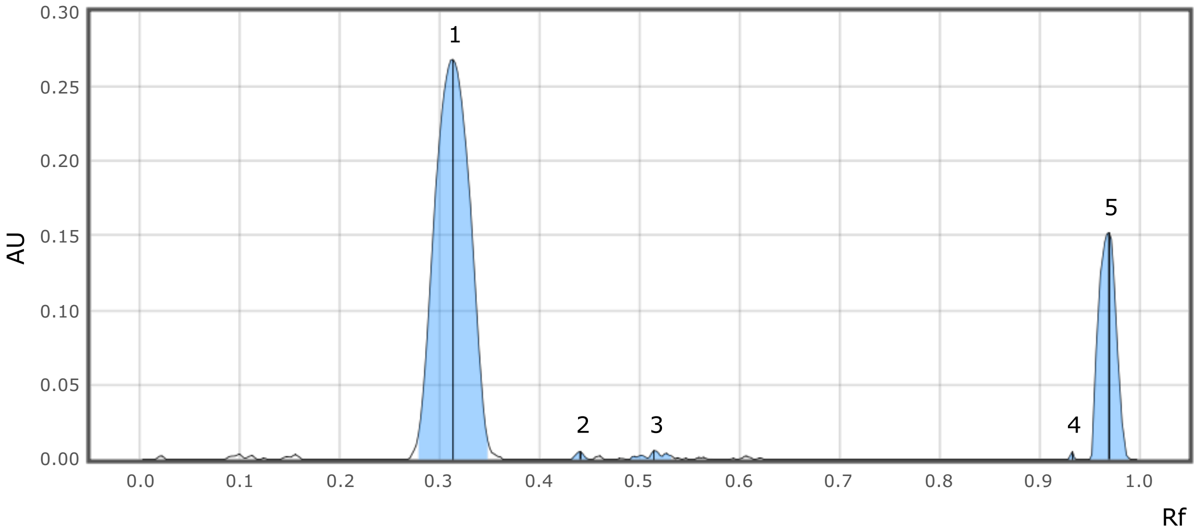

Peak #	Start		Max			End		Area		Manual peak	Substance Name
	Rf	H	Rf	H	%	Rf	H	A	%		
1	0.277	0.0099	0.313	0.2678	61.48	0.349	0.0127	0.01111	76.99	Yes	THCV
2	0.430	0.0000	0.441	0.0051	1.17	0.451	0.0000	0.00005	0.32	No	
3	0.488	0.0000	0.514	0.0058	1.32	0.536	0.0003	0.00012	0.82	No	
4	0.929	0.0000	0.933	0.0052	1.19	0.938	0.0000	0.00002	0.13	No	
5	0.951	0.0000	0.970	0.1518	34.84	0.992	0.0000	0.00314	21.74	No	

Track 9:

Type	Reference
Vial ID	CBDV 100
Description	200ng
Volume	2.0 µl

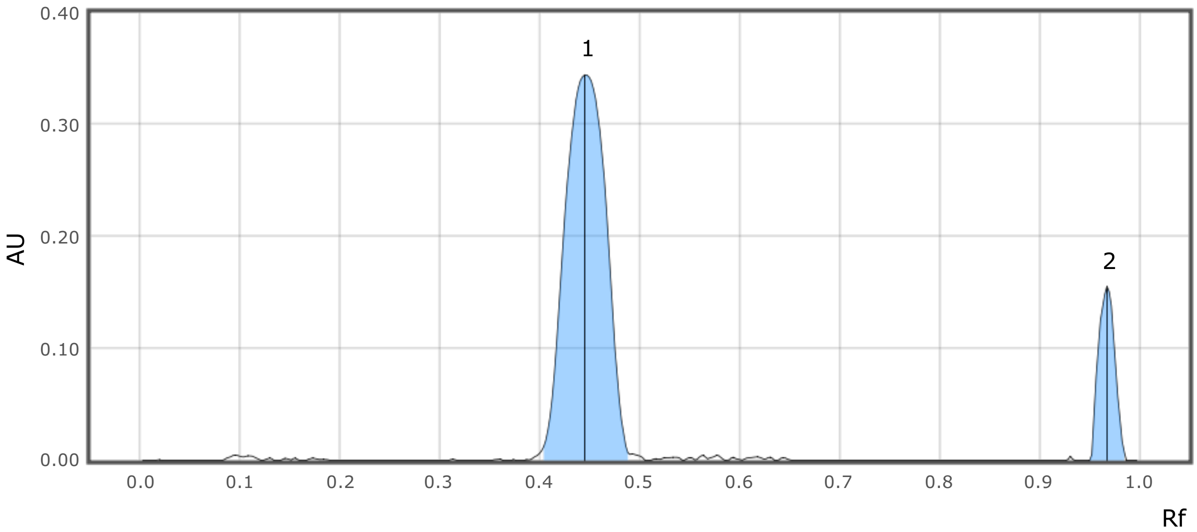

Peak	Start		Max			End		Area		Manual	Substance
#	Rf	H	Rf	H	%	Rf	H	A	%	peak	Name
1	0.403	0.0121	0.445	0.3442	68.92	0.490	0.0053	0.01659	84.67	Yes	CBDV
2	0.951	0.0000	0.968	0.1552	31.08	0.987	0.0000	0.00300	15.33	No	

Track 10:

Type	Reference
Vial ID	8-THC 100
Description	200ng
Volume	2.0 µl

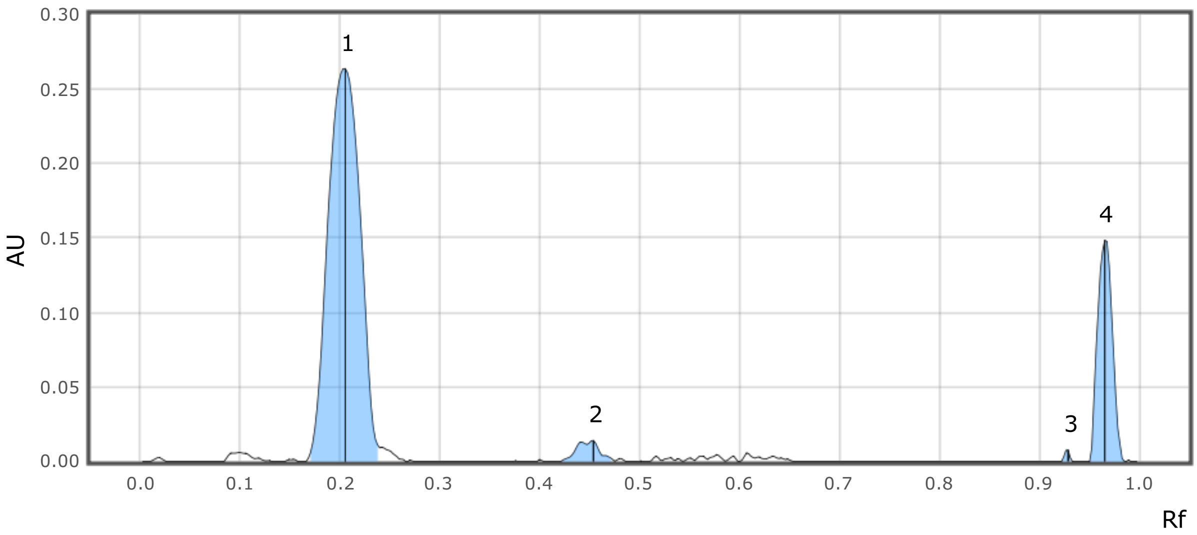

Peak #	Start Rf	Start H	Max Rf	Max H	Max %	End Rf	End H	Area A	Area %	Manual peak	Substance Name
1	0.170	0.0060	0.205	0.2634	60.80	0.238	0.0111	0.00980	76.75	Yes	8-THC
2	0.421	0.0000	0.454	0.0141	3.25	0.475	0.0001	0.00037	2.92	No	
3	0.922	0.0000	0.929	0.0075	1.74	0.933	0.0000	0.00004	0.35	No	
4	0.951	0.0000	0.966	0.1482	34.21	0.987	0.0000	0.00255	19.99	No	

Track 11:

Type	Reference
Vial ID	THCA-A 100
Description	200ng
Volume	2.0 µl

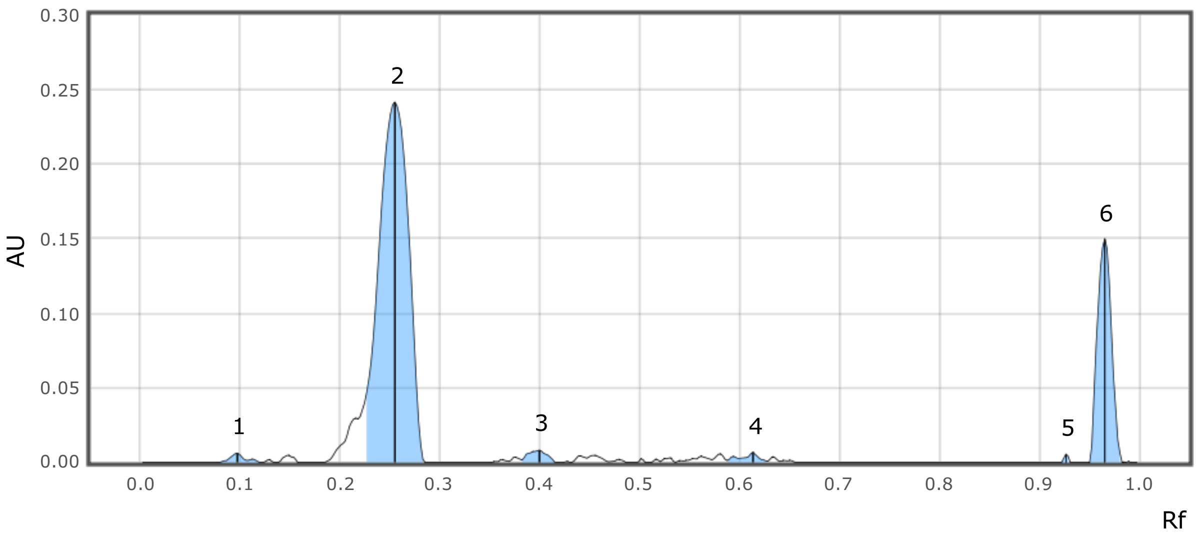

Peak #	Start Rf	Start H	Max Rf	Max H	Max %	End Rf	End H	Area A	Area %	Manual peak	Substance Name
1	0.080	0.0000	0.097	0.0061	1.47	0.121	0.0000	0.00010	0.89	No	
2	0.226	0.0468	0.255	0.2413	57.79	0.285	0.0000	0.00822	74.10	Yes	THCA-A
3	0.382	0.0015	0.400	0.0081	1.95	0.417	0.0000	0.00018	1.58	No	
4	0.588	0.0012	0.613	0.0067	1.61	0.629	0.0009	0.00013	1.18	No	
5	0.922	0.0000	0.927	0.0055	1.32	0.931	0.0000	0.00003	0.24	No	
6	0.951	0.0000	0.966	0.1497	35.86	0.983	0.0000	0.00244	21.99	No	

Track 12:

Type	Reference
Vial ID	CBDA 100
Description	200ng
Volume	2.0 µl

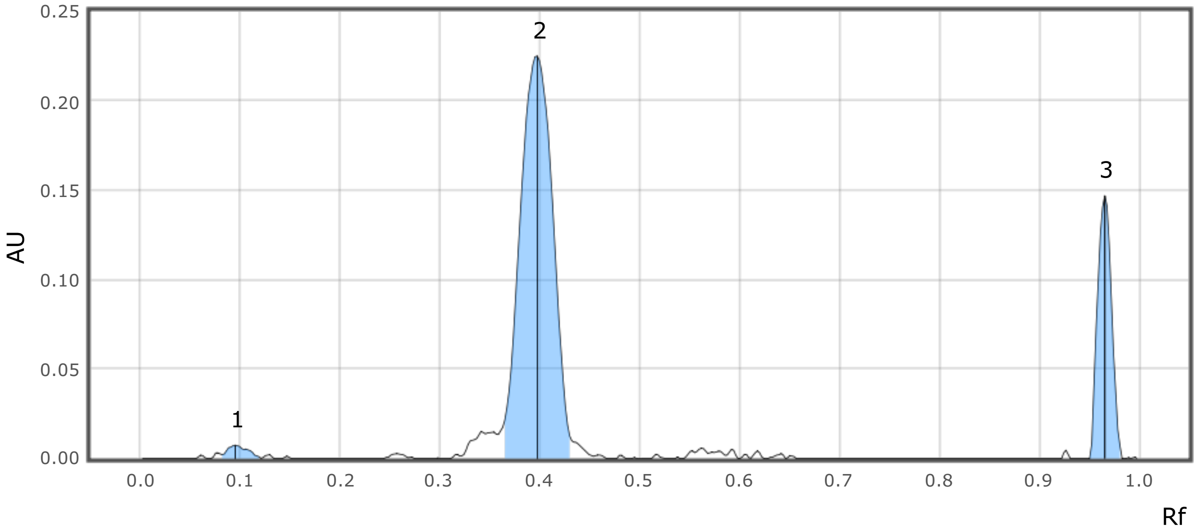

Peak #	Start		Max			End		Area		Manual peak	Substance Name
	Rf	H	Rf	H	%	Rf	H	A	%		
1	0.082	0.0021	0.095	0.0073	1.94	0.121	0.0001	0.00017	1.57	No	
2	0.363	0.0174	0.397	0.2248	59.32	0.431	0.0099	0.00853	76.92	Yes	CBDA
3	0.951	0.0000	0.966	0.1468	38.74	0.983	0.0000	0.00238	21.51	No	

Track 13:

Type	Reference
Vial ID	CBGA 100
Description	200ng
Volume	2.0 µl

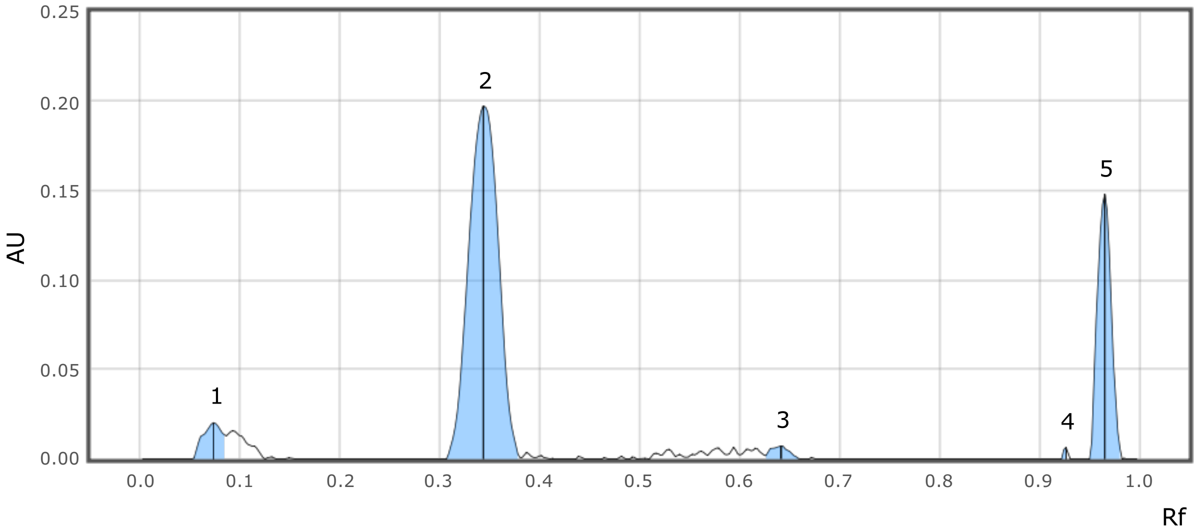

Peak #	Start		Max			End		Area		Manual peak	Substance Name
	Rf	H	Rf	H	%	Rf	H	A	%		
1	0.052	0.0000	0.073	0.0202	5.33	0.086	0.0129	0.00047	4.73	No	
2	0.307	0.0000	0.343	0.1972	52.04	0.380	0.0008	0.00683	69.30	No	CBGA
3	0.626	0.0018	0.642	0.0073	1.92	0.661	0.0000	0.00015	1.53	No	
4	0.922	0.0000	0.927	0.0064	1.70	0.931	0.0000	0.00003	0.33	No	
5	0.951	0.0000	0.966	0.1478	39.01	0.983	0.0000	0.00237	24.11	No	

Calibration results:

Height calibration for substance 8-THC @ RT White:

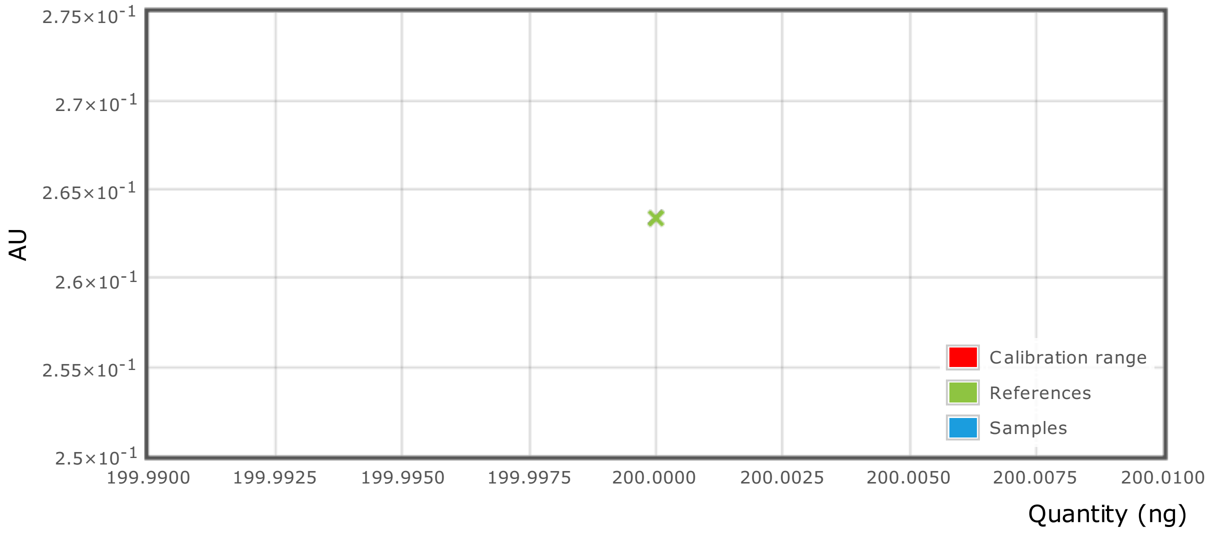

Regression mode	Linear-2
Range deviation	5.00 %
Related substances	Default
Number of references	1
Calibration function	$y=0x$
Coefficient of variation	CV 0.00 %
Correlation coefficient	n/a
⚠	Unable to compute the results for this substance because there wasn't eno ugh groups of references replicas (at least 1 for Linear-1, 2 for Linear2 and Mime-1 and 3 for Polynomial and MiMe-2)

Height calibration for substance 9-THC @ RT White:

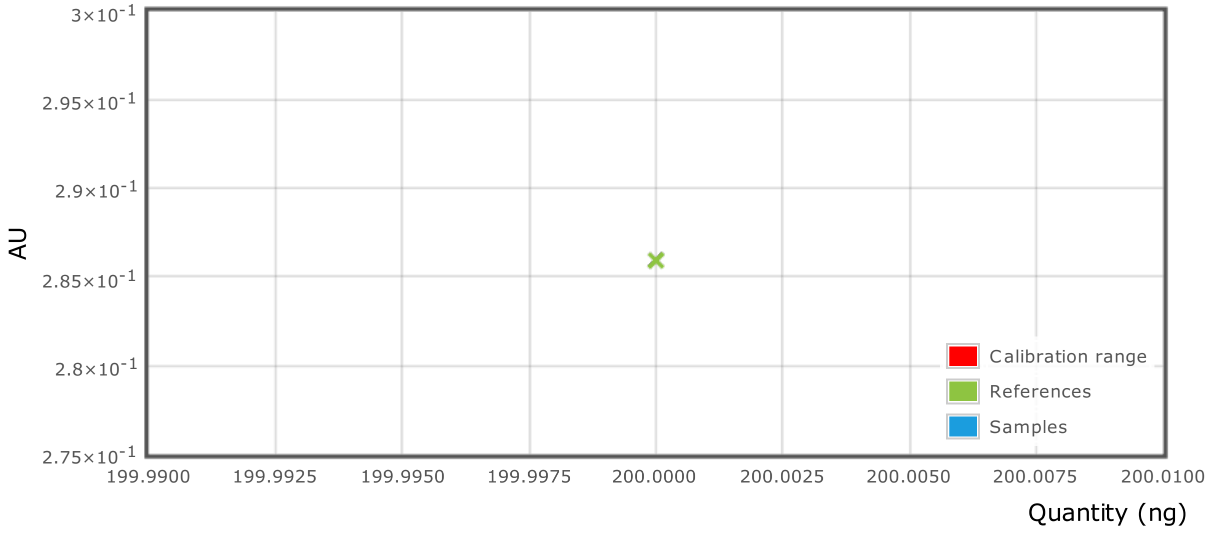

Regression mode	Linear-2
Range deviation	5.00 %
Related substances	Default
Number of references	1
Calibration function	$y=0x$
Coefficient of variation	CV 0.00 %
Correlation coefficient	n/a
⚠	Unable to compute the results for this substance because there wasn't enough groups of references replicas (at least 1 for Linear-1, 2 for Linear2 and Mime-1 and 3 for Polynomial and MiMe-2)

Height calibration for substance CBC @ RT White:

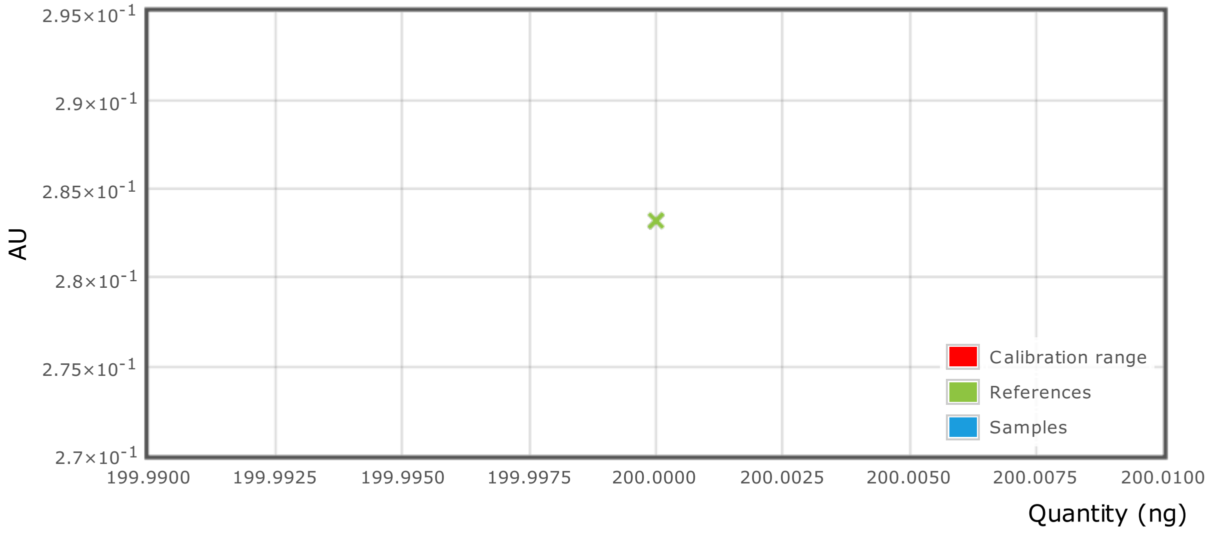

Regression mode	Linear-2
Range deviation	5.00 %
Related substances	Default
Number of references	1
Calibration function	$y=0x$
Coefficient of variation	CV 0.00 %
Correlation coefficient	n/a
⚠	Unable to compute the results for this substance because there wasn't enough groups of references replicas (at least 1 for Linear-1, 2 for Linear2 and Mime-1 and 3 for Polynomial and MiMe-2)

Height calibration for substance CBD @ RT White:

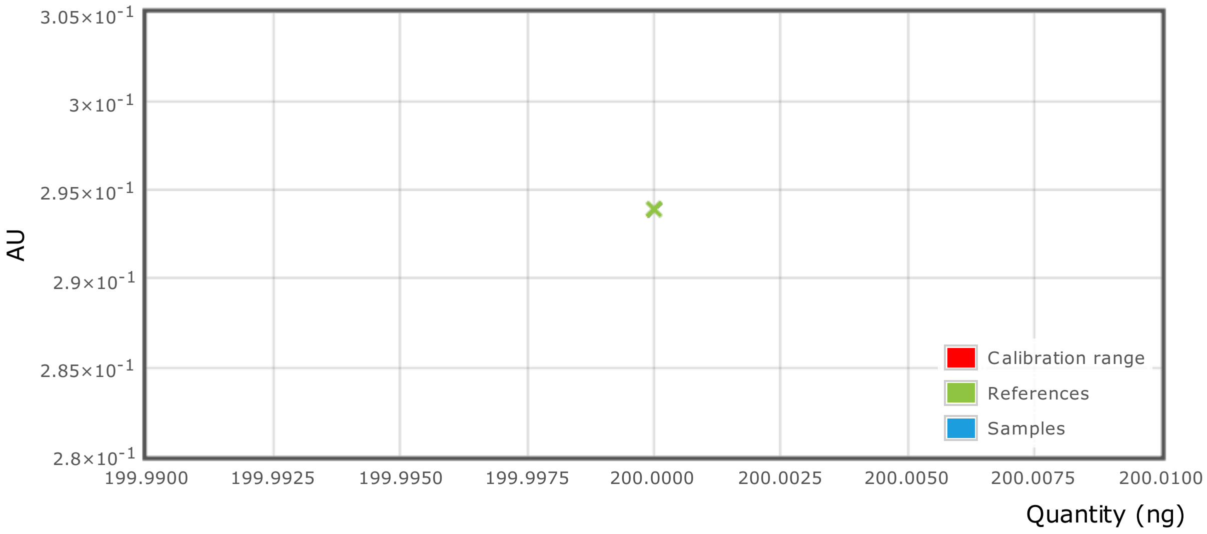

Regression mode	Linear-2
Range deviation	5.00 %
Related substances	Default
Number of references	1
Calibration function	$y=0x$
Coefficient of variation	CV 0.00 %
Correlation coefficient	n/a
⚠	Unable to compute the results for this substance because there wasn't enough groups of references replicas (at least 1 for Linear-1, 2 for Linear2 and Mime-1 and 3 for Polynomial and MiMe-2)

Height calibration for substance CBDA @ RT White:

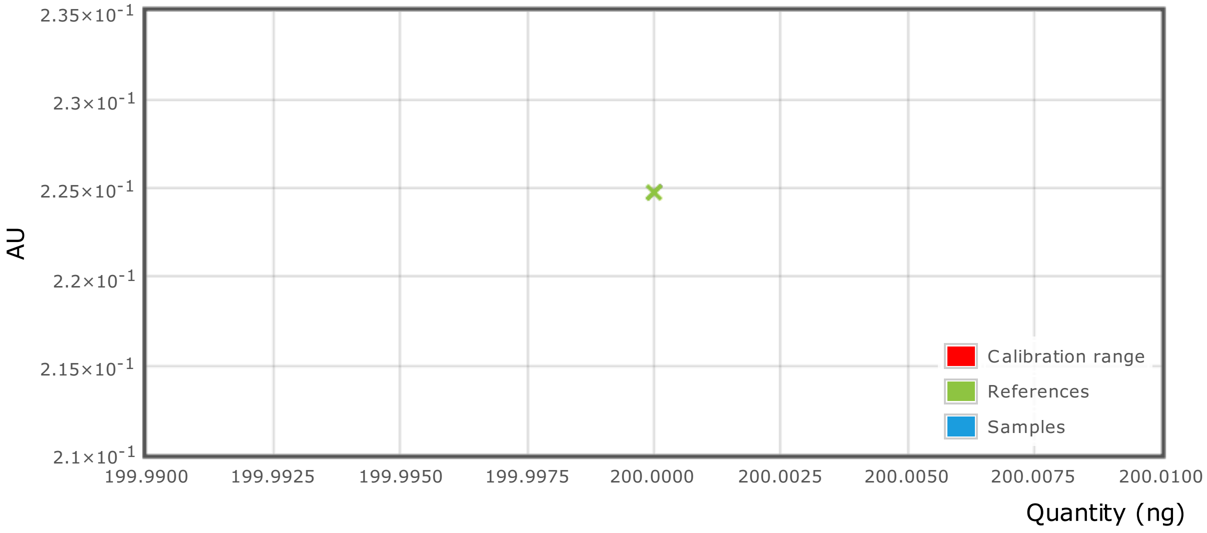

Regression mode	Linear-2
Range deviation	5.00 %
Related substances	Default
Number of references	1
Calibration function	$y = 0x$
Coefficient of variation	CV 0.00 %
Correlation coefficient	n/a
⚠	Unable to compute the results for this substance because there wasn't enough groups of references replicas (at least 1 for Linear-1, 2 for Linear2 and Mime-1 and 3 for Polynomial and MiMe-2)

Height calibration for substance CBDV @ RT White:

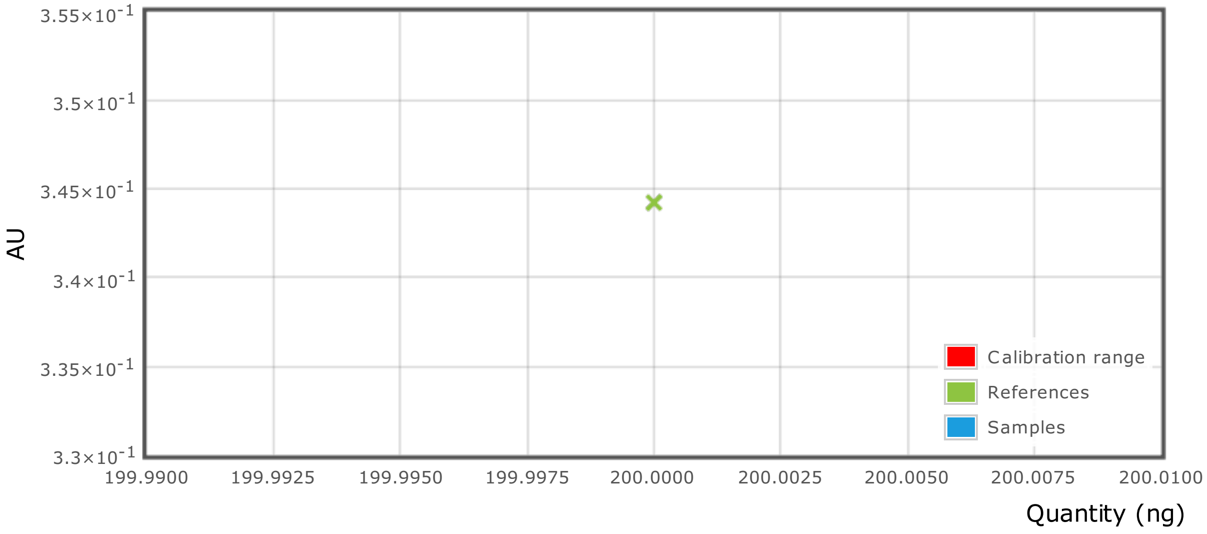

Regression mode	Linear-2
Range deviation	5.00 %
Related substances	Default
Number of references	1
Calibration function	$y=0x$
Coefficient of variation	CV 0.00 %
Correlation coefficient	n/a
⚠	Unable to compute the results for this substance because there wasn't enough groups of references replicas (at least 1 for Linear-1, 2 for Linear2 and Mime-1 and 3 for Polynomial and MiMe-2)

Height calibration for substance CBG @ RT White:

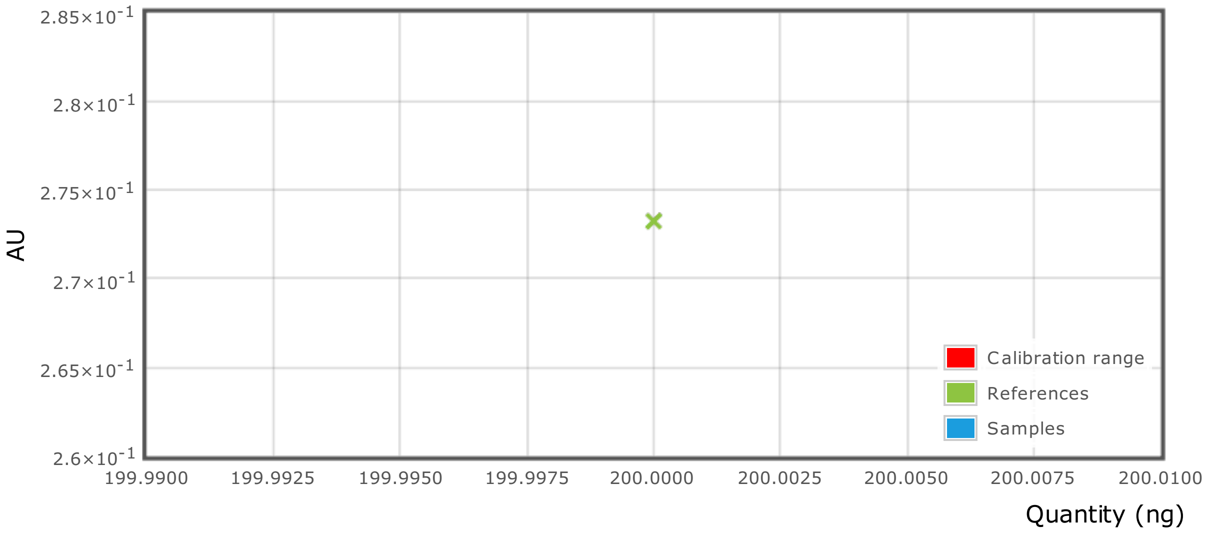

Regression mode	Linear-2
Range deviation	5.00 %
Related substances	Default
Number of references	1
Calibration function	$y=0x$
Coefficient of variation	CV 0.00 %
Correlation coefficient	n/a
⚠	Unable to compute the results for this substance because there wasn't enough groups of references replicas (at least 1 for Linear-1, 2 for Linear2 and Mime-1 and 3 for Polynomial and MiMe-2)

Height calibration for substance CBGA @ RT White:

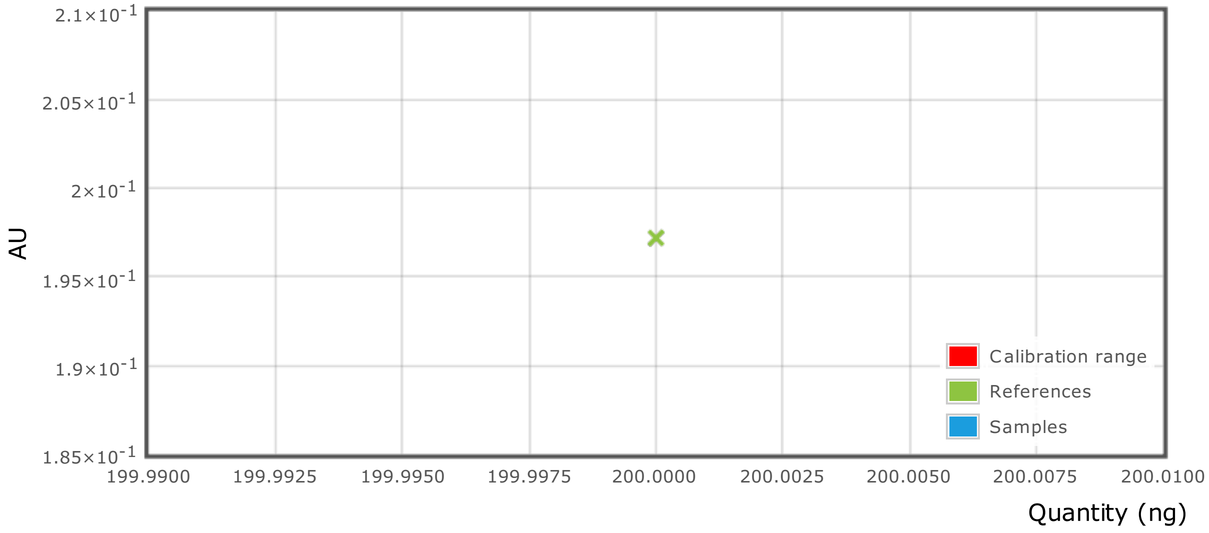

Regression mode	Linear-2
Range deviation	5.00 %
Related substances	Default
Number of references	1
Calibration function	$y=0x$
Coefficient of variation	CV 0.00 %
Correlation coefficient	n/a
⚠	Unable to compute the results for this substance because there wasn't eno ugh groups of references replicas (at least 1 for Linear-1, 2 for Linear2 and Mime-1 and 3 for Polynomial and MiMe-2)

Height calibration for substance CBN @ RT White:

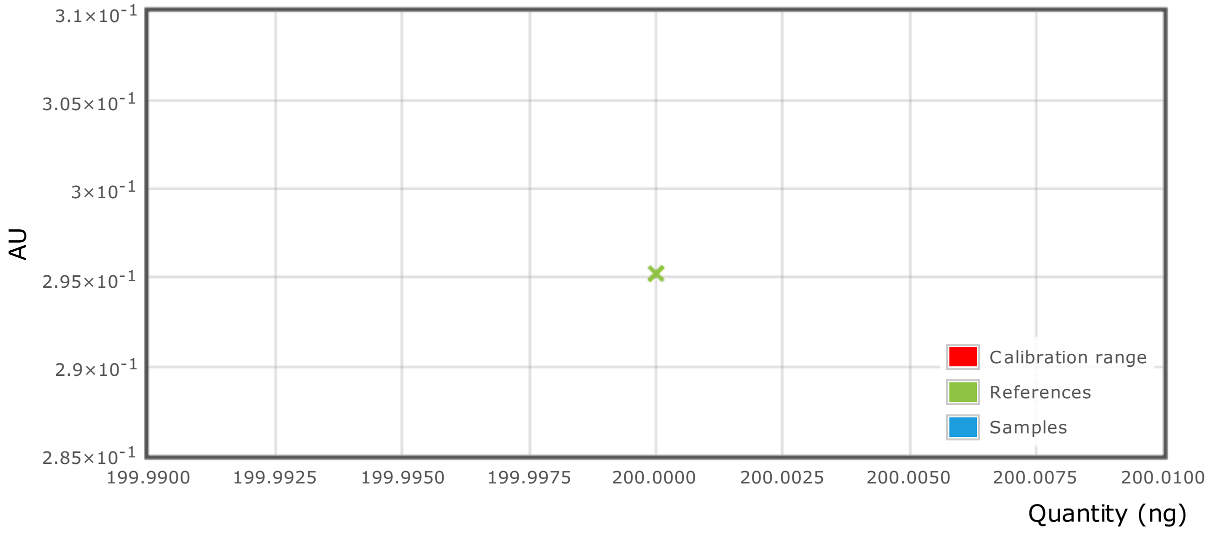

Regression mode	Linear-2
Range deviation	5.00 %
Related substances	Default
Number of references	1
Calibration function	$y=0x$
Coefficient of variation	CV 0.00 %
Correlation coefficient	n/a
⚠	Unable to compute the results for this substance because there wasn't enough groups of references replicas (at least 1 for Linear-1, 2 for Linear2 and Mime-1 and 3 for Polynomial and MiMe-2)

Height calibration for substance THCA-A @ RT White:

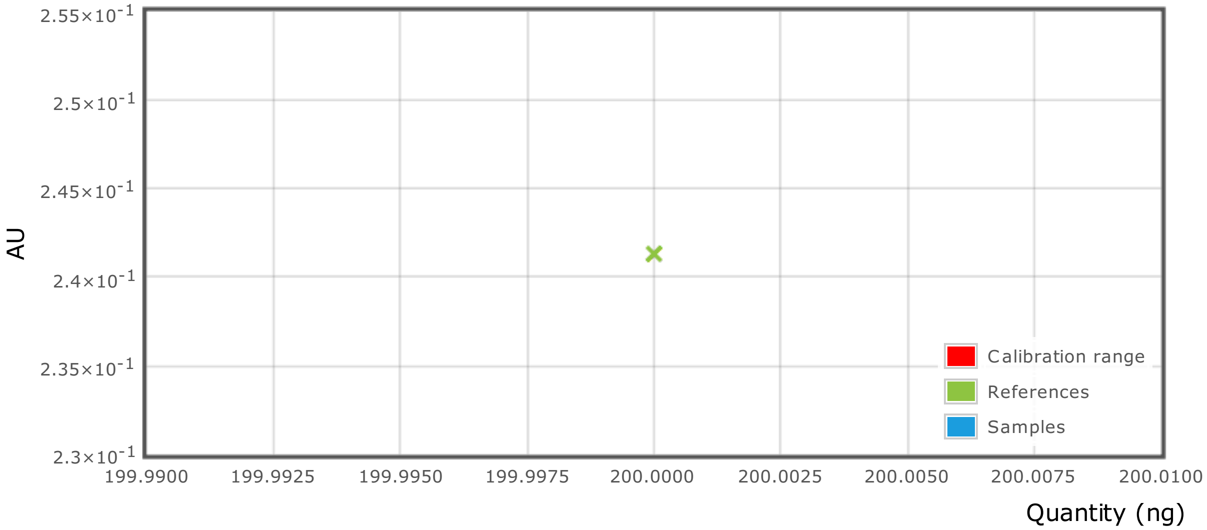

Regression mode	Linear-2
Range deviation	5.00 %
Related substances	Default
Number of references	1
Calibration function	$y=0x$
Coefficient of variation	CV 0.00 %
Correlation coefficient	n/a
⚠	Unable to compute the results for this substance because there wasn't enough groups of references replicas (at least 1 for Linear-1, 2 for Linear2 and Mime-1 and 3 for Polynomial and MiMe-2)

Height calibration for substance THCV @ RT White:

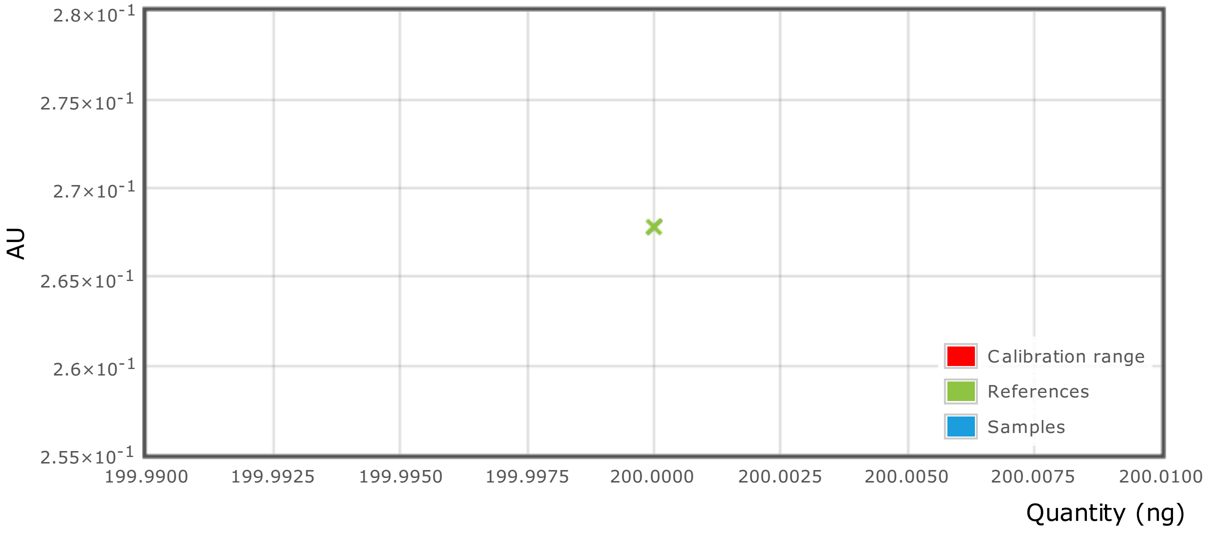

Regression mode	Linear-2
Range deviation	5.00 %
Related substances	Default
Number of references	1
Calibration function	$y=0x$
Coefficient of variation	CV 0.00 %
Correlation coefficient	n/a
⚠	Unable to compute the results for this substance because there wasn't enough groups of references replicas (at least 1 for Linear-1, 2 for Linear2 and Mime-1 and 3 for Polynomial and MiMe-2)

Results:

Substance having no available results		
⚠	8-THC	There wasn't any sample application available in the assignments for this substance. Please check that the peaks were correctly detected and assigned for this substance.
⚠	CBD	There wasn't any sample application available in the assignments for this substance. Please check that the peaks were correctly detected and assigned for this substance.
⚠	THCA-A	There wasn't any sample application available in the assignments for this substance. Please check that the peaks were correctly detected and assigned for this substance.
⚠	9-THC	There wasn't any sample application available in the assignments for this substance. Please check that the peaks were correctly detected and assigned for this substance.
⚠	CBGA	There wasn't any sample application available in the assignments for this substance. Please check that the peaks were correctly detected and assigned for this substance.
⚠	CBC	There wasn't any sample application available in the assignments for this substance. Please check that the peaks were correctly detected and assigned for this substance.
⚠	CBDA	There wasn't any sample application available in the assignments for this substance. Please check that the peaks were correctly detected and assigned for this substance.
⚠	THCV	There wasn't any sample application available in the assignments for this substance. Please check that the peaks were correctly detected and assigned for this substance.
⚠	CBDV	There wasn't any sample application available in the assignments for this substance. Please check that the peaks were correctly detected and assigned for this substance.
⚠	CBN	There wasn't any sample application available in the assignments for this substance. Please check that the peaks were correctly detected and assigned for this substance.
⚠	CBG	There wasn't any sample application available in the assignments for this substance. Please check that the peaks were correctly detected and assigned for this substance.

A track marked with ⚠ means: this result is outside the regression range given by the reference assignments, but is included in the results because it is in the allowed range deviation.

Analyst:	Reviewer:
