# Supplementary material for: High Performance Thin-Layer Chromatography (HPTLC) data of Cannabinoids in ten mobile phase systems
Source: Data Brief. 2020 Jun 30;31:105955. doi: 10.1016/j.dib.2020.105955 (PMC7352075; doi:10.1016/j.dib.2020.105955)
Supplement: Supplementary file 1 [file mmc1.zip › S1-Triplicate reports/MGW-2.pdf]

Analysis: MGW-2-R

Path: Home/YL Research

Based on method: Triplets Method

Created	01-Jul-2019 13:20:13	visionCATSuser
Modified	01-Jul-2019 15:14:54	visionCATSuser
Last HPTLC log	01-Jul-2019 15:14:54	Analysis modified
Explorer notes		

Track	Vial ID	Description	Volume	Position	Type
1	MeOH blank	200ng	2.0 µl	A1	Sample
2	Mixture 100	200ng	2.0 µl	A2	Sample
3	9-THC 100	200ng	2.0 µl	A3	Reference
4	CBD 100	200ng	2.0 µl	A4	Reference
5	CBN 100	200ng	2.0 µl	A5	Reference
6	CBG 100	200ng	2.0 µl	A6	Reference
7	CBC 100	200ng	2.0 µl	A7	Reference
8	THCV 100	200ng	2.0 µl	A8	Reference
9	CBDV 100	200ng	2.0 µl	A9	Reference
10	8-THC 100	200ng	2.0 µl	A10	Reference
11	THCA-A 100	200ng	2.0 µl	A11	Reference
12	CBDA 100	200ng	2.0 µl	B1	Reference
13	CBGA 100	200ng	2.0 µl	B2	Reference
Sequence table notes					

A track marked with ⚠ means: the application type is overridden in some evaluation(s).

System setup:

Software	Server User-PC, version 2.5.18072.1
ATS4	S/N:080713
Chamber	N/A
Derivatization dip	N/A
Scanner3	S/N:031025
Visualizer	S/N:230515

Chromatography

Plate layout:

Stationary phase	Merck, HPTLC plates RP-18 F 254s
Plate format	100.0 x 100.0 mm
Application type	Spot
Application	Position Y: 8.0 mm, length: 0.0 mm, width: 0.0 mm
Track	First position X: 20.0 mm, distance: 5.0 mm
Solvent front position	70.0 mm
Notes	

Take image clean plate 1a - Visualizer (S/N: 230515):

Quality	Enhanced
RT White	auto capture, Auto, level 85 %, Band
R 254	auto capture, Auto, level 85 %, Band
Instrument diagnostics	Valid diagnostics
Documentation step label	
Notes	

Application 1 - ATS 4 (S/N: 080713):

Spray gas	NI
Sample solvent type	Methanol
Filling speed	15 µl/s
Predosage volume	200 nl
Retraction volume	200 nl
Dosage speed	150 nl/s
Filling quality	User
Rinsing cycles / vacuum	1 / 4 s
Filling cycles / vacuum	1 / 4 s
Rinsing solvent name	Methanol
Nozzle temperature	Unheated
Rack in use	Standard
Instrument diagnostics	Valid diagnostics
Notes	

Development 1 - Chamber:

Tank	TTC 20x10
Mobile phase	Methanol, 0.1% acetic acid in water (75:25)
Saturation time	20 min
Use saturation pad	true
Use smartALERT	true
Volume front through	10 ml
Volume rear through	25 ml
Drying time	5 min
Drying temperature	Room temperature
Notes	

Take image developed plate 1a - Visualizer (S/N: 230515):

Quality	Enhanced
RT White	auto capture, Auto, level 85 %, Band
R 254	auto capture, Auto, level 85 %, Band
R 366	auto capture, Auto, level 85 %, Band
Instrument diagnostics	Valid diagnostics
Documentation step label	
Notes	

Scan developed plate 1b - Scanner 3 (S/N: 031025):

Scanner type	Single λ
Optimization for	Resolution
Measurement mode	Absorption
Filter	n/a
Detector mode	Automatic
Scanning speed	20 mm/s
Data resolution	100 µm/step
Slit	5 x 0.2 mm, micro
Partial scan	No
Lamp	Deuterium & Tungsten
Wavelength(s)	254 nm
Instrument diagnostics	Valid diagnostics
Documentation step label	
Notes	

Derivatization 1 - dip:

Reagent name	Fast Blue B salt
Dipping speed	3
Dipping time	5 s
Reagent preparation	1g Fast Blue B salt in 200mL water
Heating	none
Notes	Air dry for 5 minutes

Take image derivatized plate 1a - Visualizer (S/N: 230515):

Quality	Enhanced
RT White	auto capture, Auto, level 85 %, Band
R 366	auto capture, Auto, level 85 %, Band
Instrument diagnostics	Valid diagnostics
Documentation step label	
Notes	

System suitability tests:

SST settings:

SST tracks	

Data acquisition

Application 1 - ATS 4 (S/N: 080713):

Executed	01-Jul-2019 13:24:49 visionCATSuser

Development 1 - Chamber:

Executed	01-Jul-2019 14:58:47 visionCATSuser

Take image developed plate 1a - Visualizer (S/N: 230515):

Executed	01-Jul-2019 14:59:32 visionCATSuser

RT White

Developed, RemTransVis

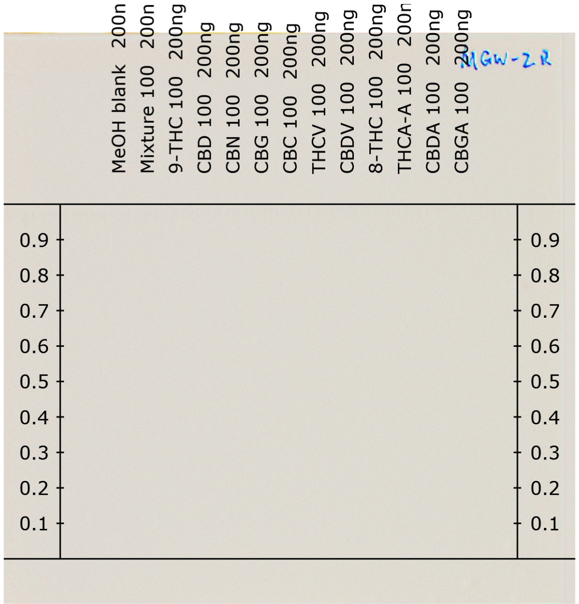

Exposure	0.051 s
Contrast	1
Normalized exposure	Disabled
Clarify	Disabled
White balance	1.00, 1.00, 1.00

R 254

Developed, Remission254

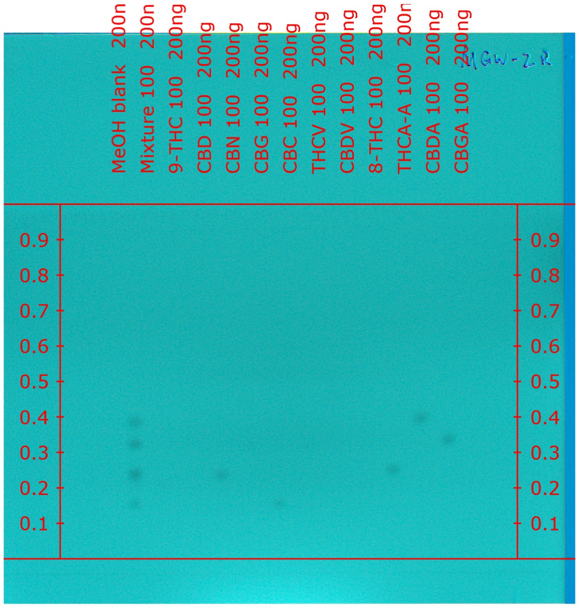

Exposure	0.220 s
Contrast	1
Normalized exposure	Disabled
Clarify	Disabled
White balance	1.00, 1.00, 1.00

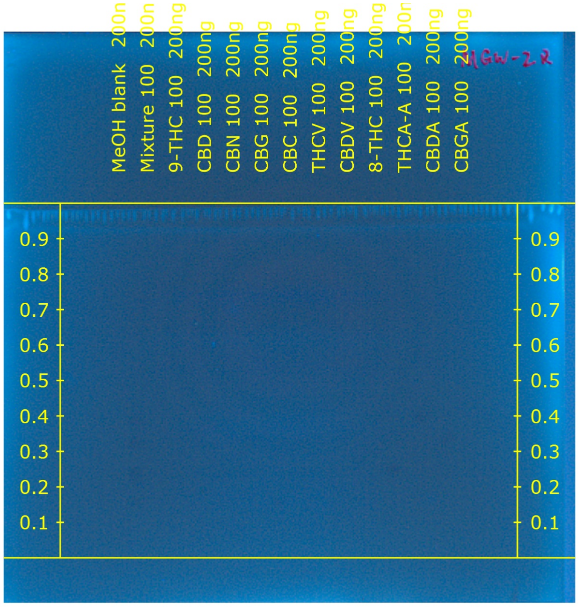

Exposure	7.105 s
Contrast	1
Normalized exposure	Disabled
Clarify	Disabled
White balance	1.00, 1.00, 1.00

Scan developed plate 1b - Scanner 3 (S/N: 031025):

Executed	01-Jul-2019 15:01:01 visionCATSuser

Scan:

Wavelength	254 nm

Track 1:

Type	Single λ

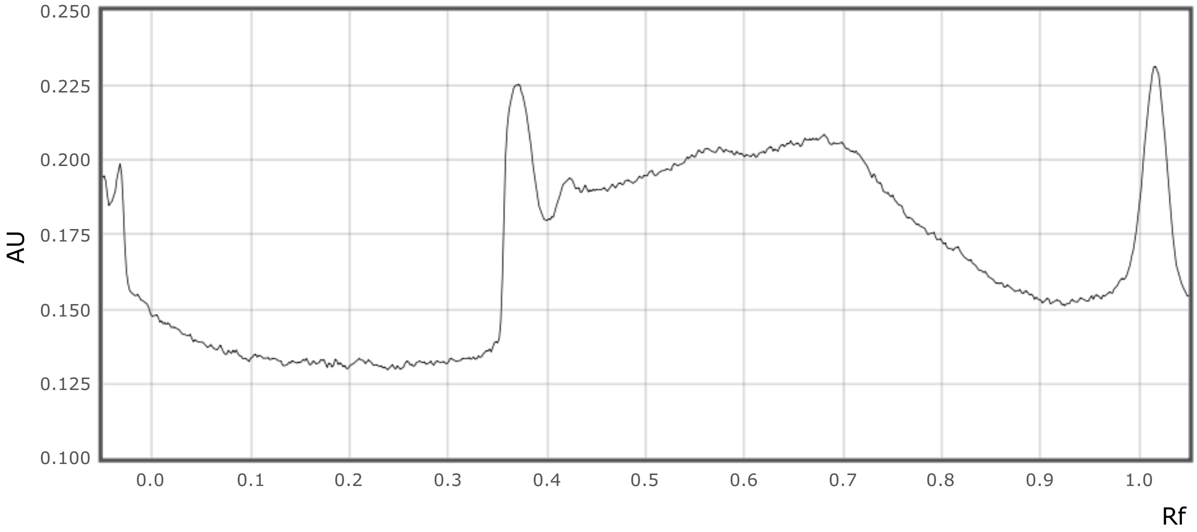

Track 2:

Type	Single λ

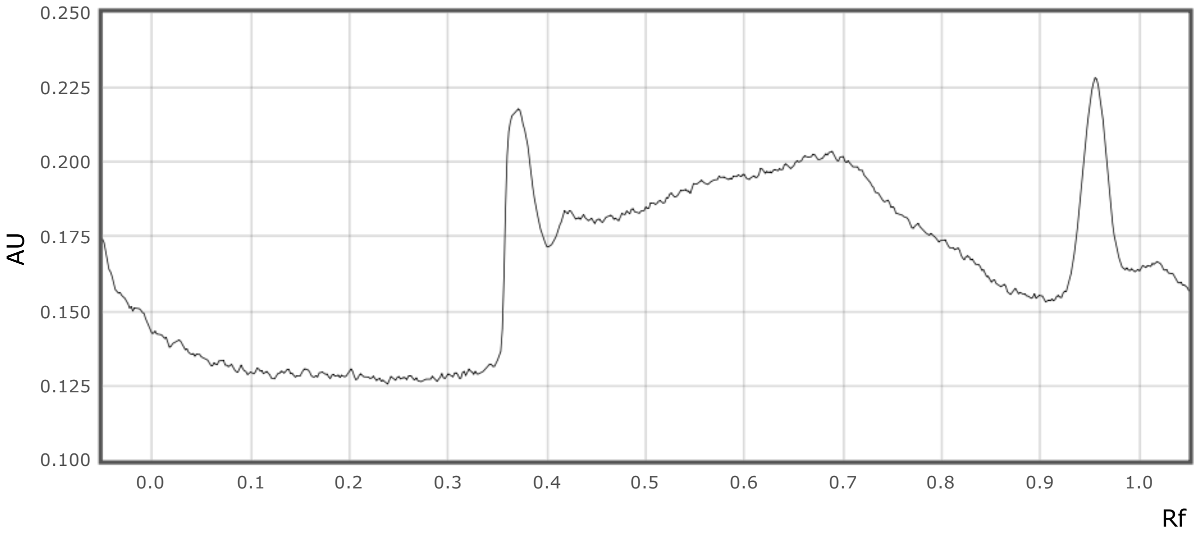

Track 3:

Type	Single λ

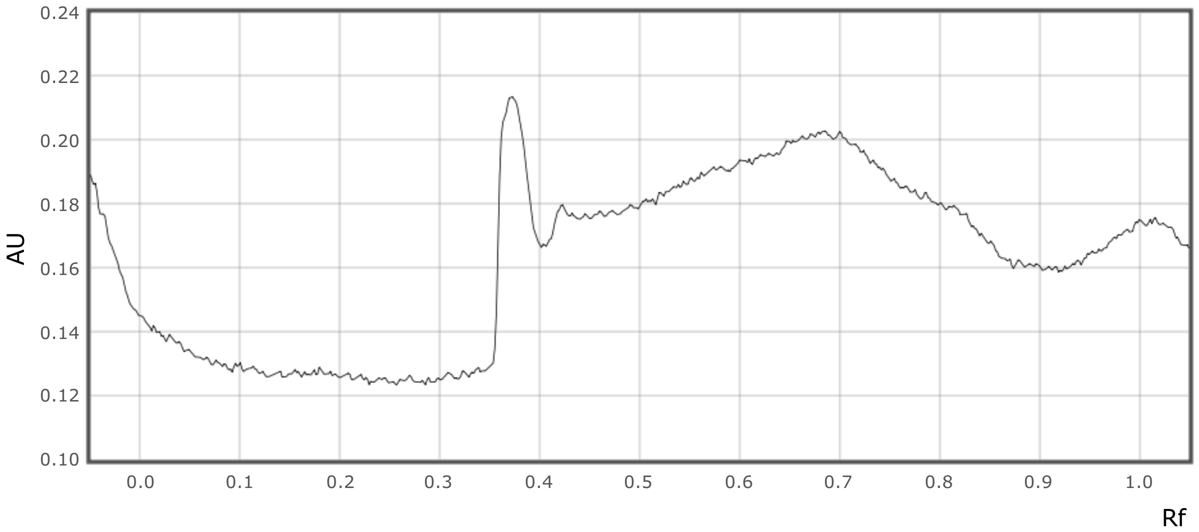

Track 4:

Type	Single λ

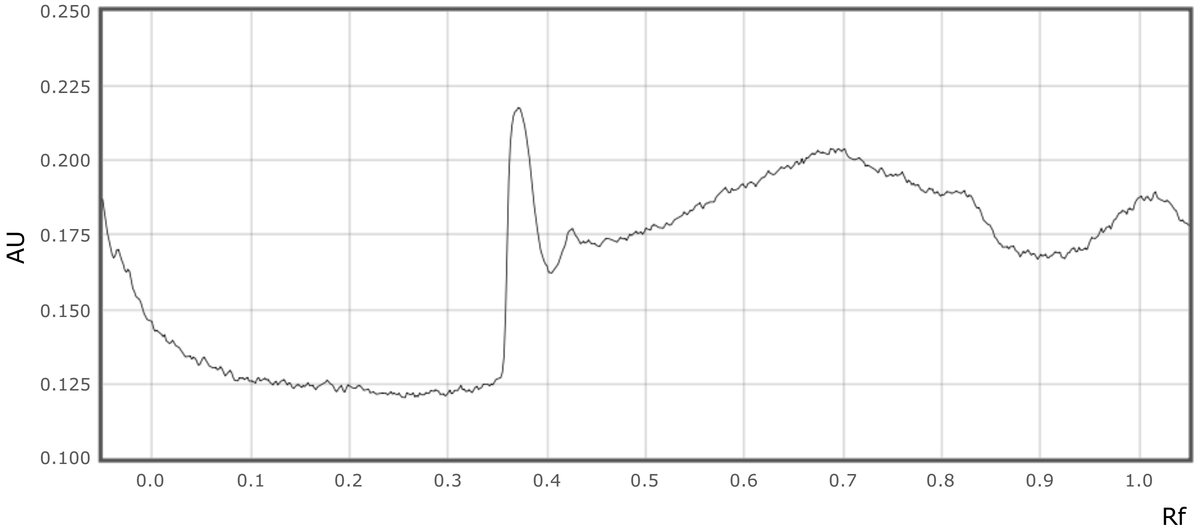

Track 5:	
Type	Single λ

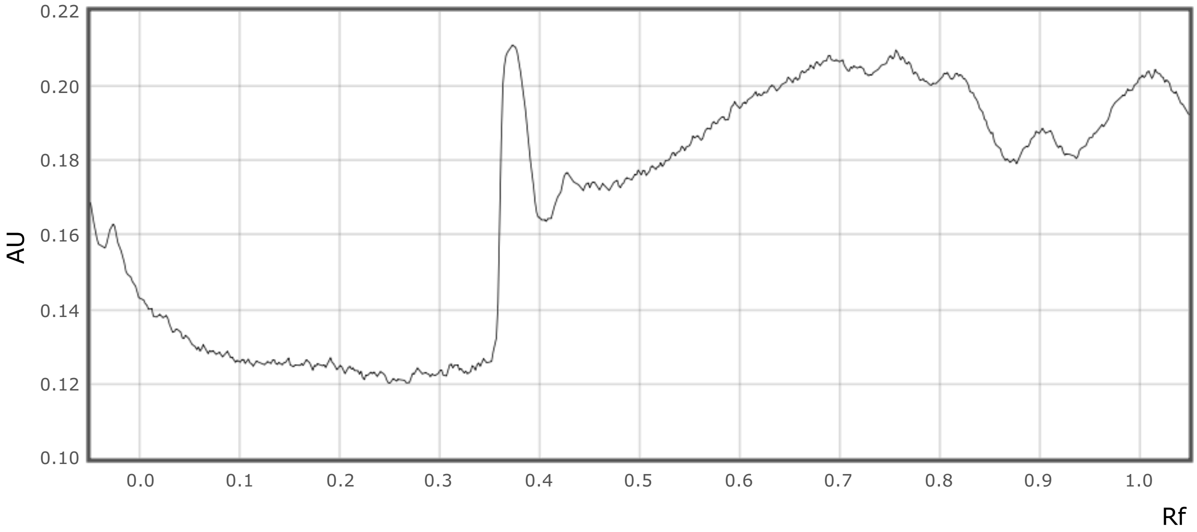

Track 6:	
Type	Single λ

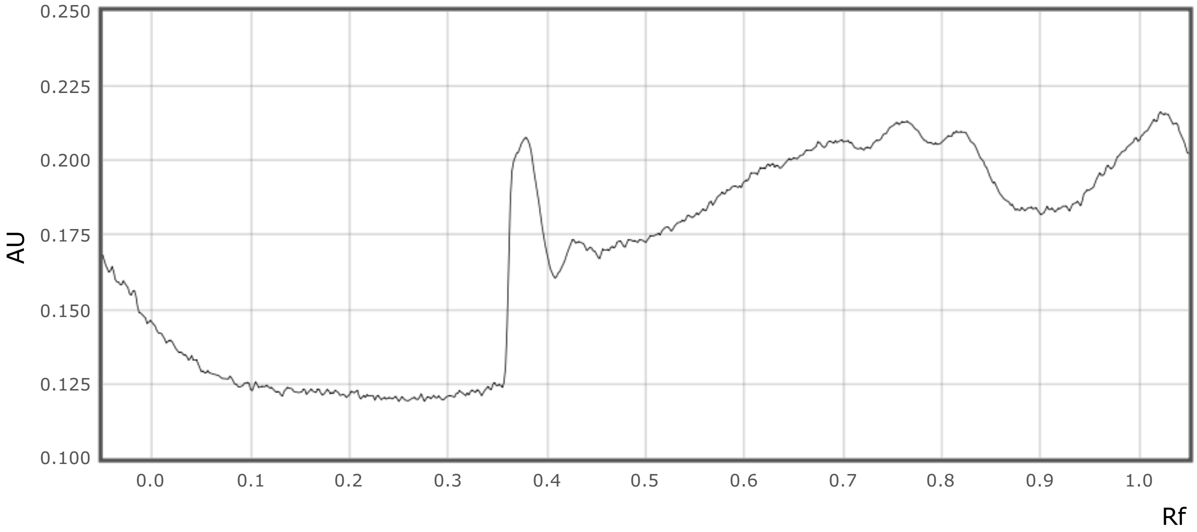

Track 7:	
Type	Single λ

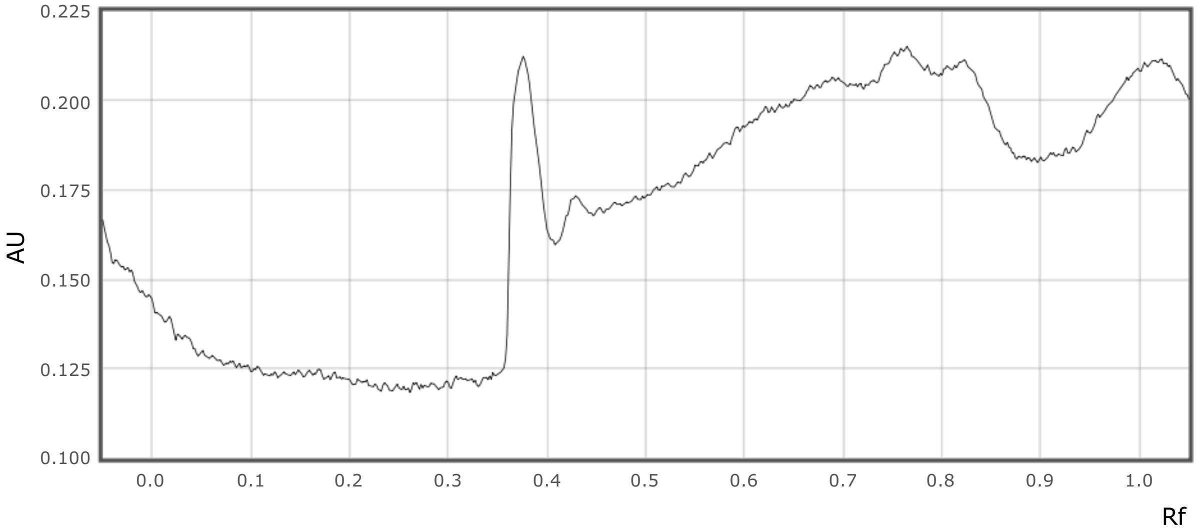

Track 8:	
Type	Single λ

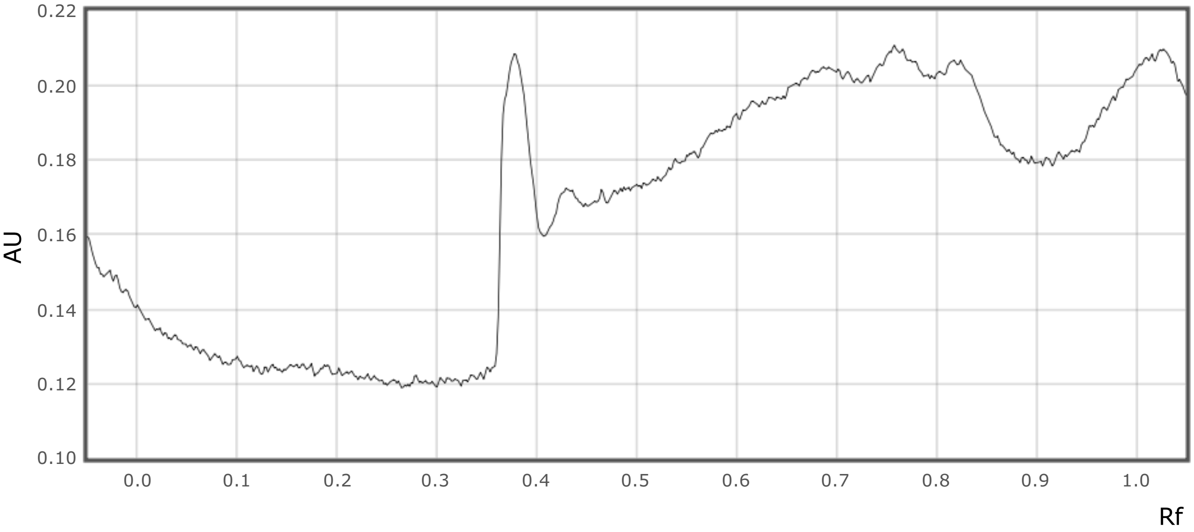

Track 9:

Type	Single λ

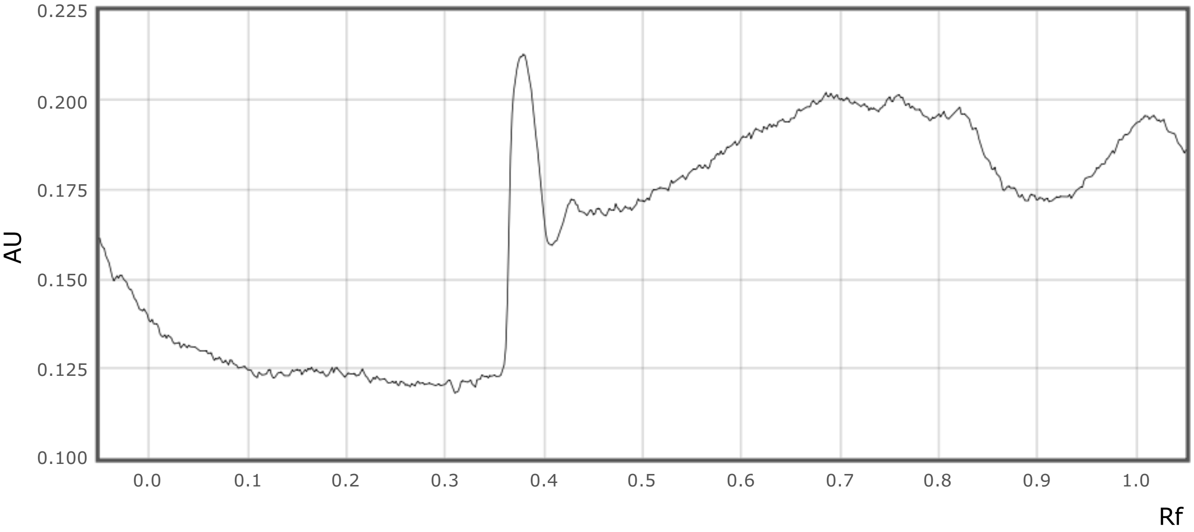

Track 10:

Type	Single λ

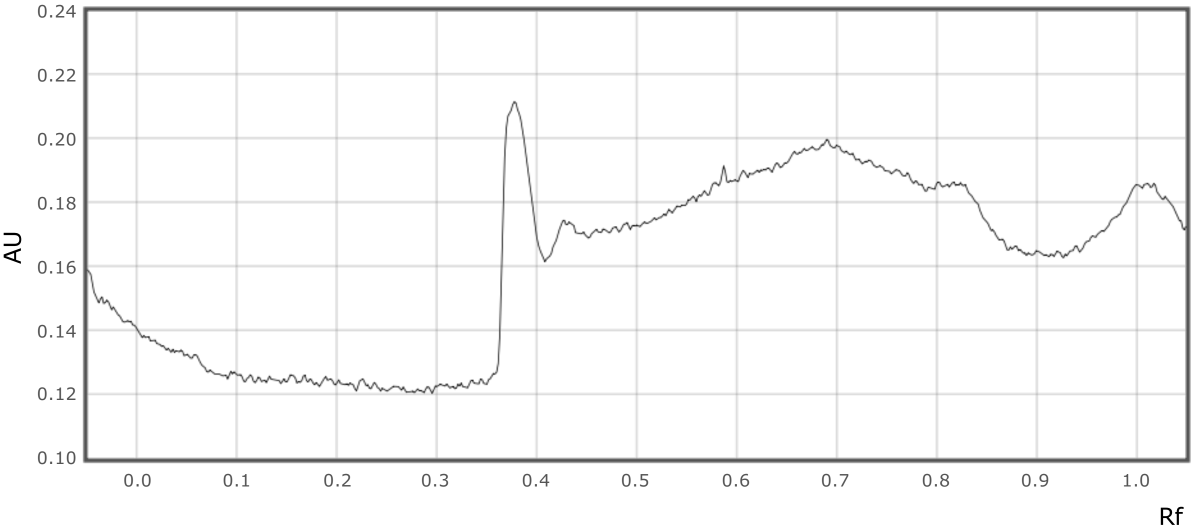

Track 11:	
Type	Single λ

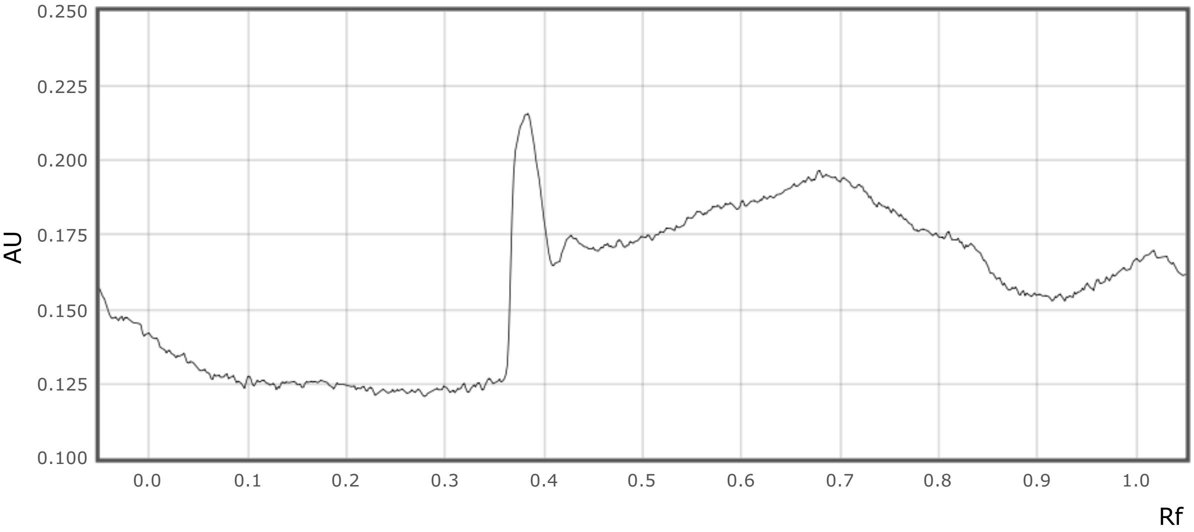

Track 12:	
Type	Single λ

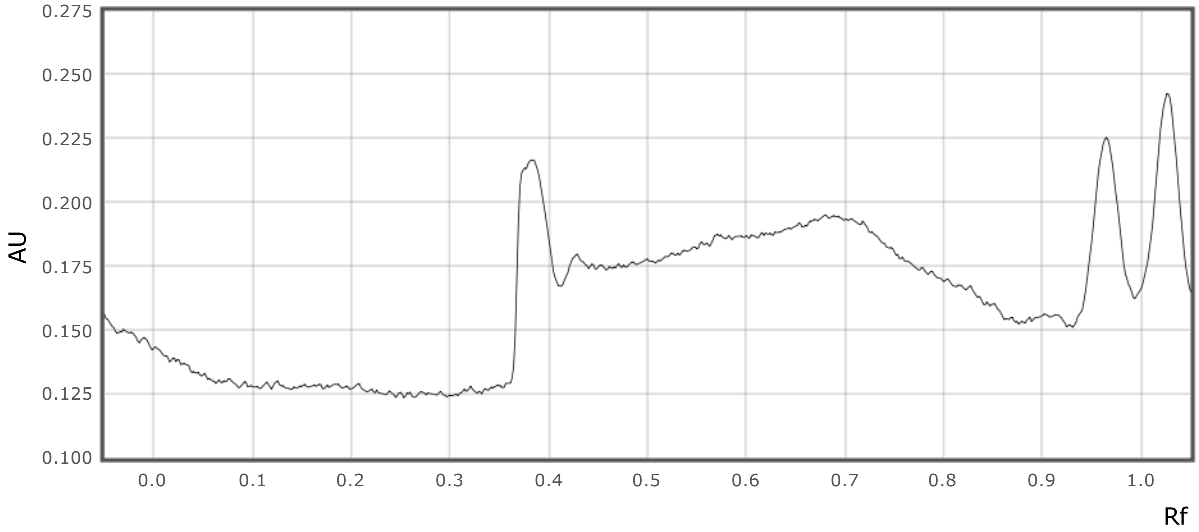

Track 13:	
Type	Single λ

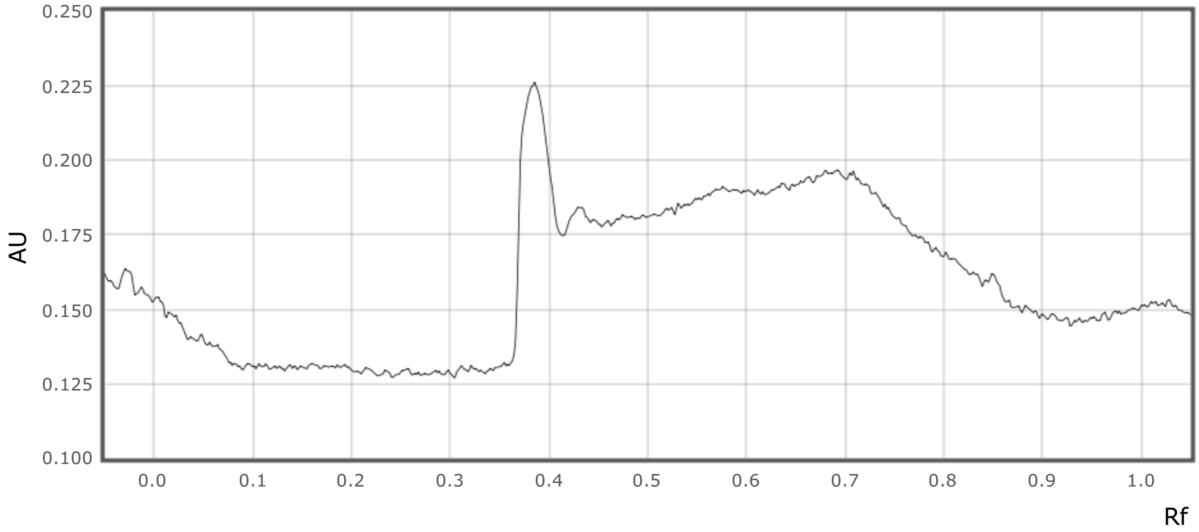

Derivatization 1 - dip:

Executed	01-Jul-2019 15:05:20 visionCATSuser

Take image derivatized plate 1a - Visualizer (S/N: 230515):

Executed	01-Jul-2019 15:09:03 visionCATSuser

RT White

Derivatized, RemTransVis

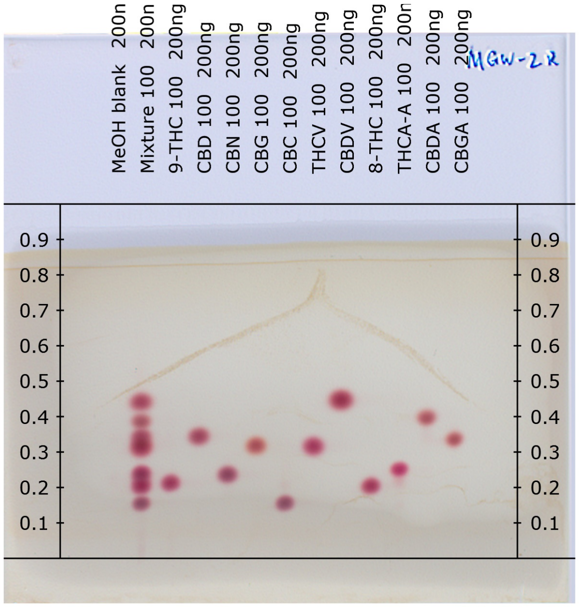

Exposure	0.055 s
Contrast	1
Normalized exposure	Disabled
Clarify	Disabled
White balance	1.14, 1.07, 0.85

R 366

Derivatized, Remission366

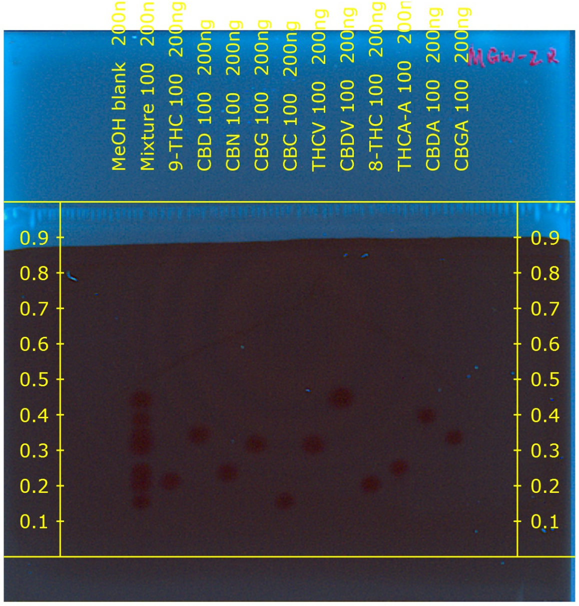

Exposure	8.206 s
Contrast	1
Normalized exposure	Disabled
Clarify	Disabled
White balance	1.00, 1.00, 1.00

Evaluation 1 :

Validated	false
Step	Take image derivatized plate 1a
Concentration unit type	Mass / volume
Notes	

Definition:

References:

9-THC 100

Substance Name	Concentration	Purity
9-THC	100.000 µg/ml	100.00 %

CBD 100

Substance Name	Concentration	Purity
CBD	100.000 µg/ml	100.00 %

CBN 100

Substance Name	Concentration	Purity
CBN	100.000 µg/ml	100.00 %

CBG 100

Substance Name	Concentration	Purity
CBG	100.000 µg/ml	100.00 %

CBC 100

Substance Name	Concentration	Purity
CBC	100.000 µg/ml	100.00 %

THCV 100

Substance Name	Concentration	Purity
THCV	100.000 µg/ml	100.00 %

CBDV 100

Substance Name	Concentration	Purity
CBDV	100.000 µg/ml	100.00 %

8-THC 100

Substance Name	Concentration	Purity
8-THC	100.000 µg/ml	100.00 %

THCA-A 100

Substance Name	Concentration	Purity
THCA-A	100.000 µg/ml	100.00 %

CBDA 100

Substance Name	Concentration	Purity
CBDA	100.000 µg/ml	100.00 %

CBGA 100

Substance Name	Concentration	Purity
CBGA	100.000 µg/ml	100.00 %

Samples:

Vial ID	Amount	Volume solution	Reference amount	Related to
MeOH blank		0.00 ml		
Mixture 100		0.00 ml		

Integration parameters:

Bounds	[0.000,1.000]
Smoothing	Savitzky–Golay of order 3 and window 7
Baseline correction	Lowest slope with noise 0.05
Profile subtraction	Profile subtraction from track 1
Peaks detection	Gauss (legacy) with sensitivity 0.1, separation 1 and threshold 0.05

Scan:

Wavelength	RT White

Track 1:

Type	Sample
Vial ID	MeOH blank
Description	200ng
Volume	2.0 µl

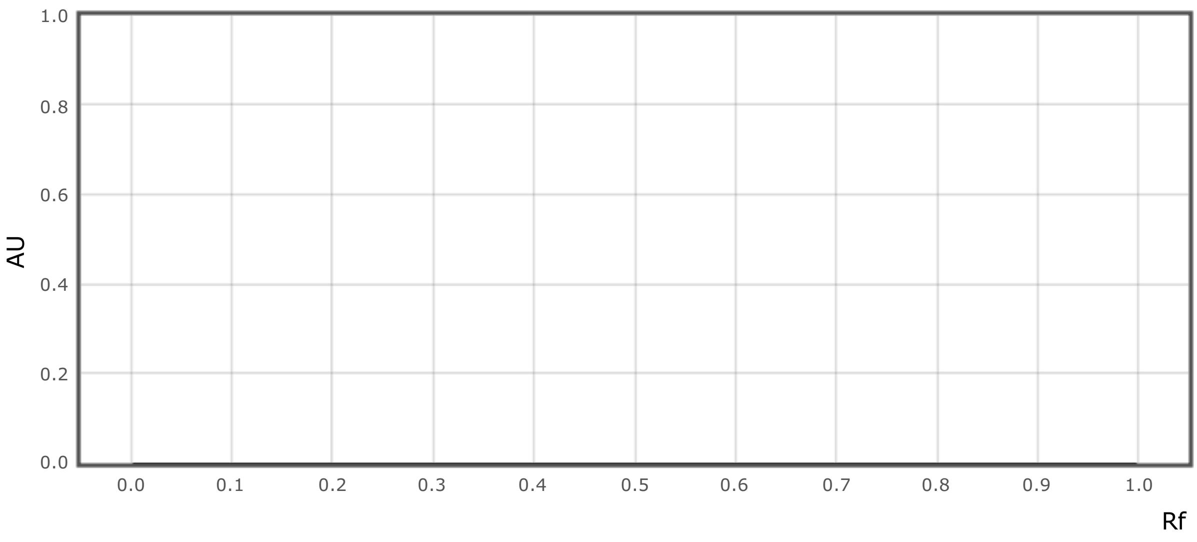

Peak #	Start		Max			End		Area		Manual peak	Substance Name
	Rf	H	Rf	H	%	Rf	H	A	%		

Track 2:

Type	Sample
Vial ID	Mixture 100
Description	200ng
Volume	2.0 µl

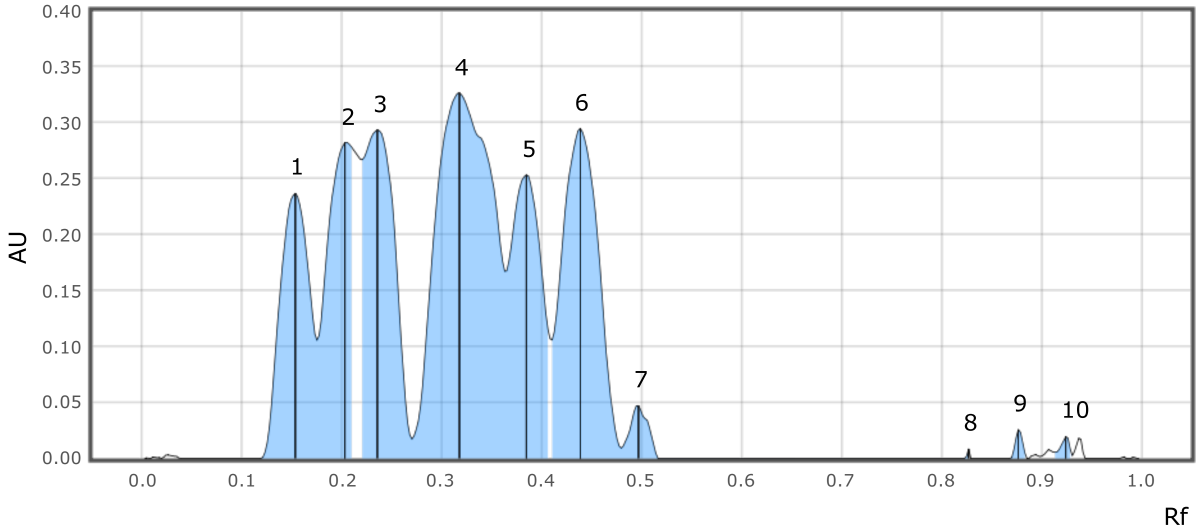

Peak #	Start		Max			End		Area		Manual peak	Substance Name
	Rf	H	Rf	H	%	Rf	H	A	%		
1	0.119	0.0000	0.153	0.2369	13.24	0.175	0.1056	0.00803	11.03	No	
2	0.175	0.1056	0.203	0.2824	15.79	0.218	0.2677	0.01004	13.79	No	
3	0.220	0.2670	0.235	0.2936	16.42	0.270	0.0172	0.01035	14.21	No	
4	0.270	0.0172	0.317	0.3267	18.26	0.363	0.1672	0.02142	29.41	No	
5	0.363	0.1672	0.384	0.2533	14.16	0.408	0.1071	0.00917	12.59	No	
6	0.410	0.1058	0.438	0.2950	16.49	0.479	0.0091	0.01240	17.03	No	
7	0.479	0.0091	0.497	0.0471	2.63	0.518	0.0000	0.00099	1.36	No	
8	0.823	0.0000	0.827	0.0084	0.47	0.830	0.0000	0.00002	0.03	No	
9	0.868	0.0000	0.877	0.0258	1.44	0.886	0.0000	0.00021	0.28	No	
10	0.914	0.0055	0.925	0.0194	1.08	0.931	0.0025	0.00020	0.27	No	

Track 3:

Type	Reference
Vial ID	9-THC 100
Description	200ng
Volume	2.0 µl

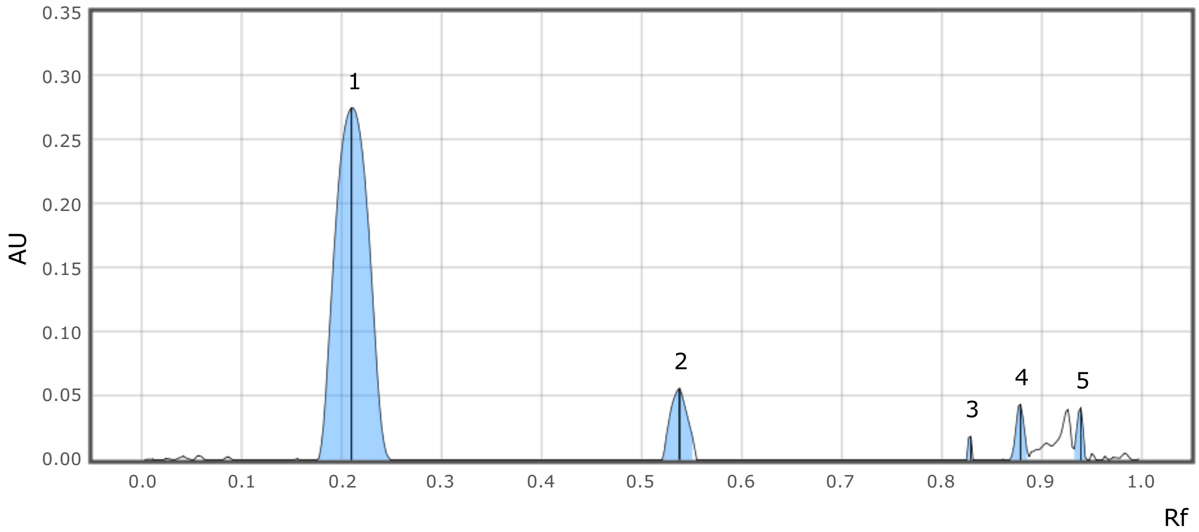

Peak #	Start		Max			End		Area		Manual peak	Substance Name
	Rf	H	Rf	H	%	Rf	H	A	%		
1	0.175	0.0000	0.209	0.2752	63.46	0.248	0.0000	0.01075	85.00	No	9-THC
2	0.521	0.0000	0.538	0.0558	12.87	0.555	0.0000	0.00112	8.90	No	
3	0.825	0.0000	0.830	0.0184	4.25	0.832	0.0000	0.00008	0.61	No	
4	0.868	0.0000	0.879	0.0435	10.03	0.888	0.0025	0.00041	3.24	No	
5	0.933	0.0084	0.940	0.0407	9.39	0.946	0.0000	0.00029	2.26	No	

Track 4:

Type	Reference
Vial ID	CBD 100
Description	200ng
Volume	2.0 µl

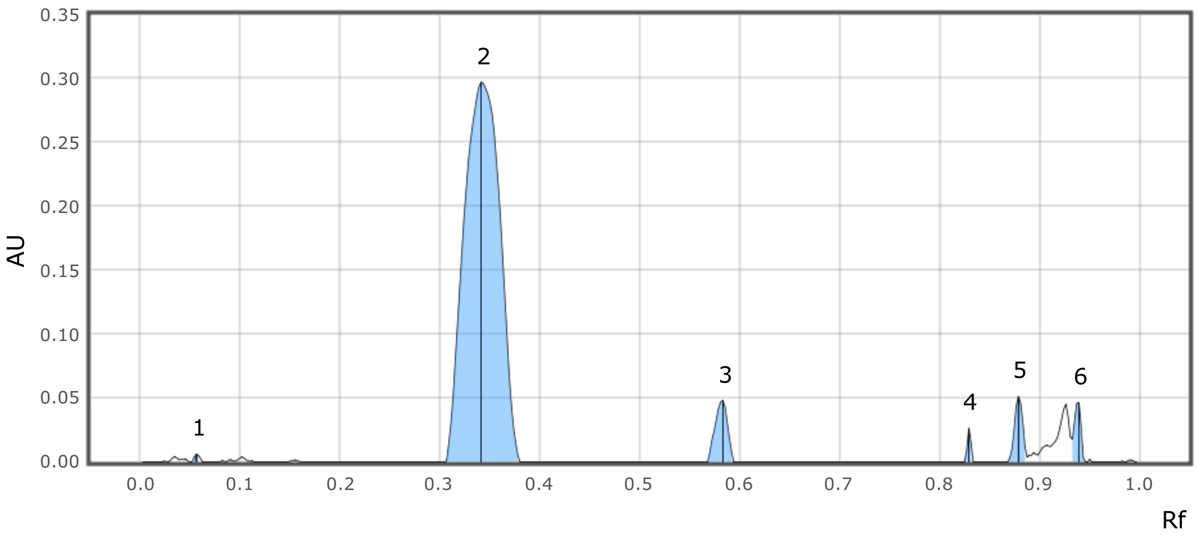

Peak #	Start		Max			End		Area		Manual peak	Substance Name
	Rf	H	Rf	H	%	Rf	H	A	%		
1	0.052	0.0000	0.056	0.0059	1.24	0.062	0.0000	0.00004	0.26	No	
2	0.304	0.0000	0.341	0.2971	62.51	0.380	0.0000	0.01240	88.09	No	CBD
3	0.568	0.0000	0.583	0.0482	10.13	0.594	0.0000	0.00069	4.93	No	
4	0.825	0.0000	0.830	0.0265	5.58	0.834	0.0000	0.00011	0.77	No	
5	0.868	0.0000	0.879	0.0511	10.76	0.888	0.0038	0.00048	3.44	No	
6	0.933	0.0176	0.940	0.0465	9.79	0.946	0.0000	0.00035	2.50	No	

Track 5:

Type	Reference
Vial ID	CBN 100
Description	200ng
Volume	2.0 µl

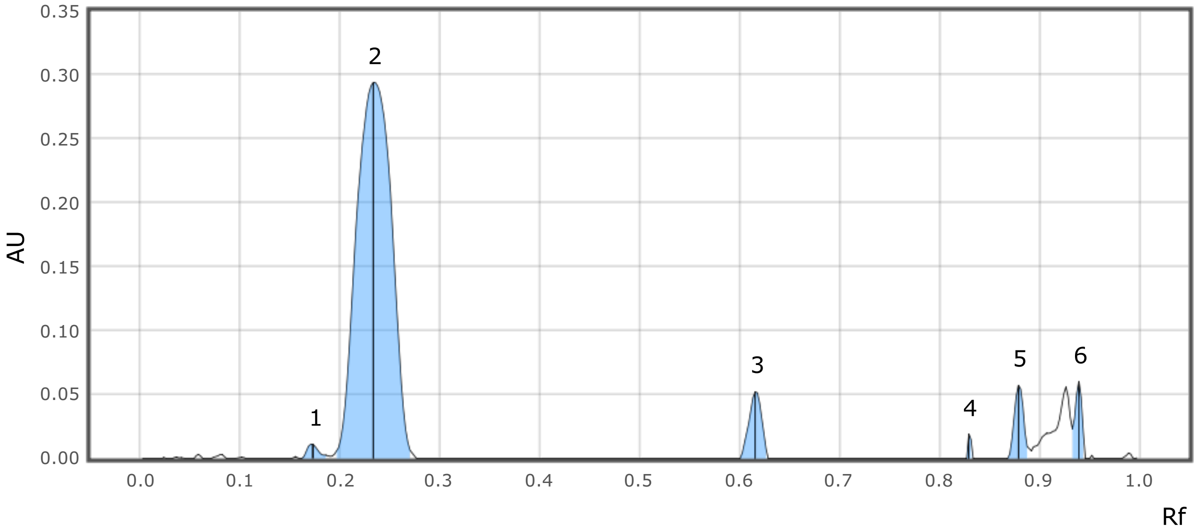

Peak #	Start		Max			End		Area		Manual peak	Substance Name
	Rf	H	Rf	H	%	Rf	H	A	%		
1	0.160	0.0000	0.173	0.0110	2.24	0.190	0.0016	0.00015	1.09	No	
2	0.196	0.0057	0.233	0.2939	59.57	0.273	0.0044	0.01198	85.04	Yes	CBN
3	0.600	0.0000	0.616	0.0520	10.54	0.629	0.0000	0.00077	5.46	No	
4	0.827	0.0000	0.830	0.0191	3.87	0.834	0.0000	0.00007	0.52	No	
5	0.868	0.0000	0.879	0.0571	11.57	0.892	0.0059	0.00063	4.47	No	
6	0.933	0.0230	0.940	0.0603	12.21	0.946	0.0000	0.00048	3.41	No	

Track 6:

Type	Reference
Vial ID	CBG 100
Description	200ng
Volume	2.0 µl

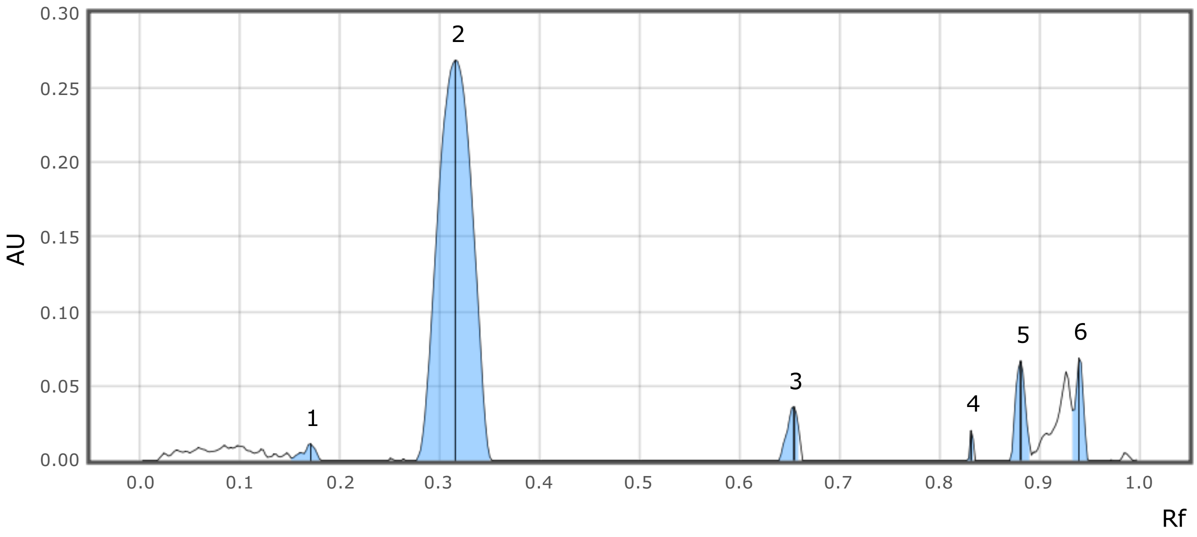

Peak #	Start		Max			End		Area		Manual peak	Substance Name
	Rf	H	Rf	H	%	Rf	H	A	%		
1	0.151	0.0015	0.170	0.0110	2.35	0.181	0.0000	0.00016	1.28	No	
2	0.276	0.0000	0.315	0.2684	57.03	0.352	0.0000	0.01073	83.62	No	CBG
3	0.639	0.0000	0.655	0.0359	7.62	0.663	0.0000	0.00047	3.64	No	
4	0.827	0.0000	0.832	0.0201	4.27	0.836	0.0000	0.00008	0.60	No	
5	0.871	0.0000	0.881	0.0667	14.18	0.892	0.0037	0.00076	5.89	No	
6	0.933	0.0335	0.940	0.0685	14.56	0.951	0.0000	0.00064	4.97	No	

Track 7:

Type	Reference
Vial ID	CBC 100
Description	200ng
Volume	2.0 µl

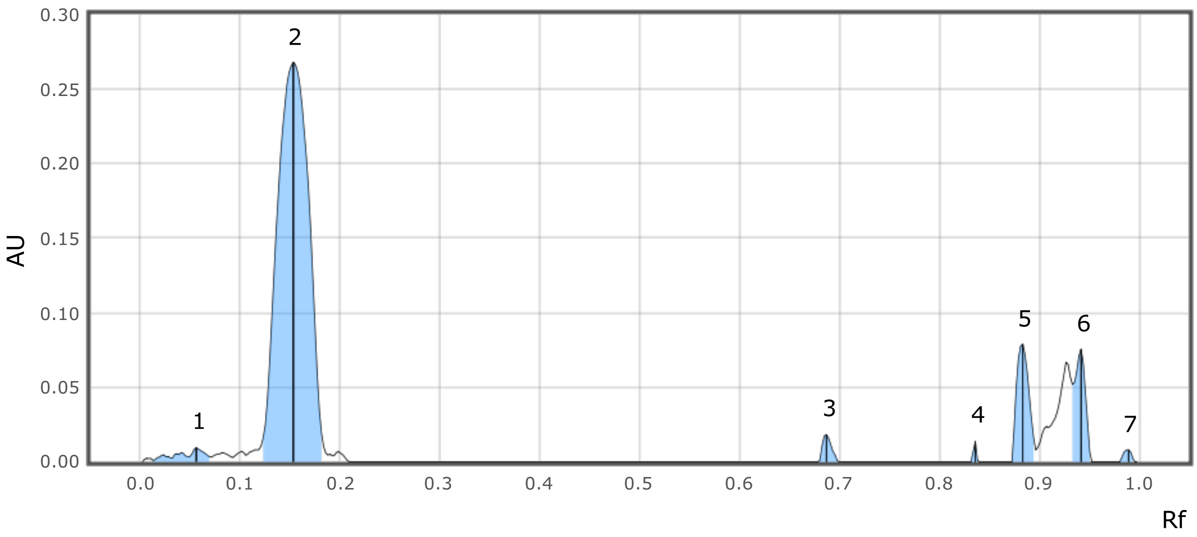

Peak #	Start		Max			End		Area		Manual peak	Substance Name
	Rf	H	Rf	H	%	Rf	H	A	%		
1	0.013	0.0009	0.056	0.0094	1.99	0.071	0.0032	0.00028	2.22	No	
2	0.122	0.0105	0.153	0.2676	56.82	0.183	0.0104	0.00978	78.66	Yes	CBC
3	0.678	0.0000	0.687	0.0183	3.89	0.700	0.0000	0.00019	1.51	No	
4	0.832	0.0000	0.836	0.0135	2.87	0.840	0.0000	0.00005	0.38	No	
5	0.873	0.0000	0.884	0.0787	16.72	0.897	0.0080	0.00113	9.12	No	
6	0.933	0.0518	0.942	0.0754	16.00	0.953	0.0000	0.00093	7.52	No	
7	0.979	0.0000	0.989	0.0081	1.71	0.996	0.0000	0.00007	0.60	No	

Track 8:

Type	Reference
Vial ID	THCV 100
Description	200ng
Volume	2.0 µl

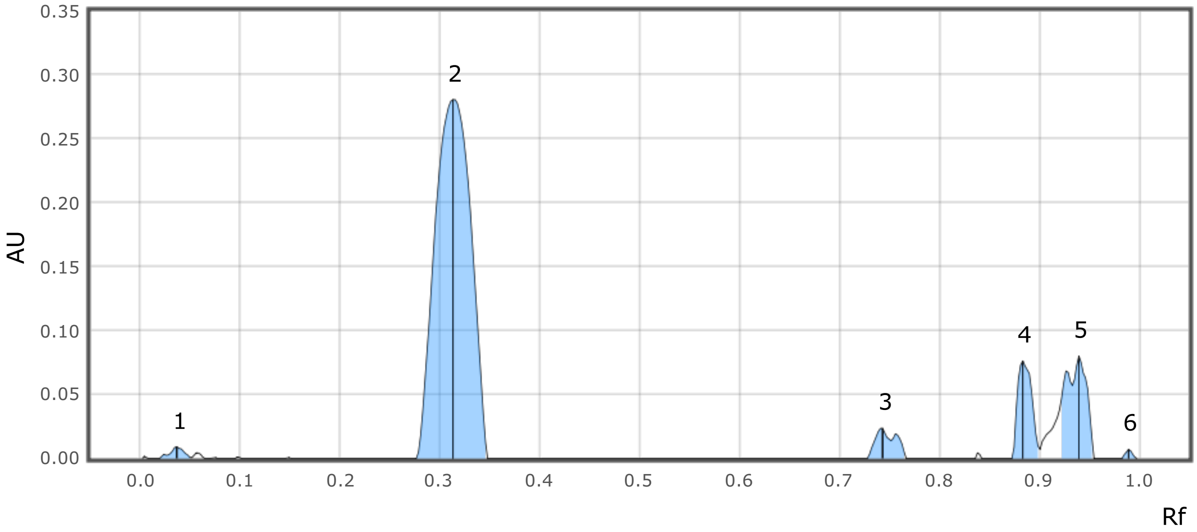

Peak #	Start		Max			End		Area		Manual peak	Substance Name
	Rf	H	Rf	H	%	Rf	H	A	%		
1	0.019	0.0000	0.036	0.0090	1.88	0.052	0.0007	0.00014	0.90	No	
2	0.276	0.0000	0.313	0.2806	58.90	0.348	0.0000	0.01179	75.22	No	THCV
3	0.728	0.0000	0.743	0.0236	4.95	0.767	0.0000	0.00056	3.56	No	
4	0.873	0.0000	0.884	0.0763	16.01	0.901	0.0071	0.00127	8.10	No	
5	0.922	0.0471	0.940	0.0799	16.78	0.955	0.0000	0.00186	11.89	No	
6	0.983	0.0000	0.989	0.0070	1.47	0.998	0.0000	0.00005	0.33	No	

Track 9:

Type	Reference
Vial ID	CBDV 100
Description	200ng
Volume	2.0 µl

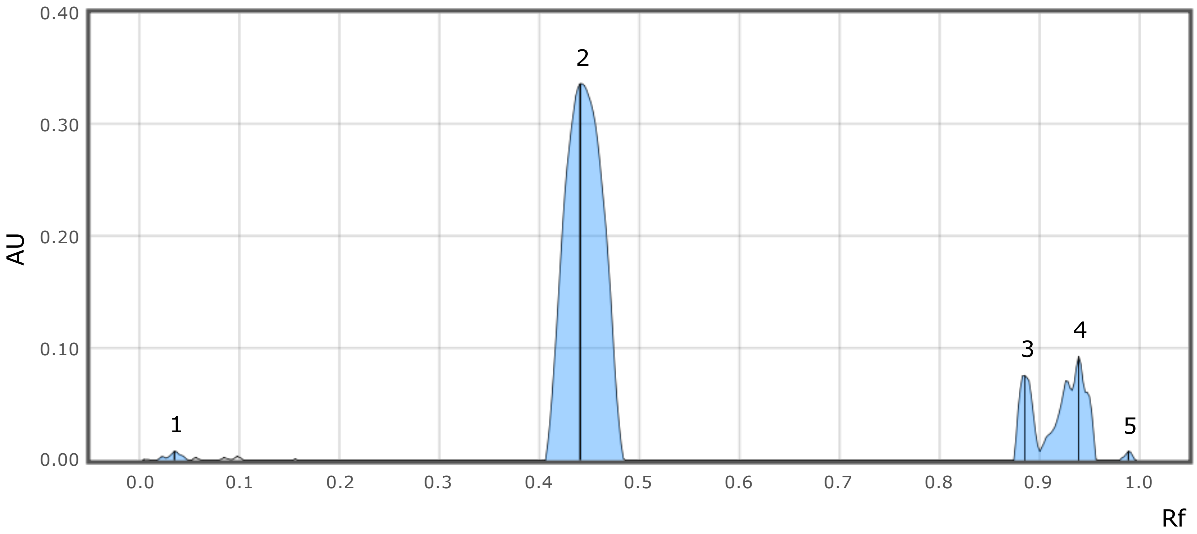

Peak #	Start Rf	Start H	Max Rf	Max H	Max %	End Rf	End H	Area A	Area %	Manual peak	Substance Name
1	0.017	0.0000	0.034	0.0079	1.51	0.049	0.0000	0.00011	0.56	No	
2	0.406	0.0000	0.441	0.3365	64.64	0.486	0.0000	0.01590	79.44	No	CBDV
3	0.875	0.0000	0.886	0.0758	14.56	0.901	0.0078	0.00122	6.10	No	
4	0.901	0.0078	0.940	0.0926	17.80	0.959	0.0000	0.00272	13.57	No	
5	0.979	0.0000	0.989	0.0078	1.49	0.998	0.0000	0.00006	0.32	No	

Track 10:

Type	Reference
Vial ID	8-THC 100
Description	200ng
Volume	2.0 µl

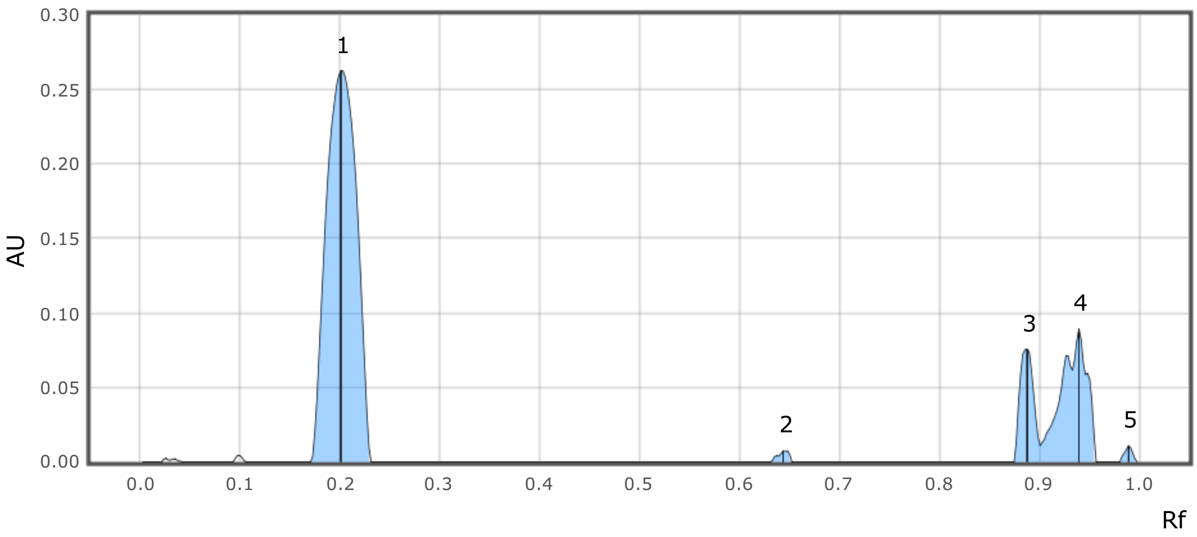

Peak #	Start Rf	Start H	Max Rf	Max H	Max %	End Rf	End H	Area A	Area %	Manual peak	Substance Name
1	0.170	0.0000	0.201	0.2622	58.92	0.231	0.0000	0.00933	69.57	No	8-THC
2	0.631	0.0000	0.644	0.0074	1.65	0.652	0.0000	0.00010	0.72	No	
3	0.875	0.0000	0.888	0.0756	16.99	0.901	0.0108	0.00122	9.13	No	
4	0.901	0.0108	0.940	0.0890	20.01	0.957	0.0000	0.00266	19.84	No	
5	0.981	0.0000	0.989	0.0108	2.43	0.998	0.0000	0.00010	0.74	No	

Track 11:

Type	Reference
Vial ID	THCA-A 100
Description	200ng
Volume	2.0 µl

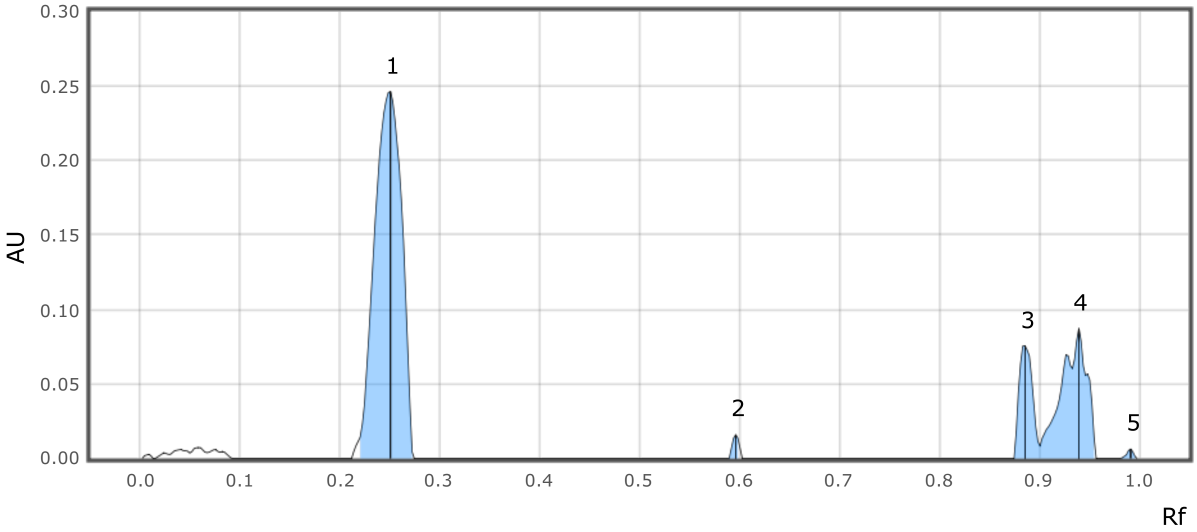

Peak #	Start Rf	Start H	Max Rf	Max H	Max %	End Rf	End H	Area A	Area %	Manual peak	Substance Name
1	0.219	0.0143	0.250	0.2459	57.08	0.274	0.0000	0.00777	66.49	Yes	THCA-A
2	0.588	0.0000	0.596	0.0159	3.69	0.603	0.0000	0.00012	1.05	No	
3	0.875	0.0000	0.886	0.0755	17.54	0.901	0.0081	0.00118	10.11	No	
4	0.901	0.0081	0.940	0.0871	20.22	0.957	0.0000	0.00256	21.95	No	
5	0.981	0.0000	0.992	0.0063	1.47	0.998	0.0000	0.00005	0.39	No	

Track 12:

Type	Reference
Vial ID	CBDA 100
Description	200ng
Volume	2.0 µl

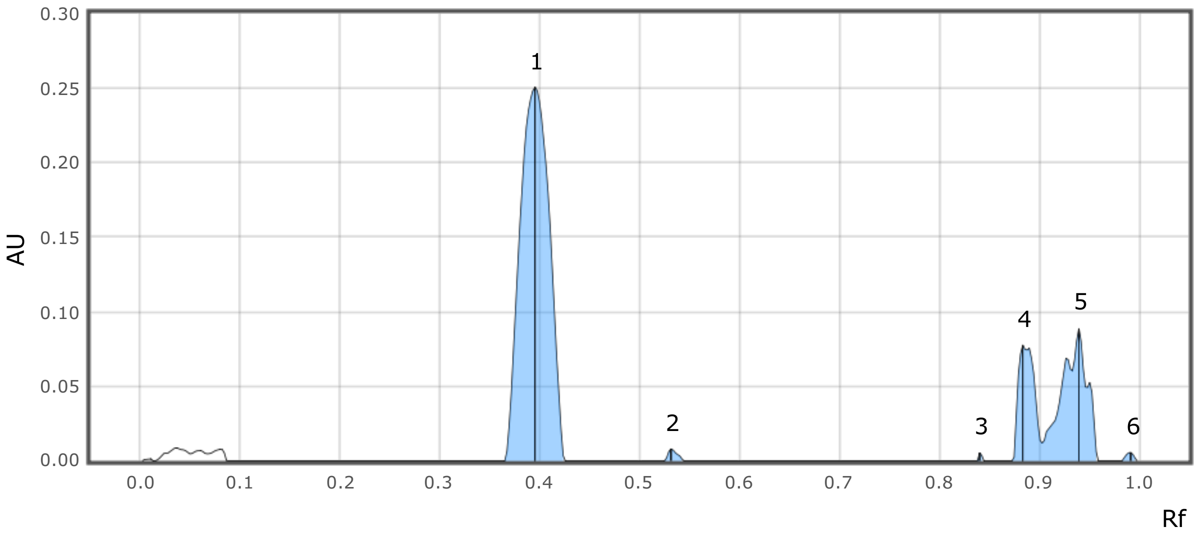

Peak #	Start Rf	Start H	Max Rf	Max H	Max %	End Rf	End H	Area A	Area %	Manual peak	Substance Name
1	0.365	0.0000	0.395	0.2503	57.52	0.425	0.0000	0.00856	67.23	No	CBDA
2	0.525	0.0000	0.531	0.0079	1.82	0.544	0.0000	0.00008	0.64	No	
3	0.838	0.0000	0.840	0.0053	1.22	0.845	0.0000	0.00002	0.15	No	
4	0.873	0.0000	0.884	0.0775	17.81	0.903	0.0119	0.00147	11.54	No	
5	0.903	0.0119	0.940	0.0886	20.35	0.959	0.0000	0.00255	20.05	No	
6	0.983	0.0000	0.992	0.0056	1.28	0.998	0.0000	0.00005	0.39	No	

Track 13:

Type	Reference
Vial ID	CBGA 100
Description	200ng
Volume	2.0 µl

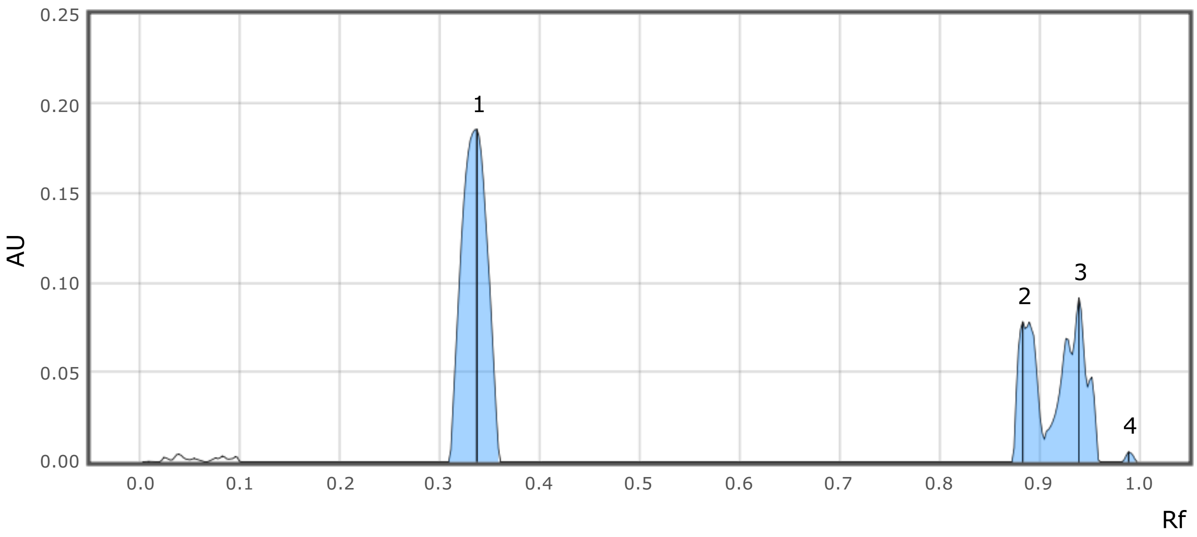

Peak #	Start Rf	Start H	Max Rf	Max H	Max %	End Rf	End H	Area A	Area %	Manual peak	Substance Name
1	0.309	0.0000	0.337	0.1855	51.41	0.361	0.0000	0.00569	57.15	No	CBGA
2	0.873	0.0000	0.884	0.0783	21.68	0.905	0.0125	0.00168	16.87	No	
3	0.905	0.0125	0.940	0.0916	25.38	0.961	0.0000	0.00254	25.54	No	
4	0.983	0.0000	0.989	0.0055	1.53	0.998	0.0000	0.00004	0.44	No	

Calibration results:

Height calibration for substance 8-THC @ RT White:

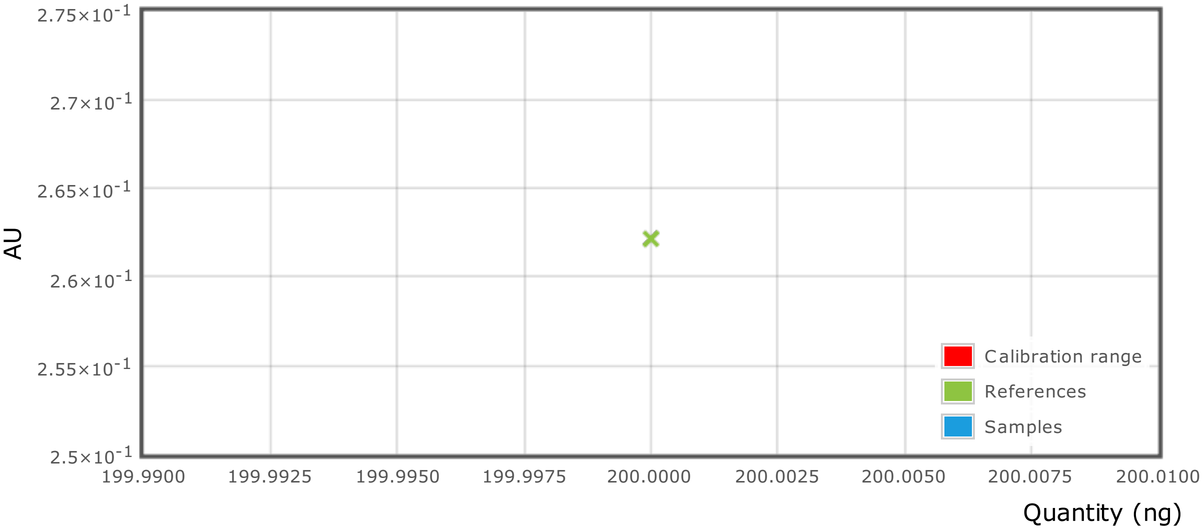

Regression mode	Linear-2
Range deviation	5.00 %
Related substances	Default
Number of references	1
Calibration function	$y=0x$
Coefficient of variation	CV 0.00 %
Correlation coefficient	n/a
⚠	Unable to compute the results for this substance because there wasn't eno ugh groups of references replicas (at least 1 for Linear-1, 2 for Linear2 and Mime-1 and 3 for Polynomial and MiMe-2)

Height calibration for substance 9-THC @ RT White:

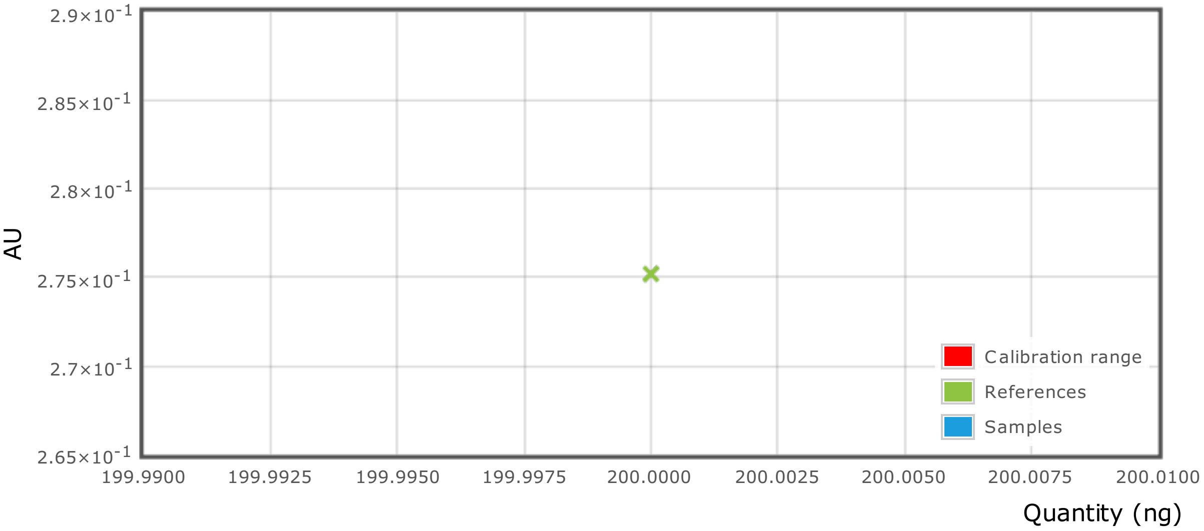

Regression mode	Linear-2
Range deviation	5.00 %
Related substances	Default
Number of references	1
Calibration function	$y=0x$
Coefficient of variation	CV 0.00 %
Correlation coefficient	n/a
⚠	Unable to compute the results for this substance because there wasn't enough groups of references replicas (at least 1 for Linear-1, 2 for Linear2 and Mime-1 and 3 for Polynomial and MiMe-2)

Height calibration for substance CBC @ RT White:

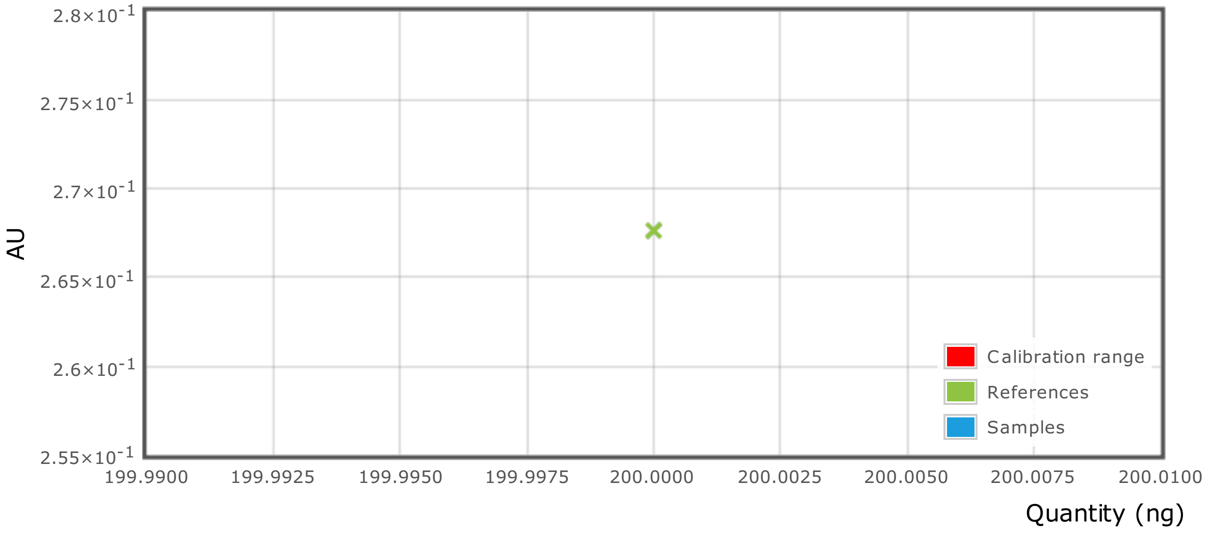

Regression mode	Linear-2
Range deviation	5.00 %
Related substances	Default
Number of references	1
Calibration function	$y=0x$
Coefficient of variation	CV 0.00 %
Correlation coefficient	n/a
⚠	Unable to compute the results for this substance because there wasn't enough groups of references replicas (at least 1 for Linear-1, 2 for Linear2 and Mime-1 and 3 for Polynomial and MiMe-2)

Height calibration for substance CBD @ RT White:

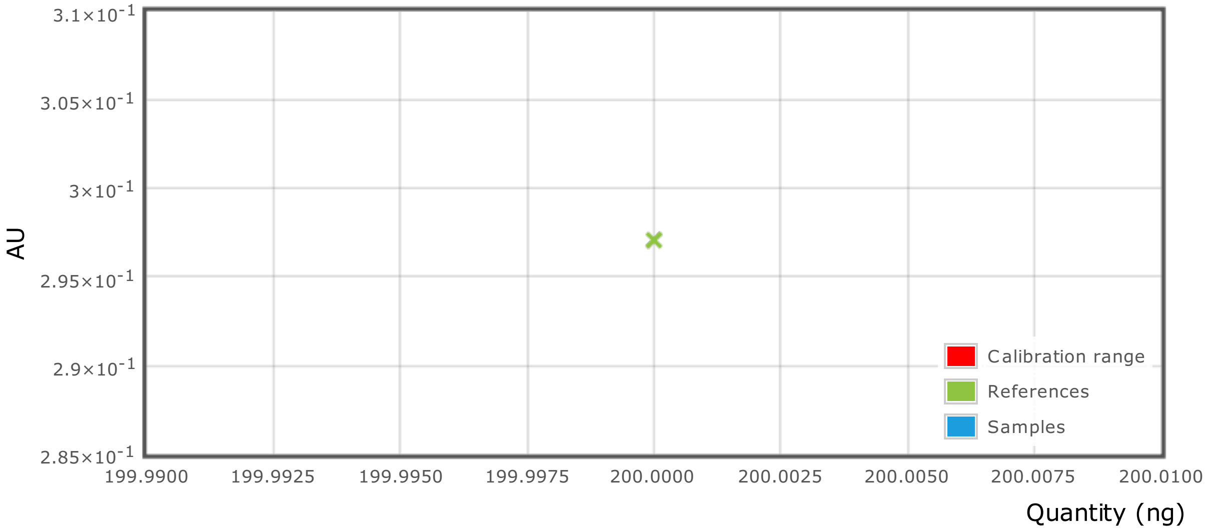

Regression mode	Linear-2
Range deviation	5.00 %
Related substances	Default
Number of references	1
Calibration function	$y=0x$
Coefficient of variation	CV 0.00 %
Correlation coefficient	n/a
⚠	Unable to compute the results for this substance because there wasn't eno ugh groups of references replicas (at least 1 for Linear-1, 2 for Linear2 and Mime-1 and 3 for Polynomial and MiMe-2)

Height calibration for substance CBDA @ RT White:

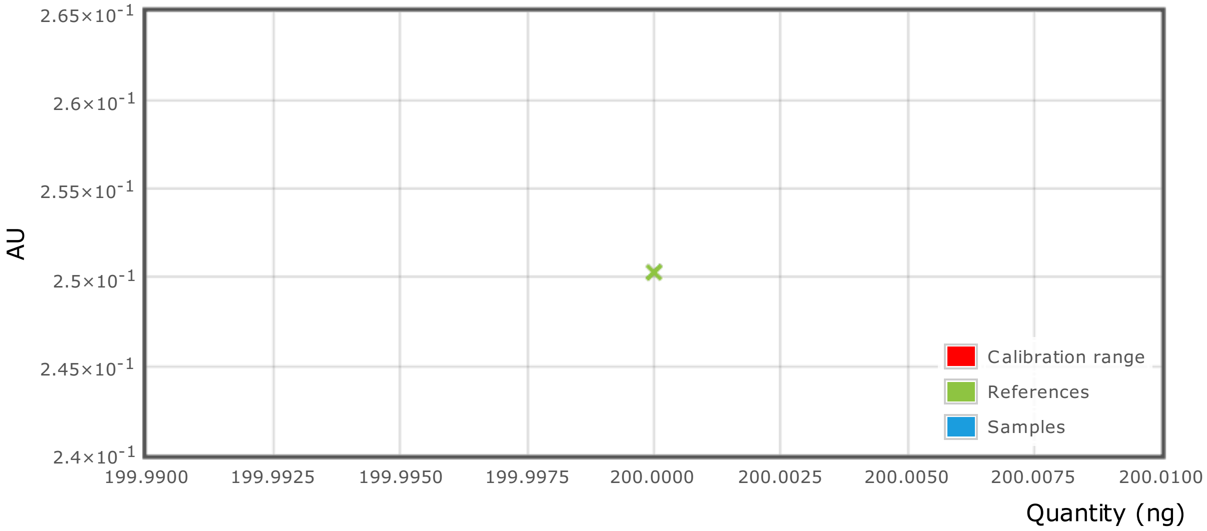

Regression mode	Linear-2
Range deviation	5.00 %
Related substances	Default
Number of references	1
Calibration function	$y=0x$
Coefficient of variation	CV 0.00 %
Correlation coefficient	n/a
⚠	Unable to compute the results for this substance because there wasn't enough groups of references replicas (at least 1 for Linear-1, 2 for Linear2 and Mime-1 and 3 for Polynomial and MiMe-2)

Height calibration for substance CBDV @ RT White:

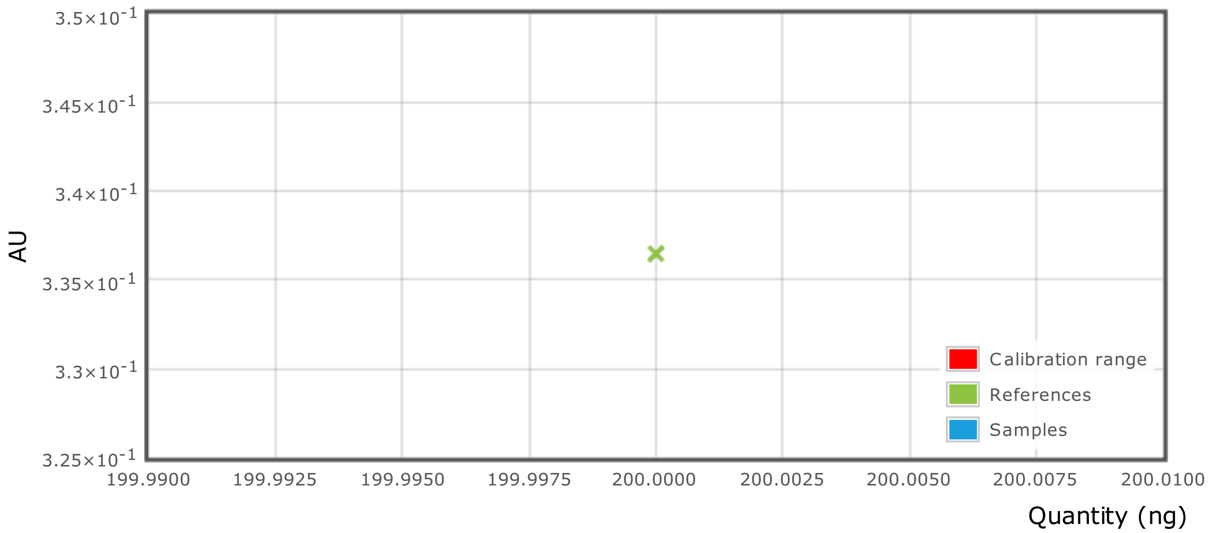

Regression mode	Linear-2
Range deviation	5.00 %
Related substances	Default
Number of references	1
Calibration function	$y=0x$
Coefficient of variation	CV 0.00 %
Correlation coefficient	n/a
⚠	Unable to compute the results for this substance because there wasn't enough groups of references replicas (at least 1 for Linear-1, 2 for Linear2 and Mime-1 and 3 for Polynomial and MiMe-2)

Height calibration for substance CBG @ RT White:

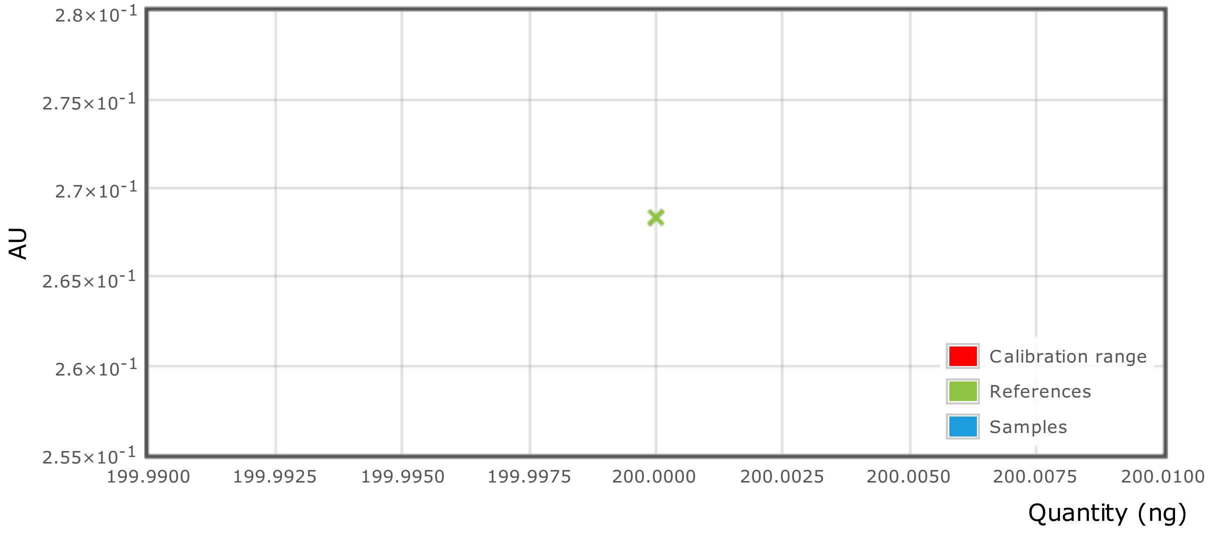

Regression mode	Linear-2
Range deviation	5.00 %
Related substances	Default
Number of references	1
Calibration function	$y=0x$
Coefficient of variation	CV 0.00 %
Correlation coefficient	n/a
⚠	Unable to compute the results for this substance because there wasn't eno ugh groups of references replicas (at least 1 for Linear-1, 2 for Linear2 and Mime-1 and 3 for Polynomial and MiMe-2)

Height calibration for substance CBGA @ RT White:

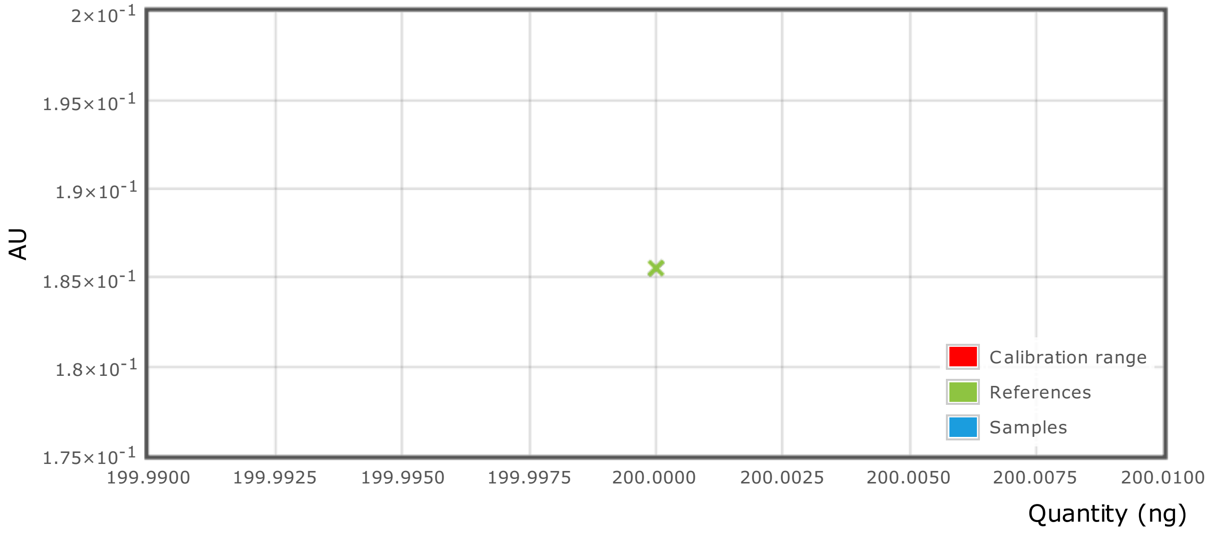

Regression mode	Linear-2
Range deviation	5.00 %
Related substances	Default
Number of references	1
Calibration function	$y=0x$
Coefficient of variation	CV 0.00 %
Correlation coefficient	n/a
⚠	Unable to compute the results for this substance because there wasn't enough groups of references replicas (at least 1 for Linear-1, 2 for Linear2 and Mime-1 and 3 for Polynomial and MiMe-2)

Height calibration for substance CBN @ RT White:

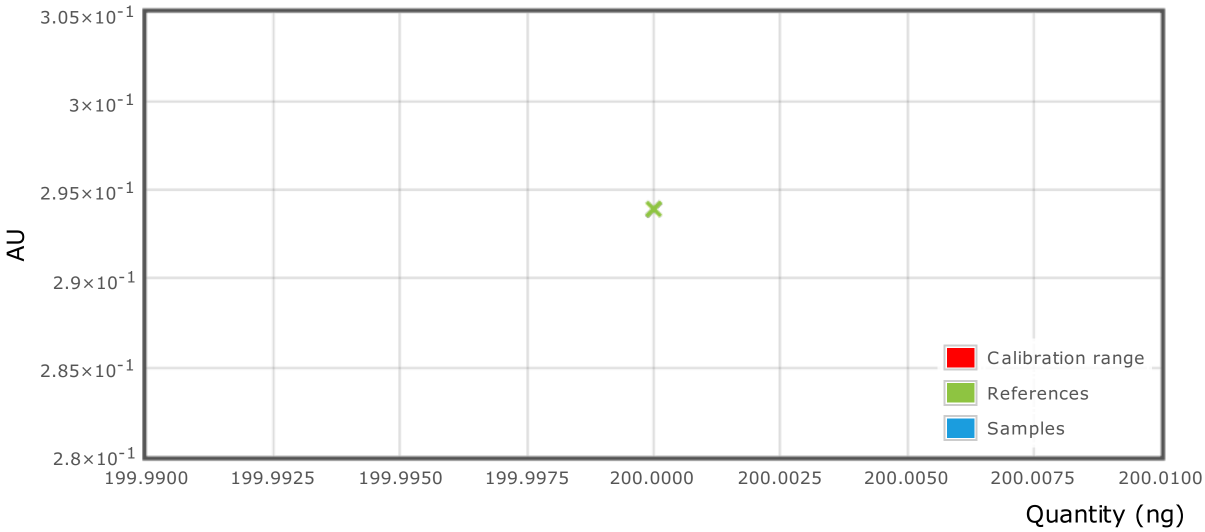

Regression mode	Linear-2
Range deviation	5.00 %
Related substances	Default
Number of references	1
Calibration function	$y=0x$
Coefficient of variation	CV 0.00 %
Correlation coefficient	n/a
⚠	Unable to compute the results for this substance because there wasn't enough groups of references replicas (at least 1 for Linear-1, 2 for Linear2 and Mime-1 and 3 for Polynomial and MiMe-2)

Height calibration for substance THCA-A @ RT White:

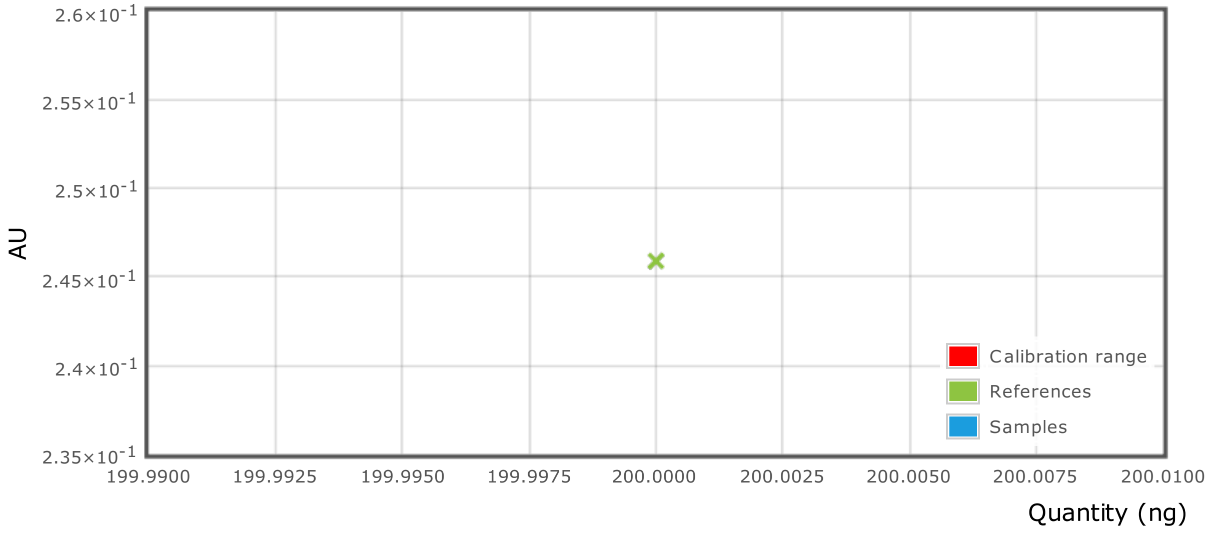

Regression mode	Linear-2
Range deviation	5.00 %
Related substances	Default
Number of references	1
Calibration function	$y=0x$
Coefficient of variation	CV 0.00 %
Correlation coefficient	n/a
⚠	Unable to compute the results for this substance because there wasn't enough groups of references replicas (at least 1 for Linear-1, 2 for Linear2 and Mime-1 and 3 for Polynomial and MiMe-2)

Height calibration for substance THCV @ RT White:

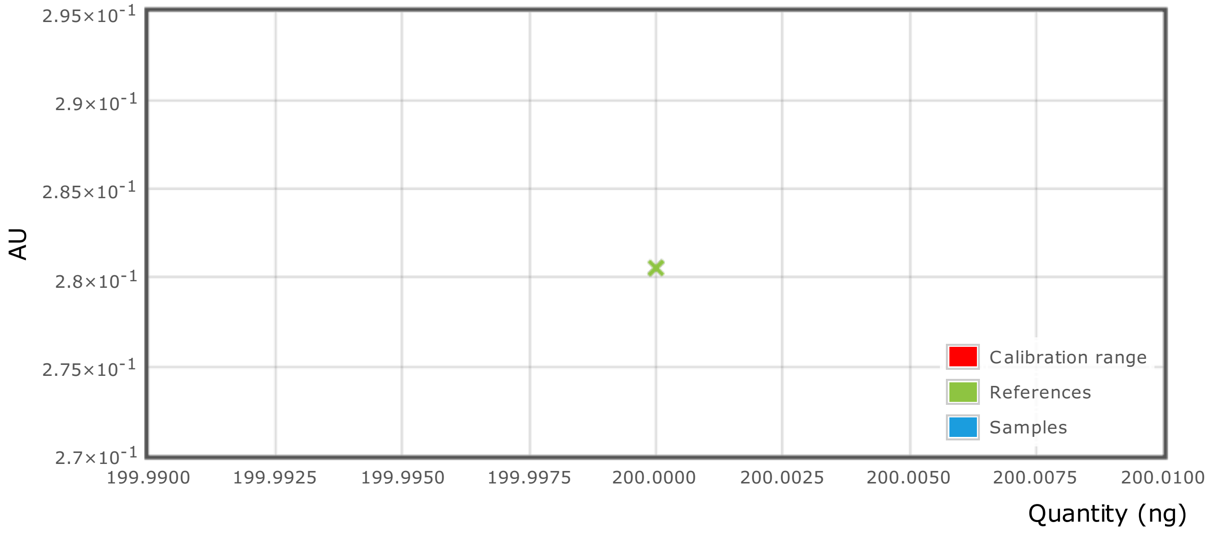

Regression mode	Linear-2
Range deviation	5.00 %
Related substances	Default
Number of references	1
Calibration function	$y=0x$
Coefficient of variation	CV 0.00 %
Correlation coefficient	n/a
⚠	Unable to compute the results for this substance because there wasn't eno ugh groups of references replicas (at least 1 for Linear-1, 2 for Linear2 and Mime-1 and 3 for Polynomial and MiMe-2)

Results:

Substance having no available results		
⚠	CBGA	There wasn't any sample application available in the assignments for this substance. Please check that the peaks were correctly detected and assig ned for this substance.
⚠	CBDV	There wasn't any sample application available in the assignments for this substance. Please check that the peaks were correctly detected and assig ned for this substance.
⚠	CBC	There wasn't any sample application available in the assignments for this substance. Please check that the peaks were correctly detected and assig ned for this substance.
⚠	CBN	There wasn't any sample application available in the assignments for this substance. Please check that the peaks were correctly detected and assig ned for this substance.
⚠	8-THC	There wasn't any sample application available in the assignments for this substance. Please check that the peaks were correctly detected and assig ned for this substance.
⚠	CBD	There wasn't any sample application available in the assignments for this substance. Please check that the peaks were correctly detected and assig ned for this substance.
⚠	THCA-A	There wasn't any sample application available in the assignments for this substance. Please check that the peaks were correctly detected and assig ned for this substance.
⚠	CBDA	There wasn't any sample application available in the assignments for this substance. Please check that the peaks were correctly detected and assig ned for this substance.
⚠	THCV	There wasn't any sample application available in the assignments for this substance. Please check that the peaks were correctly detected and assig ned for this substance.
⚠	9-THC	There wasn't any sample application available in the assignments for this substance. Please check that the peaks were correctly detected and assig ned for this substance.
⚠	CBG	There wasn't any sample application available in the assignments for this substance. Please check that the peaks were correctly detected and assig ned for this substance.

A track marked with ⚠ means: this result is outside the regression range given by the reference assignments, but is included in the results because it is in the allowed range deviation.

Analyst:	Reviewer:
