# Supplementary material for: High Performance Thin-Layer Chromatography (HPTLC) data of Cannabinoids in ten mobile phase systems
Source: Data Brief. 2020 Jun 30;31:105955. doi: 10.1016/j.dib.2020.105955 (PMC7352075; doi:10.1016/j.dib.2020.105955)
Supplement: Supplementary file 1 [file mmc1.zip › S1-Triplicate reports/MGW-3.pdf]

Analysis: MGW-3-R

Path: Home/YL Research

Based on method: Triplets Method

Created	02-Jul-2019 14:34:28	visionCATSuser
Modified	02-Jul-2019 16:23:59	visionCATSuser
Last HPTLC log	02-Jul-2019 16:23:59	Analysis modified
Explorer notes		

Track	Vial ID	Description	Volume	Position	Type
1	MeOH blank	200ng	2.0 µl	A1	Sample
2	Mixture 100	200ng	2.0 µl	A2	Sample
3	9-THC 100	200ng	2.0 µl	A3	Reference
4	CBD 100	200ng	2.0 µl	A4	Reference
5	CBN 100	200ng	2.0 µl	A5	Reference
6	CBG 100	200ng	2.0 µl	A6	Reference
7	CBC 100	200ng	2.0 µl	A7	Reference
8	THCV 100	200ng	2.0 µl	A8	Reference
9	CBDV 100	200ng	2.0 µl	A9	Reference
10	8-THC 100	200ng	2.0 µl	A10	Reference
11	THCA-A 100	200ng	2.0 µl	A11	Reference
12	CBDA 100	200ng	2.0 µl	B1	Reference
13	CBGA 100	200ng	2.0 µl	B2	Reference
Sequence table notes					

A track marked with ⚠ means: the application type is overridden in some evaluation(s).

System setup:

Software	Server User-PC, version 2.5.18072.1
ATS4	S/N:080713
Chamber	N/A
Derivatization dip	N/A
Scanner3	S/N:031025
Visualizer	S/N:230515

Chromatography

Plate layout:

Stationary phase	Merck, HPTLC plates RP-18 F 254s
Plate format	100.0 x 100.0 mm
Application type	Spot
Application	Position Y: 8.0 mm, length: 0.0 mm, width: 0.0 mm
Track	First position X: 20.0 mm, distance: 5.0 mm
Solvent front position	70.0 mm
Notes	

Take image clean plate 1a - Visualizer (S/N: 230515):

Quality	Enhanced
RT White	auto capture, Auto, level 85 %, Band
R 254	auto capture, Auto, level 85 %, Band
Instrument diagnostics	Valid diagnostics
Documentation step label	
Notes	

Application 1 - ATS 4 (S/N: 080713):

Spray gas	NI
Sample solvent type	Methanol
Filling speed	15 µl/s
Predosage volume	200 nl
Retraction volume	200 nl
Dosage speed	150 nl/s
Filling quality	User
Rinsing cycles / vacuum	1 / 4 s
Filling cycles / vacuum	1 / 4 s
Rinsing solvent name	Methanol
Nozzle temperature	Unheated
Rack in use	Standard
Instrument diagnostics	Valid diagnostics
Notes	

Development 1 - Chamber:

Tank	TTC 20x10
Mobile phase	Methanol, 0.1% acetic acid in water (75:25)
Saturation time	20 min
Use saturation pad	true
Use smartALERT	true
Volume front through	10 ml
Volume rear through	25 ml
Drying time	5 min
Drying temperature	Room temperature
Notes	

Take image developed plate 1a - Visualizer (S/N: 230515):

Quality	Enhanced
RT White	auto capture, Auto, level 85 %, Band
R 254	auto capture, Auto, level 85 %, Band
R 366	auto capture, Auto, level 85 %, Band
Instrument diagnostics	Valid diagnostics
Documentation step label	
Notes	

Scan developed plate 1b - Scanner 3 (S/N: 031025):

Scanner type	Single λ
Optimization for	Resolution
Measurement mode	Absorption
Filter	n/a
Detector mode	Automatic
Scanning speed	20 mm/s
Data resolution	100 µm/step
Slit	5 x 0.2 mm, micro
Partial scan	No
Lamp	Deuterium & Tungsten
Wavelength(s)	254 nm
Instrument diagnostics	Valid diagnostics
Documentation step label	
Notes	

Derivatization 1 - dip:

Reagent name	Fast Blue B salt
Dipping speed	3
Dipping time	5 s
Reagent preparation	1g Fast Blue B salt in 200mL water
Heating	none
Notes	Air dry for 5 minutes

Take image derivatized plate 1a - Visualizer (S/N: 230515):

Quality	Enhanced
RT White	auto capture, Auto, level 85 %, Band
R 366	auto capture, Auto, level 85 %, Band
Instrument diagnostics	Valid diagnostics
Documentation step label	
Notes	

System suitability tests:

SST settings:

SST tracks	

Data acquisition

Application 1 - ATS 4 (S/N: 080713):

Executed	02-Jul-2019 14:39:05 visionCATSuser

Development 1 - Chamber:

Executed	02-Jul-2019 15:59:51 visionCATSuser

Take image developed plate 1a - Visualizer (S/N: 230515):

Executed	02-Jul-2019 16:04:56 visionCATSuser

RT White

Developed, RemTransVis

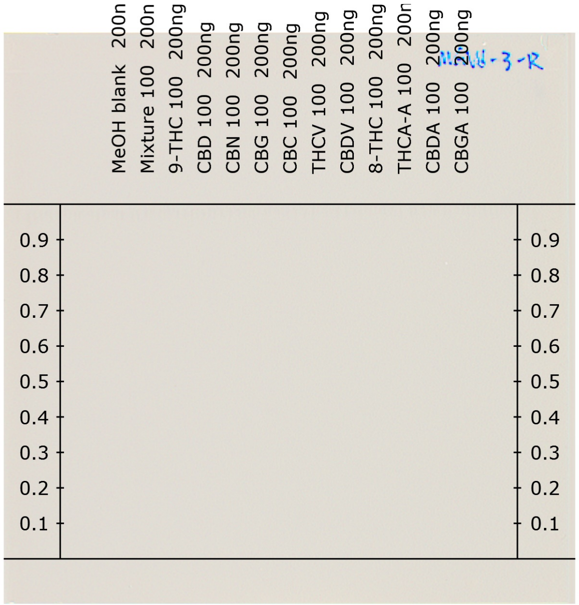

Exposure	0.050 s
Contrast	1
Normalized exposure	Disabled
Clarify	Disabled
White balance	1.00, 1.00, 1.00

R 254

Developed, Remission254

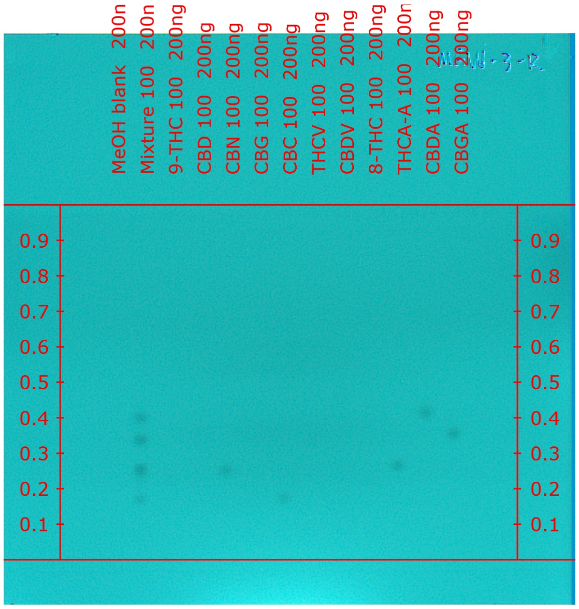

Exposure	0.219 s
Contrast	1
Normalized exposure	Disabled
Clarify	Disabled
White balance	1.00, 1.00, 1.00

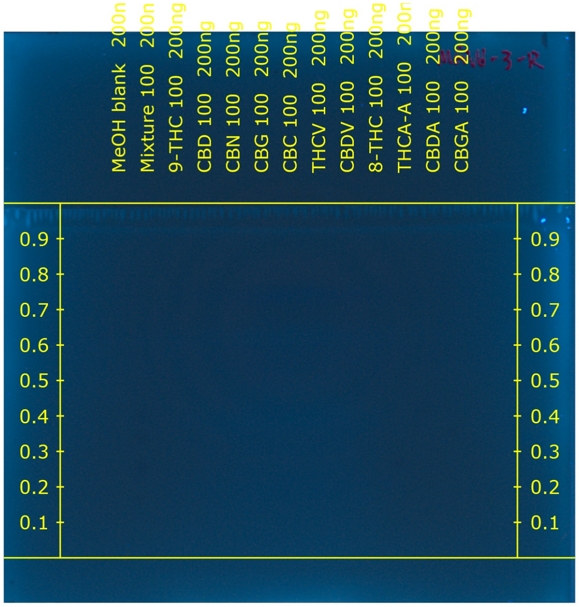

Exposure	2.650 s
Contrast	1
Normalized exposure	Disabled
Clarify	Disabled
White balance	1.00, 1.00, 1.00

Scan developed plate 1b - Scanner 3 (S/N: 031025):

Executed	02-Jul-2019 16:08:34 visionCATSuser

Scan:

Wavelength	254 nm

Track 1:

Type	Single λ

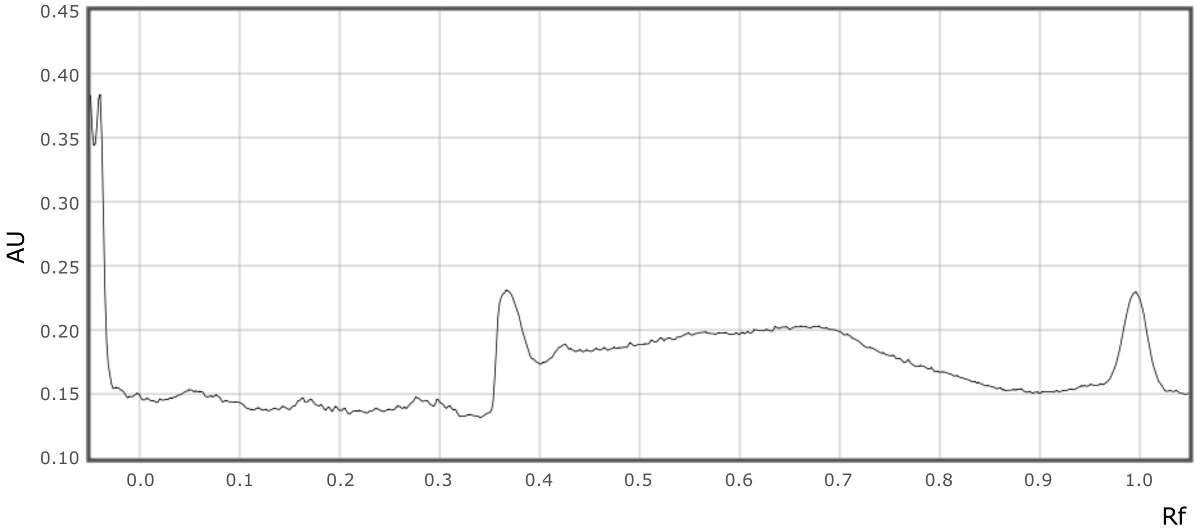

Track 2:

Type	Single λ

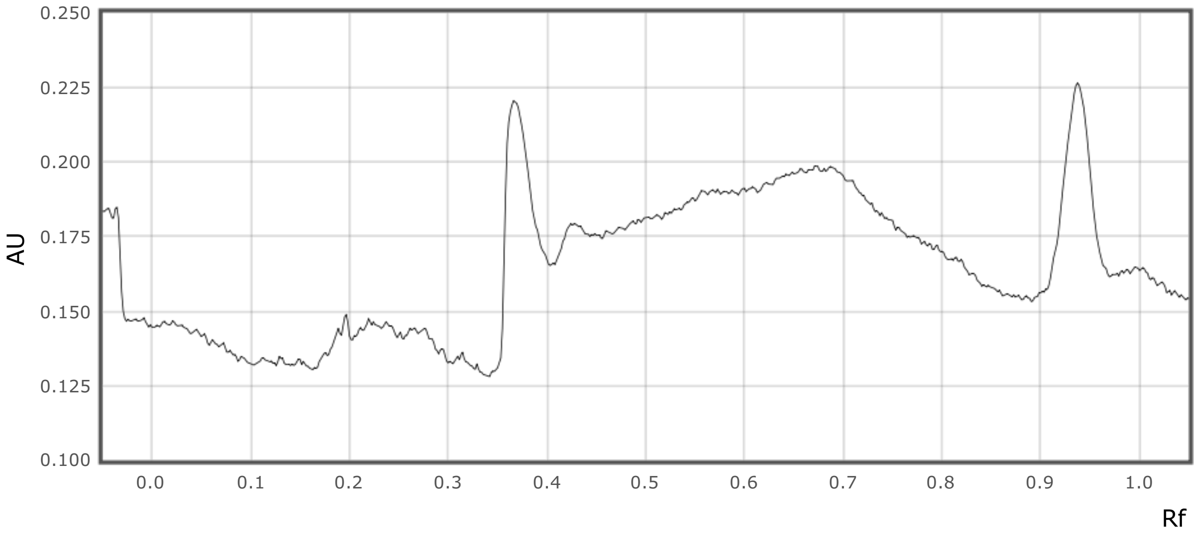

Track 3:

Type	Single λ

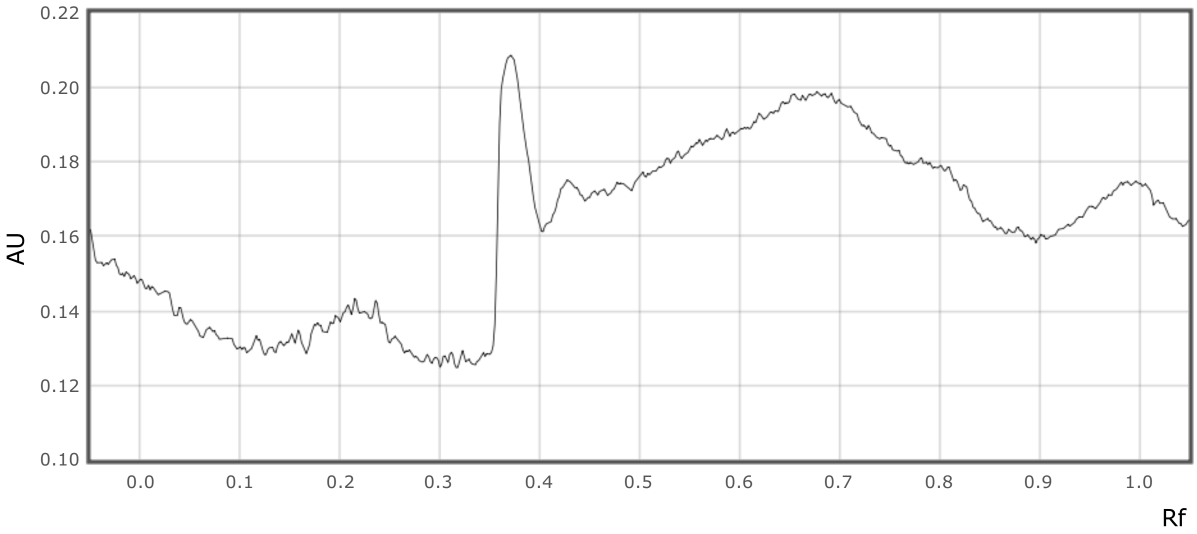

Track 4:

Type	Single λ

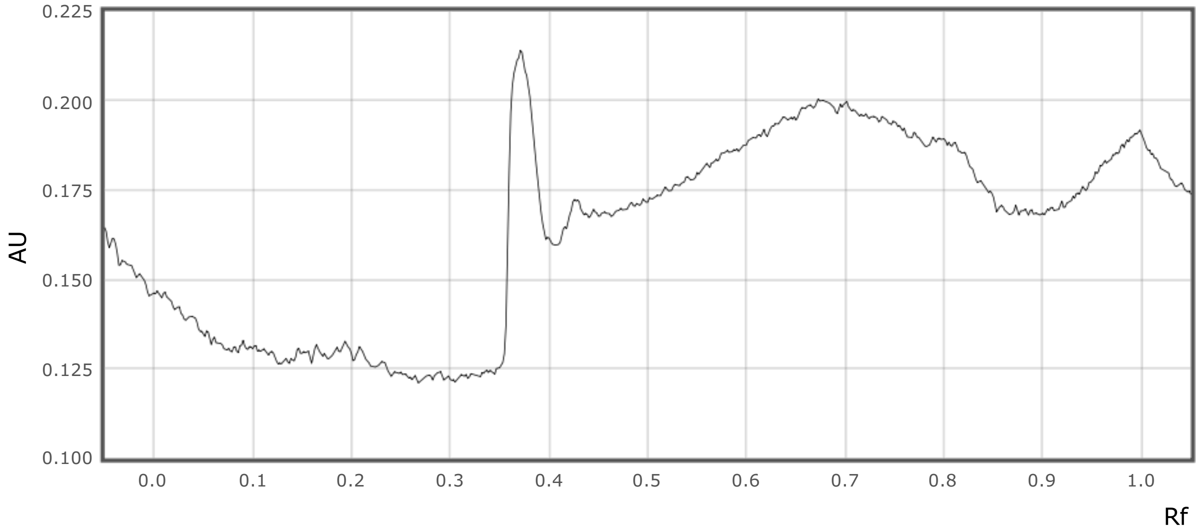

Track 5:	
Type	Single λ

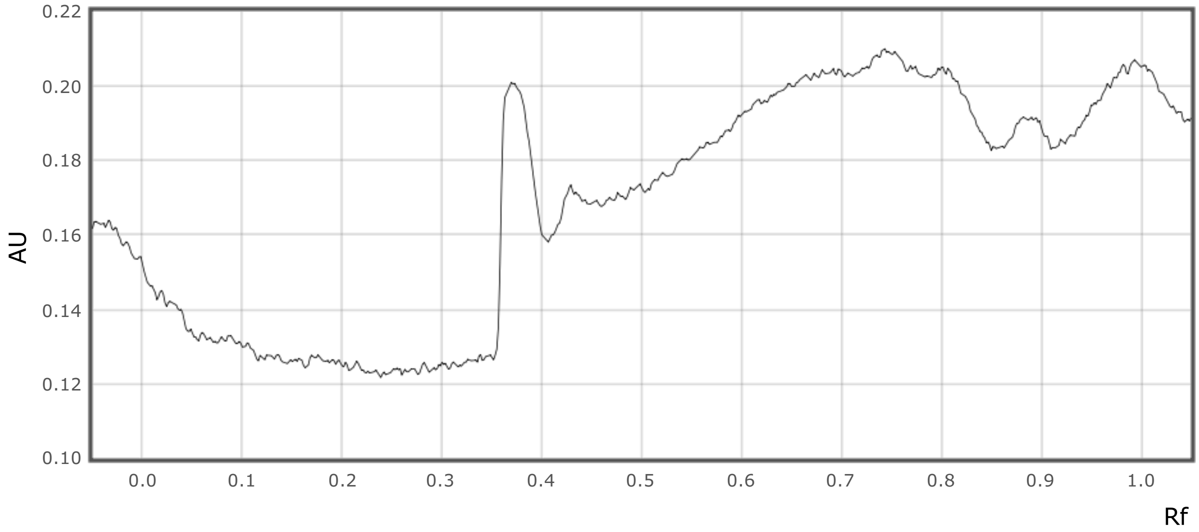

Track 6:	
Type	Single λ

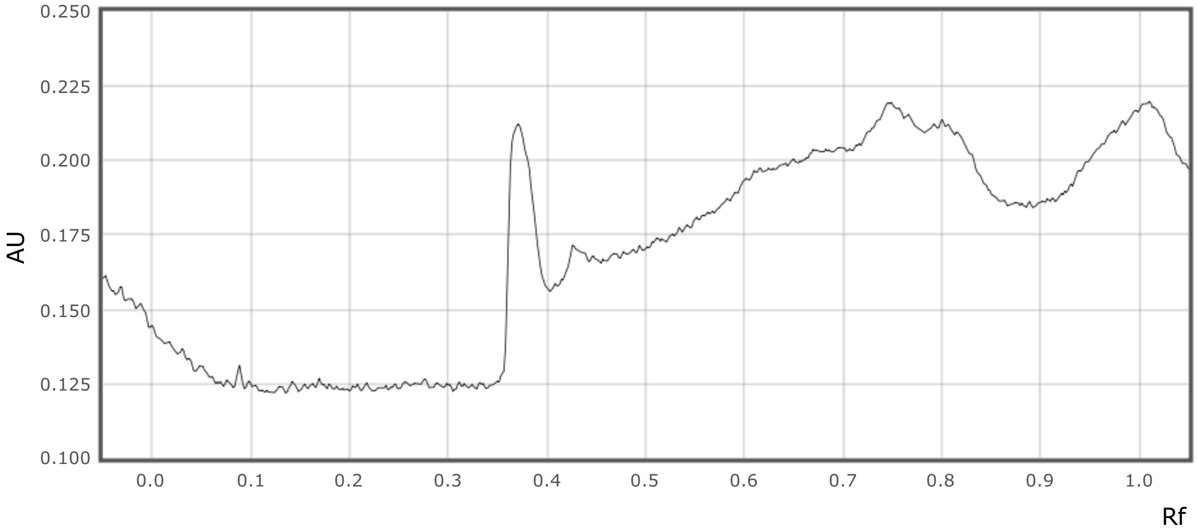

Track 7:

Type	Single λ

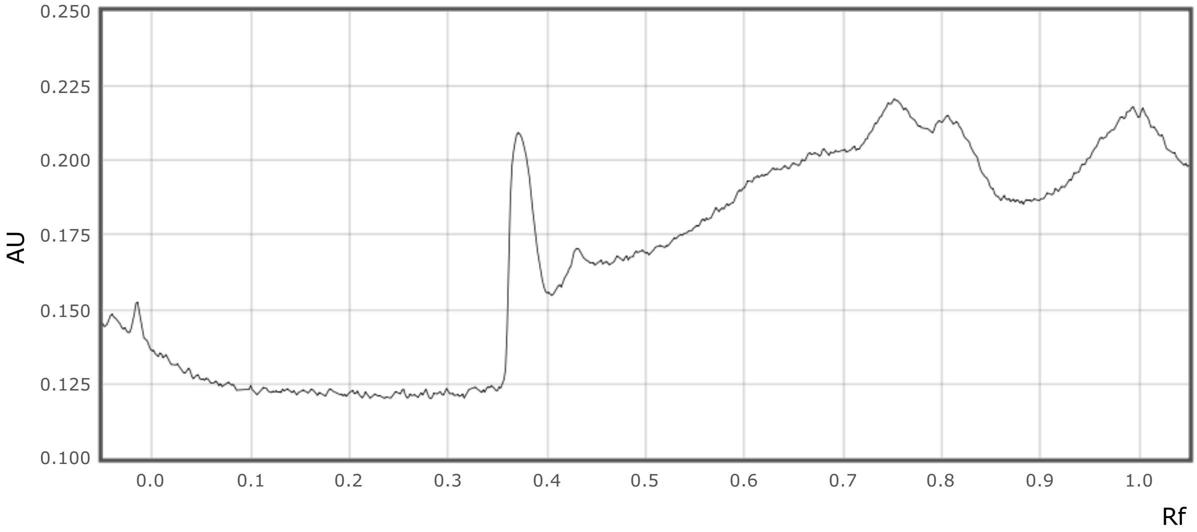

Track 8:

Type	Single λ

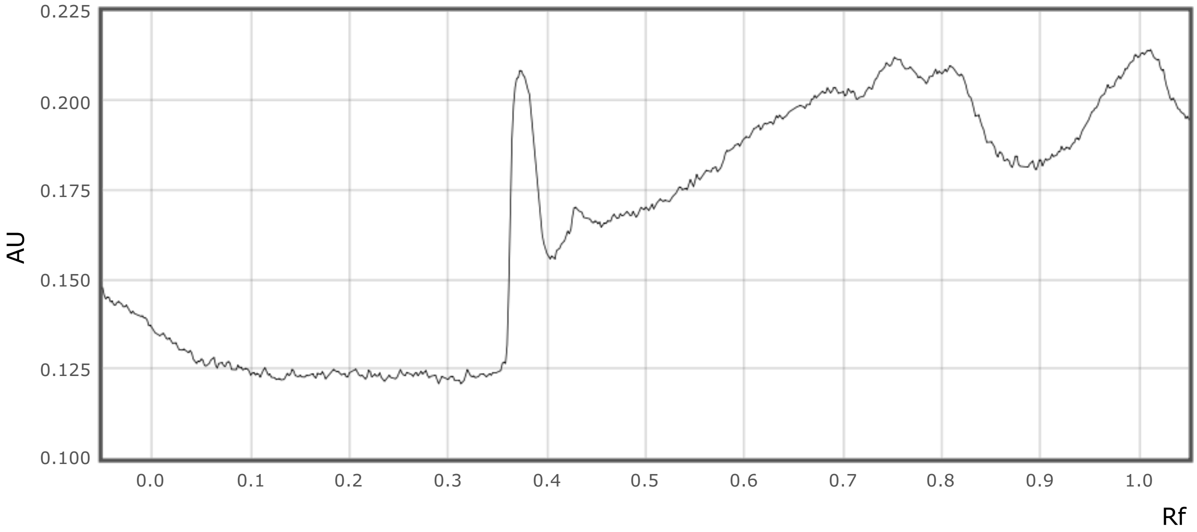

Track 9:	
Type	Single λ

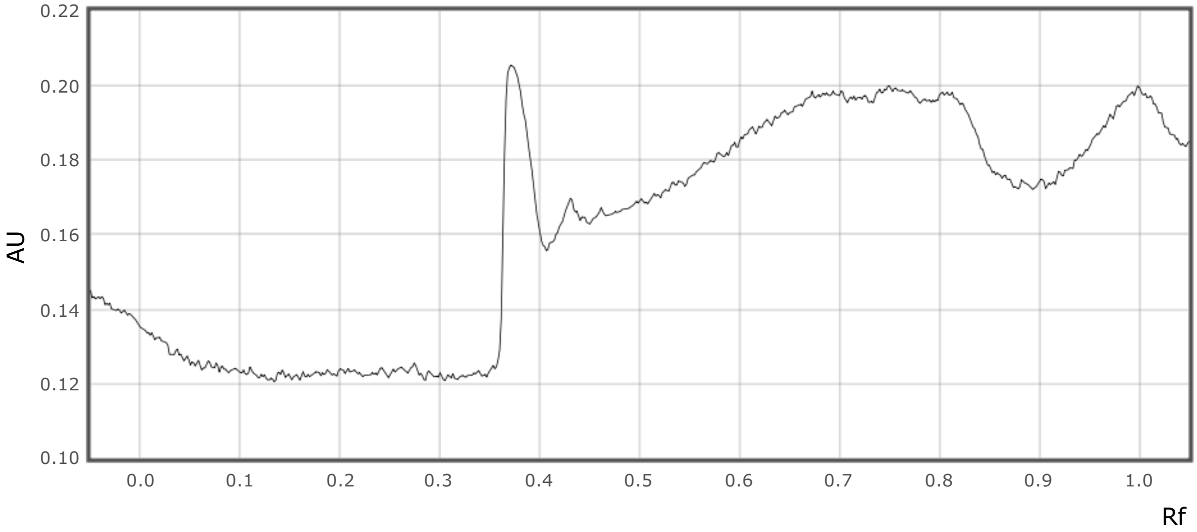

Track 10:	
Type	Single λ

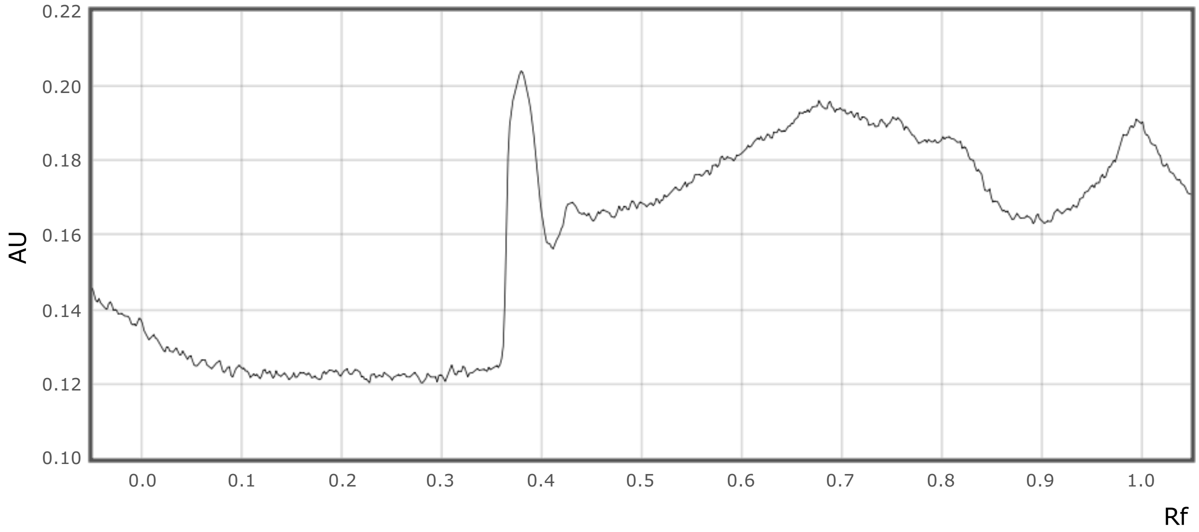

Track 11:	
Type	Single λ

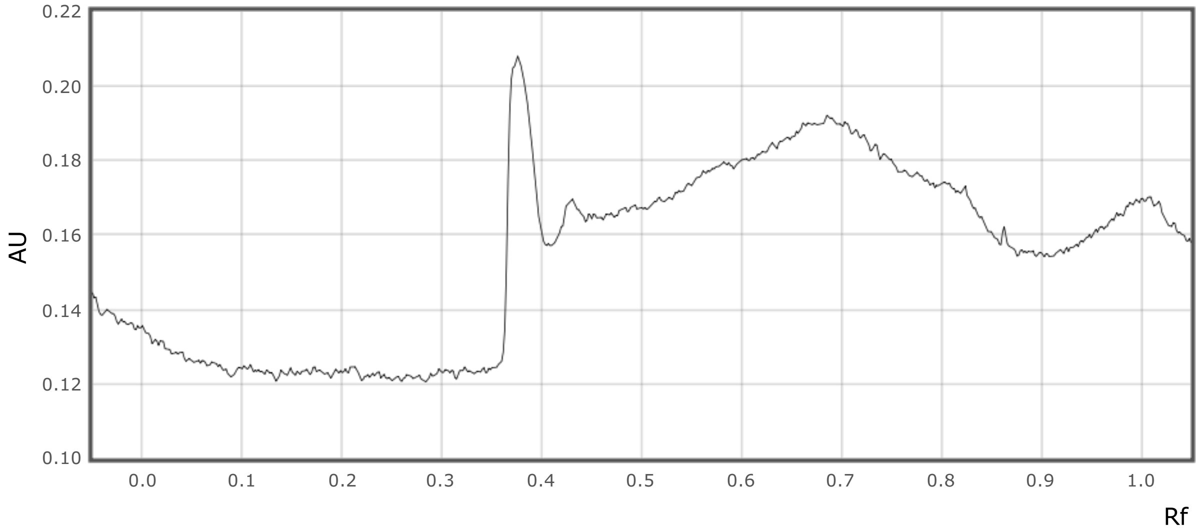

Track 12:	
Type	Single λ

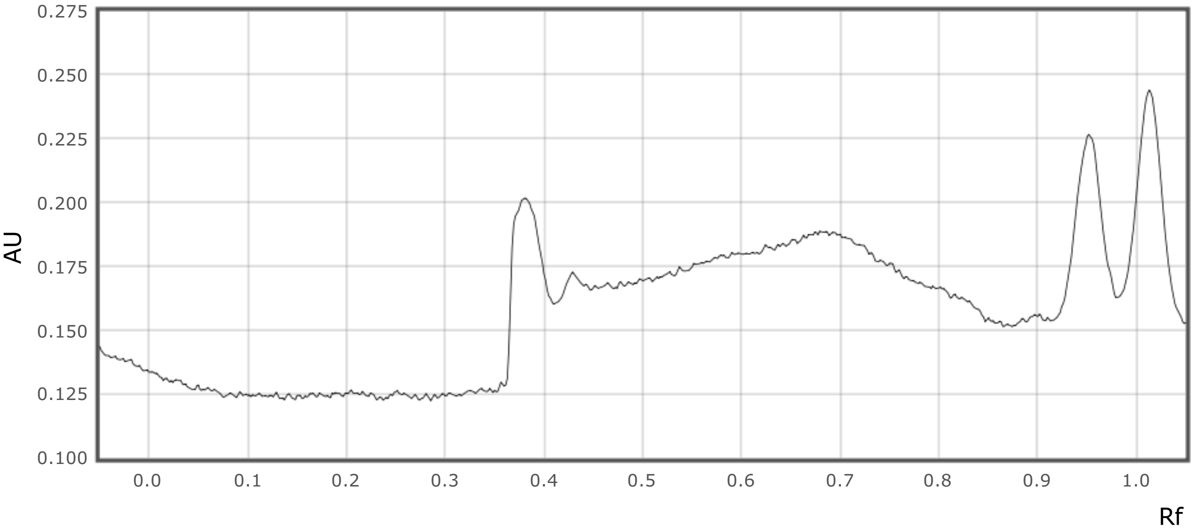

Track 13:	
Type	Single λ

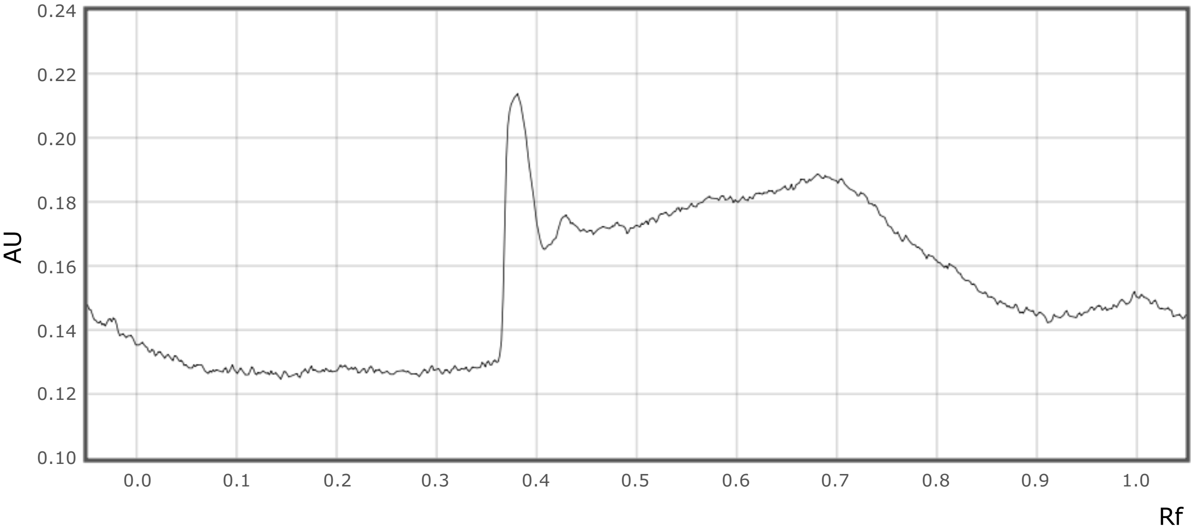

Derivatization 1 - dip:

Executed	02-Jul-2019 16:16:06 visionCATSuser

Take image derivatized plate 1a - Visualizer (S/N: 230515):

Executed	02-Jul-2019 16:17:21 visionCATSuser

RT White Derivatized, RemTransVis

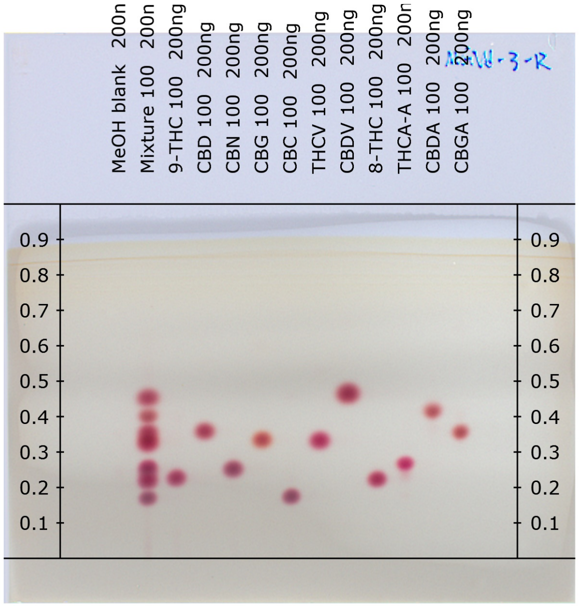

Exposure	0.051 s
Contrast	1
Normalized exposure	Disabled
Clarify	Disabled
White balance	1.13, 1.06, 0.86

R 366 Derivatized, Remission366

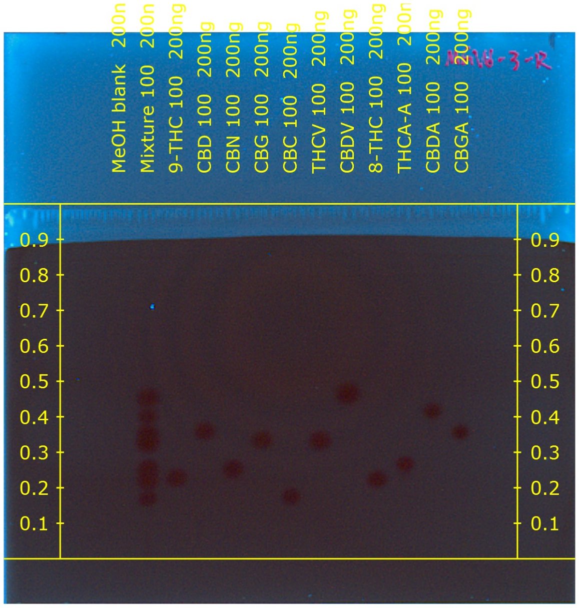

Exposure	9.607 s
Contrast	1
Normalized exposure	Disabled
Clarify	Disabled
White balance	1.00, 1.00, 1.00

Evaluation 1 :

Validated	false
Step	Take image derivatized plate 1a
Concentration unit type	Mass / volume
Notes	

Definition:

References:

9-THC 100

Substance Name	Concentration	Purity
9-THC	100.000 µg/ml	100.00 %

CBD 100

Substance Name	Concentration	Purity
CBD	100.000 µg/ml	100.00 %

CBN 100

Substance Name	Concentration	Purity
CBN	100.000 µg/ml	100.00 %

CBG 100

Substance Name	Concentration	Purity
CBG	100.000 µg/ml	100.00 %

CBC 100

Substance Name	Concentration	Purity
CBC	100.000 µg/ml	100.00 %

THCV 100

Substance Name	Concentration	Purity
THCV	100.000 µg/ml	100.00 %

CBDV 100

Substance Name	Concentration	Purity
CBDV	100.000 µg/ml	100.00 %

8-THC 100

Substance Name	Concentration	Purity
8-THC	100.000 µg/ml	100.00 %

THCA-A 100

Substance Name	Concentration	Purity
THCA-A	100.000 µg/ml	100.00 %

CBDA 100

Substance Name	Concentration	Purity
CBDA	100.000 µg/ml	100.00 %

CBGA 100

Substance Name	Concentration	Purity
CBGA	100.000 µg/ml	100.00 %

Samples:

Vial ID	Amount	Volume solution	Reference amount	Related to
MeOH blank		0.00 ml		
Mixture 100		0.00 ml		

Integration parameters:

Bounds	[0.000,1.000]
Smoothing	Savitzky–Golay of order 3 and window 7
Baseline correction	Lowest slope with noise 0.05
Profile subtraction	Profile subtraction from track 1
Peaks detection	Gauss (legacy) with sensitivity 0.1, separation 1 and threshold 0.1

Scan:

Wavelength	RT White

Track 1:

Type	Sample
Vial ID	MeOH blank
Description	200ng
Volume	2.0 µl

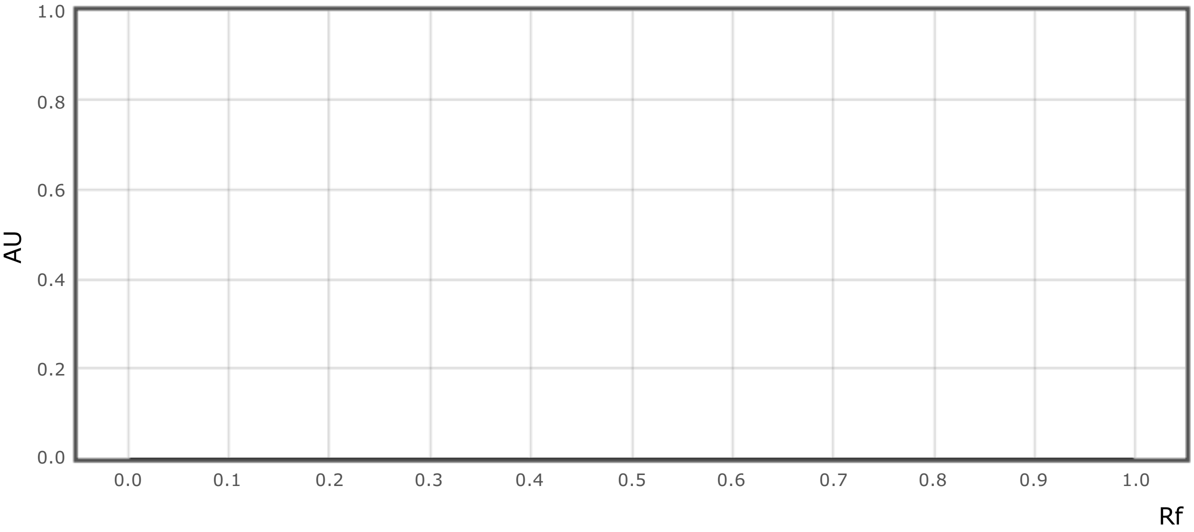

Peak #	Start		Max			End		Area		Manual peak	Substance Name
	Rf	H	Rf	H	%	Rf	H	A	%		

Track 2:

Type	Sample
Vial ID	Mixture 100
Description	200ng
Volume	2.0 µl

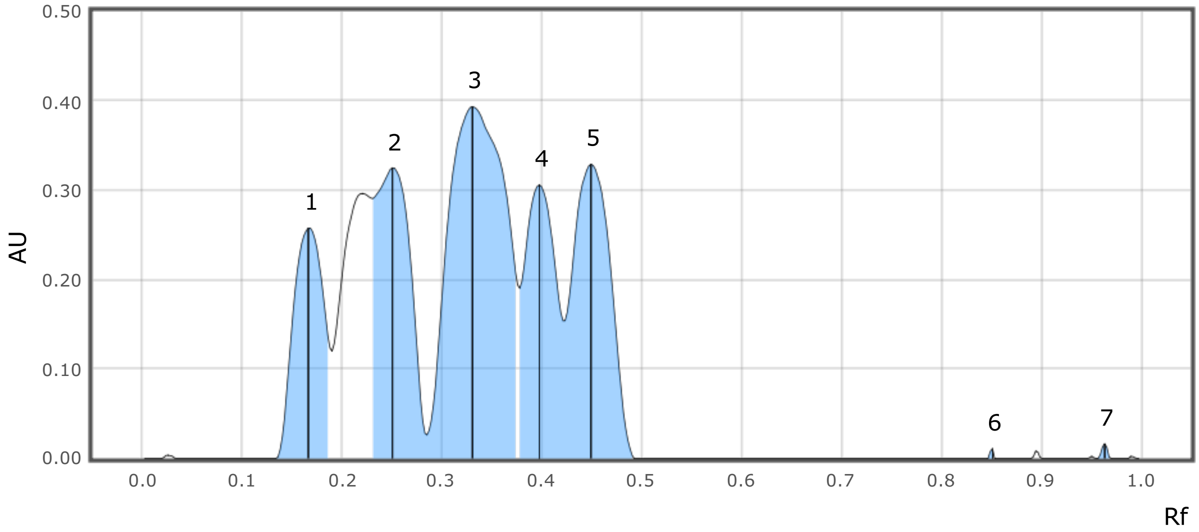

Peak #	Start		Max			End		Area		Manual peak	Substance Name
	Rf	H	Rf	H	%	Rf	H	A	%		
1	0.134	0.0000	0.166	0.2573	15.72	0.188	0.1235	0.00893	12.14	No	
2	0.231	0.2903	0.250	0.3247	19.84	0.285	0.0261	0.01298	17.65	No	
3	0.285	0.0261	0.330	0.3929	24.01	0.376	0.1937	0.02613	35.51	No	
4	0.378	0.1901	0.397	0.3056	18.67	0.421	0.1537	0.01077	14.64	No	
5	0.421	0.1537	0.449	0.3286	20.08	0.492	0.0000	0.01462	19.87	No	
6	0.847	0.0000	0.851	0.0110	0.67	0.853	0.0000	0.00004	0.05	No	
7	0.957	0.0000	0.964	0.0165	1.01	0.970	0.0000	0.00010	0.14	No	

Track 3:

Type	Reference
Vial ID	9-THC 100
Description	200ng
Volume	2.0 µl

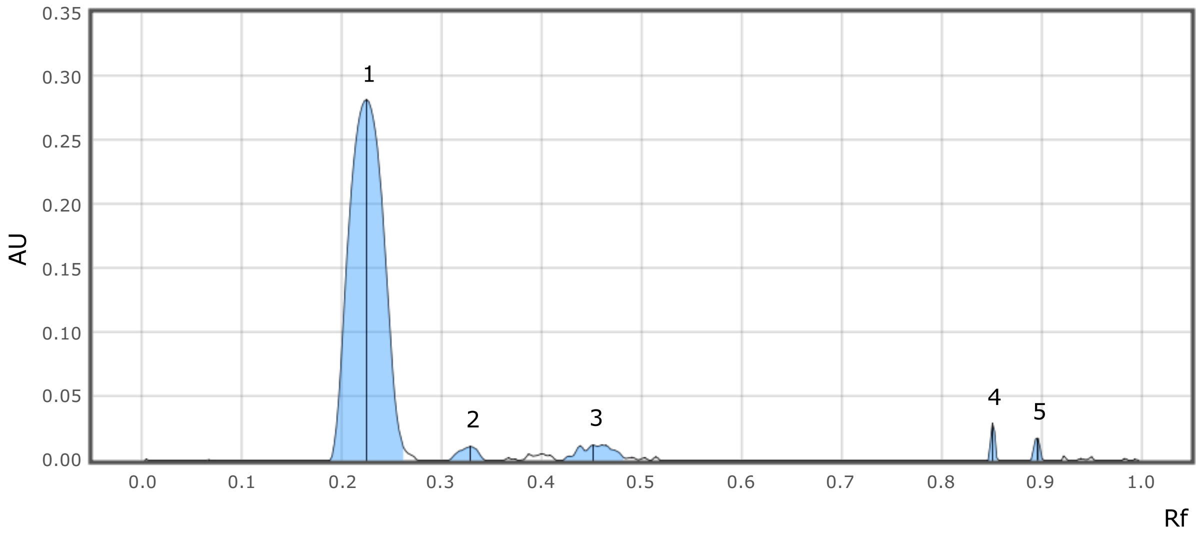

Peak	Start		Max			End		Area		Manual	Substance
#	Rf	H	Rf	H	%	Rf	H	A	%	peak	Name
1	0.188	0.0000	0.224	0.2819	80.28	0.263	0.0076	0.01151	92.14	Yes	9-THC
2	0.307	0.0000	0.328	0.0108	3.07	0.343	0.0000	0.00023	1.87	No	
3	0.421	0.0000	0.451	0.0120	3.43	0.484	0.0014	0.00049	3.90	No	
4	0.847	0.0000	0.851	0.0290	8.26	0.858	0.0000	0.00014	1.14	No	
5	0.888	0.0000	0.897	0.0174	4.96	0.903	0.0000	0.00012	0.94	No	

Track 4:

Type	Reference
Vial ID	CBD 100
Description	200ng
Volume	2.0 µl

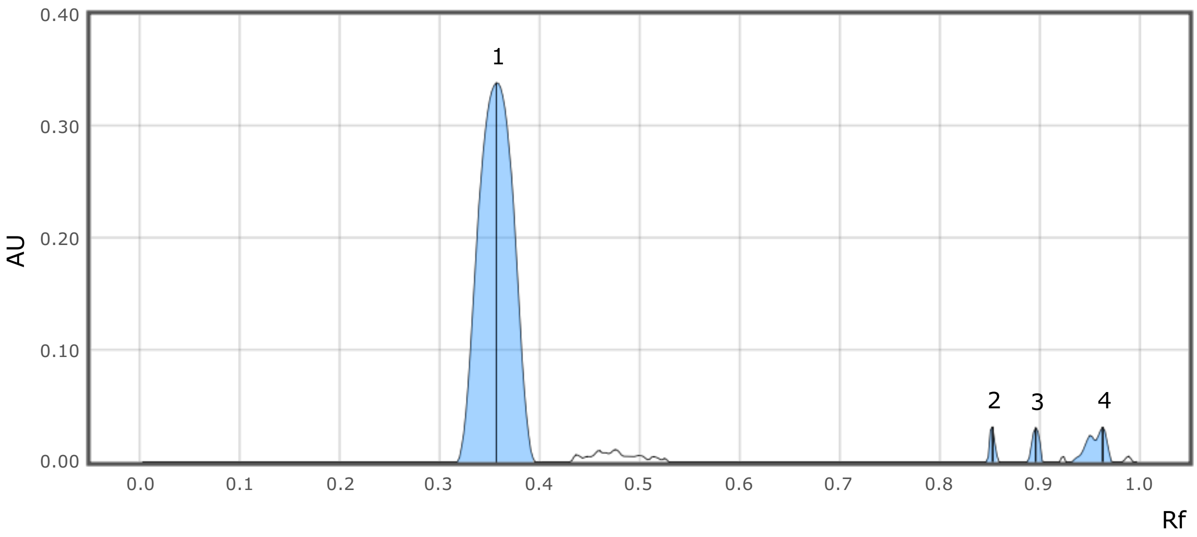

Peak	Start		Max			End		Area		Manual	Substance
#	Rf	H	Rf	H	%	Rf	H	A	%	peak	Name
1	0.315	0.0000	0.356	0.3389	78.45	0.395	0.0000	0.01416	93.00	No	CBD
2	0.847	0.0000	0.853	0.0314	7.27	0.860	0.0000	0.00019	1.23	No	
3	0.888	0.0000	0.897	0.0306	7.07	0.905	0.0000	0.00026	1.70	No	
4	0.931	0.0000	0.964	0.0311	7.20	0.974	0.0000	0.00062	4.07	No	

Track 5:

Type	Reference
Vial ID	CBN 100
Description	200ng
Volume	2.0 µl

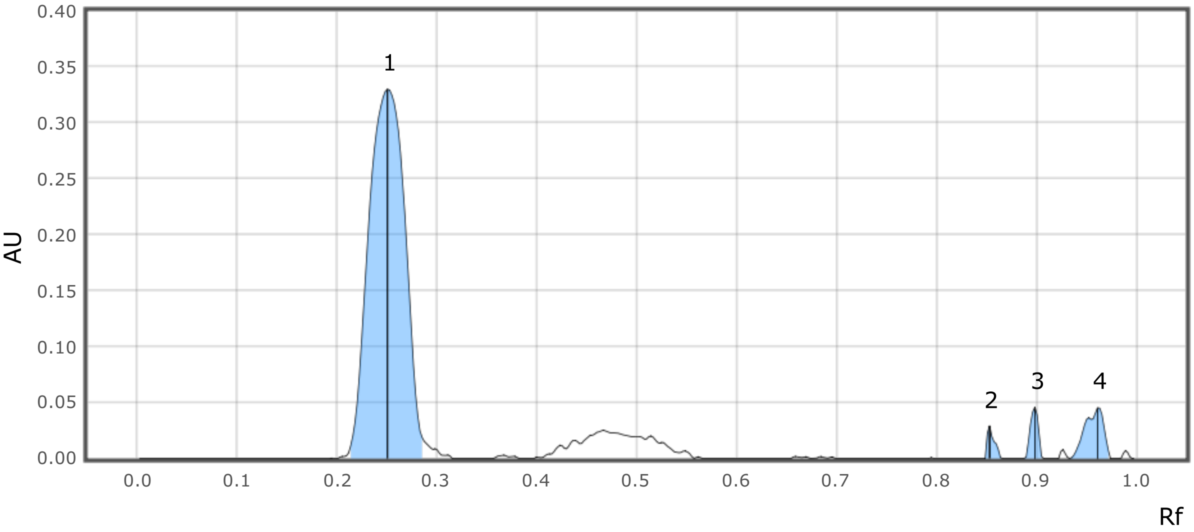

Peak #	Start Rf	Start H	Max Rf	Max H	Max %	End Rf	End H	Area A	Area %	Manual peak	Substance Name
1	0.212	0.0049	0.250	0.3300	73.32	0.286	0.0153	0.01395	89.54	Yes	CBN
2	0.849	0.0000	0.853	0.0291	6.48	0.866	0.0000	0.00023	1.50	No	
3	0.888	0.0000	0.899	0.0458	10.17	0.907	0.0000	0.00042	2.70	No	
4	0.933	0.0000	0.961	0.0451	10.03	0.974	0.0000	0.00098	6.26	No	

Track 6:

Type	Reference
Vial ID	CBG 100
Description	200ng
Volume	2.0 µl

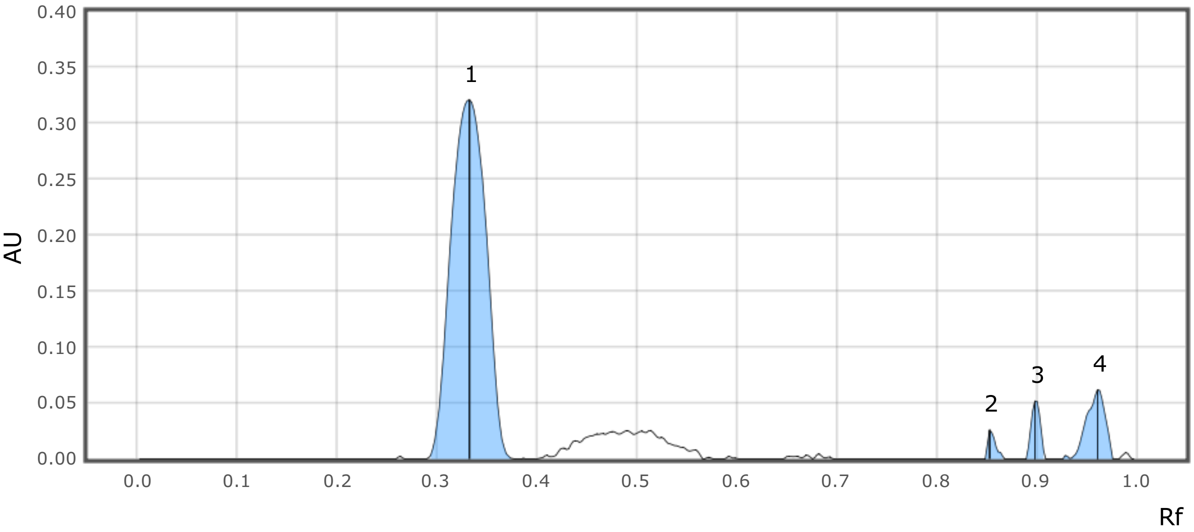

Peak	Start		Max			End		Area		Manual	Substance
#	Rf	H	Rf	H	%	Rf	H	A	%	peak	Name
1	0.293	0.0029	0.333	0.3209	69.69	0.374	0.0008	0.01310	86.13	Yes	CBG
2	0.849	0.0000	0.853	0.0259	5.63	0.868	0.0000	0.00023	1.50	No	
3	0.890	0.0000	0.899	0.0518	11.24	0.910	0.0000	0.00056	3.65	No	
4	0.927	0.0000	0.961	0.0619	13.43	0.979	0.0000	0.00133	8.72	No	

Track 7:

Type	Reference
Vial ID	CBC 100
Description	200ng
Volume	2.0 µl

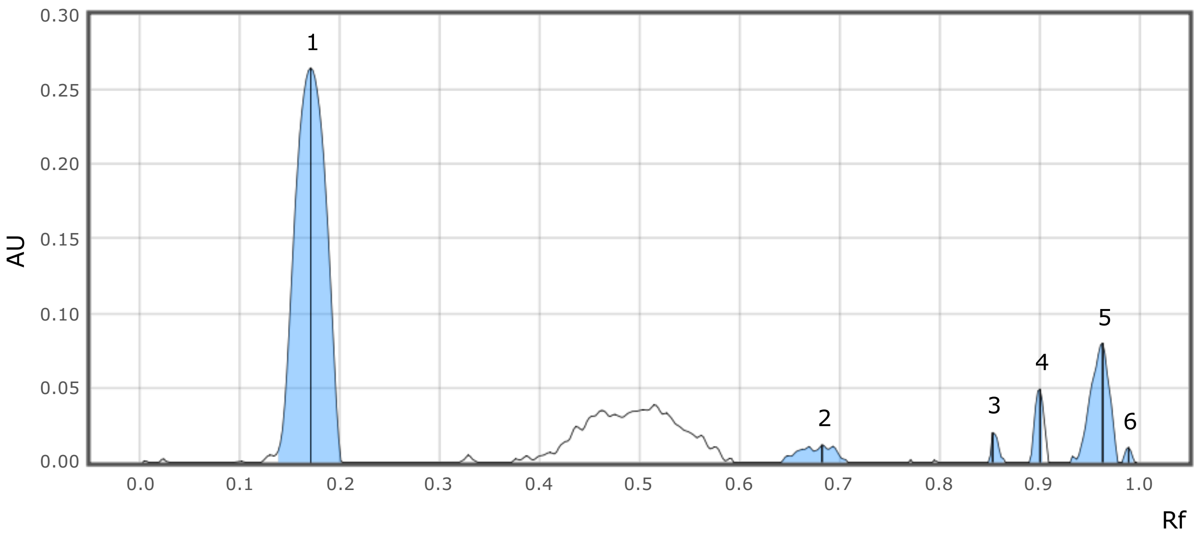

Peak	Start		Max			End		Area		Manual	Substance
#	Rf	H	Rf	H	%	Rf	H	A	%	peak	Name
1	0.136	0.0052	0.170	0.2643	60.79	0.203	0.0000	0.00966	76.51	Yes	CBC
2	0.642	0.0000	0.683	0.0117	2.70	0.709	0.0000	0.00047	3.68	No	
3	0.849	0.0000	0.853	0.0200	4.59	0.866	0.0000	0.00016	1.28	No	
4	0.890	0.0000	0.901	0.0489	11.25	0.910	0.0000	0.00053	4.19	No	
5	0.931	0.0000	0.964	0.0798	18.36	0.981	0.0000	0.00174	13.75	No	
6	0.983	0.0000	0.989	0.0100	2.30	0.996	0.0000	0.00007	0.58	No	

Track 8:

Type	Reference
Vial ID	THCV 100
Description	200ng
Volume	2.0 µl

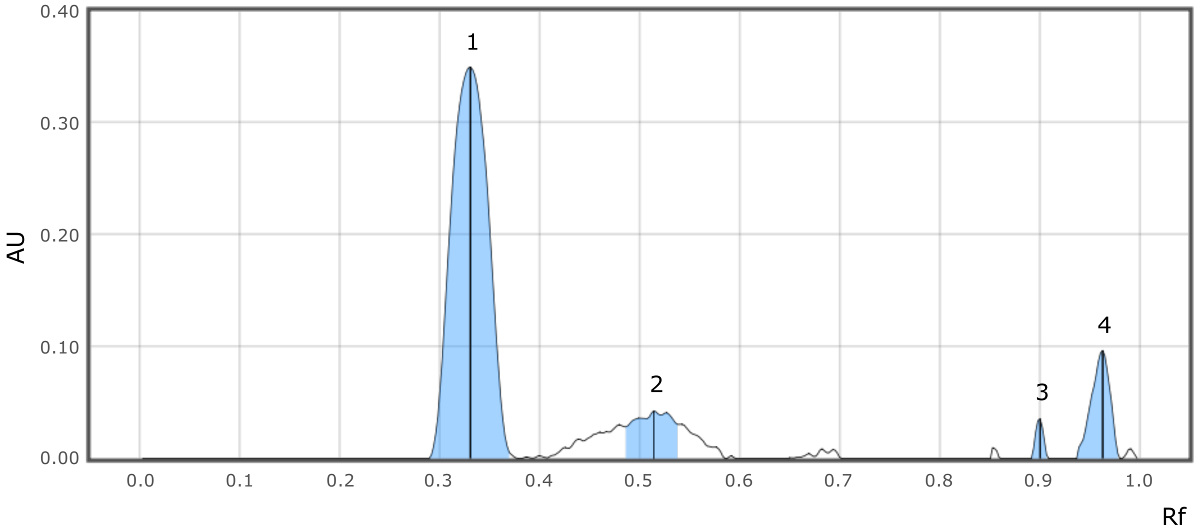

Peak #	Start Rf	Start H	Max Rf	Max H	Max %	End Rf	End H	Area A	Area %	Manual peak	Substance Name
1	0.289	0.0000	0.330	0.3497	66.71	0.371	0.0038	0.01521	77.72	Yes	THCV
2	0.486	0.0282	0.514	0.0426	8.12	0.540	0.0303	0.00196	10.00	No	
3	0.892	0.0000	0.901	0.0354	6.75	0.910	0.0000	0.00032	1.65	No	
4	0.938	0.0000	0.964	0.0966	18.42	0.981	0.0000	0.00208	10.63	No	

Track 9:

Type	Reference
Vial ID	CBDV 100
Description	200ng
Volume	2.0 µl

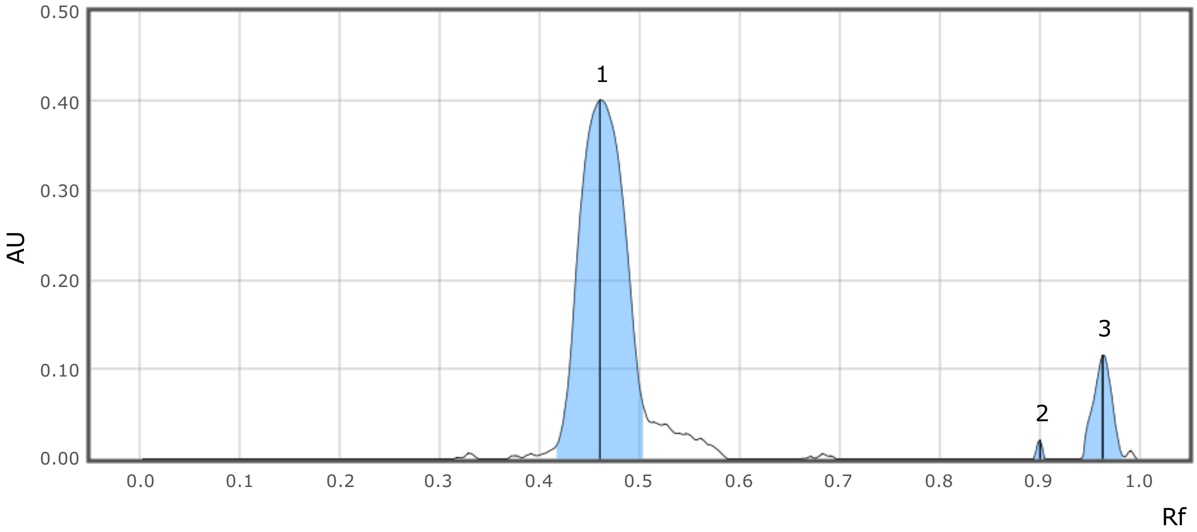

Peak #	Start Rf	Start H	Max Rf	Max H	Max %	End Rf	End H	Area A	Area %	Manual peak	Substance Name
1	0.416	0.0121	0.460	0.4009	74.51	0.507	0.0448	0.02166	89.18	Yes	CBDV
2	0.894	0.0000	0.901	0.0211	3.92	0.907	0.0000	0.00013	0.55	No	
3	0.942	0.0000	0.964	0.1161	21.57	0.985	0.0021	0.00250	10.28	No	

Track 10:

Type	Reference
Vial ID	8-THC 100
Description	200ng
Volume	2.0 µl

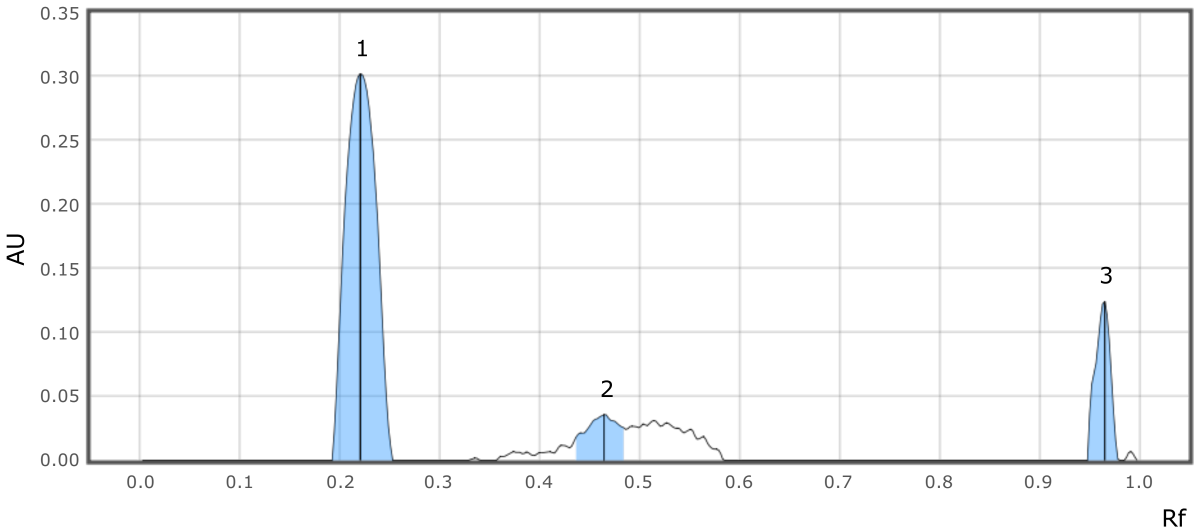

Peak #	Start		Max			End		Area		Manual peak	Substance Name
	Rf	H	Rf	H	%	Rf	H	A	%		
1	0.192	0.0000	0.220	0.3021	65.41	0.255	0.0000	0.01102	75.31	No	8-THC
2	0.436	0.0181	0.464	0.0357	7.72	0.486	0.0242	0.00141	9.64	No	
3	0.946	0.0000	0.966	0.1241	26.87	0.981	0.0000	0.00220	15.05	No	

Track 11:

Type	Reference
Vial ID	THCA-A 100
Description	200ng
Volume	2.0 µl

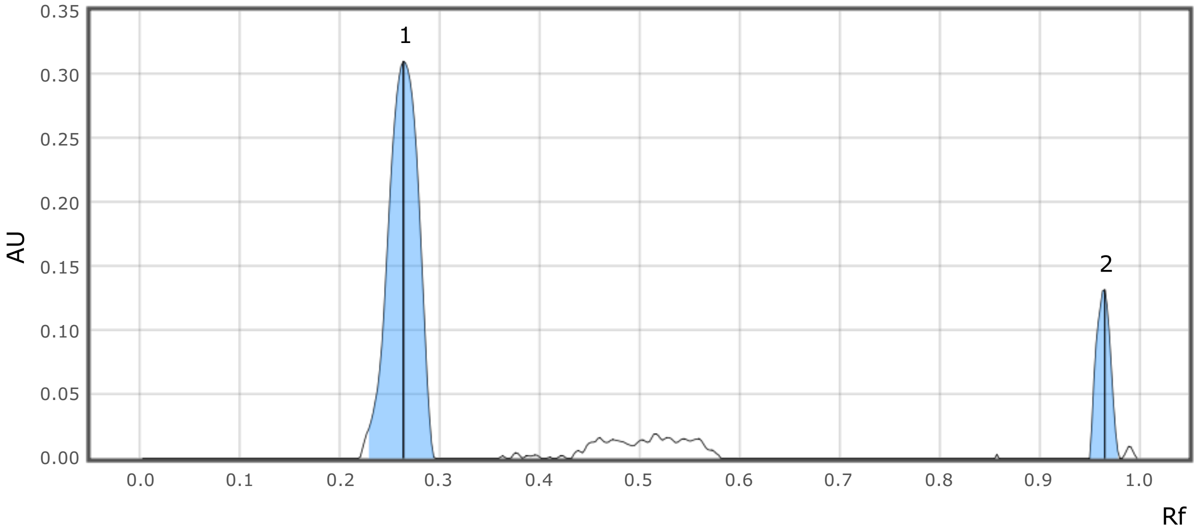

Peak #	Start		Max			End		Area		Manual peak	Substance Name
	Rf	H	Rf	H	%	Rf	H	A	%		
1	0.227	0.0195	0.263	0.3103	70.20	0.296	0.0000	0.01107	83.32	Yes	THCA-A
2	0.951	0.0000	0.966	0.1317	29.80	0.981	0.0000	0.00222	16.68	No	

Track 12:

Type	Reference
Vial ID	CBDA 100
Description	200ng
Volume	2.0 µl

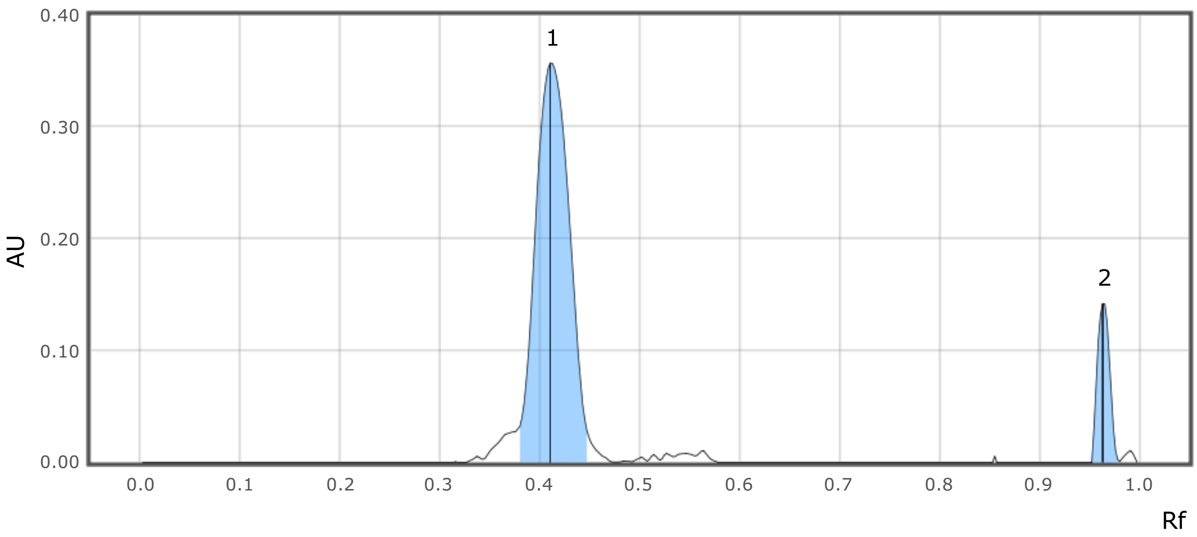

Peak #	Start		Max			End		Area		Manual peak	Substance Name
	Rf	H	Rf	H	%	Rf	H	A	%		
1	0.379	0.0323	0.410	0.3570	71.55	0.448	0.0222	0.01396	87.15	Yes	CBDA
2	0.953	0.0000	0.964	0.1420	28.45	0.981	0.0011	0.00206	12.85	No	

Track 13:

Type	Reference
Vial ID	CBGA 100
Description	200ng
Volume	2.0 µl

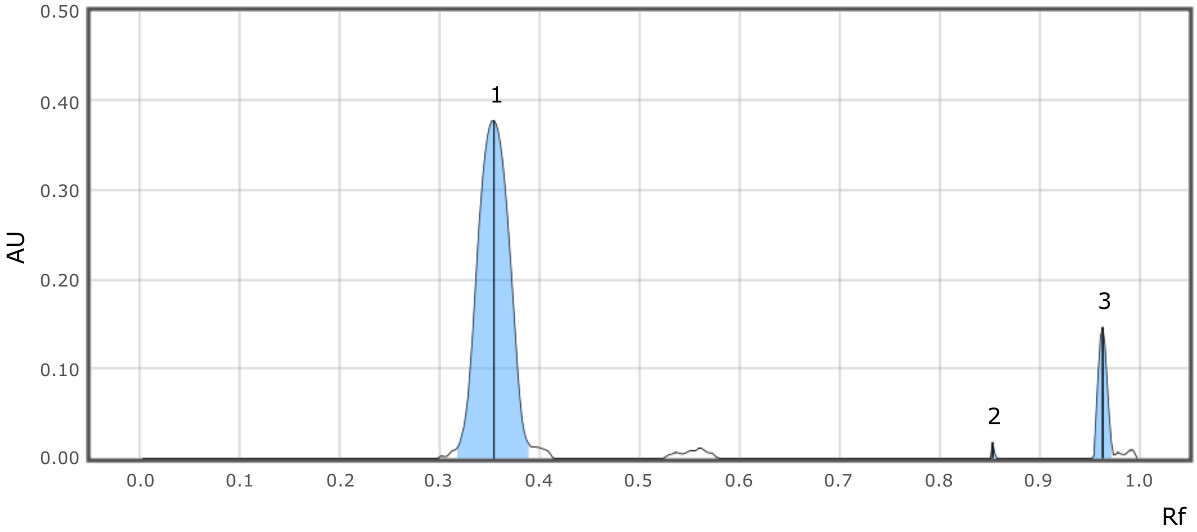

Peak #	Start Rf	Start H	Max Rf	Max H	Max %	End Rf	End H	Area A	Area %	Manual peak	Substance Name
1	0.317	0.0120	0.354	0.3775	69.63	0.389	0.0166	0.01416	89.82	Yes	CBGA
2	0.851	0.0000	0.853	0.0180	3.32	0.858	0.0000	0.00005	0.32	No	
3	0.953	0.0000	0.964	0.1467	27.05	0.974	0.0038	0.00155	9.86	No	

Calibration results:

Height calibration for substance 8-THC @ RT White:

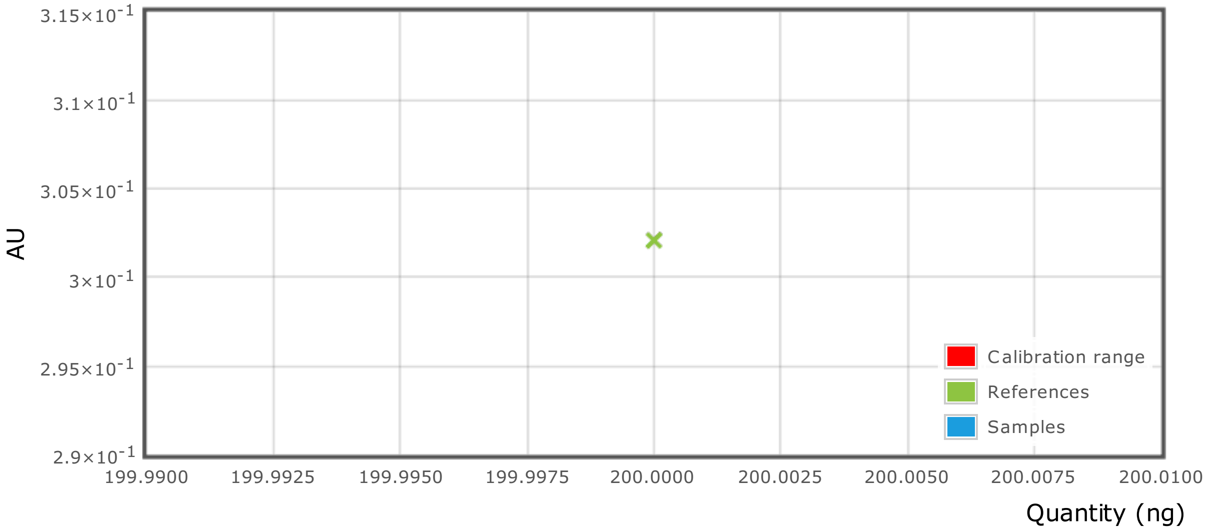

Regression mode	Linear-2
Range deviation	5.00 %
Related substances	Default
Number of references	1
Calibration function	$y=0x$
Coefficient of variation	CV 0.00 %
Correlation coefficient	n/a
⚠	Unable to compute the results for this substance because there wasn't enough groups of references replicas (at least 1 for Linear-1, 2 for Linear2 and Mime-1 and 3 for Polynomial and MiMe-2)

Height calibration for substance 9-THC @ RT White:

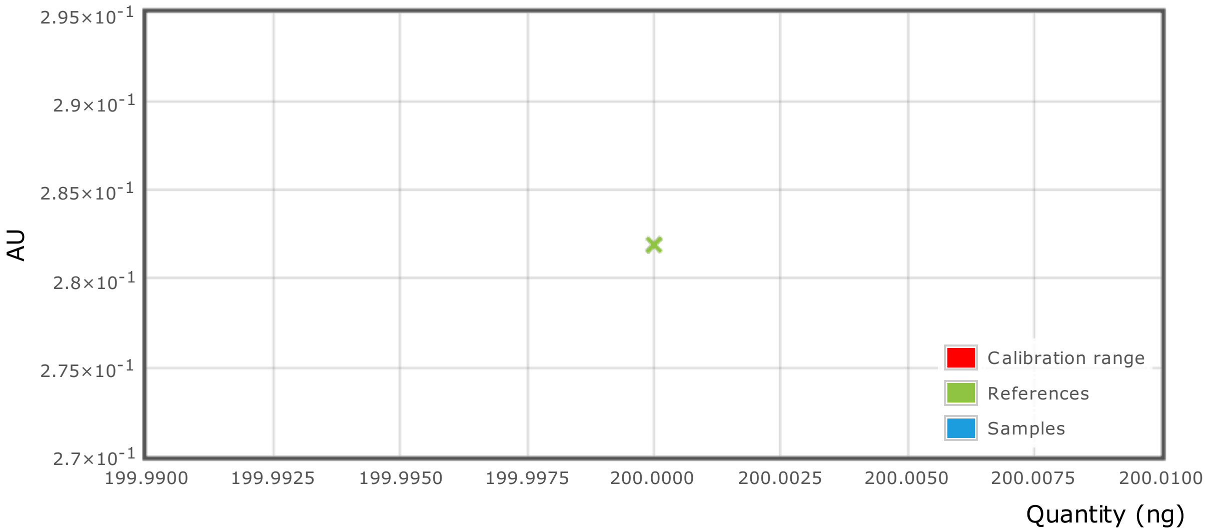

Regression mode	Linear-2
Range deviation	5.00 %
Related substances	Default
Number of references	1
Calibration function	$y=0x$
Coefficient of variation	CV 0.00 %
Correlation coefficient	n/a
⚠	Unable to compute the results for this substance because there wasn't enough groups of references replicas (at least 1 for Linear-1, 2 for Linear2 and Mime-1 and 3 for Polynomial and MiMe-2)

Height calibration for substance CBC @ RT White:

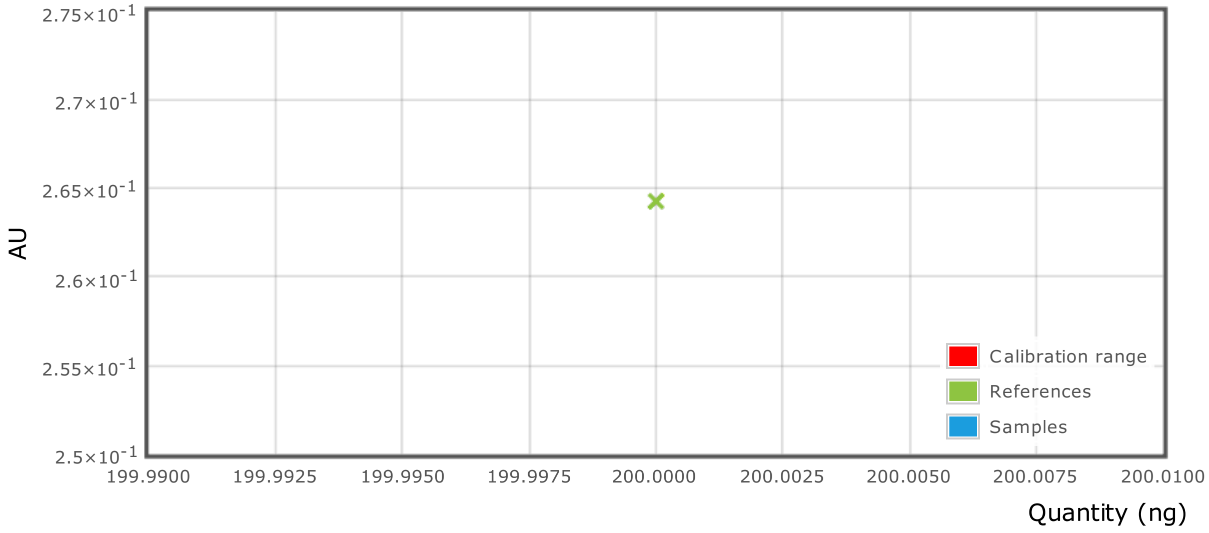

Regression mode	Linear-2
Range deviation	5.00 %
Related substances	Default
Number of references	1
Calibration function	$y=0x$
Coefficient of variation	CV 0.00 %
Correlation coefficient	n/a
⚠	Unable to compute the results for this substance because there wasn't enough groups of references replicas (at least 1 for Linear-1, 2 for Linear2 and Mime-1 and 3 for Polynomial and MiMe-2)

Height calibration for substance CBD @ RT White:

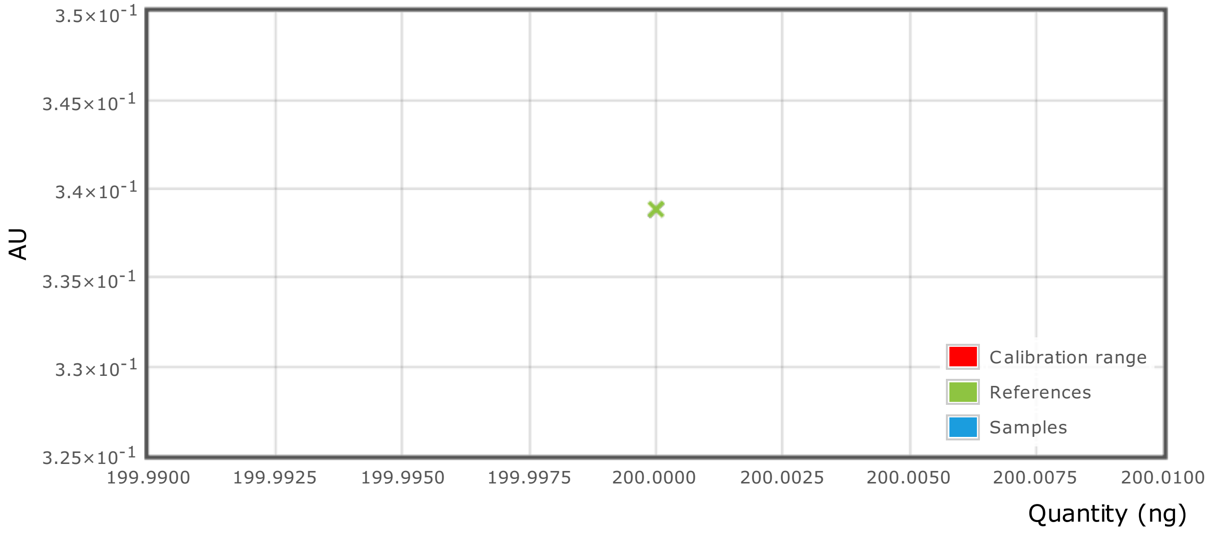

Regression mode	Linear-2
Range deviation	5.00 %
Related substances	Default
Number of references	1
Calibration function	$y=0x$
Coefficient of variation	CV 0.00 %
Correlation coefficient	n/a
⚠	Unable to compute the results for this substance because there wasn't enough groups of references replicas (at least 1 for Linear-1, 2 for Linear2 and Mime-1 and 3 for Polynomial and MiMe-2)

Height calibration for substance CBDA @ RT White:

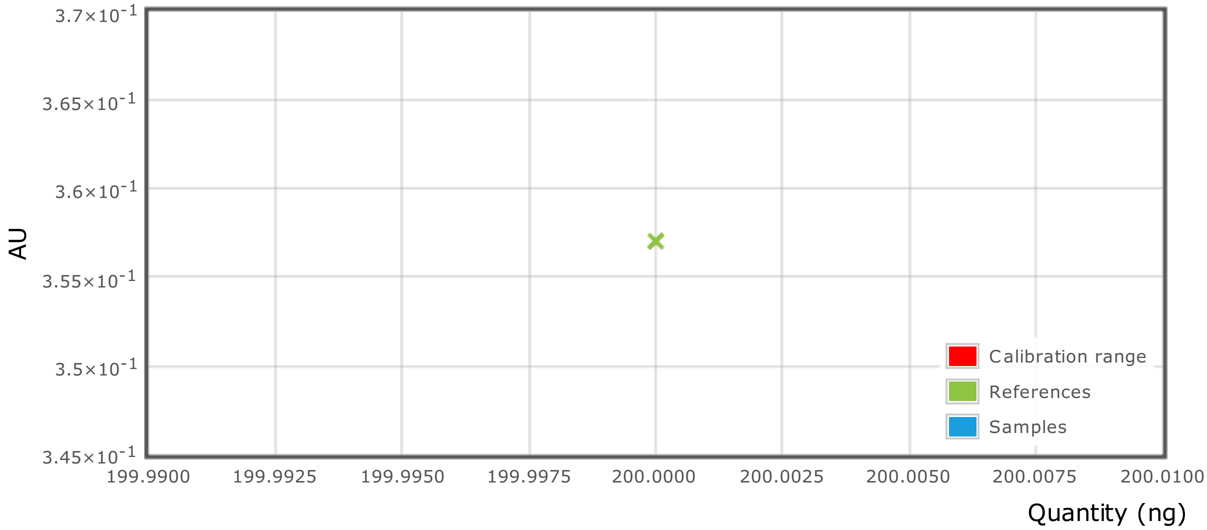

Regression mode	Linear-2
Range deviation	5.00 %
Related substances	Default
Number of references	1
Calibration function	$y=0x$
Coefficient of variation	CV 0.00 %
Correlation coefficient	n/a
⚠	Unable to compute the results for this substance because there wasn't enough groups of references replicas (at least 1 for Linear-1, 2 for Linear2 and Mime-1 and 3 for Polynomial and MiMe-2)

Height calibration for substance CBDV @ RT White:

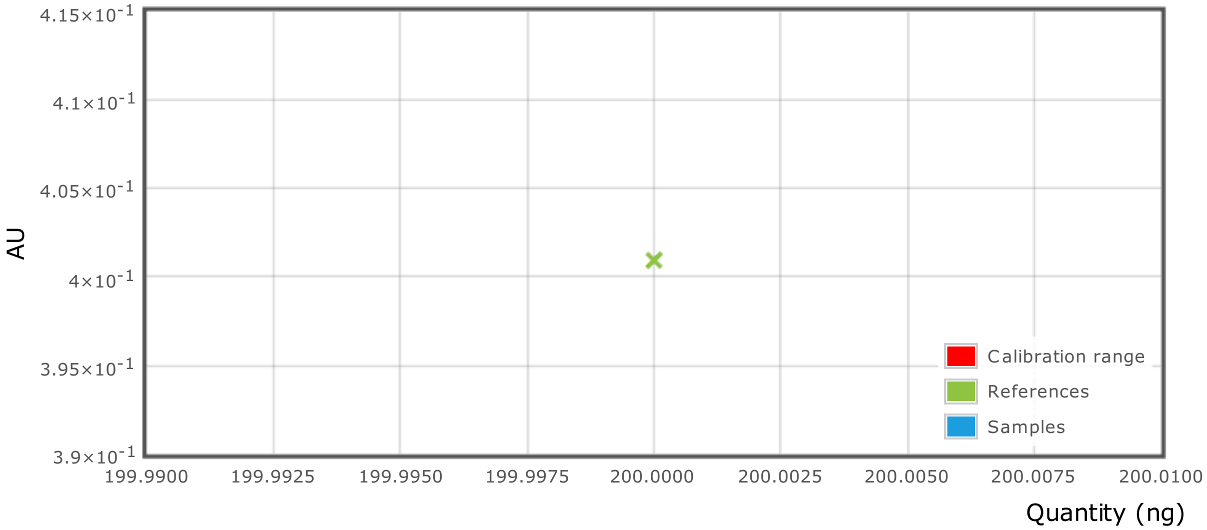

Regression mode	Linear-2
Range deviation	5.00 %
Related substances	Default
Number of references	1
Calibration function	$y=0x$
Coefficient of variation	CV 0.00 %
Correlation coefficient	n/a
⚠	Unable to compute the results for this substance because there wasn't enough groups of references replicas (at least 1 for Linear-1, 2 for Linear2 and Mime-1 and 3 for Polynomial and MiMe-2)

Height calibration for substance CBG @ RT White:

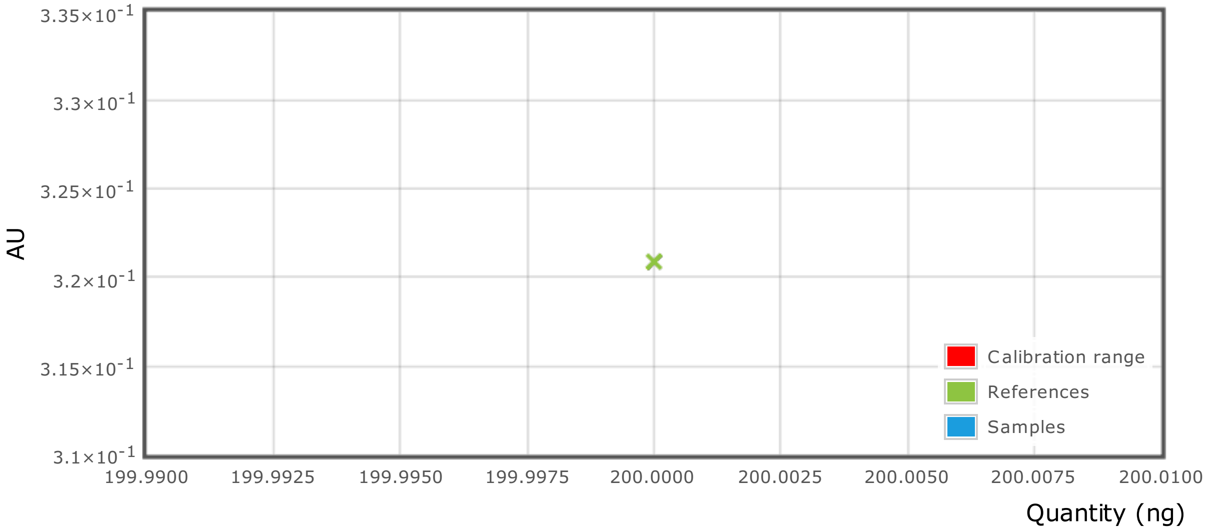

Regression mode	Linear-2
Range deviation	5.00 %
Related substances	Default
Number of references	1
Calibration function	$y=0x$
Coefficient of variation	CV 0.00 %
Correlation coefficient	n/a
⚠	Unable to compute the results for this substance because there wasn't enough groups of references replicas (at least 1 for Linear-1, 2 for Linear2 and Mime-1 and 3 for Polynomial and MiMe-2)

Height calibration for substance CBGA @ RT White:

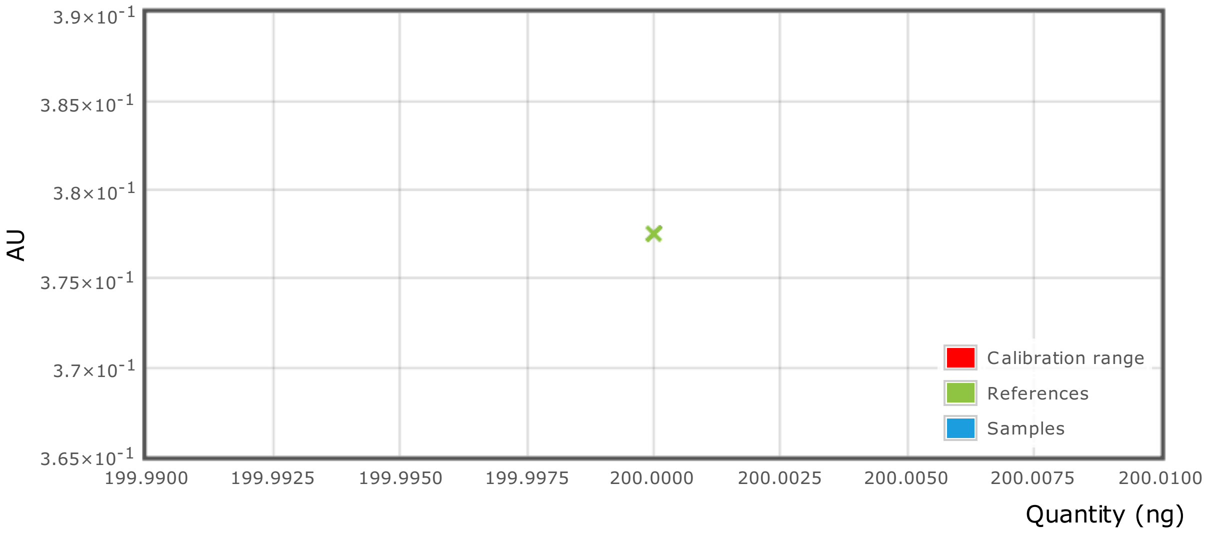

Regression mode	Linear-2
Range deviation	5.00 %
Related substances	Default
Number of references	1
Calibration function	$y=0x$
Coefficient of variation	CV 0.00 %
Correlation coefficient	n/a
⚠	Unable to compute the results for this substance because there wasn't enough groups of references replicas (at least 1 for Linear-1, 2 for Linear2 and Mime-1 and 3 for Polynomial and MiMe-2)

Height calibration for substance CBN @ RT White:

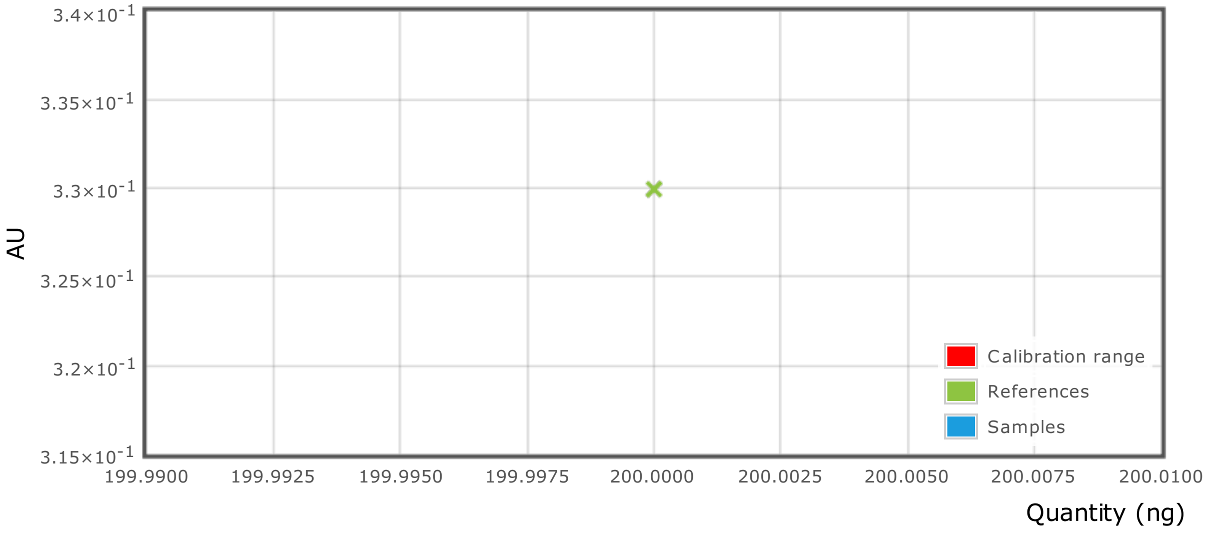

Regression mode	Linear-2
Range deviation	5.00 %
Related substances	Default
Number of references	1
Calibration function	$y=0x$
Coefficient of variation	CV 0.00 %
Correlation coefficient	n/a
⚠	Unable to compute the results for this substance because there wasn't eno ugh groups of references replicas (at least 1 for Linear-1, 2 for Linear2 and Mime-1 and 3 for Polynomial and MiMe-2)

Height calibration for substance THCA-A @ RT White:

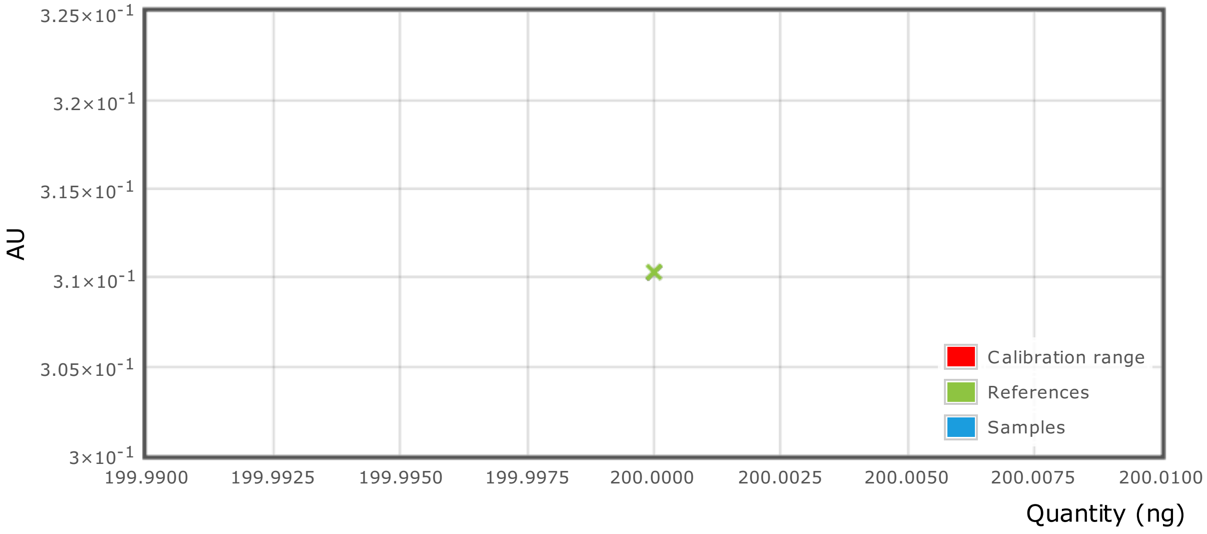

Regression mode	Linear-2
Range deviation	5.00 %
Related substances	Default
Number of references	1
Calibration function	$y=0x$
Coefficient of variation	CV 0.00 %
Correlation coefficient	n/a
⚠	Unable to compute the results for this substance because there wasn't enough groups of references replicas (at least 1 for Linear-1, 2 for Linear2 and Mime-1 and 3 for Polynomial and MiMe-2)

Height calibration for substance THCV @ RT White:

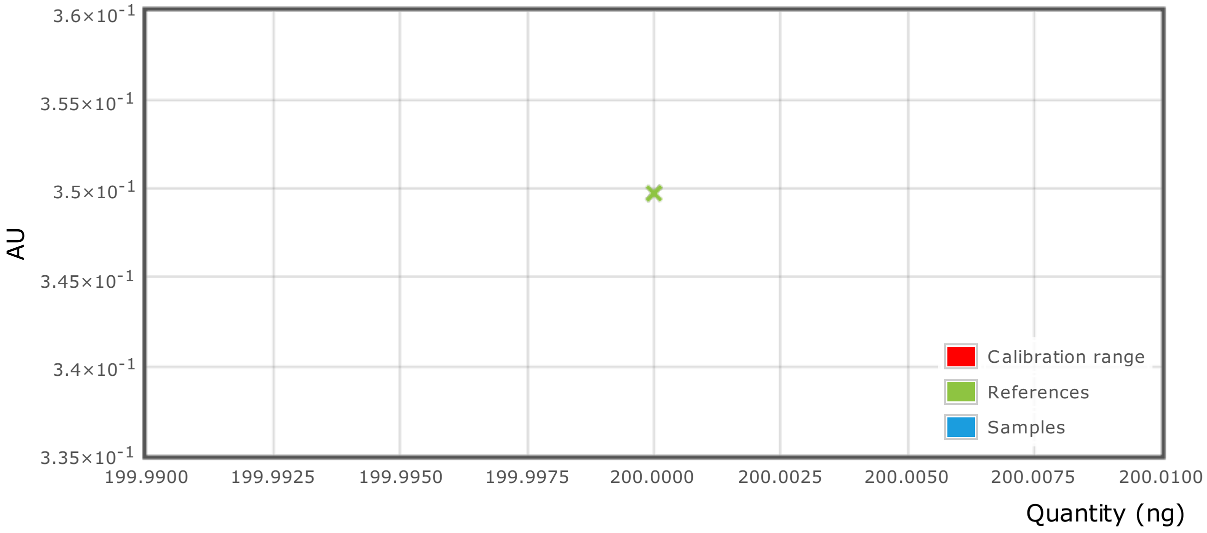

Regression mode	Linear-2
Range deviation	5.00 %
Related substances	Default
Number of references	1
Calibration function	$y=0x$
Coefficient of variation	CV 0.00 %
Correlation coefficient	n/a
⚠	Unable to compute the results for this substance because there wasn't enough groups of references replicas (at least 1 for Linear-1, 2 for Linear2 and Mime-1 and 3 for Polynomial and MiMe-2)

Results:

Substance having no available results		
⚠	8-THC	There wasn't any sample application available in the assignments for this substance. Please check that the peaks were correctly detected and assigned for this substance.
⚠	9-THC	There wasn't any sample application available in the assignments for this substance. Please check that the peaks were correctly detected and assigned for this substance.
⚠	CBD	There wasn't any sample application available in the assignments for this substance. Please check that the peaks were correctly detected and assigned for this substance.
⚠	CBC	There wasn't any sample application available in the assignments for this substance. Please check that the peaks were correctly detected and assigned for this substance.
⚠	CBDA	There wasn't any sample application available in the assignments for this substance. Please check that the peaks were correctly detected and assigned for this substance.
⚠	CBGA	There wasn't any sample application available in the assignments for this substance. Please check that the peaks were correctly detected and assigned for this substance.
⚠	CBN	There wasn't any sample application available in the assignments for this substance. Please check that the peaks were correctly detected and assigned for this substance.
⚠	THCA-A	There wasn't any sample application available in the assignments for this substance. Please check that the peaks were correctly detected and assigned for this substance.
⚠	CBDV	There wasn't any sample application available in the assignments for this substance. Please check that the peaks were correctly detected and assigned for this substance.
⚠	CBG	There wasn't any sample application available in the assignments for this substance. Please check that the peaks were correctly detected and assigned for this substance.
⚠	THCV	There wasn't any sample application available in the assignments for this substance. Please check that the peaks were correctly detected and assigned for this substance.

A track marked with ⚠ means: this result is outside the regression range given by the reference assignments, but is included in the results because it is in the allowed range deviation.

Analyst:	Reviewer:
