# Supplementary material for: High Performance Thin-Layer Chromatography (HPTLC) data of Cannabinoids in ten mobile phase systems
Source: Data Brief. 2020 Jun 30;31:105955. doi: 10.1016/j.dib.2020.105955 (PMC7352075; doi:10.1016/j.dib.2020.105955)
Supplement: Supplementary file 1 [file mmc1.zip › S1-Triplicate reports/Toluene-1.pdf]

World Leader in Planar Chromatography

Analysis: YL-toluene-1

Path: Home/YL Research

Based on method: Triplets Method

Created	13-May-2019 13:17:12	visionCATSuser
Modified	28-May-2019 16:25:31	visionCATSuser
Last HPTLC log	28-May-2019 16:25:31	Analysis modified
Explorer notes		

Track	Vial ID	Description	Volume	Position	Type
1	MeOH blank	MeOH Blank	2.0 µl	A1	Sample
2	Mixture 100	Mixture	2.0 µl	A2	Sample
3	9-THC 100	D9-THC	2.0 µl	A3	Reference
4	CBD 100	CBD	2.0 µl	A4	Reference
5	CBN 100	CBN	2.0 µl	A5	Reference
6	CBG 100	CBG	2.0 µl	A6	Reference
7	CBC 100	CBC	2.0 µl	A7	Reference
8	THCV 100	THCV	2.0 µl	A8	Reference
9	CBDV 100	CBDV	2.0 µl	A9	Reference
10	8-THC 100	D8-THC	2.0 µl	A10	Reference
11	THCA-A 100	THCA-A	2.0 µl	A11	Reference
12	CBDA 100	CBDA	2.0 µl	B1	Reference
13	CBGA 100	CBGA	2.0 µl	B2	Reference
14	Mixture 100	Mixture	2.0 µl	A2	Sample
15	MeOH blank	MeOH Blank	2.0 µl	A1	Sample
Sequence table notes					

A track marked with ⚠ means: the application type is overridden in some evaluation(s).

System setup:

Software	Server User-PC, version 2.5.18072.1
ATS4	S/N:080713
Chamber	N/A
Derivatization dip	N/A
Scanner3	S/N:031025
Visualizer	S/N:230515

Chromatography

Plate layout:

Stationary phase	Merck, HPTLC plates silica gel 60 F 254
Plate format	200.0 x 100.0 mm
Application type	Band
Application	Position Y: 8.0 mm, length: 8.0 mm, width: 0.0 mm
Track	First position X: 20.0 mm, distance: 11.4 mm
Solvent front position	70.0 mm
Notes	

Take image clean plate 1a - Visualizer (S/N: 230515):

Quality	Enhanced
RT White	auto capture, Auto, level 85 %, Band
R 254	auto capture, Auto, level 85 %, Band
Instrument diagnostics	Valid diagnostics
Documentation step label	
Notes	

Application 1 - ATS 4 (S/N: 080713):

Spray gas	Air
Sample solvent type	Methanol
Filling speed	15 µl/s
Predosage volume	200 nl
Retraction volume	200 nl
Dosage speed	150 nl/s
Filling quality	Standard
Rinsing cycles / vacuum	1 / 4 s
Filling cycles / vacuum	1 / 4 s
Rinsing solvent name	Methanol
Nozzle temperature	Unheated
Rack in use	Standard
Instrument diagnostics	Valid diagnostics
Notes	

Development 1 - Chamber:

Tank	TTC 20x10
Mobile phase	
Saturation time	20 min
Use saturation pad	true
Use smartALERT	false
Volume front through	10 ml
Volume rear through	20 ml
Drying time	5 min
Drying temperature	Room temperature
Notes	

Take image developed plate 1a - Visualizer (S/N: 230515):

Quality	Enhanced
RT White	auto capture, Auto, level 85 %, Band
R 254	auto capture, Auto, level 85 %, Band
R 366	auto capture, Auto, level 85 %, Band
Instrument diagnostics	Valid diagnostics
Documentation step label	
Notes	

Scan developed plate 1b - Scanner 3 (S/N: 031025):

Scanner type	Single λ
Optimization for	Resolution
Measurement mode	Absorption
Filter	n/a
Detector mode	Automatic
Scanning speed	20 mm/s
Data resolution	100 µm/step
Slit	5 x 0.2 mm, micro
Partial scan	No
Lamp	Deuterium & Tungsten
Wavelength(s)	254 nm
Instrument diagnostics	Valid diagnostics
Documentation step label	
Notes	

Derivatization 1 - dip:

Reagent name	
Dipping speed	5
Dipping time	0 s
Reagent preparation	
Heating	100 °C for 3 min, heated after
Notes	

Take image derivatized plate 1a - Visualizer (S/N: 230515):

Quality	Enhanced
RT White	auto capture, Auto, level 85 %, Band
R 366	auto capture, Auto, level 85 %, Band
Instrument diagnostics	Valid diagnostics
Documentation step label	
Notes	

Take image derivatized plate 1b - Visualizer (S/N: 230515):

Quality	Enhanced
RT White	auto capture, Auto, level 85 %, Band
R 366	auto capture, Auto, level 85 %, Band
Instrument diagnostics	Valid diagnostics
Documentation step label	
Notes	

System suitability tests:

SST settings:

SST tracks	

Data acquisition

Application 1 - ATS 4 (S/N: 080713):

Executed	13-May-2019 13:21:54 visionCATSuser

Development 1 - Chamber:

Executed	13-May-2019 14:33:02 visionCATSuser

Take image developed plate 1a - Visualizer (S/N: 230515):

Executed	13-May-2019 14:33:05 visionCATSuser

RT White Developed, RemTransVis

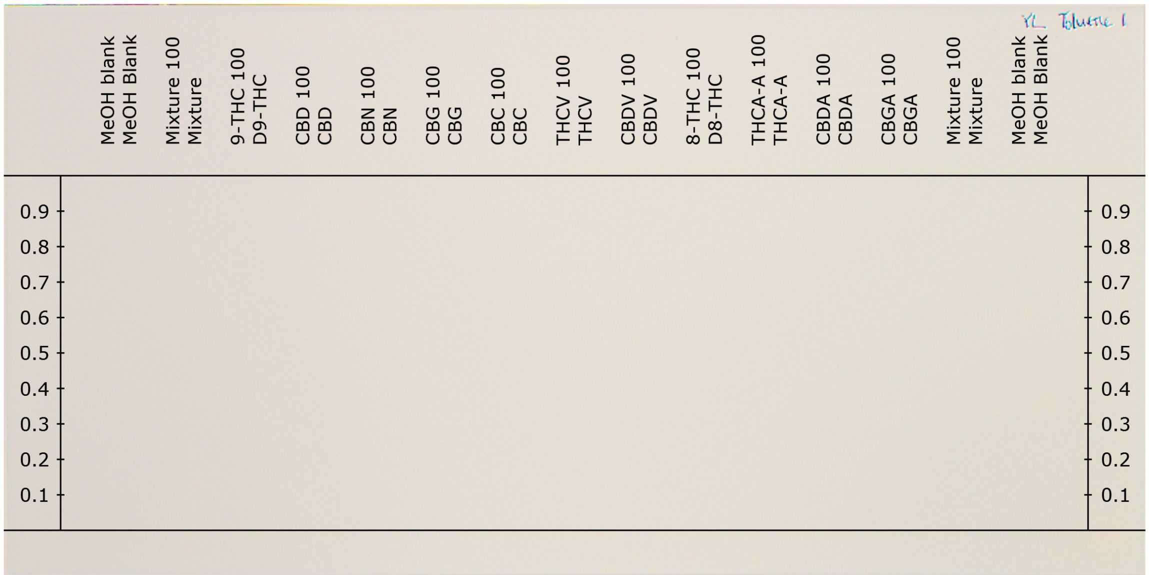

Exposure	0.081 s
Contrast	1
Normalized exposure	Disabled
Clarify	Disabled
White balance	1.00, 1.00, 1.00

R 254 Developed, Remission254

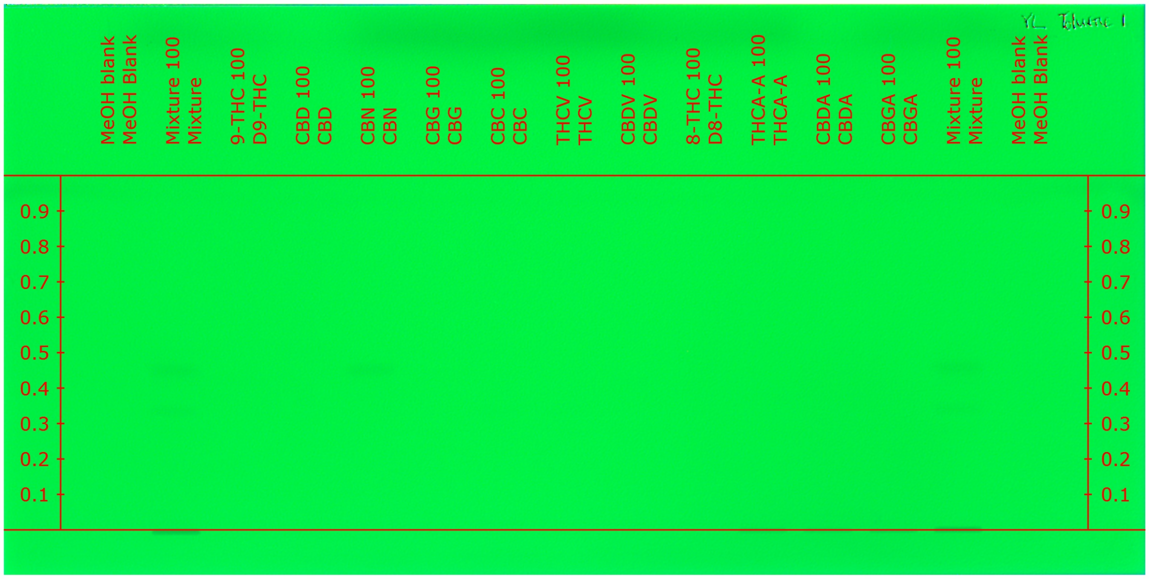

Exposure	0.290 s
Contrast	1
Normalized exposure	Disabled
Clarify	Disabled
White balance	1.00, 1.00, 1.00

R 366 Developed, Remission366

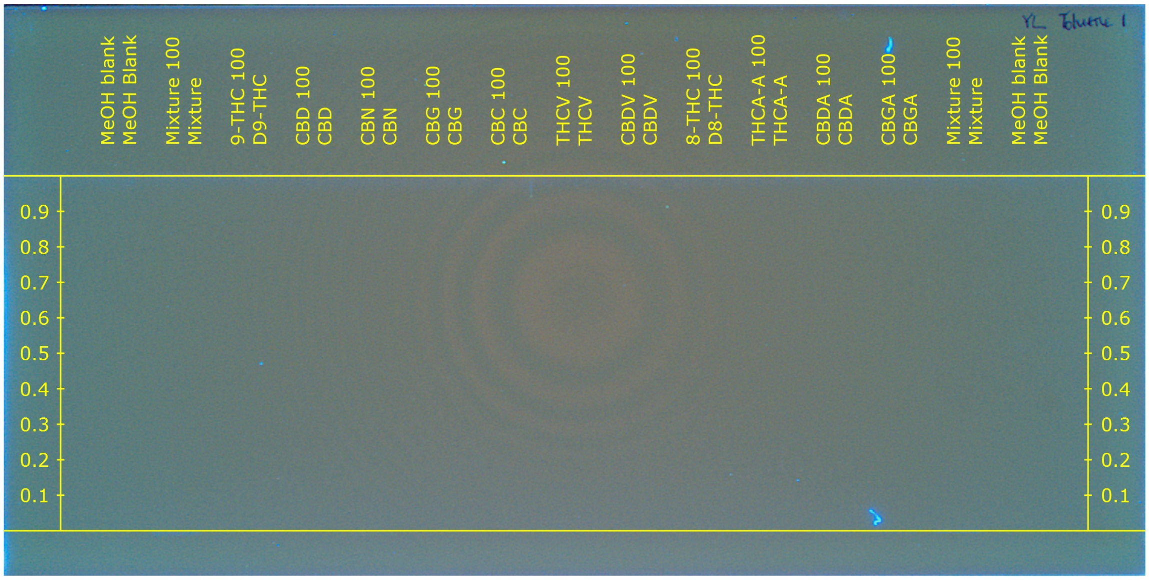

Exposure	9.999 s
Contrast	1
Normalized exposure	Disabled
Clarify	Disabled
White balance	1.00, 1.00, 1.00

Scan developed plate 1b - Scanner 3 (S/N: 031025):

Executed	13-May-2019 14:36:24 visionCATSuser

Scan:

Wavelength	254 nm

Track 1:

Type	Single λ

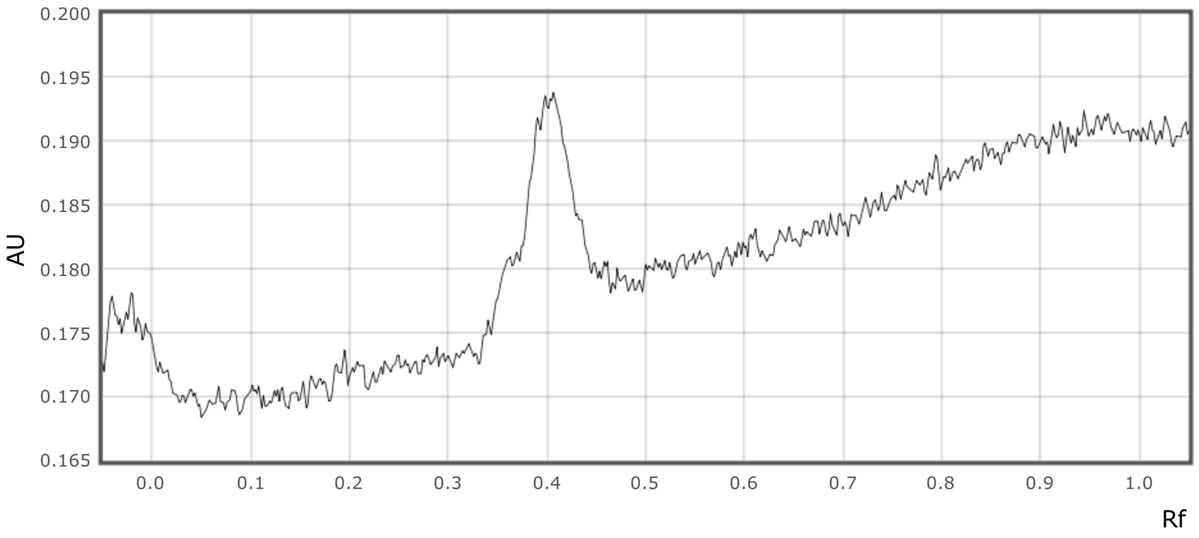

Track 2:

Type	Single λ

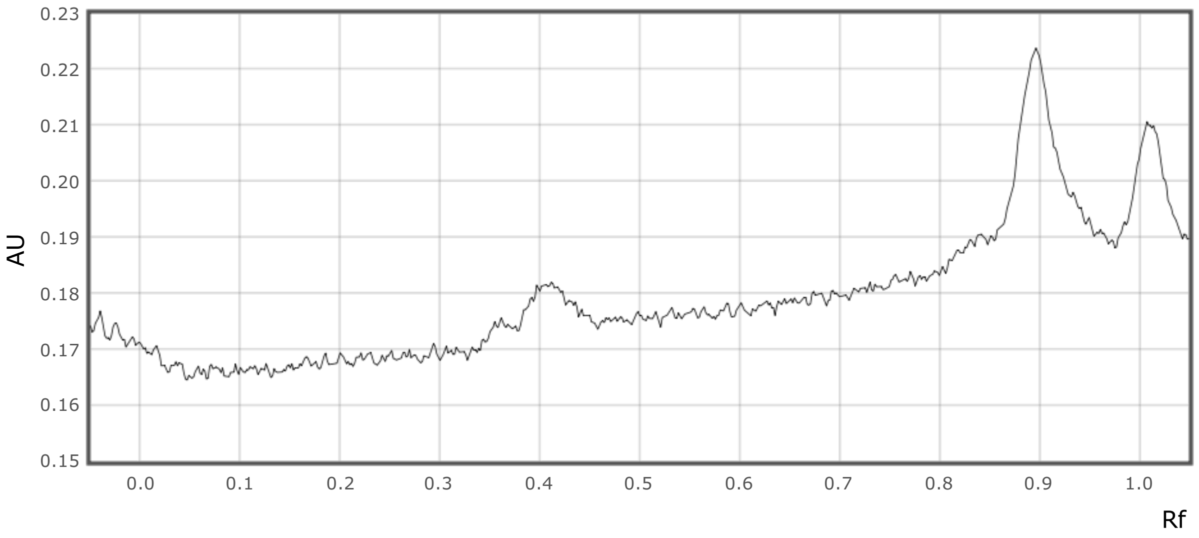

Track 3:

Type	Single λ

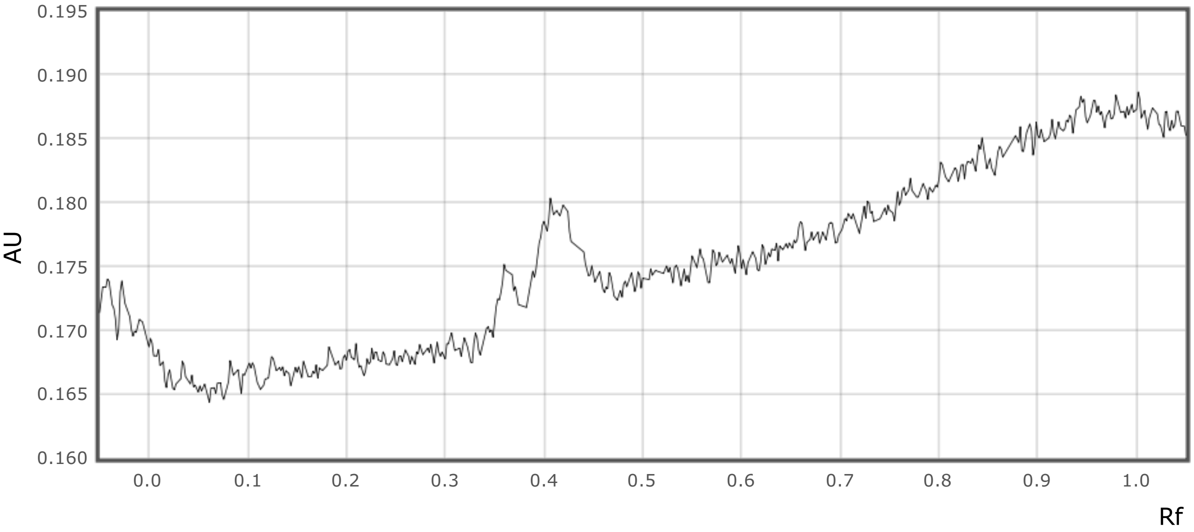

Track 4:

Type	Single λ

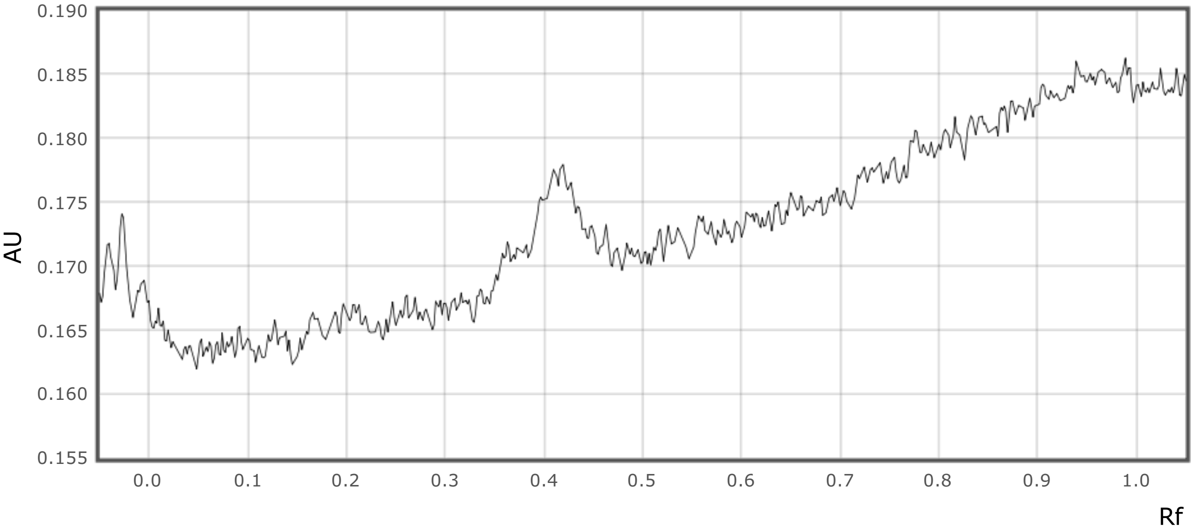

Track 5:

Type	Single λ

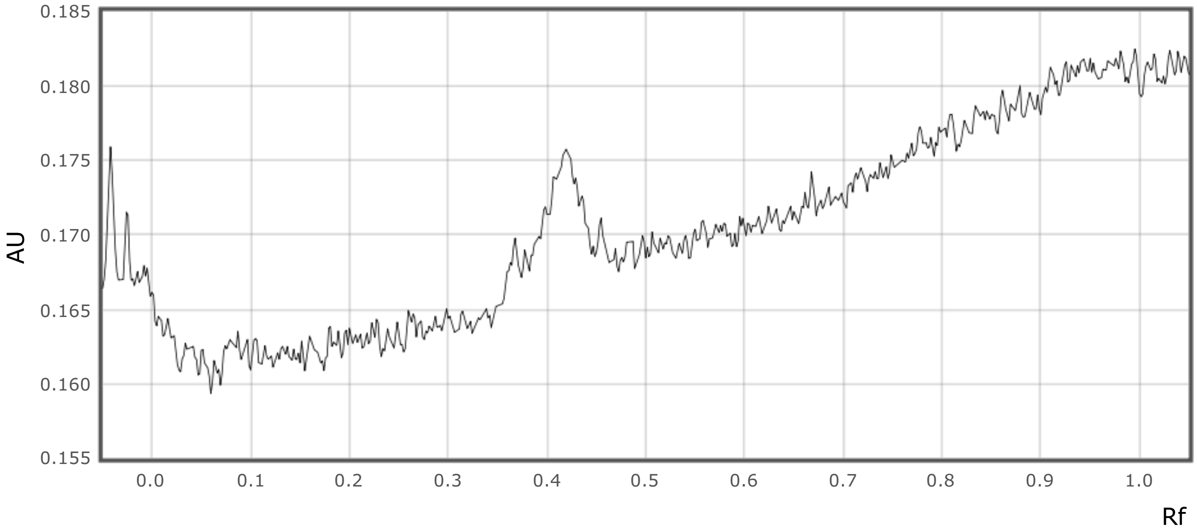

Track 6:	
Type	Single λ

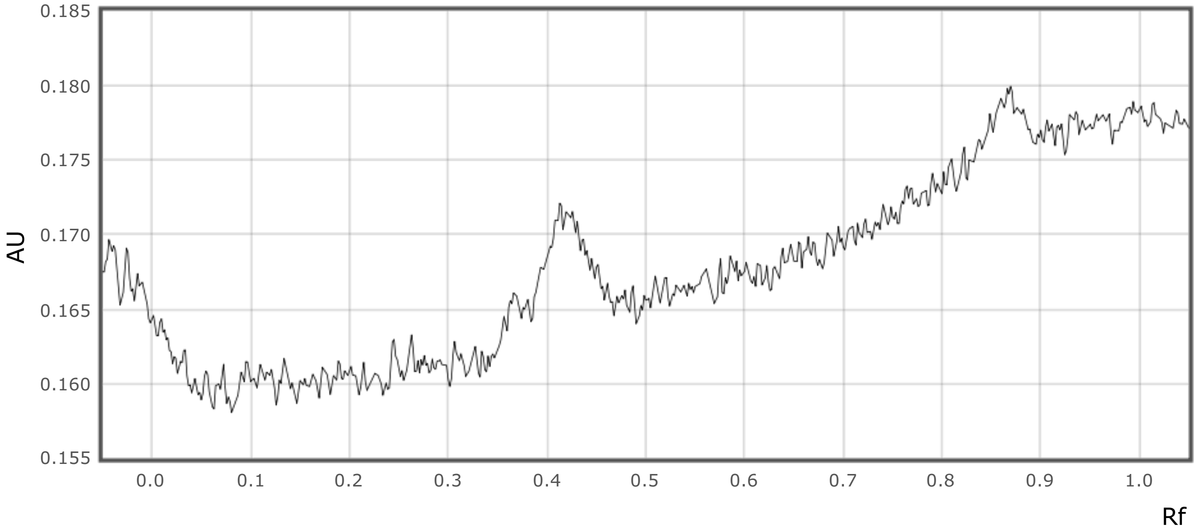

Track 7:	
Type	Single λ

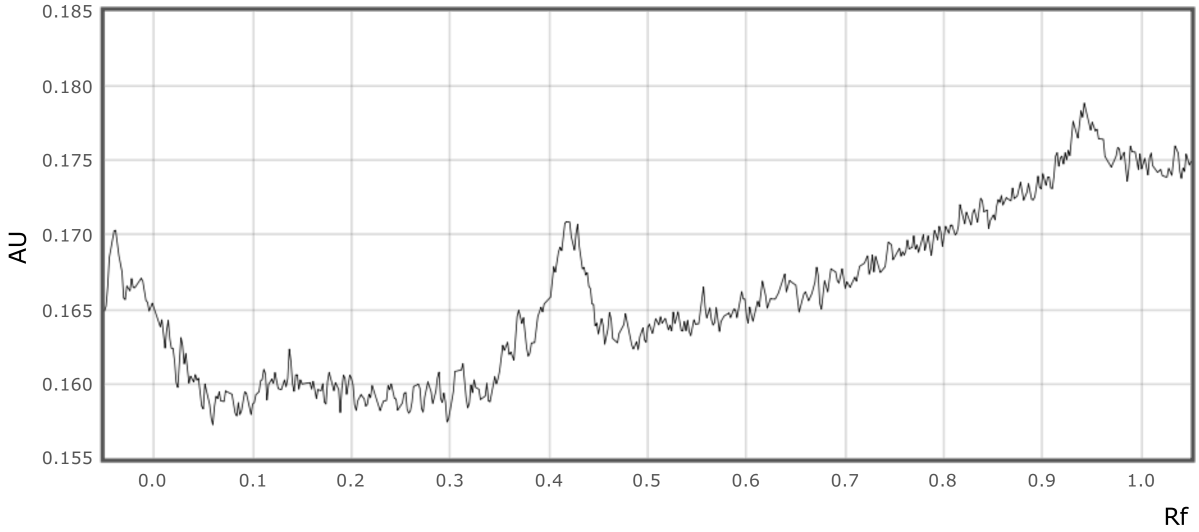

Track 8:	
Type	Single λ

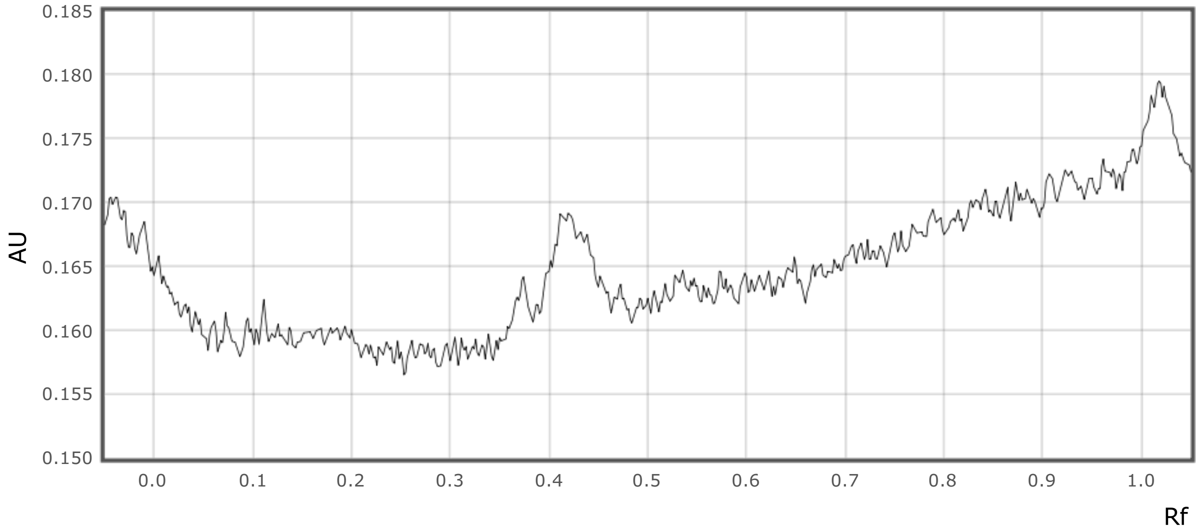

Track 9:	
Type	Single λ

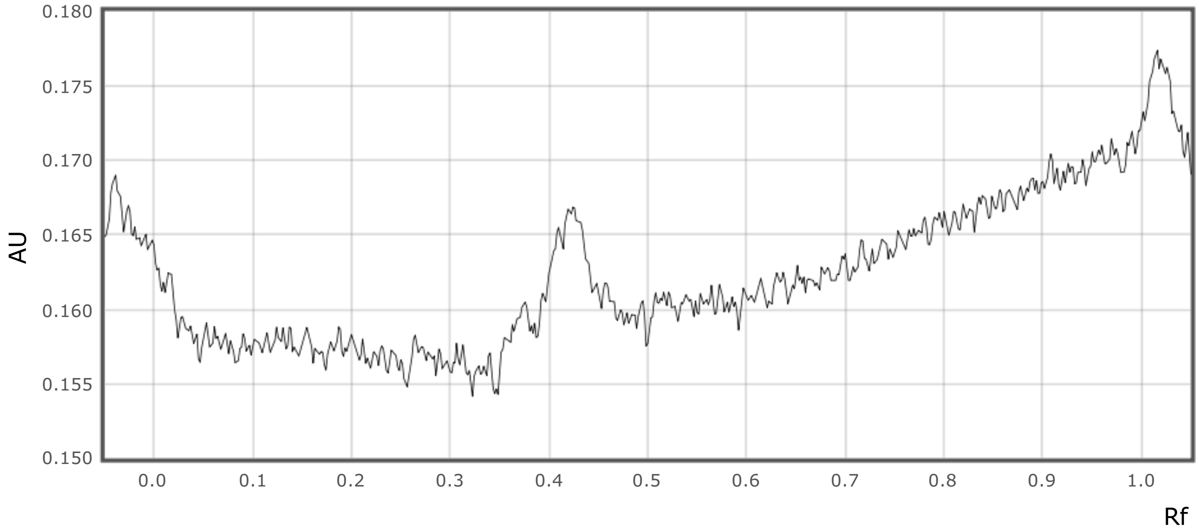

Track 10:

Type	Single λ

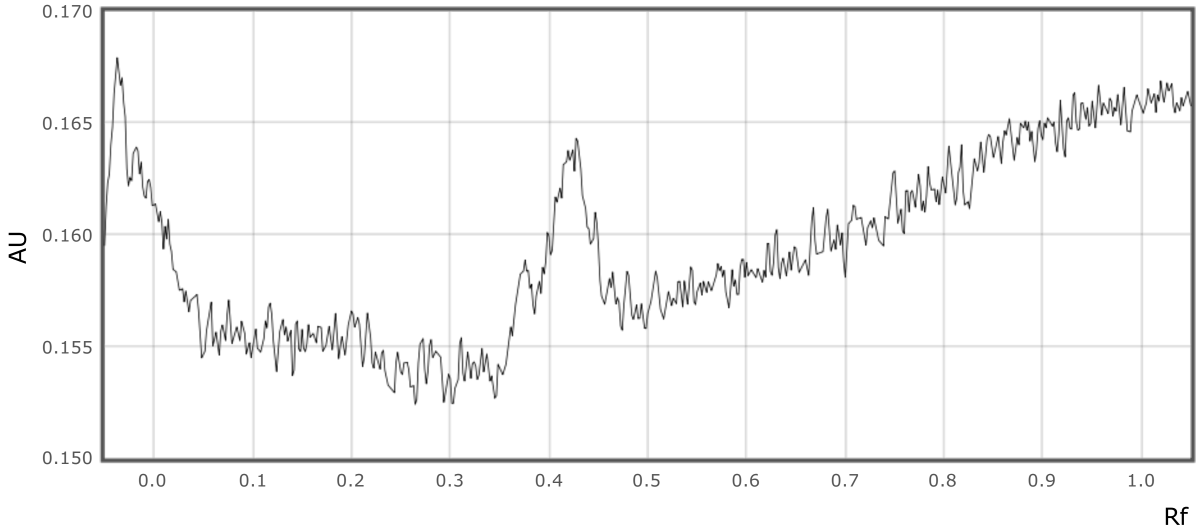

Track 11:

Type	Single λ

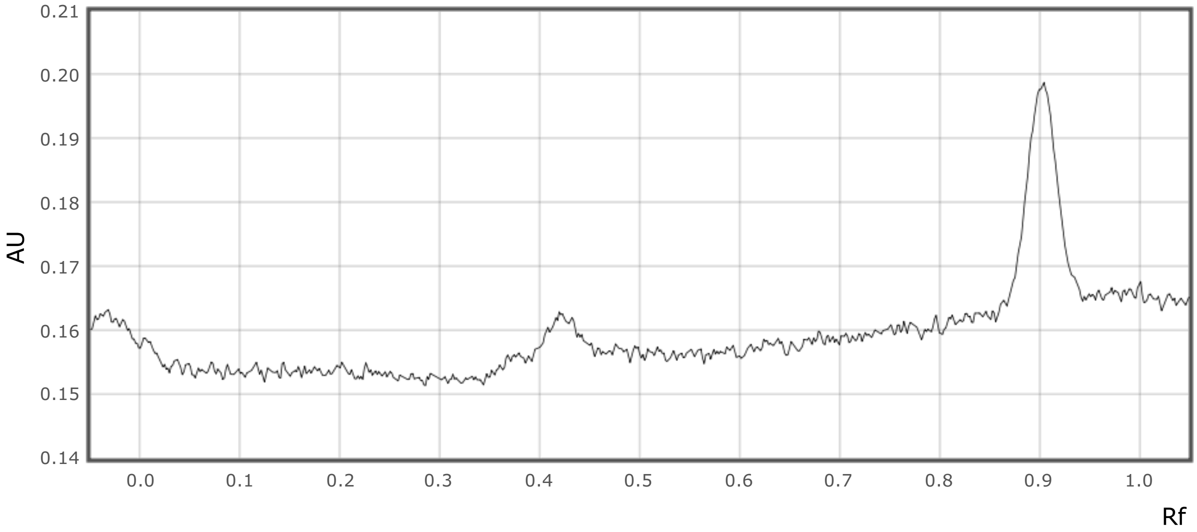

Track 12:	
Type	Single λ

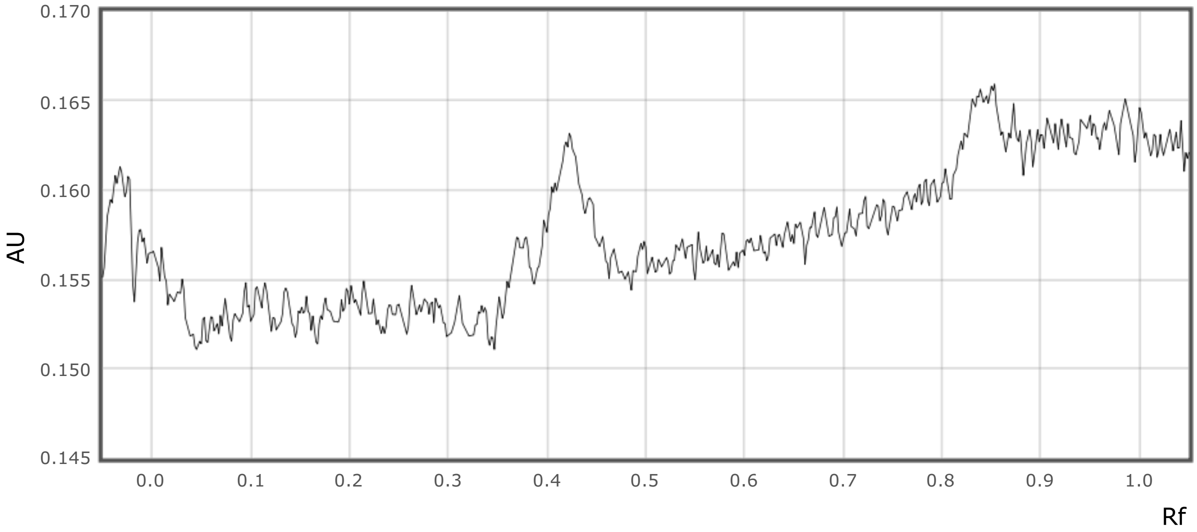

Track 13:	
Type	Single λ

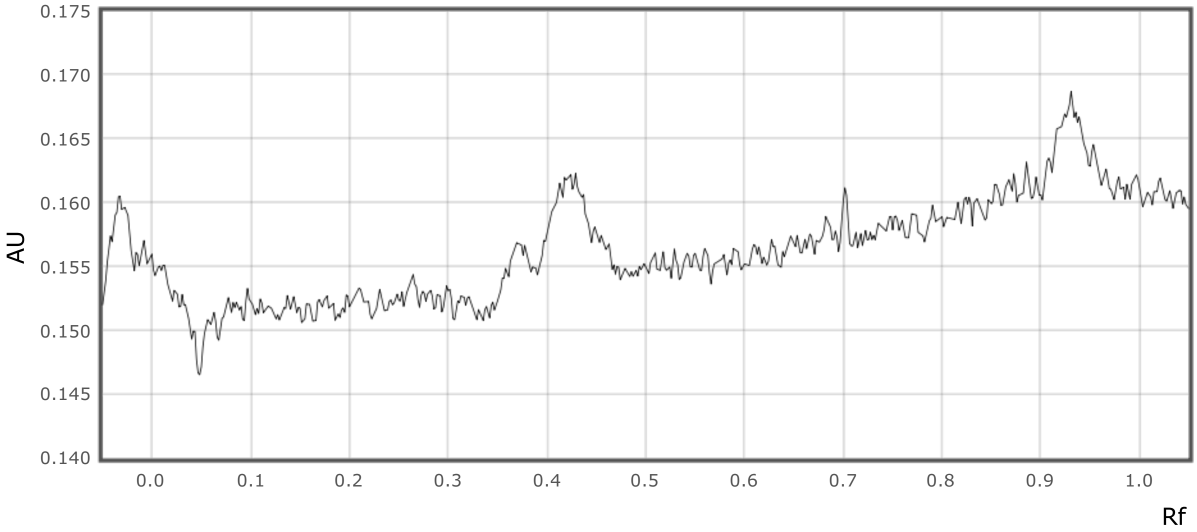

Track 14:	
Type	Single λ

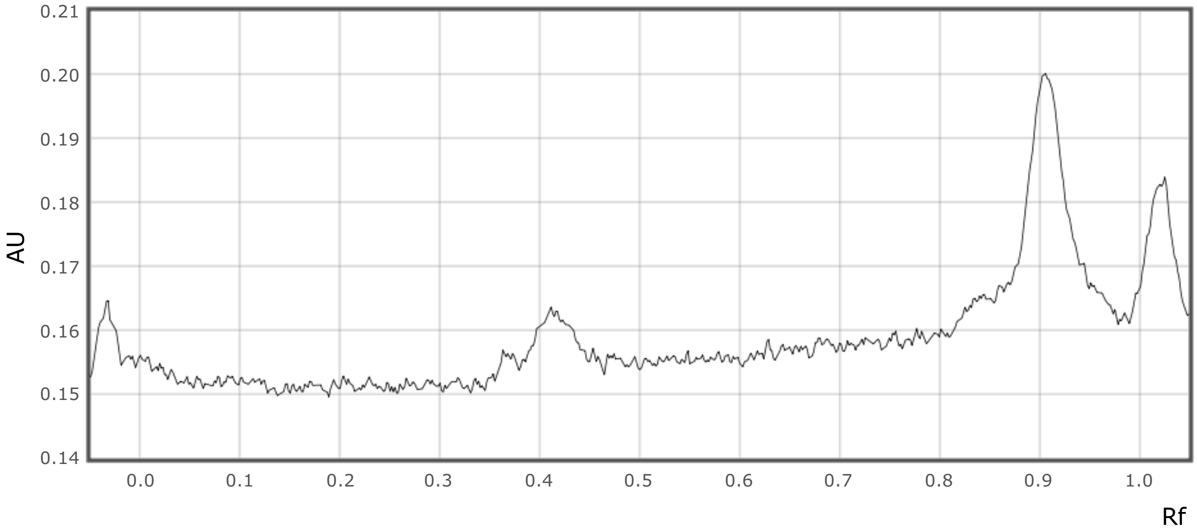

Track 15:	
Type	Single λ

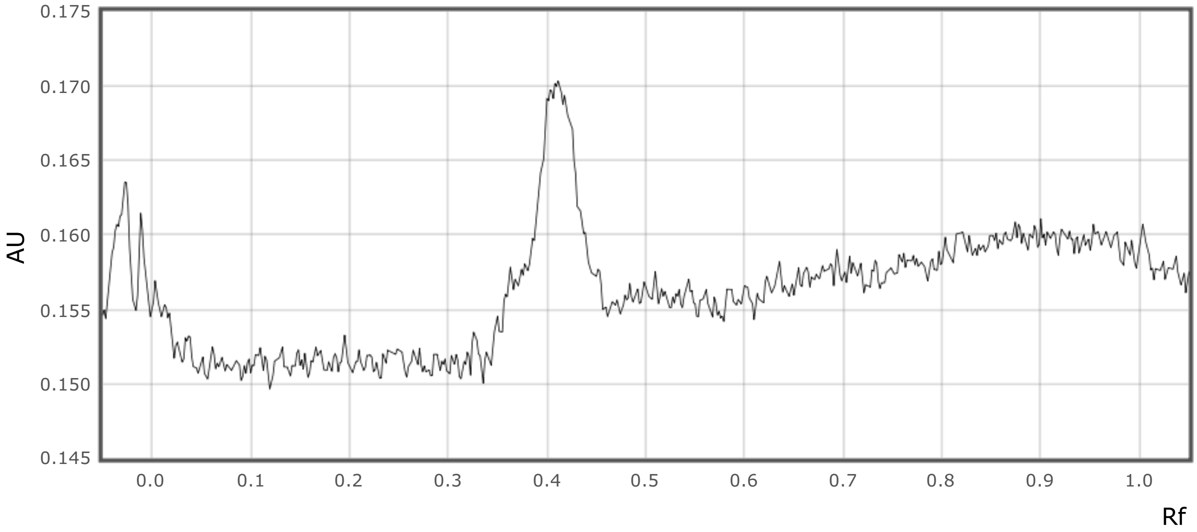

Derivatization 1 - dip:

Executed	13-May-2019 14:43:50 visionCATSuser

Take image derivatized plate 1a - Visualizer (S/N: 230515):

Executed	13-May-2019 14:50:54 visionCATSuser

RT White

Derivatized, RemTransVis

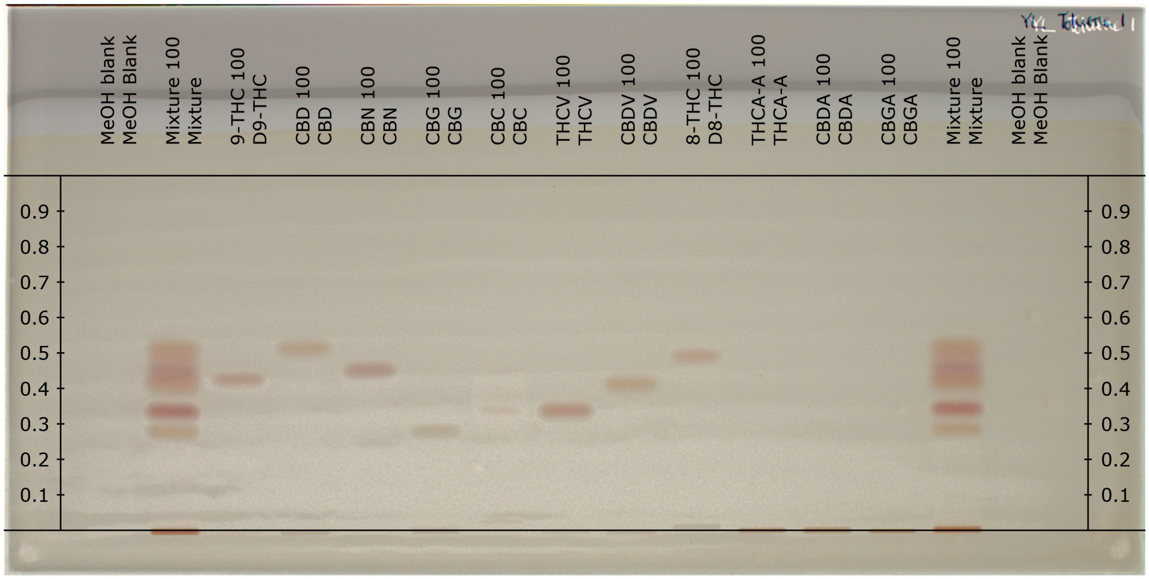

Exposure	0.034 s
Contrast	1
Normalized exposure	Disabled
Clarify	Disabled
White balance	1.00, 1.00, 1.00

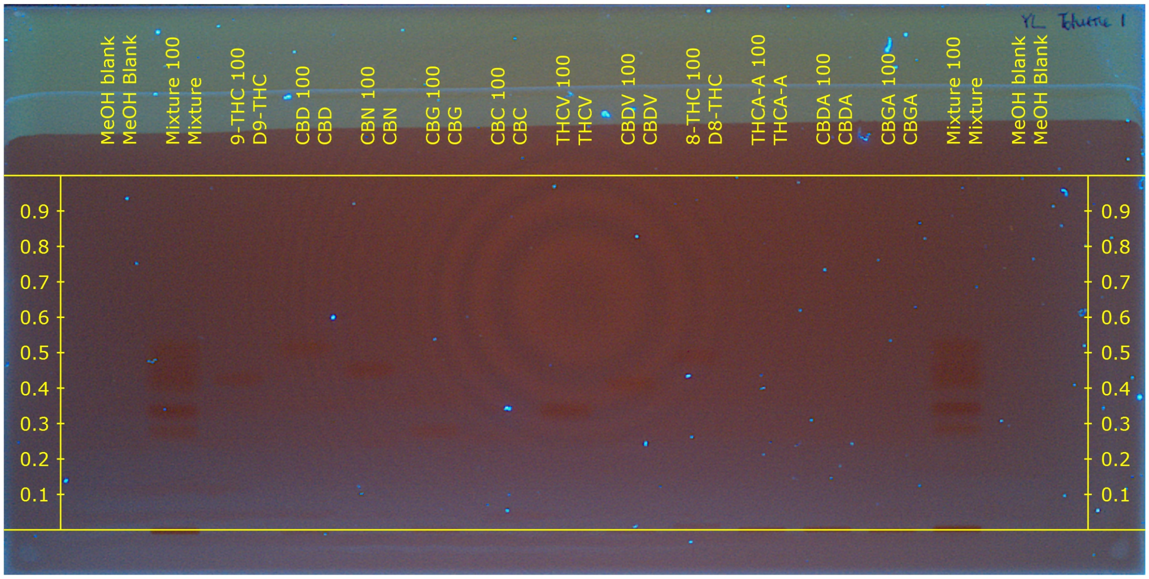

Exposure	9.999 s
Contrast	1
Normalized exposure	Disabled
Clarify	Disabled
White balance	1.00, 1.00, 1.00

Take image derivatized plate 1b - Visualizer (S/N: 230515):

Executed	13-May-2019 15:11:07 visionCATSuser

RT White — Derivatized, RemTransVis

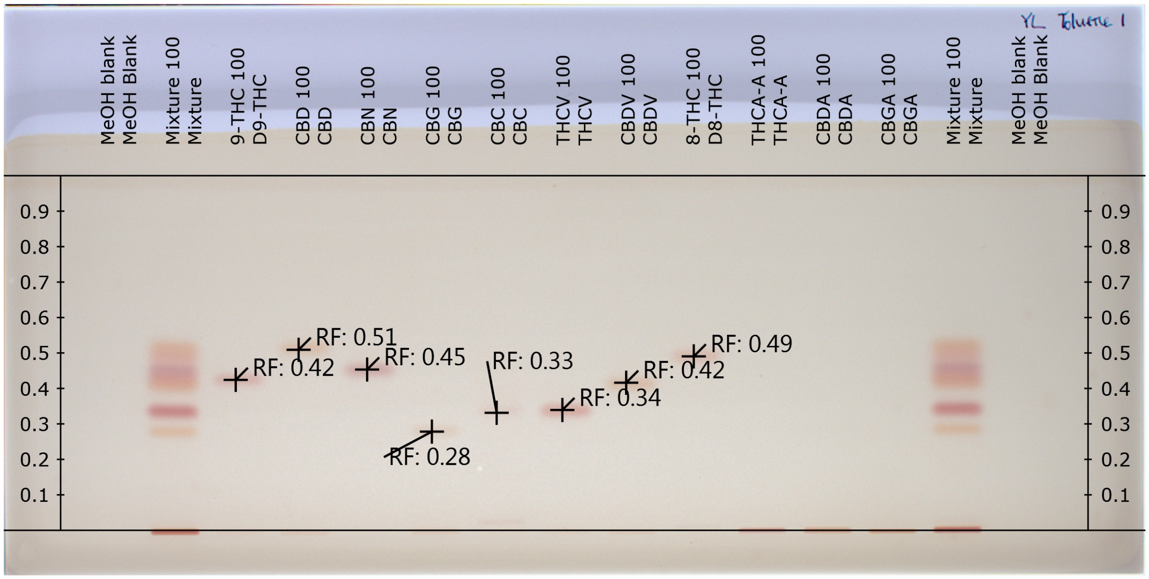

Exposure	0.055 s
Contrast	1
Normalized exposure	Disabled
Clarify	Disabled
White balance	1.19, 1.09, 0.80

R 366 — Derivatized, Remission366

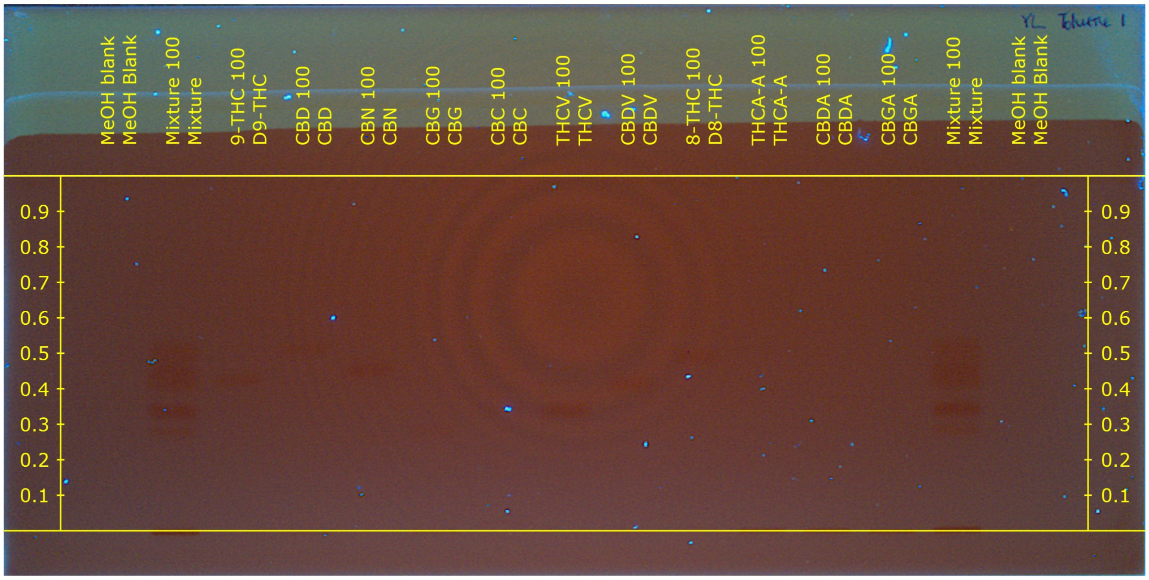

Exposure	9.999 s
Contrast	1
Normalized exposure	Disabled
Clarify	Disabled
White balance	1.00, 1.00, 1.00

Evaluation 1 :

Validated	false
Step	Take image derivatized plate 1b
Concentration unit type	Mass / volume
Notes	

Definition:

References:

9-THC 100		
Substance Name	Concentration	Purity
9-THC	100.000 µg/ml	100.00 %

CBD 100		
Substance Name	Concentration	Purity
CBD	100.000 µg/ml	100.00 %

CBN 100		
Substance Name	Concentration	Purity
CBN	100.000 µg/ml	100.00 %

CBG 100		
Substance Name	Concentration	Purity
CBG	100.000 µg/ml	100.00 %

CBC 100		
Substance Name	Concentration	Purity
CBC	100.000 µg/ml	100.00 %

THCV 100		
Substance Name	Concentration	Purity
THCV	100.000 µg/ml	100.00 %

CBDV 100		
Substance Name	Concentration	Purity
CBDV	100.000 µg/ml	100.00 %

8-THC 100		
Substance Name	Concentration	Purity
8-THC	100.000 µg/ml	100.00 %

THCA-A 100		
Substance Name	Concentration	Purity
THCA-A	100.000 µg/ml	100.00 %

CBDA 100		
Substance Name	Concentration	Purity
CBDA	100.000 µg/ml	100.00 %

CBGA 100		
Substance Name	Concentration	Purity
CBGA	100.000 µg/ml	100.00 %

Samples:

Vial ID	Amount	Volume solution	Reference amount	Related to
MeOH blank		0.00 ml		
Mixture 100		0.00 ml		

Integration parameters:

Bounds	[0.000,1.000]
Smoothing	Savitzky–Golay of order 3 and window 7
Baseline correction	Lowest slope with noise 0.05
Profile subtraction	None
Peaks detection	Gauss (legacy) with sensitivity 0.1, separation 1 and threshold 0.1

Scan:

Wavelength	RT White

Track 1:

Type	Sample
Vial ID	MeOH blank
Description	MeOH Blank
Volume	2.0 µl

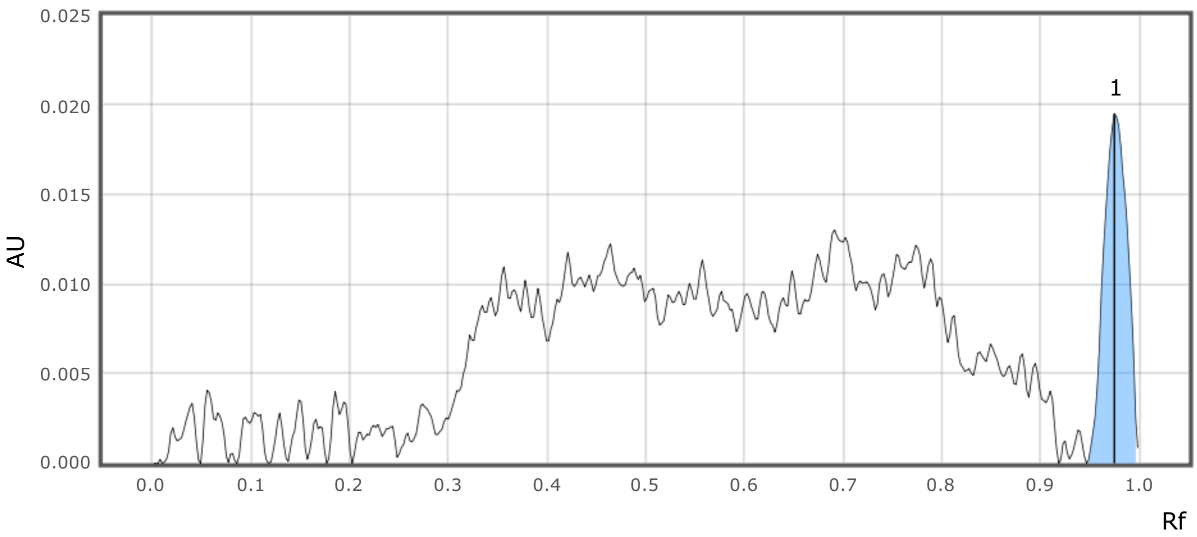

Peak #	Start		Max			End		Area		Manual peak	Substance Name
	Rf	H	Rf	H	%	Rf	H	A	%		
1	0.946	0.0000	0.974	0.0195	100.00	0.998	0.0009	0.00054	100.00	No	

Track 2:

Type	Sample
Vial ID	Mixture 100
Description	Mixture
Volume	2.0 µl

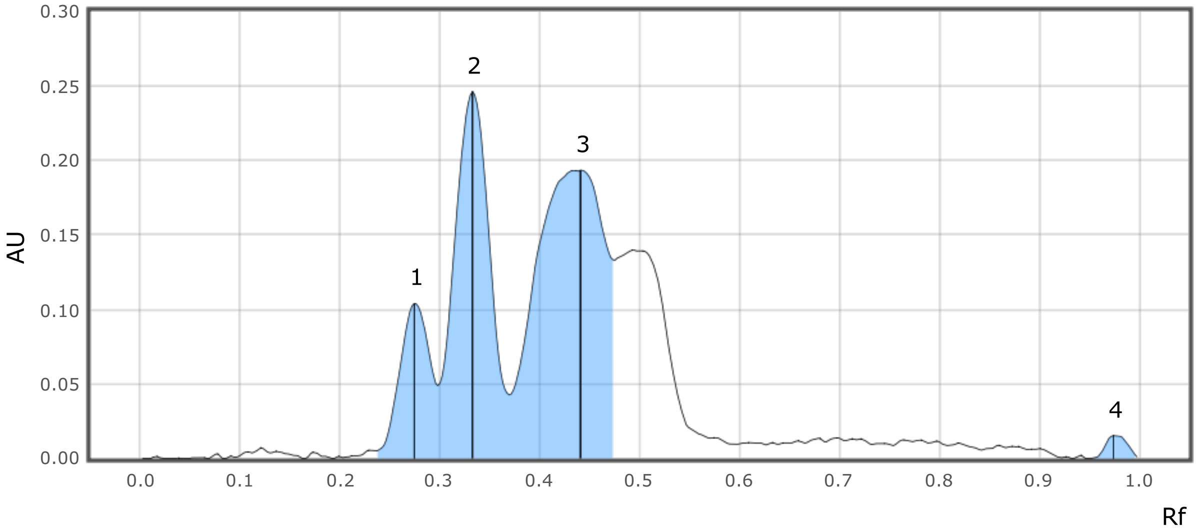

Peak #	Start Rf	Start H	Max Rf	Max H	Max %	End Rf	End H	Area A	Area %	Manual peak	Substance Name
1	0.237	0.0051	0.274	0.1038	18.60	0.296	0.0501	0.00355	11.96	No	CBG
2	0.298	0.0488	0.333	0.2459	44.06	0.369	0.0426	0.01015	34.13	No	THCV
3	0.369	0.0426	0.441	0.1932	34.61	0.475	0.1329	0.01564	52.62	No	CBN
4	0.951	0.0000	0.974	0.0152	2.73	0.998	0.0009	0.00038	1.29	No	

Track 3:	
Type	Reference
Vial ID	9-THC 100
Description	D9-THC
Volume	2.0 µl

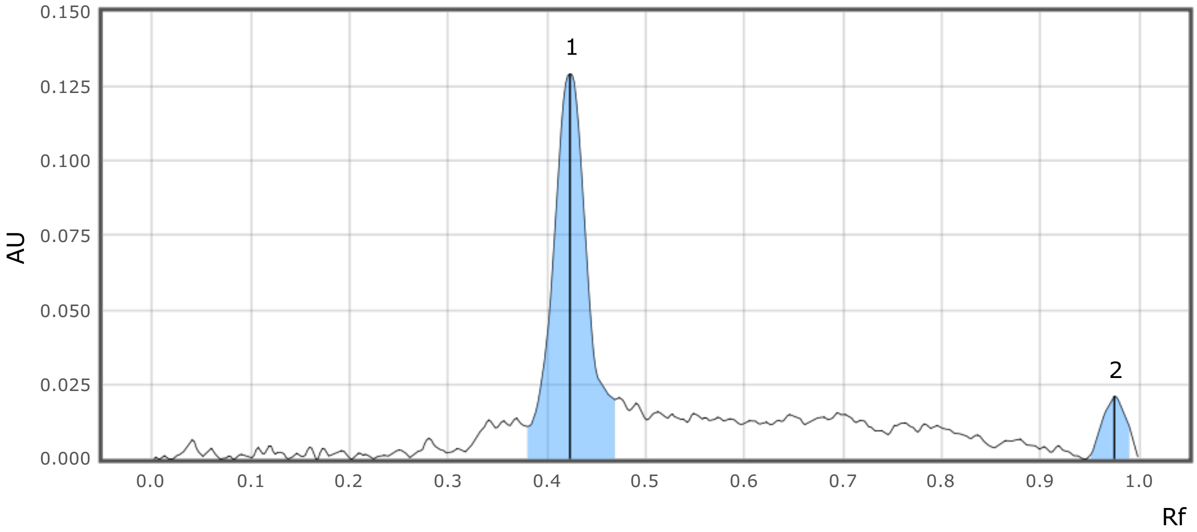

Peak #	Start Rf	Start H	Max Rf	Max H	Max %	End Rf	End H	Area A	Area %	Manual peak	Substance Name
1	0.380	0.0109	0.423	0.1291	86.03	0.469	0.0198	0.00524	89.80	No	9-THC
2	0.946	0.0000	0.974	0.0210	13.97	0.998	0.0007	0.00060	10.20	No	

Track 4:

Type	Reference
Vial ID	CBD 100
Description	CBD
Volume	2.0 µl

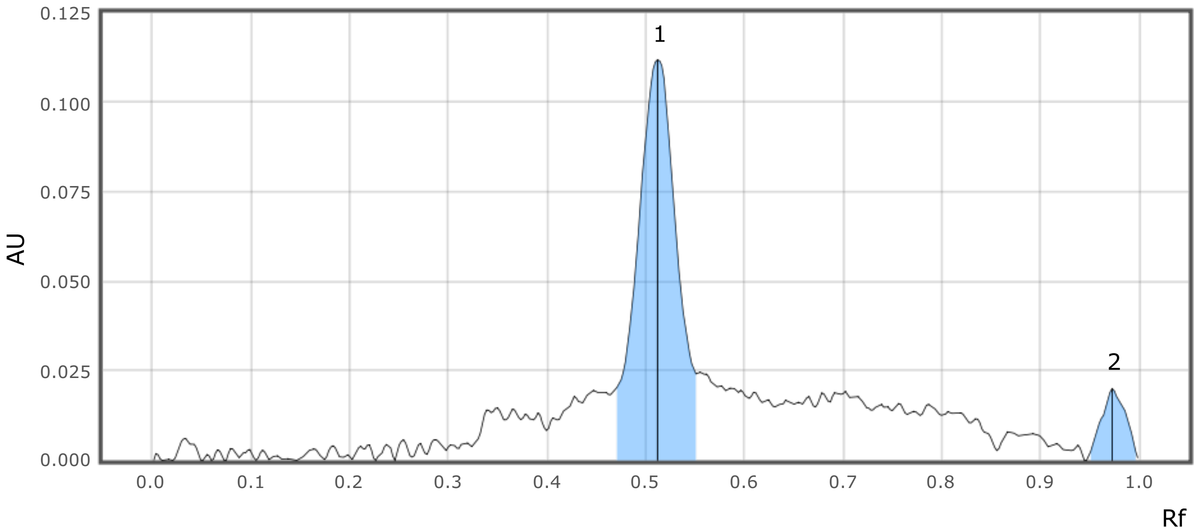

Peak	Start		Max			End		Area		Manual	Substance
#	Rf	H	Rf	H	%	Rf	H	A	%	peak	Name
1	0.464	0.0183	0.512	0.1118	84.79	0.551	0.0241	0.00524	90.19	No	CBD
2	0.946	0.0000	0.972	0.0200	15.21	0.998	0.0007	0.00057	9.81	No	

Track 5:

Type	Reference
Vial ID	CBN 100
Description	CBN
Volume	2.0 µl

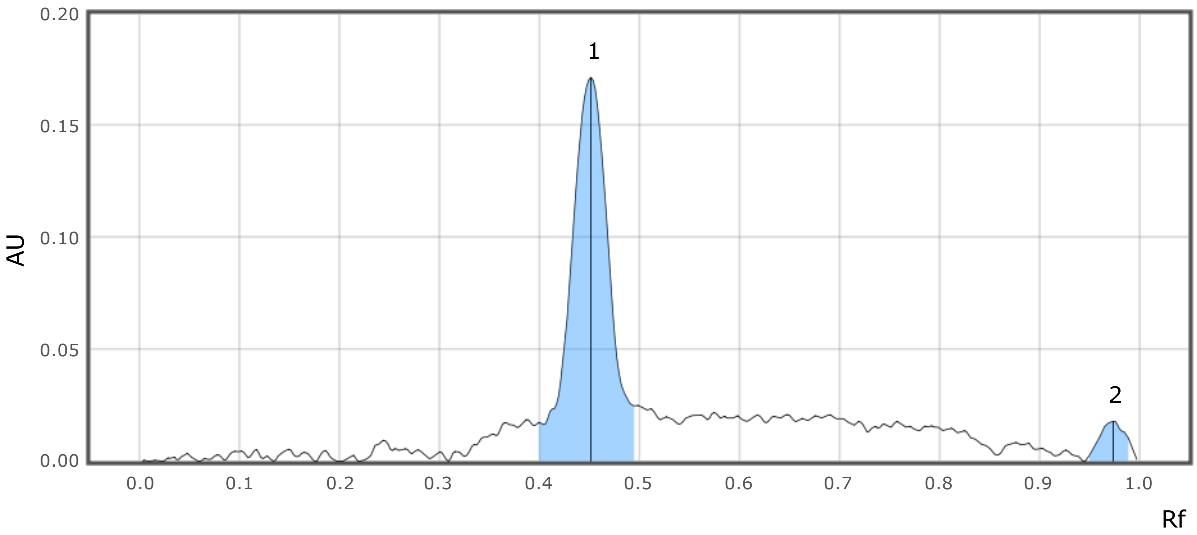

Peak #	Start Rf	Start H	Max Rf	Max H	Max %	End Rf	End H	Area A	Area %	Manual peak	Substance Name
1	0.395	0.0160	0.451	0.1716	90.62	0.495	0.0247	0.00761	93.27	No	CBN
2	0.946	0.0000	0.974	0.0178	9.38	0.998	0.0006	0.00055	6.73	No	

Track 6:

Type	Reference
Vial ID	CBG 100
Description	CBG
Volume	2.0 µl

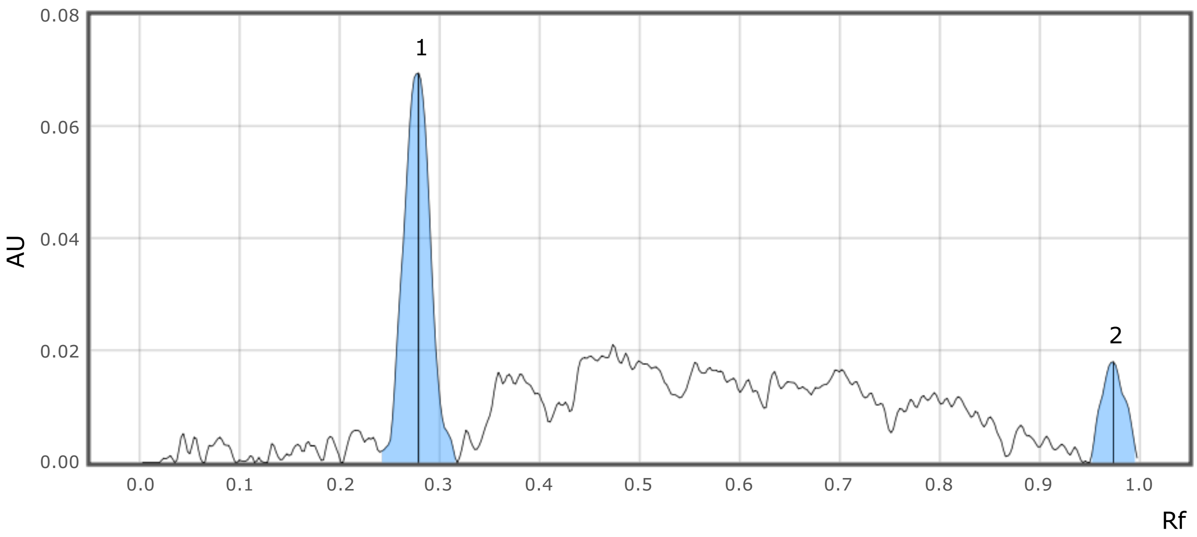

Peak #	Start Rf	Start H	Max Rf	Max H	Max %	End Rf	End H	Area A	Area %	Manual peak	Substance Name
1	0.240	0.0019	0.278	0.0696	79.49	0.317	0.0000	0.00222	81.41	No	CBG
2	0.951	0.0000	0.974	0.0180	20.51	0.998	0.0007	0.00051	18.59	No	

Track 7:

Type	Reference
Vial ID	CBC 100
Description	CBC
Volume	2.0 µl

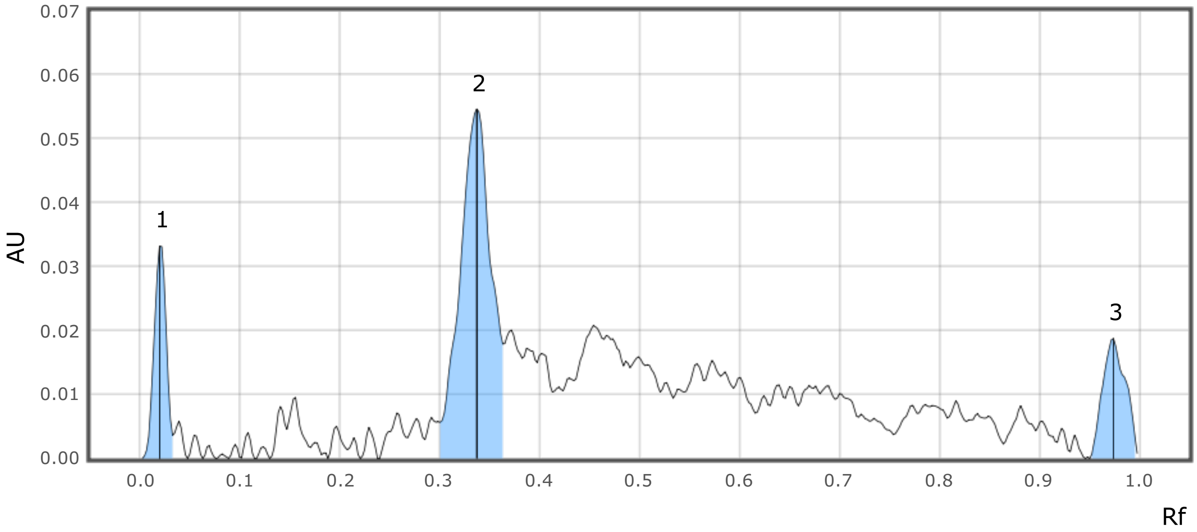

Peak #	Start Rf	Start H	Max Rf	Max H	Max %	End Rf	End H	Area A	Area %	Manual peak	Substance Name
1	0.002	0.0000	0.019	0.0332	31.14	0.032	0.0036	0.00047	15.93	No	
2	0.300	0.0056	0.337	0.0547	51.27	0.363	0.0179	0.00195	66.89	No	THCV
3	0.951	0.0000	0.974	0.0188	17.59	0.998	0.0008	0.00050	17.18	No	

Track 8:	
Type	Reference
Vial ID	THCV 100
Description	THCV
Volume	2.0 µl

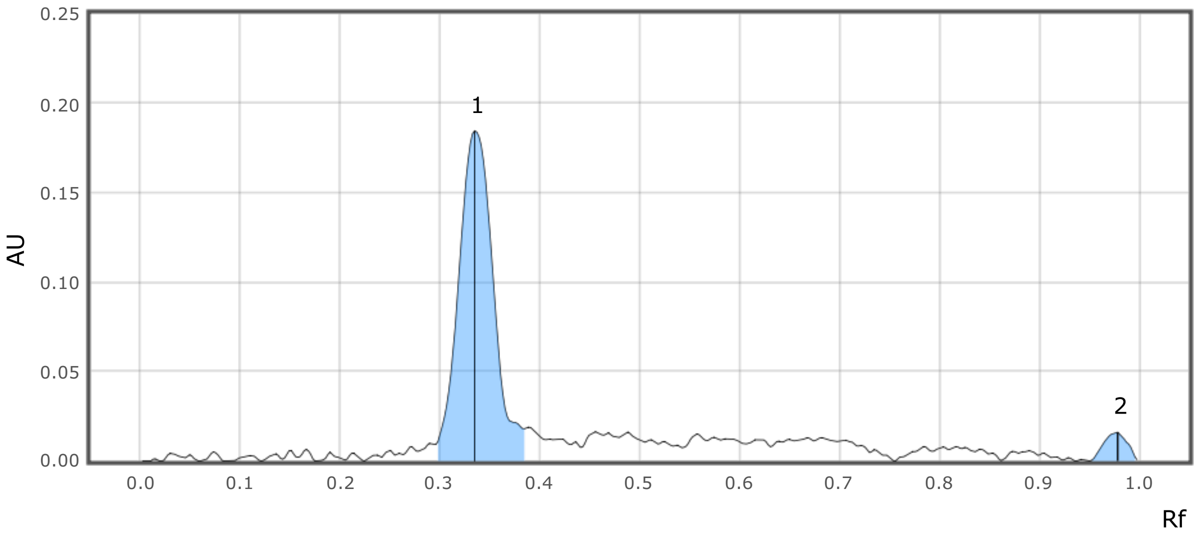

Peak #	Start Rf	Start H	Max Rf	Max H	Max %	End Rf	End H	Area A	Area %	Manual peak	Substance Name
1	0.296	0.0092	0.335	0.1845	92.02	0.384	0.0179	0.00744	94.50	No	THCV
2	0.951	0.0000	0.979	0.0160	7.98	0.998	0.0006	0.00043	5.50	No	

Track 9:

Type	Reference
Vial ID	CBDV 100
Description	CBDV
Volume	2.0 µl

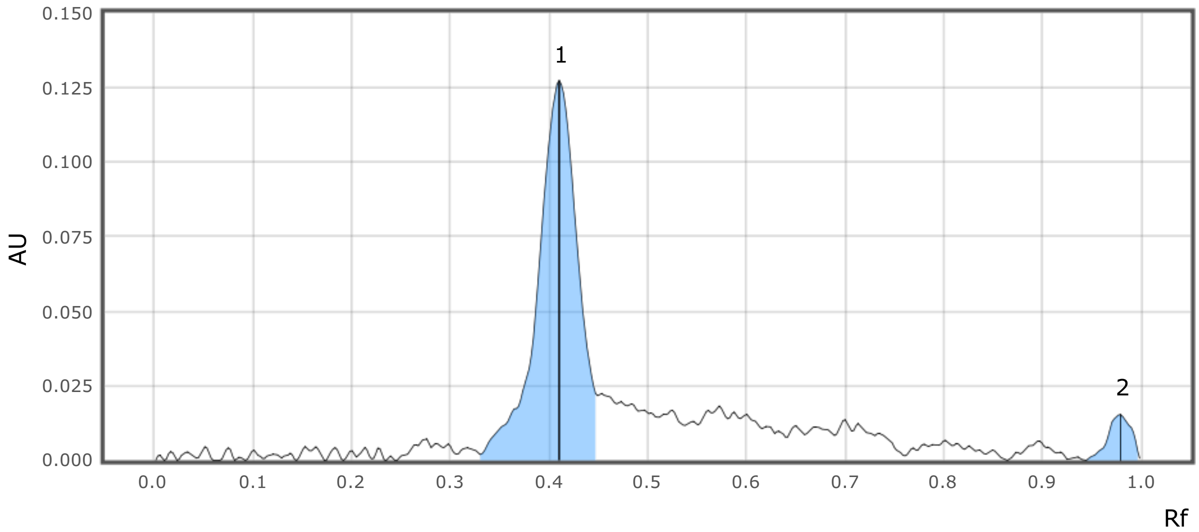

Peak	Start		Max			End		Area		Manual	Substance
#	Rf	H	Rf	H	%	Rf	H	A	%	peak	Name
1	0.330	0.0019	0.410	0.1272	89.26	0.449	0.0217	0.00596	93.37	No	CBDV
2	0.942	0.0000	0.979	0.0153	10.74	0.998	0.0006	0.00042	6.63	No	

Track 10:

Type	Reference
Vial ID	8-THC 100
Description	D8-THC
Volume	2.0 µl

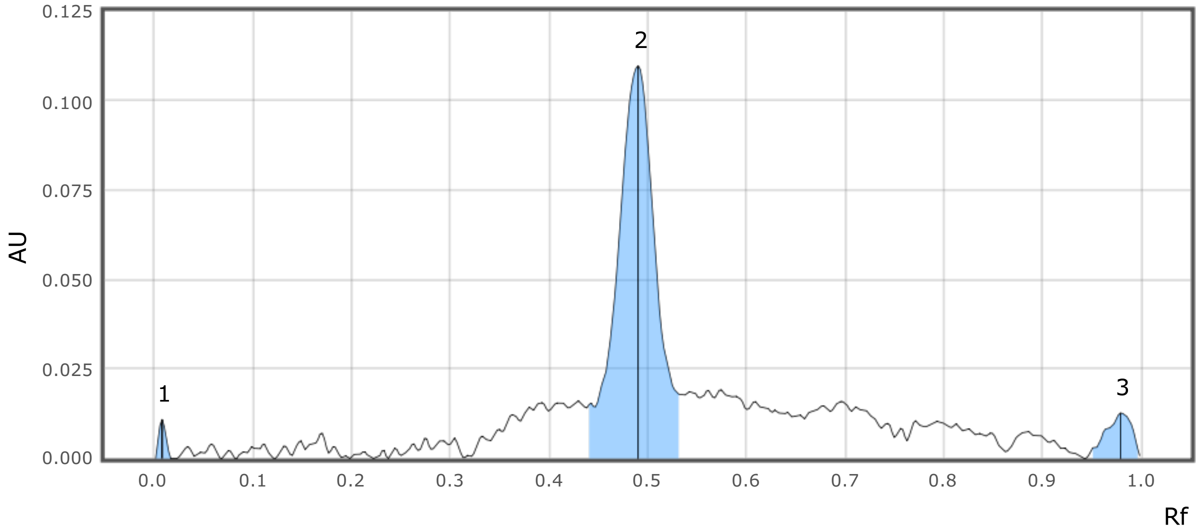

Peak #	Start		Max			End		Area		Manual peak	Substance Name
	Rf	H	Rf	H	%	Rf	H	A	%		
1	0.002	0.0000	0.008	0.0109	8.17	0.017	0.0000	0.00009	1.63	No	CBGA
2	0.438	0.0141	0.490	0.1097	82.32	0.533	0.0177	0.00494	91.16	No	8-THC
3	0.946	0.0008	0.979	0.0127	9.51	0.998	0.0007	0.00039	7.21	No	

Track 11:

Type	Reference
Vial ID	THCA-A 100
Description	THCA-A
Volume	2.0 µl

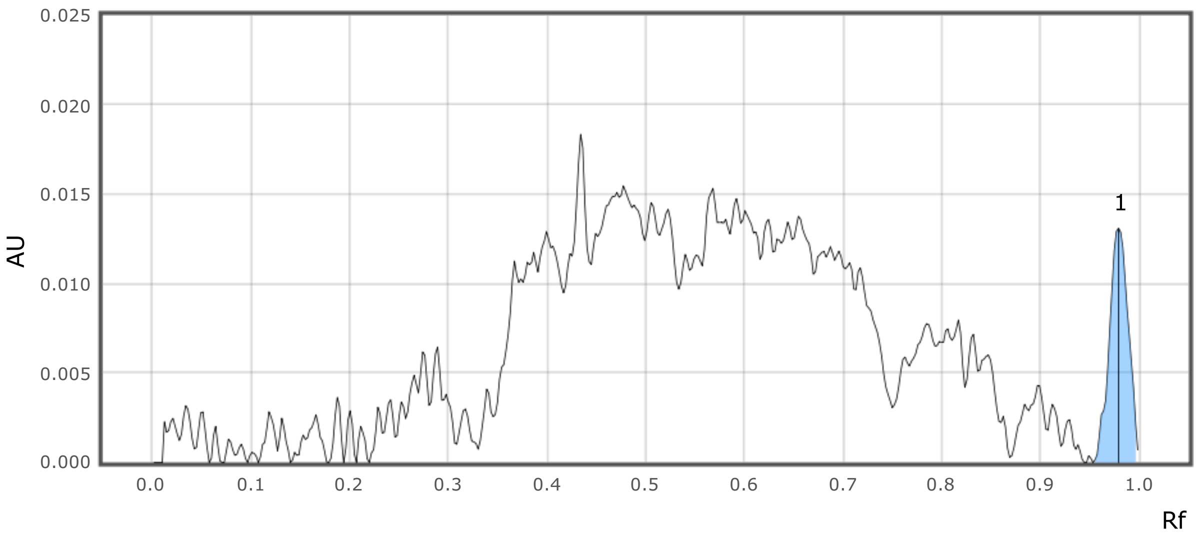

Peak #	Start		Max			End		Area		Manual peak	Substance Name
	Rf	H	Rf	H	%	Rf	H	A	%		
1	0.953	0.0000	0.979	0.0131	100.0 0	0.998	0.0007	0.00029	100.0 0	No	

Track 12:

Type	Reference
Vial ID	CBDA 100
Description	CBDA
Volume	2.0 µl

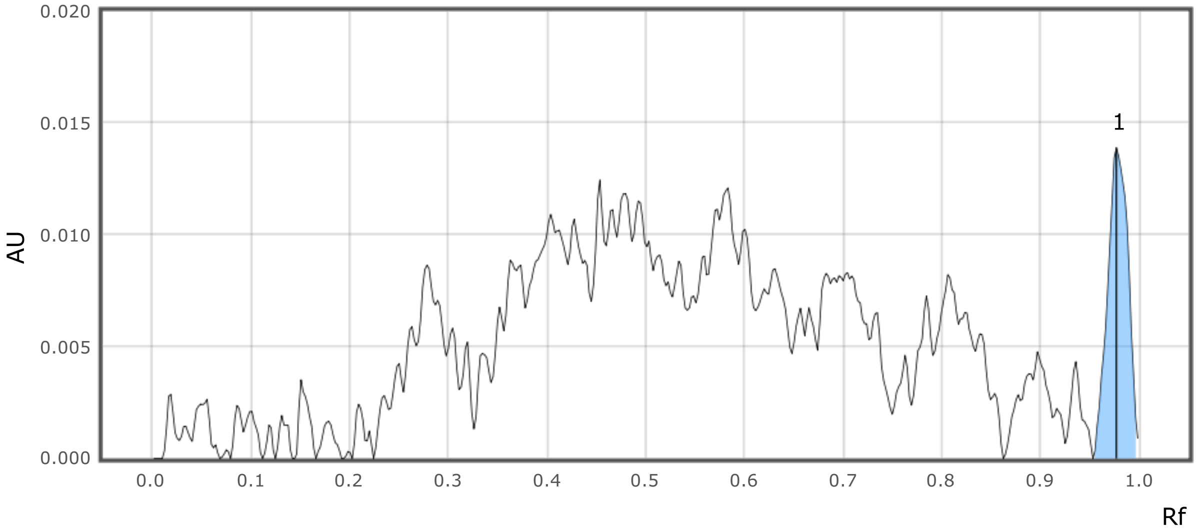

Peak #	Start		Max			End		Area		Manual peak	Substance Name
	Rf	H	Rf	H	%	Rf	H	A	%		
1	0.953	0.0000	0.977	0.0139	100.0 0	0.998	0.0009	0.00034	100.0 0	No	

Track 13:

Type	Reference
Vial ID	CBGA 100
Description	CBGA
Volume	2.0 µl

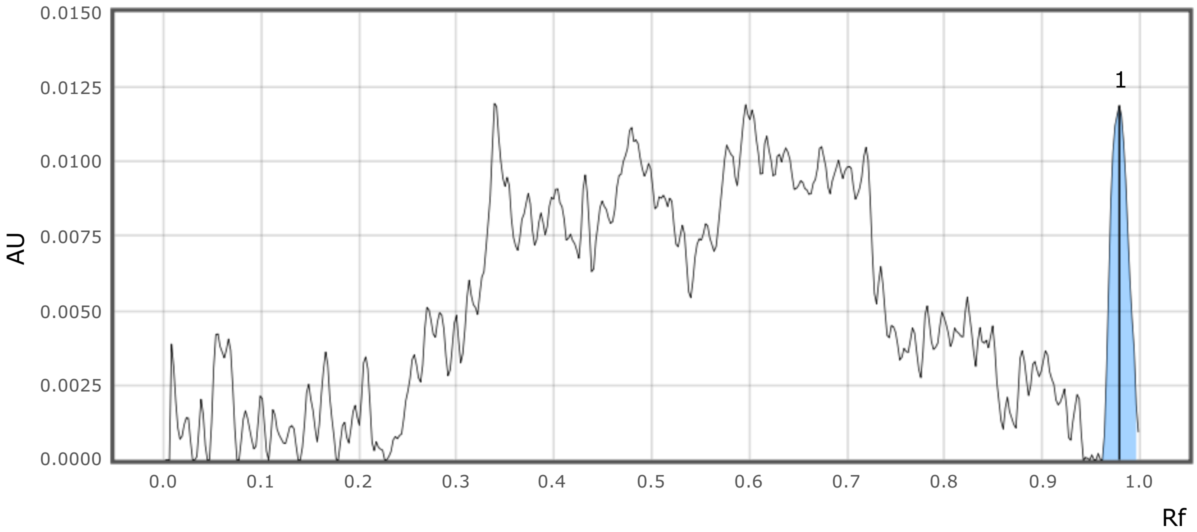

Peak #	Start		Max			End		Area		Manual peak	Substance Name
	Rf	H	Rf	H	%	Rf	H	A	%		
1	0.961	0.0000	0.979	0.0119	100.0 0	0.998	0.0009	0.00026	100.0 0	No	

Track 14:

Type	Sample
Vial ID	Mixture 100
Description	Mixture
Volume	2.0 µl

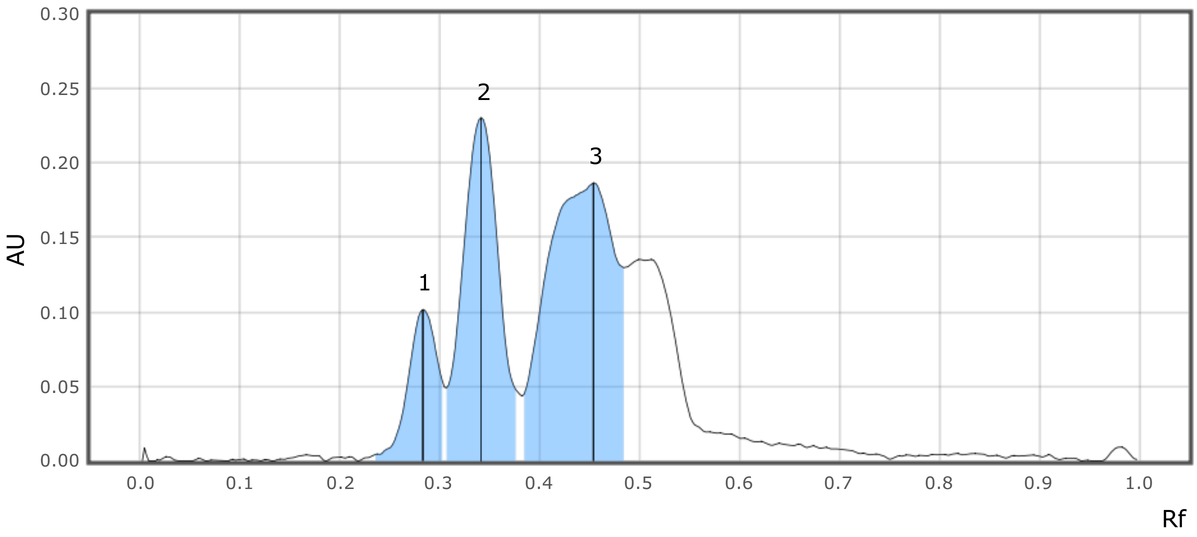

Peak #	Start Rf	Start H	Max Rf	Max H	Max %	End Rf	End H	Area A	Area %	Manual peak	Substance Name
1	0.233	0.0038	0.283	0.1015	19.59	0.304	0.0495	0.00350	12.49	No	CBG
2	0.307	0.0490	0.341	0.2302	44.43	0.380	0.0445	0.00982	35.04	No	CBC
3	0.382	0.0435	0.454	0.1864	35.98	0.484	0.1296	0.01471	52.47	No	CBN

Track 15:

Type	Sample
Vial ID	MeOH blank
Description	MeOH Blank
Volume	2.0 µl

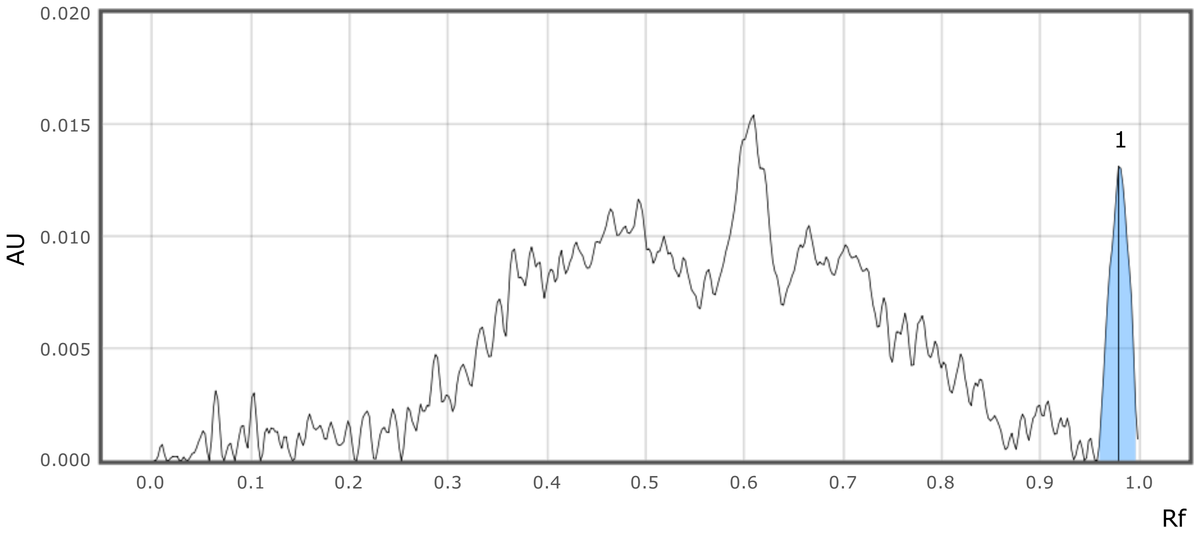

Peak #	Start		Max			End		Area		Manual peak	Substance Name
	Rf	H	Rf	H	%	Rf	H	A	%		
1	0.957	0.0000	0.979	0.0131	100.00	0.998	0.0009	0.00031	100.00	No	

Calibration results:

Height calibration for substance 8-THC @ RT White:

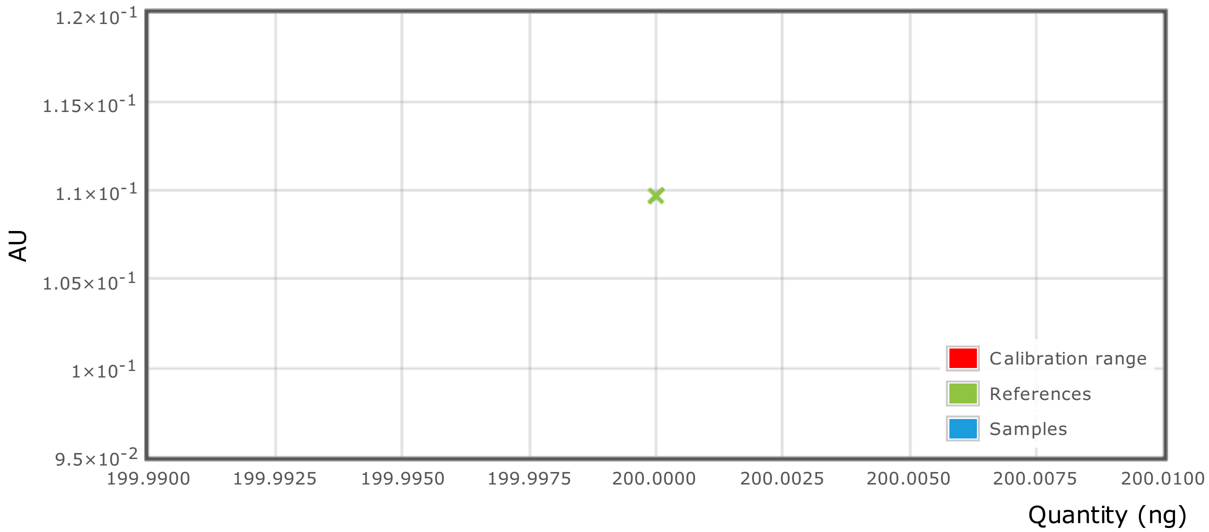

Regression mode	Linear-2
Range deviation	5.00 %
Related substances	Default
Number of references	1
Calibration function	$y=0x$
Coefficient of variation	CV 0.00 %
Correlation coefficient	n/a
⚠	Unable to compute the results for this substance because there wasn't enough groups of references replicas (at least 1 for Linear-1, 2 for Linear2 and Mime-1 and 3 for Polynomial and MiMe-2)

Height calibration for substance 9-THC @ RT White:

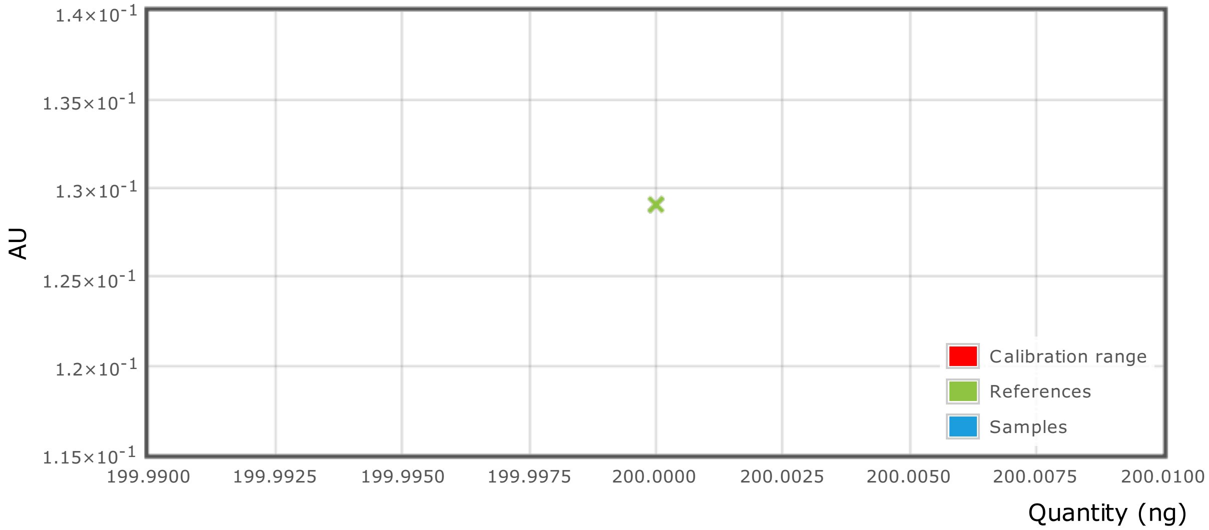

Regression mode	Linear-2
Range deviation	5.00 %
Related substances	Default
Number of references	1
Calibration function	$y=0x$
Coefficient of variation	CV 0.00 %
Correlation coefficient	n/a
⚠	Unable to compute the results for this substance because there wasn't eno ugh groups of references replicas (at least 1 for Linear-1, 2 for Linear2 and Mime-1 and 3 for Polynomial and MiMe-2)

Height calibration for substance CBC @ RT White:

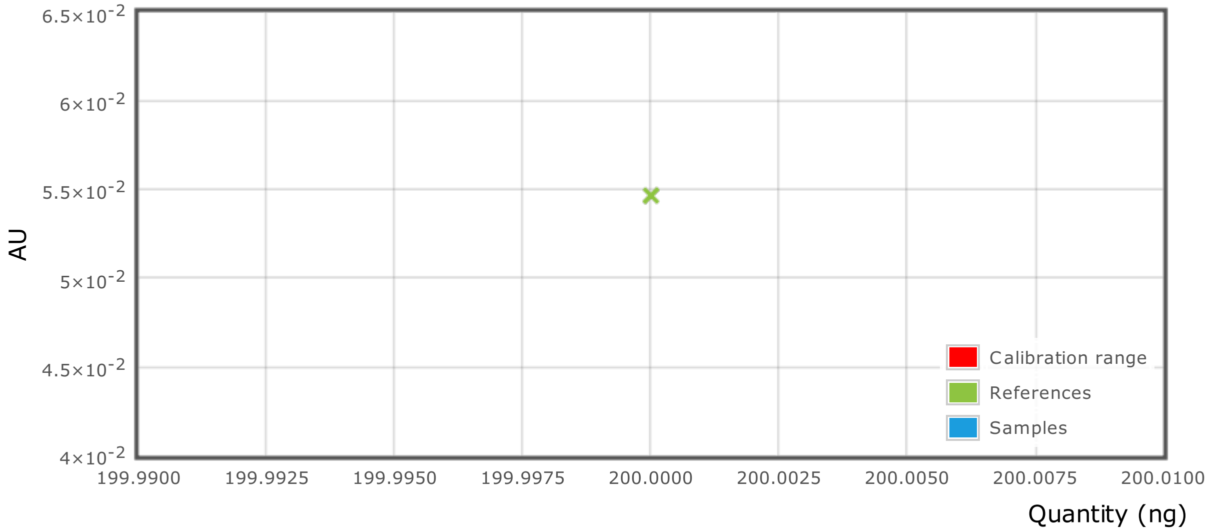

Regression mode	Linear-2
Range deviation	5.00 %
Related substances	Default
Number of references	1
Calibration function	$y=0x$
Coefficient of variation	CV 0.00 %
Correlation coefficient	n/a
⚠	Unable to compute the results for this substance because there wasn't enough groups of references replicas (at least 1 for Linear-1, 2 for Linear2 and Mime-1 and 3 for Polynomial and MiMe-2)

Height calibration for substance CBD @ RT White:

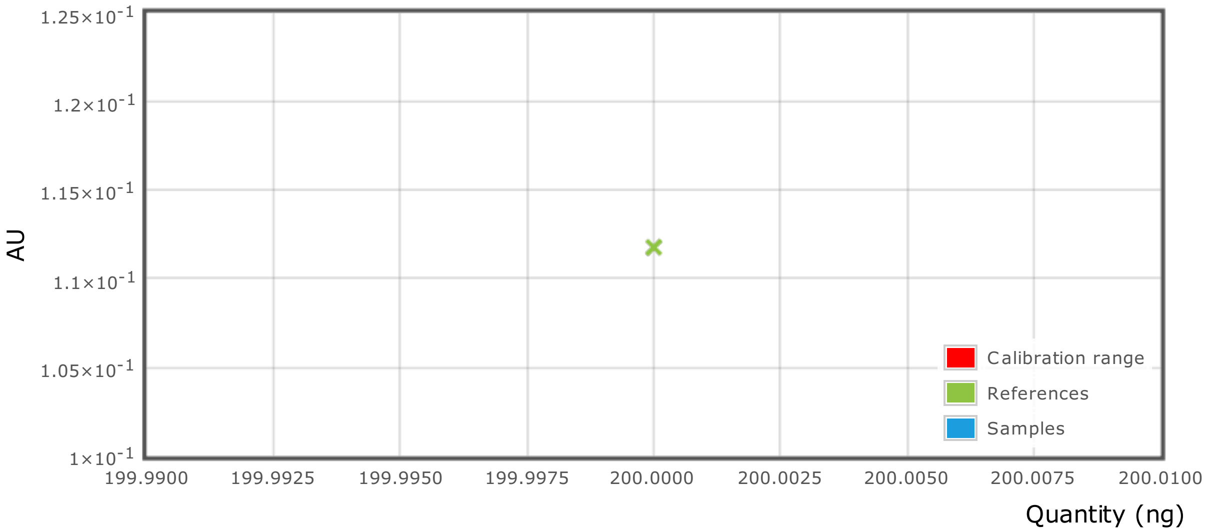

Regression mode	Linear-2
Range deviation	5.00 %
Related substances	Default
Number of references	1
Calibration function	$y=0x$
Coefficient of variation	CV 0.00 %
Correlation coefficient	n/a
⚠	Unable to compute the results for this substance because there wasn't enough groups of references replicas (at least 1 for Linear-1, 2 for Linear2 and Mime-1 and 3 for Polynomial and MiMe-2)

Height calibration for substance CBDA @ RT White:

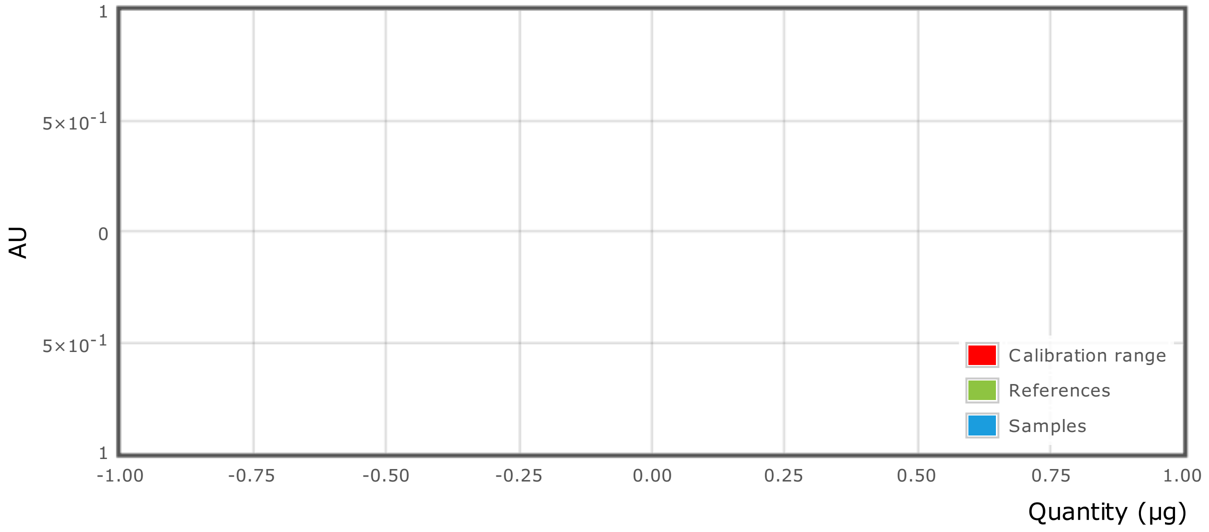

Regression mode	Linear-2
Range deviation	5.00 %
Related substances	Default
Number of references	0
Calibration function	$y=0x$
Coefficient of variation	CV 0.00 %
Correlation coefficient	n/a
⚠	Unable to compute the results for this substance because there wasn't eno ugh groups of references replicas (at least 1 for Linear-1, 2 for Linear2 and Mime-1 and 3 for Polynomial and MiMe-2)

Height calibration for substance CBDV @ RT White:

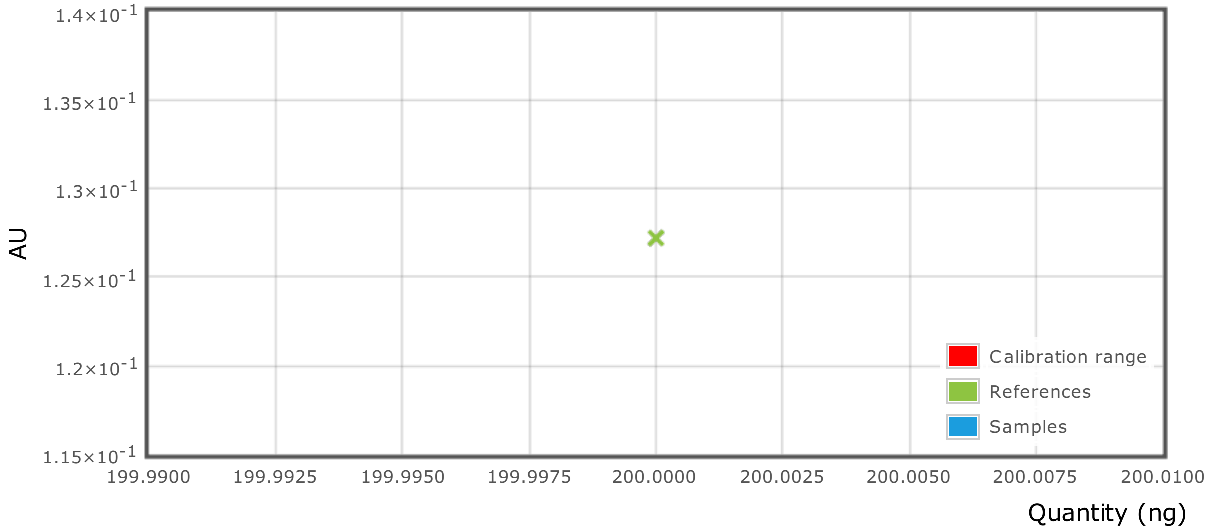

Regression mode	Linear-2
Range deviation	5.00 %
Related substances	Default
Number of references	1
Calibration function	$y=0x$
Coefficient of variation	CV 0.00 %
Correlation coefficient	n/a
⚠	Unable to compute the results for this substance because there wasn't enough groups of references replicas (at least 1 for Linear-1, 2 for Linear2 and Mime-1 and 3 for Polynomial and MiMe-2)

Height calibration for substance CBG @ RT White:

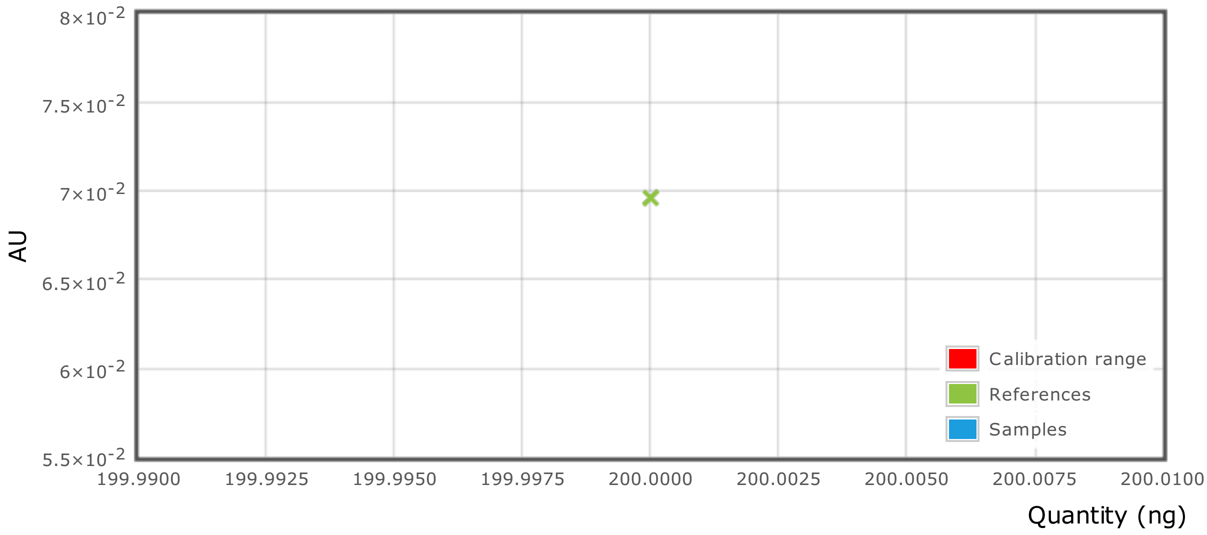

Regression mode	Linear-2
Range deviation	5.00 %
Related substances	Default
Number of references	1
Calibration function	$y=0x$
Coefficient of variation	CV 0.00 %
Correlation coefficient	n/a
⚠	Unable to compute the results for this substance because there wasn't enough groups of references replicas (at least 1 for Linear-1, 2 for Linear2 and Mime-1 and 3 for Polynomial and MiMe-2)

Height calibration for substance CBGA @ RT White:

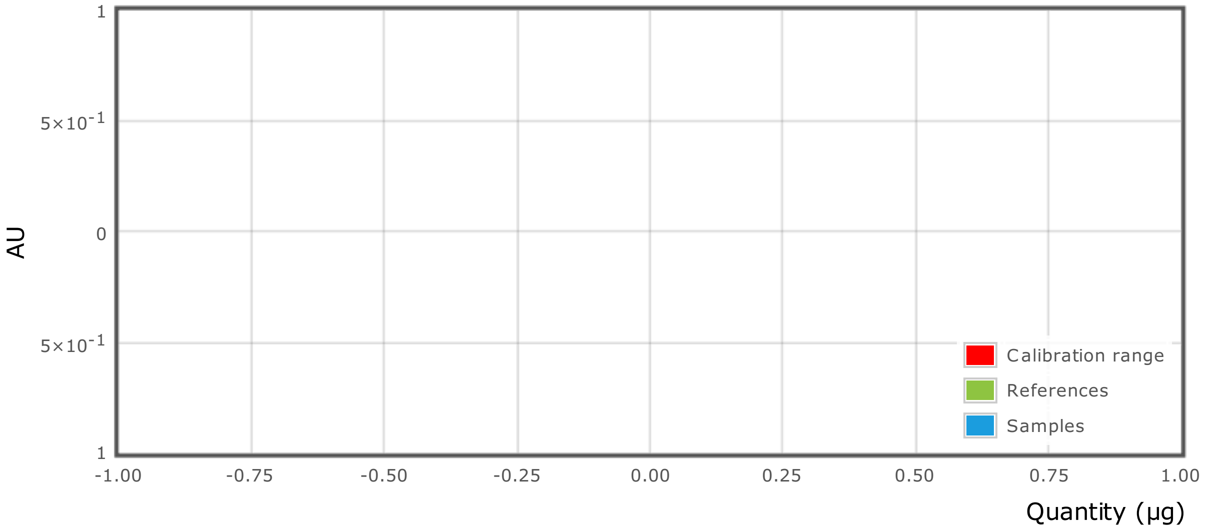

Regression mode	Linear-2
Range deviation	5.00 %
Related substances	Default
Number of references	0
Calibration function	$y=0x$
Coefficient of variation	CV 0.00 %
Correlation coefficient	n/a
⚠	Unable to compute the results for this substance because there wasn't eno ugh groups of references replicas (at least 1 for Linear-1, 2 for Linear2 and Mime-1 and 3 for Polynomial and MiMe-2)

Height calibration for substance CBN @ RT White:

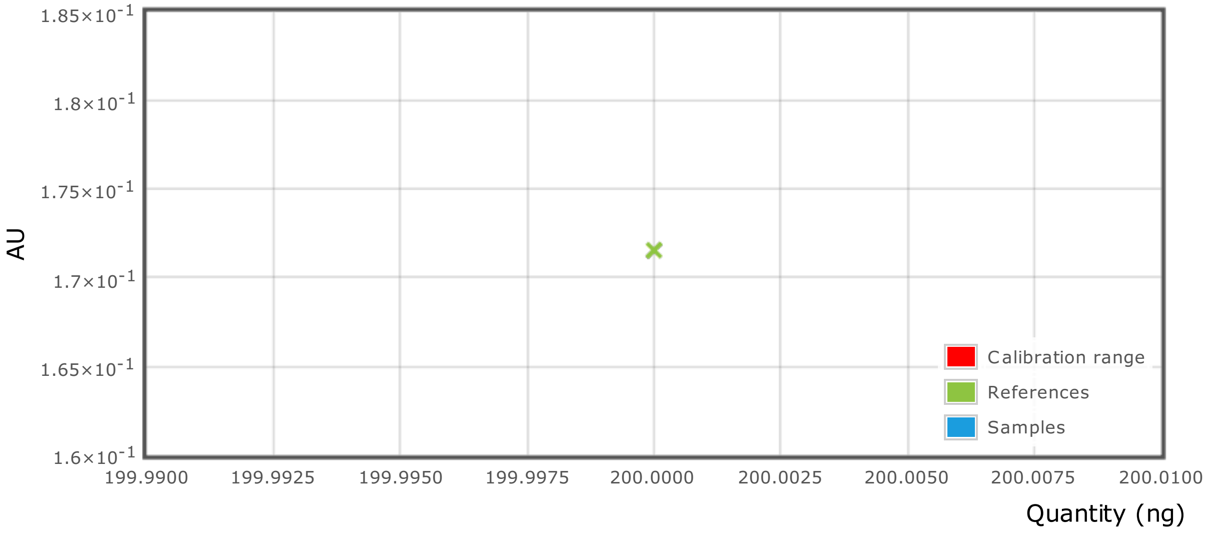

Regression mode	Linear-2
Range deviation	5.00 %
Related substances	Default
Number of references	1
Calibration function	$y=0x$
Coefficient of variation	CV 0.00 %
Correlation coefficient	n/a
⚠	Unable to compute the results for this substance because there wasn't enough groups of references replicas (at least 1 for Linear-1, 2 for Linear2 and Mime-1 and 3 for Polynomial and MiMe-2)

Height calibration for substance THCA-A @ RT White:

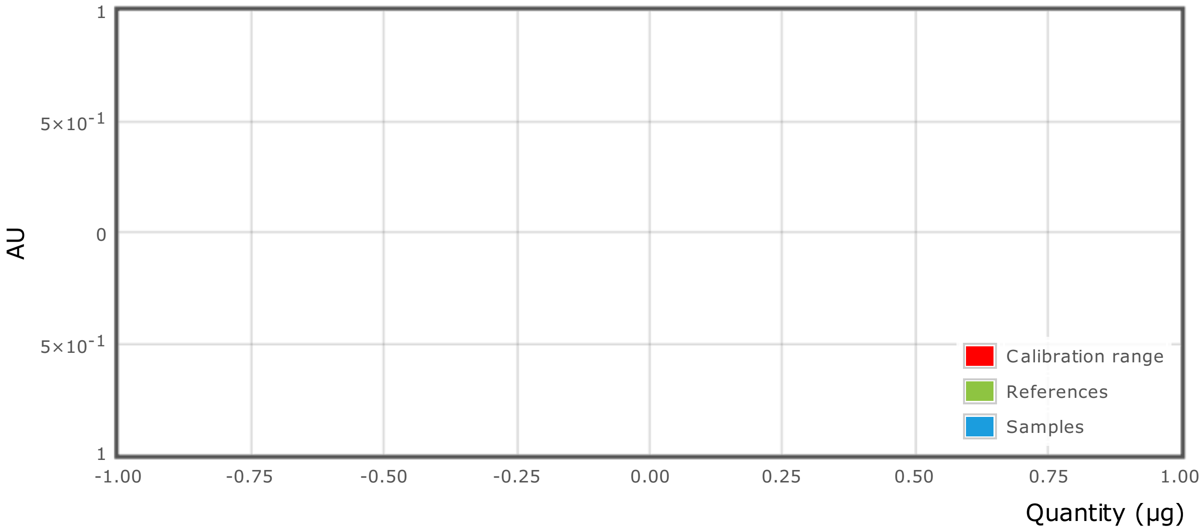

Regression mode	Linear-2
Range deviation	5.00 %
Related substances	Default
Number of references	0
Calibration function	$y=0x$
Coefficient of variation	CV 0.00 %
Correlation coefficient	n/a
⚠	Unable to compute the results for this substance because there wasn't enough groups of references replicas (at least 1 for Linear-1, 2 for Linear2 and Mime-1 and 3 for Polynomial and MiMe-2)

Height calibration for substance THCV @ RT White:

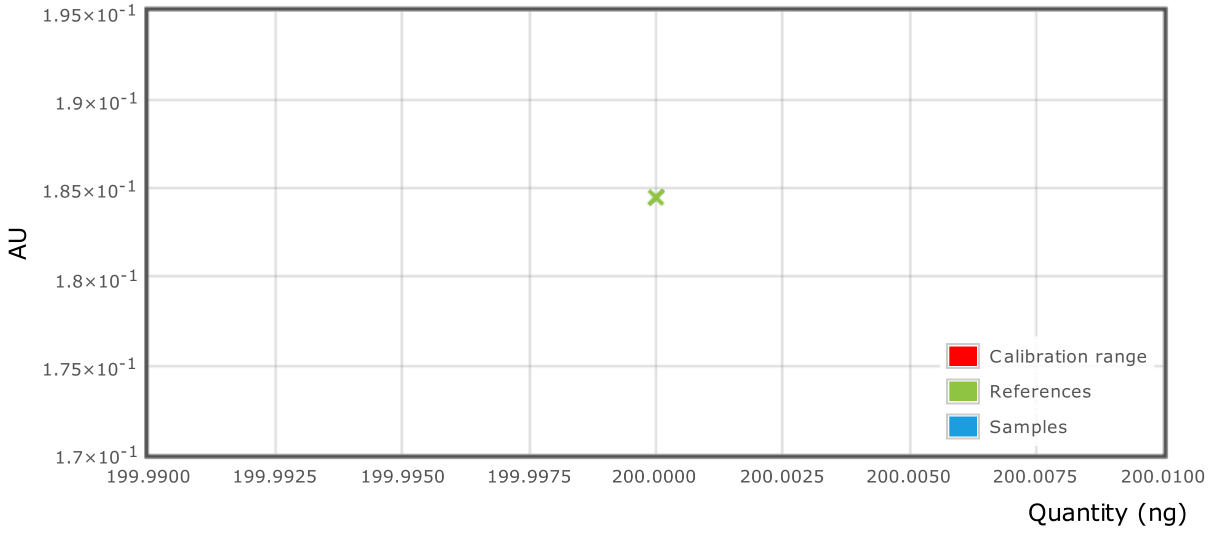

Regression mode	Linear-2
Range deviation	5.00 %
Related substances	Default
Number of references	1
Calibration function	$y=0x$
Coefficient of variation	CV 0.00 %
Correlation coefficient	n/a
⚠	Unable to compute the results for this substance because there wasn't enough groups of references replicas (at least 1 for Linear-1, 2 for Linear2 and Mime-1 and 3 for Polynomial and MiMe-2)

Results:

Substance having no available results		
⚠	CBDV	There wasn't any sample application available in the assignments for this substance. Please check that the peaks were correctly detected and assig ned for this substance.
⚠	CBC	Unable to compute the results for this substance because there wasn't en ough groups of references replicas (at least 1 for Linear-1, 2 for Linear2 a nd Mime-1 and 3 for Polynomial and MiMe-2)
⚠	CBN	Unable to compute the results for this substance because there wasn't en ough groups of references replicas (at least 1 for Linear-1, 2 for Linear2 a nd Mime-1 and 3 for Polynomial and MiMe-2)
⚠	CBGA	There wasn't any sample application available in the assignments for this substance. Please check that the peaks were correctly detected and assig ned for this substance.
⚠	CBD	There wasn't any sample application available in the assignments for this substance. Please check that the peaks were correctly detected and assig ned for this substance.
⚠	8-THC	There wasn't any sample application available in the assignments for this substance. Please check that the peaks were correctly detected and assig ned for this substance.
⚠	THCV	Unable to compute the results for this substance because there wasn't en ough groups of references replicas (at least 1 for Linear-1, 2 for Linear2 a nd Mime-1 and 3 for Polynomial and MiMe-2)
⚠	THCA-A	There wasn't any sample application available in the assignments for this substance. Please check that the peaks were correctly detected and assig ned for this substance.
⚠	9-THC	There wasn't any sample application available in the assignments for this substance. Please check that the peaks were correctly detected and assig ned for this substance.
⚠	CBDA	There wasn't any sample application available in the assignments for this substance. Please check that the peaks were correctly detected and assig ned for this substance.
⚠	CBG	Unable to compute the results for this substance because there wasn't en ough groups of references replicas (at least 1 for Linear-1, 2 for Linear2 a nd Mime-1 and 3 for Polynomial and MiMe-2)

A track marked with ⚠ means: this result is outside the regression range given by the reference assignments, but is included in the results because it is in the allowed range deviation.

Analyst:	Reviewer:
