# Supplementary material for: High Performance Thin-Layer Chromatography (HPTLC) data of Cannabinoids in ten mobile phase systems
Source: Data Brief. 2020 Jun 30;31:105955. doi: 10.1016/j.dib.2020.105955 (PMC7352075; doi:10.1016/j.dib.2020.105955)
Supplement: Supplementary file 1 [file mmc1.zip › S1-Triplicate reports/XHDa-2.pdf]

# Analysis: XHDa-re-2

**Path: Home/YL Research**

**Based on method: Triplets Method**

| | | |
|---|---|---|
| Created | 07-Jun-2019 15:46:40 | visionCATSuser |
| Modified | 07-Jun-2019 17:39:31 | visionCATSuser |
| Last HPTLC log | 07-Jun-2019 17:39:31 | Analysis modified |
| Explorer notes | | |

| Track | Vial ID | Description | Volume | Position | Type |
|---|---|---|---|---|---|
| 1 | MeOH blank | MeOH Blank | 2.0 µl | A1 | Sample |
| 2 | Mixture 100 | Mixture 500ng | 5.0 µl | A2 | Sample |
| 3 | 9-THC 100 | D9-THC 500ng | 5.0 µl | A3 | Reference |
| 4 | CBD 100 | CBD 500ng | 5.0 µl | A4 | Reference |
| 5 | CBN 100 | CBN 500ng | 5.0 µl | A5 | Reference |
| 6 | CBG 100 | CBG 500ng | 5.0 µl | A6 | Reference |
| 7 | CBC 100 | CBC 500ng | 5.0 µl | A7 | Reference |
| 8 | THCV 100 | THCV 500ng | 5.0 µl | A8 | Reference |
| 9 | CBDV 100 | CBDV 500ng | 5.0 µl | A9 | Reference |
| 10 | 8-THC 100 | D8-THC 500ng | 5.0 µl | A10 | Reference |
| 11 | THCA-A 100 | THCA-A 500ng | 5.0 µl | A11 | Reference |
| 12 | CBDA 100 | CBDA 500ng | 5.0 µl | B1 | Reference |
| 13 | CBGA 100 | CBGA 500ng | 5.0 µl | B2 | Reference |
| 14 | Mixture 100 | Mixture 500ng | 5.0 µl | A2 | Sample |
| 15 | MeOH blank | MeOH Blank | 2.0 µl | A1 | Sample |
| Sequence table notes | | | | | |

A track marked with ⚠ means: the application type is overridden in some evaluation(s).

## System setup:

| | |
|---|---|
| Software | Server User-PC, version 2.5.18072.1 |
| ATS4 | S/N:080713 |
| Chamber | N/A |
| Derivatization dip | N/A |
| Scanner3 | S/N:031025 |
| Visualizer | S/N:230515 |

# Chromatography

## Plate layout:

| | |
|---|---|
| Stationary phase | Merck, HPTLC plates silica gel 60 F 254 |
| Plate format | 200.0 x 100.0 mm |
| Application type | User |
| Application | Position Y: 10.0 mm, length: 8.0 mm, width: 0.0 mm |
| Track | First position X: 20.0 mm, distance: 11.4 mm |
| Solvent front position | 70.0 mm |
| Notes | |

## Take image clean plate 1a - Visualizer (S/N: 230515):

| | |
|---|---|
| Quality | Enhanced |
| RT White | auto capture, Auto, level 85 %, Band |
| R 254 | auto capture, Auto, level 85 %, Band |
| Instrument diagnostics | Valid diagnostics |
| Documentation step label | |
| Notes | |

## Application 1 - ATS 4 (S/N: 080713):

| | |
|---|---|
| Spray gas | NI |
| Sample solvent type | Methanol |
| Filling speed | 15 µl/s |
| Predosage volume | 200 nl |
| Retraction volume | 200 nl |
| Dosage speed | 150 nl/s |
| Filling quality | User |
| Rinsing cycles / vacuum | 1 / 4 s |
| Filling cycles / vacuum | 1 / 4 s |
| Rinsing solvent name | Methanol |
| Nozzle temperature | Unheated |
| Rack in use | Standard |
| Instrument diagnostics | Valid diagnostics |
| Notes | |

## Development 1 - Chamber:

| | |
|---|---|
| Tank | TTC 20x10 |
| Mobile phase | |
| Saturation time | 20 min |
| Use saturation pad | true |
| Use smartALERT | false |
| Volume front through | 10 ml |
| Volume rear through | 20 ml |
| Drying time | 5 min |
| Drying temperature | Room temperature |
| Notes | |

## Take image developed plate 1a - Visualizer (S/N: 230515):

| | |
|---|---|
| Quality | Enhanced |
| RT White | auto capture, Auto, level 85 %, Band |
| R 254 | auto capture, Auto, level 85 %, Band |
| R 366 | auto capture, Auto, level 85 %, Band |
| Instrument diagnostics | Valid diagnostics |
| Documentation step label | |
| Notes | |

## Scan developed plate 1b - Scanner 3 (S/N: 031025):

| | |
|---|---|
| Scanner type | Single λ |
| Optimization for | Resolution |
| Measurement mode | Absorption |
| Filter | n/a |
| Detector mode | Automatic |
| Scanning speed | 20 mm/s |
| Data resolution | 100 µm/step |
| Slit | 5 x 0.2 mm, micro |
| Partial scan | No |
| Lamp | Deuterium & Tungsten |
| Wavelength(s) | 254 nm |
| Instrument diagnostics | Valid diagnostics |
| Documentation step label | |
| Notes | |

## Derivatization 1 - dip:

| | |
|---|---|
| Reagent name | Fast Blue B salt |
| Dipping speed | 3 |
| Dipping time | 5 s |
| Reagent preparation | 1g Fast Blue B salt in 200mL water |
| Heating | none |
| Notes | Air dry for 5 minutes |

## Take image derivatized plate 1a - Visualizer (S/N: 230515):

| | |
|---|---|
| Quality | Enhanced |
| RT White | auto capture, Auto, level 85 %, Band |
| R 366 | auto capture, Auto, level 85 %, Band |
| Instrument diagnostics | Valid diagnostics |
| Documentation step label | |
| Notes | |

# System suitability tests:

## SST settings:

| | |
|---|---|
| SST tracks | |

# Data acquisition

## Application 1 - ATS 4 (S/N: 080713):

| | |
|---|---|
| Executed | 07-Jun-2019 15:48:41 visionCATSuser |

## Development 1 - Chamber:

| | |
|---|---|
| Executed | 07-Jun-2019 16:28:05 visionCATSuser |

## Take image developed plate 1a - Visualizer (S/N: 230515):

| | |
|---|---|
| Executed | 07-Jun-2019 17:18:59 visionCATSuser |

RT White Developed, RemTransVis

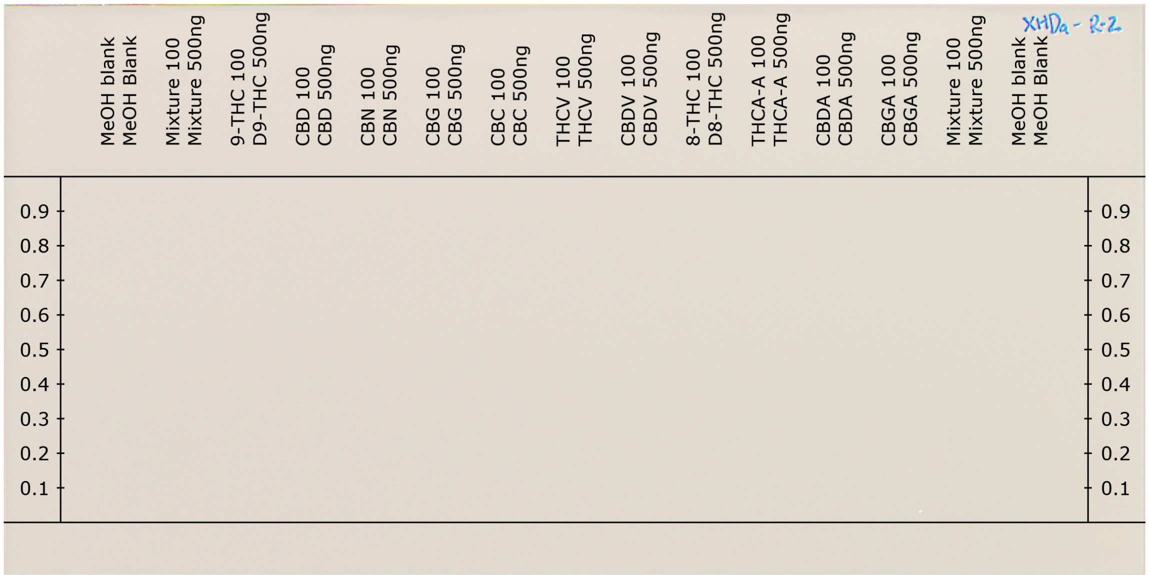


| | |
|---|---|
| Exposure | 0.095 s |
| Contrast | 1 |
| Normalized exposure | Disabled |
| Clarify | Disabled |
| White balance | 1.00, 1.00, 1.00 |

R 254 Developed, Remission254

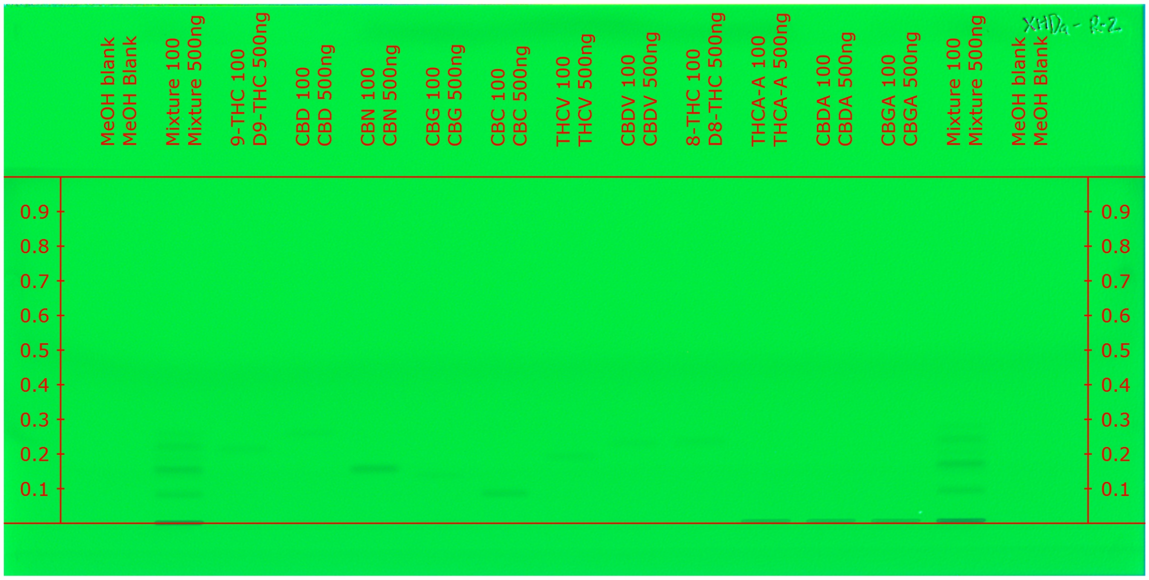


| | |
|---|---|
| Exposure | 0.272 s |
| Contrast | 1 |
| Normalized exposure | Disabled |
| Clarify | Disabled |
| White balance | 1.00, 1.00, 1.00 |

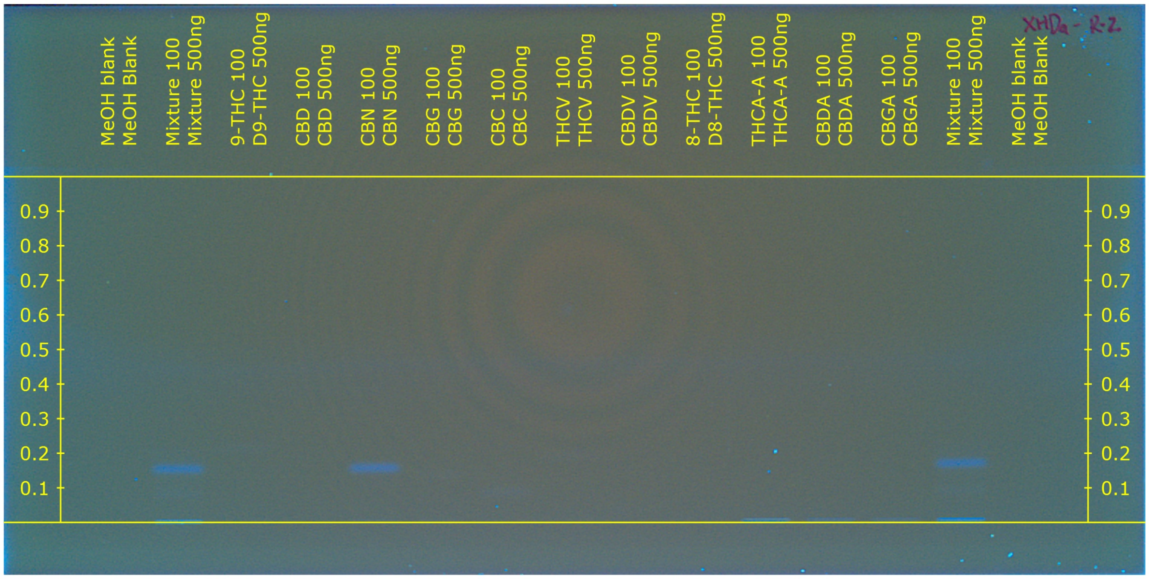


| | |
|---|---|
| Exposure | 9.999 s |
| Contrast | 1 |
| Normalized exposure | Disabled |
| Clarify | Disabled |
| White balance | 1.00, 1.00, 1.00 |

## Scan developed plate 1b - Scanner 3 (S/N: 031025):

| | |
|---|---|
| Executed | 07-Jun-2019 17:20:36 visionCATSuser |

## Scan:

| | |
|---|---|
| Wavelength | 254 nm |

## Track 1:

| | |
|---|---|
| Type | Single λ |

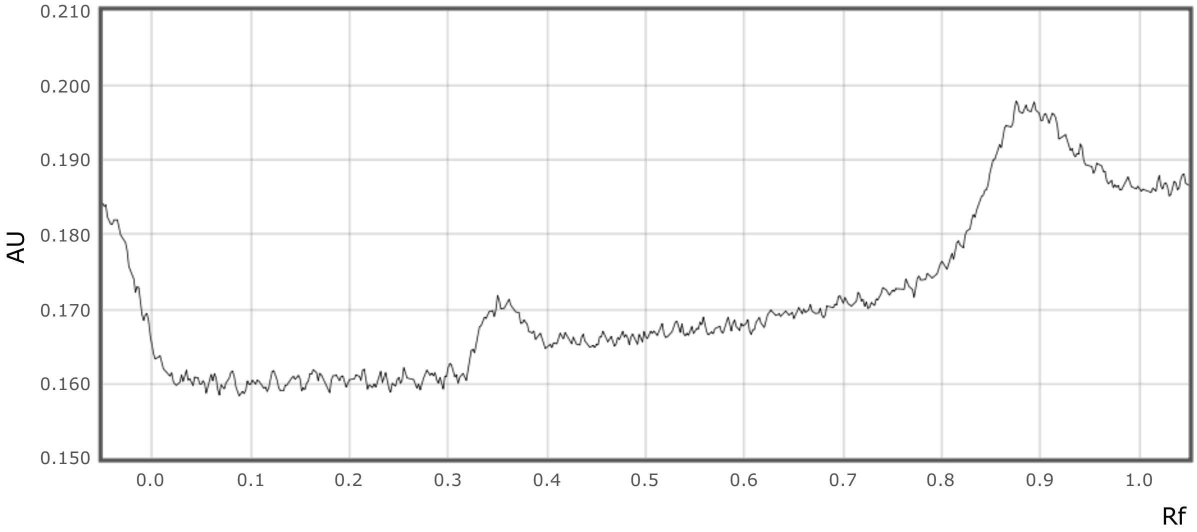

## Track 2:

| Type | Single λ |
|---|---|

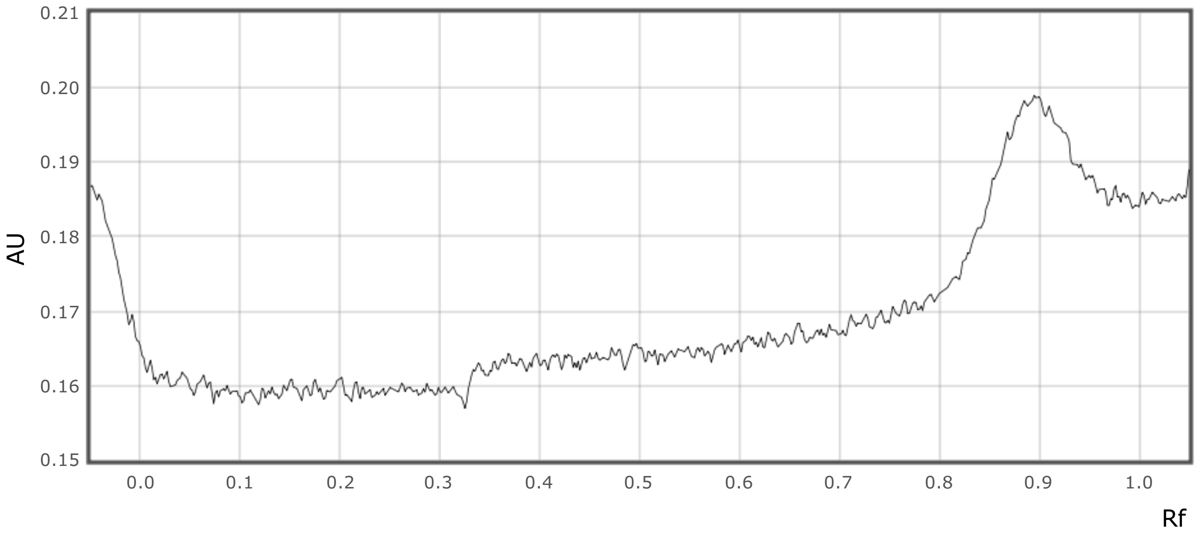


## Track 3:

| Type | Single λ |
|---|---|

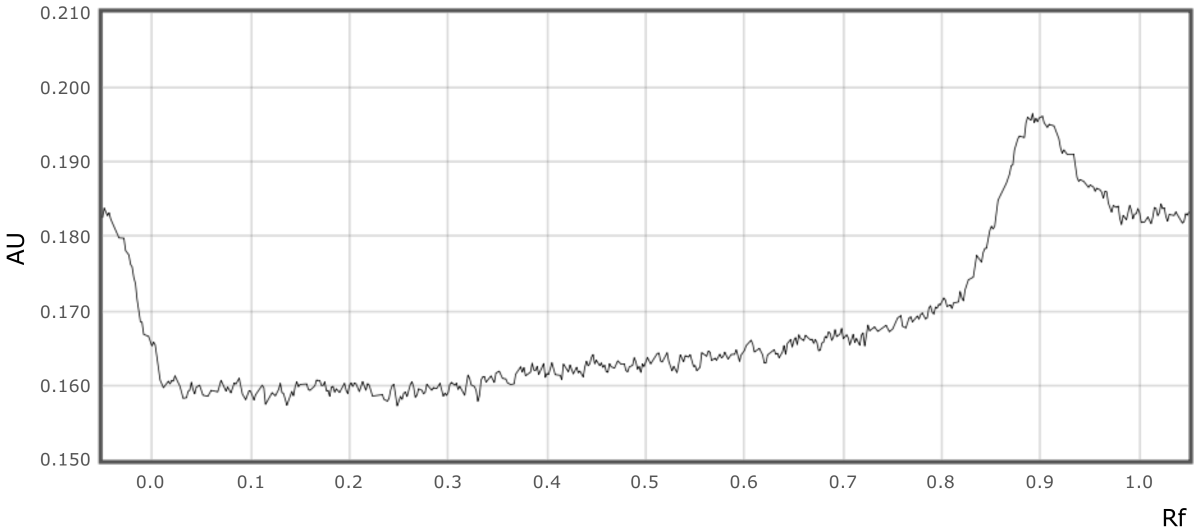


## Track 4:

| Type | Single λ |
|---|---|

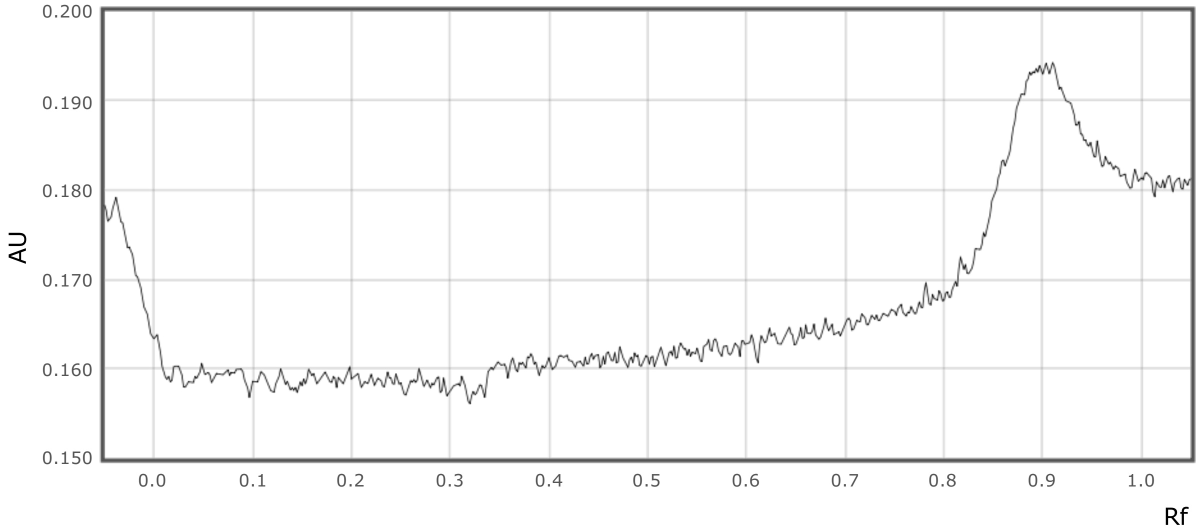


| Track 5: | |
|---|---|
| Type | Single λ |

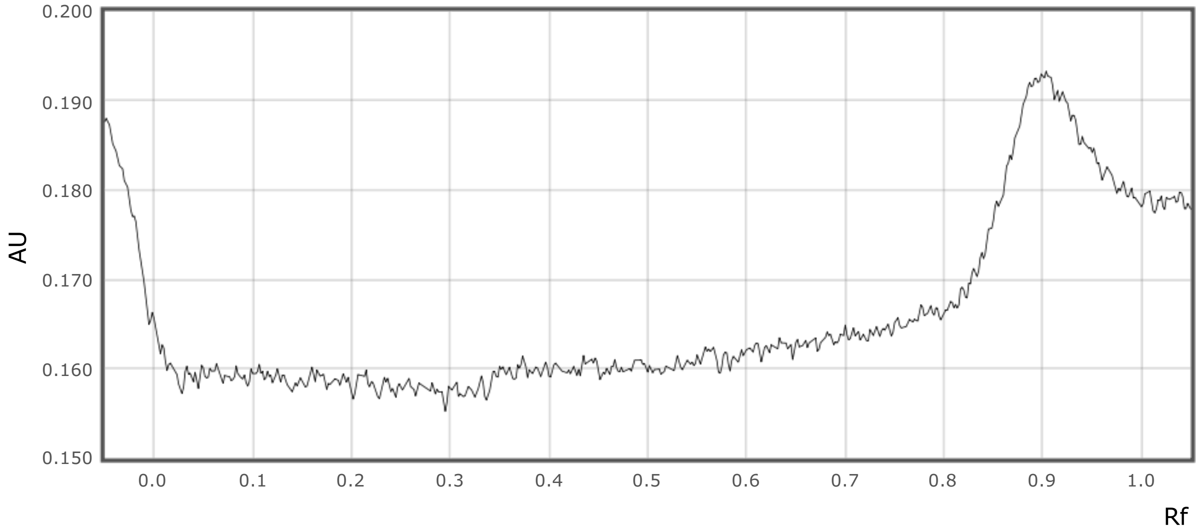


| Track 6: | |
|---|---|
| Type | Single λ |

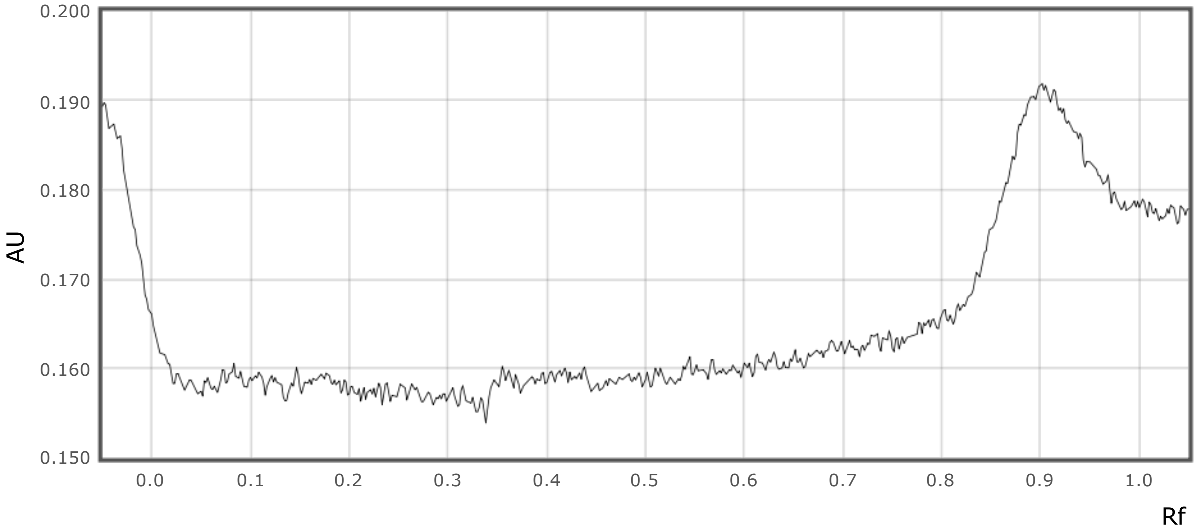


| Track 7: | |
|---|---|
| Type | Single λ |

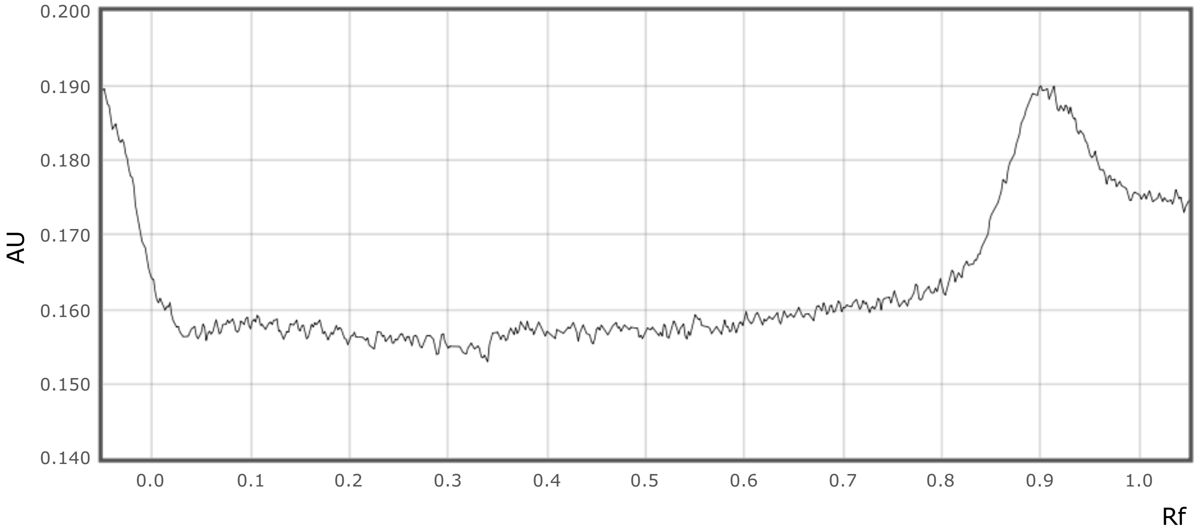


| Track 8: | |
|---|---|
| Type | Single λ |

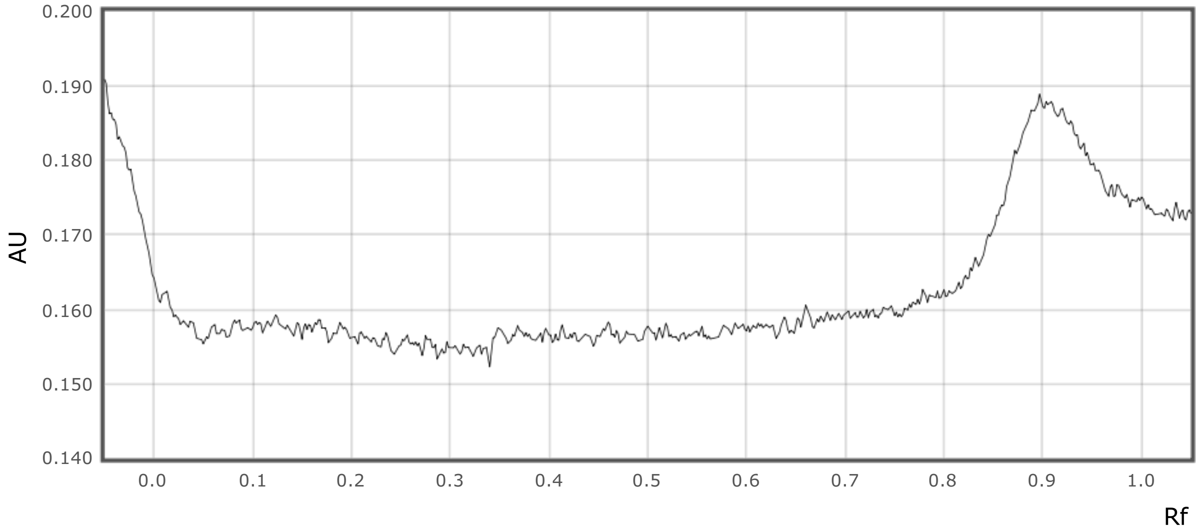


## Track 9:

| Type | Single λ |
|---|---|

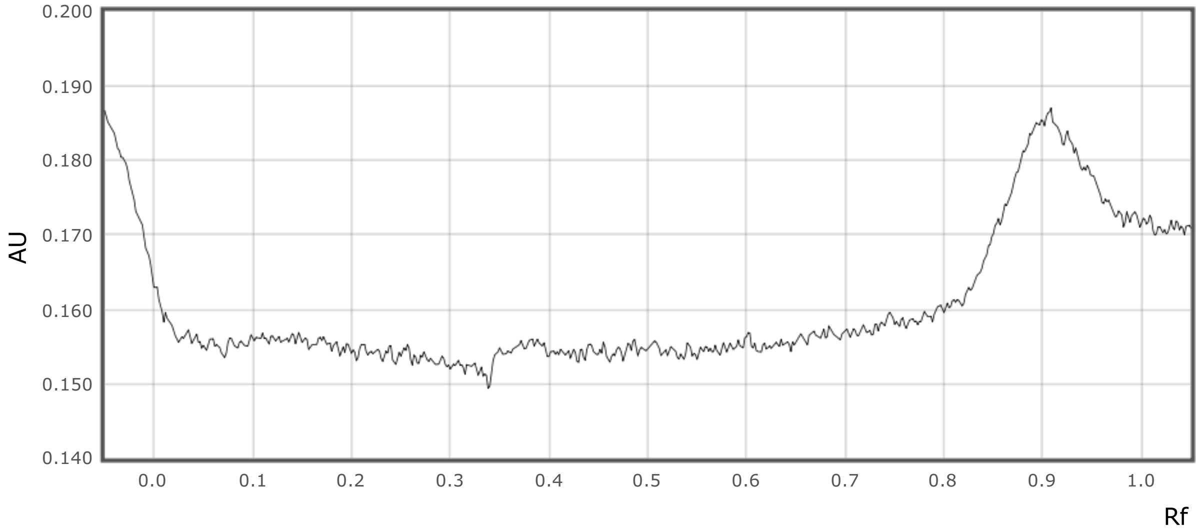


## Track 10:

| Type | Single λ |
|---|---|

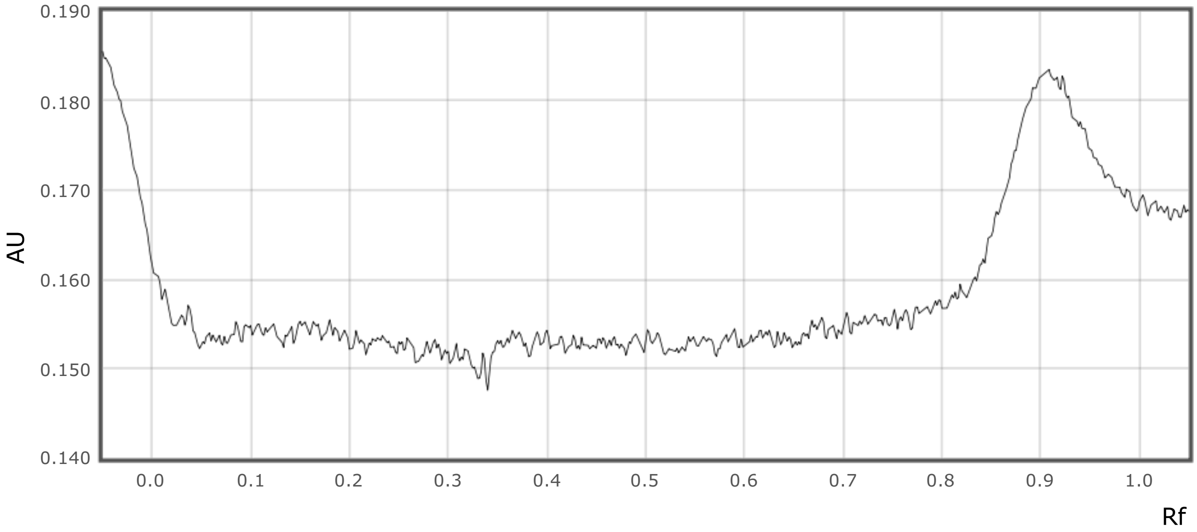


| Track 11: | |
|---|---|
| Type | Single λ |

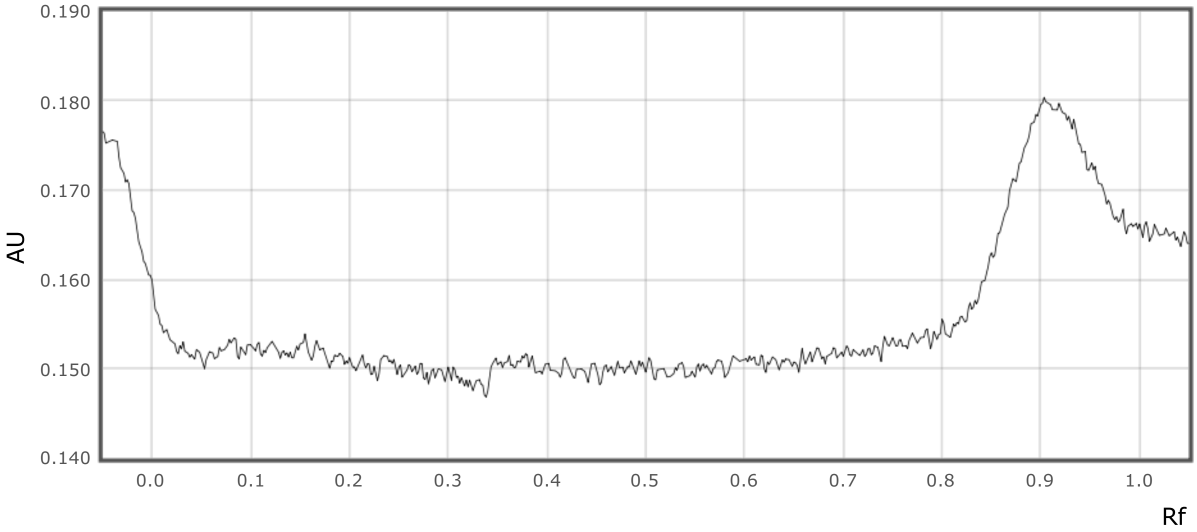


| Track 12: | |
|---|---|
| Type | Single λ |

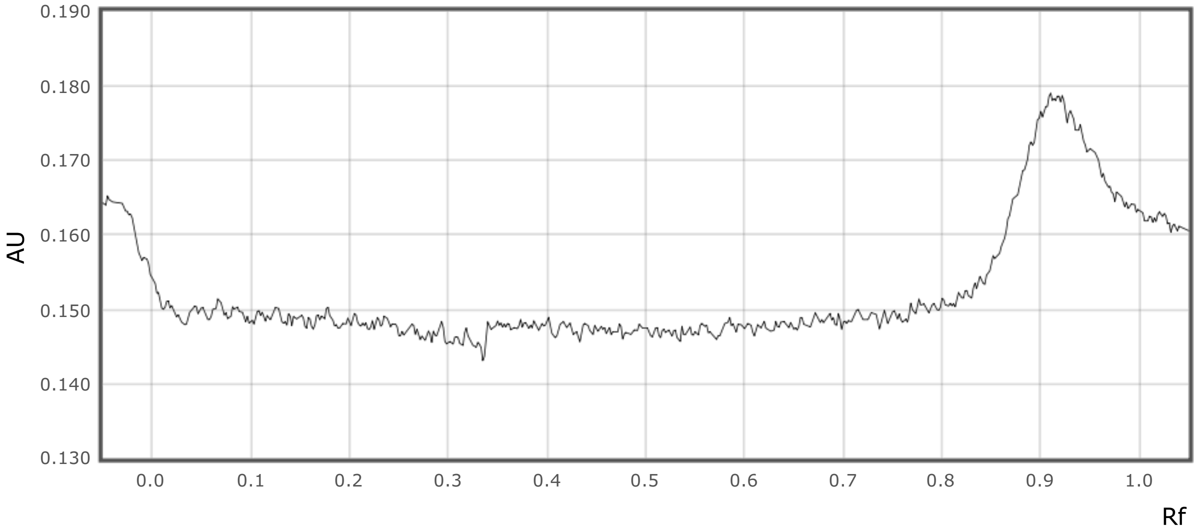


| Track 13: | |
|---|---|
| Type | Single λ |

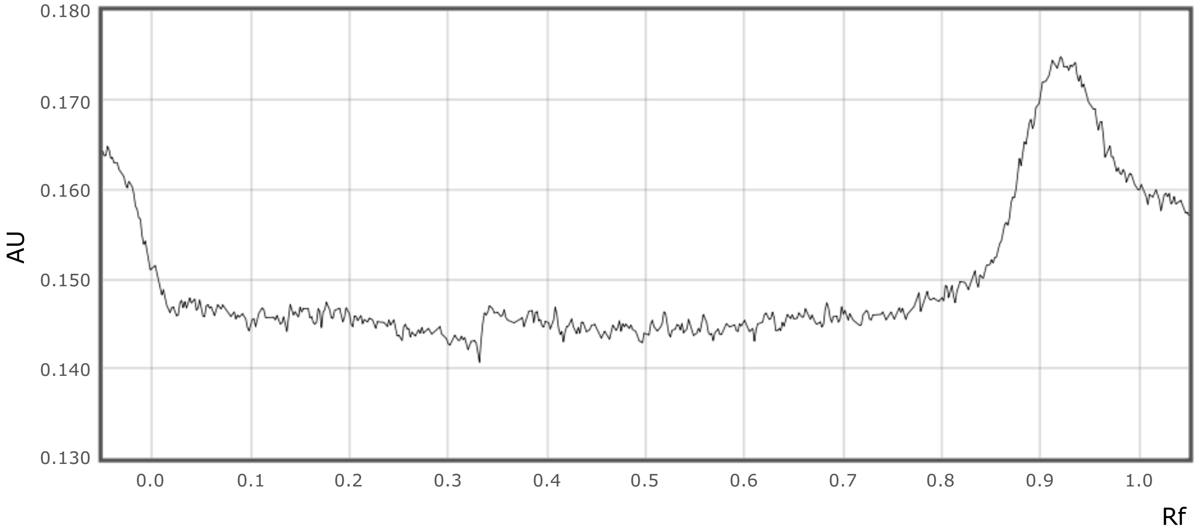


| Track 14: | |
|---|---|
| Type | Single λ |

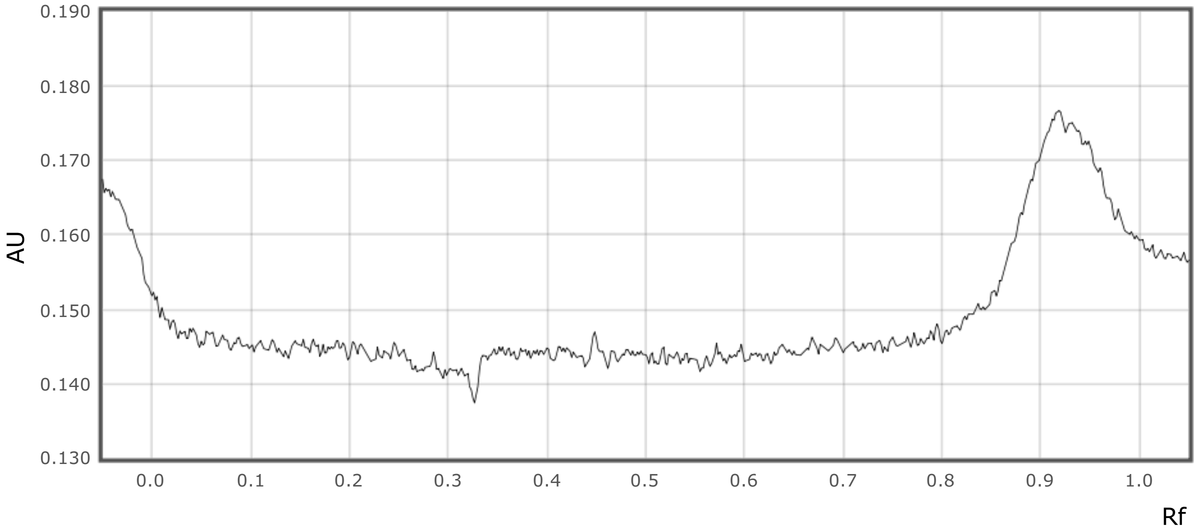


## Track 15:

| | |
|---|---|
| Type | Single λ |

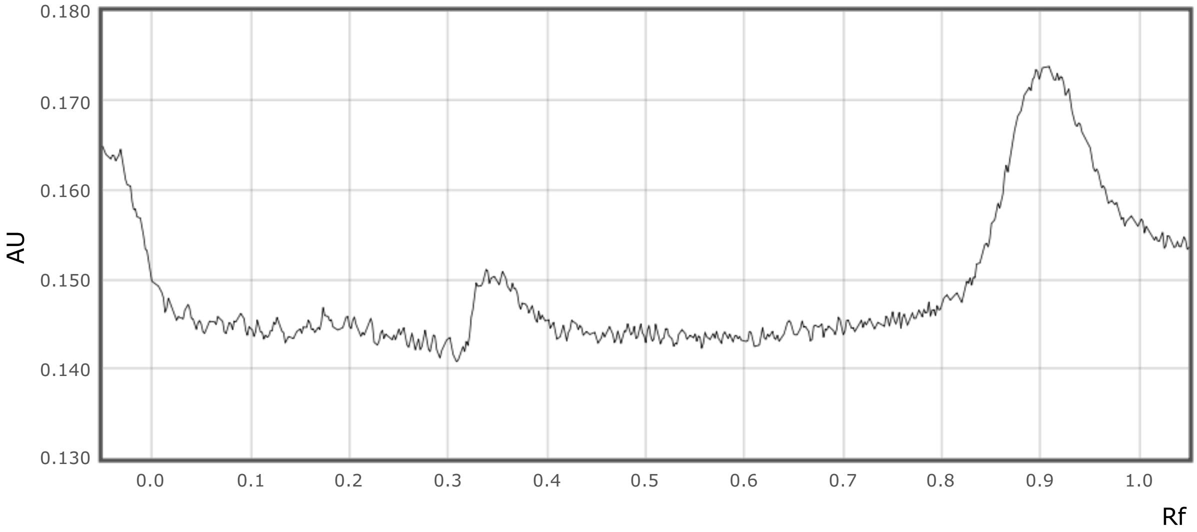


## Derivatization 1 - dip:

| | |
|---|---|
| Executed | 07-Jun-2019 17:23:18 visionCATSuser |

## Take image derivatized plate 1a - Visualizer (S/N: 230515):

| | |
|---|---|
| Executed | 07-Jun-2019 17:27:33 visionCATSuser |

RT White

Derivatized, RemTransVis

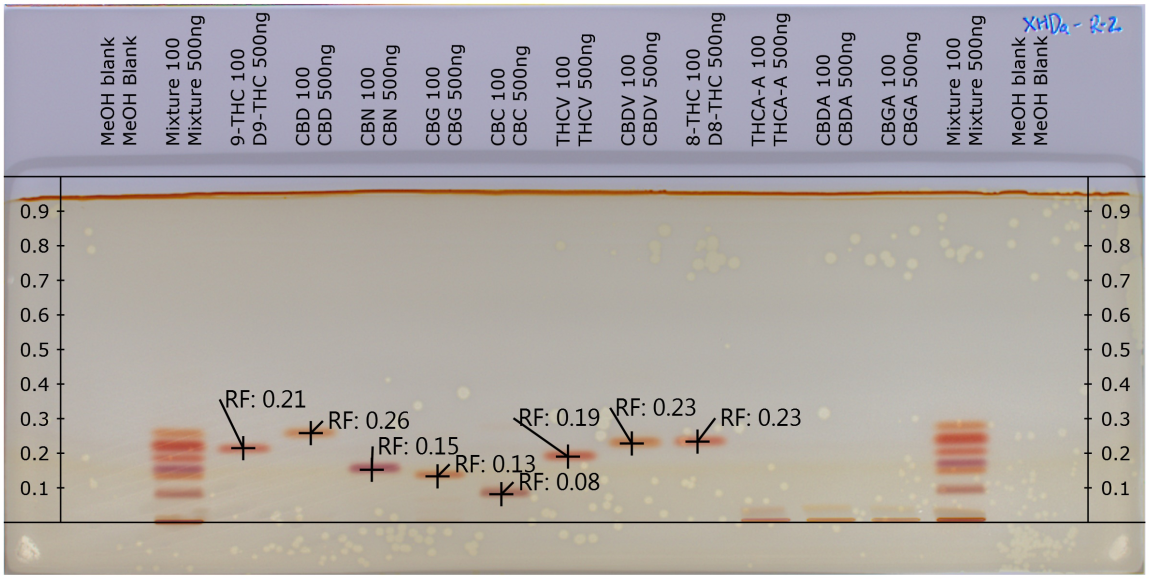


| | |
|---|---|
| Exposure | 0.054 s |
| Contrast | 1 |
| Normalized exposure | Disabled |
| Clarify | Disabled |
| White balance | 1.14, 1.10, 0.83 |

R 366

Derivatized, Remission366

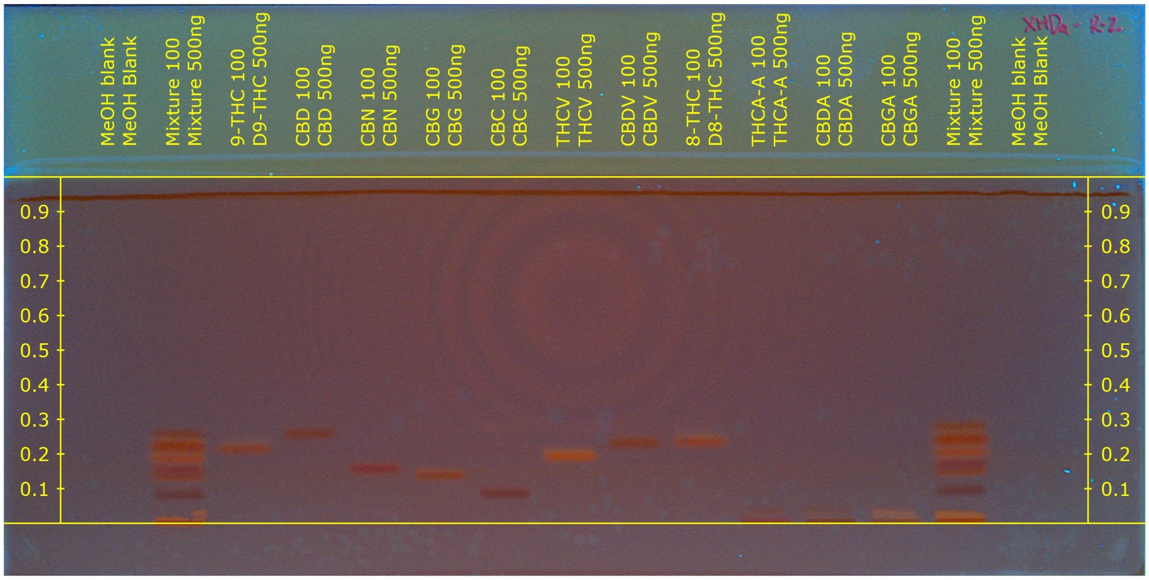


| | |
|---|---|
| Exposure | 9.999 s |
| Contrast | 1 |
| Normalized exposure | Disabled |
| Clarify | Disabled |
| White balance | 1.00, 1.00, 1.00 |

## Evaluation 1 :

| | |
|---|---|
| Validated | false |
| Step | Take image derivatized plate 1a |
| Concentration unit type | Mass / volume |
| Notes | |

# Definition:

## References:

| 9-THC 100 | | |
|---|---|---|
| Substance Name | Concentration | Purity |
| 9-THC | 100.000 µg/ml | 100.00 % |

| CBD 100 | | |
|---|---|---|
| Substance Name | Concentration | Purity |
| CBD | 100.000 µg/ml | 100.00 % |

| CBN 100 | | |
|---|---|---|
| Substance Name | Concentration | Purity |
| CBN | 100.000 µg/ml | 100.00 % |

| CBG 100 | | |
|---|---|---|
| Substance Name | Concentration | Purity |
| CBG | 100.000 µg/ml | 100.00 % |

| CBC 100 | | |
|---|---|---|
| Substance Name | Concentration | Purity |
| CBC | 100.000 µg/ml | 100.00 % |

| THCV 100 | | |
|---|---|---|
| Substance Name | Concentration | Purity |
| THCV | 100.000 µg/ml | 100.00 % |

| CBDV 100 | | |
|---|---|---|
| Substance Name | Concentration | Purity |
| CBDV | 100.000 µg/ml | 100.00 % |

| 8-THC 100 | | |
|---|---|---|
| Substance Name | Concentration | Purity |
| 8-THC | 100.000 µg/ml | 100.00 % |

| THCA-A 100 | | |
|---|---|---|
| Substance Name | Concentration | Purity |
| THCA-A | 100.000 µg/ml | 100.00 % |

| CBDA 100 | | |
|---|---|---|
| Substance Name | Concentration | Purity |
| CBDA | 100.000 µg/ml | 100.00 % |

| CBGA 100 | | |
|---|---|---|
| Substance Name | Concentration | Purity |
| CBGA | 100.000 µg/ml | 100.00 % |

## Samples:

| Vial ID | Amount | Volume solution | Reference amount | Related to |
|---|---|---|---|---|
| MeOH blank | | 0.00 ml | | |
| Mixture 100 | | 0.00 ml | | |

## Integration parameters:

| | |
|---|---|
| Bounds | [0.000,1.000] |
| Smoothing | Savitzky–Golay of order 3 and window 7 |
| Baseline correction | Lowest slope with noise 0.05 |
| Profile subtraction | Profile subtraction from track 1 |
| Peaks detection | Gauss (legacy) with sensitivity 0.1, separation 1 and threshold 0.1 |

## Scan:

| | |
|---|---|
| Wavelength | RT White |

## Track 1:

| | |
|---|---|
| Type | Sample |
| Vial ID | MeOH blank |
| Description | MeOH Blank |
| Volume | 2.0 µl |

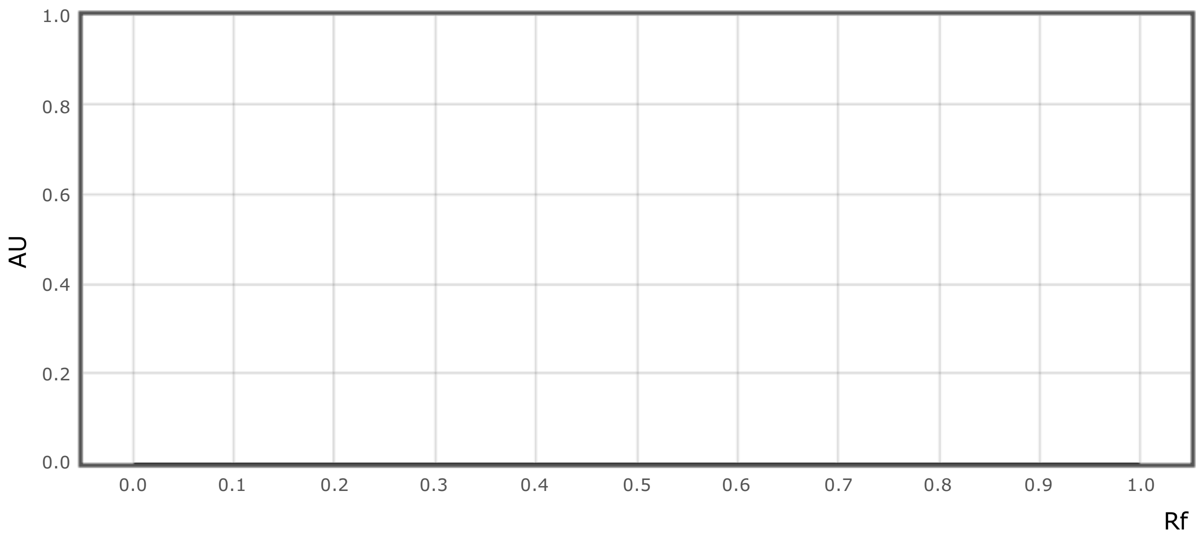


| Peak # | Start | | Max | | | End | | Area | | Manual peak | Substance Name |
|---|---|---|---|---|---|---|---|---|---|---|---|
| | Rf | H | Rf | H | % | Rf | H | A | % | | |

## Track 2:

| | |
|---|---|
| Type | Sample |
| Vial ID | Mixture 100 |
| Description | Mixture 500ng |
| Volume | 5.0 µl |

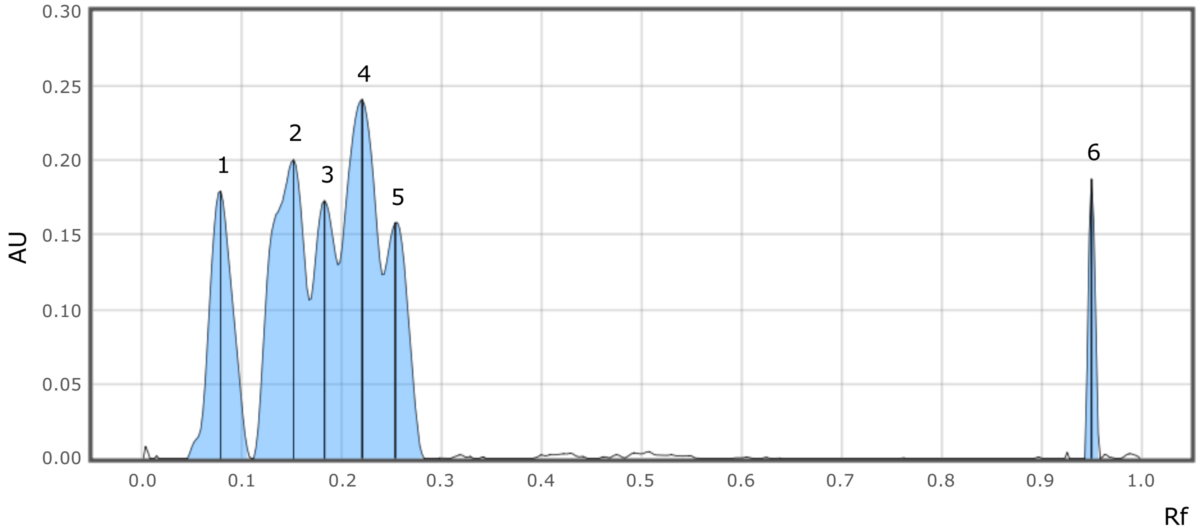


| Peak # | Start | | Max | | | End | | Area | | Manual peak | Substance Name |
|---|---|---|---|---|---|---|---|---|---|---|---|
| | Rf | H | Rf | H | % | Rf | H | A | % | | |
| 1 | 0.045 | 0.0000 | 0.078 | 0.1793 | 15.75 | 0.107 | 0.0009 | 0.00483 | 15.71 | No | |
| 2 | 0.109 | 0.0000 | 0.151 | 0.2003 | 17.59 | 0.167 | 0.1062 | 0.00760 | 24.69 | No | |
| 3 | 0.167 | 0.1062 | 0.182 | 0.1728 | 15.18 | 0.196 | 0.1297 | 0.00422 | 13.72 | No | |
| 4 | 0.196 | 0.1297 | 0.220 | 0.2407 | 21.14 | 0.240 | 0.1232 | 0.00846 | 27.50 | No | |
| 5 | 0.240 | 0.1232 | 0.253 | 0.1581 | 13.89 | 0.282 | 0.0000 | 0.00421 | 13.68 | No | |
| 6 | 0.944 | 0.0000 | 0.950 | 0.1874 | 16.46 | 0.959 | 0.0000 | 0.00145 | 4.70 | No | |

## Track 3:

| | |
|---|---|
| Type | Reference |
| Vial ID | 9-THC 100 |
| Description | D9-THC 500ng |
| Volume | 5.0 µl |

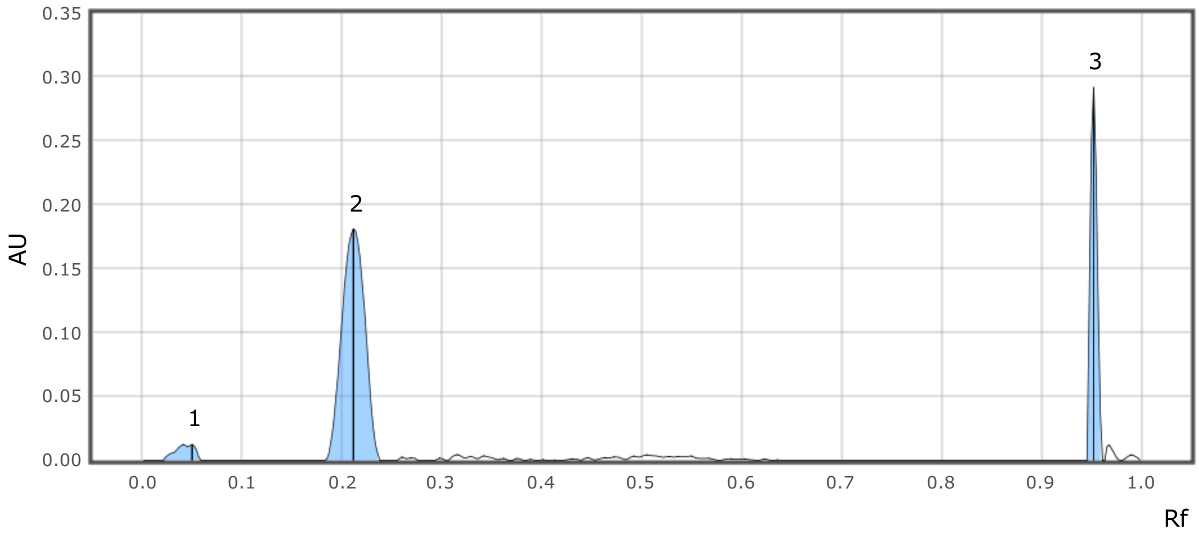

| Peak | Start | | Max | | | End | | Area | | Manual | Substance |
|---|---|---|---|---|---|---|---|---|---|---|---|
| # | Rf | H | Rf | H | % | Rf | H | A | % | peak | Name |
| 1 | 0.021 | 0.0000 | 0.050 | 0.0125 | 2.57 | 0.058 | 0.0000 | 0.00029 | 3.95 | No | |
| 2 | 0.182 | 0.0000 | 0.211 | 0.1809 | 37.30 | 0.238 | 0.0000 | 0.00485 | 64.99 | No | 9-THC |
| 3 | 0.946 | 0.0000 | 0.953 | 0.2916 | 60.12 | 0.961 | 0.0000 | 0.00232 | 31.07 | No | |

## Track 4:

| | |
|---|---|
| Type | Reference |
| Vial ID | CBD 100 |
| Description | CBD 500ng |
| Volume | 5.0 µl |

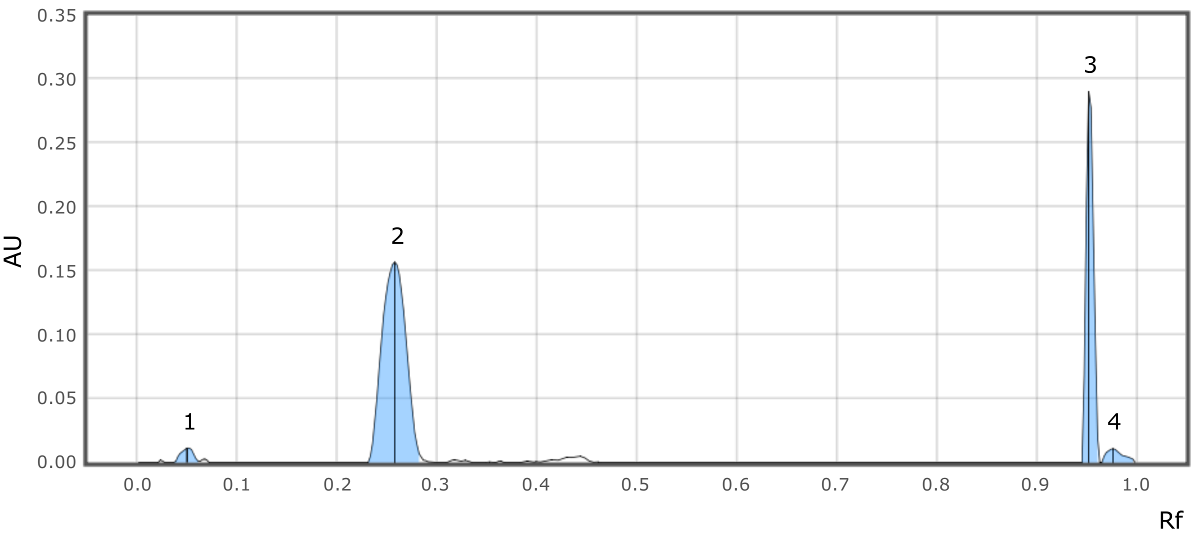


| Peak | Start | | Max | | | End | | Area | | Manual | Substance |
|---|---|---|---|---|---|---|---|---|---|---|---|
| # | Rf | H | Rf | H | % | Rf | H | A | % | peak | Name |
| 1 | 0.036 | 0.0000 | 0.050 | 0.0112 | 2.38 | 0.063 | 0.0010 | 0.00017 | 2.27 | No | |
| 2 | 0.231 | 0.0000 | 0.258 | 0.1567 | 33.44 | 0.283 | 0.0068 | 0.00440 | 60.15 | Yes | CBD |
| 3 | 0.946 | 0.0000 | 0.953 | 0.2900 | 61.90 | 0.964 | 0.0000 | 0.00254 | 34.78 | No | |
| 4 | 0.966 | 0.0000 | 0.977 | 0.0107 | 2.28 | 0.999 | 0.0000 | 0.00020 | 2.80 | No | |

## Track 5:

| | |
|---|---|
| Type | Reference |
| Vial ID | CBN 100 |
| Description | CBN 500ng |
| Volume | 5.0 µl |

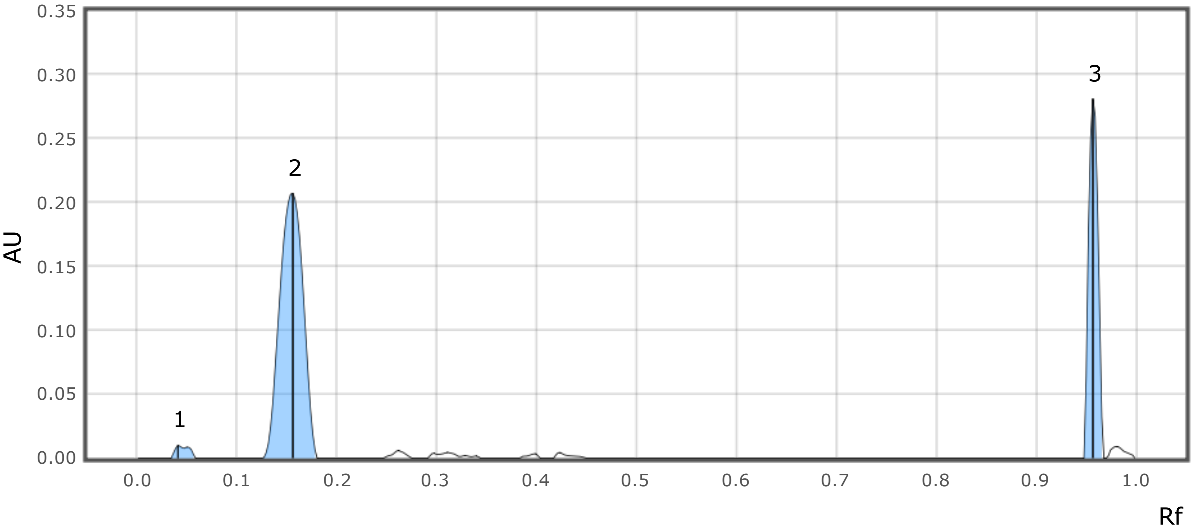


| Peak | Start | | Max | | | End | | Area | | Manual | Substance |
|---|---|---|---|---|---|---|---|---|---|---|---|
| # | Rf | H | Rf | H | % | Rf | H | A | % | peak | Name |
| 1 | 0.034 | 0.0000 | 0.041 | 0.0100 | 2.01 | 0.058 | 0.0000 | 0.00016 | 1.82 | No | |
| 2 | 0.125 | 0.0000 | 0.156 | 0.2071 | 41.57 | 0.180 | 0.0000 | 0.00564 | 63.55 | No | CBN |
| 3 | 0.948 | 0.0000 | 0.957 | 0.2811 | 56.42 | 0.968 | 0.0000 | 0.00307 | 34.63 | No | |

## Track 6:

| | |
|---|---|
| Type | Reference |
| Vial ID | CBG 100 |
| Description | CBG 500ng |
| Volume | 5.0 µl |

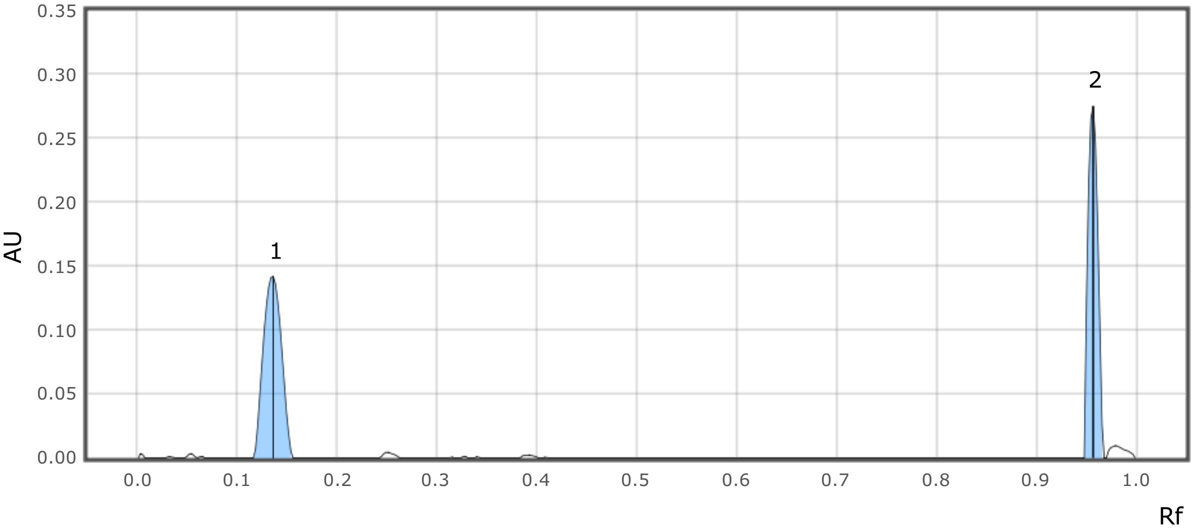


| Peak | Start | | Max | | | End | | Area | | Manual | Substance |
|---|---|---|---|---|---|---|---|---|---|---|---|
| # | Rf | H | Rf | H | % | Rf | H | A | % | peak | Name |
| 1 | 0.116 | 0.0000 | 0.136 | 0.1420 | 34.04 | 0.156 | 0.0000 | 0.00300 | 48.61 | No | CBG |
| 2 | 0.948 | 0.0000 | 0.957 | 0.2750 | 65.96 | 0.968 | 0.0000 | 0.00317 | 51.39 | No | |

## Track 7:

| | |
|---|---|
| Type | Reference |
| Vial ID | CBC 100 |
| Description | CBC 500ng |
| Volume | 5.0 µl |

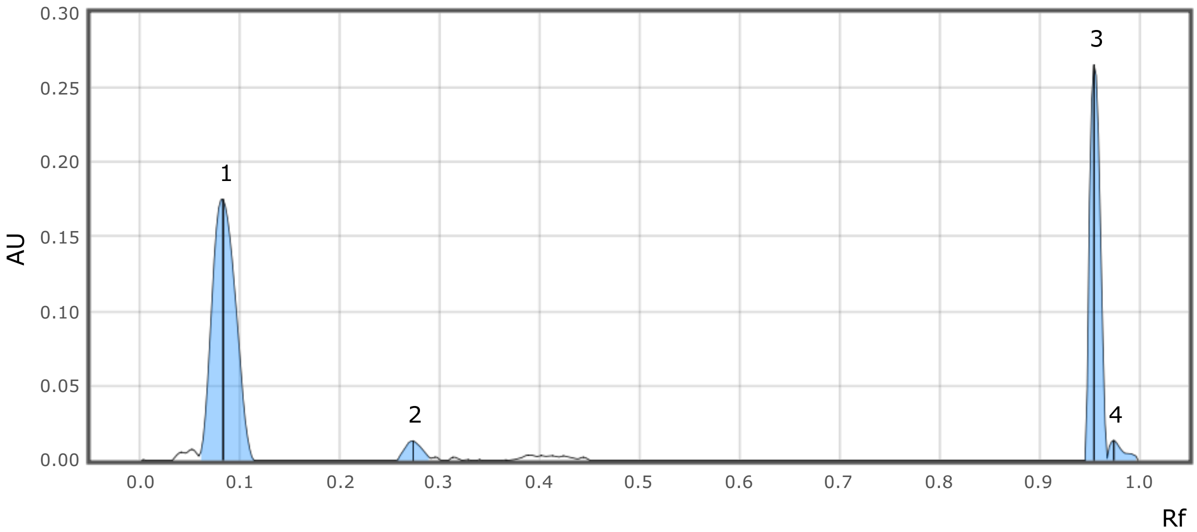


| Peak | Start | | Max | | | End | | Area | | Manual | Substance |
|---|---|---|---|---|---|---|---|---|---|---|---|
| # | Rf | H | Rf | H | % | Rf | H | A | % | peak | Name |
| 1 | 0.060 | 0.0057 | 0.083 | 0.1748 | 37.50 | 0.114 | 0.0000 | 0.00479 | 56.35 | Yes | CBC |
| 2 | 0.255 | 0.0000 | 0.273 | 0.0131 | 2.80 | 0.291 | 0.0014 | 0.00026 | 3.01 | No | |
| 3 | 0.946 | 0.0000 | 0.955 | 0.2649 | 56.83 | 0.968 | 0.0013 | 0.00324 | 38.15 | No | |
| 4 | 0.968 | 0.0013 | 0.975 | 0.0134 | 2.87 | 0.999 | 0.0000 | 0.00021 | 2.50 | No | |

## Track 8:

| | |
|---|---|
| Type | Reference |
| Vial ID | THCV 100 |
| Description | THCV 500ng |
| Volume | 5.0 µl |

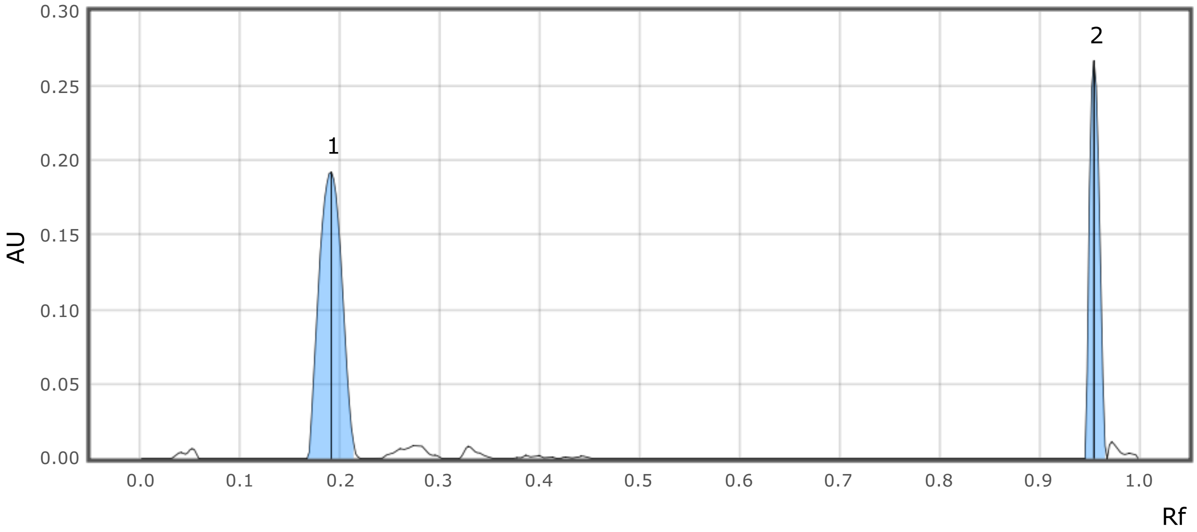


| Peak # | Start | | Max | | | End | | Area | | Manual peak | Substance Name |
|---|---|---|---|---|---|---|---|---|---|---|---|
| | Rf | H | Rf | H | % | Rf | H | A | % | | |
| 1 | 0.167 | 0.0000 | 0.191 | 0.1920 | 41.86 | 0.215 | 0.0030 | 0.00512 | 62.48 | Yes | THCV |
| 2 | 0.946 | 0.0000 | 0.955 | 0.2666 | 58.14 | 0.968 | 0.0000 | 0.00308 | 37.52 | No | |

## Track 9:

| | |
|---|---|
| Type | Reference |
| Vial ID | CBDV 100 |
| Description | CBDV 500ng |
| Volume | 5.0 µl |

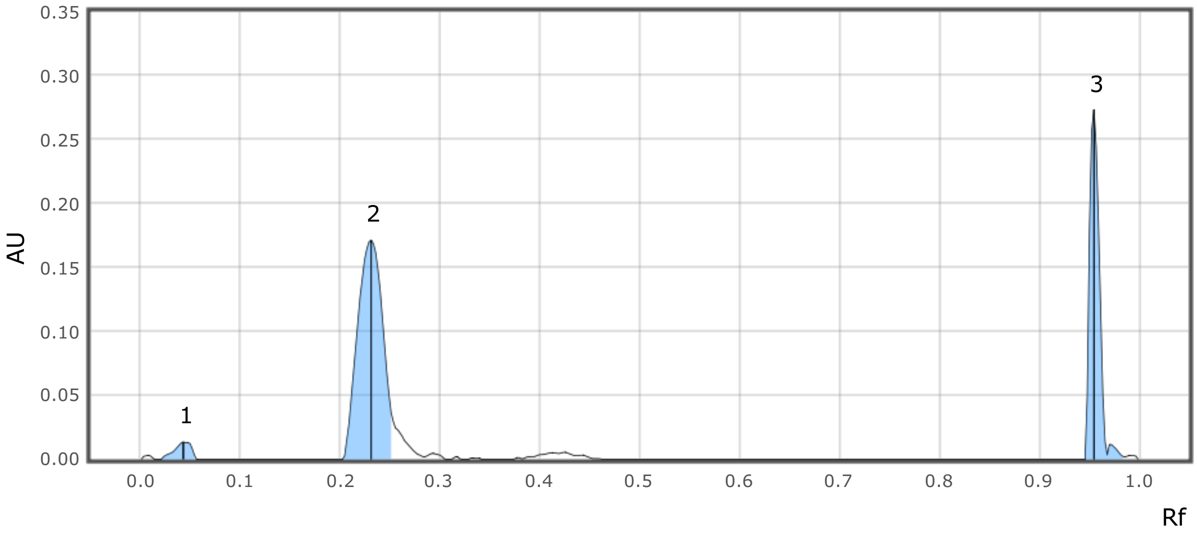


| Peak # | Start | | Max | | | End | | Area | | Manual peak | Substance Name |
|---|---|---|---|---|---|---|---|---|---|---|---|
| | Rf | H | Rf | H | % | Rf | H | A | % | | |
| 1 | 0.021 | 0.0000 | 0.043 | 0.0135 | 2.95 | 0.056 | 0.0000 | 0.00027 | 3.20 | No | |
| 2 | 0.202 | 0.0000 | 0.231 | 0.1714 | 37.41 | 0.252 | 0.0294 | 0.00502 | 59.46 | Yes | CBDV |
| 3 | 0.946 | 0.0000 | 0.955 | 0.2733 | 59.64 | 0.986 | 0.0018 | 0.00316 | 37.35 | No | |

## Track 10:

| | |
|---|---|
| Type | Reference |
| Vial ID | 8-THC 100 |
| Description | D8-THC 500ng |
| Volume | 5.0 µl |

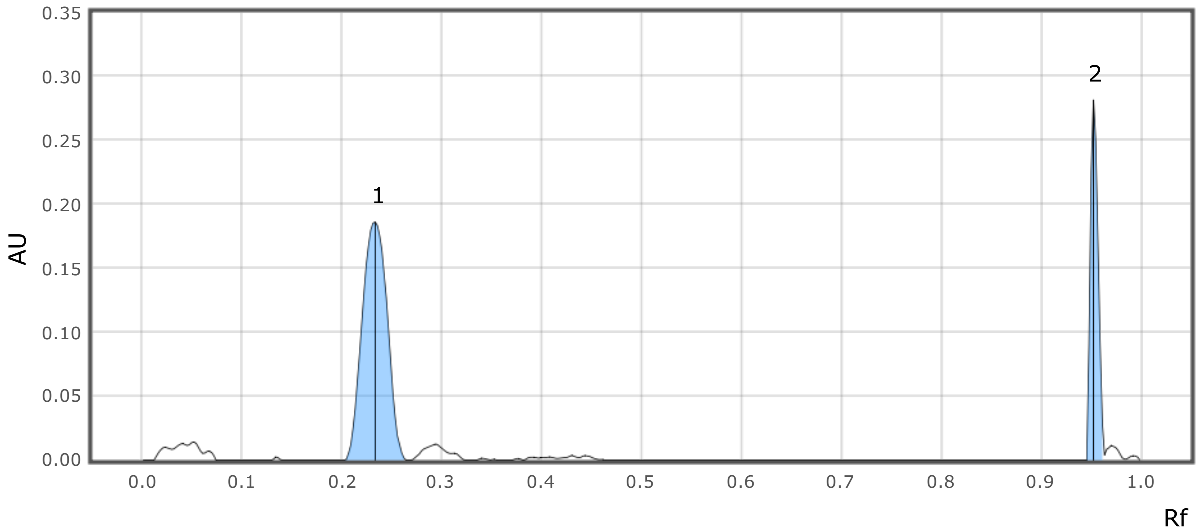


| Peak | Start | | Max | | | End | | Area | | Manual | Substance |
|---|---|---|---|---|---|---|---|---|---|---|---|
| # | Rf | H | Rf | H | % | Rf | H | A | % | peak | Name |
| 1 | 0.202 | 0.0000 | 0.233 | 0.1861 | 39.81 | 0.264 | 0.0000 | 0.00540 | 68.14 | No | 8-THC |
| 2 | 0.946 | 0.0000 | 0.953 | 0.2814 | 60.19 | 0.964 | 0.0040 | 0.00252 | 31.86 | No | |

## Track 11:

| | |
|---|---|
| Type | Reference |
| Vial ID | THCA-A 100 |
| Description | THCA-A 500ng |
| Volume | 5.0 µl |

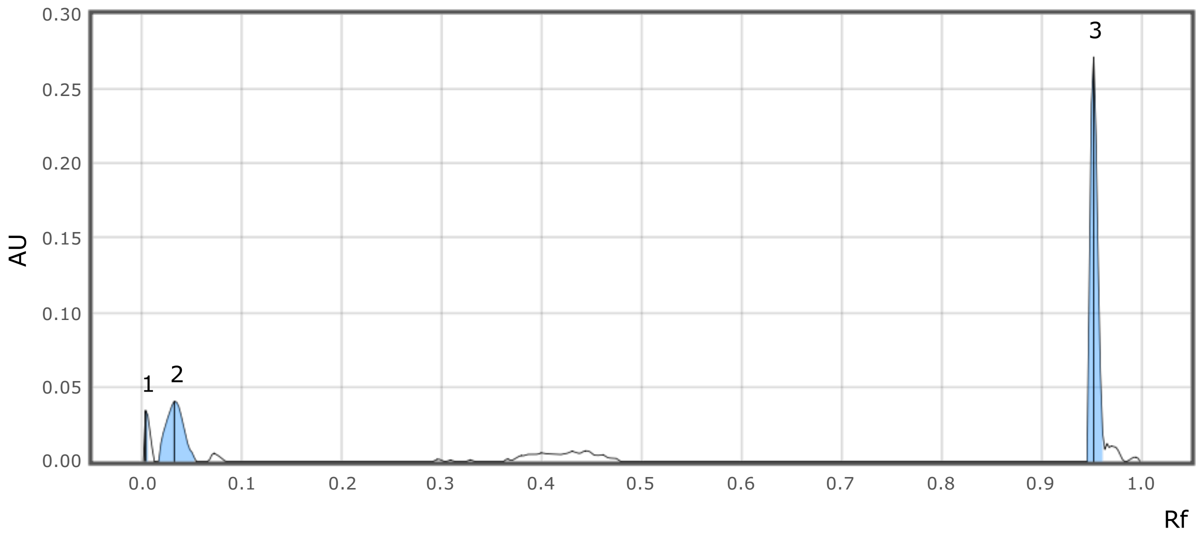

| Peak # | Start | | Max | | | End | | Area | | Manual peak | Substance Name |
|---|---|---|---|---|---|---|---|---|---|---|---|
| | Rf | H | Rf | H | % | Rf | H | A | % | | |
| 1 | 0.001 | 0.0000 | 0.003 | 0.0341 | 9.87 | 0.010 | 0.0088 | 0.00020 | 5.92 | Yes | THCA-A |
| 2 | 0.014 | 0.0000 | 0.032 | 0.0403 | 11.66 | 0.054 | 0.0000 | 0.00083 | 24.56 | No | |
| 3 | 0.946 | 0.0000 | 0.953 | 0.2712 | 78.46 | 0.964 | 0.0081 | 0.00235 | 69.52 | No | |

## Track 12:

| | |
|---|---|
| Type | Reference |
| Vial ID | CBDA 100 |
| Description | CBDA 500ng |
| Volume | 5.0 µl |

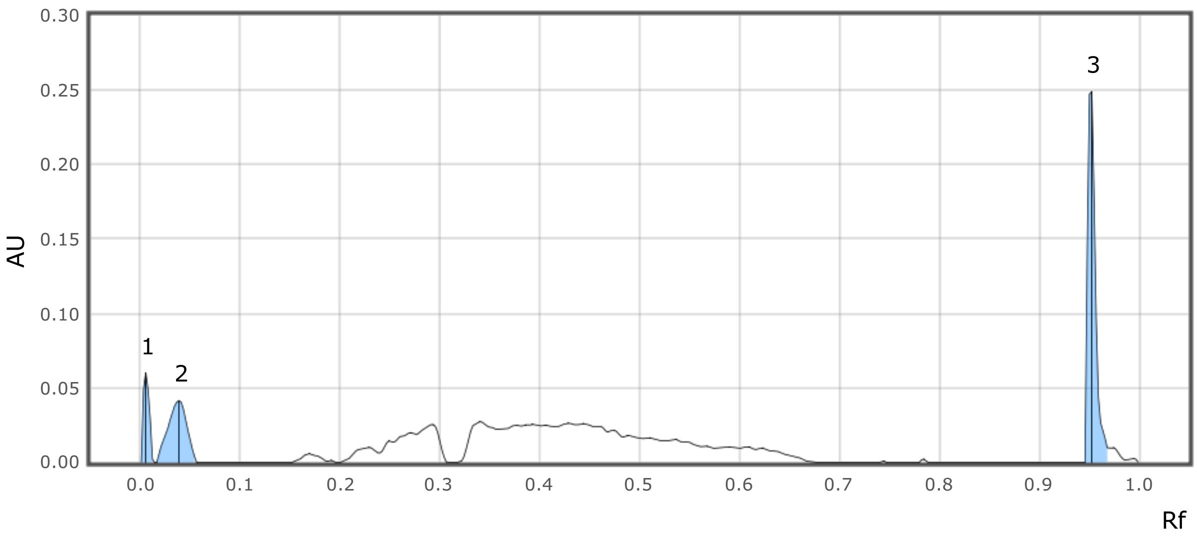


| Peak # | Start | | Max | | | End | | Area | | Manual peak | Substance Name |
|---|---|---|---|---|---|---|---|---|---|---|---|
| | Rf | H | Rf | H | % | Rf | H | A | % | | |
| 1 | 0.001 | 0.0000 | 0.005 | 0.0601 | 17.15 | 0.014 | 0.0000 | 0.00042 | 11.50 | No | CBDA |
| 2 | 0.016 | 0.0000 | 0.039 | 0.0415 | 11.84 | 0.056 | 0.0000 | 0.00091 | 24.85 | No | |
| 3 | 0.946 | 0.0000 | 0.953 | 0.2486 | 71.00 | 0.973 | 0.0096 | 0.00232 | 63.64 | No | |

## Track 13:

| | |
|---|---|
| Type | Reference |
| Vial ID | CBGA 100 |
| Description | CBGA 500ng |
| Volume | 5.0 µl |

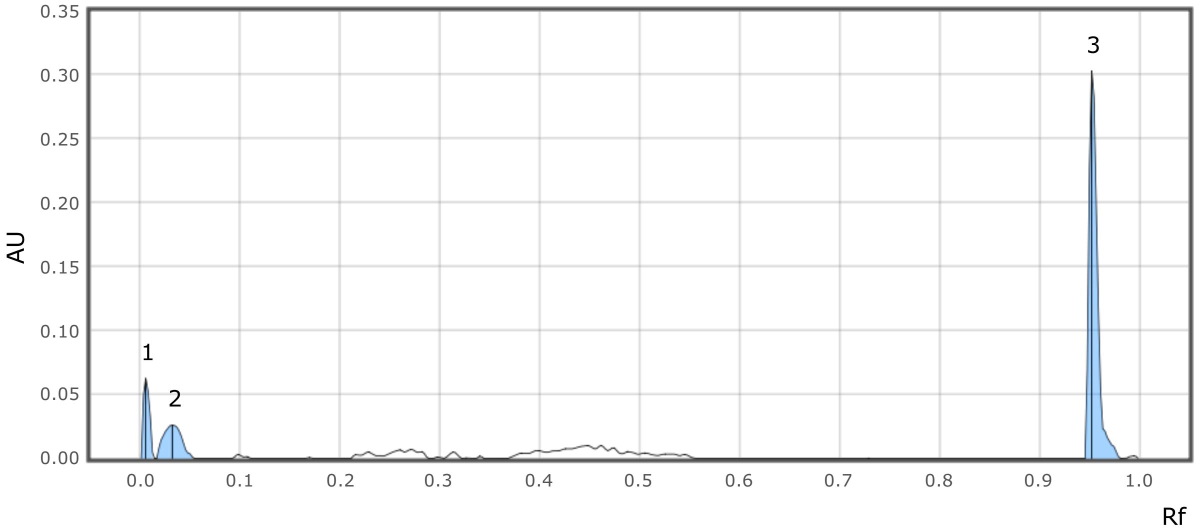


| Peak # | Start | | Max | | | End | | Area | | Manual peak | Substance Name |
|---|---|---|---|---|---|---|---|---|---|---|---|
| | Rf | H | Rf | H | % | Rf | H | A | % | | |
| 1 | 0.001 | 0.0000 | 0.005 | 0.0627 | 16.00 | 0.014 | 0.0000 | 0.00045 | 11.19 | No | CBGA |
| 2 | 0.016 | 0.0000 | 0.032 | 0.0258 | 6.60 | 0.054 | 0.0000 | 0.00056 | 14.04 | No | |
| 3 | 0.946 | 0.0000 | 0.953 | 0.3030 | 77.39 | 0.981 | 0.0000 | 0.00300 | 74.76 | No | |

## Track 14:

| | |
|---|---|
| Type | Sample |
| Vial ID | Mixture 100 |
| Description | Mixture 500ng |
| Volume | 5.0 µl |

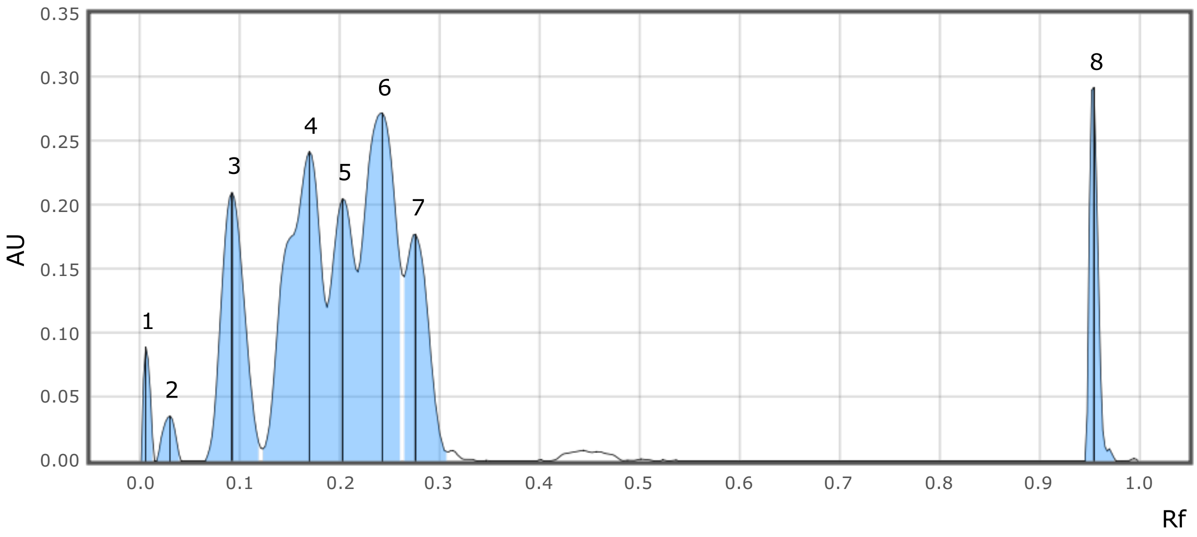

| Peak # | Start Rf | Start H | Max Rf | Max H | Max % | End Rf | End H | Area A | Area % | Manual peak | Substance Name |
|---|---|---|---|---|---|---|---|---|---|---|---|
| 1 | 0.001 | 0.0000 | 0.005 | 0.0891 | 5.85 | 0.014 | 0.0000 | 0.00068 | 1.73 | No | |
| 2 | 0.016 | 0.0000 | 0.030 | 0.0352 | 2.31 | 0.041 | 0.0000 | 0.00051 | 1.29 | No | |
| 3 | 0.065 | 0.0000 | 0.092 | 0.2098 | 13.78 | 0.120 | 0.0102 | 0.00562 | 14.35 | No | |
| 4 | 0.123 | 0.0094 | 0.169 | 0.2420 | 15.89 | 0.187 | 0.1201 | 0.00973 | 24.84 | No | |
| 5 | 0.187 | 0.1201 | 0.202 | 0.2050 | 13.46 | 0.218 | 0.1477 | 0.00533 | 13.60 | No | |
| 6 | 0.218 | 0.1477 | 0.242 | 0.2722 | 17.88 | 0.262 | 0.1458 | 0.00984 | 25.13 | No | |
| 7 | 0.264 | 0.1442 | 0.275 | 0.1774 | 11.65 | 0.309 | 0.0071 | 0.00455 | 11.62 | No | |
| 8 | 0.946 | 0.0000 | 0.955 | 0.2921 | 19.18 | 0.977 | 0.0000 | 0.00291 | 7.44 | No | |

## Track 15:

| | |
|---|---|
| Type | Sample |
| Vial ID | MeOH blank |
| Description | MeOH Blank |
| Volume | 2.0 µl |

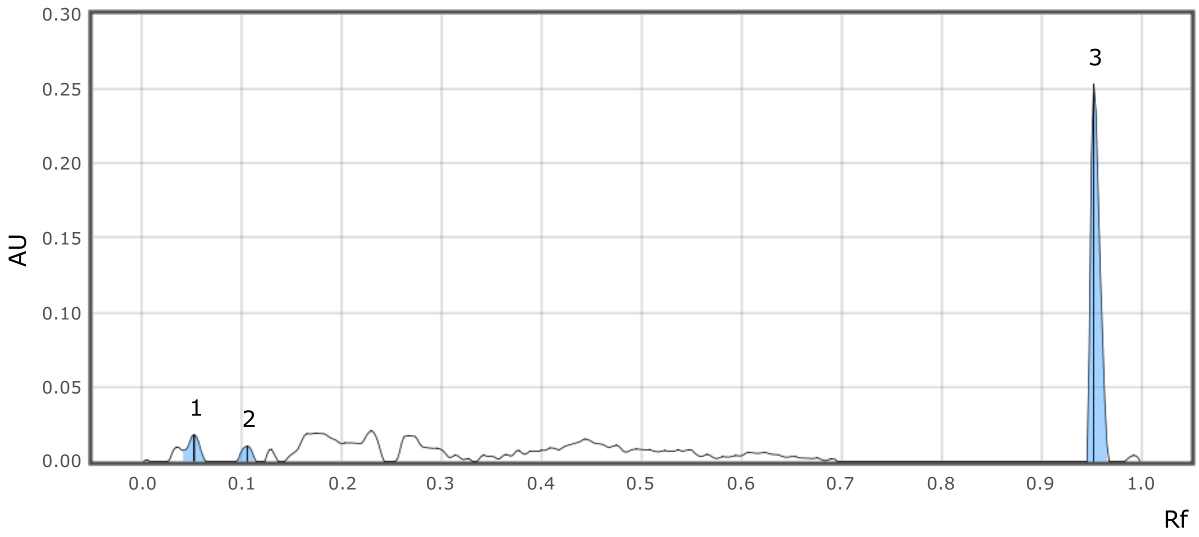


| Peak # | Start Rf | Start H | Max Rf | Max H | Max % | End Rf | End H | Area A | Area % | Manual peak | Substance Name |
|---|---|---|---|---|---|---|---|---|---|---|---|
| 1 | 0.041 | 0.0074 | 0.052 | 0.0178 | 6.34 | 0.065 | 0.0000 | 0.00024 | 7.97 | No | |
| 2 | 0.094 | 0.0000 | 0.105 | 0.0103 | 3.66 | 0.114 | 0.0000 | 0.00012 | 4.02 | No | |
| 3 | 0.946 | 0.0000 | 0.953 | 0.2532 | 89.99 | 0.968 | 0.0000 | 0.00268 | 88.01 | No | |

# Calibration results:

## Height calibration for substance 8-THC @ RT White:

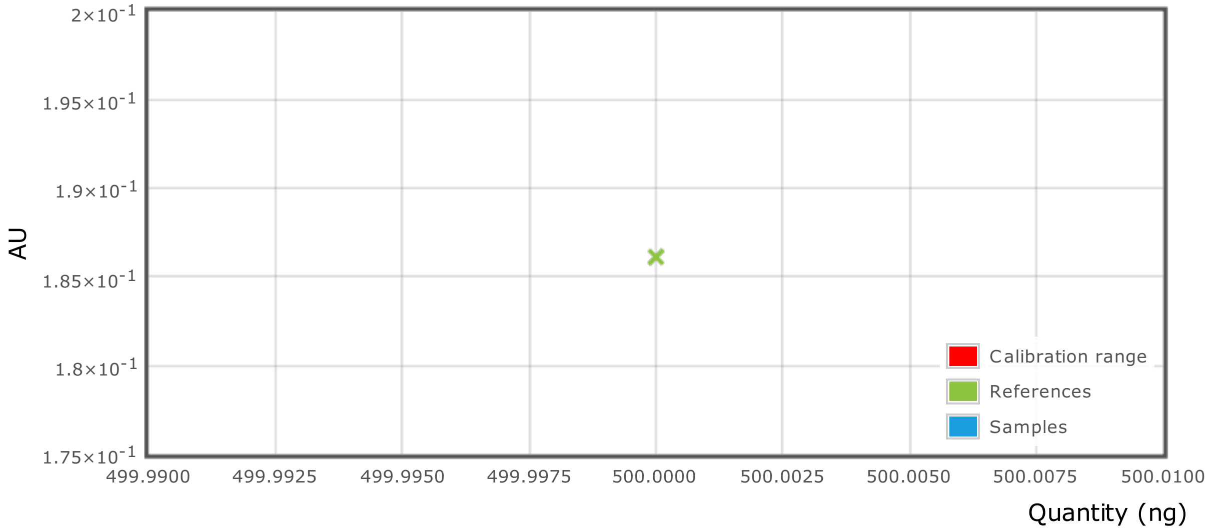


| | |
|---|---|
| Regression mode | Linear-2 |
| Range deviation | 5.00 % |
| Related substances | Default |
| Number of references | 1 |
| Calibration function | $y=0x$ |
| Coefficient of variation | CV 0.00 % |
| Correlation coefficient | n/a |
| ⚠ | Unable to compute the results for this substance because there wasn't enough groups of references replicas (at least 1 for Linear-1, 2 for Linear2 and Mime-1 and 3 for Polynomial and MiMe-2) |

## Height calibration for substance 9-THC @ RT White:

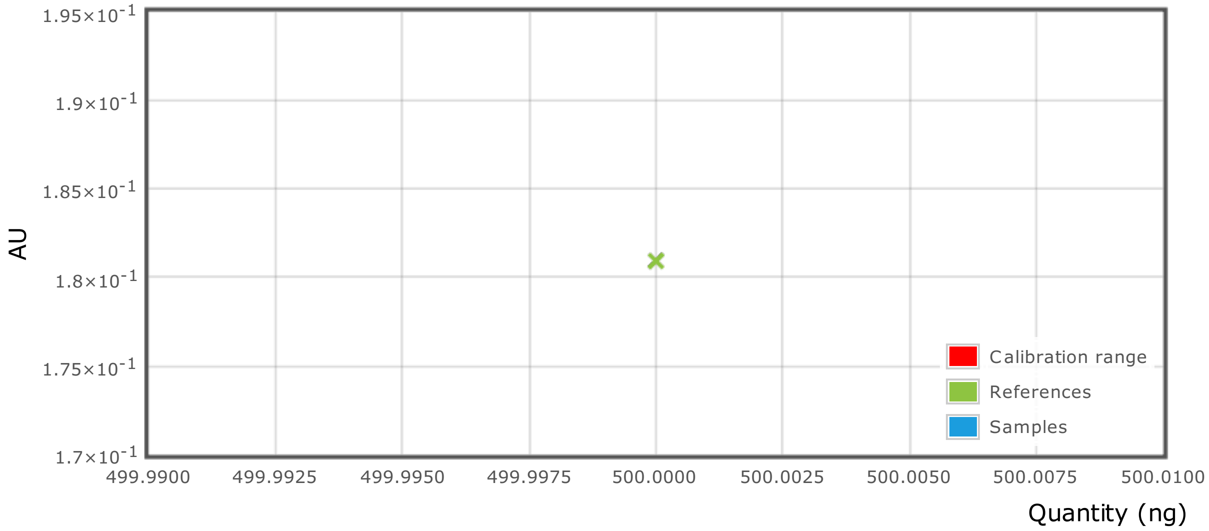

| | |
|---|---|
| Regression mode | Linear-2 |
| Range deviation | 5.00 % |
| Related substances | Default |
| Number of references | 1 |
| Calibration function | $y=0x$ |
| Coefficient of variation | CV 0.00 % |
| Correlation coefficient | n/a |
| ⚠ | Unable to compute the results for this substance because there wasn't enough groups of references replicas (at least 1 for Linear-1, 2 for Linear2 and Mime-1 and 3 for Polynomial and MiMe-2) |

## Height calibration for substance CBC @ RT White:

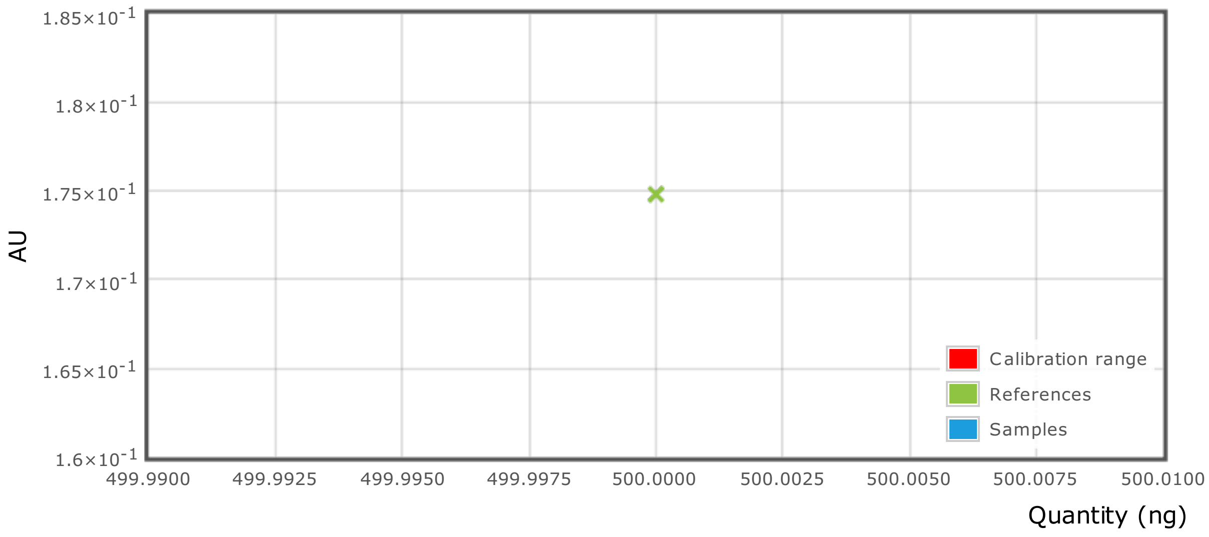


| | |
|---|---|
| Regression mode | Linear-2 |
| Range deviation | 5.00 % |
| Related substances | Default |
| Number of references | 1 |
| Calibration function | $y=0x$ |
| Coefficient of variation | CV 0.00 % |
| Correlation coefficient | n/a |
| ⚠ | Unable to compute the results for this substance because there wasn't enough groups of references replicas (at least 1 for Linear-1, 2 for Linear2 and Mime-1 and 3 for Polynomial and MiMe-2) |

## Height calibration for substance CBD @ RT White:

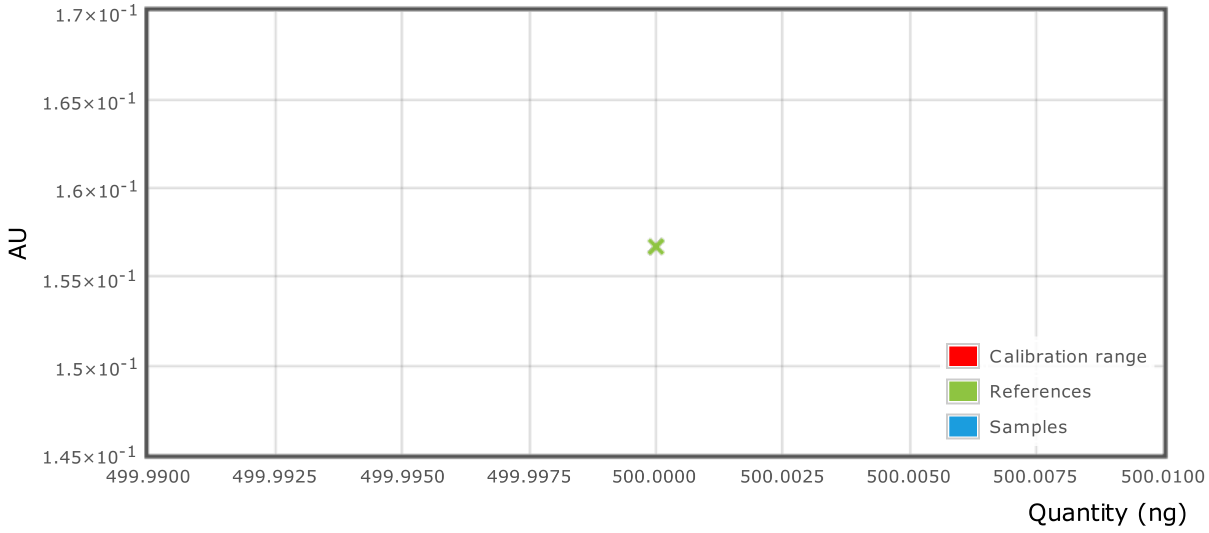


| | |
|---|---|
| Regression mode | Linear-2 |
| Range deviation | 5.00 % |
| Related substances | Default |
| Number of references | 1 |
| Calibration function | $y=0x$ |
| Coefficient of variation | CV 0.00 % |
| Correlation coefficient | n/a |
| ⚠ | Unable to compute the results for this substance because there wasn't eno ugh groups of references replicas (at least 1 for Linear-1, 2 for Linear2 and Mime-1 and 3 for Polynomial and MiMe-2) |

## Height calibration for substance CBDA @ RT White:

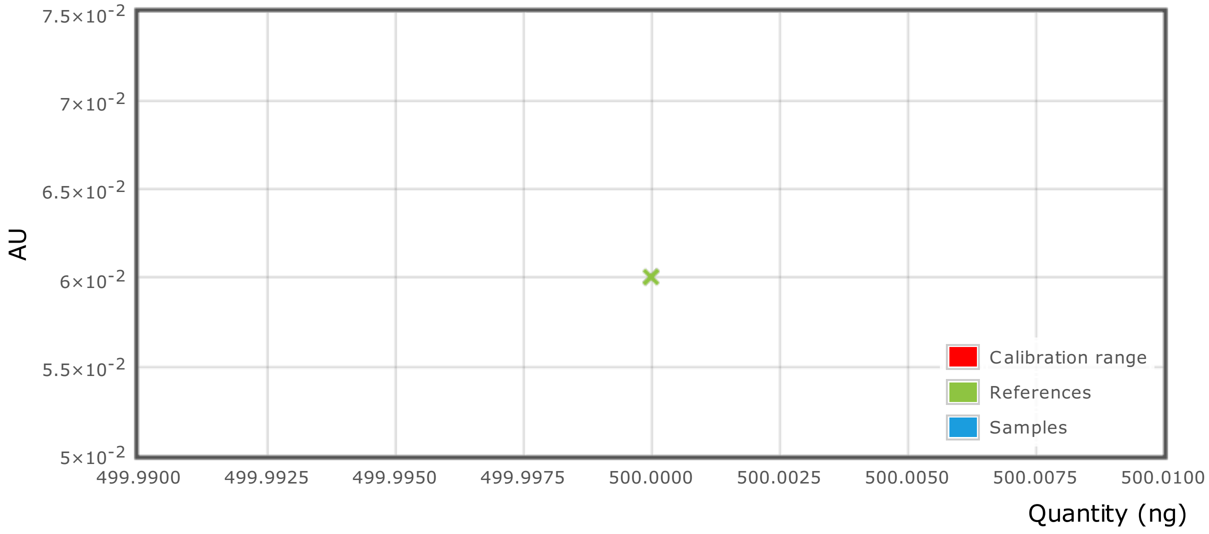

| | |
|---|---|
| Regression mode | Linear-2 |
| Range deviation | 5.00 % |
| Related substances | Default |
| Number of references | 1 |
| Calibration function | $y=0x$ |
| Coefficient of variation | CV 0.00 % |
| Correlation coefficient | n/a |
| ⚠ | Unable to compute the results for this substance because there wasn't enough groups of references replicas (at least 1 for Linear-1, 2 for Linear2 and Mime-1 and 3 for Polynomial and MiMe-2) |

## Height calibration for substance CBDV @ RT White:

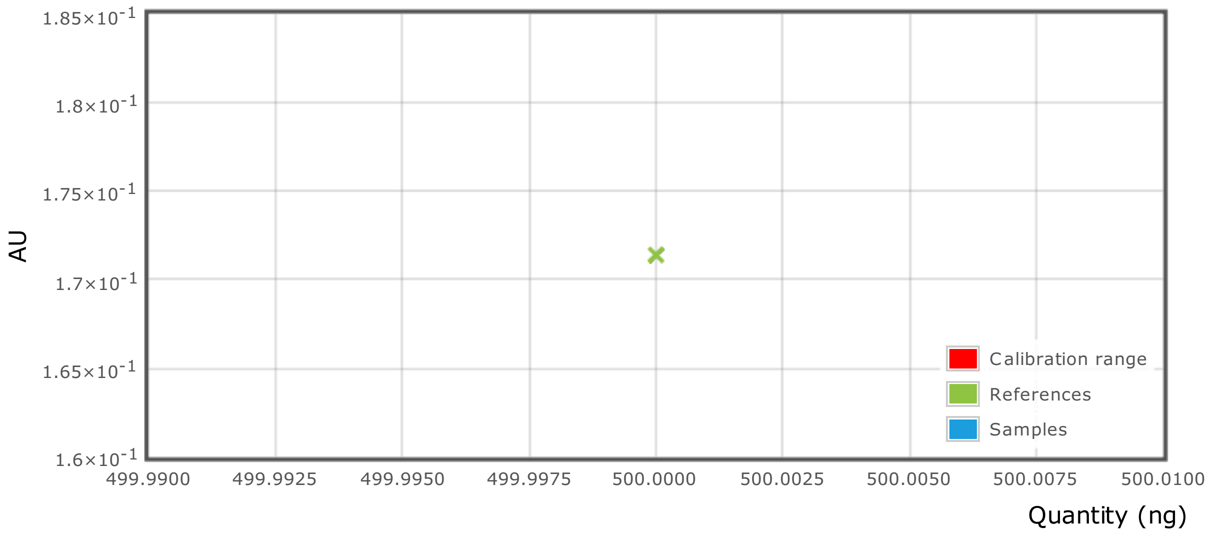


| | |
|---|---|
| Regression mode | Linear-2 |
| Range deviation | 5.00 % |
| Related substances | Default |
| Number of references | 1 |
| Calibration function | $y=0x$ |
| Coefficient of variation | CV 0.00 % |
| Correlation coefficient | n/a |
| ⚠ | Unable to compute the results for this substance because there wasn't enough groups of references replicas (at least 1 for Linear-1, 2 for Linear2 and Mime-1 and 3 for Polynomial and MiMe-2) |

## Height calibration for substance CBG @ RT White:

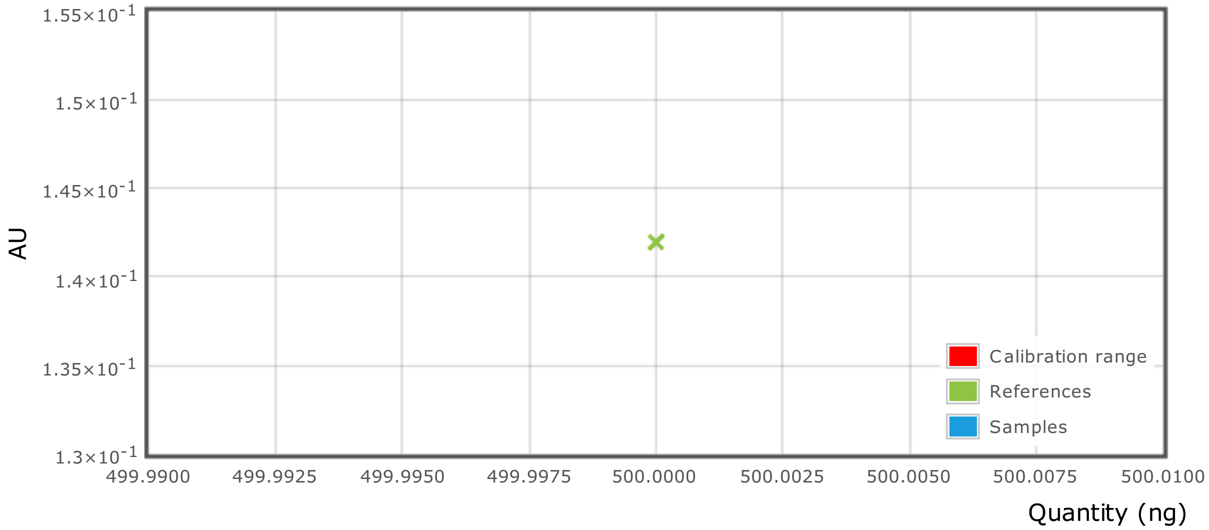


| | |
|---|---|
| Regression mode | Linear-2 |
| Range deviation | 5.00 % |
| Related substances | Default |
| Number of references | 1 |
| Calibration function | $y=0x$ |
| Coefficient of variation | CV 0.00 % |
| Correlation coefficient | n/a |
| ⚠ | Unable to compute the results for this substance because there wasn't enough groups of references replicas (at least 1 for Linear-1, 2 for Linear2 and Mime-1 and 3 for Polynomial and MiMe-2) |

## Height calibration for substance CBGA @ RT White:

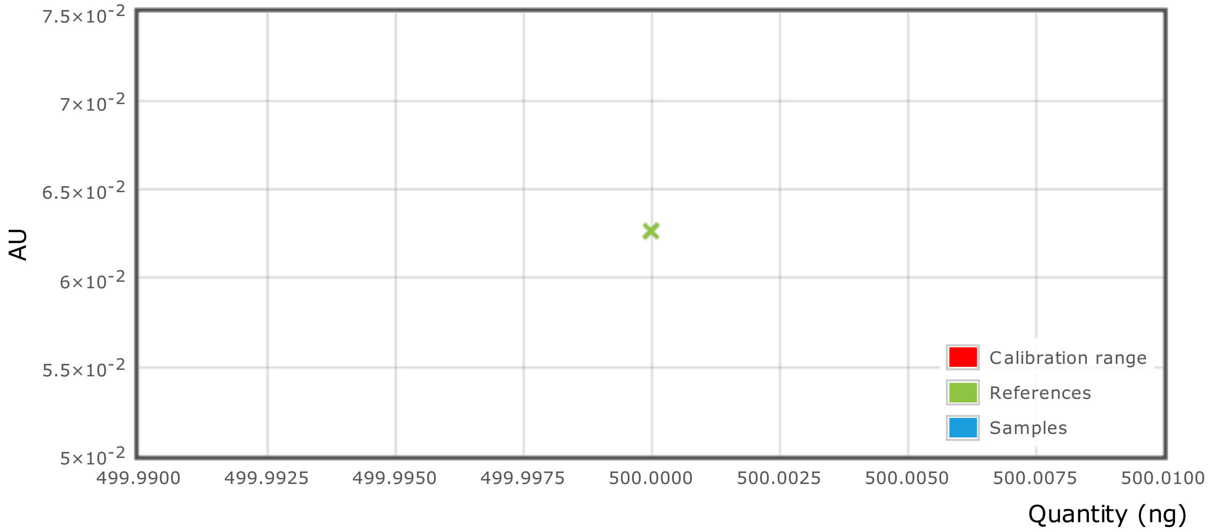

| | |
|---|---|
| Regression mode | Linear-2 |
| Range deviation | 5.00 % |
| Related substances | Default |
| Number of references | 1 |
| Calibration function | $y=0x$ |
| Coefficient of variation | CV 0.00 % |
| Correlation coefficient | n/a |
| ⚠ | Unable to compute the results for this substance because there wasn't enough groups of references replicas (at least 1 for Linear-1, 2 for Linear2 and Mime-1 and 3 for Polynomial and MiMe-2) |

## Height calibration for substance CBN @ RT White:

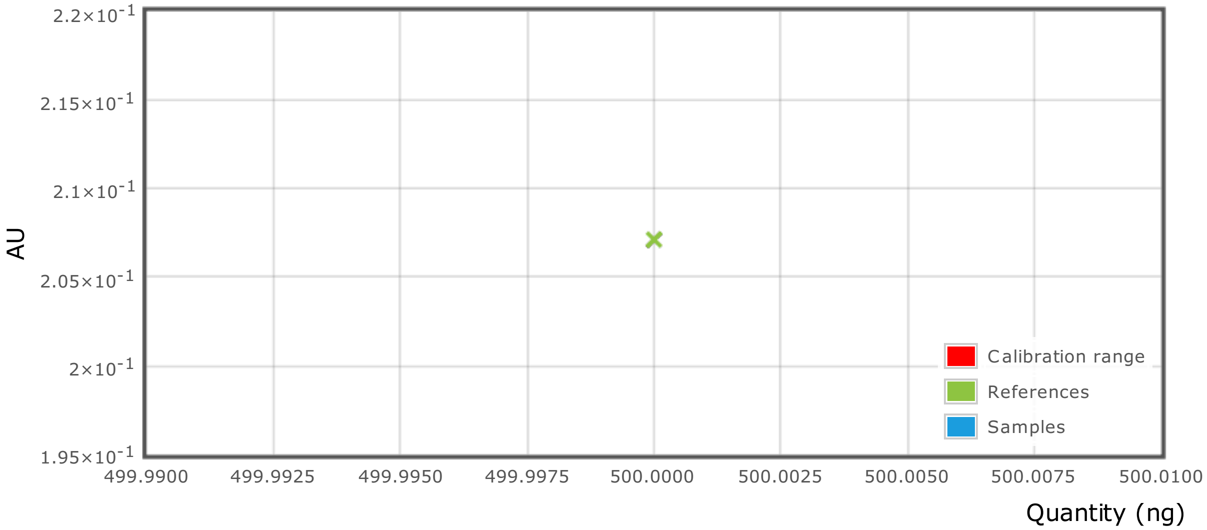


| | |
|---|---|
| Regression mode | Linear-2 |
| Range deviation | 5.00 % |
| Related substances | Default |
| Number of references | 1 |
| Calibration function | $y=0x$ |
| Coefficient of variation | CV 0.00 % |
| Correlation coefficient | n/a |
| ⚠ | Unable to compute the results for this substance because there wasn't enough groups of references replicas (at least 1 for Linear-1, 2 for Linear2 and Mime-1 and 3 for Polynomial and MiMe-2) |

## Height calibration for substance THCA-A @ RT White:

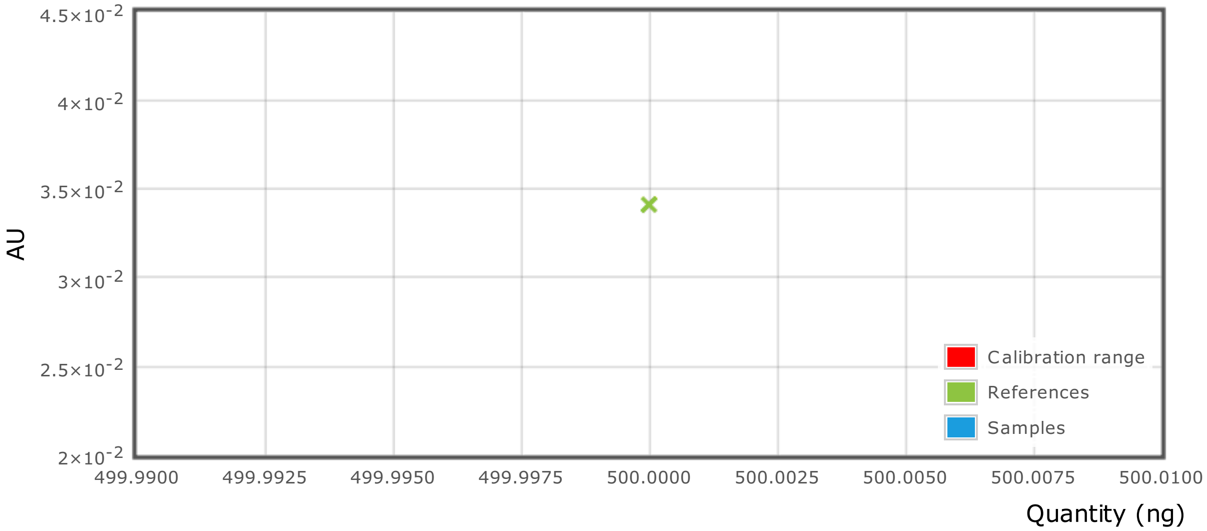


| | |
|---|---|
| Regression mode | Linear-2 |
| Range deviation | 5.00 % |
| Related substances | Default |
| Number of references | 1 |
| Calibration function | $y=0x$ |
| Coefficient of variation | CV 0.00 % |
| Correlation coefficient | n/a |
| ⚠ | Unable to compute the results for this substance because there wasn't enough groups of references replicas (at least 1 for Linear-1, 2 for Linear2 and Mime-1 and 3 for Polynomial and MiMe-2) |

## Height calibration for substance THCV @ RT White:

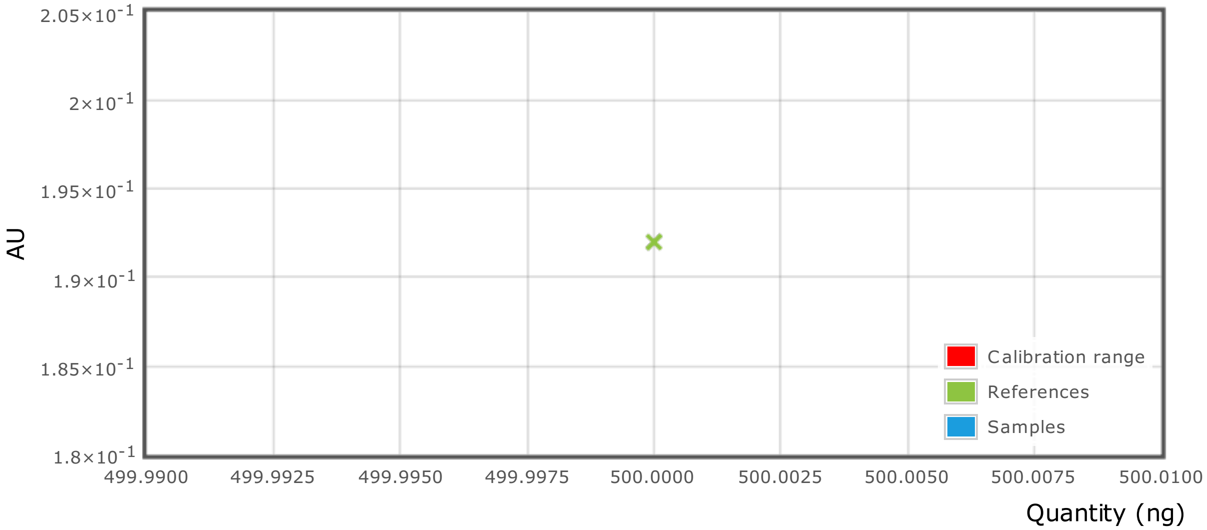

| | |
|---|---|
| Regression mode | Linear-2 |
| Range deviation | 5.00 % |
| Related substances | Default |
| Number of references | 1 |
| Calibration function | $y=0x$ |
| Coefficient of variation | CV 0.00 % |
| Correlation coefficient | n/a |
| ⚠ | Unable to compute the results for this substance because there wasn't eno ugh groups of references replicas (at least 1 for Linear-1, 2 for Linear2 and Mime-1 and 3 for Polynomial and MiMe-2) |

## Results:

| Substance having no available results | | |
|---|---|---|
| ⚠ | CBDV | There wasn't any sample application available in the assignments for this substance. Please check that the peaks were correctly detected and assig ned for this substance. |
| ⚠ | CBDA | There wasn't any sample application available in the assignments for this substance. Please check that the peaks were correctly detected and assig ned for this substance. |
| ⚠ | 9-THC | There wasn't any sample application available in the assignments for this substance. Please check that the peaks were correctly detected and assig ned for this substance. |
| ⚠ | CBC | There wasn't any sample application available in the assignments for this substance. Please check that the peaks were correctly detected and assig ned for this substance. |
| ⚠ | CBGA | There wasn't any sample application available in the assignments for this substance. Please check that the peaks were correctly detected and assig ned for this substance. |
| ⚠ | CBD | There wasn't any sample application available in the assignments for this substance. Please check that the peaks were correctly detected and assig ned for this substance. |
| ⚠ | THCV | There wasn't any sample application available in the assignments for this substance. Please check that the peaks were correctly detected and assig ned for this substance. |
| ⚠ | THCA-A | There wasn't any sample application available in the assignments for this substance. Please check that the peaks were correctly detected and assig ned for this substance. |
| ⚠ | CBG | There wasn't any sample application available in the assignments for this substance. Please check that the peaks were correctly detected and assig ned for this substance. |
| ⚠ | 8-THC | There wasn't any sample application available in the assignments for this substance. Please check that the peaks were correctly detected and assig ned for this substance. |
| ⚠ | CBN | There wasn't any sample application available in the assignments for this substance. Please check that the peaks were correctly detected and assig ned for this substance. |

A track marked with ⚠ means: this result is outside the regression range given by the reference assignments, but is included in the results because it is in the allowed range deviation.

| | |
|---|---|
| Analyst: | Reviewer: |
