# Supplementary material for: High Performance Thin-Layer Chromatography (HPTLC) data of Cannabinoids in ten mobile phase systems
Source: Data Brief. 2020 Jun 30;31:105955. doi: 10.1016/j.dib.2020.105955 (PMC7352075; doi:10.1016/j.dib.2020.105955)
Supplement: Supplementary file 1 [file mmc1.zip › S4-Case sample reports/6DaT-sample run-4.pdf]

Analysis: 6DaT-sample run-4

Path: Home/YL Research

Based on method: Samples (no cal)

Created	11-Oct-2019 13:26:25	visionCATSuser
Modified	11-Oct-2019 15:37:34	visionCATSuser
Last HPTLC log	11-Oct-2019 15:37:34	Analysis modified
Explorer notes		

Track	Vial ID	Description	Volume	Position	Type
1	MeOH blank	MeOH Blank	2.0 µl	A3	Sample
2	250ug/mL mix	250ug/mL	2.0 µl	A4	Reference
3	Tetracosane	Tetracosane IS	2.0 µl	A5	Sample
4	s1	[redacted]	2.0 µl	A1	Sample
5	s2	[redacted]	2.0 µl	B1	Sample
6	s3	[redacted]	2.0 µl	C1	Sample
7	s4	[redacted]	2.0 µl	D1	Sample
8	s5	[redacted]	2.0 µl	E1	Sample
9	s6	[redacted]	2.0 µl	F1	Sample
10	s7	[redacted]	2.0 µl	A2	Sample
11	s8	[redacted]	2.0 µl	B2	Sample
12	s9	[redacted]	2.0 µl	C2	Sample
13	s10	[redacted]	2.0 µl	D2	Sample
14	250ug/mL mix	250ug/mL	2.0 µl	A4	Reference
15	MeOH blank	MeOH Blank	2.0 µl	A3	Sample
Sequence table notes					

A track marked with ⚠ means: the application type is overridden in some evaluation(s).

System setup:

Software	Server User-PC, version 2.5.18072.1
ATS4	S/N:080713
Chamber	N/A
Derivatization dip	N/A
Scanner3	S/N:031025
Visualizer	S/N:230515

Chromatography

Plate layout:

Stationary phase	Merck, HPTLC plates silica gel 60 F 254
Plate format	200.0 x 100.0 mm
Application type	Band
Application	Position Y: 8.0 mm, length: 8.0 mm, width: 0.0 mm
Track	First position X: 20.0 mm, distance: 11.4 mm
Solvent front position	70.0 mm
Notes	

Take image clean plate 1a - Visualizer (S/N: 230515):

Quality	Enhanced
RT White	auto capture, Auto, level 85 %, Band
R 254	auto capture, Auto, level 85 %, Band
Instrument diagnostics	Valid diagnostics
Documentation step label	
Notes	

Application 1 - ATS 4 (S/N: 080713):

Spray gas	NI
Sample solvent type	Methanol
Filling speed	15 µl/s
Predosage volume	200 nl
Retraction volume	200 nl
Dosage speed	150 nl/s
Filling quality	User
Rinsing cycles / vacuum	2 / 4 s
Filling cycles / vacuum	1 / 4 s
Rinsing solvent name	Methanol
Nozzle temperature	Unheated
Rack in use	Standard
Instrument diagnostics	Valid diagnostics
Notes	

Development 1 - Chamber:

Tank	TTC 20x10
Mobile phase	6% diethylamine in toluene
Saturation time	20 min
Use saturation pad	true
Use smartALERT	false
Volume front through	10 ml
Volume rear through	25 ml
Drying time	5 min
Drying temperature	Room temperature
Notes	

Take image developed plate 1a - Visualizer (S/N: 230515):

Quality	Enhanced
RT White	auto capture, Auto, level 85 %, Band
R 254	auto capture, Auto, level 85 %, Band
R 366	auto capture, Auto, level 85 %, Band
Instrument diagnostics	Valid diagnostics
Documentation step label	
Notes	

Scan developed plate 1b - Scanner 3 (S/N: 031025):

Scanner type	Single λ
Optimization for	Resolution
Measurement mode	Absorption
Filter	n/a
Detector mode	Automatic
Scanning speed	20 mm/s
Data resolution	100 µm/step
Slit	5 x 0.2 mm, micro
Partial scan	No
Lamp	Deuterium & Tungsten
Wavelength(s)	254 nm
Instrument diagnostics	Valid diagnostics
Documentation step label	
Notes	

Derivatization 1 - dip:

Reagent name	
Dipping speed	5
Dipping time	0 s
Reagent preparation	
Heating	100 °C for 3 min, heated after
Notes	

Take image derivatized plate 1a - Visualizer (S/N: 230515):

Quality	Enhanced
RT White	auto capture, Auto, level 85 %, Band
R 366	auto capture, Auto, level 85 %, Band
Instrument diagnostics	Valid diagnostics
Documentation step label	
Notes	

System suitability tests:

SST settings:

SST tracks	

Data acquisition

Application 1 - ATS 4 (S/N: 080713):

Executed	11-Oct-2019 13:41:17 visionCATSuser

Development 1 - Chamber:

Executed	11-Oct-2019 14:20:00 visionCATSuser

Take image developed plate 1a - Visualizer (S/N: 230515):

Executed	11-Oct-2019 15:13:32 visionCATSuser

RT White Developed, RemTransVis

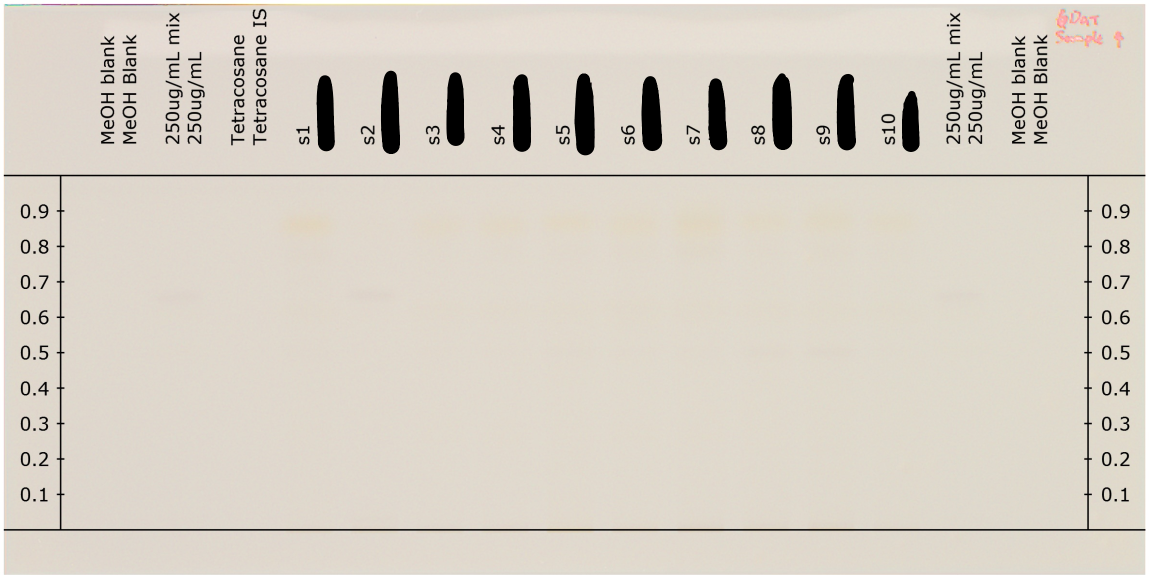

Exposure	0.082 s
Contrast	1
Normalized exposure	Disabled
Clarify	Disabled
White balance	1.00, 1.00, 1.00

R 254 Developed, Remission254

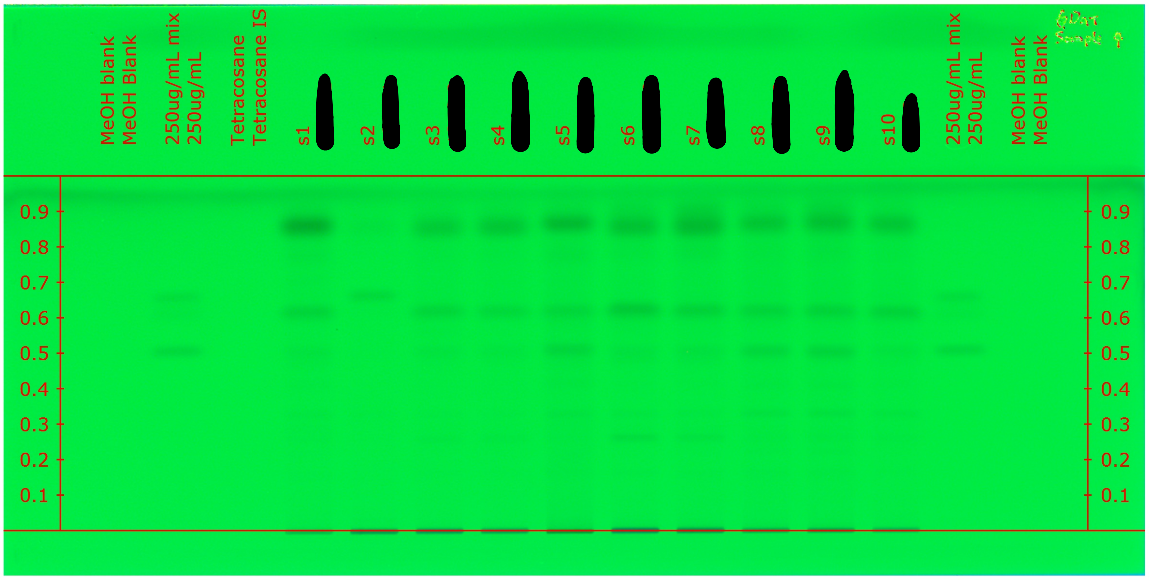

Exposure	0.266 s
Contrast	1
Normalized exposure	Disabled
Clarify	Disabled
White balance	1.00, 1.00, 1.00

R 366

Developed, Remission366

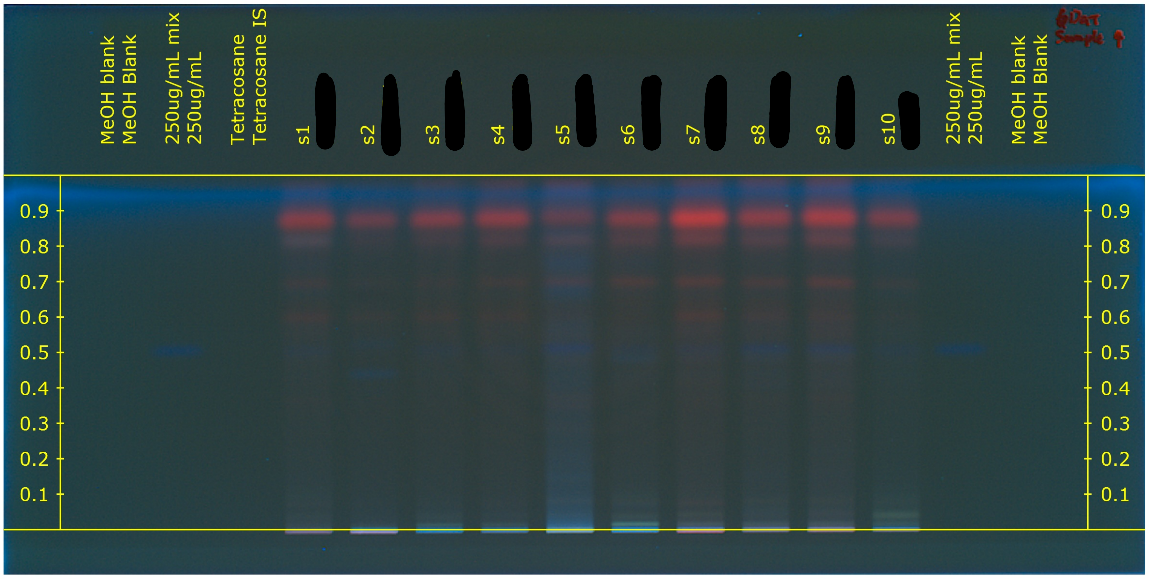

Exposure	4.498 s
Contrast	1
Normalized exposure	Disabled
Clarify	Disabled
White balance	1.00, 1.00, 1.00

Scan developed plate 1b - Scanner 3 (S/N: 031025):

Executed	11-Oct-2019 15:15:06 visionCATSuser

Scan:

Wavelength	254 nm

Track 1:

Type	Single λ

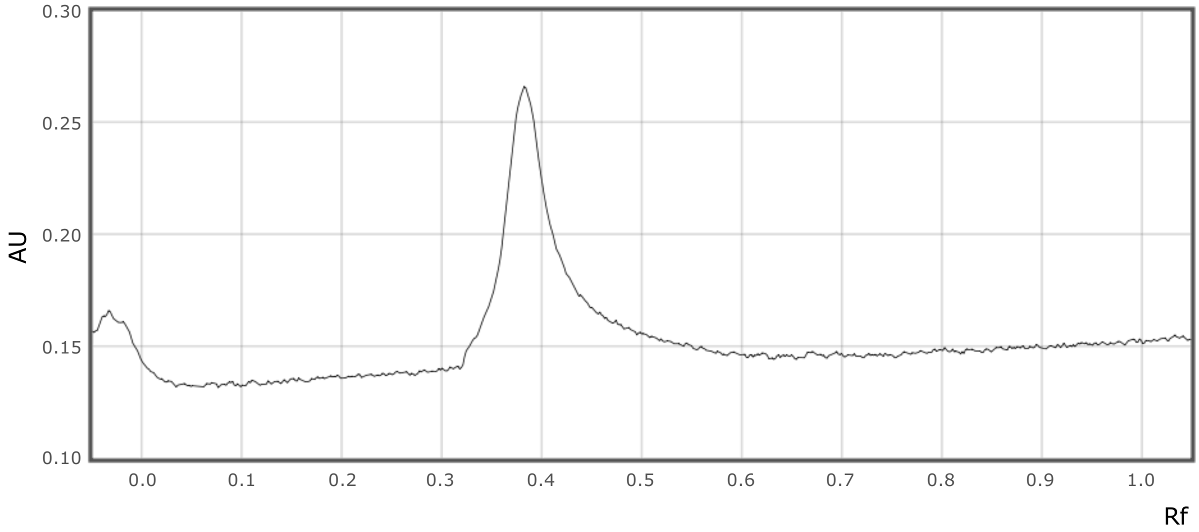

Track 2:

Type	Single λ

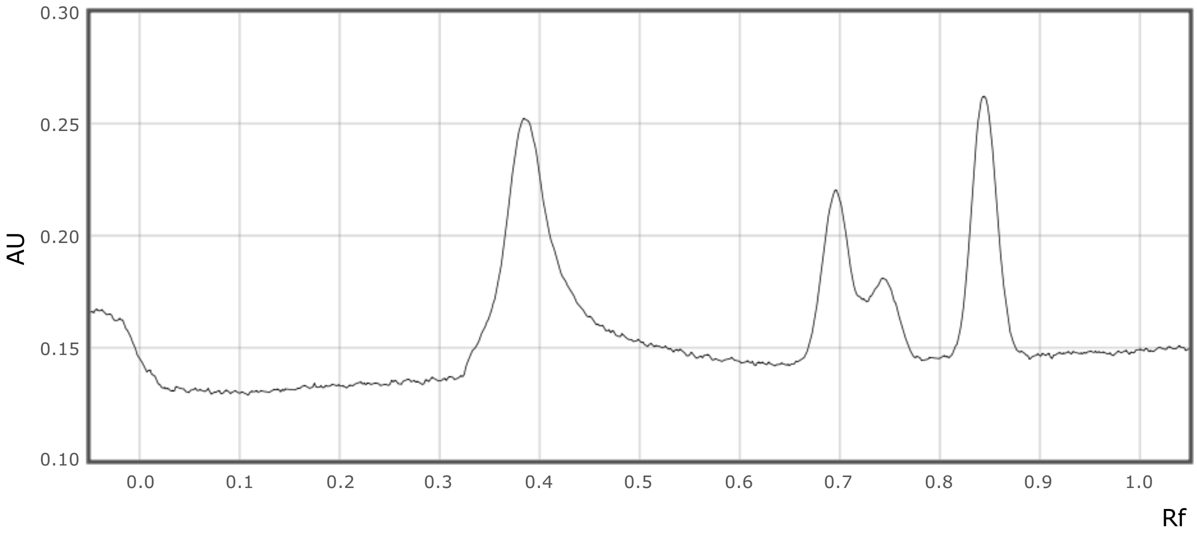

Track 3:

Type	Single λ

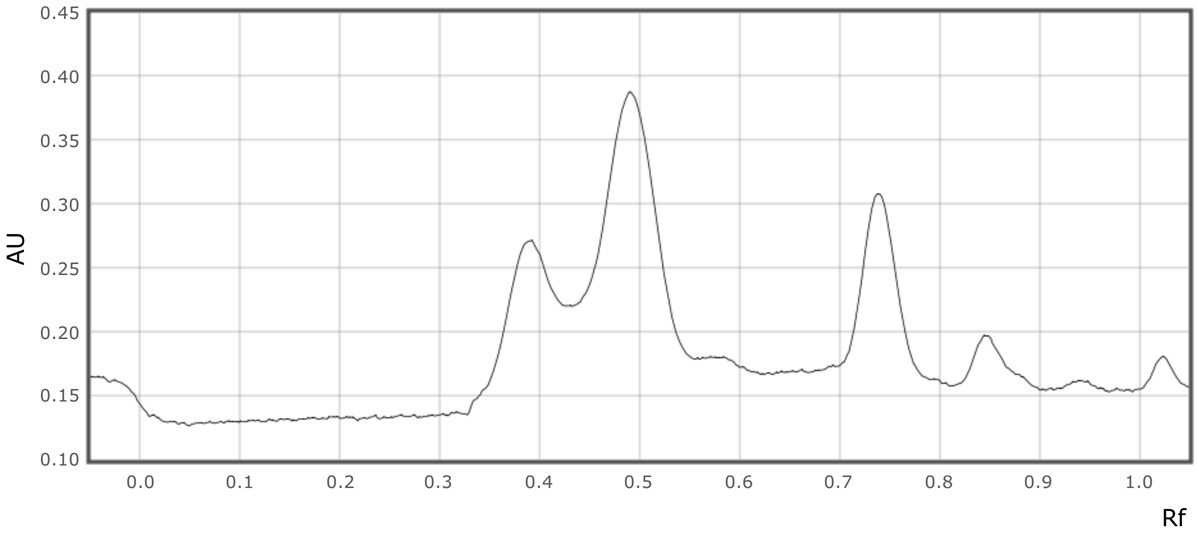

Track 4:

Type	Single λ

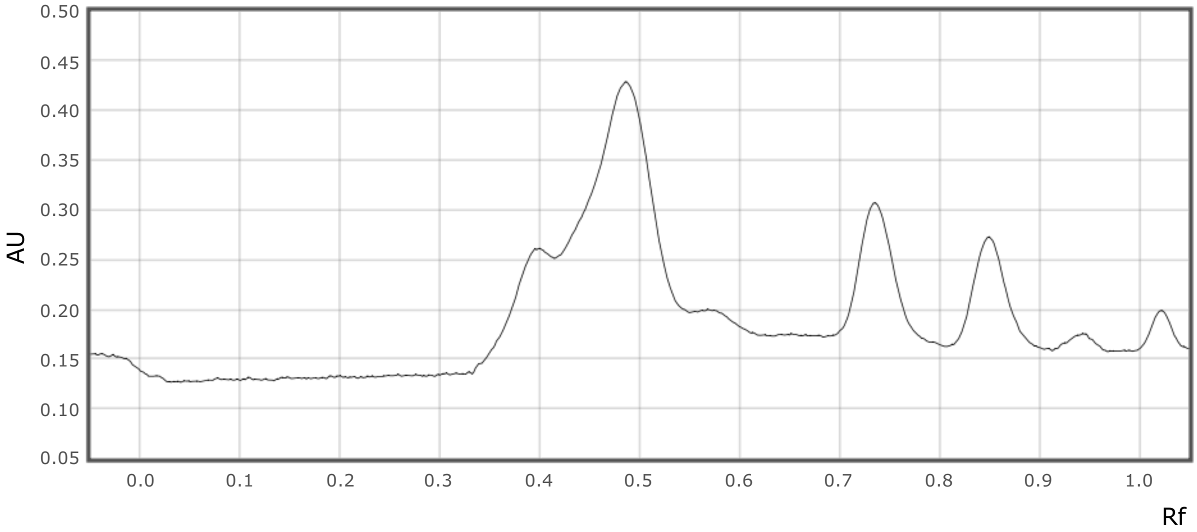

Track 5:	
Type	Single λ

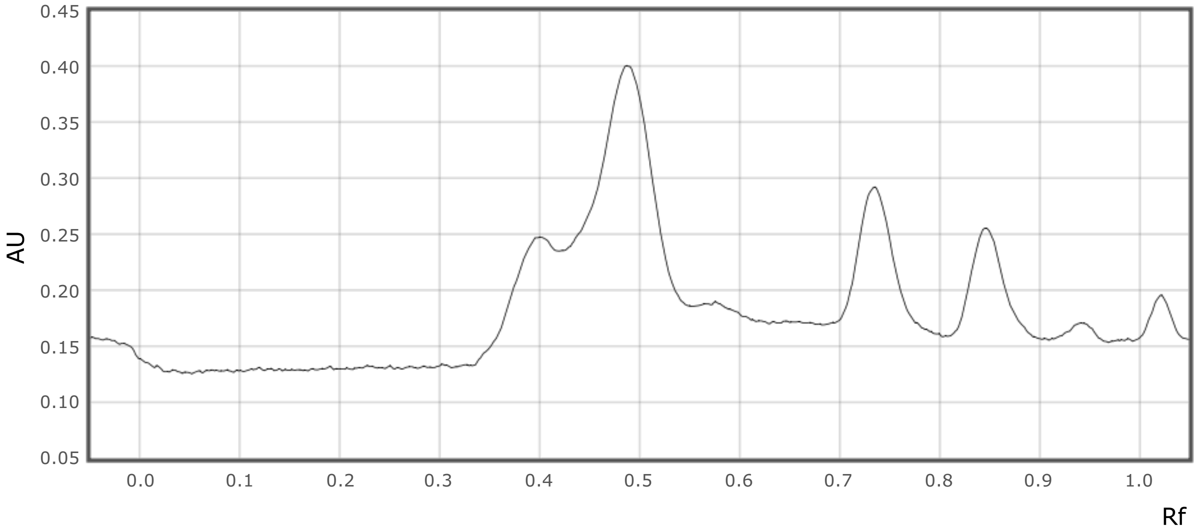

Track 6:	
Type	Single λ

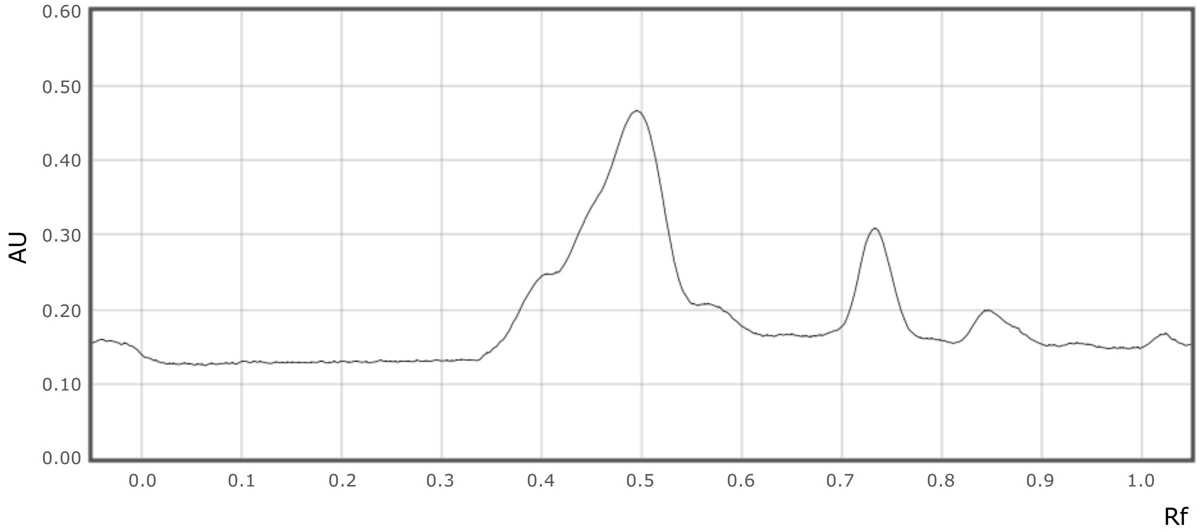

Track 7:

Type	Single λ

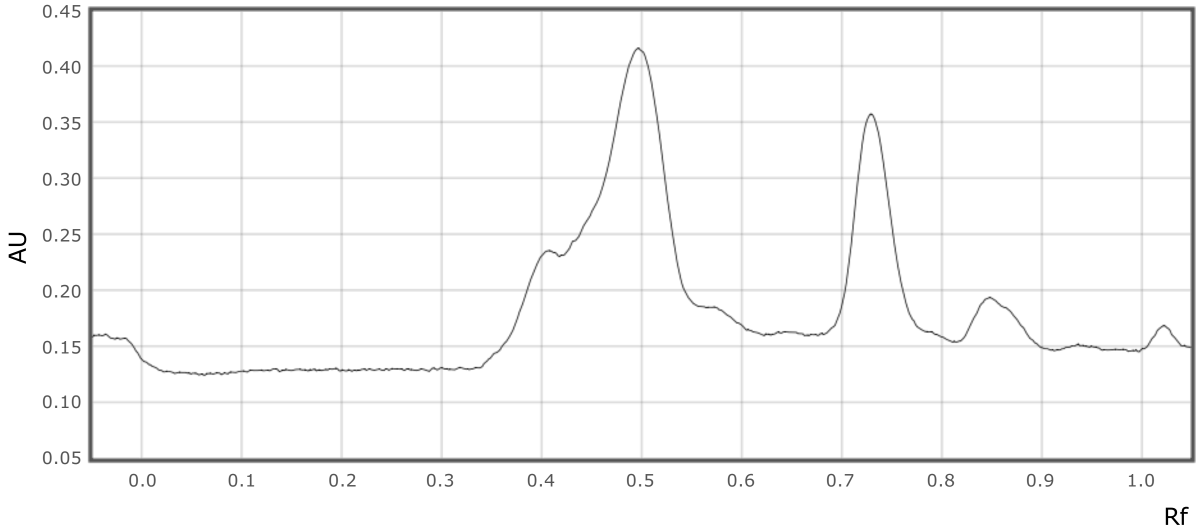

Track 8:

Type	Single λ

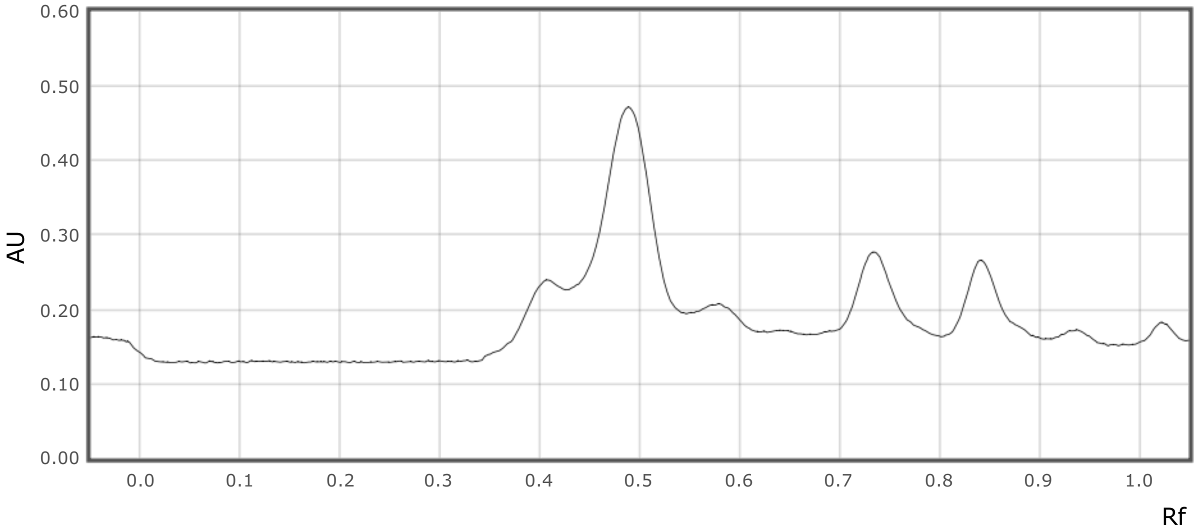

Track 9:

Type	Single λ

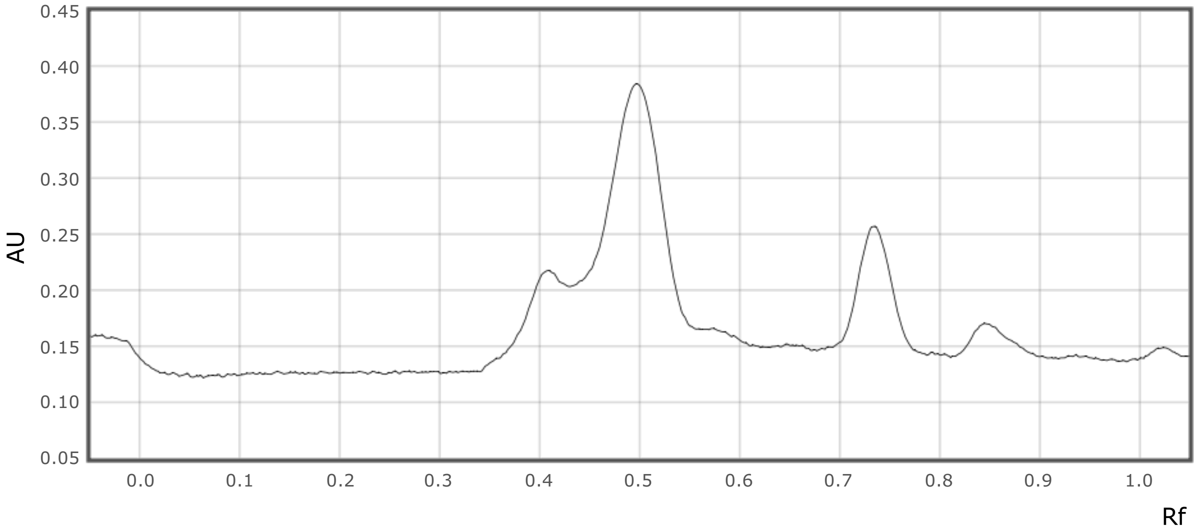

Track 10:

Type	Single λ

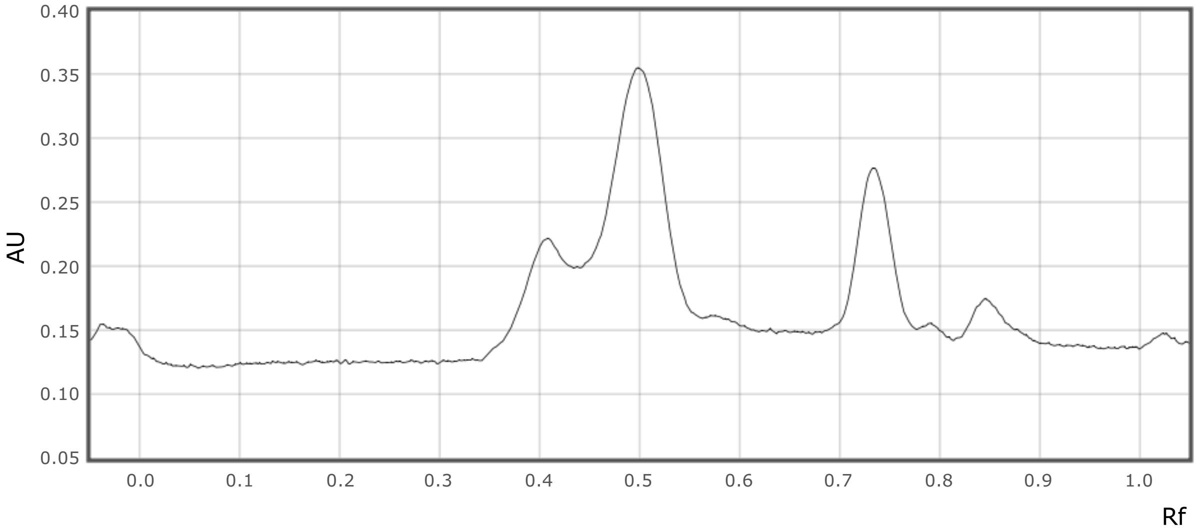

Track 11:	
Type	Single λ

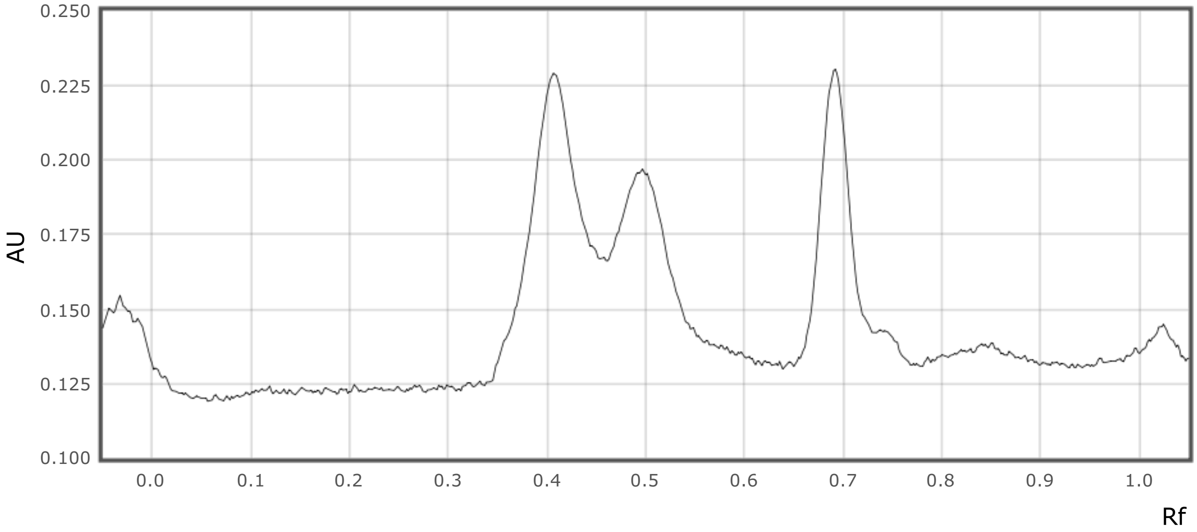

Track 12:	
Type	Single λ

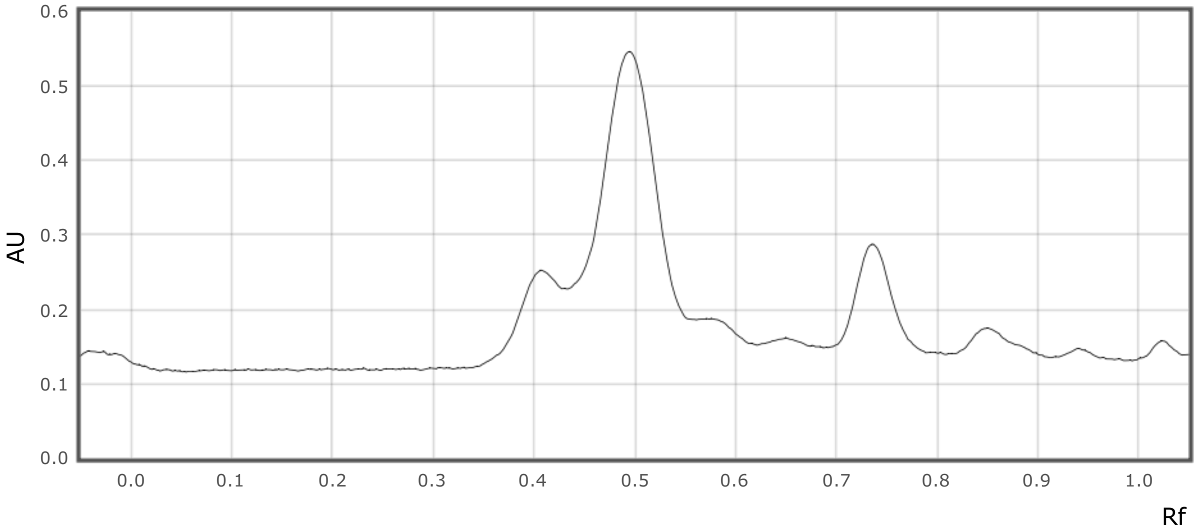

Track 13:	
Type	Single λ

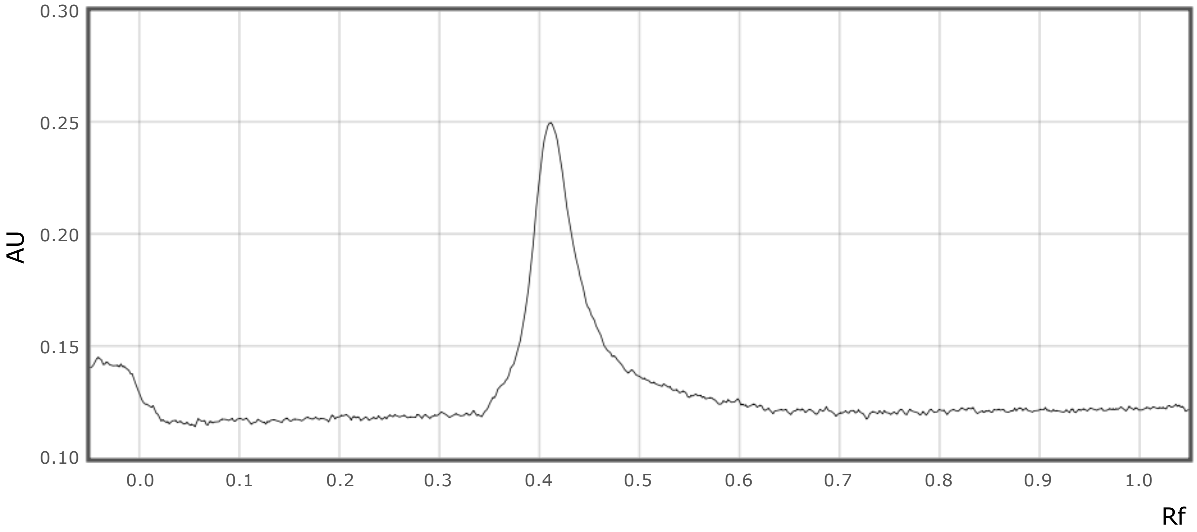

Track 14:	
Type	Single λ

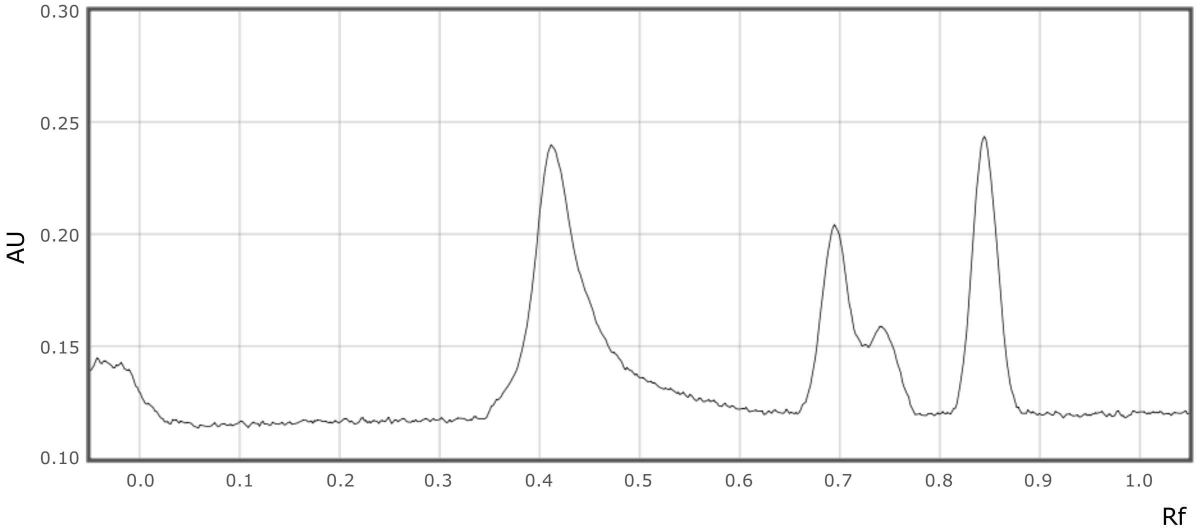

Track 15:	
Type	Single λ

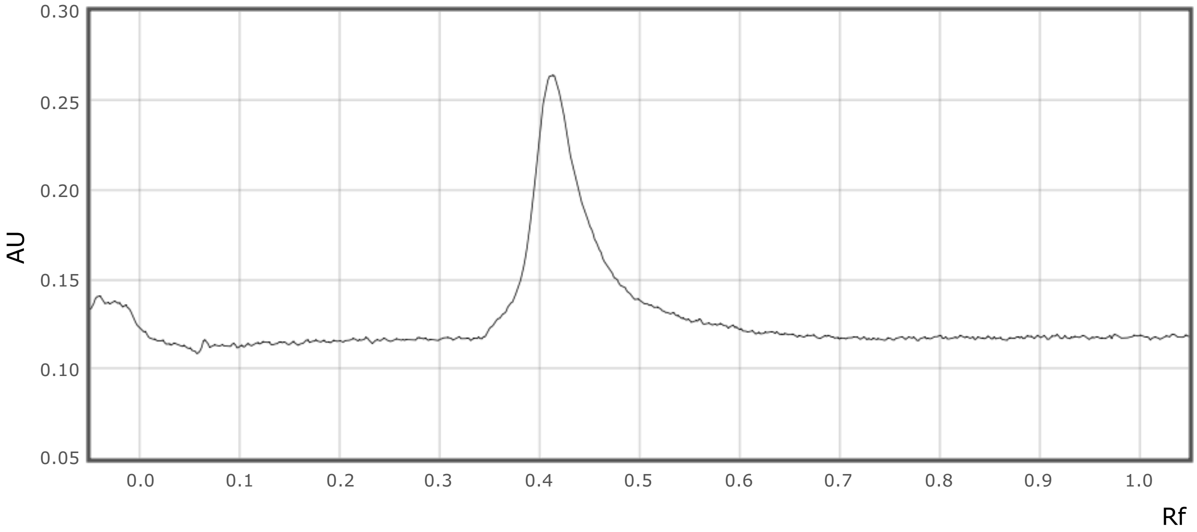

Derivatization 1 - dip:

Executed	11-Oct-2019 15:30:43 visionCATSuser

Take image derivatized plate 1a - Visualizer (S/N: 230515):

Executed	11-Oct-2019 15:33:38 visionCATSuser

RT White Derivatized, RemTransVis

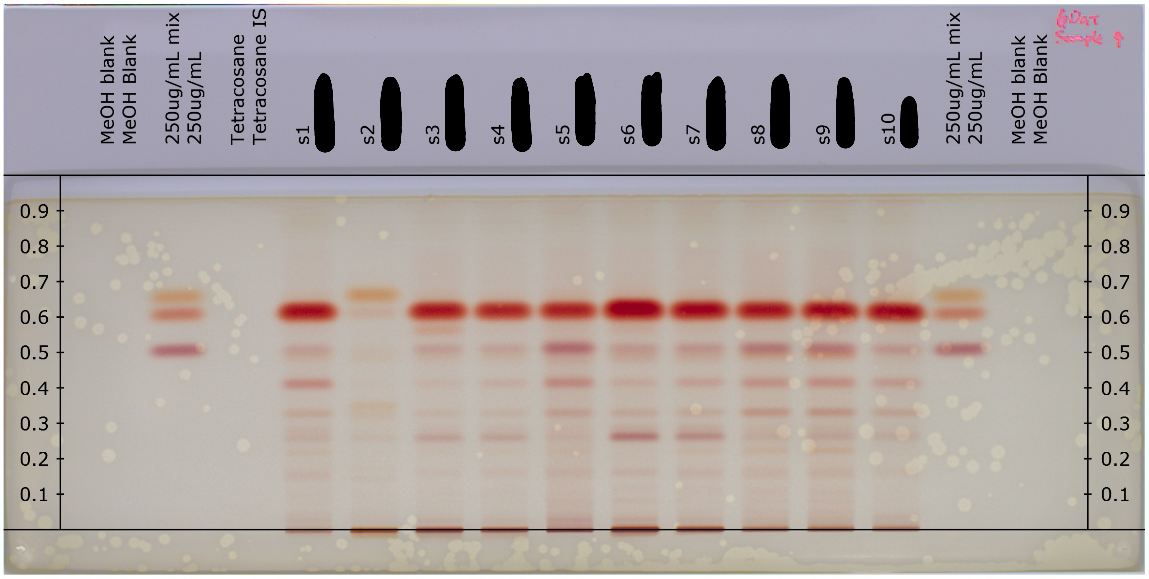

Exposure	0.046 s
Contrast	1
Normalized exposure	Disabled
Clarify	Disabled
White balance	1.12, 1.09, 0.84

R 366 Derivatized, Remission366

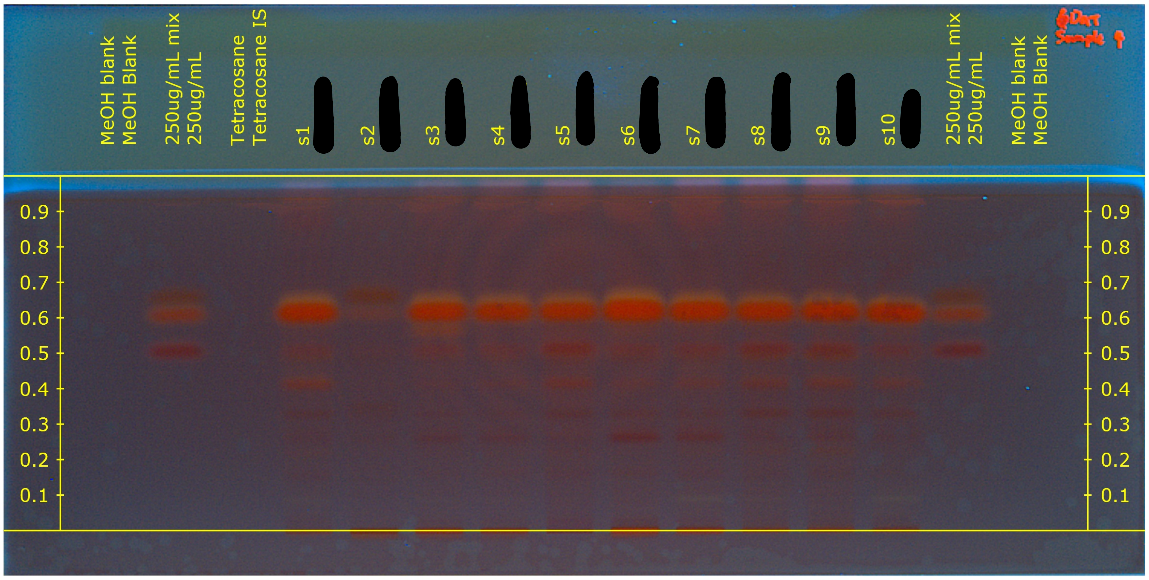

Exposure	9.999 s
Contrast	1
Normalized exposure	Disabled
Clarify	Disabled
White balance	1.00, 1.00, 1.00

Evaluation 1 :

Validated	false
Step	Take image derivatized plate 1a
Concentration unit type	Mass / volume
Notes	

Definition:

References:

250ug/mL mix

Substance Name	Concentration	Purity
9-THC	250.000 µg/ml	100.00 %
CBD	250.000 µg/ml	100.00 %
CBN	250.000 µg/ml	100.00 %

Samples:

Vial ID	Amount	Volume solution	Reference amount	Related to
MeOH blank		0.00 ml		
Tetracosane		0.00 ml		
s1		0.00 ml		
s2		0.00 ml		
s3		0.00 ml		
s4		0.00 ml		
s5		0.00 ml		
s6		0.00 ml		
s7		0.00 ml		
s8		0.00 ml		
s9		0.00 ml		
s10		0.00 ml		

Integration parameters:

Bounds	[0.000,1.000]
Smoothing	Savitzky–Golay of order 3 and window 7
Baseline correction	Lowest slope with noise 0.05
Profile subtraction	Profile subtraction from track 1
Peaks detection	Gauss (legacy) with sensitivity 0.1, separation 1 and threshold 0.1

Scan:

Wavelength	RT White

Track 1:

Type	Sample
Vial ID	MeOH blank
Description	MeOH Blank
Volume	2.0 µl

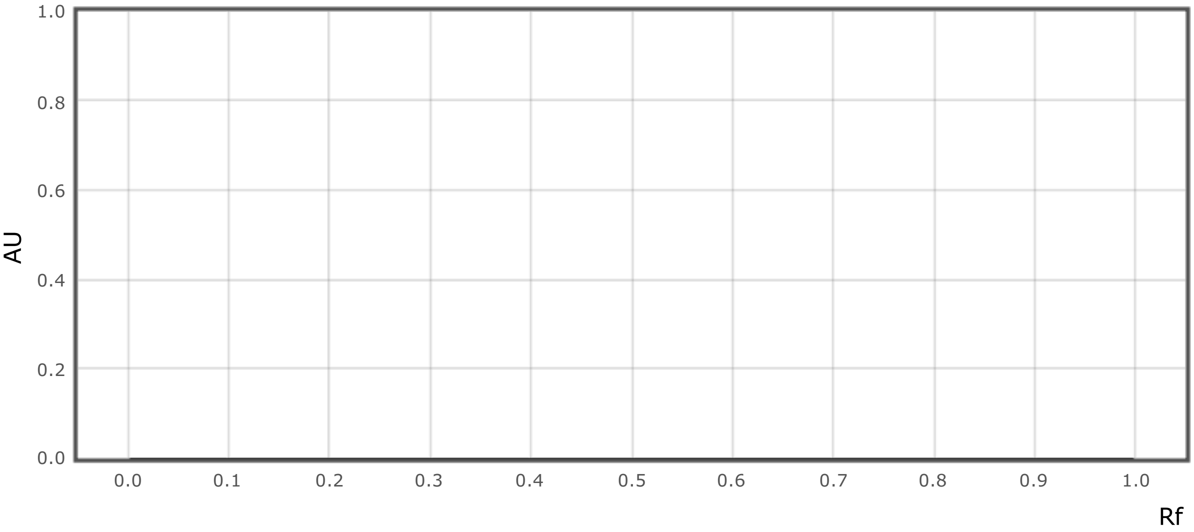

Peak #	Start		Max			End		Area		Manual peak	Substance Name
	Rf	H	Rf	H	%	Rf	H	A	%		

Track 2:

Type	Reference
Vial ID	250ug/mL mix
Description	250ug/mL
Volume	2.0 µl

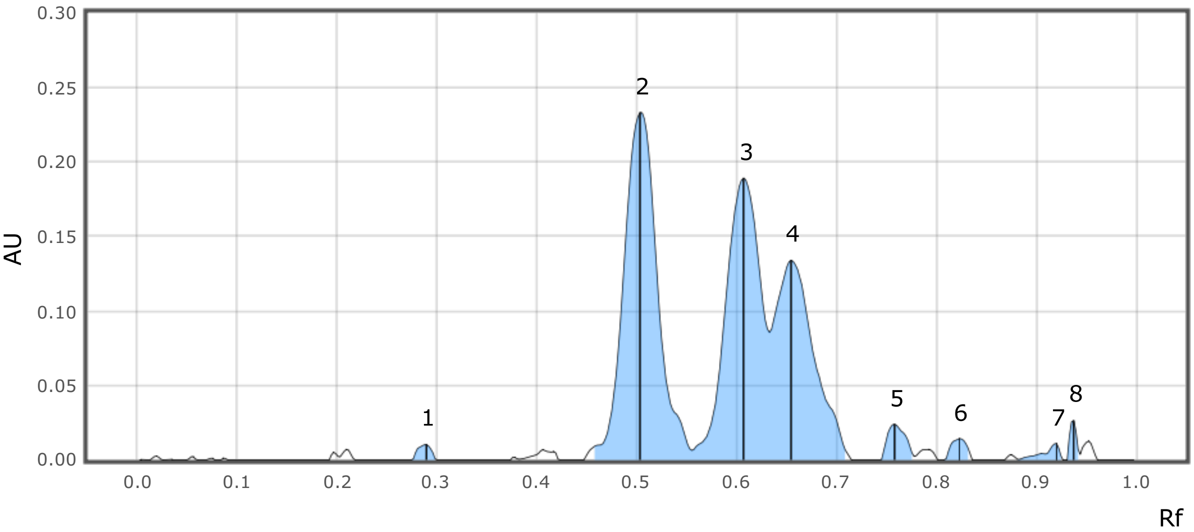

Peak	Start		Max			End		Area		Manual	Substance
#	Rf	H	Rf	H	%	Rf	H	A	%	peak	Name
1	0.274	0.0000	0.289	0.0103	1.60	0.300	0.0000	0.00016	0.66	No	
2	0.456	0.0081	0.503	0.2329	36.31	0.555	0.0063	0.00909	37.77	No	CBN
3	0.555	0.0063	0.607	0.1890	29.47	0.633	0.0855	0.00758	31.52	No	9-THC
4	0.633	0.0855	0.655	0.1338	20.87	0.715	0.0000	0.00607	25.24	No	CBD
5	0.743	0.0000	0.758	0.0238	3.71	0.778	0.0024	0.00050	2.09	No	
6	0.808	0.0000	0.823	0.0142	2.22	0.836	0.0000	0.00026	1.07	No	
7	0.881	0.0003	0.920	0.0110	1.71	0.927	0.0000	0.00019	0.79	No	
8	0.931	0.0000	0.938	0.0264	4.11	0.944	0.0037	0.00021	0.86	No	

Track 3:

Type	Sample
Vial ID	Tetracosane
Description	Tetracosane IS
Volume	2.0 µl

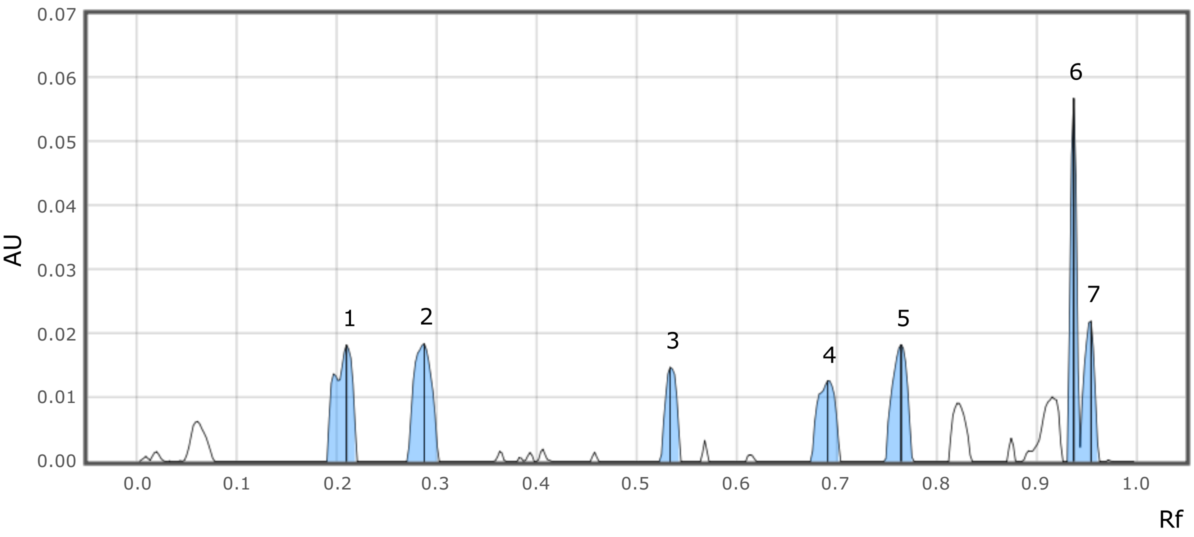

Peak	Start		Max			End		Area		Manual	Substance
#	Rf	H	Rf	H	%	Rf	H	A	%	peak	Name
1	0.188	0.0000	0.209	0.0182	11.31	0.220	0.0000	0.00037	17.17	No	
2	0.270	0.0000	0.287	0.0185	11.46	0.302	0.0000	0.00038	17.52	No	
3	0.523	0.0000	0.533	0.0147	9.13	0.544	0.0000	0.00019	8.85	No	
4	0.674	0.0000	0.691	0.0126	7.84	0.704	0.0000	0.00026	11.78	No	
5	0.747	0.0000	0.765	0.0183	11.35	0.778	0.0000	0.00032	14.47	No	
6	0.931	0.0000	0.938	0.0568	35.27	0.944	0.0022	0.00040	18.35	No	
7	0.944	0.0022	0.955	0.0219	13.62	0.964	0.0000	0.00026	11.86	No	

Track 4:

Type	Sample
Vial ID	s1
Description	[redacted]
Volume	2.0 µl

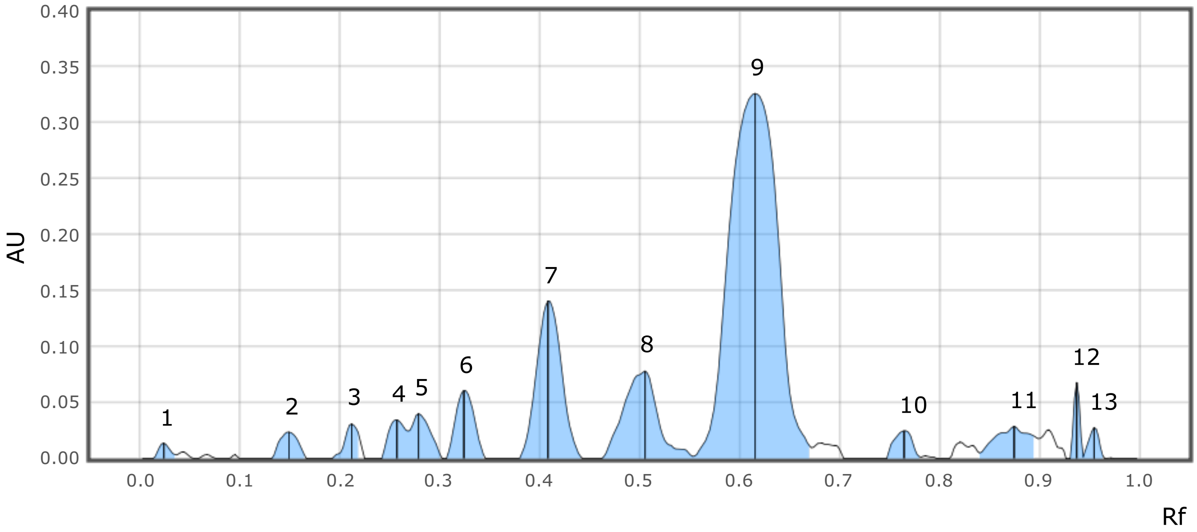

Peak #	Start		Max			End		Area		Manual peak	Substance Name
	Rf	H	Rf	H	%	Rf	H	A	%		
1	0.013	0.0000	0.023	0.0135	1.51	0.036	0.0031	0.00018	0.56	No	
2	0.132	0.0000	0.149	0.0234	2.62	0.166	0.0000	0.00050	1.58	No	
3	0.192	0.0000	0.211	0.0309	3.44	0.224	0.0000	0.00050	1.56	No	
4	0.240	0.0000	0.257	0.0345	3.84	0.268	0.0243	0.00066	2.06	No	
5	0.268	0.0243	0.278	0.0399	4.45	0.302	0.0000	0.00088	2.76	No	
6	0.304	0.0000	0.324	0.0607	6.77	0.345	0.0000	0.00130	4.07	No	
7	0.378	0.0000	0.408	0.1406	15.69	0.443	0.0000	0.00405	12.68	No	
8	0.462	0.0000	0.505	0.0782	8.72	0.553	0.0018	0.00308	9.65	No	CBN
9	0.553	0.0018	0.616	0.3262	36.38	0.672	0.0096	0.01829	57.32	No	9-THC
10	0.747	0.0000	0.765	0.0246	2.74	0.782	0.0012	0.00052	1.63	No	
11	0.840	0.0048	0.875	0.0286	3.19	0.899	0.0177	0.00117	3.66	No	
12	0.931	0.0000	0.938	0.0679	7.57	0.944	0.0012	0.00047	1.47	No	
13	0.944	0.0012	0.955	0.0275	3.07	0.966	0.0000	0.00032	1.01	No	

Track 5:	
Type	Sample
Vial ID	s2
Description	[redacted]
Volume	2.0 µl

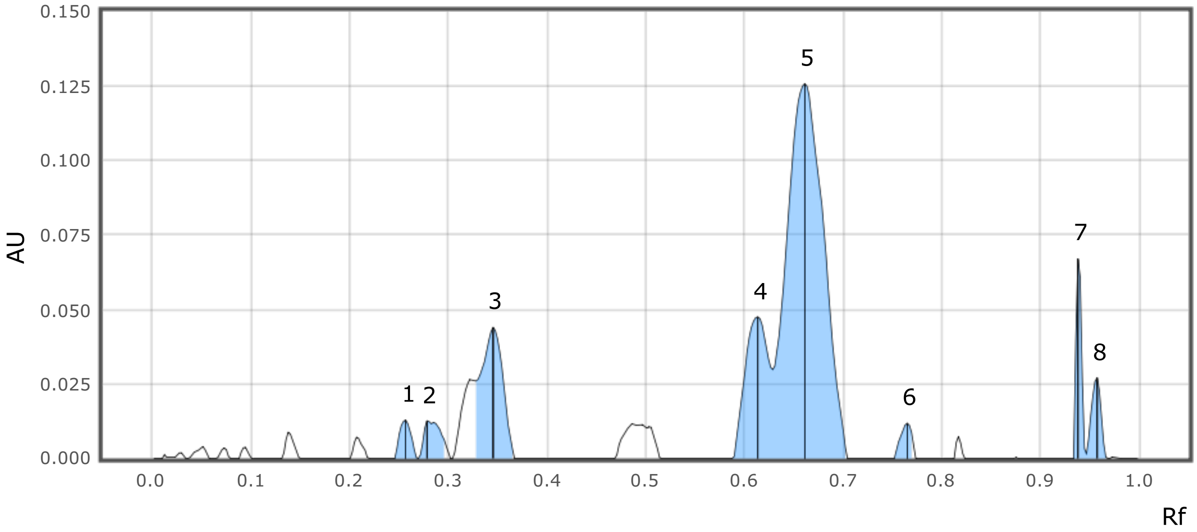

Peak #	Start		Max			End		Area		Manual peak	Substance Name
	Rf	H	Rf	H	%	Rf	H	A	%		
1	0.246	0.0000	0.257	0.0129	3.69	0.270	0.0000	0.00017	1.86	No	
2	0.270	0.0000	0.278	0.0126	3.62	0.302	0.0000	0.00025	2.79	No	
3	0.328	0.0259	0.345	0.0440	12.63	0.367	0.0000	0.00109	12.00	No	
4	0.588	0.0000	0.613	0.0474	13.62	0.629	0.0297	0.00129	14.19	No	
5	0.629	0.0297	0.661	0.1256	36.08	0.704	0.0000	0.00542	59.50	No	CBD
6	0.752	0.0000	0.765	0.0118	3.39	0.773	0.0000	0.00016	1.73	No	
7	0.933	0.0000	0.938	0.0669	19.21	0.946	0.0014	0.00043	4.69	No	
8	0.946	0.0014	0.957	0.0270	7.75	0.968	0.0000	0.00029	3.24	No	

Track 6:

Type	Sample
Vial ID	s3
Description	[redacted]
Volume	2.0 µl

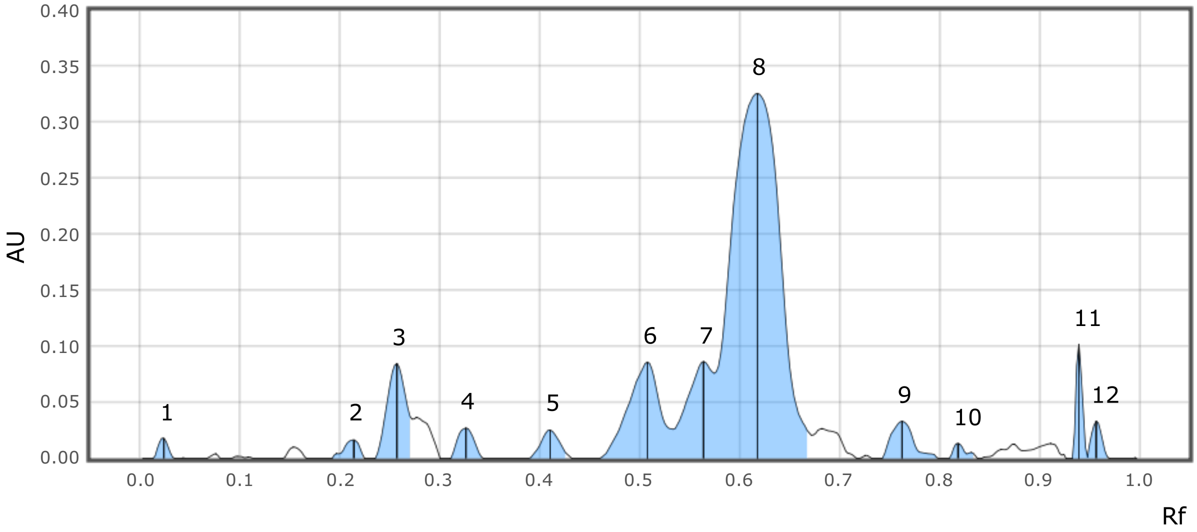

Peak	Start		Max			End		Area		Manual	Substance
#	Rf	H	Rf	H	%	Rf	H	A	%	peak	Name
1	0.013	0.0000	0.023	0.0179	2.10	0.034	0.0000	0.00020	0.67	No	
2	0.192	0.0000	0.214	0.0165	1.94	0.224	0.0000	0.00029	0.99	No	
3	0.235	0.0000	0.257	0.0846	9.94	0.272	0.0350	0.00185	6.29	No	
4	0.311	0.0000	0.326	0.0270	3.18	0.343	0.0000	0.00050	1.70	No	
5	0.389	0.0000	0.410	0.0253	2.97	0.432	0.0000	0.00053	1.80	No	
6	0.460	0.0000	0.508	0.0860	10.11	0.531	0.0262	0.00311	10.58	No	CBN
7	0.533	0.0259	0.564	0.0865	10.16	0.575	0.0758	0.00255	8.66	No	
8	0.575	0.0758	0.618	0.3260	38.31	0.672	0.0207	0.01827	62.06	No	9-THC
9	0.741	0.0000	0.763	0.0331	3.89	0.799	0.0000	0.00084	2.85	No	
10	0.810	0.0000	0.819	0.0133	1.56	0.838	0.0000	0.00019	0.63	No	
11	0.933	0.0000	0.940	0.1019	11.97	0.948	0.0009	0.00072	2.44	No	
12	0.948	0.0009	0.957	0.0331	3.89	0.970	0.0000	0.00038	1.31	No	

Track 7:

Type	Sample
Vial ID	s4
Description	[redacted]
Volume	2.0 µl

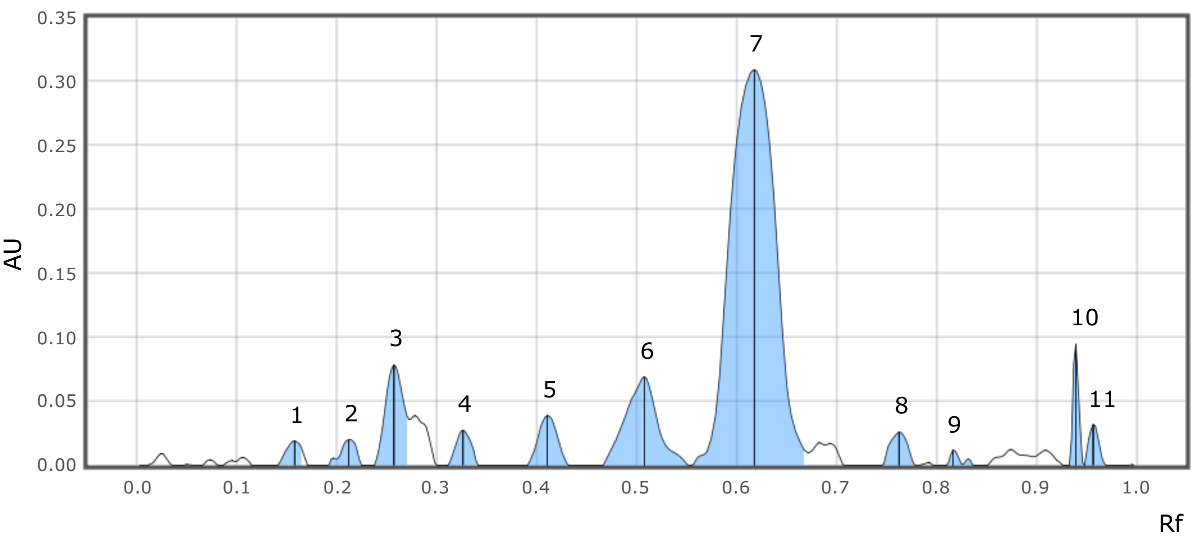

Peak	Start		Max			End		Area		Manual	Substance
#	Rf	H	Rf	H	%	Rf	H	A	%	peak	Name
1	0.140	0.0000	0.157	0.0191	2.63	0.170	0.0000	0.00034	1.43	No	
2	0.190	0.0000	0.211	0.0200	2.75	0.224	0.0000	0.00036	1.51	No	
3	0.237	0.0000	0.257	0.0786	10.81	0.272	0.0359	0.00166	6.88	No	
4	0.311	0.0000	0.326	0.0275	3.79	0.341	0.0000	0.00047	1.96	No	
5	0.391	0.0000	0.410	0.0388	5.34	0.432	0.0000	0.00084	3.49	No	
6	0.467	0.0000	0.508	0.0693	9.54	0.553	0.0000	0.00256	10.62	No	CBN
7	0.555	0.0000	0.618	0.3092	42.55	0.672	0.0097	0.01620	67.31	No	9-THC
8	0.745	0.0000	0.763	0.0259	3.57	0.780	0.0000	0.00051	2.13	No	
9	0.810	0.0000	0.817	0.0118	1.62	0.838	0.0000	0.00013	0.55	No	
10	0.933	0.0000	0.940	0.0946	13.02	0.948	0.0000	0.00063	2.62	No	
11	0.948	0.0000	0.957	0.0318	4.38	0.970	0.0000	0.00036	1.51	No	

Track 8:

Type	Sample
Vial ID	s5
Description	[redacted]
Volume	2.0 µl

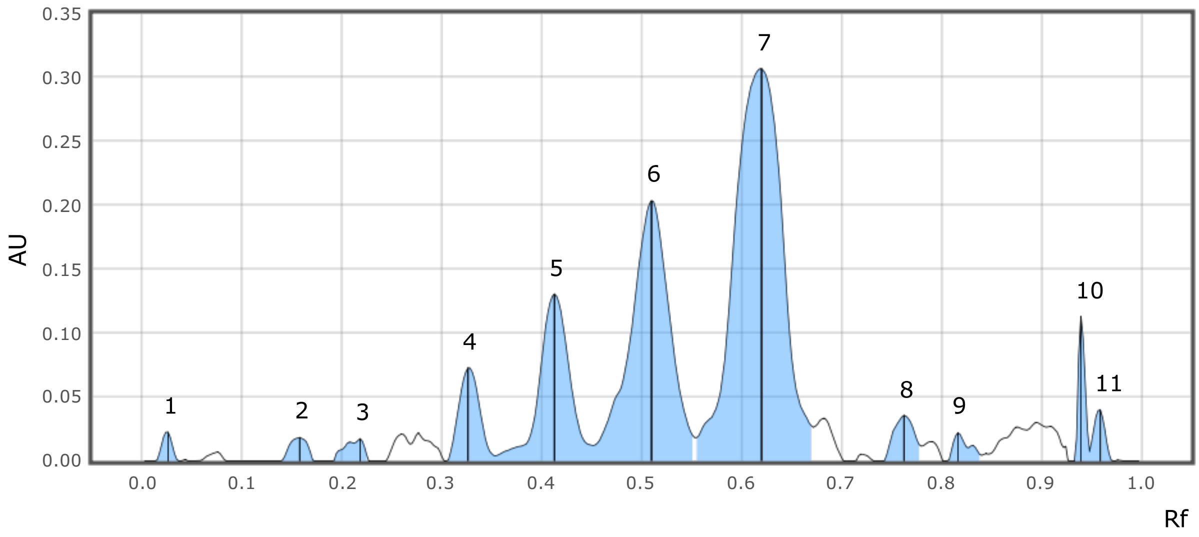

Peak #	Start Rf	Start H	Max Rf	Max H	Max %	End Rf	End H	Area A	Area %	Manual peak	Substance Name
1	0.013	0.0000	0.026	0.0225	2.29	0.036	0.0000	0.00026	0.71	No	
2	0.138	0.0000	0.157	0.0183	1.87	0.173	0.0000	0.00038	1.03	No	
3	0.190	0.0000	0.218	0.0175	1.78	0.227	0.0000	0.00039	1.06	No	
4	0.304	0.0000	0.326	0.0727	7.40	0.354	0.0040	0.00174	4.77	No	
5	0.354	0.0040	0.412	0.1305	13.29	0.449	0.0123	0.00451	12.36	No	
6	0.451	0.0120	0.510	0.2036	20.72	0.553	0.0183	0.00929	25.45	No	CBN
7	0.555	0.0182	0.620	0.3068	31.22	0.672	0.0263	0.01731	47.41	No	9-THC
8	0.741	0.0000	0.763	0.0356	3.62	0.780	0.0116	0.00085	2.34	No	
9	0.806	0.0000	0.817	0.0220	2.24	0.840	0.0044	0.00041	1.11	No	
10	0.933	0.0000	0.940	0.1132	11.52	0.948	0.0073	0.00086	2.36	No	
11	0.948	0.0073	0.959	0.0397	4.04	0.972	0.0000	0.00051	1.41	No	

Track 9:

Type	Sample
Vial ID	s6
Description	[redacted]
Volume	2.0 µl

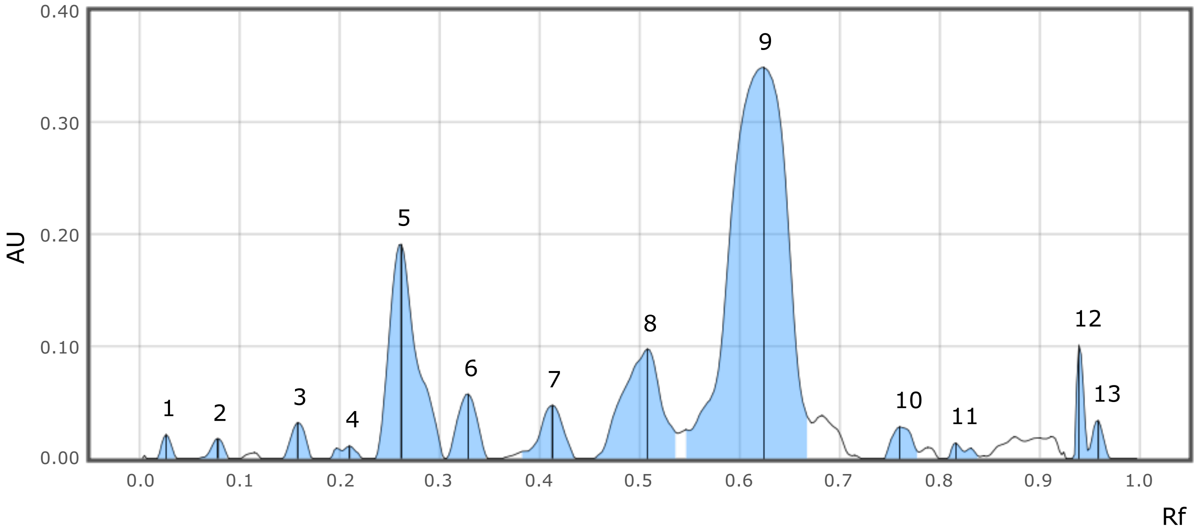

Peak #	Start		Max			End		Area		Manual peak	Substance Name
	Rf	H	Rf	H	%	Rf	H	A	%		
1	0.017	0.0000	0.026	0.0213	2.13	0.036	0.0000	0.00022	0.56	No	
2	0.058	0.0000	0.078	0.0177	1.77	0.088	0.0000	0.00024	0.62	No	
3	0.142	0.0000	0.157	0.0321	3.20	0.173	0.0000	0.00052	1.32	No	
4	0.190	0.0000	0.209	0.0111	1.10	0.222	0.0000	0.00022	0.56	No	
5	0.235	0.0000	0.261	0.1915	19.08	0.304	0.0000	0.00604	15.38	No	
6	0.307	0.0000	0.328	0.0576	5.74	0.348	0.0000	0.00128	3.26	No	
7	0.380	0.0055	0.412	0.0475	4.74	0.436	0.0000	0.00126	3.20	No	
8	0.454	0.0000	0.508	0.0978	9.75	0.538	0.0224	0.00433	11.02	No	CBN
9	0.542	0.0239	0.624	0.3494	34.82	0.672	0.0317	0.02309	58.78	No	9-THC
10	0.745	0.0000	0.760	0.0282	2.81	0.780	0.0057	0.00064	1.64	No	
11	0.808	0.0000	0.817	0.0139	1.39	0.840	0.0015	0.00024	0.61	No	
12	0.933	0.0000	0.940	0.1013	10.09	0.948	0.0071	0.00078	1.98	No	
13	0.948	0.0071	0.959	0.0339	3.38	0.972	0.0000	0.00042	1.07	No	

Track 10:	
Type	Sample
Vial ID	s7
Description	[redacted]
Volume	2.0 µl

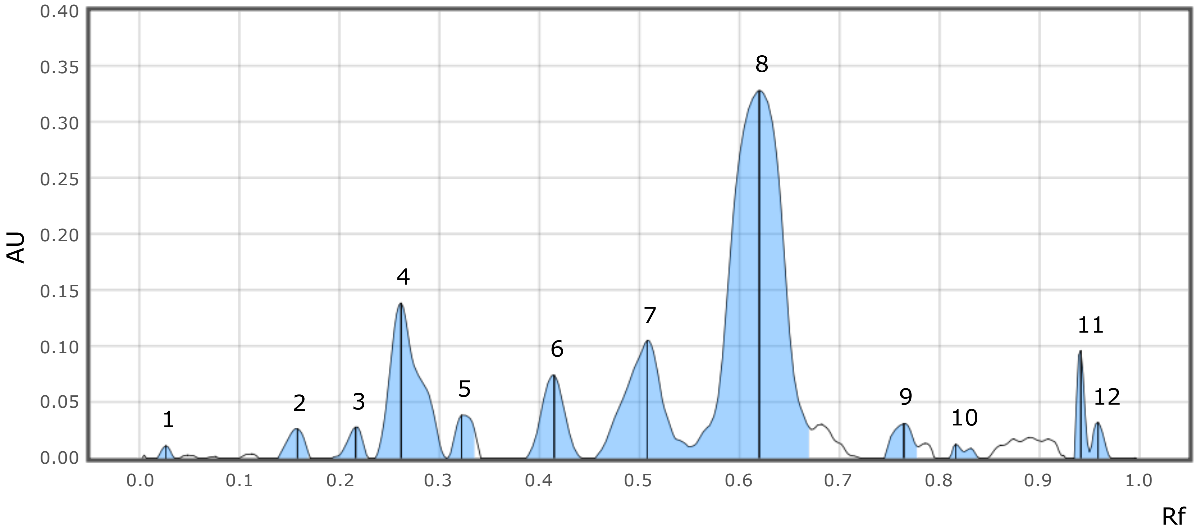

Peak #	Start		Max			End		Area		Manual peak	Substance Name
	Rf	H	Rf	H	%	Rf	H	A	%		
1	0.017	0.0000	0.026	0.0111	1.20	0.036	0.0000	0.00011	0.31	No	
2	0.138	0.0000	0.157	0.0265	2.87	0.170	0.0000	0.00051	1.47	No	
3	0.192	0.0000	0.216	0.0278	3.01	0.229	0.0000	0.00047	1.35	No	
4	0.235	0.0000	0.261	0.1387	15.04	0.307	0.0000	0.00465	13.49	No	
5	0.307	0.0000	0.322	0.0384	4.16	0.341	0.0000	0.00081	2.34	No	
6	0.384	0.0000	0.415	0.0741	8.03	0.443	0.0000	0.00194	5.62	No	
7	0.454	0.0000	0.508	0.1050	11.39	0.551	0.0103	0.00456	13.20	No	CBN
8	0.551	0.0103	0.620	0.3287	35.64	0.672	0.0256	0.01935	56.08	No	9-THC
9	0.743	0.0000	0.765	0.0309	3.35	0.780	0.0103	0.00073	2.11	No	
10	0.810	0.0000	0.817	0.0126	1.36	0.840	0.0000	0.00021	0.59	No	
11	0.935	0.0000	0.942	0.0963	10.45	0.951	0.0055	0.00080	2.32	No	
12	0.951	0.0055	0.959	0.0322	3.49	0.972	0.0000	0.00038	1.11	No	

Track 11:	
Type	Sample
Vial ID	s8
Description	[redacted]
Volume	2.0 µl

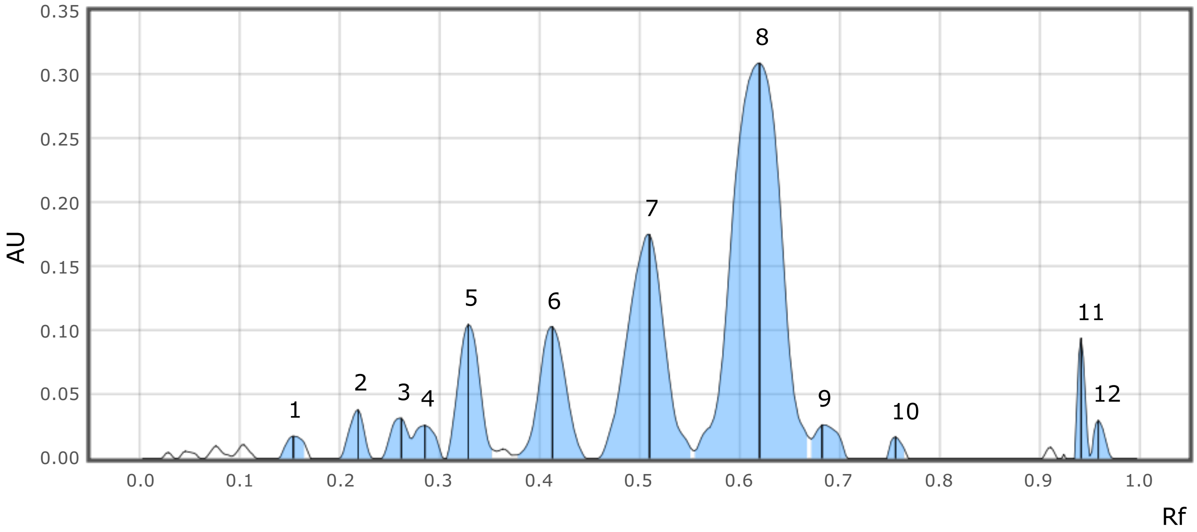

Peak #	Start Rf	Start H	Max Rf	Max H	Max %	End Rf	End H	Area A	Area %	Manual peak	Substance Name
1	0.138	0.0000	0.153	0.0175	1.80	0.170	0.0000	0.00036	1.04	No	
2	0.199	0.0000	0.218	0.0383	3.94	0.233	0.0000	0.00063	1.81	No	
3	0.242	0.0000	0.261	0.0315	3.24	0.272	0.0155	0.00062	1.81	No	
4	0.272	0.0155	0.285	0.0259	2.66	0.304	0.0000	0.00059	1.72	No	
5	0.307	0.0000	0.328	0.1048	10.77	0.356	0.0056	0.00258	7.46	No	
6	0.374	0.0025	0.412	0.1030	10.59	0.447	0.0000	0.00317	9.18	No	
7	0.458	0.0000	0.510	0.1753	18.02	0.553	0.0064	0.00739	21.37	No	CBN
8	0.555	0.0060	0.620	0.3094	31.81	0.670	0.0162	0.01718	49.70	No	9-THC
9	0.672	0.0151	0.683	0.0263	2.70	0.709	0.0000	0.00070	2.01	No	
10	0.747	0.0000	0.756	0.0168	1.73	0.769	0.0000	0.00023	0.65	No	
11	0.935	0.0000	0.942	0.0940	9.67	0.951	0.0018	0.00076	2.21	No	
12	0.951	0.0018	0.959	0.0297	3.06	0.974	0.0000	0.00036	1.04	No	

Track 12:	
Type	Sample
Vial ID	s9
Description	[redacted]
Volume	2.0 µl

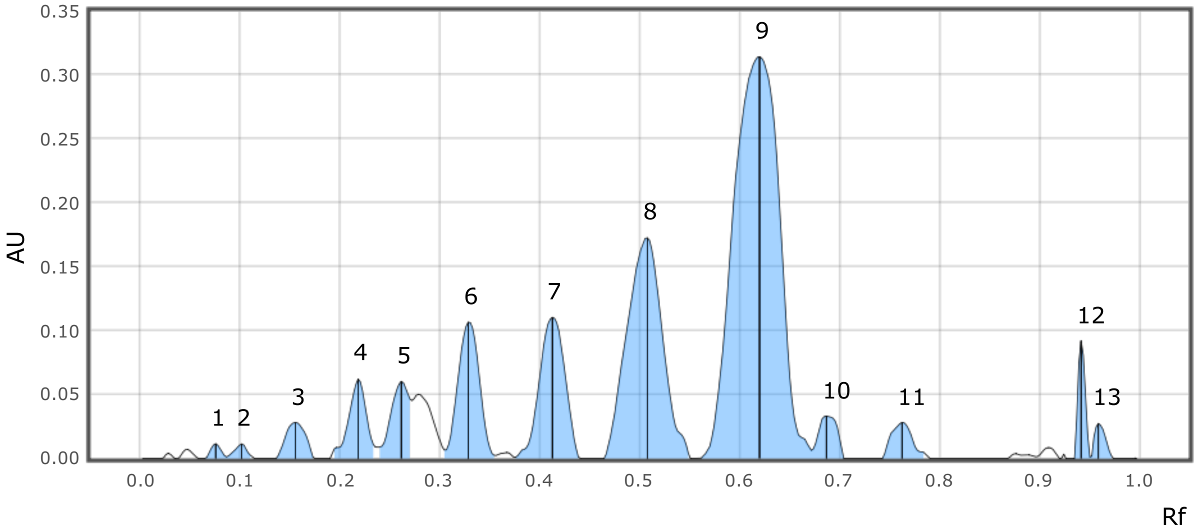

Peak #	Start Rf	Start H	Max Rf	Max H	Max %	End Rf	End H	Area A	Area %	Manual peak	Substance Name
1	0.065	0.0000	0.075	0.0115	1.09	0.086	0.0011	0.00013	0.37	No	
2	0.086	0.0011	0.101	0.0114	1.07	0.114	0.0000	0.00016	0.45	No	
3	0.136	0.0000	0.155	0.0281	2.66	0.175	0.0000	0.00064	1.80	No	
4	0.192	0.0037	0.218	0.0621	5.87	0.235	0.0088	0.00126	3.54	No	
5	0.240	0.0088	0.261	0.0602	5.69	0.272	0.0453	0.00129	3.63	No	
6	0.304	0.0065	0.328	0.1065	10.08	0.356	0.0023	0.00264	7.40	No	
7	0.376	0.0009	0.412	0.1103	10.43	0.441	0.0000	0.00324	9.10	No	
8	0.464	0.0000	0.508	0.1725	16.32	0.551	0.0000	0.00709	19.91	No	CBN
9	0.559	0.0000	0.620	0.3143	29.72	0.670	0.0088	0.01666	46.76	No	9-THC
10	0.672	0.0062	0.687	0.0331	3.13	0.706	0.0000	0.00073	2.04	No	
11	0.743	0.0000	0.763	0.0281	2.66	0.791	0.0000	0.00069	1.94	No	
12	0.935	0.0000	0.942	0.0919	8.69	0.951	0.0011	0.00075	2.11	No	
13	0.951	0.0011	0.959	0.0273	2.58	0.974	0.0000	0.00034	0.96	No	

Track 13:

Type	Sample
Vial ID	s10
Description	[redacted]
Volume	2.0 µl

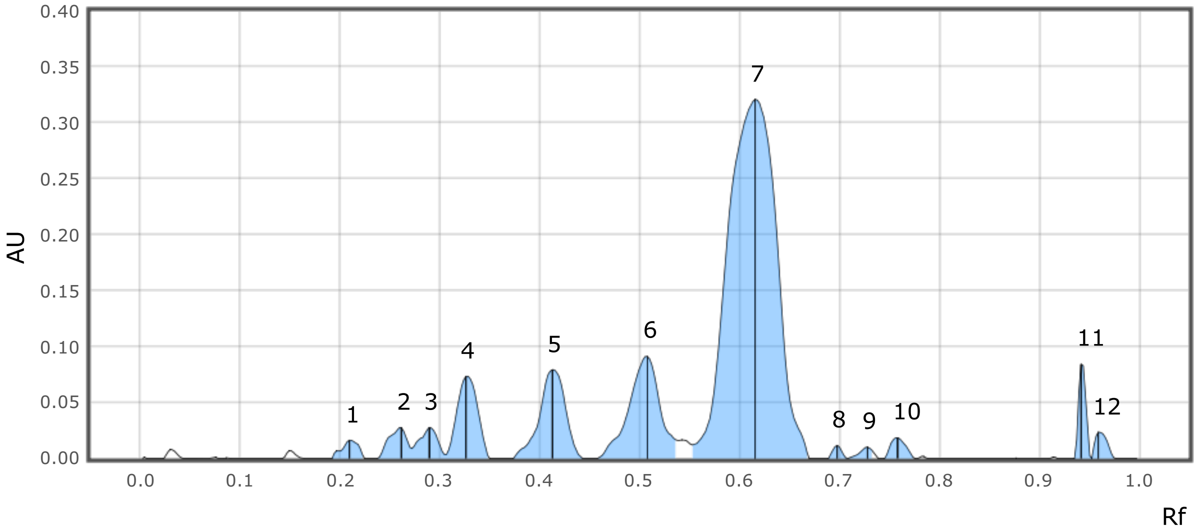

Peak #	Start Rf	Start H	Max Rf	Max H	Max %	End Rf	End H	Area A	Area %	Manual peak	Substance Name
1	0.192	0.0000	0.209	0.0163	2.08	0.224	0.0000	0.00031	1.10	No	
2	0.237	0.0000	0.261	0.0273	3.49	0.272	0.0089	0.00058	2.02	No	
3	0.272	0.0089	0.289	0.0277	3.53	0.304	0.0034	0.00057	2.00	No	
4	0.304	0.0034	0.326	0.0734	9.36	0.350	0.0000	0.00175	6.12	No	
5	0.374	0.0000	0.412	0.0792	10.11	0.445	0.0000	0.00237	8.28	No	
6	0.458	0.0000	0.508	0.0911	11.62	0.538	0.0161	0.00327	11.44	No	CBN
7	0.553	0.0121	0.616	0.3209	40.95	0.670	0.0000	0.01812	63.36	No	9-THC
8	0.689	0.0000	0.698	0.0116	1.48	0.709	0.0000	0.00012	0.40	No	
9	0.709	0.0000	0.728	0.0100	1.28	0.739	0.0000	0.00015	0.53	No	
10	0.745	0.0000	0.758	0.0184	2.34	0.776	0.0000	0.00032	1.12	No	
11	0.935	0.0000	0.942	0.0842	10.75	0.953	0.0000	0.00071	2.49	No	
12	0.953	0.0000	0.959	0.0235	3.00	0.977	0.0000	0.00032	1.14	No	

Track 14:

Type	Reference
Vial ID	250ug/mL mix
Description	250ug/mL
Volume	2.0 µl

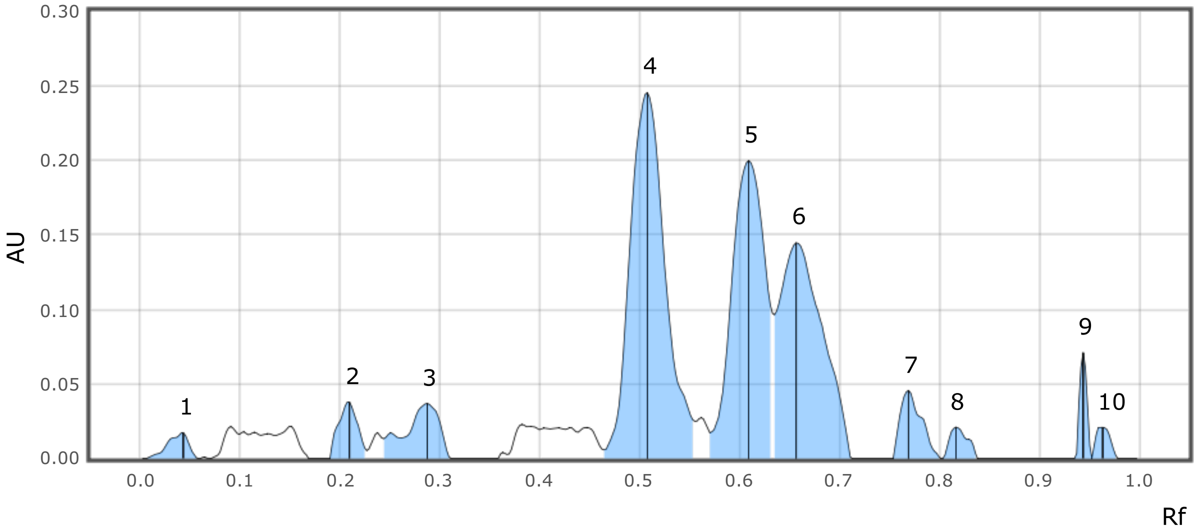

Peak #	Start		Max			End		Area		Manual peak	Substance Name
	Rf	H	Rf	H	%	Rf	H	A	%		
1	0.006	0.0000	0.043	0.0170	2.03	0.058	0.0000	0.00041	1.37	No	
2	0.190	0.0000	0.209	0.0377	4.49	0.227	0.0053	0.00084	2.80	No	
3	0.244	0.0132	0.287	0.0369	4.40	0.311	0.0000	0.00143	4.75	No	
4	0.464	0.0057	0.508	0.2453	29.22	0.555	0.0250	0.01003	33.43	No	CBN
5	0.570	0.0172	0.609	0.1998	23.80	0.633	0.0974	0.00766	25.54	No	9-THC
6	0.635	0.0963	0.657	0.1445	17.22	0.713	0.0000	0.00714	23.80	No	CBD
7	0.754	0.0000	0.769	0.0456	5.43	0.801	0.0000	0.00111	3.70	No	
8	0.804	0.0000	0.817	0.0209	2.49	0.838	0.0000	0.00044	1.46	No	
9	0.935	0.0000	0.944	0.0709	8.44	0.953	0.0000	0.00060	2.00	No	
10	0.953	0.0000	0.964	0.0207	2.47	0.979	0.0000	0.00035	1.15	No	

Track 15:	
Type	Sample
Vial ID	MeOH blank
Description	MeOH Blank
Volume	2.0 µl

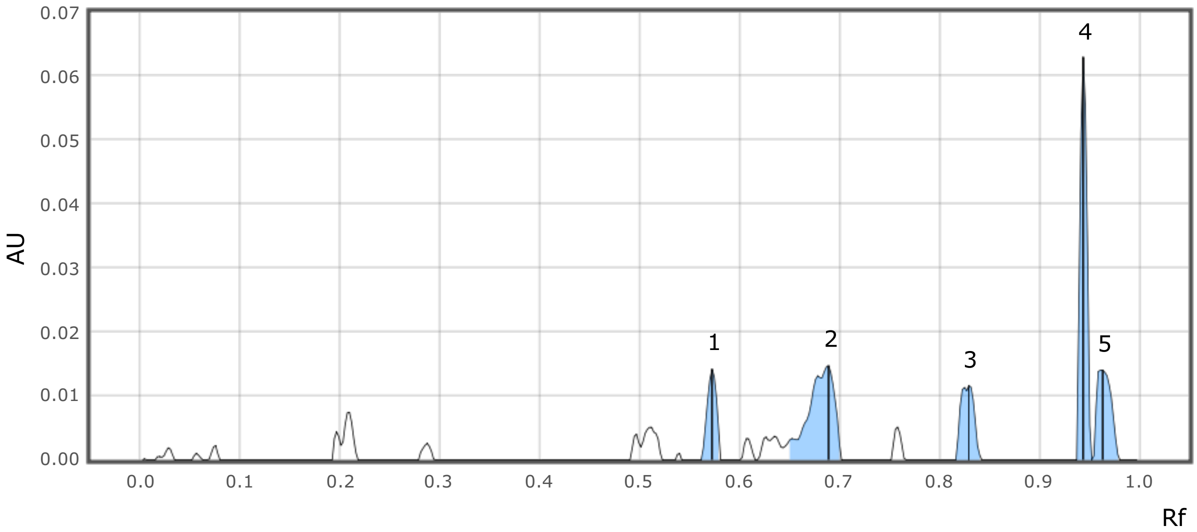

Peak	Start		Max			End		Area		Manual	Substance
#	Rf	H	Rf	H	%	Rf	H	A	%	peak	Name
1	0.559	0.0000	0.572	0.0142	12.08	0.581	0.0000	0.00015	10.17	No	
2	0.650	0.0031	0.689	0.0147	12.53	0.702	0.0000	0.00043	28.76	No	
3	0.817	0.0000	0.830	0.0116	9.88	0.843	0.0000	0.00019	12.30	No	
4	0.938	0.0000	0.944	0.0629	53.59	0.953	0.0000	0.00050	33.05	No	
5	0.953	0.0000	0.964	0.0140	11.91	0.981	0.0000	0.00024	15.72	No	

Calibration results:

Height calibration for substance 9-THC @ RT White:

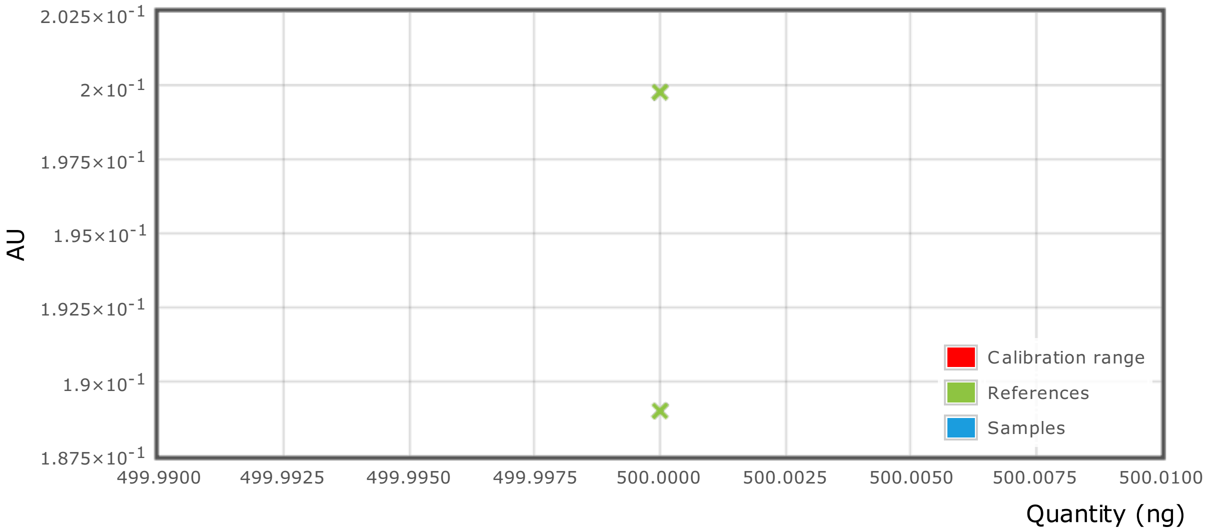

Regression mode	Linear-2
Range deviation	5.00 %
Related substances	Default
Number of references	2
Calibration function	$y=0x$
Coefficient of variation	CV 0.00 %
Correlation coefficient	n/a
⚠	Unable to compute the results for this substance because there wasn't eno ugh groups of references replicas (at least 1 for Linear-1, 2 for Linear2 and Mime-1 and 3 for Polynomial and MiMe-2)

Height calibration for substance CBD @ RT White:

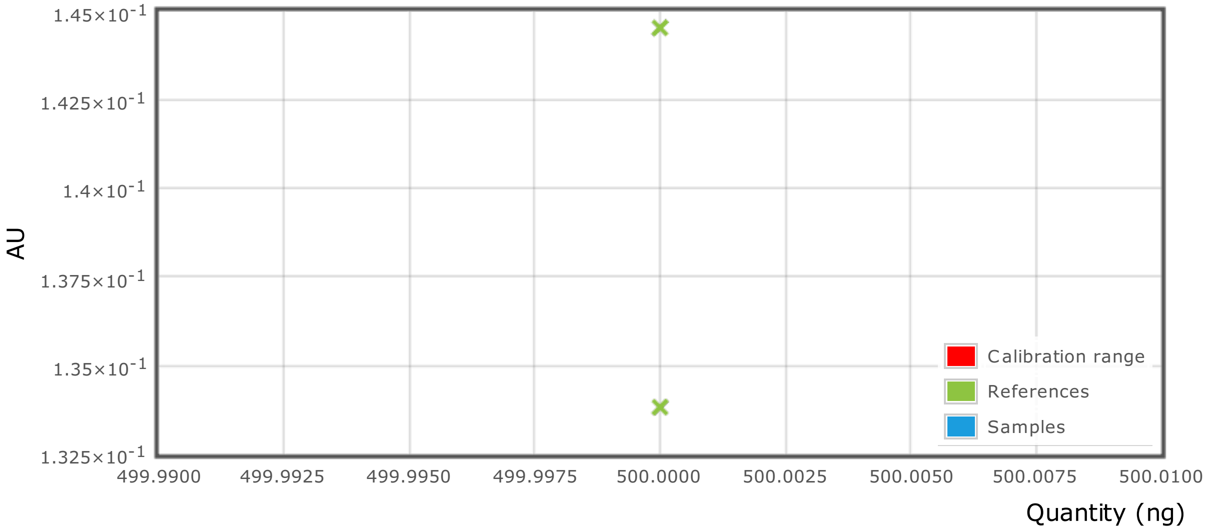

Regression mode	Linear-2
Range deviation	5.00 %
Related substances	Default
Number of references	2
Calibration function	$y=0x$
Coefficient of variation	CV 0.00 %
Correlation coefficient	n/a
⚠	Unable to compute the results for this substance because there wasn't eno ugh groups of references replicas (at least 1 for Linear-1, 2 for Linear2 and Mime-1 and 3 for Polynomial and MiMe-2)

Height calibration for substance CBN @ RT White:

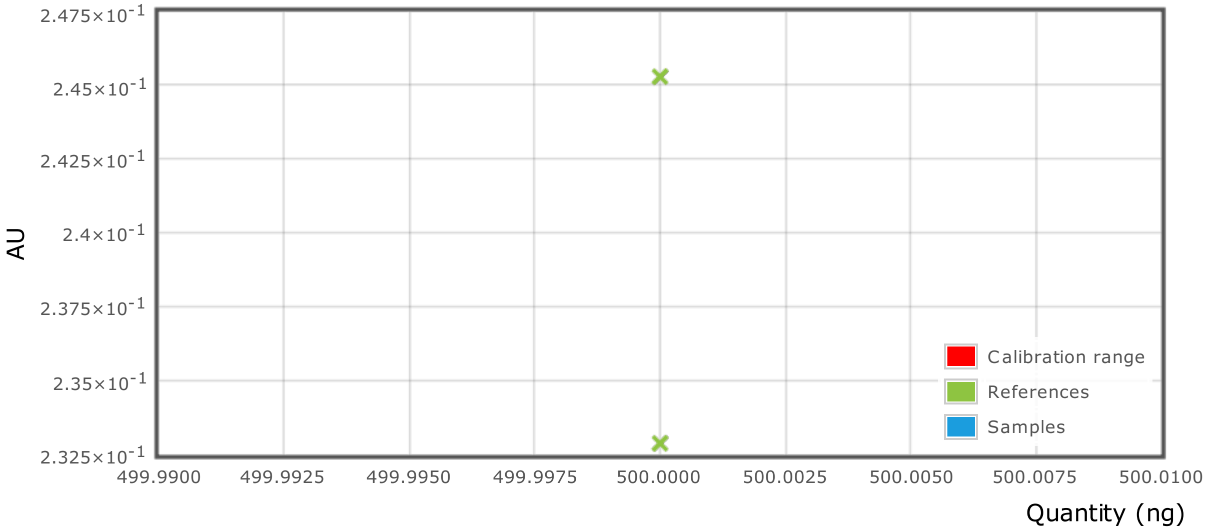

Regression mode	Linear-2
Range deviation	5.00 %
Related substances	Default
Number of references	2
Calibration function	$y=0x$
Coefficient of variation	CV 0.00 %
Correlation coefficient	n/a
⚠	Unable to compute the results for this substance because there wasn't enough groups of references replicas (at least 1 for Linear-1, 2 for Linear2 and Mime-1 and 3 for Polynomial and MiMe-2)

Results:

Substance having no available results		
⚠	CBD	Unable to compute the results for this substance because there wasn't enough groups of references replicas (at least 1 for Linear-1, 2 for Linear2 and Mime-1 and 3 for Polynomial and MiMe-2)
⚠	CBN	Unable to compute the results for this substance because there wasn't enough groups of references replicas (at least 1 for Linear-1, 2 for Linear2 and Mime-1 and 3 for Polynomial and MiMe-2)
⚠	9-THC	Unable to compute the results for this substance because there wasn't enough groups of references replicas (at least 1 for Linear-1, 2 for Linear2 and Mime-1 and 3 for Polynomial and MiMe-2)

A track marked with ⚠ means: this result is outside the regression range given by the reference assignments, but is included in the results because it is in the allowed range deviation.

Analyst:	Reviewer:
