# Supplementary material for: High Performance Thin-Layer Chromatography (HPTLC) data of Cannabinoids in ten mobile phase systems
Source: Data Brief. 2020 Jun 30;31:105955. doi: 10.1016/j.dib.2020.105955 (PMC7352075; doi:10.1016/j.dib.2020.105955)
Supplement: Supplementary file 1 [file mmc1.zip › S4-Case sample reports/6DaT-sample run-5.pdf]

Analysis: 6DaT-sample run-5

Path: Home/YL Research

Based on method: Samples (no cal)

Created	11-Oct-2019 16:06:35	visionCATSuser
Modified	11-Oct-2019 18:15:38	visionCATSuser
Last HPTLC log	11-Oct-2019 18:15:38	Analysis modified
Explorer notes		

Track	Vial ID	Description	Volume	Position	Type
1	MeOH blank	MeOH Blank	2.0 µl	A1	Sample
2	250ug/mL mix	250ug/mL	2.0 µl	A2	Reference
3	Tetracosane	Tetracosane IS	2.0 µl	A3	Sample
4	s1	[redacted]	2.0 µl	B1	Sample
5	s2	[redacted]	2.0 µl	B2	Sample
6	s3	[redacted]	2.0 µl	B3	Sample
7	s4	[redacted]	2.0 µl	B4	Sample
8	s5	[redacted]	2.0 µl	B5	Sample
9	s6	[redacted]	2.0 µl	B6	Sample
10	s7	[redacted]	2.0 µl	B7	Sample
11	s8	[redacted]	2.0 µl	B8	Sample
12	s9	[redacted]	2.0 µl	B9	Sample
13	s10	[redacted]	2.0 µl	B10	Sample
14	250ug/mL mix	250ug/mL	2.0 µl	A2	Reference
15	MeOH blank	MeOH Blank	2.0 µl	A1	Sample
Sequence table notes	*Diluted with 500uL MeOH				

A track marked with ⚠ means: the application type is overridden in some evaluation(s).

System setup:

Software	Server User-PC, version 2.5.18072.1
ATS4	S/N:080713
Chamber	N/A
Derivatization dip	N/A
Scanner3	S/N:031025
Visualizer	S/N:230515

Chromatography

Plate layout:

Stationary phase	Merck, HPTLC plates silica gel 60 F 254
Plate format	200.0 x 100.0 mm
Application type	Band
Application	Position Y: 8.0 mm, length: 8.0 mm, width: 0.0 mm
Track	First position X: 20.0 mm, distance: 11.4 mm
Solvent front position	70.0 mm
Notes	

Take image clean plate 1a - Visualizer (S/N: 230515):

Quality	Enhanced
RT White	auto capture, Auto, level 85 %, Band
R 254	auto capture, Auto, level 85 %, Band
Instrument diagnostics	Valid diagnostics
Documentation step label	
Notes	

Application 1 - ATS 4 (S/N: 080713):

Spray gas	NI
Sample solvent type	Methanol
Filling speed	15 µl/s
Predosage volume	200 nl
Retraction volume	200 nl
Dosage speed	150 nl/s
Filling quality	User
Rinsing cycles / vacuum	2 / 4 s
Filling cycles / vacuum	1 / 4 s
Rinsing solvent name	Methanol
Nozzle temperature	Unheated
Rack in use	Standard
Instrument diagnostics	Valid diagnostics
Notes	

Development 1 - Chamber:

Tank	TTC 20x10
Mobile phase	6% diethylamine in toluene
Saturation time	20 min
Use saturation pad	true
Use smartALERT	false
Volume front through	10 ml
Volume rear through	25 ml
Drying time	5 min
Drying temperature	Room temperature
Notes	

Take image developed plate 1a - Visualizer (S/N: 230515):

Quality	Enhanced
RT White	auto capture, Auto, level 85 %, Band
R 254	auto capture, Auto, level 85 %, Band
R 366	auto capture, Auto, level 85 %, Band
Instrument diagnostics	Valid diagnostics
Documentation step label	
Notes	

Scan developed plate 1b - Scanner 3 (S/N: 031025):

Scanner type	Single λ
Optimization for	Resolution
Measurement mode	Absorption
Filter	n/a
Detector mode	Automatic
Scanning speed	20 mm/s
Data resolution	100 µm/step
Slit	5 x 0.2 mm, micro
Partial scan	No
Lamp	Deuterium & Tungsten
Wavelength(s)	254 nm
Instrument diagnostics	Valid diagnostics
Documentation step label	
Notes	

Derivatization 1 - dip:

Reagent name	
Dipping speed	5
Dipping time	0 s
Reagent preparation	
Heating	100 °C for 3 min, heated after
Notes	

Take image derivatized plate 1a - Visualizer (S/N: 230515):

Quality	Enhanced
RT White	auto capture, Auto, level 85 %, Band
R 366	auto capture, Auto, level 85 %, Band
Instrument diagnostics	Valid diagnostics
Documentation step label	
Notes	

System suitability tests:

SST settings:

SST tracks	

Data acquisition

Application 1 - ATS 4 (S/N: 080713):

Executed	11-Oct-2019 16:11:14 visionCATSuser

Development 1 - Chamber:

Executed	11-Oct-2019 16:57:45 visionCATSuser

Take image developed plate 1a - Visualizer (S/N: 230515):

Executed	11-Oct-2019 17:51:49 visionCATSuser

RT White

Developed, RemTransVis

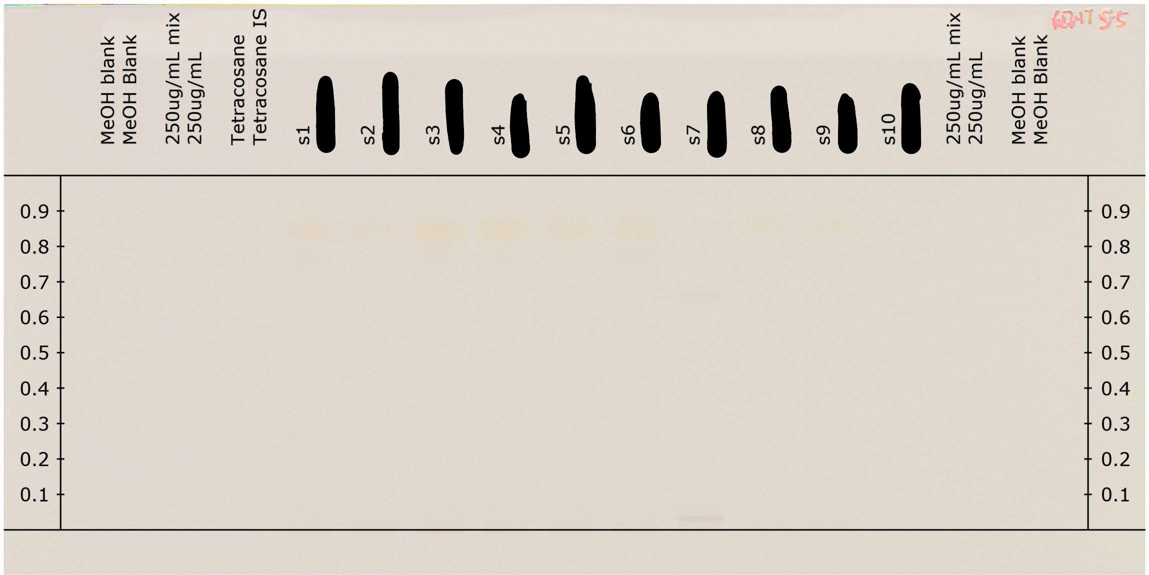

Exposure	0.083 s
Contrast	1
Normalized exposure	Disabled
Clarify	Disabled
White balance	1.00, 1.00, 1.00

R 254

Developed, Remission254

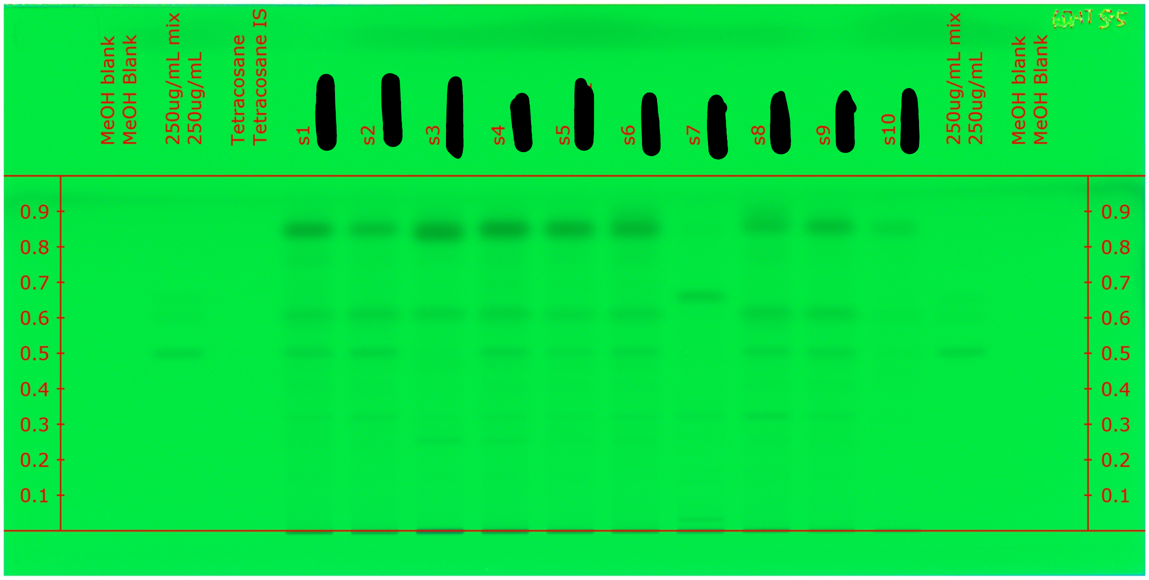

Exposure	0.266 s
Contrast	1
Normalized exposure	Disabled
Clarify	Disabled
White balance	1.00, 1.00, 1.00

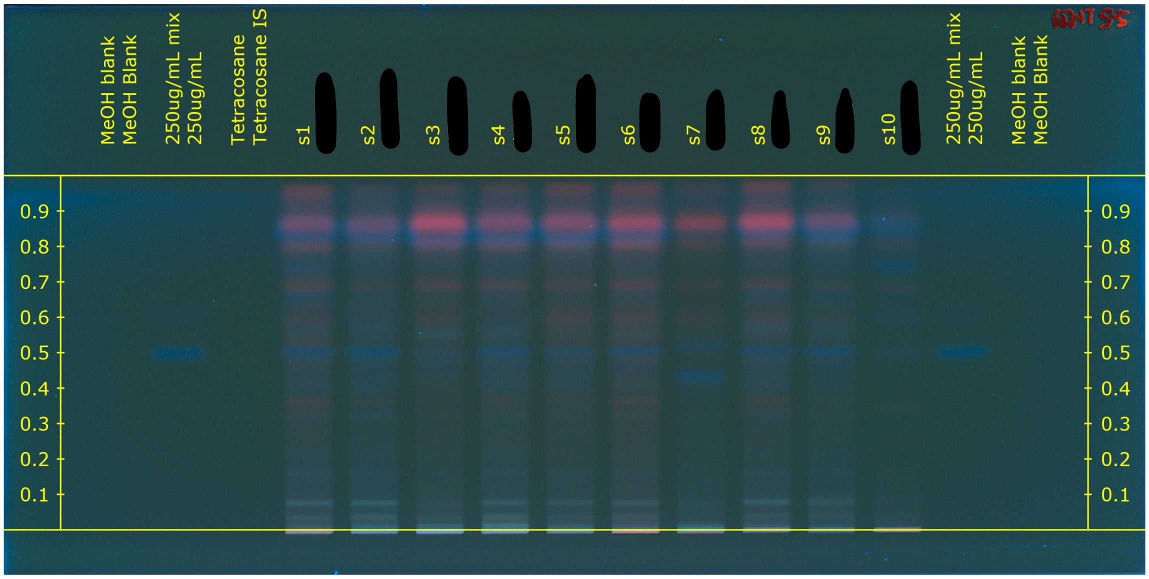

Exposure	4.906 s
Contrast	1
Normalized exposure	Disabled
Clarify	Disabled
White balance	1.00, 1.00, 1.00

Scan developed plate 1b - Scanner 3 (S/N: 031025):

Executed	11-Oct-2019 17:57:57 visionCATSuser

Scan:

Wavelength	254 nm

Track 1:

Type	Single λ

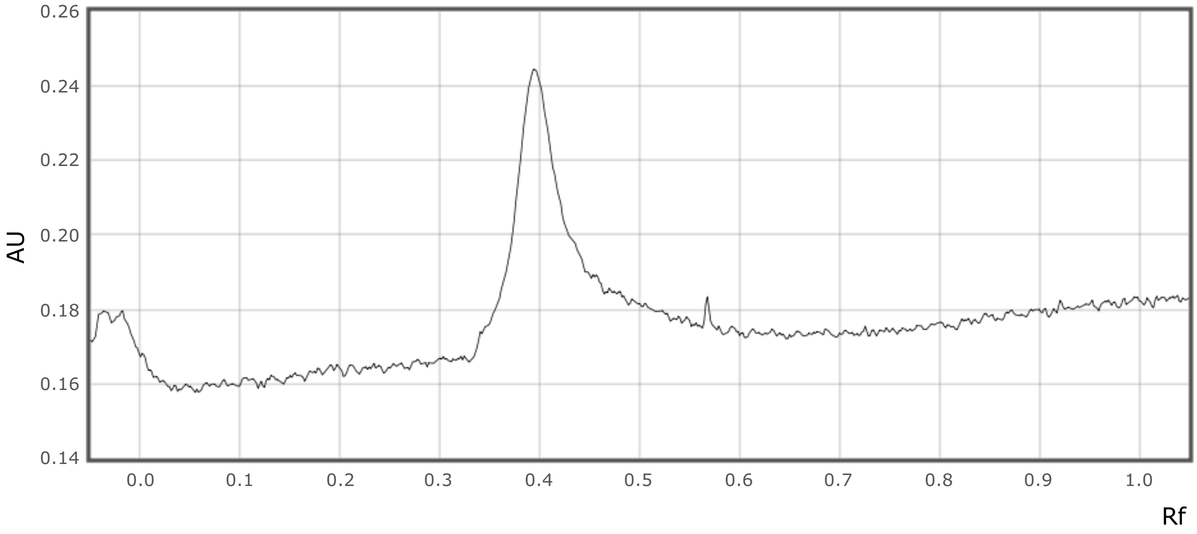

Track 2:

Type	Single λ

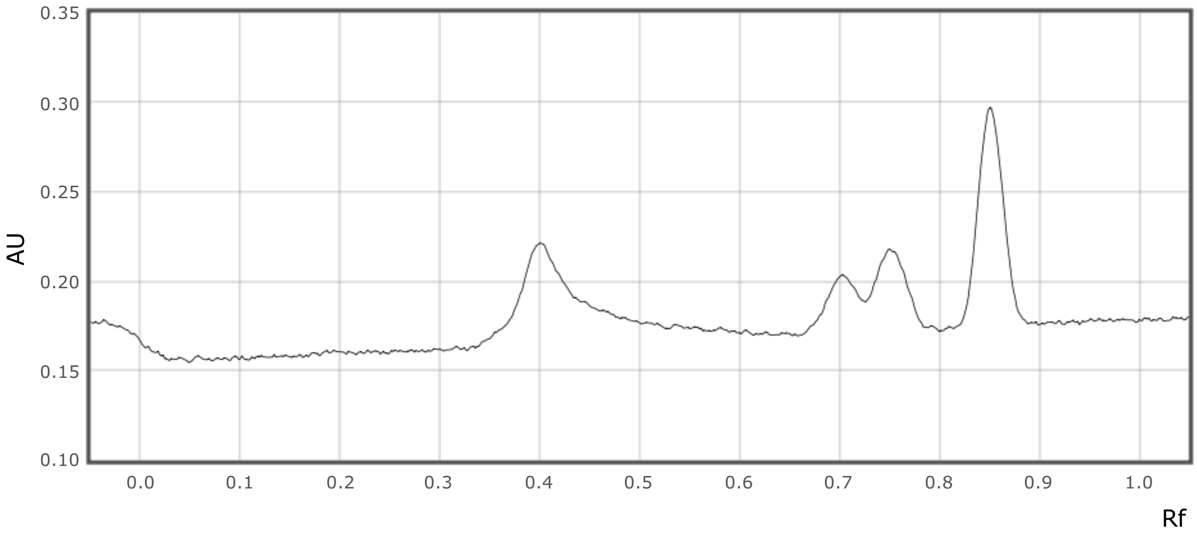

Track 3:

Type	Single λ

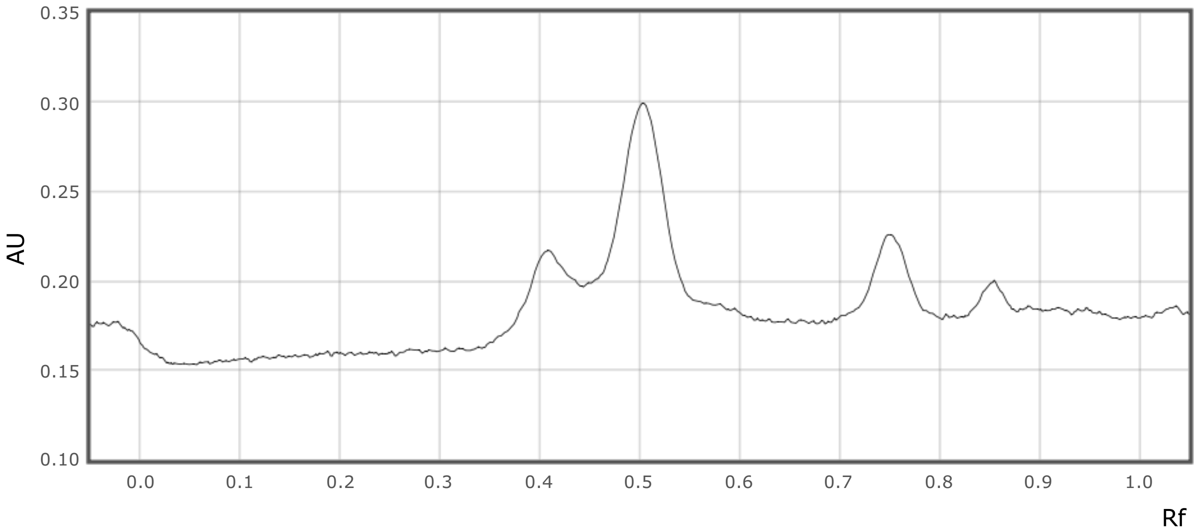

Track 4:

Type	Single λ

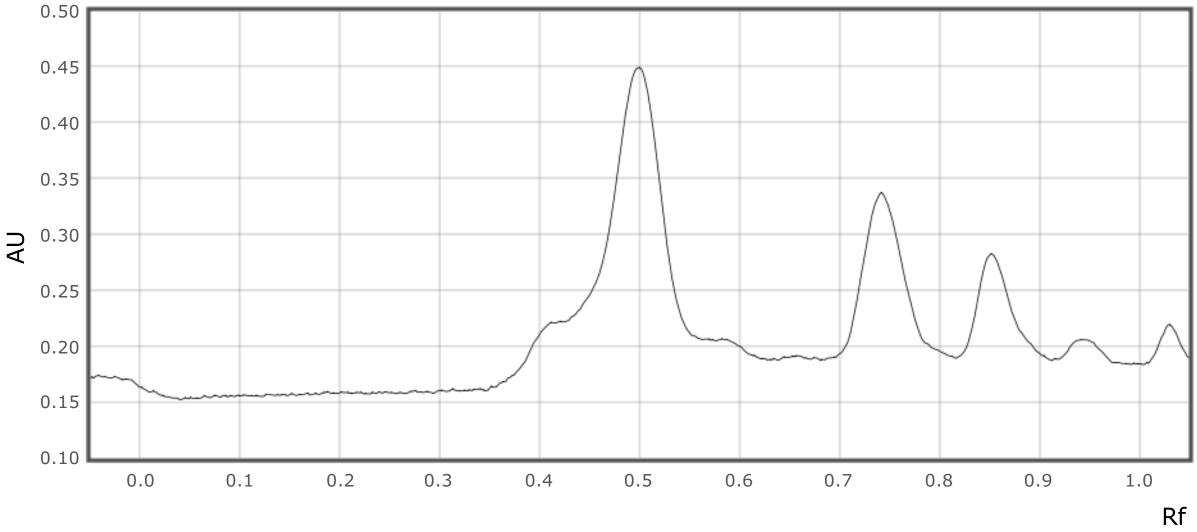

Track 5:	
Type	Single λ

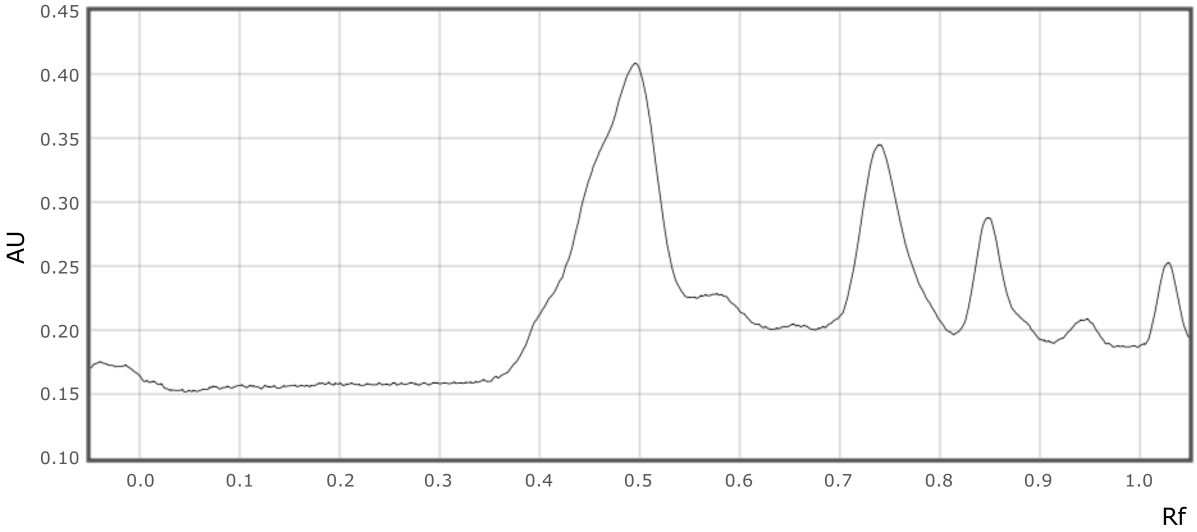

Track 6:	
Type	Single λ

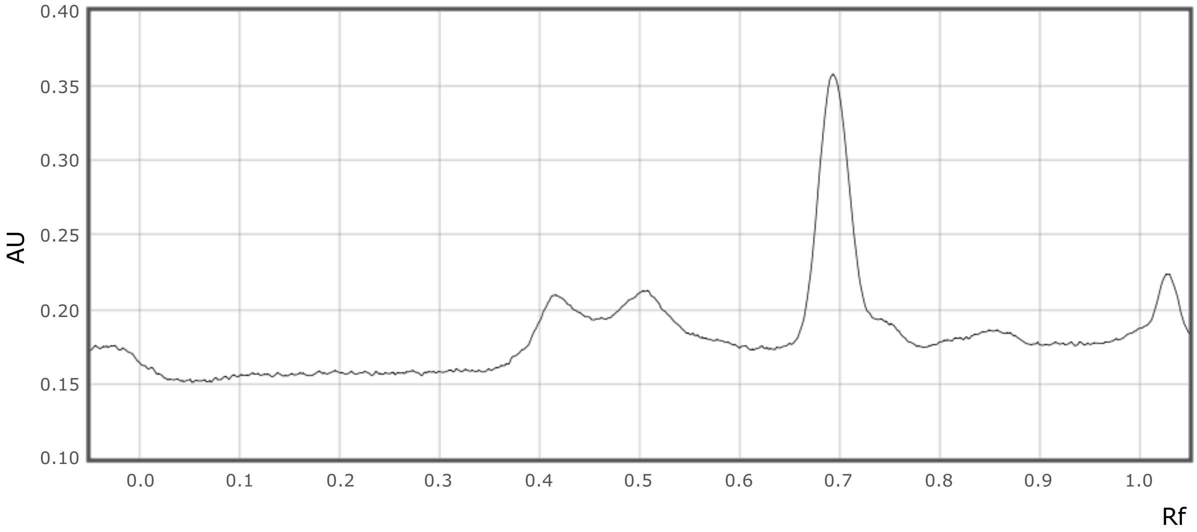

Track 7:	
Type	Single λ

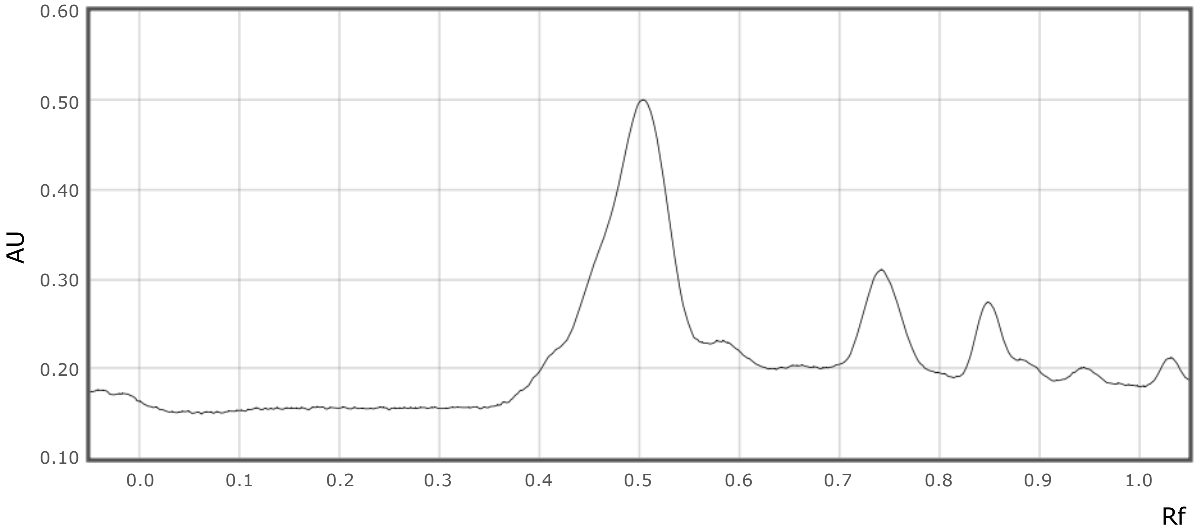

Track 8:	
Type	Single λ

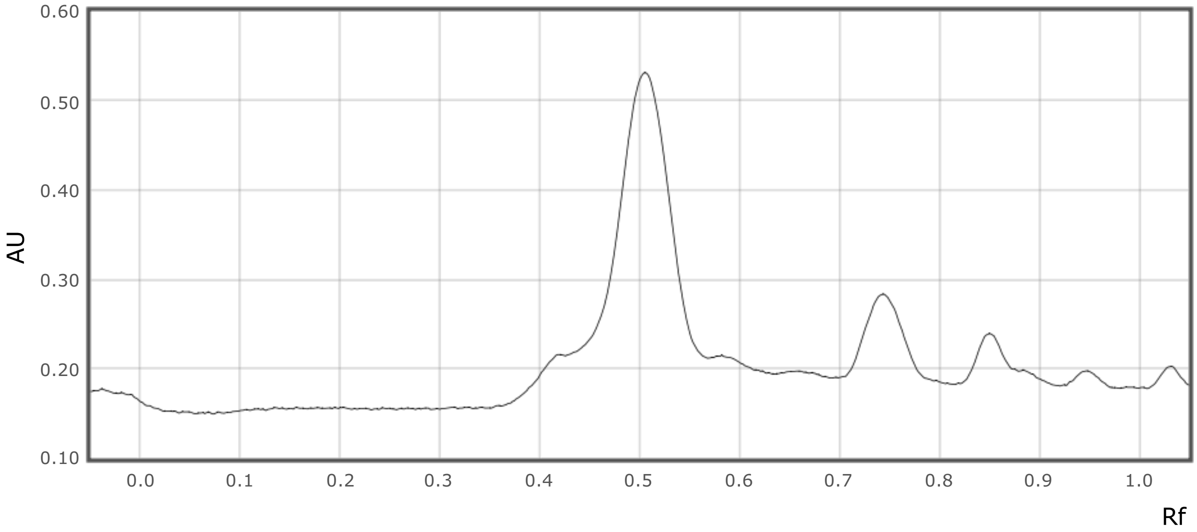

Track 9:

Type	Single λ

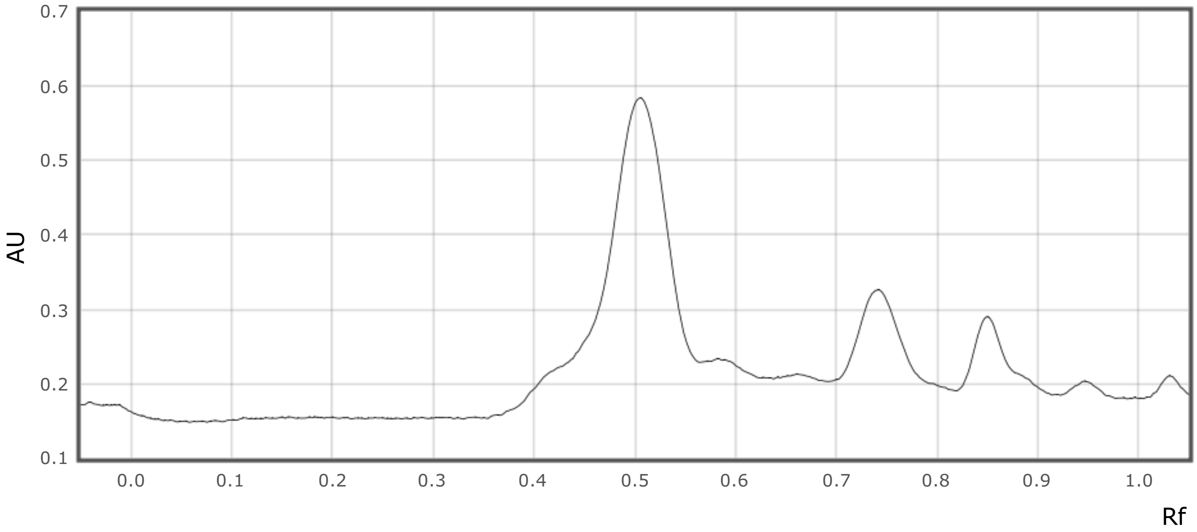

Track 10:

Type	Single λ

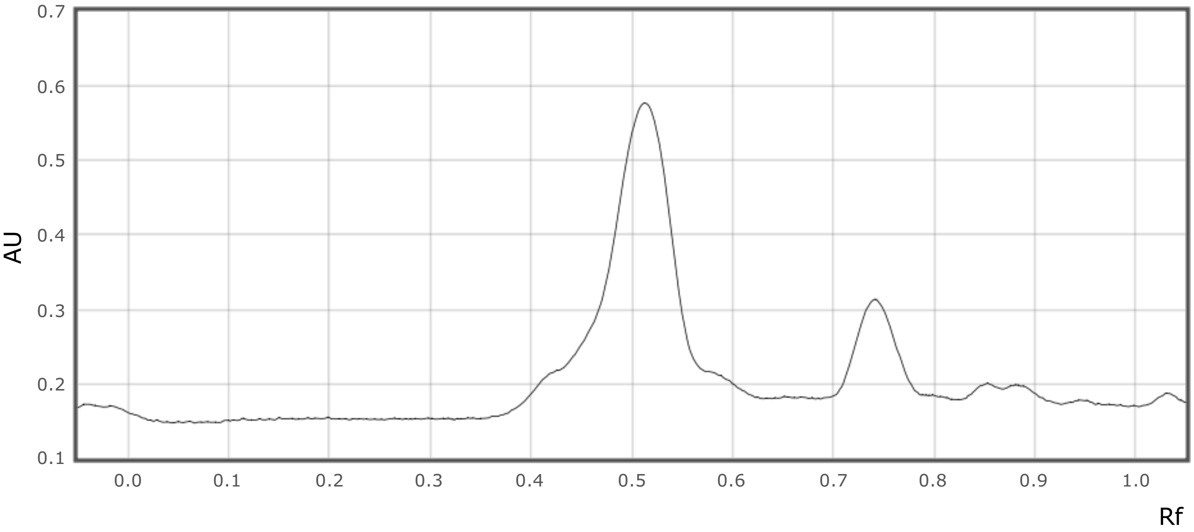

Track 11:	
Type	Single λ

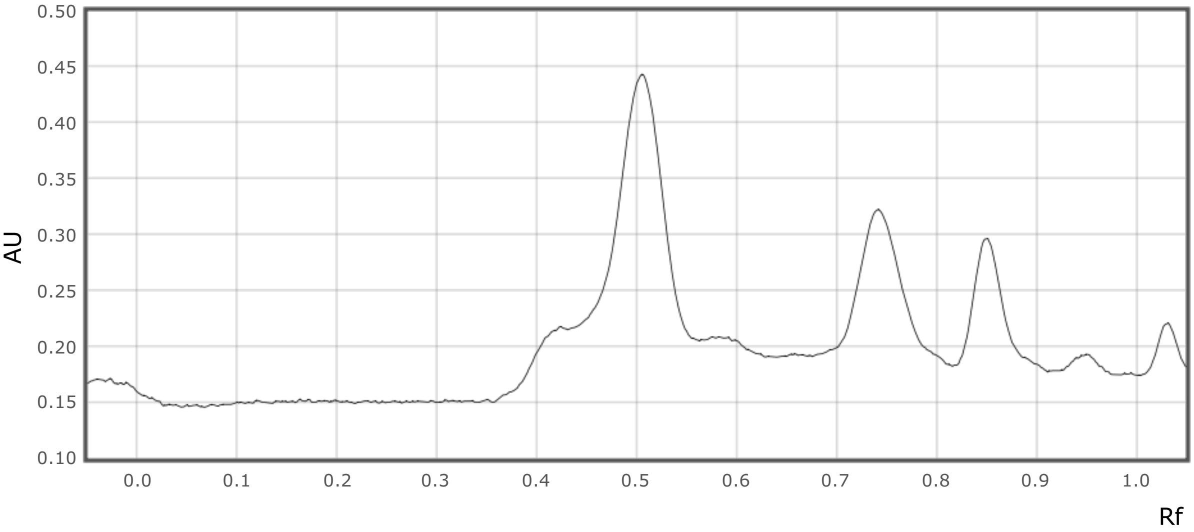

Track 12:	
Type	Single λ

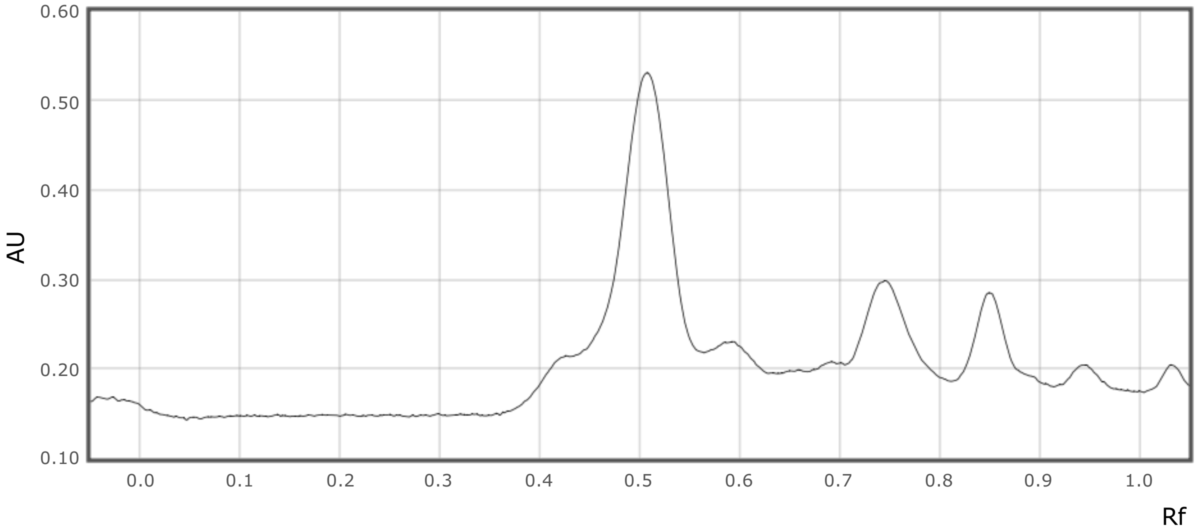

Track 13:	
Type	Single λ

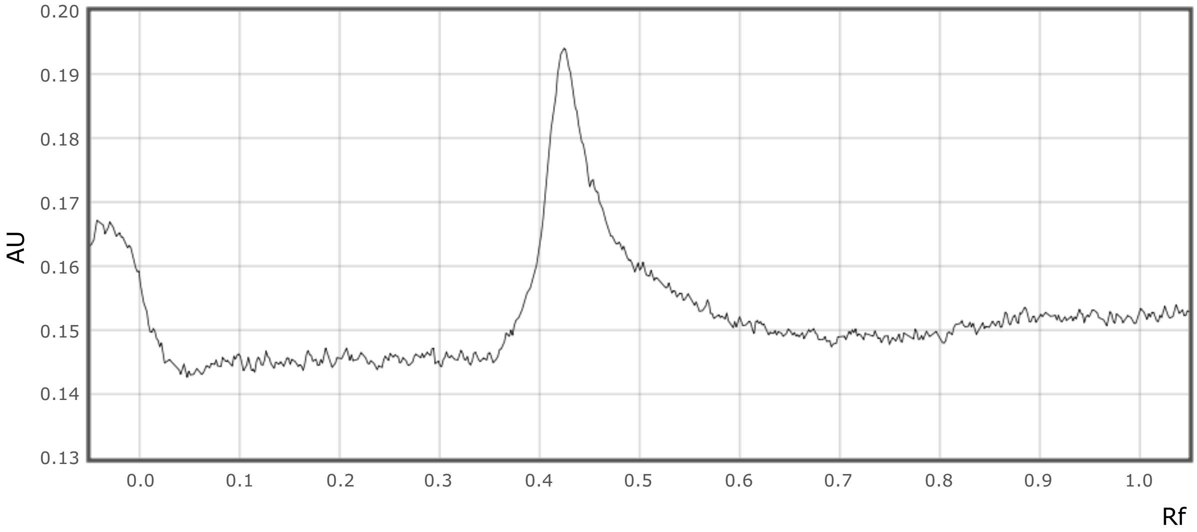

Track 14:	
Type	Single λ

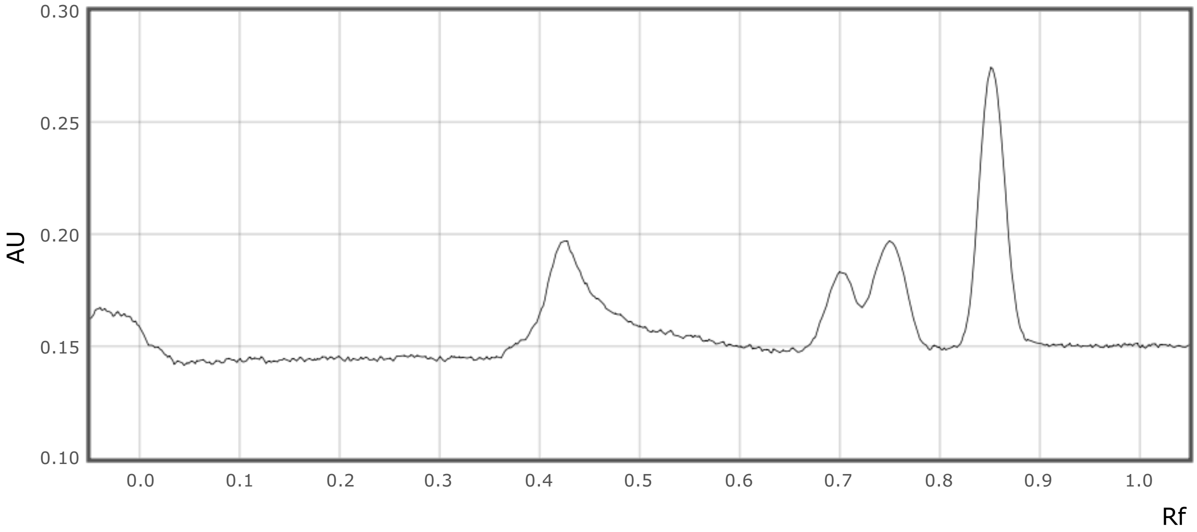

Track 15:	
Type	Single λ

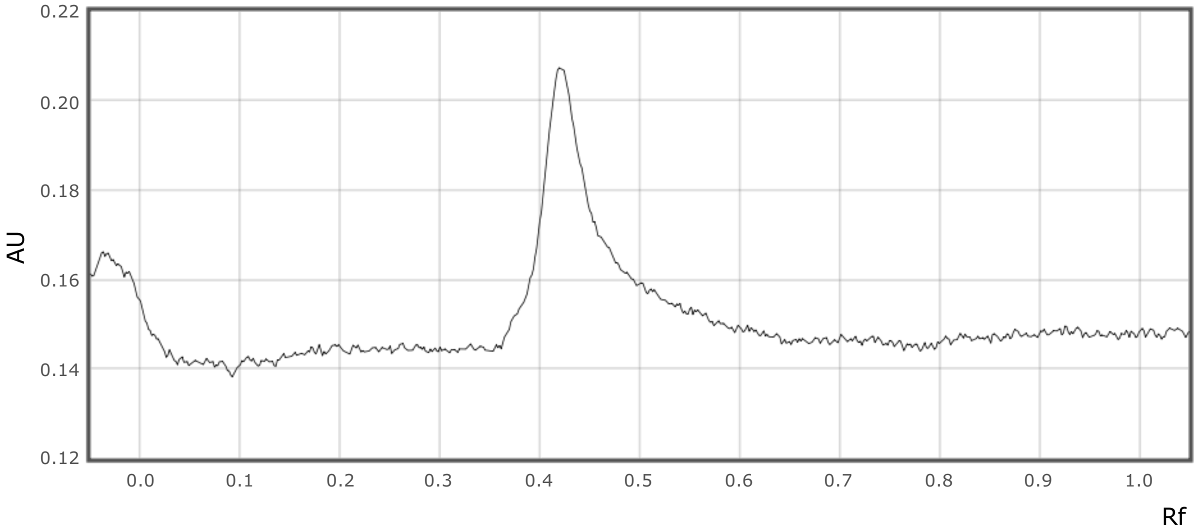

Derivatization 1 - dip:

Executed	11-Oct-2019 18:09:49 visionCATSuser

Take image derivatized plate 1a - Visualizer (S/N: 230515):

Executed	11-Oct-2019 18:12:55 visionCATSuser

RT White

Derivatized, RemTransVis

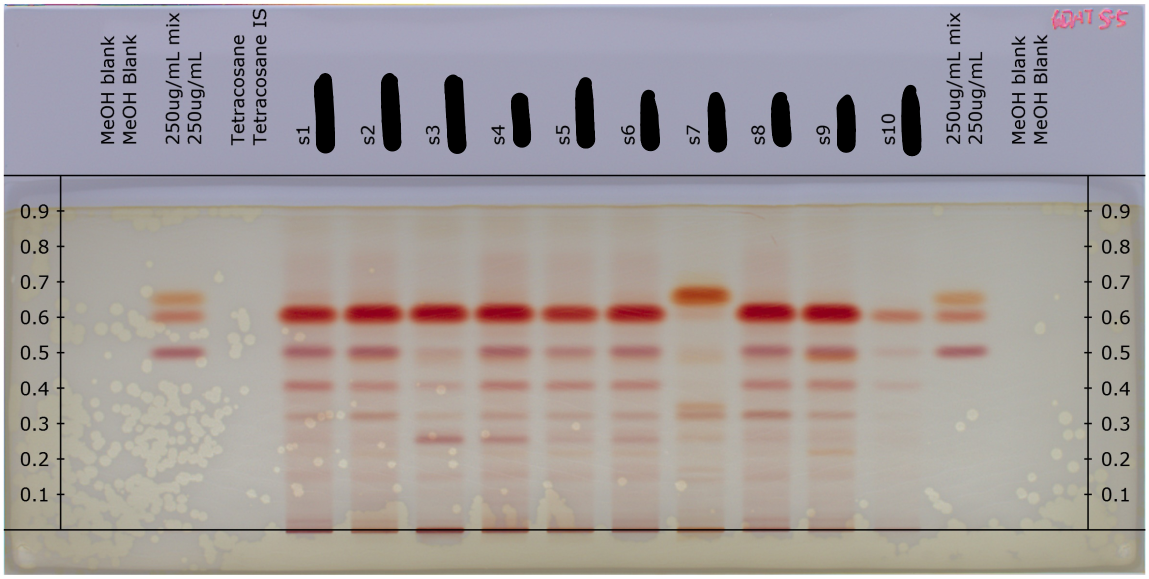

Exposure	0.047 s
Contrast	1
Normalized exposure	Disabled
Clarify	Disabled
White balance	1.15, 1.09, 0.83

R 366

Derivatized, Remission366

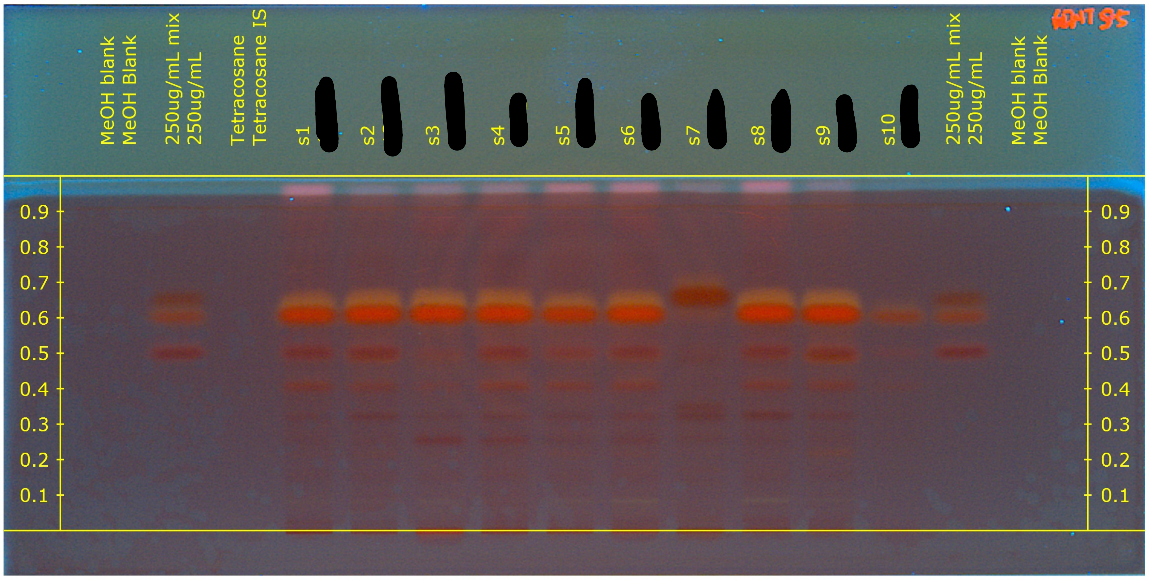

Exposure	9.999 s
Contrast	1
Normalized exposure	Disabled
Clarify	Disabled
White balance	1.00, 1.00, 1.00

Evaluation 1 :

Validated	false
Step	Take image derivatized plate 1a
Concentration unit type	Mass / volume
Notes	

Definition:

References:

250ug/mL mix

Substance Name	Concentration	Purity
9-THC	250.000 µg/ml	100.00 %
CBD	250.000 µg/ml	100.00 %
CBN	250.000 µg/ml	100.00 %

Samples:

Vial ID	Amount	Volume solution	Reference amount	Related to
MeOH blank		0.00 ml		
Tetracosane		0.00 ml		
s1		0.00 ml		
s2		0.00 ml		
s3		0.00 ml		
s4		0.00 ml		
s5		0.00 ml		
s6		0.00 ml		
s7		0.00 ml		
s8		0.00 ml		
s9		0.00 ml		
s10		0.00 ml		

Integration parameters:

Bounds	[0.000,1.000]
Smoothing	Savitzky–Golay of order 3 and window 7
Baseline correction	Lowest slope with noise 0.05
Profile subtraction	Profile subtraction from track 1
Peaks detection	Gauss (legacy) with sensitivity 0.1, separation 1 and threshold 0.1

Scan:

Wavelength	RT White

Track 1:

Type	Sample
Vial ID	MeOH blank
Description	MeOH Blank
Volume	2.0 µl

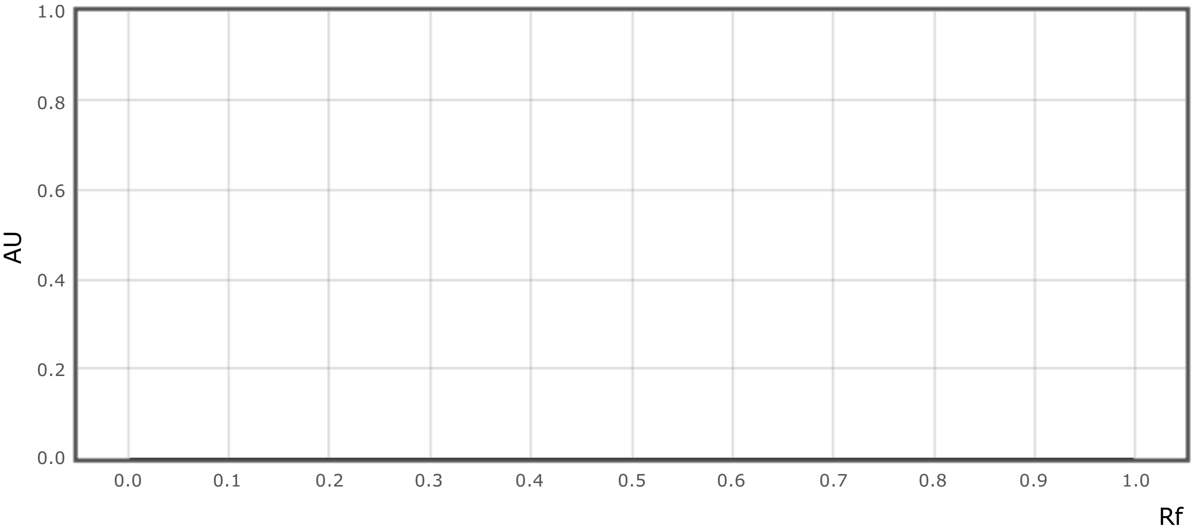

Peak #	Start		Max			End		Area		Manual peak	Substance Name
	Rf	H	Rf	H	%	Rf	H	A	%		

Track 2:

Type	Reference
Vial ID	250ug/mL mix
Description	250ug/mL
Volume	2.0 µl

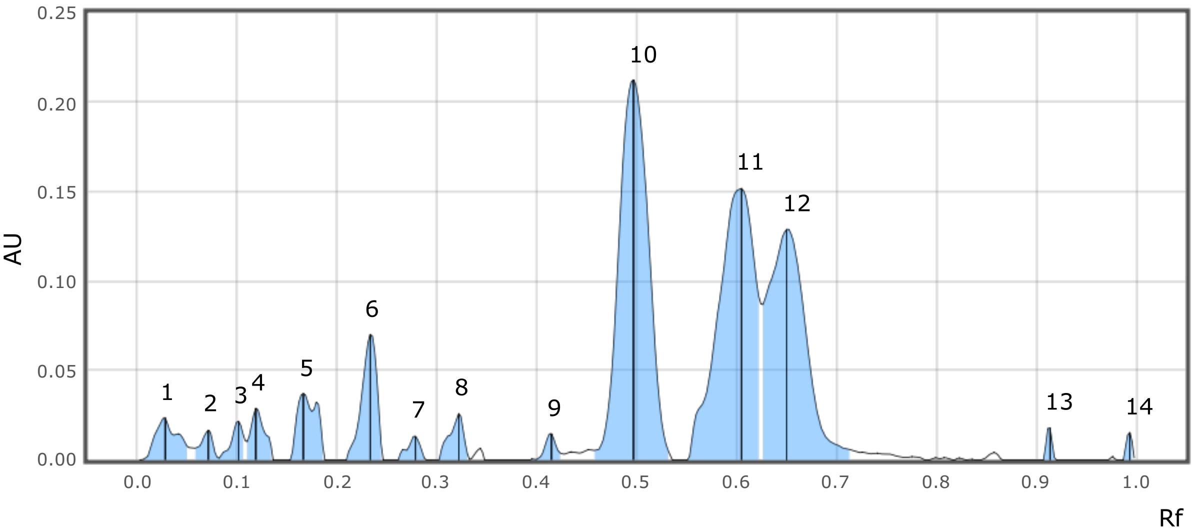

Peak #	Start Rf	Start H	Max Rf	Max H	Max %	End Rf	End H	Area A	Area %	Manual peak	Substance Name
1	0.004	0.0000	0.028	0.0234	3.01	0.052	0.0069	0.00060	2.49	No	
2	0.058	0.0066	0.071	0.0167	2.14	0.082	0.0009	0.00022	0.94	No	
3	0.082	0.0009	0.101	0.0217	2.79	0.108	0.0109	0.00029	1.22	No	
4	0.110	0.0102	0.119	0.0289	3.71	0.136	0.0000	0.00046	1.91	No	
5	0.153	0.0000	0.166	0.0370	4.76	0.188	0.0000	0.00086	3.57	No	
6	0.207	0.0000	0.233	0.0701	9.02	0.246	0.0000	0.00121	5.06	No	
7	0.261	0.0000	0.278	0.0132	1.70	0.289	0.0000	0.00019	0.81	No	
8	0.300	0.0000	0.322	0.0260	3.34	0.333	0.0002	0.00041	1.73	No	
9	0.393	0.0000	0.415	0.0146	1.88	0.425	0.0034	0.00019	0.78	No	
10	0.458	0.0053	0.497	0.2124	27.31	0.536	0.0000	0.00723	30.17	No	CBN
11	0.551	0.0000	0.605	0.1517	19.51	0.624	0.0872	0.00648	27.03	No	9-THC
12	0.626	0.0868	0.650	0.1288	16.56	0.724	0.0041	0.00560	23.38	No	CBD
13	0.907	0.0000	0.914	0.0177	2.28	0.920	0.0000	0.00012	0.49	No	
14	0.987	0.0000	0.994	0.0154	1.98	0.998	0.0010	0.00010	0.42	No	

Track 3:

Type	Sample
Vial ID	Tetracosane
Description	Tetracosane IS
Volume	2.0 µl

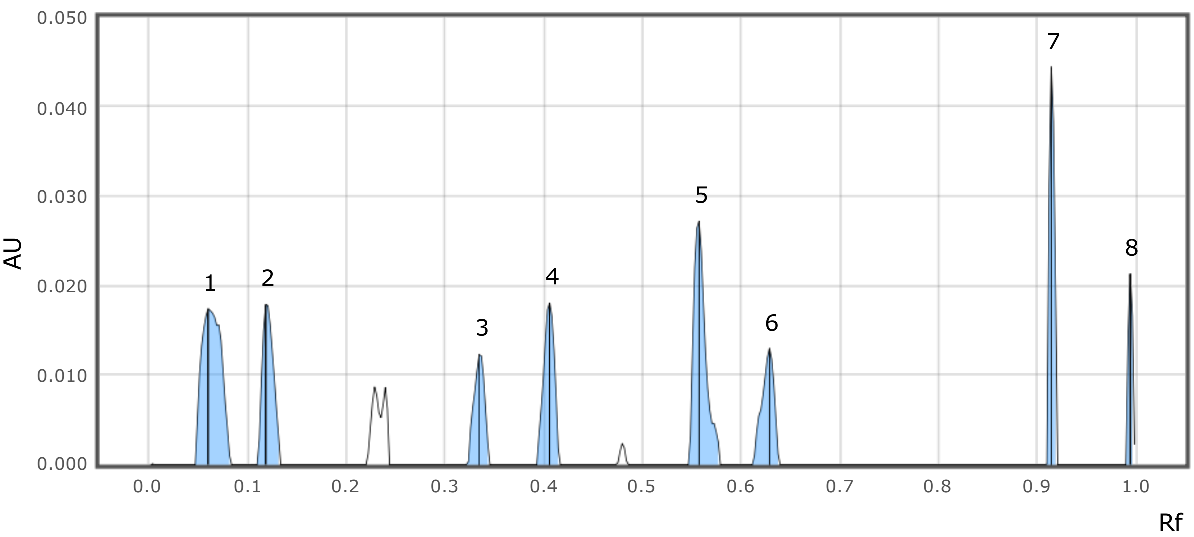

Peak #	Start Rf	Start H	Max Rf	Max H	Max %	End Rf	End H	Area A	Area %	Manual peak	Substance Name
1	0.047	0.0000	0.060	0.0174	10.15	0.084	0.0000	0.00042	20.94	No	
2	0.110	0.0000	0.119	0.0179	10.42	0.134	0.0000	0.00024	11.81	No	
3	0.322	0.0000	0.335	0.0123	7.17	0.345	0.0000	0.00016	7.72	No	
4	0.393	0.0000	0.406	0.0180	10.53	0.417	0.0000	0.00023	11.43	No	
5	0.546	0.0000	0.557	0.0271	15.84	0.579	0.0000	0.00038	18.91	No	
6	0.611	0.0000	0.629	0.0130	7.57	0.639	0.0000	0.00018	9.17	No	
7	0.907	0.0000	0.914	0.0444	25.90	0.920	0.0000	0.00029	14.30	No	
8	0.987	0.0000	0.994	0.0213	12.42	0.998	0.0022	0.00012	5.72	No	

Track 4:

Type	Sample
Vial ID	s1
Description	[redacted]
Volume	2.0 µl

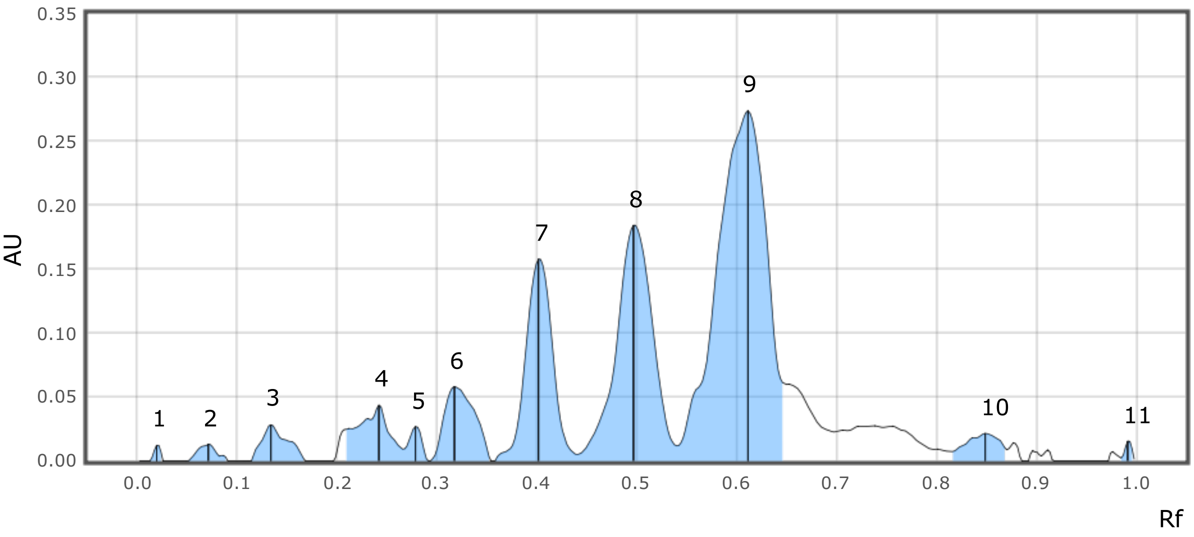

Peak #	Start		Max			End		Area		Manual peak	Substance Name
	Rf	H	Rf	H	%	Rf	H	A	%		
1	0.013	0.0000	0.019	0.0122	1.46	0.026	0.0000	0.00009	0.26	No	
2	0.049	0.0000	0.071	0.0130	1.55	0.090	0.0000	0.00028	0.81	No	
3	0.112	0.0000	0.134	0.0282	3.38	0.168	0.0000	0.00083	2.38	No	
4	0.209	0.0247	0.242	0.0436	5.22	0.266	0.0092	0.00152	4.38	No	
5	0.266	0.0092	0.278	0.0268	3.21	0.291	0.0000	0.00041	1.17	No	
6	0.291	0.0000	0.317	0.0581	6.96	0.354	0.0000	0.00205	5.90	No	
7	0.356	0.0000	0.402	0.1578	18.91	0.441	0.0050	0.00487	14.02	No	
8	0.441	0.0050	0.497	0.1843	22.08	0.540	0.0118	0.00779	22.43	No	
9	0.540	0.0118	0.611	0.2739	32.81	0.650	0.0598	0.01593	45.84	No	9-THC
10	0.817	0.0073	0.849	0.0215	2.57	0.871	0.0087	0.00085	2.45	No	
11	0.985	0.0022	0.992	0.0155	1.85	0.998	0.0012	0.00012	0.35	No	

Track 5:

Type	Sample
Vial ID	s2
Description	[redacted]
Volume	2.0 µl

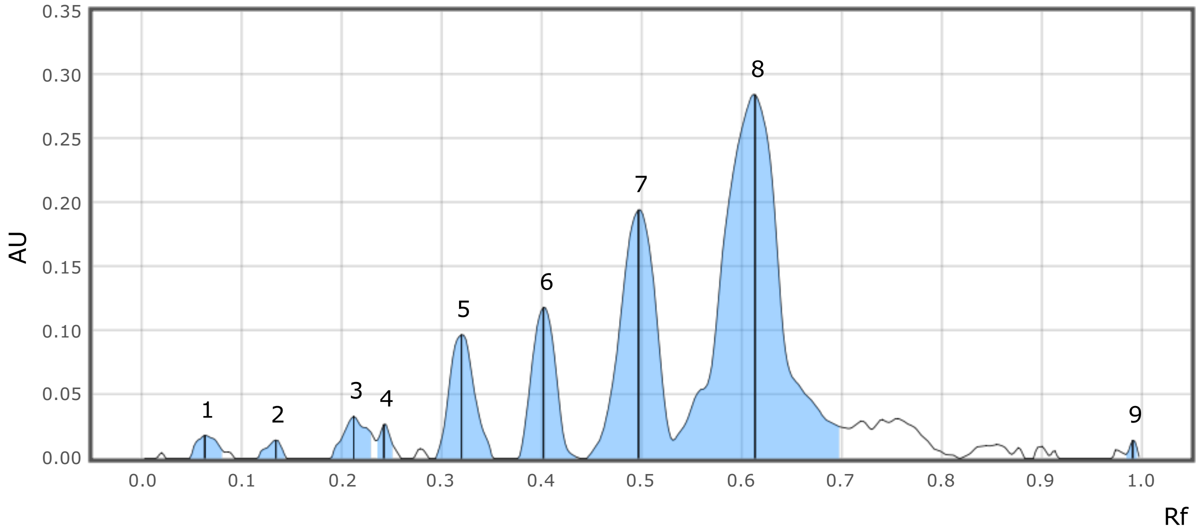

Peak #	Start Rf	Start H	Max Rf	Max H	Max %	End Rf	End H	Area A	Area %	Manual peak	Substance Name
1	0.047	0.0000	0.062	0.0180	2.25	0.082	0.0049	0.00043	1.21	No	
2	0.114	0.0000	0.134	0.0143	1.79	0.144	0.0000	0.00025	0.69	No	
3	0.188	0.0000	0.211	0.0329	4.11	0.233	0.0141	0.00092	2.59	No	
4	0.235	0.0141	0.242	0.0266	3.32	0.259	0.0000	0.00035	1.00	No	
5	0.294	0.0000	0.320	0.0967	12.09	0.352	0.0000	0.00280	7.85	No	
6	0.376	0.0000	0.402	0.1183	14.79	0.438	0.0000	0.00324	9.08	No	
7	0.445	0.0000	0.497	0.1944	24.30	0.531	0.0141	0.00795	22.31	No	
8	0.531	0.0141	0.613	0.2846	35.57	0.704	0.0238	0.01958	54.95	No	9-THC
9	0.985	0.0031	0.992	0.0142	1.77	0.998	0.0010	0.00012	0.32	No	

Track 6:

Type	Sample
Vial ID	s3
Description	[redacted]
Volume	2.0 µl

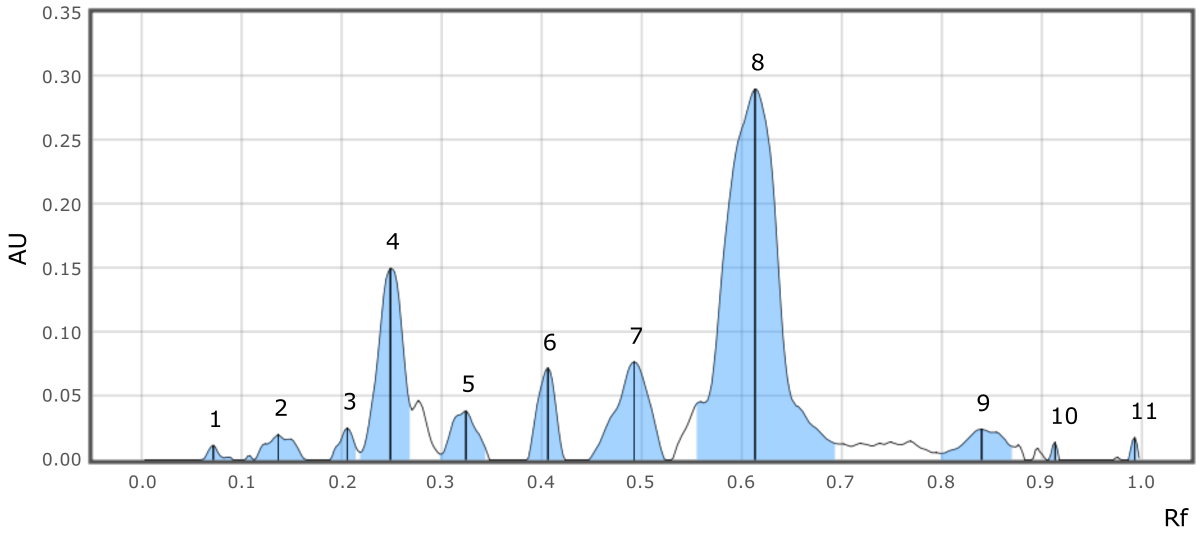

Peak #	Start Rf	Start H	Max Rf	Max H	Max %	End Rf	End H	Area A	Area %	Manual peak	Substance Name
1	0.054	0.0000	0.071	0.0116	1.56	0.093	0.0000	0.00014	0.47	No	
2	0.101	0.0000	0.136	0.0199	2.69	0.166	0.0000	0.00063	2.09	No	
3	0.188	0.0000	0.205	0.0250	3.38	0.216	0.0070	0.00041	1.34	No	
4	0.218	0.0058	0.248	0.1498	20.27	0.270	0.0390	0.00434	14.37	No	
5	0.298	0.0045	0.324	0.0385	5.22	0.348	0.0000	0.00113	3.73	No	
6	0.384	0.0000	0.406	0.0720	9.74	0.423	0.0000	0.00148	4.90	No	
7	0.445	0.0000	0.492	0.0767	10.38	0.525	0.0000	0.00293	9.69	No	
8	0.555	0.0441	0.613	0.2901	39.25	0.698	0.0124	0.01782	58.92	No	9-THC
9	0.799	0.0051	0.840	0.0240	3.25	0.875	0.0102	0.00117	3.86	No	
10	0.907	0.0000	0.914	0.0138	1.87	0.918	0.0000	0.00008	0.27	No	
11	0.987	0.0000	0.994	0.0176	2.39	0.998	0.0011	0.00011	0.38	No	

Track 7:

Type	Sample
Vial ID	s4
Description	[redacted]
Volume	2.0 µl

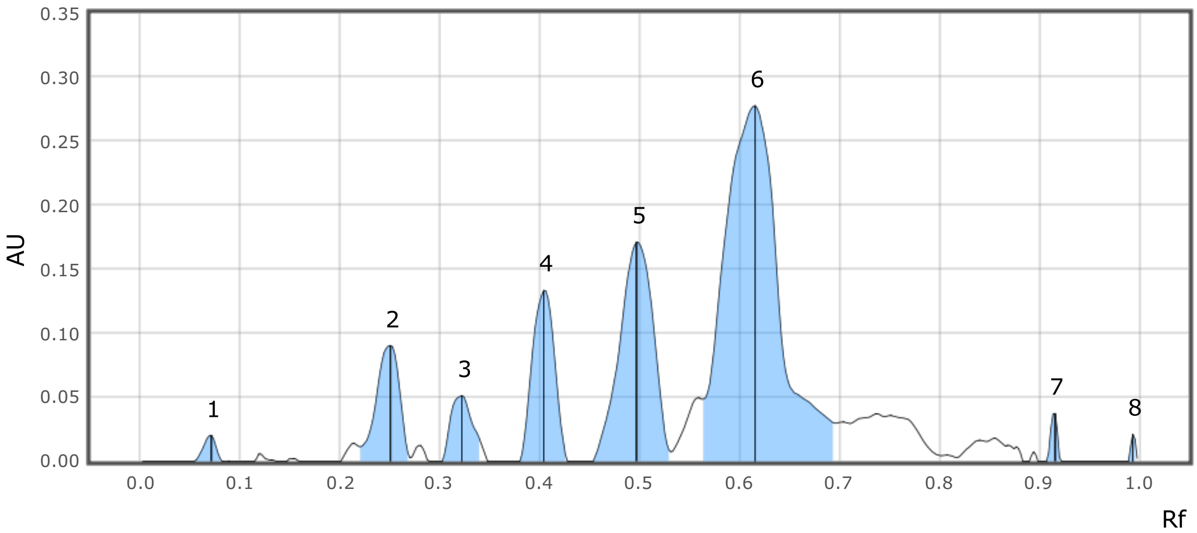

Peak #	Start Rf	Start H	Max Rf	Max H	Max %	End Rf	End H	Area A	Area %	Manual peak	Substance Name
1	0.054	0.0000	0.071	0.0203	2.53	0.082	0.0000	0.00028	0.88	No	
2	0.220	0.0113	0.250	0.0904	11.26	0.270	0.0026	0.00244	7.57	No	
3	0.302	0.0000	0.322	0.0513	6.40	0.348	0.0000	0.00133	4.14	No	
4	0.380	0.0000	0.404	0.1334	16.63	0.428	0.0000	0.00341	10.60	No	
5	0.451	0.0000	0.497	0.1710	21.31	0.531	0.0072	0.00667	20.72	No	
6	0.564	0.0485	0.616	0.2776	34.59	0.696	0.0297	0.01767	54.86	No	9-THC
7	0.907	0.0000	0.916	0.0374	4.66	0.922	0.0000	0.00029	0.89	No	
8	0.989	0.0000	0.994	0.0211	2.63	0.998	0.0020	0.00011	0.34	No	

Track 8:

Type	Sample
Vial ID	s5
Description	[redacted]
Volume	2.0 µl

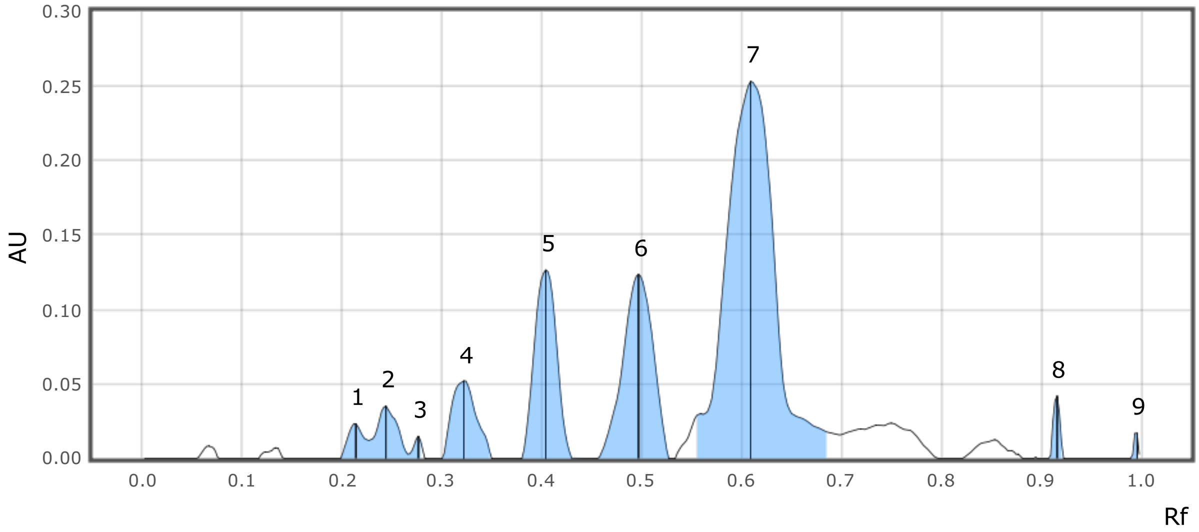

Peak #	Start		Max			End		Area		Manual peak	Substance Name
	Rf	H	Rf	H	%	Rf	H	A	%		
1	0.199	0.0000	0.214	0.0231	3.37	0.227	0.0119	0.00042	1.70	No	
2	0.227	0.0119	0.244	0.0351	5.11	0.266	0.0028	0.00083	3.31	No	
3	0.266	0.0028	0.276	0.0150	2.18	0.283	0.0000	0.00014	0.54	No	
4	0.300	0.0000	0.322	0.0519	7.56	0.350	0.0000	0.00149	5.94	No	
5	0.380	0.0000	0.404	0.1264	18.41	0.432	0.0000	0.00325	12.99	No	
6	0.456	0.0000	0.497	0.1234	17.97	0.529	0.0000	0.00430	17.18	No	
7	0.555	0.0288	0.609	0.2529	36.82	0.696	0.0160	0.01421	56.74	No	9-THC
8	0.907	0.0000	0.916	0.0419	6.11	0.922	0.0000	0.00032	1.28	No	
9	0.989	0.0000	0.996	0.0169	2.46	0.998	0.0026	0.00008	0.32	No	

Track 9:

Type	Sample
Vial ID	s6
Description	[redacted]
Volume	2.0 µl

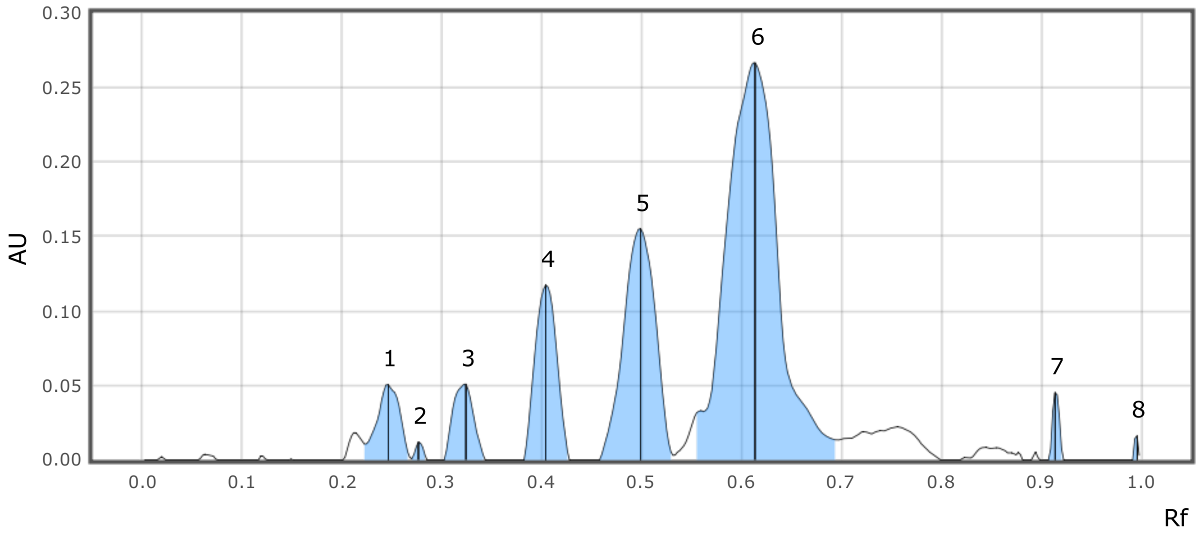

Peak #	Start Rf	Start H	Max Rf	Max H	Max %	End Rf	End H	Area A	Area %	Manual peak	Substance Name
1	0.222	0.0106	0.246	0.0505	7.08	0.268	0.0018	0.00133	4.80	No	
2	0.270	0.0008	0.276	0.0119	1.68	0.285	0.0000	0.00010	0.37	No	
3	0.302	0.0000	0.324	0.0509	7.13	0.343	0.0000	0.00120	4.36	No	
4	0.382	0.0000	0.404	0.1173	16.45	0.428	0.0000	0.00294	10.64	No	
5	0.458	0.0000	0.499	0.1550	21.74	0.531	0.0031	0.00572	20.71	No	
6	0.555	0.0317	0.613	0.2662	37.33	0.696	0.0133	0.01591	57.58	No	9-THC
7	0.907	0.0000	0.914	0.0452	6.34	0.922	0.0000	0.00036	1.29	No	
8	0.992	0.0000	0.996	0.0161	2.26	0.998	0.0029	0.00007	0.25	No	

Track 10:

Type	Sample
Vial ID	s7
Description	[redacted]
Volume	2.0 µl

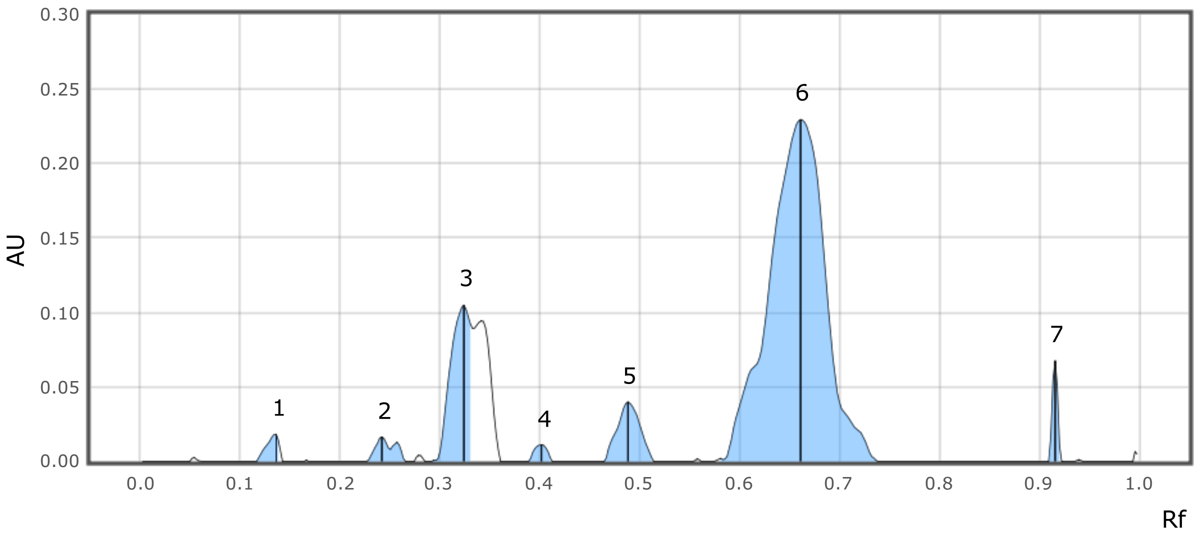

Peak #	Start Rf	Start H	Max Rf	Max H	Max %	End Rf	End H	Area A	Area %	Manual peak	Substance Name
1	0.116	0.0000	0.136	0.0183	3.76	0.142	0.0000	0.00027	1.36	No	
2	0.227	0.0000	0.242	0.0163	3.35	0.266	0.0000	0.00036	1.81	No	
3	0.291	0.0000	0.324	0.1048	21.51	0.333	0.0891	0.00245	12.47	No	
4	0.389	0.0000	0.402	0.0111	2.28	0.412	0.0000	0.00017	0.86	No	
5	0.464	0.0000	0.488	0.0399	8.19	0.514	0.0000	0.00106	5.39	No	
6	0.577	0.0009	0.661	0.2292	47.06	0.739	0.0000	0.01488	75.91	No	CBD
7	0.910	0.0000	0.916	0.0675	13.85	0.922	0.0000	0.00043	2.19	No	

Track 11:

Type	Sample
Vial ID	s8
Description	[redacted]
Volume	2.0 µl

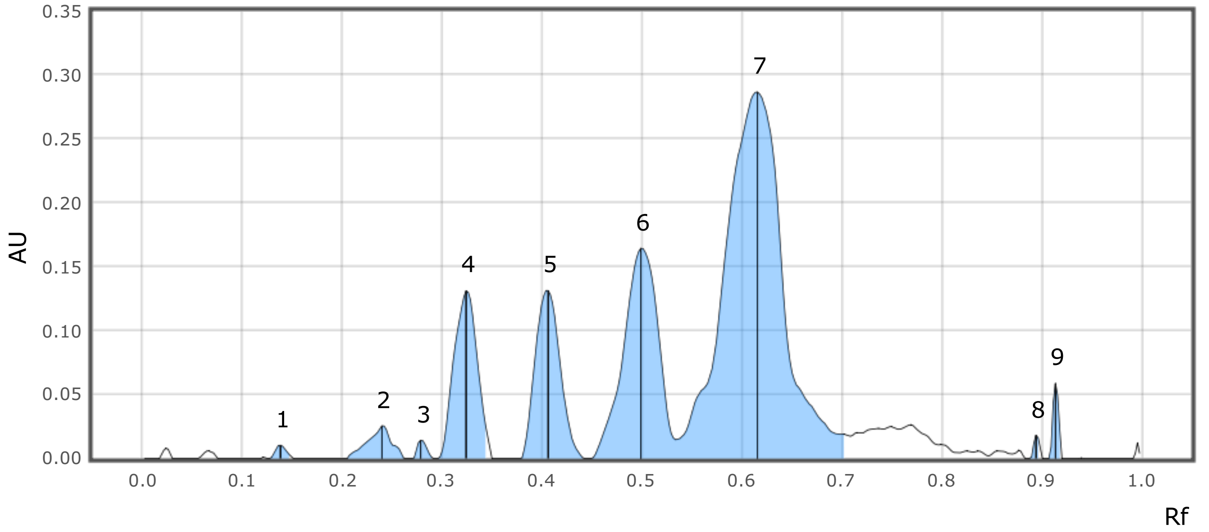

Peak #	Start		Max			End		Area		Manual peak	Substance Name
	Rf	H	Rf	H	%	Rf	H	A	%		
1	0.127	0.0000	0.138	0.0103	1.22	0.151	0.0000	0.00013	0.36	No	
2	0.205	0.0000	0.240	0.0254	3.02	0.263	0.0000	0.00069	1.97	No	
3	0.270	0.0000	0.278	0.0142	1.69	0.291	0.0000	0.00015	0.43	No	
4	0.296	0.0000	0.324	0.1310	15.60	0.350	0.0000	0.00355	10.16	No	
5	0.378	0.0000	0.406	0.1313	15.63	0.443	0.0000	0.00378	10.82	No	
6	0.449	0.0000	0.499	0.1644	19.57	0.533	0.0146	0.00661	18.91	No	
7	0.533	0.0146	0.616	0.2865	34.12	0.706	0.0177	0.01953	55.88	No	9-THC
8	0.888	0.0000	0.894	0.0181	2.15	0.901	0.0000	0.00012	0.34	No	
9	0.907	0.0000	0.914	0.0587	6.99	0.920	0.0000	0.00039	1.13	No	

Track 12:

Type	Sample
Vial ID	s9
Description	[redacted]
Volume	2.0 µl

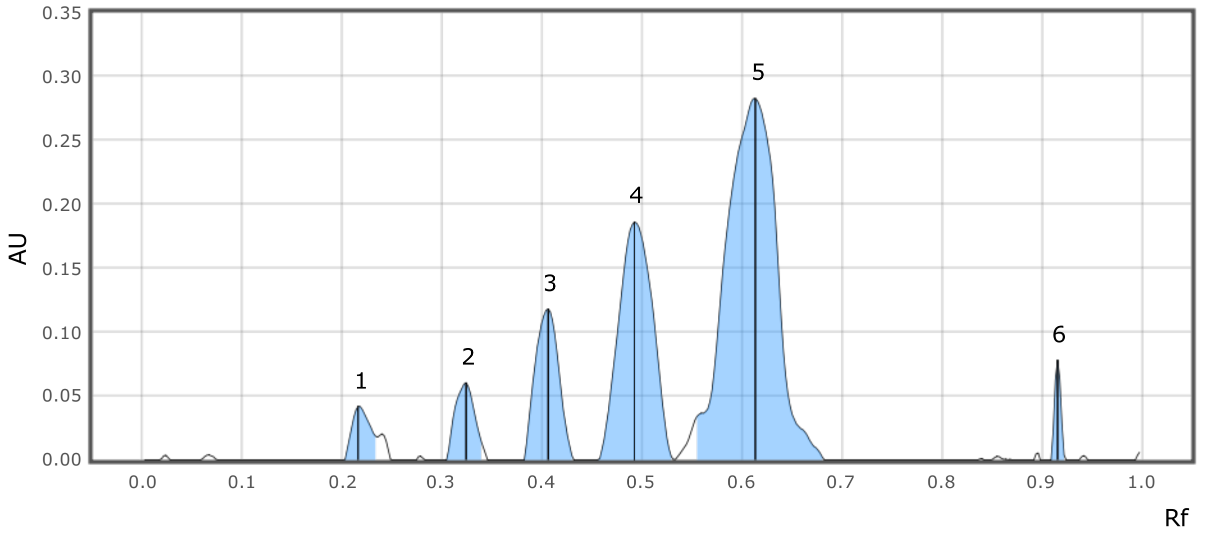

Peak #	Start		Max			End		Area		Manual peak	Substance Name
	Rf	H	Rf	H	%	Rf	H	A	%		
1	0.201	0.0000	0.216	0.0419	5.45	0.235	0.0178	0.00089	3.00	No	
2	0.304	0.0000	0.324	0.0605	7.88	0.345	0.0000	0.00139	4.67	No	
3	0.380	0.0000	0.406	0.1180	15.38	0.432	0.0000	0.00323	10.82	No	
4	0.456	0.0000	0.492	0.1862	24.26	0.531	0.0004	0.00731	24.50	No	
5	0.555	0.0341	0.613	0.2827	36.84	0.683	0.0000	0.01646	55.14	No	9-THC
6	0.910	0.0000	0.916	0.0782	10.19	0.925	0.0000	0.00056	1.87	No	

Track 13:

Type	Sample
Vial ID	s10
Description	[redacted]
Volume	2.0 µl

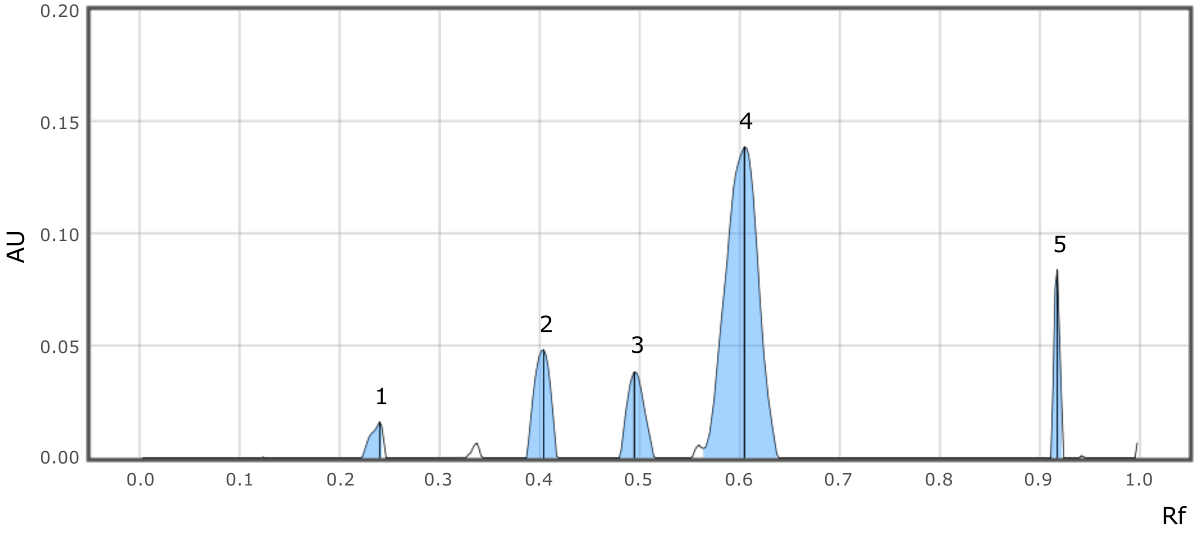

Peak #	Start		Max			End		Area		Manual peak	Substance Name
	Rf	H	Rf	H	%	Rf	H	A	%		
1	0.220	0.0000	0.240	0.0161	4.94	0.246	0.0000	0.00023	2.92	No	
2	0.387	0.0000	0.404	0.0483	14.84	0.419	0.0000	0.00092	11.80	No	
3	0.479	0.0000	0.495	0.0384	11.79	0.516	0.0000	0.00076	9.81	No	
4	0.564	0.0042	0.605	0.1388	42.65	0.639	0.0000	0.00528	67.93	No	9-THC
5	0.912	0.0000	0.918	0.0839	25.78	0.925	0.0000	0.00059	7.54	No	

Track 14:

Type	Reference
Vial ID	250ug/mL mix
Description	250ug/mL
Volume	2.0 µl

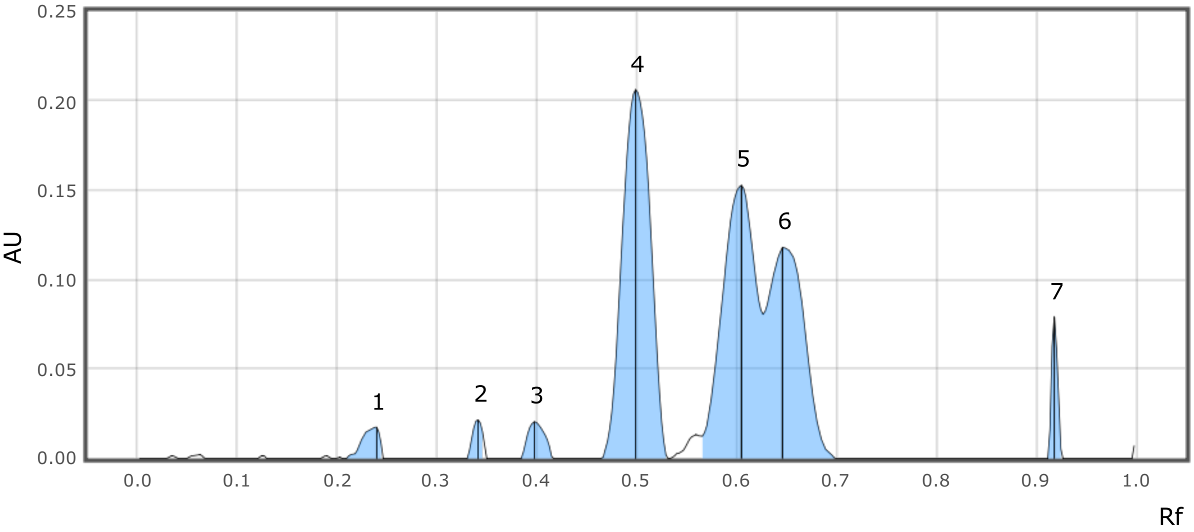

Peak #	Start		Max			End		Area		Manual peak	Substance Name
	Rf	H	Rf	H	%	Rf	H	A	%		
1	0.209	0.0000	0.240	0.0175	2.84	0.246	0.0000	0.00035	1.85	No	
2	0.330	0.0000	0.341	0.0216	3.51	0.350	0.0000	0.00025	1.32	No	
3	0.384	0.0000	0.397	0.0204	3.32	0.417	0.0000	0.00040	2.10	No	
4	0.464	0.0000	0.499	0.2061	33.52	0.531	0.0000	0.00671	35.11	No	CBN
5	0.566	0.0124	0.605	0.1526	24.81	0.626	0.0805	0.00587	30.69	No	9-THC
6	0.626	0.0805	0.646	0.1177	19.14	0.700	0.0000	0.00497	25.99	No	CBD
7	0.912	0.0000	0.918	0.0791	12.86	0.927	0.0000	0.00056	2.93	No	

Track 15:

Type	Sample
Vial ID	MeOH blank
Description	MeOH Blank
Volume	2.0 µl

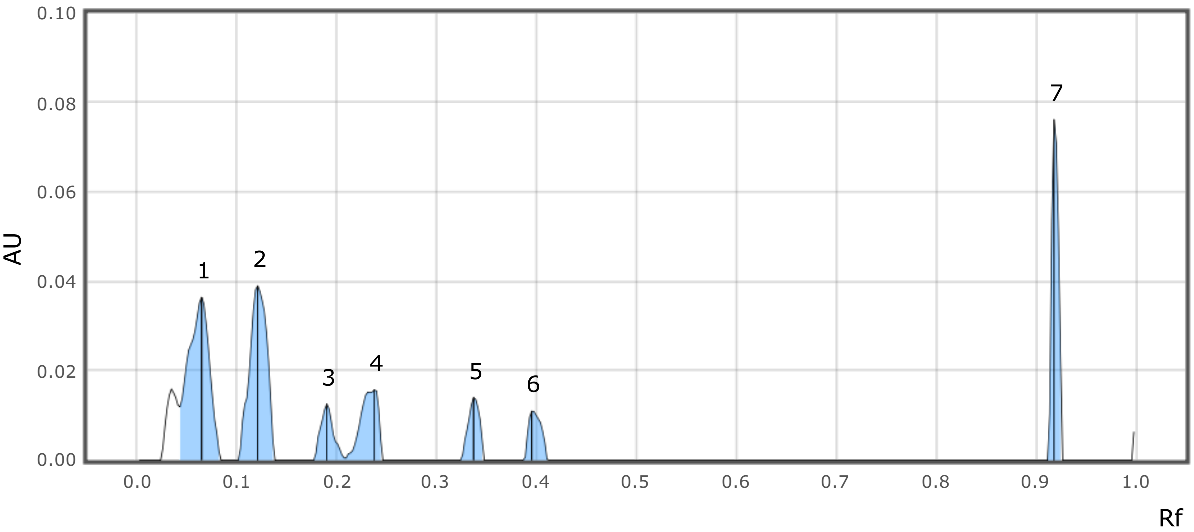

Peak #	Start		Max			End		Area		Manual peak	Substance Name
	Rf	H	Rf	H	%	Rf	H	A	%		
1	0.043	0.0119	0.065	0.0364	17.79	0.084	0.0000	0.00089	28.06	No	
2	0.101	0.0000	0.121	0.0390	19.04	0.138	0.0000	0.00080	25.18	No	
3	0.177	0.0000	0.190	0.0125	6.13	0.209	0.0005	0.00018	5.79	No	
4	0.209	0.0005	0.237	0.0157	7.65	0.246	0.0000	0.00033	10.38	No	
5	0.324	0.0000	0.337	0.0140	6.82	0.348	0.0000	0.00019	5.94	No	
6	0.387	0.0000	0.395	0.0110	5.35	0.412	0.0000	0.00016	5.20	No	
7	0.912	0.0000	0.918	0.0761	37.21	0.927	0.0000	0.00062	19.47	No	

Calibration results:

Height calibration for substance 9-THC @ RT White:

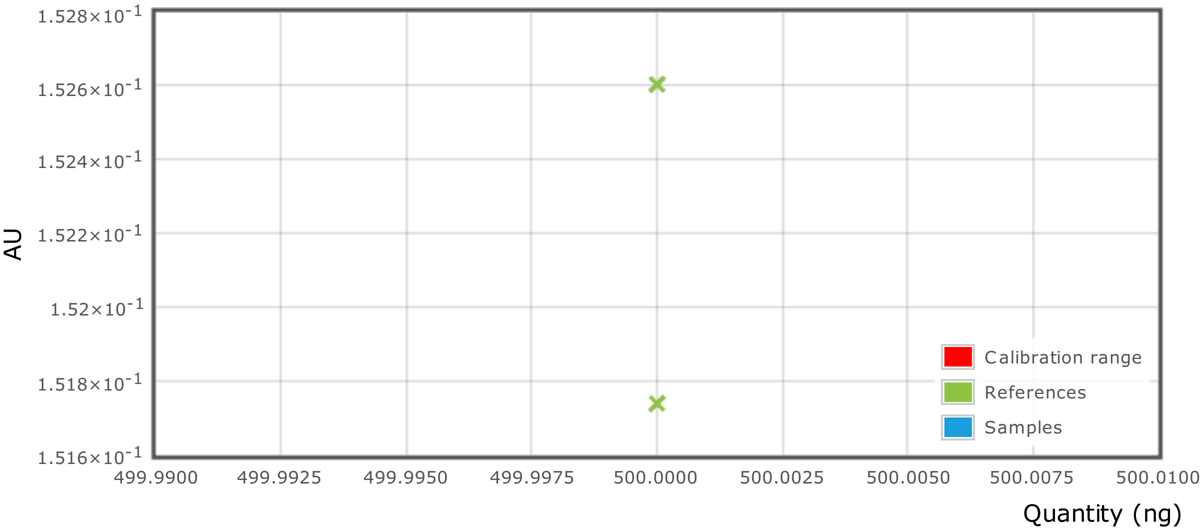

Regression mode	Linear-2
Range deviation	5.00 %
Related substances	Default
Number of references	2
Calibration function	$y=0x$
Coefficient of variation	CV 0.00 %
Correlation coefficient	n/a
⚠	Unable to compute the results for this substance because there wasn't eno ugh groups of references replicas (at least 1 for Linear-1, 2 for Linear2 and Mime-1 and 3 for Polynomial and MiMe-2)

Height calibration for substance CBD @ RT White:

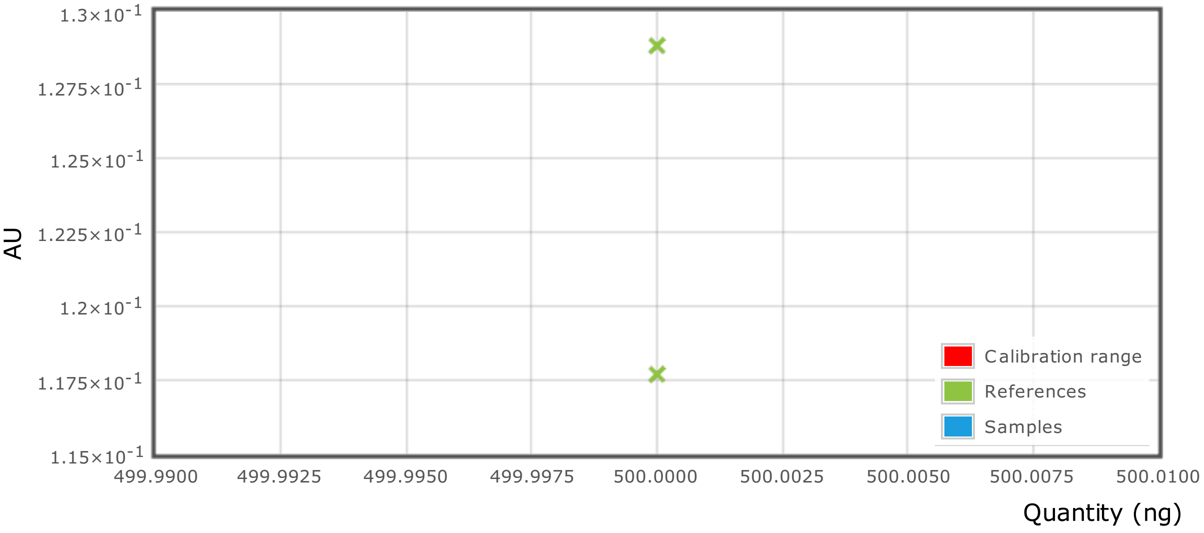

Regression mode	Linear-2
Range deviation	5.00 %
Related substances	Default
Number of references	2
Calibration function	$y=0x$
Coefficient of variation	CV 0.00 %
Correlation coefficient	n/a
⚠	Unable to compute the results for this substance because there wasn't eno ugh groups of references replicas (at least 1 for Linear-1, 2 for Linear2 and Mime-1 and 3 for Polynomial and MiMe-2)

Height calibration for substance CBN @ RT White:

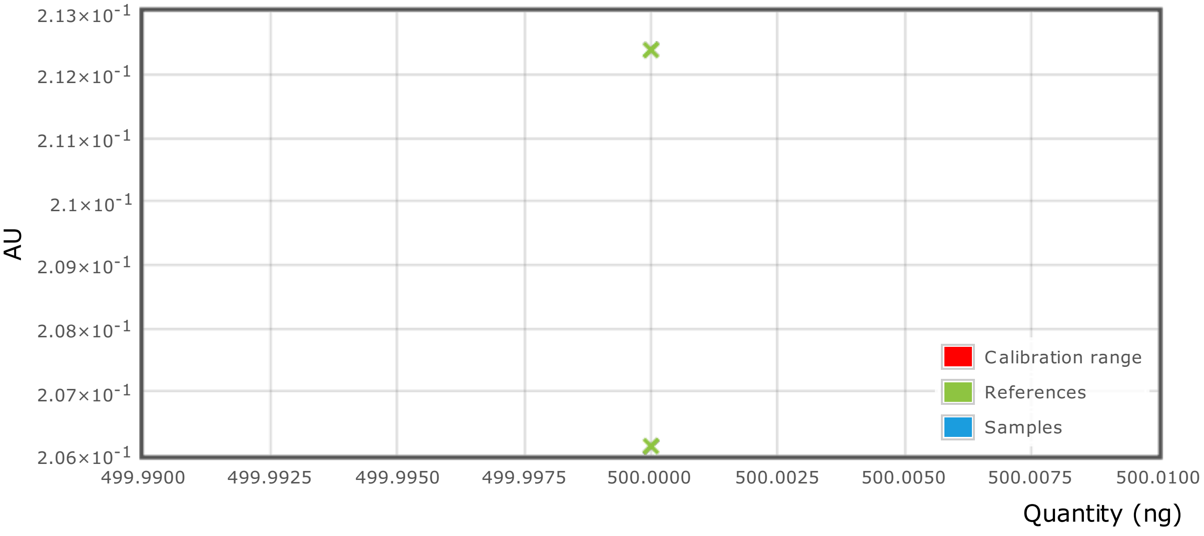

Regression mode	Linear-2
Range deviation	5.00 %
Related substances	Default
Number of references	2
Calibration function	$y=0x$
Coefficient of variation	CV 0.00 %
Correlation coefficient	n/a
⚠	Unable to compute the results for this substance because there wasn't enough groups of references replicas (at least 1 for Linear-1, 2 for Linear2 and Mime-1 and 3 for Polynomial and MiMe-2)

Results:

Substance having no available results		
⚠	CBD	Unable to compute the results for this substance because there wasn't enough groups of references replicas (at least 1 for Linear-1, 2 for Linear2 and Mime-1 and 3 for Polynomial and MiMe-2)
⚠	9-THC	Unable to compute the results for this substance because there wasn't enough groups of references replicas (at least 1 for Linear-1, 2 for Linear2 and Mime-1 and 3 for Polynomial and MiMe-2)
⚠	CBN	There wasn't any sample application available in the assignments for this substance. Please check that the peaks were correctly detected and assigned for this substance.

A track marked with ⚠ means: this result is outside the regression range given by the reference assignments, but is included in the results because it is in the allowed range deviation.

Analyst:	Reviewer:
