# Supplementary material for: High Performance Thin-Layer Chromatography (HPTLC) data of Cannabinoids in ten mobile phase systems
Source: Data Brief. 2020 Jun 30;31:105955. doi: 10.1016/j.dib.2020.105955 (PMC7352075; doi:10.1016/j.dib.2020.105955)
Supplement: Supplementary file 1 [file mmc1.zip › S4-Case sample reports/6DaT-sample run-6.pdf]

# Analysis: 6DaT-sample run-6

**Path: Home/YL Research**

**Based on method: Samples (no cal)**

| | | |
|---|---|---|
| Created | 12-Oct-2019 17:40:19 | visionCATSuser |
| Modified | 12-Oct-2019 19:34:57 | visionCATSuser |
| Last HPTLC log | 12-Oct-2019 19:34:57 | Analysis modified |
| Explorer notes | | |

| Track | Vial ID | Description | Volume | Position | Type |
|---|---|---|---|---|---|
| 1 | MeOH blank | MeOH Blank | 2.0 µl | A1 | Sample |
| 2 | 250ug/mL mix | 250ug/mL | 2.0 µl | A2 | Reference |
| 3 | Tetracosane | Tetracosane IS | 2.0 µl | A3 | Sample |
| 4 | s1 | [redacted] | 2.0 µl | B1 | Sample |
| 5 | s2 | [redacted] | 2.0 µl | B2 | Sample |
| 6 | s3 | [redacted] | 2.0 µl | B3 | Sample |
| 7 | s4 | [redacted] | 2.0 µl | B4 | Sample |
| 8 | s5 | [redacted] | 2.0 µl | B5 | Sample |
| 9 | s6 | [redacted] | 2.0 µl | B6 | Sample |
| 10 | s7 | [redacted] | 2.0 µl | B7 | Sample |
| 11 | s8 | [redacted] | 2.0 µl | B8 | Sample |
| 12 | s9 | [redacted] | 2.0 µl | B9 | Sample |
| 13 | s10 | [redacted] | 2.0 µl | B10 | Sample |
| 14 | 250ug/mL mix | 250ug/mL | 2.0 µl | A2 | Reference |
| 15 | MeOH blank | MeOH Blank | 2.0 µl | A1 | Sample |
| Sequence table notes | | | | | |

A track marked with ⚠ means: the application type is overridden in some evaluation(s).

## System setup:

| | |
|---|---|
| Software | Server User-PC, version 2.5.18072.1 |
| ATS4 | S/N:080713 |
| Chamber | N/A |
| Derivatization dip | N/A |
| Scanner3 | S/N:031025 |
| Visualizer | S/N:230515 |

# Chromatography

## Plate layout:

| | |
|---|---|
| Stationary phase | Merck, HPTLC plates silica gel 60 F 254 |
| Plate format | 200.0 x 100.0 mm |
| Application type | Band |
| Application | Position Y: 8.0 mm, length: 8.0 mm, width: 0.0 mm |
| Track | First position X: 20.0 mm, distance: 11.4 mm |
| Solvent front position | 70.0 mm |
| Notes | |

## Take image clean plate 1a - Visualizer (S/N: 230515):

| | |
|---|---|
| Quality | Enhanced |
| RT White | auto capture, Auto, level 85 %, Band |
| R 254 | auto capture, Auto, level 85 %, Band |
| Instrument diagnostics | Valid diagnostics |
| Documentation step label | |
| Notes | |

## Application 1 - ATS 4 (S/N: 080713):

| | |
|---|---|
| Spray gas | NI |
| Sample solvent type | Methanol |
| Filling speed | 15 µl/s |
| Predosage volume | 200 nl |
| Retraction volume | 200 nl |
| Dosage speed | 150 nl/s |
| Filling quality | User |
| Rinsing cycles / vacuum | 2 / 4 s |
| Filling cycles / vacuum | 1 / 4 s |
| Rinsing solvent name | Methanol |
| Nozzle temperature | Unheated |
| Rack in use | Standard |
| Instrument diagnostics | Valid diagnostics |
| Notes | |

## Development 1 - Chamber:

| | |
|---|---|
| Tank | TTC 20x10 |
| Mobile phase | 6% diethylamine in toluene |
| Saturation time | 20 min |
| Use saturation pad | true |
| Use smartALERT | false |
| Volume front through | 10 ml |
| Volume rear through | 25 ml |
| Drying time | 5 min |
| Drying temperature | Room temperature |
| Notes | |

## Take image developed plate 1a - Visualizer (S/N: 230515):

| | |
|---|---|
| Quality | Enhanced |
| RT White | auto capture, Auto, level 85 %, Band |
| R 254 | auto capture, Auto, level 85 %, Band |
| R 366 | auto capture, Auto, level 85 %, Band |
| Instrument diagnostics | Valid diagnostics |
| Documentation step label | |
| Notes | |

## Scan developed plate 1b - Scanner 3 (S/N: 031025):

| | |
|---|---|
| Scanner type | Single λ |
| Optimization for | Resolution |
| Measurement mode | Absorption |
| Filter | n/a |
| Detector mode | Automatic |
| Scanning speed | 20 mm/s |
| Data resolution | 100 µm/step |
| Slit | 5 x 0.2 mm, micro |
| Partial scan | No |
| Lamp | Deuterium & Tungsten |
| Wavelength(s) | 254 nm |
| Instrument diagnostics | Valid diagnostics |
| Documentation step label | |
| Notes | |

## Derivatization 1 - dip:

| | |
|---|---|
| Reagent name | |
| Dipping speed | 5 |
| Dipping time | 0 s |
| Reagent preparation | |
| Heating | 100 °C for 3 min, heated after |
| Notes | |

## Take image derivatized plate 1a - Visualizer (S/N: 230515):

| | |
|---|---|
| Quality | Enhanced |
| RT White | auto capture, Auto, level 85 %, Band |
| R 366 | auto capture, Auto, level 85 %, Band |
| Instrument diagnostics | Valid diagnostics |
| Documentation step label | |
| Notes | |

# System suitability tests:

## SST settings:

| | |
|---|---|
| SST tracks | |

# Data acquisition

## Application 1 - ATS 4 (S/N: 080713):

| | |
|---|---|
| Executed | 12-Oct-2019 17:44:41 visionCATSuser |

## Development 1 - Chamber:

| | |
|---|---|
| Executed | 12-Oct-2019 18:21:01 visionCATSuser |

## Take image developed plate 1a - Visualizer (S/N: 230515):

| | |
|---|---|
| Executed | 12-Oct-2019 19:11:06 visionCATSuser |

RT White

Developed, RemTransVis

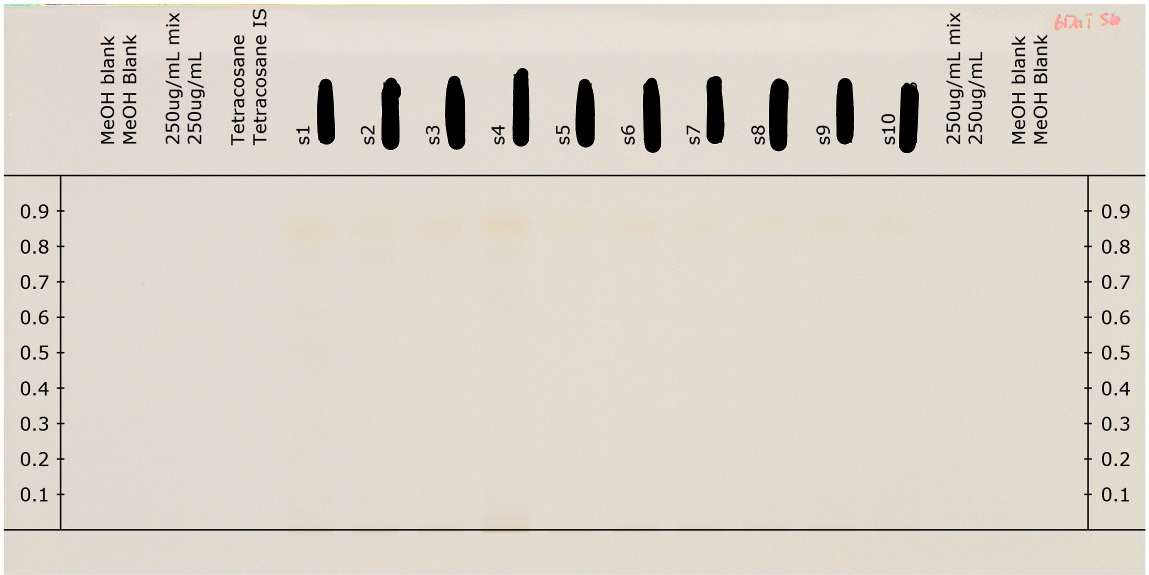


| | |
|---|---|
| Exposure | 0.086 s |
| Contrast | 1 |
| Normalized exposure | Disabled |
| Clarify | Disabled |
| White balance | 1.00, 1.00, 1.00 |

R 254

Developed, Remission254

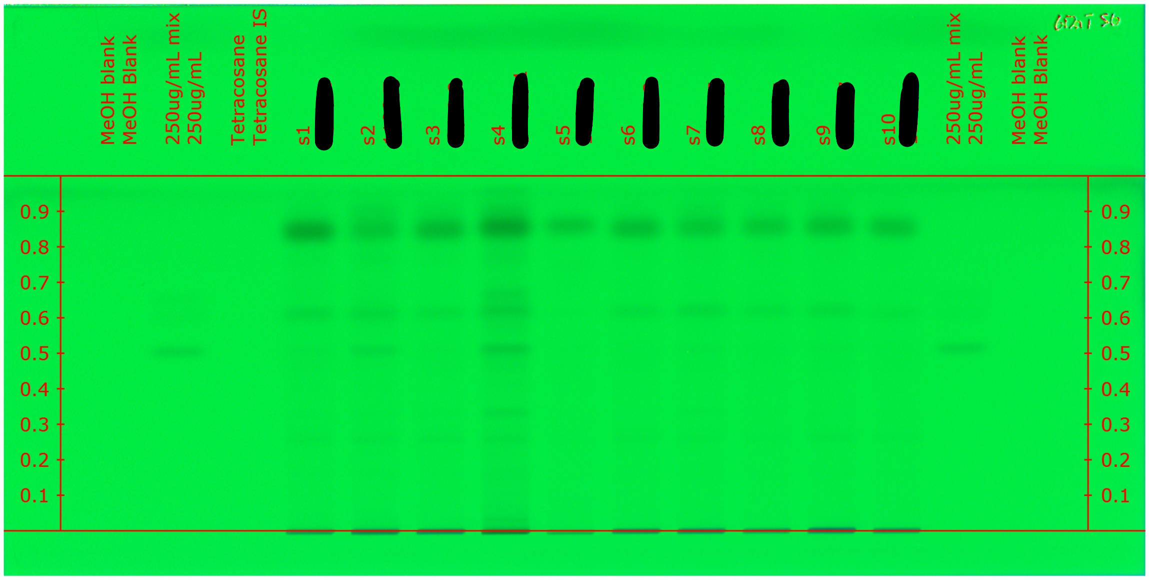


| | |
|---|---|
| Exposure | 0.275 s |
| Contrast | 1 |
| Normalized exposure | Disabled |
| Clarify | Disabled |
| White balance | 1.00, 1.00, 1.00 |

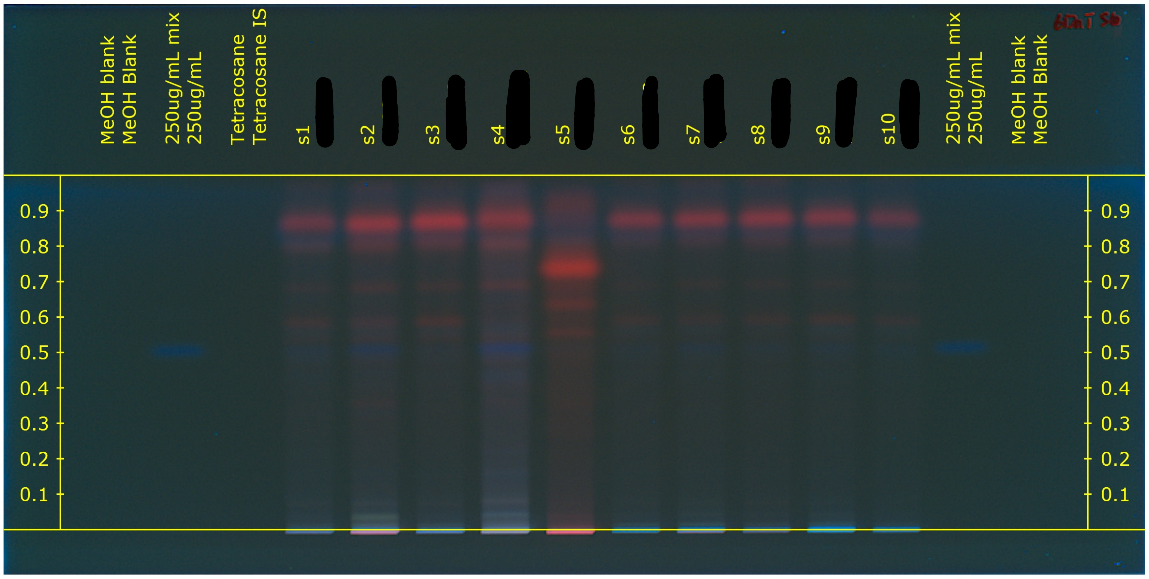


| | |
|---|---|
| Exposure | 3.525 s |
| Contrast | 1 |
| Normalized exposure | Disabled |
| Clarify | Disabled |
| White balance | 1.00, 1.00, 1.00 |

## Scan developed plate 1b - Scanner 3 (S/N: 031025):

| | |
|---|---|
| Executed | 12-Oct-2019 19:12:51 visionCATSuser |

## Scan:

| | |
|---|---|
| Wavelength | 254 nm |

## Track 1:

| | |
|---|---|
| Type | Single λ |

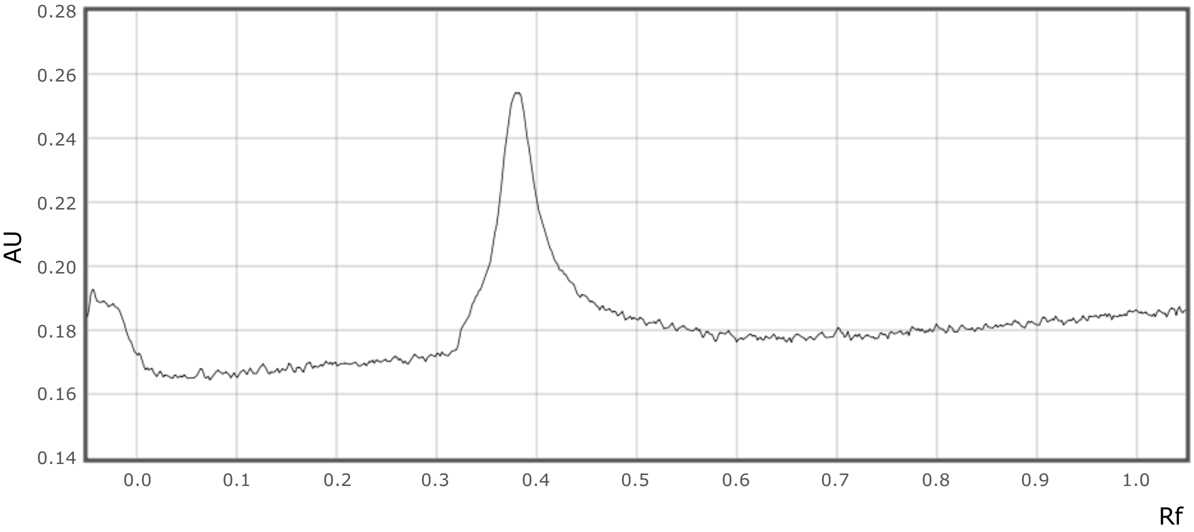

## Track 2:

| Type | Single λ |
|---|---|

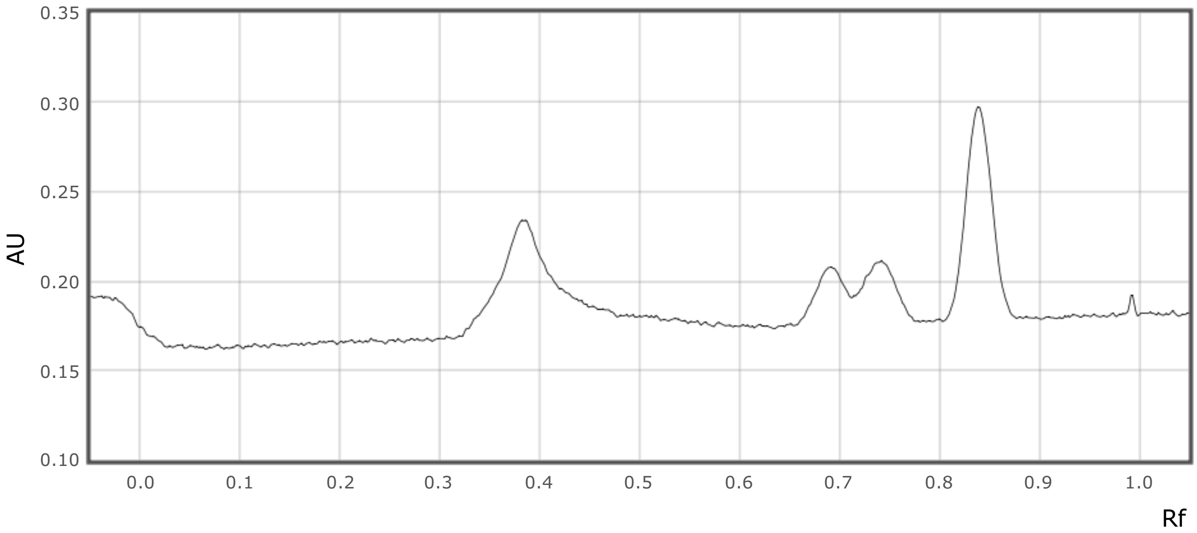


## Track 3:

| Type | Single λ |
|---|---|

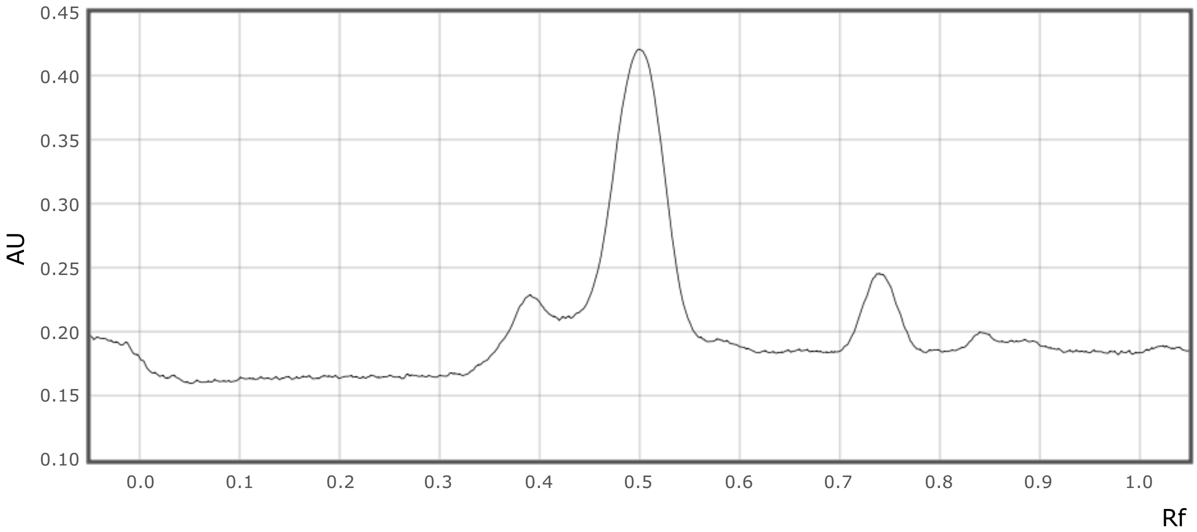


## Track 4:

| Type | Single λ |
|---|---|

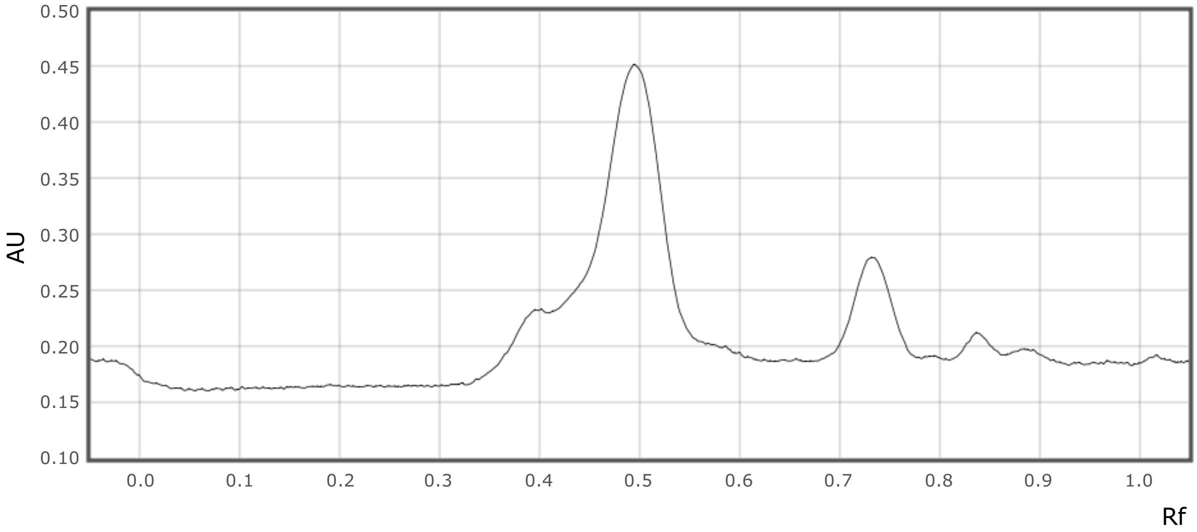


| Track 5: | |
|---|---|
| Type | Single λ |

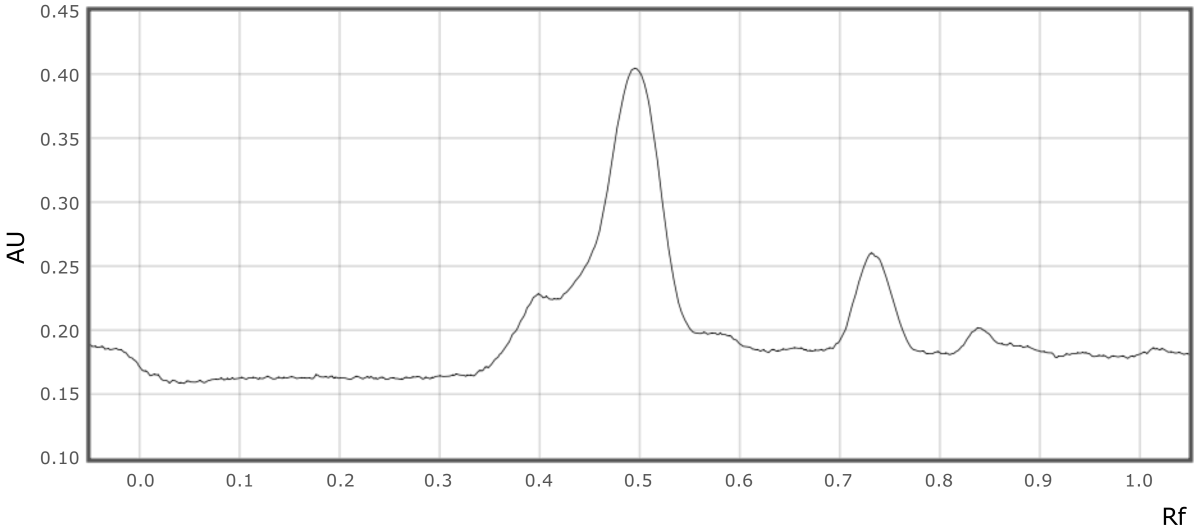


| Track 6: | |
|---|---|
| Type | Single λ |

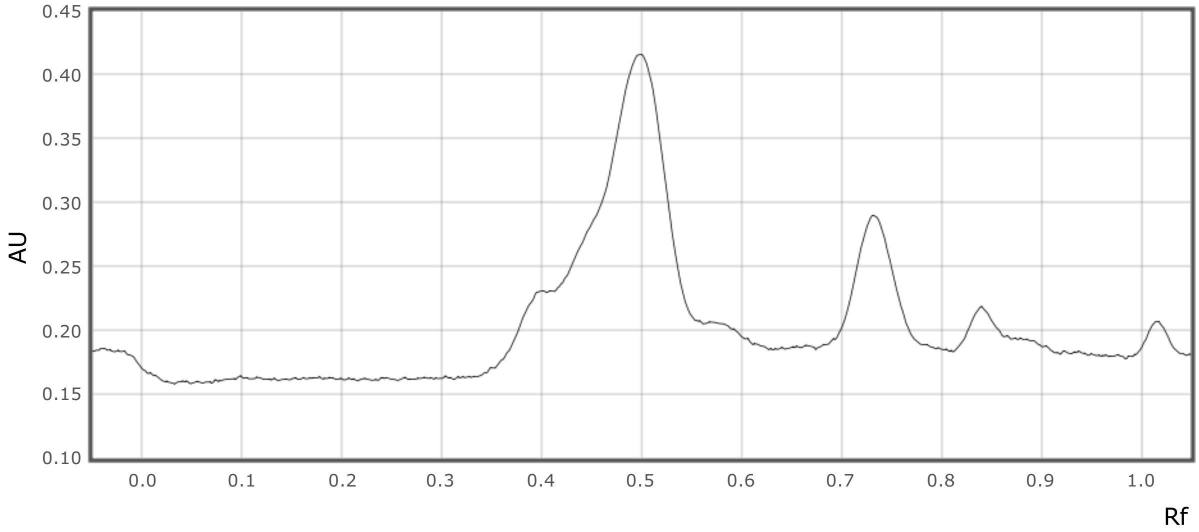


| Track 7: | |
|---|---|
| Type | Single λ |

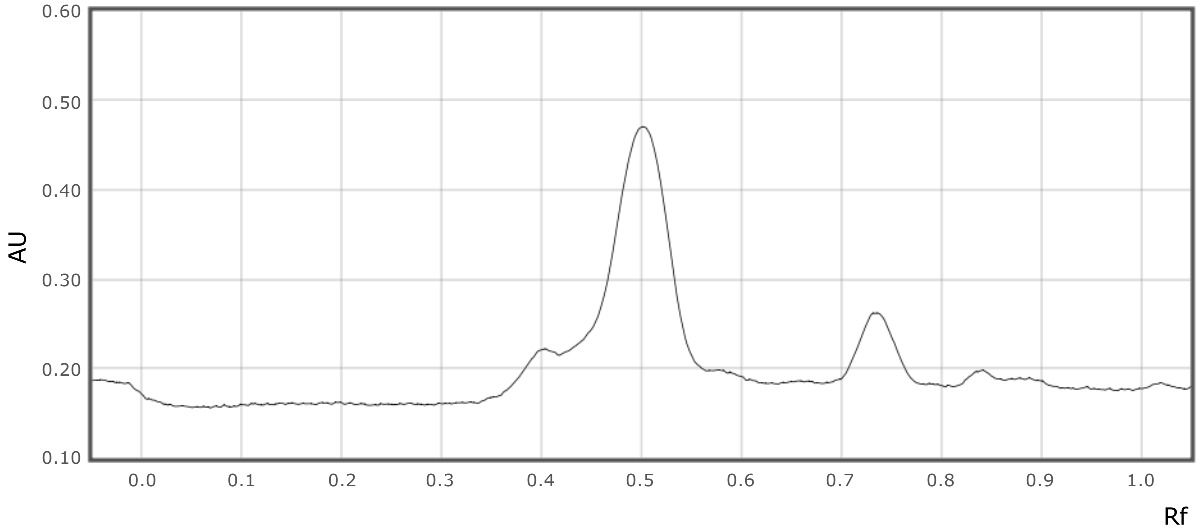


| Track 8: | |
|---|---|
| Type | Single λ |

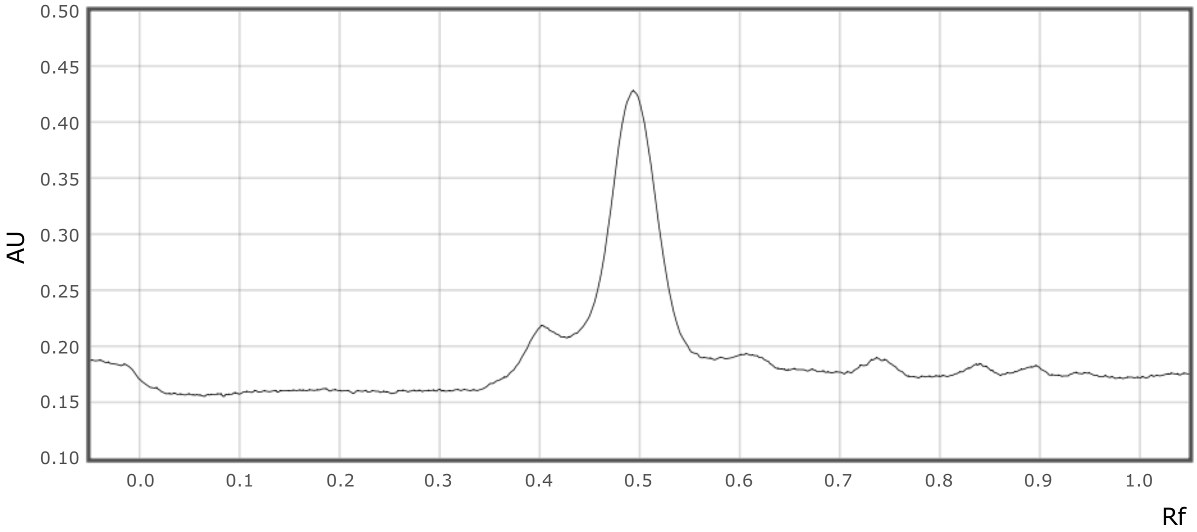


## Track 9:

| | |
|---|---|
| Type | Single λ |

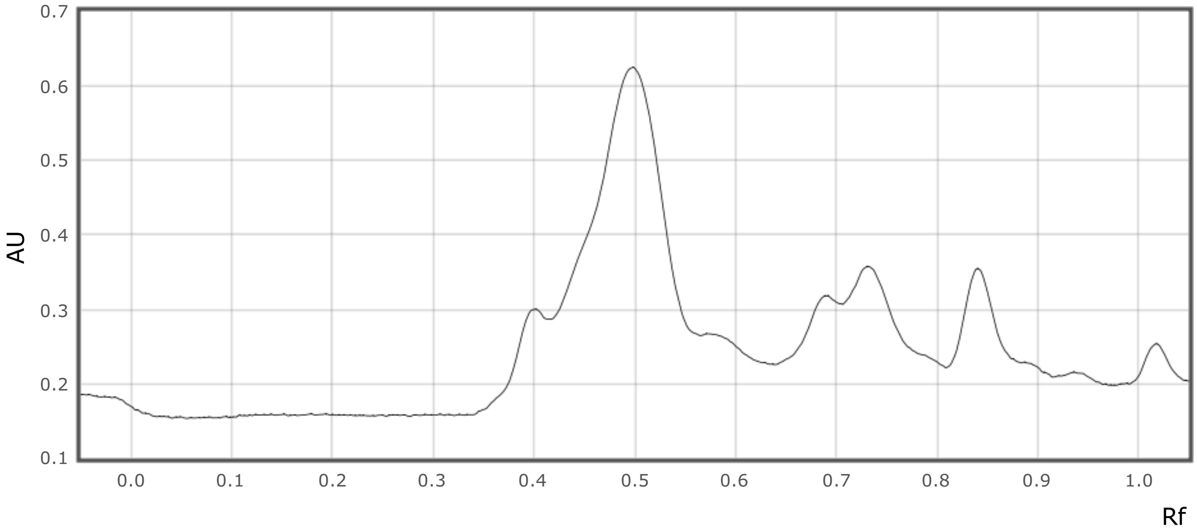


## Track 10:

| | |
|---|---|
| Type | Single λ |

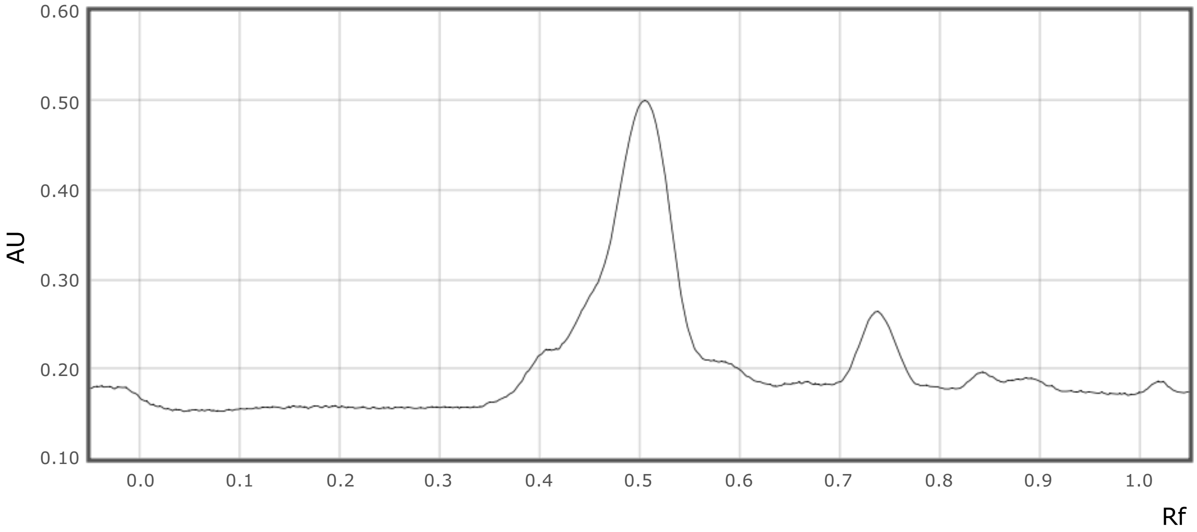


## Track 11:

| Type | Single λ |
| --- | --- |

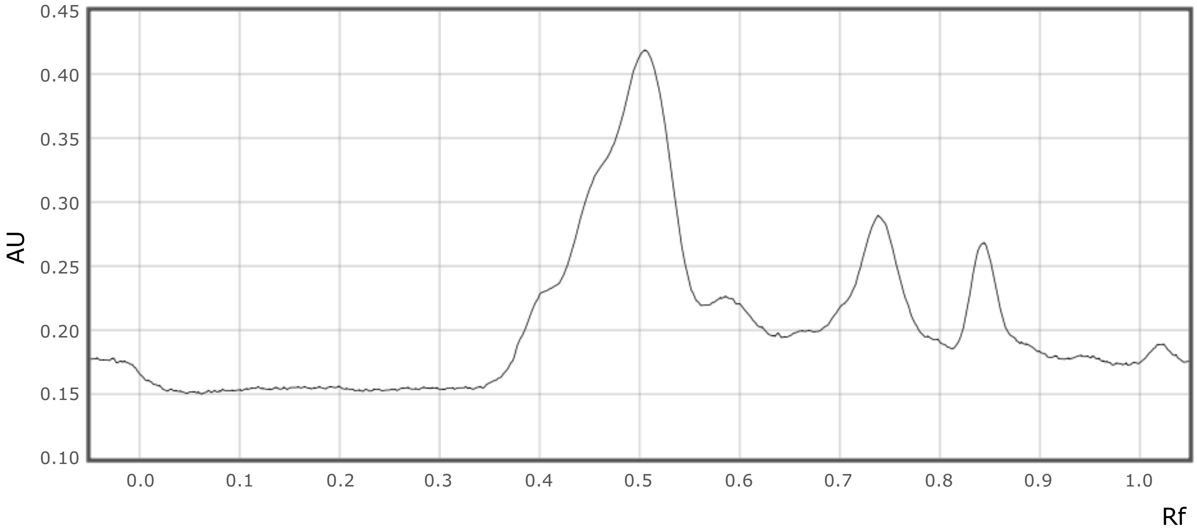


## Track 12:

| Type | Single λ |
| --- | --- |

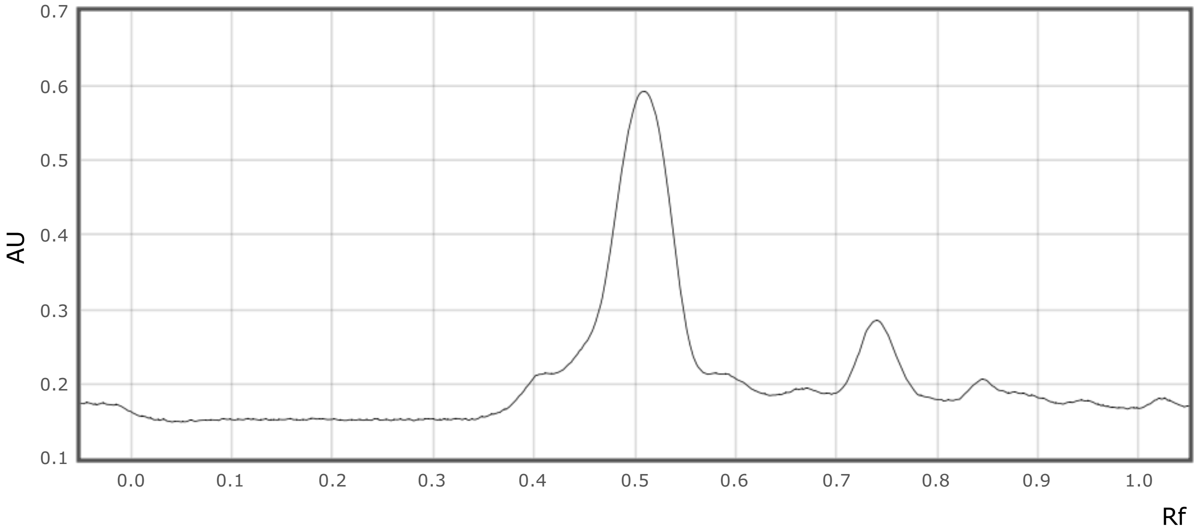


| Track 13: | |
|---|---|
| Type | Single λ |

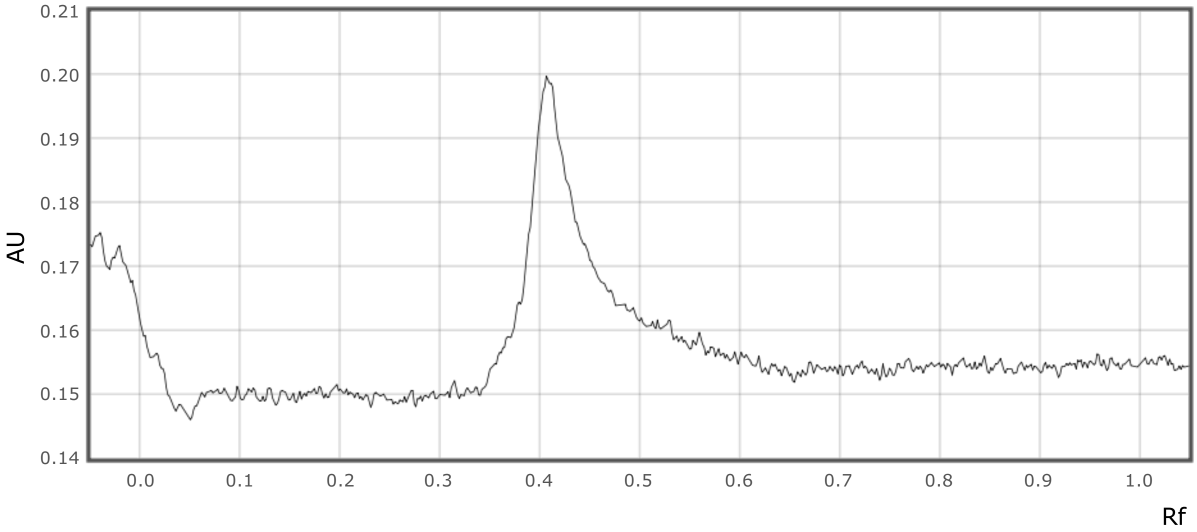


| Track 14: | |
|---|---|
| Type | Single λ |

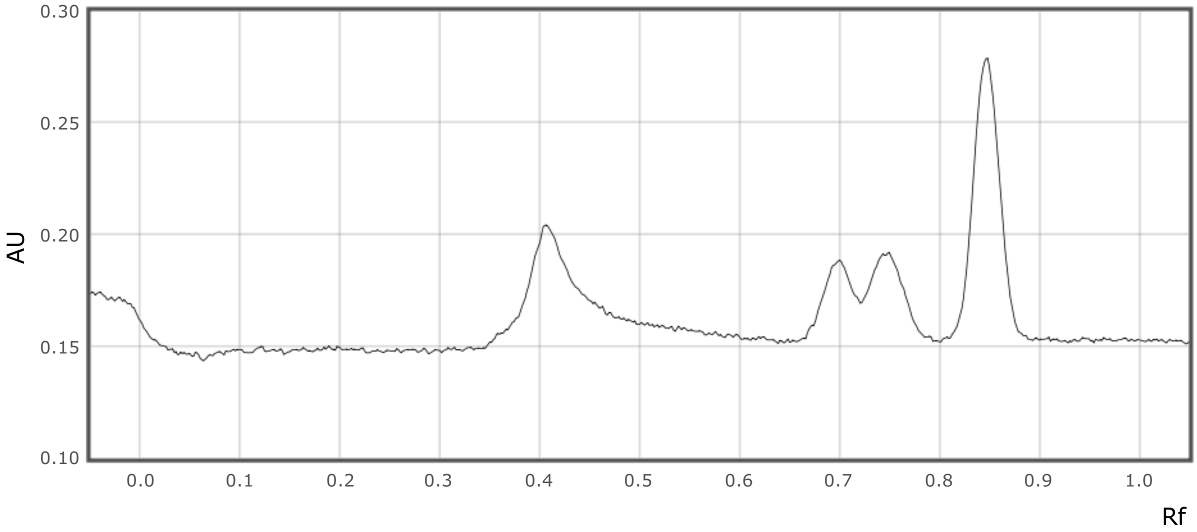


| Track 15: | |
|---|---|
| Type | Single λ |

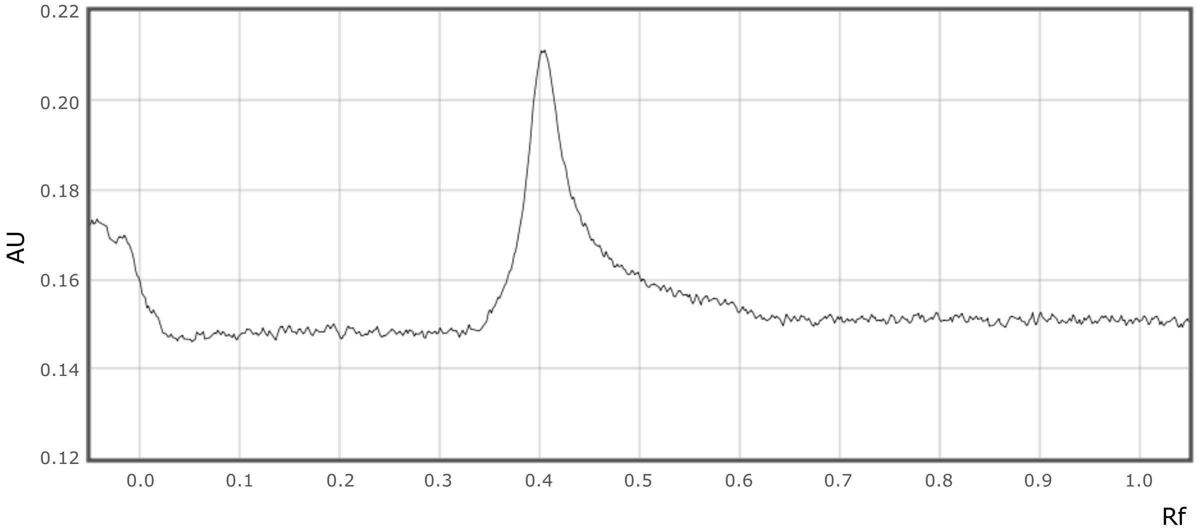


## Derivatization 1 - dip:

| Executed | 12-Oct-2019 19:28:27 visionCATSuser |
|---|---|

## Take image derivatized plate 1a - Visualizer (S/N: 230515):

| Executed | 12-Oct-2019 19:28:28 visionCATSuser |
|---|---|

RT White Derivatized, RemTransVis

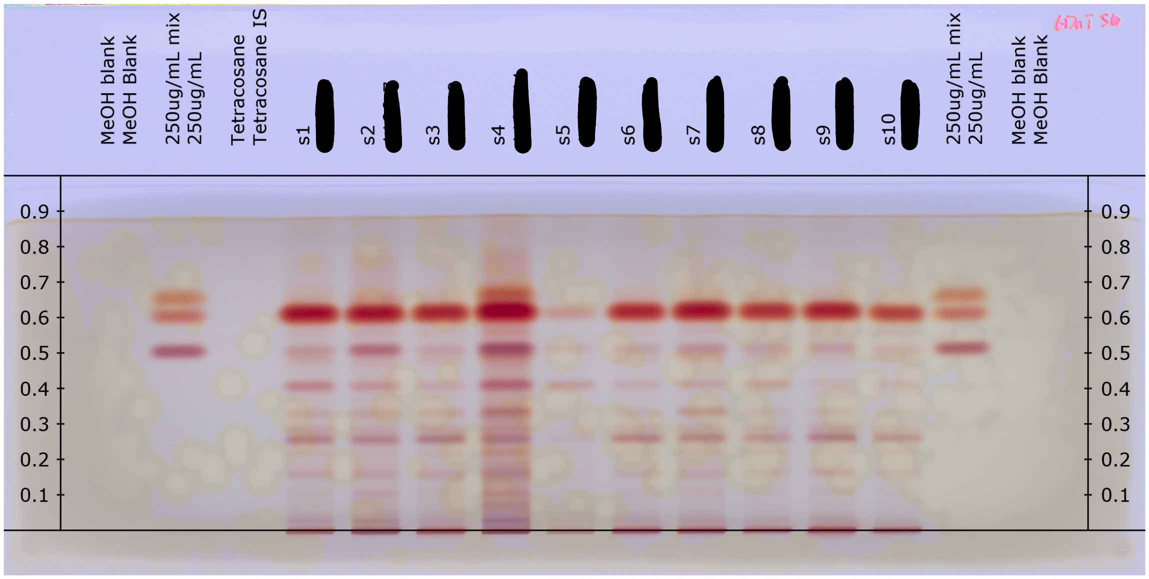


| | |
|---|---|
| Exposure | 0.084 s |
| Contrast | 1 |
| Normalized exposure | Disabled |
| Clarify | Disabled |
| White balance | 1.24, 1.16, 0.75 |

R 366 Derivatized, Remission366

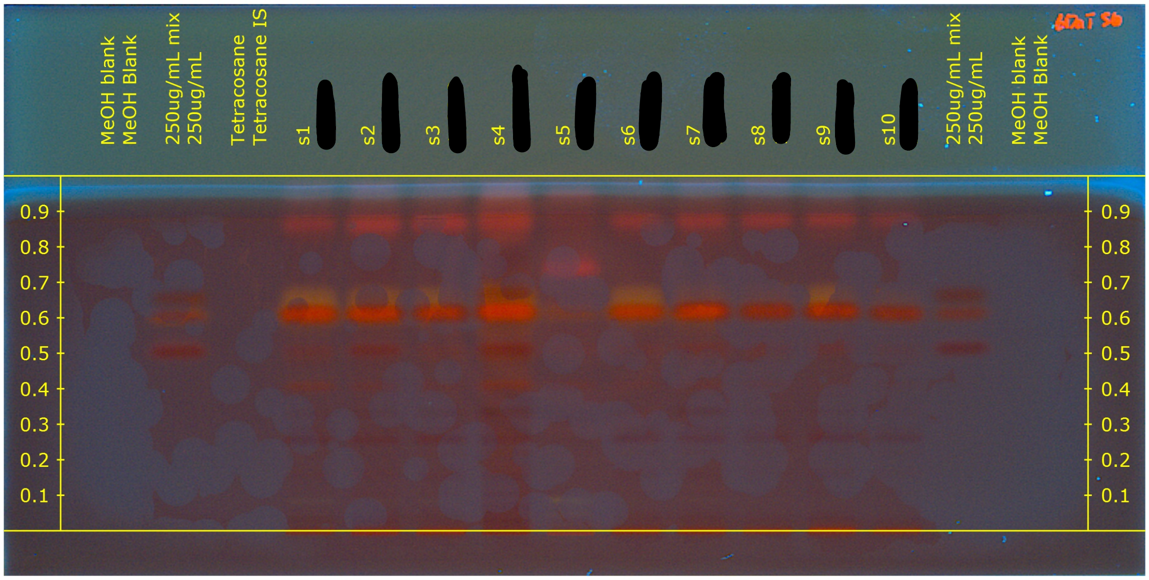


| | |
|---|---|
| Exposure | 9.999 s |
| Contrast | 1 |
| Normalized exposure | Disabled |
| Clarify | Disabled |
| White balance | 1.00, 1.00, 1.00 |

# Evaluation 1 :

| | |
|---|---|
| Validated | false |
| Step | Take image derivatized plate 1a |
| Concentration unit type | Mass / volume |
| Notes | |

# Definition:

## References:

250ug/mL mix

| Substance Name | Concentration | Purity |
|---|---|---|
| 9-THC | 250.000 µg/ml | 100.00 % |
| CBD | 250.000 µg/ml | 100.00 % |
| CBN | 250.000 µg/ml | 100.00 % |

## Samples:

| Vial ID | Amount | Volume solution | Reference amount | Related to |
|---|---|---|---|---|
| MeOH blank | | 0.00 ml | | |
| Tetracosane | | 0.00 ml | | |
| s1 | | 0.00 ml | | |
| s2 | | 0.00 ml | | |
| s3 | | 0.00 ml | | |
| s4 | | 0.00 ml | | |
| s5 | | 0.00 ml | | |
| s6 | | 0.00 ml | | |
| s7 | | 0.00 ml | | |
| s8 | | 0.00 ml | | |
| s9 | | 0.00 ml | | |
| s10 | | 0.00 ml | | |

# Integration parameters:

| | |
|---|---|
| Bounds | [0.000,1.000] |
| Smoothing | Savitzky–Golay of order 3 and window 7 |
| Baseline correction | Lowest slope with noise 0.05 |
| Profile subtraction | Profile subtraction from track 1 |
| Peaks detection | Gauss (legacy) with sensitivity 0.1, separation 1 and threshold 0.1 |

## Scan:

| | |
|---|---|
| Wavelength | RT White |

## Track 1:

| | |
|---|---|
| Type | Sample |
| Vial ID | MeOH blank |
| Description | MeOH Blank |
| Volume | 2.0 µl |

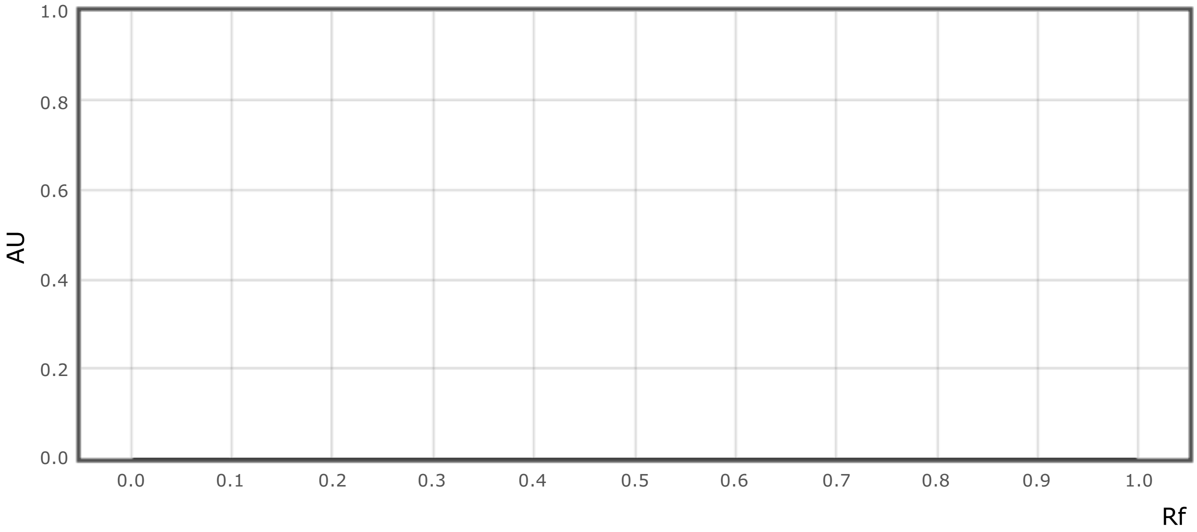


| Peak # | Start | | Max | | | End | | Area | | Manual peak | Substance Name |
|---|---|---|---|---|---|---|---|---|---|---|---|
| | Rf | H | Rf | H | % | Rf | H | A | % | | |

## Track 2:

| | |
|---|---|
| Type | Reference |
| Vial ID | 250ug/mL mix |
| Description | 250ug/mL |
| Volume | 2.0 µl |

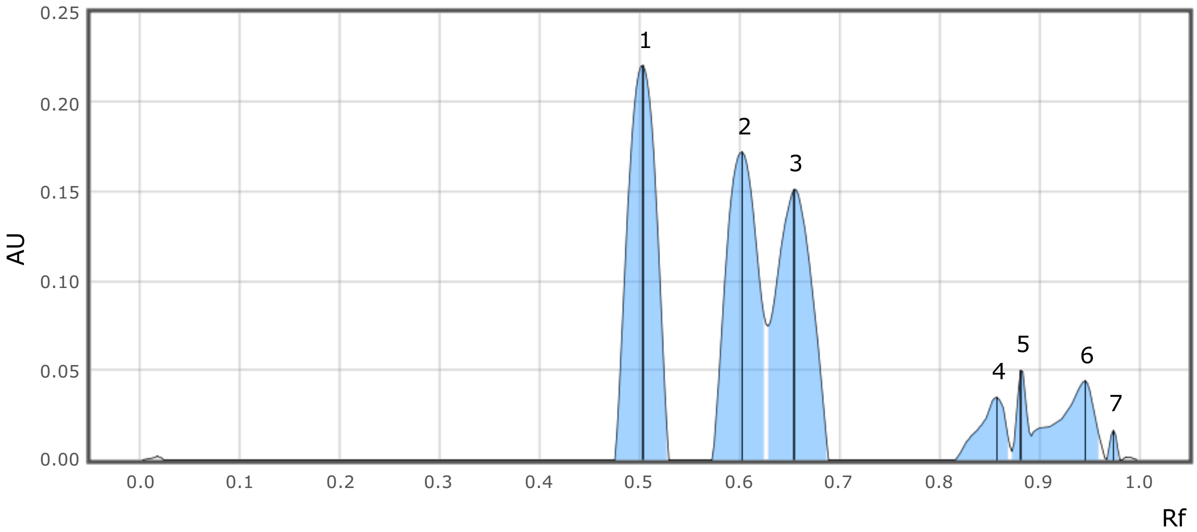


| Peak # | Start | | Max | | | End | | Area | | Manual peak | Substance Name |
|---|---|---|---|---|---|---|---|---|---|---|---|
| | Rf | H | Rf | H | % | Rf | H | A | % | | |
| 1 | 0.475 | 0.0000 | 0.503 | 0.2205 | 31.98 | 0.529 | 0.0000 | 0.00726 | 31.29 | No | CBN |
| 2 | 0.572 | 0.0000 | 0.603 | 0.1720 | 24.95 | 0.626 | 0.0759 | 0.00625 | 26.91 | No | 9-THC |
| 3 | 0.629 | 0.0748 | 0.655 | 0.1511 | 21.93 | 0.689 | 0.0000 | 0.00616 | 26.55 | No | CBD |
| 4 | 0.814 | 0.0000 | 0.858 | 0.0349 | 5.06 | 0.871 | 0.0077 | 0.00103 | 4.44 | No | |
| 5 | 0.873 | 0.0047 | 0.881 | 0.0500 | 7.26 | 0.892 | 0.0133 | 0.00056 | 2.40 | No | |
| 6 | 0.892 | 0.0133 | 0.946 | 0.0442 | 6.41 | 0.966 | 0.0011 | 0.00183 | 7.88 | No | |
| 7 | 0.968 | 0.0002 | 0.974 | 0.0166 | 2.41 | 0.981 | 0.0000 | 0.00012 | 0.53 | No | |

## Track 3:

| | |
|---|---|
| Type | Sample |
| Vial ID | Tetracosane |
| Description | Tetracosane IS |
| Volume | 2.0 µl |

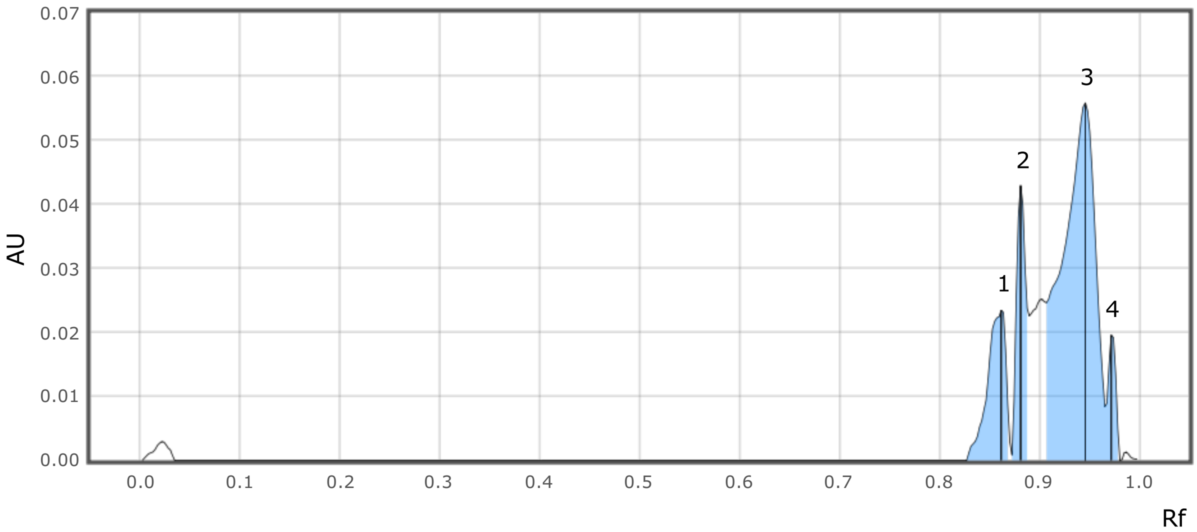


| Peak | Start | | Max | | | End | | Area | | Manual | Substance |
|---|---|---|---|---|---|---|---|---|---|---|---|
| # | Rf | H | Rf | H | % | Rf | H | A | % | peak | Name |
| 1 | 0.827 | 0.0000 | 0.862 | 0.0234 | 16.53 | 0.871 | 0.0028 | 0.00050 | 15.34 | No | |
| 2 | 0.873 | 0.0008 | 0.881 | 0.0429 | 30.28 | 0.890 | 0.0226 | 0.00048 | 14.66 | No | |
| 3 | 0.907 | 0.0246 | 0.946 | 0.0559 | 39.39 | 0.966 | 0.0084 | 0.00210 | 64.38 | No | |
| 4 | 0.966 | 0.0084 | 0.972 | 0.0196 | 13.79 | 0.981 | 0.0000 | 0.00018 | 5.62 | No | |

## Track 4:

| | |
|---|---|
| Type | Sample |
| Vial ID | s1 |
| Description | [redacted] |
| Volume | 2.0 µl |

CAMAG®
World Leader in Planar Chromatography



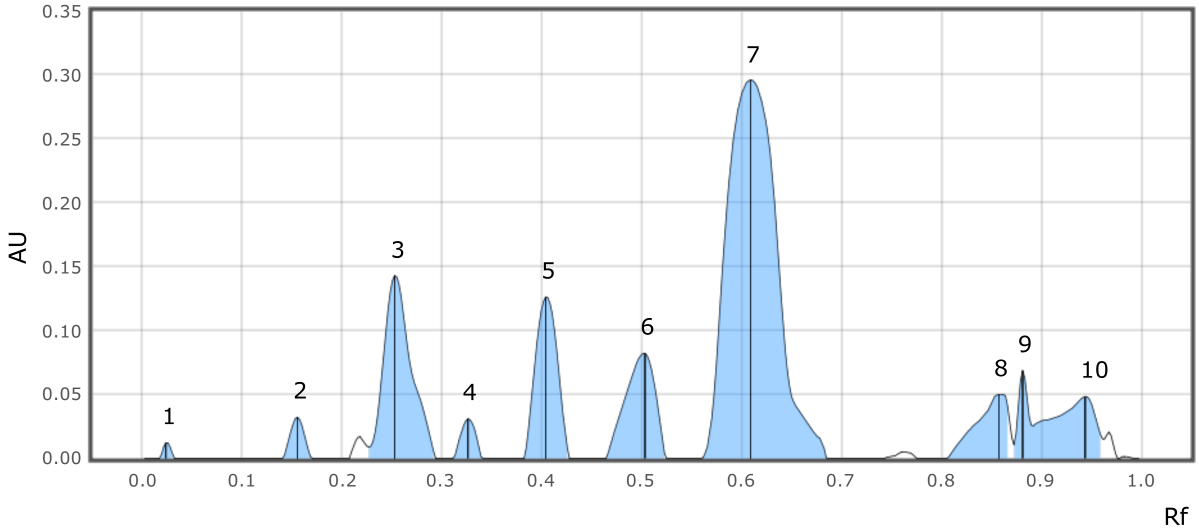


| Peak | Start | | Max | | | End | | Area | | Manual | Substance |
|---|---|---|---|---|---|---|---|---|---|---|---|
| # | Rf | H | Rf | H | % | Rf | H | A | % | peak | Name |
| 1 | 0.017 | 0.0000 | 0.023 | 0.0122 | 1.37 | 0.032 | 0.0000 | 0.00011 | 0.31 | No | |
| 2 | 0.140 | 0.0000 | 0.155 | 0.0323 | 3.63 | 0.170 | 0.0000 | 0.00051 | 1.50 | No | |
| 3 | 0.227 | 0.0087 | 0.253 | 0.1430 | 16.07 | 0.294 | 0.0000 | 0.00452 | 13.28 | No | |
| 4 | 0.311 | 0.0000 | 0.326 | 0.0308 | 3.47 | 0.341 | 0.0000 | 0.00052 | 1.52 | No | |
| 5 | 0.382 | 0.0000 | 0.404 | 0.1263 | 14.20 | 0.428 | 0.0000 | 0.00322 | 9.45 | No | |
| 6 | 0.464 | 0.0000 | 0.503 | 0.0822 | 9.24 | 0.525 | 0.0000 | 0.00283 | 8.29 | No | |
| 7 | 0.559 | 0.0000 | 0.609 | 0.2960 | 33.26 | 0.687 | 0.0000 | 0.01730 | 50.78 | No | 9-THC |
| 8 | 0.806 | 0.0000 | 0.858 | 0.0500 | 5.62 | 0.871 | 0.0161 | 0.00184 | 5.40 | No | |
| 9 | 0.873 | 0.0106 | 0.881 | 0.0687 | 7.72 | 0.892 | 0.0252 | 0.00081 | 2.38 | No | |
| 10 | 0.892 | 0.0252 | 0.944 | 0.0484 | 5.43 | 0.961 | 0.0153 | 0.00242 | 7.09 | No | |

| Track 5: | |
|---|---|
| Type | Sample |
| Vial ID | s2 |
| Description | [redacted] |
| Volume | 2.0 µl |

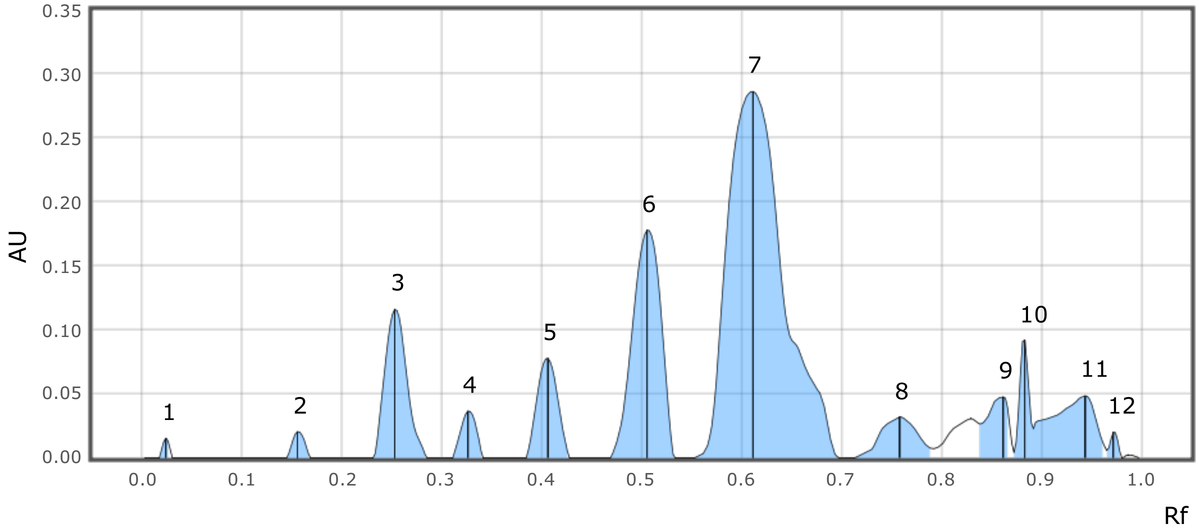

| Peak | Start | | Max | | | End | | Area | | Manual | Substance |
| --- | --- | --- | --- | --- | --- | --- | --- | --- | --- | --- | --- |
| # | Rf | H | Rf | H | % | Rf | H | A | % | peak | Name |
| 1 | 0.017 | 0.0000 | 0.023 | 0.0154 | 1.59 | 0.030 | 0.0000 | 0.00012 | 0.32 | No | |
| 2 | 0.144 | 0.0000 | 0.155 | 0.0205 | 2.11 | 0.168 | 0.0000 | 0.00028 | 0.78 | No | |
| 3 | 0.231 | 0.0000 | 0.253 | 0.1159 | 11.95 | 0.285 | 0.0000 | 0.00293 | 8.05 | No | |
| 4 | 0.309 | 0.0000 | 0.326 | 0.0366 | 3.77 | 0.341 | 0.0000 | 0.00065 | 1.79 | No | |
| 5 | 0.384 | 0.0000 | 0.406 | 0.0777 | 8.02 | 0.428 | 0.0000 | 0.00185 | 5.08 | No | |
| 6 | 0.469 | 0.0000 | 0.505 | 0.1778 | 18.34 | 0.533 | 0.0000 | 0.00583 | 16.03 | No | |
| 7 | 0.553 | 0.0000 | 0.611 | 0.2863 | 29.52 | 0.698 | 0.0000 | 0.01852 | 50.90 | No | 9-THC |
| 8 | 0.713 | 0.0000 | 0.758 | 0.0319 | 3.29 | 0.793 | 0.0072 | 0.00137 | 3.75 | No | |
| 9 | 0.838 | 0.0264 | 0.862 | 0.0475 | 4.90 | 0.871 | 0.0114 | 0.00120 | 3.29 | No | |
| 10 | 0.873 | 0.0042 | 0.884 | 0.0919 | 9.48 | 0.892 | 0.0226 | 0.00098 | 2.71 | No | |
| 11 | 0.892 | 0.0226 | 0.944 | 0.0484 | 4.99 | 0.964 | 0.0088 | 0.00248 | 6.81 | No | |
| 12 | 0.966 | 0.0058 | 0.972 | 0.0199 | 2.05 | 0.981 | 0.0000 | 0.00018 | 0.49 | No | |

## Track 6:

| | |
| --- | --- |
| Type | Sample |
| Vial ID | s3 |
| Description | [redacted] |
| Volume | 2.0 µl |

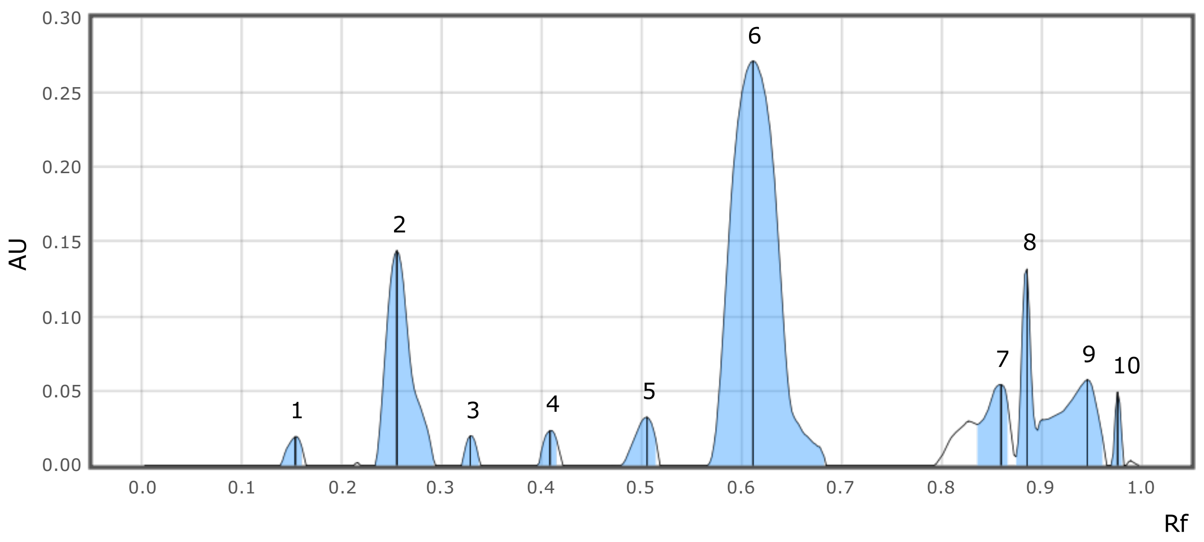


| Peak | Start | | Max | | | End | | Area | | Manual | Substance |
| --- | --- | --- | --- | --- | --- | --- | --- | --- | --- | --- | --- |
| # | Rf | H | Rf | H | % | Rf | H | A | % | peak | Name |
| 1 | 0.138 | 0.0000 | 0.153 | 0.0192 | 2.40 | 0.164 | 0.0000 | 0.00031 | 1.18 | No | |
| 2 | 0.233 | 0.0000 | 0.255 | 0.1438 | 17.95 | 0.294 | 0.0000 | 0.00408 | 15.62 | No | |
| 3 | 0.320 | 0.0000 | 0.328 | 0.0197 | 2.46 | 0.339 | 0.0000 | 0.00024 | 0.91 | No | |
| 4 | 0.395 | 0.0000 | 0.408 | 0.0233 | 2.91 | 0.421 | 0.0000 | 0.00037 | 1.41 | No | |
| 5 | 0.477 | 0.0000 | 0.505 | 0.0322 | 4.02 | 0.518 | 0.0000 | 0.00072 | 2.76 | No | |
| 6 | 0.566 | 0.0000 | 0.611 | 0.2708 | 33.79 | 0.685 | 0.0000 | 0.01456 | 55.72 | No | 9-THC |
| 7 | 0.836 | 0.0273 | 0.860 | 0.0541 | 6.75 | 0.873 | 0.0069 | 0.00145 | 5.55 | No | |
| 8 | 0.875 | 0.0058 | 0.886 | 0.1315 | 16.41 | 0.897 | 0.0235 | 0.00144 | 5.50 | No | |
| 9 | 0.897 | 0.0235 | 0.946 | 0.0574 | 7.17 | 0.966 | 0.0000 | 0.00264 | 10.10 | No | |
| 10 | 0.970 | 0.0000 | 0.977 | 0.0492 | 6.13 | 0.983 | 0.0000 | 0.00033 | 1.26 | No | |

## Track 7:

| | |
|---|---|
| Type | Sample |
| Vial ID | s4 |
| Description | [redacted] |
| Volume | 2.0 µl |

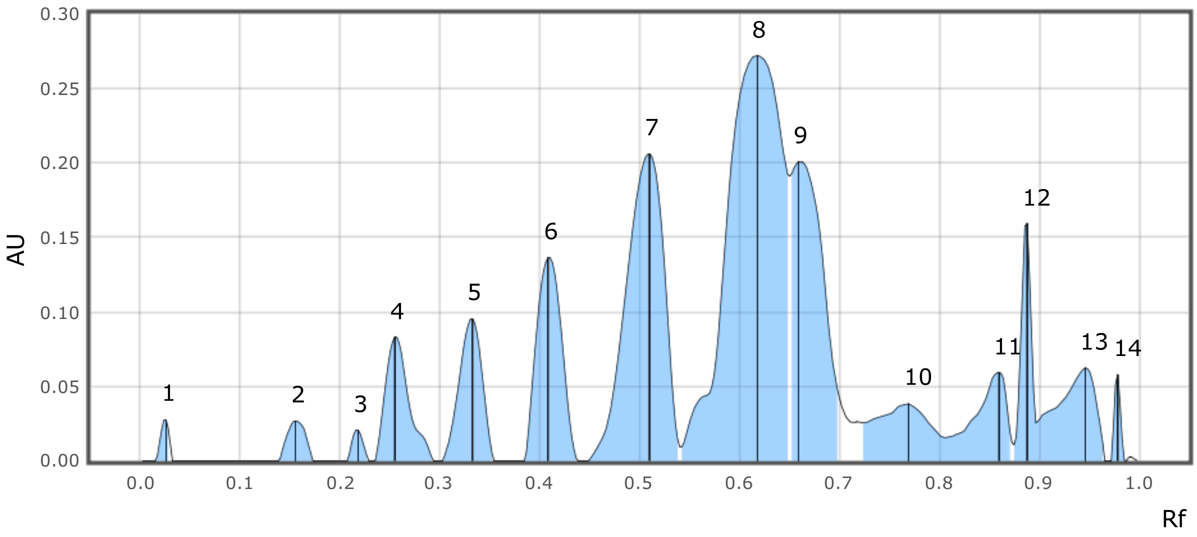


| Peak | Start | | Max | | | End | | Area | | Manual | Substance |
|---|---|---|---|---|---|---|---|---|---|---|---|
| # | Rf | H | Rf | H | % | Rf | H | A | % | peak | Name |
| 1 | 0.015 | 0.0000 | 0.026 | 0.0275 | 1.90 | 0.032 | 0.0000 | 0.00027 | 0.50 | No | |
| 2 | 0.138 | 0.0000 | 0.155 | 0.0268 | 1.85 | 0.175 | 0.0000 | 0.00058 | 1.07 | No | |
| 3 | 0.207 | 0.0000 | 0.218 | 0.0207 | 1.43 | 0.229 | 0.0000 | 0.00026 | 0.48 | No | |
| 4 | 0.233 | 0.0000 | 0.255 | 0.0830 | 5.75 | 0.294 | 0.0000 | 0.00225 | 4.19 | No | |
| 5 | 0.302 | 0.0000 | 0.333 | 0.0953 | 6.60 | 0.354 | 0.0000 | 0.00252 | 4.68 | No | |
| 6 | 0.384 | 0.0000 | 0.408 | 0.1362 | 9.43 | 0.438 | 0.0000 | 0.00397 | 7.37 | No | |
| 7 | 0.447 | 0.0000 | 0.510 | 0.2059 | 14.25 | 0.540 | 0.0095 | 0.00907 | 16.86 | No | |
| 8 | 0.542 | 0.0094 | 0.618 | 0.2716 | 18.80 | 0.650 | 0.1910 | 0.01762 | 32.75 | No | 9-THC |
| 9 | 0.650 | 0.1910 | 0.659 | 0.2008 | 13.90 | 0.701 | 0.0368 | 0.00754 | 14.03 | Yes | CBD |
| 10 | 0.724 | 0.0254 | 0.769 | 0.0382 | 2.65 | 0.806 | 0.0155 | 0.00242 | 4.49 | No | |
| 11 | 0.806 | 0.0155 | 0.860 | 0.0594 | 4.11 | 0.873 | 0.0122 | 0.00222 | 4.12 | No | |
| 12 | 0.875 | 0.0109 | 0.888 | 0.1593 | 11.02 | 0.897 | 0.0255 | 0.00183 | 3.41 | No | |
| 13 | 0.897 | 0.0255 | 0.946 | 0.0623 | 4.31 | 0.966 | 0.0000 | 0.00285 | 5.30 | No | |
| 14 | 0.972 | 0.0000 | 0.979 | 0.0579 | 4.01 | 0.987 | 0.0000 | 0.00040 | 0.75 | No | |

## Track 8:

| | |
|---|---|
| Type | Sample |
| Vial ID | s5 |
| Description | [redacted] |
| Volume | 2.0 µl |

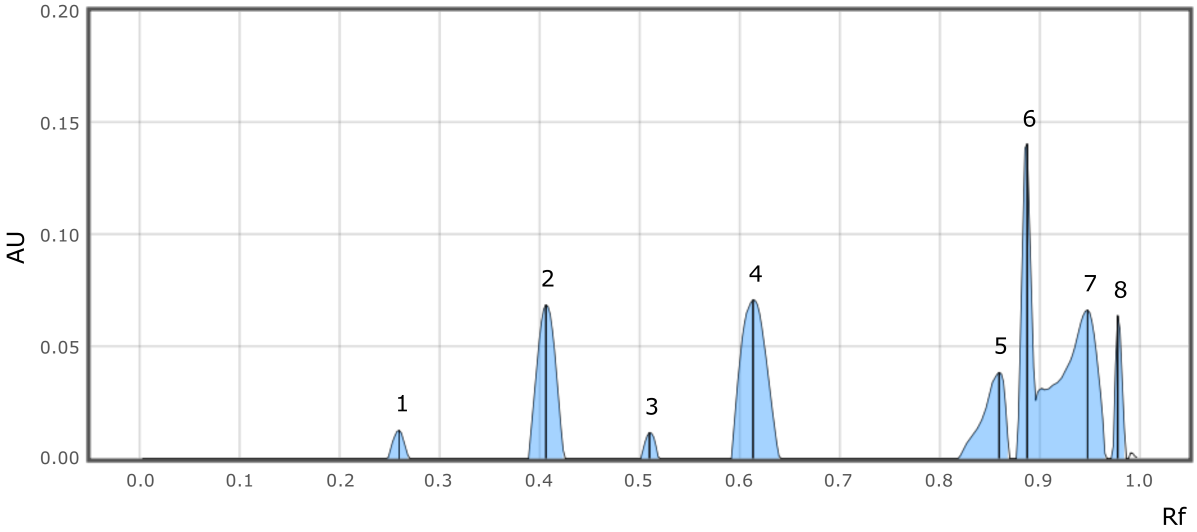


| Peak # | Start Rf | Start H | Max Rf | Max H | Max % | End Rf | End H | Area A | Area % | Manual peak | Substance Name |
|---|---|---|---|---|---|---|---|---|---|---|---|
| 1 | 0.246 | 0.0000 | 0.259 | 0.0125 | 2.65 | 0.270 | 0.0000 | 0.00016 | 1.62 | No | |
| 2 | 0.389 | 0.0000 | 0.406 | 0.0687 | 14.53 | 0.425 | 0.0000 | 0.00145 | 15.01 | No | |
| 3 | 0.501 | 0.0000 | 0.510 | 0.0116 | 2.45 | 0.521 | 0.0000 | 0.00013 | 1.33 | No | |
| 4 | 0.592 | 0.0000 | 0.613 | 0.0708 | 14.97 | 0.642 | 0.0000 | 0.00209 | 21.67 | No | 9-THC |
| 5 | 0.819 | 0.0000 | 0.860 | 0.0384 | 8.12 | 0.871 | 0.0000 | 0.00098 | 10.12 | No | |
| 6 | 0.877 | 0.0000 | 0.888 | 0.1406 | 29.74 | 0.897 | 0.0259 | 0.00148 | 15.34 | No | |
| 7 | 0.897 | 0.0259 | 0.948 | 0.0663 | 14.02 | 0.968 | 0.0000 | 0.00290 | 30.01 | No | |
| 8 | 0.972 | 0.0000 | 0.979 | 0.0638 | 13.51 | 0.987 | 0.0000 | 0.00047 | 4.90 | No | |

## Track 9:

| | |
|---|---|
| Type | Sample |
| Vial ID | s6 |
| Description | [redacted] |
| Volume | 2.0 µl |

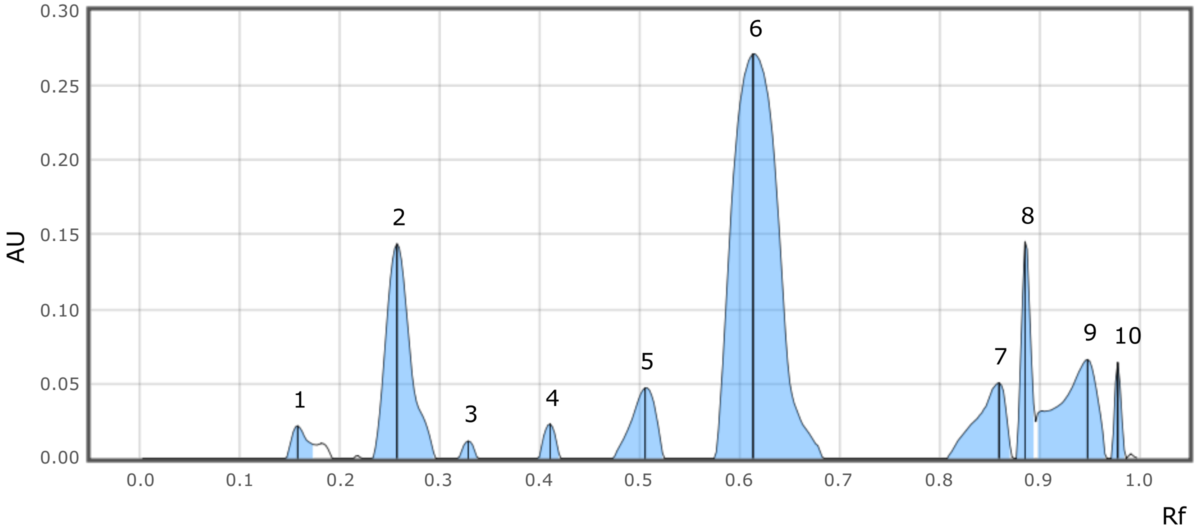

| Peak | Start | | Max | | | End | | Area | | Manual | Substance |
|---|---|---|---|---|---|---|---|---|---|---|---|
| # | Rf | H | Rf | H | % | Rf | H | A | % | peak | Name |
| 1 | 0.144 | 0.0000 | 0.157 | 0.0218 | 2.59 | 0.177 | 0.0090 | 0.00041 | 1.52 | No | |
| 2 | 0.231 | 0.0000 | 0.257 | 0.1438 | 17.02 | 0.296 | 0.0000 | 0.00401 | 14.84 | No | |
| 3 | 0.317 | 0.0000 | 0.328 | 0.0116 | 1.37 | 0.339 | 0.0000 | 0.00014 | 0.51 | No | |
| 4 | 0.397 | 0.0000 | 0.410 | 0.0230 | 2.72 | 0.421 | 0.0000 | 0.00030 | 1.13 | No | |
| 5 | 0.473 | 0.0000 | 0.505 | 0.0473 | 5.60 | 0.525 | 0.0000 | 0.00130 | 4.83 | No | |
| 6 | 0.575 | 0.0000 | 0.613 | 0.2709 | 32.08 | 0.685 | 0.0000 | 0.01429 | 52.95 | No | 9-THC |
| 7 | 0.808 | 0.0000 | 0.860 | 0.0505 | 5.98 | 0.873 | 0.0010 | 0.00169 | 6.26 | No | |
| 8 | 0.877 | 0.0000 | 0.886 | 0.1453 | 17.20 | 0.897 | 0.0246 | 0.00152 | 5.63 | No | |
| 9 | 0.899 | 0.0303 | 0.948 | 0.0662 | 7.84 | 0.968 | 0.0000 | 0.00287 | 10.62 | No | |
| 10 | 0.972 | 0.0000 | 0.979 | 0.0643 | 7.61 | 0.987 | 0.0000 | 0.00046 | 1.71 | No | |

## Track 10:

| | |
|---|---|
| Type | Sample |
| Vial ID | s7 |
| Description | [redacted] |
| Volume | 2.0 µl |

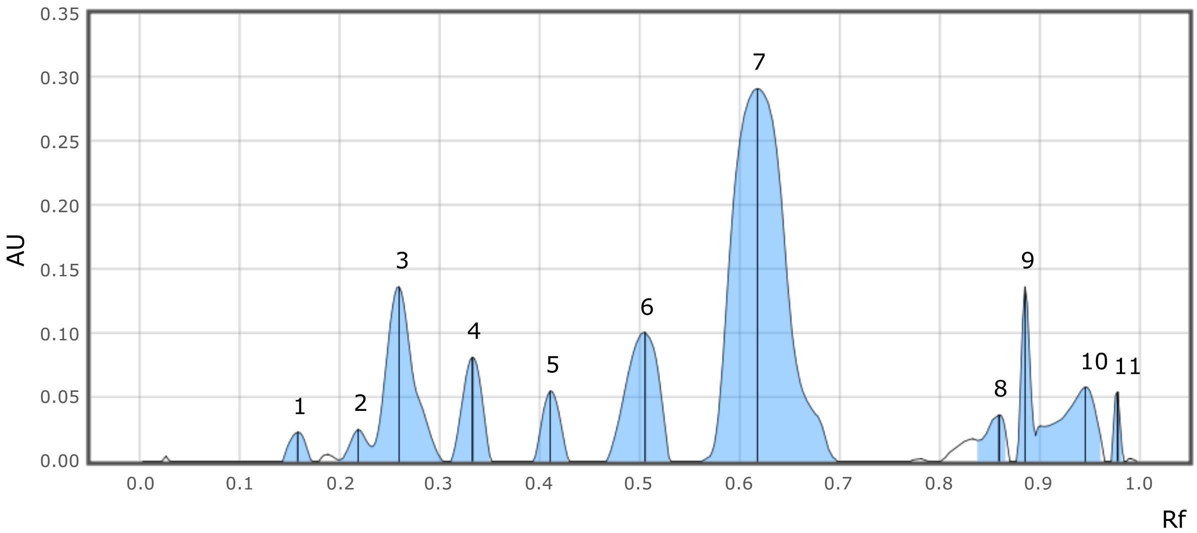


| Peak | Start | | Max | | | End | | Area | | Manual | Substance |
|---|---|---|---|---|---|---|---|---|---|---|---|
| # | Rf | H | Rf | H | % | Rf | H | A | % | peak | Name |
| 1 | 0.142 | 0.0000 | 0.157 | 0.0227 | 2.27 | 0.173 | 0.0000 | 0.00041 | 1.18 | No | |
| 2 | 0.199 | 0.0011 | 0.218 | 0.0248 | 2.49 | 0.231 | 0.0115 | 0.00047 | 1.34 | No | |
| 3 | 0.231 | 0.0115 | 0.259 | 0.1365 | 13.68 | 0.304 | 0.0000 | 0.00433 | 12.37 | No | |
| 4 | 0.311 | 0.0000 | 0.333 | 0.0815 | 8.17 | 0.352 | 0.0000 | 0.00181 | 5.17 | No | |
| 5 | 0.393 | 0.0000 | 0.410 | 0.0548 | 5.49 | 0.430 | 0.0000 | 0.00111 | 3.17 | No | |
| 6 | 0.467 | 0.0000 | 0.505 | 0.1007 | 10.09 | 0.531 | 0.0000 | 0.00375 | 10.71 | No | |
| 7 | 0.562 | 0.0000 | 0.618 | 0.2913 | 29.19 | 0.698 | 0.0000 | 0.01801 | 51.53 | No | 9-THC |
| 8 | 0.838 | 0.0163 | 0.860 | 0.0363 | 3.64 | 0.871 | 0.0000 | 0.00082 | 2.33 | No | |
| 9 | 0.877 | 0.0000 | 0.886 | 0.1365 | 13.68 | 0.897 | 0.0201 | 0.00135 | 3.85 | No | |
| 10 | 0.897 | 0.0201 | 0.946 | 0.0582 | 5.83 | 0.966 | 0.0000 | 0.00254 | 7.26 | No | |
| 11 | 0.972 | 0.0000 | 0.979 | 0.0545 | 5.46 | 0.985 | 0.0000 | 0.00038 | 1.09 | No | |

## Track 11:

| | |
|---|---|
| Type | Sample |
| Vial ID | s8 |
| Description | [redacted] |
| Volume | 2.0 µl |

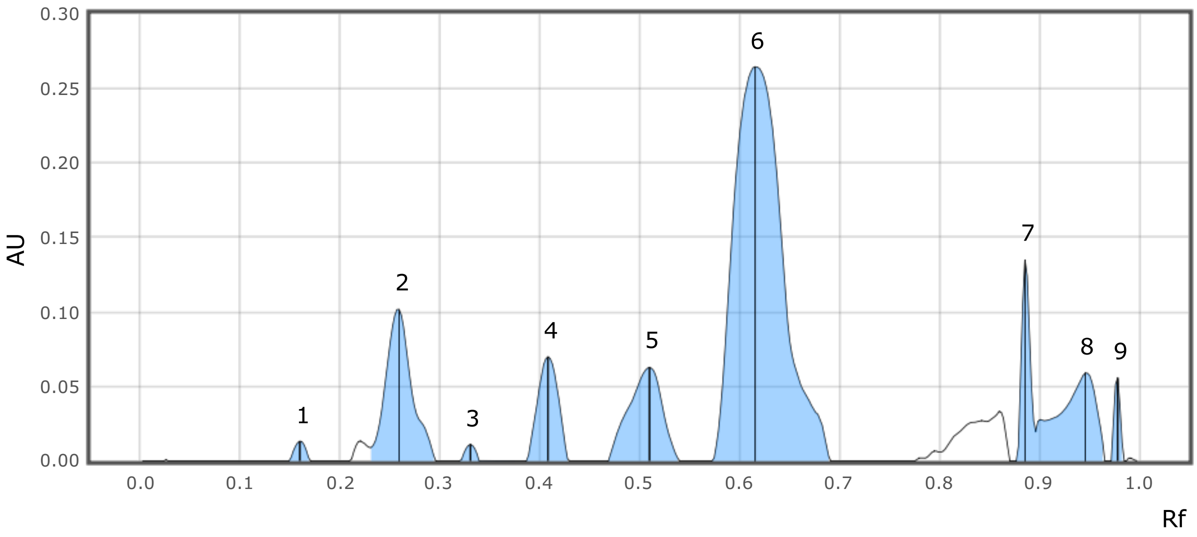


| Peak # | Start | | Max | | | End | | Area | | Manual peak | Substance Name |
|---|---|---|---|---|---|---|---|---|---|---|---|
| | Rf | H | Rf | H | % | Rf | H | A | % | | |
| 1 | 0.149 | 0.0000 | 0.160 | 0.0132 | 1.71 | 0.170 | 0.0000 | 0.00017 | 0.62 | No | |
| 2 | 0.231 | 0.0089 | 0.259 | 0.1016 | 13.16 | 0.296 | 0.0000 | 0.00302 | 11.32 | No | |
| 3 | 0.320 | 0.0000 | 0.330 | 0.0110 | 1.43 | 0.341 | 0.0000 | 0.00013 | 0.48 | No | |
| 4 | 0.387 | 0.0000 | 0.408 | 0.0699 | 9.05 | 0.430 | 0.0000 | 0.00164 | 6.17 | No | |
| 5 | 0.469 | 0.0000 | 0.510 | 0.0628 | 8.14 | 0.540 | 0.0000 | 0.00242 | 9.07 | No | |
| 6 | 0.572 | 0.0000 | 0.616 | 0.2643 | 34.23 | 0.691 | 0.0000 | 0.01504 | 56.44 | No | 9-THC |
| 7 | 0.877 | 0.0000 | 0.886 | 0.1348 | 17.45 | 0.897 | 0.0195 | 0.00130 | 4.89 | No | |
| 8 | 0.897 | 0.0195 | 0.946 | 0.0589 | 7.62 | 0.966 | 0.0000 | 0.00255 | 9.55 | No | |
| 9 | 0.972 | 0.0000 | 0.979 | 0.0556 | 7.21 | 0.985 | 0.0000 | 0.00039 | 1.45 | No | |

## Track 12:

| | |
|---|---|
| Type | Sample |
| Vial ID | s9 |
| Description | [redacted] |
| Volume | 2.0 µl |

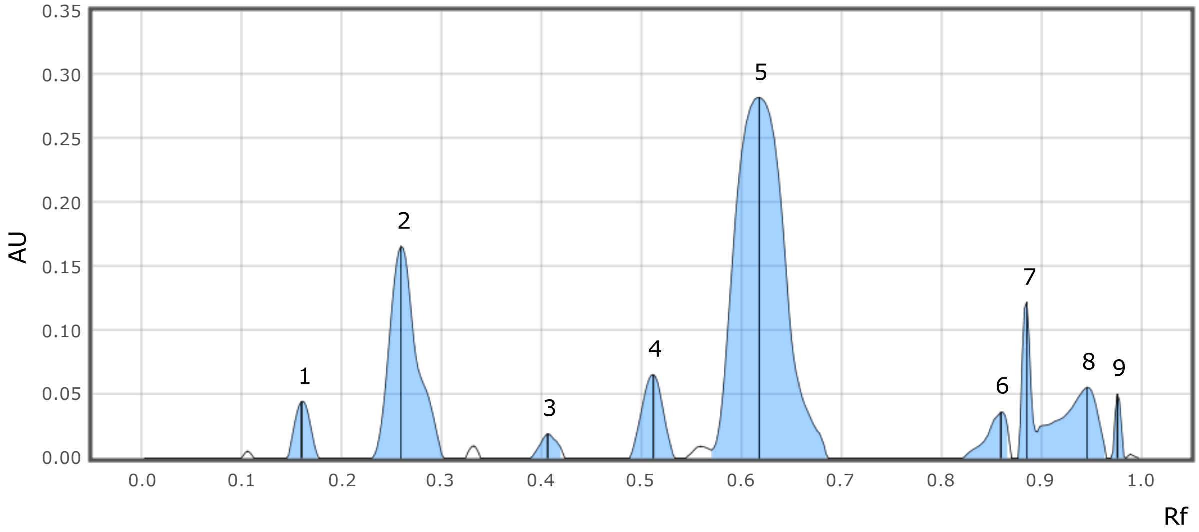


| Peak # | Start Rf | Start H | Max Rf | Max H | Max % | End Rf | End H | Area A | Area % | Manual peak | Substance Name |
|---|---|---|---|---|---|---|---|---|---|---|---|
| 1 | 0.144 | 0.0000 | 0.160 | 0.0443 | 5.28 | 0.177 | 0.0000 | 0.00078 | 2.70 | No | |
| 2 | 0.229 | 0.0000 | 0.259 | 0.1657 | 19.73 | 0.302 | 0.0000 | 0.00524 | 18.03 | No | |
| 3 | 0.389 | 0.0000 | 0.406 | 0.0191 | 2.27 | 0.423 | 0.0000 | 0.00037 | 1.28 | No | |
| 4 | 0.488 | 0.0000 | 0.512 | 0.0653 | 7.78 | 0.533 | 0.0000 | 0.00158 | 5.44 | No | |
| 5 | 0.570 | 0.0062 | 0.618 | 0.2820 | 33.58 | 0.687 | 0.0000 | 0.01629 | 56.01 | No | 9-THC |
| 6 | 0.821 | 0.0000 | 0.860 | 0.0359 | 4.27 | 0.871 | 0.0000 | 0.00085 | 2.91 | No | |
| 7 | 0.877 | 0.0000 | 0.886 | 0.1219 | 14.51 | 0.897 | 0.0205 | 0.00120 | 4.14 | No | |
| 8 | 0.897 | 0.0205 | 0.946 | 0.0554 | 6.60 | 0.966 | 0.0000 | 0.00242 | 8.33 | No | |
| 9 | 0.970 | 0.0000 | 0.977 | 0.0502 | 5.98 | 0.985 | 0.0000 | 0.00034 | 1.17 | No | |

## Track 13:

| | |
|---|---|
| Type | Sample |
| Vial ID | s10 |
| Description | [redacted] |
| Volume | 2.0 µl |

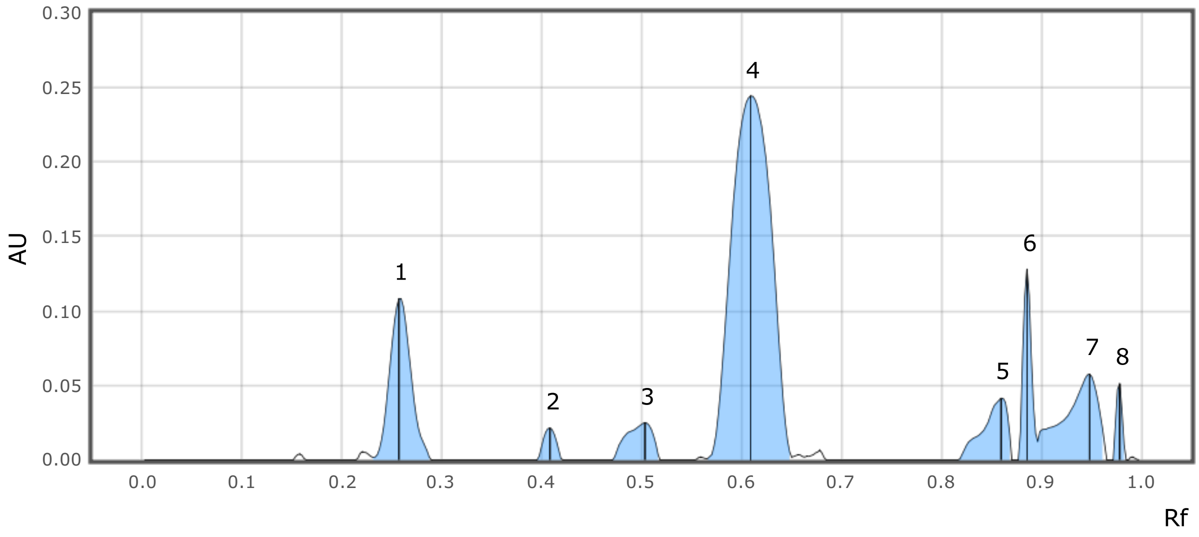

| Peak # | Start | | Max | | | End | | Area | | Manual peak | Substance Name |
|---|---|---|---|---|---|---|---|---|---|---|---|
| | Rf | H | Rf | H | % | Rf | H | A | % | | |
| 1 | 0.231 | 0.0017 | 0.257 | 0.1081 | 15.99 | 0.289 | 0.0000 | 0.00263 | 13.23 | No | |
| 2 | 0.395 | 0.0000 | 0.408 | 0.0214 | 3.16 | 0.421 | 0.0000 | 0.00031 | 1.56 | No | |
| 3 | 0.471 | 0.0000 | 0.503 | 0.0250 | 3.70 | 0.518 | 0.0000 | 0.00076 | 3.83 | No | |
| 4 | 0.566 | 0.0008 | 0.609 | 0.2440 | 36.07 | 0.650 | 0.0018 | 0.01120 | 56.31 | No | 9-THC |
| 5 | 0.817 | 0.0000 | 0.860 | 0.0413 | 6.11 | 0.871 | 0.0000 | 0.00117 | 5.86 | No | |
| 6 | 0.877 | 0.0000 | 0.886 | 0.1280 | 18.92 | 0.897 | 0.0122 | 0.00122 | 6.14 | No | |
| 7 | 0.897 | 0.0122 | 0.948 | 0.0575 | 8.49 | 0.966 | 0.0000 | 0.00225 | 11.30 | No | |
| 8 | 0.972 | 0.0000 | 0.979 | 0.0511 | 7.56 | 0.985 | 0.0000 | 0.00035 | 1.77 | No | |

## Track 14:

| | |
|---|---|
| Type | Reference |
| Vial ID | 250ug/mL mix |
| Description | 250ug/mL |
| Volume | 2.0 µl |

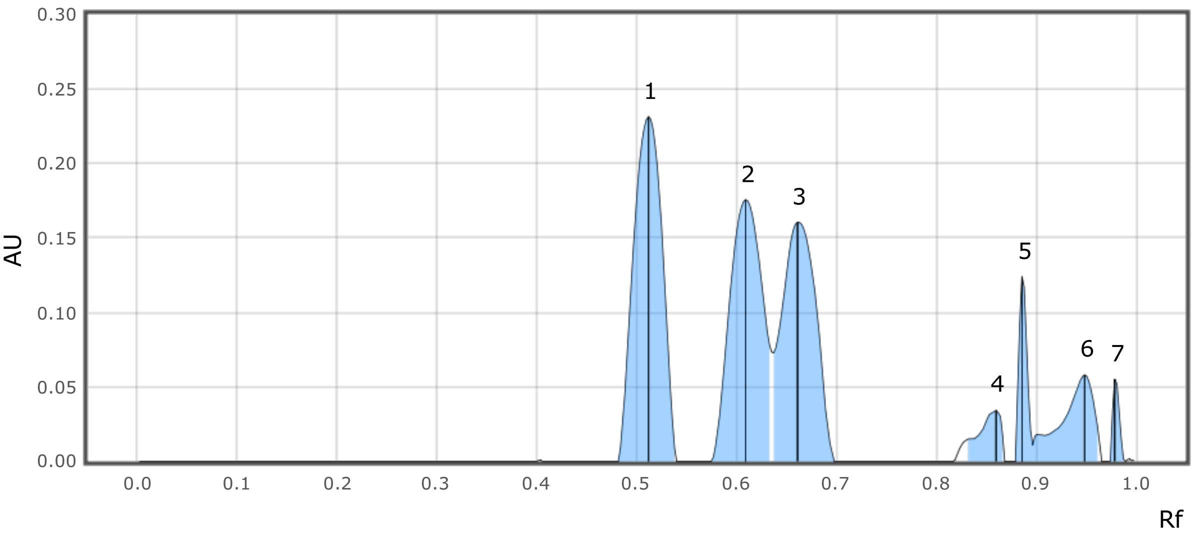


| Peak # | Start | | Max | | | End | | Area | | Manual peak | Substance Name |
|---|---|---|---|---|---|---|---|---|---|---|---|
| | Rf | H | Rf | H | % | Rf | H | A | % | | |
| 1 | 0.482 | 0.0000 | 0.512 | 0.2312 | 27.59 | 0.540 | 0.0000 | 0.00768 | 30.49 | No | CBN |
| 2 | 0.575 | 0.0000 | 0.609 | 0.1754 | 20.93 | 0.635 | 0.0733 | 0.00664 | 26.35 | No | 9-THC |
| 3 | 0.637 | 0.0728 | 0.661 | 0.1602 | 19.12 | 0.700 | 0.0000 | 0.00630 | 24.98 | No | CBD |
| 4 | 0.830 | 0.0142 | 0.860 | 0.0341 | 4.07 | 0.868 | 0.0000 | 0.00088 | 3.50 | No | |
| 5 | 0.879 | 0.0000 | 0.886 | 0.1239 | 14.79 | 0.897 | 0.0108 | 0.00115 | 4.55 | No | |
| 6 | 0.897 | 0.0108 | 0.948 | 0.0579 | 6.91 | 0.966 | 0.0000 | 0.00214 | 8.48 | No | |
| 7 | 0.972 | 0.0000 | 0.979 | 0.0551 | 6.58 | 0.989 | 0.0000 | 0.00041 | 1.65 | No | |

## Track 15:

| | |
|---|---|
| Type | Sample |
| Vial ID | MeOH blank |
| Description | MeOH Blank |
| Volume | 2.0 µl |

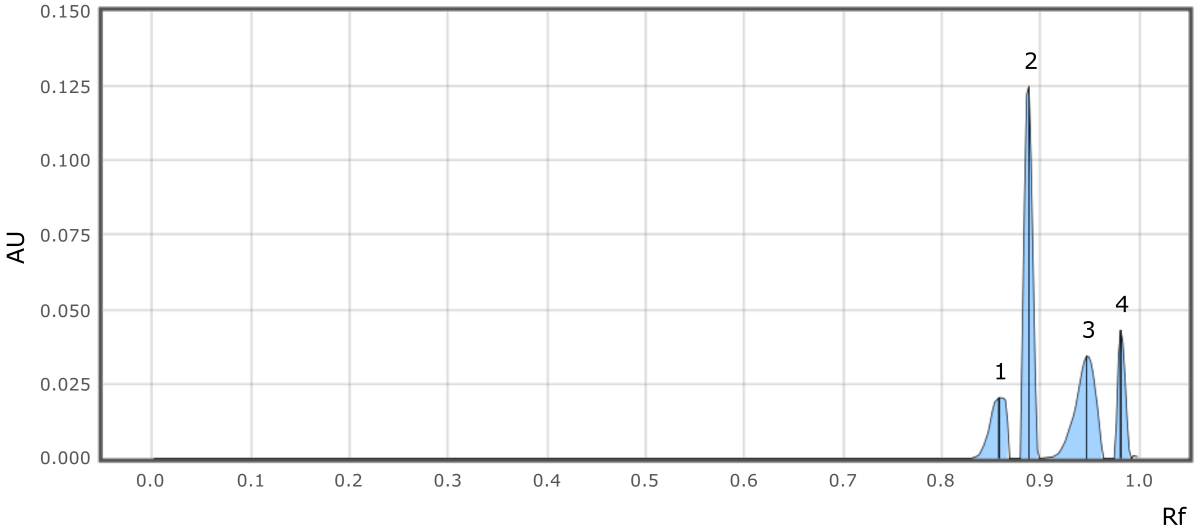


| Peak # | Start | | Max | | | End | | Area | | Manual peak | Substance Name |
|---|---|---|---|---|---|---|---|---|---|---|---|
| | Rf | H | Rf | H | % | Rf | H | A | % | | |
| 1 | 0.827 | 0.0000 | 0.858 | 0.0204 | 9.16 | 0.868 | 0.0000 | 0.00040 | 14.31 | No | |
| 2 | 0.879 | 0.0000 | 0.888 | 0.1246 | 56.09 | 0.899 | 0.0000 | 0.00123 | 43.75 | No | |
| 3 | 0.903 | 0.0000 | 0.946 | 0.0342 | 15.40 | 0.964 | 0.0000 | 0.00081 | 28.83 | No | |
| 4 | 0.974 | 0.0000 | 0.981 | 0.0430 | 19.34 | 0.992 | 0.0000 | 0.00037 | 13.11 | No | |

## Calibration results:

### Height calibration for substance 9-THC @ RT White:

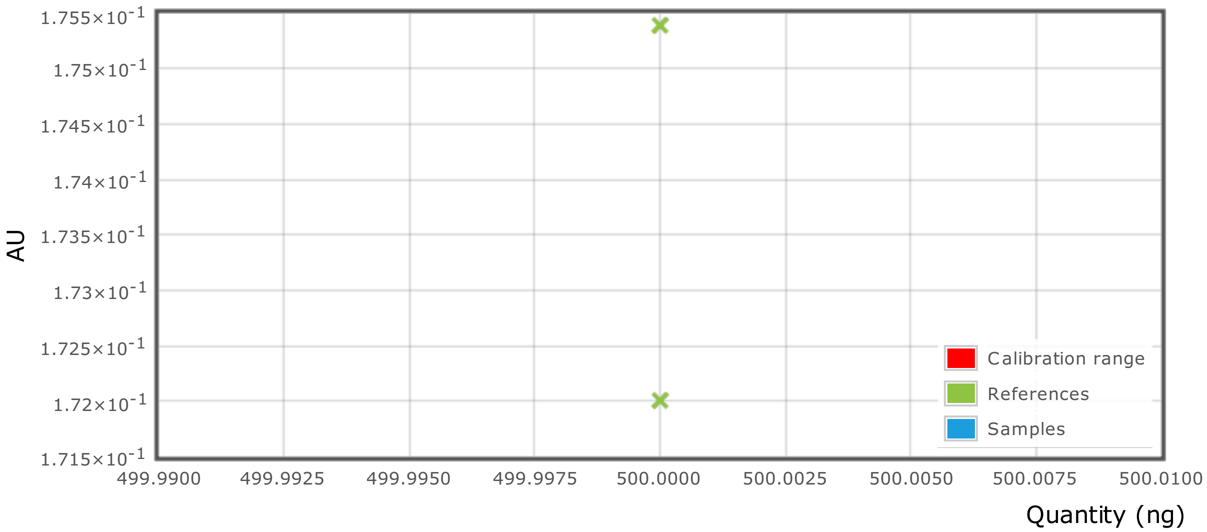

| | |
|---|---|
| Regression mode | Linear-2 |
| Range deviation | 5.00 % |
| Related substances | Default |
| Number of references | 2 |
| Calibration function | $y=0x$ |
| Coefficient of variation | CV 0.00 % |
| Correlation coefficient | n/a |
| ⚠ | Unable to compute the results for this substance because there wasn't enough groups of references replicas (at least 1 for Linear-1, 2 for Linear2 and Mime-1 and 3 for Polynomial and MiMe-2) |

## Height calibration for substance CBD @ RT White:

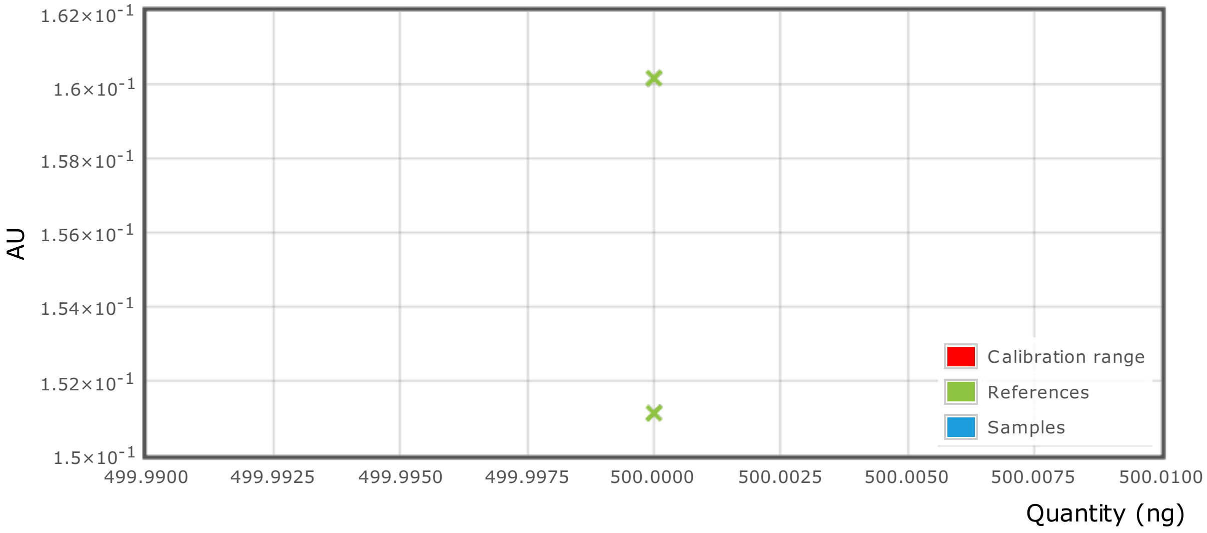


| | |
|---|---|
| Regression mode | Linear-2 |
| Range deviation | 5.00 % |
| Related substances | Default |
| Number of references | 2 |
| Calibration function | $y=0x$ |
| Coefficient of variation | CV 0.00 % |
| Correlation coefficient | n/a |
| ⚠ | Unable to compute the results for this substance because there wasn't enough groups of references replicas (at least 1 for Linear-1, 2 for Linear2 and Mime-1 and 3 for Polynomial and MiMe-2) |

## Height calibration for substance CBN @ RT White:

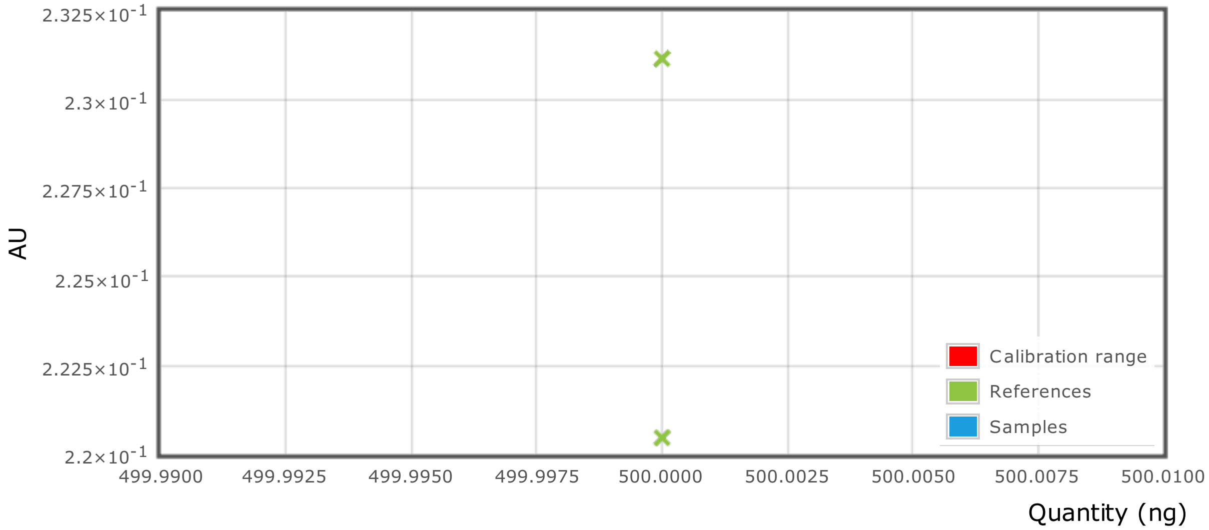


| | |
|---|---|
| Regression mode | Linear-2 |
| Range deviation | 5.00 % |
| Related substances | Default |
| Number of references | 2 |
| Calibration function | $y=0x$ |
| Coefficient of variation | CV 0.00 % |
| Correlation coefficient | n/a |
| ⚠ | Unable to compute the results for this substance because there wasn't enough groups of references replicas (at least 1 for Linear-1, 2 for Linear2 and Mime-1 and 3 for Polynomial and MiMe-2) |

## Results:

| Substance having no available results | | |
|---|---|---|
| ⚠ | 9-THC | Unable to compute the results for this substance because there wasn't enough groups of references replicas (at least 1 for Linear-1, 2 for Linear2 and Mime-1 and 3 for Polynomial and MiMe-2) |
| ⚠ | CBD | Unable to compute the results for this substance because there wasn't enough groups of references replicas (at least 1 for Linear-1, 2 for Linear2 and Mime-1 and 3 for Polynomial and MiMe-2) |
| ⚠ | CBN | There wasn't any sample application available in the assignments for this substance. Please check that the peaks were correctly detected and assigned for this substance. |

A track marked with ⚠ means: this result is outside the regression range given by the reference assignments, but is included in the results because it is in the allowed range deviation.

| Analyst: | Reviewer: |
|---|---|
