# Supplementary material for: High Performance Thin-Layer Chromatography (HPTLC) data of Cannabinoids in ten mobile phase systems
Source: Data Brief. 2020 Jun 30;31:105955. doi: 10.1016/j.dib.2020.105955 (PMC7352075; doi:10.1016/j.dib.2020.105955)
Supplement: Supplementary file 1 [file mmc1.zip › S4-Case sample reports/6DaT-sample run-7.pdf]

Analysis: 6DaT-sample run-7

Path: Home/YL Research

Based on method: Samples (no cal)

Created	14-Oct-2019 18:20:17	visionCATSuser
Modified	14-Oct-2019 20:24:41	visionCATSuser
Last HPTLC log	14-Oct-2019 20:24:41	Analysis modified
Explorer notes		

Track	Vial ID	Description	Volume	Position	Type
1	MeOH blank	MeOH Blank	2.0 µl	A1	Sample
2	250ug/mL mix	250ug/mL	2.0 µl	A2	Reference
3	Tetracosane	Tetracosane IS	2.0 µl	A3	Sample
4	s1	[redacted]	2.0 µl	B1	Sample
5	s2	[redacted]	2.0 µl	B2	Sample
6	s3	[redacted]	2.0 µl	B3	Sample
7	s4	[redacted]	2.0 µl	B4	Sample
8	s5	[redacted]	2.0 µl	B5	Sample
9	s6	[redacted]	2.0 µl	B6	Sample
10	s7	[redacted]	2.0 µl	B7	Sample
11	s8	[redacted]	2.0 µl	B8	Sample
12	s9	[redacted]	2.0 µl	B9	Sample
13	s10	[redacted]	2.0 µl	B10	Sample
14	250ug/mL mix	250ug/mL	2.0 µl	A2	Reference
15	MeOH blank	MeOH Blank	2.0 µl	A1	Sample
Sequence table notes					

A track marked with ⚠ means: the application type is overridden in some evaluation(s).

System setup:

Software	Server User-PC, version 2.5.18072.1
ATS4	S/N:080713
Chamber	N/A
Derivatization dip	N/A
Scanner3	S/N:031025
Visualizer	S/N:230515

Chromatography

Plate layout:

Stationary phase	Merck, HPTLC plates silica gel 60 F 254
Plate format	200.0 x 100.0 mm
Application type	Band
Application	Position Y: 8.0 mm, length: 8.0 mm, width: 0.0 mm
Track	First position X: 20.0 mm, distance: 11.4 mm
Solvent front position	70.0 mm
Notes	

Take image clean plate 1a - Visualizer (S/N: 230515):

Quality	Enhanced
RT White	auto capture, Auto, level 85 %, Band
R 254	auto capture, Auto, level 85 %, Band
Instrument diagnostics	Valid diagnostics
Documentation step label	
Notes	

Application 1 - ATS 4 (S/N: 080713):

Spray gas	NI
Sample solvent type	Methanol
Filling speed	15 µl/s
Predosage volume	200 nl
Retraction volume	200 nl
Dosage speed	150 nl/s
Filling quality	User
Rinsing cycles / vacuum	2 / 4 s
Filling cycles / vacuum	1 / 4 s
Rinsing solvent name	Methanol
Nozzle temperature	Unheated
Rack in use	Standard
Instrument diagnostics	Valid diagnostics
Notes	

Development 1 - Chamber:

Tank	TTC 20x10
Mobile phase	6% diethylamine in toluene
Saturation time	20 min
Use saturation pad	true
Use smartALERT	false
Volume front through	10 ml
Volume rear through	25 ml
Drying time	5 min
Drying temperature	Room temperature
Notes	

Take image developed plate 1a - Visualizer (S/N: 230515):

Quality	Enhanced
RT White	auto capture, Auto, level 85 %, Band
R 254	auto capture, Auto, level 85 %, Band
R 366	auto capture, Auto, level 85 %, Band
Instrument diagnostics	Valid diagnostics
Documentation step label	
Notes	

Scan developed plate 1b - Scanner 3 (S/N: 031025):

Scanner type	Single λ
Optimization for	Resolution
Measurement mode	Absorption
Filter	n/a
Detector mode	Automatic
Scanning speed	20 mm/s
Data resolution	100 µm/step
Slit	5 x 0.2 mm, micro
Partial scan	No
Lamp	Deuterium & Tungsten
Wavelength(s)	254 nm
Instrument diagnostics	Valid diagnostics
Documentation step label	
Notes	

Derivatization 1 - dip:

Reagent name	
Dipping speed	5
Dipping time	0 s
Reagent preparation	
Heating	100 °C for 3 min, heated after
Notes	

Take image derivatized plate 1a - Visualizer (S/N: 230515):

Quality	Enhanced
RT White	auto capture, Auto, level 85 %, Band
R 366	auto capture, Auto, level 85 %, Band
Instrument diagnostics	Valid diagnostics
Documentation step label	
Notes	

System suitability tests:

SST settings:

SST tracks	

Data acquisition

Application 1 - ATS 4 (S/N: 080713):

Executed	14-Oct-2019 18:31:20 visionCATSuser

Development 1 - Chamber:

Executed	14-Oct-2019 19:06:19 visionCATSuser

Take image developed plate 1a - Visualizer (S/N: 230515):

Executed	14-Oct-2019 19:58:01 visionCATSuser

RT White

Developed, RemTransVis

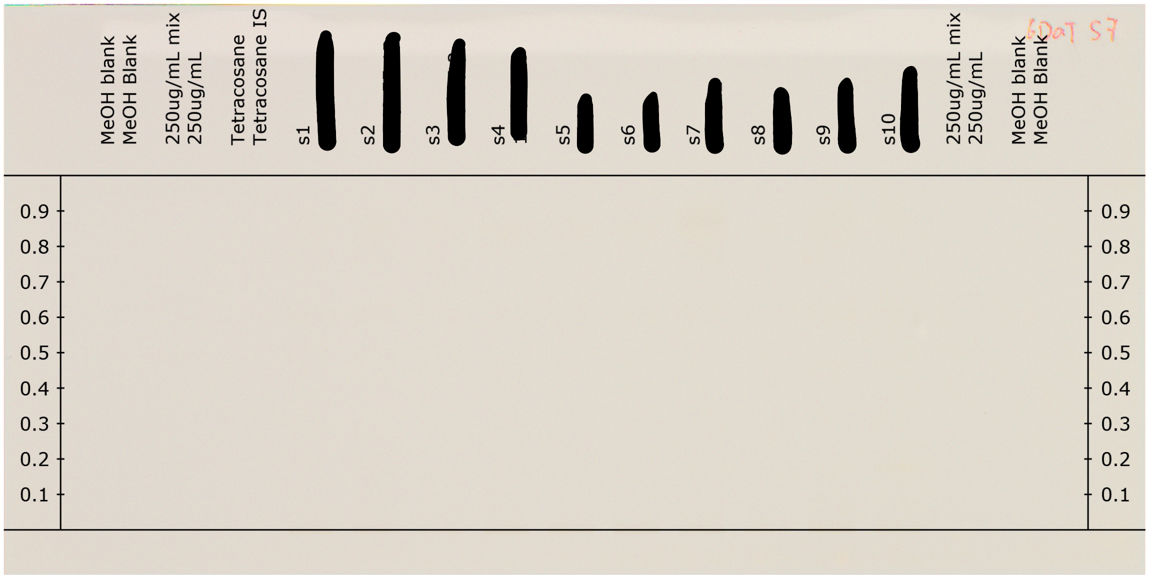

Exposure	0.086 s
Contrast	1
Normalized exposure	Disabled
Clarify	Disabled
White balance	1.00, 1.00, 1.00

R 254

Developed, Remission254

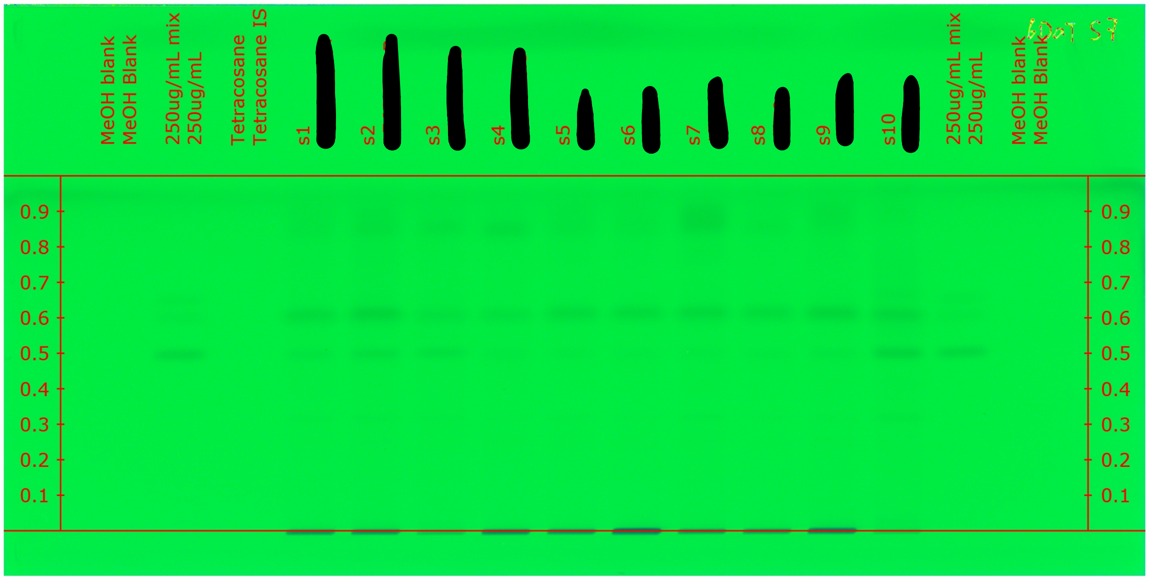

Exposure	0.273 s
Contrast	1
Normalized exposure	Disabled
Clarify	Disabled
White balance	1.00, 1.00, 1.00

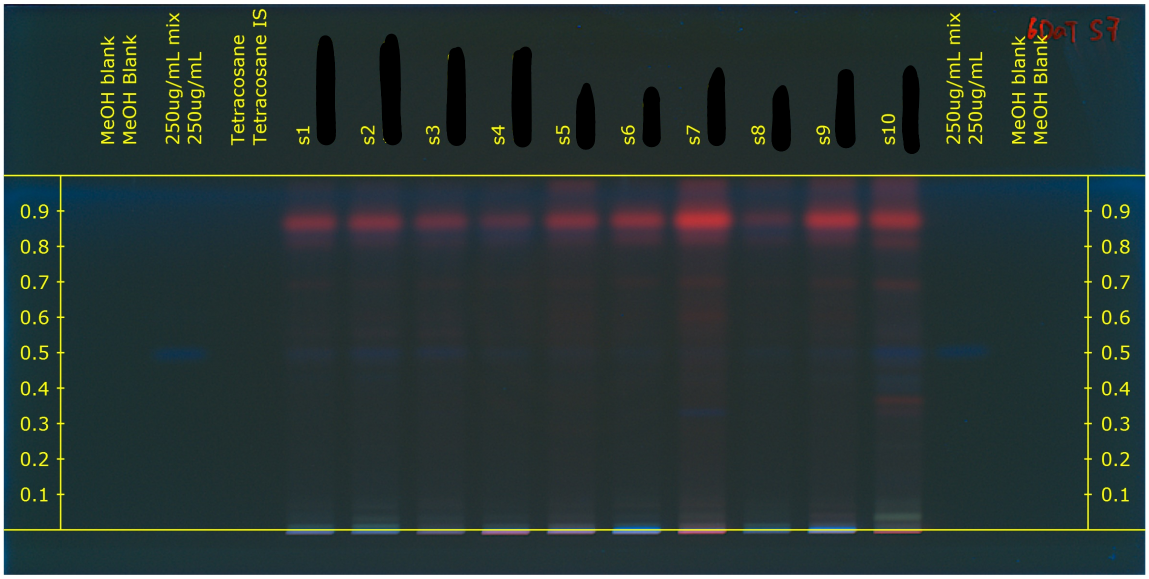

Exposure	2.707 s
Contrast	1
Normalized exposure	Disabled
Clarify	Disabled
White balance	1.00, 1.00, 1.00

Scan developed plate 1b - Scanner 3 (S/N: 031025):

Executed	14-Oct-2019 20:00:23 visionCATSuser

Scan:

Wavelength	254 nm

Track 1:

Type	Single λ

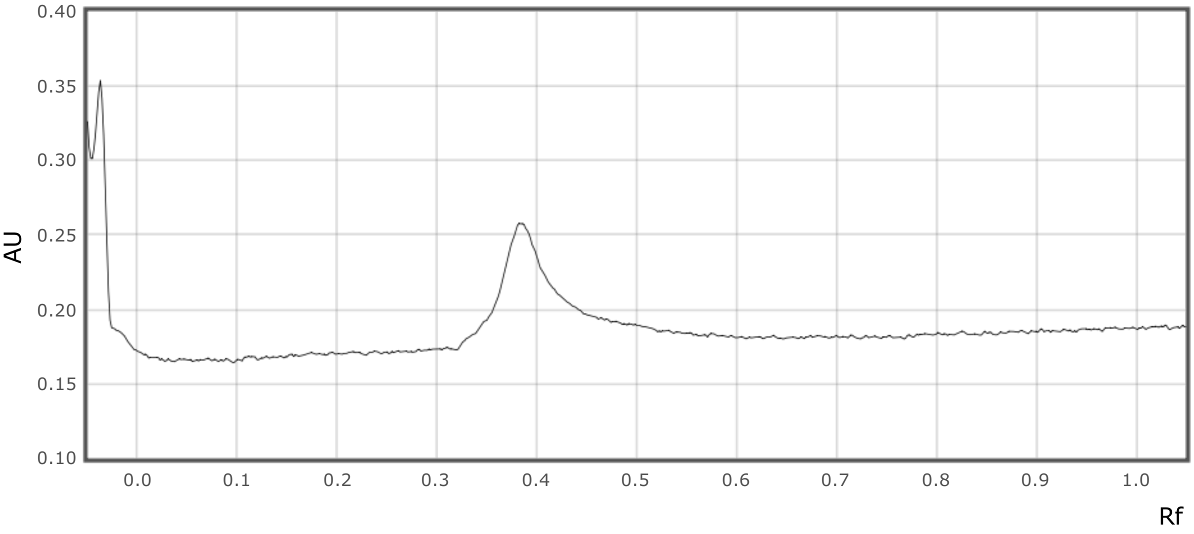

Track 2:

Type	Single λ

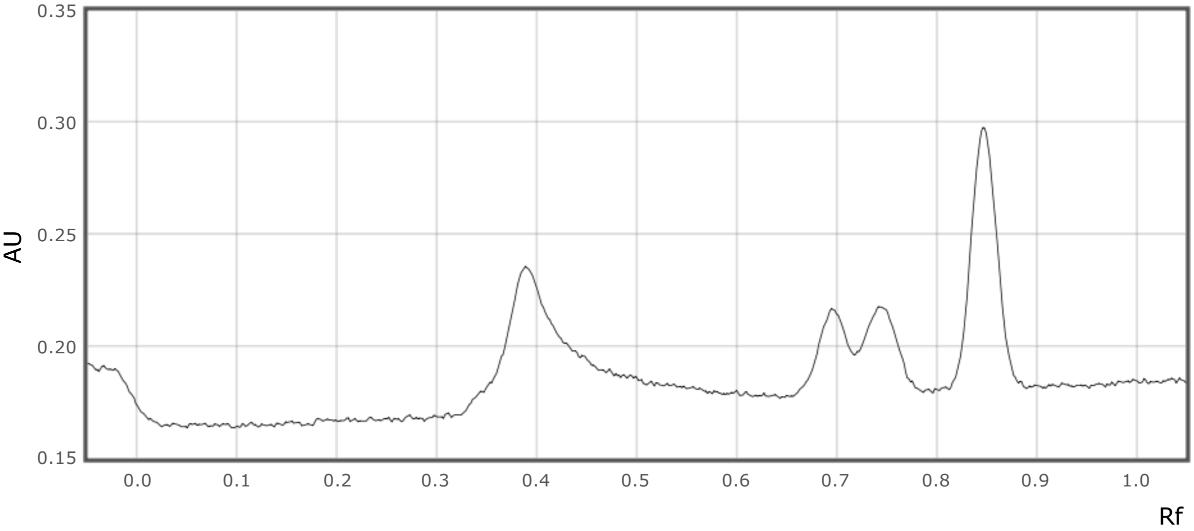

Track 3:

Type	Single λ

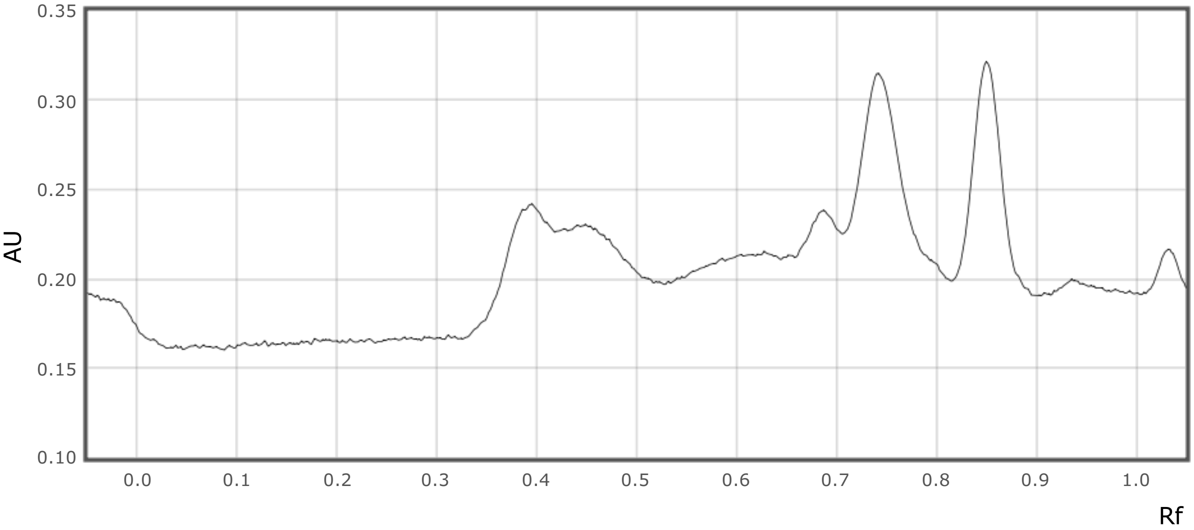

Track 4:

Type	Single λ

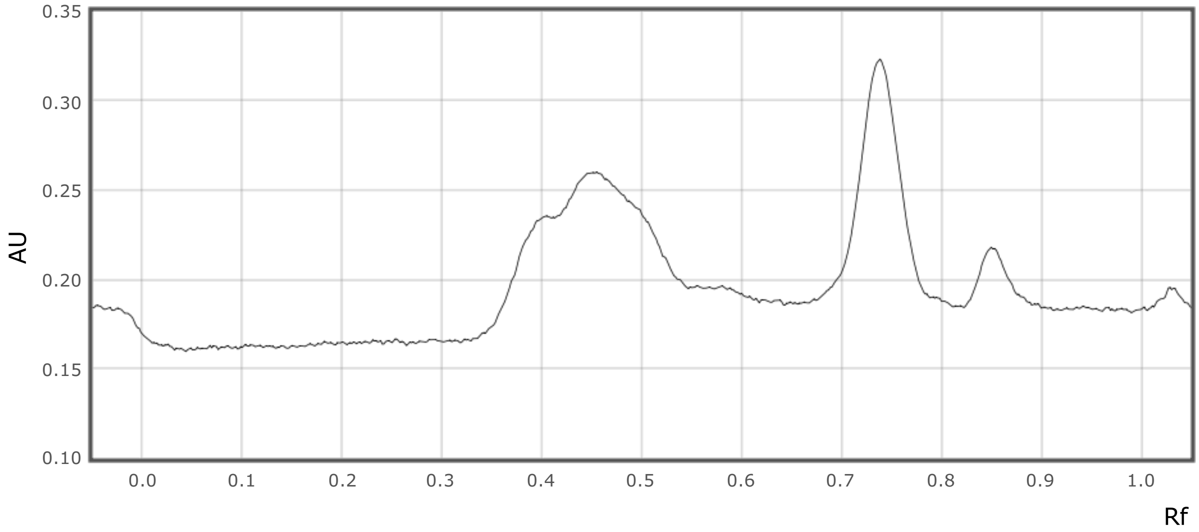

Track 5:	
Type	Single λ

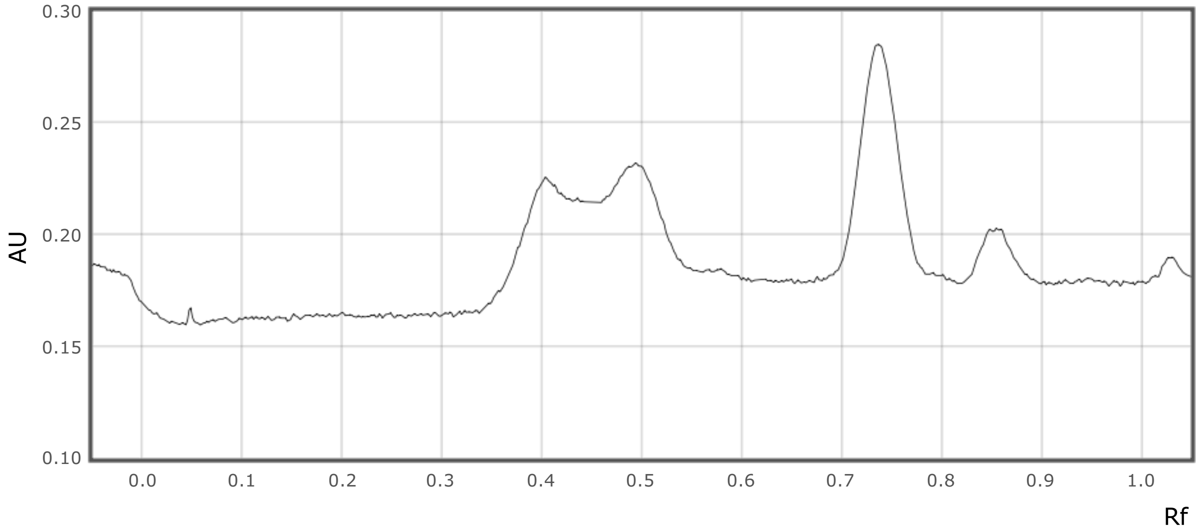

Track 6:	
Type	Single λ

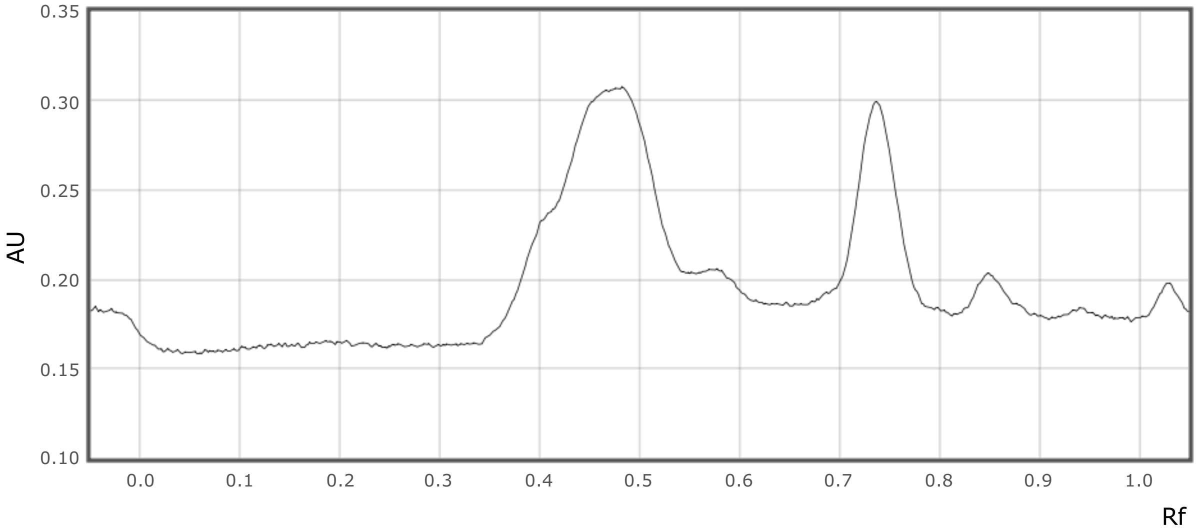

Track 7:	
Type	Single λ

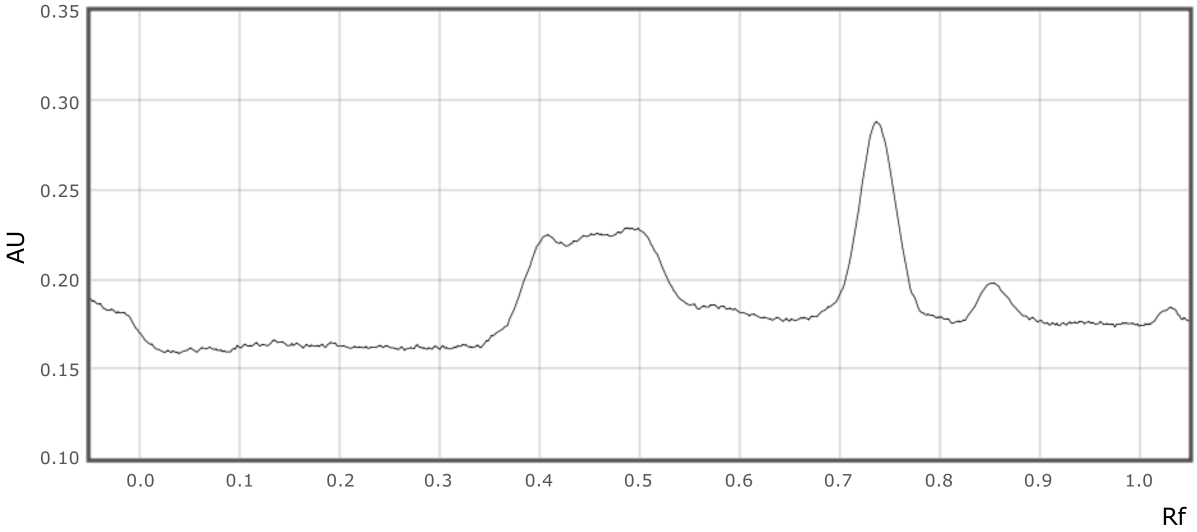

Track 8:	
Type	Single λ

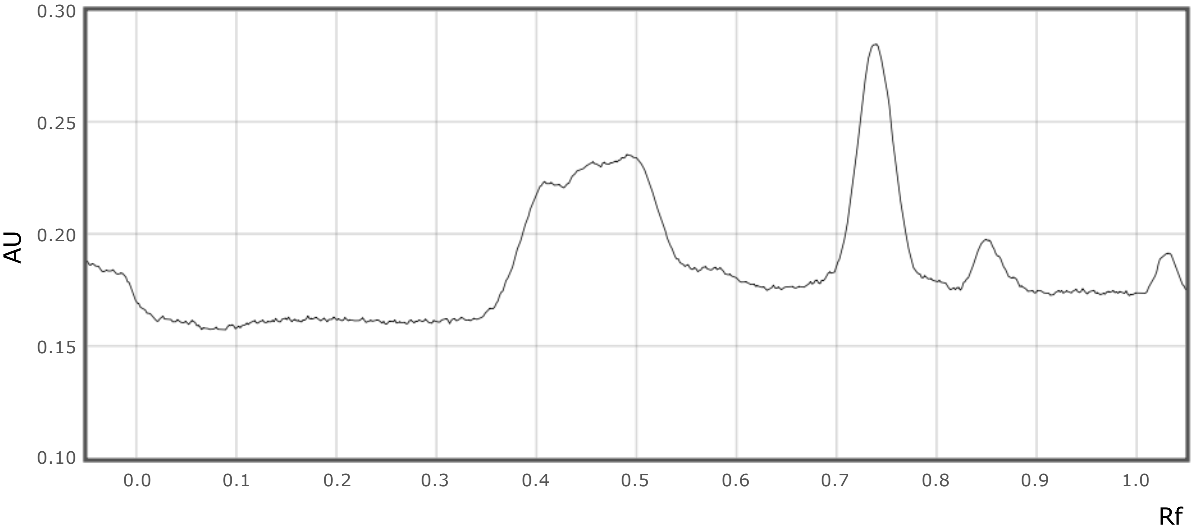

Track 9:	
Type	Single λ

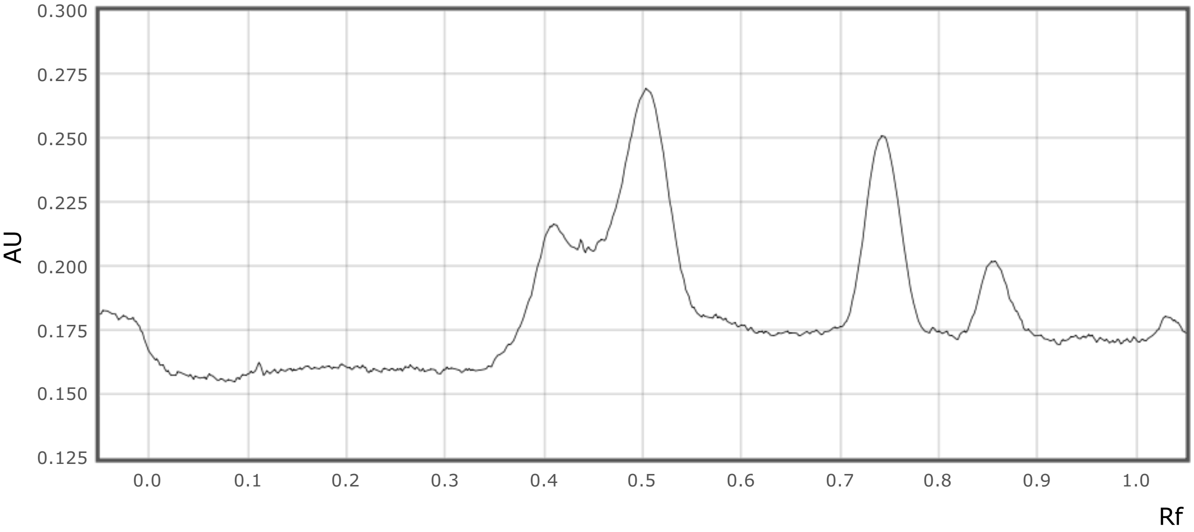

Track 10:	
Type	Single λ

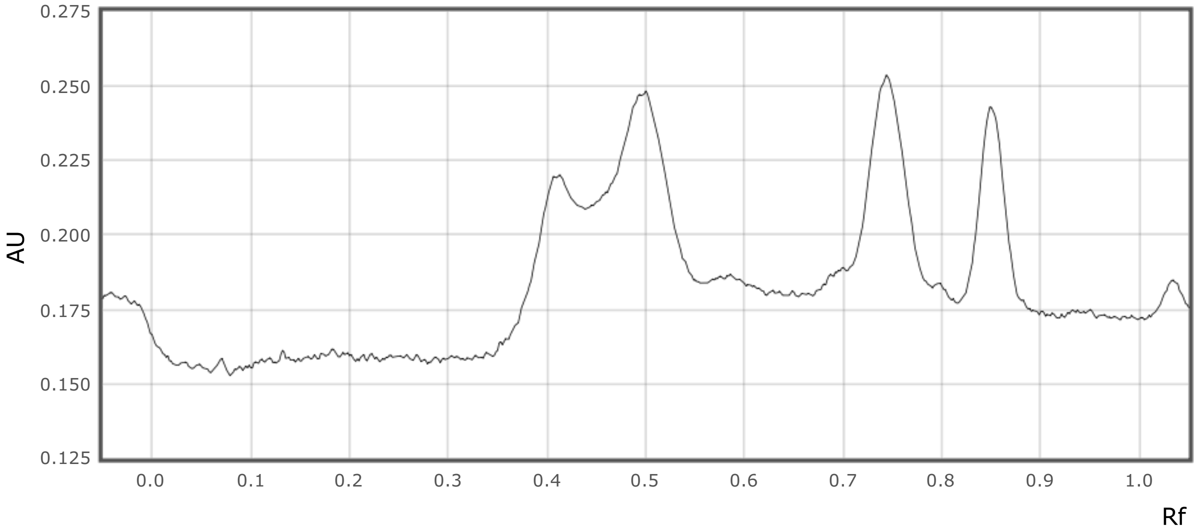

Track 11:	
Type	Single λ

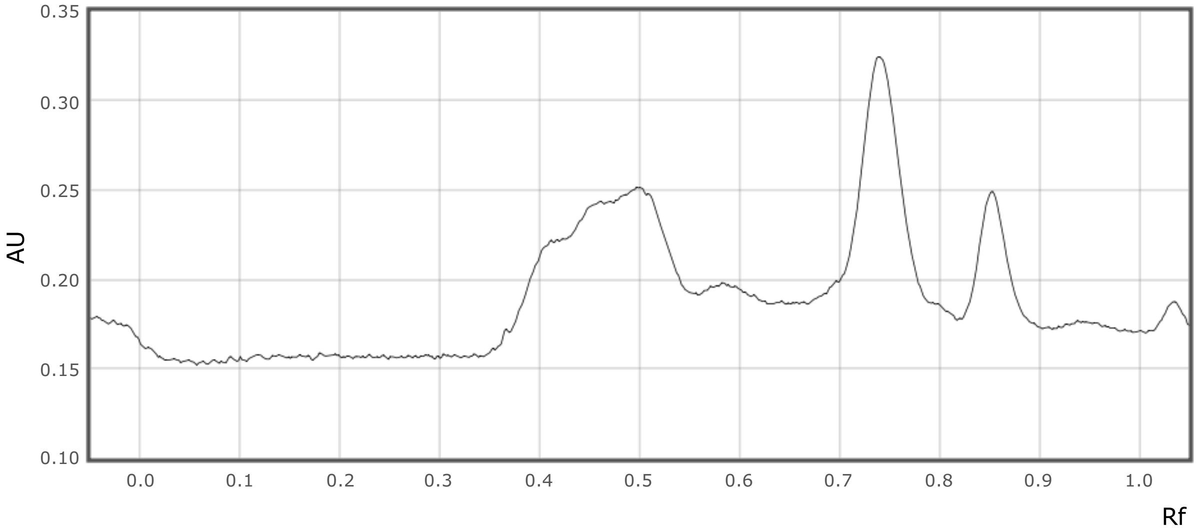

Track 12:	
Type	Single λ

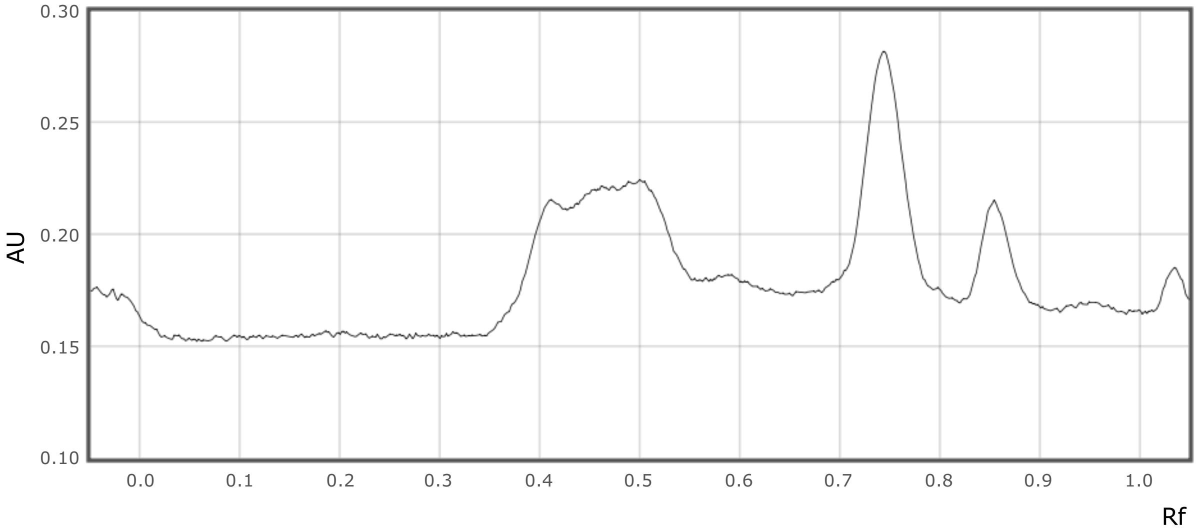

Track 13:	
Type	Single λ

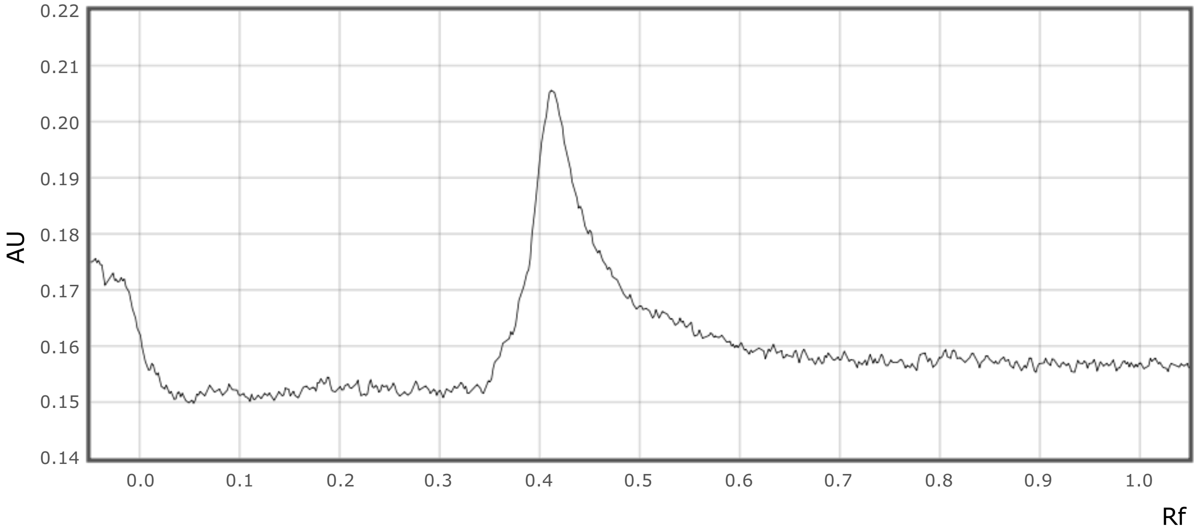

Track 14:	
Type	Single λ

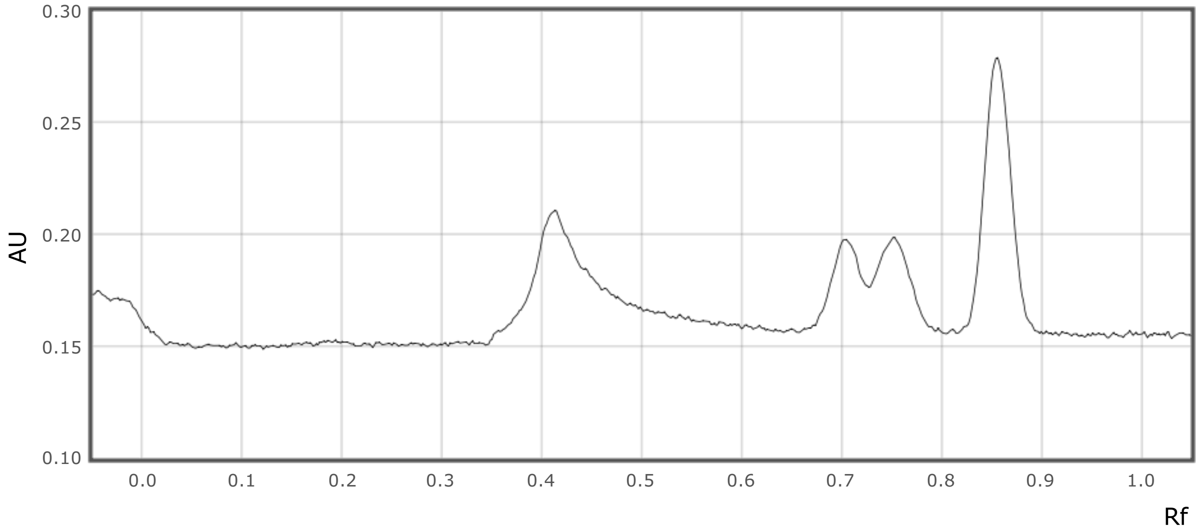

Track 15:

Type	Single λ

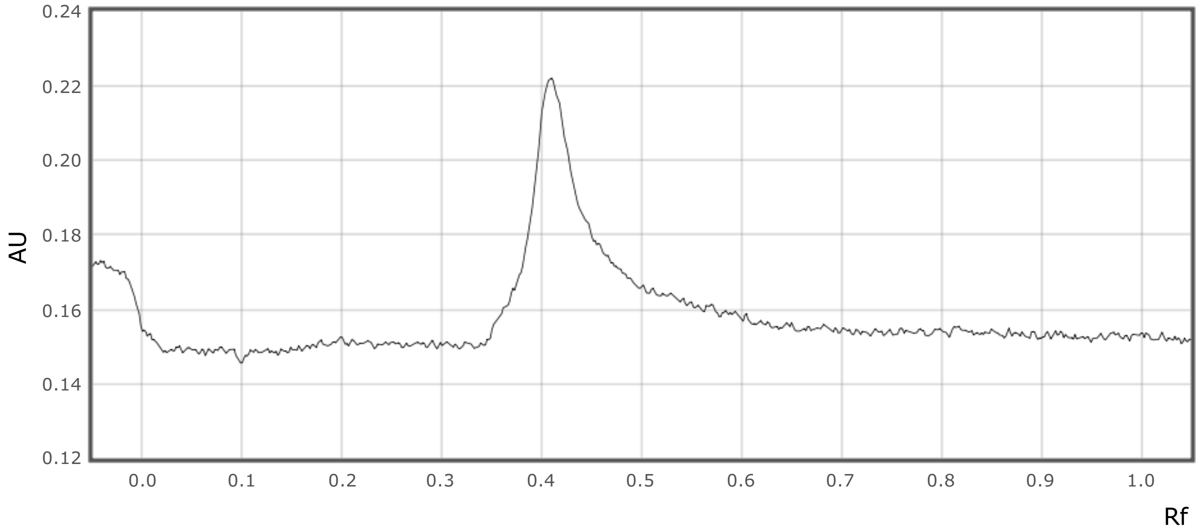

Derivatization 1 - dip:

Executed	14-Oct-2019 20:18:46 visionCATSuser

Take image derivatized plate 1a - Visualizer (S/N: 230515):

Executed	14-Oct-2019 20:21:35 visionCATSuser

RT White

Derivatized, RemTransVis

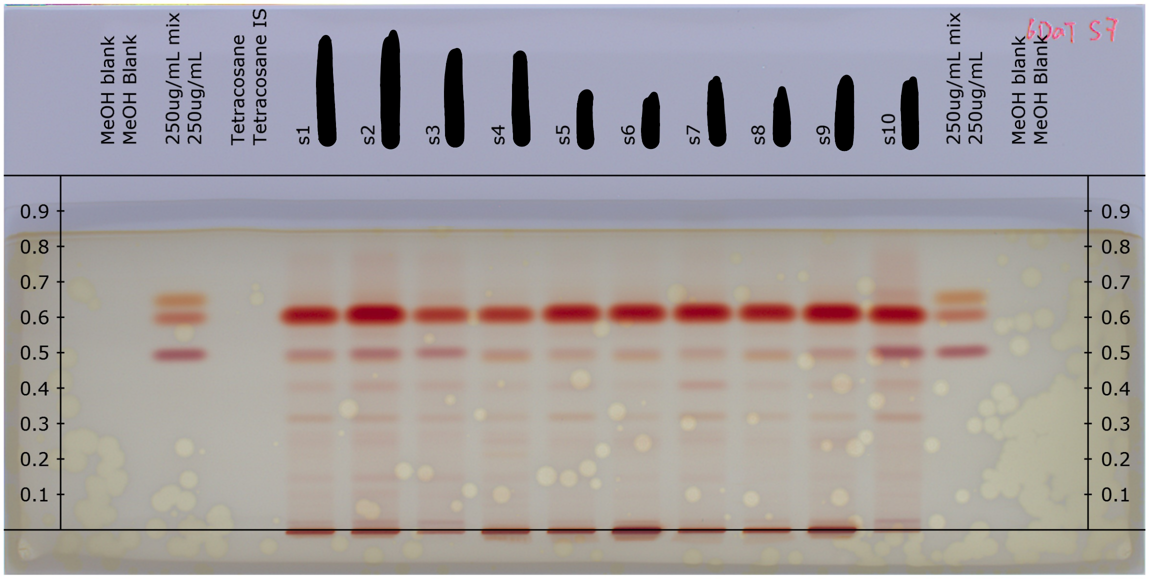

Exposure	0.053 s
Contrast	1
Normalized exposure	Disabled
Clarify	Disabled
White balance	1.17, 1.10, 0.81

R 366

Derivatized, Remission366

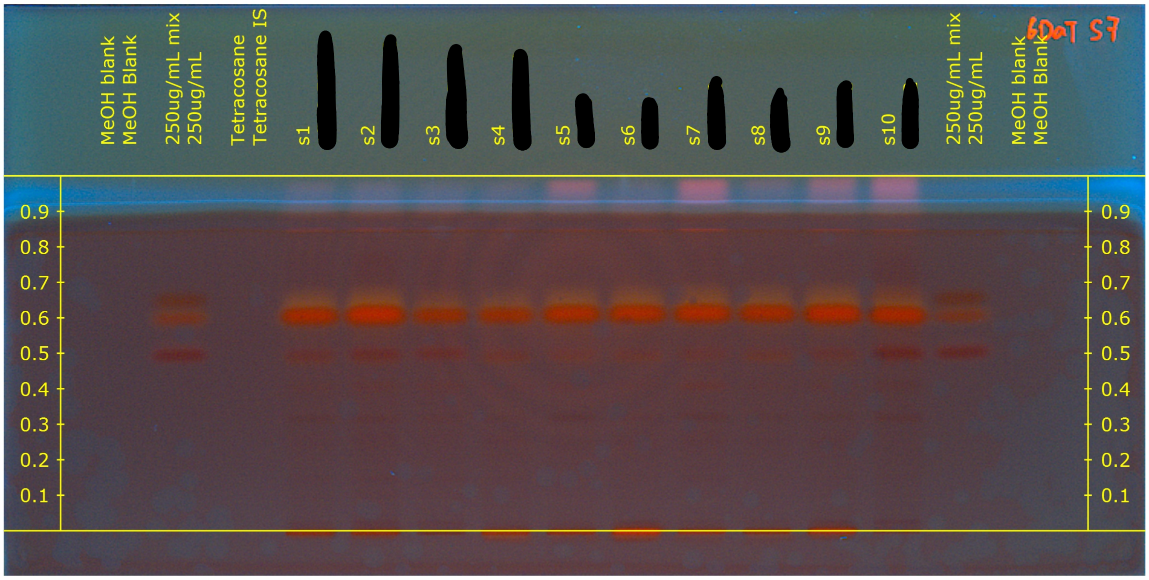

Exposure	9.999 s
Contrast	1
Normalized exposure	Disabled
Clarify	Disabled
White balance	1.00, 1.00, 1.00

Evaluation 1 :

Validated	false
Step	Take image derivatized plate 1a
Concentration unit type	Mass / volume
Notes	

Definition:

References:

250ug/mL mix

Substance Name	Concentration	Purity
9-THC	250.000 µg/ml	100.00 %
CBD	250.000 µg/ml	100.00 %
CBN	250.000 µg/ml	100.00 %

Samples:

Vial ID	Amount	Volume solution	Reference amount	Related to
MeOH blank		0.00 ml		
Tetracosane		0.00 ml		
s1		0.00 ml		
s2		0.00 ml		
s3		0.00 ml		
s4		0.00 ml		
s5		0.00 ml		
s6		0.00 ml		
s7		0.00 ml		
s8		0.00 ml		
s9		0.00 ml		
s10		0.00 ml		

Integration parameters:

Bounds	[0.000,1.000]
Smoothing	Savitzky–Golay of order 3 and window 7
Baseline correction	Lowest slope with noise 0.05
Profile subtraction	Profile subtraction from track 1
Peaks detection	Gauss (legacy) with sensitivity 0.1, separation 1 and threshold 0.1

Scan:

Wavelength	RT White

Track 1:

Type	Sample
Vial ID	MeOH blank
Description	MeOH Blank
Volume	2.0 µl

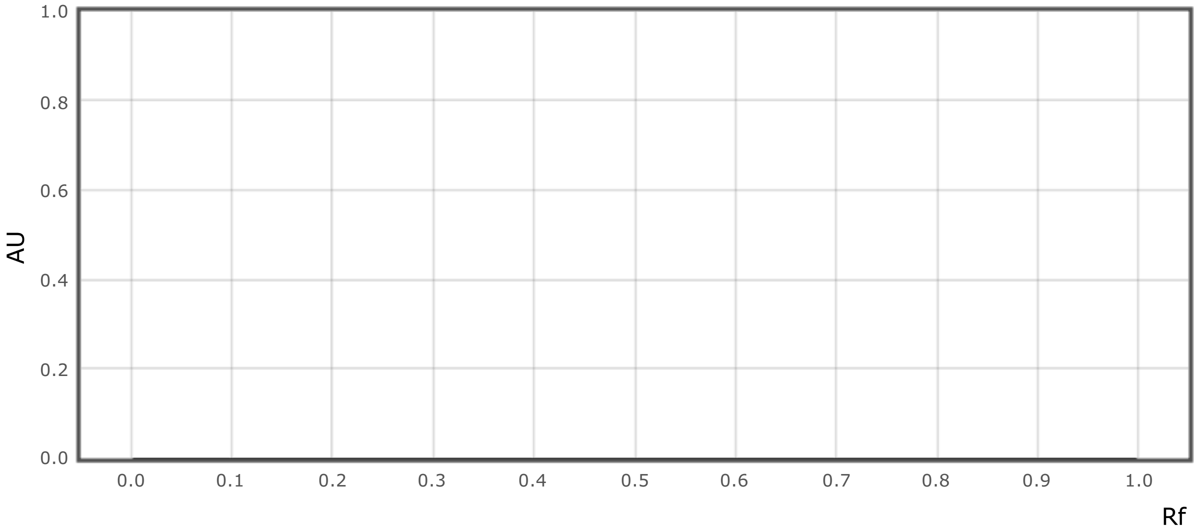

Peak #	Start		Max			End		Area		Manual peak	Substance Name
	Rf	H	Rf	H	%	Rf	H	A	%		

Track 2:

Type	Reference
Vial ID	250ug/mL mix
Description	250ug/mL
Volume	2.0 µl

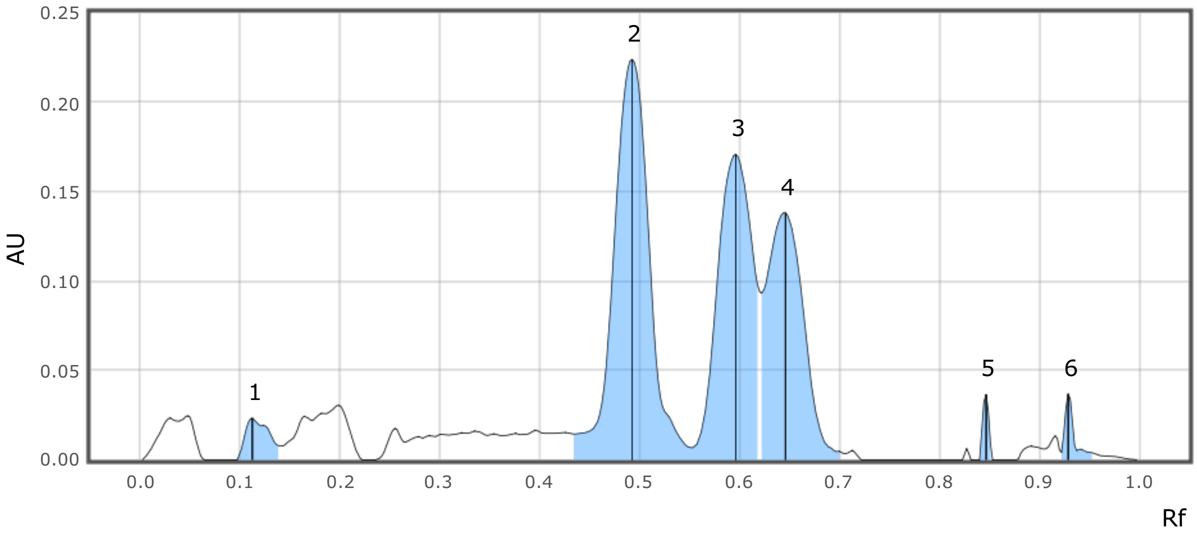

Peak #	Start		Max			End		Area		Manual peak	Substance Name
	Rf	H	Rf	H	%	Rf	H	A	%		
1	0.097	0.0000	0.112	0.0234	3.71	0.140	0.0078	0.00065	2.79	No	
2	0.434	0.0144	0.492	0.2239	35.56	0.551	0.0068	0.00925	39.66	No	CBN
3	0.553	0.0067	0.596	0.1708	27.12	0.620	0.0941	0.00688	29.50	No	9-THC
4	0.622	0.0931	0.646	0.1382	21.95	0.704	0.0036	0.00586	25.11	No	CBD
5	0.840	0.0000	0.847	0.0365	5.80	0.853	0.0000	0.00026	1.13	No	
6	0.922	0.0042	0.929	0.0369	5.86	0.961	0.0024	0.00042	1.81	No	

Track 3:

Type	Sample
Vial ID	Tetracosane
Description	Tetracosane IS
Volume	2.0 µl

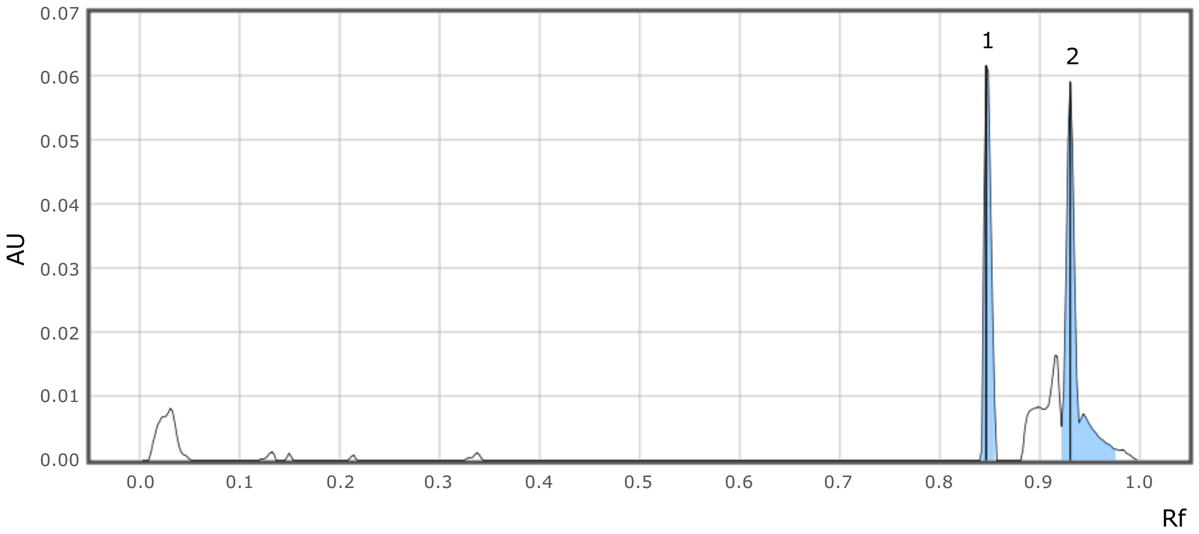

Peak #	Start Rf	Start H	Max Rf	Max H	Max %	End Rf	End H	Area A	Area %	Manual peak	Substance Name
1	0.840	0.0000	0.847	0.0617	51.06	0.858	0.0000	0.00051	42.40	No	
2	0.922	0.0053	0.931	0.0591	48.94	0.981	0.0015	0.00069	57.60	No	

Track 4:

Type	Sample
Vial ID	s1
Description	[redacted]
Volume	2.0 µl

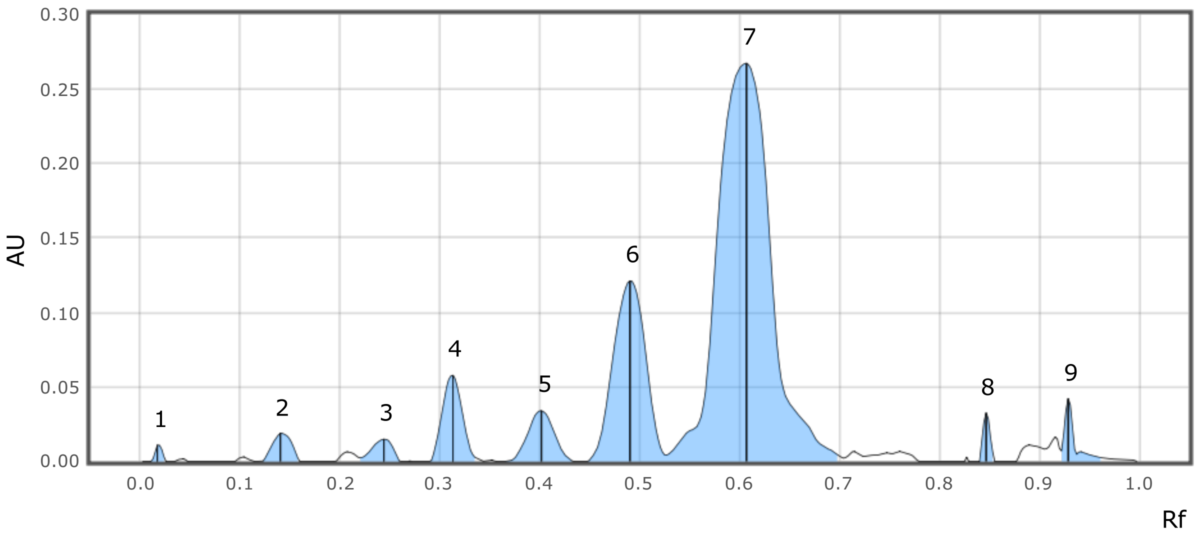

Peak #	Start		Max			End		Area		Manual peak	Substance Name
	Rf	H	Rf	H	%	Rf	H	A	%		
1	0.011	0.0000	0.017	0.0109	1.82	0.026	0.0000	0.00009	0.35	No	
2	0.121	0.0000	0.140	0.0188	3.13	0.160	0.0000	0.00041	1.66	No	
3	0.220	0.0024	0.244	0.0148	2.48	0.261	0.0000	0.00035	1.41	No	
4	0.289	0.0000	0.313	0.0577	9.65	0.343	0.0001	0.00133	5.33	No	
5	0.365	0.0000	0.402	0.0339	5.66	0.434	0.0000	0.00102	4.09	No	
6	0.447	0.0000	0.490	0.1210	20.22	0.527	0.0042	0.00453	18.13	No	
7	0.527	0.0042	0.607	0.2669	44.59	0.702	0.0028	0.01648	65.95	No	9-THC
8	0.840	0.0000	0.847	0.0325	5.43	0.856	0.0000	0.00027	1.07	No	
9	0.922	0.0071	0.929	0.0420	7.02	0.974	0.0015	0.00050	2.00	No	

Track 5:

Type	Sample
Vial ID	s2
Description	[redacted]
Volume	2.0 µl

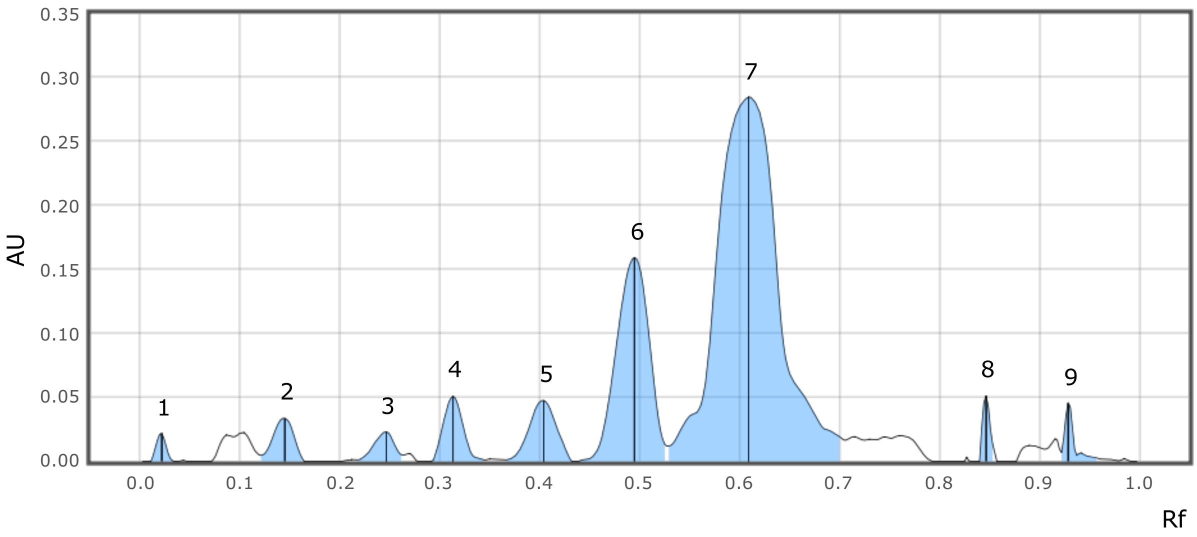

Peak #	Start		Max			End		Area		Manual peak	Substance Name
	Rf	H	Rf	H	%	Rf	H	A	%		
1	0.008	0.0000	0.021	0.0219	3.05	0.034	0.0000	0.00024	0.76	No	
2	0.121	0.0047	0.144	0.0335	4.67	0.164	0.0000	0.00081	2.54	No	
3	0.218	0.0012	0.246	0.0229	3.19	0.263	0.0047	0.00055	1.74	No	
4	0.291	0.0000	0.313	0.0508	7.08	0.345	0.0011	0.00115	3.61	No	
5	0.367	0.0012	0.404	0.0475	6.62	0.434	0.0000	0.00149	4.70	No	
6	0.438	0.0000	0.495	0.1593	22.20	0.527	0.0120	0.00605	19.02	No	
7	0.529	0.0119	0.609	0.2847	39.68	0.704	0.0167	0.02057	64.66	No	9-THC
8	0.840	0.0000	0.847	0.0512	7.13	0.858	0.0000	0.00046	1.44	No	
9	0.922	0.0069	0.929	0.0456	6.36	0.961	0.0018	0.00049	1.54	No	

Track 6:

Type	Sample
Vial ID	s3
Description	[redacted]
Volume	2.0 µl

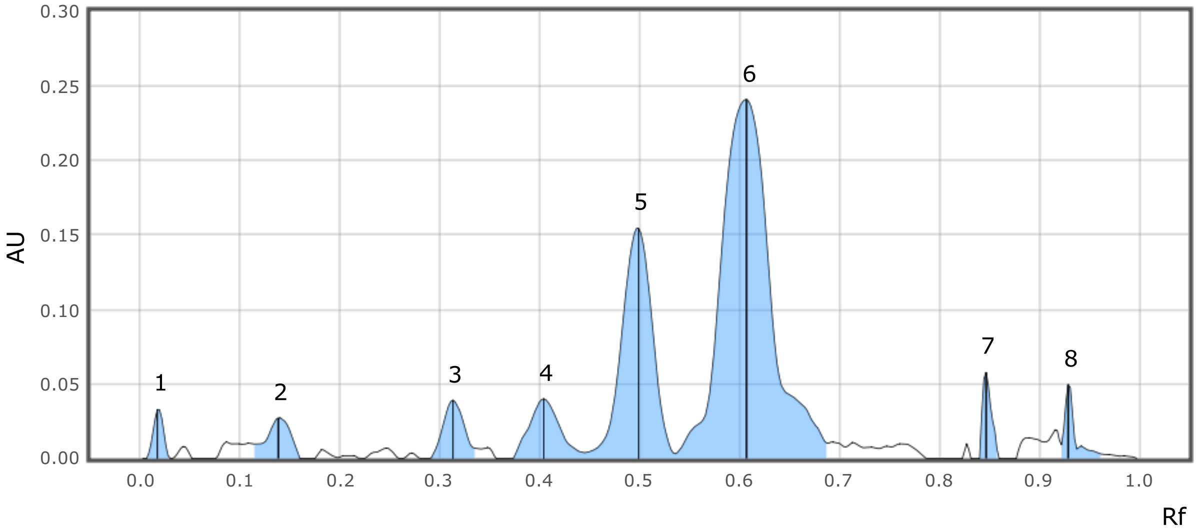

Peak #	Start		Max			End		Area		Manual peak	Substance Name
	Rf	H	Rf	H	%	Rf	H	A	%		
1	0.006	0.0000	0.017	0.0328	5.12	0.030	0.0000	0.00038	1.59	No	
2	0.114	0.0096	0.138	0.0271	4.23	0.162	0.0000	0.00074	3.08	No	
3	0.289	0.0000	0.313	0.0391	6.09	0.339	0.0065	0.00096	3.99	No	
4	0.371	0.0000	0.404	0.0400	6.23	0.445	0.0038	0.00140	5.83	No	
5	0.445	0.0038	0.499	0.1544	24.08	0.536	0.0031	0.00548	22.78	No	
6	0.536	0.0031	0.607	0.2406	37.53	0.689	0.0102	0.01388	57.76	No	9-THC
7	0.840	0.0000	0.847	0.0576	8.99	0.860	0.0000	0.00060	2.48	No	
8	0.922	0.0091	0.929	0.0496	7.73	0.968	0.0025	0.00060	2.49	No	

Track 7:

Type	Sample
Vial ID	s4
Description	[redacted]
Volume	2.0 µl

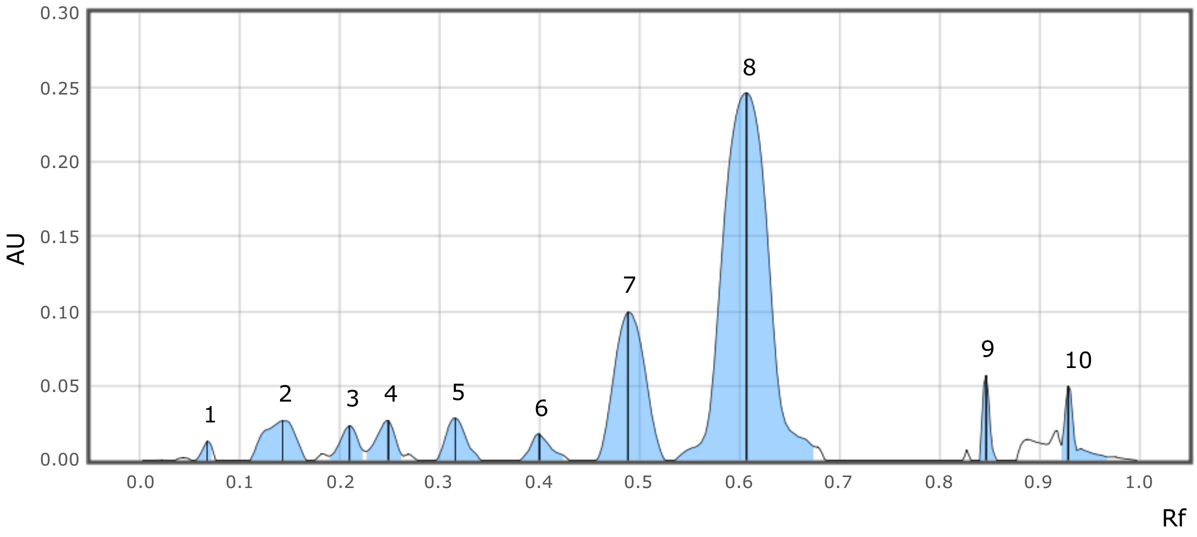

Peak #	Start		Max			End		Area		Manual peak	Substance Name
	Rf	H	Rf	H	%	Rf	H	A	%		
1	0.054	0.0000	0.067	0.0127	2.17	0.075	0.0000	0.00014	0.67	No	
2	0.110	0.0000	0.142	0.0264	4.49	0.166	0.0000	0.00096	4.65	No	
3	0.190	0.0028	0.209	0.0232	3.95	0.224	0.0061	0.00047	2.28	No	
4	0.227	0.0060	0.248	0.0267	4.54	0.263	0.0028	0.00057	2.78	No	
5	0.296	0.0000	0.315	0.0285	4.84	0.341	0.0000	0.00064	3.09	No	
6	0.380	0.0000	0.400	0.0177	3.02	0.430	0.0000	0.00040	1.95	No	
7	0.456	0.0000	0.488	0.0994	16.92	0.527	0.0000	0.00353	17.06	No	
8	0.533	0.0000	0.607	0.2462	41.90	0.676	0.0089	0.01289	62.32	No	9-THC
9	0.840	0.0000	0.847	0.0570	9.70	0.858	0.0000	0.00046	2.21	No	
10	0.922	0.0102	0.929	0.0498	8.48	0.974	0.0021	0.00062	2.99	No	

Track 8:

Type	Sample
Vial ID	s5
Description	[redacted]
Volume	2.0 µl

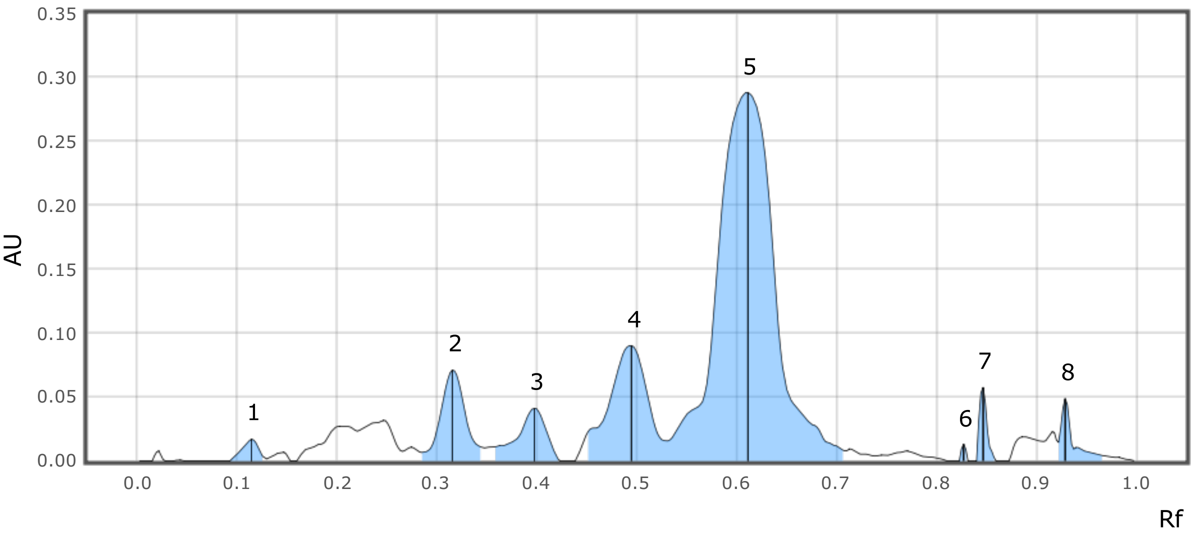

Peak #	Start		Max			End		Area		Manual peak	Substance Name
	Rf	H	Rf	H	%	Rf	H	A	%		
1	0.093	0.0000	0.114	0.0170	2.72	0.129	0.0015	0.00032	1.14	No	
2	0.285	0.0066	0.315	0.0710	11.33	0.348	0.0102	0.00204	7.26	No	
3	0.358	0.0109	0.397	0.0410	6.54	0.423	0.0000	0.00136	4.85	No	
4	0.451	0.0230	0.495	0.0901	14.38	0.529	0.0156	0.00397	14.12	No	
5	0.531	0.0156	0.611	0.2881	45.99	0.709	0.0079	0.01920	68.22	No	9-THC
6	0.823	0.0000	0.827	0.0131	2.08	0.832	0.0000	0.00007	0.24	No	
7	0.840	0.0000	0.847	0.0575	9.18	0.860	0.0000	0.00050	1.78	No	
8	0.922	0.0145	0.929	0.0487	7.78	0.968	0.0040	0.00067	2.38	No	

Track 9:

Type	Sample
Vial ID	s6
Description	[redacted]
Volume	2.0 µl

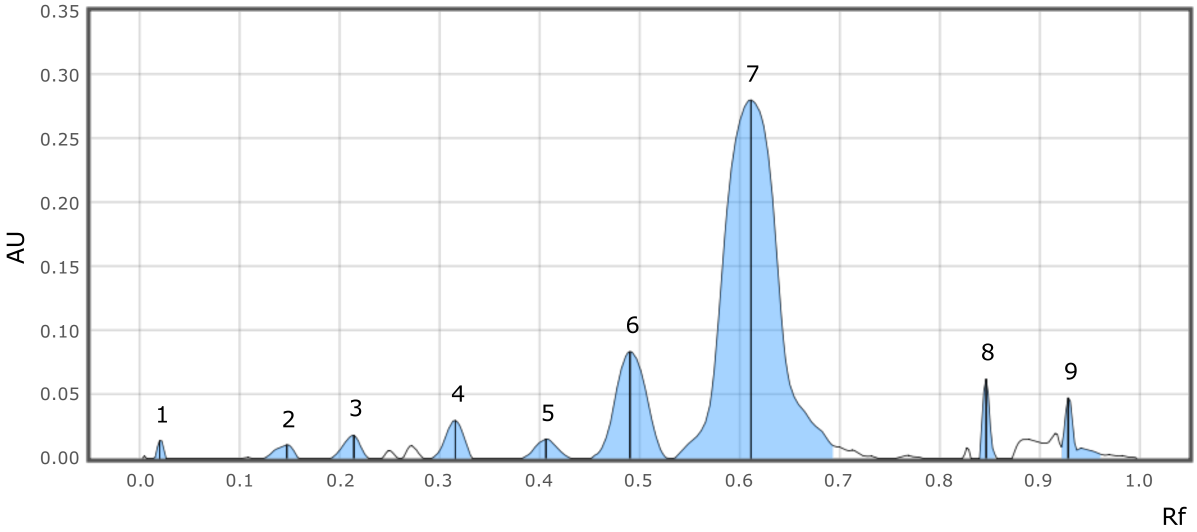

Peak #	Start Rf	Start H	Max Rf	Max H	Max %	End Rf	End H	Area A	Area %	Manual peak	Substance Name
1	0.013	0.0000	0.019	0.0139	2.47	0.028	0.0000	0.00009	0.41	No	
2	0.123	0.0000	0.147	0.0106	1.90	0.160	0.0000	0.00021	0.92	No	
3	0.190	0.0000	0.214	0.0182	3.25	0.229	0.0000	0.00035	1.49	No	
4	0.291	0.0000	0.315	0.0296	5.28	0.333	0.0000	0.00061	2.62	No	
5	0.382	0.0000	0.406	0.0152	2.71	0.432	0.0000	0.00037	1.60	No	
6	0.449	0.0000	0.490	0.0836	14.90	0.529	0.0000	0.00297	12.82	No	
7	0.533	0.0000	0.611	0.2803	49.98	0.698	0.0090	0.01752	75.54	No	9-THC
8	0.840	0.0000	0.847	0.0621	11.07	0.858	0.0000	0.00049	2.09	No	
9	0.922	0.0088	0.929	0.0474	8.46	0.966	0.0025	0.00058	2.52	No	

Track 10:

Type	Sample
Vial ID	s7
Description	[redacted]
Volume	2.0 µl

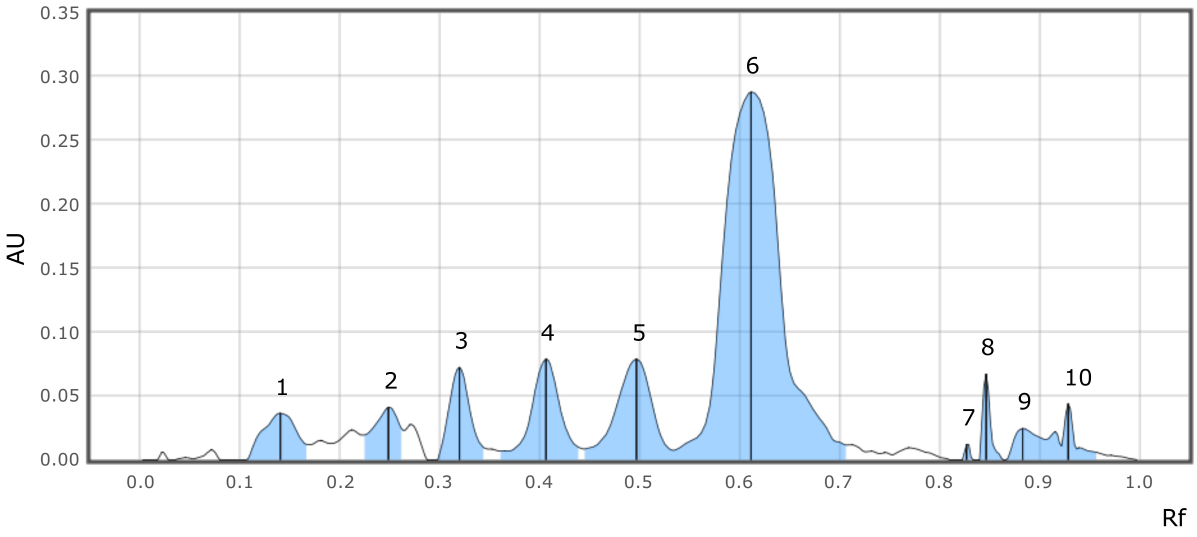

Peak #	Start Rf	Start H	Max Rf	Max H	Max %	End Rf	End H	Area A	Area %	Manual peak	Substance Name
1	0.106	0.0000	0.140	0.0365	4.91	0.170	0.0120	0.00147	4.63	No	
2	0.222	0.0194	0.248	0.0412	5.55	0.263	0.0230	0.00124	3.90	No	
3	0.296	0.0000	0.320	0.0725	9.75	0.348	0.0085	0.00177	5.57	No	
4	0.361	0.0068	0.406	0.0789	10.61	0.443	0.0087	0.00267	8.40	No	
5	0.445	0.0086	0.497	0.0788	10.60	0.533	0.0074	0.00330	10.38	No	
6	0.533	0.0074	0.611	0.2876	38.69	0.709	0.0115	0.01911	60.05	No	9-THC
7	0.823	0.0000	0.827	0.0121	1.63	0.834	0.0000	0.00007	0.22	No	
8	0.840	0.0000	0.847	0.0672	9.04	0.864	0.0000	0.00058	1.84	No	
9	0.866	0.0000	0.884	0.0245	3.30	0.922	0.0108	0.00099	3.10	No	
10	0.922	0.0108	0.929	0.0440	5.92	0.968	0.0034	0.00061	1.90	No	

Track 11:

Type	Sample
Vial ID	s8
Description	[redacted]
Volume	2.0 µl

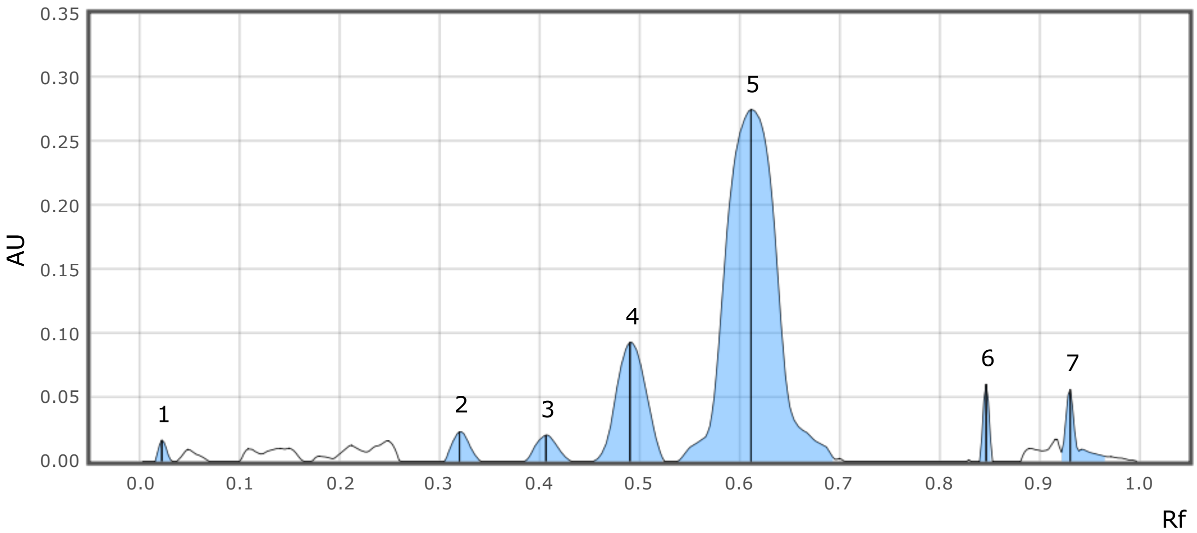

Peak #	Start Rf	Start H	Max Rf	Max H	Max %	End Rf	End H	Area A	Area %	Manual peak	Substance Name
1	0.015	0.0000	0.021	0.0166	3.05	0.032	0.0000	0.00015	0.68	No	
2	0.304	0.0000	0.320	0.0232	4.26	0.341	0.0000	0.00045	2.10	No	
3	0.384	0.0000	0.406	0.0204	3.76	0.432	0.0000	0.00050	2.32	No	
4	0.454	0.0000	0.490	0.0929	17.07	0.525	0.0000	0.00317	14.70	No	
5	0.538	0.0000	0.611	0.2748	50.48	0.696	0.0016	0.01615	75.02	No	9-THC
6	0.840	0.0000	0.847	0.0602	11.06	0.853	0.0000	0.00041	1.92	No	
7	0.922	0.0071	0.931	0.0562	10.32	0.968	0.0036	0.00070	3.27	No	

Track 12:

Type	Sample
Vial ID	s9
Description	[redacted]
Volume	2.0 µl

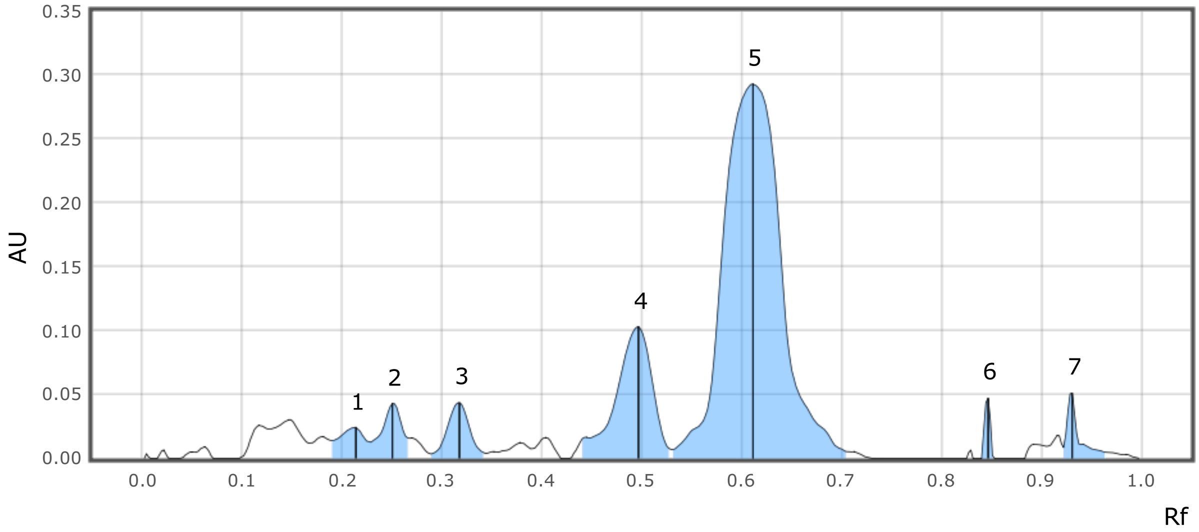

Peak #	Start		Max			End		Area		Manual peak	Substance Name
	Rf	H	Rf	H	%	Rf	H	A	%		
1	0.190	0.0139	0.214	0.0244	4.03	0.229	0.0126	0.00072	2.59	No	
2	0.229	0.0126	0.250	0.0429	7.10	0.268	0.0157	0.00102	3.64	No	
3	0.289	0.0035	0.317	0.0436	7.21	0.343	0.0040	0.00113	4.04	No	
4	0.441	0.0155	0.497	0.1030	17.03	0.529	0.0071	0.00431	15.40	No	
5	0.531	0.0068	0.611	0.2927	48.40	0.711	0.0052	0.01977	70.68	No	9-THC
6	0.840	0.0000	0.847	0.0471	7.78	0.853	0.0000	0.00031	1.11	No	
7	0.922	0.0085	0.931	0.0511	8.45	0.968	0.0045	0.00071	2.54	No	

Track 13:

Type	Sample
Vial ID	s10
Description	[redacted]
Volume	2.0 µl

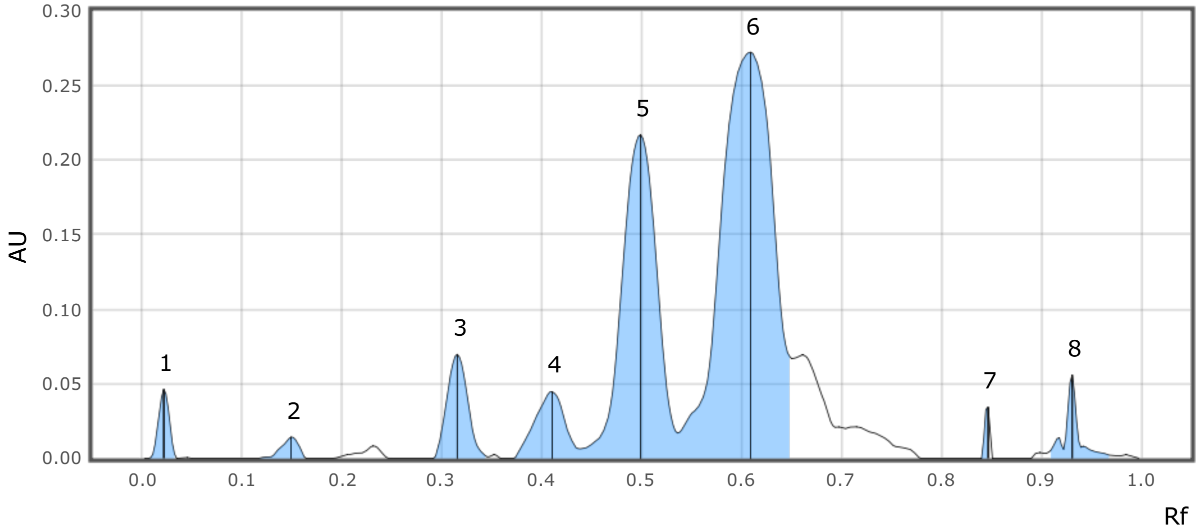

Peak #	Start Rf	Start H	Max Rf	Max H	Max %	End Rf	End H	Area A	Area %	Manual peak	Substance Name
1	0.006	0.0000	0.021	0.0463	6.14	0.034	0.0000	0.00056	1.84	No	
2	0.114	0.0000	0.149	0.0142	1.88	0.164	0.0000	0.00028	0.92	No	
3	0.291	0.0000	0.315	0.0697	9.25	0.345	0.0010	0.00167	5.45	No	
4	0.371	0.0000	0.410	0.0446	5.92	0.438	0.0063	0.00154	5.05	No	
5	0.438	0.0063	0.499	0.2168	28.76	0.536	0.0169	0.00876	28.70	No	
6	0.536	0.0169	0.609	0.2720	36.09	0.650	0.0667	0.01671	54.73	No	9-THC
7	0.840	0.0000	0.847	0.0343	4.55	0.851	0.0000	0.00021	0.69	No	
8	0.910	0.0052	0.931	0.0559	7.42	0.977	0.0017	0.00080	2.62	No	

Track 14:

Type	Reference
Vial ID	250ug/mL mix
Description	250ug/mL
Volume	2.0 µl

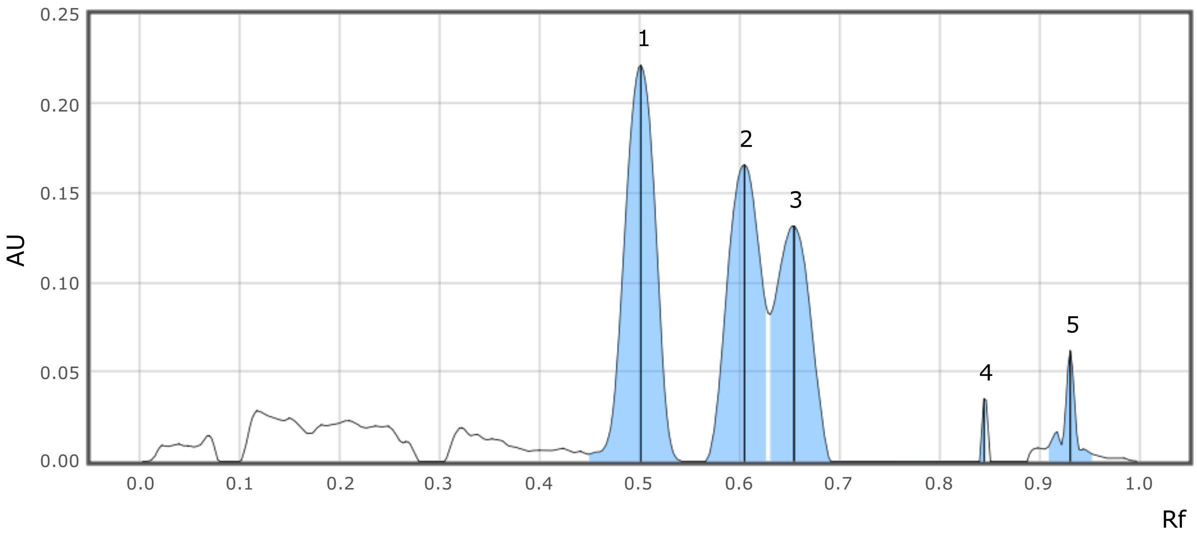

Peak #	Start Rf	Start H	Max Rf	Max H	Max %	End Rf	End H	Area A	Area %	Manual peak	Substance Name
1	0.449	0.0040	0.501	0.2214	35.97	0.544	0.0000	0.00786	38.44	No	CBN
2	0.566	0.0000	0.605	0.1657	26.91	0.629	0.0828	0.00631	30.85	No	9-THC
3	0.631	0.0822	0.655	0.1316	21.38	0.693	0.0000	0.00518	25.31	No	CBD
4	0.840	0.0000	0.845	0.0350	5.68	0.851	0.0000	0.00023	1.11	No	
5	0.910	0.0083	0.931	0.0619	10.06	0.968	0.0018	0.00087	4.28	No	

Track 15:

Type	Sample
Vial ID	MeOH blank
Description	MeOH Blank
Volume	2.0 µl

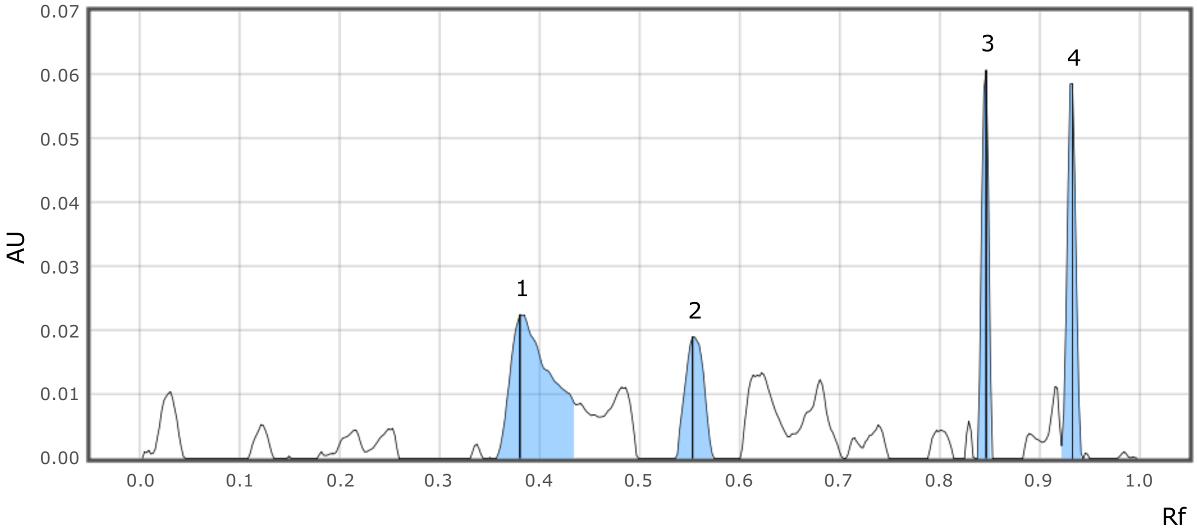

Peak #	Start		Max			End		Area		Manual peak	Substance Name
	Rf	H	Rf	H	%	Rf	H	A	%		
1	0.356	0.0000	0.380	0.0225	13.99	0.436	0.0084	0.00108	42.09	No	
2	0.536	0.0000	0.553	0.0190	11.82	0.575	0.0000	0.00041	16.04	No	
3	0.838	0.0000	0.847	0.0607	37.75	0.853	0.0000	0.00049	19.21	No	
4	0.922	0.0020	0.933	0.0586	36.44	0.944	0.0000	0.00058	22.66	No	

Calibration results:

Height calibration for substance 9-THC @ RT White:

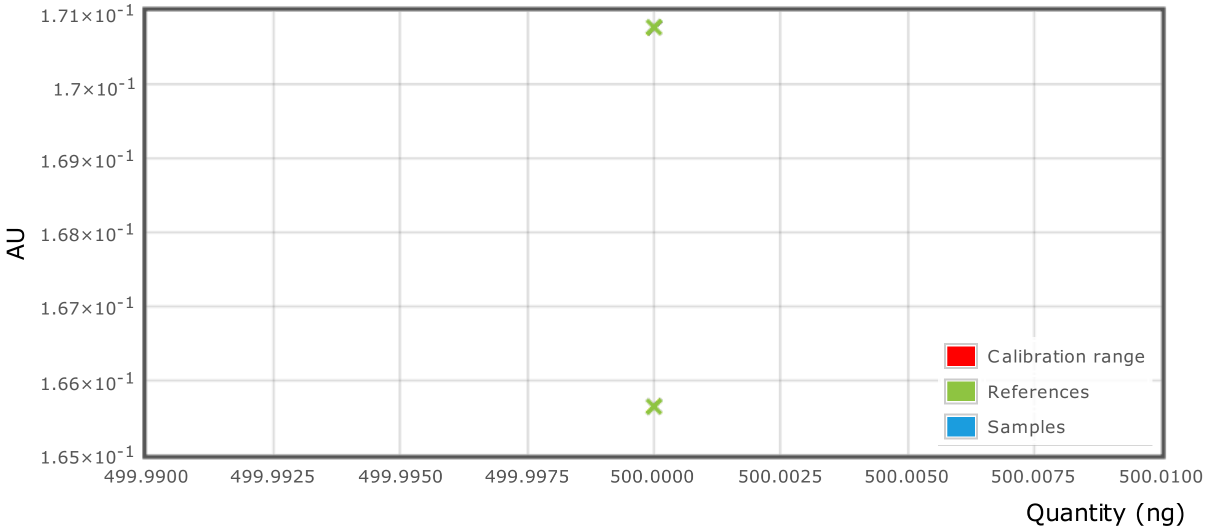

Regression mode	Linear-2
Range deviation	5.00 %
Related substances	Default
Number of references	2
Calibration function	$y=0x$
Coefficient of variation	CV 0.00 %
Correlation coefficient	n/a
⚠	Unable to compute the results for this substance because there wasn't eno ugh groups of references replicas (at least 1 for Linear-1, 2 for Linear2 and Mime-1 and 3 for Polynomial and MiMe-2)

Height calibration for substance CBD @ RT White:

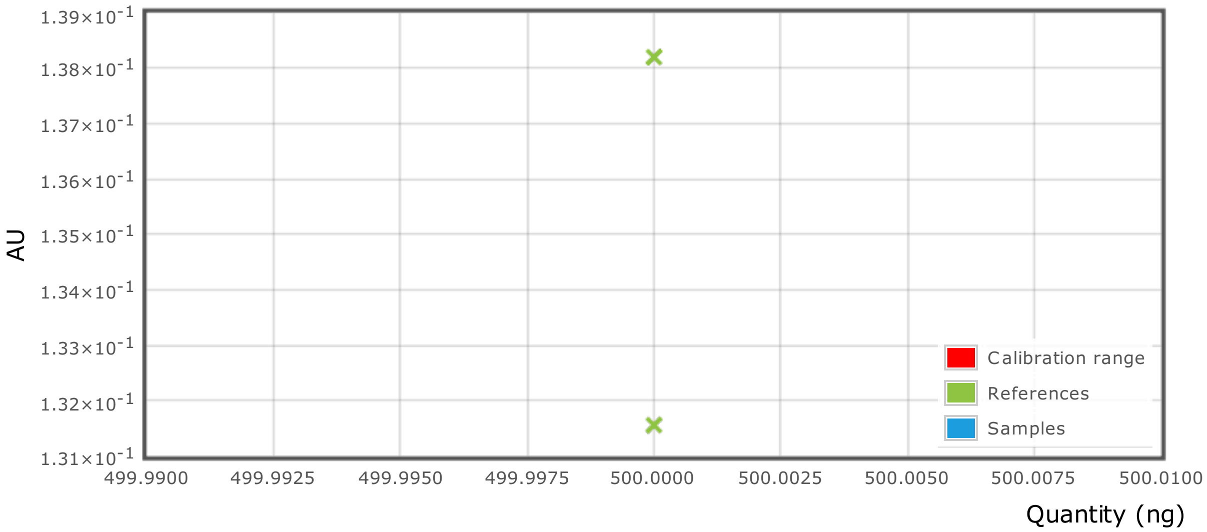

Regression mode	Linear-2
Range deviation	5.00 %
Related substances	Default
Number of references	2
Calibration function	$y=0x$
Coefficient of variation	CV 0.00 %
Correlation coefficient	n/a
⚠	Unable to compute the results for this substance because there wasn't eno ugh groups of references replicas (at least 1 for Linear-1, 2 for Linear2 and Mime-1 and 3 for Polynomial and MiMe-2)

Height calibration for substance CBN @ RT White:

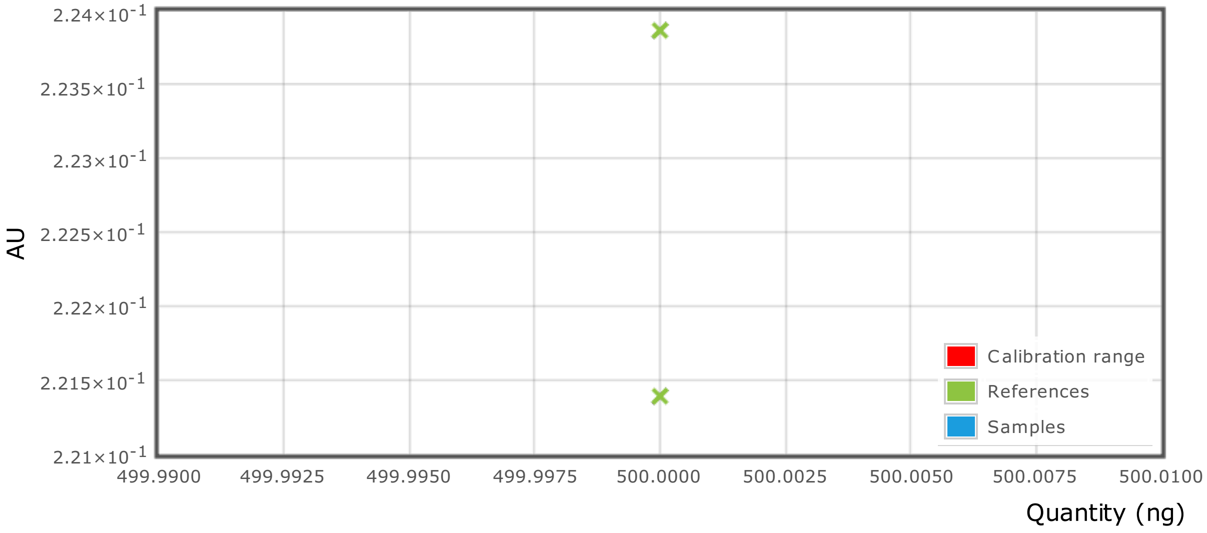

Regression mode	Linear-2
Range deviation	5.00 %
Related substances	Default
Number of references	2
Calibration function	$y=0x$
Coefficient of variation	CV 0.00 %
Correlation coefficient	n/a
⚠	Unable to compute the results for this substance because there wasn't enough groups of references replicas (at least 1 for Linear-1, 2 for Linear2 and Mime-1 and 3 for Polynomial and MiMe-2)

Results:

Substance having no available results		
⚠	CBD	There wasn't any sample application available in the assignments for this substance. Please check that the peaks were correctly detected and assigned for this substance.
⚠	CBN	There wasn't any sample application available in the assignments for this substance. Please check that the peaks were correctly detected and assigned for this substance.
⚠	9-THC	Unable to compute the results for this substance because there wasn't enough groups of references replicas (at least 1 for Linear-1, 2 for Linear2 and Mime-1 and 3 for Polynomial and MiMe-2)

A track marked with ⚠ means: this result is outside the regression range given by the reference assignments, but is included in the results because it is in the allowed range deviation.

Analyst:	Reviewer:
