# Supplementary material for: High Performance Thin-Layer Chromatography (HPTLC) data of Cannabinoids in ten mobile phase systems
Source: Data Brief. 2020 Jun 30;31:105955. doi: 10.1016/j.dib.2020.105955 (PMC7352075; doi:10.1016/j.dib.2020.105955)
Supplement: Supplementary file 1 [file mmc1.zip › S4-Case sample reports/6DaT-sample run-8.pdf]

Analysis: 6DaT-sample run-8

Path: Home/YL Research

Based on method: Samples (no cal)

Created	15-Oct-2019 12:23:34	visionCATSuser
Modified	15-Oct-2019 14:09:21	visionCATSuser
Last HPTLC log	15-Oct-2019 14:09:21	Analysis modified
Explorer notes		

Track	Vial ID	Description	Volume	Position	Type
1	MeOH blank	MeOH Blank	2.0 µl	A1	Sample
2	250ug/mL mix	250ug/mL	2.0 µl	A2	Reference
3	Tetracosane	Tetracosane IS	2.0 µl	A3	Sample
4	s1	[redacted]	2.0 µl	B1	Sample
5	s2	[redacted]	2.0 µl	B2	Sample
6	s3	[redacted]	2.0 µl	B3	Sample
7	s4	[redacted]	2.0 µl	B4	Sample
8	s5	[redacted]	2.0 µl	B5	Sample
9	s6	[redacted]	2.0 µl	B6	Sample
10	s7	[redacted]	2.0 µl	B7	Sample
11	s8	[redacted]	2.0 µl	B8	Sample
12	s9	[redacted]	2.0 µl	B9	Sample
13	s10	[redacted]	2.0 µl	B10	Sample
14	250ug/mL mix	250ug/mL	2.0 µl	A2	Reference
15	MeOH blank	MeOH Blank	2.0 µl	A1	Sample
Sequence table notes					

A track marked with ⚠ means: the application type is overridden in some evaluation(s).

System setup:

Software	Server User-PC, version 2.5.18072.1
ATS4	S/N:080713
Chamber	N/A
Derivatization dip	N/A
Scanner3	S/N:031025
Visualizer	S/N:230515

Chromatography

Plate layout:

Stationary phase	Merck, HPTLC plates silica gel 60 F 254
Plate format	200.0 x 100.0 mm
Application type	Band
Application	Position Y: 8.0 mm, length: 8.0 mm, width: 0.0 mm
Track	First position X: 20.0 mm, distance: 11.4 mm
Solvent front position	70.0 mm
Notes	

Take image clean plate 1a - Visualizer (S/N: 230515):

Quality	Enhanced
RT White	auto capture, Auto, level 85 %, Band
R 254	auto capture, Auto, level 85 %, Band
Instrument diagnostics	Valid diagnostics
Documentation step label	
Notes	

Application 1 - ATS 4 (S/N: 080713):

Spray gas	NI
Sample solvent type	Methanol
Filling speed	15 µl/s
Predosage volume	200 nl
Retraction volume	200 nl
Dosage speed	150 nl/s
Filling quality	User
Rinsing cycles / vacuum	2 / 4 s
Filling cycles / vacuum	1 / 4 s
Rinsing solvent name	Methanol
Nozzle temperature	Unheated
Rack in use	Standard
Instrument diagnostics	Valid diagnostics
Notes	

Development 1 - Chamber:

Tank	TTC 20x10
Mobile phase	6% diethylamine in toluene
Saturation time	20 min
Use saturation pad	true
Use smartALERT	false
Volume front through	10 ml
Volume rear through	25 ml
Drying time	5 min
Drying temperature	Room temperature
Notes	

Take image developed plate 1a - Visualizer (S/N: 230515):

Quality	Enhanced
RT White	auto capture, Auto, level 85 %, Band
R 254	auto capture, Auto, level 85 %, Band
R 366	auto capture, Auto, level 85 %, Band
Instrument diagnostics	Valid diagnostics
Documentation step label	
Notes	

Scan developed plate 1b - Scanner 3 (S/N: 031025):

Scanner type	Single λ
Optimization for	Resolution
Measurement mode	Absorption
Filter	n/a
Detector mode	Automatic
Scanning speed	20 mm/s
Data resolution	100 µm/step
Slit	5 x 0.2 mm, micro
Partial scan	No
Lamp	Deuterium & Tungsten
Wavelength(s)	254 nm
Instrument diagnostics	Valid diagnostics
Documentation step label	
Notes	

Derivatization 1 - dip:

Reagent name	
Dipping speed	5
Dipping time	0 s
Reagent preparation	
Heating	100 °C for 3 min, heated after
Notes	

Take image derivatized plate 1a - Visualizer (S/N: 230515):

Quality	Enhanced
RT White	auto capture, Auto, level 85 %, Band
R 366	auto capture, Auto, level 85 %, Band
Instrument diagnostics	Valid diagnostics
Documentation step label	
Notes	

System suitability tests:

SST settings:

SST tracks	

Data acquisition

Application 1 - ATS 4 (S/N: 080713):

Executed	15-Oct-2019 12:28:32 visionCATSuser

Development 1 - Chamber:

Executed	15-Oct-2019 12:54:56 visionCATSuser

Take image developed plate 1a - Visualizer (S/N: 230515):

Executed	15-Oct-2019 13:52:46 visionCATSuser

RT White

Developed, RemTransVis

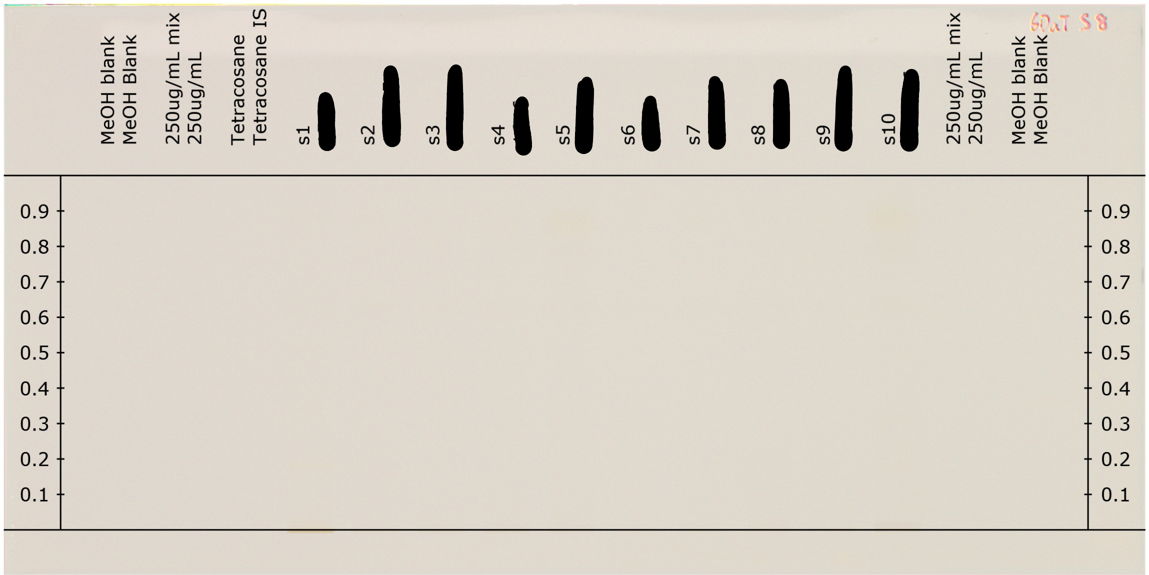

Exposure	0.086 s
Contrast	1
Normalized exposure	Disabled
Clarify	Disabled
White balance	1.00, 1.00, 1.00

R 254

Developed, Remission254

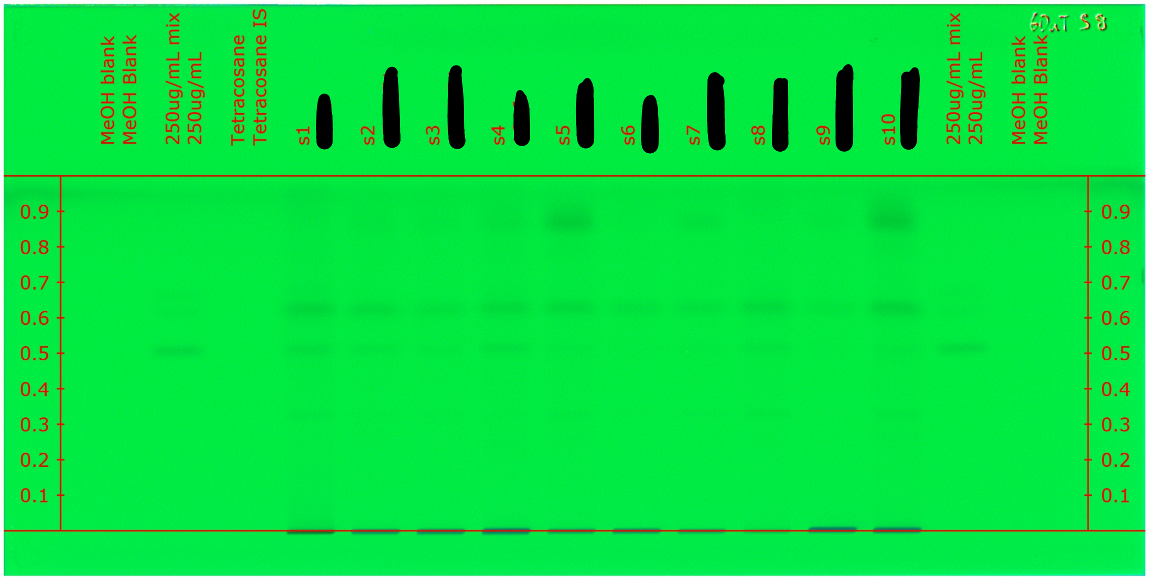

Exposure	0.272 s
Contrast	1
Normalized exposure	Disabled
Clarify	Disabled
White balance	1.00, 1.00, 1.00

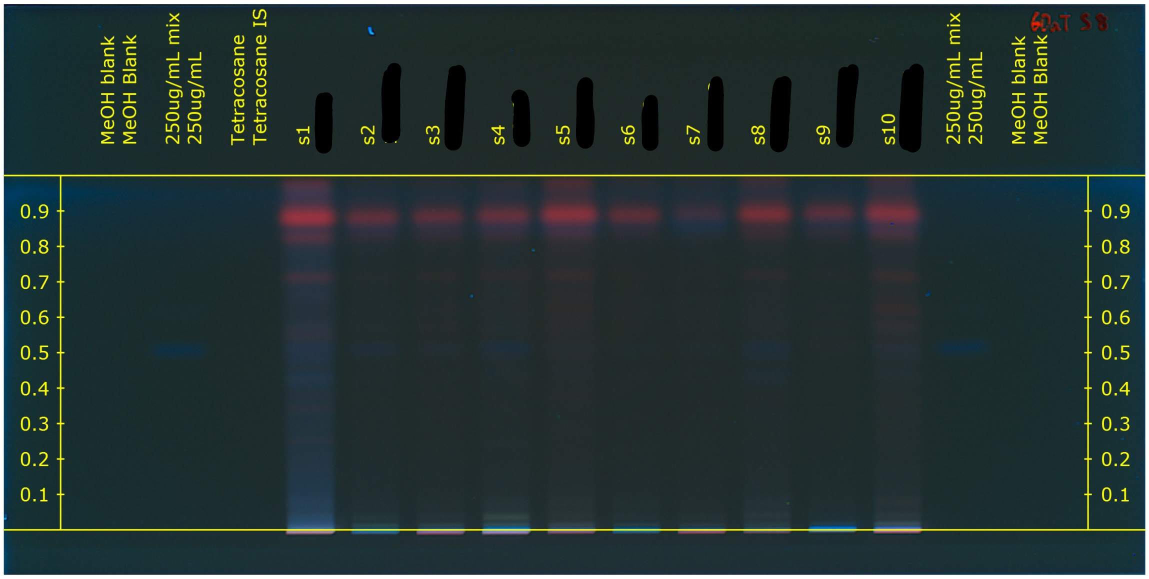

Exposure	2.329 s
Contrast	1
Normalized exposure	Disabled
Clarify	Disabled
White balance	1.00, 1.00, 1.00

Scan developed plate 1b - Scanner 3 (S/N: 031025):

Executed	15-Oct-2019 13:55:32 visionCATSuser

Scan:

Wavelength	254 nm

Track 1:

Type	Single λ

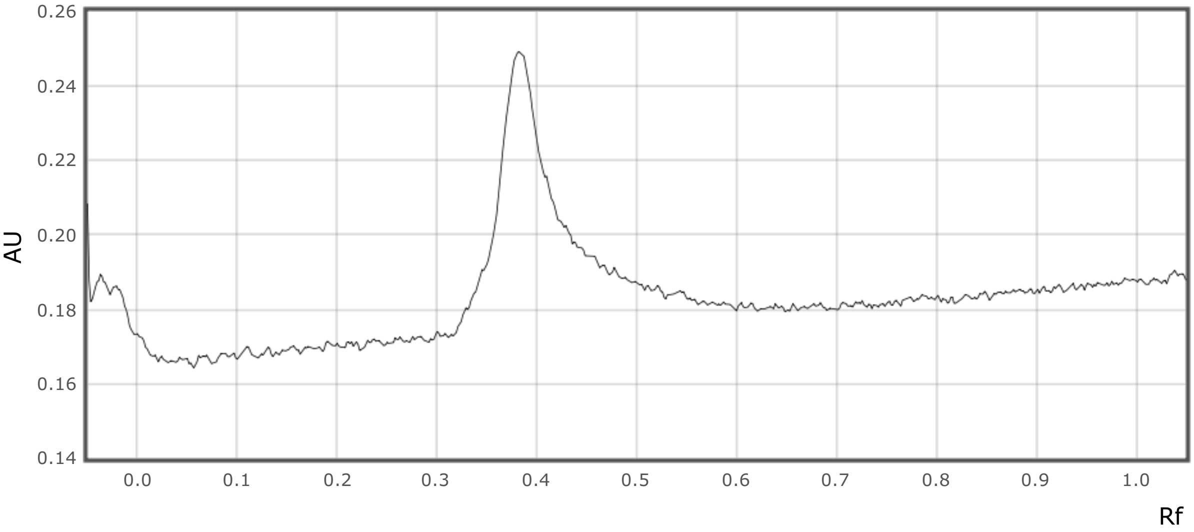

Track 2:	
Type	Single λ

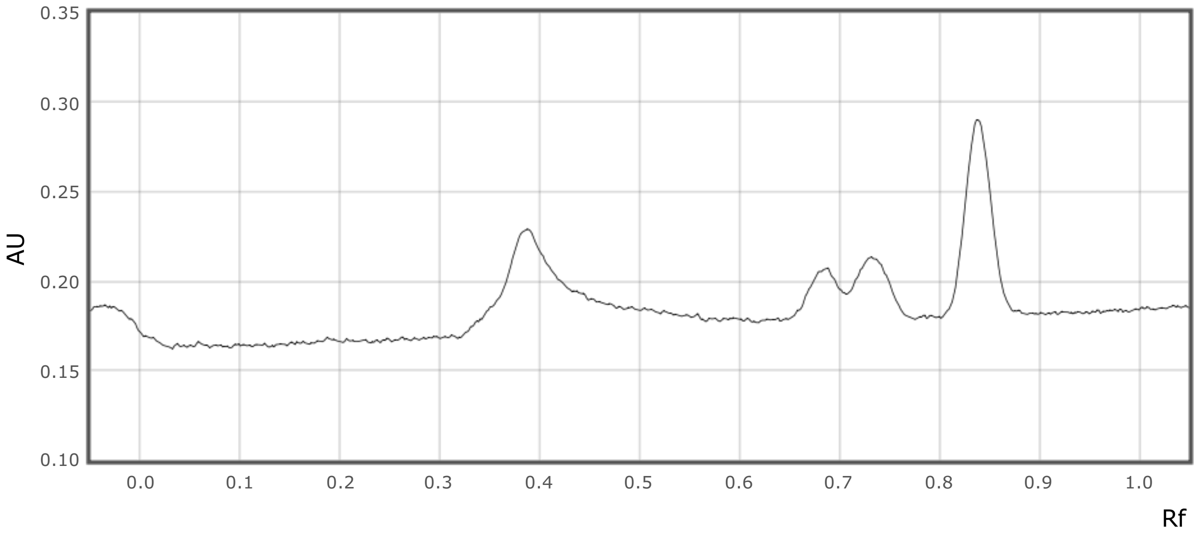

Track 3:	
Type	Single λ

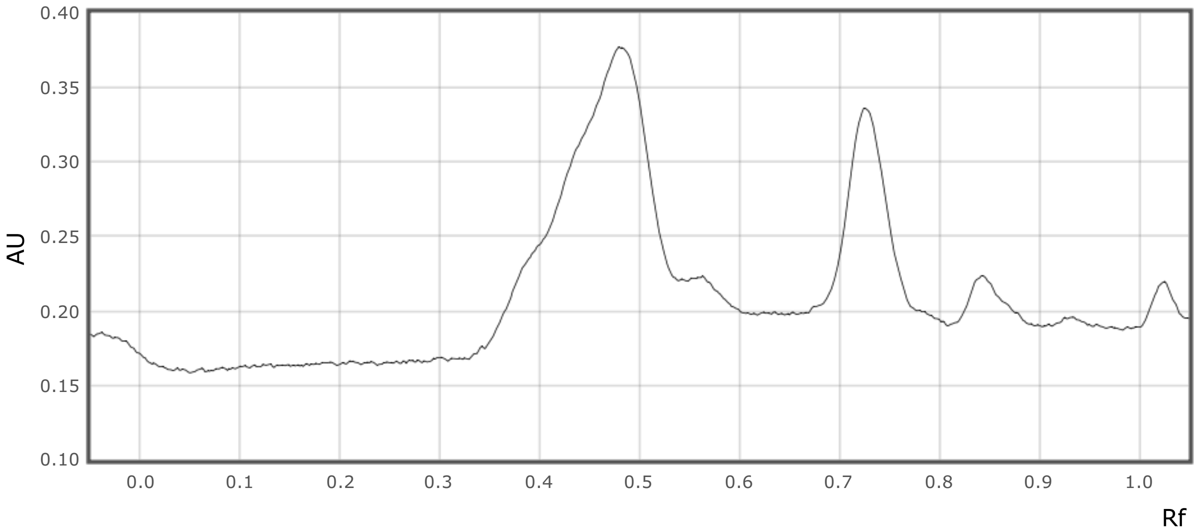

Track 4:	
Type	Single λ

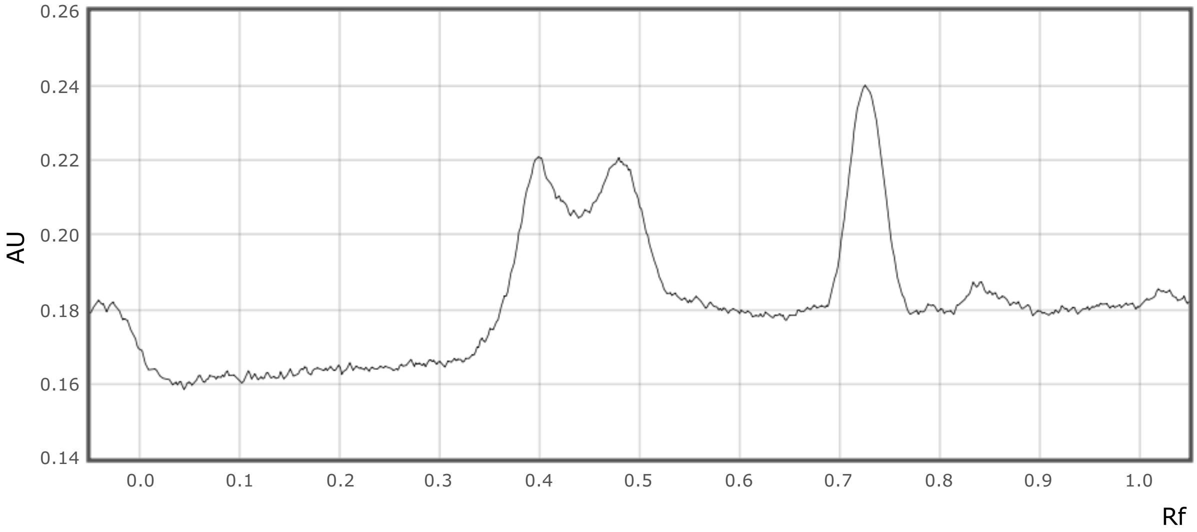

Track 5:

Type	Single λ

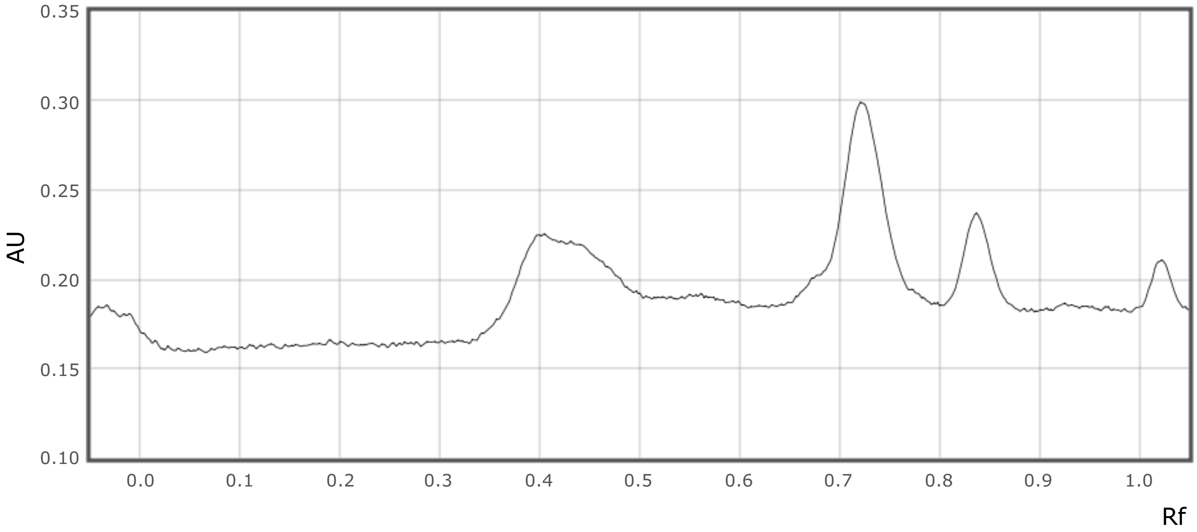

Track 6:

Type	Single λ

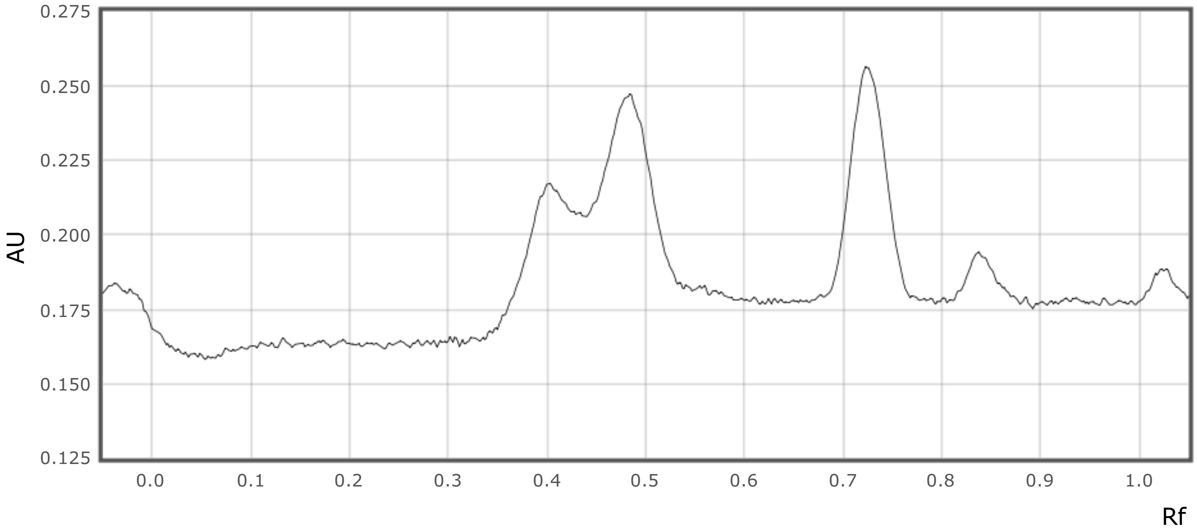

Track 7:

Type	Single λ

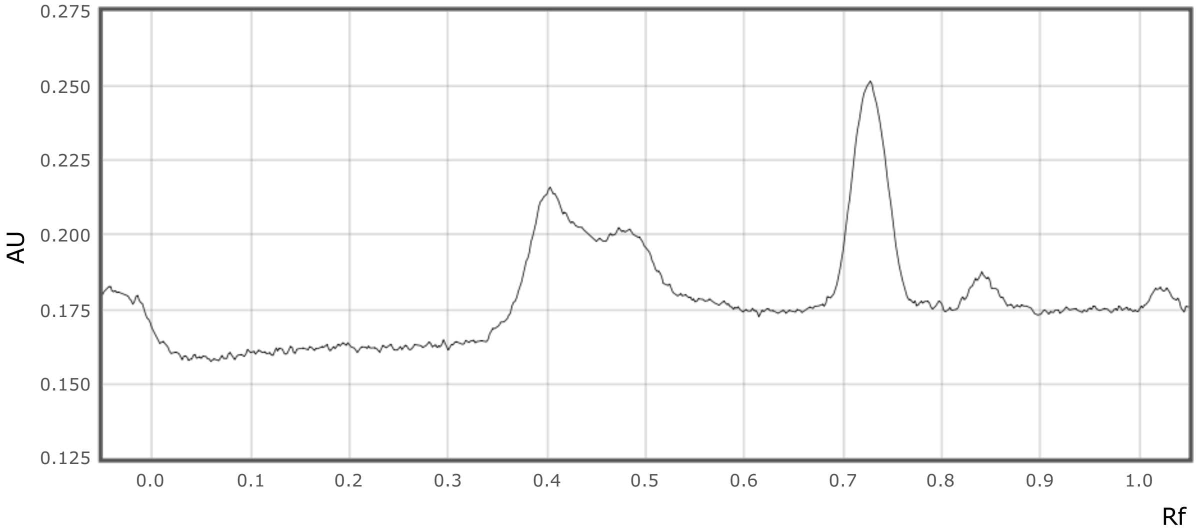

Track 8:

Type	Single λ

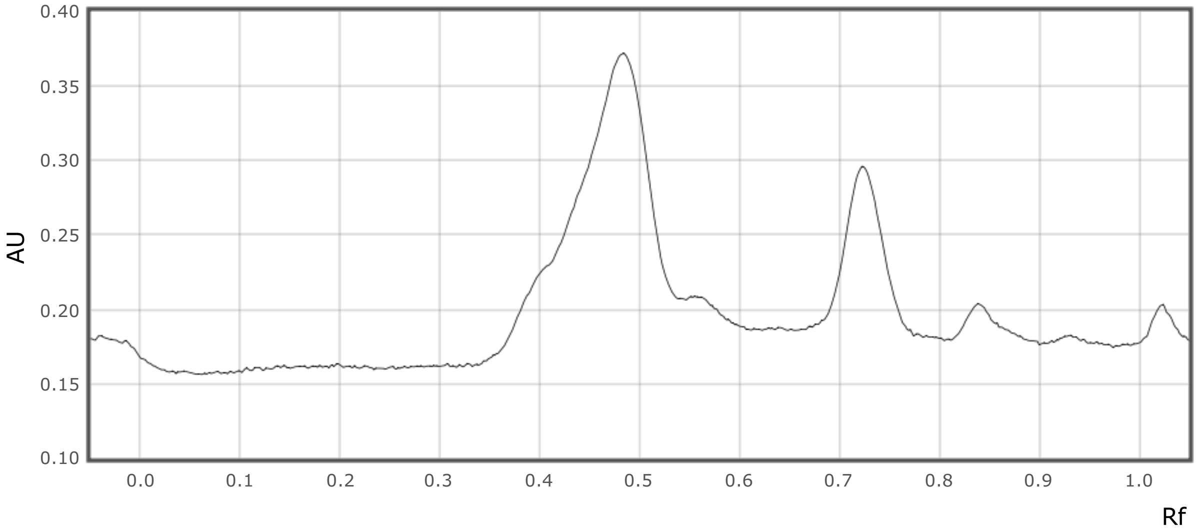

Track 9:	
Type	Single λ

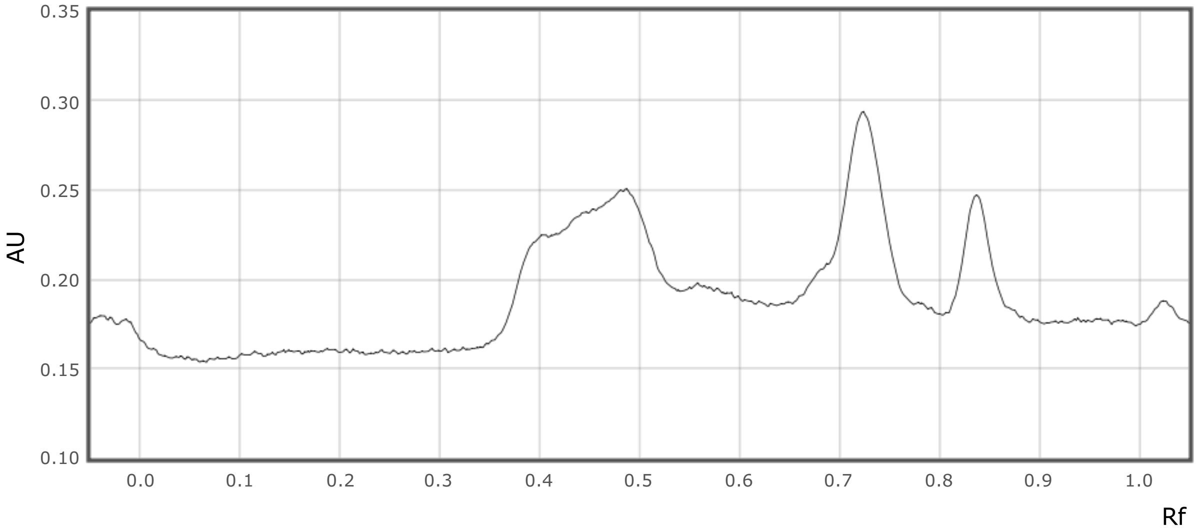

Track 10:	
Type	Single λ

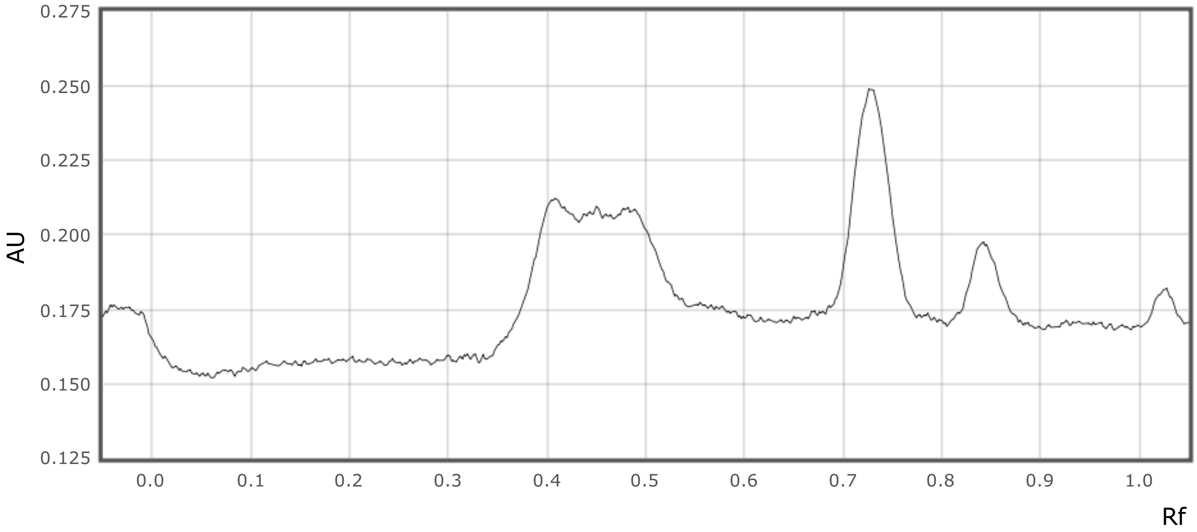

Track 11:

Type	Single λ

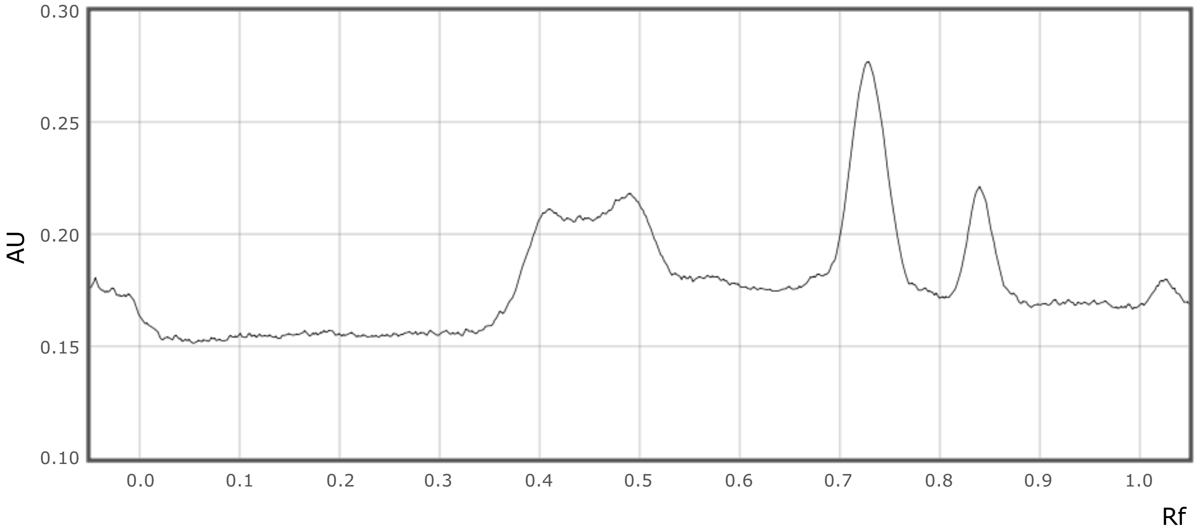

Track 12:

Type	Single λ

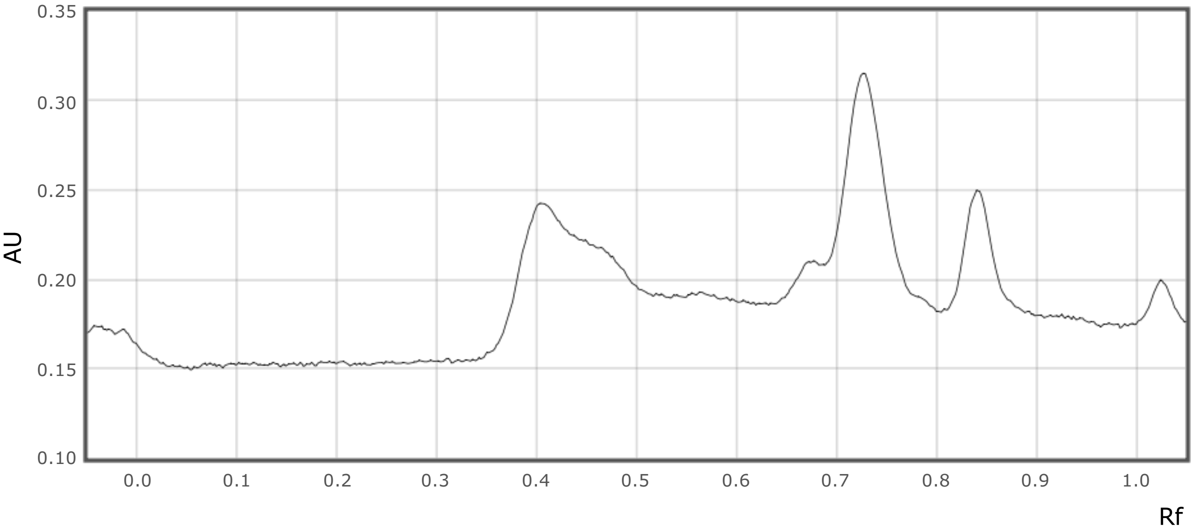

Track 13:	
Type	Single λ

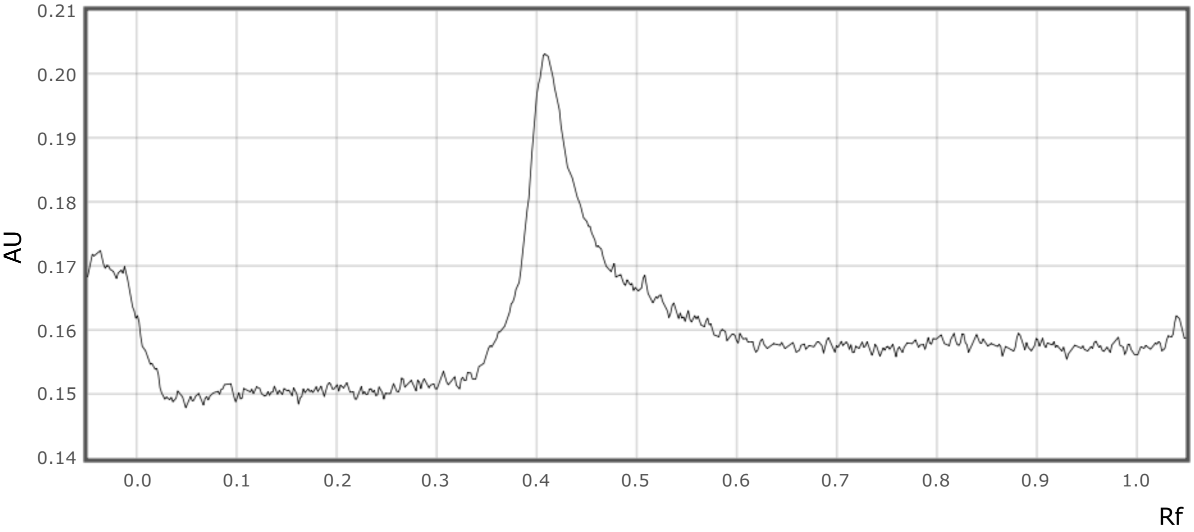

Track 14:	
Type	Single λ

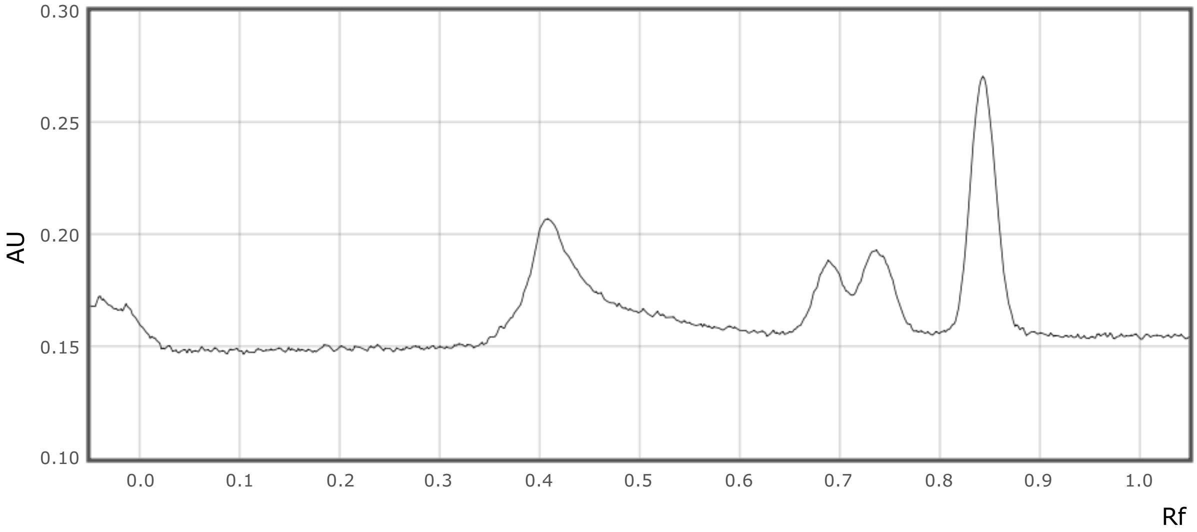

Track 15:

Type	Single λ

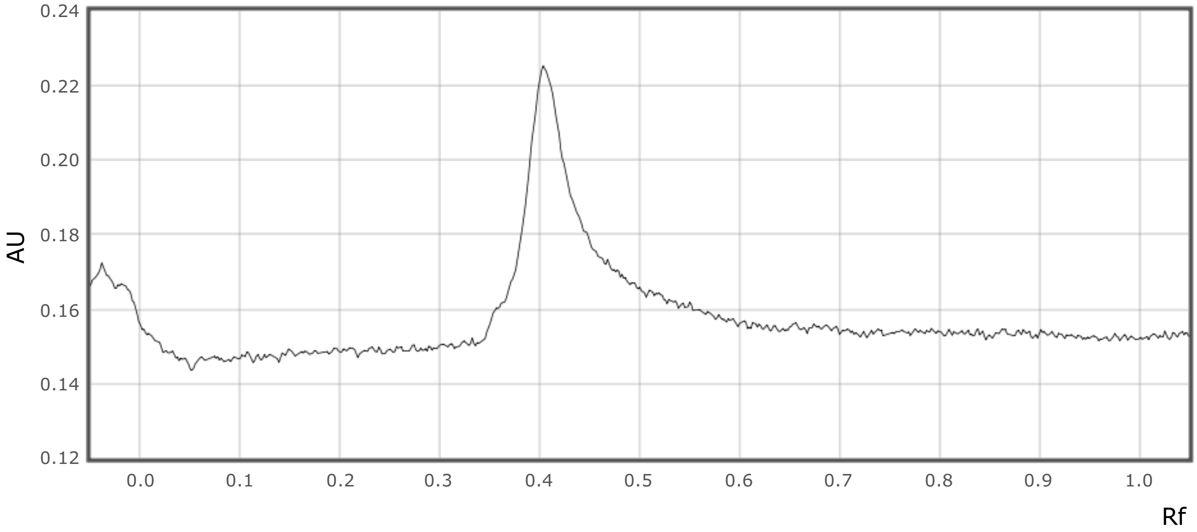

Derivatization 1 - dip:

Executed	15-Oct-2019 14:03:19 visionCATSuser

Take image derivatized plate 1a - Visualizer (S/N: 230515):

Executed	15-Oct-2019 14:04:38 visionCATSuser

RT White Derivatized, RemTransVis

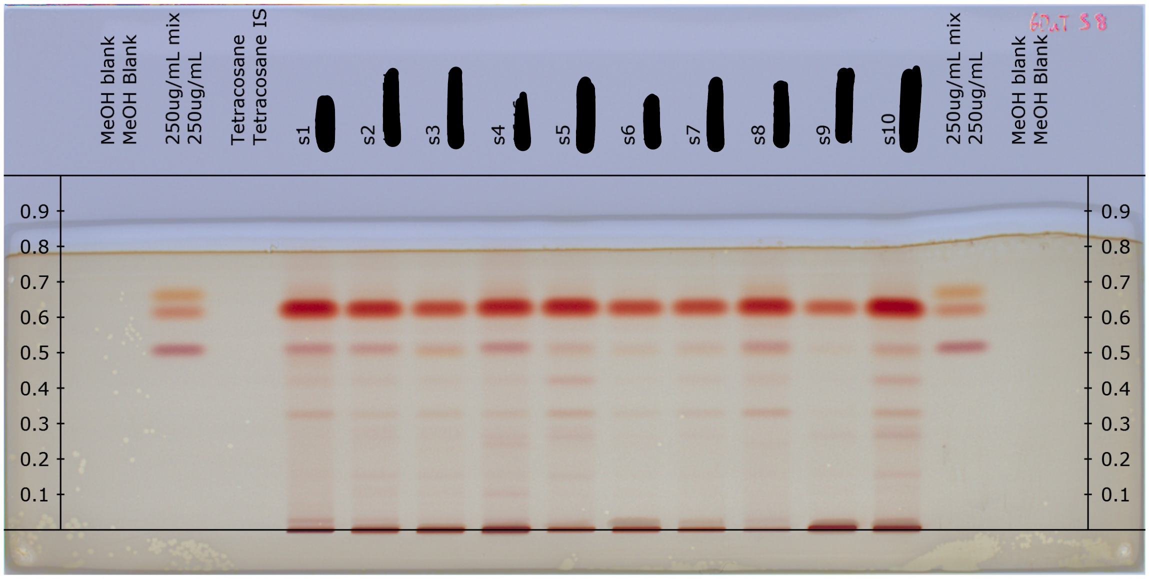

Exposure	0.051 s
Contrast	1
Normalized exposure	Disabled
Clarify	Disabled
White balance	1.23, 1.11, 0.78

R 366 Derivatized, Remission366

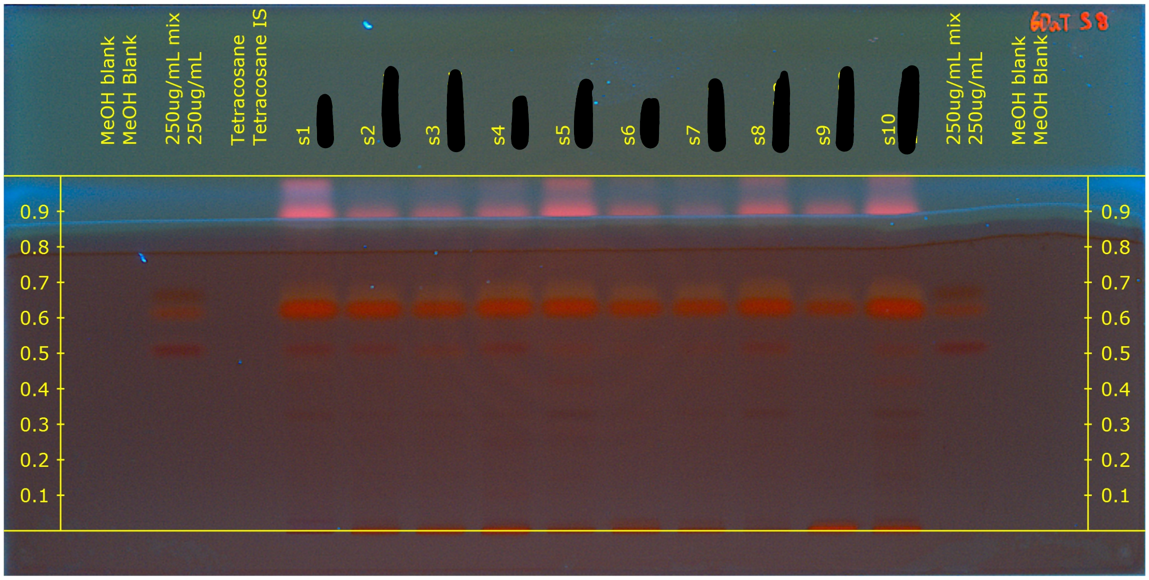

Exposure	9.999 s
Contrast	1
Normalized exposure	Disabled
Clarify	Disabled
White balance	1.00, 1.00, 1.00

Evaluation 1 :

Validated	false
Step	Take image derivatized plate 1a
Concentration unit type	Mass / volume
Notes	

Definition:

References:

250ug/mL mix

Substance Name	Concentration	Purity
9-THC	250.000 µg/ml	100.00 %
CBD	250.000 µg/ml	100.00 %
CBN	250.000 µg/ml	100.00 %

Samples:

Vial ID	Amount	Volume solution	Reference amount	Related to
MeOH blank		0.00 ml		
Tetracosane		0.00 ml		
s1		0.00 ml		
s2		0.00 ml		
s3		0.00 ml		
s4		0.00 ml		
s5		0.00 ml		
s6		0.00 ml		
s7		0.00 ml		
s8		0.00 ml		
s9		0.00 ml		
s10		0.00 ml		

Integration parameters:

Bounds	[0.000,1.000]
Smoothing	Savitzky–Golay of order 3 and window 7
Baseline correction	Lowest slope with noise 0.05
Profile subtraction	Profile subtraction from track 1
Peaks detection	Gauss (legacy) with sensitivity 0.1, separation 1 and threshold 0.1

Scan:

Wavelength	RT White

Track 1:

Type	Sample
Vial ID	MeOH blank
Description	MeOH Blank
Volume	2.0 µl

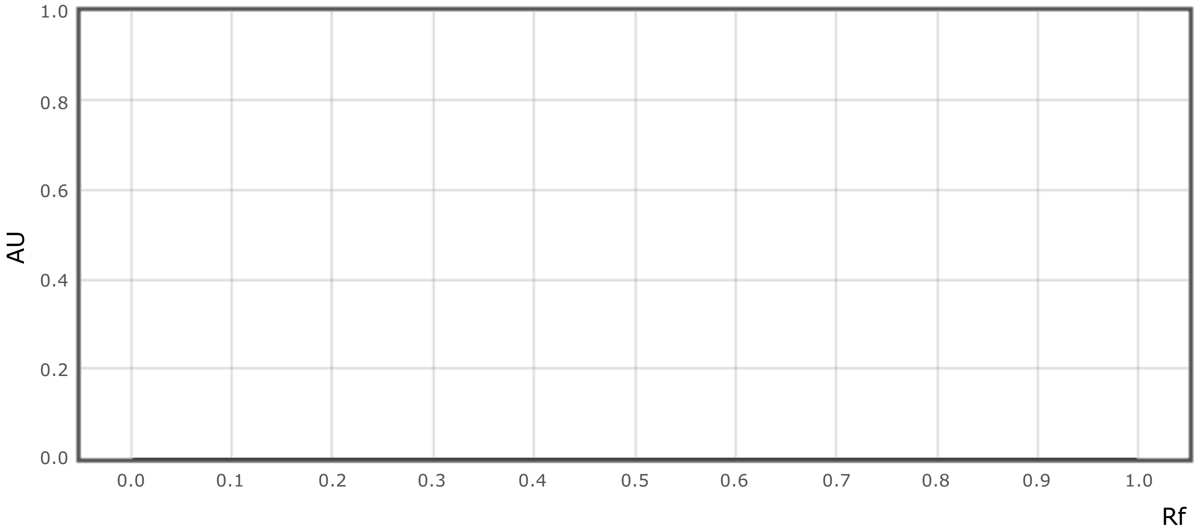

Peak #	Start		Max			End		Area		Manual peak	Substance Name
	Rf	H	Rf	H	%	Rf	H	A	%		

Track 2:

Type	Reference
Vial ID	250ug/mL mix
Description	250ug/mL
Volume	2.0 µl

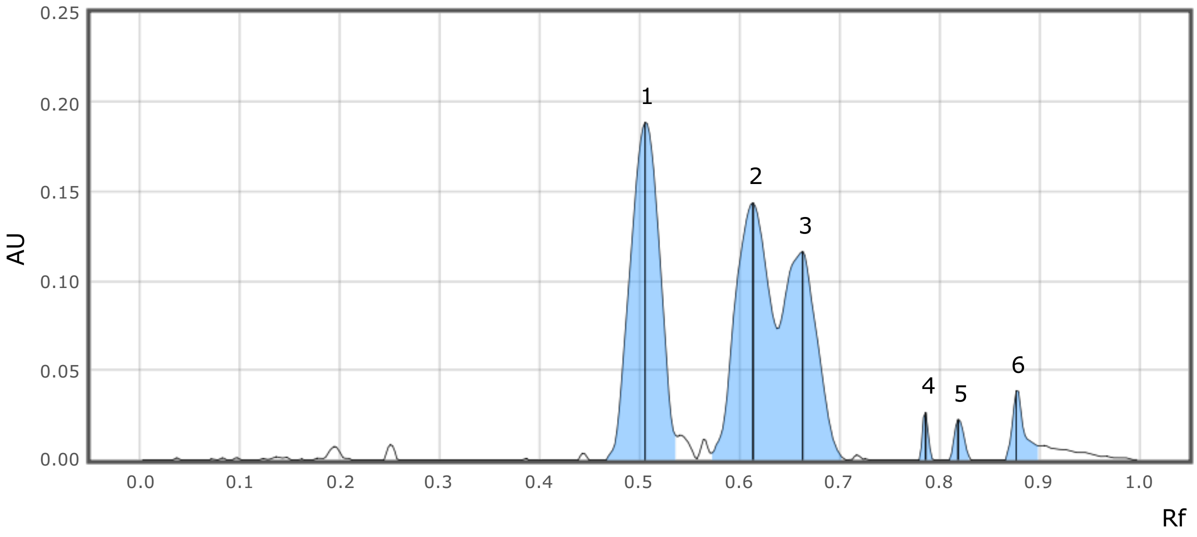

Peak #	Start		Max			End		Area		Manual peak	Substance Name
	Rf	H	Rf	H	%	Rf	H	A	%		
1	0.467	0.0000	0.505	0.1887	35.17	0.538	0.0130	0.00645	36.71	No	CBN
2	0.572	0.0038	0.613	0.1436	26.77	0.637	0.0733	0.00554	31.54	No	9-THC
3	0.637	0.0733	0.663	0.1165	21.71	0.706	0.0000	0.00459	26.12	No	CBD
4	0.780	0.0000	0.786	0.0265	4.93	0.795	0.0000	0.00017	0.99	No	
5	0.810	0.0000	0.819	0.0226	4.22	0.832	0.0000	0.00025	1.42	No	
6	0.866	0.0000	0.877	0.0386	7.20	0.901	0.0077	0.00057	3.22	No	

Track 3:

Type	Sample
Vial ID	Tetracosane
Description	Tetracosane IS
Volume	2.0 µl

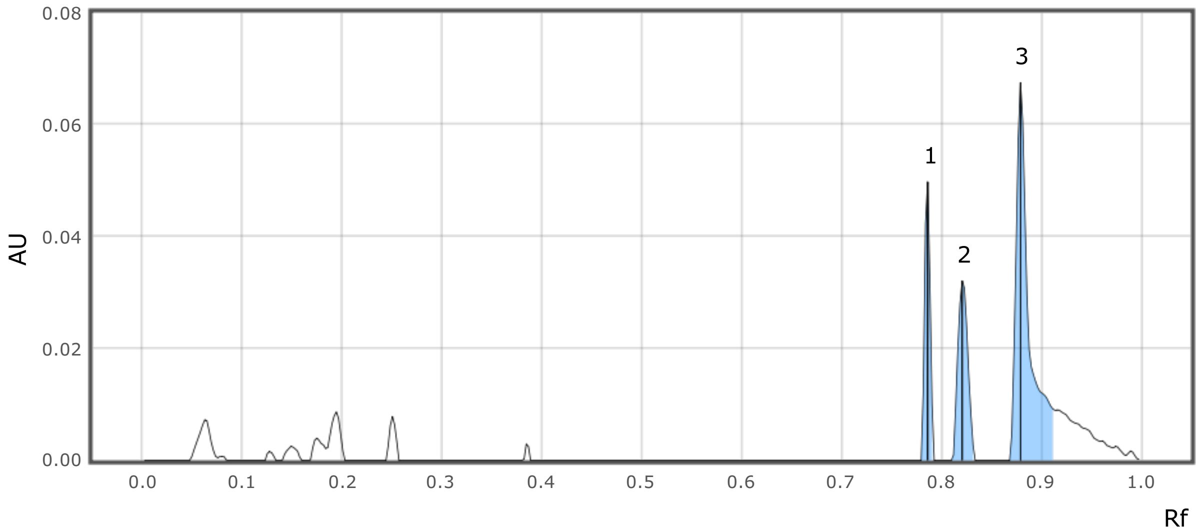

Peak	Start		Max			End		Area		Manual	Substance
#	Rf	H	Rf	H	%	Rf	H	A	%	peak	Name
1	0.780	0.0000	0.786	0.0497	33.32	0.793	0.0000	0.00032	18.34	No	
2	0.810	0.0000	0.821	0.0321	21.48	0.834	0.0000	0.00037	21.52	No	
3	0.868	0.0000	0.879	0.0674	45.19	0.914	0.0089	0.00104	60.14	No	

Track 4:

Type	Sample
Vial ID	s1
Description	[redacted]
Volume	2.0 µl

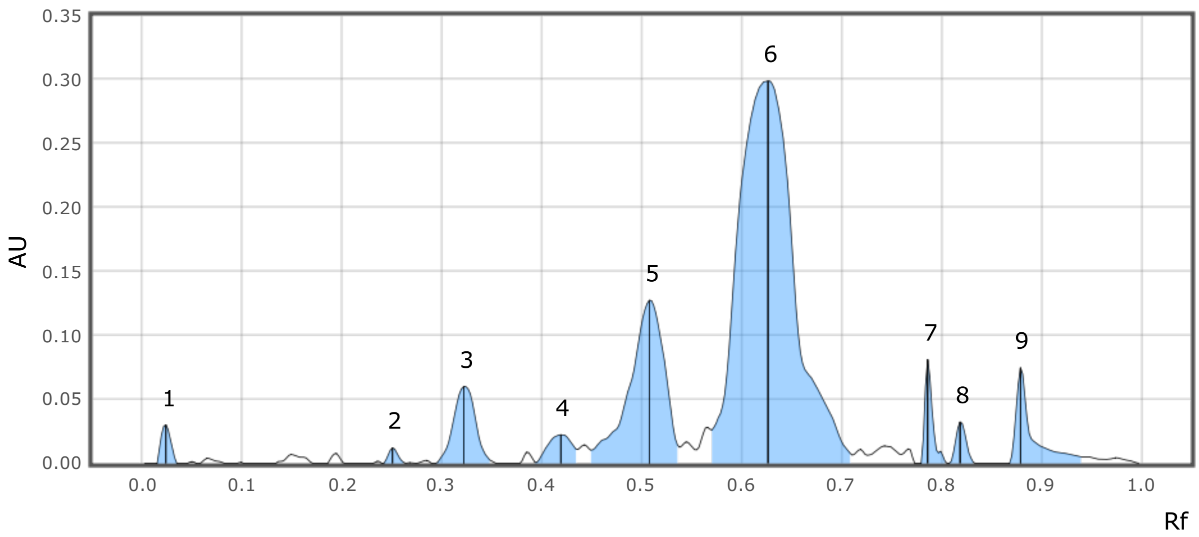

Peak #	Start Rf	Start H	Max Rf	Max H	Max %	End Rf	End H	Area A	Area %	Manual peak	Substance Name
1	0.015	0.0000	0.023	0.0301	4.08	0.036	0.0000	0.00033	1.12	No	
2	0.242	0.0000	0.250	0.0120	1.63	0.263	0.0001	0.00013	0.43	No	
3	0.294	0.0000	0.322	0.0600	8.12	0.354	0.0000	0.00153	5.14	No	
4	0.393	0.0007	0.419	0.0223	3.02	0.436	0.0109	0.00063	2.13	No	
5	0.449	0.0102	0.508	0.1275	17.25	0.538	0.0125	0.00517	17.37	No	
6	0.570	0.0260	0.626	0.2987	40.43	0.711	0.0065	0.01952	65.59	No	9-THC
7	0.780	0.0000	0.786	0.0810	10.97	0.806	0.0000	0.00071	2.38	No	
8	0.808	0.0000	0.819	0.0324	4.39	0.834	0.0000	0.00038	1.26	No	
9	0.868	0.0000	0.879	0.0747	10.11	0.944	0.0049	0.00136	4.58	No	

Track 5:

Type	Sample
Vial ID	s2
Description	[redacted]
Volume	2.0 µl

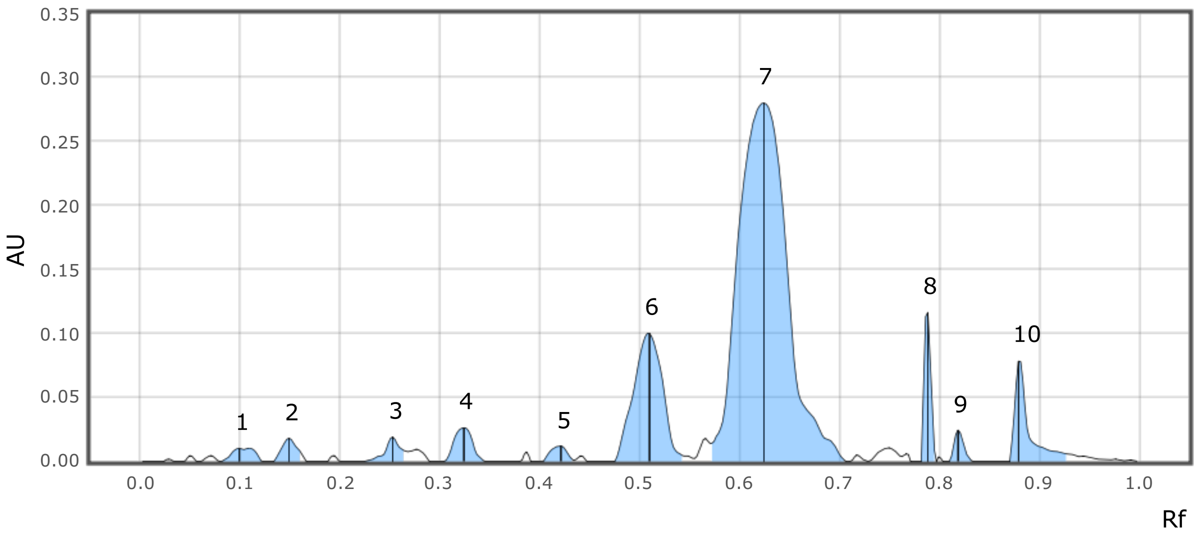

Peak #	Start Rf	Start H	Max Rf	Max H	Max %	End Rf	End H	Area A	Area %	Manual peak	Substance Name
1	0.080	0.0000	0.099	0.0102	1.49	0.123	0.0000	0.00027	1.15	No	
2	0.134	0.0000	0.149	0.0181	2.64	0.166	0.0000	0.00032	1.36	No	
3	0.224	0.0000	0.253	0.0190	2.78	0.268	0.0075	0.00034	1.45	No	
4	0.304	0.0000	0.324	0.0262	3.83	0.345	0.0000	0.00055	2.36	No	
5	0.402	0.0000	0.421	0.0119	1.74	0.434	0.0007	0.00023	0.97	No	
6	0.475	0.0000	0.510	0.1001	14.62	0.544	0.0042	0.00337	14.44	No	
7	0.572	0.0141	0.624	0.2800	40.90	0.706	0.0000	0.01584	67.87	No	9-THC
8	0.782	0.0000	0.789	0.1164	17.00	0.797	0.0000	0.00091	3.90	No	
9	0.810	0.0000	0.819	0.0244	3.57	0.834	0.0000	0.00026	1.12	No	
10	0.871	0.0000	0.879	0.0783	11.44	0.929	0.0056	0.00126	5.39	No	

Track 6:

Type	Sample
Vial ID	s3
Description	[redacted]
Volume	2.0 µl

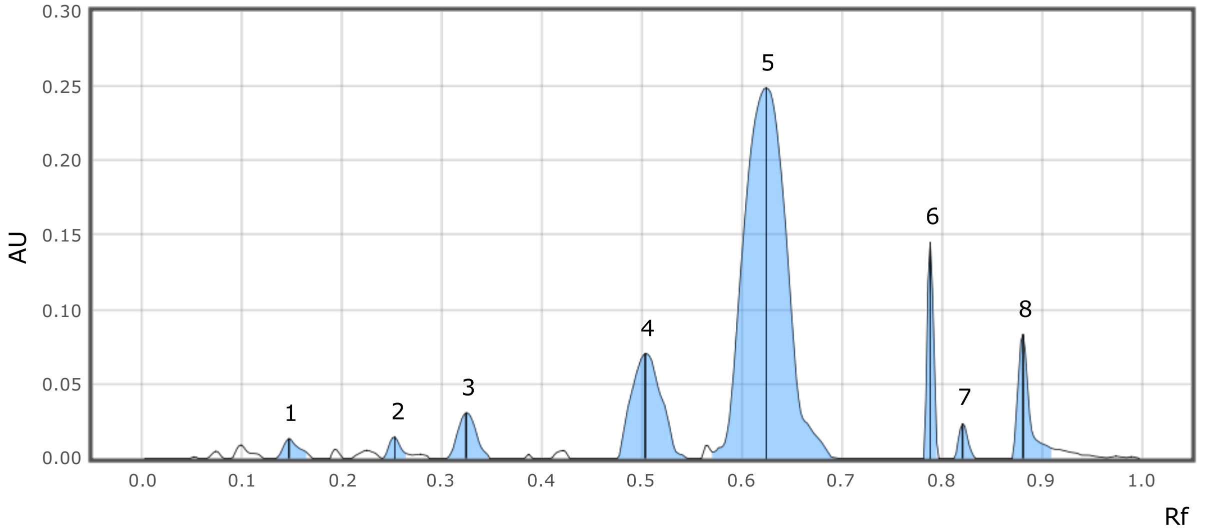

Peak #	Start Rf	Start H	Max Rf	Max H	Max %	End Rf	End H	Area A	Area %	Manual peak	Substance Name
1	0.134	0.0000	0.147	0.0133	2.12	0.170	0.0000	0.00024	1.31	No	
2	0.240	0.0000	0.253	0.0143	2.28	0.270	0.0022	0.00020	1.11	No	
3	0.304	0.0000	0.324	0.0305	4.86	0.348	0.0000	0.00065	3.54	No	
4	0.475	0.0000	0.503	0.0702	11.18	0.546	0.0000	0.00243	13.35	No	
5	0.570	0.0043	0.624	0.2484	39.56	0.698	0.0000	0.01227	67.31	No	9-THC
6	0.782	0.0000	0.789	0.1447	23.05	0.797	0.0000	0.00105	5.77	No	
7	0.812	0.0000	0.821	0.0232	3.70	0.834	0.0000	0.00024	1.33	No	
8	0.871	0.0000	0.881	0.0831	13.24	0.914	0.0061	0.00115	6.29	No	

Track 7:

Type	Sample
Vial ID	s4
Description	[redacted]
Volume	2.0 µl

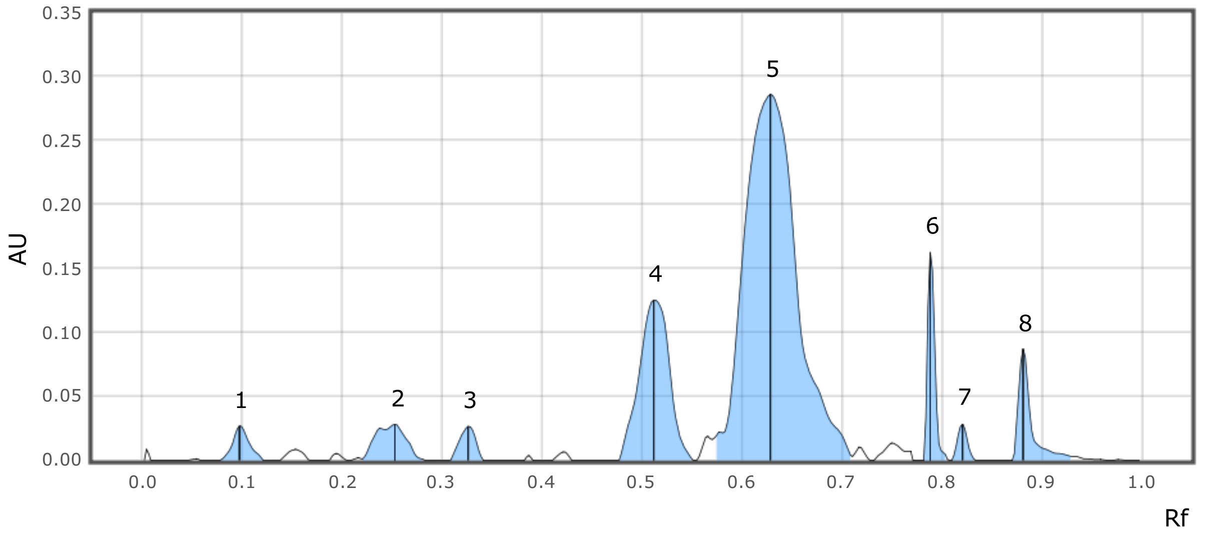

Peak #	Start		Max			End		Area		Manual peak	Substance Name
	Rf	H	Rf	H	%	Rf	H	A	%		
1	0.078	0.0000	0.097	0.0267	3.47	0.123	0.0000	0.00050	1.89	No	
2	0.220	0.0010	0.253	0.0281	3.65	0.283	0.0000	0.00100	3.73	No	
3	0.307	0.0000	0.326	0.0265	3.45	0.341	0.0000	0.00049	1.83	No	
4	0.475	0.0000	0.512	0.1252	16.25	0.551	0.0003	0.00440	16.46	No	
5	0.575	0.0193	0.629	0.2860	37.12	0.711	0.0030	0.01740	65.05	No	9-THC
6	0.782	0.0000	0.789	0.1625	21.10	0.806	0.0006	0.00136	5.10	No	
7	0.810	0.0000	0.821	0.0283	3.67	0.834	0.0000	0.00032	1.18	No	
8	0.871	0.0000	0.881	0.0870	11.29	0.931	0.0031	0.00127	4.77	No	

Track 8:

Type	Sample
Vial ID	s5
Description	[redacted]
Volume	2.0 µl

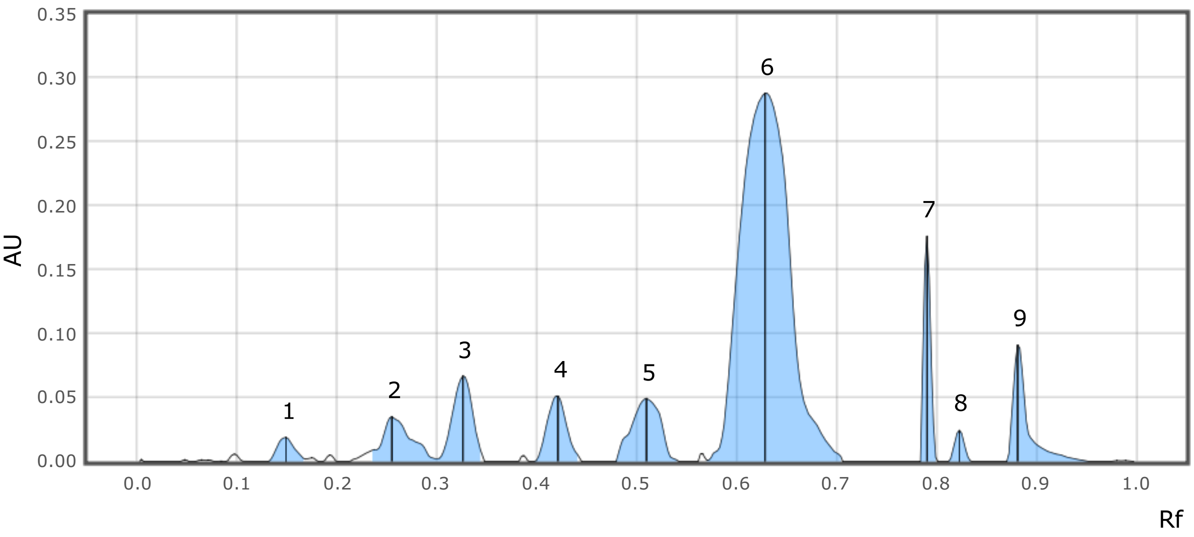

Peak #	Start		Max			End		Area		Manual peak	Substance Name
	Rf	H	Rf	H	%	Rf	H	A	%		
1	0.132	0.0000	0.149	0.0188	2.35	0.168	0.0018	0.00037	1.46	No	
2	0.233	0.0080	0.255	0.0350	4.36	0.300	0.0022	0.00116	4.54	No	
3	0.300	0.0022	0.326	0.0669	8.35	0.348	0.0000	0.00154	6.01	No	
4	0.397	0.0000	0.421	0.0512	6.39	0.445	0.0000	0.00115	4.49	No	
5	0.479	0.0000	0.510	0.0494	6.17	0.542	0.0000	0.00165	6.47	No	
6	0.570	0.0008	0.629	0.2880	35.94	0.706	0.0000	0.01649	64.55	No	9-THC
7	0.784	0.0000	0.791	0.1763	22.01	0.801	0.0000	0.00143	5.59	No	
8	0.812	0.0000	0.823	0.0245	3.06	0.836	0.0000	0.00027	1.04	No	
9	0.871	0.0000	0.881	0.0911	11.37	0.955	0.0000	0.00149	5.84	No	

Track 9:

Type	Sample
Vial ID	s6
Description	[redacted]
Volume	2.0 µl

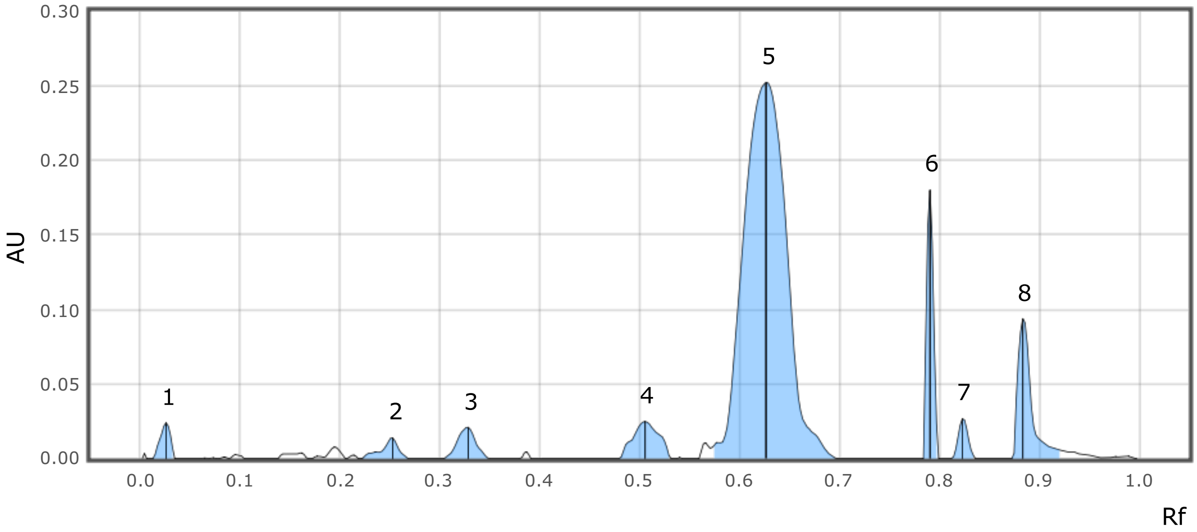

Peak #	Start Rf	Start H	Max Rf	Max H	Max %	End Rf	End H	Area A	Area %	Manual peak	Substance Name
1	0.013	0.0000	0.026	0.0239	3.76	0.034	0.0000	0.00028	1.61	No	
2	0.222	0.0000	0.253	0.0137	2.15	0.270	0.0000	0.00026	1.46	No	
3	0.304	0.0000	0.328	0.0209	3.29	0.350	0.0000	0.00043	2.49	No	
4	0.479	0.0000	0.505	0.0246	3.88	0.531	0.0000	0.00075	4.31	No	
5	0.575	0.0087	0.626	0.2519	39.66	0.698	0.0000	0.01250	71.63	No	9-THC
6	0.784	0.0000	0.791	0.1799	28.32	0.799	0.0000	0.00143	8.19	No	
7	0.812	0.0000	0.823	0.0266	4.19	0.836	0.0000	0.00029	1.67	No	
8	0.873	0.0000	0.884	0.0937	14.75	0.929	0.0043	0.00151	8.64	No	

Track 10:

Type	Sample
Vial ID	s7
Description	[redacted]
Volume	2.0 µl

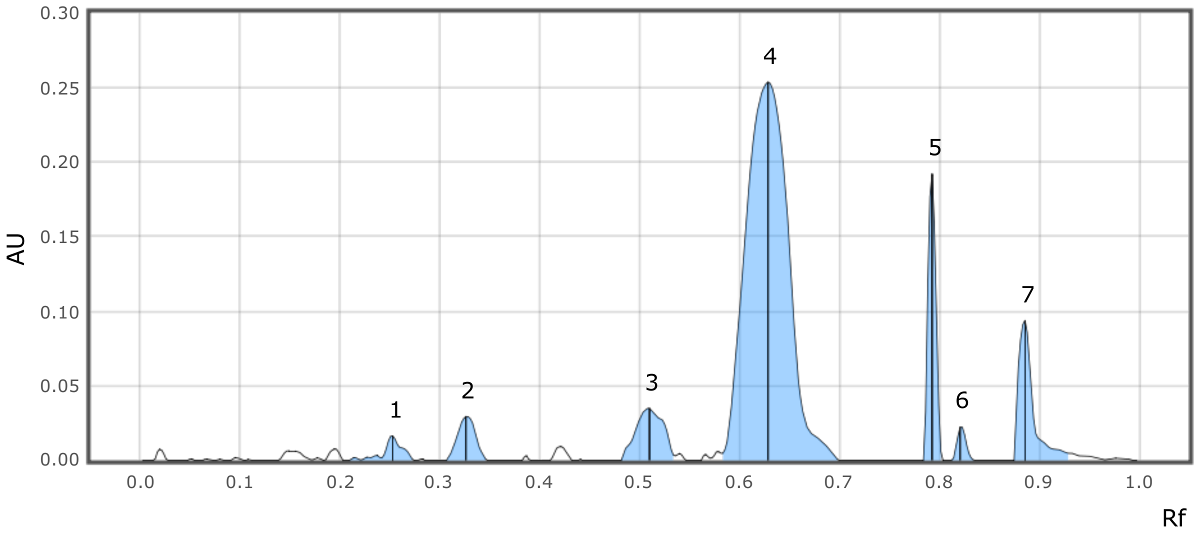

World Leader in Planar Chromatography

Peak	Start		Max			End		Area		Manual	Substance
#	Rf	H	Rf	H	%	Rf	H	A	%	peak	Name
1	0.207	0.0000	0.253	0.0164	2.56	0.274	0.0000	0.00032	1.77	No	
2	0.304	0.0000	0.326	0.0294	4.58	0.348	0.0000	0.00061	3.37	No	
3	0.482	0.0000	0.510	0.0348	5.43	0.536	0.0030	0.00113	6.22	No	
4	0.583	0.0046	0.629	0.2534	39.49	0.700	0.0000	0.01257	69.01	No	9-THC
5	0.784	0.0000	0.793	0.1918	29.90	0.804	0.0000	0.00175	9.63	No	
6	0.812	0.0000	0.821	0.0221	3.45	0.836	0.0000	0.00023	1.24	No	
7	0.875	0.0000	0.886	0.0937	14.60	0.931	0.0046	0.00160	8.77	No	

Track 11:

Type	Sample
Vial ID	s8
Description	[redacted]
Volume	2.0 µl

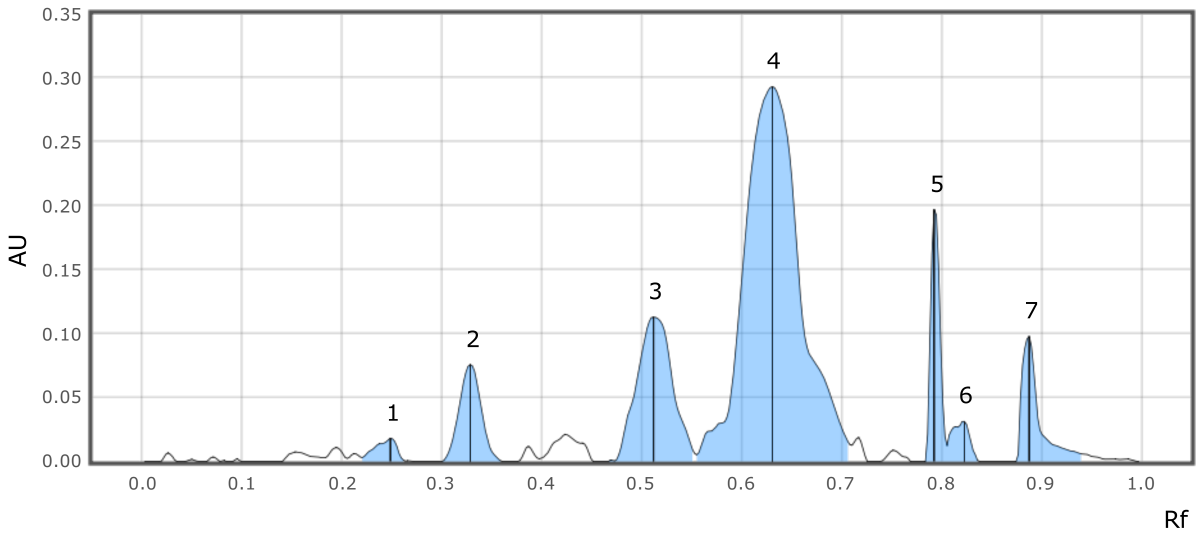

Peak	Start		Max			End		Area		Manual	Substance
#	Rf	H	Rf	H	%	Rf	H	A	%	peak	Name
1	0.220	0.0030	0.248	0.0182	2.20	0.263	0.0002	0.00045	1.46	No	
2	0.300	0.0000	0.328	0.0757	9.17	0.361	0.0000	0.00191	6.19	No	
3	0.467	0.0000	0.512	0.1127	13.65	0.553	0.0063	0.00471	15.25	No	
4	0.555	0.0051	0.631	0.2931	35.49	0.709	0.0130	0.01909	61.74	No	9-THC
5	0.784	0.0000	0.793	0.1970	23.86	0.806	0.0121	0.00214	6.91	No	
6	0.806	0.0121	0.823	0.0312	3.78	0.838	0.0000	0.00065	2.11	No	
7	0.875	0.0000	0.888	0.0978	11.85	0.942	0.0057	0.00196	6.34	No	

Track 12:

Type	Sample
Vial ID	s9
Description	[redacted]
Volume	2.0 µl

World Leader in Planar Chromatography

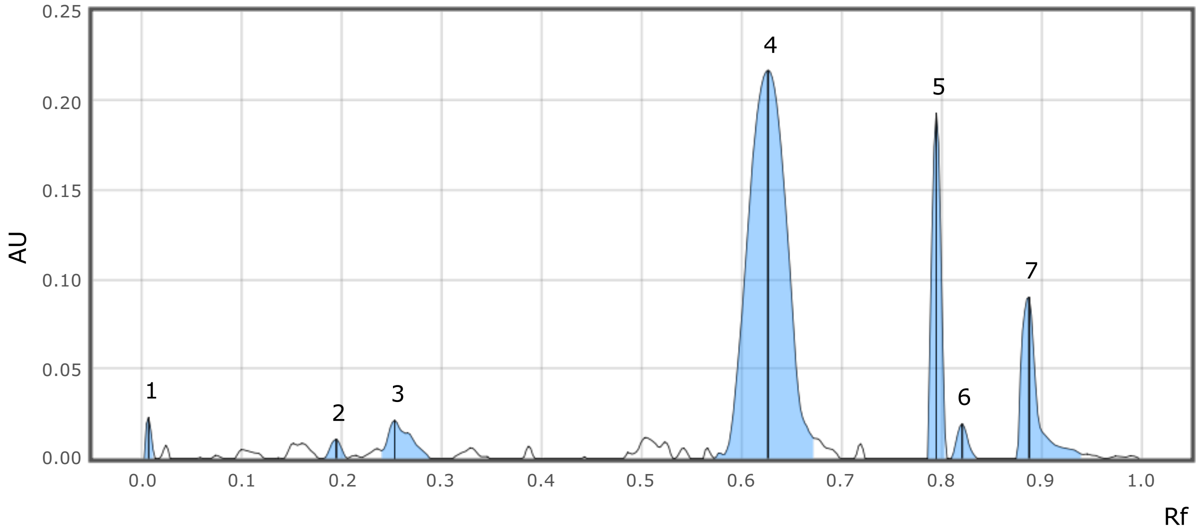

Peak #	Start Rf	Start H	Max Rf	Max H	Max %	End Rf	End H	Area A	Area %	Manual peak	Substance Name
1	0.002	0.0000	0.006	0.0229	4.00	0.013	0.0000	0.00014	0.95	No	
2	0.181	0.0000	0.194	0.0107	1.86	0.205	0.0001	0.00013	0.89	No	
3	0.240	0.0043	0.253	0.0213	3.71	0.289	0.0000	0.00053	3.67	No	
4	0.572	0.0001	0.626	0.2169	37.77	0.674	0.0110	0.00975	67.56	No	9-THC
5	0.786	0.0000	0.795	0.1929	33.60	0.806	0.0000	0.00202	14.00	No	
6	0.810	0.0000	0.821	0.0194	3.37	0.836	0.0000	0.00024	1.66	No	
7	0.875	0.0000	0.888	0.0901	15.69	0.942	0.0022	0.00163	11.27	No	

Track 13:

Type	Sample
Vial ID	s10
Description	[redacted]
Volume	2.0 µl

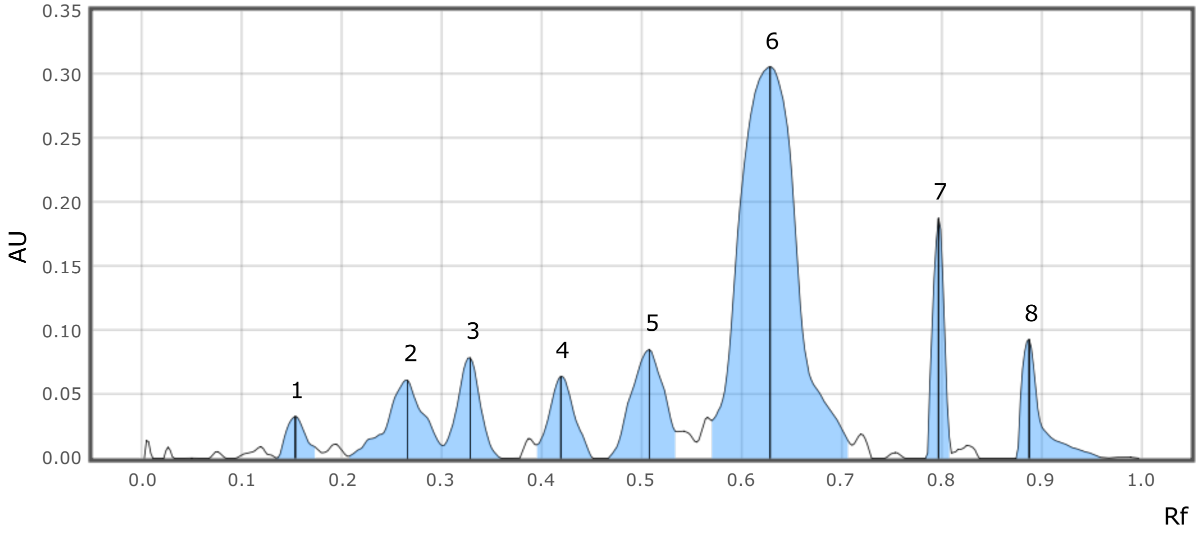

Peak	Start		Max			End		Area		Manual	Substance
#	Rf	H	Rf	H	%	Rf	H	A	%	peak	Name
1	0.134	0.0003	0.153	0.0328	3.61	0.179	0.0044	0.00077	2.16	No	
2	0.207	0.0016	0.266	0.0611	6.73	0.300	0.0099	0.00263	7.40	No	
3	0.300	0.0099	0.328	0.0786	8.65	0.361	0.0000	0.00210	5.92	No	
4	0.395	0.0106	0.419	0.0641	7.05	0.451	0.0000	0.00185	5.22	No	
5	0.467	0.0000	0.508	0.0850	9.36	0.536	0.0208	0.00324	9.13	No	
6	0.570	0.0294	0.629	0.3062	33.70	0.709	0.0115	0.02040	57.49	No	9-THC
7	0.784	0.0000	0.797	0.1877	20.67	0.810	0.0041	0.00244	6.89	No	
8	0.875	0.0000	0.888	0.0929	10.23	0.968	0.0003	0.00206	5.79	No	

Track 14:

Type	Reference
Vial ID	250ug/mL mix
Description	250ug/mL
Volume	2.0 µl

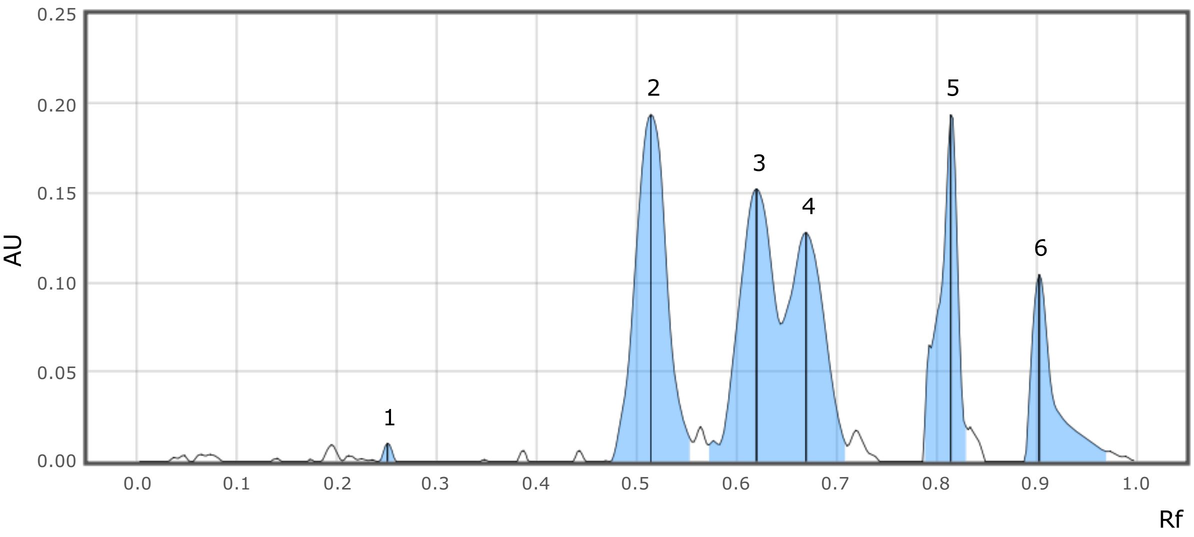

Peak	Start		Max			End		Area		Manual	Substance
#	Rf	H	Rf	H	%	Rf	H	A	%	peak	Name
1	0.242	0.0000	0.250	0.0102	1.30	0.259	0.0000	0.00009	0.36	No	
2	0.473	0.0000	0.514	0.1939	24.78	0.555	0.0110	0.00713	28.02	No	CBN
3	0.572	0.0092	0.620	0.1523	19.46	0.644	0.0767	0.00599	23.55	No	9-THC
4	0.644	0.0767	0.670	0.1279	16.35	0.711	0.0086	0.00543	21.35	No	CBD
5	0.789	0.0215	0.814	0.1937	24.75	0.832	0.0178	0.00406	15.94	No	
6	0.888	0.0000	0.903	0.1044	13.35	0.972	0.0055	0.00274	10.78	No	

Track 15:

Type	Sample
Vial ID	MeOH blank
Description	MeOH Blank
Volume	2.0 µl

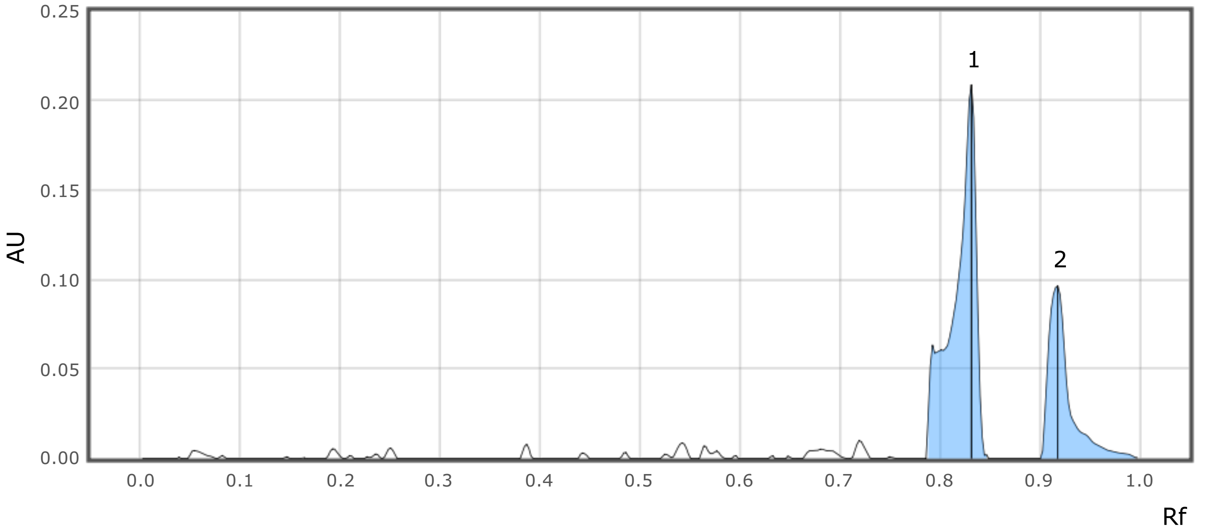

Peak #	Start Rf	Start H	Max Rf	Max H	Max %	End Rf	End H	Area A	Area %	Manual peak	Substance Name
1	0.789	0.0233	0.832	0.2088	68.41	0.849	0.0000	0.00515	69.11	No	
2	0.901	0.0000	0.918	0.0964	31.59	0.998	0.0004	0.00230	30.89	No	

Calibration results:

Height calibration for substance 9-THC @ RT White:

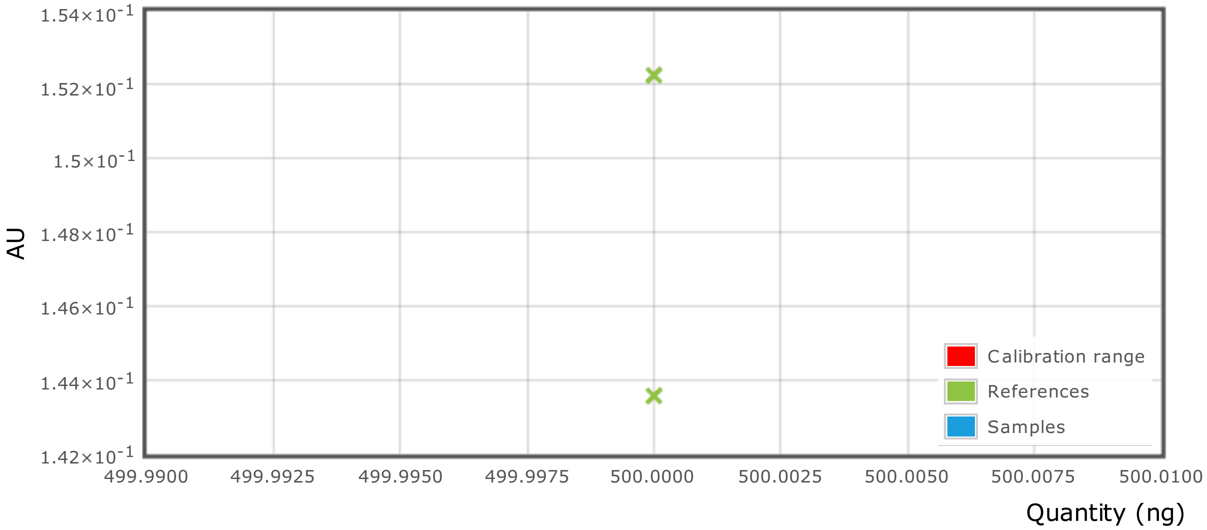

Regression mode	Linear-2
Range deviation	5.00 %
Related substances	Default
Number of references	2
Calibration function	$y=0x$
Coefficient of variation	CV 0.00 %
Correlation coefficient	n/a
⚠	Unable to compute the results for this substance because there wasn't enough groups of references replicas (at least 1 for Linear-1, 2 for Linear2 and Mime-1 and 3 for Polynomial and MiMe-2)

Height calibration for substance CBD @ RT White:

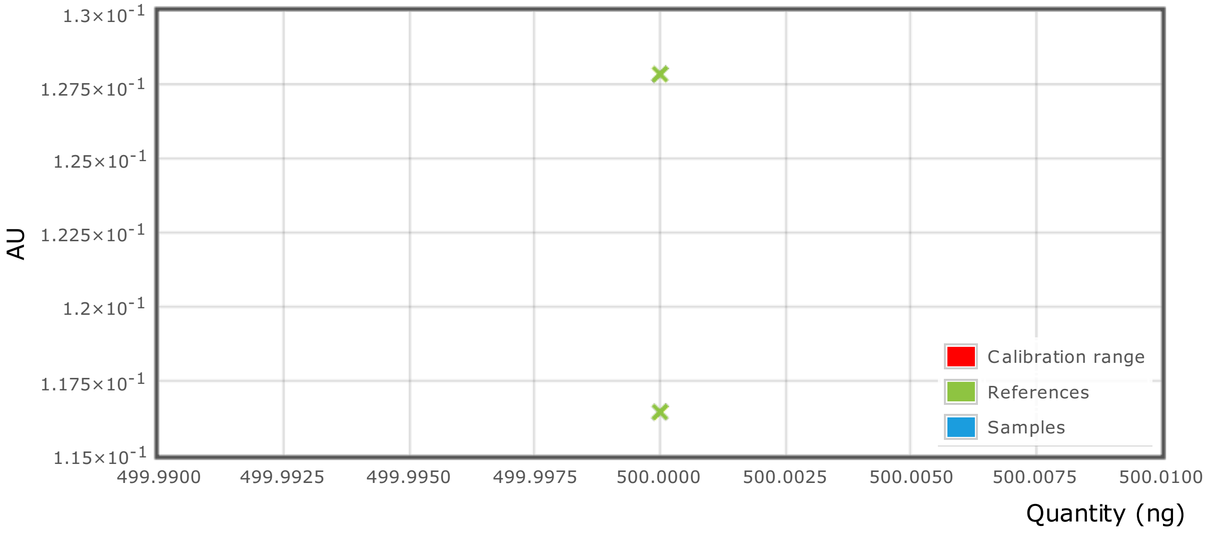

Regression mode	Linear-2
Range deviation	5.00 %
Related substances	Default
Number of references	2
Calibration function	$y=0x$
Coefficient of variation	CV 0.00 %
Correlation coefficient	n/a
⚠	Unable to compute the results for this substance because there wasn't enough groups of references replicas (at least 1 for Linear-1, 2 for Linear2 and Mime-1 and 3 for Polynomial and MiMe-2)

Height calibration for substance CBN @ RT White:

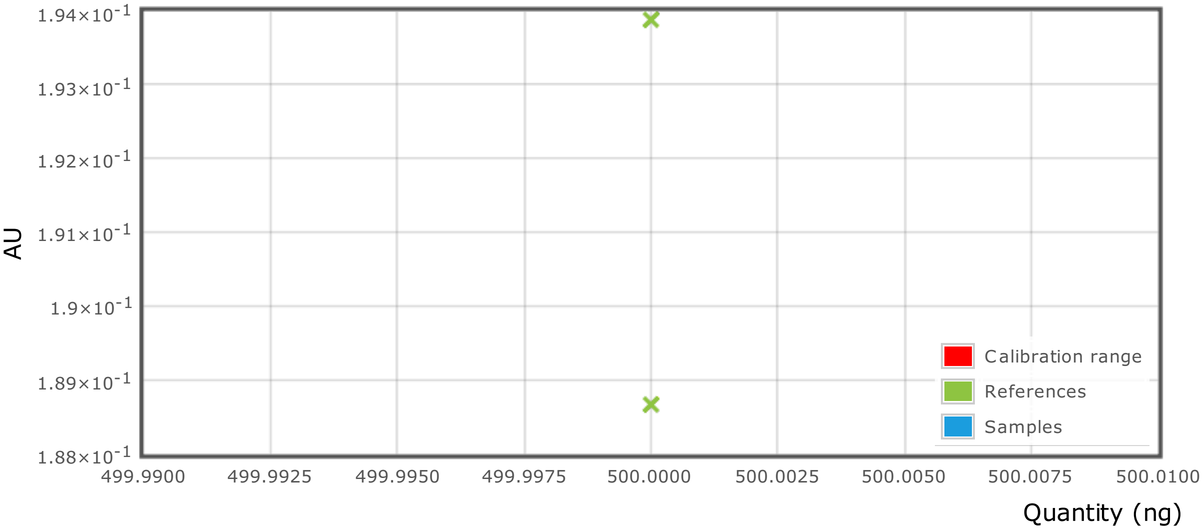

Regression mode	Linear-2
Range deviation	5.00 %
Related substances	Default
Number of references	2
Calibration function	$y=0x$
Coefficient of variation	CV 0.00 %
Correlation coefficient	n/a
⚠	Unable to compute the results for this substance because there wasn't enough groups of references replicas (at least 1 for Linear-1, 2 for Linear2 and Mime-1 and 3 for Polynomial and MiMe-2)

Results:

Substance having no available results		
⚠	9-THC	Unable to compute the results for this substance because there wasn't enough groups of references replicas (at least 1 for Linear-1, 2 for Linear2 and Mime-1 and 3 for Polynomial and MiMe-2)
⚠	CBD	There wasn't any sample application available in the assignments for this substance. Please check that the peaks were correctly detected and assigned for this substance.
⚠	CBN	There wasn't any sample application available in the assignments for this substance. Please check that the peaks were correctly detected and assigned for this substance.

A track marked with ⚠ means: this result is outside the regression range given by the reference assignments, but is included in the results because it is in the allowed range deviation.

Analyst:	Reviewer:
