# Supplementary material for: High Performance Thin-Layer Chromatography (HPTLC) data of Cannabinoids in ten mobile phase systems
Source: Data Brief. 2020 Jun 30;31:105955. doi: 10.1016/j.dib.2020.105955 (PMC7352075; doi:10.1016/j.dib.2020.105955)
Supplement: Supplementary file 1 [file mmc1.zip › S4-Case sample reports/6DaT-sample run-9.pdf]

Analysis: 6DaT-sample run-9

Path: Home/YL Research

Based on method: Samples (no cal)

Created	15-Oct-2019 14:10:10	visionCATSuser
Modified	15-Oct-2019 16:16:56	visionCATSuser
Last HPTLC log	15-Oct-2019 16:16:56	Analysis modified
Explorer notes		

Track	Vial ID	Description	Volume	Position	Type
1	MeOH blank	MeOH Blank	2.0 µl	A1	Sample
2	250ug/mL mix	250ug/mL	2.0 µl	A2	Reference
3	Tetracosane	Tetracosane IS	2.0 µl	A3	Sample
4	s1	[redacted]	2.0 µl	B1	Sample
5	s2	[redacted]	2.0 µl	B2	Sample
6	s3	[redacted]	2.0 µl	B3	Sample
7	s4	[redacted]	2.0 µl	B4	Sample
8	s5	[redacted]	2.0 µl	B5	Sample
9	s6	[redacted]	2.0 µl	B6	Sample
10	s7	[redacted]	2.0 µl	B7	Sample
11	s8	[redacted]	2.0 µl	B8	Sample
12	s9	[redacted]	2.0 µl	B9	Sample
13	s10	[redacted]	2.0 µl	B10	Sample
14	250ug/mL mix	250ug/mL	2.0 µl	A2	Reference
15	MeOH blank	MeOH Blank	2.0 µl	A1	Sample
Sequence table notes					

A track marked with ⚠ means: the application type is overridden in some evaluation(s).

System setup:

Software	Server User-PC, version 2.5.18072.1
ATS4	S/N:080713
Chamber	N/A
Derivatization dip	N/A
Scanner3	S/N:031025
Visualizer	S/N:230515

Chromatography

Plate layout:

Stationary phase	Merck, HPTLC plates silica gel 60 F 254
Plate format	200.0 x 100.0 mm
Application type	Band
Application	Position Y: 8.0 mm, length: 8.0 mm, width: 0.0 mm
Track	First position X: 20.0 mm, distance: 11.4 mm
Solvent front position	70.0 mm
Notes	

Take image clean plate 1a - Visualizer (S/N: 230515):

Quality	Enhanced
RT White	auto capture, Auto, level 85 %, Band
R 254	auto capture, Auto, level 85 %, Band
Instrument diagnostics	Valid diagnostics
Documentation step label	
Notes	

Application 1 - ATS 4 (S/N: 080713):

Spray gas	NI
Sample solvent type	Methanol
Filling speed	15 µl/s
Predosage volume	200 nl
Retraction volume	200 nl
Dosage speed	150 nl/s
Filling quality	User
Rinsing cycles / vacuum	2 / 4 s
Filling cycles / vacuum	1 / 4 s
Rinsing solvent name	Methanol
Nozzle temperature	Unheated
Rack in use	Standard
Instrument diagnostics	Valid diagnostics
Notes	

Development 1 - Chamber:

Tank	TTC 20x10
Mobile phase	6% diethylamine in toluene
Saturation time	20 min
Use saturation pad	true
Use smartALERT	false
Volume front through	10 ml
Volume rear through	25 ml
Drying time	5 min
Drying temperature	Room temperature
Notes	

Take image developed plate 1a - Visualizer (S/N: 230515):

Quality	Enhanced
RT White	auto capture, Auto, level 85 %, Band
R 254	auto capture, Auto, level 85 %, Band
R 366	auto capture, Auto, level 85 %, Band
Instrument diagnostics	Valid diagnostics
Documentation step label	
Notes	

Scan developed plate 1b - Scanner 3 (S/N: 031025):

Scanner type	Single λ
Optimization for	Resolution
Measurement mode	Absorption
Filter	n/a
Detector mode	Automatic
Scanning speed	20 mm/s
Data resolution	100 µm/step
Slit	5 x 0.2 mm, micro
Partial scan	No
Lamp	Deuterium & Tungsten
Wavelength(s)	254 nm
Instrument diagnostics	Valid diagnostics
Documentation step label	
Notes	

Derivatization 1 - dip:

Reagent name	
Dipping speed	5
Dipping time	0 s
Reagent preparation	
Heating	100 °C for 3 min, heated after
Notes	

Take image derivatized plate 1a - Visualizer (S/N: 230515):

Quality	Enhanced
RT White	auto capture, Auto, level 85 %, Band
R 366	auto capture, Auto, level 85 %, Band
Instrument diagnostics	Valid diagnostics
Documentation step label	
Notes	

System suitability tests:

SST settings:

SST tracks	

Data acquisition

Application 1 - ATS 4 (S/N: 080713):

Executed	15-Oct-2019 14:16:21 visionCATSuser

Development 1 - Chamber:

Executed	15-Oct-2019 15:25:15 visionCATSuser

Take image developed plate 1a - Visualizer (S/N: 230515):

Executed	15-Oct-2019 15:53:30 visionCATSuser

RT White Developed, RemTransVis

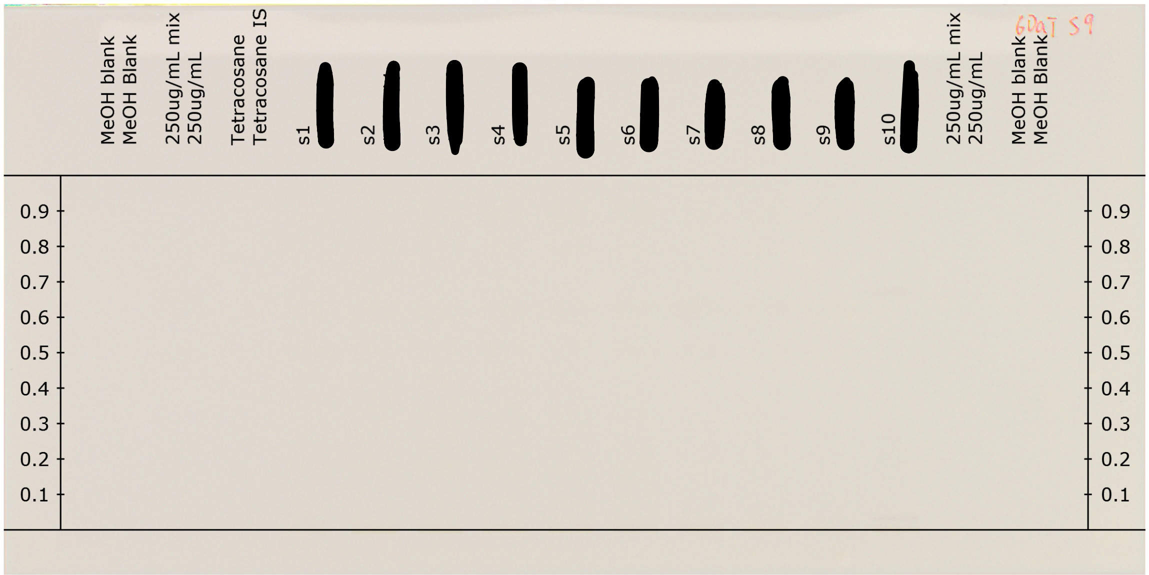

Exposure	0.079 s
Contrast	1
Normalized exposure	Disabled
Clarify	Disabled
White balance	1.00, 1.00, 1.00

R 254 Developed, Remission254

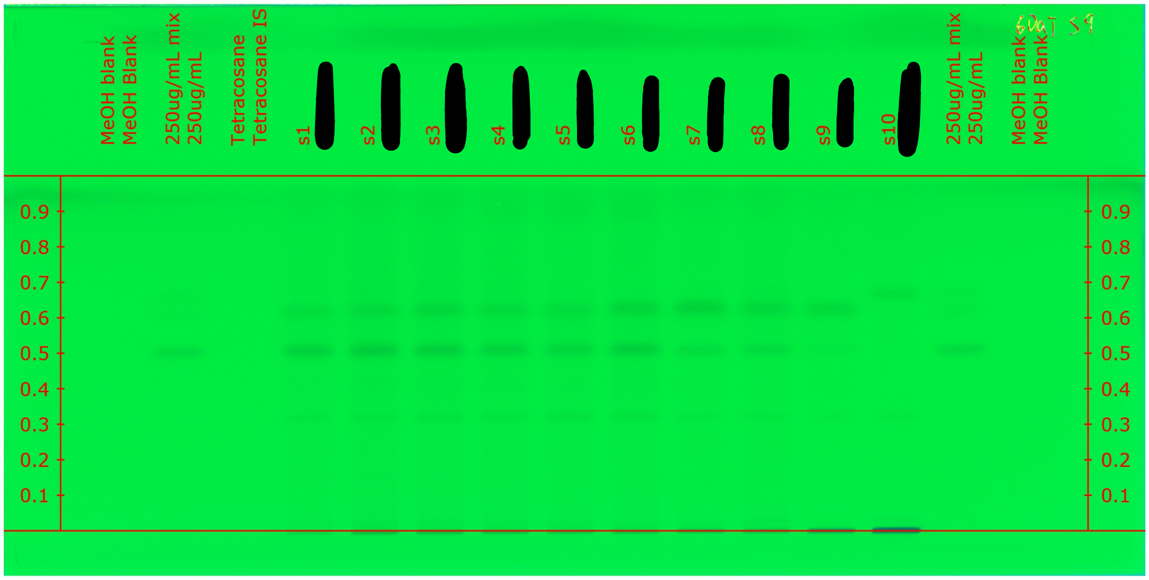

Exposure	0.260 s
Contrast	1
Normalized exposure	Disabled
Clarify	Disabled
White balance	1.00, 1.00, 1.00

R 366

Developed, Remission366

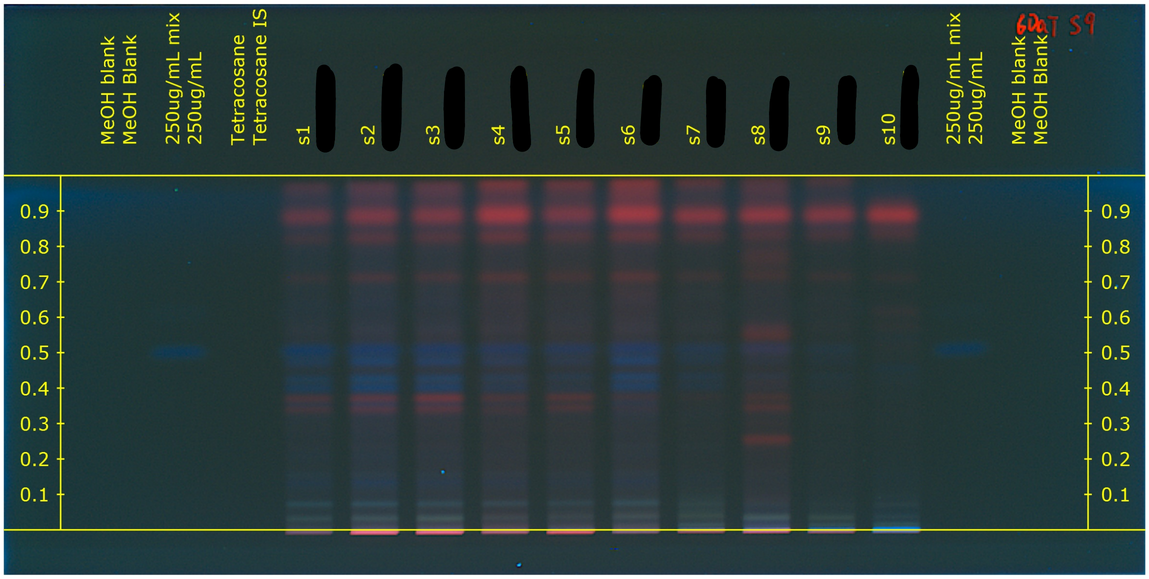

Exposure	3.471 s
Contrast	1
Normalized exposure	Disabled
Clarify	Disabled
White balance	1.00, 1.00, 1.00

Scan developed plate 1b - Scanner 3 (S/N: 031025):

Executed	15-Oct-2019 15:55:49 visionCATSuser

Scan:

Wavelength	254 nm

Track 1:

Type	Single λ

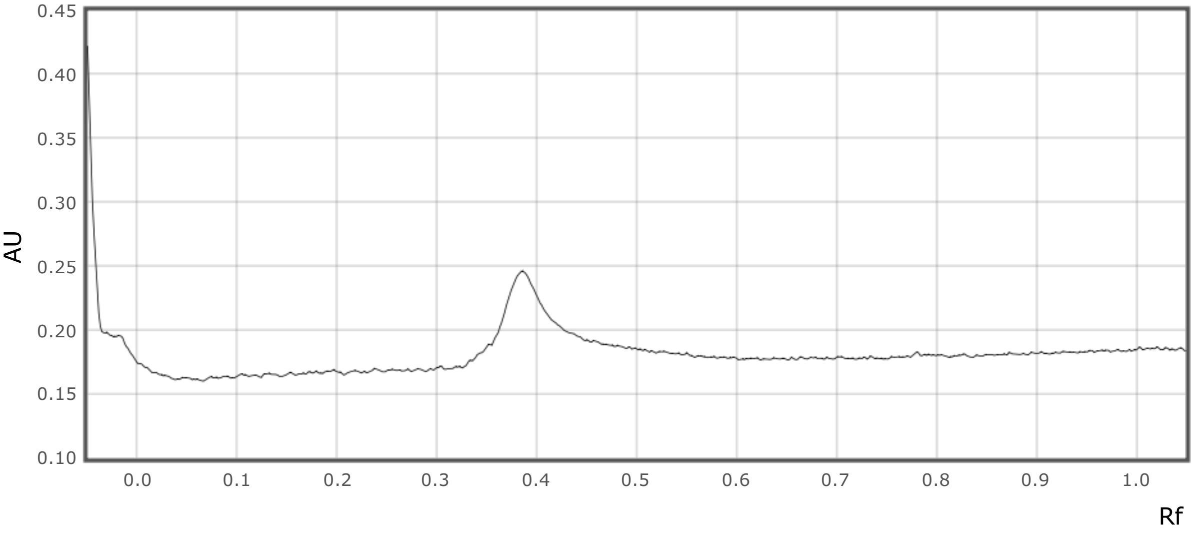

Track 2:

Type	Single λ

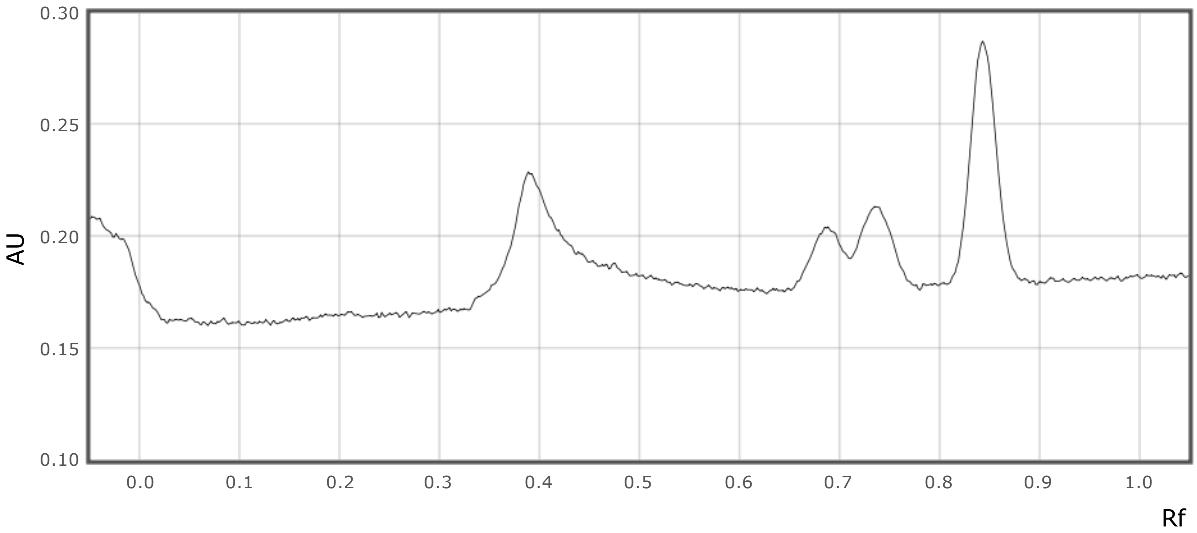

Track 3:

Type	Single λ

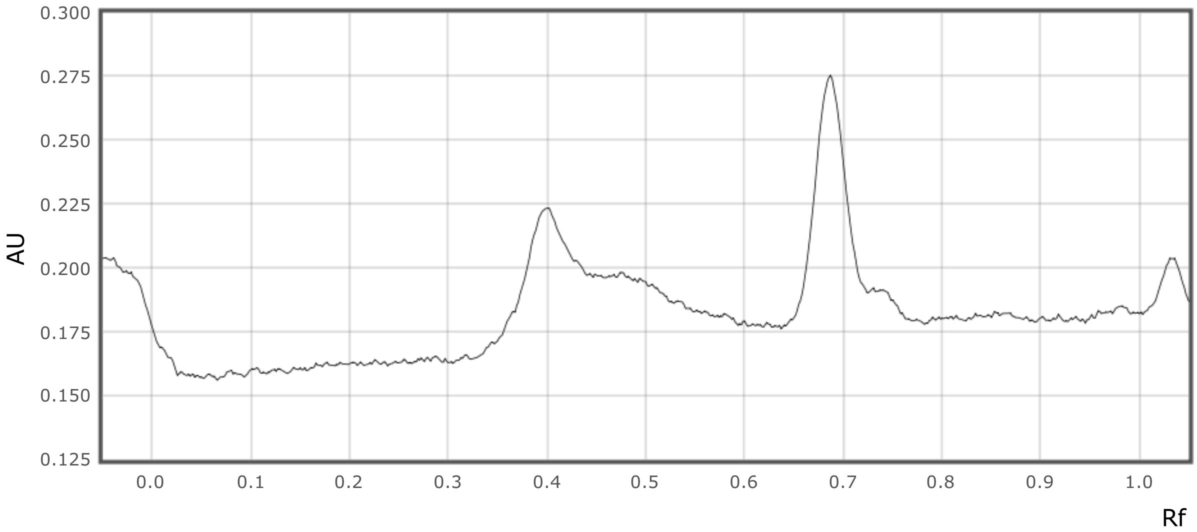

Track 4:

Type	Single λ

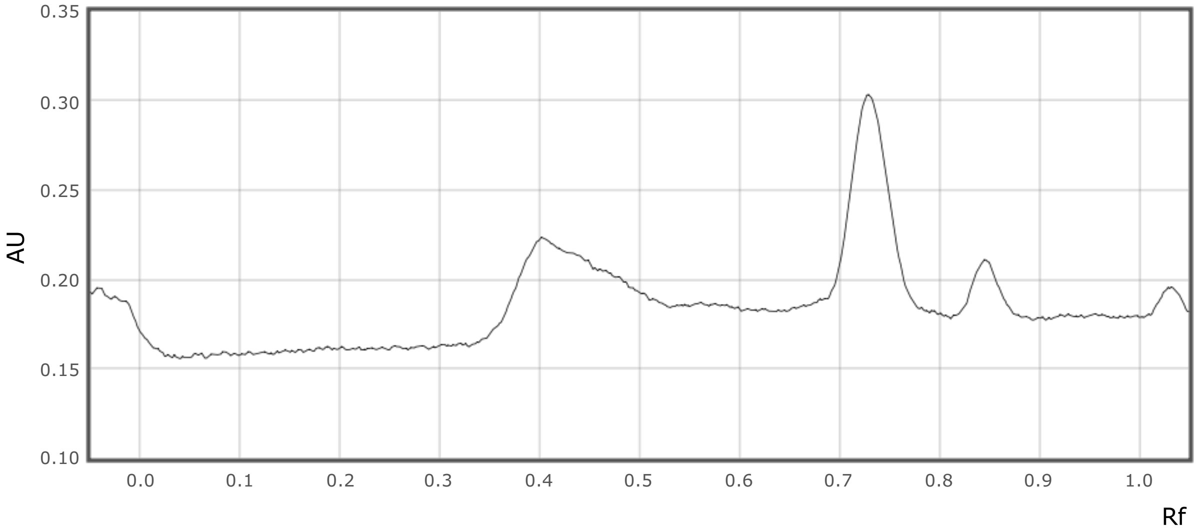

Track 5:

Type	Single λ

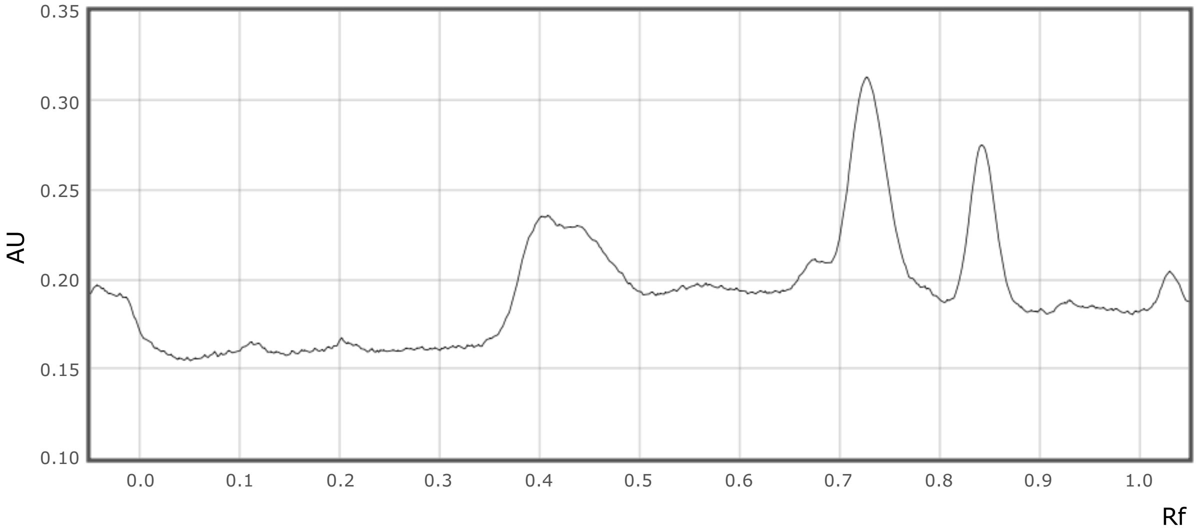

Track 6:

Type	Single λ

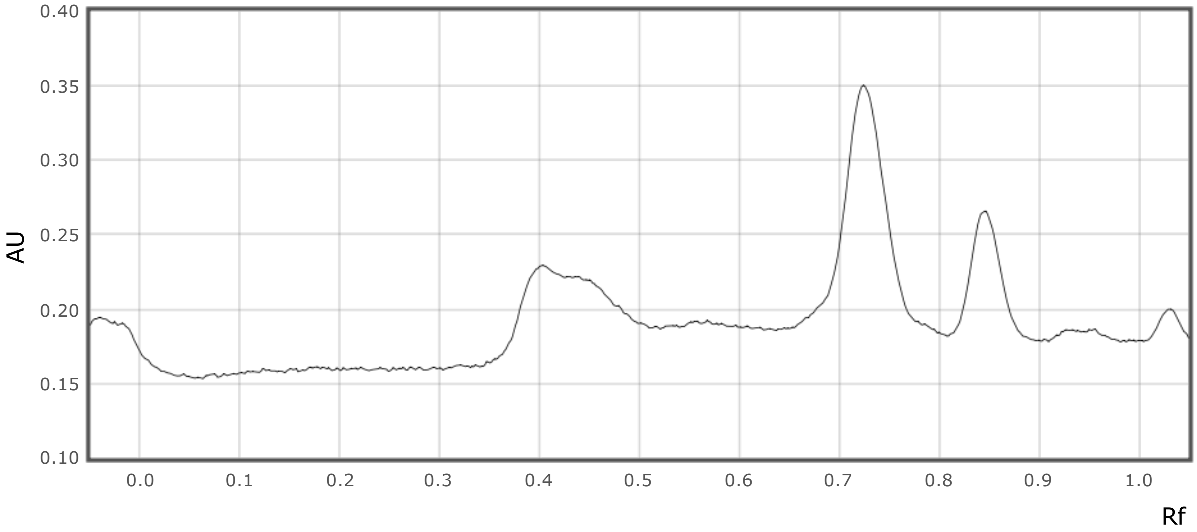

Track 7:

Type	Single λ

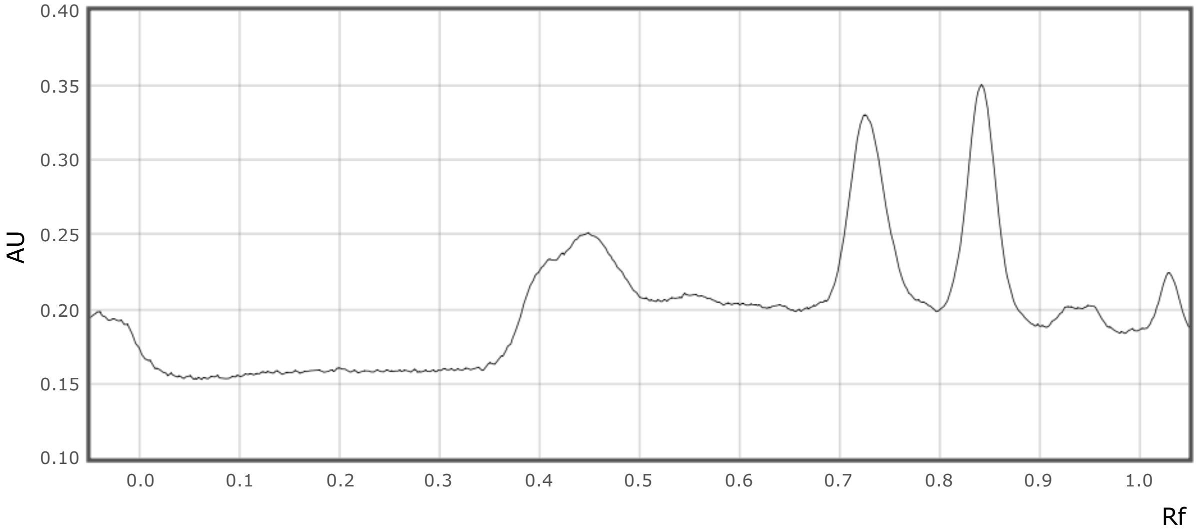

Track 8:

Type	Single λ

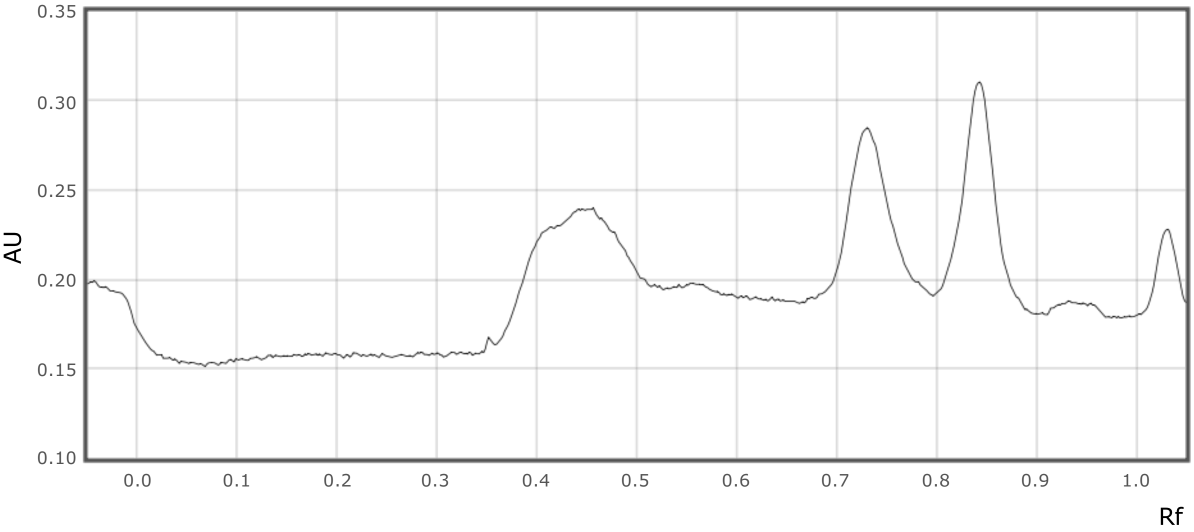

Track 9:	
Type	Single λ

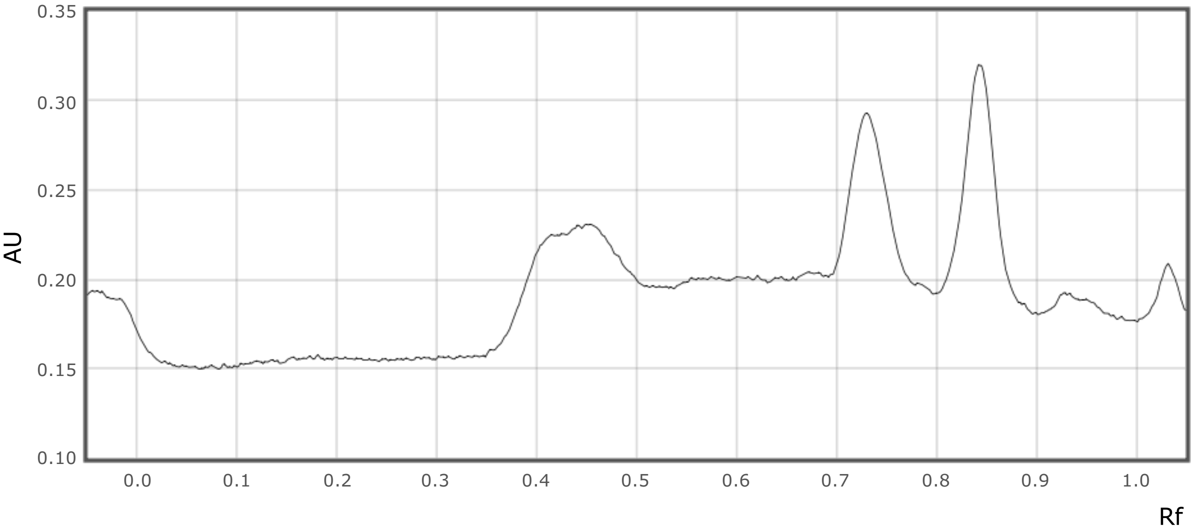

Track 10:	
Type	Single λ

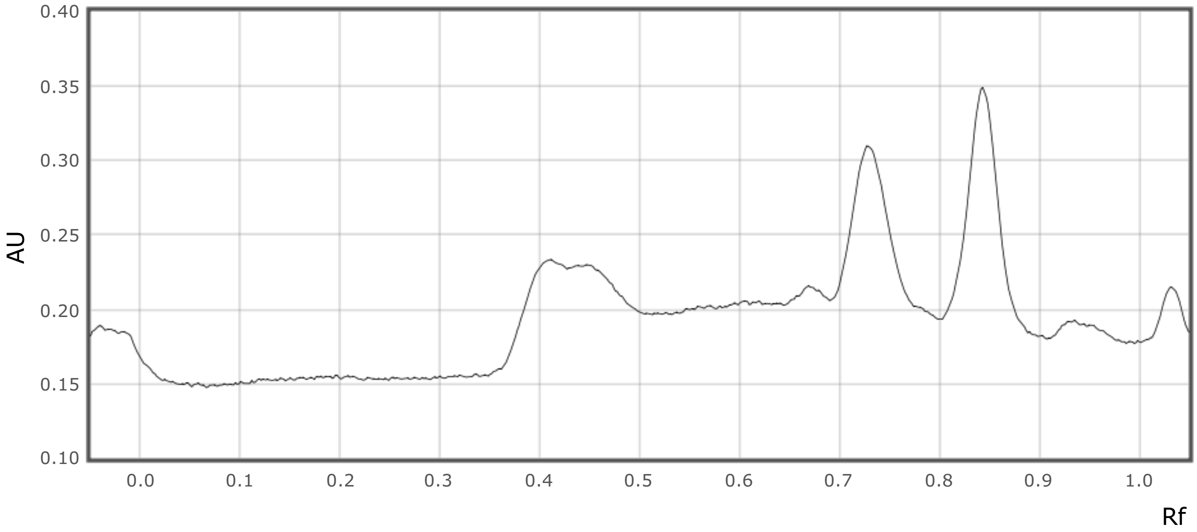

Track 11:	
Type	Single λ

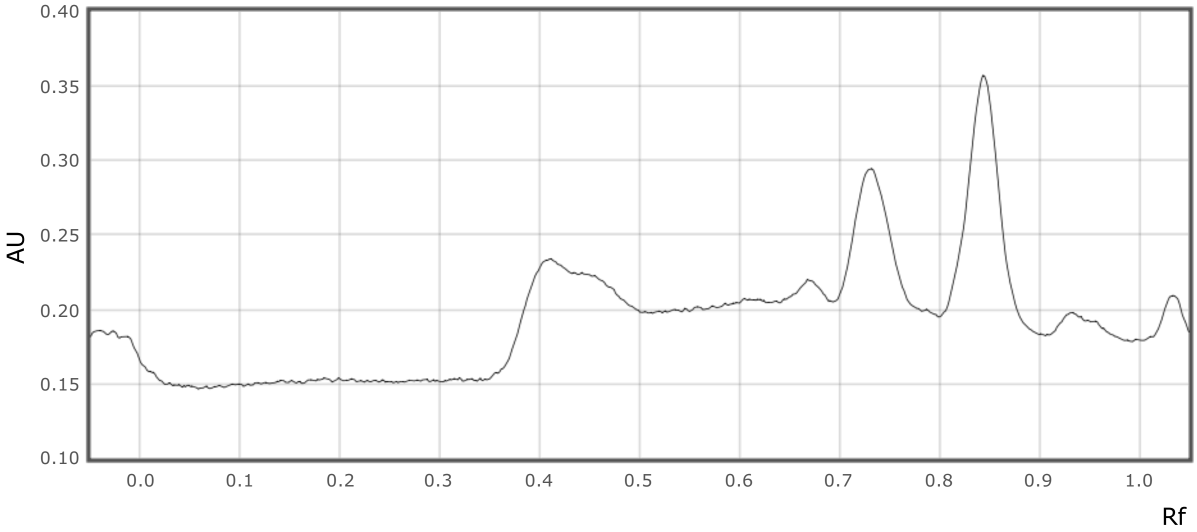

Track 12:	
Type	Single λ

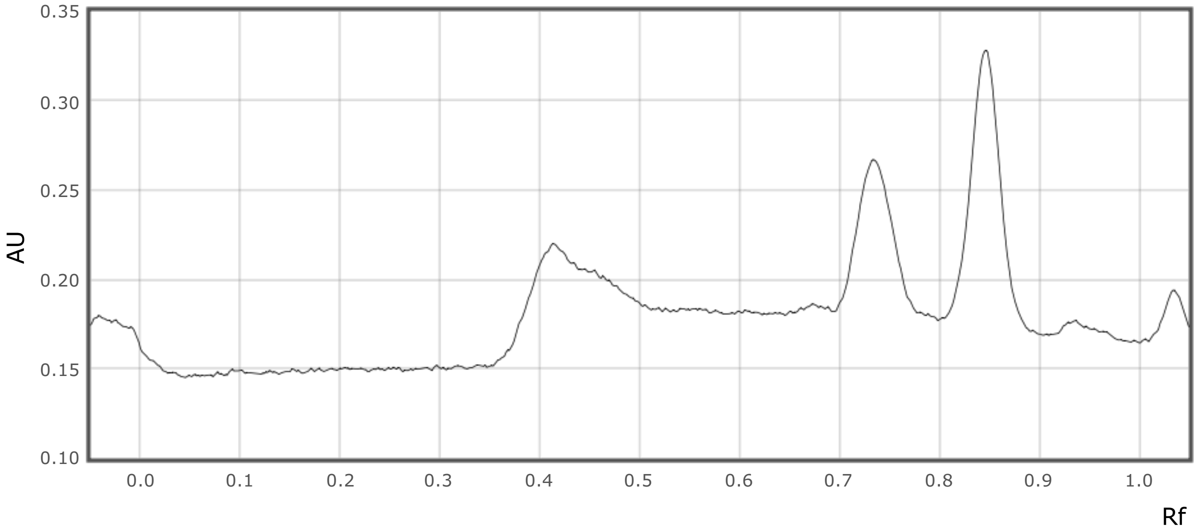

Track 13:	
Type	Single λ

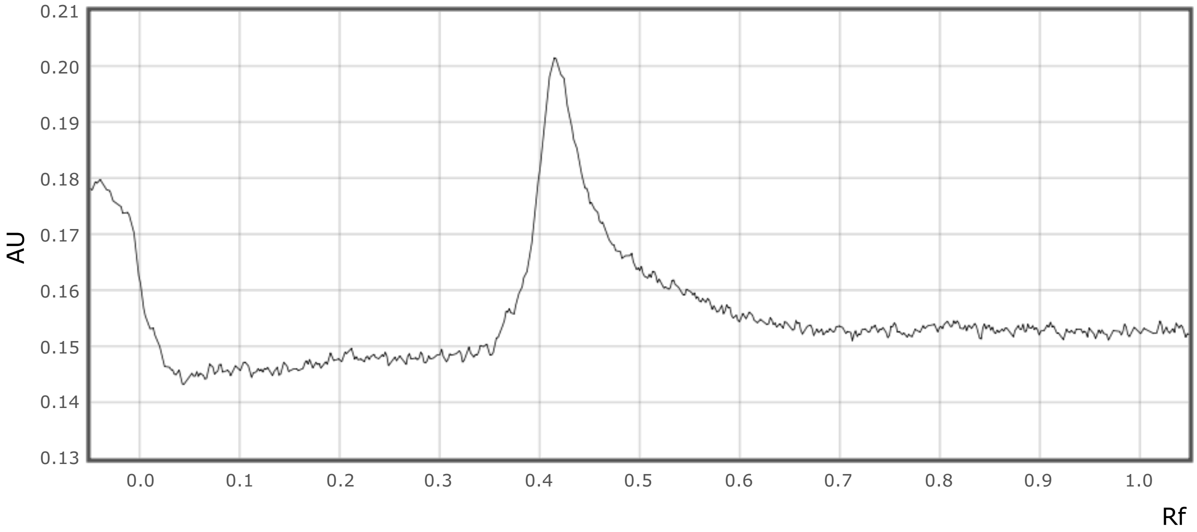

Track 14:	
Type	Single λ

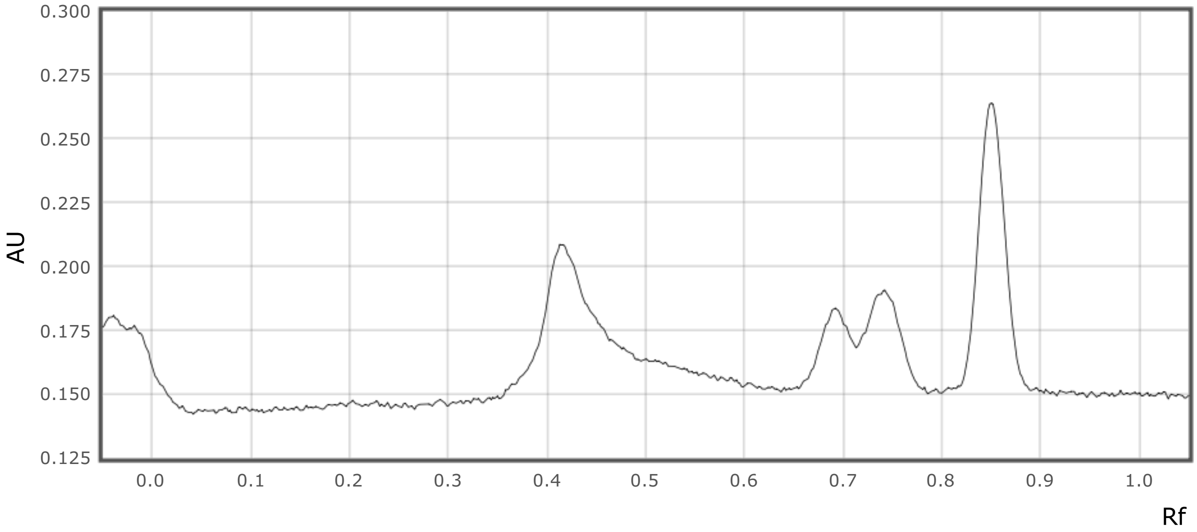

Track 15:	
Type	Single λ

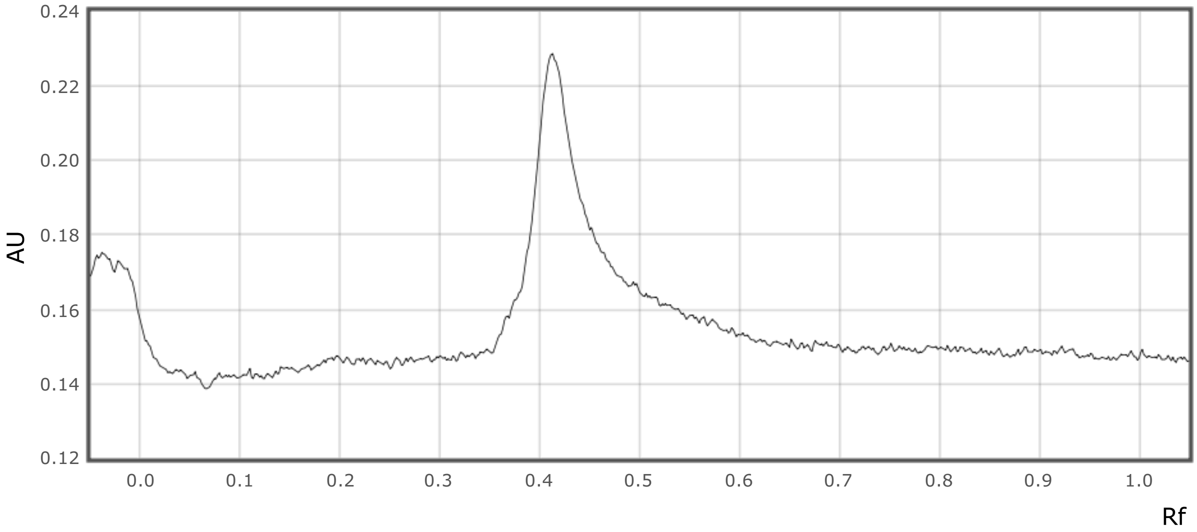

Derivatization 1 - dip:

Executed	15-Oct-2019 15:59:48 visionCATSuser

Take image derivatized plate 1a - Visualizer (S/N: 230515):

Executed	15-Oct-2019 16:06:44 visionCATSuser

RT White

Derivatized, RemTransVis

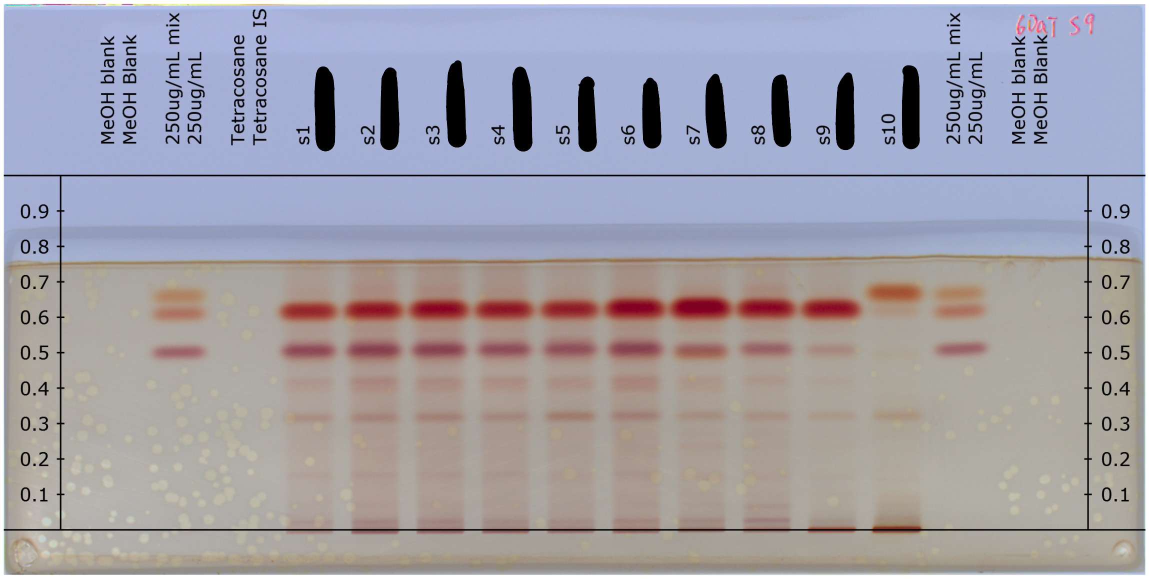

Exposure	0.057 s
Contrast	1
Normalized exposure	Disabled
Clarify	Disabled
White balance	1.28, 1.13, 0.75

R 366

Derivatized, Remission366

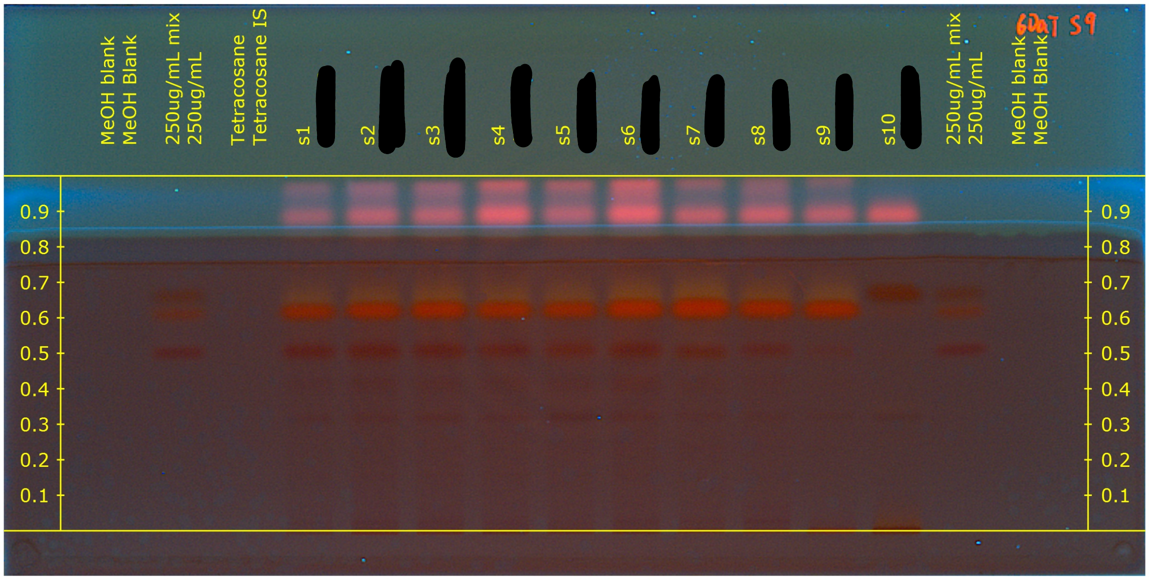

Exposure	9.999 s
Contrast	1
Normalized exposure	Disabled
Clarify	Disabled
White balance	1.00, 1.00, 1.00

Evaluation 1 :

Validated	false
Step	Take image derivatized plate 1a
Concentration unit type	Mass / volume
Notes	

Definition:

References:

250ug/mL mix

Substance Name	Concentration	Purity
9-THC	250.000 µg/ml	100.00 %
CBD	250.000 µg/ml	100.00 %
CBN	250.000 µg/ml	100.00 %

Samples:

Vial ID	Amount	Volume solution	Reference amount	Related to
MeOH blank		0.00 ml		
Tetracosane		0.00 ml		
s1		0.00 ml		
s2		0.00 ml		
s3		0.00 ml		
s4		0.00 ml		
s5		0.00 ml		
s6		0.00 ml		
s7		0.00 ml		
s8		0.00 ml		
s9		0.00 ml		
s10		0.00 ml		

Integration parameters:

Bounds	[0.000,1.000]
Smoothing	Savitzky–Golay of order 3 and window 7
Baseline correction	Lowest slope with noise 0.05
Profile subtraction	Profile subtraction from track 1
Peaks detection	Gauss (legacy) with sensitivity 0.1, separation 1 and threshold 0.1

Scan:

Wavelength	RT White

Track 1:

Type	Sample
Vial ID	MeOH blank
Description	MeOH Blank
Volume	2.0 µl

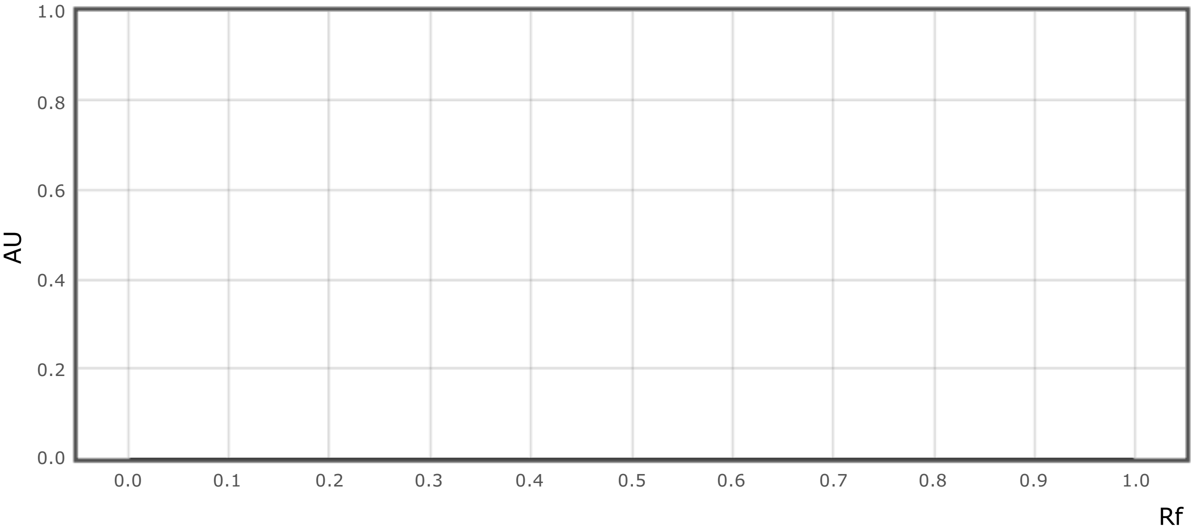

Peak #	Start		Max			End		Area		Manual peak	Substance Name
	Rf	H	Rf	H	%	Rf	H	A	%		

Track 2:

Type	Reference
Vial ID	250ug/mL mix
Description	250ug/mL
Volume	2.0 µl

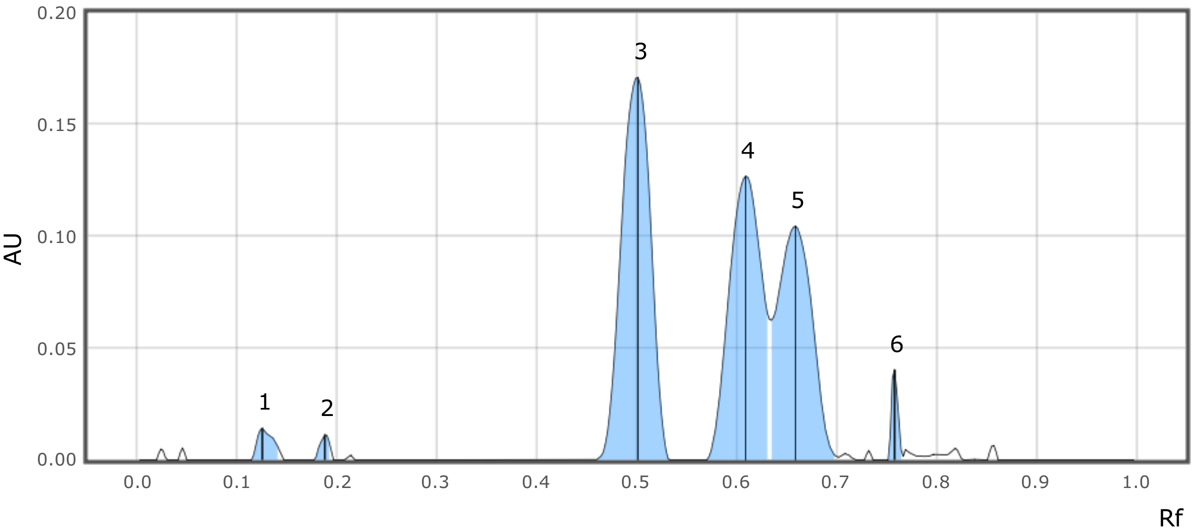

Peak #	Start		Max			End		Area		Manual peak	Substance Name
	Rf	H	Rf	H	%	Rf	H	A	%		
1	0.114	0.0000	0.125	0.0142	3.03	0.147	0.0000	0.00027	1.74	No	
2	0.177	0.0000	0.188	0.0113	2.41	0.196	0.0000	0.00012	0.81	No	
3	0.458	0.0000	0.501	0.1710	36.54	0.533	0.0000	0.00580	37.73	No	
4	0.570	0.0000	0.609	0.1268	27.09	0.633	0.0628	0.00476	30.96	No	9-THC
5	0.635	0.0625	0.659	0.1045	22.32	0.702	0.0012	0.00412	26.79	No	CBD
6	0.752	0.0000	0.758	0.0404	8.62	0.767	0.0017	0.00030	1.97	No	

Track 3:

Type	Sample
Vial ID	Tetracosane
Description	Tetracosane IS
Volume	2.0 µl

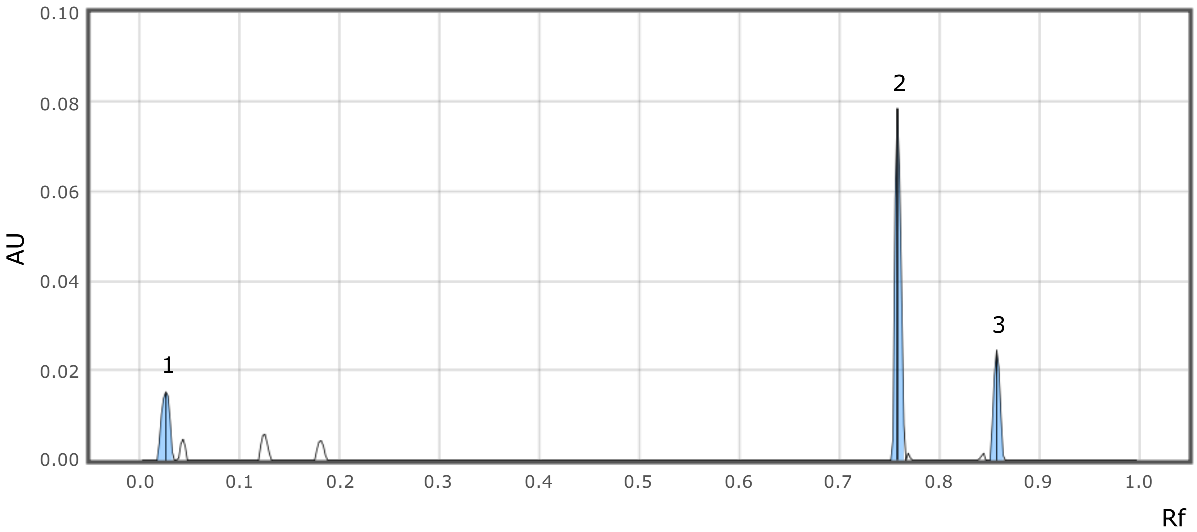

Peak #	Start Rf	Start H	Max Rf	Max H	Max %	End Rf	End H	Area A	Area %	Manual peak	Substance Name
1	0.017	0.0000	0.026	0.0152	12.88	0.034	0.0000	0.00015	16.98	No	
2	0.752	0.0000	0.758	0.0785	66.39	0.767	0.0000	0.00054	62.32	No	
3	0.851	0.0000	0.858	0.0245	20.72	0.866	0.0000	0.00018	20.70	No	

Track 4:

Type	Sample
Vial ID	s1
Description	[redacted]
Volume	2.0 µl

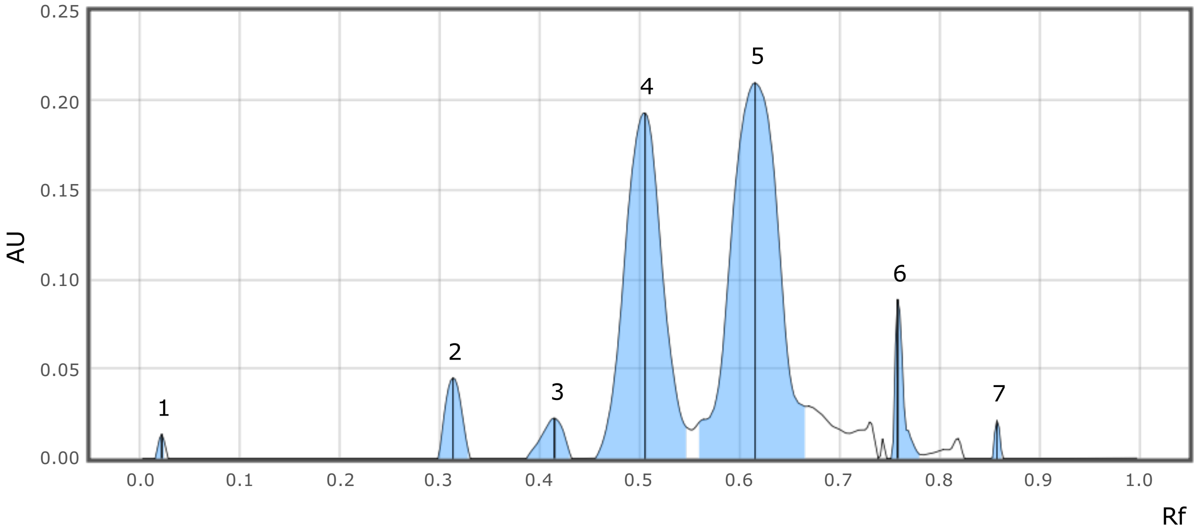

Peak	Start		Max			End		Area		Manual	Substance
#	Rf	H	Rf	H	%	Rf	H	A	%	peak	Name
1	0.015	0.0000	0.021	0.0135	2.28	0.028	0.0000	0.00010	0.43	No	
2	0.298	0.0000	0.313	0.0450	7.58	0.330	0.0000	0.00084	3.71	No	
3	0.387	0.0000	0.415	0.0225	3.79	0.432	0.0000	0.00057	2.50	No	
4	0.454	0.0000	0.505	0.1930	32.50	0.551	0.0160	0.00849	37.52	No	CBN
5	0.559	0.0202	0.616	0.2098	35.32	0.667	0.0289	0.01166	51.50	No	9-THC
6	0.752	0.0000	0.758	0.0889	14.97	0.782	0.0020	0.00086	3.78	No	
7	0.851	0.0000	0.858	0.0212	3.57	0.864	0.0000	0.00013	0.55	No	

Track 5:

Type	Sample
Vial ID	s2
Description	[redacted]
Volume	2.0 µl

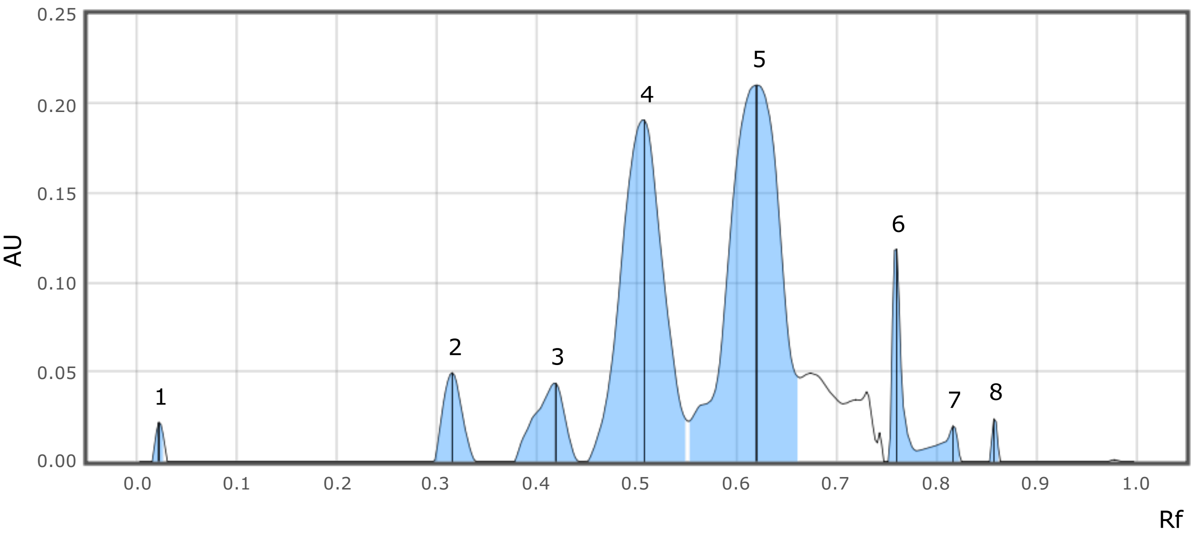

Peak	Start		Max			End		Area		Manual	Substance
#	Rf	H	Rf	H	%	Rf	H	A	%	peak	Name
1	0.015	0.0000	0.021	0.0219	3.22	0.030	0.0000	0.00019	0.73	No	
2	0.296	0.0000	0.315	0.0496	7.32	0.339	0.0000	0.00107	4.03	No	
3	0.378	0.0000	0.419	0.0437	6.44	0.443	0.0000	0.00149	5.61	No	
4	0.449	0.0000	0.508	0.1908	28.13	0.551	0.0225	0.00929	35.06	No	
5	0.553	0.0225	0.620	0.2102	30.99	0.663	0.0467	0.01264	47.71	No	9-THC
6	0.752	0.0000	0.760	0.1185	17.46	0.780	0.0059	0.00123	4.64	No	
7	0.780	0.0059	0.817	0.0198	2.92	0.825	0.0000	0.00045	1.69	No	
8	0.853	0.0000	0.858	0.0238	3.51	0.864	0.0000	0.00014	0.54	No	

Track 6:

Type	Sample
Vial ID	s3
Description	[redacted]
Volume	2.0 µl

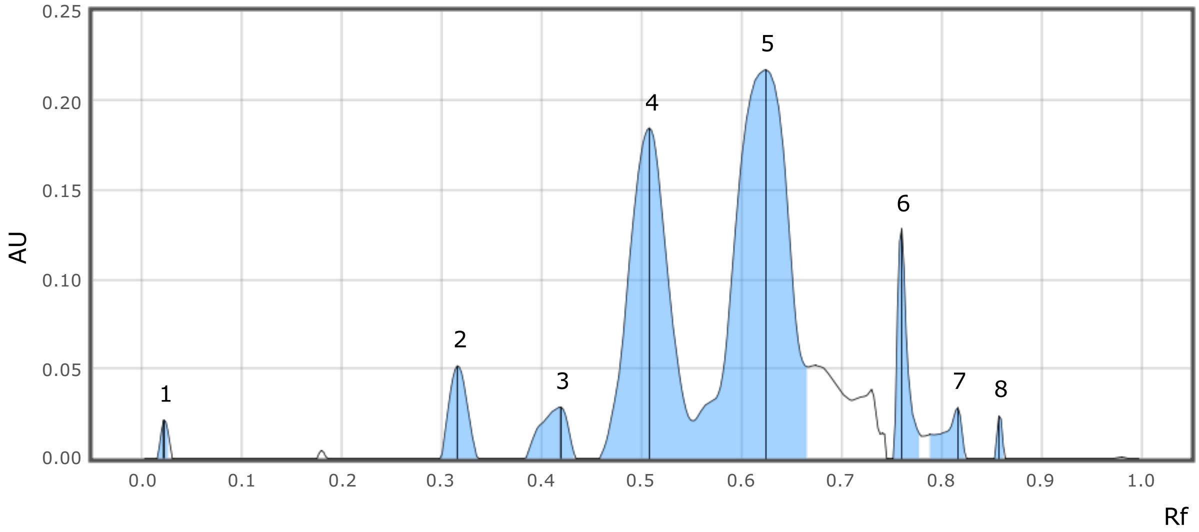

Peak #	Start Rf	Start H	Max Rf	Max H	Max %	End Rf	End H	Area A	Area %	Manual peak	Substance Name
1	0.015	0.0000	0.021	0.0214	3.13	0.030	0.0000	0.00019	0.70	No	
2	0.298	0.0000	0.315	0.0517	7.56	0.337	0.0000	0.00106	4.00	No	
3	0.382	0.0000	0.419	0.0288	4.20	0.434	0.0000	0.00090	3.41	No	
4	0.458	0.0000	0.508	0.1847	26.99	0.551	0.0210	0.00837	31.62	No	
5	0.551	0.0210	0.624	0.2171	31.74	0.667	0.0511	0.01369	51.75	No	9-THC
6	0.752	0.0000	0.760	0.1284	18.76	0.780	0.0124	0.00150	5.67	No	
7	0.786	0.0130	0.817	0.0284	4.14	0.825	0.0000	0.00062	2.33	No	
8	0.853	0.0000	0.858	0.0238	3.48	0.864	0.0000	0.00014	0.53	No	

Track 7:

Type	Sample
Vial ID	s4
Description	[redacted]
Volume	2.0 µl

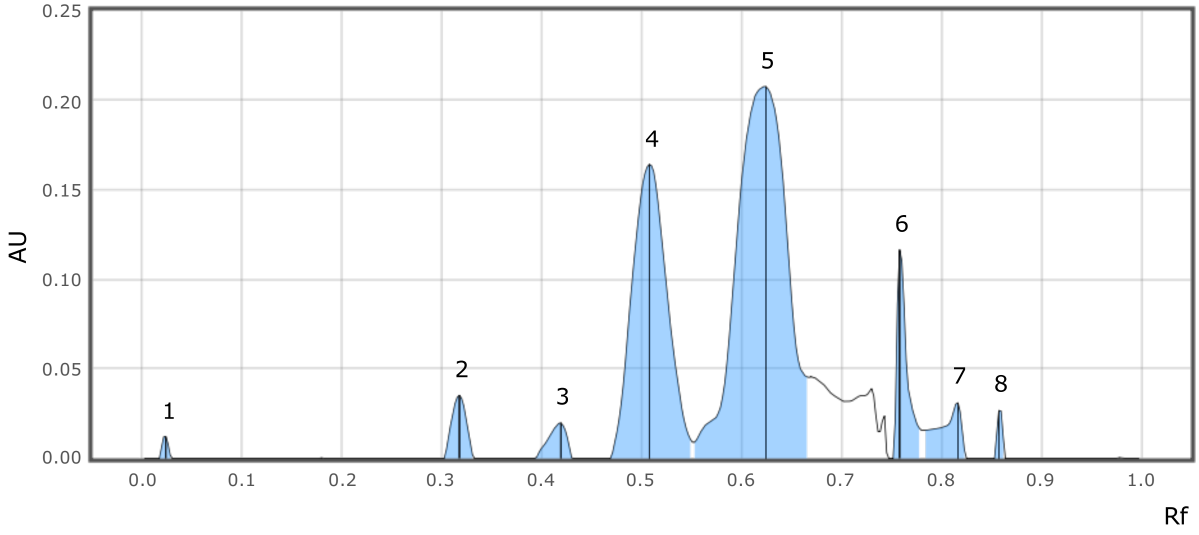

Peak #	Start Rf	Start H	Max Rf	Max H	Max %	End Rf	End H	Area A	Area %	Manual peak	Substance Name
1	0.017	0.0000	0.023	0.0120	1.96	0.030	0.0000	0.00009	0.39	No	
2	0.302	0.0000	0.317	0.0352	5.74	0.333	0.0000	0.00059	2.65	No	
3	0.393	0.0000	0.419	0.0198	3.22	0.432	0.0000	0.00041	1.83	No	
4	0.469	0.0000	0.508	0.1641	26.78	0.551	0.0090	0.00668	29.93	No	
5	0.553	0.0090	0.624	0.2075	33.87	0.667	0.0452	0.01224	54.85	No	9-THC
6	0.752	0.0000	0.758	0.1164	18.99	0.780	0.0158	0.00138	6.20	No	
7	0.784	0.0156	0.817	0.0310	5.07	0.825	0.0000	0.00076	3.40	No	
8	0.853	0.0000	0.858	0.0267	4.36	0.864	0.0000	0.00017	0.75	No	

Track 8:

Type	Sample
Vial ID	s5
Description	[redacted]
Volume	2.0 µl

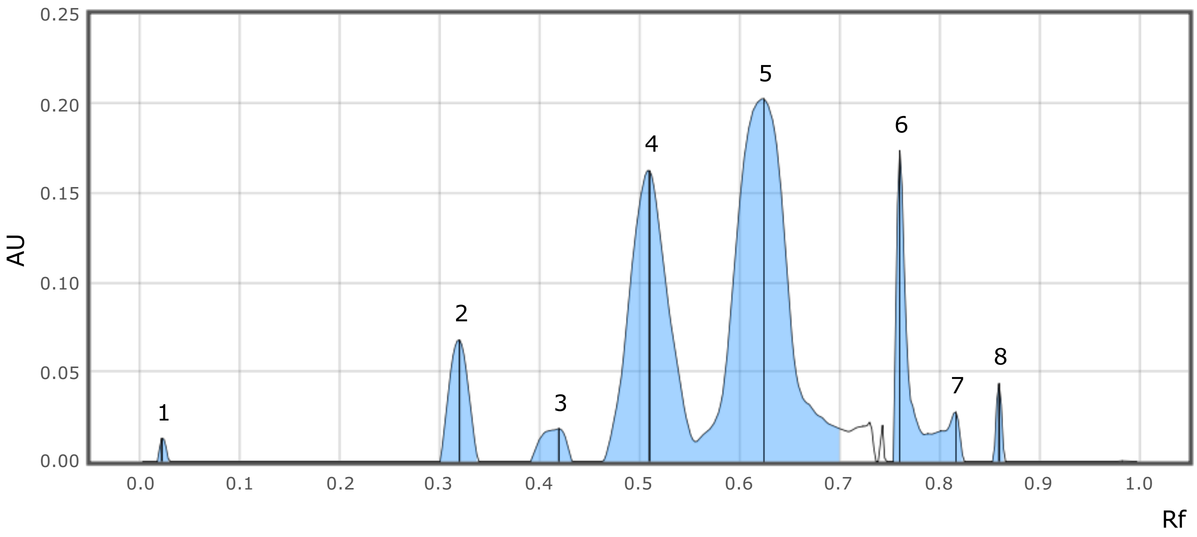

Peak #	Start Rf	Start H	Max Rf	Max H	Max %	End Rf	End H	Area A	Area %	Manual peak	Substance Name
1	0.017	0.0000	0.021	0.0130	1.83	0.030	0.0000	0.00009	0.36	No	
2	0.300	0.0000	0.320	0.0679	9.57	0.339	0.0000	0.00149	6.01	No	
3	0.389	0.0000	0.419	0.0183	2.58	0.434	0.0000	0.00053	2.13	No	
4	0.462	0.0000	0.510	0.1625	22.91	0.555	0.0110	0.00725	29.22	No	
5	0.555	0.0110	0.624	0.2026	28.57	0.706	0.0170	0.01250	50.36	No	9-THC
6	0.754	0.0000	0.760	0.1737	24.50	0.784	0.0149	0.00199	8.02	No	
7	0.784	0.0149	0.817	0.0276	3.89	0.825	0.0000	0.00070	2.81	No	
8	0.853	0.0000	0.860	0.0435	6.14	0.866	0.0000	0.00027	1.09	No	

Track 9:

Type	Sample
Vial ID	s6
Description	[redacted]
Volume	2.0 µl

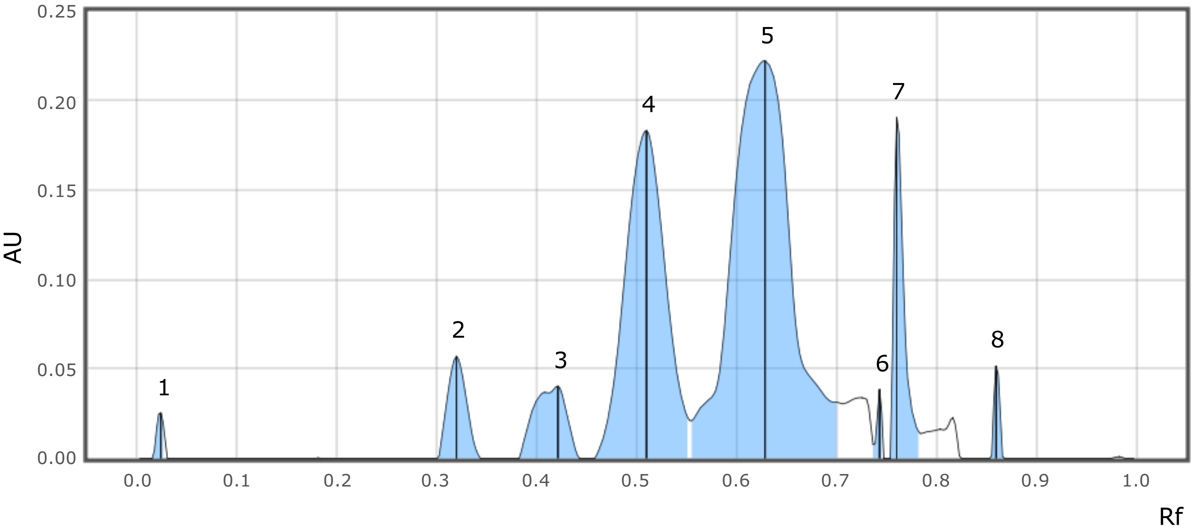

Peak #	Start		Max			End		Area		Manual peak	Substance Name
	Rf	H	Rf	H	%	Rf	H	A	%		
1	0.015	0.0000	0.023	0.0255	3.15	0.030	0.0000	0.00022	0.73	No	
2	0.300	0.0000	0.320	0.0570	7.04	0.345	0.0000	0.00123	4.10	No	
3	0.382	0.0000	0.421	0.0404	4.99	0.443	0.0000	0.00153	5.11	No	
4	0.458	0.0000	0.510	0.1833	22.65	0.553	0.0212	0.00856	28.61	No	
5	0.555	0.0210	0.629	0.2222	27.46	0.704	0.0307	0.01556	52.00	No	9-THC
6	0.737	0.0077	0.743	0.0386	4.77	0.747	0.0000	0.00021	0.71	No	
7	0.754	0.0000	0.760	0.1905	23.54	0.784	0.0138	0.00228	7.61	No	
8	0.853	0.0000	0.860	0.0517	6.39	0.868	0.0000	0.00034	1.13	No	

Track 10:

Type	Sample
Vial ID	s7
Description	[redacted]
Volume	2.0 µl

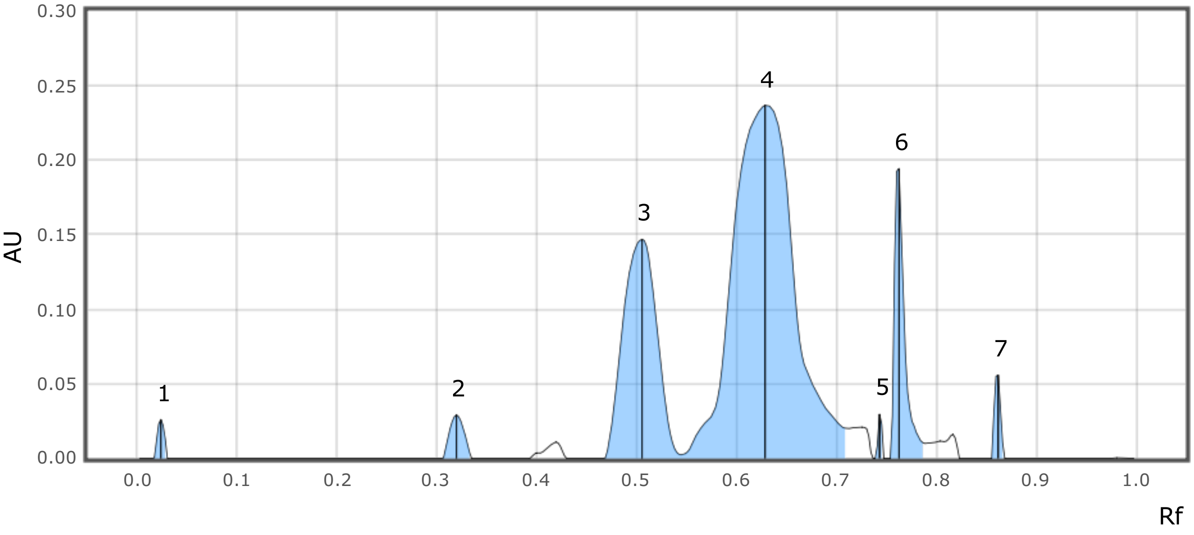

Peak #	Start Rf	Start H	Max Rf	Max H	Max %	End Rf	End H	Area A	Area %	Manual peak	Substance Name
1	0.015	0.0000	0.023	0.0261	3.63	0.030	0.0000	0.00021	0.80	No	
2	0.304	0.0000	0.320	0.0290	4.05	0.335	0.0000	0.00049	1.85	No	
3	0.467	0.0000	0.505	0.1468	20.45	0.544	0.0023	0.00574	21.84	No	CBN
4	0.544	0.0023	0.629	0.2364	32.93	0.711	0.0200	0.01703	64.78	No	9-THC
5	0.739	0.0000	0.743	0.0296	4.12	0.747	0.0000	0.00013	0.50	No	
6	0.754	0.0000	0.763	0.1941	27.05	0.789	0.0100	0.00229	8.70	No	
7	0.856	0.0000	0.862	0.0558	7.77	0.868	0.0000	0.00040	1.54	No	

Track 11:

Type	Sample
Vial ID	s8
Description	[redacted]
Volume	2.0 µl

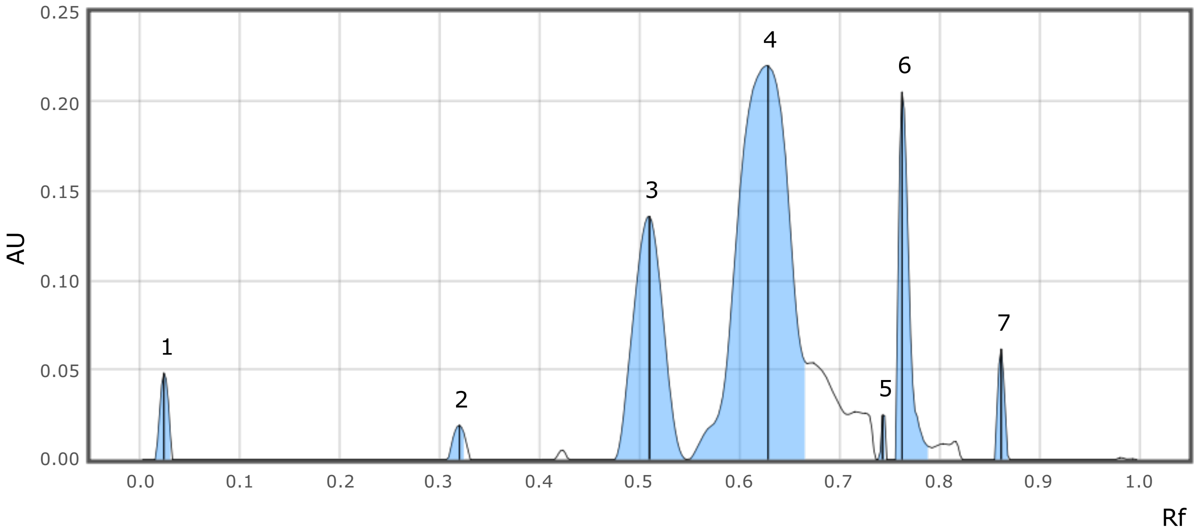

Peak #	Start Rf	Start H	Max Rf	Max H	Max %	End Rf	End H	Area A	Area %	Manual peak	Substance Name
1	0.015	0.0000	0.023	0.0483	6.75	0.032	0.0000	0.00047	2.16	No	
2	0.307	0.0000	0.320	0.0191	2.67	0.330	0.0000	0.00026	1.21	No	
3	0.475	0.0000	0.510	0.1359	19.00	0.546	0.0000	0.00461	21.29	No	
4	0.549	0.0000	0.629	0.2201	30.77	0.667	0.0535	0.01323	61.12	No	9-THC
5	0.739	0.0000	0.743	0.0248	3.47	0.747	0.0000	0.00012	0.54	No	
6	0.756	0.0000	0.763	0.2054	28.71	0.793	0.0065	0.00250	11.55	No	
7	0.856	0.0000	0.862	0.0617	8.63	0.871	0.0000	0.00046	2.13	No	

Track 12:

Type	Sample
Vial ID	s9
Description	[redacted]
Volume	2.0 µl

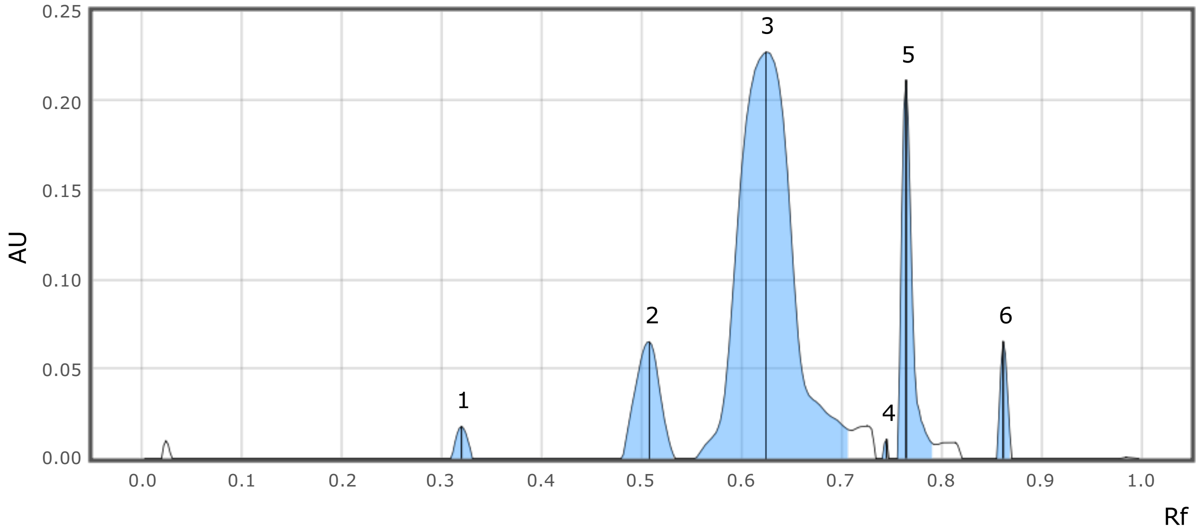

Peak #	Start		Max			End		Area		Manual peak	Substance Name
	Rf	H	Rf	H	%	Rf	H	A	%		
1	0.309	0.0000	0.320	0.0179	3.00	0.333	0.0000	0.00023	1.19	No	
2	0.479	0.0000	0.508	0.0652	10.91	0.533	0.0000	0.00184	9.47	No	
3	0.553	0.0000	0.624	0.2269	37.96	0.709	0.0156	0.01422	73.06	No	9-THC
4	0.741	0.0000	0.745	0.0107	1.79	0.747	0.0000	0.00004	0.21	No	
5	0.756	0.0000	0.765	0.2114	35.38	0.793	0.0077	0.00259	13.28	No	
6	0.856	0.0000	0.862	0.0655	10.95	0.871	0.0000	0.00054	2.80	No	

Track 13:

Type	Sample
Vial ID	s10
Description	[redacted]
Volume	2.0 µl

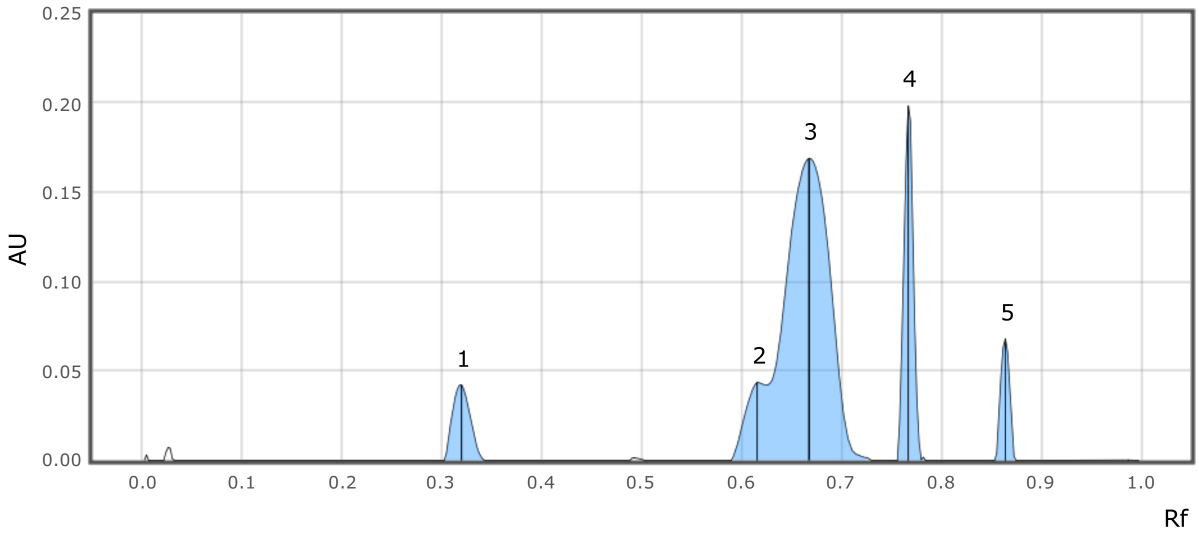

Peak #	Start Rf	Start H	Max Rf	Max H	Max %	End Rf	End H	Area A	Area %	Manual peak	Substance Name
1	0.302	0.0000	0.320	0.0421	8.10	0.343	0.0000	0.00087	6.48	No	
2	0.592	0.0022	0.616	0.0435	8.36	0.626	0.0421	0.00106	7.90	Yes	9-THC
3	0.626	0.0421	0.667	0.1687	32.42	0.730	0.0000	0.00858	63.97	No	CBD
4	0.756	0.0000	0.767	0.1982	38.07	0.784	0.0000	0.00219	16.30	No	
5	0.853	0.0000	0.864	0.0679	13.04	0.875	0.0000	0.00072	5.35	No	

Track 14:

Type	Reference
Vial ID	250ug/mL mix
Description	250ug/mL
Volume	2.0 µl

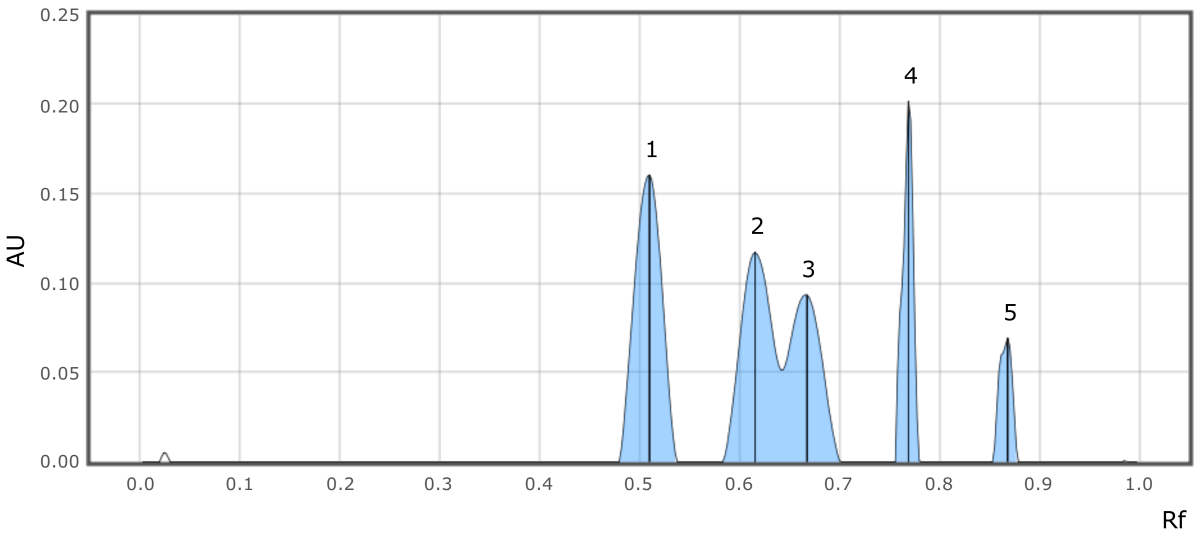

Peak #	Start Rf	Start H	Max Rf	Max H	Max %	End Rf	End H	Area A	Area %	Manual peak	Substance Name
1	0.479	0.0000	0.510	0.1604	25.00	0.538	0.0000	0.00511	30.96	No	
2	0.583	0.0000	0.616	0.1171	18.25	0.642	0.0512	0.00427	25.90	No	9-THC
3	0.642	0.0512	0.667	0.0933	14.54	0.702	0.0000	0.00357	21.65	No	CBD
4	0.756	0.0000	0.769	0.2016	31.42	0.782	0.0000	0.00248	15.02	No	
5	0.853	0.0000	0.868	0.0692	10.79	0.879	0.0000	0.00107	6.46	No	

Track 15:

Type	Sample
Vial ID	MeOH blank
Description	MeOH Blank
Volume	2.0 µl

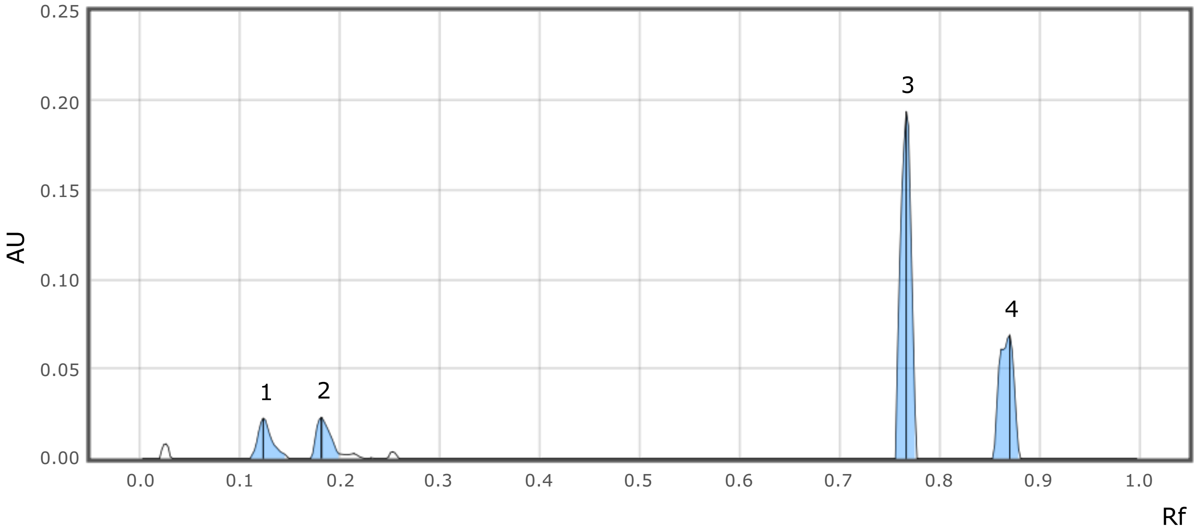

Peak #	Start		Max			End		Area		Manual peak	Substance Name
	Rf	H	Rf	H	%	Rf	H	A	%		
1	0.110	0.0000	0.123	0.0225	7.29	0.149	0.0000	0.00036	8.24	No	
2	0.170	0.0000	0.181	0.0230	7.46	0.207	0.0020	0.00039	8.91	No	
3	0.756	0.0000	0.767	0.1938	62.87	0.778	0.0000	0.00241	55.79	No	
4	0.853	0.0000	0.871	0.0690	22.38	0.881	0.0000	0.00117	27.06	No	

Calibration results:

Height calibration for substance 9-THC @ RT White:

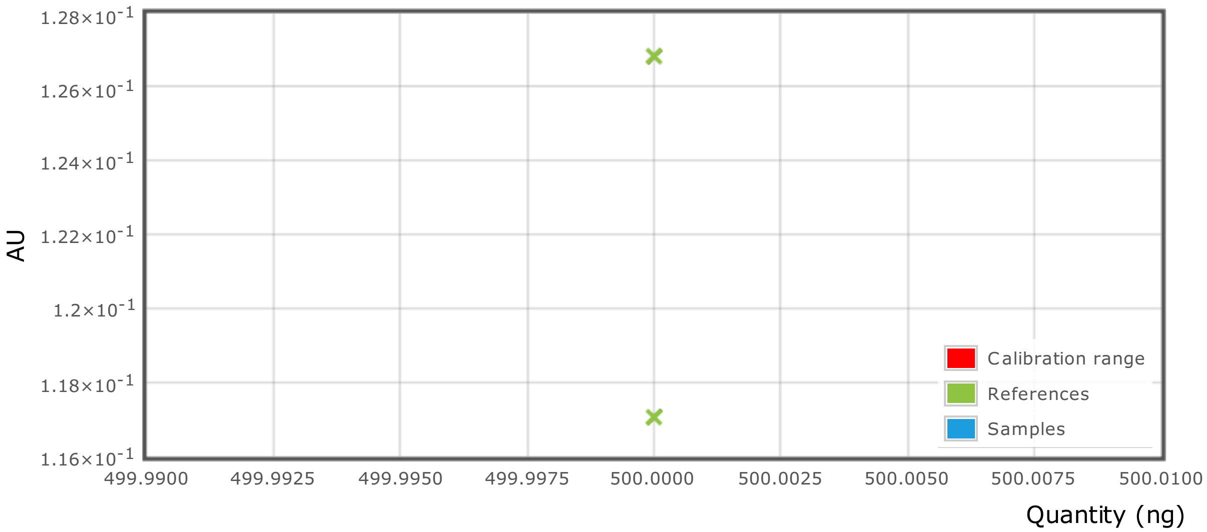

Regression mode	Linear-2
Range deviation	5.00 %
Related substances	Default
Number of references	2
Calibration function	$y=0x$
Coefficient of variation	CV 0.00 %
Correlation coefficient	n/a
⚠	Unable to compute the results for this substance because there wasn't enough groups of references replicas (at least 1 for Linear-1, 2 for Linear2 and Mime-1 and 3 for Polynomial and MiMe-2)

Height calibration for substance CBD @ RT White:

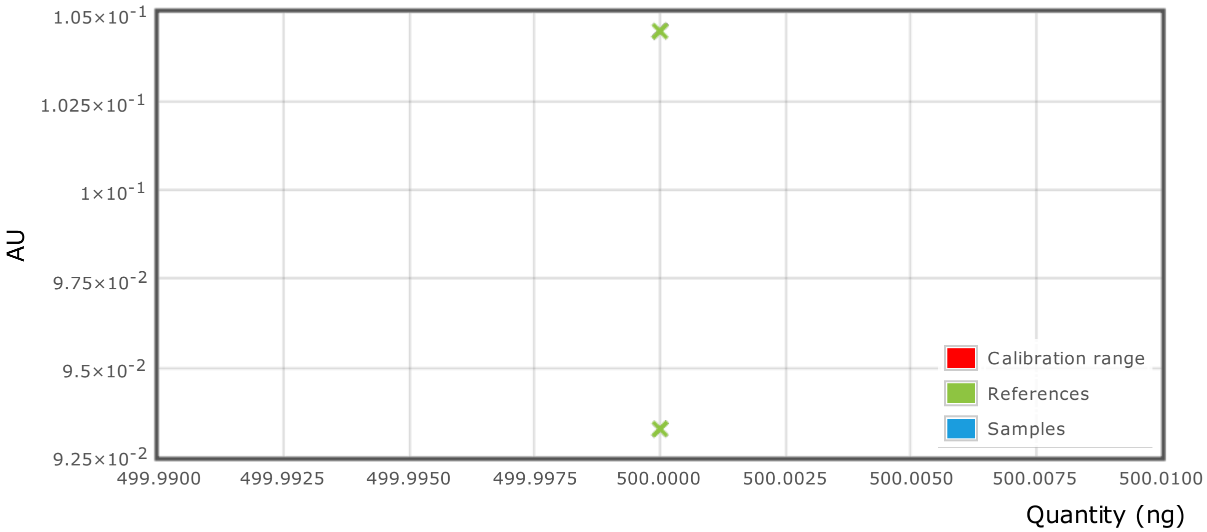

Regression mode	Linear-2
Range deviation	5.00 %
Related substances	Default
Number of references	2
Calibration function	$y=0x$
Coefficient of variation	CV 0.00 %
Correlation coefficient	n/a
⚠	Unable to compute the results for this substance because there wasn't enough groups of references replicas (at least 1 for Linear-1, 2 for Linear2 and Mime-1 and 3 for Polynomial and MiMe-2)

Height calibration for substance CBN @ RT White:

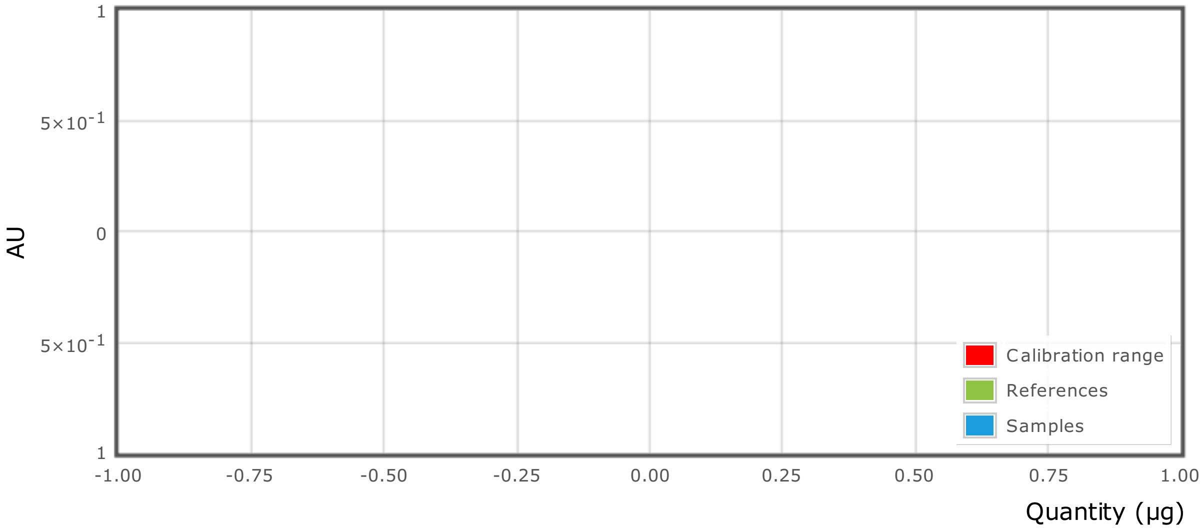

Regression mode	Linear-2
Range deviation	5.00 %
Related substances	Default
Number of references	0
Calibration function	$y=0x$
Coefficient of variation	CV 0.00 %
Correlation coefficient	n/a
⚠	There wasn't any reference application available in the assignments for this substance. Please check that the peaks were correctly detected and assigne d for this substance.

Results:

Substance having no available results		
⚠	9-THC	Unable to compute the results for this substance because there wasn't en ough groups of references replicas (at least 1 for Linear-1, 2 for Linear2 a nd Mime-1 and 3 for Polynomial and MiMe-2)
⚠	CBN	There wasn't any reference application available in the assignments for thi s substance. Please check that the peaks were correctly detected and assi gned for this substance.
⚠	CBD	Unable to compute the results for this substance because there wasn't en ough groups of references replicas (at least 1 for Linear-1, 2 for Linear2 a nd Mime-1 and 3 for Polynomial and MiMe-2)

A track marked with ⚠ means: this result is outside the regression range given by the reference assignments, but is included in the results because it is in the allowed range deviation.

Analyst:	Reviewer:
