# Supplementary material for: High Performance Thin-Layer Chromatography (HPTLC) data of Cannabinoids in ten mobile phase systems
Source: Data Brief. 2020 Jun 30;31:105955. doi: 10.1016/j.dib.2020.105955 (PMC7352075; doi:10.1016/j.dib.2020.105955)
Supplement: Supplementary file 1 [file mmc1.zip › S4-Case sample reports/XHDa-sample run-4.pdf]

Analysis: XHDa-sample run-4

Path: Home/YL Research

Based on method: Samples (no cal)

Created	11-Oct-2019 14:20:26	visionCATSuser
Modified	11-Oct-2019 16:59:39	visionCATSuser
Last HPTLC log	11-Oct-2019 16:59:39	Analysis modified
Explorer notes		

Track	Vial ID	Description	Volume	Position	Type
1	MeOH blank	MeOH Blank	2.0 µl	A1	Sample
2	250ug/mL mix	250ug/mL	2.0 µl	A2	Reference
3	Tetracosane	Tetracosane IS	2.0 µl	A3	Sample
4	s1	[redacted]	2.0 µl	B1	Sample
5	s2	[redacted]	2.0 µl	B2	Sample
6	s3	[redacted]	2.0 µl	B3	Sample
7	s4	[redacted]	2.0 µl	B4	Sample
8	s5	[redacted]	2.0 µl	B5	Sample
9	s6	[redacted]	2.0 µl	B6	Sample
10	s7	[redacted]	2.0 µl	B7	Sample
11	s8	[redacted]	2.0 µl	B8	Sample
12	s9	[redacted]	2.0 µl	B9	Sample
13	s10	[redacted]	2.0 µl	B10	Sample
14	250ug/mL mix	250ug/mL	2.0 µl	A2	Reference
15	MeOH blank	MeOH Blank	2.0 µl	A1	Sample
Sequence table notes					

A track marked with ⚠ means: the application type is overridden in some evaluation(s).

System setup:

Software	Server User-PC, version 2.5.18072.1
ATS4	S/N:080713
Chamber	N/A
Derivatization dip	N/A
Scanner3	S/N:031025
Visualizer	S/N:230515

Chromatography

Plate layout:

Stationary phase	Merck, HPTLC plates silica gel 60 F 254
Plate format	200.0 x 100.0 mm
Application type	Band
Application	Position Y: 8.0 mm, length: 8.0 mm, width: 0.0 mm
Track	First position X: 20.0 mm, distance: 11.4 mm
Solvent front position	70.0 mm
Notes	

Take image clean plate 1a - Visualizer (S/N: 230515):

Quality	Enhanced
RT White	auto capture, Auto, level 85 %, Band
R 254	auto capture, Auto, level 85 %, Band
Instrument diagnostics	Valid diagnostics
Documentation step label	
Notes	

Application 1 - ATS 4 (S/N: 080713):

Spray gas	NI
Sample solvent type	Methanol
Filling speed	15 µl/s
Predosage volume	200 nl
Retraction volume	200 nl
Dosage speed	150 nl/s
Filling quality	User
Rinsing cycles / vacuum	2 / 4 s
Filling cycles / vacuum	1 / 4 s
Rinsing solvent name	Methanol
Nozzle temperature	Unheated
Rack in use	Standard
Instrument diagnostics	Valid diagnostics
Notes	

Development 1 - Chamber:

Tank	TTC 20x10
Mobile phase	Xylene:hexane:diethylamine (25:10:1)
Saturation time	20 min
Use saturation pad	true
Use smartALERT	false
Volume front through	10 ml
Volume rear through	25 ml
Drying time	5 min
Drying temperature	Room temperature
Notes	

Take image developed plate 1a - Visualizer (S/N: 230515):

Quality	Enhanced
RT White	auto capture, Auto, level 85 %, Band
R 254	auto capture, Auto, level 85 %, Band
R 366	auto capture, Auto, level 85 %, Band
Instrument diagnostics	Valid diagnostics
Documentation step label	
Notes	

Scan developed plate 1b - Scanner 3 (S/N: 031025):

Scanner type	Single λ
Optimization for	Resolution
Measurement mode	Absorption
Filter	n/a
Detector mode	Automatic
Scanning speed	20 mm/s
Data resolution	100 µm/step
Slit	5 x 0.2 mm, micro
Partial scan	No
Lamp	Deuterium & Tungsten
Wavelength(s)	254 nm
Instrument diagnostics	Valid diagnostics
Documentation step label	
Notes	

Derivatization 1 - dip:

Reagent name	
Dipping speed	5
Dipping time	0 s
Reagent preparation	
Heating	100 °C for 3 min, heated after
Notes	

Take image derivatized plate 1a - Visualizer (S/N: 230515):

Quality	Enhanced
RT White	auto capture, Auto, level 85 %, Band
R 366	auto capture, Auto, level 85 %, Band
Instrument diagnostics	Valid diagnostics
Documentation step label	
Notes	

System suitability tests:

SST settings:

SST tracks	

Data acquisition

Application 1 - ATS 4 (S/N: 080713):

Executed	11-Oct-2019 14:55:24 visionCATSuser

Development 1 - Chamber:

Executed	11-Oct-2019 15:30:51 visionCATSuser

Take image developed plate 1a - Visualizer (S/N: 230515):

Executed	11-Oct-2019 16:25:17 visionCATSuser

RT White Developed, RemTransVis

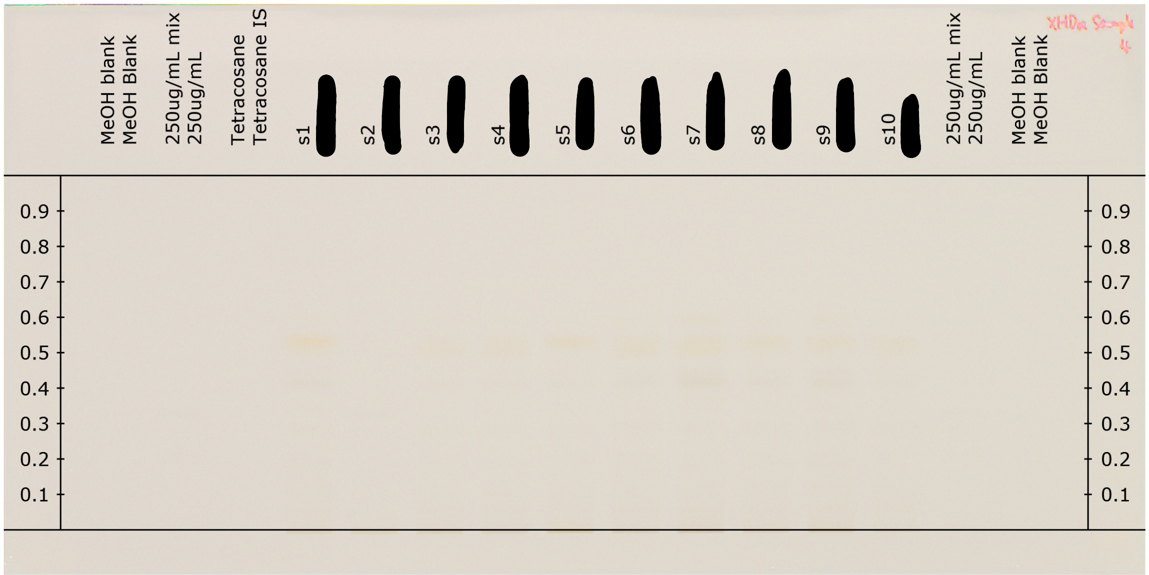

Exposure	0.081 s
Contrast	1
Normalized exposure	Disabled
Clarify	Disabled
White balance	1.00, 1.00, 1.00

R 254 Developed, Remission254

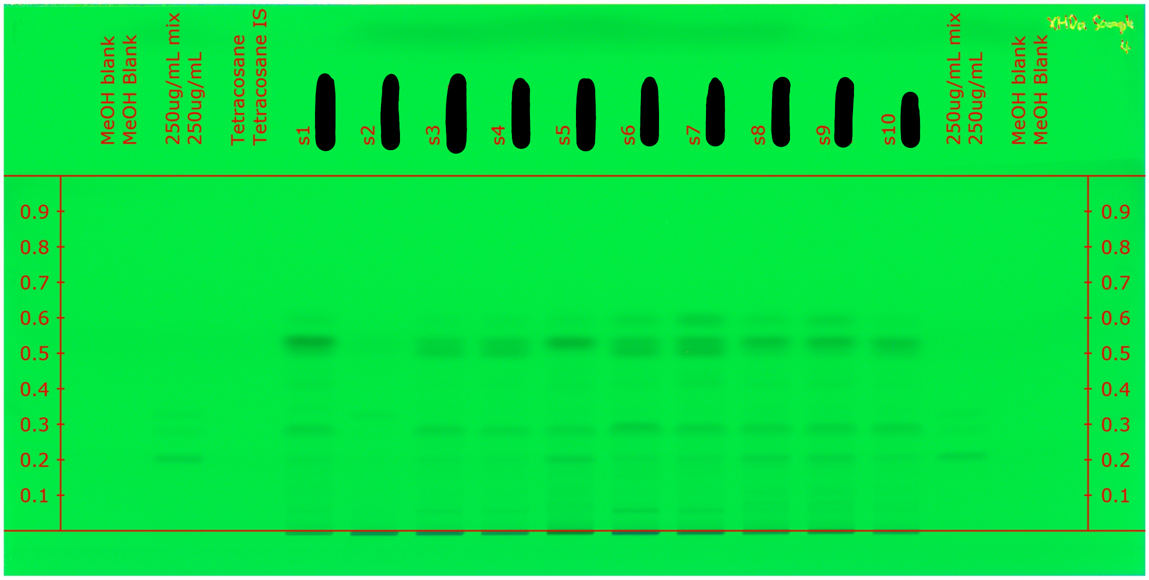

Exposure	0.270 s
Contrast	1
Normalized exposure	Disabled
Clarify	Disabled
White balance	1.00, 1.00, 1.00

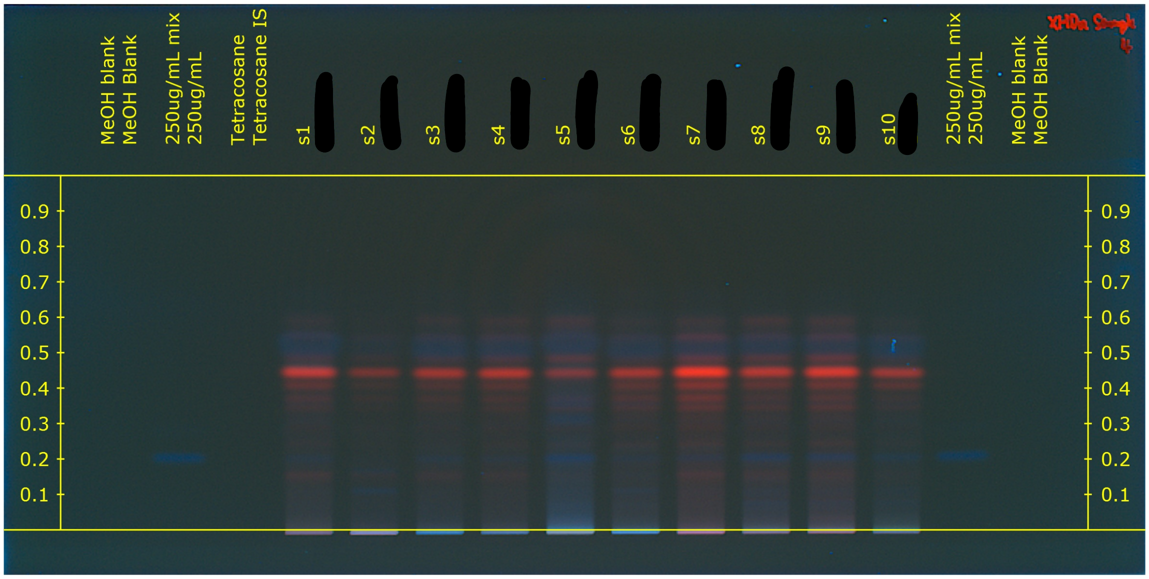

Exposure	3.722 s
Contrast	1
Normalized exposure	Disabled
Clarify	Disabled
White balance	1.00, 1.00, 1.00

Scan developed plate 1b - Scanner 3 (S/N: 031025):

Executed	11-Oct-2019 16:26:41 visionCATSuser

Scan:

Wavelength	254 nm

Track 1:

Type	Single λ

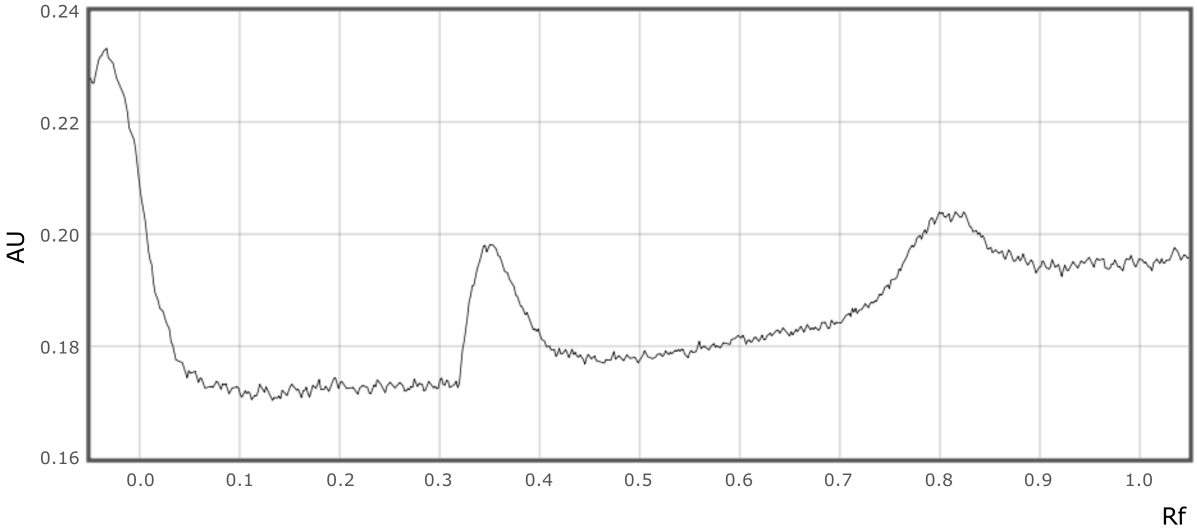

Track 2:

Type	Single λ

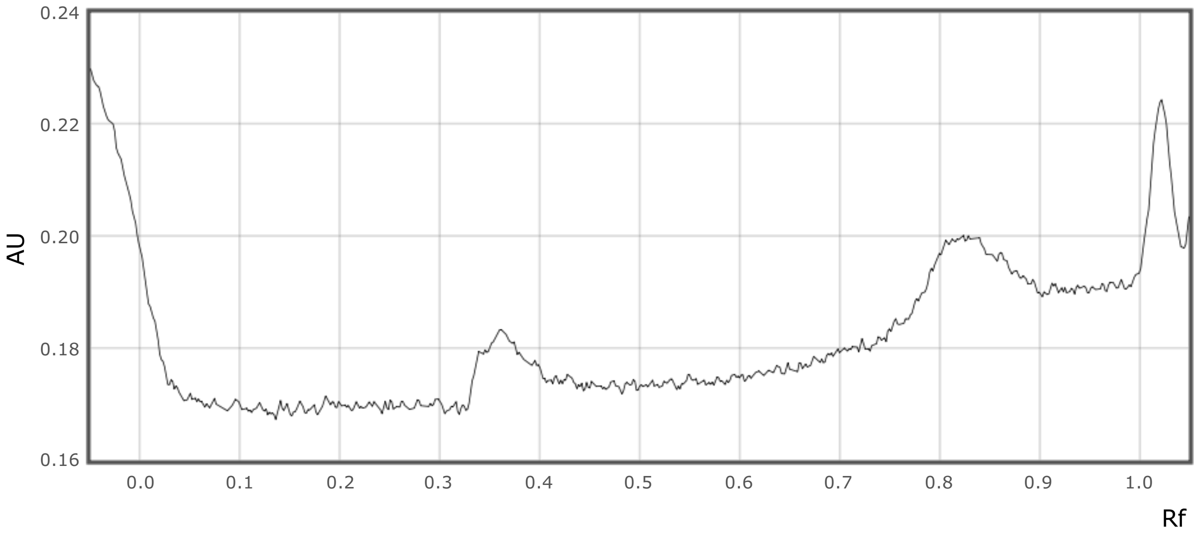

Track 3:

Type	Single λ

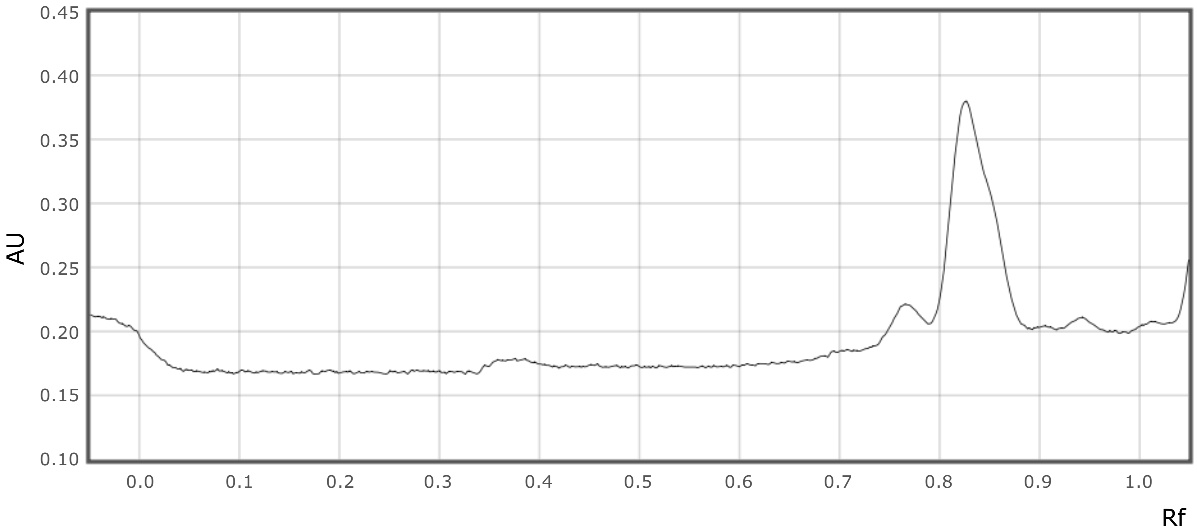

Track 4:

Type	Single λ

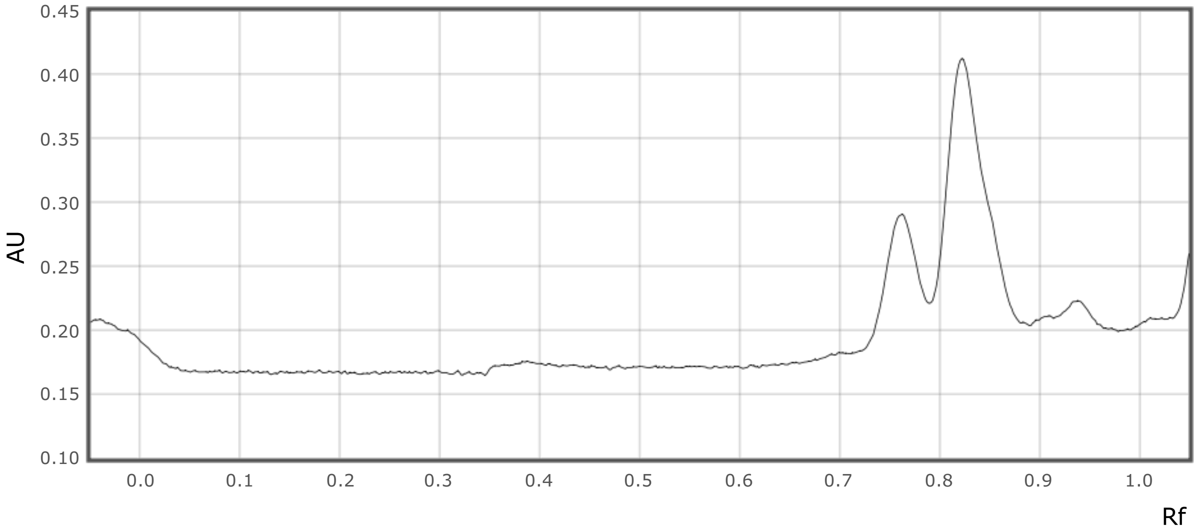

Track 5:	
Type	Single λ

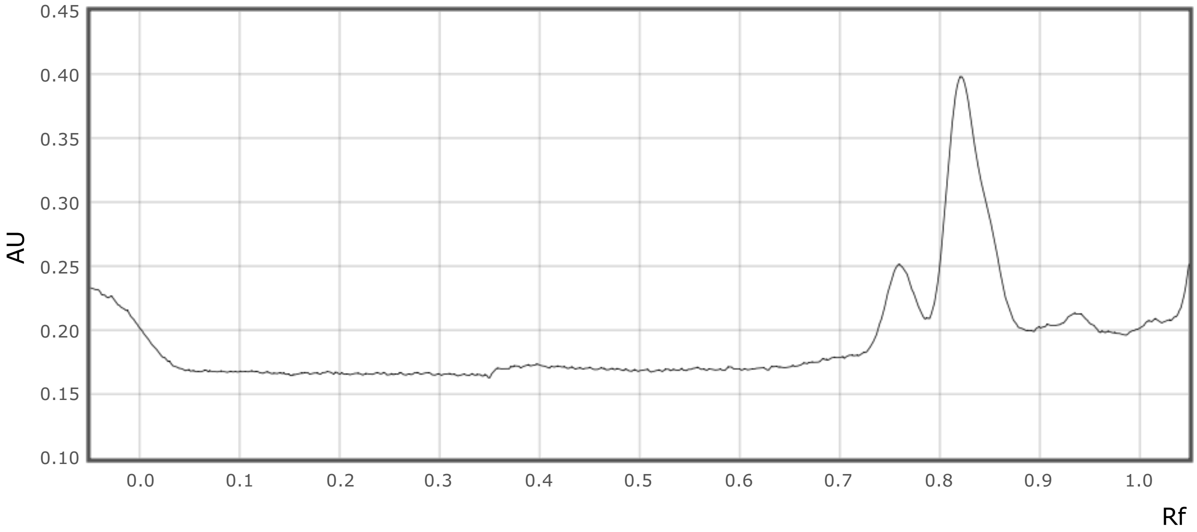

Track 6:	
Type	Single λ

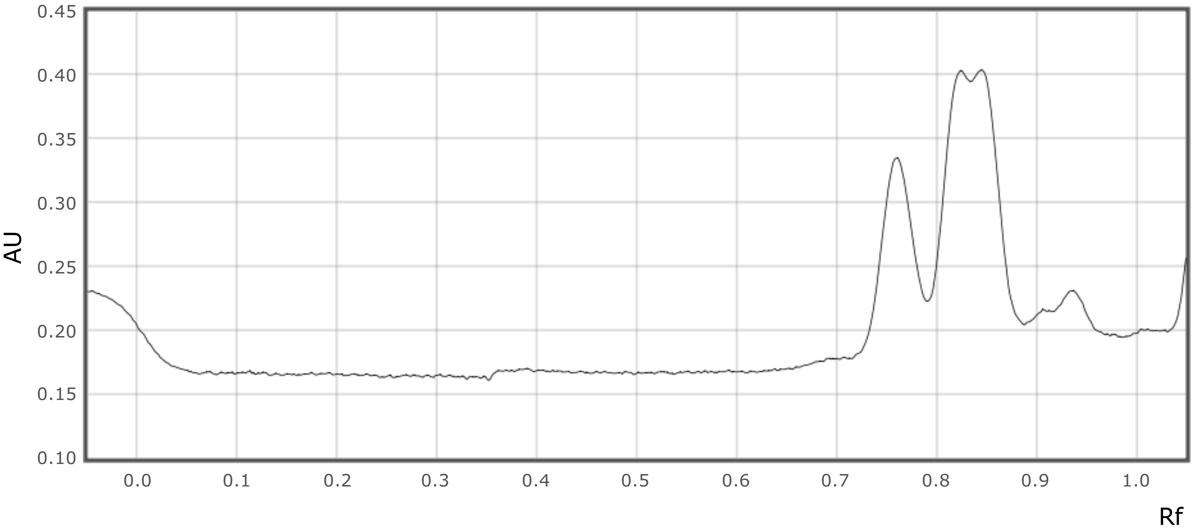

Track 7:

Type	Single λ

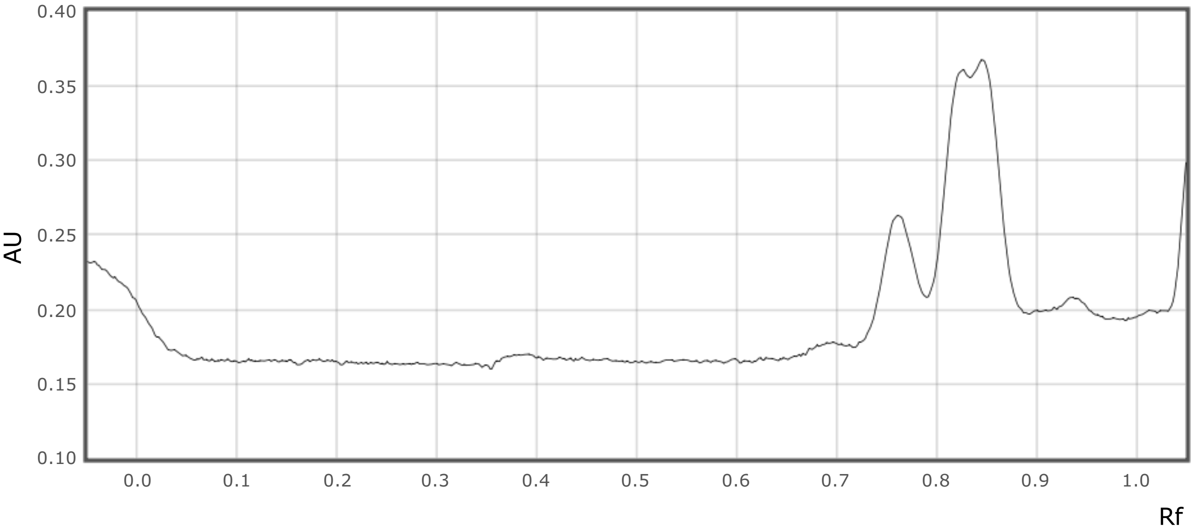

Track 8:

Type	Single λ

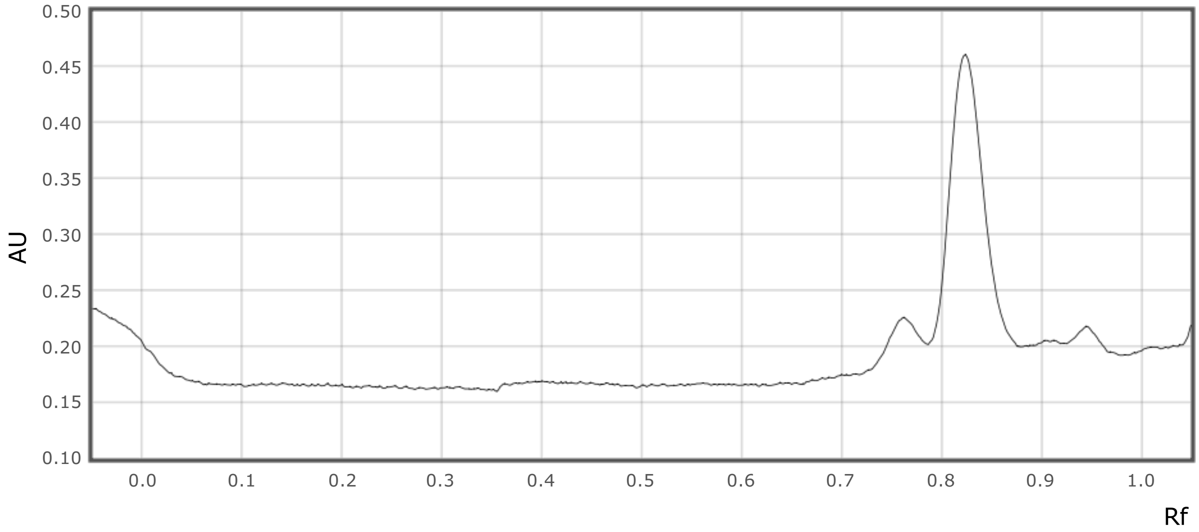

Track 9:	
Type	Single λ

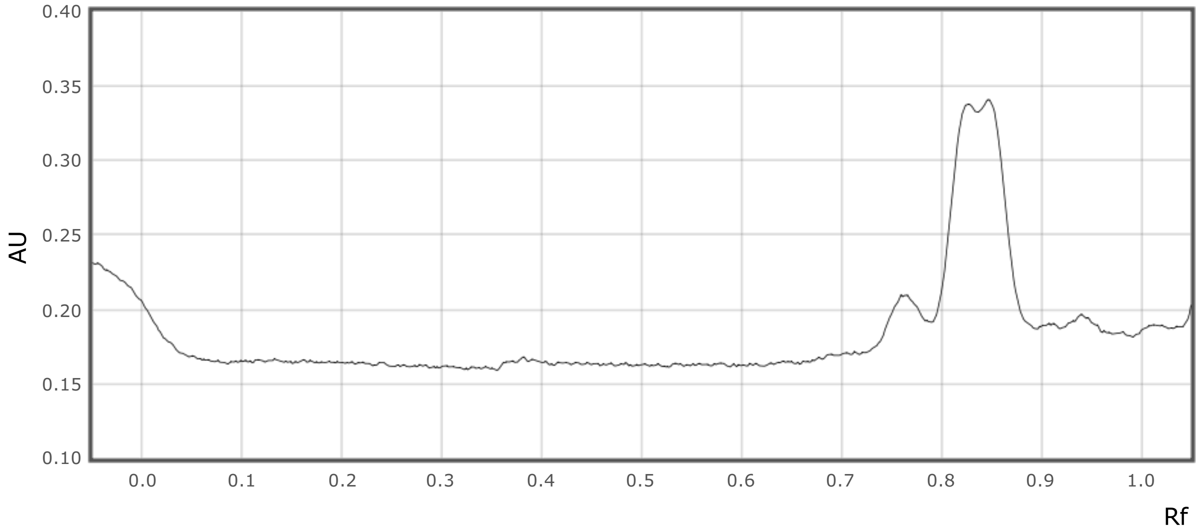

Track 10:	
Type	Single λ

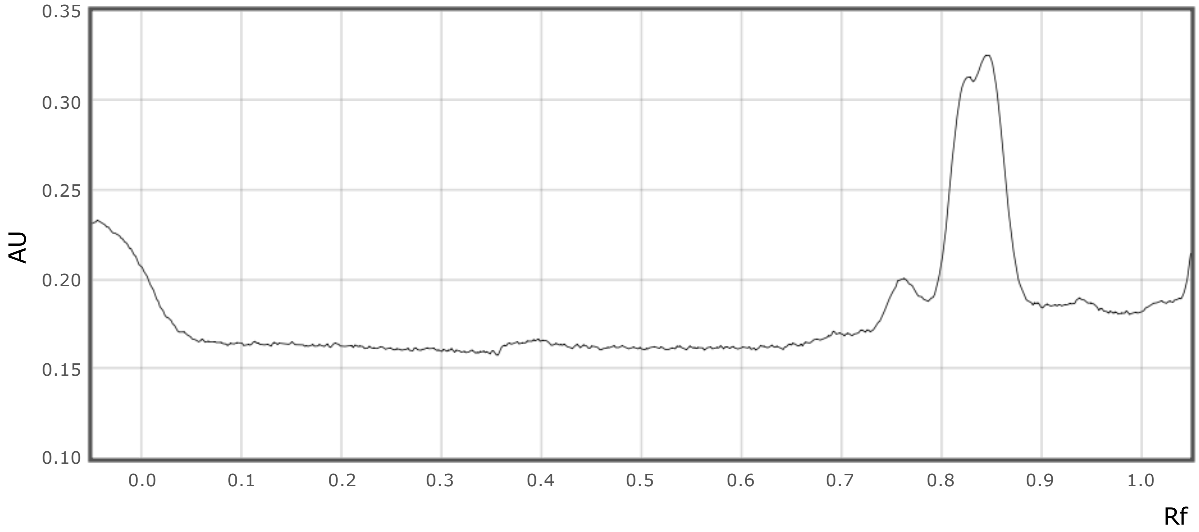

Track 11:

Type	Single λ

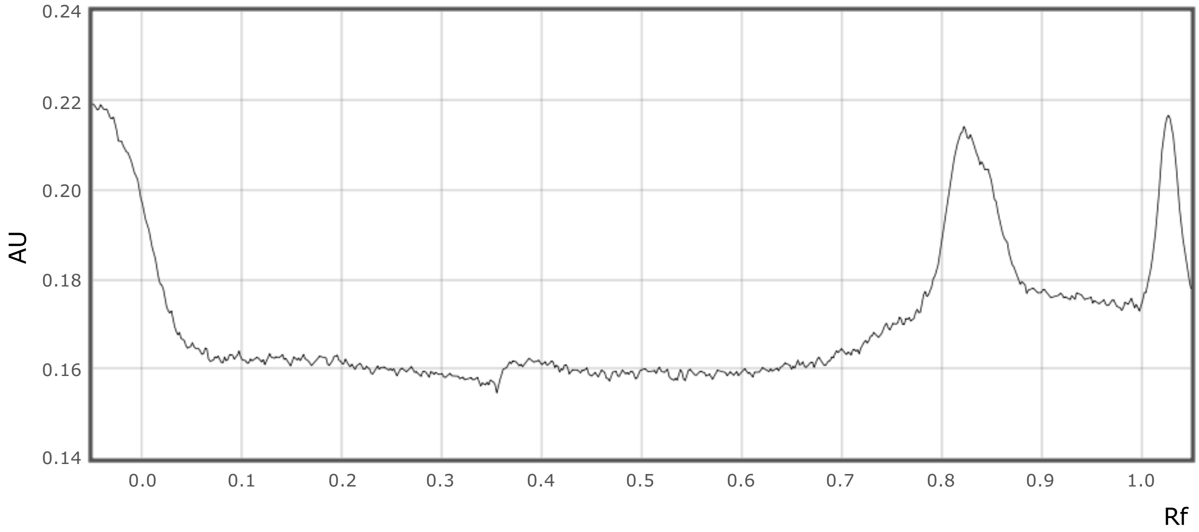

Track 12:

Type	Single λ

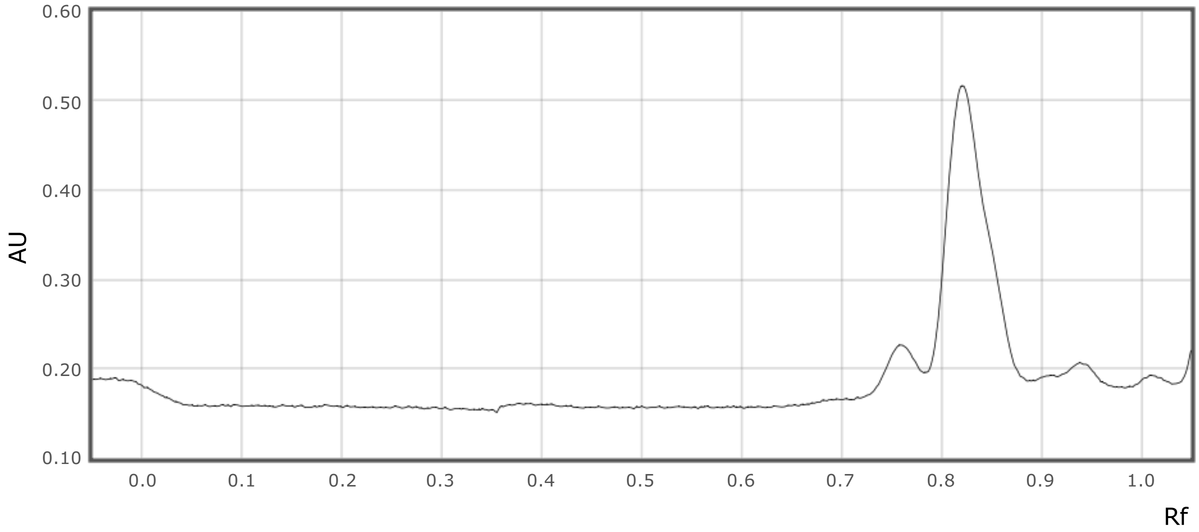

Track 13:	
Type	Single λ

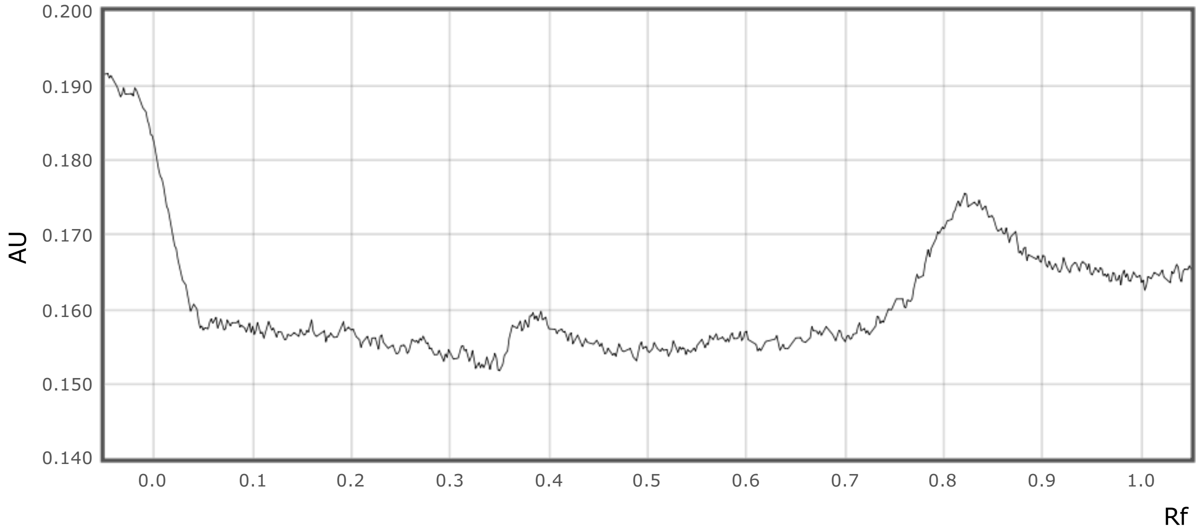

Track 14:	
Type	Single λ

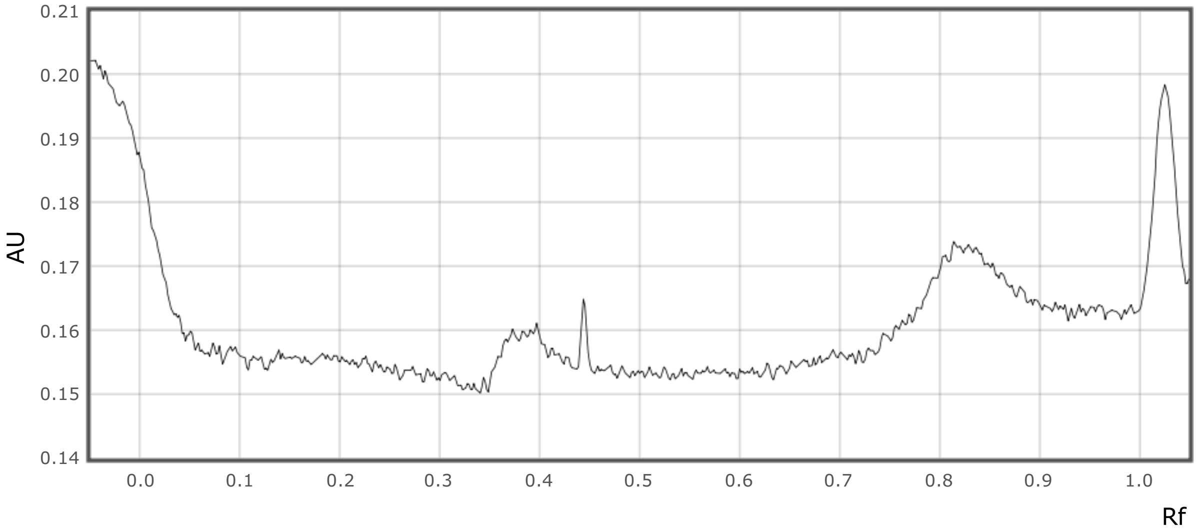

Track 15:	
Type	Single λ

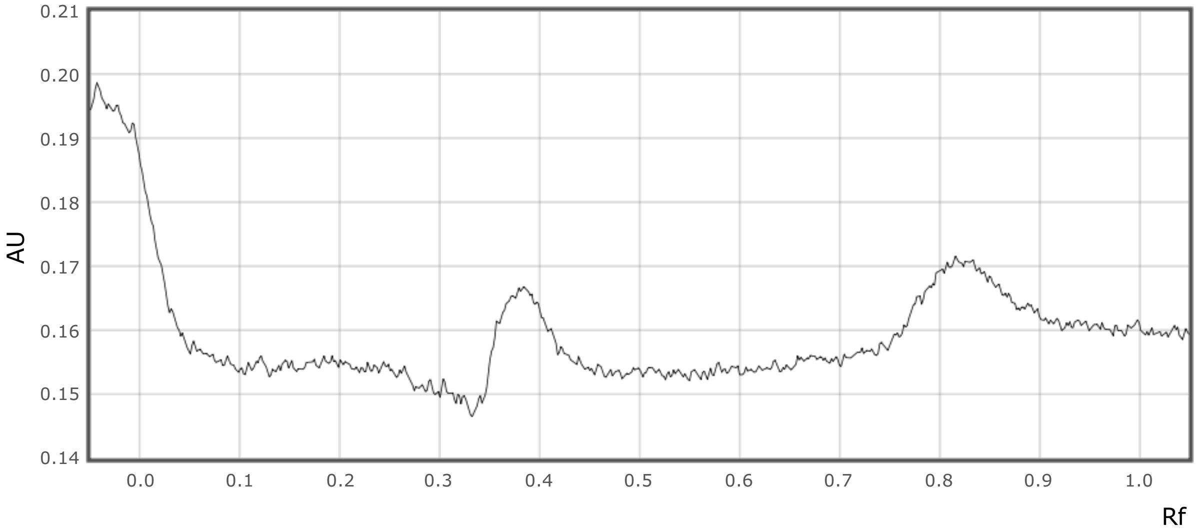

Derivatization 1 - dip:

Executed	11-Oct-2019 16:30:27 visionCATSuser

Take image derivatized plate 1a - Visualizer (S/N: 230515):

Executed	11-Oct-2019 16:31:08 visionCATSuser

RT White Derivatized, RemTransVis

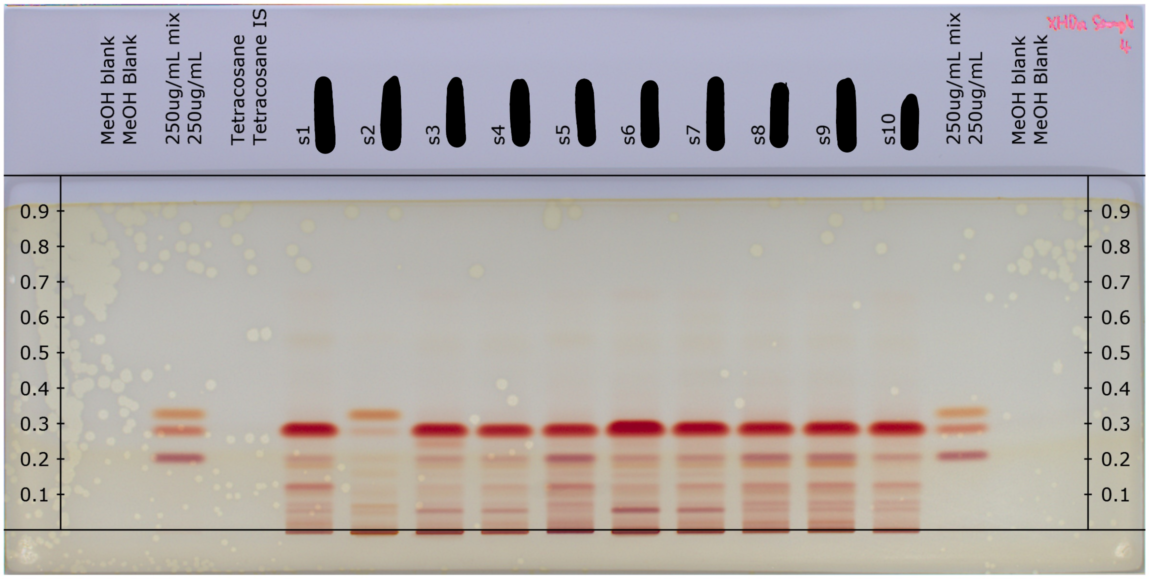

Exposure	0.052 s
Contrast	1
Normalized exposure	Disabled
Clarify	Disabled
White balance	1.16, 1.09, 0.82

R 366 Derivatized, Remission366

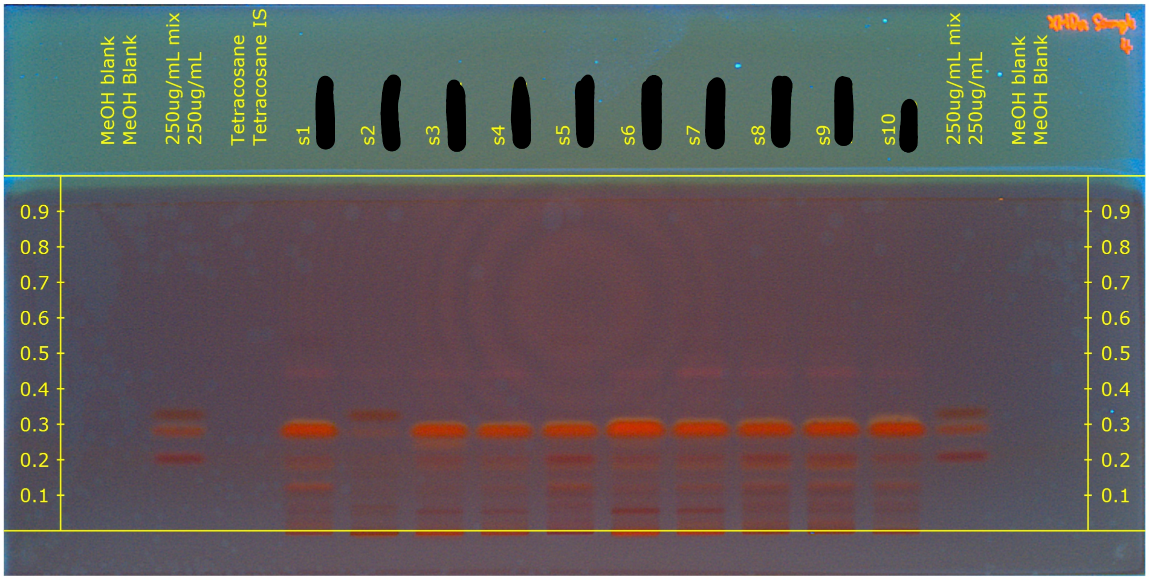

Exposure	9.999 s
Contrast	1
Normalized exposure	Disabled
Clarify	Disabled
White balance	1.00, 1.00, 1.00

Evaluation 1 :

Validated	false
Step	Take image derivatized plate 1a
Concentration unit type	Mass / volume
Notes	

Definition:

References:

250ug/mL mix

Substance Name	Concentration	Purity
9-THC	250.000 µg/ml	100.00 %
CBD	250.000 µg/ml	100.00 %
CBN	250.000 µg/ml	100.00 %

Samples:

Vial ID	Amount	Volume solution	Reference amount	Related to
MeOH blank		0.00 ml		
Tetracosane		0.00 ml		
s1		0.00 ml		
s2		0.00 ml		
s3		0.00 ml		
s4		0.00 ml		
s5		0.00 ml		
s6		0.00 ml		
s7		0.00 ml		
s8		0.00 ml		
s9		0.00 ml		
s10		0.00 ml		

Integration parameters:

Bounds	[0.000,1.000]
Smoothing	Savitzky–Golay of order 3 and window 7
Baseline correction	Lowest slope with noise 0.05
Profile subtraction	Profile subtraction from track 1
Peaks detection	Gauss (legacy) with sensitivity 0.1, separation 1 and threshold 0.1

Scan:

Wavelength	RT White

Track 1:

Type	Sample
Vial ID	MeOH blank
Description	MeOH Blank
Volume	2.0 µl

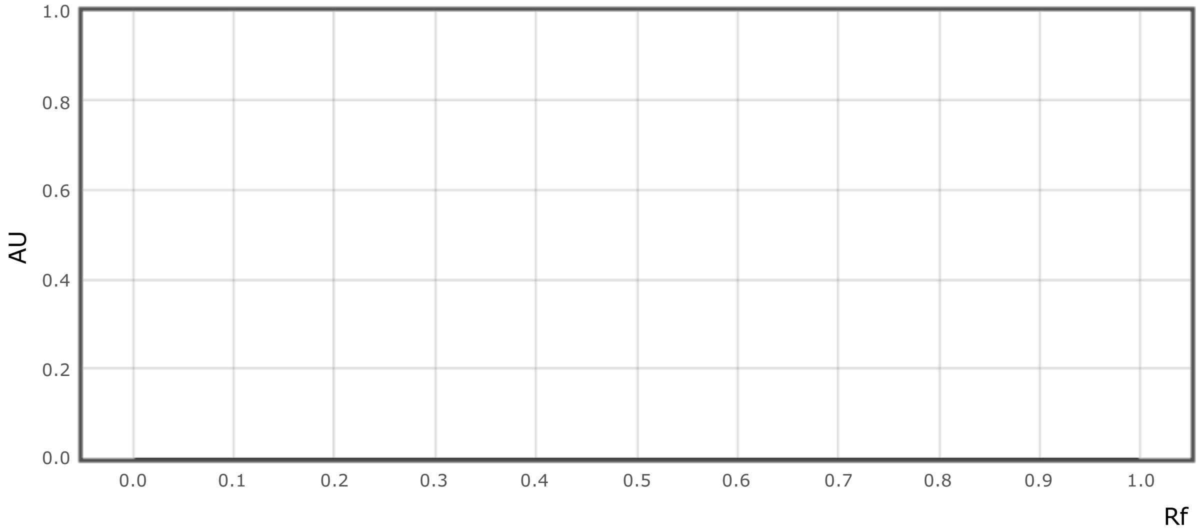

Peak #	Start		Max			End		Area		Manual peak	Substance Name
	Rf	H	Rf	H	%	Rf	H	A	%		

Track 2:

Type	Reference
Vial ID	250ug/mL mix
Description	250ug/mL
Volume	2.0 µl

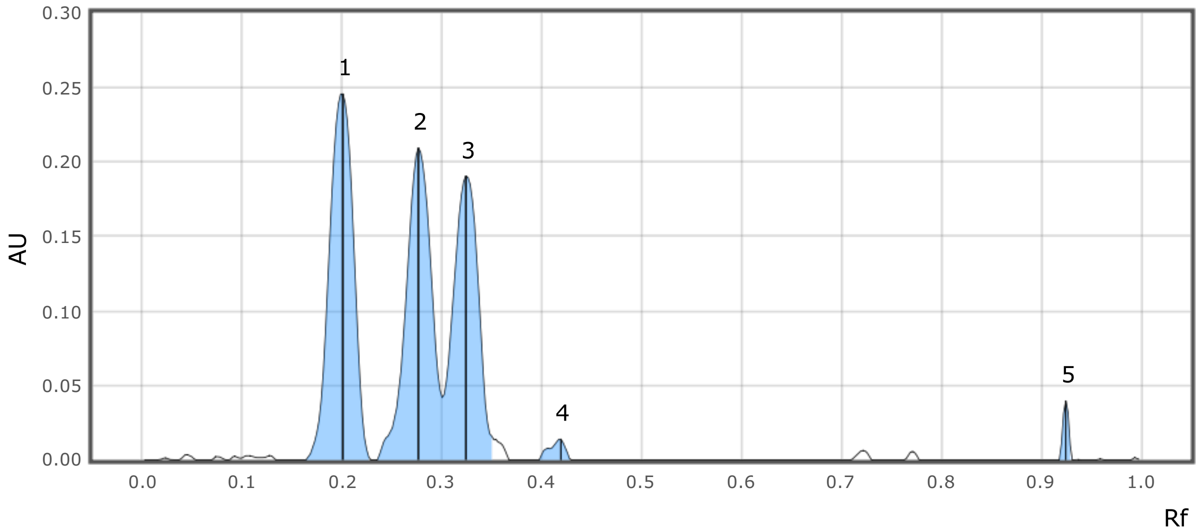

Peak #	Start		Max			End		Area		Manual peak	Substance Name
	Rf	H	Rf	H	%	Rf	H	A	%		
1	0.162	0.0000	0.201	0.2454	35.13	0.229	0.0000	0.00684	35.99	No	CBN
2	0.235	0.0000	0.276	0.2092	29.95	0.300	0.0417	0.00612	32.19	No	9-THC
3	0.300	0.0417	0.324	0.1904	27.25	0.352	0.0135	0.00553	29.11	No	CBD
4	0.397	0.0000	0.419	0.0140	2.00	0.430	0.0000	0.00025	1.30	No	
5	0.918	0.0000	0.925	0.0396	5.67	0.931	0.0000	0.00027	1.41	No	

Track 3:

Type	Sample
Vial ID	Tetracosane
Description	Tetracosane IS
Volume	2.0 µl

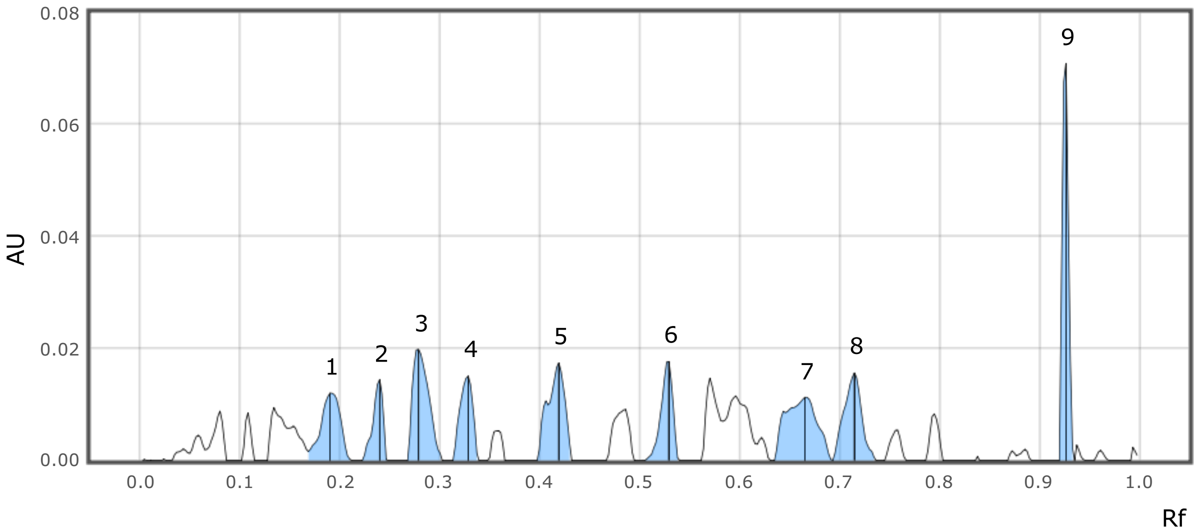

Peak	Start		Max			End		Area		Manual	Substance
#	Rf	H	Rf	H	%	Rf	H	A	%	peak	Name
1	0.168	0.0016	0.190	0.0120	6.18	0.211	0.0000	0.00027	9.48	No	
2	0.222	0.0000	0.240	0.0144	7.43	0.248	0.0000	0.00016	5.72	No	
3	0.268	0.0000	0.278	0.0198	10.21	0.302	0.0000	0.00037	12.97	No	
4	0.313	0.0000	0.328	0.0150	7.75	0.339	0.0000	0.00022	7.82	No	
5	0.397	0.0000	0.419	0.0174	8.96	0.432	0.0000	0.00034	12.04	No	
6	0.505	0.0000	0.529	0.0176	9.09	0.540	0.0000	0.00024	8.32	No	
7	0.635	0.0000	0.665	0.0112	5.79	0.693	0.0000	0.00042	14.67	No	
8	0.693	0.0000	0.715	0.0156	8.04	0.737	0.0000	0.00031	11.00	No	
9	0.920	0.0000	0.927	0.0709	36.55	0.935	0.0000	0.00051	17.99	No	

Track 4:

Type	Sample
Vial ID	s1
Description	[redacted]
Volume	2.0 µl

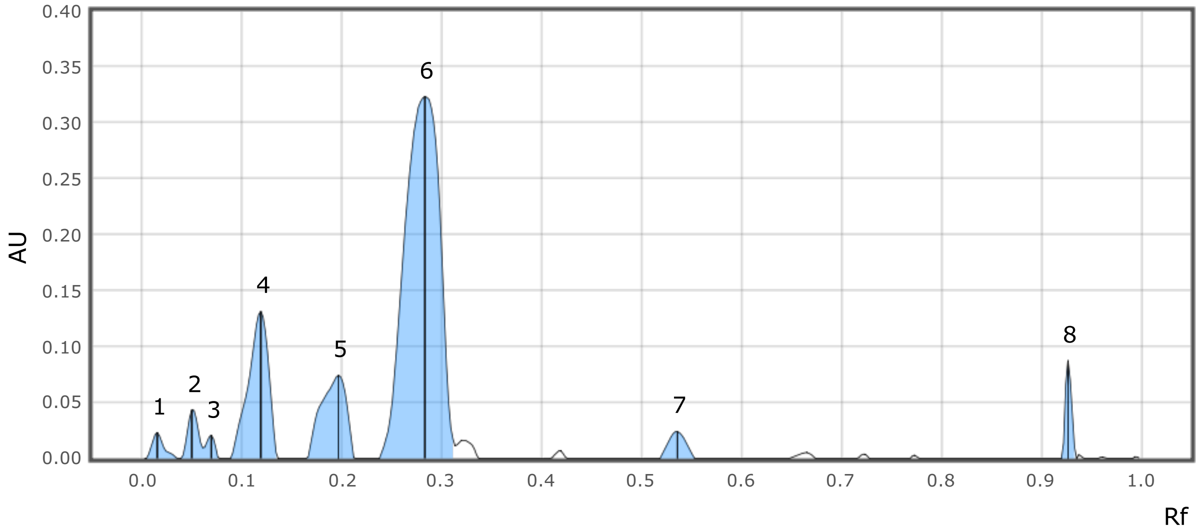

Peak #	Start Rf	Start H	Max Rf	Max H	Max %	End Rf	End H	Area A	Area %	Manual peak	Substance Name
1	0.004	0.0000	0.015	0.0231	3.18	0.036	0.0000	0.00030	1.47	No	
2	0.039	0.0000	0.049	0.0433	5.94	0.060	0.0094	0.00052	2.53	No	
3	0.060	0.0094	0.069	0.0208	2.85	0.078	0.0000	0.00021	1.02	No	
4	0.088	0.0000	0.119	0.1313	18.04	0.136	0.0000	0.00306	14.82	No	
5	0.164	0.0000	0.196	0.0743	10.21	0.214	0.0000	0.00217	10.52	No	
6	0.237	0.0000	0.283	0.3234	44.44	0.313	0.0113	0.01327	64.29	No	9-THC
7	0.518	0.0000	0.536	0.0241	3.31	0.555	0.0000	0.00048	2.34	No	
8	0.920	0.0000	0.927	0.0875	12.03	0.935	0.0000	0.00063	3.03	No	

Track 5:

Type	Sample
Vial ID	s2
Description	[redacted]
Volume	2.0 µl

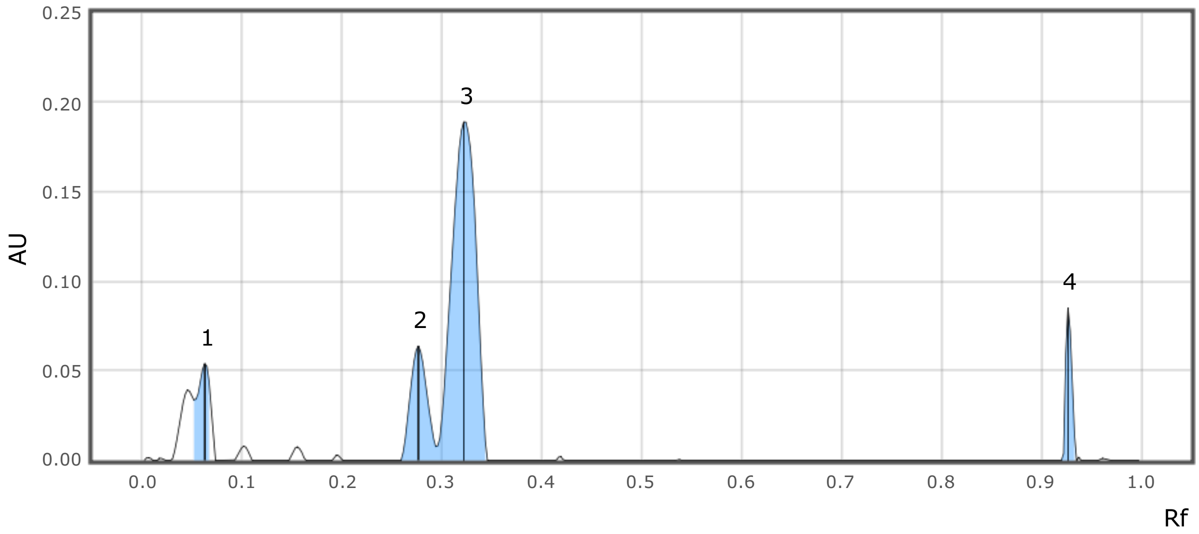

Peak #	Start Rf	Start H	Max Rf	Max H	Max %	End Rf	End H	Area A	Area %	Manual peak	Substance Name
1	0.052	0.0336	0.062	0.0540	13.79	0.073	0.0000	0.00081	10.30	No	
2	0.259	0.0000	0.276	0.0638	16.30	0.294	0.0077	0.00121	15.32	No	9-THC
3	0.294	0.0077	0.322	0.1889	48.22	0.345	0.0000	0.00527	66.70	No	CBD
4	0.920	0.0000	0.927	0.0849	21.69	0.935	0.0000	0.00061	7.68	No	

Track 6:

Type	Sample
Vial ID	s3
Description	[redacted]
Volume	2.0 µl

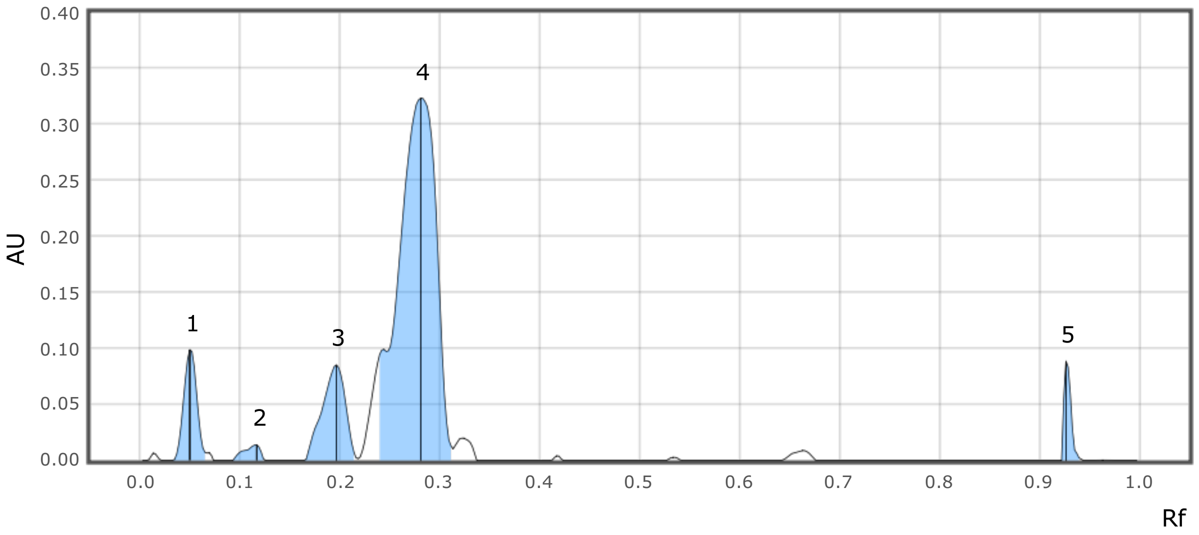

Peak #	Start Rf	Start H	Max Rf	Max H	Max %	End Rf	End H	Area A	Area %	Manual peak	Substance Name
1	0.032	0.0000	0.049	0.0989	16.21	0.067	0.0065	0.00150	7.98	No	
2	0.093	0.0000	0.116	0.0139	2.28	0.125	0.0000	0.00026	1.40	No	
3	0.164	0.0000	0.196	0.0852	13.96	0.216	0.0024	0.00228	12.14	No	
4	0.240	0.0945	0.281	0.3234	53.02	0.313	0.0103	0.01402	74.66	No	9-THC
5	0.922	0.0000	0.927	0.0886	14.52	0.944	0.0000	0.00072	3.83	No	

Track 7:

Type	Sample
Vial ID	s4
Description	[redacted]
Volume	2.0 µl

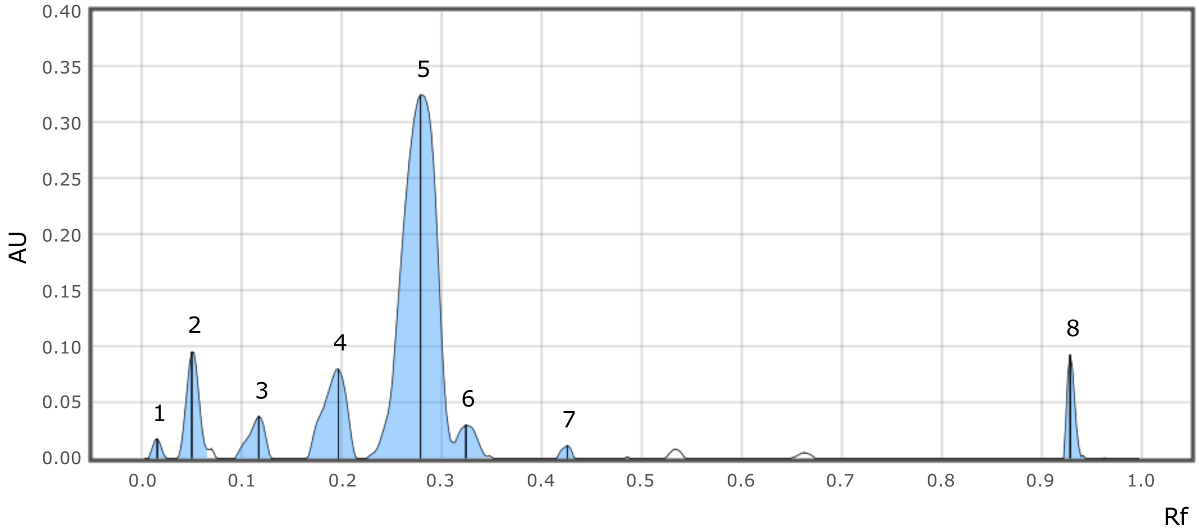

Peak #	Start		Max			End		Area		Manual peak	Substance Name
	Rf	H	Rf	H	%	Rf	H	A	%		
1	0.006	0.0000	0.015	0.0175	2.55	0.026	0.0000	0.00017	0.89	No	
2	0.034	0.0000	0.049	0.0953	13.83	0.067	0.0081	0.00141	7.46	No	
3	0.093	0.0000	0.116	0.0376	5.46	0.129	0.0000	0.00071	3.74	No	
4	0.164	0.0000	0.196	0.0796	11.55	0.216	0.0000	0.00216	11.38	No	
5	0.224	0.0000	0.278	0.3250	47.16	0.311	0.0141	0.01289	68.03	No	9-THC
6	0.311	0.0141	0.324	0.0299	4.33	0.345	0.0016	0.00067	3.51	No	CBD
7	0.415	0.0000	0.425	0.0115	1.67	0.434	0.0000	0.00013	0.67	No	
8	0.922	0.0000	0.929	0.0926	13.43	0.946	0.0000	0.00082	4.32	No	

Track 8:

Type	Sample
Vial ID	s5
Description	[redacted]
Volume	2.0 µl

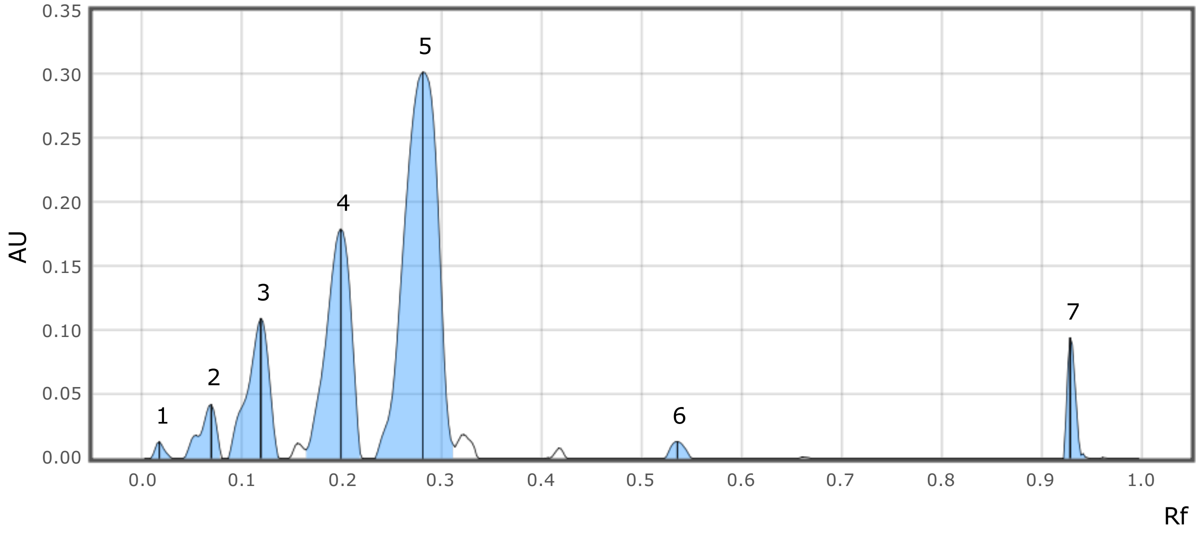

Peak	Start		Max			End		Area		Manual	Substance
#	Rf	H	Rf	H	%	Rf	H	A	%	peak	Name
1	0.008	0.0000	0.017	0.0129	1.72	0.030	0.0000	0.00013	0.61	No	
2	0.041	0.0000	0.069	0.0420	5.58	0.080	0.0000	0.00079	3.67	No	
3	0.086	0.0000	0.119	0.1094	14.54	0.138	0.0000	0.00270	12.48	No	
4	0.164	0.0065	0.199	0.1793	23.82	0.220	0.0000	0.00510	23.63	No	
5	0.233	0.0000	0.281	0.3017	40.08	0.313	0.0089	0.01179	54.56	No	9-THC
6	0.523	0.0000	0.536	0.0131	1.74	0.551	0.0000	0.00021	0.98	No	
7	0.922	0.0000	0.929	0.0943	12.53	0.948	0.0000	0.00088	4.06	No	

Track 9:

Type	Sample
Vial ID	s6
Description	[redacted]
Volume	2.0 µl

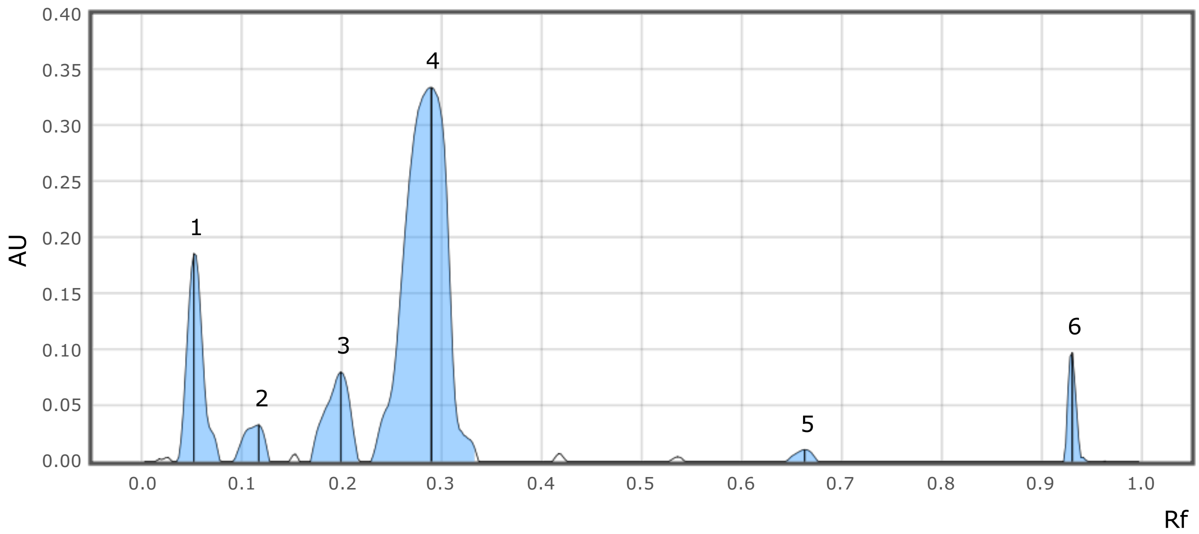

Peak	Start		Max			End		Area		Manual	Substance
#	Rf	H	Rf	H	%	Rf	H	A	%	peak	Name
1	0.034	0.0000	0.052	0.1858	25.10	0.080	0.0000	0.00338	14.01	No	
2	0.090	0.0000	0.116	0.0327	4.42	0.129	0.0000	0.00078	3.23	No	
3	0.168	0.0000	0.199	0.0799	10.80	0.218	0.0000	0.00223	9.23	No	
4	0.229	0.0000	0.289	0.3343	45.15	0.337	0.0000	0.01665	68.93	No	9-THC
5	0.644	0.0000	0.663	0.0105	1.42	0.678	0.0000	0.00021	0.86	No	
6	0.922	0.0000	0.931	0.0971	13.12	0.948	0.0000	0.00090	3.74	No	

Track 10:

Type	Sample
Vial ID	s7
Description	[redacted]
Volume	2.0 µl

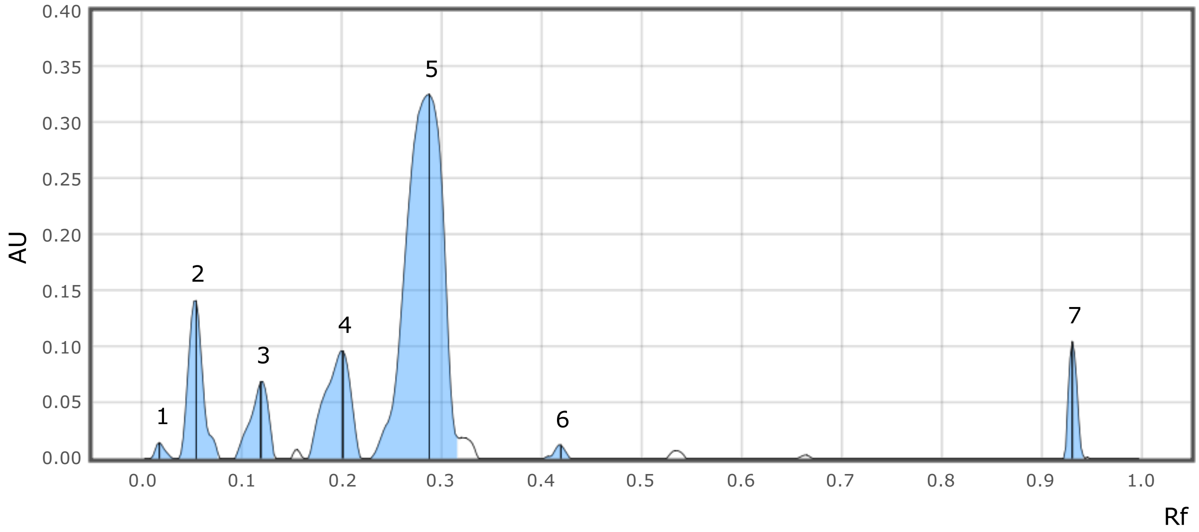

Peak #	Start Rf	Start H	Max Rf	Max H	Max %	End Rf	End H	Area A	Area %	Manual peak	Substance Name
1	0.008	0.0000	0.017	0.0142	1.86	0.032	0.0000	0.00015	0.69	No	
2	0.036	0.0000	0.054	0.1411	18.51	0.078	0.0000	0.00236	10.76	No	
3	0.093	0.0000	0.119	0.0686	8.99	0.134	0.0000	0.00141	6.43	No	
4	0.164	0.0000	0.201	0.0962	12.62	0.220	0.0000	0.00283	12.90	No	
5	0.227	0.0000	0.287	0.3255	42.70	0.317	0.0178	0.01401	63.92	No	9-THC
6	0.402	0.0000	0.419	0.0121	1.58	0.430	0.0000	0.00015	0.67	No	
7	0.922	0.0000	0.931	0.1046	13.73	0.944	0.0000	0.00101	4.62	No	

Track 11:

Type	Sample
Vial ID	s8
Description	[redacted]
Volume	2.0 µl

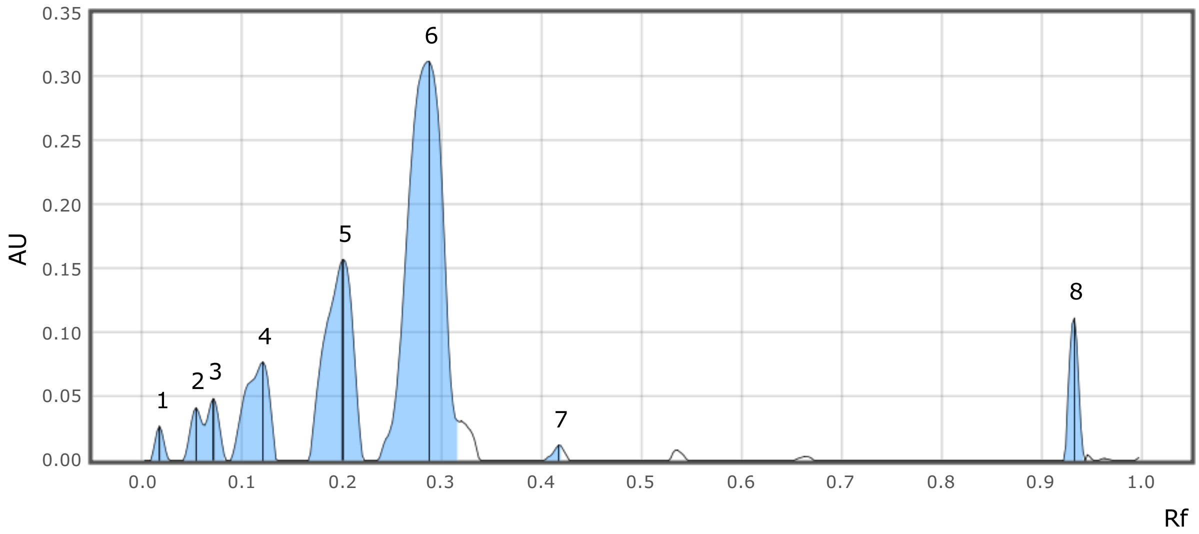

Peak #	Start Rf	Start H	Max Rf	Max H	Max %	End Rf	End H	Area A	Area %	Manual peak	Substance Name
1	0.006	0.0000	0.017	0.0267	3.40	0.028	0.0000	0.00026	1.13	No	
2	0.041	0.0000	0.054	0.0413	5.25	0.062	0.0273	0.00056	2.48	No	
3	0.062	0.0273	0.071	0.0484	6.16	0.084	0.0000	0.00065	2.87	No	
4	0.088	0.0000	0.121	0.0770	9.80	0.136	0.0000	0.00214	9.42	No	
5	0.166	0.0000	0.201	0.1571	19.99	0.222	0.0000	0.00502	22.07	No	
6	0.235	0.0000	0.287	0.3123	39.72	0.317	0.0302	0.01275	56.11	No	9-THC
7	0.402	0.0000	0.417	0.0118	1.51	0.428	0.0000	0.00015	0.67	No	
8	0.922	0.0000	0.933	0.1114	14.17	0.944	0.0000	0.00119	5.25	No	

Track 12:

Type	Sample
Vial ID	s9
Description	[redacted]
Volume	2.0 µl

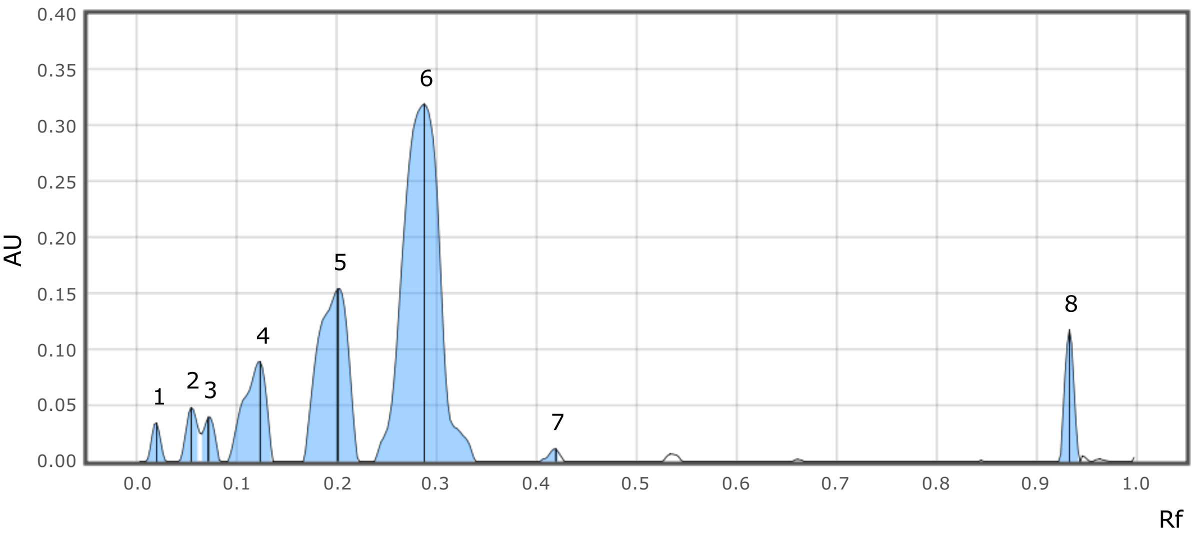

Peak #	Start Rf	Start H	Max Rf	Max H	Max %	End Rf	End H	Area A	Area %	Manual peak	Substance Name
1	0.008	0.0000	0.019	0.0344	4.23	0.030	0.0000	0.00034	1.41	No	
2	0.041	0.0000	0.054	0.0483	5.93	0.062	0.0254	0.00061	2.53	No	
3	0.065	0.0245	0.071	0.0396	4.86	0.084	0.0000	0.00049	2.04	No	
4	0.090	0.0000	0.123	0.0891	10.94	0.136	0.0000	0.00229	9.52	No	
5	0.166	0.0000	0.201	0.1543	18.95	0.222	0.0000	0.00534	22.19	No	
6	0.237	0.0000	0.287	0.3196	39.26	0.339	0.0000	0.01362	56.60	No	9-THC
7	0.402	0.0000	0.419	0.0112	1.37	0.428	0.0000	0.00014	0.57	No	
8	0.922	0.0000	0.933	0.1177	14.45	0.944	0.0000	0.00124	5.14	No	

Track 13:

Type	Sample
Vial ID	s10
Description	[redacted]
Volume	2.0 µl

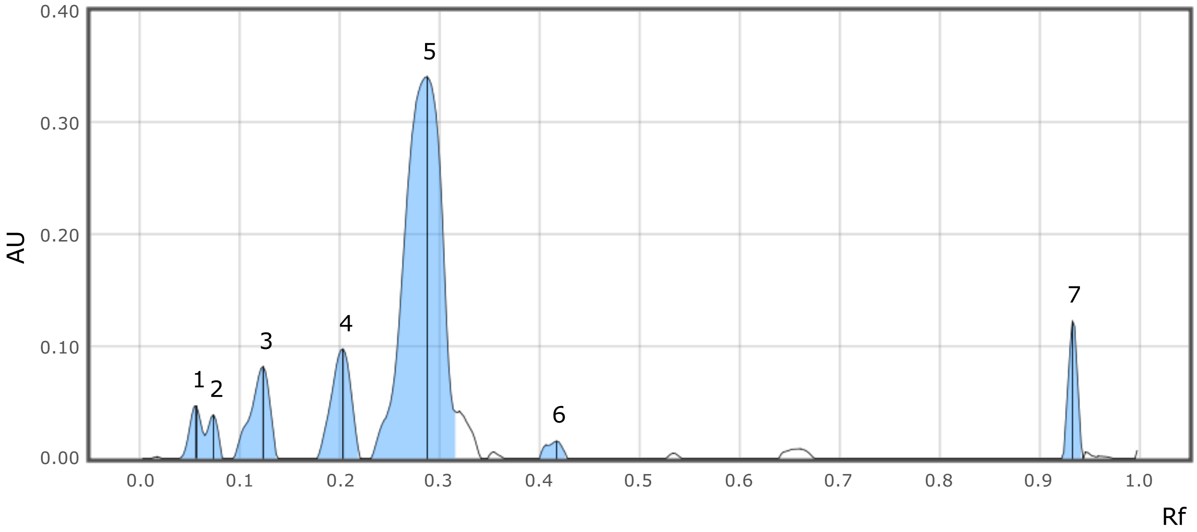

Peak #	Start		Max			End		Area		Manual peak	Substance Name
	Rf	H	Rf	H	%	Rf	H	A	%		
1	0.039	0.0000	0.056	0.0470	6.30	0.065	0.0204	0.00064	2.98	No	
2	0.065	0.0204	0.073	0.0391	5.25	0.084	0.0000	0.00046	2.15	No	
3	0.093	0.0000	0.123	0.0820	11.00	0.138	0.0000	0.00180	8.38	No	
4	0.177	0.0000	0.203	0.0978	13.12	0.220	0.0000	0.00223	10.35	No	CBN
5	0.231	0.0000	0.287	0.3413	45.77	0.317	0.0411	0.01481	68.78	No	9-THC
6	0.400	0.0000	0.417	0.0154	2.07	0.428	0.0000	0.00029	1.35	No	
7	0.922	0.0000	0.933	0.1229	16.49	0.944	0.0000	0.00129	6.01	No	

Track 14:

Type	Reference
Vial ID	250ug/mL mix
Description	250ug/mL
Volume	2.0 µl

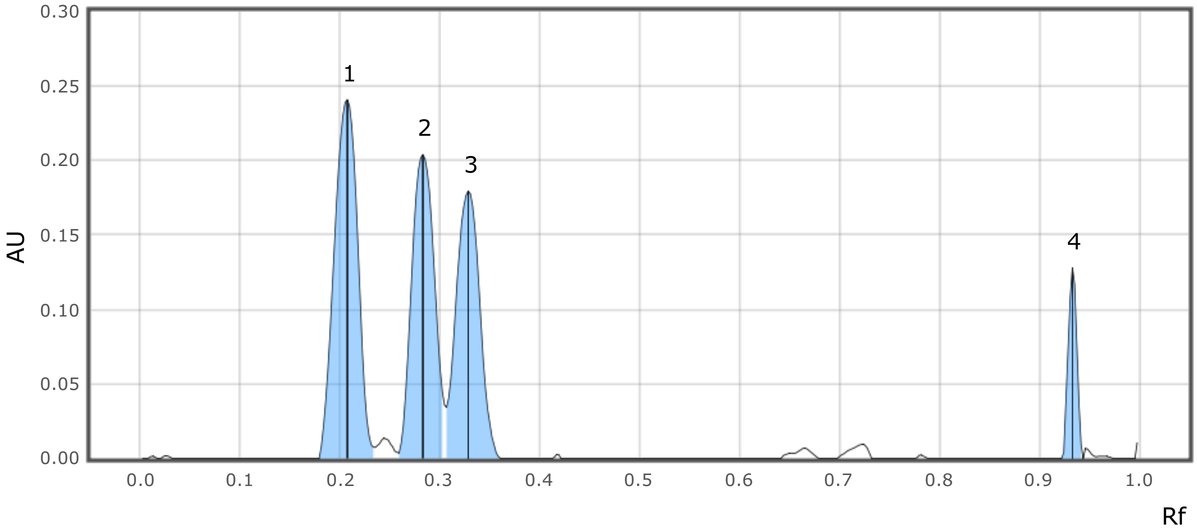

Peak #	Start Rf	Start H	Max Rf	Max H	Max %	End Rf	End H	Area A	Area %	Manual peak	Substance Name
1	0.179	0.0000	0.207	0.2405	32.02	0.235	0.0074	0.00650	36.07	No	CBN
2	0.259	0.0035	0.283	0.2037	27.11	0.304	0.0359	0.00535	29.66	No	9-THC
3	0.307	0.0342	0.328	0.1793	23.87	0.361	0.0000	0.00489	27.15	No	CBD
4	0.922	0.0000	0.933	0.1277	17.00	0.944	0.0000	0.00128	7.11	No	

Track 15:

Type	Sample
Vial ID	MeOH blank
Description	MeOH Blank
Volume	2.0 µl

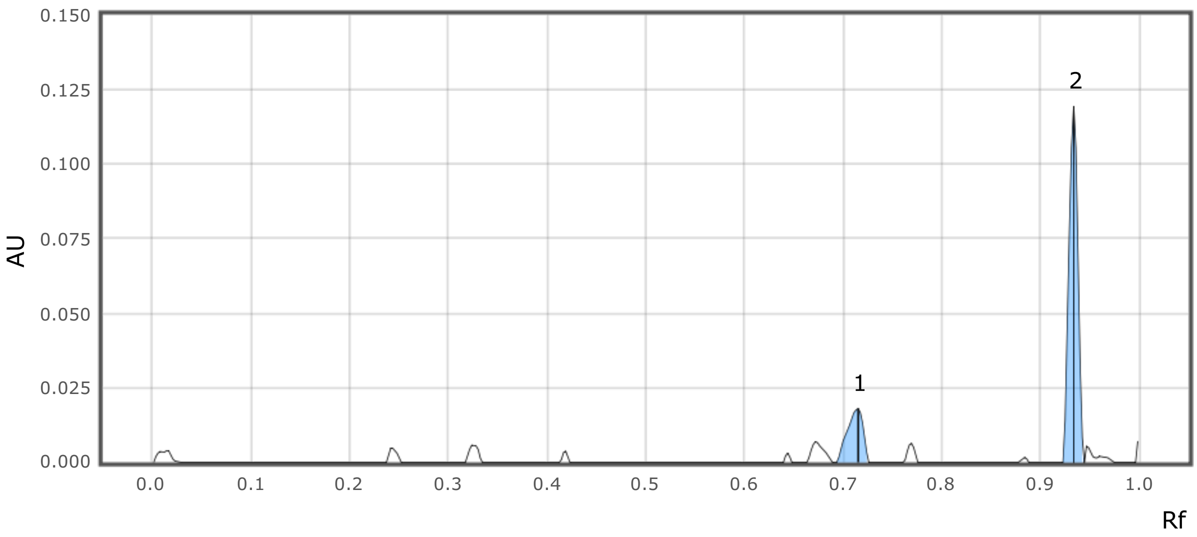

Peak #	Start Rf	Start H	Max Rf	Max H	Max %	End Rf	End H	Area A	Area %	Manual peak	Substance Name
1	0.693	0.0000	0.715	0.0180	13.10	0.726	0.0000	0.00033	20.99	No	
2	0.922	0.0000	0.933	0.1191	86.90	0.944	0.0000	0.00125	79.01	No	

Calibration results:

Height calibration for substance 9-THC @ RT White:

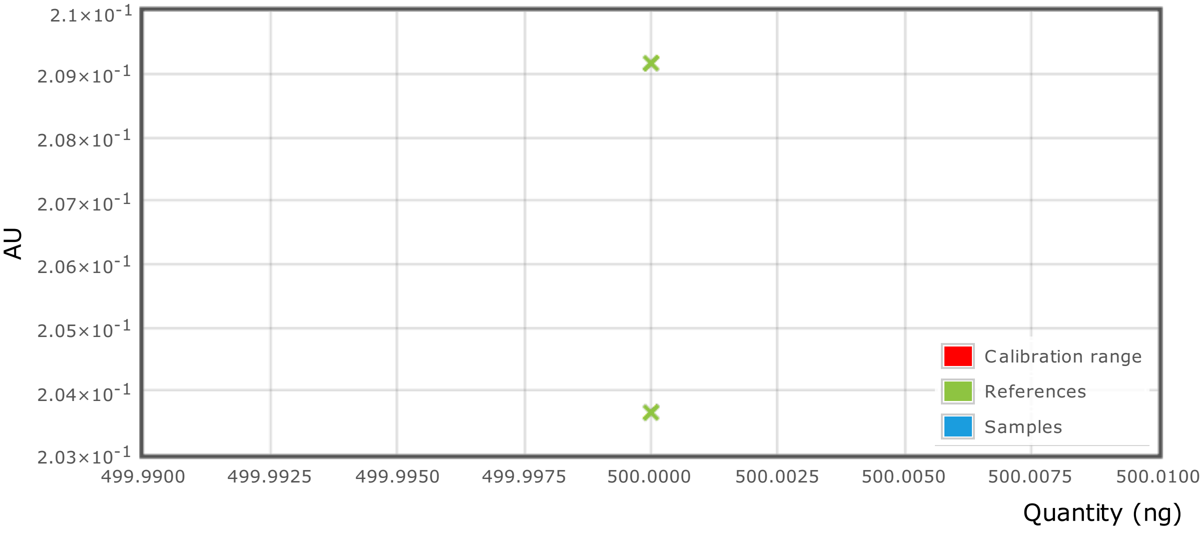

Regression mode	Linear-2
Range deviation	5.00 %
Related substances	Default
Number of references	2
Calibration function	$y=0x$
Coefficient of variation	CV 0.00 %
Correlation coefficient	n/a
⚠	Unable to compute the results for this substance because there wasn't eno ugh groups of references replicas (at least 1 for Linear-1, 2 for Linear2 and Mime-1 and 3 for Polynomial and MiMe-2)

Height calibration for substance CBD @ RT White:

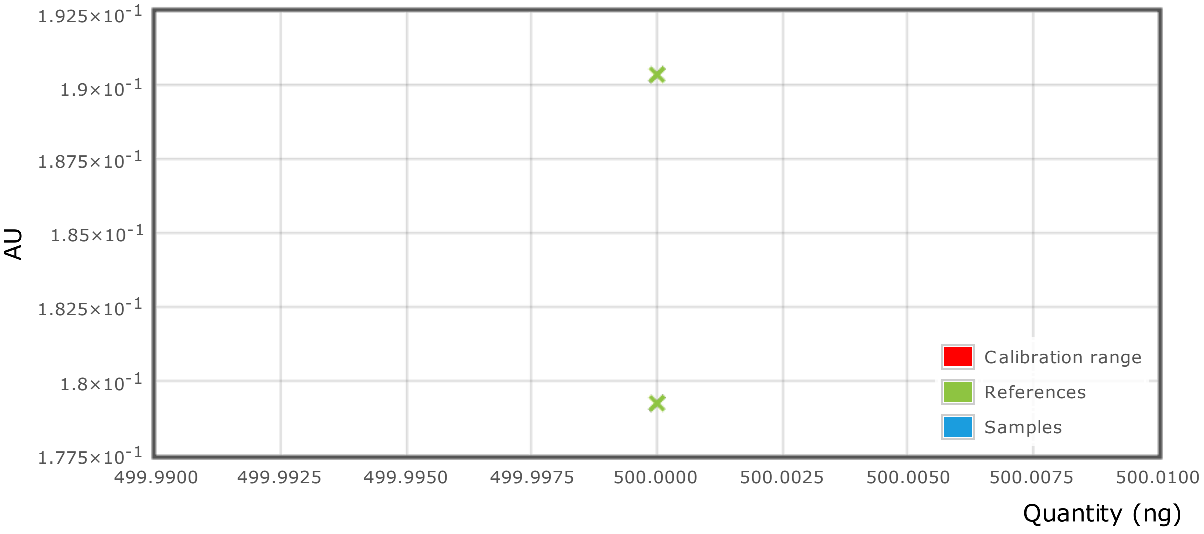

Regression mode	Linear-2
Range deviation	5.00 %
Related substances	Default
Number of references	2
Calibration function	$y=0x$
Coefficient of variation	CV 0.00 %
Correlation coefficient	n/a
	Unable to compute the results for this substance because there wasn't enough groups of references replicas (at least 1 for Linear-1, 2 for Linear2 and Mime-1 and 3 for Polynomial and MiMe-2)

Height calibration for substance CBN @ RT White:

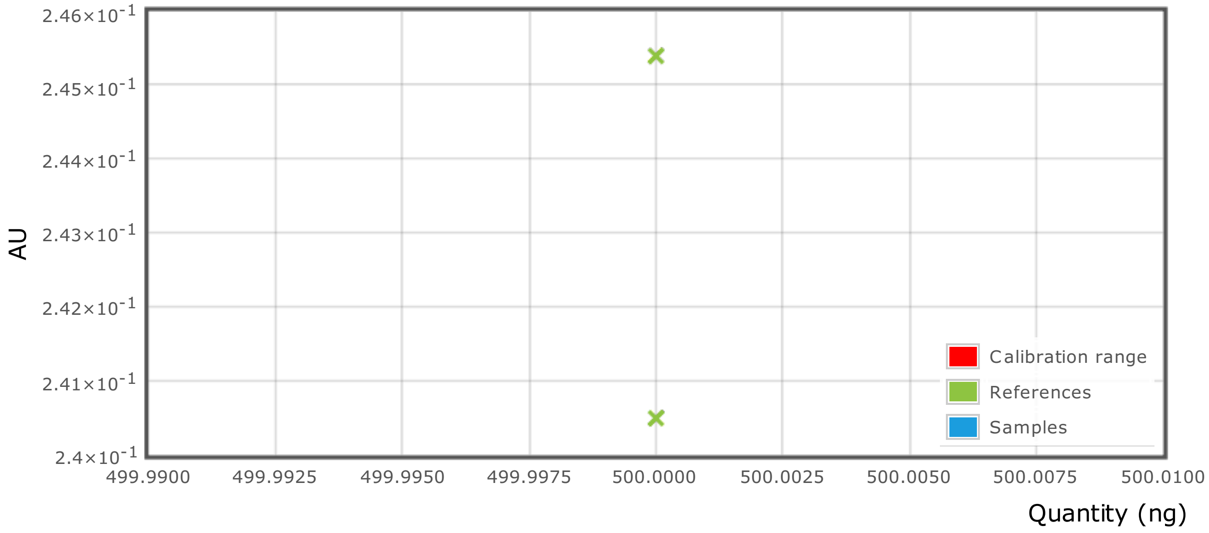

Regression mode	Linear-2
Range deviation	5.00 %
Related substances	Default
Number of references	2
Calibration function	$y=0x$
Coefficient of variation	CV 0.00 %
Correlation coefficient	n/a
	Unable to compute the results for this substance because there wasn't enough groups of references replicas (at least 1 for Linear-1, 2 for Linear2 and Mime-1 and 3 for Polynomial and MiMe-2)

Results:

Substance having no available results		
	CBD	Unable to compute the results for this substance because there wasn't enough groups of references replicas (at least 1 for Linear-1, 2 for Linear2 and Mime-1 and 3 for Polynomial and MiMe-2)
	9-THC	Unable to compute the results for this substance because there wasn't enough groups of references replicas (at least 1 for Linear-1, 2 for Linear2 and Mime-1 and 3 for Polynomial and MiMe-2)
	CBN	Unable to compute the results for this substance because there wasn't enough groups of references replicas (at least 1 for Linear-1, 2 for Linear2 and Mime-1 and 3 for Polynomial and MiMe-2)

A track marked with ⚠ means: this result is outside the regression range given by the reference assignments, but is included in the results because it is in the allowed range deviation.

Analyst:	Reviewer:
