# Supplementary material for: High Performance Thin-Layer Chromatography (HPTLC) data of Cannabinoids in ten mobile phase systems
Source: Data Brief. 2020 Jun 30;31:105955. doi: 10.1016/j.dib.2020.105955 (PMC7352075; doi:10.1016/j.dib.2020.105955)
Supplement: Supplementary file 1 [file mmc1.zip › S4-Case sample reports/XHDa-sample run-5.pdf]

Analysis: XHDa-sample run-5

Path: Home/YL Research

Based on method: Samples (no cal)

Created	11-Oct-2019 17:00:32	visionCATSuser
Modified	11-Oct-2019 19:14:45	visionCATSuser
Last HPTLC log	11-Oct-2019 19:14:45	Analysis modified
Explorer notes		

Track	Vial ID	Description	Volume	Position	Type
1	MeOH blank	MeOH Blank	2.0 µl	A1	Sample
2	250ug/mL mix	250ug/mL	2.0 µl	A2	Reference
3	Tetracosane	Tetracosane IS	2.0 µl	A3	Sample
4	s1	[redacted]	2.0 µl	B1	Sample
5	s2	[redacted]	2.0 µl	B2	Sample
6	s3	[redacted]	2.0 µl	B3	Sample
7	s4	[redacted]	2.0 µl	B4	Sample
8	s5	[redacted]	2.0 µl	B5	Sample
9	s6	[redacted]	2.0 µl	B6	Sample
10	s7	[redacted]	2.0 µl	B7	Sample
11	s8	[redacted]	2.0 µl	B8	Sample
12	s9	[redacted]	2.0 µl	B9	Sample
13	s10	[redacted]	2.0 µl	B10	Sample
14	250ug/mL mix	250ug/mL	2.0 µl	A2	Reference
15	MeOH blank	MeOH Blank	2.0 µl	A1	Sample
Sequence table notes	* Diluted with 500uL MeOH				

A track marked with ⚠ means: the application type is overridden in some evaluation(s).

System setup:

Software	Server User-PC, version 2.5.18072.1
ATS4	S/N:080713
Chamber	N/A
Derivatization dip	N/A
Scanner3	S/N:031025
Visualizer	S/N:230515

Chromatography

Plate layout:

Stationary phase	Merck, HPTLC plates silica gel 60 F 254
Plate format	200.0 x 100.0 mm
Application type	Band
Application	Position Y: 8.0 mm, length: 8.0 mm, width: 0.0 mm
Track	First position X: 20.0 mm, distance: 11.4 mm
Solvent front position	70.0 mm
Notes	

Take image clean plate 1a - Visualizer (S/N: 230515):

Quality	Enhanced
RT White	auto capture, Auto, level 85 %, Band
R 254	auto capture, Auto, level 85 %, Band
Instrument diagnostics	Valid diagnostics
Documentation step label	
Notes	

Application 1 - ATS 4 (S/N: 080713):

Spray gas	NI
Sample solvent type	Methanol
Filling speed	15 µl/s
Predosage volume	200 nl
Retraction volume	200 nl
Dosage speed	150 nl/s
Filling quality	User
Rinsing cycles / vacuum	2 / 4 s
Filling cycles / vacuum	1 / 4 s
Rinsing solvent name	Methanol
Nozzle temperature	Unheated
Rack in use	Standard
Instrument diagnostics	Valid diagnostics
Notes	

Development 1 - Chamber:

Tank	TTC 20x10
Mobile phase	Xylene:hexane:diethylamine (25:10:1)
Saturation time	20 min
Use saturation pad	true
Use smartALERT	false
Volume front through	10 ml
Volume rear through	25 ml
Drying time	5 min
Drying temperature	Room temperature
Notes	

Take image developed plate 1a - Visualizer (S/N: 230515):

Quality	Enhanced
RT White	auto capture, Auto, level 85 %, Band
R 254	auto capture, Auto, level 85 %, Band
R 366	auto capture, Auto, level 85 %, Band
Instrument diagnostics	Valid diagnostics
Documentation step label	
Notes	

Scan developed plate 1b - Scanner 3 (S/N: 031025):

Scanner type	Single λ
Optimization for	Resolution
Measurement mode	Absorption
Filter	n/a
Detector mode	Automatic
Scanning speed	20 mm/s
Data resolution	100 µm/step
Slit	5 x 0.2 mm, micro
Partial scan	No
Lamp	Deuterium & Tungsten
Wavelength(s)	254 nm
Instrument diagnostics	Valid diagnostics
Documentation step label	
Notes	

Derivatization 1 - dip:

Reagent name	
Dipping speed	5
Dipping time	0 s
Reagent preparation	
Heating	100 °C for 3 min, heated after
Notes	

Take image derivatized plate 1a - Visualizer (S/N: 230515):

Quality	Enhanced
RT White	auto capture, Auto, level 85 %, Band
R 366	auto capture, Auto, level 85 %, Band
Instrument diagnostics	Valid diagnostics
Documentation step label	
Notes	

System suitability tests:

SST settings:

SST tracks	

Data acquisition

Application 1 - ATS 4 (S/N: 080713):

Executed	11-Oct-2019 17:28:09 visionCATSuser

Development 1 - Chamber:

Executed	11-Oct-2019 18:09:52 visionCATSuser

Take image developed plate 1a - Visualizer (S/N: 230515):

Executed	11-Oct-2019 19:02:26 visionCATSuser

RT White

Developed, RemTransVis

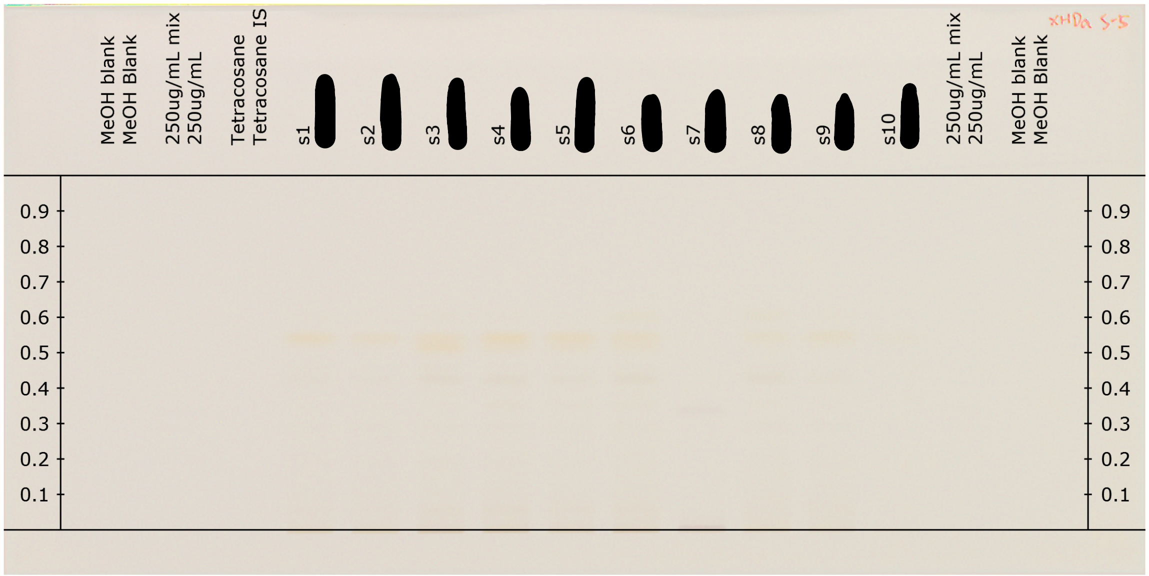

Exposure	0.084 s
Contrast	1
Normalized exposure	Disabled
Clarify	Disabled
White balance	1.00, 1.00, 1.00

R 254

Developed, Remission254

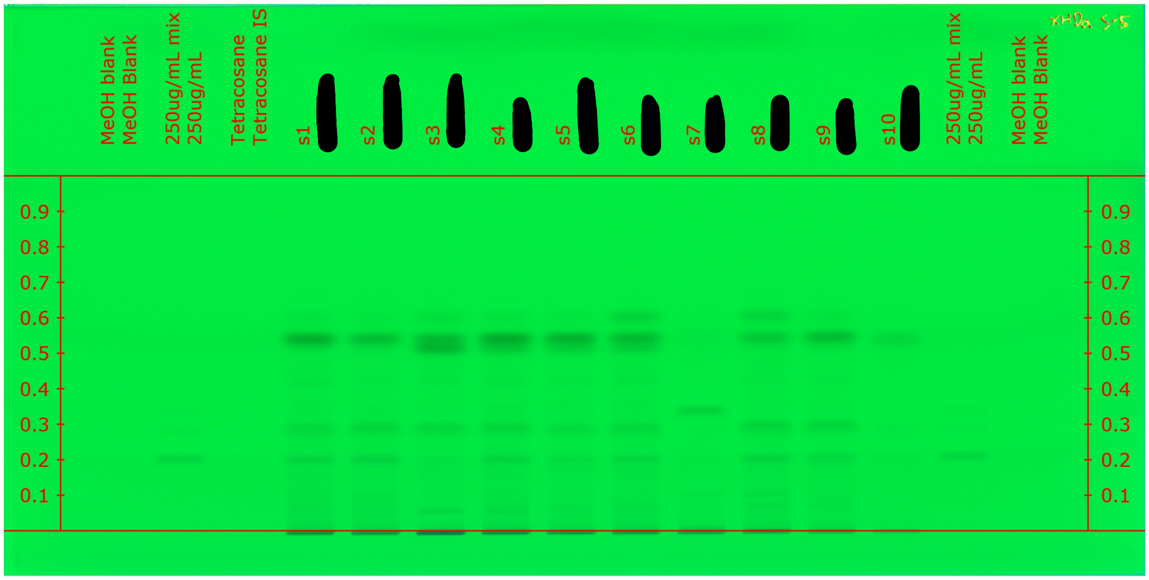

Exposure	0.269 s
Contrast	1
Normalized exposure	Disabled
Clarify	Disabled
White balance	1.00, 1.00, 1.00

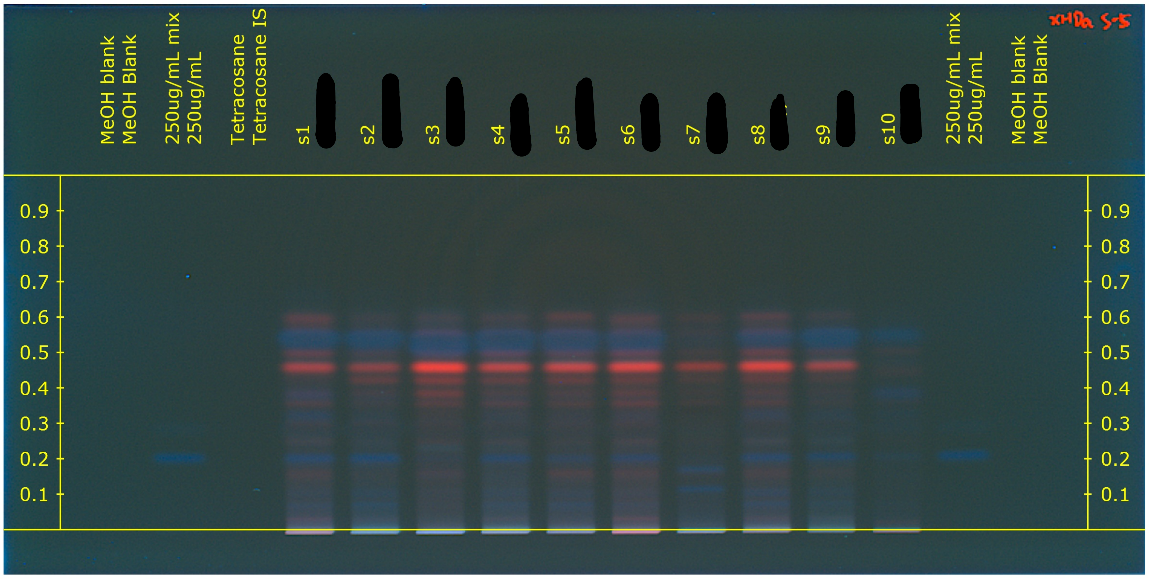

Exposure	5.168 s
Contrast	1
Normalized exposure	Disabled
Clarify	Disabled
White balance	1.00, 1.00, 1.00

Scan developed plate 1b - Scanner 3 (S/N: 031025):

Executed	11-Oct-2019 19:04:05 visionCATSuser

Scan:

Wavelength	254 nm

Track 1:

Type	Single λ

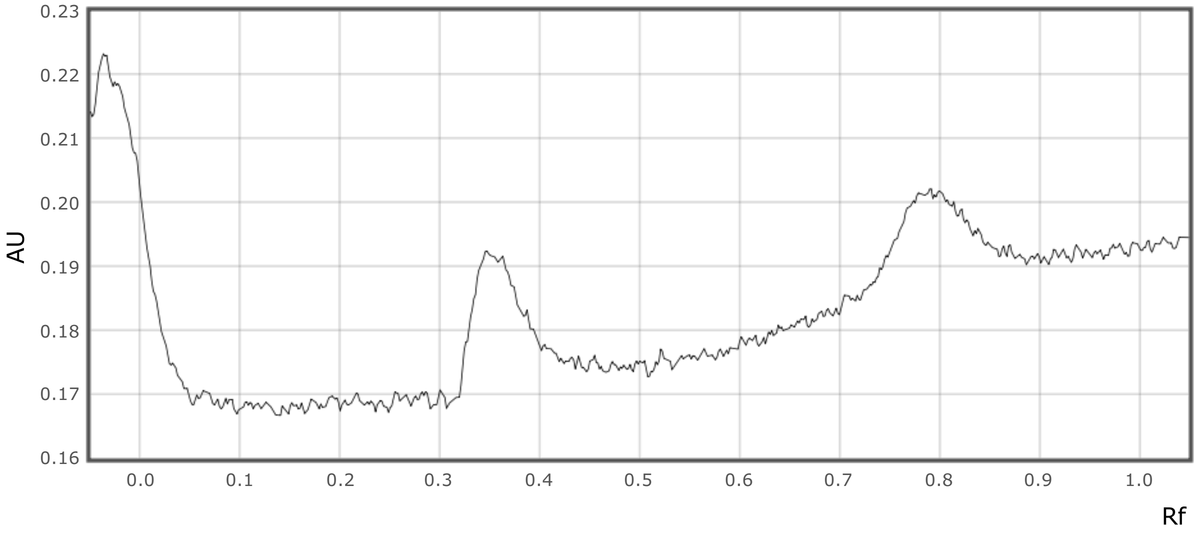

Track 2:

Type	Single λ

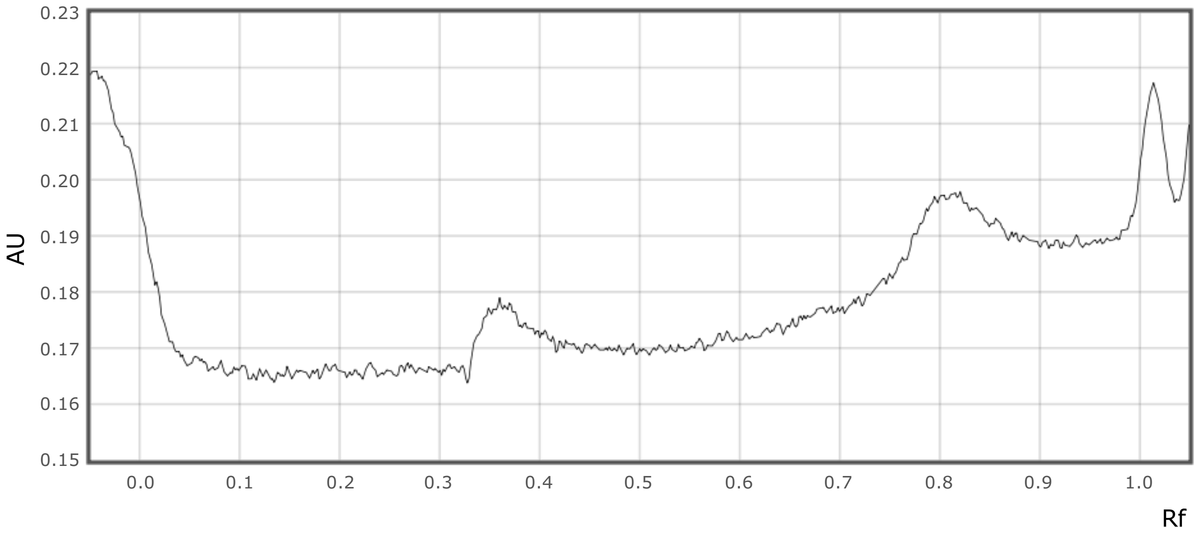

Track 3:

Type	Single λ

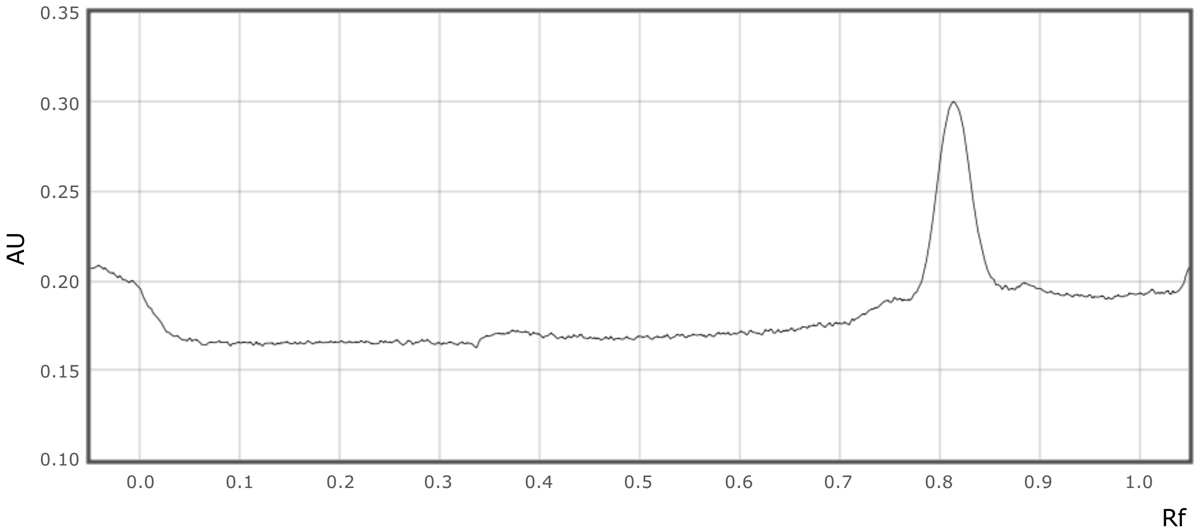

Track 4:

Type	Single λ

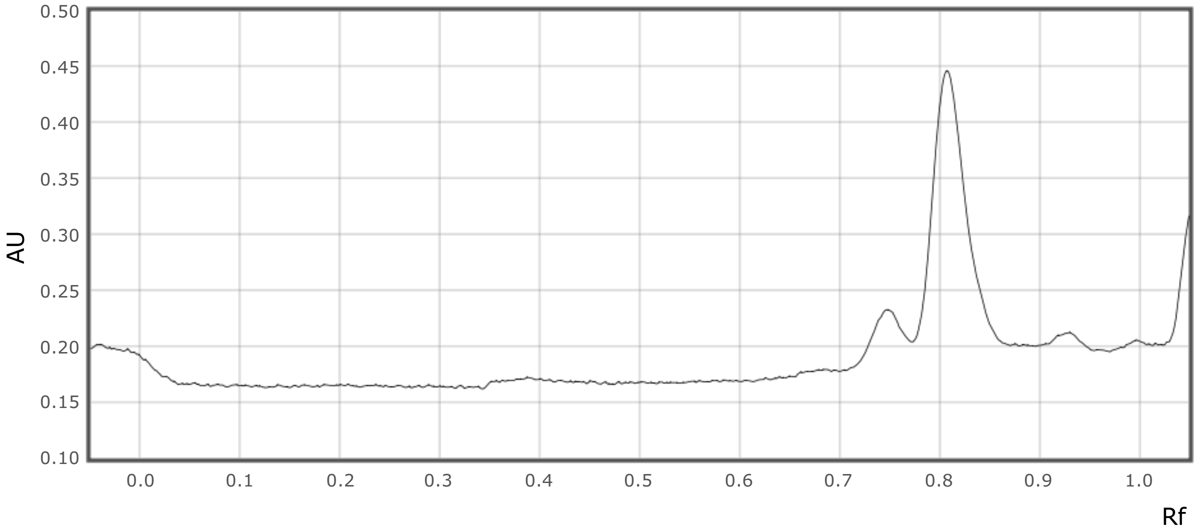

Track 5:	
Type	Single λ

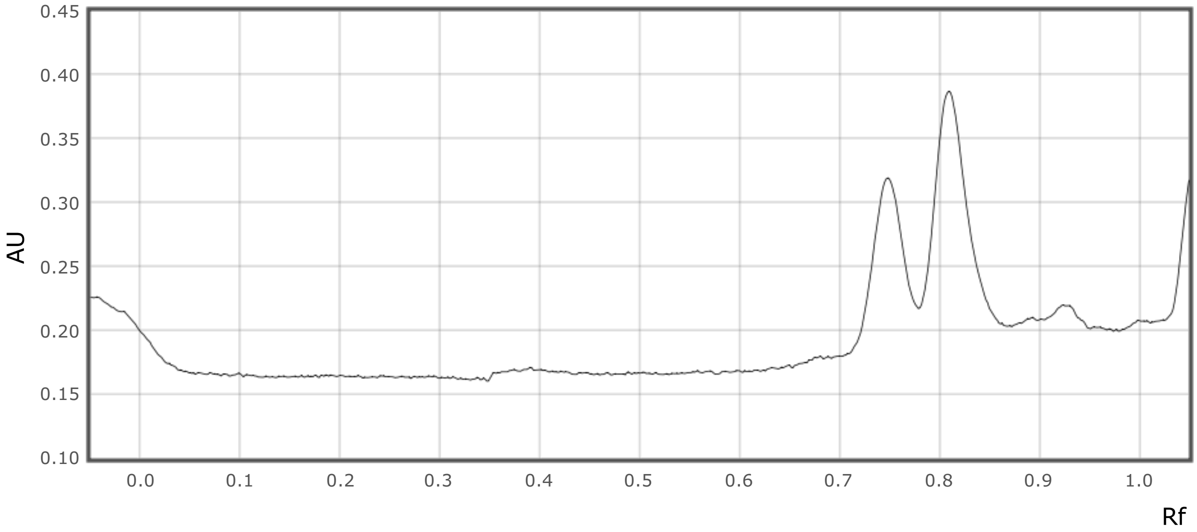

Track 6:	
Type	Single λ

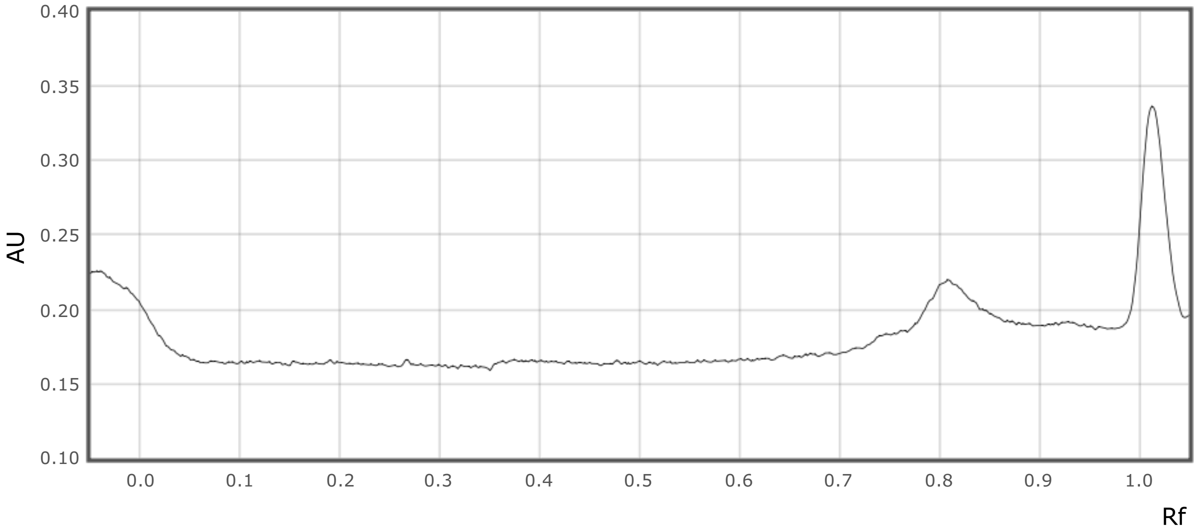

Track 7:

Type	Single λ

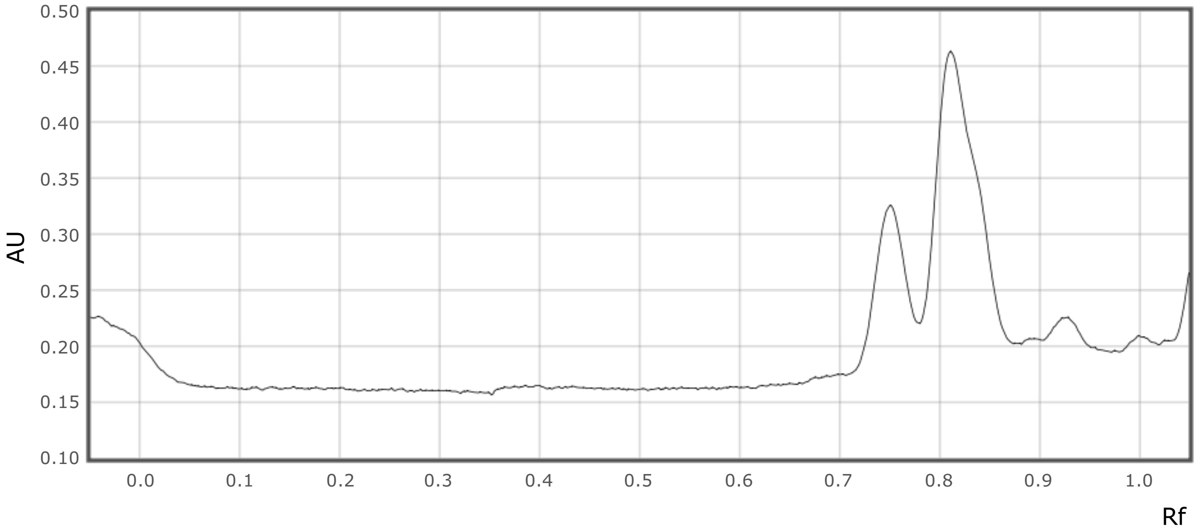

Track 8:

Type	Single λ

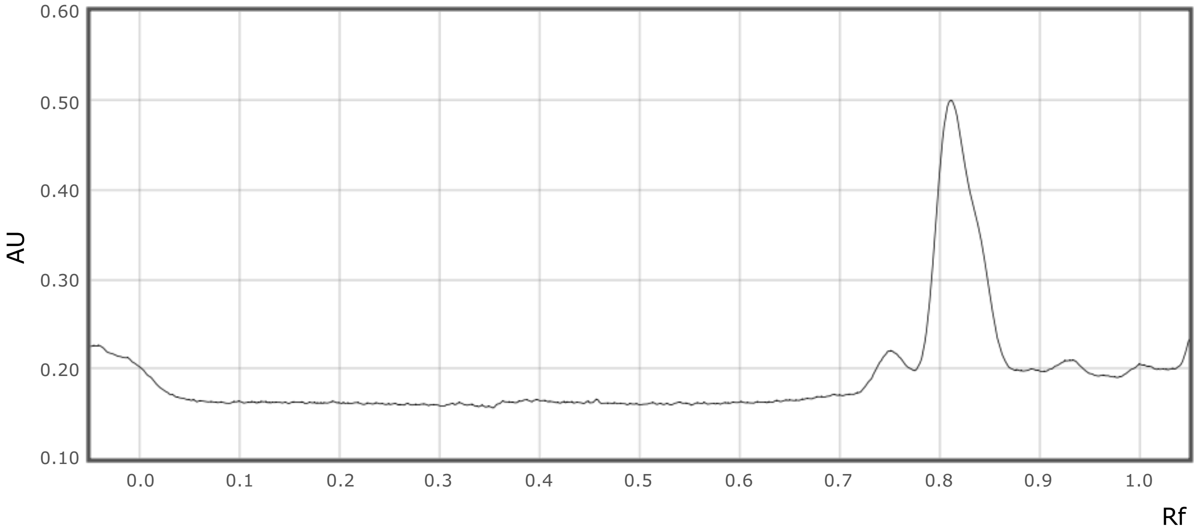

Track 9:	
Type	Single λ

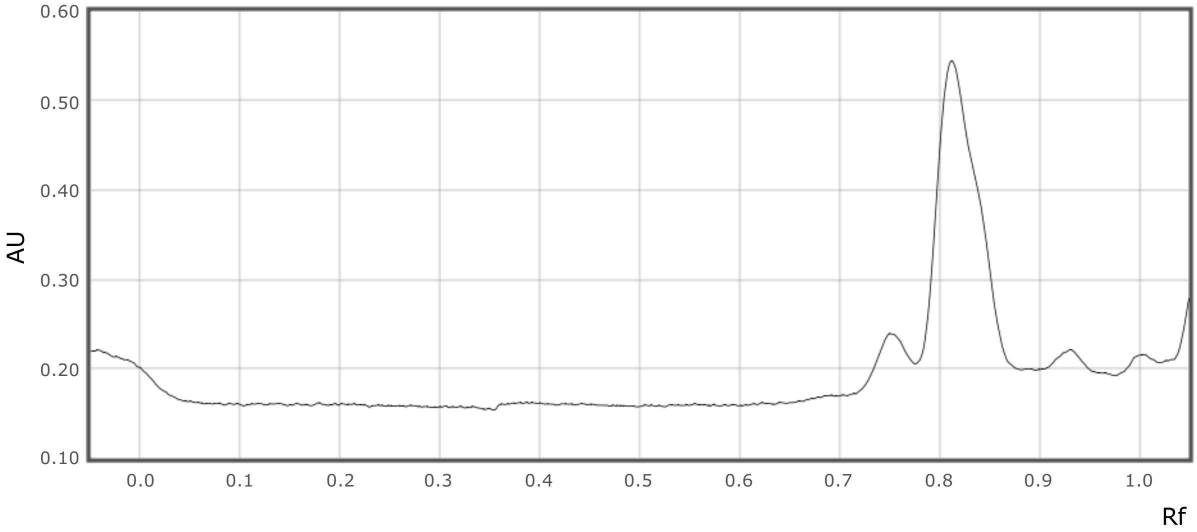

Track 10:	
Type	Single λ

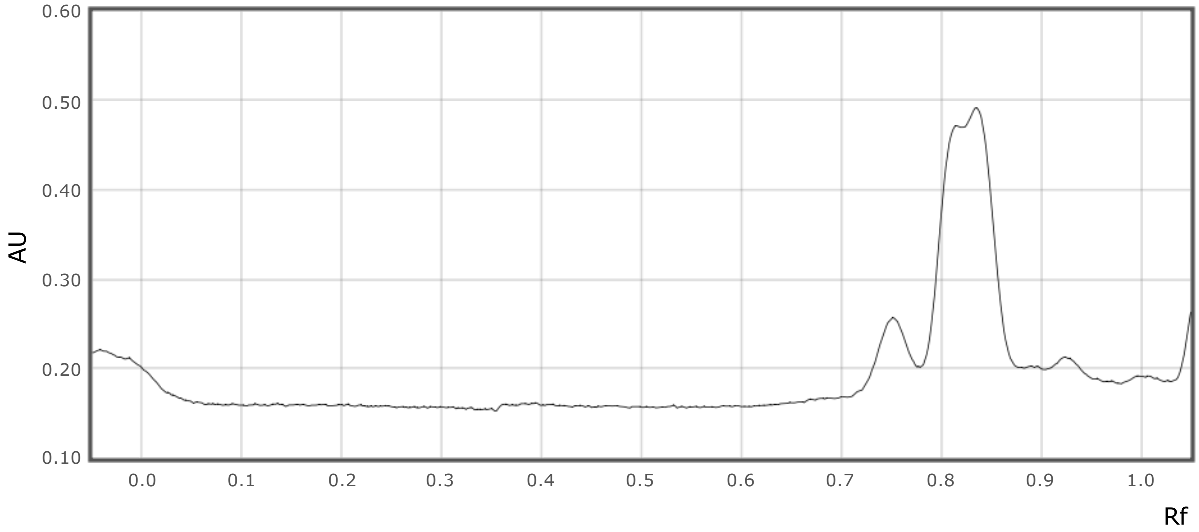

Track 11:	
Type	Single λ

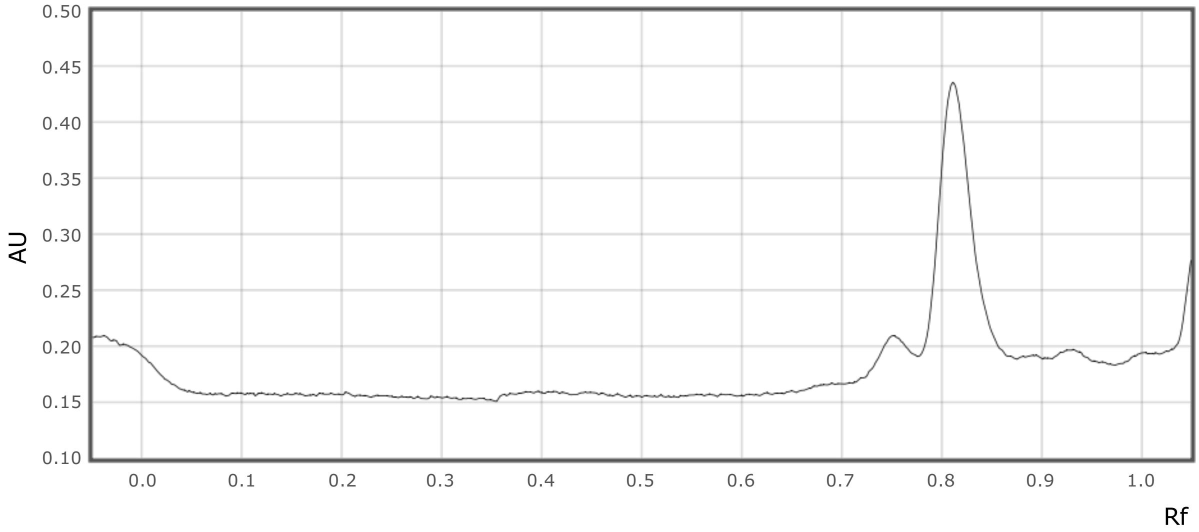

Track 12:	
Type	Single λ

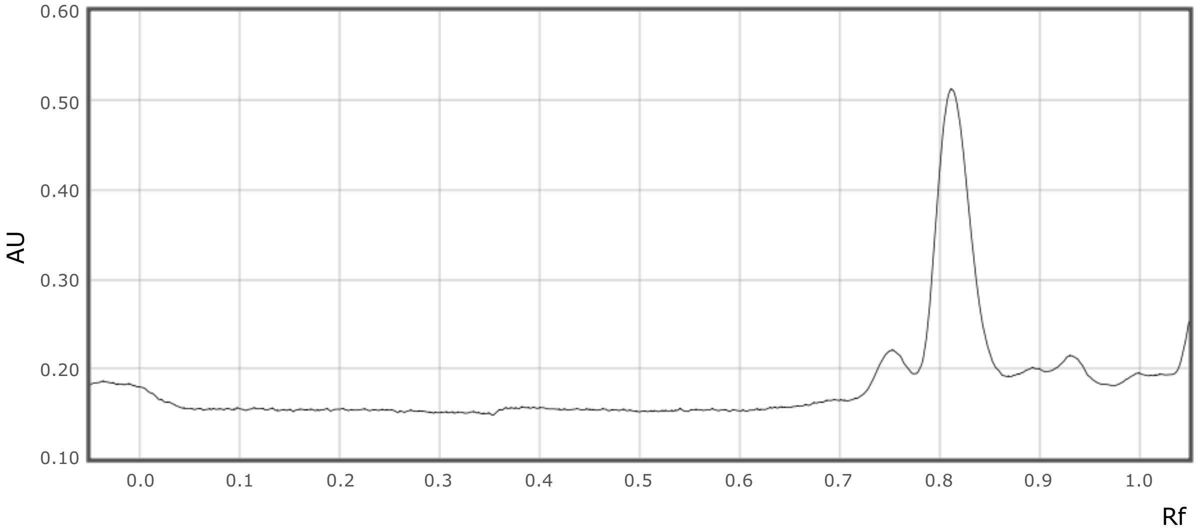

Track 13:	
Type	Single λ

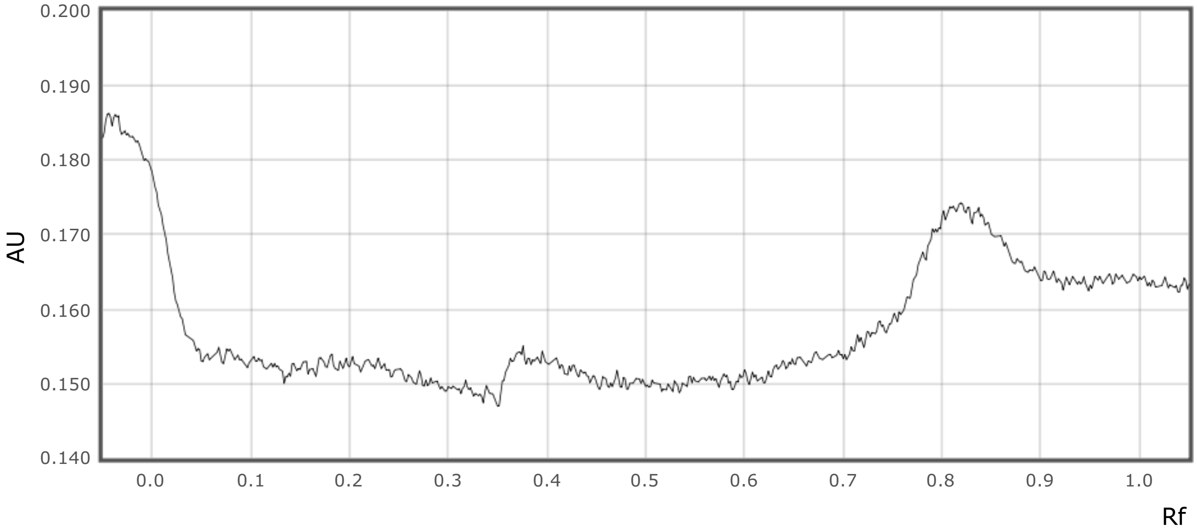

Track 14:	
Type	Single λ

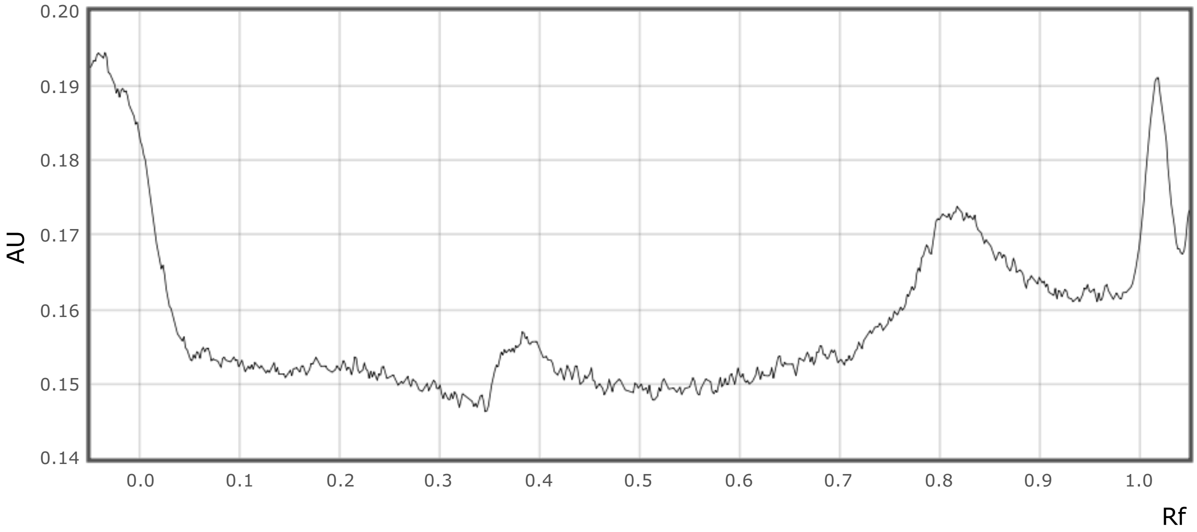

Track 15:

Type	Single λ

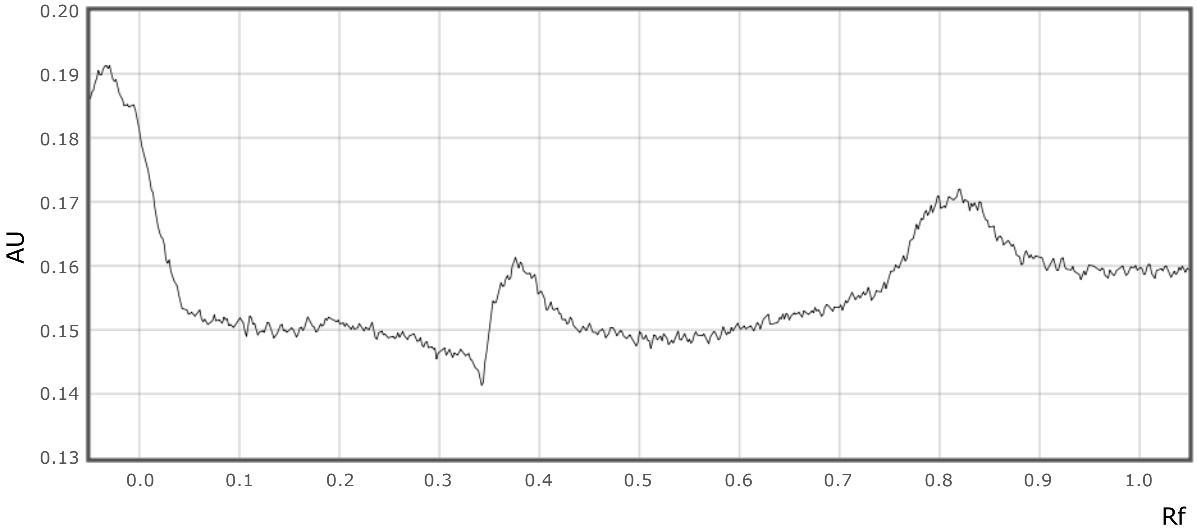

Derivatization 1 - dip:

Executed	11-Oct-2019 19:09:11 visionCATSuser

Take image derivatized plate 1a - Visualizer (S/N: 230515):

Executed	11-Oct-2019 19:11:59 visionCATSuser

RT White

Derivatized, RemTransVis

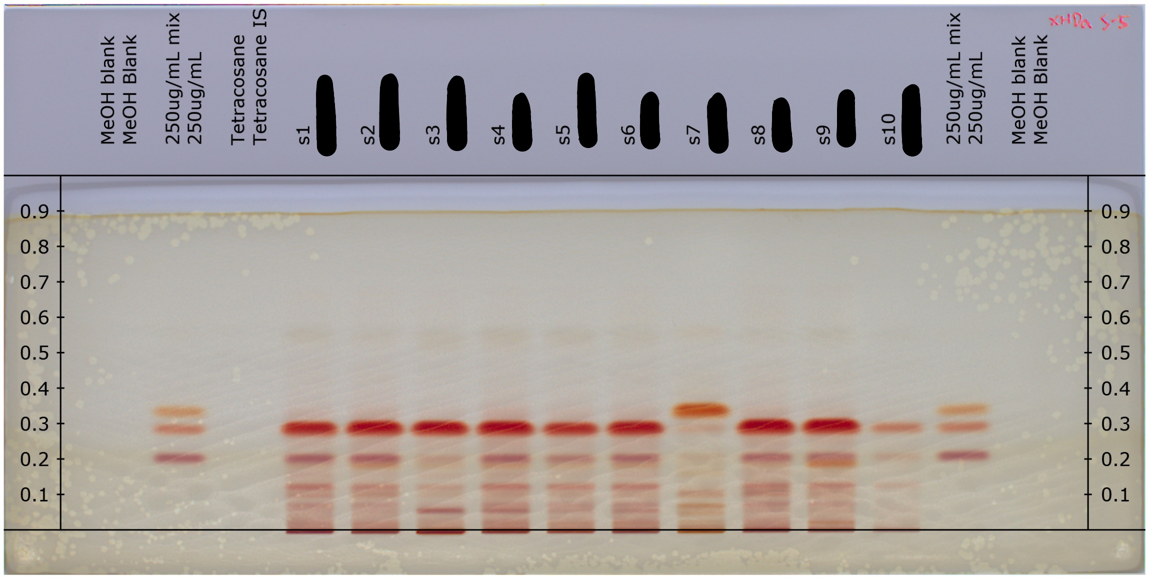

Exposure	0.041 s
Contrast	1
Normalized exposure	Disabled
Clarify	Disabled
White balance	1.15, 1.09, 0.82

R 366

Derivatized, Remission366

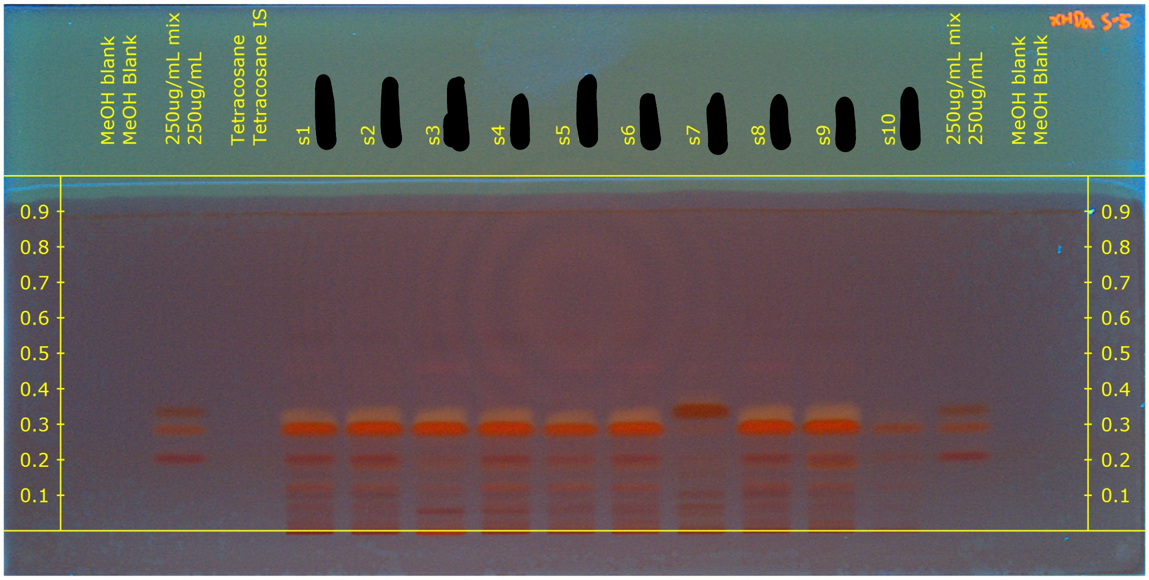

Exposure	9.999 s
Contrast	1
Normalized exposure	Disabled
Clarify	Disabled
White balance	1.00, 1.00, 1.00

Evaluation 1 :

Validated	false
Step	Take image derivatized plate 1a
Concentration unit type	Mass / volume
Notes	

Definition:

References:

250ug/mL mix

Substance Name	Concentration	Purity
9-THC	250.000 µg/ml	100.00 %
CBD	250.000 µg/ml	100.00 %
CBN	250.000 µg/ml	100.00 %

Samples:

Vial ID	Amount	Volume solution	Reference amount	Related to
MeOH blank		0.00 ml		
Tetracosane		0.00 ml		
s1		0.00 ml		
s2		0.00 ml		
s3		0.00 ml		
s4		0.00 ml		
s5		0.00 ml		
s6		0.00 ml		
s7		0.00 ml		
s8		0.00 ml		
s9		0.00 ml		
s10		0.00 ml		

Integration parameters:

Bounds	[0.000,1.000]
Smoothing	Savitzky–Golay of order 3 and window 7
Baseline correction	Lowest slope with noise 0.05
Profile subtraction	Profile subtraction from track 1
Peaks detection	Gauss (legacy) with sensitivity 0.1, separation 1 and threshold 0.1

Scan:

Wavelength	RT White

Track 1:

Type	Sample
Vial ID	MeOH blank
Description	MeOH Blank
Volume	2.0 µl

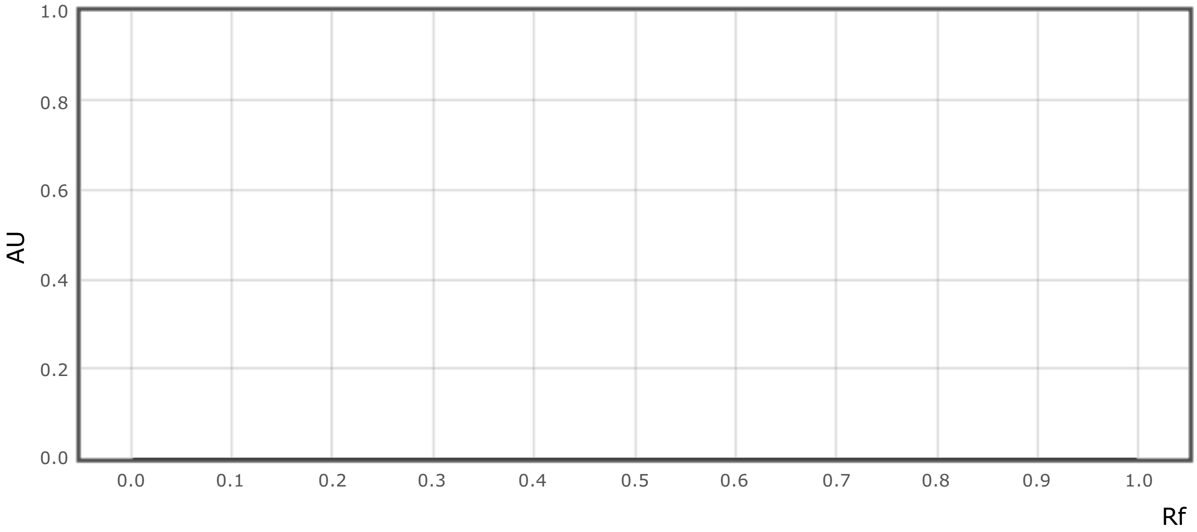

Peak #	Start		Max			End		Area		Manual peak	Substance Name
	Rf	H	Rf	H	%	Rf	H	A	%		

Track 2:	
Type	Reference
Vial ID	250ug/mL mix
Description	250ug/mL
Volume	2.0 µl

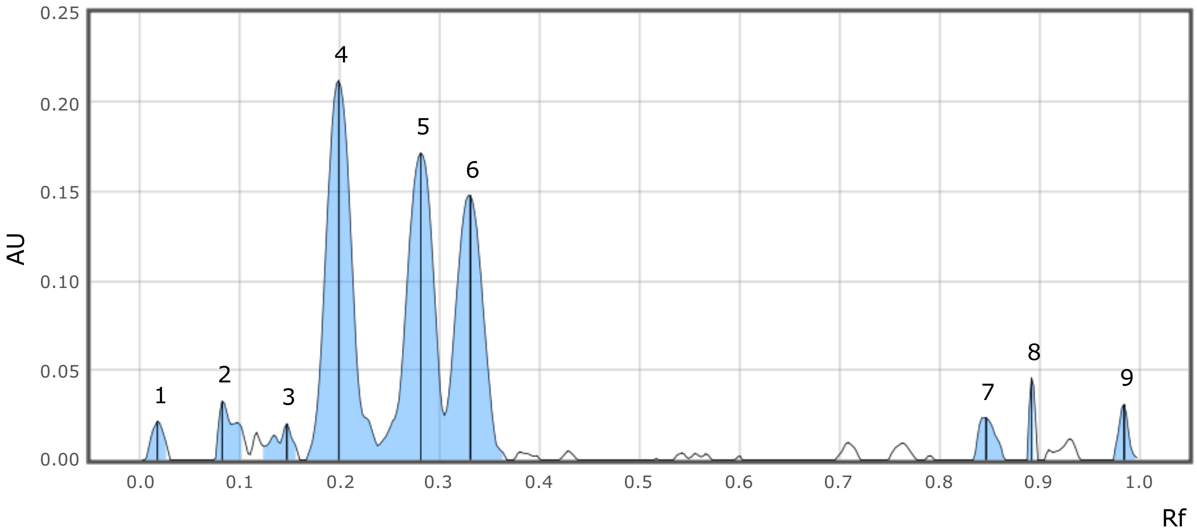

Peak #	Start Rf	Start H	Max Rf	Max H	Max %	End Rf	End H	Area A	Area %	Manual peak	Substance Name
1	0.004	0.0000	0.017	0.0217	3.07	0.030	0.0000	0.00031	1.68	No	
2	0.073	0.0000	0.082	0.0328	4.64	0.108	0.0035	0.00065	3.47	No	
3	0.123	0.0077	0.147	0.0202	2.86	0.160	0.0000	0.00041	2.22	No	
4	0.166	0.0000	0.199	0.2120	30.01	0.237	0.0079	0.00626	33.46	No	CBN
5	0.237	0.0079	0.281	0.1715	24.28	0.304	0.0248	0.00538	28.74	No	9-THC
6	0.304	0.0248	0.330	0.1477	20.91	0.367	0.0000	0.00459	24.55	No	CBD
7	0.834	0.0000	0.847	0.0235	3.32	0.866	0.0000	0.00046	2.46	No	
8	0.888	0.0000	0.892	0.0459	6.50	0.899	0.0000	0.00030	1.60	No	
9	0.974	0.0000	0.985	0.0311	4.40	0.998	0.0012	0.00034	1.82	No	

Track 3:

Type	Sample
Vial ID	Tetracosane
Description	Tetracosane IS
Volume	2.0 µl

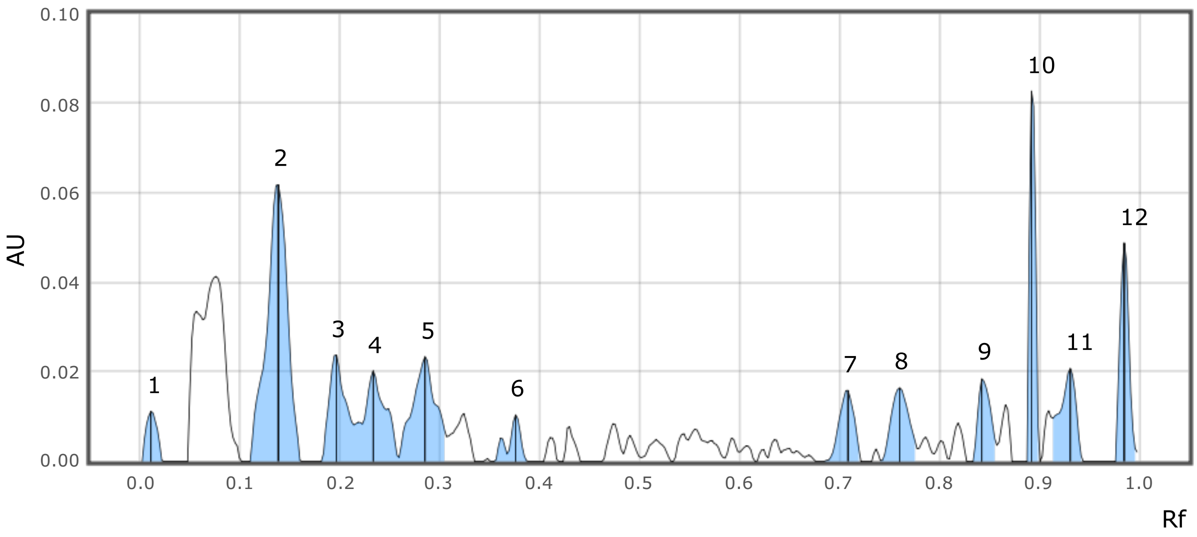

Peak #	Start Rf	Start H	Max Rf	Max H	Max %	End Rf	End H	Area A	Area %	Manual peak	Substance Name
1	0.002	0.0000	0.011	0.0112	3.16	0.023	0.0000	0.00014	2.48	No	
2	0.110	0.0000	0.138	0.0617	17.46	0.162	0.0000	0.00147	25.99	No	
3	0.181	0.0000	0.196	0.0238	6.72	0.214	0.0081	0.00045	7.88	No	
4	0.214	0.0081	0.233	0.0202	5.72	0.259	0.0008	0.00051	9.02	No	
5	0.259	0.0008	0.285	0.0233	6.60	0.307	0.0055	0.00062	10.98	No	
6	0.354	0.0000	0.376	0.0104	2.94	0.389	0.0000	0.00014	2.53	No	
7	0.685	0.0000	0.709	0.0158	4.48	0.722	0.0000	0.00027	4.78	No	
8	0.741	0.0002	0.760	0.0164	4.65	0.778	0.0028	0.00034	5.94	No	
9	0.834	0.0000	0.843	0.0184	5.22	0.858	0.0036	0.00026	4.66	No	
10	0.888	0.0000	0.892	0.0827	23.39	0.901	0.0000	0.00058	10.24	No	
11	0.914	0.0096	0.931	0.0208	5.87	0.944	0.0000	0.00037	6.53	No	
12	0.977	0.0000	0.985	0.0488	13.79	0.998	0.0020	0.00051	8.95	No	

Track 4:

Type	Sample
Vial ID	s1
Description	[redacted]
Volume	2.0 µl

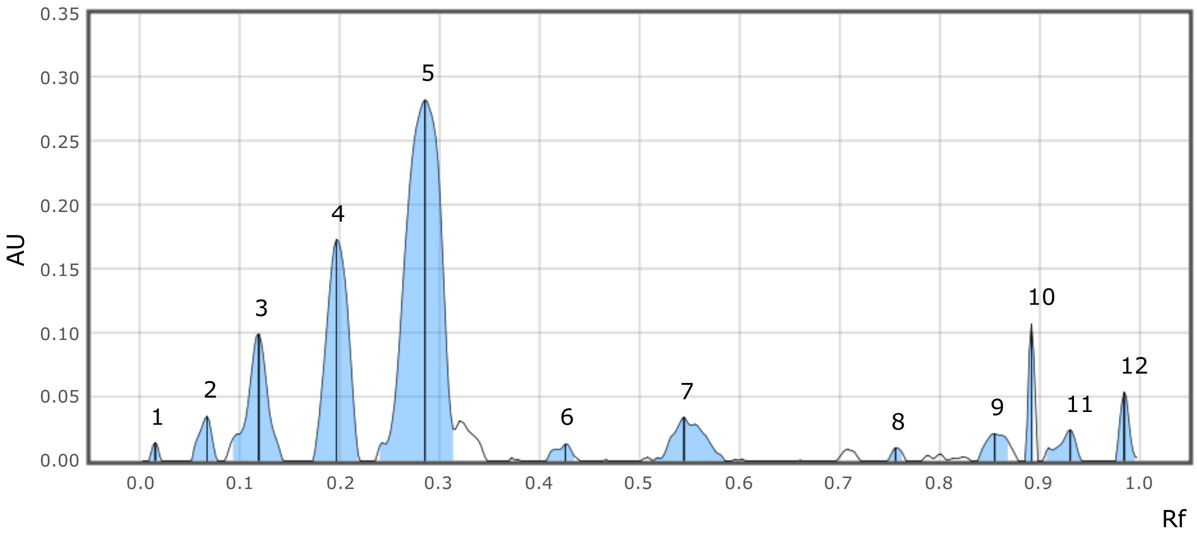

Peak #	Start Rf	Start H	Max Rf	Max H	Max %	End Rf	End H	Area A	Area %	Manual peak	Substance Name
1	0.008	0.0000	0.015	0.0144	1.65	0.021	0.0000	0.00011	0.47	No	
2	0.049	0.0000	0.067	0.0349	4.01	0.078	0.0000	0.00051	2.24	No	
3	0.093	0.0174	0.119	0.0995	11.45	0.144	0.0000	0.00225	9.93	No	
4	0.173	0.0000	0.196	0.1734	19.95	0.220	0.0000	0.00435	19.22	No	
5	0.240	0.0118	0.285	0.2825	32.50	0.315	0.0246	0.01148	50.72	No	9-THC
6	0.406	0.0000	0.425	0.0133	1.53	0.441	0.0000	0.00025	1.12	No	
7	0.514	0.0003	0.544	0.0343	3.95	0.585	0.0000	0.00121	5.34	No	
8	0.747	0.0000	0.756	0.0103	1.18	0.767	0.0000	0.00012	0.53	No	
9	0.838	0.0000	0.856	0.0213	2.45	0.879	0.0000	0.00057	2.53	No	
10	0.886	0.0000	0.892	0.1071	12.33	0.899	0.0000	0.00075	3.33	No	
11	0.907	0.0080	0.931	0.0244	2.81	0.944	0.0000	0.00047	2.08	No	
12	0.977	0.0000	0.985	0.0537	6.18	0.998	0.0024	0.00057	2.50	No	

Track 5:

Type	Sample
Vial ID	s2
Description	[redacted]
Volume	2.0 µl

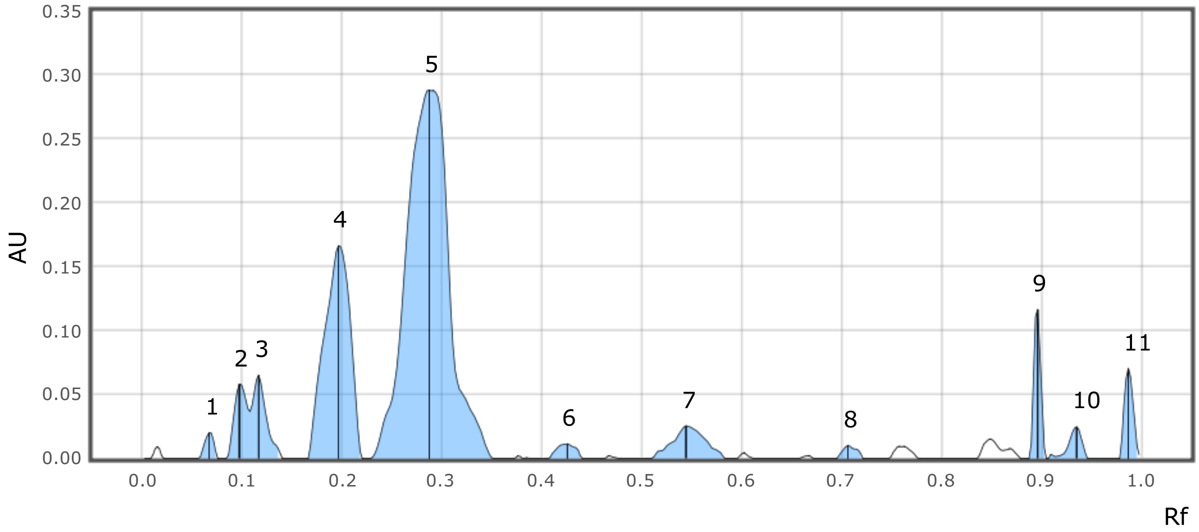

Peak #	Start Rf	Start H	Max Rf	Max H	Max %	End Rf	End H	Area A	Area %	Manual peak	Substance Name
1	0.056	0.0000	0.067	0.0201	2.34	0.075	0.0000	0.00021	0.85	No	
2	0.084	0.0000	0.097	0.0580	6.77	0.108	0.0366	0.00088	3.52	No	
3	0.108	0.0366	0.116	0.0650	7.59	0.140	0.0000	0.00103	4.15	No	
4	0.166	0.0000	0.196	0.1664	19.44	0.220	0.0000	0.00498	20.01	No	
5	0.229	0.0000	0.287	0.2881	33.65	0.352	0.0000	0.01438	57.79	No	9-THC
6	0.406	0.0000	0.425	0.0114	1.33	0.441	0.0000	0.00025	1.01	No	
7	0.510	0.0000	0.544	0.0255	2.98	0.583	0.0000	0.00095	3.82	No	
8	0.696	0.0000	0.706	0.0101	1.18	0.722	0.0000	0.00016	0.65	No	
9	0.888	0.0000	0.897	0.1165	13.61	0.905	0.0000	0.00094	3.78	No	
10	0.907	0.0000	0.935	0.0247	2.88	0.948	0.0000	0.00037	1.47	No	
11	0.979	0.0000	0.987	0.0705	8.23	0.998	0.0029	0.00073	2.94	No	

Track 6:	
Type	Sample
Vial ID	s3
Description	[redacted]
Volume	2.0 µl

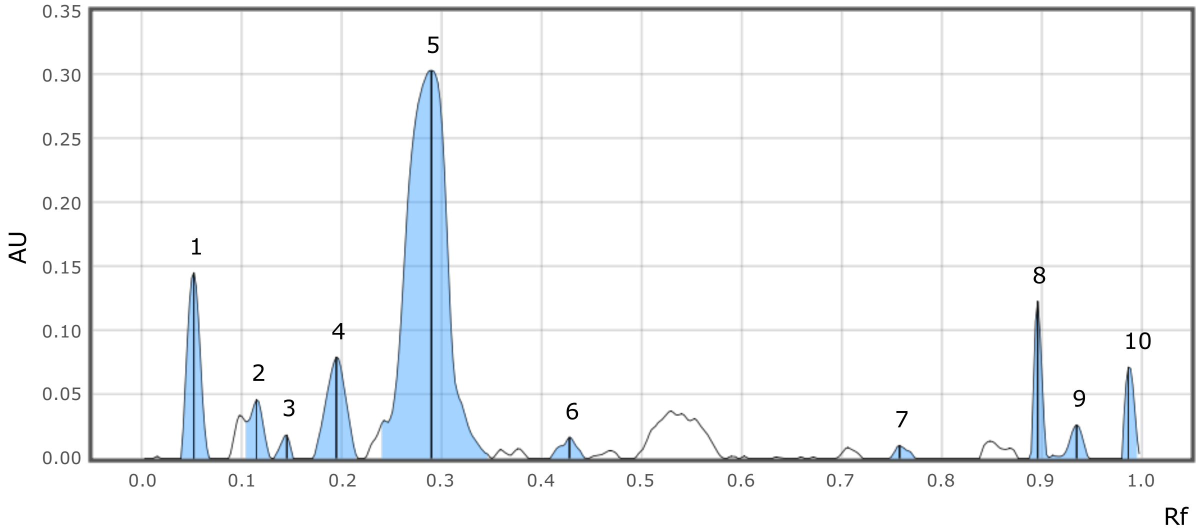

Peak #	Start		Max			End		Area		Manual peak	Substance Name
	Rf	H	Rf	H	%	Rf	H	A	%		
1	0.039	0.0000	0.052	0.1454	17.31	0.069	0.0000	0.00213	9.77	No	
2	0.103	0.0287	0.114	0.0460	5.47	0.129	0.0000	0.00072	3.31	No	
3	0.132	0.0000	0.144	0.0184	2.19	0.153	0.0000	0.00019	0.89	No	
4	0.170	0.0000	0.194	0.0793	9.45	0.216	0.0000	0.00181	8.32	No	
5	0.240	0.0272	0.289	0.3034	36.13	0.348	0.0013	0.01425	65.50	No	9-THC
6	0.408	0.0000	0.428	0.0166	1.98	0.445	0.0000	0.00030	1.40	No	
7	0.747	0.0000	0.758	0.0100	1.19	0.776	0.0000	0.00015	0.67	No	
8	0.888	0.0000	0.897	0.1229	14.64	0.907	0.0007	0.00105	4.84	No	
9	0.907	0.0007	0.935	0.0263	3.13	0.948	0.0000	0.00040	1.83	No	
10	0.981	0.0000	0.987	0.0714	8.50	0.998	0.0034	0.00075	3.46	No	

Track 7:

Type	Sample
Vial ID	s4
Description	[redacted]
Volume	2.0 µl

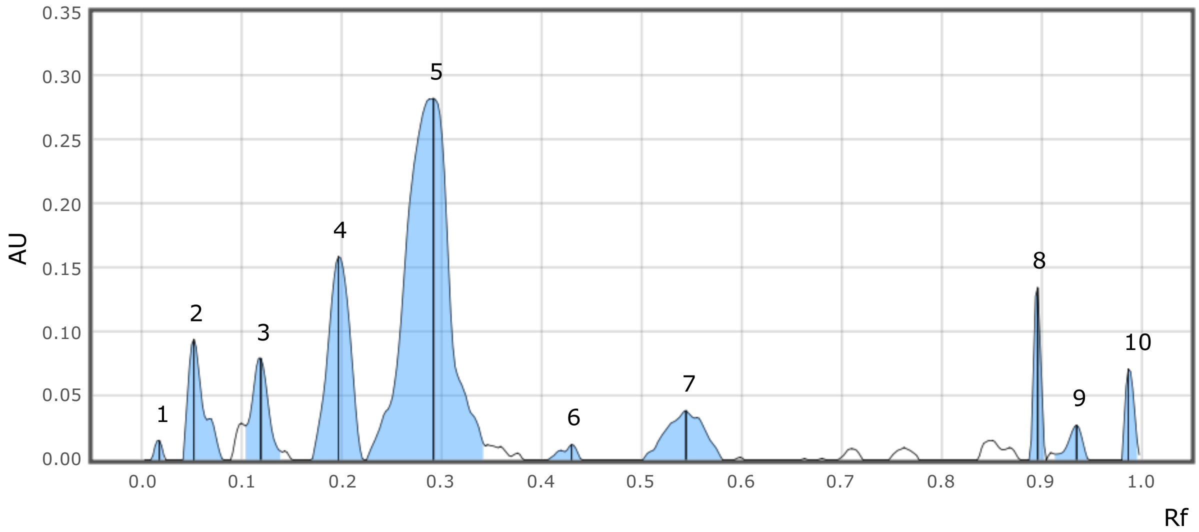

Peak #	Start Rf	Start H	Max Rf	Max H	Max %	End Rf	End H	Area A	Area %	Manual peak	Substance Name
1	0.008	0.0000	0.017	0.0152	1.67	0.023	0.0000	0.00013	0.49	No	
2	0.039	0.0000	0.052	0.0943	10.32	0.082	0.0000	0.00170	6.56	No	
3	0.103	0.0264	0.119	0.0795	8.70	0.140	0.0061	0.00153	5.88	No	
4	0.168	0.0000	0.196	0.1589	17.39	0.222	0.0000	0.00405	15.61	No	
5	0.224	0.0000	0.291	0.2825	30.91	0.343	0.0108	0.01438	55.41	No	9-THC
6	0.406	0.0000	0.430	0.0120	1.32	0.441	0.0000	0.00021	0.81	No	
7	0.501	0.0000	0.544	0.0385	4.22	0.581	0.0000	0.00169	6.52	No	
8	0.888	0.0000	0.897	0.1346	14.73	0.905	0.0000	0.00110	4.23	No	
9	0.914	0.0044	0.935	0.0272	2.98	0.948	0.0000	0.00044	1.71	No	
10	0.981	0.0000	0.987	0.0710	7.77	0.998	0.0038	0.00073	2.79	No	

Track 8:

Type	Sample
Vial ID	s5
Description	[redacted]
Volume	2.0 µl

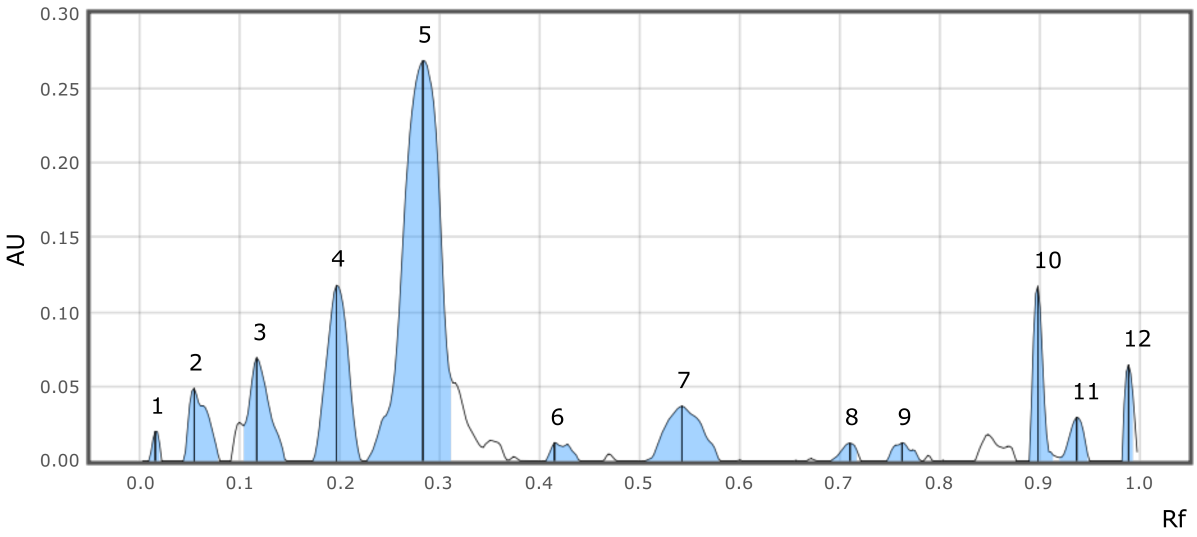

Peak #	Start Rf	Start H	Max Rf	Max H	Max %	End Rf	End H	Area A	Area %	Manual peak	Substance Name
1	0.008	0.0000	0.015	0.0198	2.46	0.021	0.0000	0.00015	0.71	No	
2	0.043	0.0000	0.054	0.0487	6.03	0.080	0.0000	0.00102	4.74	No	
3	0.103	0.0235	0.116	0.0693	8.57	0.147	0.0000	0.00156	7.24	No	
4	0.173	0.0000	0.196	0.1178	14.58	0.222	0.0000	0.00289	13.38	No	
5	0.227	0.0000	0.283	0.2683	33.22	0.313	0.0522	0.01132	52.41	No	9-THC
6	0.406	0.0000	0.415	0.0123	1.52	0.441	0.0000	0.00026	1.21	No	
7	0.505	0.0000	0.542	0.0367	4.54	0.581	0.0000	0.00153	7.06	No	
8	0.691	0.0000	0.711	0.0118	1.46	0.722	0.0000	0.00020	0.93	No	
9	0.747	0.0000	0.763	0.0122	1.51	0.782	0.0000	0.00026	1.18	No	
10	0.890	0.0000	0.899	0.1171	14.50	0.918	0.0026	0.00130	6.01	No	
11	0.920	0.0023	0.938	0.0293	3.63	0.951	0.0000	0.00048	2.20	No	
12	0.981	0.0000	0.989	0.0644	7.98	0.998	0.0058	0.00063	2.92	No	

Track 9:

Type	Sample
Vial ID	s6
Description	[redacted]
Volume	2.0 µl

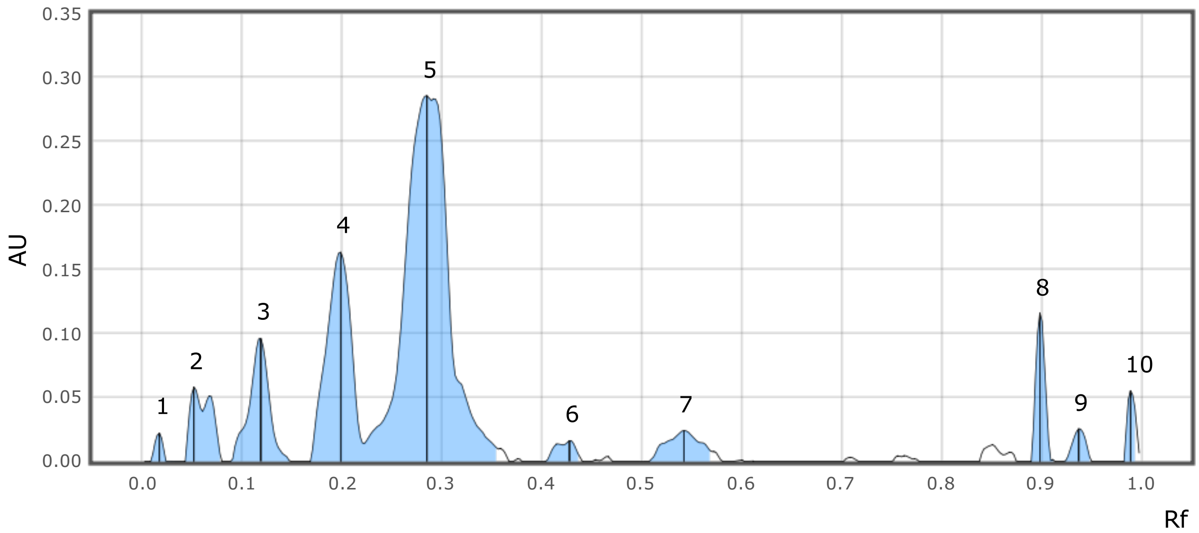

Peak	Start		Max			End		Area		Manual	Substance
#	Rf	H	Rf	H	%	Rf	H	A	%	peak	Name
1	0.008	0.0000	0.017	0.0220	2.55	0.026	0.0000	0.00020	0.74	No	
2	0.043	0.0000	0.052	0.0580	6.73	0.080	0.0000	0.00139	5.20	No	
3	0.088	0.0000	0.119	0.0959	11.12	0.149	0.0000	0.00219	8.18	No	
4	0.168	0.0000	0.199	0.1636	18.98	0.222	0.0138	0.00470	17.58	No	
5	0.222	0.0138	0.285	0.2857	33.14	0.356	0.0097	0.01483	55.43	No	9-THC
6	0.404	0.0000	0.428	0.0162	1.87	0.441	0.0000	0.00037	1.39	No	
7	0.508	0.0000	0.542	0.0240	2.79	0.570	0.0076	0.00095	3.53	No	
8	0.890	0.0000	0.899	0.1158	13.44	0.914	0.0000	0.00122	4.55	No	
9	0.922	0.0000	0.938	0.0256	2.96	0.951	0.0000	0.00038	1.40	No	
10	0.983	0.0000	0.989	0.0552	6.41	0.998	0.0063	0.00054	2.00	No	

Track 10:

Type	Sample
Vial ID	s7
Description	[redacted]
Volume	2.0 µl

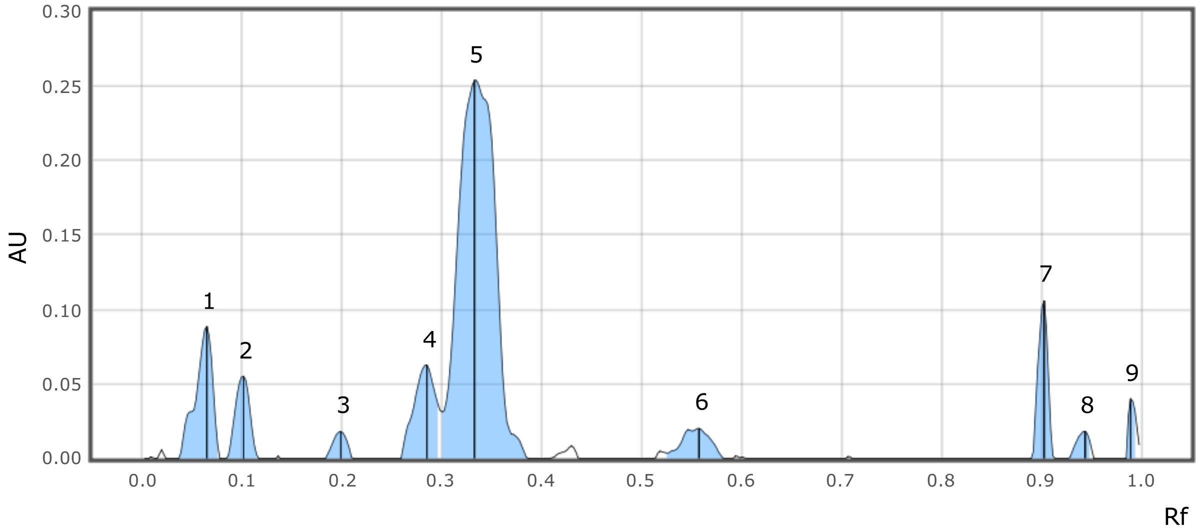

Peak #	Start		Max			End		Area		Manual peak	Substance Name
	Rf	H	Rf	H	%	Rf	H	A	%		
1	0.036	0.0000	0.065	0.0886	13.38	0.078	0.0000	0.00173	9.71	No	
2	0.084	0.0000	0.101	0.0552	8.34	0.116	0.0000	0.00092	5.14	No	
3	0.183	0.0000	0.199	0.0181	2.73	0.211	0.0000	0.00029	1.61	No	
4	0.259	0.0000	0.285	0.0625	9.44	0.298	0.0324	0.00156	8.76	No	9-THC
5	0.300	0.0311	0.333	0.2535	38.30	0.387	0.0000	0.01085	60.80	No	CBD
6	0.525	0.0036	0.557	0.0201	3.04	0.583	0.0000	0.00068	3.80	No	
7	0.890	0.0000	0.903	0.1057	15.98	0.914	0.0000	0.00119	6.66	No	
8	0.927	0.0000	0.944	0.0182	2.75	0.953	0.0000	0.00027	1.54	No	
9	0.985	0.0000	0.989	0.0400	6.05	0.998	0.0089	0.00035	1.98	No	

Track 11:

Type	Sample
Vial ID	s8
Description	[redacted]
Volume	2.0 µl

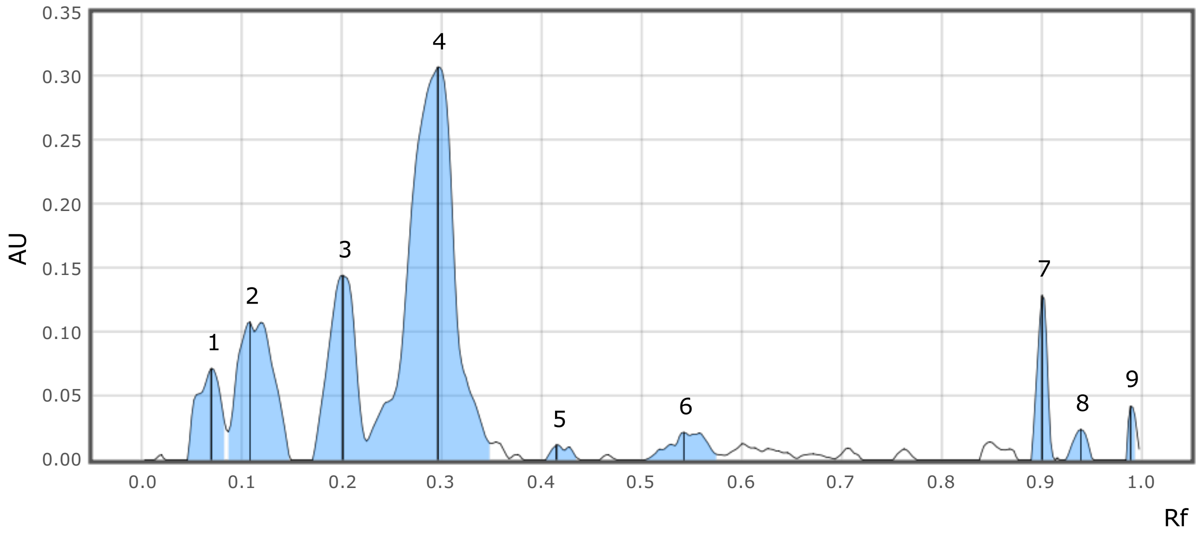

Peak	Start		Max			End		Area		Manual	Substance
#	Rf	H	Rf	H	%	Rf	H	A	%	peak	Name
1	0.045	0.0000	0.069	0.0715	8.33	0.084	0.0237	0.00194	6.37	No	
2	0.086	0.0220	0.108	0.1079	12.56	0.149	0.0000	0.00450	14.73	No	
3	0.170	0.0000	0.201	0.1444	16.81	0.224	0.0146	0.00447	14.64	No	
4	0.224	0.0146	0.296	0.3074	35.78	0.350	0.0128	0.01626	53.25	No	9-THC
5	0.404	0.0000	0.415	0.0117	1.37	0.438	0.0000	0.00024	0.79	No	
6	0.503	0.0000	0.542	0.0215	2.50	0.577	0.0040	0.00090	2.95	No	
7	0.890	0.0000	0.901	0.1288	14.99	0.914	0.0000	0.00146	4.80	No	
8	0.922	0.0000	0.940	0.0237	2.76	0.953	0.0000	0.00038	1.25	No	
9	0.985	0.0000	0.989	0.0421	4.91	0.998	0.0082	0.00038	1.23	No	

Track 12:

Type	Sample
Vial ID	s9
Description	[redacted]
Volume	2.0 µl

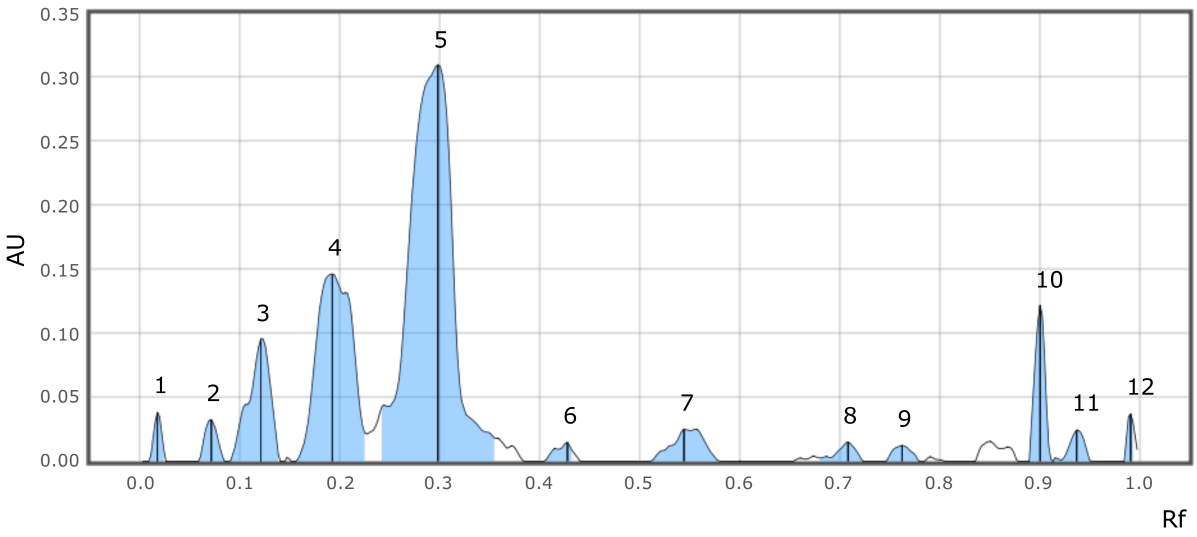

Peak	Start		Max			End		Area		Manual	Substance
#	Rf	H	Rf	H	%	Rf	H	A	%	peak	Name
1	0.008	0.0000	0.017	0.0382	4.37	0.026	0.0000	0.00034	1.19	No	
2	0.058	0.0000	0.071	0.0325	3.73	0.086	0.0000	0.00047	1.64	No	
3	0.088	0.0000	0.121	0.0956	10.95	0.140	0.0000	0.00240	8.29	No	
4	0.155	0.0000	0.192	0.1464	16.76	0.227	0.0214	0.00617	21.35	No	
5	0.242	0.0427	0.298	0.3098	35.47	0.356	0.0179	0.01556	53.87	No	9-THC
6	0.404	0.0000	0.428	0.0147	1.69	0.441	0.0000	0.00028	0.98	No	
7	0.510	0.0000	0.544	0.0251	2.87	0.581	0.0000	0.00095	3.27	No	
8	0.680	0.0027	0.709	0.0151	1.72	0.724	0.0000	0.00032	1.09	No	
9	0.745	0.0000	0.763	0.0125	1.43	0.780	0.0000	0.00026	0.90	No	
10	0.890	0.0000	0.901	0.1219	13.96	0.914	0.0000	0.00142	4.90	No	
11	0.914	0.0000	0.938	0.0245	2.81	0.953	0.0000	0.00041	1.42	No	
12	0.985	0.0000	0.992	0.0370	4.23	0.998	0.0090	0.00032	1.09	No	

Track 13:

Type	Sample
Vial ID	s10
Description	[redacted]
Volume	2.0 µl

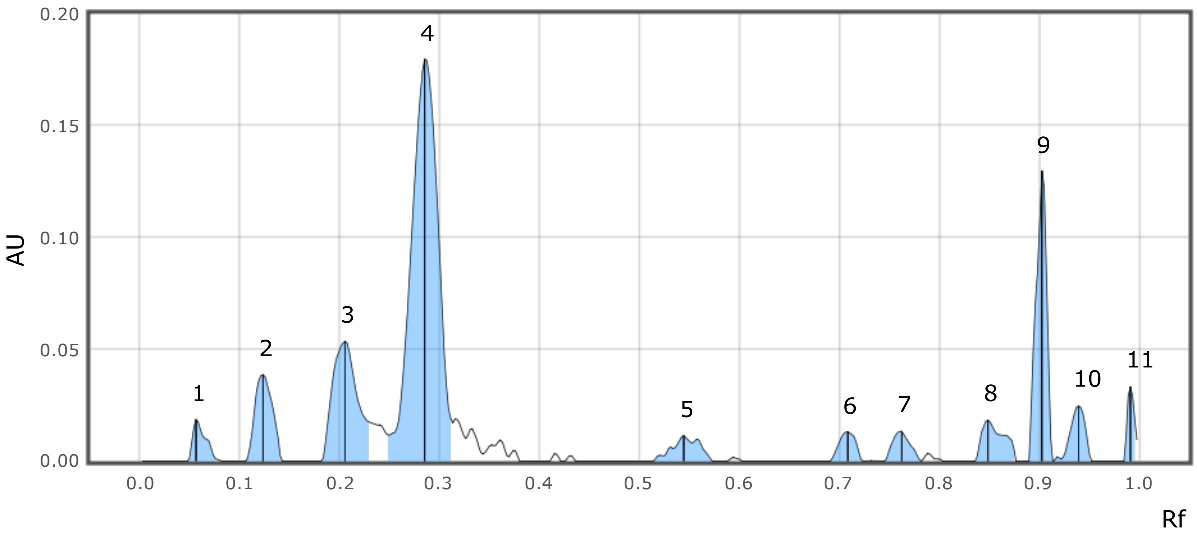

Peak #	Start Rf	Start H	Max Rf	Max H	Max %	End Rf	End H	Area A	Area %	Manual peak	Substance Name
1	0.047	0.0000	0.056	0.0187	3.49	0.082	0.0000	0.00027	2.29	No	
2	0.106	0.0000	0.123	0.0387	7.23	0.142	0.0000	0.00079	6.66	No	
3	0.181	0.0000	0.205	0.0535	10.01	0.237	0.0162	0.00168	14.10	No	
4	0.248	0.0116	0.285	0.1798	33.63	0.313	0.0172	0.00553	46.53	No	9-THC
5	0.514	0.0000	0.544	0.0114	2.14	0.575	0.0000	0.00035	2.97	No	
6	0.691	0.0000	0.709	0.0131	2.46	0.724	0.0000	0.00025	2.11	No	
7	0.745	0.0000	0.763	0.0134	2.50	0.782	0.0000	0.00027	2.27	No	
8	0.836	0.0000	0.849	0.0182	3.41	0.877	0.0000	0.00047	3.93	No	
9	0.890	0.0000	0.903	0.1297	24.27	0.914	0.0000	0.00159	13.33	No	
10	0.914	0.0000	0.940	0.0247	4.62	0.953	0.0000	0.00042	3.53	No	
11	0.985	0.0000	0.992	0.0333	6.23	0.998	0.0095	0.00027	2.28	No	

Track 14:

Type	Reference
Vial ID	250ug/mL mix
Description	250ug/mL
Volume	2.0 µl

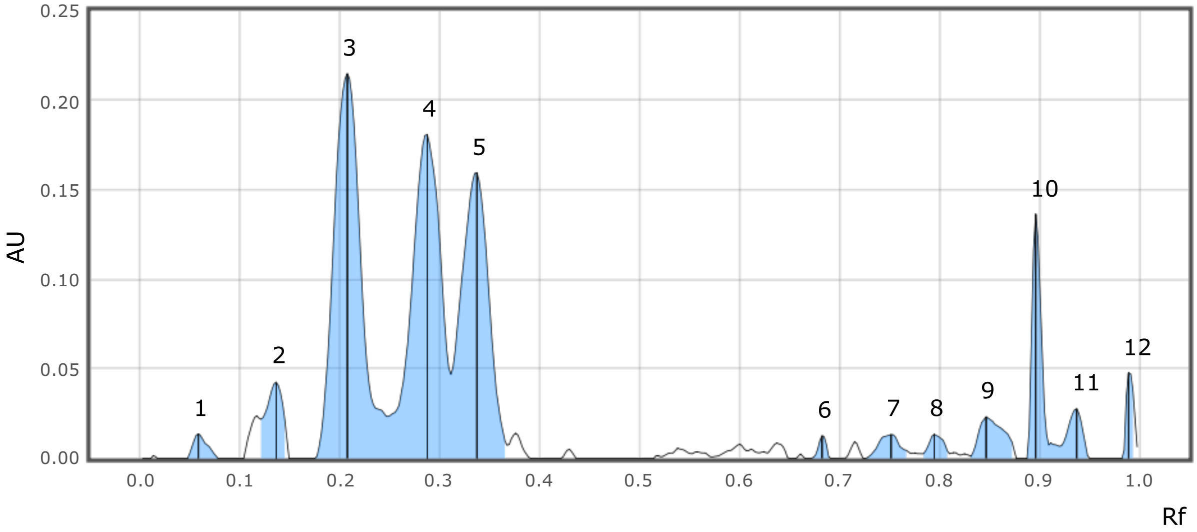

Peak #	Start Rf	Start H	Max Rf	Max H	Max %	End Rf	End H	Area A	Area %	Manual peak	Substance Name
1	0.047	0.0000	0.058	0.0137	1.54	0.080	0.0000	0.00022	0.95	No	
2	0.121	0.0218	0.136	0.0426	4.81	0.149	0.0000	0.00083	3.63	No	
3	0.175	0.0000	0.207	0.2147	24.26	0.248	0.0234	0.00678	29.79	No	CBN
4	0.248	0.0234	0.287	0.1808	20.42	0.311	0.0468	0.00612	26.90	No	9-THC
5	0.311	0.0468	0.337	0.1595	18.01	0.367	0.0074	0.00509	22.38	No	CBD
6	0.672	0.0000	0.683	0.0126	1.43	0.691	0.0000	0.00011	0.48	No	
7	0.726	0.0000	0.752	0.0132	1.50	0.771	0.0028	0.00033	1.47	No	
8	0.782	0.0025	0.795	0.0133	1.51	0.810	0.0026	0.00024	1.04	No	
9	0.832	0.0010	0.847	0.0232	2.62	0.877	0.0000	0.00067	2.93	No	
10	0.888	0.0000	0.897	0.1363	15.40	0.918	0.0071	0.00148	6.49	No	
11	0.920	0.0067	0.938	0.0276	3.12	0.951	0.0000	0.00047	2.06	No	
12	0.983	0.0000	0.989	0.0477	5.39	0.998	0.0063	0.00043	1.87	No	

Track 15:	
Type	Sample
Vial ID	MeOH blank
Description	MeOH Blank
Volume	2.0 µl

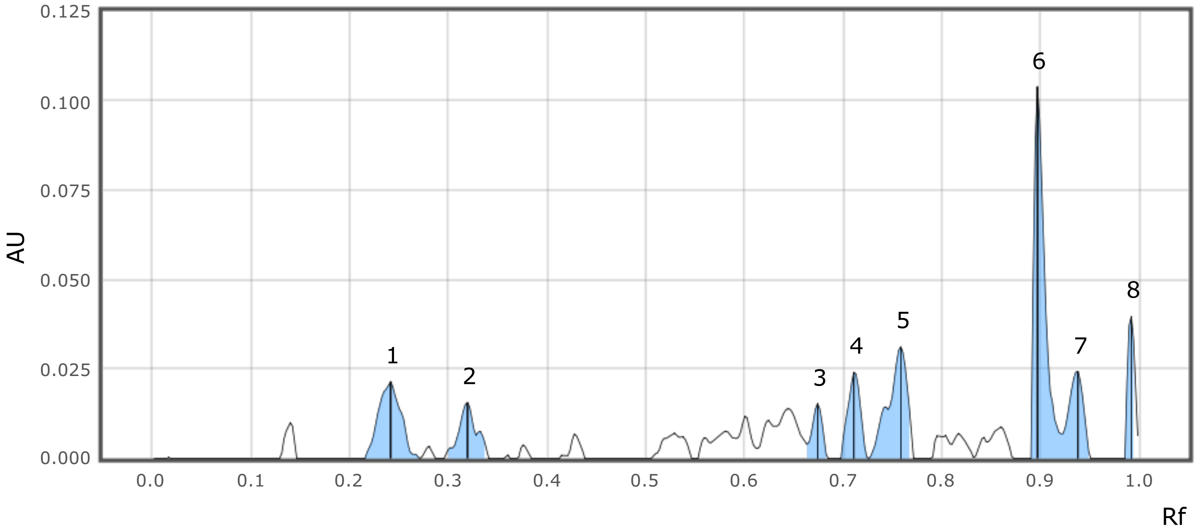

Peak #	Start		Max			End		Area		Manual peak	Substance Name
	Rf	H	Rf	H	%	Rf	H	A	%		
1	0.216	0.0000	0.242	0.0214	7.78	0.272	0.0000	0.00056	13.50	No	
2	0.298	0.0015	0.320	0.0156	5.67	0.341	0.0000	0.00032	7.70	No	
3	0.663	0.0039	0.674	0.0153	5.56	0.685	0.0000	0.00018	4.35	No	
4	0.698	0.0000	0.711	0.0241	8.74	0.726	0.0000	0.00035	8.41	No	
5	0.726	0.0000	0.758	0.0312	11.34	0.771	0.0000	0.00068	16.35	No	
6	0.890	0.0000	0.897	0.1038	37.71	0.920	0.0068	0.00130	31.39	No	
7	0.920	0.0068	0.938	0.0243	8.81	0.951	0.0000	0.00043	10.26	No	
8	0.985	0.0000	0.992	0.0396	14.39	0.998	0.0063	0.00033	8.04	No	

Calibration results:

Height calibration for substance 9-THC @ RT White:

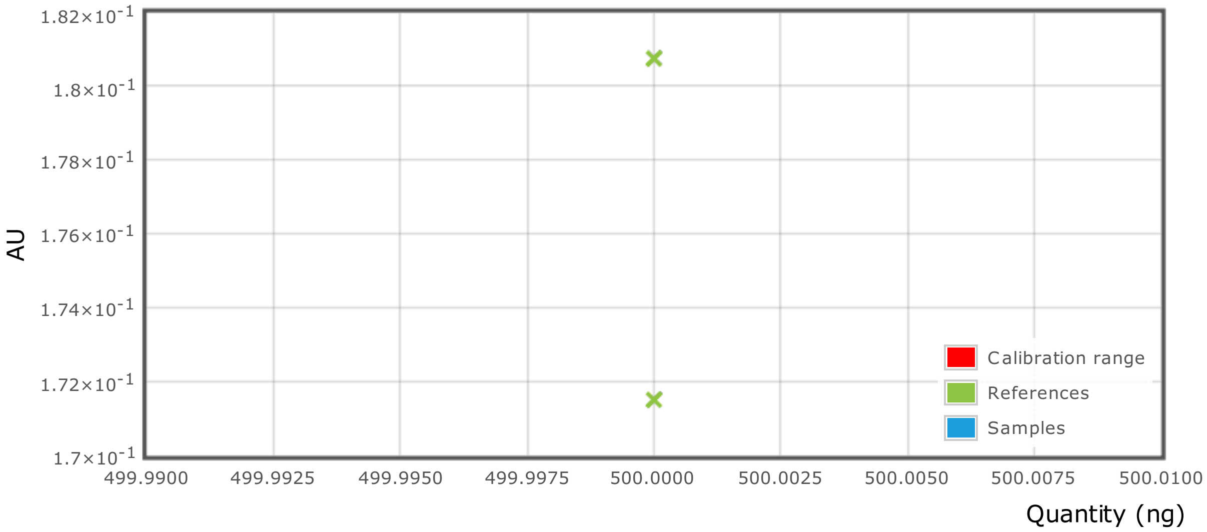

Regression mode	Linear-2
Range deviation	5.00 %
Related substances	Default
Number of references	2
Calibration function	$y=0x$
Coefficient of variation	CV 0.00 %
Correlation coefficient	n/a
⚠	Unable to compute the results for this substance because there wasn't enough groups of references replicas (at least 1 for Linear-1, 2 for Linear2 and Mime-1 and 3 for Polynomial and MiMe-2)

Height calibration for substance CBD @ RT White:

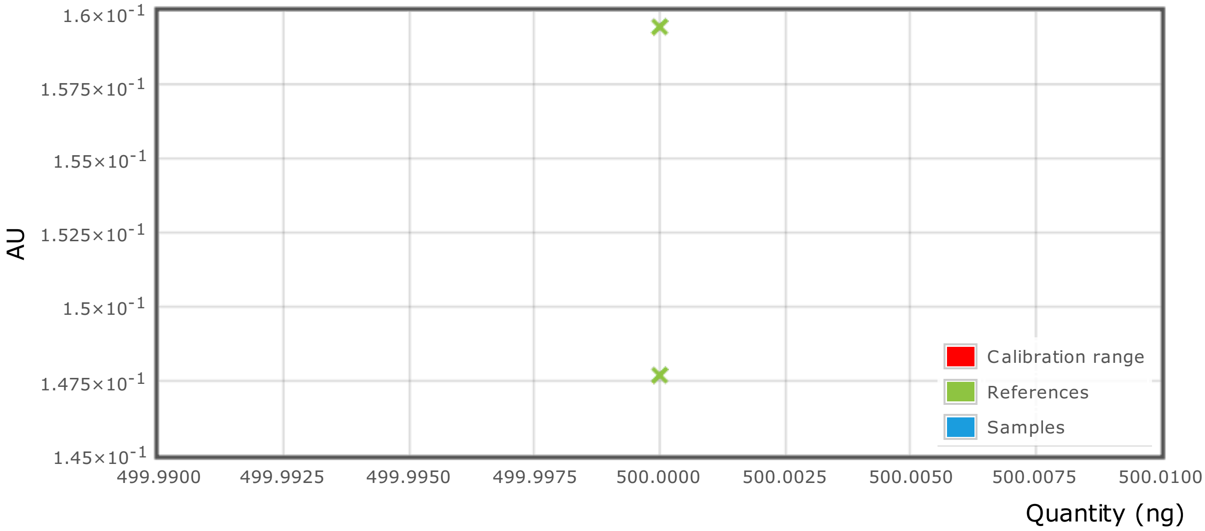

Regression mode	Linear-2
Range deviation	5.00 %
Related substances	Default
Number of references	2
Calibration function	$y=0x$
Coefficient of variation	CV 0.00 %
Correlation coefficient	n/a
⚠	Unable to compute the results for this substance because there wasn't enough groups of references replicas (at least 1 for Linear-1, 2 for Linear2 and Mime-1 and 3 for Polynomial and MiMe-2)

Height calibration for substance CBN @ RT White:

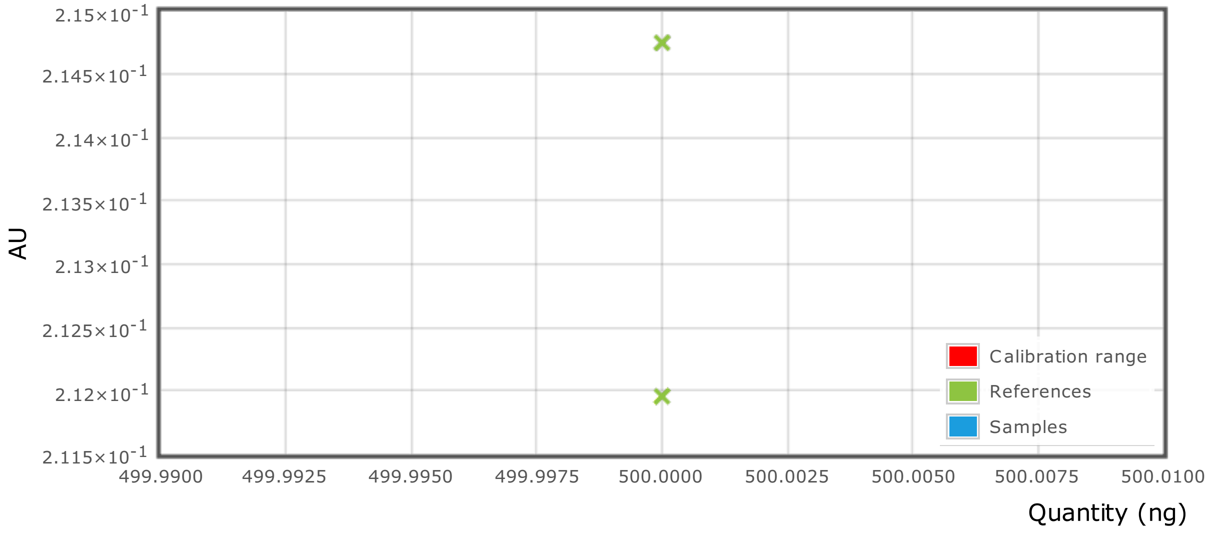

Regression mode	Linear-2
Range deviation	5.00 %
Related substances	Default
Number of references	2
Calibration function	$y=0x$
Coefficient of variation	CV 0.00 %
Correlation coefficient	n/a
⚠	Unable to compute the results for this substance because there wasn't enough groups of references replicas (at least 1 for Linear-1, 2 for Linear2 and Mime-1 and 3 for Polynomial and MiMe-2)

Results:

Substance having no available results		
⚠	9-THC	Unable to compute the results for this substance because there wasn't enough groups of references replicas (at least 1 for Linear-1, 2 for Linear2 and Mime-1 and 3 for Polynomial and MiMe-2)
⚠	CBD	Unable to compute the results for this substance because there wasn't enough groups of references replicas (at least 1 for Linear-1, 2 for Linear2 and Mime-1 and 3 for Polynomial and MiMe-2)
⚠	CBN	There wasn't any sample application available in the assignments for this substance. Please check that the peaks were correctly detected and assigned for this substance.

A track marked with ⚠ means: this result is outside the regression range given by the reference assignments, but is included in the results because it is in the allowed range deviation.

Analyst:	Reviewer:
