# Supplementary material for: High Performance Thin-Layer Chromatography (HPTLC) data of Cannabinoids in ten mobile phase systems
Source: Data Brief. 2020 Jun 30;31:105955. doi: 10.1016/j.dib.2020.105955 (PMC7352075; doi:10.1016/j.dib.2020.105955)
Supplement: Supplementary file 1 [file mmc1.zip › S4-Case sample reports/XHDa-sample run-6.pdf]

Analysis: XHDa-sample run-6

Path: Home/YL Research

Based on method: Samples (no cal)

Created	12-Oct-2019 18:48:31	visionCATSuser
Modified	12-Oct-2019 20:27:42	visionCATSuser
Last HPTLC log	12-Oct-2019 20:27:42	Analysis modified
Explorer notes		

Track	Vial ID	Description	Volume	Position	Type
1	MeOH blank	MeOH Blank	2.0 µl	A1	Sample
2	250ug/mL mix	250ug/mL	2.0 µl	A2	Reference
3	Tetracosane	Tetracosane IS	2.0 µl	A3	Sample
4	s1	[redacted]	2.0 µl	B1	Sample
5	s2	[redacted]	2.0 µl	B2	Sample
6	s3	[redacted]	2.0 µl	B3	Sample
7	s4	[redacted]	2.0 µl	B4	Sample
8	s5	[redacted]	2.0 µl	B5	Sample
9	s6	[redacted]	2.0 µl	B6	Sample
10	s7	[redacted]	2.0 µl	B7	Sample
11	s8	[redacted]	2.0 µl	B8	Sample
12	s9	[redacted]	2.0 µl	B9	Sample
13	s10	[redacted]	2.0 µl	B10	Sample
14	250ug/mL mix	250ug/mL	2.0 µl	A2	Reference
15	MeOH blank	MeOH Blank	2.0 µl	A1	Sample
Sequence table notes					

A track marked with ⚠ means: the application type is overridden in some evaluation(s).

System setup:

Software	Server User-PC, version 2.5.18072.1
ATS4	S/N:080713
Chamber	N/A
Derivatization dip	N/A
Scanner3	S/N:031025
Visualizer	S/N:230515

Chromatography

Plate layout:

Stationary phase	Merck, HPTLC plates silica gel 60 F 254
Plate format	200.0 x 100.0 mm
Application type	Band
Application	Position Y: 8.0 mm, length: 8.0 mm, width: 0.0 mm
Track	First position X: 20.0 mm, distance: 11.4 mm
Solvent front position	70.0 mm
Notes	

Take image clean plate 1a - Visualizer (S/N: 230515):

Quality	Enhanced
RT White	auto capture, Auto, level 85 %, Band
R 254	auto capture, Auto, level 85 %, Band
Instrument diagnostics	Valid diagnostics
Documentation step label	
Notes	

Application 1 - ATS 4 (S/N: 080713):

Spray gas	NI
Sample solvent type	Methanol
Filling speed	15 µl/s
Predosage volume	200 nl
Retraction volume	200 nl
Dosage speed	150 nl/s
Filling quality	User
Rinsing cycles / vacuum	2 / 4 s
Filling cycles / vacuum	1 / 4 s
Rinsing solvent name	Methanol
Nozzle temperature	Unheated
Rack in use	Standard
Instrument diagnostics	Valid diagnostics
Notes	

Development 1 - Chamber:

Tank	TTC 20x10
Mobile phase	Xylene:hexane:diethylamine (25:10:1)
Saturation time	20 min
Use saturation pad	true
Use smartALERT	false
Volume front through	10 ml
Volume rear through	25 ml
Drying time	5 min
Drying temperature	Room temperature
Notes	

Take image developed plate 1a - Visualizer (S/N: 230515):

Quality	Enhanced
RT White	auto capture, Auto, level 85 %, Band
R 254	auto capture, Auto, level 85 %, Band
R 366	auto capture, Auto, level 85 %, Band
Instrument diagnostics	Valid diagnostics
Documentation step label	
Notes	

Scan developed plate 1b - Scanner 3 (S/N: 031025):

Scanner type	Single λ
Optimization for	Resolution
Measurement mode	Absorption
Filter	n/a
Detector mode	Automatic
Scanning speed	20 mm/s
Data resolution	100 µm/step
Slit	5 x 0.2 mm, micro
Partial scan	No
Lamp	Deuterium & Tungsten
Wavelength(s)	254 nm
Instrument diagnostics	Valid diagnostics
Documentation step label	
Notes	

Derivatization 1 - dip:

Reagent name	
Dipping speed	5
Dipping time	0 s
Reagent preparation	
Heating	100 °C for 3 min, heated after
Notes	

Take image derivatized plate 1a - Visualizer (S/N: 230515):

Quality	Enhanced
RT White	auto capture, Auto, level 85 %, Band
R 366	auto capture, Auto, level 85 %, Band
Instrument diagnostics	Valid diagnostics
Documentation step label	
Notes	

System suitability tests:

SST settings:

SST tracks	

Data acquisition

Application 1 - ATS 4 (S/N: 080713):

Executed	12-Oct-2019 18:53:02 visionCATSuser

Development 1 - Chamber:

Executed	12-Oct-2019 19:19:21 visionCATSuser

Take image developed plate 1a - Visualizer (S/N: 230515):

Executed	12-Oct-2019 20:12:24 visionCATSuser

RT White

Developed, RemTransVis

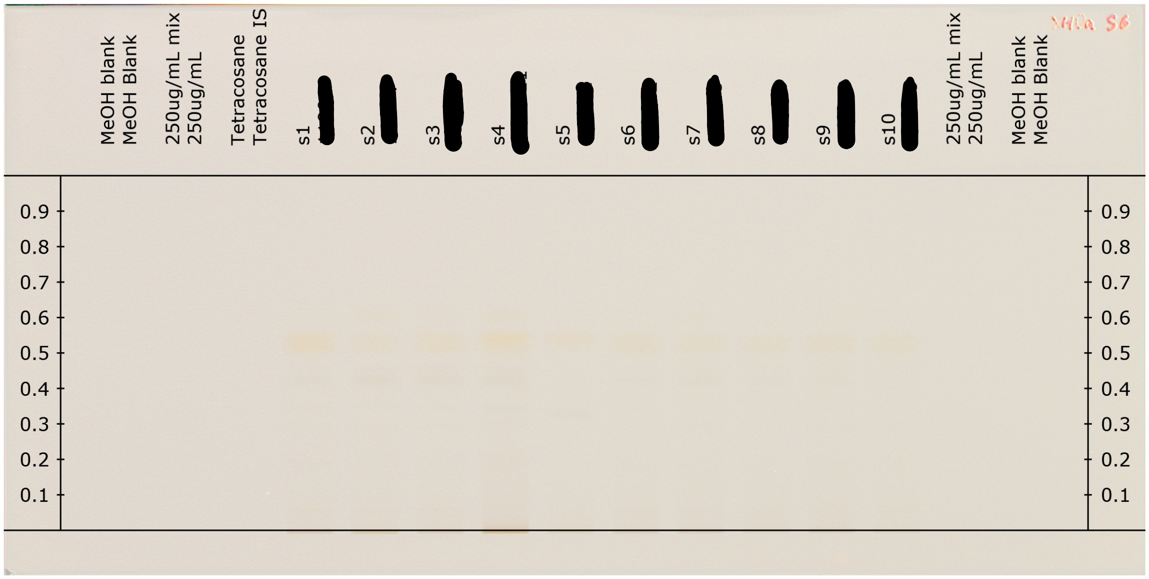

Exposure	0.085 s
Contrast	1
Normalized exposure	Disabled
Clarify	Disabled
White balance	1.00, 1.00, 1.00

R 254

Developed, Remission254

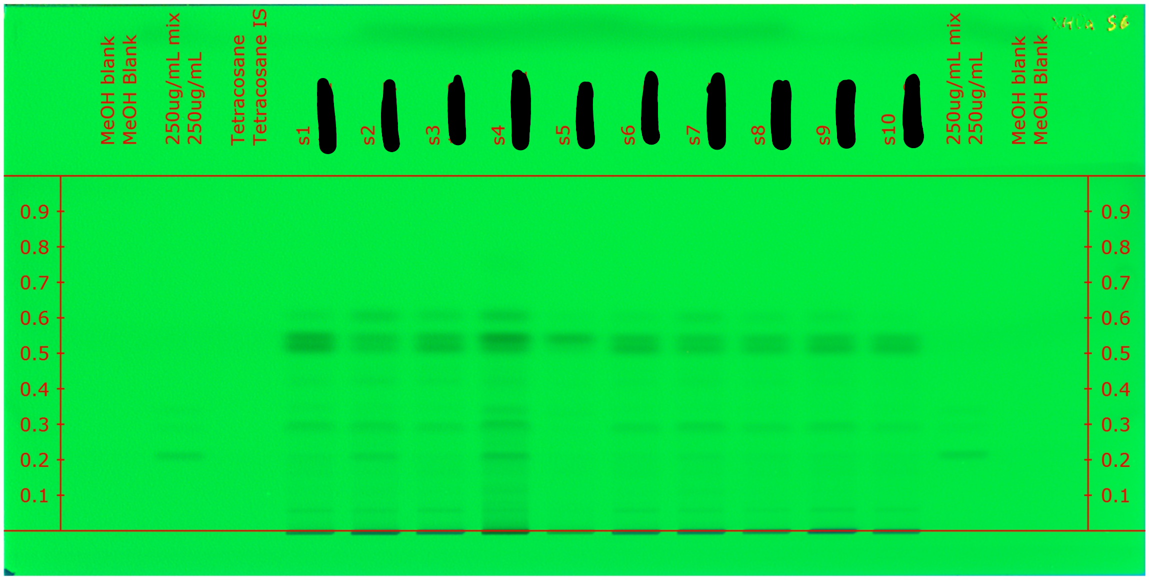

Exposure	0.272 s
Contrast	1
Normalized exposure	Disabled
Clarify	Disabled
White balance	1.00, 1.00, 1.00

CAMAG®
World Leader in Planar Chromatography

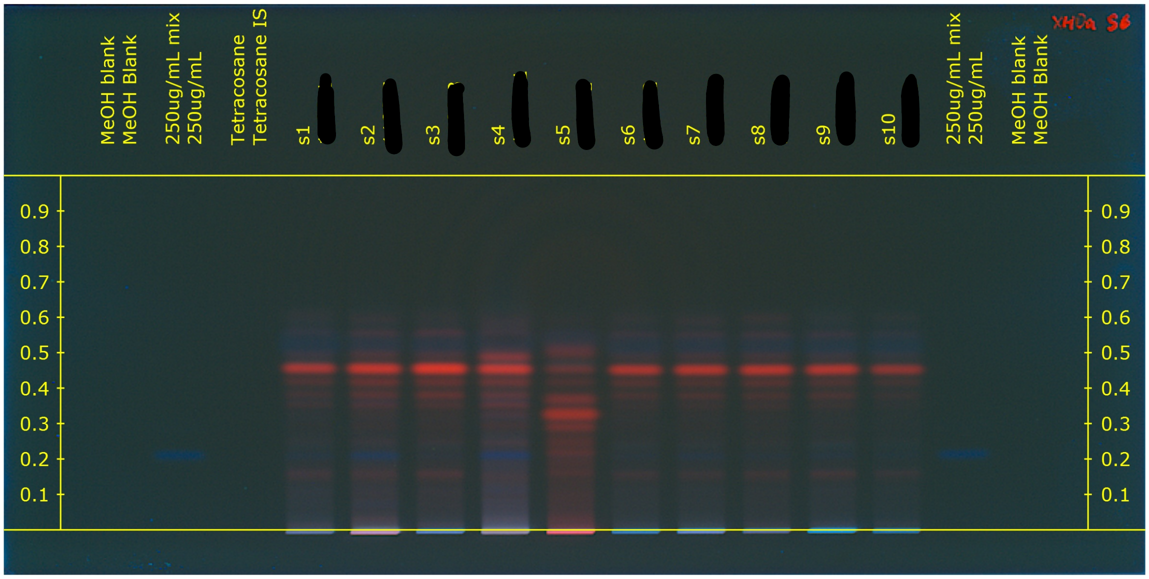

Exposure	3.613 s
Contrast	1
Normalized exposure	Disabled
Clarify	Disabled
White balance	1.00, 1.00, 1.00

Scan developed plate 1b - Scanner 3 (S/N: 031025):

Executed	12-Oct-2019 20:15:17 visionCATSuser

Scan:

Wavelength	254 nm

Track 1:

Type	Single λ

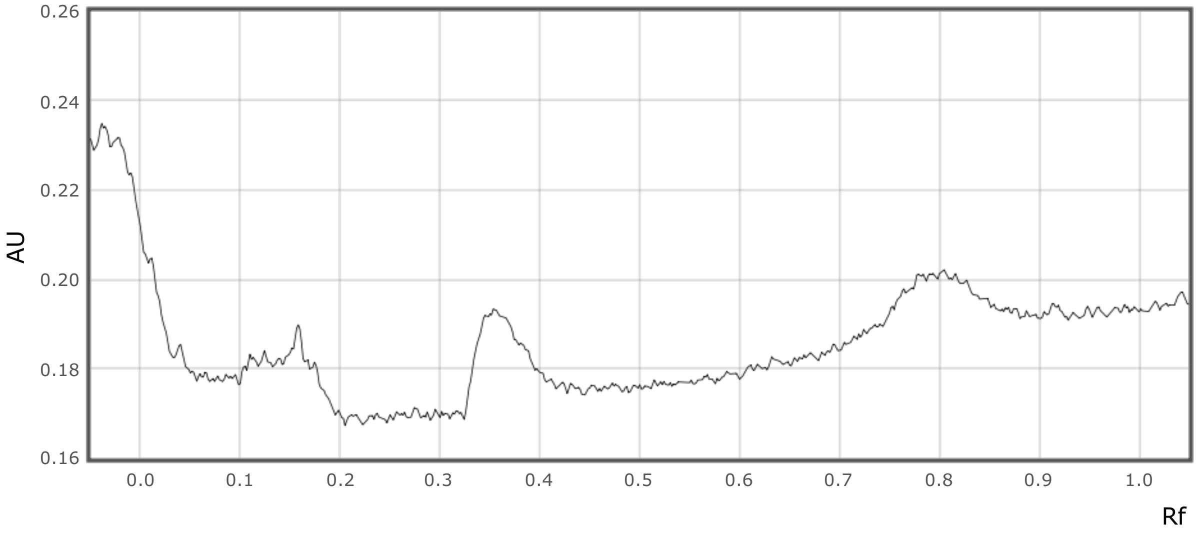

Track 2:

Type	Single λ

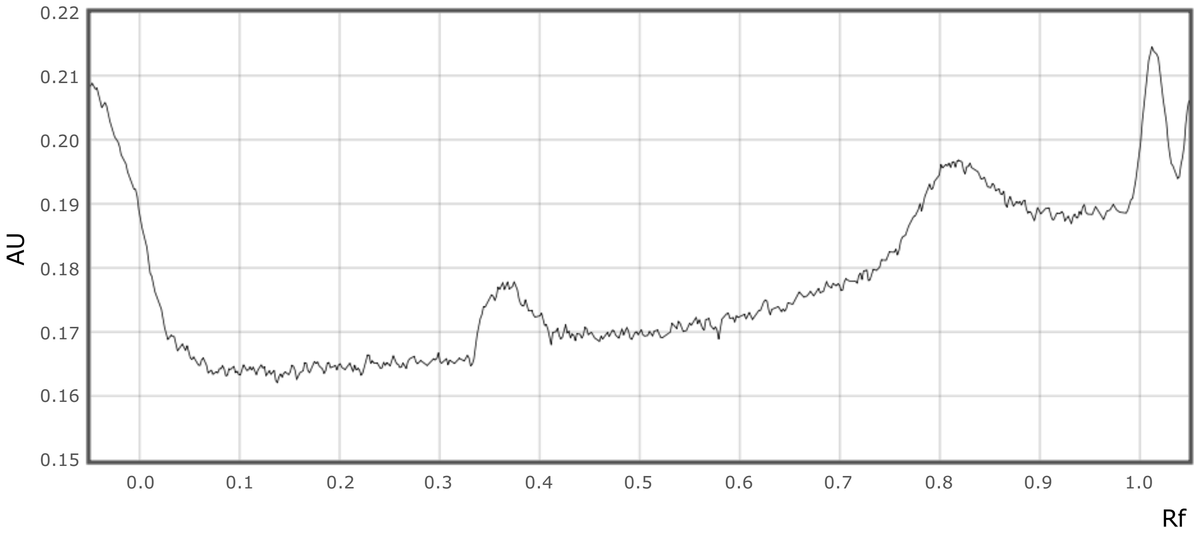

Track 3:

Type	Single λ

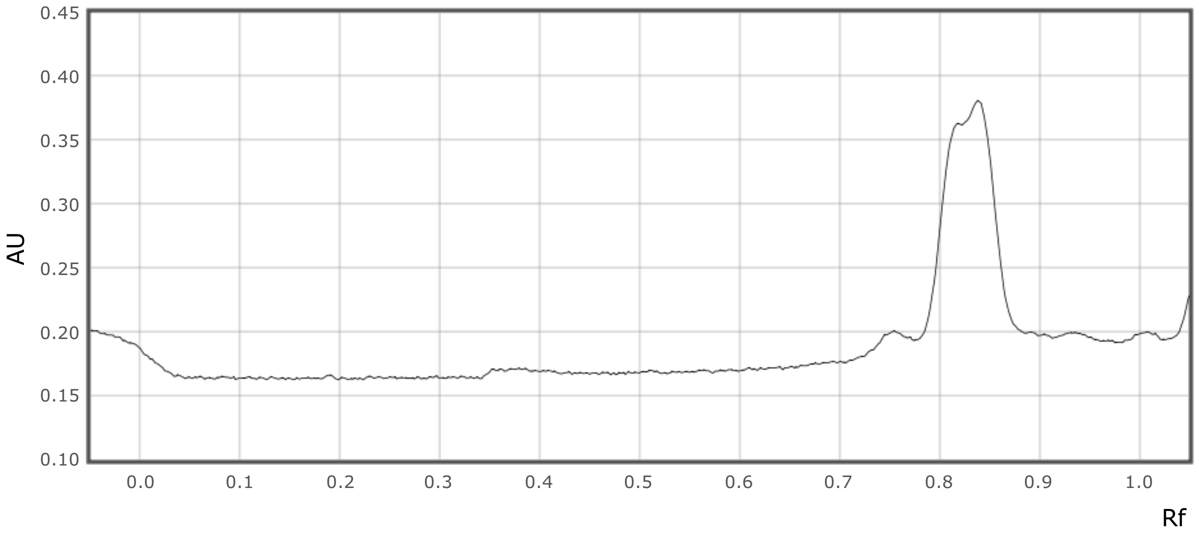

Track 4:

Type	Single λ

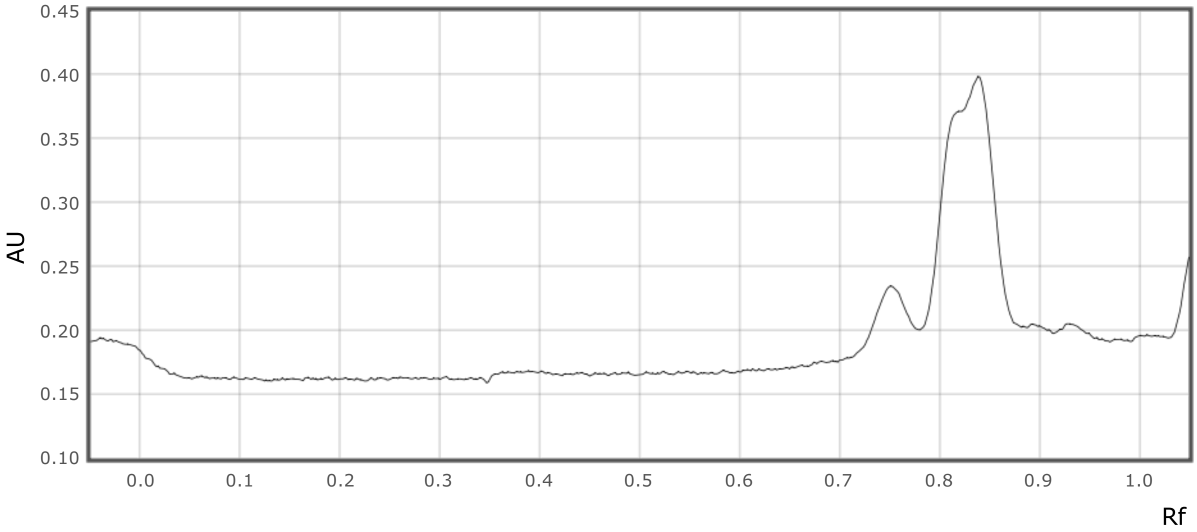

Track 5:	
Type	Single λ

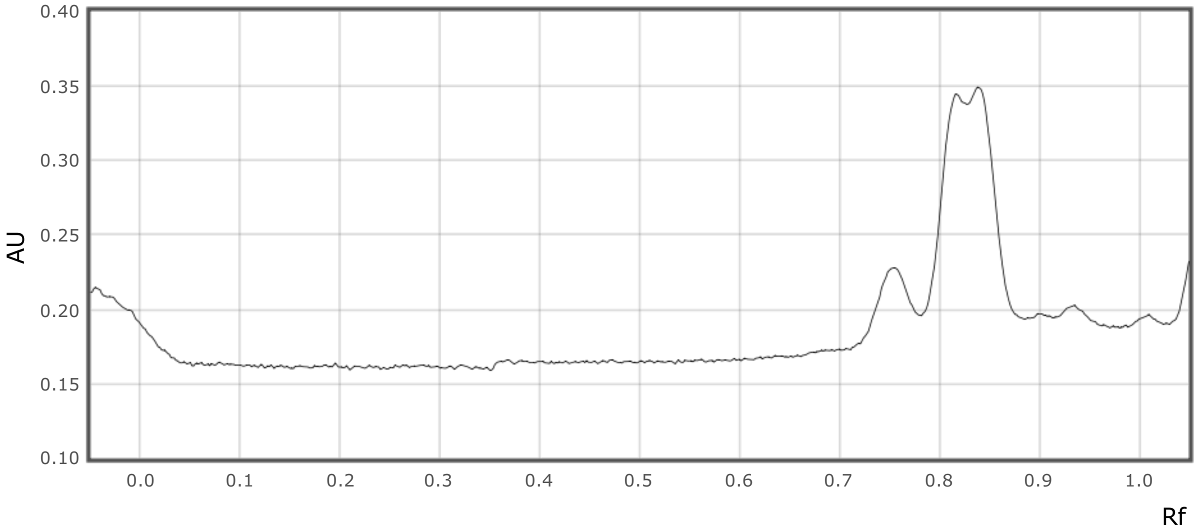

Track 6:	
Type	Single λ

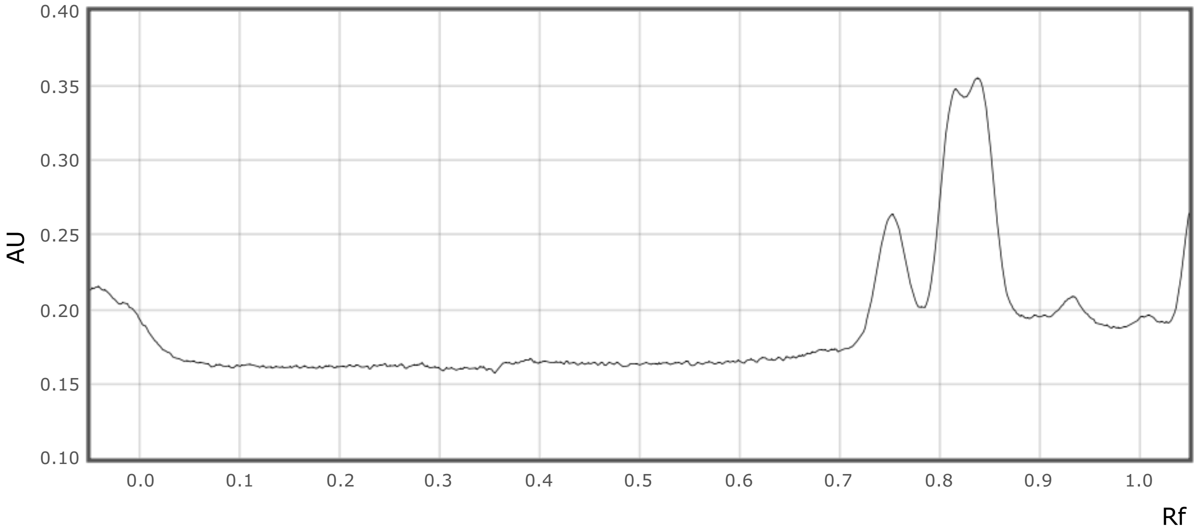

Track 7:

Type	Single λ

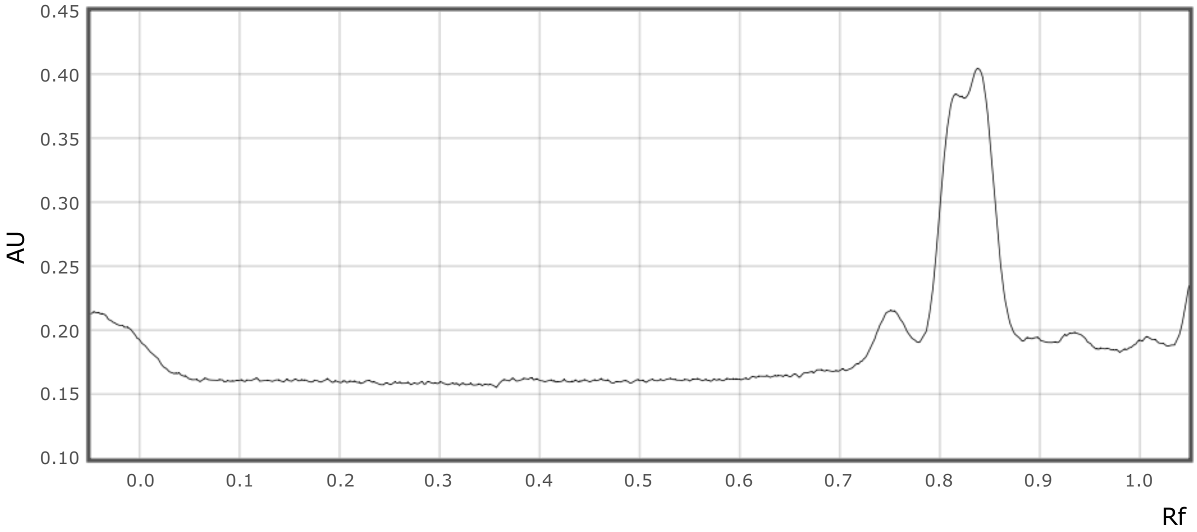

Track 8:

Type	Single λ

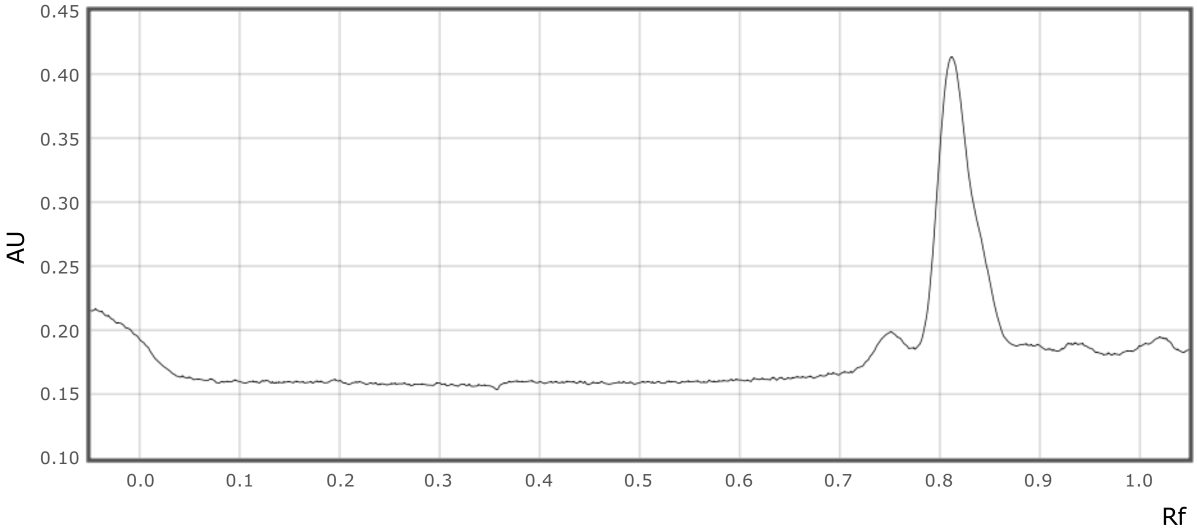

Track 9:	
Type	Single λ

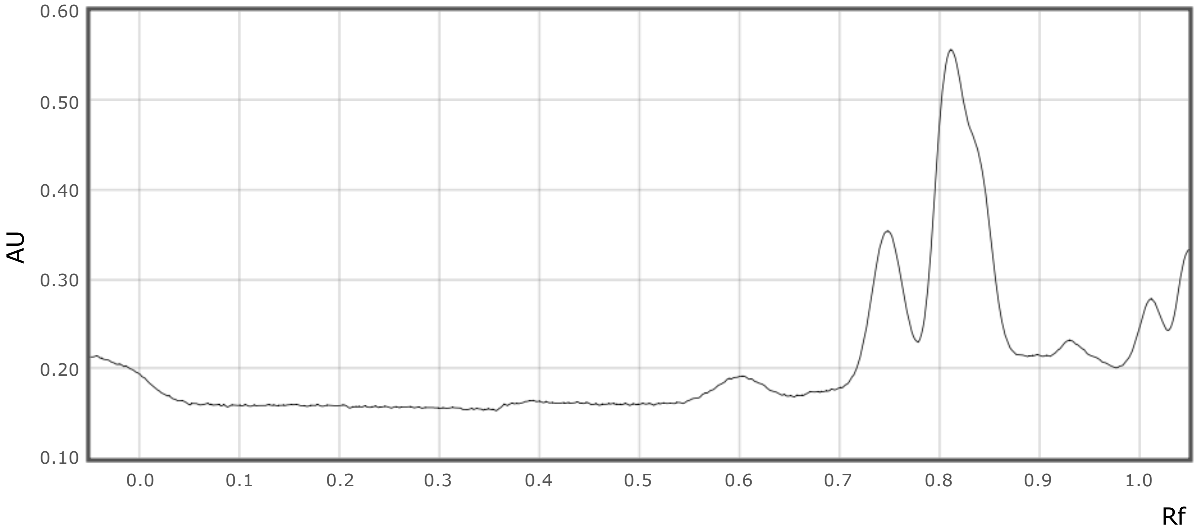

Track 10:	
Type	Single λ

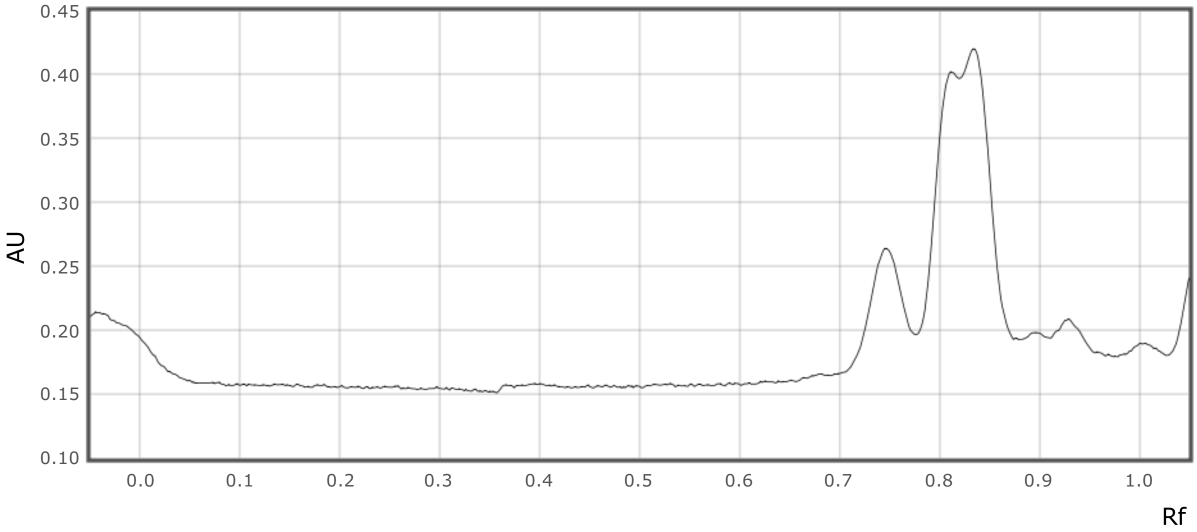

Track 11:

Type	Single λ

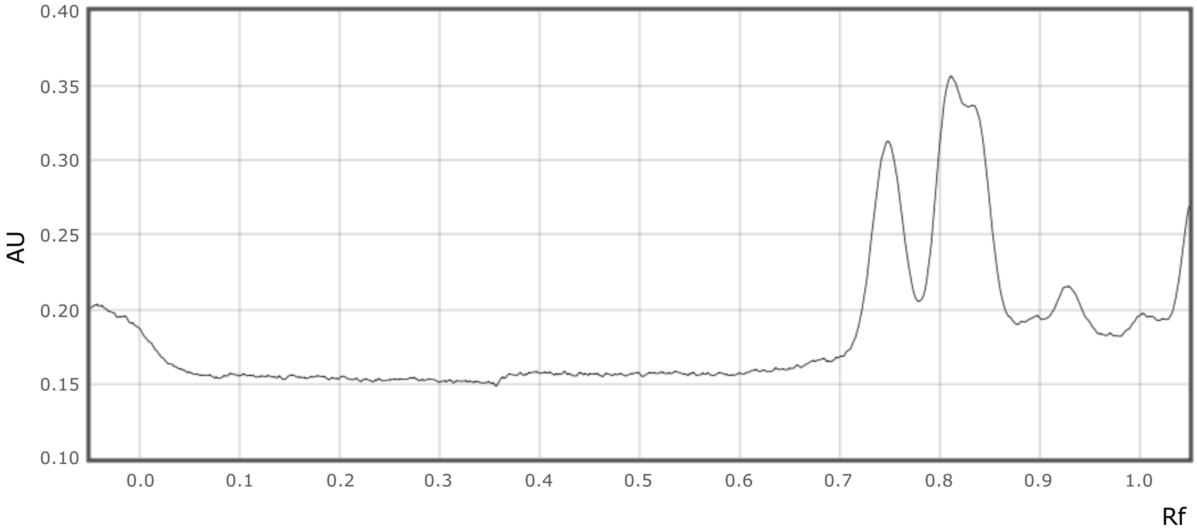

Track 12:

Type	Single λ

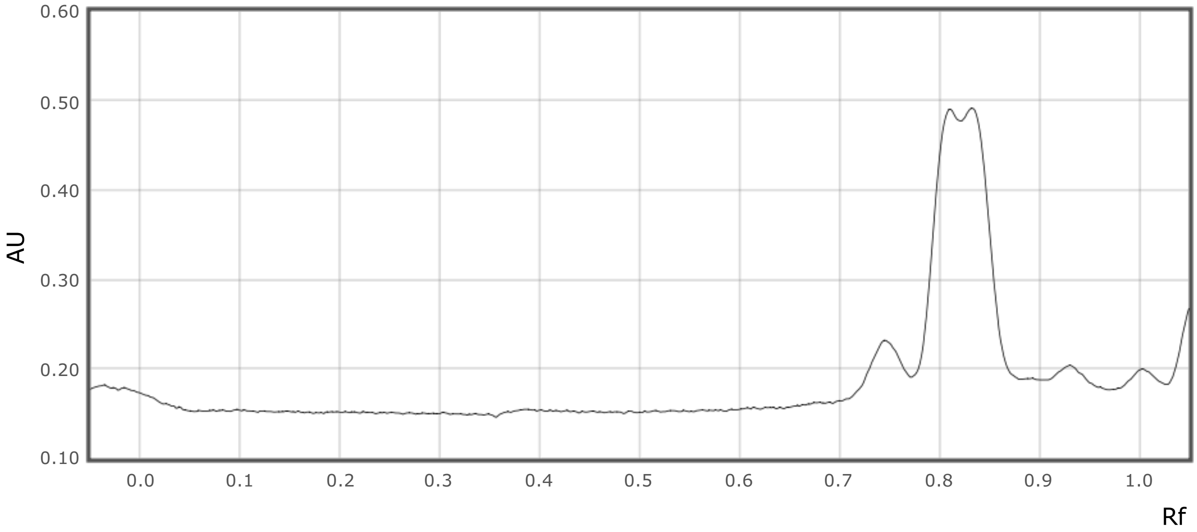

Track 13:	
Type	Single λ

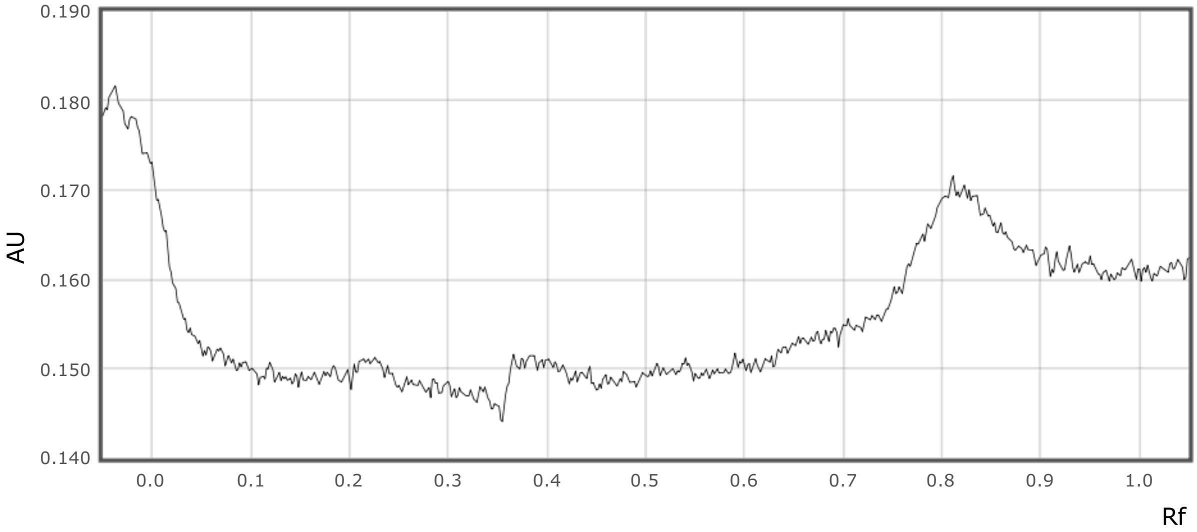

Track 14:	
Type	Single λ

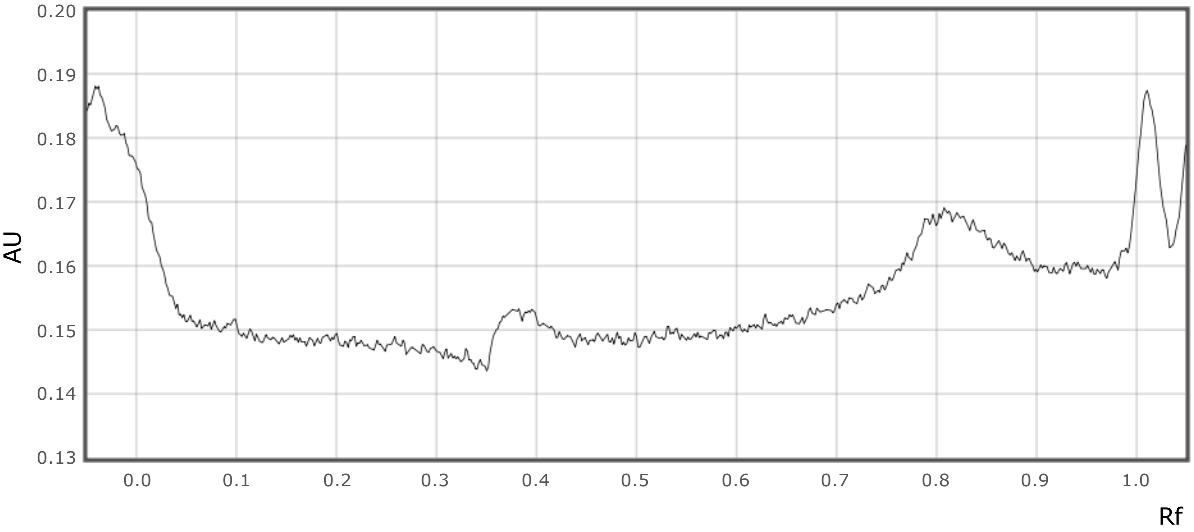

Track 15:

Type	Single λ

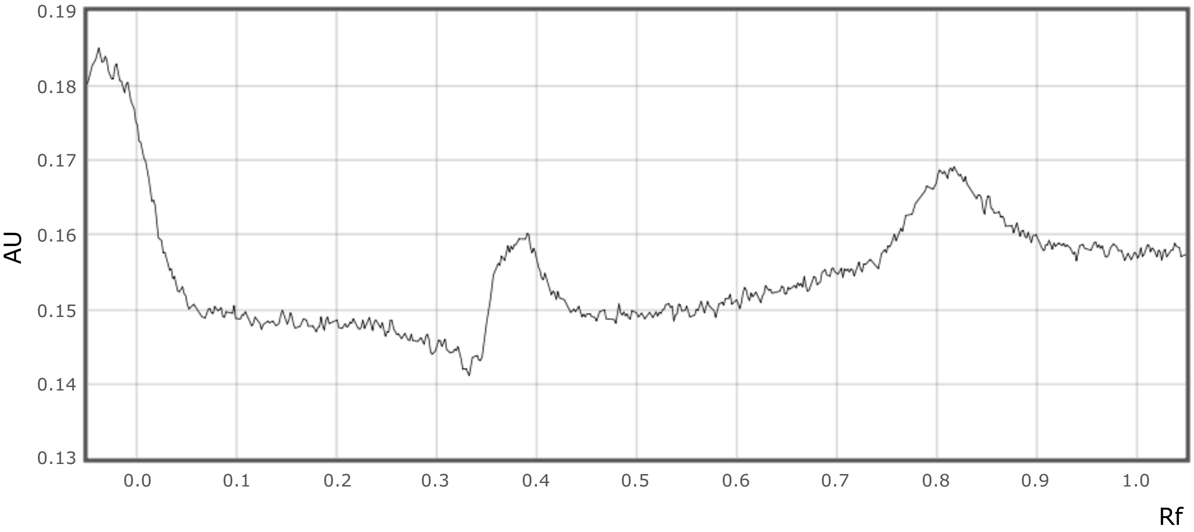

Derivatization 1 - dip:

Executed	12-Oct-2019 20:19:11 visionCATSuser

Take image derivatized plate 1a - Visualizer (S/N: 230515):

Executed	12-Oct-2019 20:24:24 visionCATSuser

RT White

Derivatized, RemTransVis

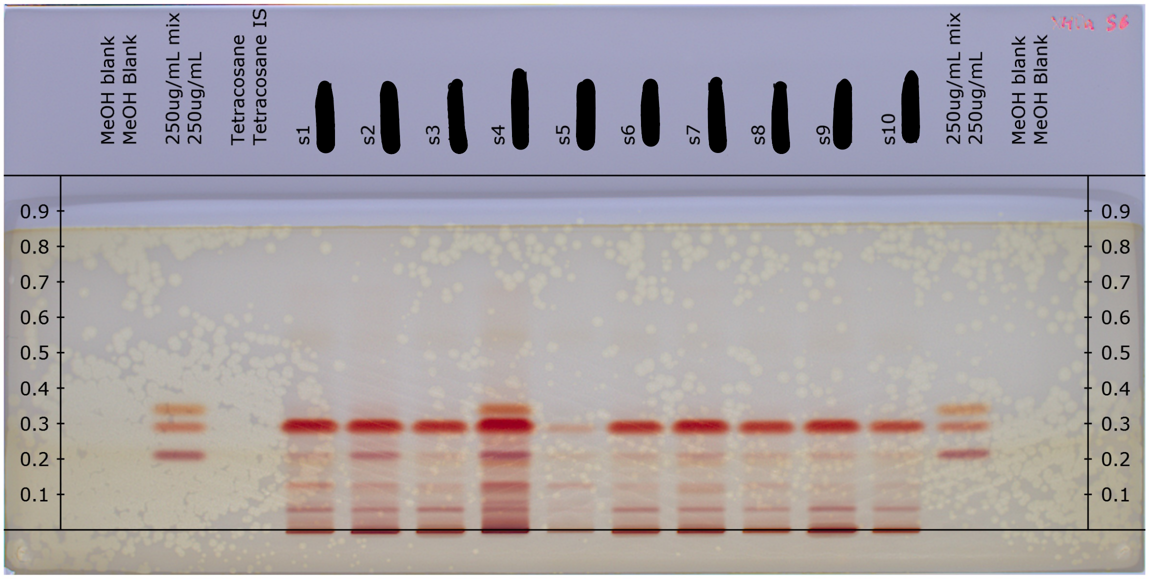

Exposure	0.041 s
Contrast	1
Normalized exposure	Disabled
Clarify	Disabled
White balance	1.18, 1.12, 0.79

R 366

Derivatized, Remission366

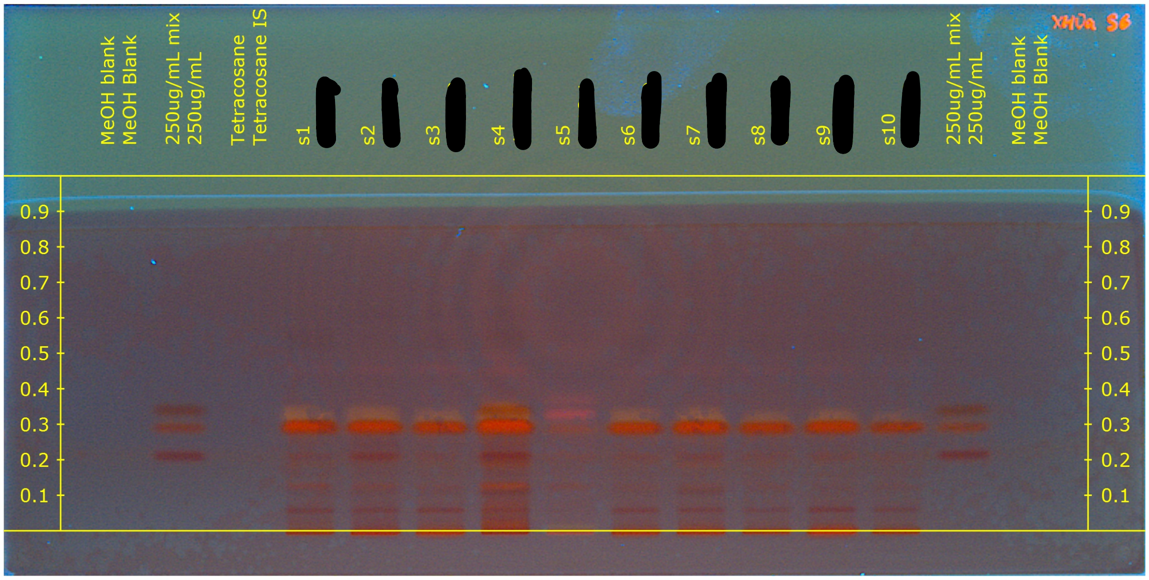

Exposure	9.999 s
Contrast	1
Normalized exposure	Disabled
Clarify	Disabled
White balance	1.00, 1.00, 1.00

Evaluation 1 :

Validated	false
Step	Take image derivatized plate 1a
Concentration unit type	Mass / volume
Notes	

Definition:

References:

250ug/mL mix

Substance Name	Concentration	Purity
9-THC	250.000 µg/ml	100.00 %
CBD	250.000 µg/ml	100.00 %
CBN	250.000 µg/ml	100.00 %

Samples:

Vial ID	Amount	Volume solution	Reference amount	Related to
MeOH blank		0.00 ml		
Tetracosane		0.00 ml		
s1		0.00 ml		
s2		0.00 ml		
s3		0.00 ml		
s4		0.00 ml		
s5		0.00 ml		
s6		0.00 ml		
s7		0.00 ml		
s8		0.00 ml		
s9		0.00 ml		
s10		0.00 ml		

Integration parameters:

Bounds	[0.000,1.000]
Smoothing	Savitzky–Golay of order 3 and window 7
Baseline correction	Lowest slope with noise 0.05
Profile subtraction	Profile subtraction from track 1
Peaks detection	Gauss (legacy) with sensitivity 0.1, separation 1 and threshold 0.1

Scan:

Wavelength	RT White

Track 1:

Type	Sample
Vial ID	MeOH blank
Description	MeOH Blank
Volume	2.0 µl

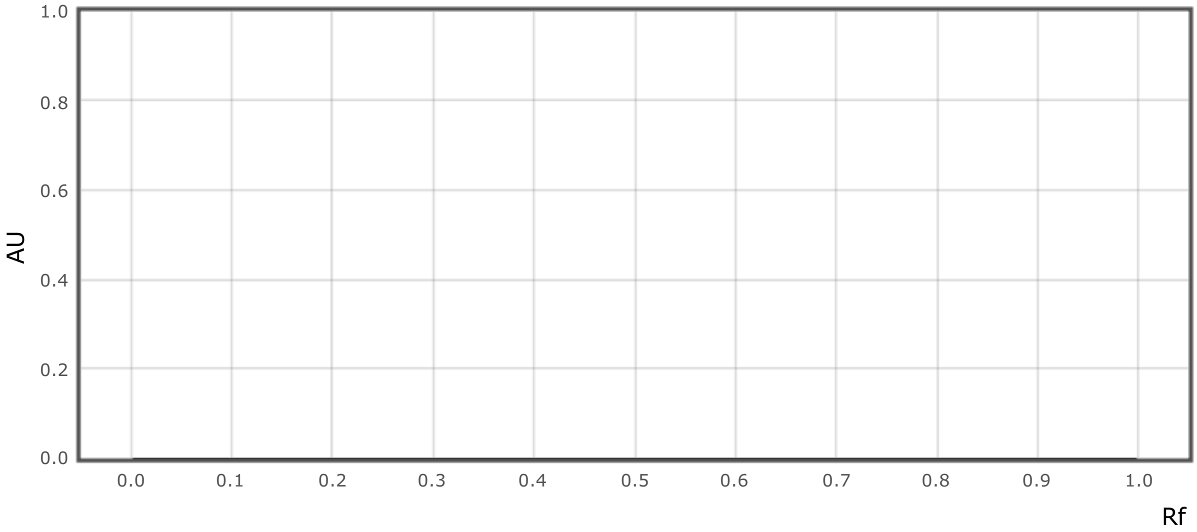

Peak #	Start		Max			End		Area		Manual peak	Substance Name
	Rf	H	Rf	H	%	Rf	H	A	%		

Track 2:	
Type	Reference
Vial ID	250ug/mL mix
Description	250ug/mL
Volume	2.0 µl

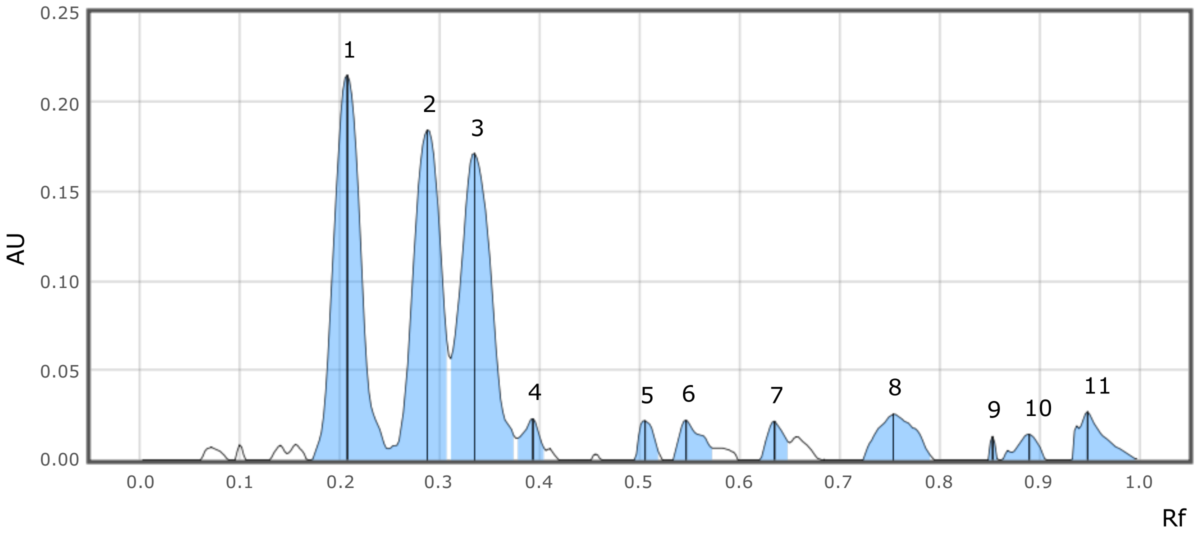

Peak	Start		Max			End		Area		Manual	Substance
#	Rf	H	Rf	H	%	Rf	H	A	%	peak	Name
1	0.170	0.0000	0.207	0.2150	29.08	0.248	0.0062	0.00666	29.33	No	CBN
2	0.248	0.0062	0.287	0.1844	24.94	0.309	0.0583	0.00583	25.65	No	9-THC
3	0.311	0.0566	0.335	0.1712	23.17	0.376	0.0123	0.00611	26.91	No	CBD
4	0.378	0.0121	0.393	0.0230	3.11	0.406	0.0053	0.00044	1.93	No	
5	0.495	0.0000	0.505	0.0219	2.96	0.523	0.0000	0.00037	1.61	No	
6	0.531	0.0000	0.546	0.0222	3.00	0.575	0.0064	0.00056	2.46	No	
7	0.620	0.0000	0.635	0.0217	2.93	0.650	0.0095	0.00042	1.83	No	
8	0.724	0.0000	0.754	0.0255	3.45	0.795	0.0000	0.00111	4.90	No	
9	0.849	0.0000	0.853	0.0132	1.78	0.860	0.0000	0.00007	0.32	No	
10	0.862	0.0000	0.890	0.0142	1.93	0.907	0.0000	0.00034	1.51	No	
11	0.931	0.0000	0.948	0.0269	3.64	0.998	0.0006	0.00081	3.55	No	

Track 3:

Type	Sample
Vial ID	Tetracosane
Description	Tetracosane IS
Volume	2.0 µl

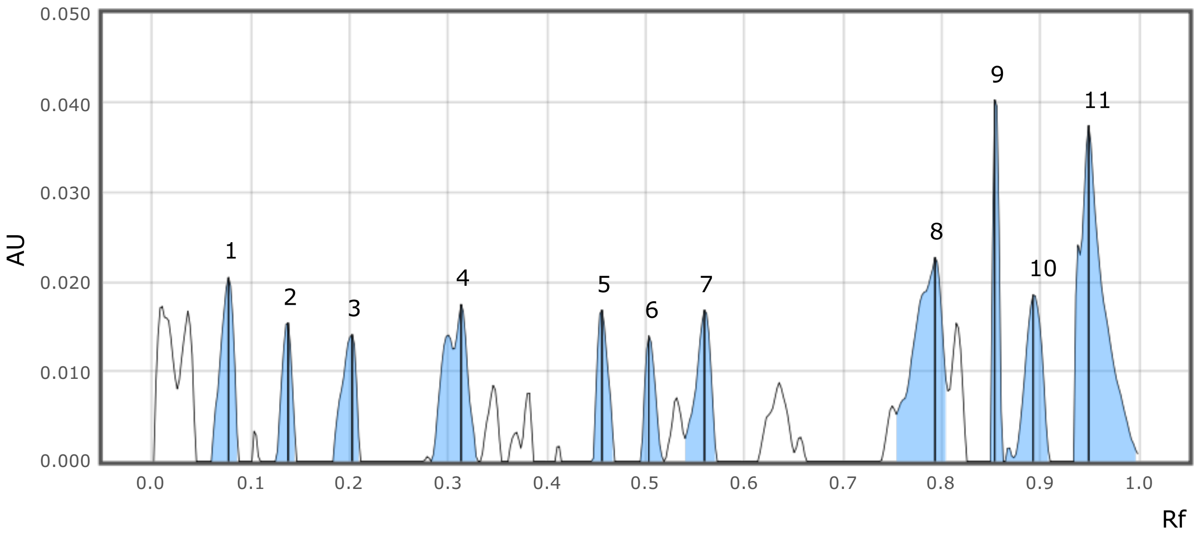

Peak	Start		Max			End		Area		Manual	Substance
#	Rf	H	Rf	H	%	Rf	H	A	%	peak	Name
1	0.060	0.0000	0.078	0.0205	8.75	0.088	0.0000	0.00032	7.39	No	
2	0.125	0.0000	0.138	0.0154	6.58	0.147	0.0000	0.00018	4.18	No	
3	0.183	0.0000	0.203	0.0141	6.04	0.211	0.0000	0.00024	5.45	No	
4	0.283	0.0000	0.313	0.0175	7.47	0.330	0.0000	0.00046	10.53	No	
5	0.445	0.0000	0.456	0.0169	7.20	0.469	0.0000	0.00022	4.99	No	
6	0.495	0.0000	0.503	0.0140	5.97	0.518	0.0000	0.00017	3.84	No	
7	0.540	0.0026	0.559	0.0169	7.20	0.572	0.0000	0.00029	6.75	No	
8	0.754	0.0052	0.793	0.0227	9.70	0.806	0.0079	0.00074	17.23	No	
9	0.849	0.0000	0.853	0.0403	17.20	0.862	0.0000	0.00029	6.76	No	
10	0.862	0.0000	0.892	0.0185	7.91	0.910	0.0000	0.00037	8.46	No	
11	0.933	0.0000	0.948	0.0374	15.97	0.998	0.0008	0.00106	24.42	No	

Track 4:

Type	Sample
Vial ID	s1
Description	[redacted]
Volume	2.0 µl

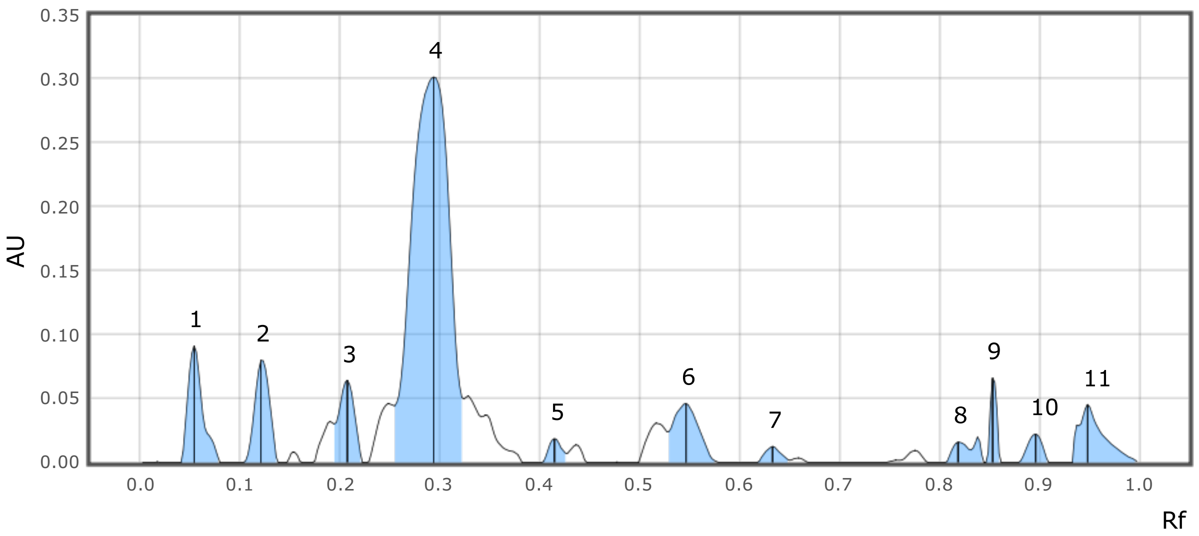

Peak #	Start		Max			End		Area		Manual peak	Substance Name
	Rf	H	Rf	H	%	Rf	H	A	%		
1	0.041	0.0000	0.054	0.0910	11.93	0.080	0.0000	0.00153	7.07	No	
2	0.103	0.0000	0.121	0.0797	10.46	0.138	0.0000	0.00135	6.27	No	
3	0.194	0.0299	0.207	0.0642	8.42	0.222	0.0000	0.00114	5.29	No	
4	0.255	0.0442	0.294	0.3014	39.52	0.324	0.0497	0.01316	60.98	No	9-THC
5	0.402	0.0000	0.415	0.0186	2.43	0.428	0.0070	0.00028	1.30	No	
6	0.529	0.0240	0.546	0.0460	6.04	0.579	0.0000	0.00132	6.14	No	
7	0.618	0.0000	0.633	0.0125	1.64	0.650	0.0017	0.00022	1.04	No	
8	0.806	0.0000	0.819	0.0158	2.07	0.845	0.0000	0.00044	2.03	No	
9	0.847	0.0000	0.853	0.0660	8.65	0.862	0.0000	0.00049	2.29	No	
10	0.879	0.0000	0.897	0.0220	2.89	0.910	0.0000	0.00037	1.71	No	
11	0.933	0.0000	0.948	0.0453	5.94	0.998	0.0009	0.00127	5.90	No	

Track 5:

Type	Sample
Vial ID	s2
Description	[redacted]
Volume	2.0 µl

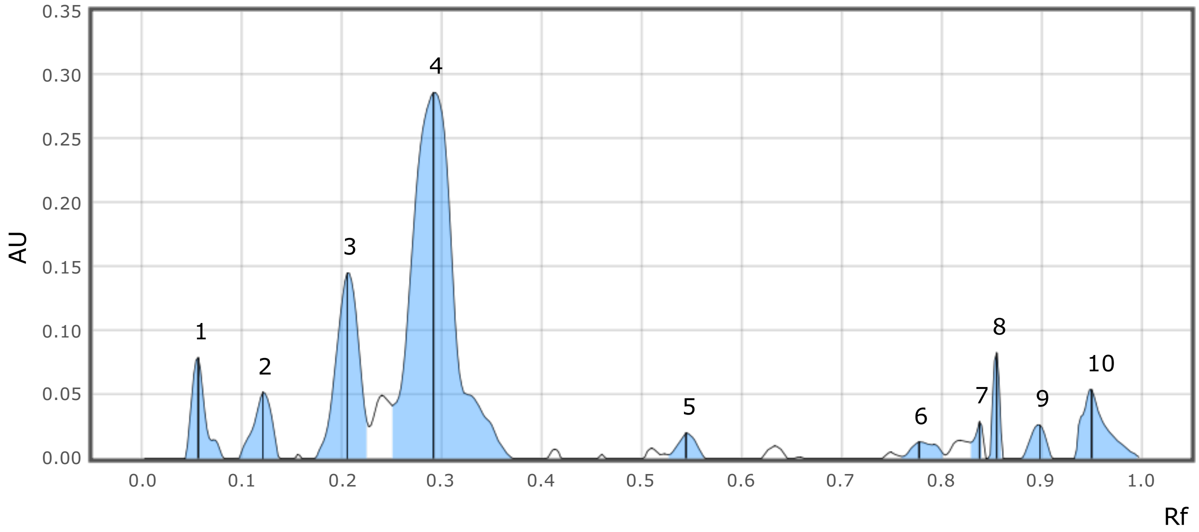

Peak #	Start Rf	Start H	Max Rf	Max H	Max %	End Rf	End H	Area A	Area %	Manual peak	Substance Name
1	0.043	0.0000	0.056	0.0788	10.03	0.082	0.0000	0.00123	5.25	No	
2	0.095	0.0000	0.121	0.0519	6.60	0.138	0.0000	0.00107	4.55	No	
3	0.173	0.0000	0.205	0.1449	18.43	0.227	0.0247	0.00381	16.22	No	
4	0.250	0.0411	0.291	0.2862	36.42	0.371	0.0000	0.01378	58.66	No	9-THC
5	0.527	0.0028	0.544	0.0199	2.54	0.564	0.0000	0.00041	1.72	No	
6	0.760	0.0015	0.778	0.0129	1.64	0.804	0.0026	0.00037	1.59	No	
7	0.830	0.0124	0.838	0.0288	3.66	0.845	0.0000	0.00027	1.15	No	
8	0.847	0.0000	0.856	0.0827	10.52	0.862	0.0000	0.00062	2.66	No	
9	0.879	0.0000	0.899	0.0262	3.33	0.912	0.0000	0.00045	1.92	No	
10	0.933	0.0000	0.951	0.0537	6.84	0.998	0.0011	0.00147	6.27	No	

Track 6:

Type	Sample
Vial ID	s3
Description	[redacted]
Volume	2.0 µl

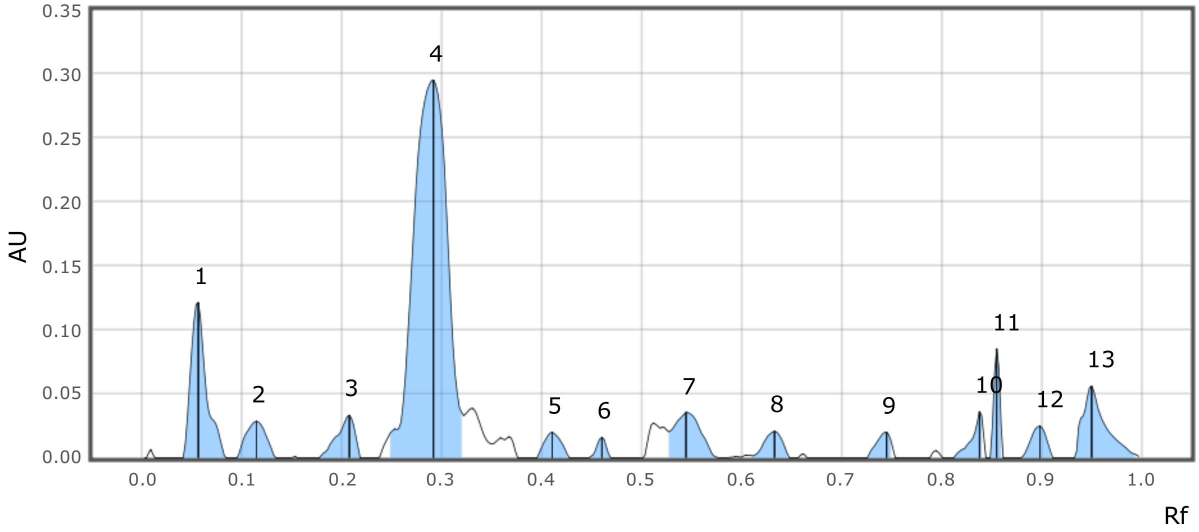

Peak #	Start		Max			End		Area		Manual peak	Substance Name
	Rf	H	Rf	H	%	Rf	H	A	%		
1	0.041	0.0000	0.056	0.1215	15.30	0.084	0.0000	0.00217	10.71	No	
2	0.095	0.0000	0.114	0.0286	3.60	0.134	0.0000	0.00066	3.25	No	
3	0.177	0.0000	0.207	0.0334	4.20	0.220	0.0000	0.00067	3.31	No	
4	0.248	0.0196	0.291	0.2954	37.19	0.322	0.0330	0.01147	56.48	No	9-THC
5	0.395	0.0000	0.410	0.0201	2.53	0.428	0.0000	0.00038	1.89	No	
6	0.447	0.0000	0.460	0.0159	2.00	0.471	0.0000	0.00017	0.83	No	
7	0.527	0.0203	0.544	0.0357	4.49	0.577	0.0000	0.00108	5.32	No	
8	0.611	0.0015	0.633	0.0208	2.62	0.648	0.0000	0.00043	2.12	No	
9	0.726	0.0000	0.745	0.0201	2.54	0.754	0.0000	0.00033	1.65	No	
10	0.812	0.0000	0.838	0.0363	4.58	0.845	0.0000	0.00045	2.22	No	
11	0.849	0.0000	0.856	0.0854	10.75	0.862	0.0000	0.00059	2.89	No	
12	0.879	0.0000	0.899	0.0249	3.13	0.912	0.0000	0.00044	2.16	No	
13	0.933	0.0000	0.951	0.0561	7.06	0.998	0.0010	0.00146	7.18	No	

Track 7:

Type	Sample
Vial ID	s4
Description	[redacted]
Volume	2.0 µl

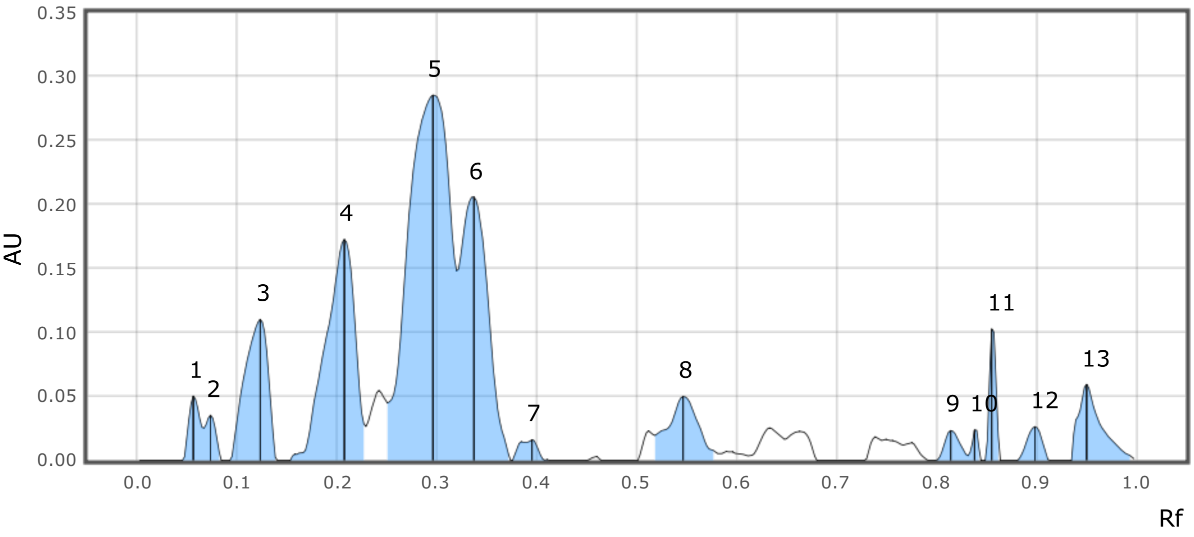

Peak #	Start		Max			End		Area		Manual peak	Substance Name
	Rf	H	Rf	H	%	Rf	H	A	%		
1	0.045	0.0000	0.056	0.0503	4.33	0.067	0.0250	0.00066	1.86	No	
2	0.067	0.0250	0.073	0.0354	3.04	0.084	0.0000	0.00041	1.16	No	
3	0.093	0.0000	0.123	0.1102	9.49	0.140	0.0000	0.00295	8.28	No	
4	0.153	0.0000	0.207	0.1727	14.87	0.229	0.0264	0.00581	16.31	No	
5	0.250	0.0448	0.296	0.2854	24.58	0.320	0.1479	0.01369	38.44	No	9-THC
6	0.320	0.1479	0.337	0.2058	17.72	0.374	0.0002	0.00664	18.64	No	CBD
7	0.376	0.0000	0.395	0.0162	1.39	0.412	0.0000	0.00033	0.93	No	
8	0.518	0.0194	0.546	0.0498	4.29	0.581	0.0053	0.00175	4.93	No	
9	0.799	0.0000	0.814	0.0233	2.01	0.832	0.0036	0.00040	1.13	No	
10	0.832	0.0036	0.838	0.0236	2.04	0.845	0.0000	0.00017	0.48	No	
11	0.849	0.0000	0.856	0.1027	8.84	0.864	0.0000	0.00078	2.20	No	
12	0.881	0.0000	0.899	0.0264	2.27	0.914	0.0000	0.00045	1.26	No	
13	0.935	0.0000	0.951	0.0595	5.12	0.998	0.0011	0.00157	4.39	No	

Track 8:

Type	Sample
Vial ID	s5
Description	[redacted]
Volume	2.0 µl

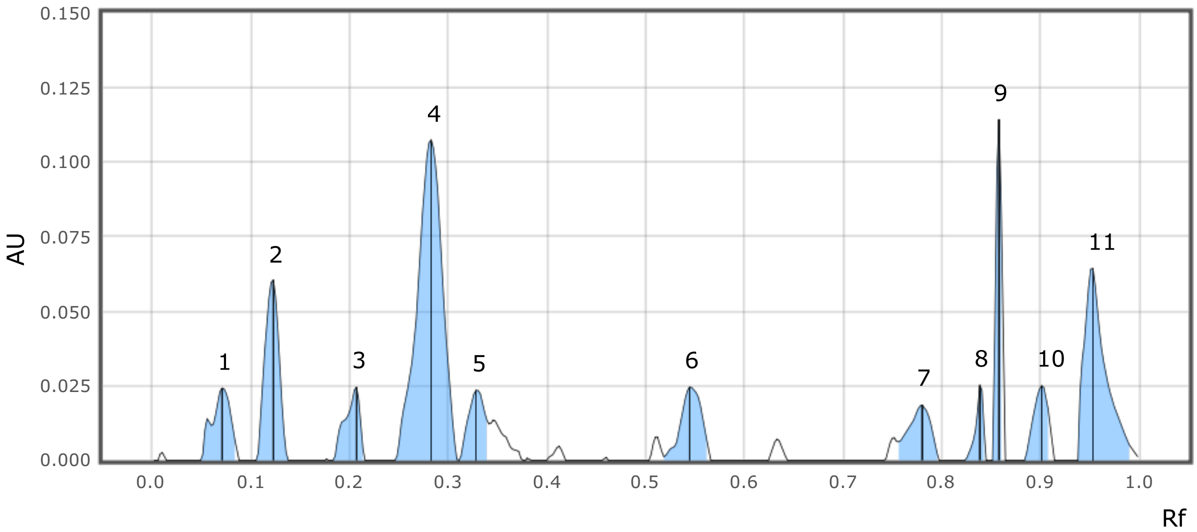

Peak	Start		Max			End		Area		Manual	Substance
#	Rf	H	Rf	H	%	Rf	H	A	%	peak	Name
1	0.049	0.0000	0.071	0.0242	4.73	0.088	0.0000	0.00053	5.50	No	
2	0.106	0.0000	0.123	0.0604	11.80	0.140	0.0000	0.00100	10.29	No	
3	0.183	0.0000	0.207	0.0246	4.80	0.216	0.0000	0.00042	4.31	No	
4	0.246	0.0000	0.283	0.1074	20.99	0.311	0.0000	0.00314	32.26	No	9-THC
5	0.311	0.0000	0.328	0.0234	4.58	0.341	0.0122	0.00046	4.72	No	CBD
6	0.518	0.0012	0.544	0.0246	4.80	0.566	0.0000	0.00060	6.22	No	
7	0.756	0.0062	0.780	0.0185	3.62	0.797	0.0000	0.00048	4.89	No	
8	0.823	0.0000	0.838	0.0252	4.92	0.845	0.0000	0.00022	2.22	No	
9	0.851	0.0000	0.858	0.1142	22.31	0.864	0.0000	0.00081	8.34	No	
10	0.884	0.0000	0.901	0.0251	4.90	0.914	0.0000	0.00044	4.50	No	
11	0.938	0.0000	0.953	0.0642	12.54	0.998	0.0012	0.00163	16.75	No	

Track 9:

Type	Sample
Vial ID	s6
Description	[redacted]
Volume	2.0 µl

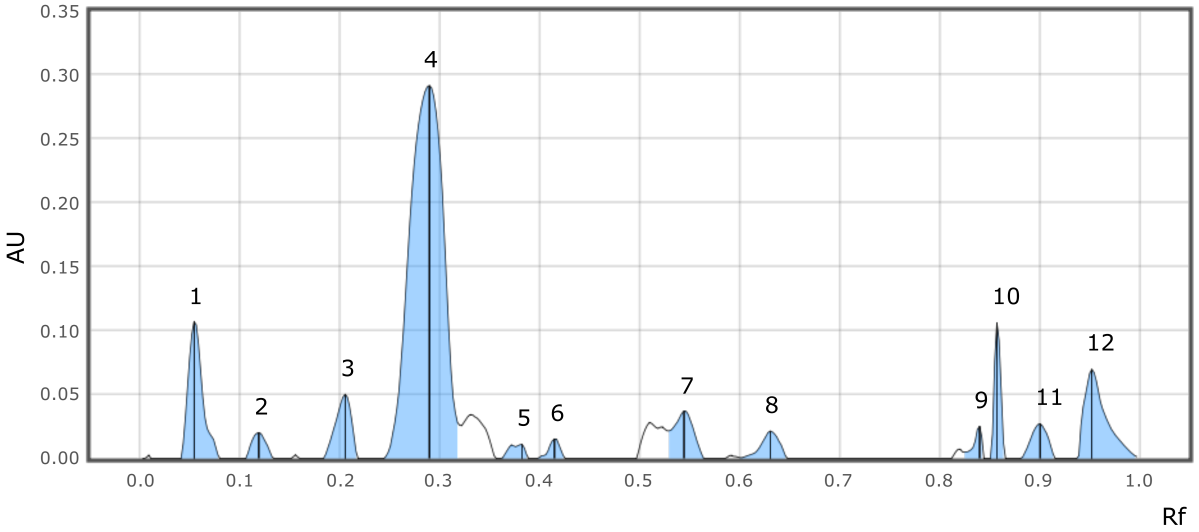

Peak #	Start		Max			End		Area		Manual peak	Substance Name
	Rf	H	Rf	H	%	Rf	H	A	%		
1	0.041	0.0000	0.054	0.1069	13.68	0.080	0.0000	0.00176	9.06	No	
2	0.106	0.0000	0.119	0.0200	2.55	0.134	0.0000	0.00033	1.69	No	
3	0.183	0.0000	0.205	0.0503	6.44	0.218	0.0000	0.00090	4.66	No	
4	0.244	0.0000	0.289	0.2916	37.34	0.320	0.0261	0.01148	59.21	No	9-THC
5	0.361	0.0000	0.382	0.0109	1.40	0.389	0.0000	0.00019	1.00	No	
6	0.397	0.0000	0.415	0.0152	1.95	0.425	0.0000	0.00019	0.98	No	
7	0.529	0.0216	0.544	0.0370	4.73	0.566	0.0000	0.00084	4.31	No	
8	0.600	0.0006	0.631	0.0214	2.74	0.648	0.0000	0.00044	2.29	No	
9	0.825	0.0049	0.840	0.0251	3.21	0.845	0.0000	0.00023	1.18	No	
10	0.851	0.0000	0.858	0.1059	13.56	0.866	0.0000	0.00078	4.03	No	
11	0.881	0.0000	0.901	0.0271	3.48	0.916	0.0000	0.00051	2.64	No	
12	0.938	0.0000	0.953	0.0697	8.92	0.998	0.0013	0.00173	8.94	No	

Track 10:	
Type	Sample
Vial ID	s7
Description	[redacted]
Volume	2.0 µl

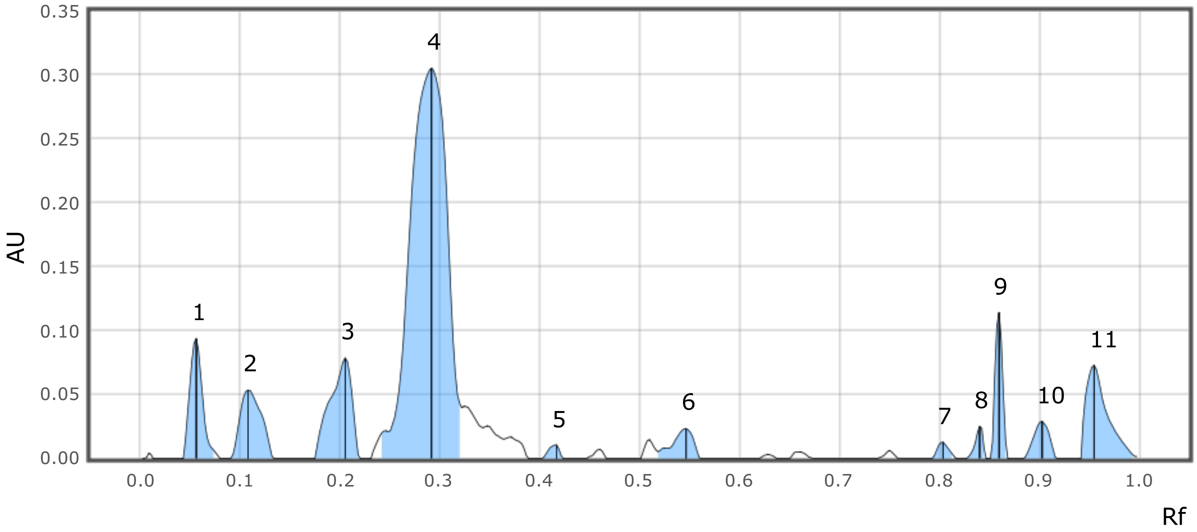

Peak #	Start		Max			End		Area		Manual peak	Substance Name
	Rf	H	Rf	H	%	Rf	H	A	%		
1	0.043	0.0000	0.056	0.0937	11.45	0.080	0.0000	0.00137	6.23	No	
2	0.090	0.0000	0.108	0.0533	6.52	0.134	0.0000	0.00131	5.95	No	
3	0.175	0.0000	0.205	0.0786	9.61	0.220	0.0000	0.00196	8.86	No	
4	0.242	0.0201	0.291	0.3054	37.32	0.322	0.0397	0.01308	59.23	No	9-THC
5	0.402	0.0000	0.417	0.0105	1.29	0.423	0.0000	0.00013	0.58	No	
6	0.518	0.0052	0.546	0.0233	2.85	0.562	0.0000	0.00055	2.51	No	
7	0.793	0.0000	0.804	0.0126	1.54	0.817	0.0000	0.00016	0.74	No	
8	0.827	0.0000	0.840	0.0249	3.05	0.847	0.0000	0.00022	0.99	No	
9	0.851	0.0000	0.860	0.1141	13.95	0.868	0.0000	0.00097	4.41	No	
10	0.884	0.0000	0.903	0.0288	3.52	0.918	0.0000	0.00052	2.34	No	
11	0.942	0.0000	0.955	0.0728	8.90	0.996	0.0015	0.00180	8.15	No	

Track 11:	
Type	Sample
Vial ID	s8
Description	[redacted]
Volume	2.0 µl

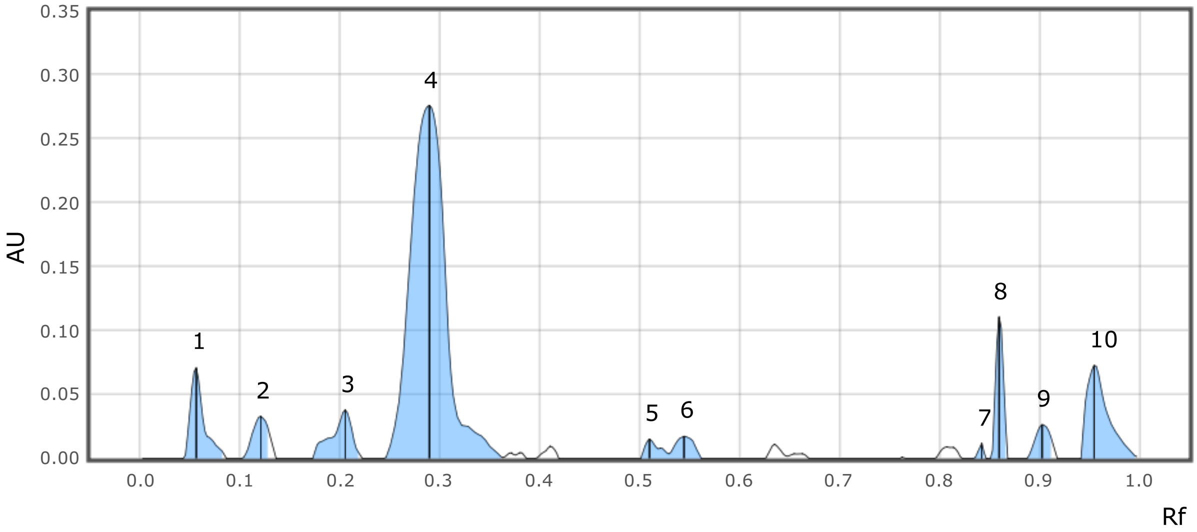

Peak #	Start Rf	Start H	Max Rf	Max H	Max %	End Rf	End H	Area A	Area %	Manual peak	Substance Name
1	0.043	0.0000	0.056	0.0708	10.54	0.086	0.0000	0.00110	6.16	No	
2	0.101	0.0000	0.121	0.0328	4.88	0.136	0.0000	0.00062	3.49	No	
3	0.173	0.0000	0.205	0.0379	5.65	0.222	0.0000	0.00088	4.92	No	
4	0.244	0.0000	0.289	0.2761	41.09	0.363	0.0007	0.01131	63.42	No	9-THC
5	0.501	0.0000	0.510	0.0151	2.25	0.529	0.0035	0.00024	1.36	No	
6	0.529	0.0035	0.544	0.0173	2.58	0.564	0.0000	0.00037	2.07	No	
7	0.834	0.0000	0.843	0.0119	1.76	0.847	0.0000	0.00006	0.36	No	
8	0.851	0.0000	0.860	0.1107	16.48	0.868	0.0000	0.00098	5.49	No	
9	0.888	0.0000	0.903	0.0265	3.95	0.918	0.0000	0.00046	2.60	No	
10	0.942	0.0000	0.955	0.0726	10.81	0.998	0.0014	0.00180	10.12	No	

Track 12:

Field	Value
Type	Sample
Vial ID	s9
Description	[redacted]
Volume	2.0 µl

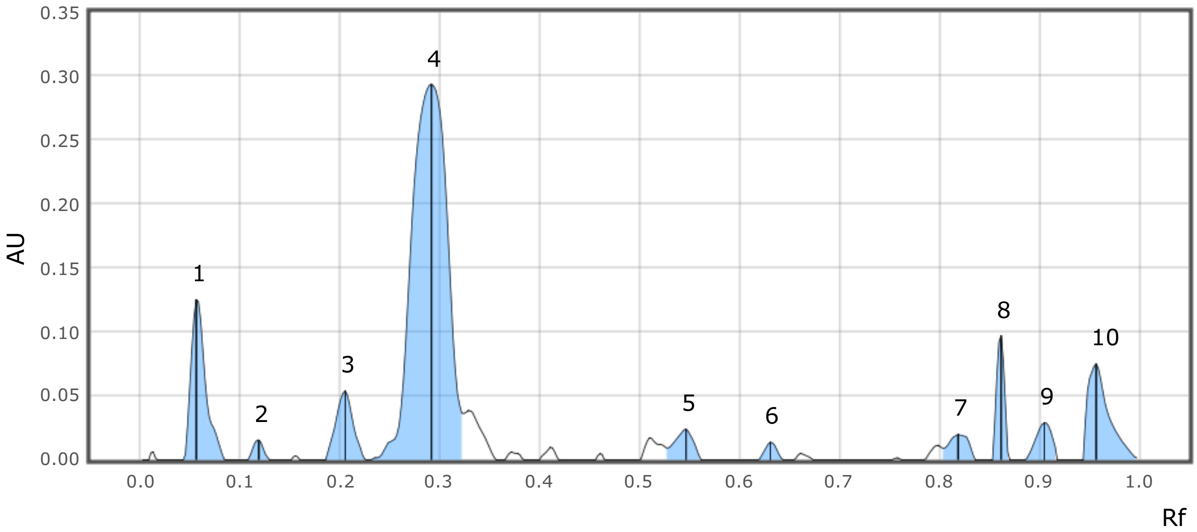

Peak #	Start Rf	Start H	Max Rf	Max H	Max %	End Rf	End H	Area A	Area %	Manual peak	Substance Name
1	0.043	0.0000	0.056	0.1253	16.78	0.086	0.0000	0.00211	10.62	No	
2	0.108	0.0000	0.119	0.0154	2.07	0.129	0.0000	0.00018	0.90	No	
3	0.186	0.0000	0.205	0.0539	7.22	0.227	0.0000	0.00105	5.30	No	
4	0.231	0.0000	0.291	0.2936	39.33	0.324	0.0362	0.01214	61.09	No	9-THC
5	0.527	0.0092	0.546	0.0240	3.21	0.562	0.0000	0.00051	2.59	No	
6	0.618	0.0000	0.631	0.0137	1.84	0.644	0.0000	0.00018	0.91	No	
7	0.804	0.0092	0.819	0.0198	2.65	0.836	0.0000	0.00046	2.31	No	
8	0.853	0.0000	0.862	0.0970	12.99	0.871	0.0000	0.00088	4.44	No	
9	0.886	0.0000	0.905	0.0289	3.87	0.918	0.0000	0.00053	2.69	No	
10	0.944	0.0000	0.957	0.0749	10.04	0.998	0.0015	0.00182	9.14	No	

Track 13:

Type	Sample
Vial ID	s10
Description	[redacted]
Volume	2.0 µl

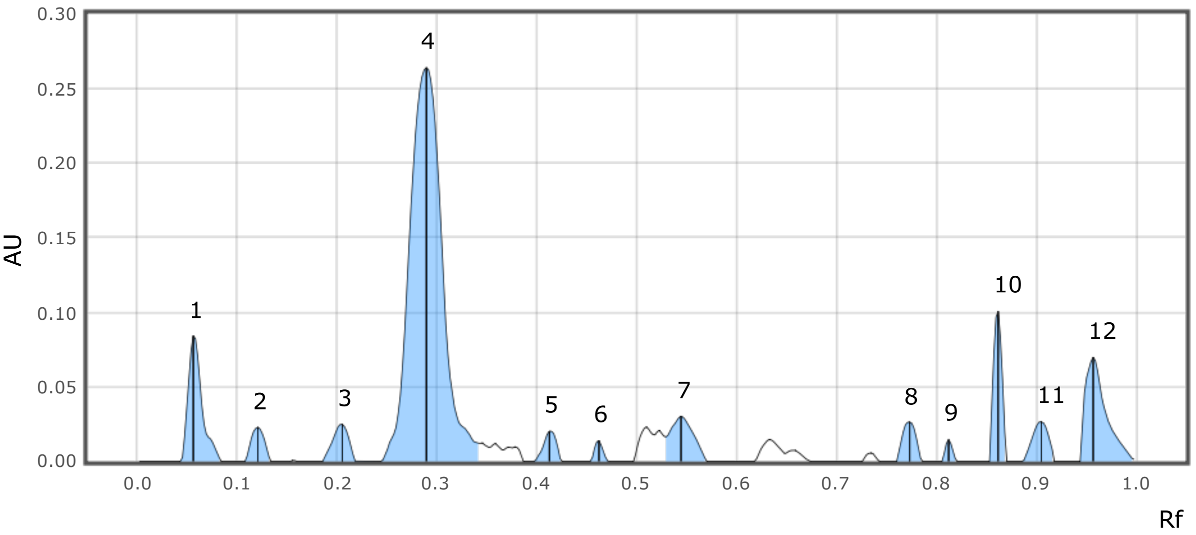

Peak #	Start Rf	Start H	Max Rf	Max H	Max %	End Rf	End H	Area A	Area %	Manual peak	Substance Name
1	0.043	0.0000	0.056	0.0841	12.06	0.084	0.0000	0.00133	7.85	No	
2	0.108	0.0000	0.121	0.0228	3.27	0.134	0.0000	0.00035	2.04	No	
3	0.186	0.0000	0.205	0.0248	3.56	0.220	0.0000	0.00046	2.69	No	
4	0.244	0.0000	0.289	0.2637	37.85	0.343	0.0121	0.00992	58.53	No	9-THC
5	0.397	0.0000	0.412	0.0201	2.88	0.425	0.0000	0.00029	1.74	No	
6	0.454	0.0000	0.462	0.0138	1.99	0.473	0.0000	0.00013	0.75	No	
7	0.529	0.0163	0.544	0.0302	4.33	0.570	0.0000	0.00079	4.67	No	
8	0.760	0.0000	0.773	0.0263	3.77	0.786	0.0000	0.00041	2.42	No	
9	0.806	0.0000	0.812	0.0145	2.08	0.821	0.0000	0.00012	0.68	No	
10	0.853	0.0000	0.862	0.1005	14.42	0.871	0.0000	0.00099	5.86	No	
11	0.886	0.0000	0.905	0.0264	3.78	0.918	0.0000	0.00049	2.92	No	
12	0.944	0.0000	0.957	0.0696	10.00	0.998	0.0013	0.00167	9.85	No	

Track 14:

Type	Reference
Vial ID	250ug/mL mix
Description	250ug/mL
Volume	2.0 µl

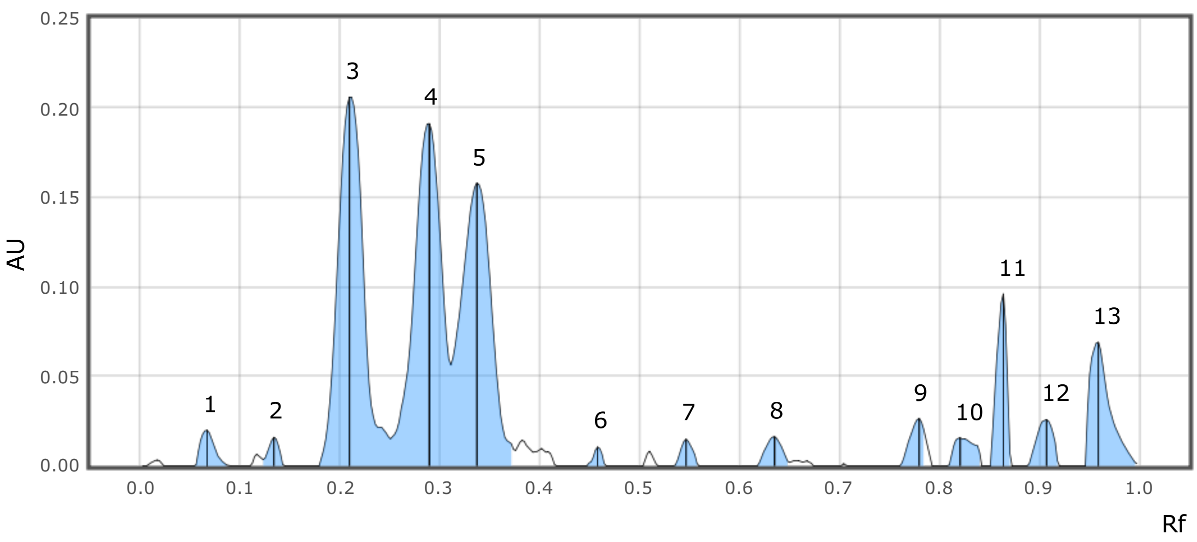

Peak	Start		Max			End		Area		Manual	Substance
#	Rf	H	Rf	H	%	Rf	H	A	%	peak	Name
1	0.054	0.0000	0.067	0.0198	2.29	0.090	0.0000	0.00032	1.39	No	
2	0.123	0.0034	0.134	0.0160	1.84	0.144	0.0000	0.00019	0.84	No	
3	0.179	0.0000	0.209	0.2057	23.78	0.250	0.0150	0.00611	26.68	No	CBN
4	0.250	0.0150	0.289	0.1911	22.09	0.311	0.0563	0.00613	26.75	No	9-THC
5	0.311	0.0563	0.337	0.1578	18.25	0.374	0.0092	0.00551	24.05	No	CBD
6	0.445	0.0000	0.458	0.0105	1.22	0.467	0.0000	0.00010	0.43	No	
7	0.533	0.0000	0.546	0.0149	1.72	0.559	0.0000	0.00020	0.85	No	
8	0.618	0.0000	0.635	0.0162	1.88	0.650	0.0020	0.00031	1.33	No	
9	0.760	0.0000	0.780	0.0264	3.05	0.793	0.0000	0.00047	2.05	No	
10	0.808	0.0000	0.821	0.0156	1.80	0.843	0.0000	0.00038	1.66	No	
11	0.851	0.0000	0.864	0.0961	11.11	0.873	0.0000	0.00110	4.80	No	
12	0.888	0.0000	0.907	0.0258	2.99	0.920	0.0000	0.00049	2.13	No	
13	0.946	0.0000	0.959	0.0690	7.98	0.998	0.0013	0.00161	7.03	No	

Track 15:

Type	Sample
Vial ID	MeOH blank
Description	MeOH Blank
Volume	2.0 µl

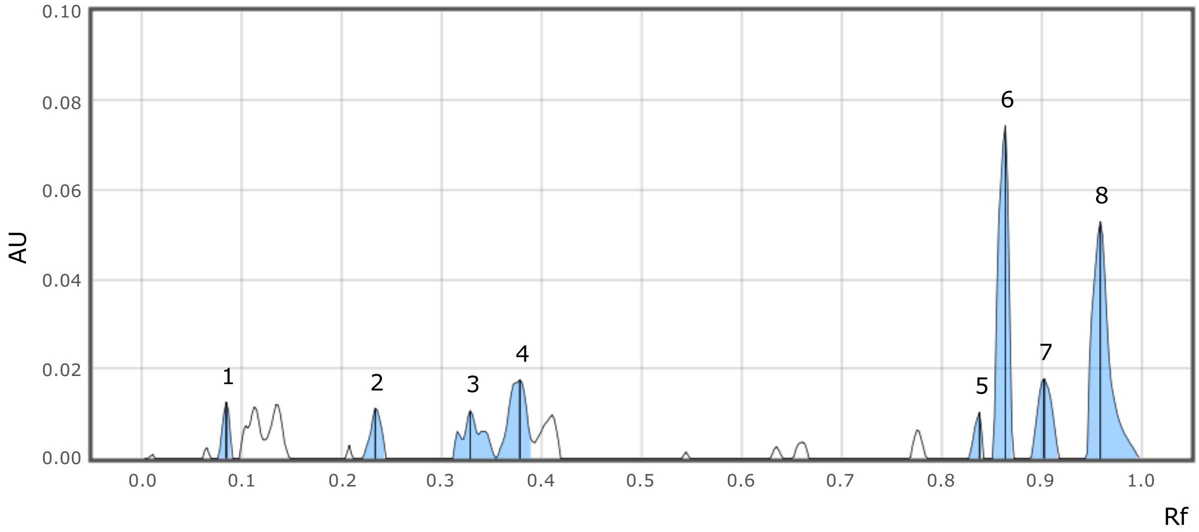

Peak #	Start Rf	Start H	Max Rf	Max H	Max %	End Rf	End H	Area A	Area %	Manual peak	Substance Name
1	0.075	0.0000	0.084	0.0125	6.04	0.090	0.0000	0.00010	3.22	No	
2	0.220	0.0000	0.233	0.0112	5.41	0.244	0.0000	0.00014	4.45	No	
3	0.311	0.0000	0.328	0.0107	5.16	0.354	0.0001	0.00024	7.54	No	
4	0.354	0.0001	0.378	0.0176	8.47	0.391	0.0037	0.00037	11.71	No	
5	0.827	0.0000	0.838	0.0103	4.95	0.843	0.0000	0.00008	2.66	No	
6	0.851	0.0000	0.864	0.0744	35.87	0.873	0.0000	0.00088	28.12	No	
7	0.888	0.0000	0.903	0.0178	8.60	0.918	0.0000	0.00029	9.44	No	
8	0.944	0.0000	0.959	0.0529	25.50	0.998	0.0002	0.00103	32.87	No	

Calibration results:

Height calibration for substance 9-THC @ RT White:

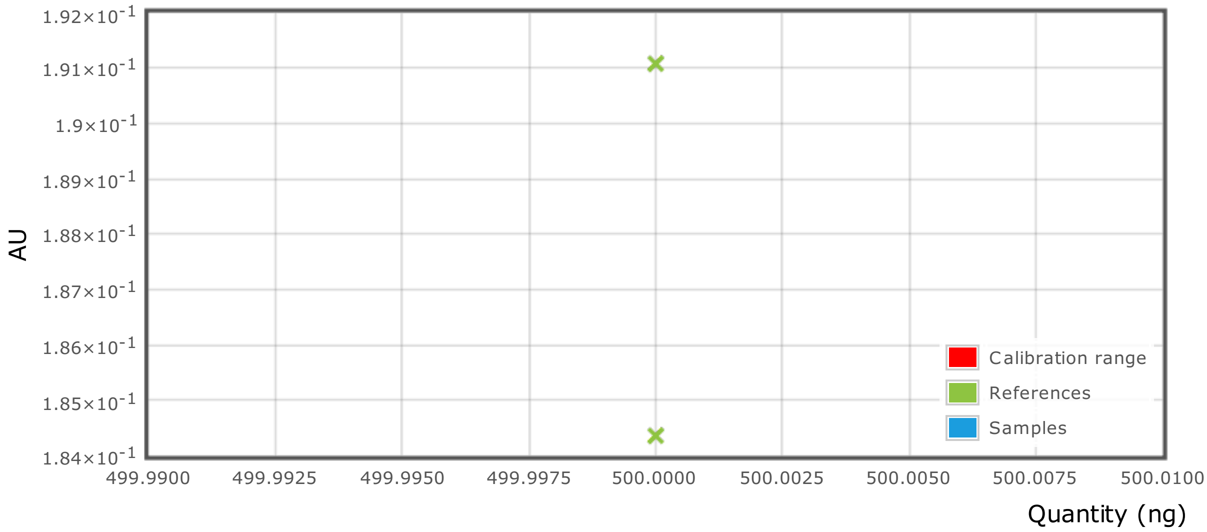

Regression mode	Linear-2
Range deviation	5.00 %
Related substances	Default
Number of references	2
Calibration function	$y=0x$
Coefficient of variation	CV 0.00 %
Correlation coefficient	n/a
⚠	Unable to compute the results for this substance because there wasn't enough groups of references replicas (at least 1 for Linear-1, 2 for Linear2 and Mime-1 and 3 for Polynomial and MiMe-2)

Height calibration for substance CBD @ RT White:

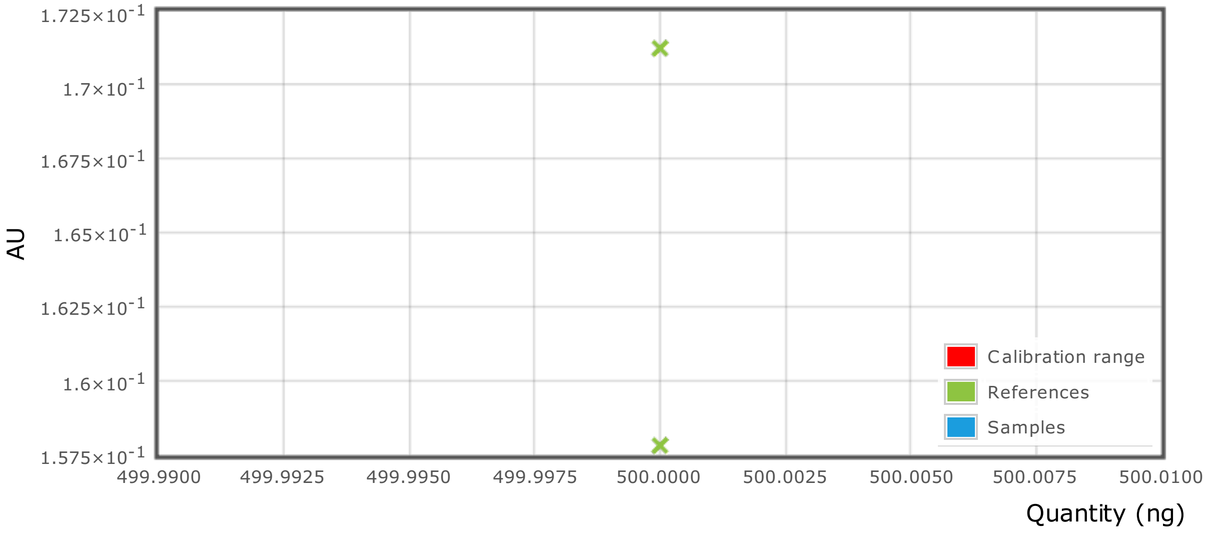

Regression mode	Linear-2
Range deviation	5.00 %
Related substances	Default
Number of references	2
Calibration function	$y=0x$
Coefficient of variation	CV 0.00 %
Correlation coefficient	n/a
⚠	Unable to compute the results for this substance because there wasn't enough groups of references replicas (at least 1 for Linear-1, 2 for Linear2 and Mime-1 and 3 for Polynomial and MiMe-2)

Height calibration for substance CBN @ RT White:

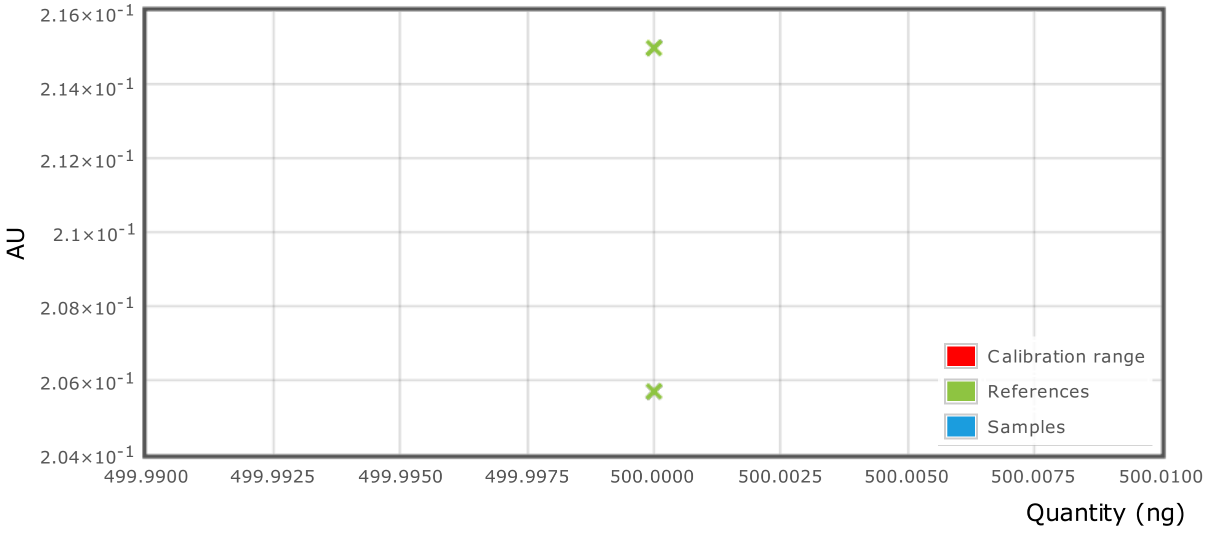

Regression mode	Linear-2
Range deviation	5.00 %
Related substances	Default
Number of references	2
Calibration function	$y=0x$
Coefficient of variation	CV 0.00 %
Correlation coefficient	n/a
⚠	Unable to compute the results for this substance because there wasn't enough groups of references replicas (at least 1 for Linear-1, 2 for Linear2 and Mime-1 and 3 for Polynomial and MiMe-2)

Results:

Substance having no available results		
⚠	CBD	Unable to compute the results for this substance because there wasn't enough groups of references replicas (at least 1 for Linear-1, 2 for Linear2 and Mime-1 and 3 for Polynomial and MiMe-2)
⚠	CBN	There wasn't any sample application available in the assignments for this substance. Please check that the peaks were correctly detected and assigned for this substance.
⚠	9-THC	Unable to compute the results for this substance because there wasn't enough groups of references replicas (at least 1 for Linear-1, 2 for Linear2 and Mime-1 and 3 for Polynomial and MiMe-2)

A track marked with ⚠ means: this result is outside the regression range given by the reference assignments, but is included in the results because it is in the allowed range deviation.

Analyst:	Reviewer:
