# Supplementary material for: High Performance Thin-Layer Chromatography (HPTLC) data of Cannabinoids in ten mobile phase systems
Source: Data Brief. 2020 Jun 30;31:105955. doi: 10.1016/j.dib.2020.105955 (PMC7352075; doi:10.1016/j.dib.2020.105955)
Supplement: Supplementary file 1 [file mmc1.zip › S4-Case sample reports/XHDa-sample run-7.pdf]

Analysis: XHDa-sample run-7

Path: Home/YL Research

Based on method: Samples (no cal)

Created	14-Oct-2019 19:06:32	visionCATSuser
Modified	14-Oct-2019 21:15:51	visionCATSuser
Last HPTLC log	14-Oct-2019 21:15:51	Analysis modified
Explorer notes		

Track	Vial ID	Description	Volume	Position	Type
1	MeOH blank	MeOH Blank	2.0 µl	A1	Sample
2	250ug/mL mix	250ug/mL	2.0 µl	A2	Reference
3	Tetracosane	Tetracosane IS	2.0 µl	A3	Sample
4	s1	[redacted]	2.0 µl	B1	Sample
5	s2	[redacted]	2.0 µl	B2	Sample
6	s3	[redacted]	2.0 µl	B3	Sample
7	s4	[redacted]	2.0 µl	B4	Sample
8	s5	[redacted]	2.0 µl	B5	Sample
9	s6	[redacted]	2.0 µl	B6	Sample
10	s7	[redacted]	2.0 µl	B7	Sample
11	s8	[redacted]	2.0 µl	B8	Sample
12	s9	[redacted]	2.0 µl	B9	Sample
13	s10	[redacted]	2.0 µl	B10	Sample
14	250ug/mL mix	250ug/mL	2.0 µl	A2	Reference
15	MeOH blank	MeOH Blank	2.0 µl	A1	Sample
Sequence table notes					

A track marked with ⚠ means: the application type is overridden in some evaluation(s).

System setup:

Software	Server User-PC, version 2.5.18072.1
ATS4	S/N:080713
Chamber	N/A
Derivatization dip	N/A
Scanner3	S/N:031025
Visualizer	S/N:230515

Chromatography

Plate layout:

Stationary phase	Merck, HPTLC plates silica gel 60 F 254
Plate format	200.0 x 100.0 mm
Application type	Band
Application	Position Y: 8.0 mm, length: 8.0 mm, width: 0.0 mm
Track	First position X: 20.0 mm, distance: 11.4 mm
Solvent front position	70.0 mm
Notes	

Take image clean plate 1a - Visualizer (S/N: 230515):

Quality	Enhanced
RT White	auto capture, Auto, level 85 %, Band
R 254	auto capture, Auto, level 85 %, Band
Instrument diagnostics	Valid diagnostics
Documentation step label	
Notes	

Application 1 - ATS 4 (S/N: 080713):

Spray gas	NI
Sample solvent type	Methanol
Filling speed	15 µl/s
Predosage volume	200 nl
Retraction volume	200 nl
Dosage speed	150 nl/s
Filling quality	User
Rinsing cycles / vacuum	2 / 4 s
Filling cycles / vacuum	1 / 4 s
Rinsing solvent name	Methanol
Nozzle temperature	Unheated
Rack in use	Standard
Instrument diagnostics	Valid diagnostics
Notes	

Development 1 - Chamber:

Tank	TTC 20x10
Mobile phase	Xylene:hexane:diethylamine (25:10:1)
Saturation time	20 min
Use saturation pad	true
Use smartALERT	false
Volume front through	10 ml
Volume rear through	25 ml
Drying time	5 min
Drying temperature	Room temperature
Notes	

Take image developed plate 1a - Visualizer (S/N: 230515):

Quality	Enhanced
RT White	auto capture, Auto, level 85 %, Band
R 254	auto capture, Auto, level 85 %, Band
R 366	auto capture, Auto, level 85 %, Band
Instrument diagnostics	Valid diagnostics
Documentation step label	
Notes	

Scan developed plate 1b - Scanner 3 (S/N: 031025):

Scanner type	Single λ
Optimization for	Resolution
Measurement mode	Absorption
Filter	n/a
Detector mode	Automatic
Scanning speed	20 mm/s
Data resolution	100 µm/step
Slit	5 x 0.2 mm, micro
Partial scan	No
Lamp	Deuterium & Tungsten
Wavelength(s)	254 nm
Instrument diagnostics	Valid diagnostics
Documentation step label	
Notes	

Derivatization 1 - dip:

Reagent name	
Dipping speed	5
Dipping time	0 s
Reagent preparation	
Heating	100 °C for 3 min, heated after
Notes	

Take image derivatized plate 1a - Visualizer (S/N: 230515):

Quality	Enhanced
RT White	auto capture, Auto, level 85 %, Band
R 366	auto capture, Auto, level 85 %, Band
Instrument diagnostics	Valid diagnostics
Documentation step label	
Notes	

System suitability tests:

SST settings:

SST tracks	

Data acquisition

Application 1 - ATS 4 (S/N: 080713):

Executed	14-Oct-2019 19:30:30 visionCATSuser

Development 1 - Chamber:

Executed	14-Oct-2019 20:18:50 visionCATSuser

Take image developed plate 1a - Visualizer (S/N: 230515):

Executed	14-Oct-2019 21:06:17 visionCATSuser

RT White

Developed, RemTransVis

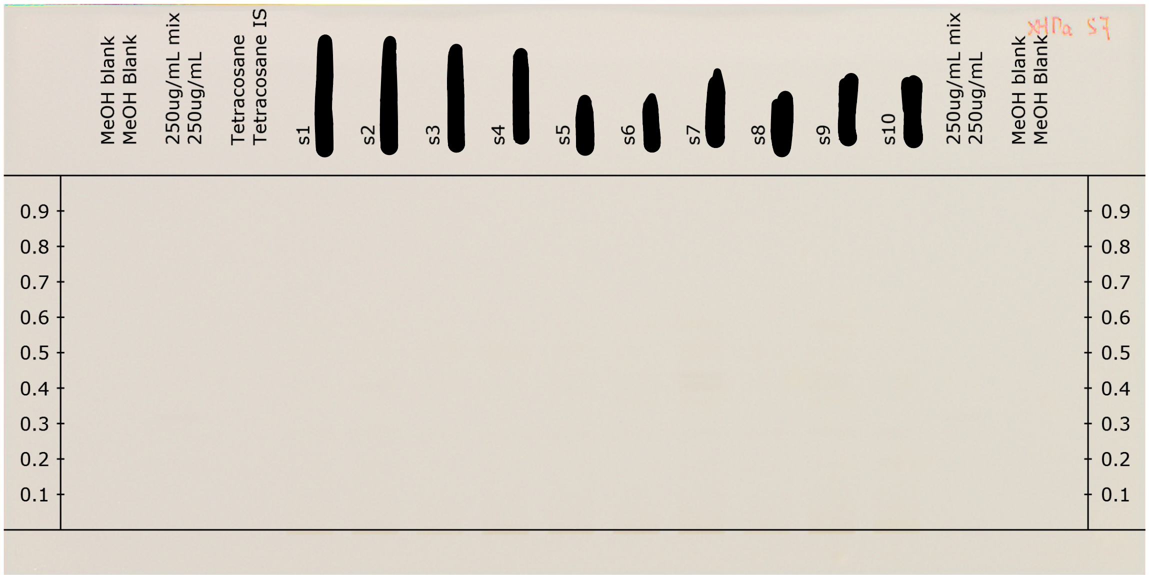

Exposure	0.082 s
Contrast	1
Normalized exposure	Disabled
Clarify	Disabled
White balance	1.00, 1.00, 1.00

R 254

Developed, Remission254

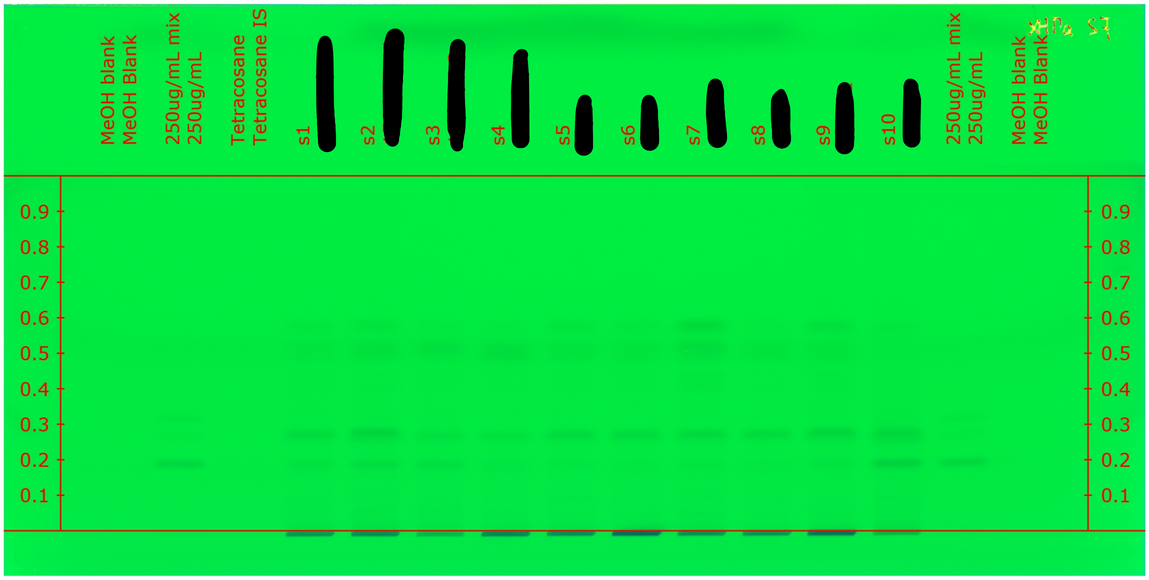

Exposure	0.268 s
Contrast	1
Normalized exposure	Disabled
Clarify	Disabled
White balance	1.00, 1.00, 1.00

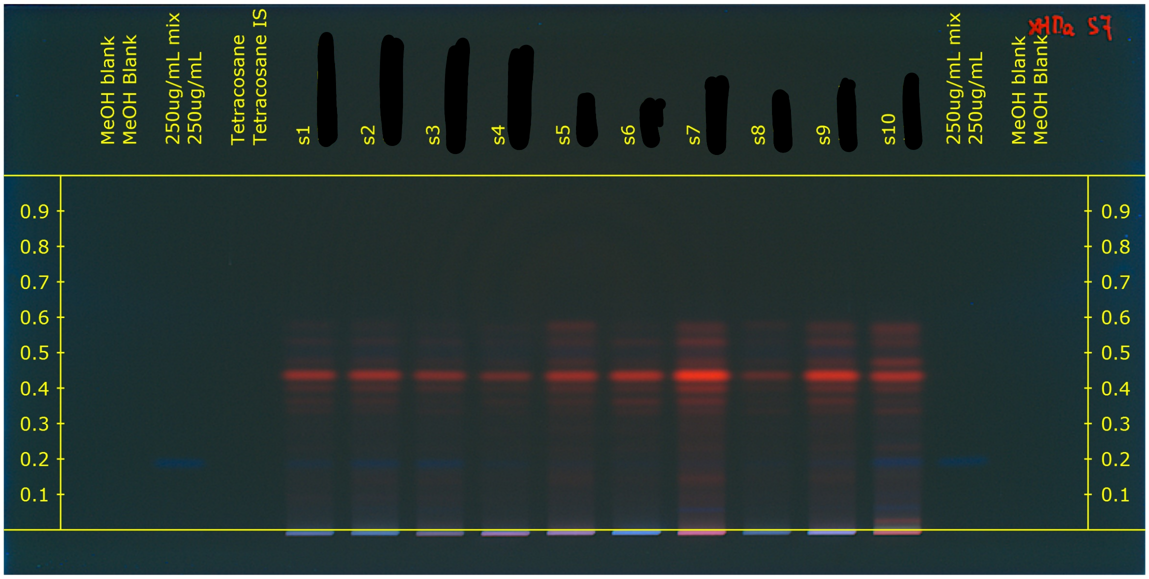

Exposure	2.835 s
Contrast	1
Normalized exposure	Disabled
Clarify	Disabled
White balance	1.00, 1.00, 1.00

Scan developed plate 1b - Scanner 3 (S/N: 031025):

Executed	14-Oct-2019 21:07:54 visionCATSuser

Scan:

Wavelength	254 nm

Track 1:

Type	Single λ

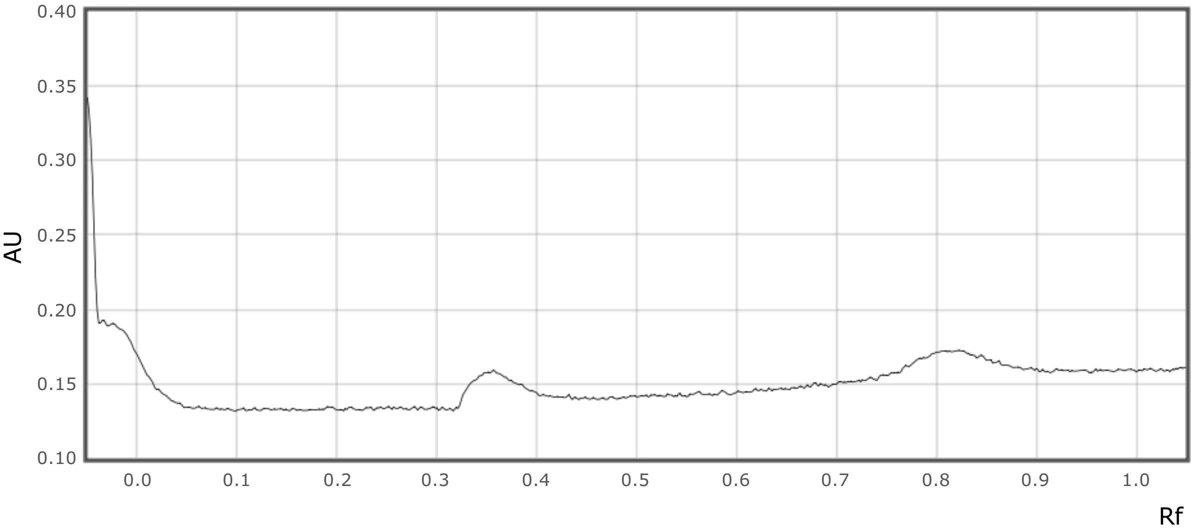

Track 2:	
Type	Single λ

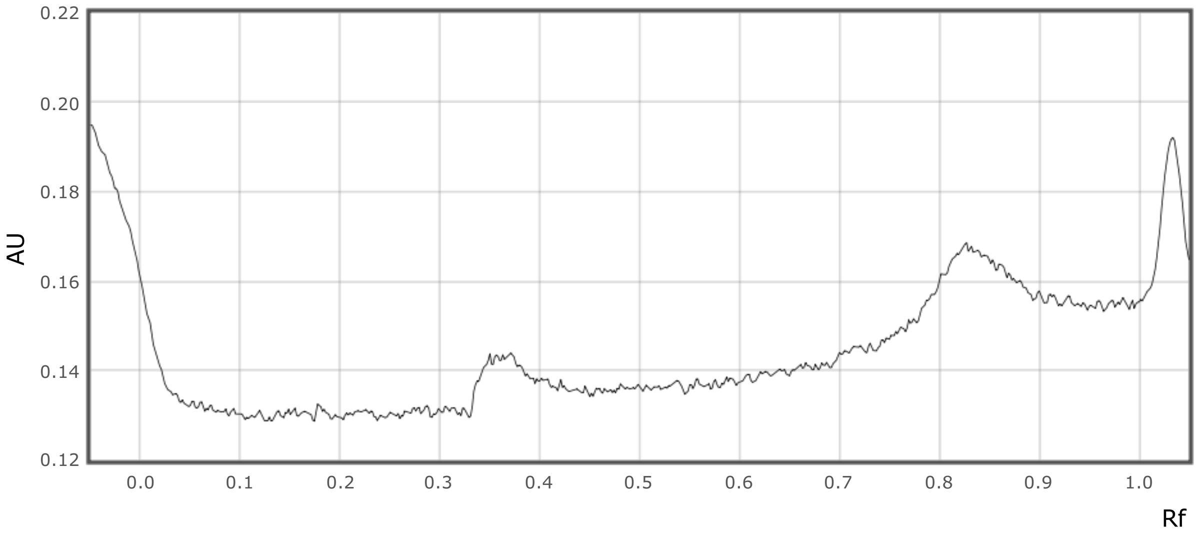

Track 3:	
Type	Single λ

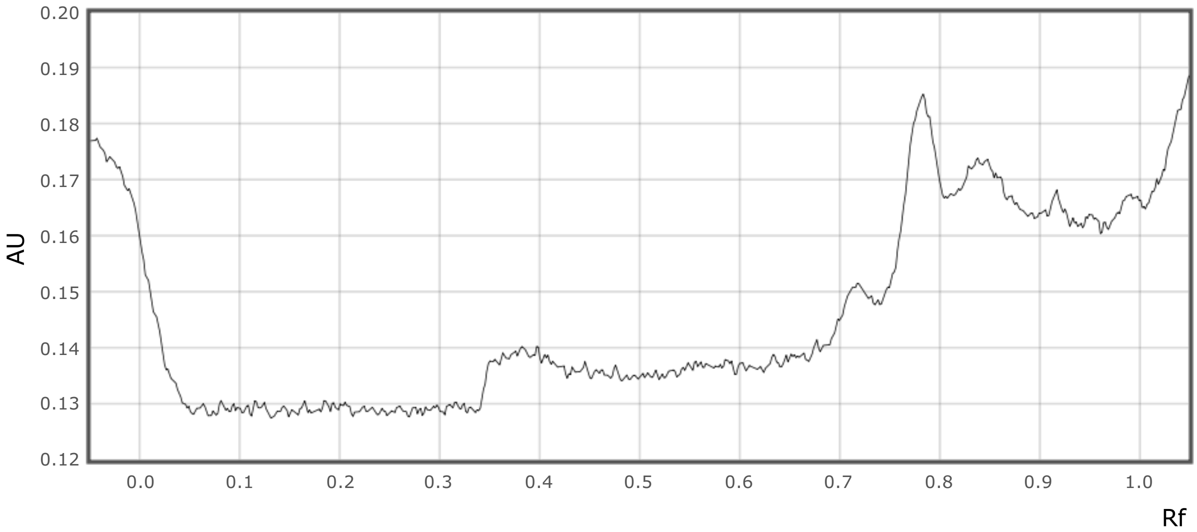

Track 4:	
Type	Single λ

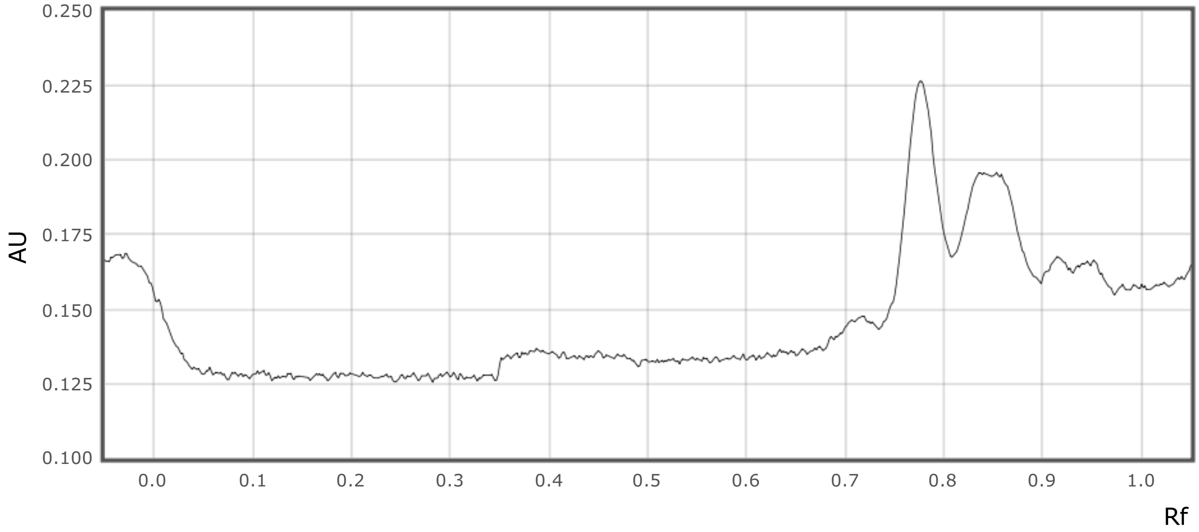

Track 5:	
Type	Single λ

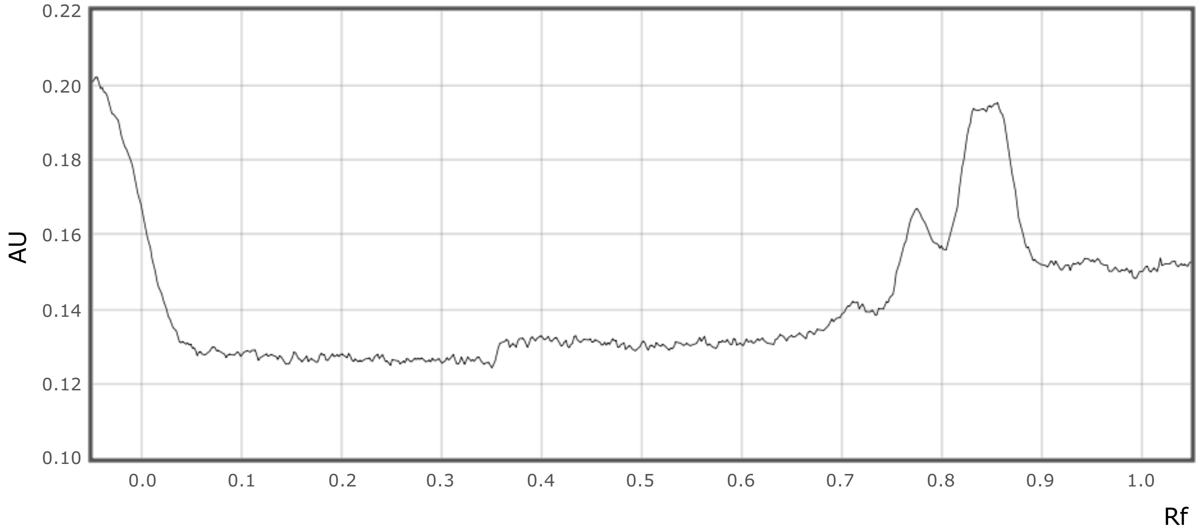

Track 6:	
Type	Single λ

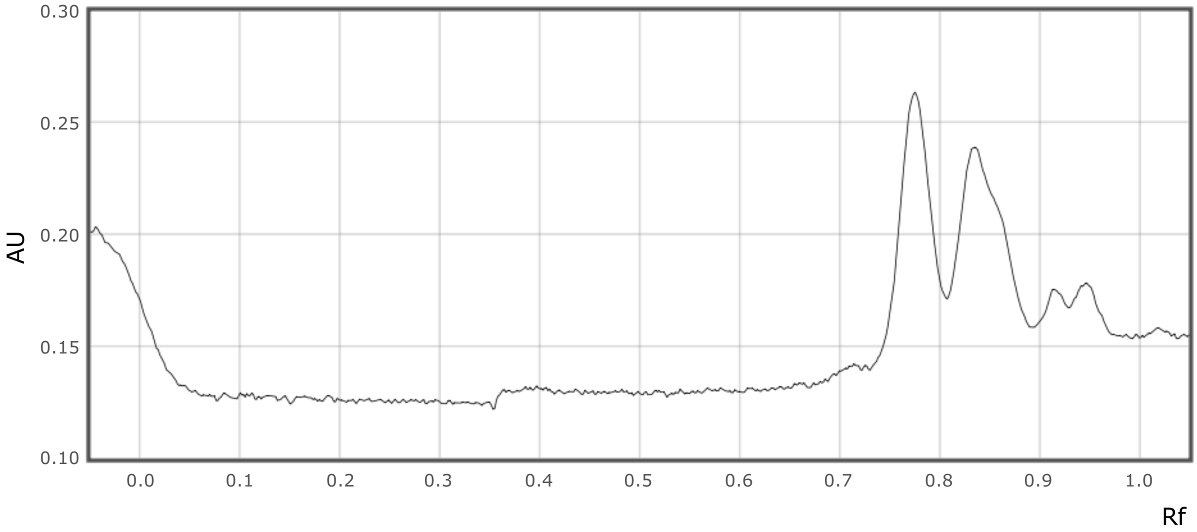

Track 7:	
Type	Single λ

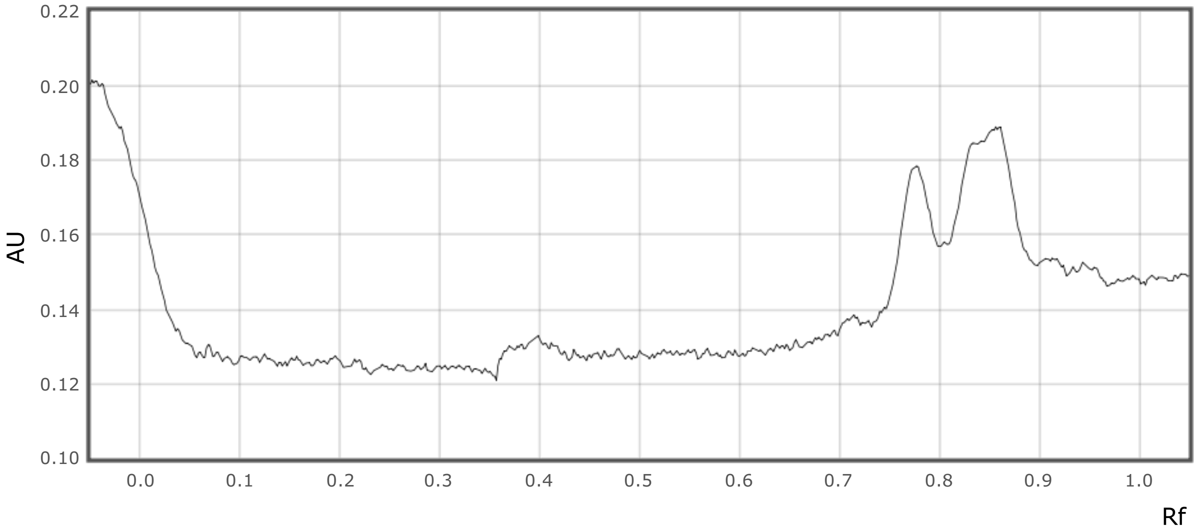

Track 8:	
Type	Single λ

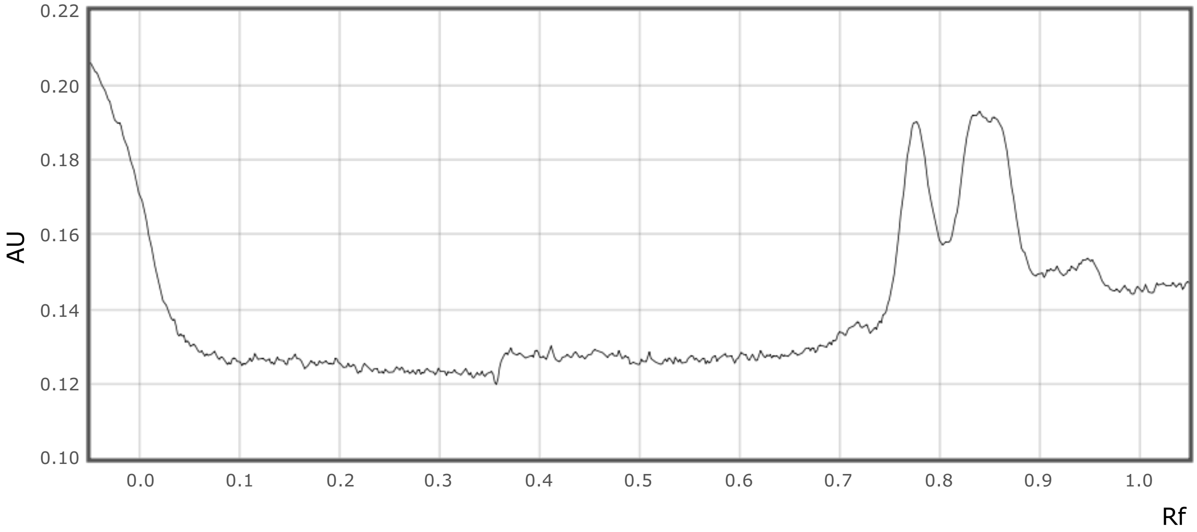

Track 9:

Type	Single λ

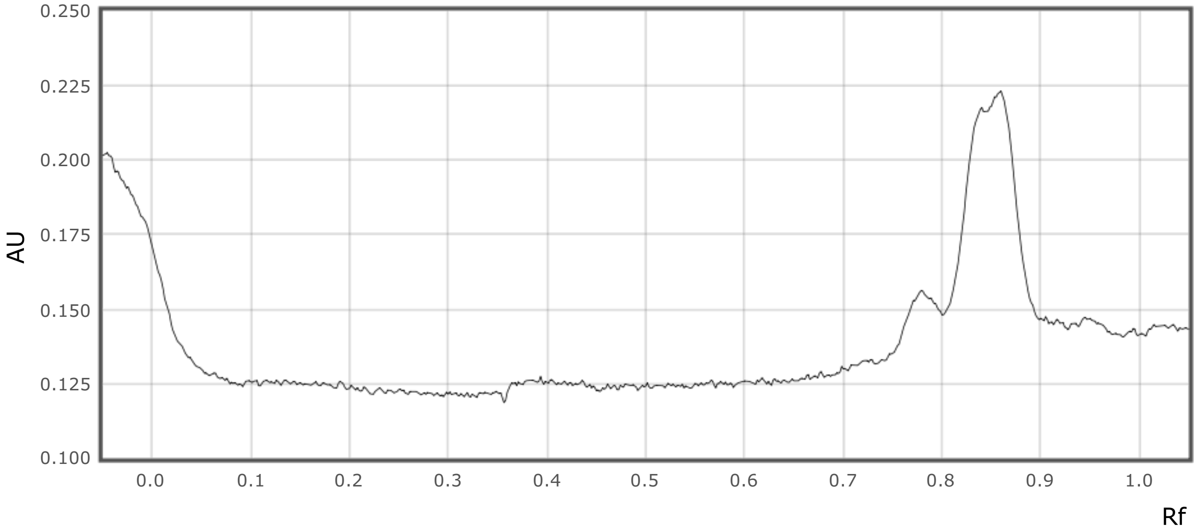

Track 10:

Type	Single λ

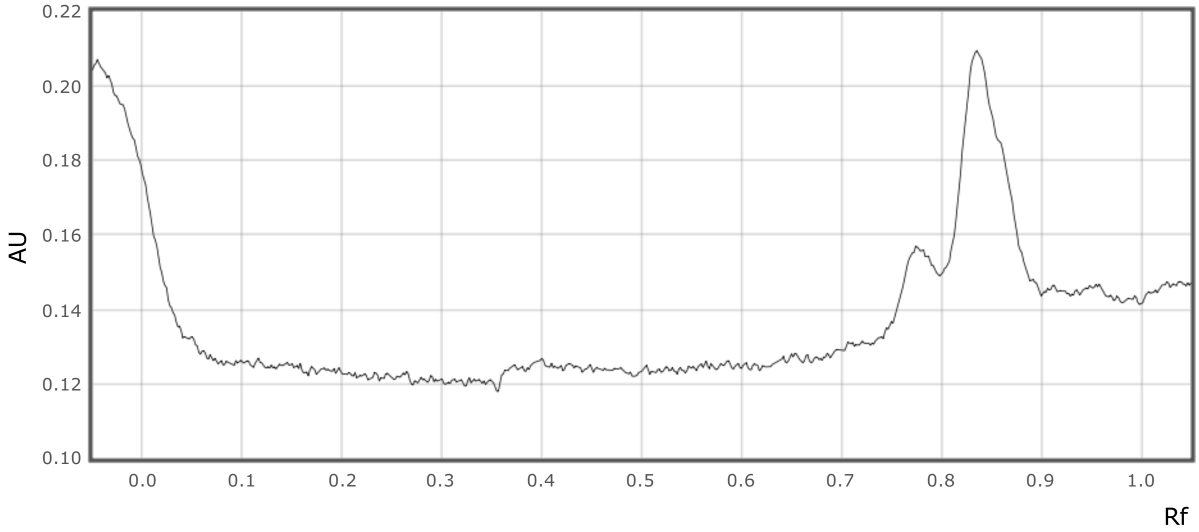

Track 11:

Type	Single λ

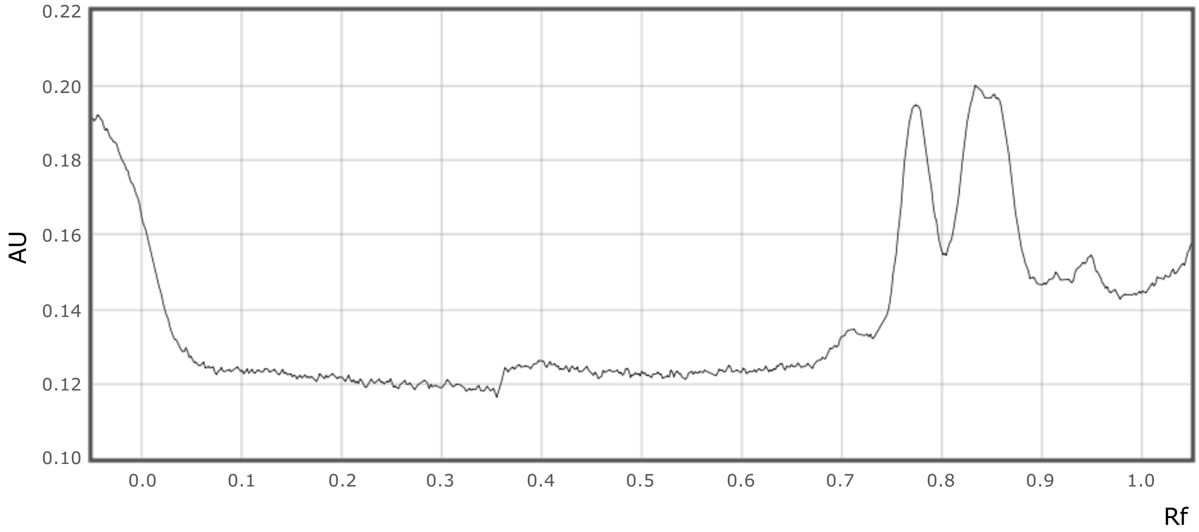

Track 12:

Type	Single λ

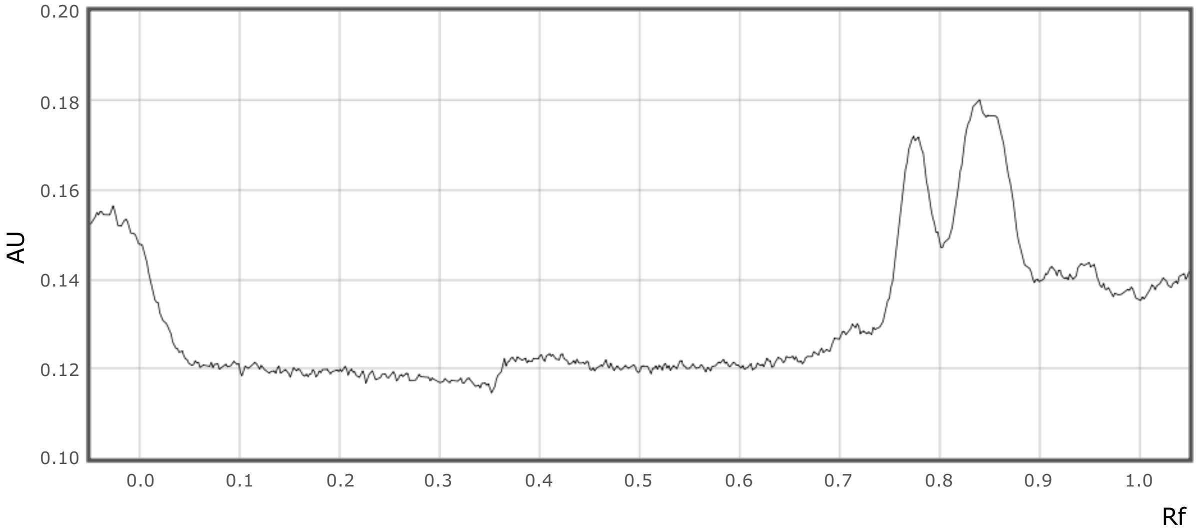

Track 13:

Type	Single λ

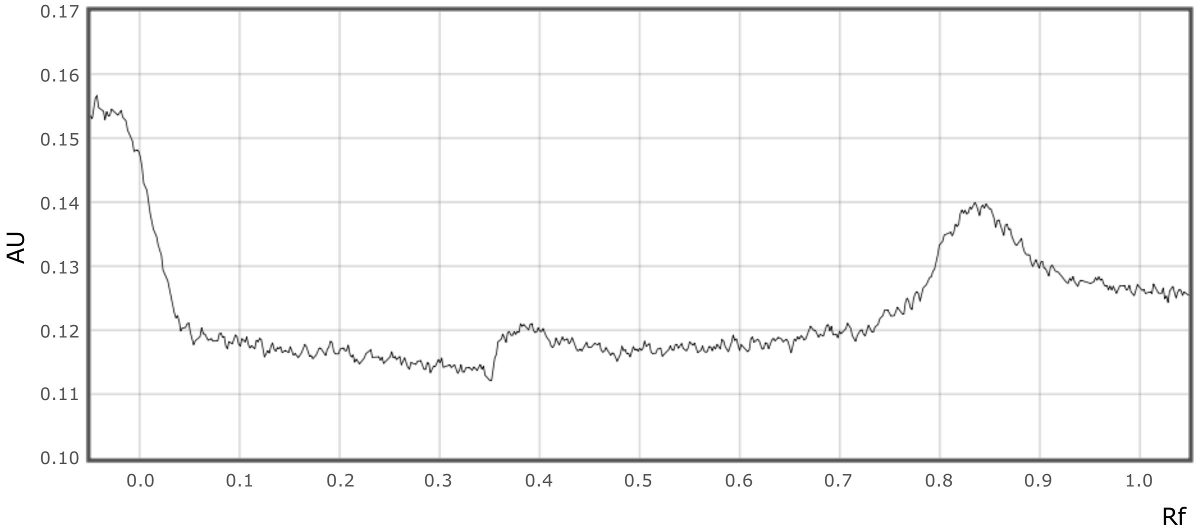

Track 14:

Type	Single λ

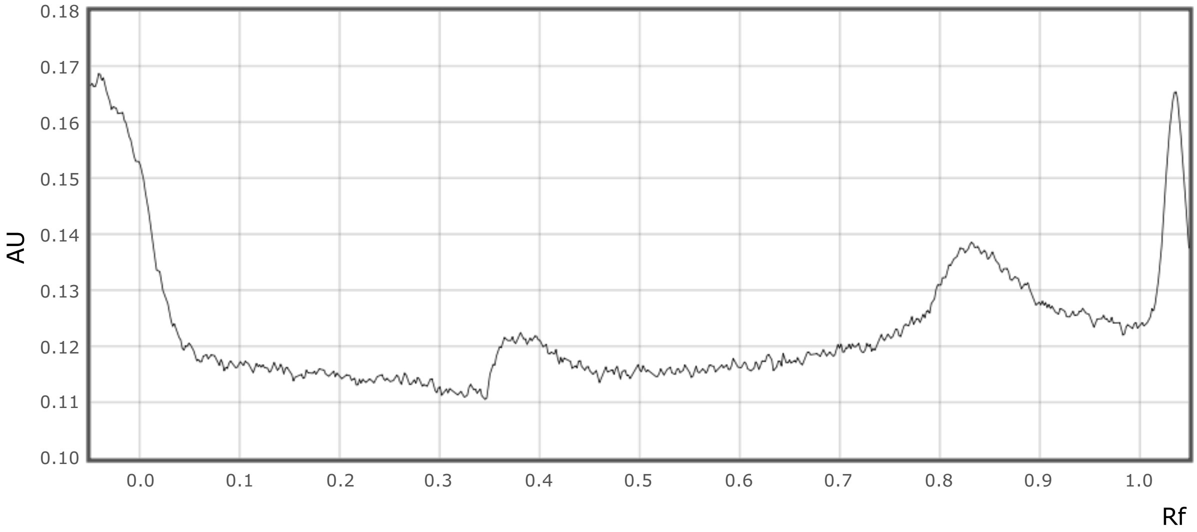

Track 15:

Type	Single λ

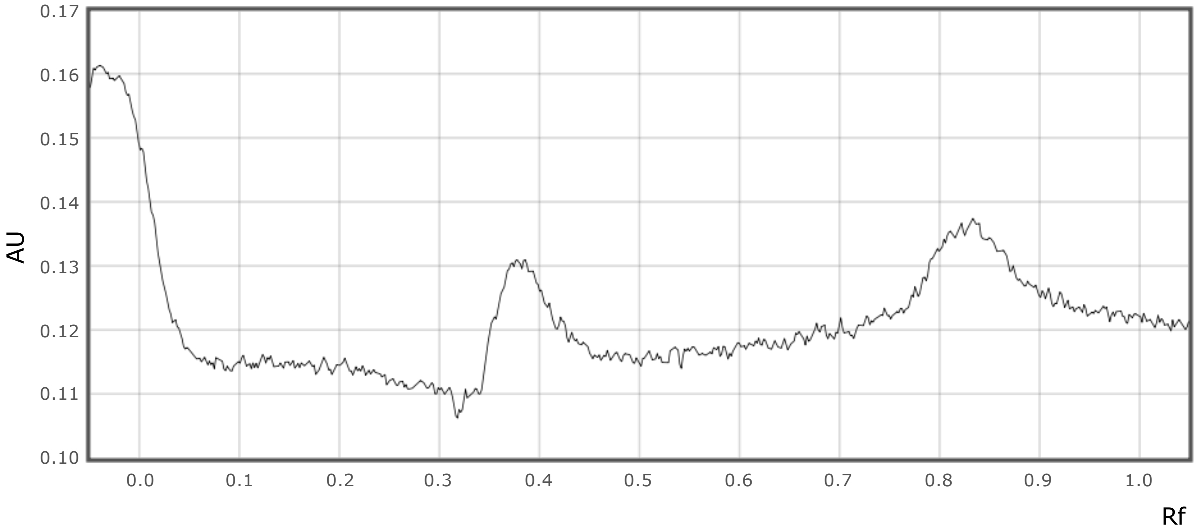

Derivatization 1 - dip:

Executed	14-Oct-2019 21:10:34 visionCATSuser

Take image derivatized plate 1a - Visualizer (S/N: 230515):

Executed	14-Oct-2019 21:13:33 visionCATSuser

RT White Derivatized, RemTransVis

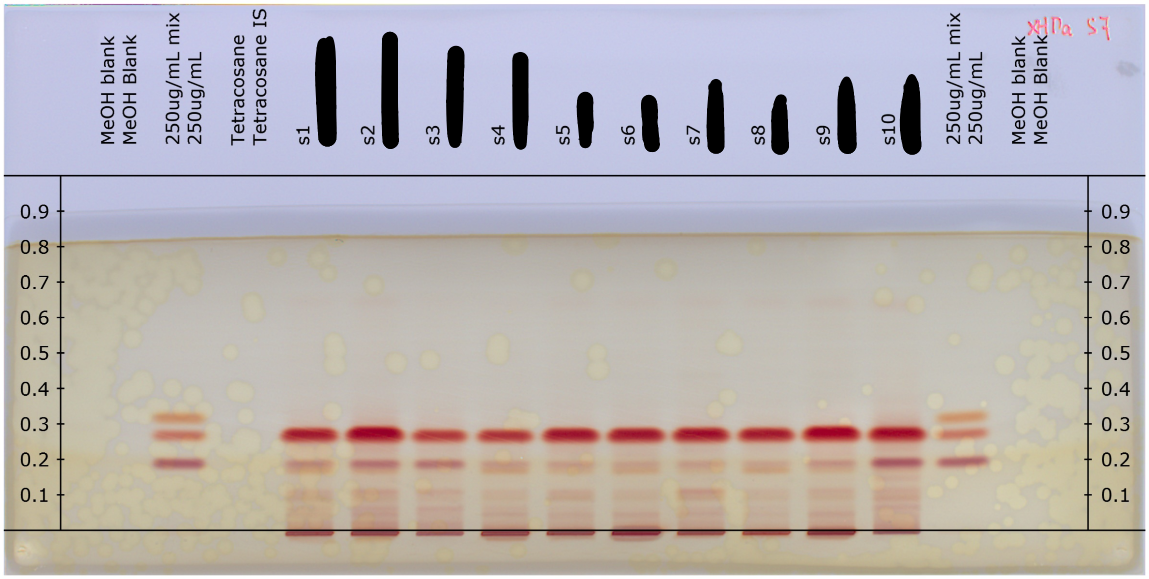

Exposure	0.072 s
Contrast	1
Normalized exposure	Disabled
Clarify	Disabled
White balance	1.18, 1.10, 0.80

R 366 Derivatized, Remission366

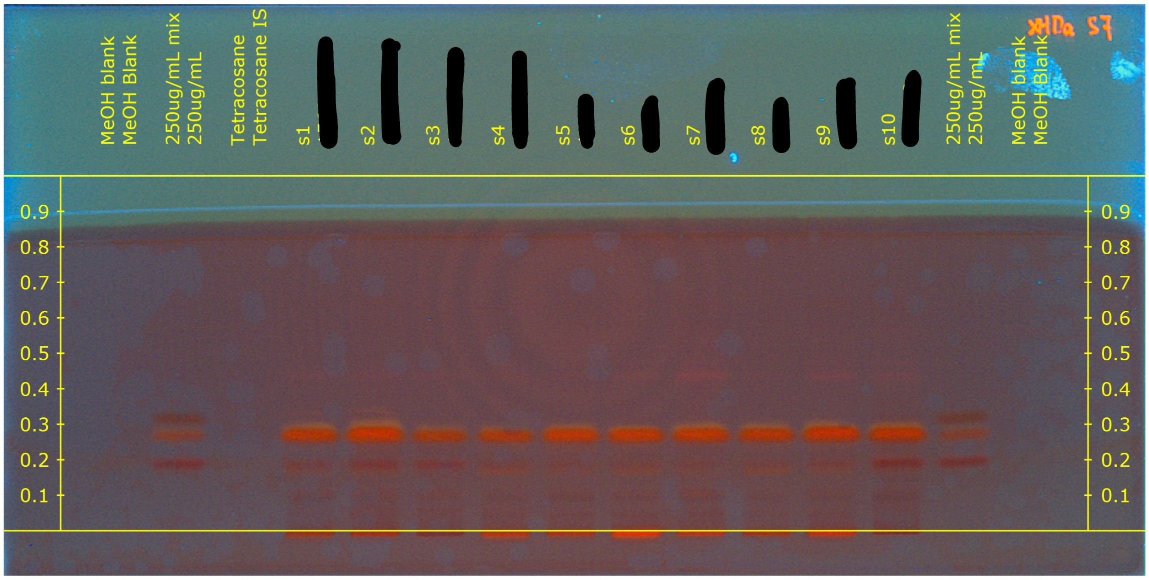

Exposure	9.999 s
Contrast	1
Normalized exposure	Disabled
Clarify	Disabled
White balance	1.00, 1.00, 1.00

Evaluation 1 :

Validated	false
Step	Take image derivatized plate 1a
Concentration unit type	Mass / volume
Notes	

Definition:

References:

250ug/mL mix

Substance Name	Concentration	Purity
9-THC	250.000 µg/ml	100.00 %
CBD	250.000 µg/ml	100.00 %
CBN	250.000 µg/ml	100.00 %

Samples:

Vial ID	Amount	Volume solution	Reference amount	Related to
MeOH blank		0.00 ml		
Tetracosane		0.00 ml		
s1		0.00 ml		
s2		0.00 ml		
s3		0.00 ml		
s4		0.00 ml		
s5		0.00 ml		
s6		0.00 ml		
s7		0.00 ml		
s8		0.00 ml		
s9		0.00 ml		
s10		0.00 ml		

Integration parameters:

Bounds	[0.000,1.000]
Smoothing	Savitzky–Golay of order 3 and window 7
Baseline correction	Lowest slope with noise 0.05
Profile subtraction	Profile subtraction from track 1
Peaks detection	Gauss (legacy) with sensitivity 0.1, separation 1 and threshold 0.1

Scan:

Wavelength	RT White

Track 1:

Type	Sample
Vial ID	MeOH blank
Description	MeOH Blank
Volume	2.0 µl

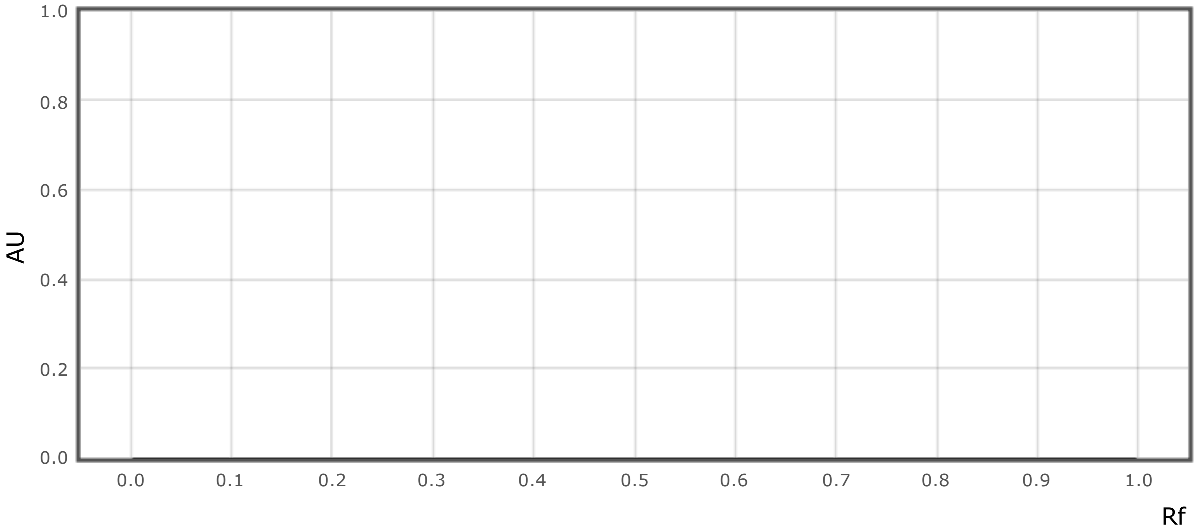

Peak	Start		Max			End		Area		Manual	Substance
#	Rf	H	Rf	H	%	Rf	H	A	%	peak	Name

Track 2:

Type	Reference
Vial ID	250ug/mL mix
Description	250ug/mL
Volume	2.0 µl

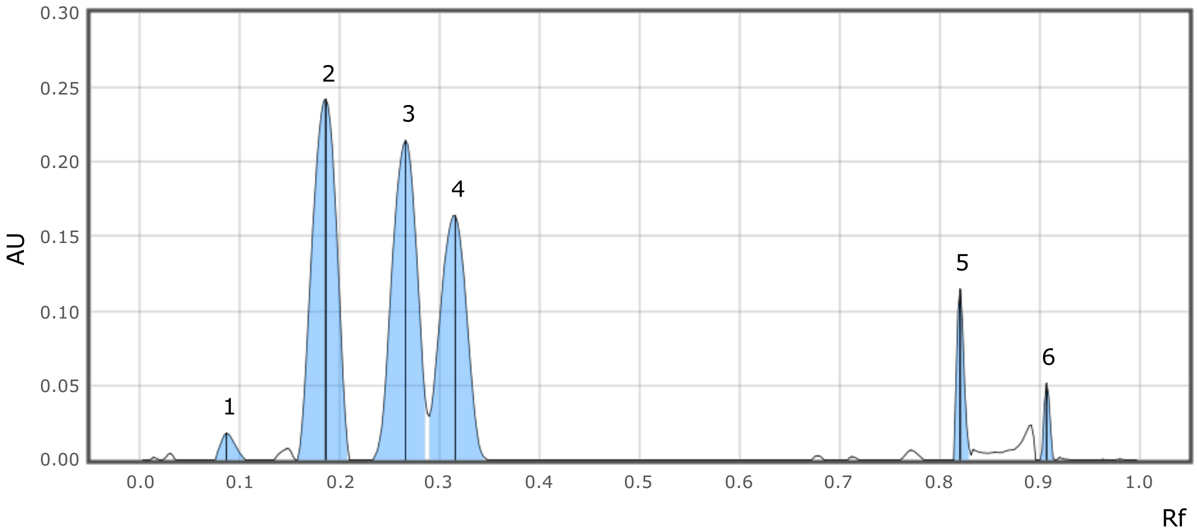

Peak	Start		Max			End		Area		Manual	Substance
#	Rf	H	Rf	H	%	Rf	H	A	%	peak	Name
1	0.073	0.0000	0.086	0.0179	2.23	0.106	0.0000	0.00029	1.50	No	
2	0.155	0.0000	0.186	0.2421	30.10	0.209	0.0000	0.00681	34.87	No	CBN
3	0.231	0.0000	0.266	0.2143	26.64	0.287	0.0313	0.00614	31.45	No	9-THC
4	0.289	0.0292	0.315	0.1640	20.40	0.348	0.0000	0.00499	25.52	No	CBD
5	0.814	0.0000	0.821	0.1146	14.25	0.832	0.0034	0.00096	4.92	No	
6	0.901	0.0000	0.907	0.0514	6.39	0.916	0.0000	0.00034	1.74	No	

Track 3:

Type	Sample
Vial ID	Tetracosane
Description	Tetracosane IS
Volume	2.0 µl

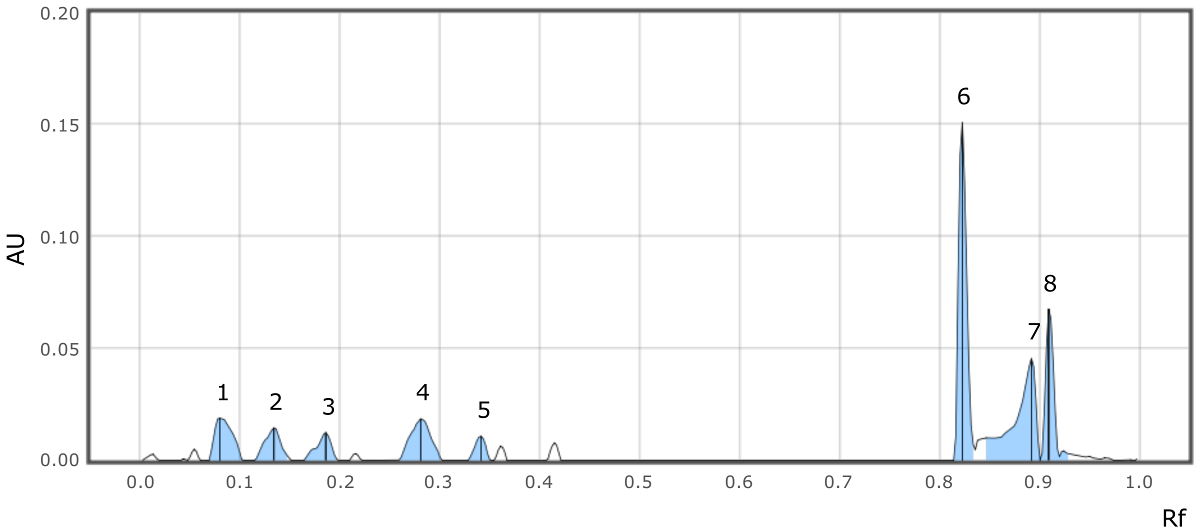

Peak	Start		Max			End		Area		Manual	Substance
#	Rf	H	Rf	H	%	Rf	H	A	%	peak	Name
1	0.069	0.0000	0.080	0.0188	5.54	0.103	0.0000	0.00039	8.58	No	
2	0.114	0.0000	0.134	0.0145	4.29	0.151	0.0000	0.00026	5.78	No	
3	0.164	0.0000	0.186	0.0124	3.66	0.199	0.0000	0.00020	4.43	No	
4	0.257	0.0000	0.281	0.0186	5.47	0.302	0.0000	0.00044	9.51	No	
5	0.328	0.0000	0.341	0.0107	3.16	0.352	0.0000	0.00013	2.86	No	
6	0.814	0.0000	0.823	0.1510	44.52	0.836	0.0047	0.00141	30.73	No	
7	0.843	0.0094	0.892	0.0455	13.42	0.901	0.0000	0.00107	23.41	No	
8	0.901	0.0000	0.910	0.0676	19.95	0.946	0.0015	0.00067	14.70	No	

Track 4:

Type	Sample
Vial ID	s1
Description	[redacted]
Volume	2.0 µl

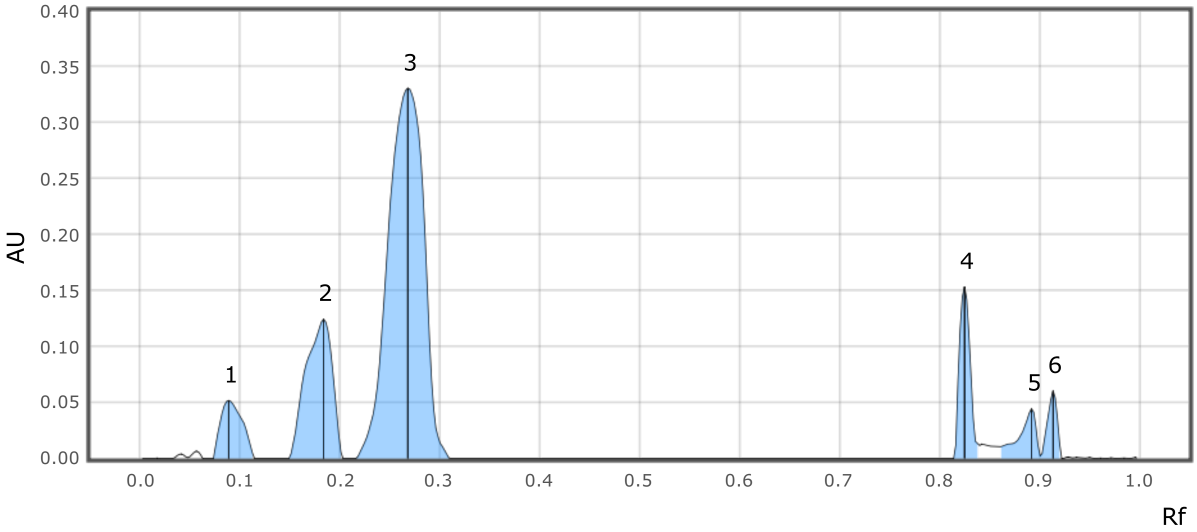

Peak #	Start		Max			End		Area		Manual peak	Substance Name
	Rf	H	Rf	H	%	Rf	H	A	%		
1	0.073	0.0000	0.088	0.0518	6.76	0.114	0.0000	0.00132	5.91	No	
2	0.149	0.0000	0.183	0.1244	16.26	0.203	0.0000	0.00381	17.12	No	
3	0.216	0.0000	0.268	0.3310	43.25	0.311	0.0000	0.01373	61.64	No	9-THC
4	0.814	0.0000	0.825	0.1534	20.05	0.840	0.0116	0.00192	8.62	No	
5	0.862	0.0105	0.892	0.0443	5.80	0.901	0.0021	0.00084	3.76	No	
6	0.901	0.0021	0.914	0.0604	7.89	0.922	0.0000	0.00066	2.96	No	

Track 5:

Type	Sample
Vial ID	s2
Description	[redacted]
Volume	2.0 µl

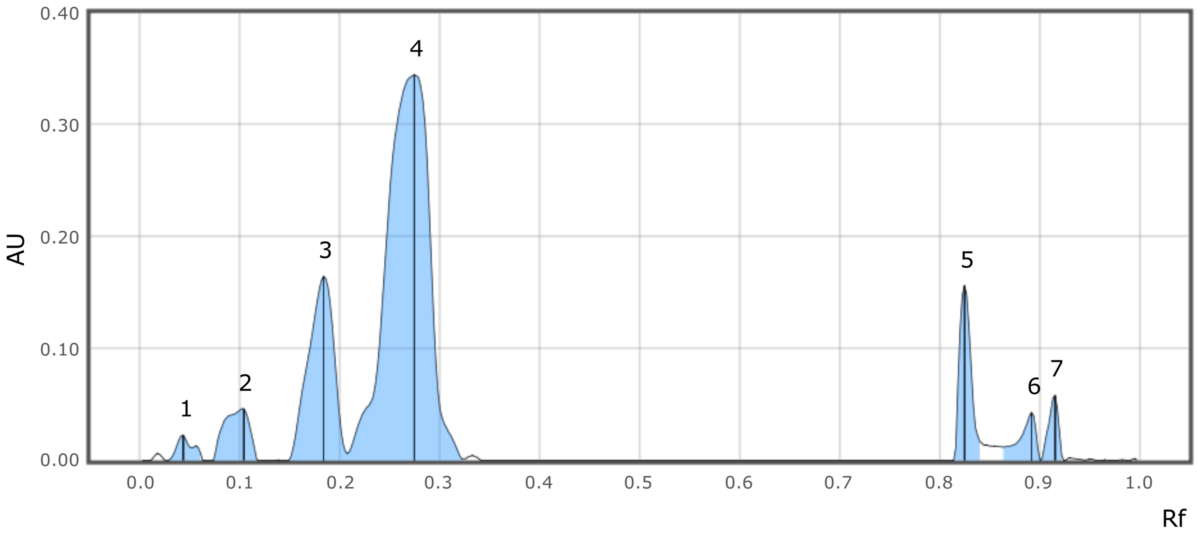

Peak	Start		Max			End		Area		Manual	Substance
#	Rf	H	Rf	H	%	Rf	H	A	%	peak	Name
1	0.028	0.0000	0.043	0.0225	2.69	0.062	0.0000	0.00041	1.48	No	
2	0.073	0.0000	0.103	0.0462	5.53	0.119	0.0000	0.00138	5.02	No	
3	0.147	0.0000	0.183	0.1647	19.71	0.207	0.0060	0.00485	17.66	No	
4	0.207	0.0060	0.274	0.3448	41.26	0.324	0.0014	0.01725	62.77	No	9-THC
5	0.814	0.0000	0.825	0.1563	18.71	0.845	0.0136	0.00215	7.84	No	
6	0.864	0.0119	0.892	0.0430	5.14	0.901	0.0000	0.00079	2.88	No	
7	0.901	0.0000	0.916	0.0581	6.95	0.925	0.0000	0.00065	2.36	No	

Track 6:

Type	Sample
Vial ID	s3
Description	[redacted]
Volume	2.0 µl

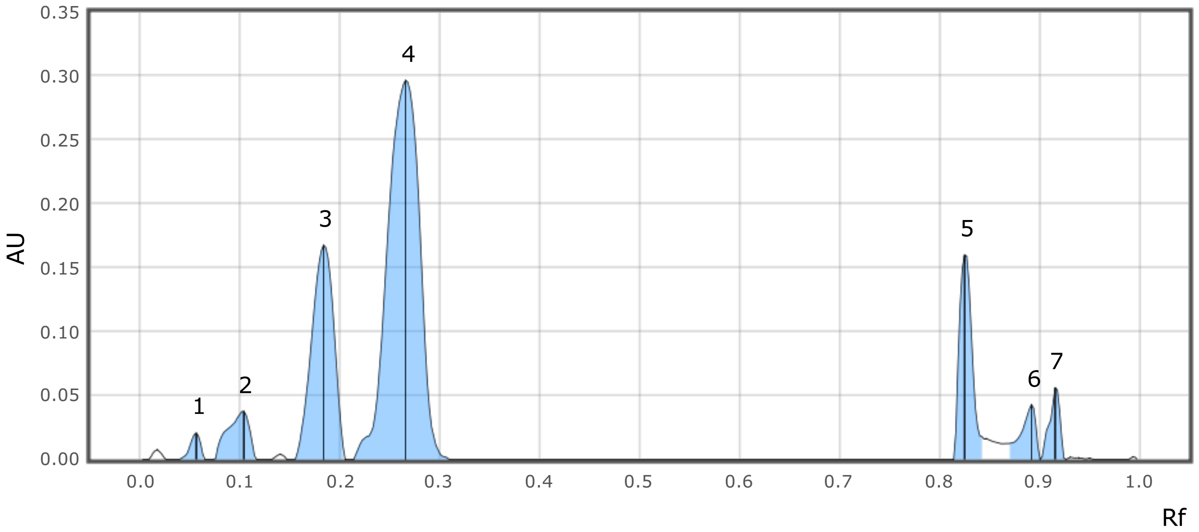

Peak	Start		Max			End		Area		Manual	Substance
#	Rf	H	Rf	H	%	Rf	H	A	%	peak	Name
1	0.039	0.0000	0.056	0.0207	2.65	0.065	0.0000	0.00024	1.18	No	
2	0.073	0.0000	0.103	0.0377	4.82	0.116	0.0000	0.00095	4.67	No	
3	0.153	0.0000	0.183	0.1677	21.45	0.205	0.0000	0.00427	21.06	No	
4	0.214	0.0000	0.266	0.2966	37.96	0.311	0.0000	0.01099	54.16	No	9-THC
5	0.814	0.0000	0.825	0.1599	20.46	0.845	0.0160	0.00239	11.80	No	
6	0.864	0.0122	0.892	0.0429	5.49	0.901	0.0000	0.00079	3.87	No	
7	0.901	0.0000	0.916	0.0560	7.17	0.927	0.0000	0.00066	3.26	No	

Track 7:

Type	Sample
Vial ID	s4
Description	[redacted]
Volume	2.0 µl

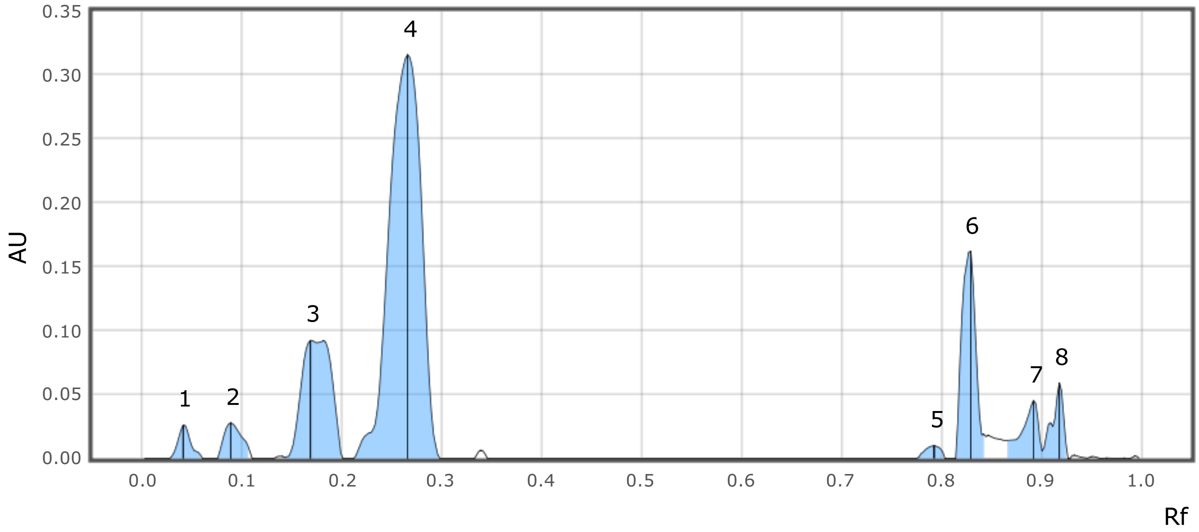

Peak #	Start Rf	Start H	Max Rf	Max H	Max %	End Rf	End H	Area A	Area %	Manual peak	Substance Name
1	0.026	0.0000	0.041	0.0259	3.51	0.060	0.0000	0.00037	1.83	No	
2	0.075	0.0000	0.088	0.0278	3.76	0.110	0.0000	0.00058	2.86	No	
3	0.142	0.0008	0.168	0.0923	12.51	0.201	0.0000	0.00323	15.90	No	
4	0.211	0.0000	0.266	0.3158	42.78	0.298	0.0000	0.01172	57.59	No	9-THC
5	0.776	0.0000	0.793	0.0100	1.36	0.804	0.0000	0.00018	0.90	No	
6	0.814	0.0000	0.830	0.1619	21.93	0.845	0.0172	0.00263	12.94	No	
7	0.864	0.0141	0.892	0.0454	6.15	0.901	0.0057	0.00087	4.29	No	
8	0.901	0.0057	0.918	0.0591	8.00	0.927	0.0000	0.00075	3.69	No	

Track 8:

Type	Sample
Vial ID	s5
Description	[redacted]
Volume	2.0 µl

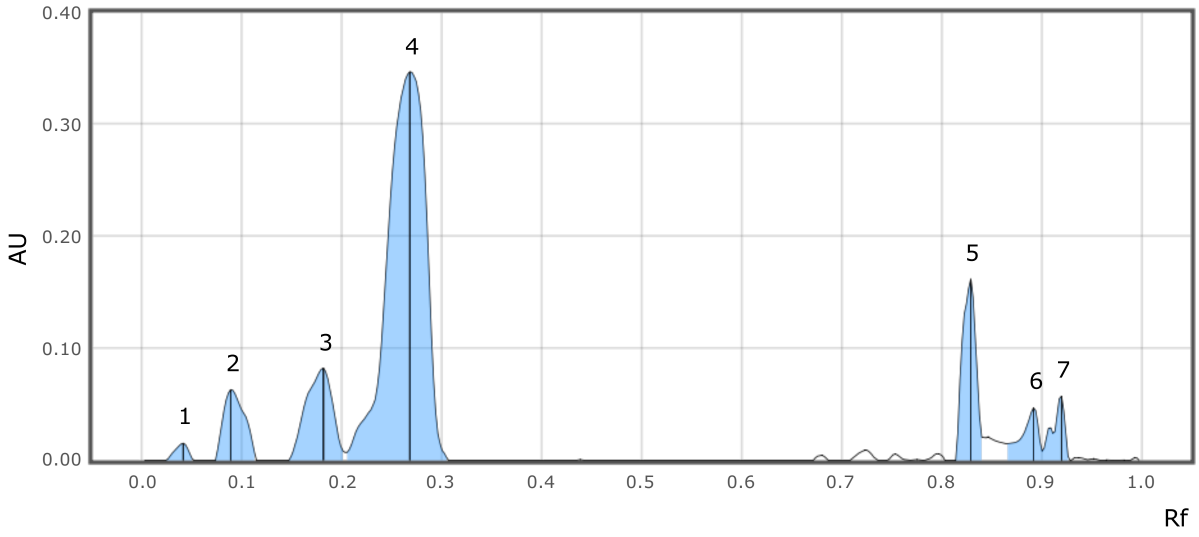

Peak #	Start Rf	Start H	Max Rf	Max H	Max %	End Rf	End H	Area A	Area %	Manual peak	Substance Name
1	0.023	0.0000	0.041	0.0153	1.97	0.052	0.0000	0.00023	0.95	No	
2	0.073	0.0000	0.088	0.0631	8.16	0.114	0.0000	0.00157	6.52	No	
3	0.147	0.0000	0.181	0.0822	10.63	0.203	0.0072	0.00255	10.61	No	
4	0.205	0.0071	0.268	0.3469	44.85	0.307	0.0000	0.01541	64.09	No	9-THC
5	0.814	0.0000	0.830	0.1619	20.92	0.845	0.0201	0.00262	10.89	No	
6	0.866	0.0149	0.892	0.0468	6.05	0.901	0.0081	0.00088	3.67	No	
7	0.901	0.0081	0.920	0.0573	7.41	0.929	0.0000	0.00079	3.28	No	

Track 9:

Type	Sample
Vial ID	s6
Description	[redacted]
Volume	2.0 µl

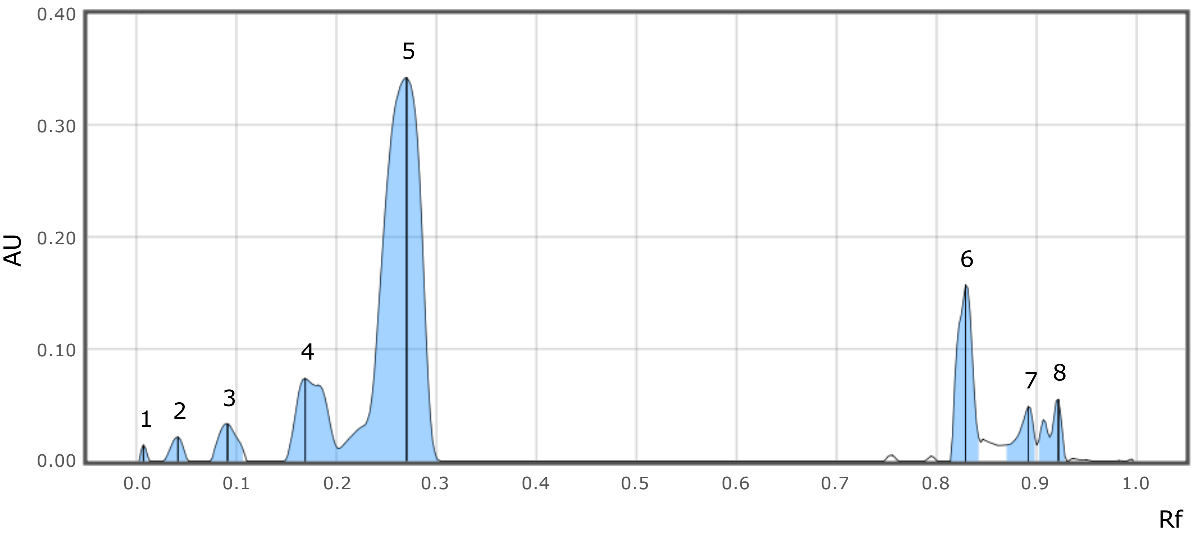

Peak #	Start Rf	Start H	Max Rf	Max H	Max %	End Rf	End H	Area A	Area %	Manual peak	Substance Name
1	0.002	0.0000	0.006	0.0145	1.94	0.013	0.0000	0.00009	0.37	No	
2	0.026	0.0000	0.041	0.0215	2.88	0.052	0.0000	0.00030	1.27	No	
3	0.073	0.0000	0.090	0.0335	4.48	0.110	0.0000	0.00073	3.16	No	
4	0.147	0.0000	0.168	0.0739	9.88	0.201	0.0114	0.00253	10.91	No	
5	0.201	0.0114	0.270	0.3428	45.84	0.304	0.0000	0.01502	64.71	No	9-THC
6	0.814	0.0000	0.830	0.1575	21.07	0.845	0.0169	0.00274	11.79	No	
7	0.864	0.0140	0.892	0.0488	6.52	0.901	0.0143	0.00094	4.07	No	
8	0.903	0.0186	0.922	0.0554	7.41	0.931	0.0000	0.00086	3.72	No	

Track 10:

Type	Sample
Vial ID	s7
Description	[redacted]
Volume	2.0 µl

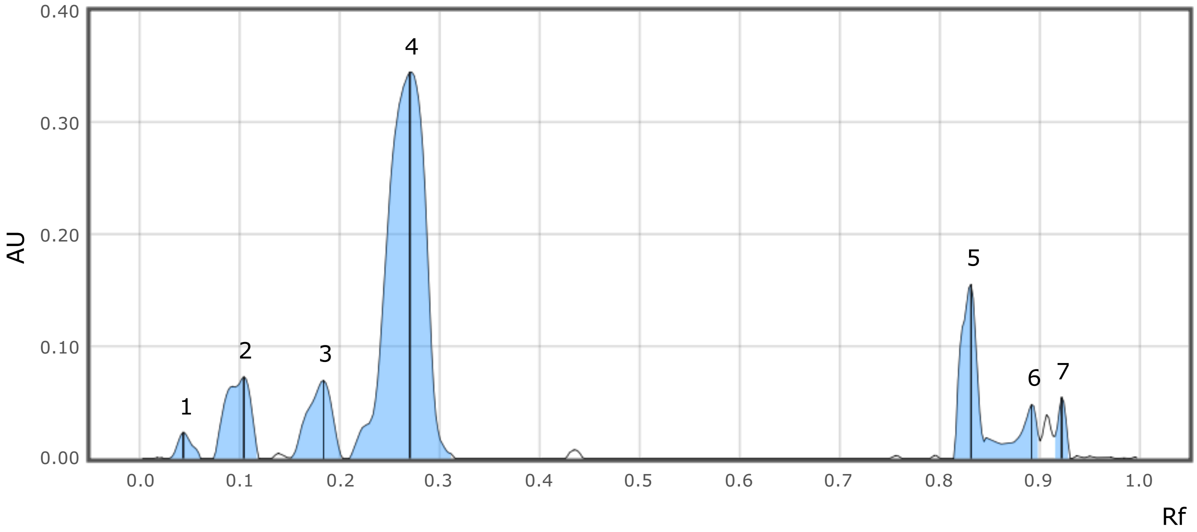

Peak #	Start		Max			End		Area		Manual peak	Substance Name
	Rf	H	Rf	H	%	Rf	H	A	%		
1	0.028	0.0000	0.043	0.0235	3.05	0.062	0.0000	0.00037	1.52	No	
2	0.073	0.0000	0.103	0.0729	9.47	0.119	0.0000	0.00213	8.84	No	
3	0.151	0.0005	0.183	0.0696	9.05	0.203	0.0000	0.00194	8.02	No	
4	0.209	0.0000	0.270	0.3455	44.87	0.315	0.0000	0.01519	62.95	No	9-THC
5	0.814	0.0000	0.832	0.1555	20.19	0.862	0.0128	0.00304	12.59	No	
6	0.862	0.0128	0.892	0.0482	6.27	0.901	0.0156	0.00096	3.96	No	
7	0.916	0.0197	0.922	0.0547	7.11	0.931	0.0000	0.00051	2.10	No	

Track 11:

Type	Sample
Vial ID	s8
Description	[redacted]
Volume	2.0 µl

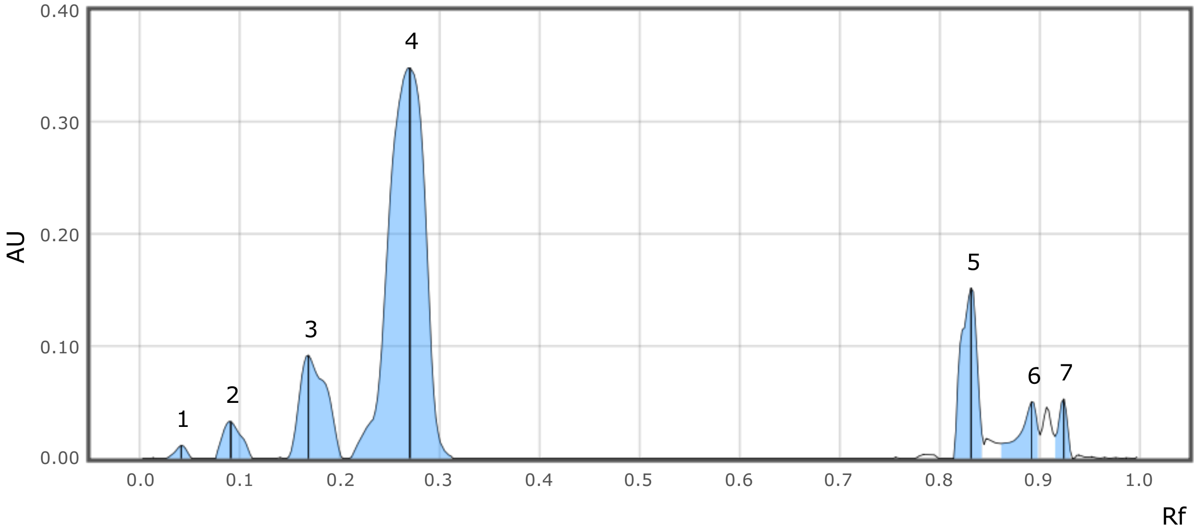

Peak #	Start Rf	Start H	Max Rf	Max H	Max %	End Rf	End H	Area A	Area %	Manual peak	Substance Name
1	0.026	0.0000	0.041	0.0115	1.55	0.052	0.0000	0.00015	0.65	No	
2	0.075	0.0000	0.090	0.0328	4.43	0.112	0.0000	0.00071	3.13	No	
3	0.147	0.0000	0.168	0.0920	12.42	0.203	0.0000	0.00283	12.45	No	
4	0.209	0.0000	0.270	0.3487	47.07	0.313	0.0000	0.01472	64.77	No	9-THC
5	0.814	0.0000	0.832	0.1521	20.53	0.845	0.0125	0.00278	12.22	No	
6	0.862	0.0132	0.892	0.0506	6.83	0.901	0.0209	0.00101	4.45	No	
7	0.916	0.0196	0.925	0.0531	7.17	0.933	0.0000	0.00053	2.34	No	

Track 12:

Type	Sample
Vial ID	s9
Description	[redacted]
Volume	2.0 µl

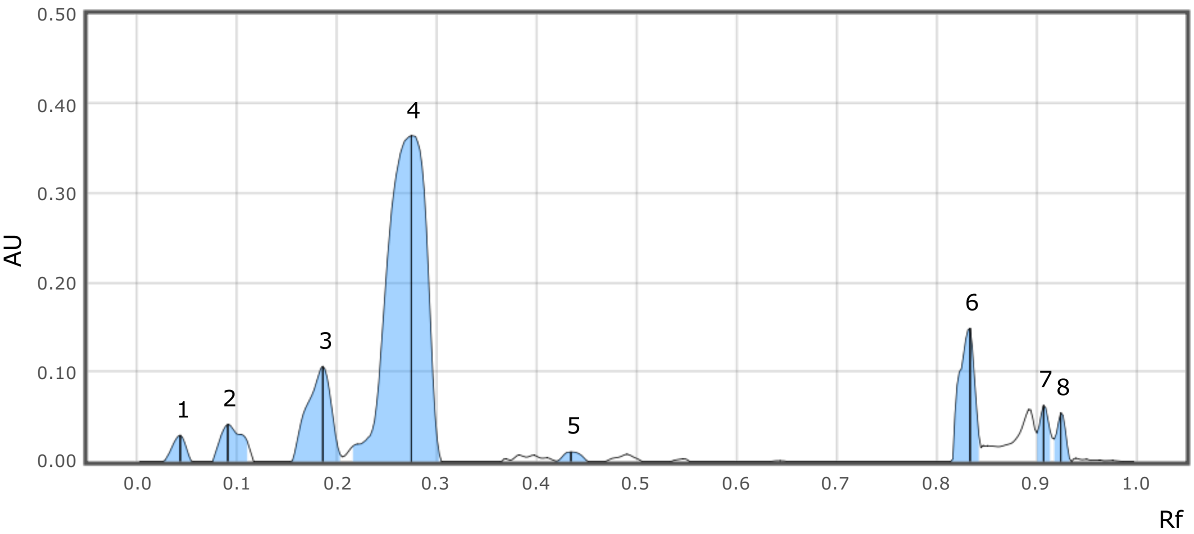

Peak #	Start Rf	Start H	Max Rf	Max H	Max %	End Rf	End H	Area A	Area %	Manual peak	Substance Name
1	0.026	0.0000	0.043	0.0293	3.59	0.056	0.0000	0.00044	1.76	No	
2	0.075	0.0000	0.090	0.0413	5.05	0.116	0.0000	0.00107	4.29	No	
3	0.153	0.0000	0.186	0.1064	13.01	0.205	0.0051	0.00289	11.62	No	
4	0.216	0.0171	0.274	0.3639	44.53	0.304	0.0000	0.01643	65.98	No	9-THC
5	0.419	0.0004	0.434	0.0106	1.29	0.451	0.0000	0.00021	0.84	No	
6	0.814	0.0000	0.834	0.1488	18.20	0.845	0.0153	0.00262	10.52	No	
7	0.901	0.0316	0.907	0.0627	7.67	0.916	0.0264	0.00070	2.82	No	
8	0.918	0.0249	0.925	0.0544	6.66	0.935	0.0000	0.00054	2.18	No	

Track 13:

Type	Sample
Vial ID	s10
Description	[redacted]
Volume	2.0 µl

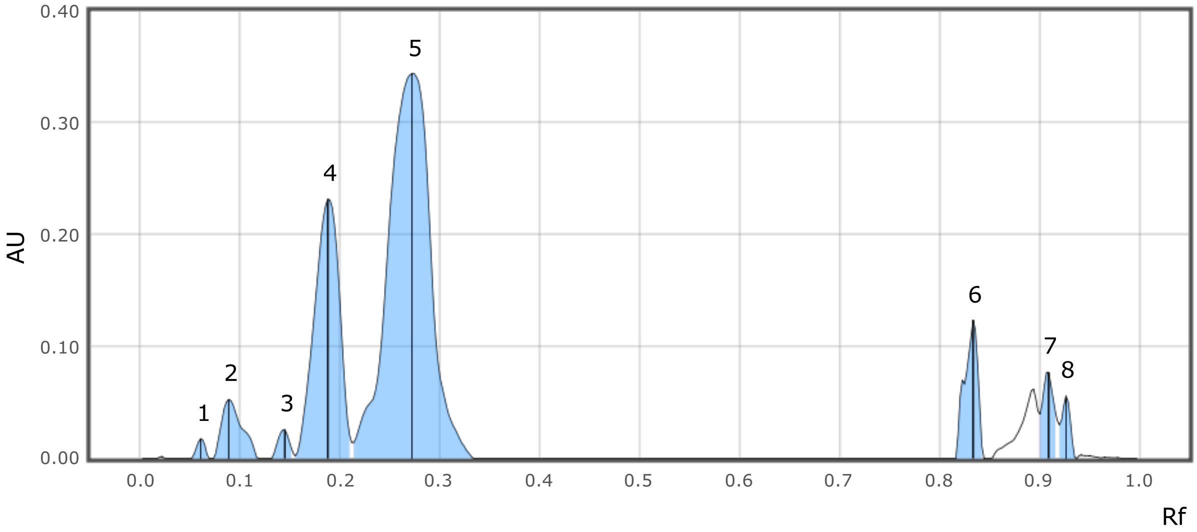

Peak #	Start		Max			End		Area		Manual peak	Substance Name
	Rf	H	Rf	H	%	Rf	H	A	%		
1	0.049	0.0000	0.060	0.0176	1.90	0.069	0.0000	0.00017	0.57	No	
2	0.073	0.0000	0.088	0.0527	5.67	0.119	0.0000	0.00123	4.26	No	
3	0.132	0.0000	0.144	0.0258	2.78	0.153	0.0043	0.00034	1.16	No	
4	0.155	0.0022	0.188	0.2317	24.97	0.211	0.0143	0.00674	23.31	No	
5	0.214	0.0141	0.272	0.3440	37.07	0.335	0.0000	0.01700	58.81	No	9-THC
6	0.817	0.0000	0.834	0.1236	13.32	0.845	0.0000	0.00188	6.51	No	
7	0.901	0.0395	0.910	0.0770	8.30	0.918	0.0338	0.00101	3.49	No	
8	0.920	0.0300	0.927	0.0557	6.00	0.938	0.0000	0.00055	1.89	No	

Track 14:

Type	Reference
Vial ID	250ug/mL mix
Description	250ug/mL
Volume	2.0 µl

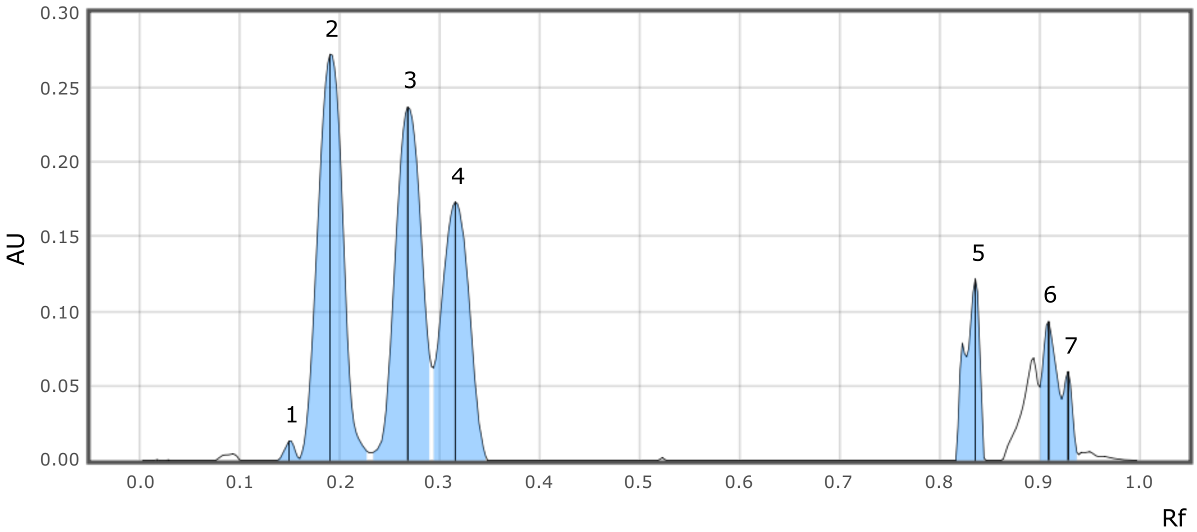

Peak #	Start		Max			End		Area		Manual peak	Substance Name
	Rf	H	Rf	H	%	Rf	H	A	%		
1	0.136	0.0000	0.149	0.0130	1.34	0.160	0.0011	0.00015	0.58	No	
2	0.160	0.0011	0.190	0.2720	28.07	0.229	0.0053	0.00789	31.78	No	CBN
3	0.231	0.0051	0.268	0.2368	24.43	0.291	0.0629	0.00723	29.10	No	9-THC
4	0.294	0.0619	0.315	0.1730	17.86	0.348	0.0000	0.00544	21.92	No	CBD
5	0.817	0.0000	0.836	0.1215	12.54	0.847	0.0000	0.00201	8.09	No	
6	0.901	0.0490	0.910	0.0931	9.61	0.922	0.0409	0.00149	6.01	No	
7	0.922	0.0409	0.929	0.0596	6.15	0.940	0.0038	0.00062	2.51	No	

Track 15:

Type	Sample
Vial ID	MeOH blank
Description	MeOH Blank
Volume	2.0 µl

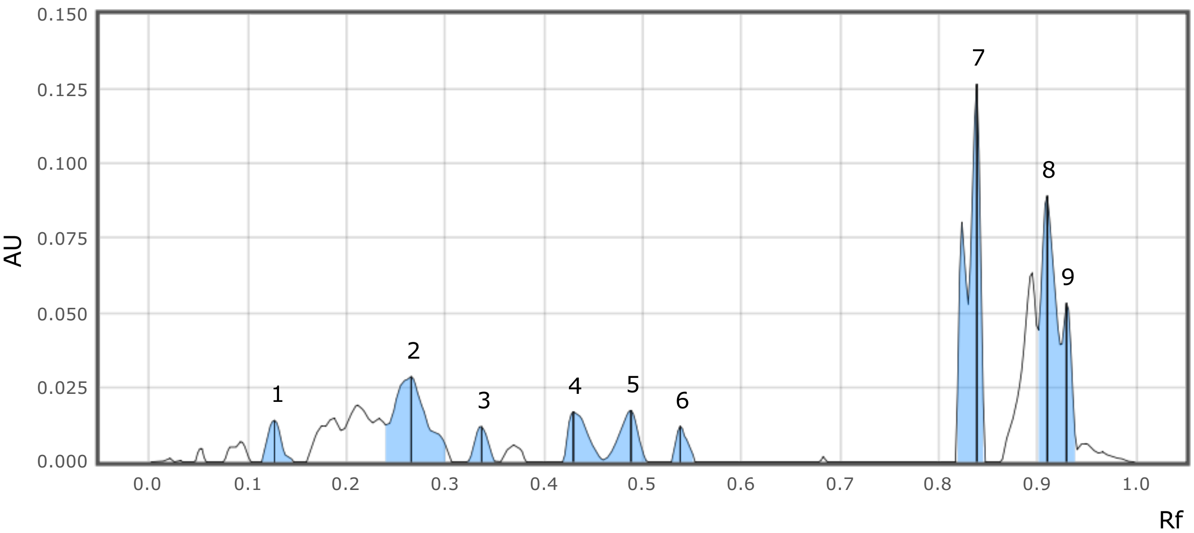

Peak #	Start		Max			End		Area		Manual peak	Substance Name
	Rf	H	Rf	H	%	Rf	H	A	%		
1	0.114	0.0000	0.127	0.0139	3.78	0.147	0.0000	0.00021	3.35	No	
2	0.240	0.0124	0.266	0.0286	7.75	0.307	0.0000	0.00110	17.11	No	
3	0.322	0.0000	0.337	0.0118	3.20	0.352	0.0000	0.00018	2.74	No	
4	0.419	0.0000	0.430	0.0168	4.55	0.460	0.0006	0.00037	5.82	No	
5	0.460	0.0006	0.488	0.0172	4.67	0.505	0.0000	0.00037	5.70	No	
6	0.529	0.0000	0.538	0.0119	3.23	0.553	0.0000	0.00015	2.41	No	
7	0.819	0.0232	0.838	0.1265	34.27	0.847	0.0000	0.00199	31.11	No	
8	0.901	0.0441	0.910	0.0891	24.14	0.922	0.0392	0.00143	22.26	No	
9	0.922	0.0392	0.929	0.0532	14.41	0.940	0.0041	0.00061	9.51	No	

Calibration results:

Height calibration for substance 9-THC @ RT White:

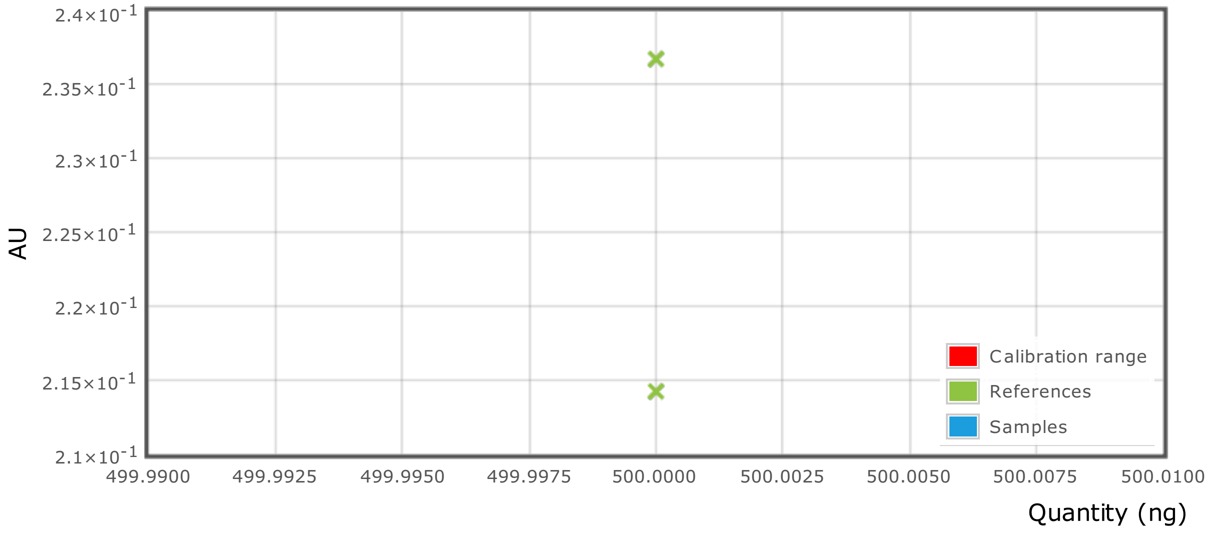

Regression mode	Linear-2
Range deviation	5.00 %
Related substances	Default
Number of references	2
Calibration function	$y=0x$
Coefficient of variation	CV 0.00 %
Correlation coefficient	n/a
	Unable to compute the results for this substance because there wasn't enough groups of references replicas (at least 1 for Linear-1, 2 for Linear2 and Mime-1 and 3 for Polynomial and MiMe-2)

Height calibration for substance CBD @ RT White:

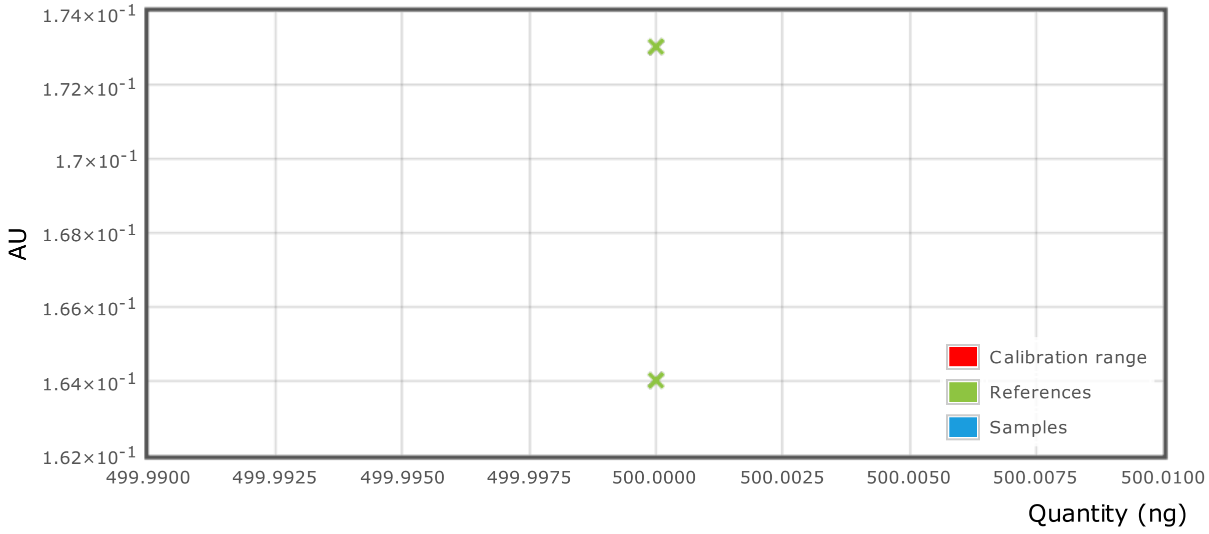

Regression mode	Linear-2
Range deviation	5.00 %
Related substances	Default
Number of references	2
Calibration function	$y=0x$
Coefficient of variation	CV 0.00 %
Correlation coefficient	n/a
	Unable to compute the results for this substance because there wasn't eno ugh groups of references replicas (at least 1 for Linear-1, 2 for Linear2 and Mime-1 and 3 for Polynomial and MiMe-2)

Height calibration for substance CBN @ RT White:

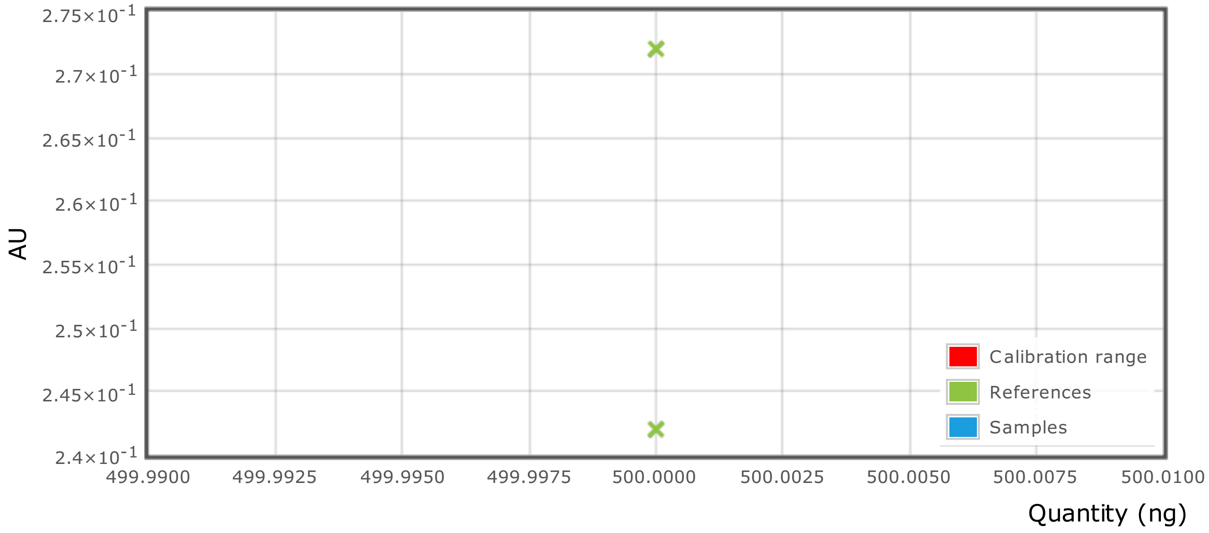

Regression mode	Linear-2
Range deviation	5.00 %
Related substances	Default
Number of references	2
Calibration function	$y=0x$
Coefficient of variation	CV 0.00 %
Correlation coefficient	n/a
	Unable to compute the results for this substance because there wasn't eno ugh groups of references replicas (at least 1 for Linear-1, 2 for Linear2 and Mime-1 and 3 for Polynomial and MiMe-2)

Results:

Substance having no available results		
	CBD	There wasn't any sample application available in the assignments for this substance. Please check that the peaks were correctly detected and assig ned for this substance.
	9-THC	Unable to compute the results for this substance because there wasn't en ough groups of references replicas (at least 1 for Linear-1, 2 for Linear2 a nd Mime-1 and 3 for Polynomial and MiMe-2)
	CBN	There wasn't any sample application available in the assignments for this substance. Please check that the peaks were correctly detected and assig ned for this substance.

A track marked with ⚠ means: this result is outside the regression range given by the reference assignments, but is included in the results because it is in the allowed range deviation.

Analyst:	Reviewer:
