# Supplementary material for: High Performance Thin-Layer Chromatography (HPTLC) data of Cannabinoids in ten mobile phase systems
Source: Data Brief. 2020 Jun 30;31:105955. doi: 10.1016/j.dib.2020.105955 (PMC7352075; doi:10.1016/j.dib.2020.105955)
Supplement: Supplementary file 1 [file mmc1.zip › S4-Case sample reports/XHDa-sample run-8.pdf]

Analysis: XHDa-sample run-8

Path: Home/YL Research

Based on method: Samples (no cal)

Created	15-Oct-2019 12:55:54	visionCATSuser
Modified	15-Oct-2019 15:24:38	visionCATSuser
Last HPTLC log	15-Oct-2019 15:24:38	Analysis modified
Explorer notes		

Track	Vial ID	Description	Volume	Position	Type
1	MeOH blank	MeOH Blank	2.0 µl	A1	Sample
2	250ug/mL mix	250ug/mL	2.0 µl	A2	Reference
3	Tetracosane	Tetracosane IS	2.0 µl	A3	Sample
4	s1	[redacted]	2.0 µl	B1	Sample
5	s2	[redacted]	2.0 µl	B2	Sample
6	s3	[redacted]	2.0 µl	B3	Sample
7	s4	[redacted]	2.0 µl	B4	Sample
8	s5	[redacted]	2.0 µl	B5	Sample
9	s6	[redacted]	2.0 µl	B6	Sample
10	s7	[redacted]	2.0 µl	B7	Sample
11	s8	[redacted]	2.0 µl	B8	Sample
12	s9	[redacted]	2.0 µl	B9	Sample
13	s10	[redacted]	2.0 µl	B10	Sample
14	250ug/mL mix	250ug/mL	2.0 µl	A2	Reference
15	MeOH blank	MeOH Blank	2.0 µl	A1	Sample
Sequence table notes					

A track marked with ⚠ means: the application type is overridden in some evaluation(s).

System setup:

Software	Server User-PC, version 2.5.18072.1
ATS4	S/N:080713
Chamber	N/A
Derivatization dip	N/A
Scanner3	S/N:031025
Visualizer	S/N:230515

Chromatography

Plate layout:

Stationary phase	Merck, HPTLC plates silica gel 60 F 254
Plate format	200.0 x 100.0 mm
Application type	Band
Application	Position Y: 8.0 mm, length: 8.0 mm, width: 0.0 mm
Track	First position X: 20.0 mm, distance: 11.4 mm
Solvent front position	70.0 mm
Notes	

Take image clean plate 1a - Visualizer (S/N: 230515):

Quality	Enhanced
RT White	auto capture, Auto, level 85 %, Band
R 254	auto capture, Auto, level 85 %, Band
Instrument diagnostics	Valid diagnostics
Documentation step label	
Notes	

Application 1 - ATS 4 (S/N: 080713):

Spray gas	NI
Sample solvent type	Methanol
Filling speed	15 µl/s
Predosage volume	200 nl
Retraction volume	200 nl
Dosage speed	150 nl/s
Filling quality	User
Rinsing cycles / vacuum	2 / 4 s
Filling cycles / vacuum	1 / 4 s
Rinsing solvent name	Methanol
Nozzle temperature	Unheated
Rack in use	Standard
Instrument diagnostics	Valid diagnostics
Notes	

Development 1 - Chamber:

Tank	TTC 20x10
Mobile phase	Xylene:hexane:diethylamine (25:10:1)
Saturation time	20 min
Use saturation pad	true
Use smartALERT	false
Volume front through	10 ml
Volume rear through	25 ml
Drying time	5 min
Drying temperature	Room temperature
Notes	

Take image developed plate 1a - Visualizer (S/N: 230515):

Quality	Enhanced
RT White	auto capture, Auto, level 85 %, Band
R 254	auto capture, Auto, level 85 %, Band
R 366	auto capture, Auto, level 85 %, Band
Instrument diagnostics	Valid diagnostics
Documentation step label	
Notes	

Scan developed plate 1b - Scanner 3 (S/N: 031025):

Scanner type	Single λ
Optimization for	Resolution
Measurement mode	Absorption
Filter	n/a
Detector mode	Automatic
Scanning speed	20 mm/s
Data resolution	100 µm/step
Slit	5 x 0.2 mm, micro
Partial scan	No
Lamp	Deuterium & Tungsten
Wavelength(s)	254 nm
Instrument diagnostics	Valid diagnostics
Documentation step label	
Notes	

Derivatization 1 - dip:

Reagent name	
Dipping speed	5
Dipping time	0 s
Reagent preparation	
Heating	100 °C for 3 min, heated after
Notes	

Take image derivatized plate 1a - Visualizer (S/N: 230515):

Quality	Enhanced
RT White	auto capture, Auto, level 85 %, Band
R 366	auto capture, Auto, level 85 %, Band
Instrument diagnostics	Valid diagnostics
Documentation step label	
Notes	

System suitability tests:

SST settings:

SST tracks	

Data acquisition

Application 1 - ATS 4 (S/N: 080713):

Executed	15-Oct-2019 13:15:59 visionCATSuser

Development 1 - Chamber:

Executed	15-Oct-2019 14:09:25 visionCATSuser

Take image developed plate 1a - Visualizer (S/N: 230515):

Executed	15-Oct-2019 14:51:44 visionCATSuser

RT White Developed, RemTransVis

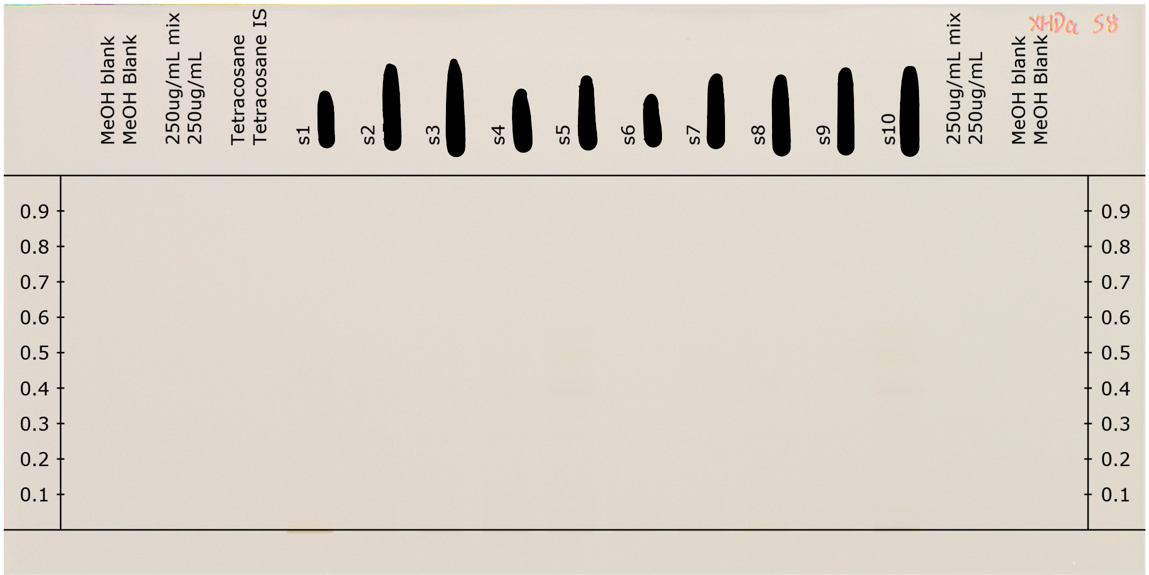

Exposure	0.080 s
Contrast	1
Normalized exposure	Disabled
Clarify	Disabled
White balance	1.00, 1.00, 1.00

R 254 Developed, Remission254

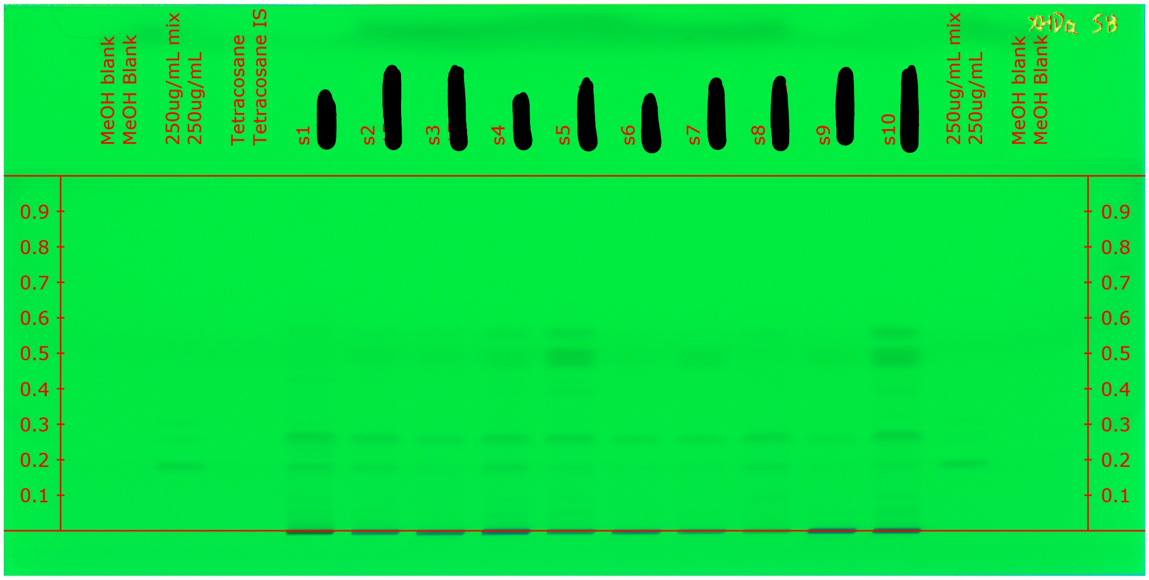

Exposure	0.271 s
Contrast	1
Normalized exposure	Disabled
Clarify	Disabled
White balance	1.00, 1.00, 1.00

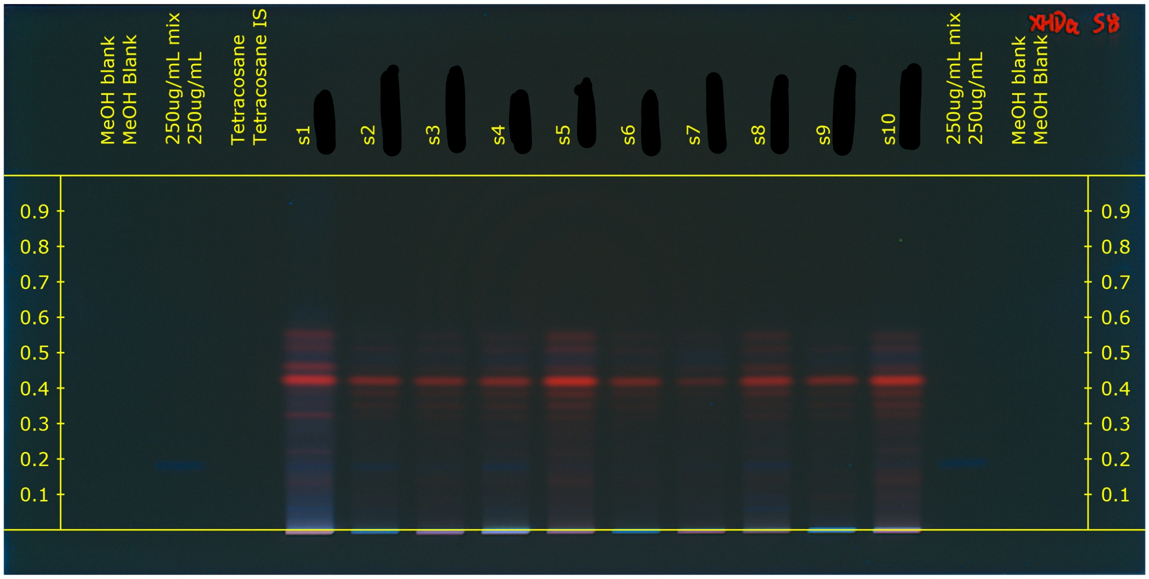

Exposure	2.081 s
Contrast	1
Normalized exposure	Disabled
Clarify	Disabled
White balance	1.00, 1.00, 1.00

Scan developed plate 1b - Scanner 3 (S/N: 031025):

Executed	15-Oct-2019 15:01:18 visionCATSuser

Scan:

Wavelength	254 nm

Track 1:

Type	Single λ

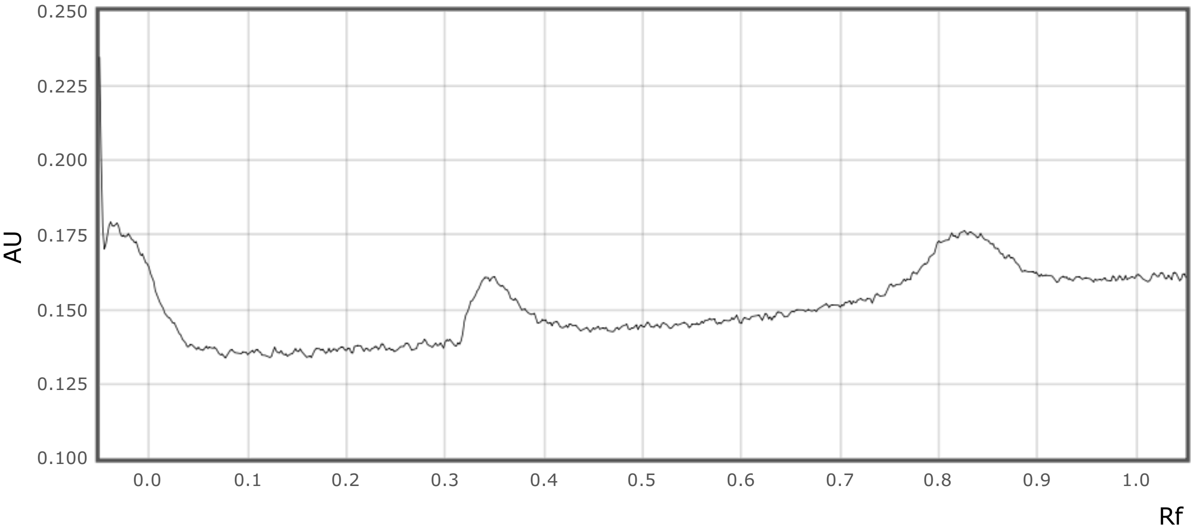

Track 2:

Type	Single λ

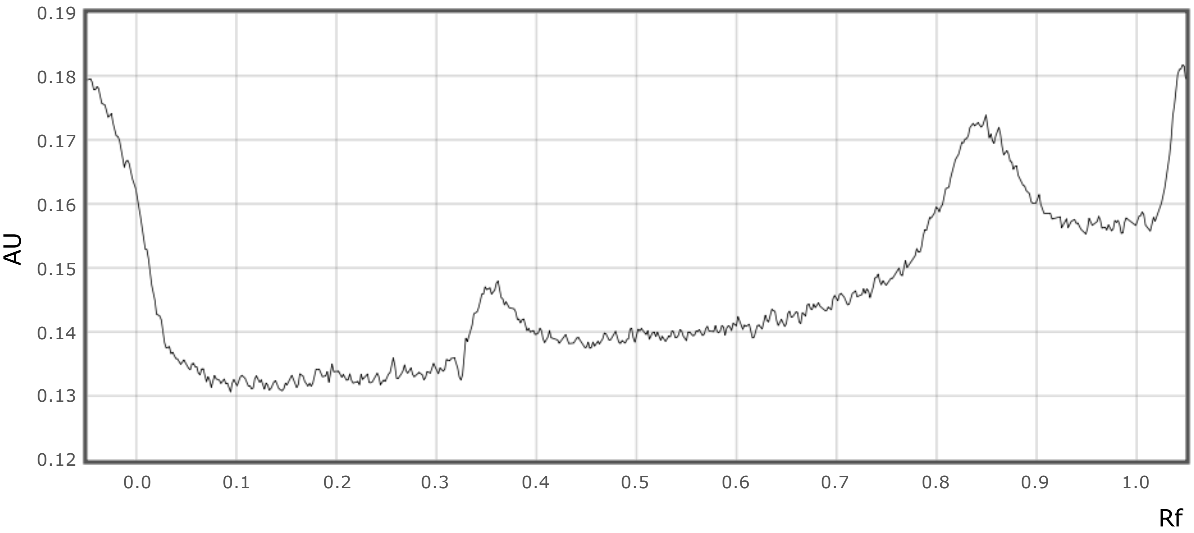

Track 3:

Type	Single λ

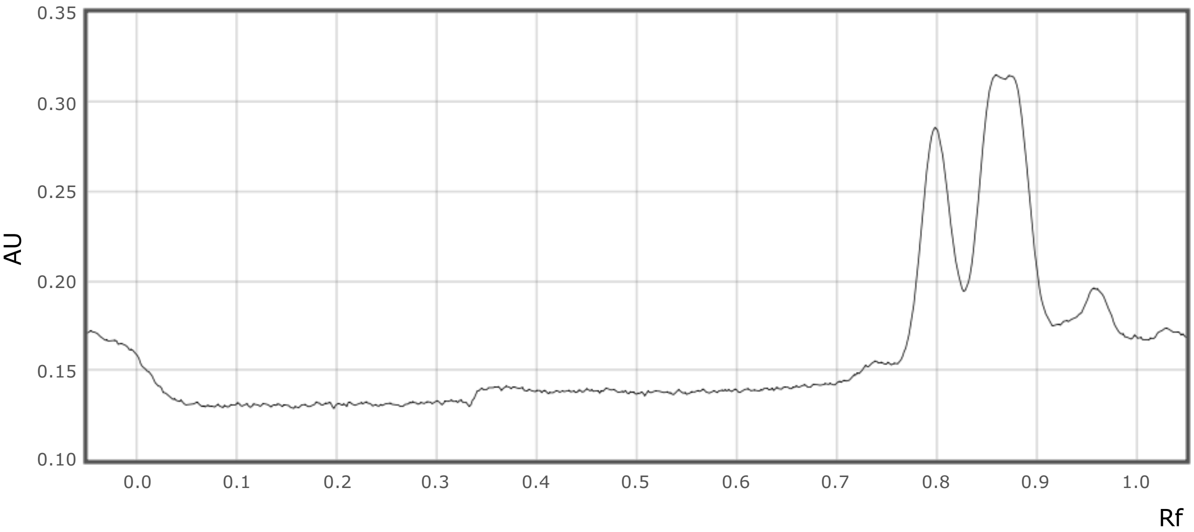

Track 4:

Type	Single λ

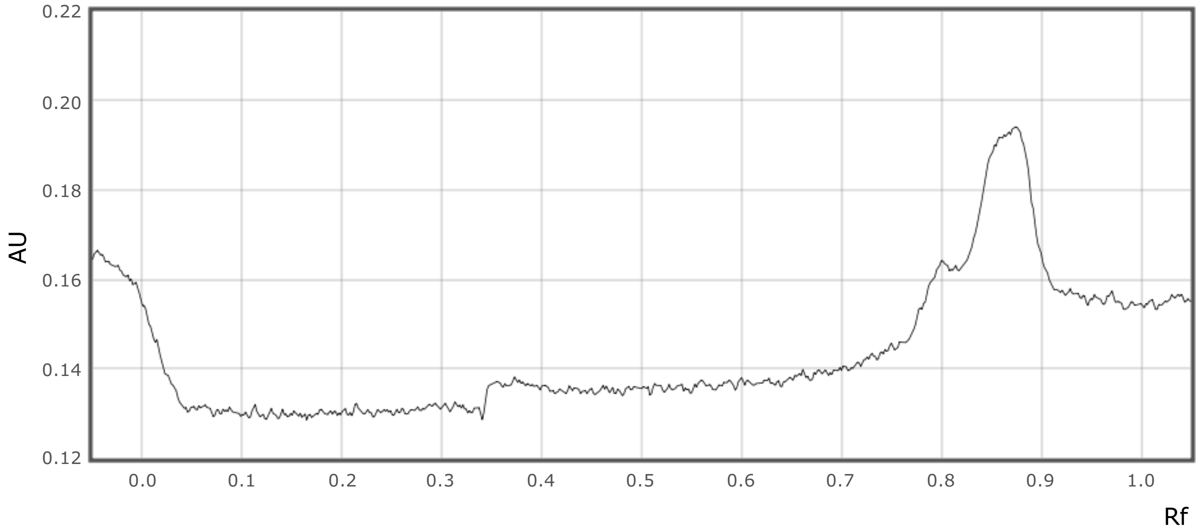

Track 5:	
Type	Single λ

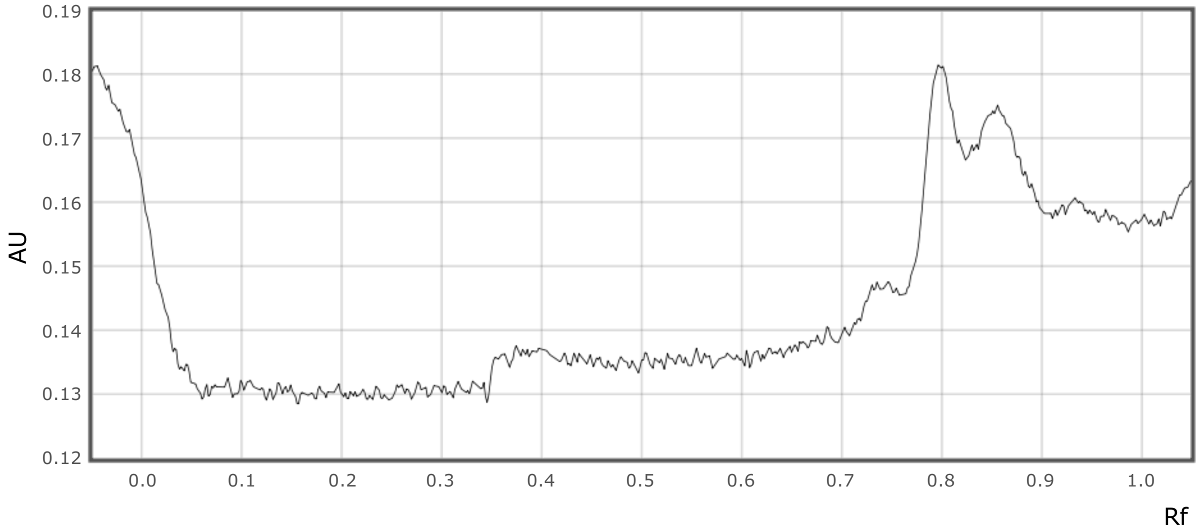

Track 6:	
Type	Single λ

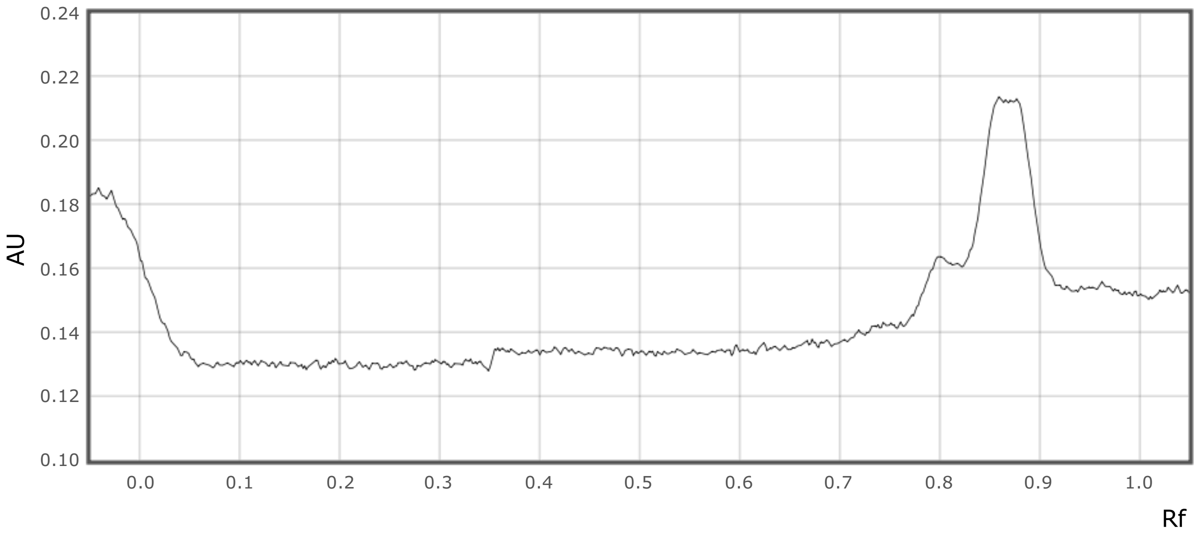

Track 7:

Type	Single λ

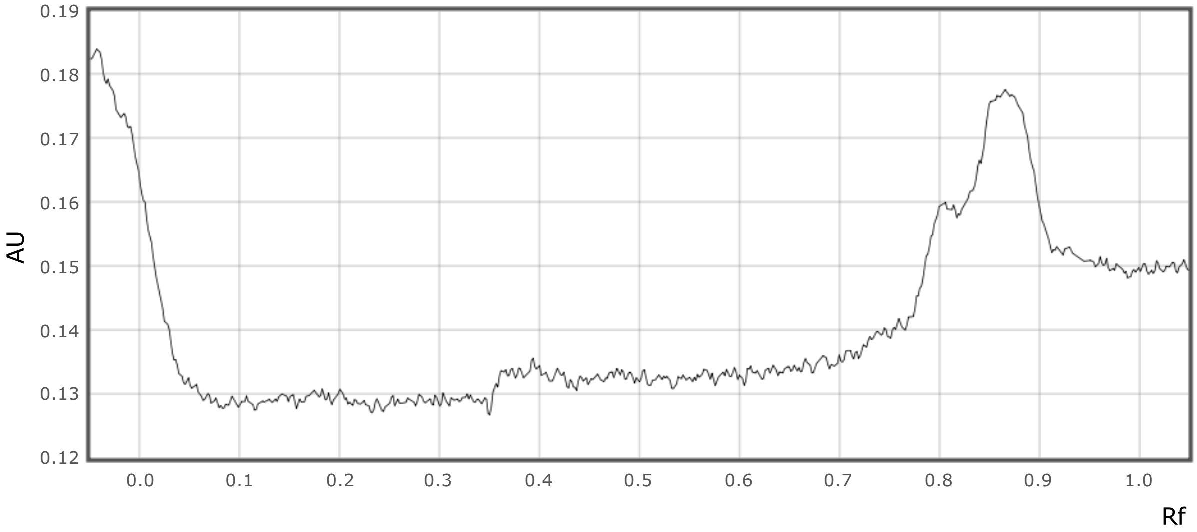

Track 8:

Type	Single λ

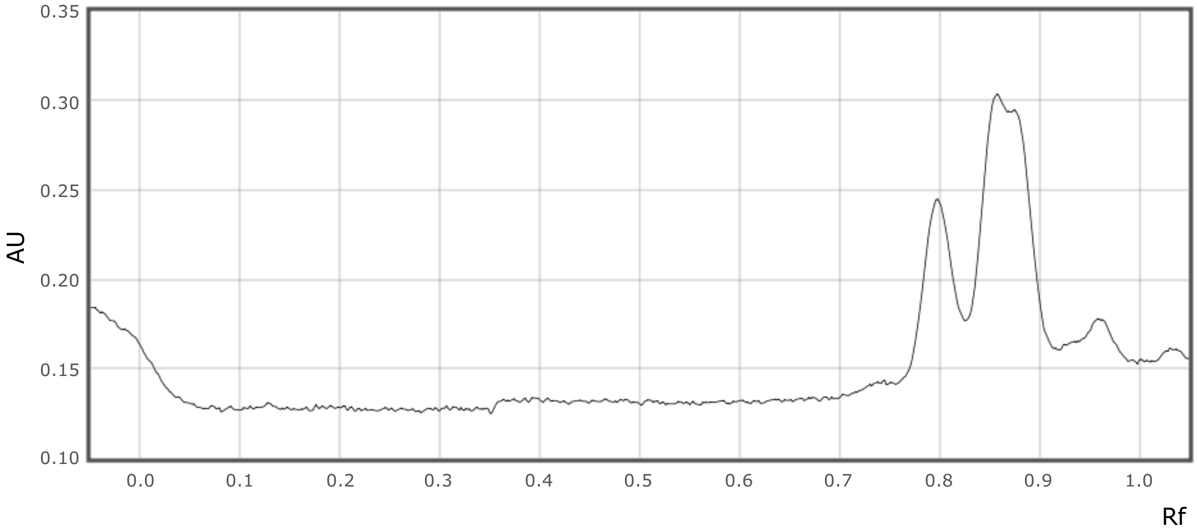

Track 9:	
Type	Single λ

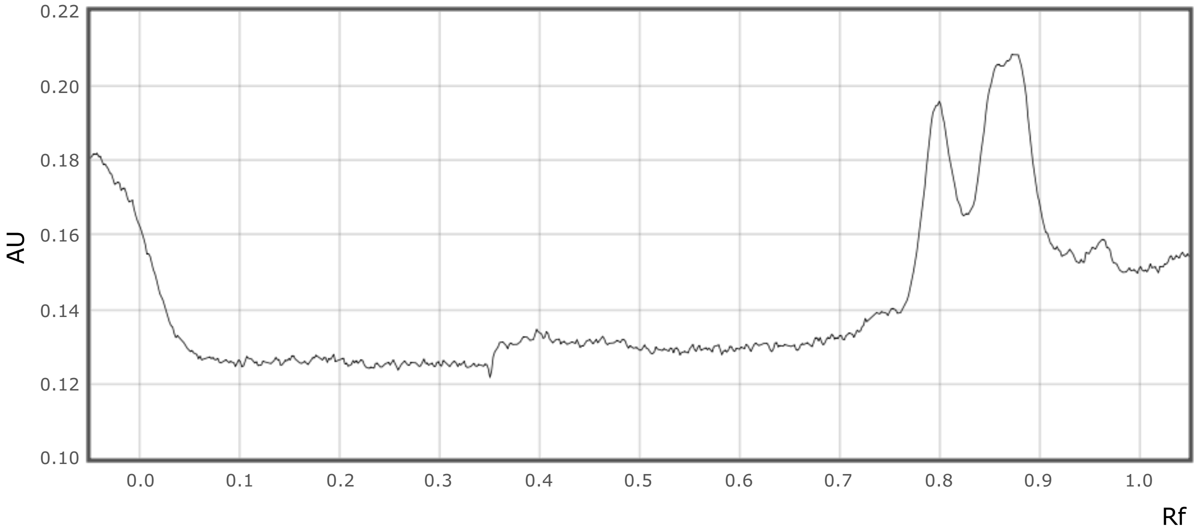

Track 10:	
Type	Single λ

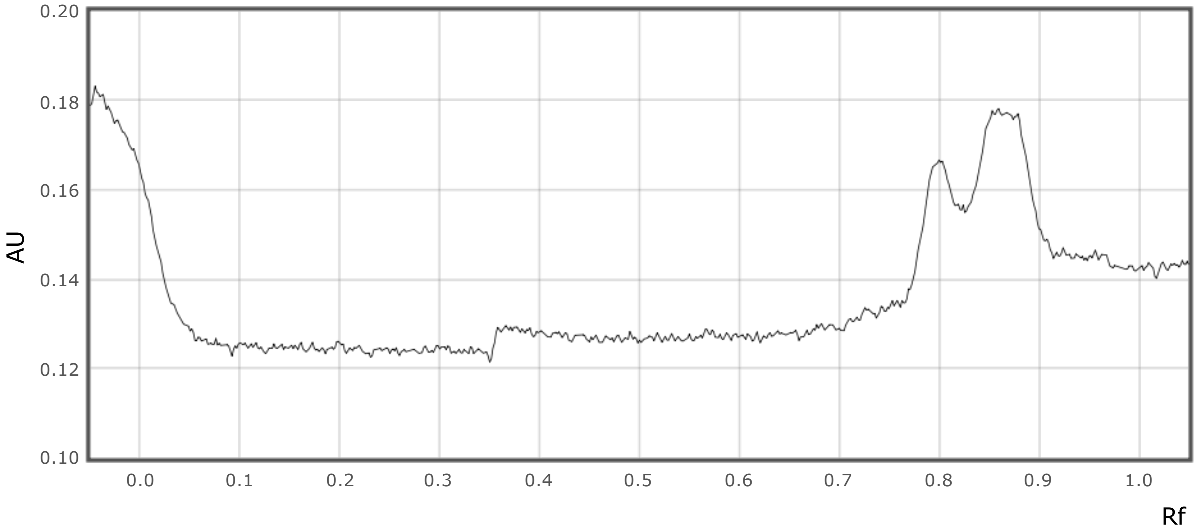

Track 11:

Type	Single λ

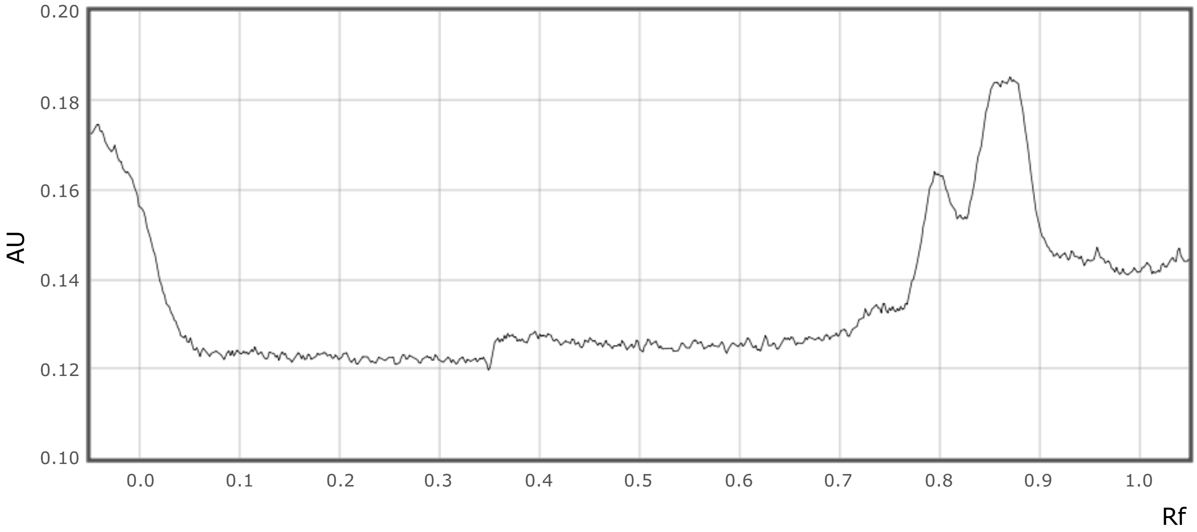

Track 12:

Type	Single λ

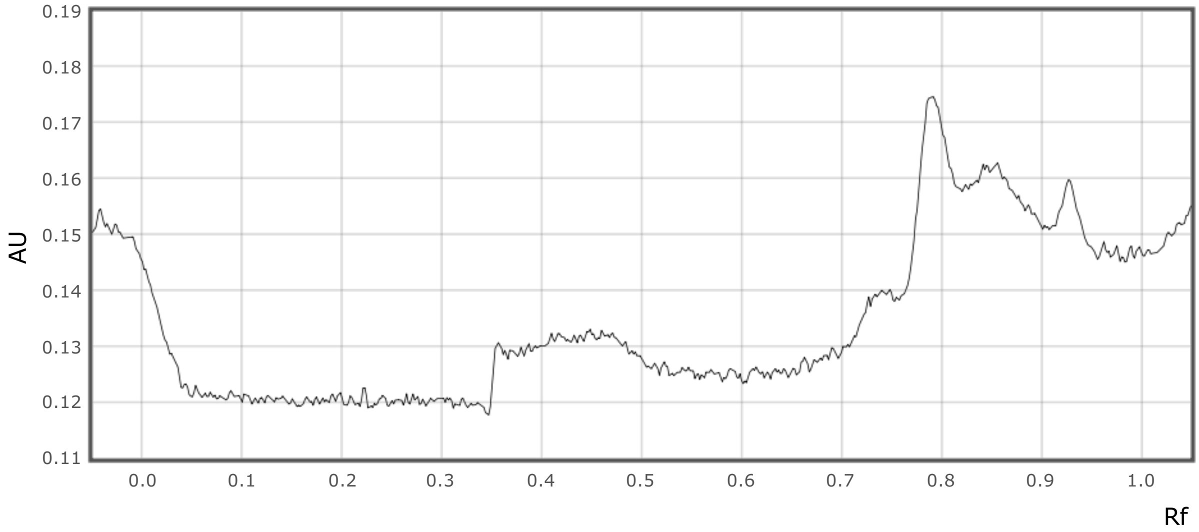

Track 13:	
Type	Single λ

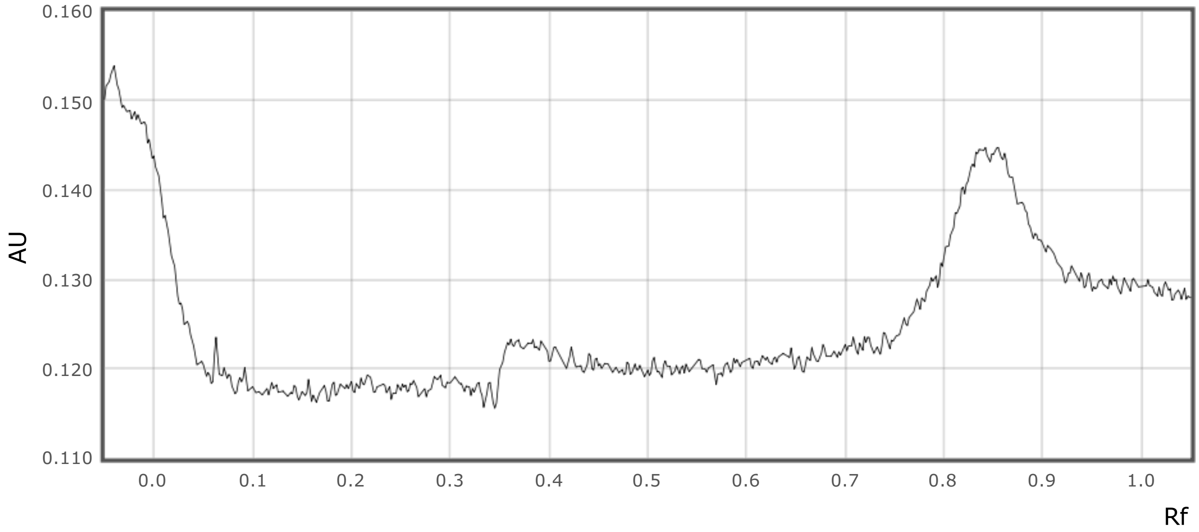

Track 14:	
Type	Single λ

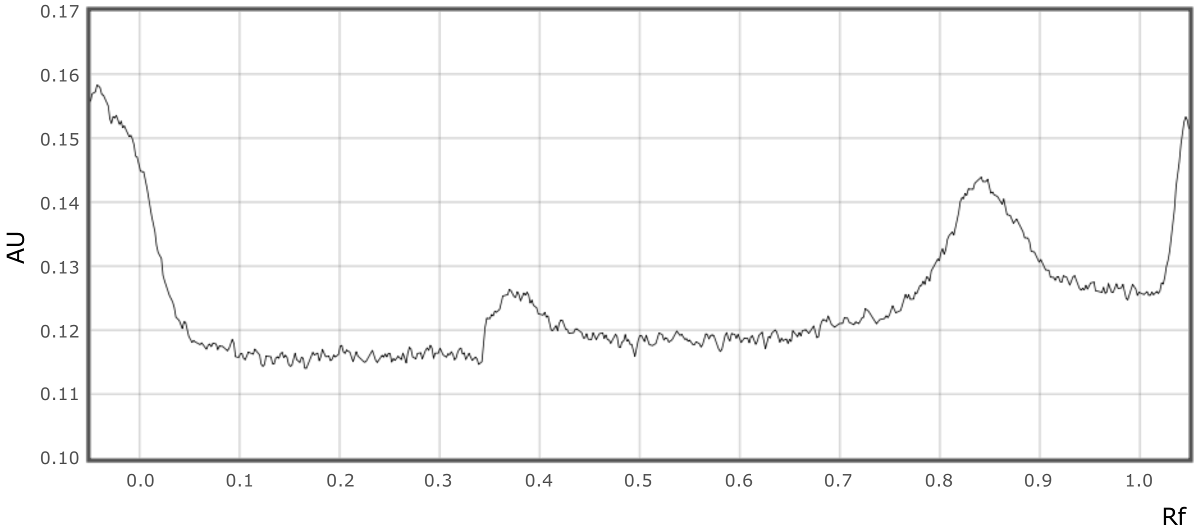

Track 15:

Type	Single λ

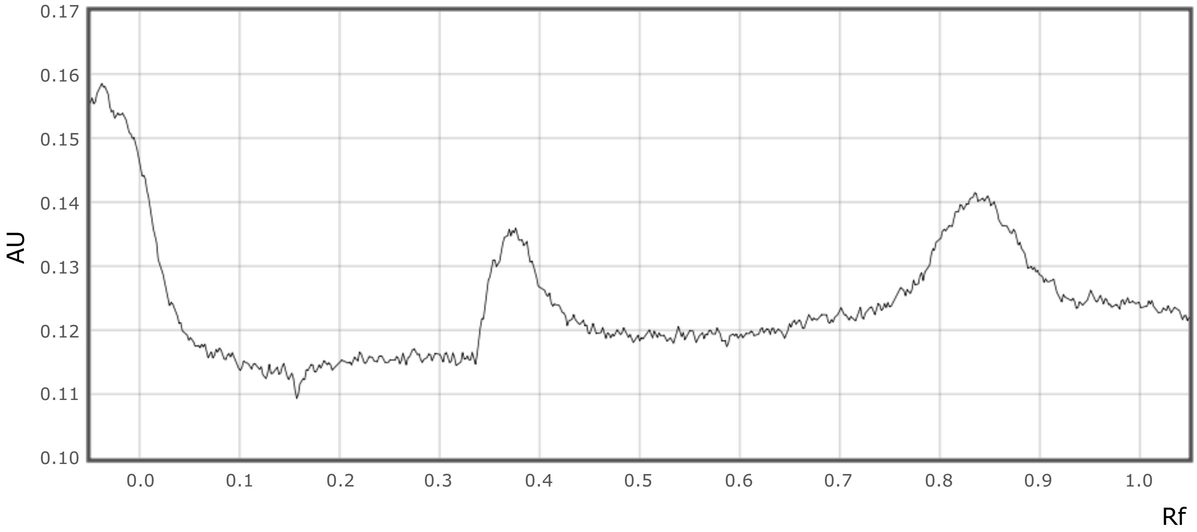

Derivatization 1 - dip:

Executed	15-Oct-2019 15:04:39 visionCATSuser

Take image derivatized plate 1a - Visualizer (S/N: 230515):

Executed	15-Oct-2019 15:12:32 visionCATSuser

RT White

Derivatized, RemTransVis

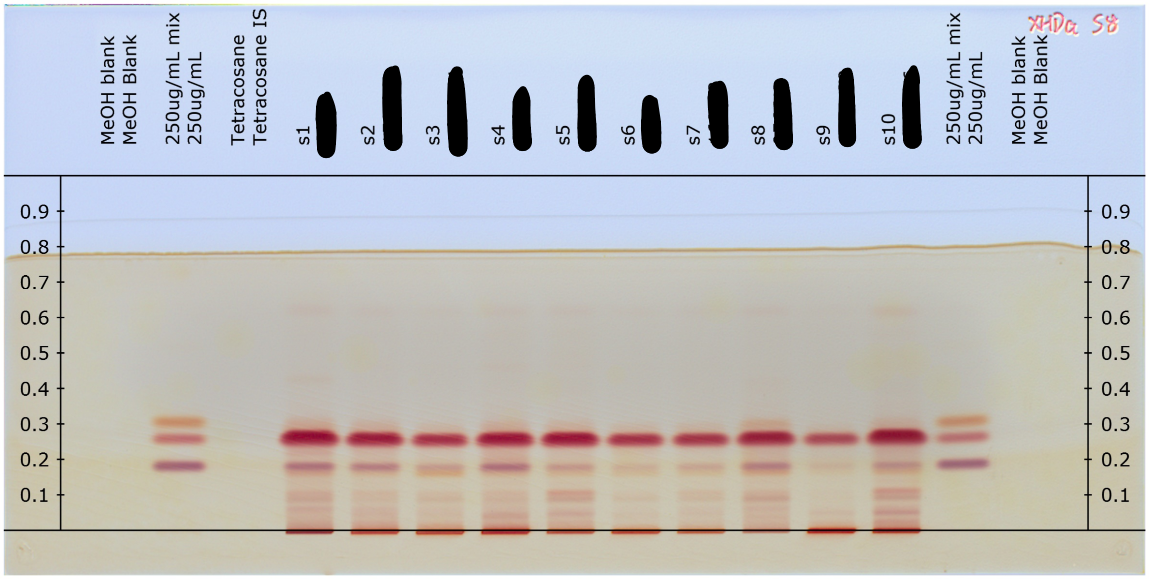

Exposure	0.074 s
Contrast	1
Normalized exposure	Disabled
Clarify	Disabled
White balance	1.25, 1.08, 0.78

R 366

Derivatized, Remission366

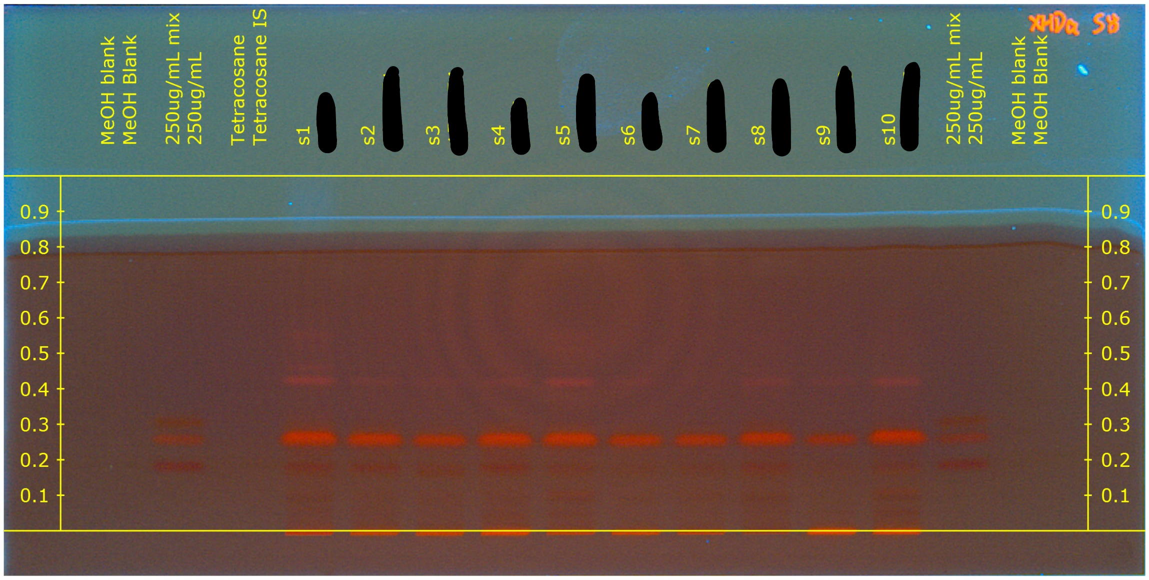

Exposure	9.999 s
Contrast	1
Normalized exposure	Disabled
Clarify	Disabled
White balance	1.00, 1.00, 1.00

Evaluation 1 :

Validated	false
Step	Take image derivatized plate 1a
Concentration unit type	Mass / volume
Notes	

Definition:

References:

250ug/mL mix

Substance Name	Concentration	Purity
9-THC	250.000 µg/ml	100.00 %
CBD	250.000 µg/ml	100.00 %
CBN	250.000 µg/ml	100.00 %

Samples:

Vial ID	Amount	Volume solution	Reference amount	Related to
MeOH blank		0.00 ml		
Tetracosane		0.00 ml		
s1		0.00 ml		
s2		0.00 ml		
s3		0.00 ml		
s4		0.00 ml		
s5		0.00 ml		
s6		0.00 ml		
s7		0.00 ml		
s8		0.00 ml		
s9		0.00 ml		
s10		0.00 ml		

Integration parameters:

Bounds	[0.000,1.000]
Smoothing	Savitzky–Golay of order 3 and window 7
Baseline correction	Lowest slope with noise 0.05
Profile subtraction	Profile subtraction from track 1
Peaks detection	Gauss (legacy) with sensitivity 0.1, separation 1 and threshold 0.1

Scan:

Wavelength	RT White

Track 1:

Type	Sample
Vial ID	MeOH blank
Description	MeOH Blank
Volume	2.0 µl

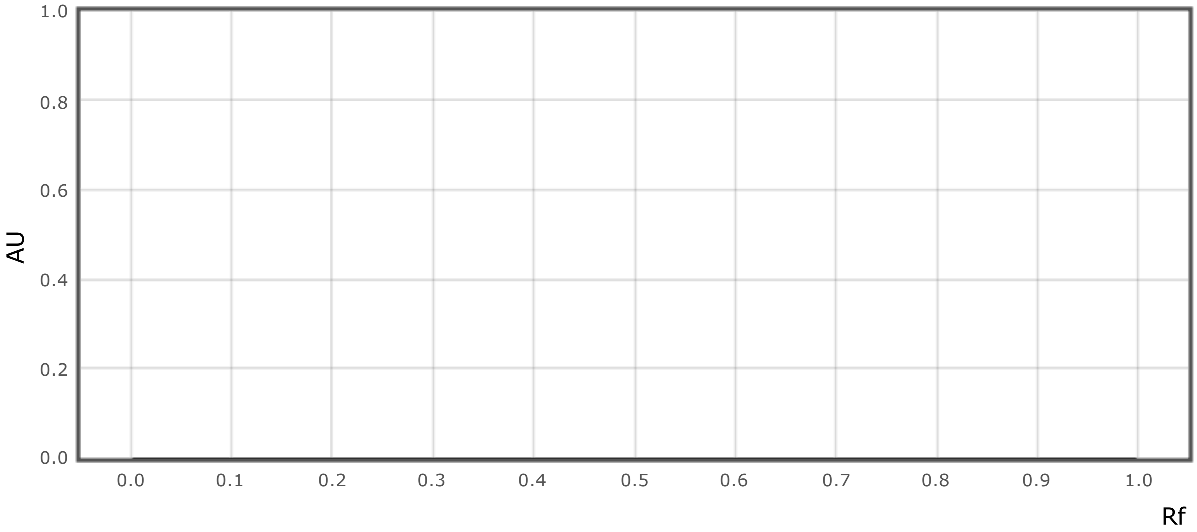

Peak #	Start		Max			End		Area		Manual peak	Substance Name
	Rf	H	Rf	H	%	Rf	H	A	%		

Track 2:

Type	Reference
Vial ID	250ug/mL mix
Description	250ug/mL
Volume	2.0 µl

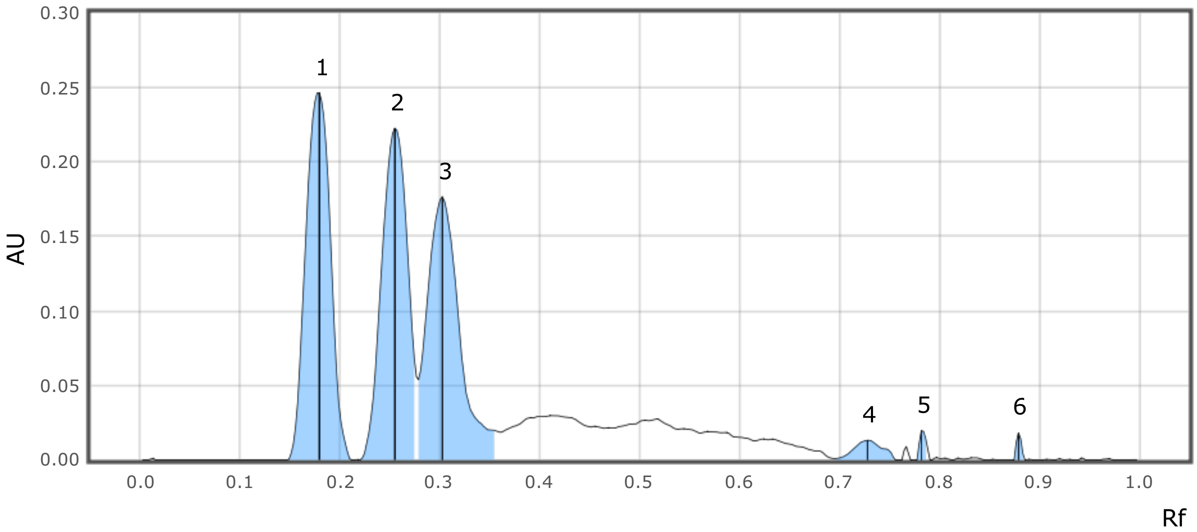

Peak #	Start		Max			End		Area		Manual peak	Substance Name
	Rf	H	Rf	H	%	Rf	H	A	%		
1	0.147	0.0000	0.179	0.2462	35.40	0.211	0.0000	0.00706	33.57	No	CBN
2	0.220	0.0000	0.255	0.2224	31.97	0.276	0.0558	0.00665	31.61	No	9-THC
3	0.278	0.0538	0.302	0.1764	25.35	0.358	0.0188	0.00663	31.51	No	CBD
4	0.696	0.0008	0.728	0.0131	1.89	0.756	0.0000	0.00044	2.09	No	
5	0.778	0.0000	0.782	0.0195	2.81	0.791	0.0000	0.00015	0.73	No	
6	0.875	0.0000	0.879	0.0179	2.58	0.886	0.0000	0.00010	0.50	No	

Track 3:

Type	Sample
Vial ID	Tetracosane
Description	Tetracosane IS
Volume	2.0 µl

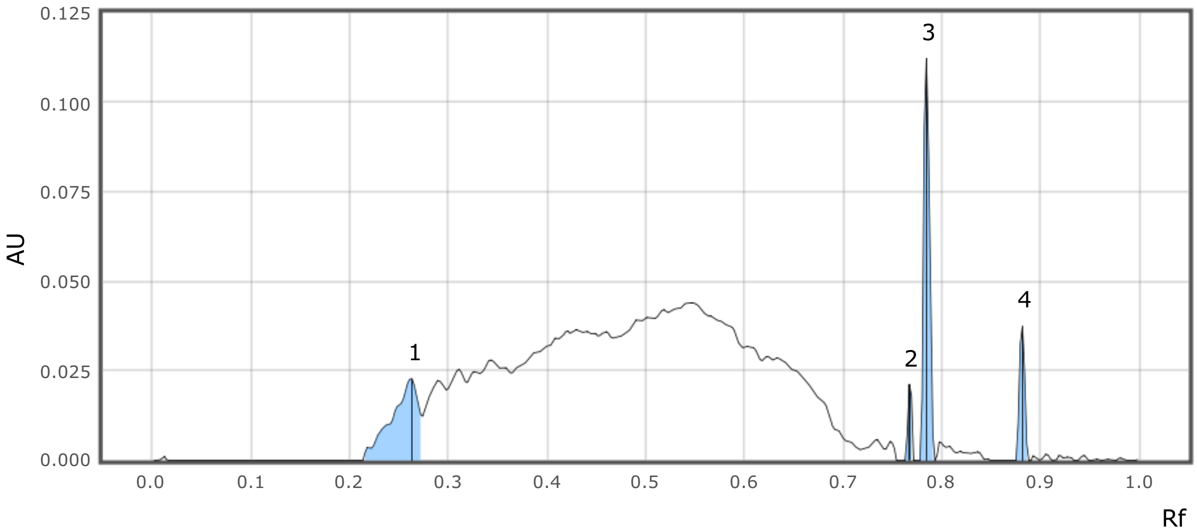

Peak #	Start Rf	Start H	Max Rf	Max H	Max %	End Rf	End H	Area A	Area %	Manual peak	Substance Name
1	0.216	0.0022	0.263	0.0227	11.74	0.274	0.0123	0.00072	39.07	No	
2	0.763	0.0000	0.767	0.0212	10.97	0.771	0.0000	0.00010	5.43	No	
3	0.778	0.0000	0.784	0.1121	57.97	0.793	0.0000	0.00079	42.73	No	
4	0.875	0.0000	0.881	0.0374	19.32	0.888	0.0000	0.00023	12.77	No	

Track 4:

Type	Sample
Vial ID	s1
Description	[redacted]
Volume	2.0 µl

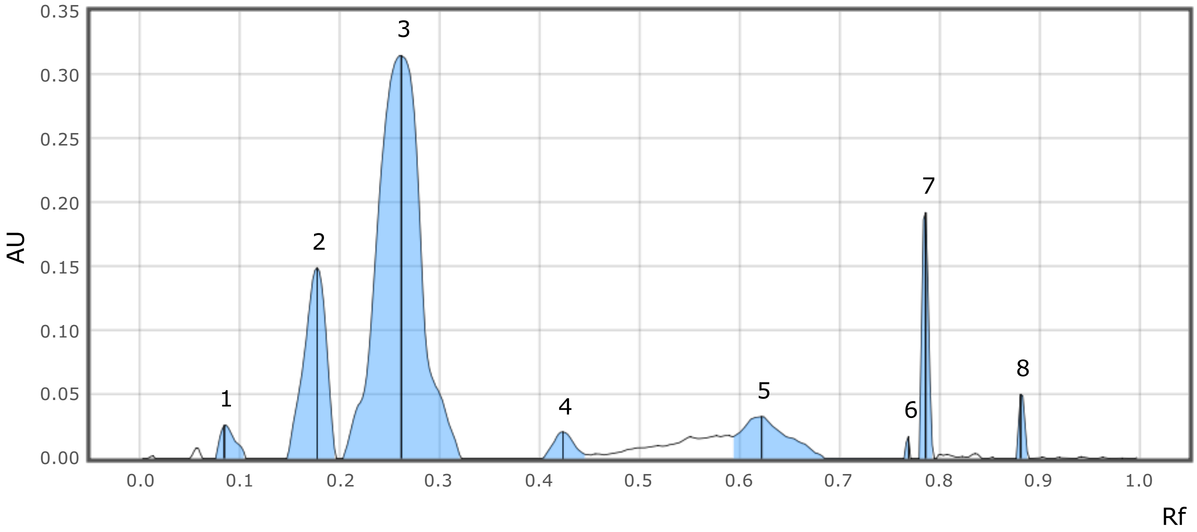

Peak #	Start		Max			End		Area		Manual peak	Substance Name
	Rf	H	Rf	H	%	Rf	H	A	%		
1	0.075	0.0000	0.084	0.0258	3.22	0.106	0.0000	0.00045	1.84	No	
2	0.147	0.0000	0.177	0.1493	18.59	0.196	0.0000	0.00382	15.67	No	
3	0.203	0.0000	0.261	0.3151	39.23	0.322	0.0000	0.01601	65.63	No	9-THC
4	0.402	0.0000	0.423	0.0206	2.57	0.451	0.0026	0.00051	2.09	No	
5	0.594	0.0170	0.622	0.0326	4.06	0.687	0.0000	0.00172	7.06	No	
6	0.765	0.0000	0.769	0.0171	2.12	0.771	0.0000	0.00006	0.26	No	
7	0.780	0.0000	0.786	0.1924	23.95	0.795	0.0000	0.00148	6.07	No	
8	0.877	0.0000	0.881	0.0502	6.26	0.890	0.0000	0.00034	1.39	No	

Track 5:

Type	Sample
Vial ID	s2
Description	[redacted]
Volume	2.0 µl

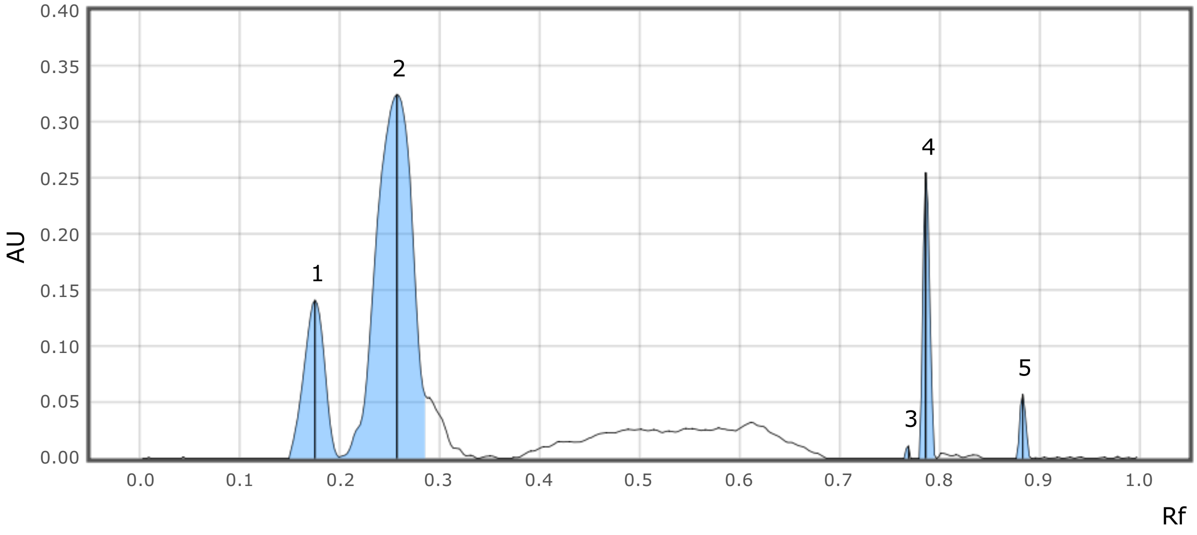

Peak #	Start Rf	Start H	Max Rf	Max H	Max %	End Rf	End H	Area A	Area %	Manual peak	Substance Name
1	0.149	0.0000	0.175	0.1413	17.89	0.199	0.0011	0.00336	17.30	No	
2	0.199	0.0011	0.257	0.3252	41.17	0.287	0.0539	0.01365	70.31	No	9-THC
3	0.765	0.0000	0.769	0.0110	1.40	0.771	0.0000	0.00004	0.22	No	
4	0.780	0.0000	0.786	0.2553	32.32	0.797	0.0000	0.00197	10.16	No	
5	0.877	0.0000	0.884	0.0571	7.23	0.892	0.0000	0.00039	2.01	No	

Track 6:

Type	Sample
Vial ID	s3
Description	[redacted]
Volume	2.0 µl

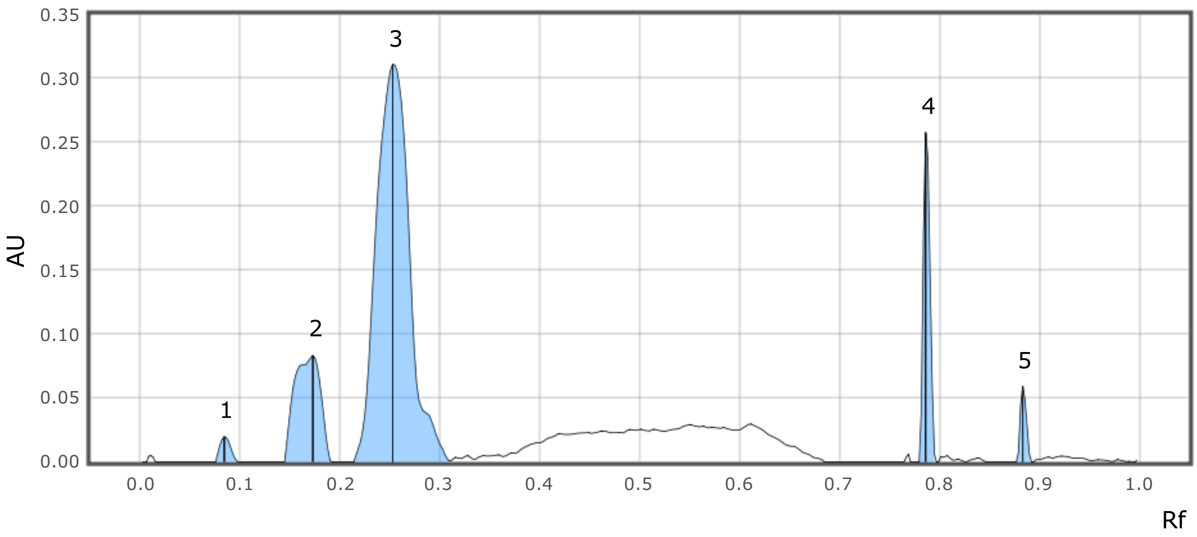

Peak #	Start Rf	Start H	Max Rf	Max H	Max %	End Rf	End H	Area A	Area %	Manual peak	Substance Name
1	0.075	0.0000	0.084	0.0197	2.69	0.099	0.0000	0.00024	1.41	No	
2	0.142	0.0000	0.173	0.0833	11.40	0.192	0.0000	0.00256	14.94	No	
3	0.214	0.0000	0.253	0.3110	42.55	0.311	0.0009	0.01190	69.57	No	9-THC
4	0.780	0.0000	0.786	0.2577	35.26	0.797	0.0000	0.00199	11.64	No	
5	0.877	0.0000	0.884	0.0591	8.09	0.892	0.0000	0.00042	2.44	No	

Track 7:

Type	Sample
Vial ID	s4
Description	[redacted]
Volume	2.0 µl

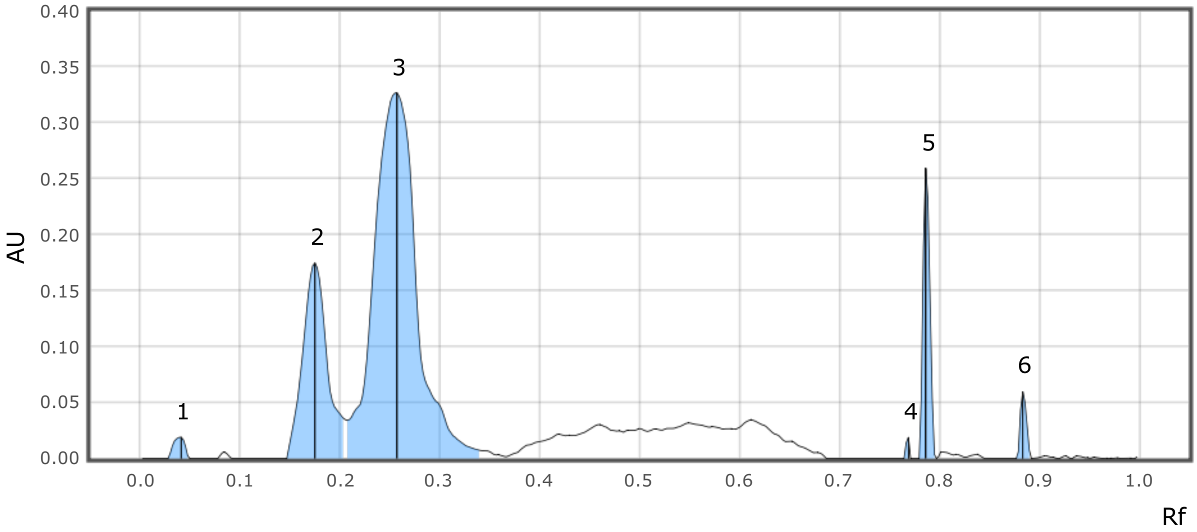

Peak #	Start Rf	Start H	Max Rf	Max H	Max %	End Rf	End H	Area A	Area %	Manual peak	Substance Name
1	0.028	0.0000	0.041	0.0189	2.21	0.049	0.0000	0.00025	1.06	No	
2	0.147	0.0000	0.175	0.1746	20.35	0.205	0.0348	0.00500	20.95	No	
3	0.207	0.0340	0.257	0.3267	38.07	0.343	0.0067	0.01611	67.53	No	9-THC
4	0.765	0.0000	0.769	0.0188	2.19	0.771	0.0000	0.00007	0.30	No	
5	0.780	0.0000	0.786	0.2594	30.24	0.797	0.0000	0.00200	8.40	No	
6	0.877	0.0000	0.884	0.0595	6.94	0.892	0.0000	0.00042	1.77	No	

Track 8:

Type	Sample
Vial ID	s5
Description	[redacted]
Volume	2.0 µl

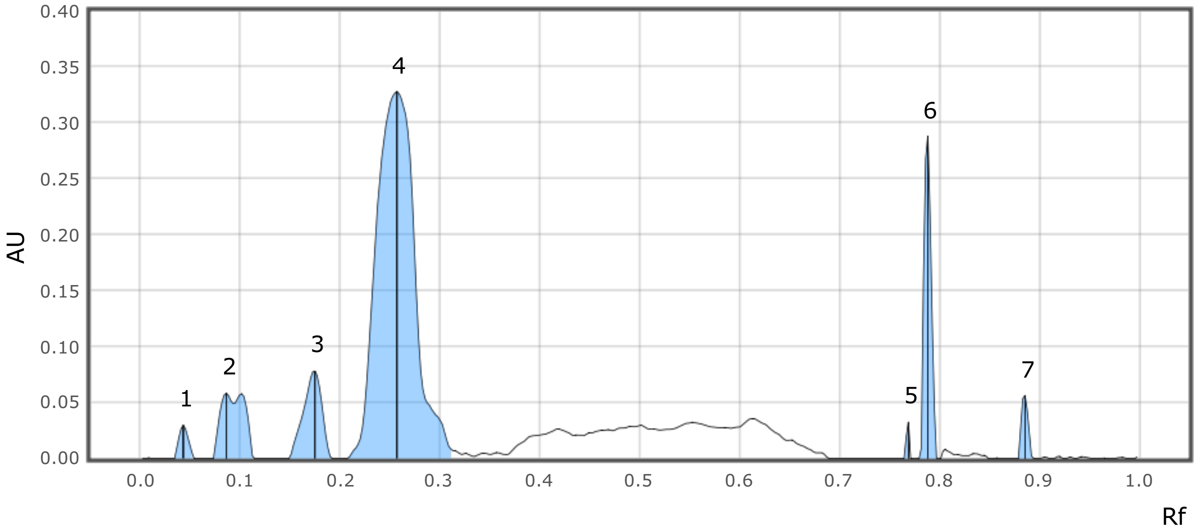

Peak #	Start Rf	Start H	Max Rf	Max H	Max %	End Rf	End H	Area A	Area %	Manual peak	Substance Name
1	0.034	0.0000	0.043	0.0296	3.40	0.054	0.0000	0.00032	1.49	No	
2	0.073	0.0000	0.086	0.0582	6.68	0.114	0.0000	0.00169	7.94	No	
3	0.149	0.0000	0.175	0.0781	8.97	0.192	0.0000	0.00166	7.78	No	
4	0.207	0.0000	0.257	0.3279	37.65	0.313	0.0070	0.01469	68.94	No	9-THC
5	0.765	0.0000	0.769	0.0325	3.73	0.771	0.0000	0.00011	0.52	No	
6	0.780	0.0000	0.789	0.2882	33.09	0.799	0.0000	0.00240	11.26	No	
7	0.879	0.0000	0.886	0.0563	6.47	0.894	0.0000	0.00044	2.07	No	

Track 9:

Type	Sample
Vial ID	s6
Description	[redacted]
Volume	2.0 µl

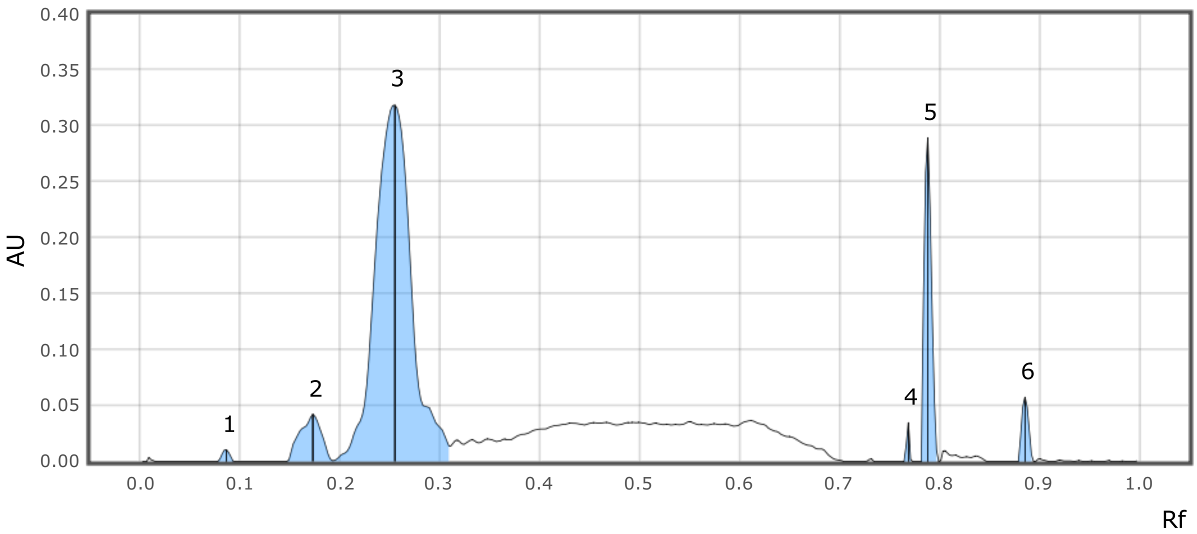

Peak #	Start Rf	Start H	Max Rf	Max H	Max %	End Rf	End H	Area A	Area %	Manual peak	Substance Name
1	0.078	0.0000	0.086	0.0104	1.38	0.095	0.0000	0.00009	0.51	No	
2	0.147	0.0000	0.173	0.0421	5.60	0.192	0.0006	0.00104	5.92	No	
3	0.192	0.0006	0.255	0.3184	42.32	0.311	0.0139	0.01341	76.57	No	9-THC
4	0.765	0.0000	0.769	0.0347	4.61	0.773	0.0000	0.00011	0.63	No	
5	0.782	0.0000	0.789	0.2894	38.46	0.799	0.0000	0.00241	13.78	No	
6	0.879	0.0000	0.886	0.0574	7.63	0.894	0.0000	0.00045	2.59	No	

Track 10:

Type	Sample
Vial ID	s7
Description	[redacted]
Volume	2.0 µl

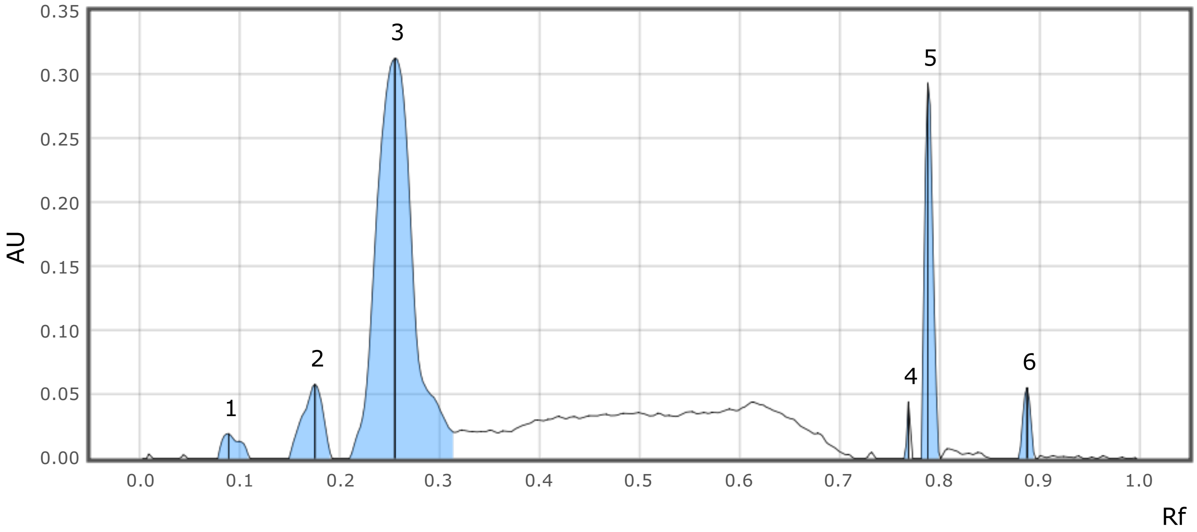

Peak #	Start		Max			End		Area		Manual peak	Substance Name
	Rf	H	Rf	H	%	Rf	H	A	%		
1	0.078	0.0000	0.088	0.0194	2.47	0.110	0.0000	0.00040	2.18	No	
2	0.149	0.0000	0.175	0.0579	7.39	0.192	0.0000	0.00136	7.33	No	
3	0.209	0.0000	0.255	0.3128	39.93	0.315	0.0204	0.01338	72.05	No	9-THC
4	0.765	0.0000	0.769	0.0443	5.66	0.773	0.0000	0.00017	0.90	No	
5	0.782	0.0000	0.789	0.2936	37.48	0.801	0.0000	0.00278	14.96	No	
6	0.879	0.0000	0.888	0.0555	7.08	0.897	0.0000	0.00048	2.58	No	

Track 11:

Type	Sample
Vial ID	s8
Description	[redacted]
Volume	2.0 µl

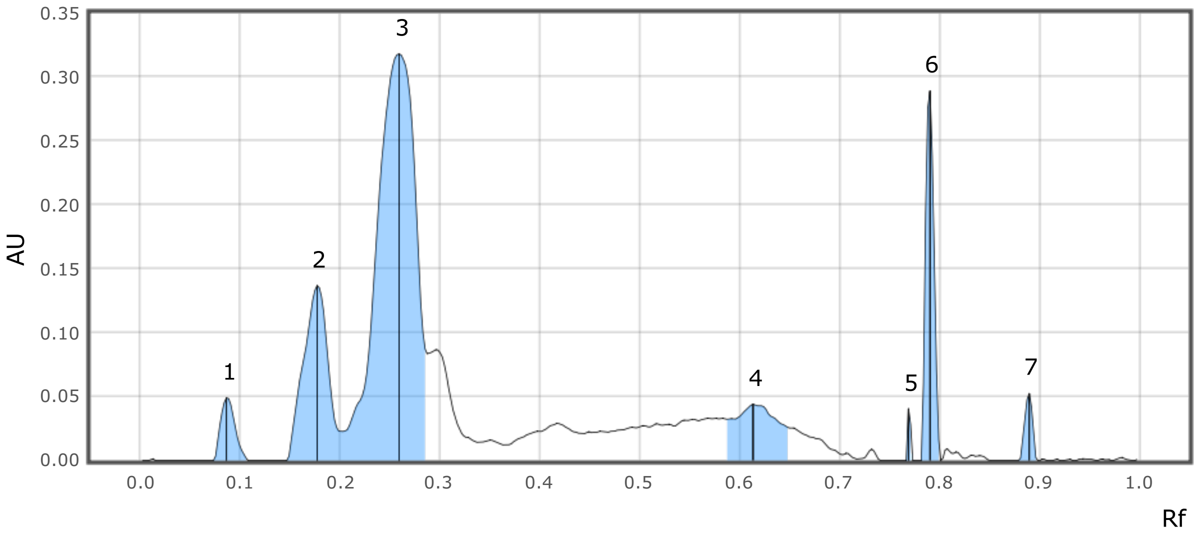

CAMAG®
World Leader in Planar Chromatography

Peak	Start		Max			End		Area		Manual	Substance
#	Rf	H	Rf	H	%	Rf	H	A	%	peak	Name
1	0.073	0.0000	0.086	0.0489	5.26	0.108	0.0000	0.00082	3.30	No	
2	0.147	0.0000	0.177	0.1367	14.71	0.201	0.0227	0.00391	15.80	No	
3	0.203	0.0226	0.259	0.3180	34.21	0.287	0.0834	0.01438	58.14	No	9-THC
4	0.588	0.0322	0.613	0.0438	4.72	0.650	0.0253	0.00223	9.01	No	
5	0.767	0.0000	0.769	0.0406	4.37	0.773	0.0000	0.00015	0.60	No	
6	0.782	0.0000	0.791	0.2890	31.09	0.801	0.0000	0.00279	11.28	No	
7	0.879	0.0000	0.890	0.0524	5.64	0.899	0.0000	0.00046	1.87	No	

Track 12:

Type	Sample
Vial ID	s9
Description	[redacted]
Volume	2.0 µl

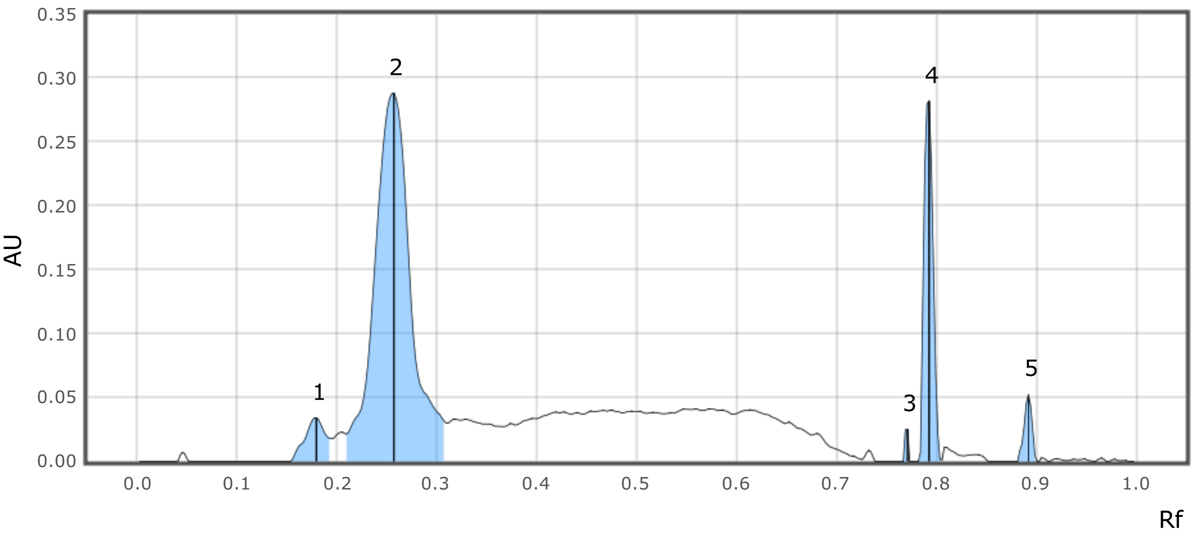

Peak	Start		Max			End		Area		Manual	Substance
#	Rf	H	Rf	H	%	Rf	H	A	%	peak	Name
1	0.153	0.0000	0.179	0.0340	4.99	0.194	0.0178	0.00082	4.98	No	
2	0.209	0.0213	0.257	0.2880	42.28	0.309	0.0302	0.01207	73.19	No	9-THC
3	0.767	0.0000	0.771	0.0251	3.69	0.773	0.0000	0.00011	0.65	No	
4	0.782	0.0000	0.793	0.2819	41.37	0.806	0.0012	0.00302	18.30	No	
5	0.881	0.0000	0.892	0.0522	7.67	0.901	0.0000	0.00047	2.87	No	

Track 13:

Type	Sample
Vial ID	s10
Description	[redacted]
Volume	2.0 µl

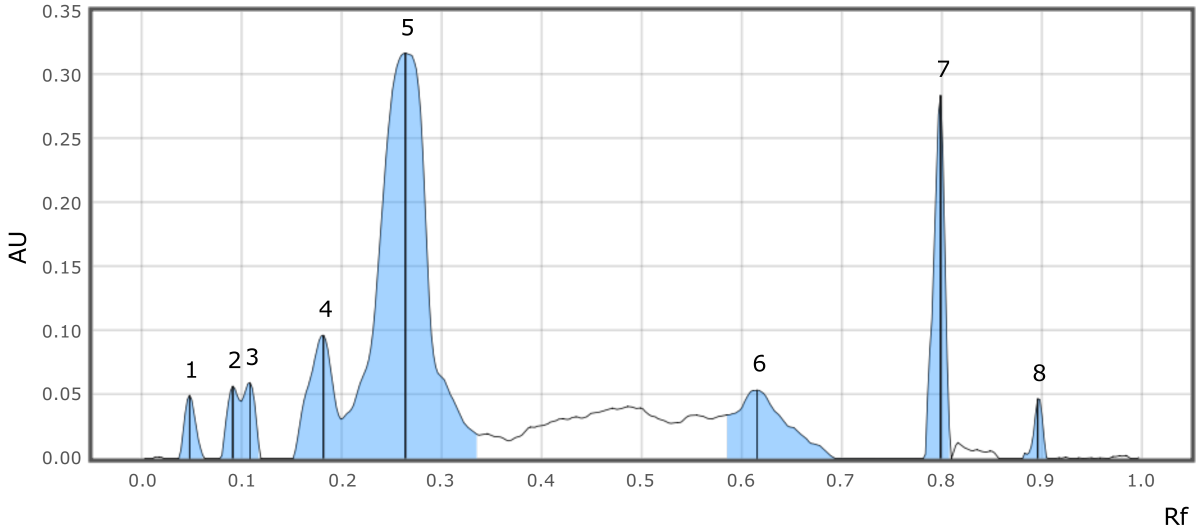

Peak #	Start		Max			End		Area		Manual peak	Substance Name
	Rf	H	Rf	H	%	Rf	H	A	%		
1	0.036	0.0000	0.047	0.0495	5.14	0.062	0.0000	0.00063	2.05	No	
2	0.078	0.0000	0.090	0.0565	5.87	0.099	0.0446	0.00081	2.64	No	
3	0.099	0.0446	0.108	0.0594	6.17	0.119	0.0000	0.00082	2.68	No	
4	0.151	0.0000	0.181	0.0964	10.01	0.199	0.0307	0.00274	8.93	No	
5	0.199	0.0307	0.263	0.3170	32.93	0.337	0.0182	0.01831	59.71	No	9-THC
6	0.581	0.0332	0.616	0.0534	5.54	0.696	0.0000	0.00329	10.71	No	
7	0.782	0.0000	0.799	0.2840	29.49	0.810	0.0000	0.00361	11.77	No	
8	0.881	0.0000	0.897	0.0467	4.85	0.907	0.0000	0.00046	1.51	No	

Track 14:

Type	Reference
Vial ID	250ug/mL mix
Description	250ug/mL
Volume	2.0 µl

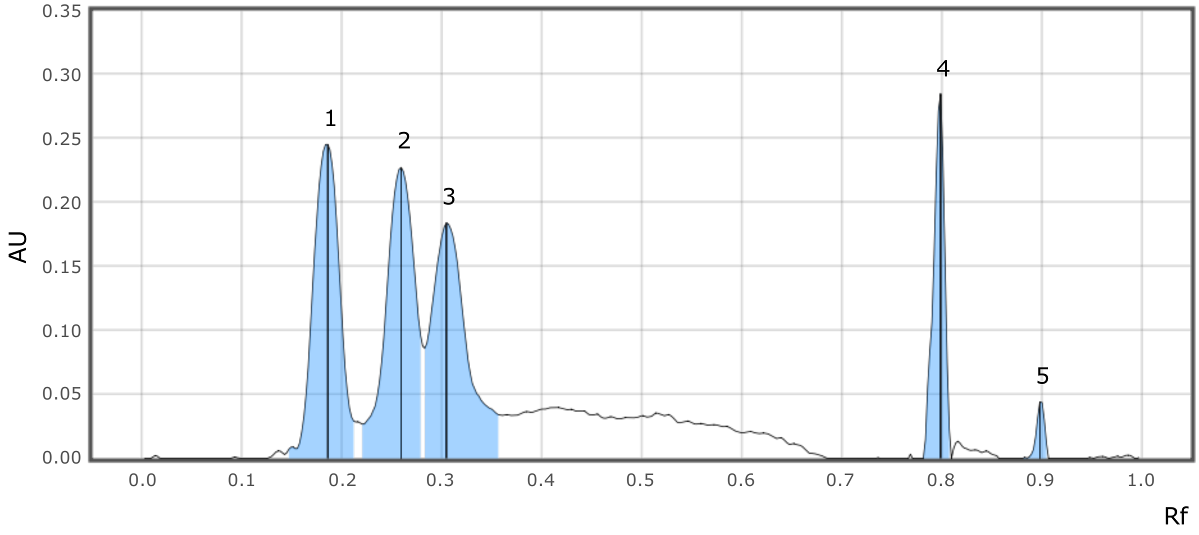

Peak #	Start Rf	Start H	Max Rf	Max H	Max %	End Rf	End H	Area A	Area %	Manual peak	Substance Name
1	0.147	0.0071	0.186	0.2453	24.89	0.214	0.0284	0.00748	27.94	No	CBN
2	0.220	0.0268	0.259	0.2273	23.07	0.281	0.0877	0.00761	28.40	No	9-THC
3	0.283	0.0861	0.304	0.1839	18.66	0.361	0.0336	0.00765	28.57	No	CBD
4	0.782	0.0000	0.799	0.2849	28.90	0.810	0.0000	0.00363	13.56	No	
5	0.881	0.0000	0.899	0.0442	4.48	0.907	0.0000	0.00041	1.53	No	

Track 15:

Type	Sample
Vial ID	MeOH blank
Description	MeOH Blank
Volume	2.0 µl

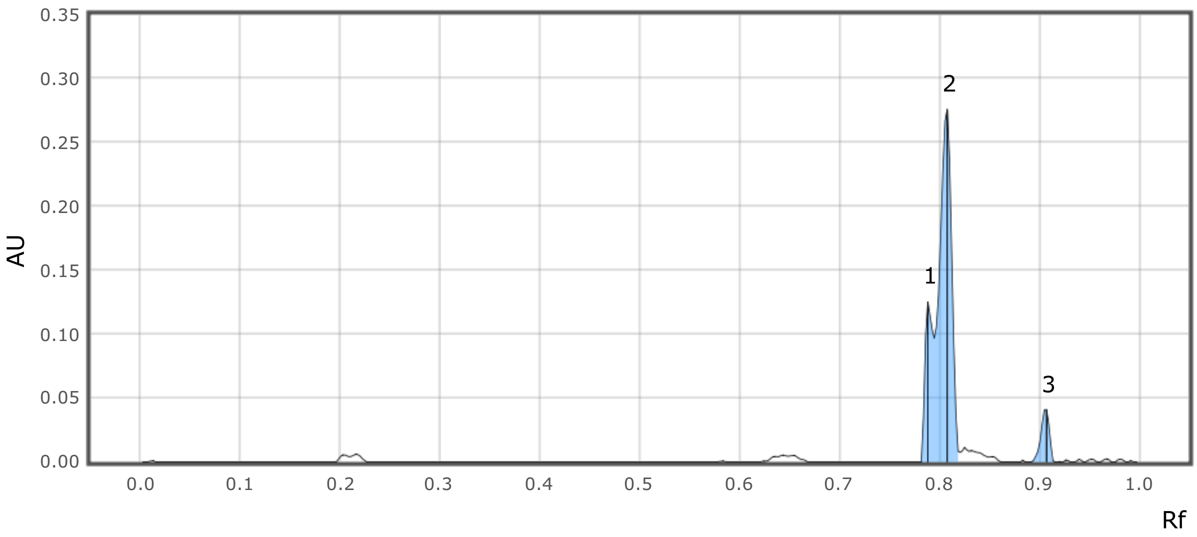

Peak #	Start Rf	Start H	Max Rf	Max H	Max %	End Rf	End H	Area A	Area %	Manual peak	Substance Name
1	0.782	0.0000	0.789	0.1250	28.31	0.795	0.0966	0.00113	21.08	No	
2	0.795	0.0966	0.808	0.2757	62.47	0.821	0.0074	0.00384	71.50	No	
3	0.892	0.0000	0.907	0.0407	9.22	0.916	0.0000	0.00040	7.43	No	

Calibration results:

Height calibration for substance 9-THC @ RT White:

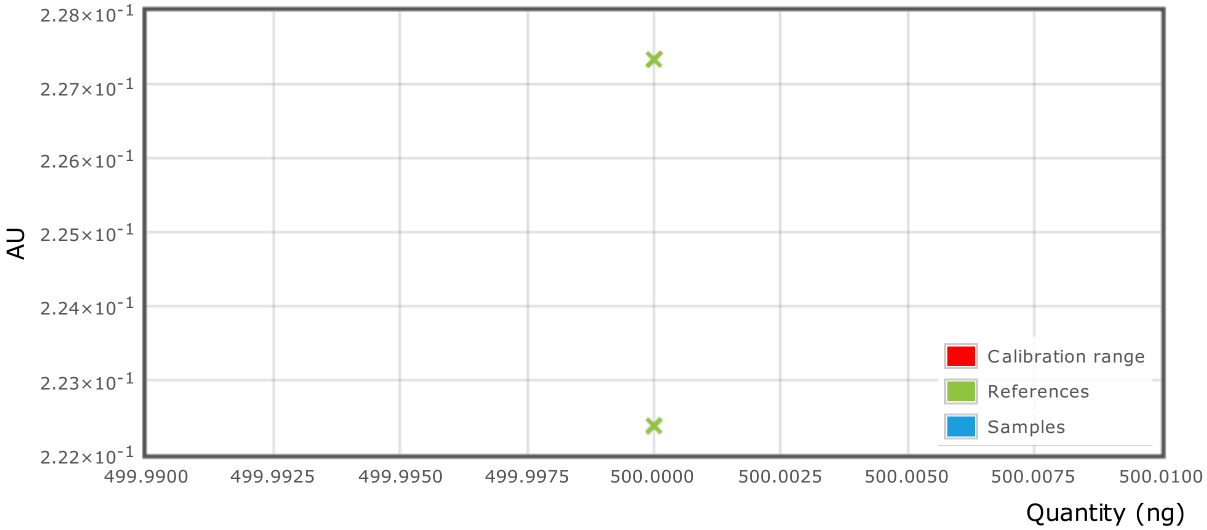

Regression mode	Linear-2
Range deviation	5.00 %
Related substances	Default
Number of references	2
Calibration function	$y=0x$
Coefficient of variation	CV 0.00 %
Correlation coefficient	n/a
⚠	Unable to compute the results for this substance because there wasn't eno ugh groups of references replicas (at least 1 for Linear-1, 2 for Linear2 and Mime-1 and 3 for Polynomial and MiMe-2)

Height calibration for substance CBD @ RT White:

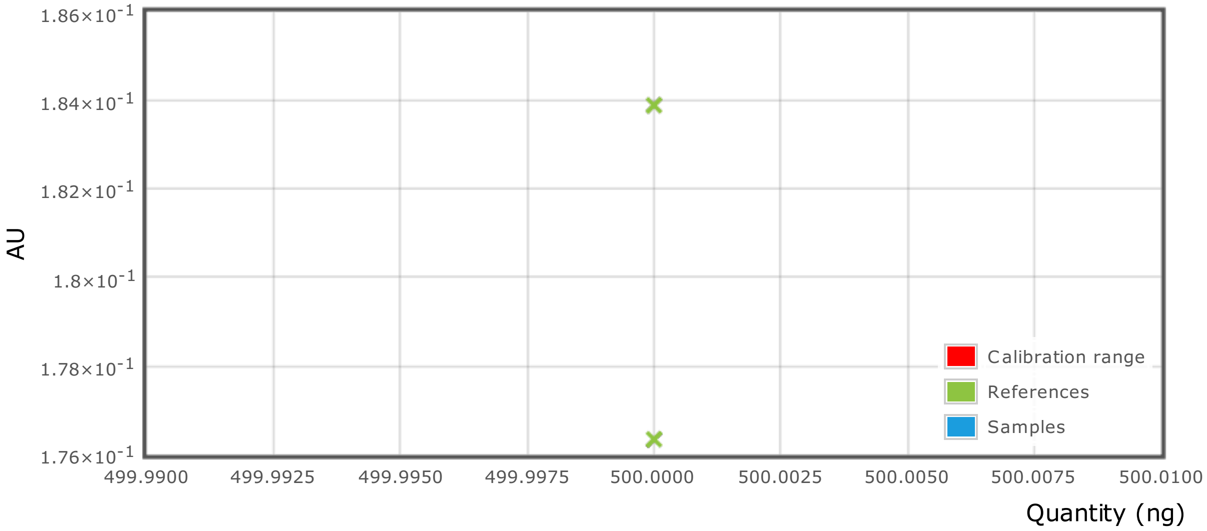

Regression mode	Linear-2
Range deviation	5.00 %
Related substances	Default
Number of references	2
Calibration function	$y=0x$
Coefficient of variation	CV 0.00 %
Correlation coefficient	n/a
⚠	Unable to compute the results for this substance because there wasn't eno ugh groups of references replicas (at least 1 for Linear-1, 2 for Linear2 and Mime-1 and 3 for Polynomial and MiMe-2)

Height calibration for substance CBN @ RT White:

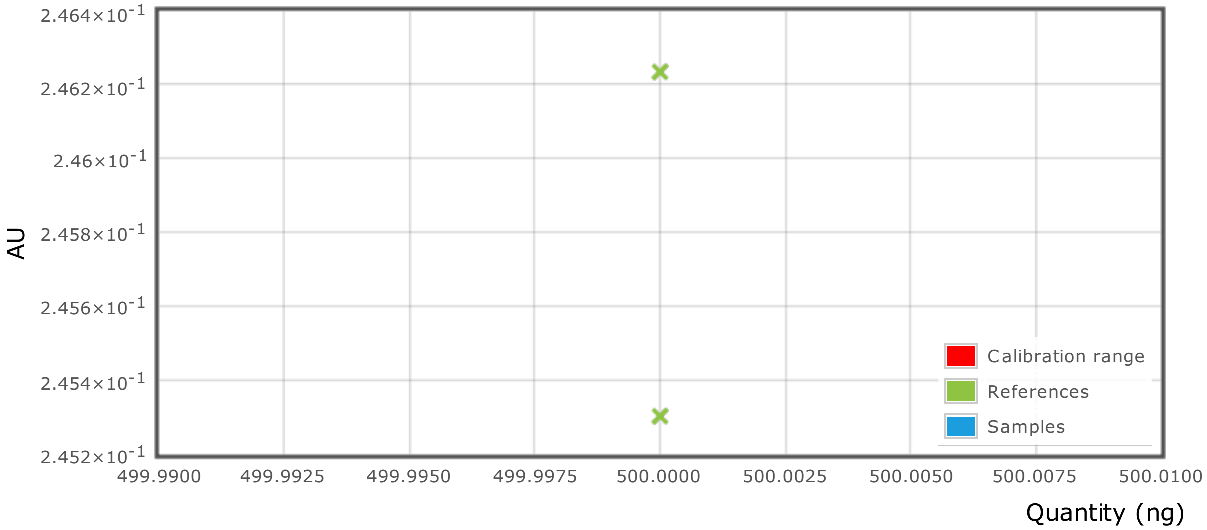

Regression mode	Linear-2
Range deviation	5.00 %
Related substances	Default
Number of references	2
Calibration function	$y=0x$
Coefficient of variation	CV 0.00 %
Correlation coefficient	n/a
⚠	Unable to compute the results for this substance because there wasn't eno ugh groups of references replicas (at least 1 for Linear-1, 2 for Linear2 and Mime-1 and 3 for Polynomial and MiMe-2)

Results:

Substance having no available results		
⚠	CBD	There wasn't any sample application available in the assignments for this substance. Please check that the peaks were correctly detected and assig ned for this substance.
⚠	9-THC	Unable to compute the results for this substance because there wasn't en ough groups of references replicas (at least 1 for Linear-1, 2 for Linear2 a nd Mime-1 and 3 for Polynomial and MiMe-2)
⚠	CBN	There wasn't any sample application available in the assignments for this substance. Please check that the peaks were correctly detected and assig ned for this substance.

A track marked with ⚠ means: this result is outside the regression range given by the reference assignments, but is included in the results because it is in the allowed range deviation.

Analyst:	Reviewer:
