# Supplementary material for: High Performance Thin-Layer Chromatography (HPTLC) data of Cannabinoids in ten mobile phase systems
Source: Data Brief. 2020 Jun 30;31:105955. doi: 10.1016/j.dib.2020.105955 (PMC7352075; doi:10.1016/j.dib.2020.105955)
Supplement: Supplementary file 1 [file mmc1.zip › S4-Case sample reports/XHDa-sample run-9.pdf]

Analysis: XHDa-sample run-9

Path: Home/YL Research

Based on method: Samples (no cal)

Created	15-Oct-2019 15:02:05	visionCATSuser
Modified	15-Oct-2019 17:08:50	visionCATSuser
Last HPTLC log	15-Oct-2019 17:08:50	Analysis modified
Explorer notes		

Track	Vial ID	Description	Volume	Position	Type
1	MeOH blank	MeOH Blank	2.0 µl	A1	Sample
2	250ug/mL mix	250ug/mL	2.0 µl	A2	Reference
3	Tetracosane	Tetracosane IS	2.0 µl	A3	Sample
4	s1	[redacted]	2.0 µl	B1	Sample
5	s2	[redacted]	2.0 µl	B2	Sample
6	s3	[redacted]	2.0 µl	B3	Sample
7	s4	[redacted]	2.0 µl	B4	Sample
8	s5	[redacted]	2.0 µl	B5	Sample
9	s6	[redacted]	2.0 µl	B6	Sample
10	s7	[redacted]	2.0 µl	B7	Sample
11	s8	[redacted]	2.0 µl	B8	Sample
12	s9	[redacted]	2.0 µl	B9	Sample
13	s10	[redacted]	2.0 µl	B10	Sample
14	250ug/mL mix	250ug/mL	2.0 µl	A2	Reference
15	MeOH blank	MeOH Blank	2.0 µl	A1	Sample
Sequence table notes					

A track marked with ⚠ means: the application type is overridden in some evaluation(s).

System setup:

Software	Server User-PC, version 2.5.18072.1
ATS4	S/N:080713
Chamber	N/A
Derivatization dip	N/A
Scanner3	S/N:031025
Visualizer	S/N:230515

Chromatography

Plate layout:

Stationary phase	Merck, HPTLC plates silica gel 60 F 254
Plate format	200.0 x 100.0 mm
Application type	Band
Application	Position Y: 8.0 mm, length: 8.0 mm, width: 0.0 mm
Track	First position X: 20.0 mm, distance: 11.4 mm
Solvent front position	70.0 mm
Notes	

Take image clean plate 1a - Visualizer (S/N: 230515):

Quality	Enhanced
RT White	auto capture, Auto, level 85 %, Band
R 254	auto capture, Auto, level 85 %, Band
Instrument diagnostics	Valid diagnostics
Documentation step label	
Notes	

Application 1 - ATS 4 (S/N: 080713):

Spray gas	NI
Sample solvent type	Methanol
Filling speed	15 µl/s
Predosage volume	200 nl
Retraction volume	200 nl
Dosage speed	150 nl/s
Filling quality	User
Rinsing cycles / vacuum	2 / 4 s
Filling cycles / vacuum	1 / 4 s
Rinsing solvent name	Methanol
Nozzle temperature	Unheated
Rack in use	Standard
Instrument diagnostics	Valid diagnostics
Notes	

Development 1 - Chamber:

Tank	TTC 20x10
Mobile phase	Xylene:hexane:diethylamine (25:10:1)
Saturation time	20 min
Use saturation pad	true
Use smartALERT	false
Volume front through	10 ml
Volume rear through	25 ml
Drying time	5 min
Drying temperature	Room temperature
Notes	

Take image developed plate 1a - Visualizer (S/N: 230515):

Quality	Enhanced
RT White	auto capture, Auto, level 85 %, Band
R 254	auto capture, Auto, level 85 %, Band
R 366	auto capture, Auto, level 85 %, Band
Instrument diagnostics	Valid diagnostics
Documentation step label	
Notes	

Scan developed plate 1b - Scanner 3 (S/N: 031025):

Scanner type	Single λ
Optimization for	Resolution
Measurement mode	Absorption
Filter	n/a
Detector mode	Automatic
Scanning speed	20 mm/s
Data resolution	100 µm/step
Slit	5 x 0.2 mm, micro
Partial scan	No
Lamp	Deuterium & Tungsten
Wavelength(s)	254 nm
Instrument diagnostics	Valid diagnostics
Documentation step label	
Notes	

Derivatization 1 - dip:

Reagent name	
Dipping speed	5
Dipping time	0 s
Reagent preparation	
Heating	100 °C for 3 min, heated after
Notes	

Take image derivatized plate 1a - Visualizer (S/N: 230515):

Quality	Enhanced
RT White	auto capture, Auto, level 85 %, Band
R 366	auto capture, Auto, level 85 %, Band
Instrument diagnostics	Valid diagnostics
Documentation step label	
Notes	

System suitability tests:

SST settings:

SST tracks	

Data acquisition

Application 1 - ATS 4 (S/N: 080713):

Executed	15-Oct-2019 15:25:25 visionCATSuser

Development 1 - Chamber:

Executed	15-Oct-2019 16:05:52 visionCATSuser

Take image developed plate 1a - Visualizer (S/N: 230515):

Executed	15-Oct-2019 16:54:19 visionCATSuser

RT White Developed, RemTransVis

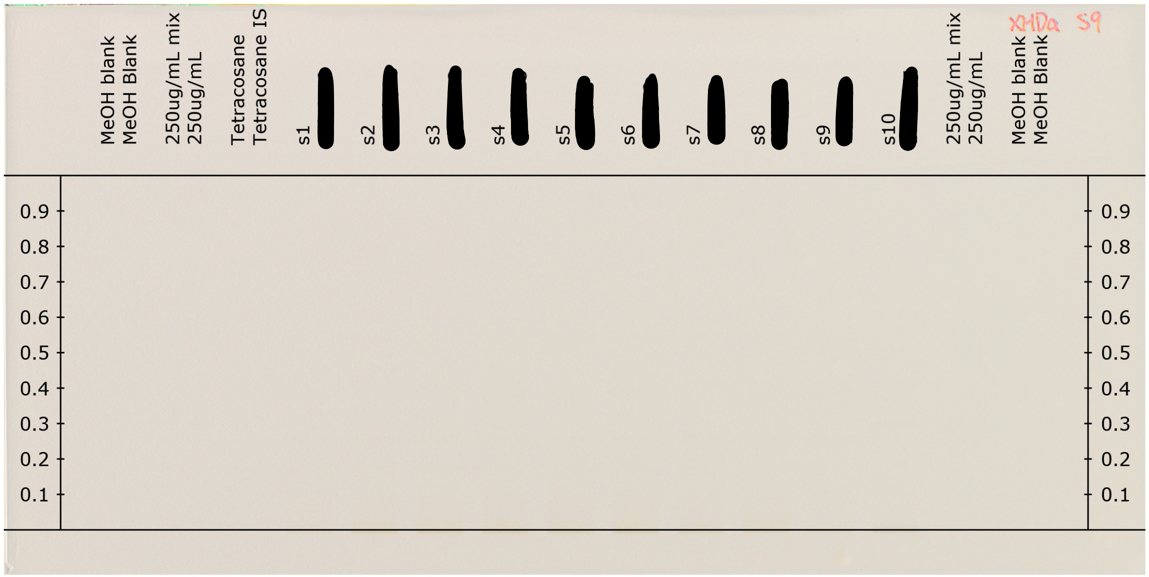

Exposure	0.082 s
Contrast	1
Normalized exposure	Disabled
Clarify	Disabled
White balance	1.00, 1.00, 1.00

R 254 Developed, Remission254

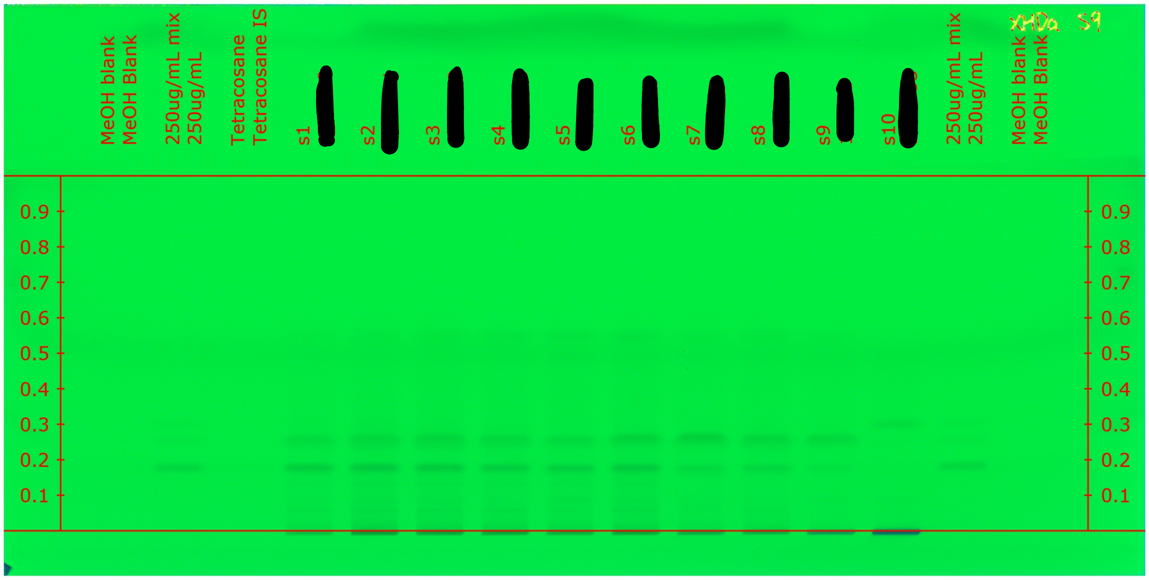

Exposure	0.270 s
Contrast	1
Normalized exposure	Disabled
Clarify	Disabled
White balance	1.00, 1.00, 1.00

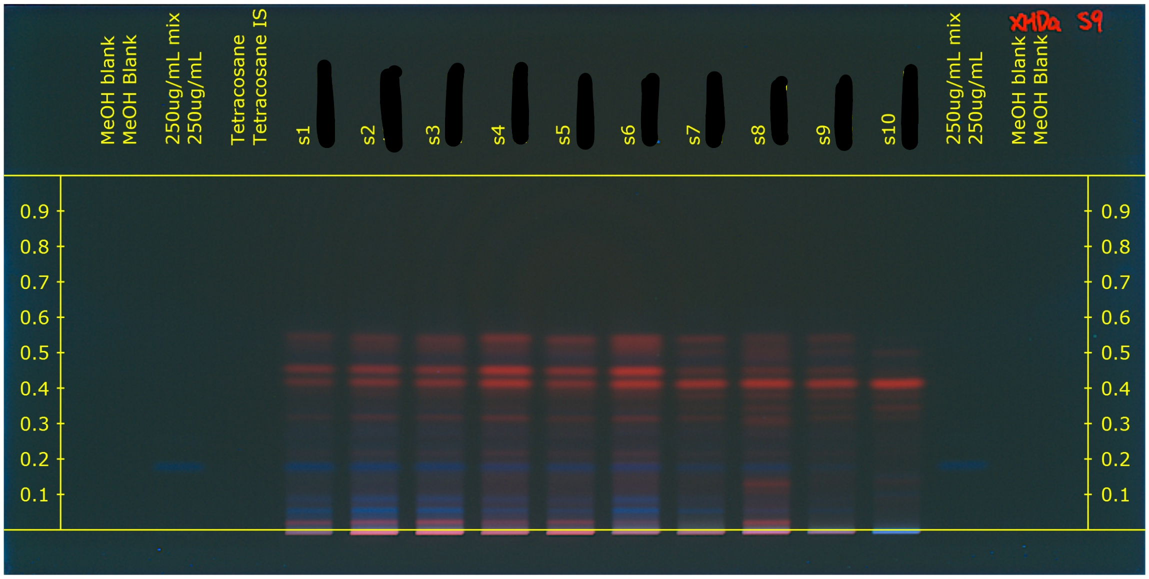

Exposure	3.097 s
Contrast	1
Normalized exposure	Disabled
Clarify	Disabled
White balance	1.00, 1.00, 1.00

Scan developed plate 1b - Scanner 3 (S/N: 031025):

Executed	15-Oct-2019 16:56:21 visionCATSuser

Scan:

Wavelength	254 nm

Track 1:

Type	Single λ

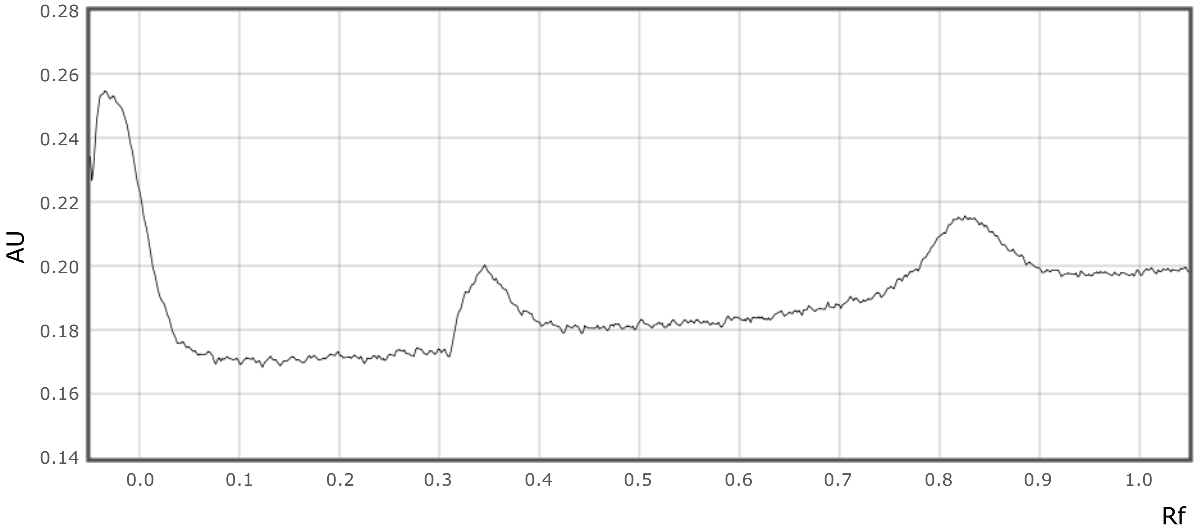

Track 2:

Type	Single λ

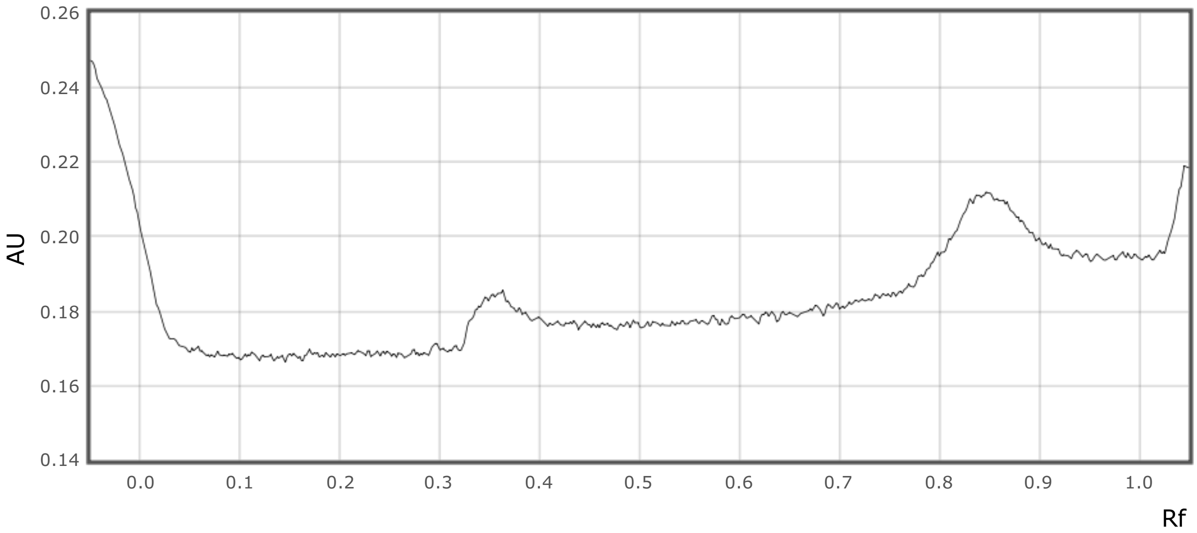

Track 3:

Type	Single λ

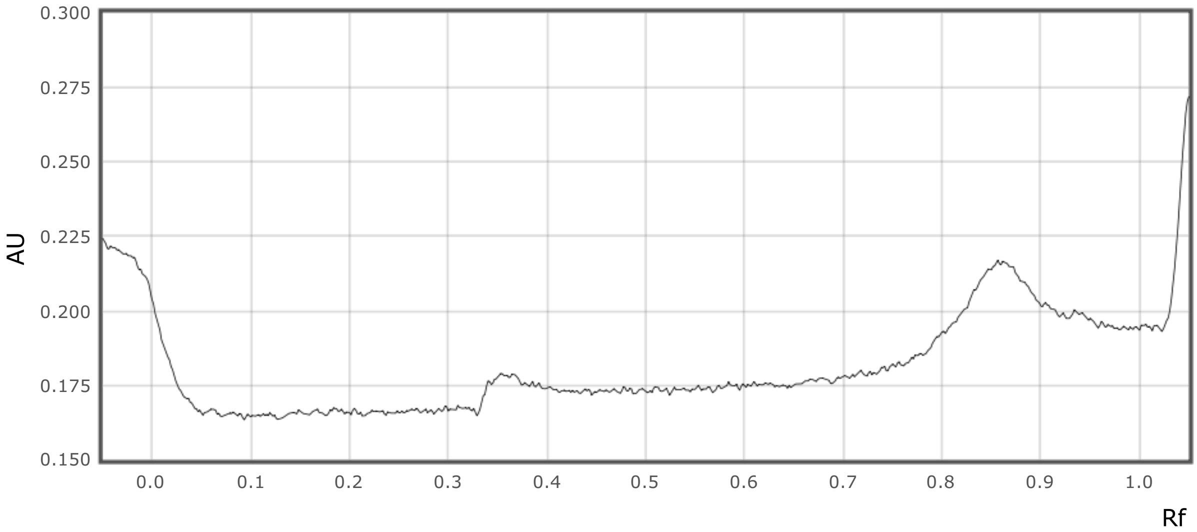

Track 4:

Type	Single λ

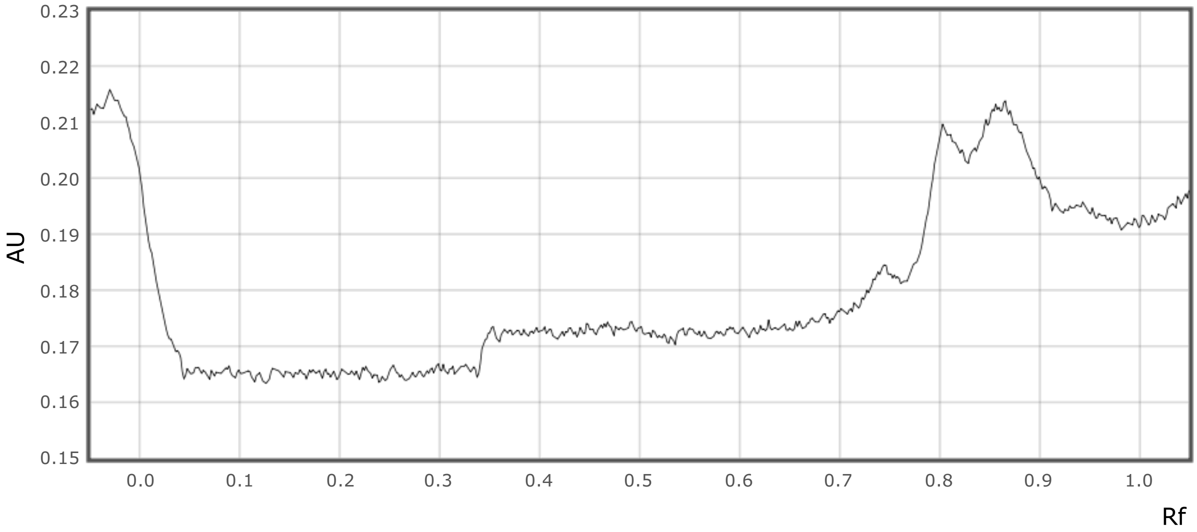

Track 5:	
Type	Single λ

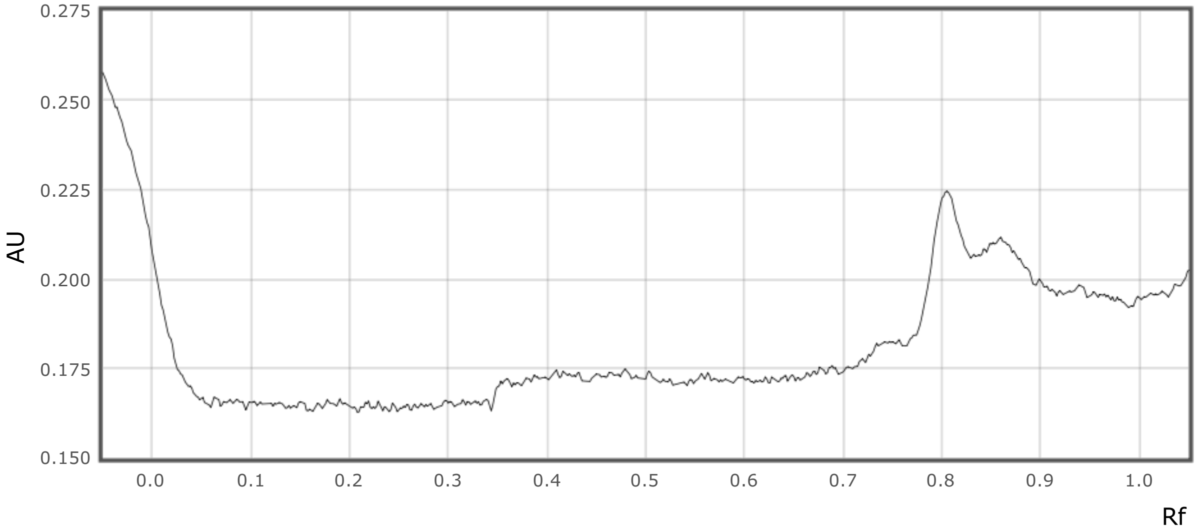

Track 6:	
Type	Single λ

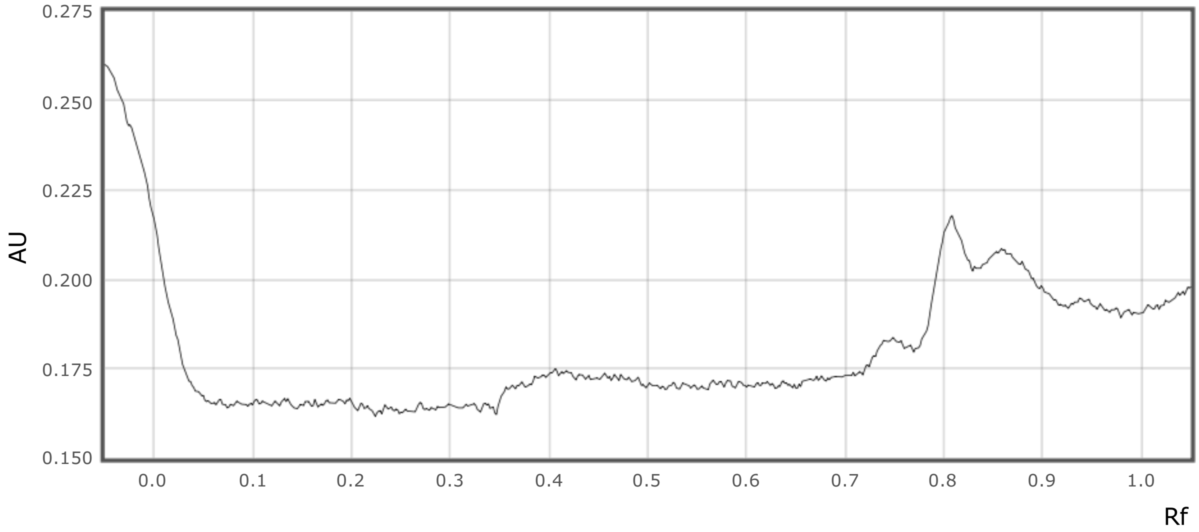

Track 7:	
Type	Single λ

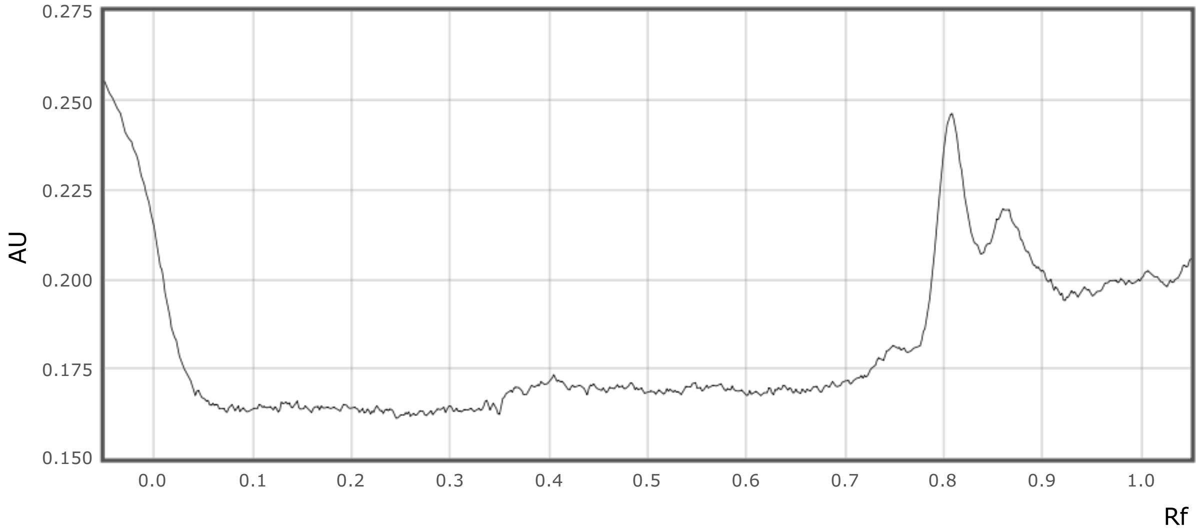

Track 8:	
Type	Single λ

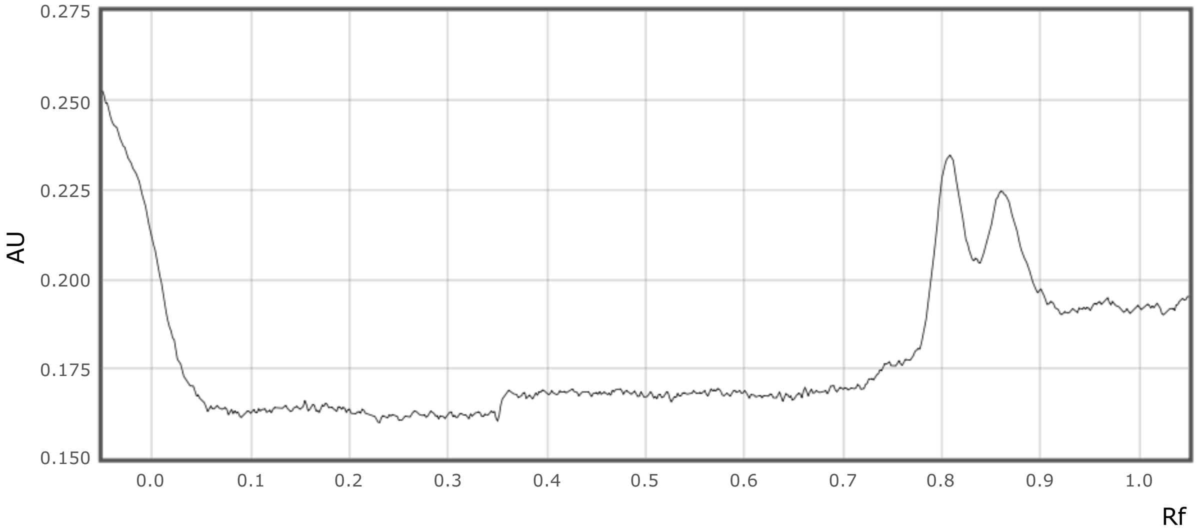

Track 9:	
Type	Single λ

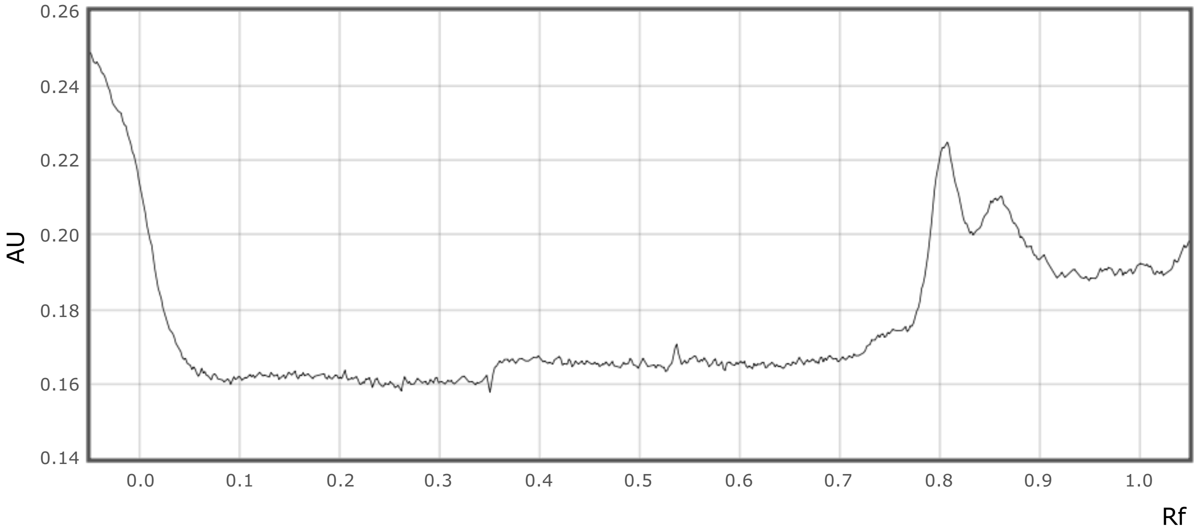

Track 10:	
Type	Single λ

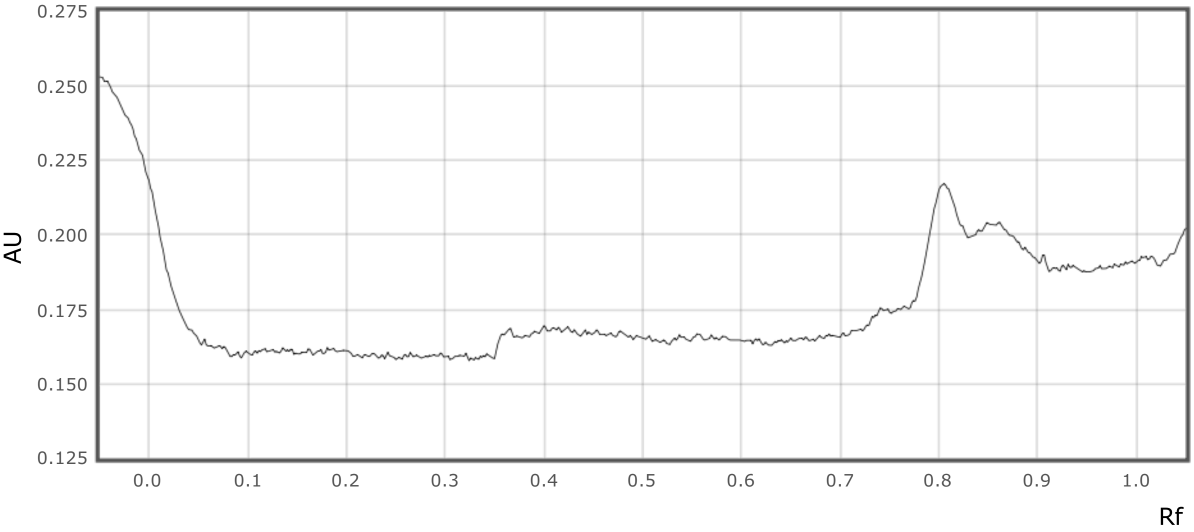

Track 11:

Type	Single λ

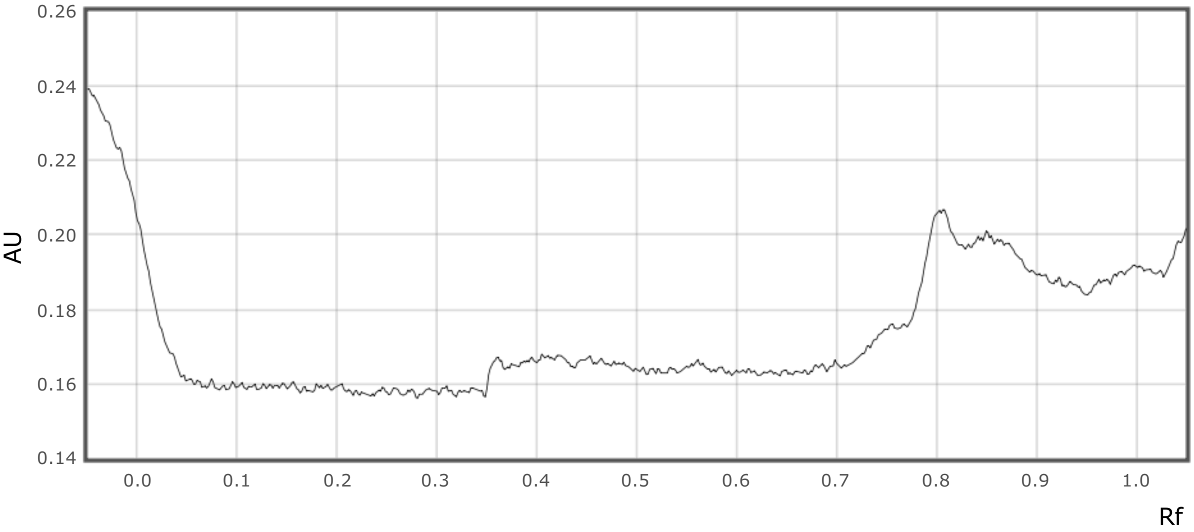

Track 12:

Type	Single λ

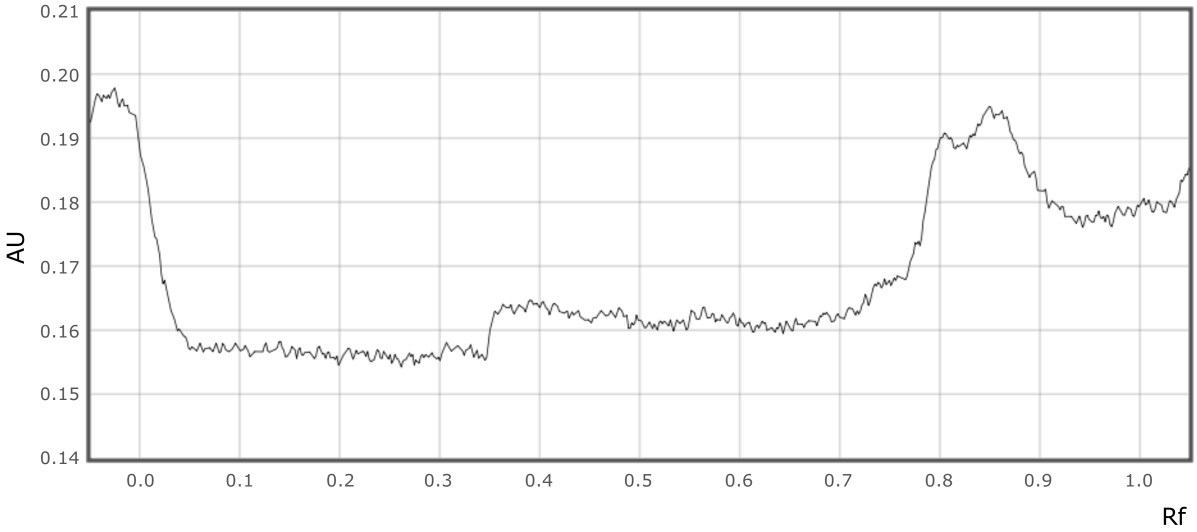

Track 13:	
Type	Single λ

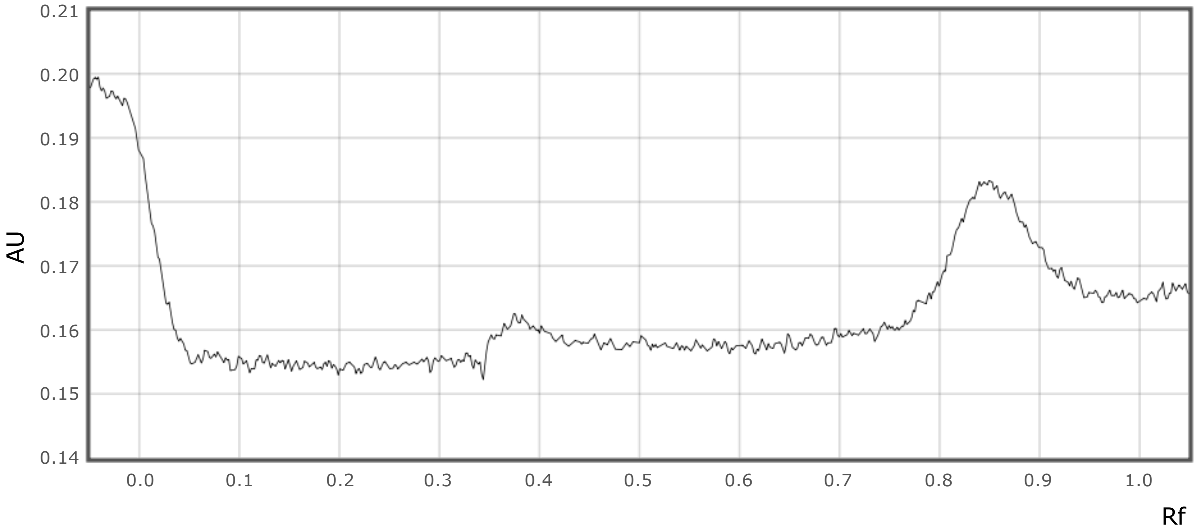

Track 14:	
Type	Single λ

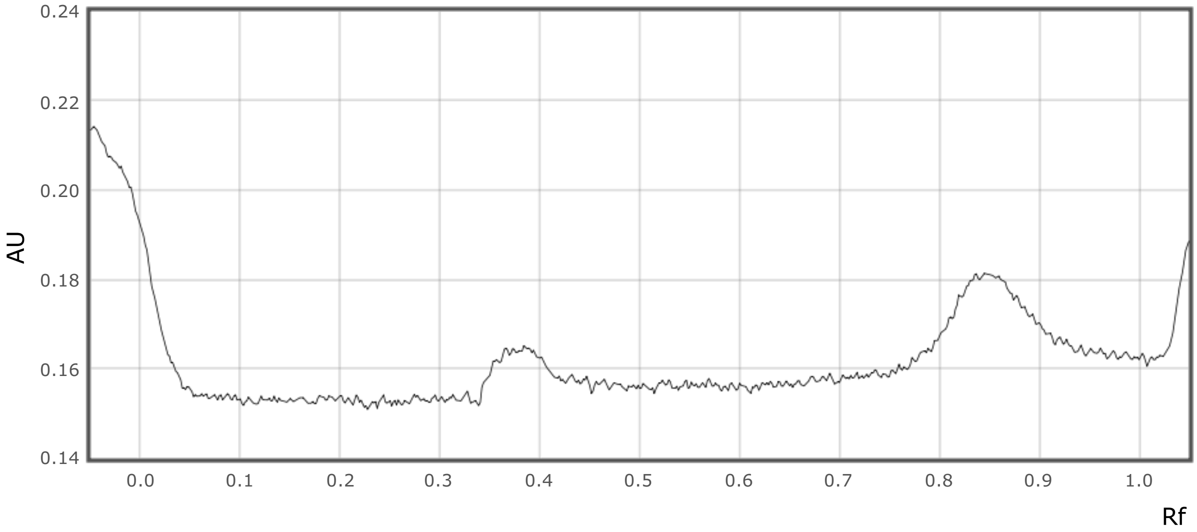

Track 15:	
Type	Single λ

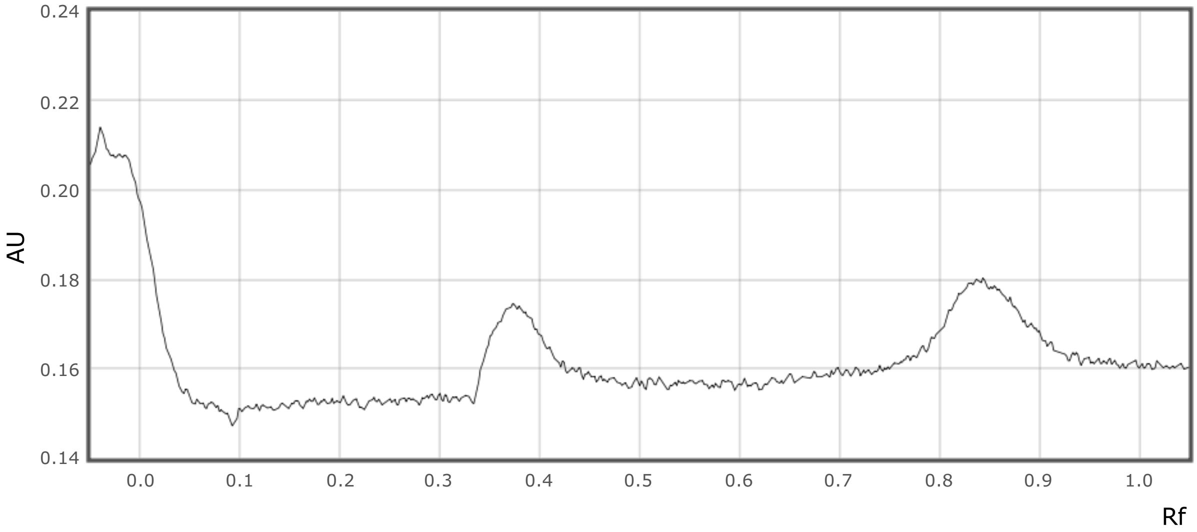

Derivatization 1 - dip:

Executed	15-Oct-2019 17:02:44 visionCATSuser

Take image derivatized plate 1a - Visualizer (S/N: 230515):

Executed	15-Oct-2019 17:06:18 visionCATSuser

RT White Derivatized, RemTransVis

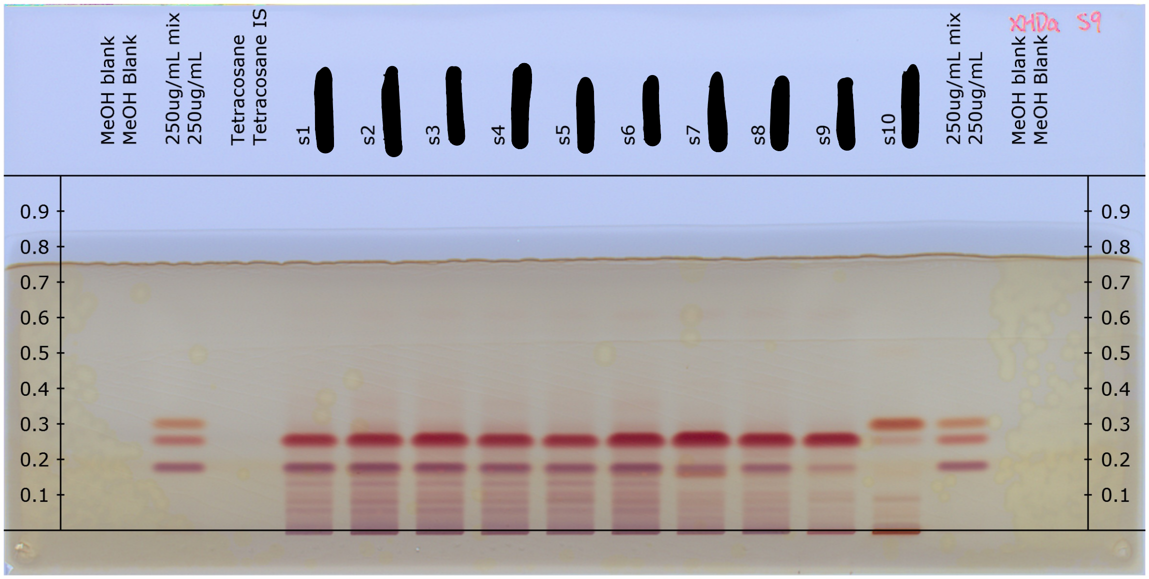

Exposure	0.077 s
Contrast	1
Normalized exposure	Disabled
Clarify	Disabled
White balance	1.30, 1.12, 0.75

R 366 Derivatized, Remission366

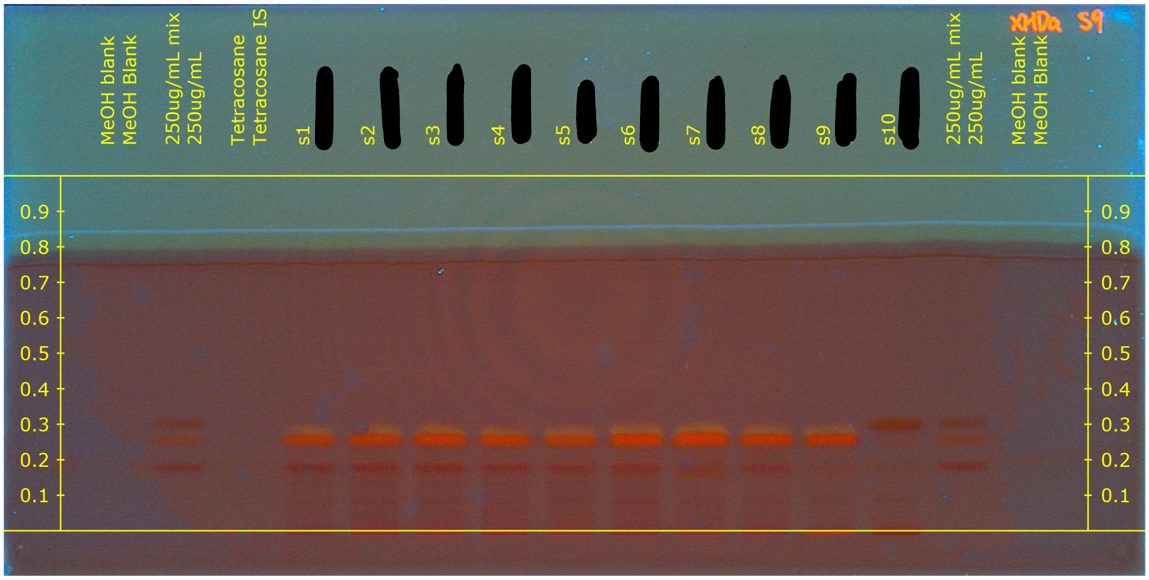

Exposure	9.999 s
Contrast	1
Normalized exposure	Disabled
Clarify	Disabled
White balance	1.00, 1.00, 1.00

Evaluation 1 :

Validated	false
Step	Take image derivatized plate 1a
Concentration unit type	Mass / volume
Notes	

Definition:

References:

250ug/mL mix

Substance Name	Concentration	Purity
9-THC	250.000 µg/ml	100.00 %
CBD	250.000 µg/ml	100.00 %
CBN	250.000 µg/ml	100.00 %

Samples:

Vial ID	Amount	Volume solution	Reference amount	Related to
MeOH blank		0.00 ml		
Tetracosane		0.00 ml		
s1		0.00 ml		
s2		0.00 ml		
s3		0.00 ml		
s4		0.00 ml		
s5		0.00 ml		
s6		0.00 ml		
s7		0.00 ml		
s8		0.00 ml		
s9		0.00 ml		
s10		0.00 ml		

Integration parameters:

Bounds	[0.000,1.000]
Smoothing	Savitzky–Golay of order 3 and window 7
Baseline correction	Lowest slope with noise 0.05
Profile subtraction	Profile subtraction from track 1
Peaks detection	Gauss (legacy) with sensitivity 0.1, separation 1 and threshold 0.1

Scan:

Wavelength	RT White

Track 1:

Type	Sample
Vial ID	MeOH blank
Description	MeOH Blank
Volume	2.0 µl

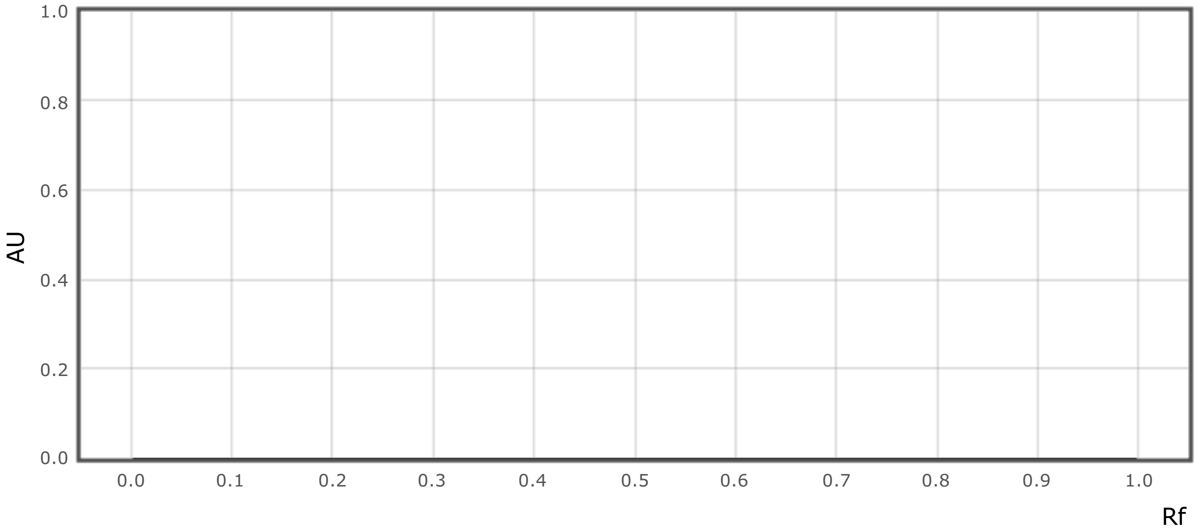

Peak #	Start Rf	Start H	Max Rf	Max H	Max %	End Rf	End H	Area A	Area %	Manual peak	Substance Name

Track 2:

Type	Reference
Vial ID	250ug/mL mix
Description	250ug/mL
Volume	2.0 µl

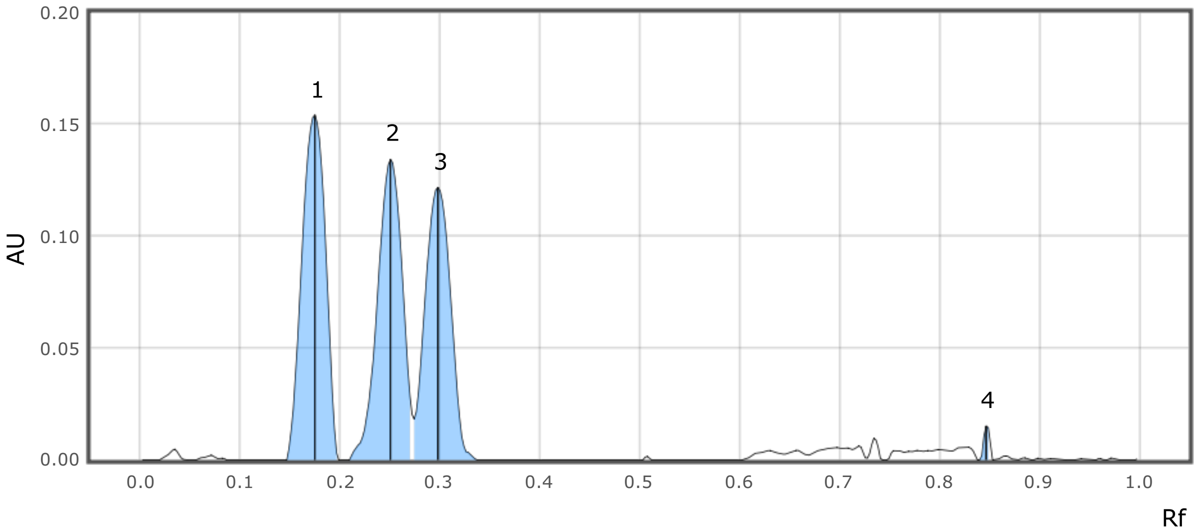

Peak #	Start Rf	Start H	Max Rf	Max H	Max %	End Rf	End H	Area A	Area %	Manual peak	Substance Name
1	0.147	0.0000	0.175	0.1542	36.23	0.199	0.0000	0.00418	35.68	No	CBN
2	0.209	0.0000	0.250	0.1343	31.57	0.272	0.0203	0.00384	32.84	No	9-THC
3	0.274	0.0182	0.298	0.1218	28.63	0.339	0.0000	0.00358	30.58	No	CBD
4	0.840	0.0000	0.847	0.0152	3.57	0.853	0.0000	0.00011	0.91	No	

Track 3:

Type	Sample
Vial ID	Tetracosane
Description	Tetracosane IS
Volume	2.0 µl

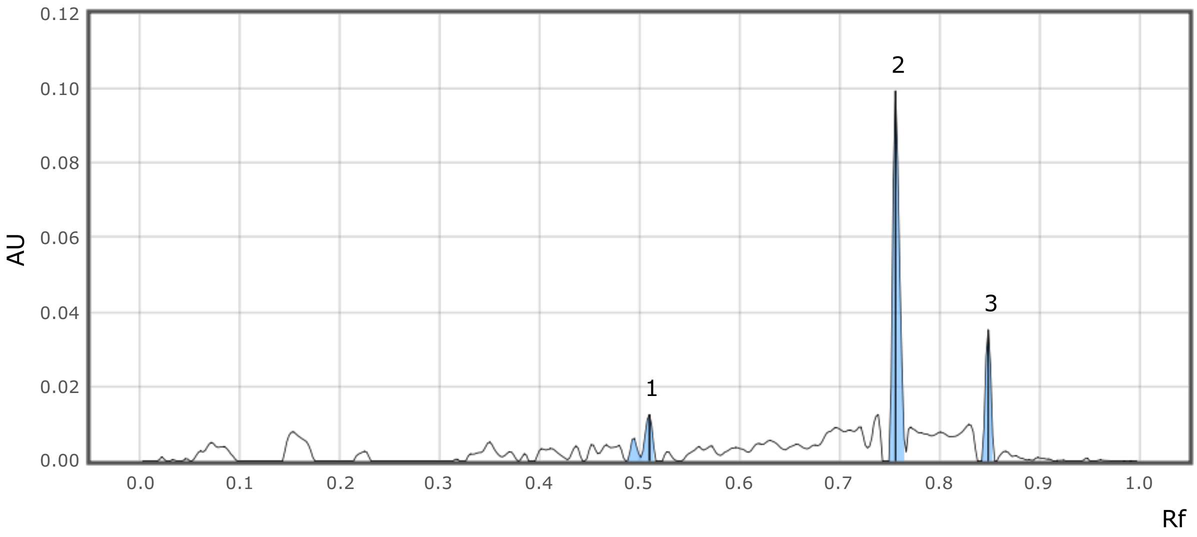

Peak #	Start		Max			End		Area		Manual peak	Substance Name
	Rf	H	Rf	H	%	Rf	H	A	%		
1	0.486	0.0000	0.510	0.0124	8.46	0.516	0.0000	0.00015	12.77	No	
2	0.750	0.0000	0.756	0.0992	67.58	0.767	0.0025	0.00077	67.49	No	
3	0.843	0.0000	0.849	0.0352	23.96	0.856	0.0000	0.00023	19.74	No	

Track 4:

Type	Sample
Vial ID	s1
Description	[redacted]
Volume	2.0 µl

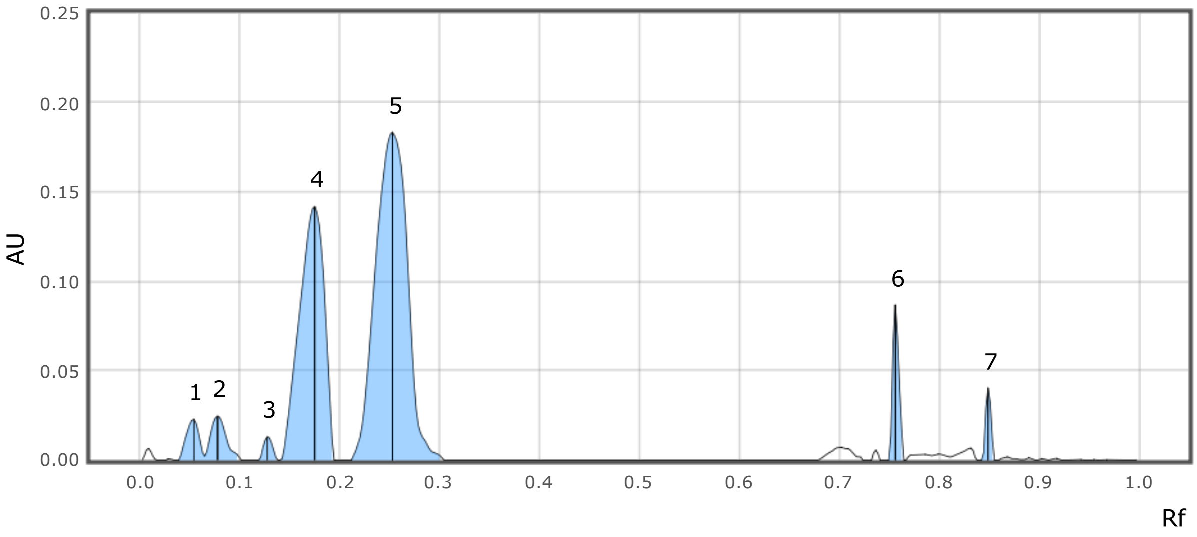

Peak	Start		Max			End		Area		Manual	Substance
#	Rf	H	Rf	H	%	Rf	H	A	%	peak	Name
1	0.039	0.0000	0.054	0.0228	4.45	0.065	0.0022	0.00033	2.64	No	
2	0.065	0.0022	0.078	0.0247	4.81	0.101	0.0000	0.00044	3.47	No	
3	0.119	0.0000	0.127	0.0131	2.56	0.138	0.0000	0.00013	1.06	No	
4	0.140	0.0000	0.175	0.1418	27.64	0.194	0.0000	0.00408	32.24	No	CBN
5	0.211	0.0000	0.253	0.1833	35.73	0.304	0.0000	0.00679	53.62	No	9-THC
6	0.750	0.0000	0.756	0.0868	16.92	0.765	0.0000	0.00063	4.98	No	
7	0.843	0.0000	0.849	0.0404	7.88	0.856	0.0000	0.00025	2.00	No	

Track 5:

Type	Sample
Vial ID	s2
Description	[redacted]
Volume	2.0 µl

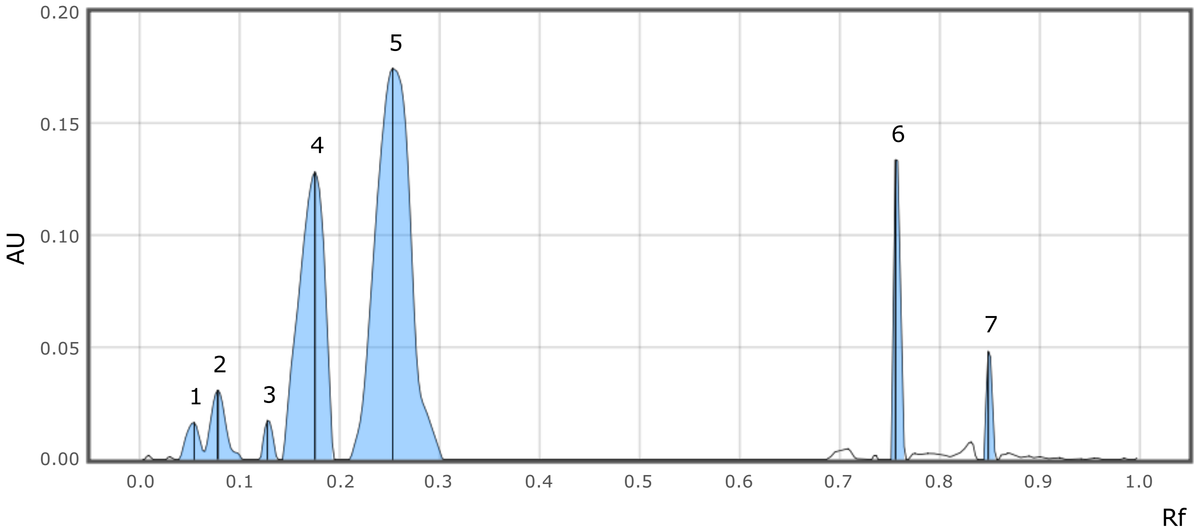

Peak	Start		Max			End		Area		Manual	Substance
#	Rf	H	Rf	H	%	Rf	H	A	%	peak	Name
1	0.039	0.0000	0.054	0.0165	2.99	0.065	0.0036	0.00025	1.89	No	
2	0.065	0.0036	0.078	0.0310	5.63	0.103	0.0000	0.00052	3.93	No	
3	0.119	0.0000	0.127	0.0174	3.17	0.138	0.0000	0.00017	1.30	No	
4	0.142	0.0000	0.175	0.1285	23.34	0.194	0.0000	0.00379	28.53	No	CBN
5	0.209	0.0000	0.253	0.1749	31.77	0.304	0.0000	0.00719	54.08	No	9-THC
6	0.752	0.0000	0.756	0.1340	24.33	0.767	0.0000	0.00104	7.83	No	
7	0.845	0.0000	0.849	0.0483	8.78	0.858	0.0000	0.00032	2.43	No	

Track 6:

Type	Sample
Vial ID	s3
Description	[redacted]
Volume	2.0 µl

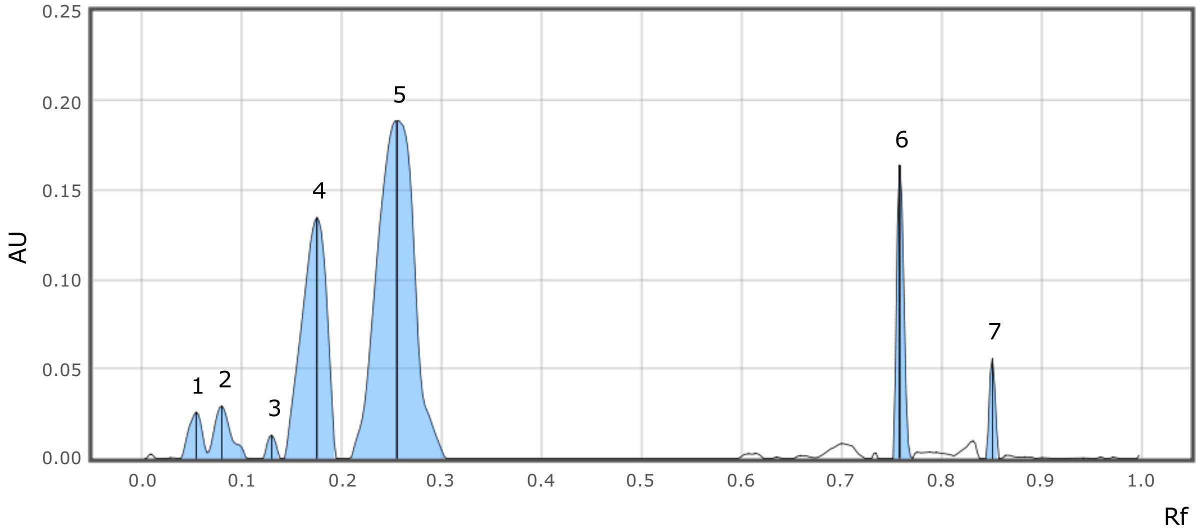

Peak #	Start		Max			End		Area		Manual peak	Substance Name
	Rf	H	Rf	H	%	Rf	H	A	%		
1	0.039	0.0000	0.054	0.0260	4.25	0.065	0.0031	0.00039	2.65	No	
2	0.065	0.0031	0.080	0.0293	4.79	0.106	0.0000	0.00056	3.76	No	
3	0.121	0.0000	0.129	0.0131	2.14	0.138	0.0000	0.00013	0.87	No	
4	0.140	0.0000	0.175	0.1347	22.03	0.194	0.0000	0.00385	25.99	No	CBN
5	0.207	0.0000	0.255	0.1887	30.85	0.304	0.0000	0.00818	55.14	No	9-THC
6	0.752	0.0000	0.758	0.1639	26.80	0.771	0.0000	0.00134	9.06	No	
7	0.845	0.0000	0.851	0.0560	9.15	0.858	0.0000	0.00038	2.53	No	

Track 7:

Type	Sample
Vial ID	s4
Description	[redacted]
Volume	2.0 µl

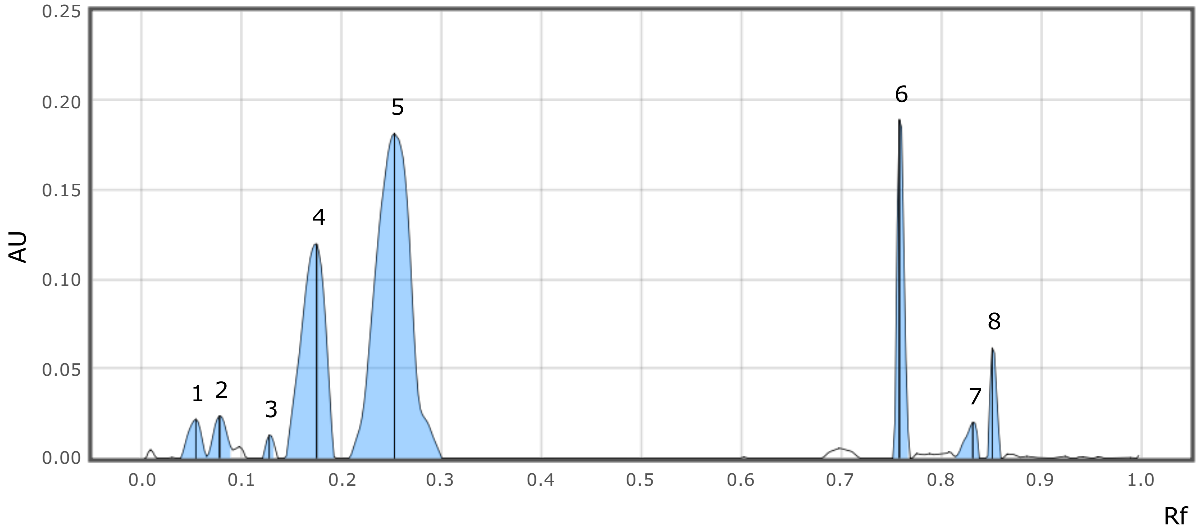

Peak #	Start		Max			End		Area		Manual peak	Substance Name
	Rf	H	Rf	H	%	Rf	H	A	%		
1	0.039	0.0000	0.054	0.0219	3.47	0.065	0.0009	0.00033	2.38	No	
2	0.065	0.0009	0.078	0.0236	3.75	0.090	0.0046	0.00036	2.62	No	
3	0.121	0.0000	0.127	0.0129	2.04	0.136	0.0000	0.00012	0.85	No	
4	0.142	0.0000	0.175	0.1197	18.99	0.194	0.0000	0.00331	24.09	No	CBN
5	0.207	0.0000	0.253	0.1814	28.78	0.302	0.0000	0.00733	53.44	No	9-THC
6	0.752	0.0000	0.758	0.1892	30.01	0.769	0.0000	0.00156	11.36	No	
7	0.814	0.0008	0.832	0.0201	3.18	0.840	0.0000	0.00026	1.91	No	
8	0.845	0.0000	0.851	0.0616	9.78	0.862	0.0000	0.00046	3.35	No	

Track 8:	
Type	Sample
Vial ID	s5
Description	[redacted]
Volume	2.0 µl

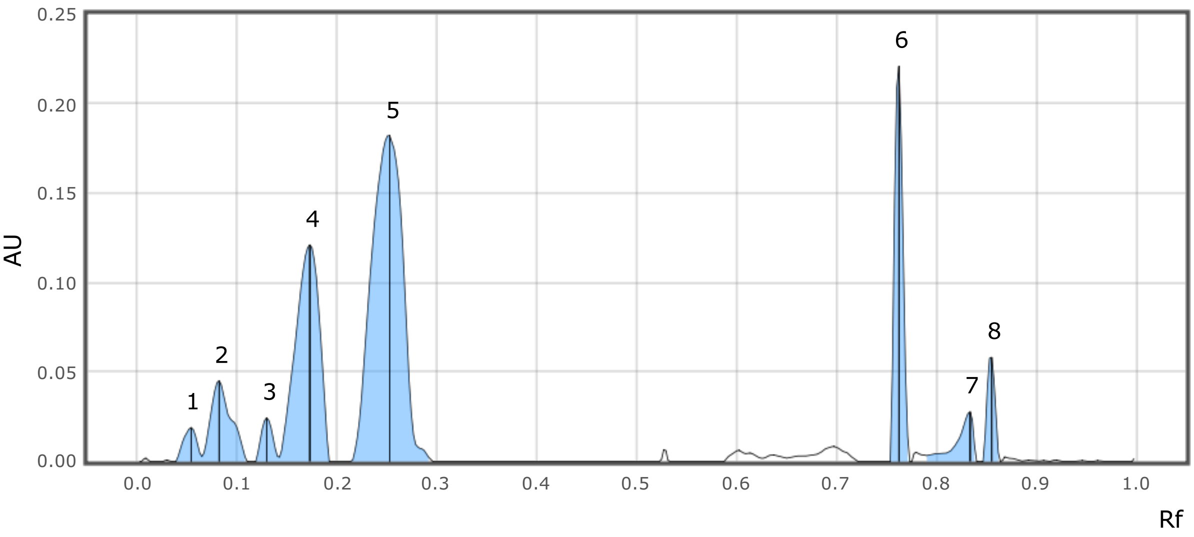

Peak #	Start		Max			End		Area		Manual peak	Substance Name
	Rf	H	Rf	H	%	Rf	H	A	%		
1	0.039	0.0000	0.054	0.0189	2.71	0.065	0.0027	0.00029	1.97	No	
2	0.065	0.0027	0.082	0.0451	6.46	0.110	0.0000	0.00104	7.06	No	
3	0.119	0.0000	0.129	0.0244	3.49	0.142	0.0023	0.00032	2.16	No	
4	0.142	0.0023	0.173	0.1210	17.34	0.194	0.0000	0.00333	22.52	No	CBN
5	0.214	0.0000	0.253	0.1820	26.07	0.296	0.0000	0.00662	44.84	No	9-THC
6	0.754	0.0000	0.763	0.2208	31.63	0.773	0.0000	0.00213	14.40	No	
7	0.791	0.0033	0.834	0.0277	3.96	0.840	0.0000	0.00051	3.44	No	
8	0.847	0.0000	0.856	0.0582	8.33	0.864	0.0000	0.00053	3.60	No	

Track 9:	
Type	Sample
Vial ID	s6
Description	[redacted]
Volume	2.0 µl

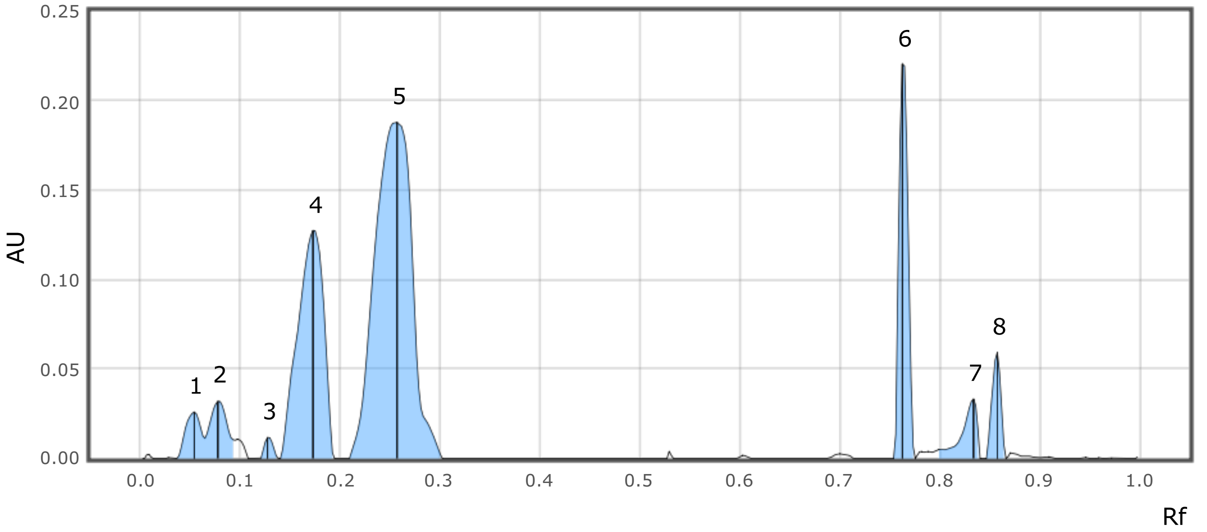

Peak #	Start		Max			End		Area		Manual peak	Substance Name
	Rf	H	Rf	H	%	Rf	H	A	%		
1	0.036	0.0000	0.054	0.0260	3.73	0.065	0.0112	0.00046	2.76	No	
2	0.065	0.0112	0.078	0.0319	4.57	0.095	0.0102	0.00065	3.89	No	
3	0.119	0.0000	0.127	0.0117	1.68	0.138	0.0000	0.00012	0.69	No	
4	0.140	0.0000	0.173	0.1274	18.26	0.194	0.0000	0.00380	22.82	No	CBN
5	0.207	0.0000	0.257	0.1877	26.91	0.302	0.0000	0.00816	49.01	No	9-THC
6	0.754	0.0000	0.763	0.2205	31.61	0.776	0.0000	0.00230	13.79	No	
7	0.797	0.0043	0.834	0.0333	4.77	0.840	0.0000	0.00058	3.46	No	
8	0.847	0.0000	0.858	0.0592	8.48	0.866	0.0000	0.00060	3.58	No	

Track 10:

Type	Sample
Vial ID	s7
Description	[redacted]
Volume	2.0 µl

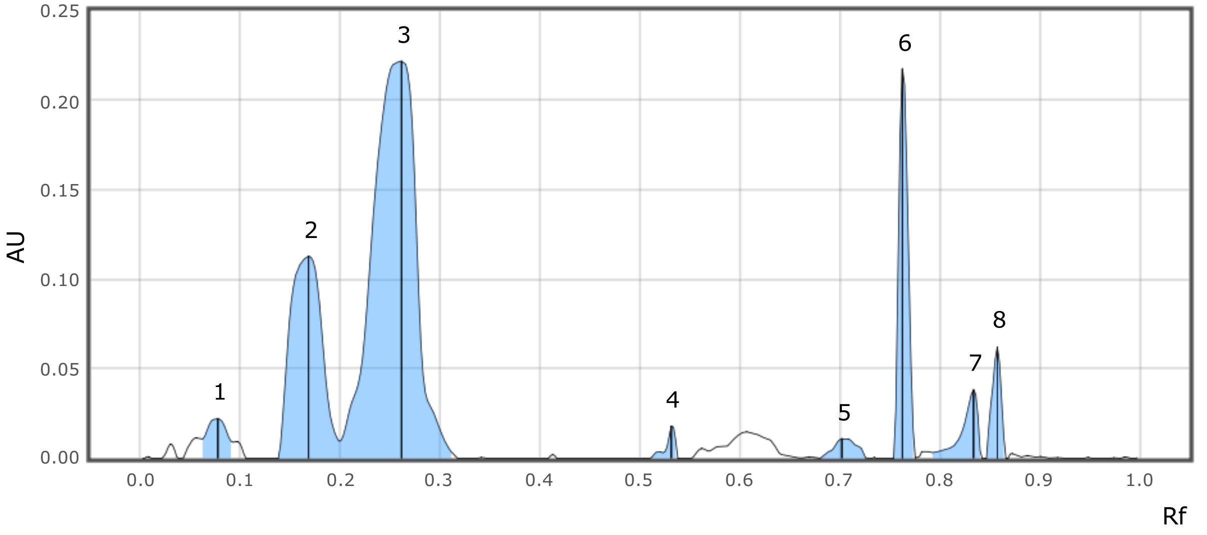

Peak #	Start		Max			End		Area		Manual peak	Substance Name
	Rf	H	Rf	H	%	Rf	H	A	%		
1	0.060	0.0108	0.078	0.0221	3.13	0.093	0.0090	0.00054	2.68	No	
2	0.138	0.0000	0.168	0.1129	16.04	0.199	0.0096	0.00419	20.74	No	CBN
3	0.199	0.0096	0.261	0.2218	31.51	0.317	0.0000	0.01134	56.09	No	9-THC
4	0.510	0.0000	0.531	0.0181	2.57	0.538	0.0000	0.00018	0.90	No	
5	0.680	0.0000	0.702	0.0110	1.57	0.728	0.0000	0.00030	1.47	No	
6	0.754	0.0000	0.763	0.2176	30.91	0.776	0.0002	0.00233	11.54	No	
7	0.793	0.0032	0.834	0.0384	5.45	0.843	0.0000	0.00068	3.34	No	
8	0.847	0.0000	0.858	0.0622	8.83	0.866	0.0000	0.00065	3.24	No	

Track 11:

Type	Sample
Vial ID	s8
Description	[redacted]
Volume	2.0 µl

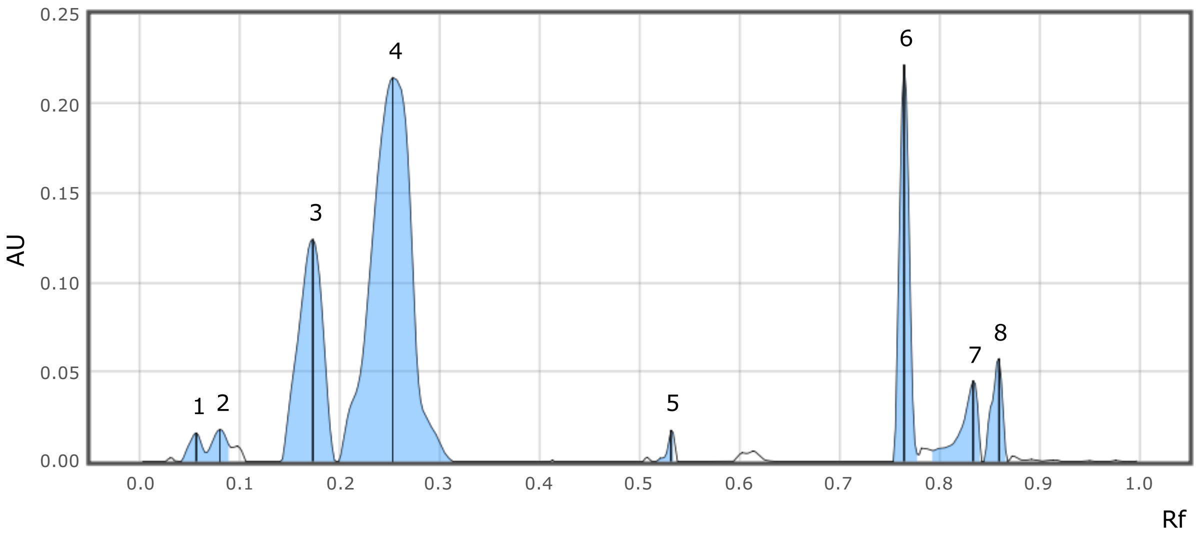

Peak #	Start		Max			End		Area		Manual peak	Substance Name
	Rf	H	Rf	H	%	Rf	H	A	%		
1	0.041	0.0000	0.056	0.0159	2.23	0.067	0.0050	0.00024	1.30	No	
2	0.067	0.0050	0.080	0.0179	2.51	0.090	0.0078	0.00030	1.66	No	
3	0.140	0.0000	0.173	0.1241	17.38	0.196	0.0000	0.00353	19.36	No	CBN
4	0.199	0.0000	0.253	0.2145	30.03	0.313	0.0000	0.00997	54.69	No	9-THC
5	0.516	0.0000	0.531	0.0175	2.45	0.538	0.0000	0.00014	0.75	No	
6	0.754	0.0000	0.765	0.2217	31.03	0.780	0.0037	0.00247	13.57	No	
7	0.793	0.0061	0.834	0.0451	6.31	0.843	0.0000	0.00088	4.82	No	
8	0.845	0.0000	0.860	0.0575	8.05	0.868	0.0000	0.00070	3.85	No	

Track 12:

Type	Sample
Vial ID	s9
Description	[redacted]
Volume	2.0 µl

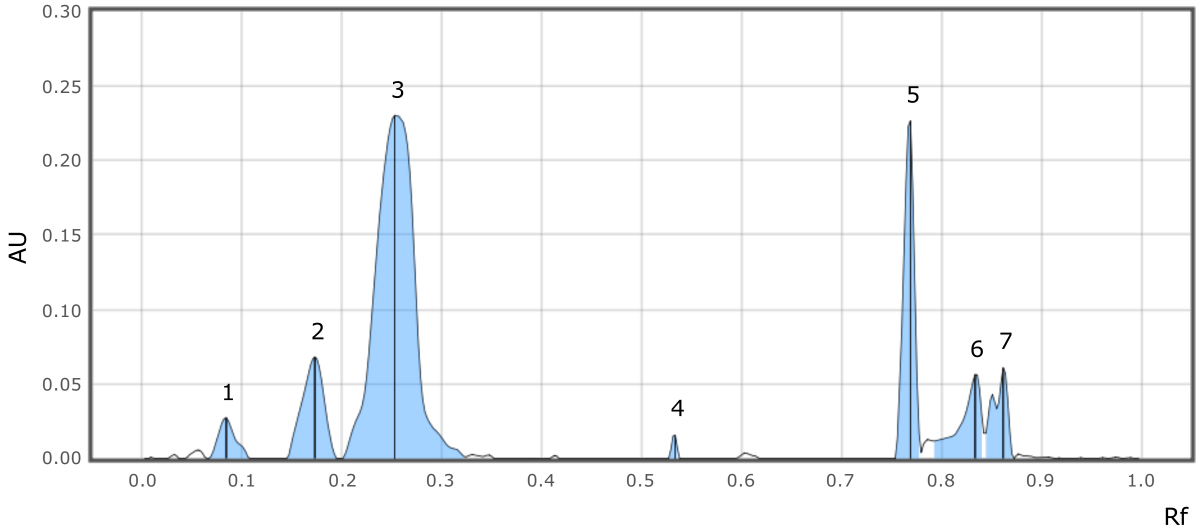

Peak #	Start		Max			End		Area		Manual peak	Substance Name
	Rf	H	Rf	H	%	Rf	H	A	%		
1	0.067	0.0000	0.084	0.0272	3.98	0.108	0.0000	0.00053	2.96	No	
2	0.144	0.0000	0.173	0.0681	9.95	0.196	0.0000	0.00172	9.68	No	CBN
3	0.201	0.0000	0.253	0.2299	33.62	0.324	0.0009	0.01037	58.37	No	9-THC
4	0.527	0.0000	0.533	0.0156	2.28	0.538	0.0000	0.00009	0.53	No	
5	0.754	0.0000	0.769	0.2263	33.08	0.780	0.0039	0.00278	15.64	No	
6	0.793	0.0116	0.834	0.0562	8.22	0.843	0.0169	0.00129	7.27	No	
7	0.845	0.0169	0.862	0.0607	8.87	0.873	0.0000	0.00098	5.54	No	

Track 13:

Type	Sample
Vial ID	s10
Description	[redacted]
Volume	2.0 µl

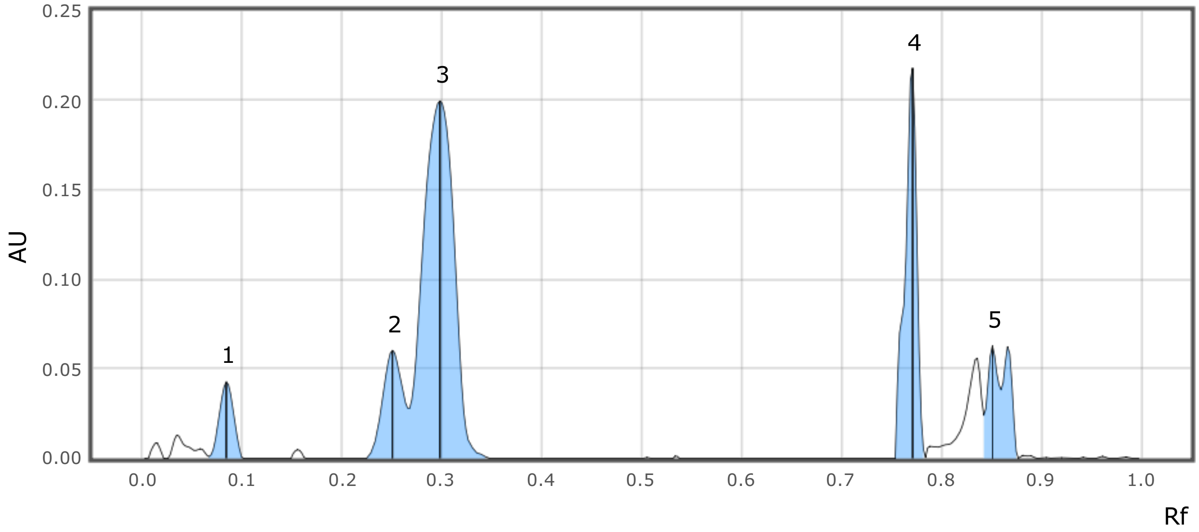

Peak #	Start Rf	Start H	Max Rf	Max H	Max %	End Rf	End H	Area A	Area %	Manual peak	Substance Name
1	0.067	0.0009	0.084	0.0426	7.31	0.101	0.0000	0.00069	5.09	No	
2	0.224	0.0000	0.250	0.0602	10.34	0.268	0.0279	0.00142	10.43	No	9-THC
3	0.268	0.0279	0.298	0.1995	34.22	0.348	0.0000	0.00708	52.05	No	CBD
4	0.754	0.0000	0.771	0.2178	37.36	0.784	0.0005	0.00297	21.84	No	
5	0.843	0.0240	0.851	0.0628	10.77	0.877	0.0000	0.00144	10.58	No	

Track 14:

Type	Reference
Vial ID	250ug/mL mix
Description	250ug/mL
Volume	2.0 µl

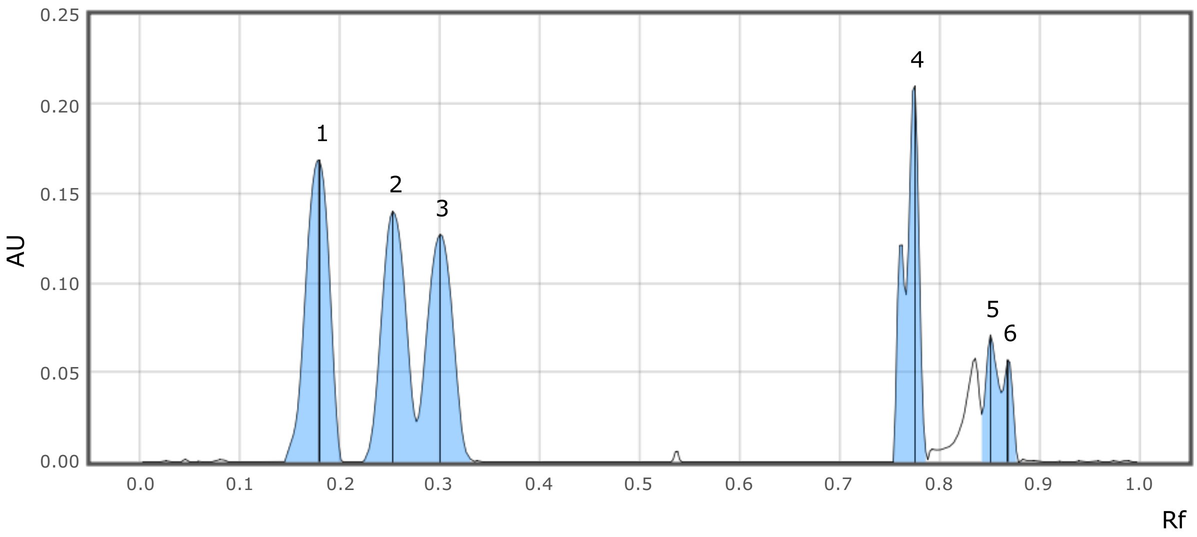

Peak #	Start Rf	Start H	Max Rf	Max H	Max %	End Rf	End H	Area A	Area %	Manual peak	Substance Name
1	0.142	0.0000	0.179	0.1687	21.80	0.203	0.0000	0.00463	26.58	No	CBN
2	0.222	0.0000	0.253	0.1400	18.10	0.276	0.0225	0.00389	22.32	No	9-THC
3	0.276	0.0225	0.300	0.1273	16.44	0.335	0.0002	0.00374	21.50	No	CBD
4	0.754	0.0000	0.776	0.2100	27.13	0.789	0.0013	0.00353	20.28	No	
5	0.843	0.0266	0.851	0.0710	9.17	0.862	0.0387	0.00099	5.71	No	
6	0.862	0.0387	0.868	0.0569	7.36	0.879	0.0000	0.00063	3.61	No	

Track 15:

Type	Sample
Vial ID	MeOH blank
Description	MeOH Blank
Volume	2.0 µl

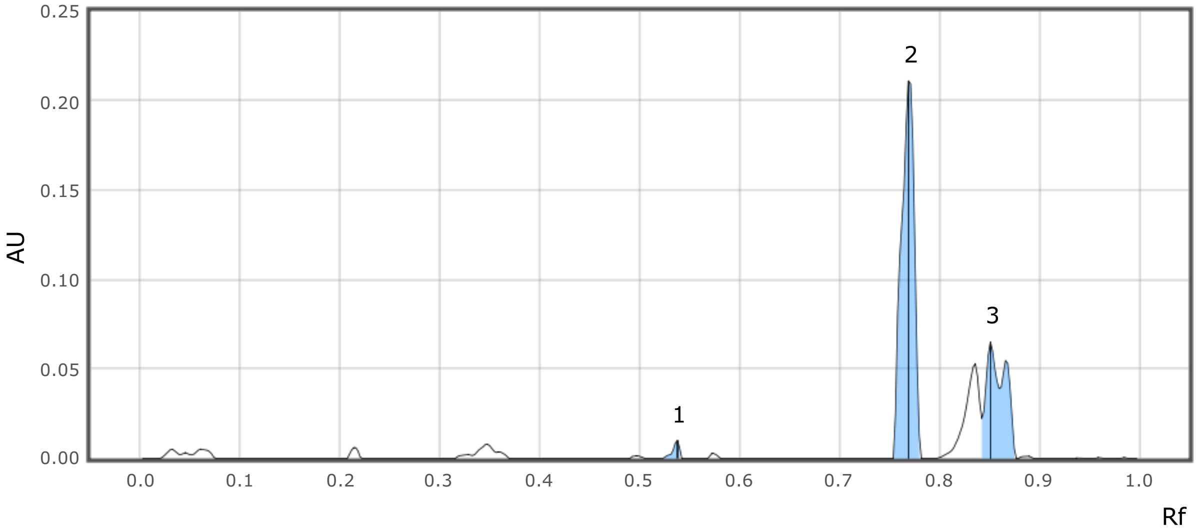

Peak #	Start		Max			End		Area		Manual peak	Substance Name
	Rf	H	Rf	H	%	Rf	H	A	%		
1	0.523	0.0000	0.538	0.0100	3.50	0.542	0.0000	0.00008	1.63	No	
2	0.754	0.0000	0.769	0.2110	73.77	0.782	0.0000	0.00316	67.95	No	
3	0.843	0.0223	0.851	0.0650	22.73	0.877	0.0000	0.00142	30.42	No	

Calibration results:

Height calibration for substance 9-THC @ RT White:

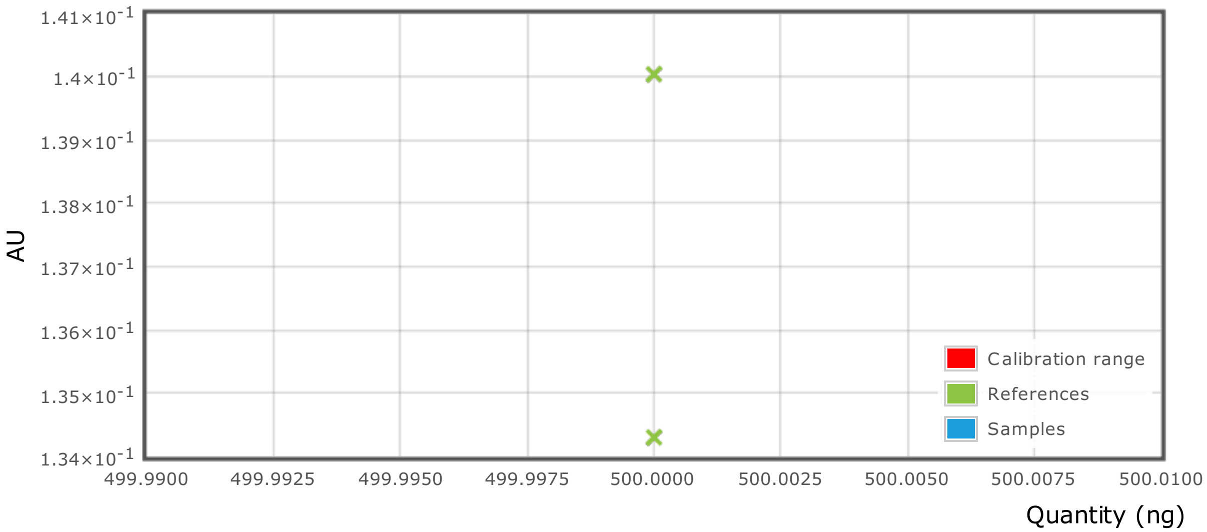

Regression mode	Linear-2
Range deviation	5.00 %
Related substances	Default
Number of references	2
Calibration function	$y=0x$
Coefficient of variation	CV 0.00 %
Correlation coefficient	n/a
⚠	Unable to compute the results for this substance because there wasn't enough groups of references replicas (at least 1 for Linear-1, 2 for Linear2 and Mime-1 and 3 for Polynomial and MiMe-2)

Height calibration for substance CBD @ RT White:

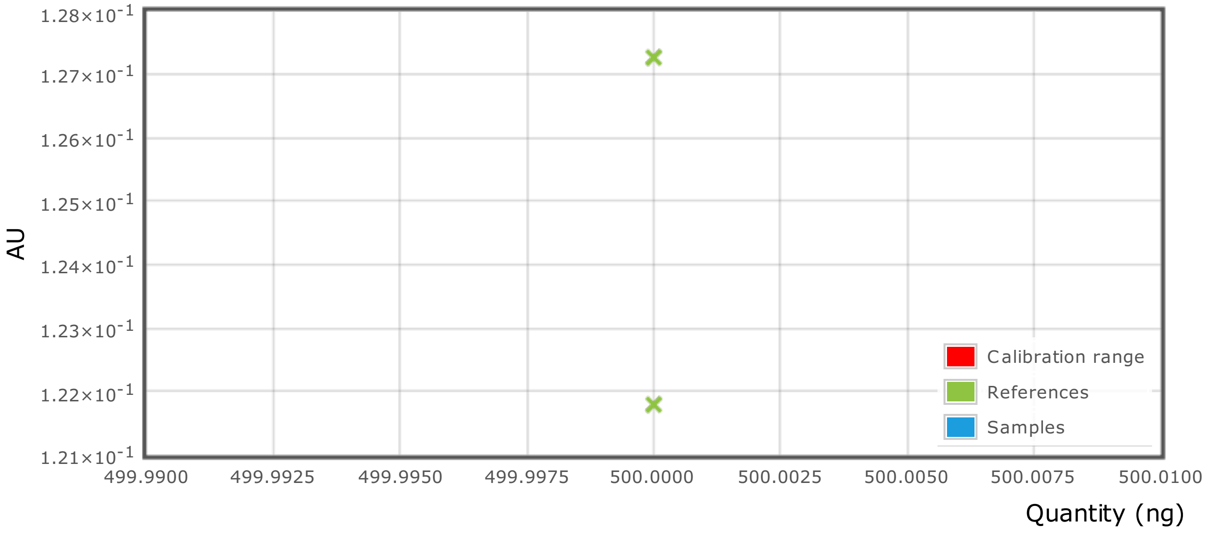

Regression mode	Linear-2
Range deviation	5.00 %
Related substances	Default
Number of references	2
Calibration function	$y=0x$
Coefficient of variation	CV 0.00 %
Correlation coefficient	n/a
⚠	Unable to compute the results for this substance because there wasn't enough groups of references replicas (at least 1 for Linear-1, 2 for Linear2 and Mime-1 and 3 for Polynomial and MiMe-2)

Height calibration for substance CBN @ RT White:

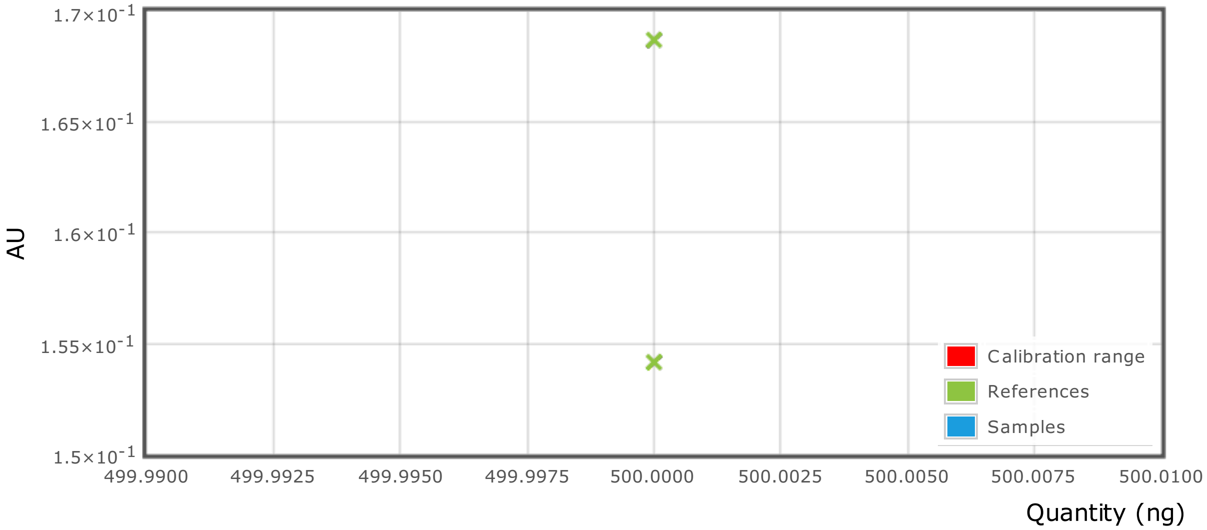

Regression mode	Linear-2
Range deviation	5.00 %
Related substances	Default
Number of references	2
Calibration function	$y=0x$
Coefficient of variation	CV 0.00 %
Correlation coefficient	n/a
⚠	Unable to compute the results for this substance because there wasn't enough groups of references replicas (at least 1 for Linear-1, 2 for Linear2 and Mime-1 and 3 for Polynomial and MiMe-2)

Results:

Substance having no available results		
⚠	9-THC	Unable to compute the results for this substance because there wasn't enough groups of references replicas (at least 1 for Linear-1, 2 for Linear2 and Mime-1 and 3 for Polynomial and MiMe-2)
⚠	CBN	Unable to compute the results for this substance because there wasn't enough groups of references replicas (at least 1 for Linear-1, 2 for Linear2 and Mime-1 and 3 for Polynomial and MiMe-2)
⚠	CBD	Unable to compute the results for this substance because there wasn't enough groups of references replicas (at least 1 for Linear-1, 2 for Linear2 and Mime-1 and 3 for Polynomial and MiMe-2)

A track marked with ⚠ means: this result is outside the regression range given by the reference assignments, but is included in the results because it is in the allowed range deviation.

Analyst:	Reviewer:
